# The rapamycin-regulated gene expression signature determines prognosis for breast cancer

**DOI:** 10.1186/1476-4598-8-75

**Published:** 2009-09-24

**Authors:** Argun Akcakanat, Li Zhang, Spiridon Tsavachidis, Funda Meric-Bernstam

**Affiliations:** 1 Department of Surgical Oncology, The University of Texas MD Anderson Cancer Center, Houston, Texas, 77030, USA; 2 Department of Bioinformatics and Computational Biology, The University of Texas MD Anderson Cancer Center, Houston, Texas, 77030, USA

## Abstract

**Background:**

Mammalian target of rapamycin (mTOR) is a serine/threonine kinase involved in multiple intracellular signaling pathways promoting tumor growth. mTOR is aberrantly activated in a significant portion of breast cancers and is a promising target for treatment. Rapamycin and its analogues are in clinical trials for breast cancer treatment. Patterns of gene expression (metagenes) may also be used to simulate a biologic process or effects of a drug treatment. In this study, we tested the hypothesis that the gene-expression signature regulated by rapamycin could predict disease outcome for patients with breast cancer.

**Results:**

Colony formation and sulforhodamine B (IC_50 _< 1 nM) assays, and xenograft animals showed that MDA-MB-468 cells were sensitive to treatment with rapamycin. The comparison of *in vitro *and *in vivo *gene expression data identified a signature, termed rapamycin metagene index (RMI), of 31 genes upregulated by rapamycin treatment *in vitro *as well as *in vivo *(false discovery rate of 10%). In the Miller dataset, RMI did not correlate with tumor size or lymph node status. High (>75th percentile) RMI was significantly associated with longer survival (*P *= 0.015). On multivariate analysis, RMI (*P *= 0.029), tumor size (*P *= 0.015) and lymph node status (*P *= 0.001) were prognostic. In van 't Veer study, RMI was not associated with the time to develop distant metastasis (*P *= 0.41). In the Wang dataset, RMI predicted time to disease relapse (*P *= 0.009).

**Conclusion:**

Rapamycin-regulated gene expression signature predicts clinical outcome in breast cancer. This supports the central role of mTOR signaling in breast cancer biology and provides further impetus to pursue mTOR-targeted therapies for breast cancer treatment.

## Background

Mammalian target of rapamycin (mTOR) is a serine/threonine kinase involved in multiple intracellular signaling pathways promoting tumor growth [1]. The phosphatidylinositol 3-kinase (PI3K)/Akt/mTOR signaling pathway in particular is deregulated in many cancers, including breast cancer. PI3K activates Akt, which regulates various cellular processes and promotes cell survival. mTOR is a downstream effector of the PI3K/Akt pathway and phosphorylates S6 kinase (S6K1) and 4E-binding protein-1 (4E-BP1), which control cell growth and proliferation and protein translation. Furthermore, PI3K is a mediator of oncogenesis in breast cancer cases. Mutations in the PI3K catalytic subunit p110α [2,3] and overexpression of growth factor receptors such as HER2/neu [4], epidermal growth factor receptor [5], insulin-like growth factor receptor [6], and integrins [7] may activate PI3K signaling. Additionally, phosphatase and tensin homologue deleted from chromosome 10 (PTEN) is a negative regulator of the PI3K/Akt pathway. Germ-line PTEN mutations lead to Cowden disease, which predisposes patients to breast cancer. PTEN is downregulated in one third of patients with breast cancer [8] and PTEN loss is associated with poor prognosis for this malignancy [9]. In addition, authors have reported Akt1 mutations [10], increased Akt1 kinase activity [11], genomic amplification of Akt2 [12], and overexpression of phosphorylated Akt protein [13]. Thus, various aberrations activate mTOR, which has a key role in translation, cell growth, apoptosis and angiogenesis.

Rapamycin is an antibiotic and fungicide isolated from *Streptomyces hygroscopicus *[14]. It forms a complex with FK506-binding protein 12 that binds and inhibits mammalian target of TOR kinase activity, leading to dephosphorylation of downstream targets of mTOR, S6K1, and 4E-BP1 [15]. S6K1 and 4E-BP1 regulate ribosomal component biogenesis and cap-dependent mRNA translation, and their dephosphorylation inhibits translation of mRNAs involved in cell cycle, proliferation, and induction of growth arrest at G1 phase.

The U.S. Food and Drug Administration approved rapamycin analog temsirolimus (Toricel, CCI-779; Wyeth) and everolimus (Afinitor, RAD001, Novartis) for patients with advanced renal cell carcinoma. Clinical trials evaluating the efficacy of rapamycin and its analogs alone or in combination with other agents in patients with breast cancer are ongoing. However, in the Phase II trial of temsirolimus in heavily pretreated locally advanced or metastatic breast cancer, temsirolimus produced an objective response rate of 9.2% in the intent-to-treat population [16]. Thus there is an urgent need to identify minority subpopulations of patients that are sensitive to certain pathway inhibition, better understand the mechanism of action of rapamycin and its analogs, and identify markers of pathway activity.

Researchers are actively pursuing transcriptional profiling as a prognostic and predictive tool in breast cancer therapy. Transcriptional response to modulation of a gene or signaling pathway may not only allow identification of novel targets of well-characterized genes but may also define a pattern of mRNA expression (metagene), which can serve as a molecular indicator of gene and/or pathway activation [17]. Recent studies identified gene expression signatures of several pathways, such as Akt [18], cyclin D1 [19], KRAS2 [20], Myc, Ras, E2F3, Src, β-catenin [21], ErbB2, epidermal growth factor receptor, Raf, and MEK [22]. In the study described herein, we defined a rapamycin-regulated gene signature as a set of genes whose expression is upregulated when mTOR activity is inhibited by rapamycin *in vitro *as well as *in vivo*. We hypothesized that this rapamycin-regulated gene signature determines prognosis for breast cancer, and we tested its ability to predict the outcome of this disease using three independent publicly available primary breast cancer data sets.

## Results

### Identification of differentially expressed genes in breast cancer cells and generation of a rapamycin-regulated gene expression signature

**Figure 1:**
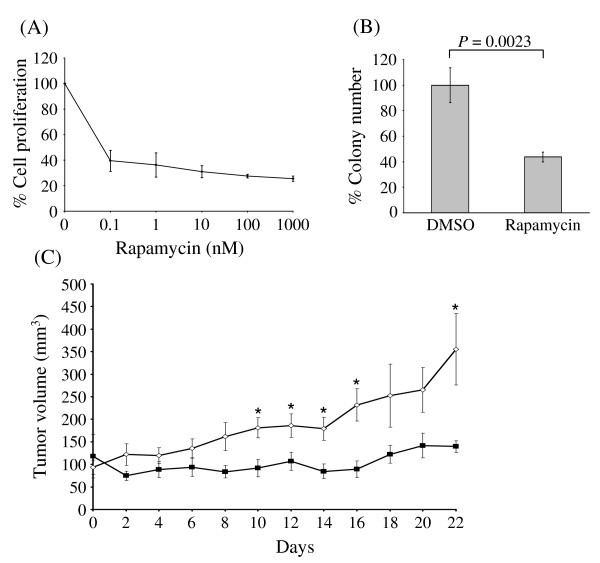
**Rapamycin sensitivity of the MDA-MB-468 breast cancer cell line**. **(A) **MDA-MB-468 cells were treated with rapamycin at various concentrations. SRB assay was performed 5 days later. The assay results shown are the mean (± standard deviation) of three independent experiments. **(B) **2 × 10^3 ^MDA-MB-468 cells were plated in 60-mm plates in triplicate and treated with DMSO or 100 nM rapamycin. Two weeks later, cell colonies were stained with crystal violet, and the plates were scanned and colonies quantitated. The results shown are the mean (± standard deviation) for three plates. **(C) **Mice with established MDA-MB-468 tumor xenografts received DMSO or rapamycin (15 mg/kg) intraperitoneally once a week for 3 weeks. The tumor volumes were then measured using calipers every other day and presented as the mean (± standard error of the mean). Solid line, rapamycin; dashed line, DMSO. * *P *< 0.05.

We sought to identify genes differentially expressed in response to treatment with rapamycin in MDA-MB-468 cells, a PTEN-null human breast cancer cell line with constitutive activation of PI3K/Akt/mTOR signaling [23-25]. To confirm the rapamycin sensitivity of MDA-MB-468 cells *in vitro*, we treated them with rapamycin at concentrations ranging from 0.1 to 1000 nM for 5 days. Fig. 1A shows the inhibitory effect of rapamycin on cell growth. The IC_50 _of rapamycin was less than 1 nM. We also assessed the effect of rapamycin on anchorage-dependent growth of MDA-MB-468 cells using a colony formation assay. Rapamycin treatment resulted in a significant decline in colony-forming ability in these cells (*P *= 0.0023) (Fig. 1B).

To determine rapamycin's effects on *in vivo *tumor growth, we injected MDA-MB-468 cells into mammary fat pads of athymic nude mice. We then gave the animals injections of DMSO or rapamycin (15 mg/kg weekly) intraperitoneally for 3 weeks. We observed a statistically significantly lower mean tumor volume (*P *< 0.05) on day 22 after injection in the mice given rapamycin (140 mm^3^) than in the control mice (355 mm^3^) (Fig. 1C). This demonstrated that MDA-MB-468 cells are sensitive to the growth-inhibitory effect of rapamycin *in vivo*.

The ratio of total expression of rapamycin-treated RNA to that DMSO-treated RNA defined the changes in the transcriptional states for individual RNAs. Of the 1271 differentially expressed genes by rapamycin treatment, 477 showed upregulation and 794 showed downregulation *in vitro *(false discovery rate [FDR] = 10%). To examine early and late rapamycin-mediated gene-expression changes *in vivo*, we assessed the effect of rapamycin on MDA-MB-468 xenografts in nude mice after 24 h and 3 weeks of treatment. These specific time-points were selected as 24 h and 3 week post-treatment biopsies have been incorporated into some of the ongoing clinical trials with rapamycin and its analogues. There was no significant interaction between treatment (vehicle and rapamycin) and time (1-day and 22-day) *in vivo *study. However, treatment and time regulated expression of several genes. Gene set enrichment analysis (GSEA) results show upregulated and downregulated gene sets (See Additional files 1, 2, 3: Gene set enrichment analysis of *in vivo *data) [26]. Treatment effect is regulating genes sets that are involved in immune response and metabolism, whereas time effect regulates gene sets that are involved in hypoxia, cancer and metabolism.

We used the averages of rapamycin and vehicle treatment over two time points, of the 377 differentially expressed genes, 303 showed upregulation and 74 showed downregulation *in vivo *(FDR = 10%). To identify genes whose expression was regulated *in vitro *and *in vivo*, we compared differentially expressed genes using Affymetrix probe set identifiers which generated a list of 34 entries. Treatment with rapamycin upregulated the expression of 31 of these probes and downregulated that of 3. We then used these 31 probe sequences belonging to 29 genes whose expression was upregulated by rapamycin and designated this gene signature as the rapamycin metagene index (RMI) (Table 1). One of these probe sequences did not have a matching gene sequence, and granulin had two hits; expression of both probe sets was upregulated. The three downregulated genes that were not included in the RMI were DDIT4, GPR107 and ZNF419.

**Table 1:** The 31 probe sets (29 genes) in Rapamycin Metagene Index listed by probe set identifier.

**Probe set ID**	**Gene symbol**	**Gene title**	**Included in HG-U133 array**
202050_s_at	ZMYM4	Zinc finger, MYM-type 4	Yes

202623_at	C14orf1	E2F-associated phosphoprotein	Yes

203985_at	ZNF212	Zinc finger protein 212	Yes

204279_at	PSMB9	Proteasome (prosome, macropain) subunit, beta type, 9 (large multifunctional protease 2)	Yes

204985_s_at	MGC2650	Trafficking protein particle complex 6A	Yes

208669_s_at	CRI1	CREBBP/EP300	Yes

209101_at	CTGF	Connective tissue growth factor	Yes

209102_s_at	HBP1	HMG-box transcription factor 1	Yes

209216_at	WDR45	WD repeat domain 45	Yes

210296_s_at	PXMP3	Peroxisomal membrane protein 3, 35 kDa (Zellweger syndrome)	Yes

211284_s_at	GRN	Granulin	Yes

214177_s_at	PBXIP1	Pre-B-cell leukemia transcription factor-interacting protein 1	Yes

215464_s_at	TAX1IP3	Tax1 (human T-cell leukemia virus type I)-binding protein 3	Yes

216041_x_at	GRN	Granulin	Yes

217906_at	KLHDC2	Kelch domain containing 2	Yes

218550_s_at	LRRC20	Leucine-rich repeat containing 20	Yes

219630_at	MAP17	PDZK1-interacting protein 1	Yes

221087_s_at	APOL3	Apolipoprotein L, 3	Yes

221476_s_at	RPL15	Ribosomal protein L15	Yes

46256_at	SSB3	SPRY domain-containing SOCS box protein SSB-3	Yes

1555852_at	-	-	No

222574_s_at	DHX40	DEAH (Asp-Glu-Ala-His) box polypeptide 40	No

222802_at	EDN1	Endothelin 1	No

223042_s_at	FUNDC2	FUN14 domain-containing 2	No

223493_at	FBXO4	F-box protein 4	No

224564_s_at	RTN3	Reticulon 3	No

224785_at	MGC29814	Hypothetical protein MGC29814	No

225076_s_at	KIAA1404	Zinc finger, NFX1-type containing 1	No

225898_at	WDR54	WD repeat domain 54	No

226157_at	TFDP2	Transcription factor Dp-2 (EF2 dimerization partner 2)	No

227475_at	FOXQ1	Forkhead box Q1	No

ID, identifier.

### The RMI as a prognostic factor for breast cancer in the independent primary breast cancer data sets

**Figure 2:**
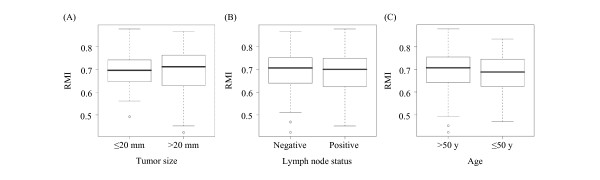
**Lack of correlation of the RMI with prognostic factors for breast cancer in Miller et al. data set **[27]. The nonparametric box plots show interquartile range, horizontal line is mean. The RMI is distributed according to **(A) **tumor size, **(B) **lymph node status, and **(C) **patient age. o, outlier.

**Figure 3:**
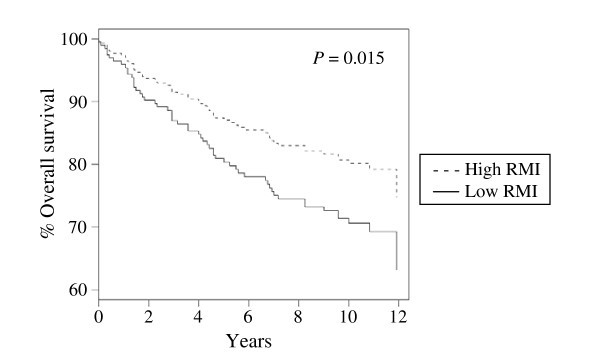
**Overall survival rate according to the RMI in patients with breast cancer in Miller et al. data set **[27]. Overall survival rate based on high and low RMI values were calculated using Cox proportional hazards analysis.

**Figure 4:**
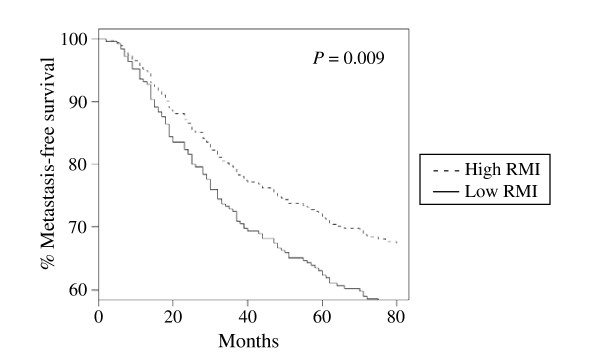
**Metastasis-free survival rate according to the RMI in patients with breast cancer in Wang et al. data set **[28]. Metastasis-free survival rate based on high and low RMI values were calculated using of Cox proportional hazards analysis.

We hypothesized that if rapamycin indeed regulates a critical oncogenic pathway in breast cancer, then RMI would correlate with breast cancer outcome. To determine whether the RMI can provide prognostic information about breast cancer, we applied it to the three well-described, publicly available primary breast cancer data sets described above. The sets described by Miller et al. [27] and by Wang et al. [28] were Affymetrix-based data sets, and we correlated the gene-expression levels with our study using the corresponding probe set identifiers. We analyzed the HG-U133A probe set in the data set described by Miller and colleagues. Of the 31 probes in the HG-U133 Plus 2.0 chips, we included 20 that were present in HG-U133A array and used them for cross-study comparisons. We also applied RMI to van 't Veer data set which was performed by using Hu25K microarray chip (Agilent platform) [29]. The probes in our and Wang data sets were matched by using gene symbols and 26 of the 29 genes were present. The data set used by Miller et al. represents 251 patients with primary breast cancer who underwent surgery. They used no patient selection criteria. In this data set, the RMI did not correlate with the following known prognostic factors for breast cancer: tumor size (*P *= 0.36), lymph node status (*P *= 0.93), and patient age (*P *= 0.22) (Fig. 2). However, the overall survival rate based on the high and low RMI values showed a significant difference in between the two values (*P *= 0.015), with the high RMI group having longer survival rates (Fig. 3). Multivariate analysis indicated that RMI (*P *= 0.029), tumor size (*P *= 0.015), and lymph node status (*P *= 0.001) were prognostic for overall survival in breast cancer (Table 2). van 't Veer et al. [29] selected 97 patients with sporadic primary breast cancer who had lymph node-negative disease and were younger than 55 years of age at the time of diagnosis. RMI was not associated with time to development of distant metastasis in these patients (*P *= 0.41). Wang et al. [28] included in their data set 286 patients with lymph node-negative breast cancer who did not receive systemic neoadjuvant or adjuvant therapy. In this data set, the RMI predicted the metastasis-free survival rate (*P *= 0.009), with the high RMI value associated with a better disease course than the low RMI value was (Fig. 4).

**Table 2:** Cox multivariate regression analysis of survival according to clinical factors in the primary breast cancer data sets used.

	**Miller et al**. [27]			**Wang et al**. [28]			**van 't Veer et al**. [29]		
**Factor**	**HR**	**95% CI**	***P***	**HR**	**95% CI**	***P***	**HR**	**95% CI**	***P***

RMI	0.03^a^	0.00-0.69	0.029	0.27	0.10-0.71	0.008	0.23	NS	0.28

LN status (negative versus positive)	2.74	1.50-5.01	0.001						

Tumor size^b^	1.03	1.01-1.06	0.015						

Age^b^	1.01	0.99-1.03	NS						

Grade (low [1 or 2] versus high 3)	1.43	0.90-2.26	NS						

ER status (negative versus positive)	1.44	0.60-3.48	NS	1.07	0.69-1.65	NS	0.57	0.32-1.03	NS

Clinical data and the RMI with relative risk (hazards ratio), confidence interval, and *P *value were fitted to each of the clinical factors.

^a^A coefficient of 0.03 means that the model gives a 97% decrease of the predicted hazard for an increase of 1 unit of RMI.

^b^There was no stratification of the variables, actual values were used in analysis.

HR, hazards ratio; CI, confidence interval; LN, lymph node; NS, not significant; ER, estrogen receptor.

## Discussion

The mTOR pathway is activated in breast cancer and has become a promising target for breast cancer therapy. mTOR activation contributes to the malignant phenotype by increasing protein synthesis, cell proliferation, angiogenesis, and nutrient uptake. Herein we show that the RMI is associated with overall and metastasis-free survival rate in patients with breast cancer. Furthermore, our multivariate analysis showed that the RMI is prognostic for breast cancer. These data indicate that the mTOR pathway is important to breast carcinogenesis.

By identifying human microarray probe sets corresponding to the genes in the three data sets impacted by rapamycin treatment, we identified a rapamycin-regulated gene expression signature that predicts prognosis for breast cancer. Several studies have characterized transcriptional response to treatment using cell culture experiments, whereas others have related *in vitro *experiments with *in vivo *experimental models [20,30]. Gene expression signatures generated in cell lines may be predictive of clinical response, suggesting that despite major differences in tumor microenvironment, at least some key oncogenic signatures are conserved *in vitro *and *in vivo*. Thus, we may be able to effectively use preclinical data to discover clinically relevant biomarkers. Our strategy described above of combining preclinical data obtained in cell culture experiments as well using established xenograft models may generate a robust gene expression signature that may be useful for both *in vitro *and *in vivo *studies. We also applied GSEA and determined the effect of treatment and time *in vivo*. Compared to 1-day treatment, 22-day treatment (time effect) increased the expression of gene sets involved in response to hypoxia and cancer. These findings further support importance of mTOR as a central controller integrating signals coming from separate pathways.

Other researchers have also investigated the effect of treatment with rapamycin and its analogues on gene expression. Gera et al. [31] studied Akt activation and mTOR inhibition by rapamycin in prostate cancer and glioblastoma cell lines *in vitro*. They identified 62 regulated genes and expression of 29 them were upregulated; however, none of these genes were on our RMI list. Majumder et al. [32] used a transgene to produce activated Akt1 in luminal epithelial cells in the ventral murine prostate. A prostatic intraepithelial neoplasia (PIN) phenotype developed in the transgenic mice, which was completely reversed by mTOR inhibition by the rapamycin analogue everolimus (RAD001; Novartis), by inducing apoptosis. They identified 571 genes or ESTs whose expression was altered by Akt expression and mTOR inhibition. Further analysis by using gene set enrichment analysis (GSEA) revealed inactivation of hypoxia inducible factor 1α (HIF-1α) and its target genes, including genes coding enzymes involved in glycolysis pathway, which are all regulated by mTOR. We used our rapamycin-responsive gene set to probe the gene set used in that study and identified only endothelin 1 gene common in both sets. Interestingly, in our study endothelin 1 gene expression was downregulated whereas in Majumder et al. study upregulated. Besides, rapamycin treatment does not induce apoptosis in breast cancer cell lines [25], thus the downstream effects of rapamycin in these two models may be different. Absence of concordance may not be surprising considering this is a comparison of gene expression in a breast cancer cell line with that of a model of Akt-activated mouse PIN. As stated by Majumder et al., cell lines and xenografts show a more complex genetic background than an Akt-activation model as survival and adaptive events have already taken place. Creighton re-analyzed the Majumder et al. study data and identified "Akt-mTOR-dependent (RAD001-sensitive)" genes, which were higher in human breast tumors having high Akt mRNA [18]. This signature of 101 genes was applied to five publicly available breast cancer databases and high expression of these genes in several datasets were associated with more metastasis, shorter time of disease free survival, ER negative status, higher grade, and increase in tumor size. This was an application of Akt-mTOR signature derived from a mouse model of Akt activation in prostate to human breast cancer showing that the genes were not tissue or model specific. There were no matches between RMI and Akt-mTOR-dependent gene signatures. Also of note, Saal et al. generated an "immunohistochemistry-detectable PTEN loss" signature in breast cancer showing activation of PI3K/Akt signaling pathway [33]. This signature of 246 genes was applied to two established breast cancer datasets and identified metastasis and poor prognosis [33]. There were no matches between RMI and PTEN loss gene signatures. Thus, although we and Creighton, and Saal et al. used different gene expression signatures, all mTOR-regulated gene sets were prognostic for breast cancer, supporting an important role for mTOR in breast cancer. This agrees with the results of studies of the prognostic role of mTOR pathway activation in breast cancer using immunohistochemistry. In a tissue array-based analysis of 285 patients with breast cancer, Bose et al. [13] showed that overexpression of phosphorylated mTOR increased the risk of recurrence threefold. Similarly, using immunohistochemistry, Zhou et al. [34] showed that overexpression of phosphorylated mTOR protein in breast cancer is an indicator of decreased disease-free survival rate, whereas decreased expression of phosphorylated Akt and phosphorylated 4E-BP1, which is an mTOR downstream target, are indicators of increased disease-free survival rate.

Use of microarrays enables simultaneous analysis of thousands of genes in a single step, which leads to identification of groups of genes working in a similar way. Because several genes are involved in the same biological processes, the fact that several gene sets carry prognostic information for cancer and that gene signatures generated in different studies may not overlap is not surprising. Technical differences among the studies contribute to the discrepancy in gene expression data, such as different microarray platforms, probes, RNA-labeling methods, and gene sets [35]. Microarray-based studies of breast cancer usually focus on three main uses of gene expression profiling [36]. First, gene expression profiling may can generate a molecular classification of breast cancer into different subsets according to clinical subtype, such as high versus low grade [37-41]. Second, profiling of genes associated with clinical outcome of patients, such as time to death or relapse, may help clinicians predict risk of failure after surgery [28,29,42,43] and individualize the use of adjuvant therapy based on the predicted risk of relapse. Third, gene expression profiling may be used to predict breast cancer response to specific treatment regimens, which is potentially best studied in the neoadjuvant setting [21,44-46]. A predictive gene signature may be used to identify patients, whose disease will not respond to one drug regimen but will to another regimen, thereby making breast cancer treatment more precise and individualized.

In this study, we applied RMI to independent primary breast cancer data sets to confirm the importance of mTOR signaling in breast cancer biology. We identified a rapamycin-regulated gene signature that is a significant predictor of breast cancer prognosis. For clinical use, identifying rapamycin-mediated gene expression changes in a variety of tumors responsive to mTOR inhibition would be ideal. Although several clinical trials using correlative studies are ongoing, the results have been slow to arrive. The reason for this is that many of these trials are conducted in the metastatic setting, in which accessibility and the relative tumor cellularity of metastatic tumors are limiting, as is the relatively modest objective response rates achieved using single-agent therapy. Thus, identification of oncogenic gene expression signatures in the preclinical setting using well-characterized rapamycin-sensitive cancer models may facilitate discovery of profiles that can then be tested prospectively in the clinic and retrospectively.

Although researchers are actively studying mTOR inhibitors in the treatment of many tumor types in hundreds of clinical trials, which patients will have a response and/or clinically benefit from mTOR inhibition remains unclear. Thus, the need to identify markers of response to mTOR inhibitors for patient selection and pharmacodynamic markers for early response assessment is a pressing one. Further work is needed to determine whether examination of the RMI can identify patients with breast cancer who have baseline activation of mTOR signaling and thus would benefit from treatment with rapamycin or its analogues. It also needs to be determined whether an increase in the RMI in response to treatment to rapamycin may serve as an early indicator of clinical response to mTOR inhibition. Because rapamycin modulates gene expression postranscriptionally [31,47,48], we are also seeking to determine whether incorporation of functional proteomics complements gene expression profiling in identification of patients with breast cancer who have activation of mTOR signaling and monitoring response of breast cancer to therapy.

## Methods

### Cell line and reagents

MDA-MB-468 cells were obtained from the American Type Tissue Culture Collection and cultured in Dulbecco's modified Eagle's medium/F12 medium supplemented with 10% fetal bovine serum at 37°C and humidified in 5% CO_2_. Rapamycin was purchased from LC Laboratories. All other chemicals were purchased from Sigma Chemical Company and Fisher Scientific.

### Cell proliferation assay and dose-effect analysis

To test the effect of rapamycin, 5 × 10^3 ^MDA-MB-468 cells per 100 μL per well were plated in 96-well flat-bottomed plates. After overnight incubation, cells in triplicate wells were treated with rapamycin at various concentrations for 5 days. Cell proliferation was then analyzed by comparing the protein content of rapamycin-treated cells with that of vehicle (dimethyl sulfoxide [DMSO])-treated cells using a sulforhodamine B (SRB) assay. The assay results were assessed using a 96-well plate reader by measuring the absorbance at a wavelength of 570 nm. The IC_50 _of rapamycin was determined based on dose-response curves using the SRB assay with the CalcuSyn software program (Biosoft). Experiments were repeated three times, and the mean IC_50 _values are reported.

### Colony formation assay

MDA-MB-468 cells were plated at a density of 2 × 10^3 ^cells per 60-mm plate in triplicate. After overnight incubation, cells were treated with DMSO or 100 nM rapamycin. Two weeks later, plates were fixed, stained with crystal violet, and scanned, and the cell colonies in the plates were counted using the ImageJ software program (National Institutes of Health).

### Animal studies

All animal studies were conducted according to the guidelines of the American Association of Laboratory Animal Care under an approved protocol. Eight-week-old female athymic nude (*nu/nu*) mice (Harlan Sprague Dawley Inc.) were inoculated with 1.5 × 10^7 ^MDA-MB-468 cells in the mammary fat pad. Thirty days after inoculation, the resulting breast tumor volumes had reached 75-150 mm^3^, and the mice were placed in four experimental groups. The mice in the first and second groups (five per group) received a single injection of DMSO or rapamycin (15 mg/kg) intraperitoneally. The mice in the third and fourth groups (five per group) received weekly injections of DMSO or rapamycin for 3 weeks. The tumors were measured every other day using calipers and the formula 1/2 × a^2 ^× b, in which a is the short axis and b is the long axis. Twenty-four hours after the last injection, the mice were killed using cervical dislocation. Samples of the tumors were collected in RNAlater (Ambion) for RNA extraction.

### Total RNA extraction, amplification, labeling, and hybridization

Total RNA was extracted from MDA-MB-468 cells using TRIzol reagent (Invitrogen) according to the manufacturer's recommendations. Total RNA was also extracted from the breast tumor xenografts described above using an RNeasy kit (Qiagen) following the manufacturer's recommendations. RNA purity and integrity were controlled using a 2100 Bioanalyzer (Agilent Technologies). Total RNA was extracted from three separate MDA-MB-468 cell culture plates or breast tumor samples for each treatment condition, as described above, generating 18 RNA-extraction experiments (6 with MDA-MB-468 cell line and 12 with xenograft samples).

Microarray hybridization analysis was performed according to the protocol described in the Affymetrix Expression Analysis Technical Manual. Briefly, 5 μg of total RNA extracted from cell culture or xenograft was reverse-transcribed and amplified. The RNA was labeled using the BioArray high-yield RNA transcript labeling kit (Enzo Biochem Inc.) following the manufacturer's recommendations. Biotin-labeled cRNA was purified, quantified, and fragmented. Hybridization and scanning were performed at The University of Texas M. D. Anderson Cancer Center Microarray Core Facility. Fifteen micrograms of labeled cRNA was then hybridized to Affymetrix Human Genome U133 (HG-U133) Plus 2.0 chips (Affymetrix, Inc.). The chips were washed and stained according to the Affymetrix Expression Analysis Technical Manual.

### Microarray gene expression analysis

All data preprocessing and statistical analyses were performed in R software. As part of standard quality control analysis, the .CEL files were quantified using the MAS5 algorithm. The probe intensities were processed using a position-dependent nearest neighbor (PDNN) model to estimate gene expression values [49]. Array images, markers bar plot, box plot, and sample cluster figures were generated to confirm the data quality. Paired and unpaired Student *t*-tests were used to determine the effect of rapamycin in our cell culture study and animal study, respectively. T statistics, fold change, and *P *values were computed for all probe sets separately. A beta-uniform mixture analysis was performed to assess statistical significance and control the false-discovery rate (FDR) [50].

### Independent data sets

Publicly available primary breast cancer data sets described by Miller et al. [27], van 't Veer et al. [29], and Wang et al. [28] were used in this study.

### Statistical analysis

For *in vitro *and *in vivo *studies, treatment groups of mice were compared using the Student *t*-test. Rapamycin metagene index is calculated as the mean of the log-expression values of 29 genes (represented by 31 probe sets). A Cox proportional hazards model was used to examine whether the (RMI) is an independent prognostic factor for breast cancer. To show the association of RMI with survival, Cox regression analysis of the samples that have "high" (>75th percentile) and "low" (<25th percentile) RMI values was performed. Traditional proportional hazards analysis was established and quantified the prognostic relevance of clinical and biological factors, including lymph node status, tumor size, age, grade, and estrogen receptor status, to the RMI using traditional proportional hazards analysis. The Wilcoxon rank test was used to determine how clinical factors were correlated with the high and low RMI values. All *P *values were two-sided, and *P *values less than 0.05 were considered significant.

## Competing interests

Research funding for clinical trials: Abraxis, Novartis, Merck, (Meric-Bernstam), Honorarium: Novartis (Meric-Bernstam). The other authors declare that they have no competing interests.

## Authors' contributions

FMB designed the experiments. AA performed the experimental work. AA and FMB wrote the manuscript. ST and LZ analyzed the microarray data and helped draft the manuscript. All authors read and approved the final manuscript.

## Supplementary Material

Additional file 1 **Gene set enrichment analysis of *in vivo *data, methods**. Explanation of analysis and interpretation of the data. Click here for file

Additional file 2 **Gene set enrichment analysis of *in vivo *data, time series**. The data provided represent the time series of GSEA. This compressed file contains "Time" shortcut file and "GSEA_time" folder. Clicking on "Time" shortcut opens the index file providing access to analysis files contained in the "GSEA_time" folder. Click here for file

Additional file 3 **Gene set enrichment analysis of *in vivo *data, treatment series**. The data provided represent the treatment series of GSEA. This compressed file contains "Treatment" shortcut file and "GSEA_treatment" folder. Clicking on "Treatment" shortcut opens the index file providing access to analysis files contained in the "GSEA_treatment" folder. Click here for file
