# Supplementary material for: The rapamycin-regulated gene expression signature determines prognosis for breast cancer
Source: Mol Cancer. 2009 Sep 24;8:75. doi: 10.1186/1476-4598-8-75 (PMC2761377; doi:10.1186/1476-4598-8-75)
Supplement: Additional file 1 — Gene set enrichment analysis of in vivo data, methods. Explanation of analysis and interpretation of the data. [file 1476-4598-8-75-S1.doc]

**Gene set enrichment analysis (GSEA) methods**

We investigated the effect of treatment (DMSO or rapamycin) and time (1-day or 3-week) and modeled the microarray gene expression as:

Expression = β_0_ + β_1_ Treatment + β_2_ Time + β_3_ Treatment/Time + error

The model coefficient β_3_ indicates the interaction effect and there is no statistically significant interaction effect. On the other hand, treatment and time have significant effects on several gene sets. For the GSEA we used the values of the β_1_ and β_2_ coefficients for all genes. A positive value of β_1_ means that when rapamycin group has higher mean expression than DMSO group. Similarly, positive β_2_ means that the 3-week group has higher mean expression than the 1-day group.

We applied GSEA for Treatment and Time effects. The starting point is a list (L) of probe sets ranked by the appropriate linear model coefficient (β_1_ or β_2_). Given an a priori defined set of genes (e.g., genes encoding products in a pathway), GSEA determines whether the members of S are randomly distributed within the ordered list of genes L or found mostly at the top of bottom of L. It is expected that the interesting gene sets S will be the ones exhibiting the latter distribution. For this project, we used a curated database of 1687 gene sets; it can be downloaded at <ftp://ftp.broad.mit.edu/pub/gsea/gene_sets/c2.v2.symbols.gmt>.

GSEA has three steps:

- **Computation of an Enrichment Score.** The enrichment score (ES) reflects the degree to which a set S is overrepresented at the top or bottom of the list L.
- **Significance level estimation of ES.** A nominal p-value is computed by performing 1000 permutations to arrive to an estimation of the null distribution of ES.
- **Adjustment for multiple testing.**  A Normalized Enrichment Score is computed (NES). A False Discovery Rate is computed for all sets S. The FDR is the estimated probability that a set with a given NES represents a false positive finding.

The data is presented in GSEA_time.zip and GSEA_treatment.zip files. In each .zip file, there is a shortcut named “Time” and “Treatment,”respectively. Clicking on “Time” and “Treatment” files opens the analysis files. The figures in the directory correspond to the top 50 gene sets with an overabundance of genes at the top of the gene list (large positive values) and similarly the top 50 gene sets corresponding to the bottom of the gene list (large negative values). Positive values mean rapamycin is greater than DMSO for the treatment analysis and 3-week is greater than 1-day for the time analysis. For negative values, it is the opposite. Gene sets showing overabundance of genes with high positive values, are listed under “na_pos” heading and high negative values are listed under “na_neg” heading.
