# Supplementary material for: The rapamycin-regulated gene expression signature determines prognosis for breast cancer
Source: Mol Cancer. 2009 Sep 24;8:75. doi: 10.1186/1476-4598-8-75 (PMC2761377; doi:10.1186/1476-4598-8-75)
Supplement: Additional file 2 — Gene set enrichment analysis of in vivo data, time series. The data provided represent the time series of GSEA. This compressed file contains "Time" shortcut file and "GSEA_time" folder. Clicking on "Time" shortcut opens the index file providing access to analysis files contained in the "GSEA_time" folder. [file 1476-4598-8-75-S2.zip › GSEA_time/ADIPOCYTE_PPARG_UP.html]

Details for gene set ADIPOCYTE\_PPARG\_UP[GSEA]

|  || Dataset | gsea\_time\_collapsed |
| Phenotype | NoPhenotypeAvailable |
| Upregulated in class | na\_neg |
| GeneSet | ADIPOCYTE\_PPARG\_UP |
| Enrichment Score (ES) | -0.63639796 |
| Normalized Enrichment Score (NES) | -1.9233725 |
| Nominal p-value | 0.0 |
| FDR q-value | 0.059729166 |
| FWER p-Value | 0.171 |
Table: GSEA Results Summary

  

Fig 1: Enrichment plot: ADIPOCYTE\_PPARG\_UP      
 Profile of the Running ES Score & Positions of GeneSet Members on the Rank Ordered List

  

| PROBE | GENE SYMBOL | GENE\_TITLE | RANK IN GENE LIST | RANK METRIC SCORE | RUNNING ES | CORE ENRICHMENT || 1 | ABP1 |  |  | 513 | 0.529 | 0.1501 | No |
| 2 | ACSL4 |  |  | 5038 | 0.136 | -0.0246 | No |
| 3 | LIPE |  |  | 10357 | 0.037 | -0.2708 | No |
| 4 | AGT |  |  | 12350 | 0.009 | -0.3646 | No |
| 5 | ADRB3 |  |  | 13897 | -0.014 | -0.4349 | No |
| 6 | RETN |  |  | 14391 | -0.022 | -0.4517 | No |
| 7 | ADIPOQ |  |  | 16202 | -0.053 | -0.5221 | No |
| 8 | GAPDH |  |  | 16748 | -0.064 | -0.5272 | No |
| 9 | CFD |  |  | 17896 | -0.096 | -0.5513 | No |
| 10 | AEBP1 |  |  | 19650 | -0.194 | -0.5721 | Yes |
| 11 | PPARG |  |  | 19907 | -0.226 | -0.5097 | Yes |
| 12 | PC |  |  | 20000 | -0.241 | -0.4344 | Yes |
| 13 | NR1H3 |  |  | 20189 | -0.291 | -0.3472 | Yes |
| 14 | CEBPA |  |  | 20197 | -0.295 | -0.2499 | Yes |
| 15 | FASN |  |  | 20596 | -0.815 | 0.0004 | Yes |
Table: GSEA details [plain text format]

  

Fig 2: ADIPOCYTE\_PPARG\_UP: Random ES distribution      
 Gene set null distribution of ES for **ADIPOCYTE\_PPARG\_UP**

  
