# Supplementary material for: The rapamycin-regulated gene expression signature determines prognosis for breast cancer
Source: Mol Cancer. 2009 Sep 24;8:75. doi: 10.1186/1476-4598-8-75 (PMC2761377; doi:10.1186/1476-4598-8-75)
Supplement: Additional file 2 — Gene set enrichment analysis of in vivo data, time series. The data provided represent the time series of GSEA. This compressed file contains "Time" shortcut file and "GSEA_time" folder. Clicking on "Time" shortcut opens the index file providing access to analysis files contained in the "GSEA_time" folder. [file 1476-4598-8-75-S2.zip › GSEA_time/ADIP_DIFF_CLUSTER3.html]

Details for gene set ADIP\_DIFF\_CLUSTER3[GSEA]

|  || Dataset | gsea\_time\_collapsed |
| Phenotype | NoPhenotypeAvailable |
| Upregulated in class | na\_neg |
| GeneSet | ADIP\_DIFF\_CLUSTER3 |
| Enrichment Score (ES) | -0.3245248 |
| Normalized Enrichment Score (NES) | -1.2095349 |
| Nominal p-value | 0.16239317 |
| FDR q-value | 0.39729732 |
| FWER p-Value | 1.0 |
Table: GSEA Results Summary

  

Fig 1: Enrichment plot: ADIP\_DIFF\_CLUSTER3      
 Profile of the Running ES Score & Positions of GeneSet Members on the Rank Ordered List

  

| PROBE | GENE SYMBOL | GENE\_TITLE | RANK IN GENE LIST | RANK METRIC SCORE | RUNNING ES | CORE ENRICHMENT || 1 | SLC16A1 |  |  | 187 | 0.746 | 0.1044 | No |
| 2 | CD44 |  |  | 641 | 0.487 | 0.1565 | No |
| 3 | PPP1R2 |  |  | 1183 | 0.374 | 0.1871 | No |
| 4 | KLF5 |  |  | 1827 | 0.298 | 0.2011 | No |
| 5 | RCL1 |  |  | 3507 | 0.193 | 0.1489 | No |
| 6 | TIAM1 |  |  | 4045 | 0.172 | 0.1489 | No |
| 7 | FOSL1 |  |  | 4191 | 0.165 | 0.1669 | No |
| 8 | VDR |  |  | 4278 | 0.162 | 0.1874 | No |
| 9 | SAMHD1 |  |  | 4537 | 0.153 | 0.1981 | No |
| 10 | TOP1 |  |  | 4714 | 0.147 | 0.2119 | No |
| 11 | SLC23A2 |  |  | 4745 | 0.146 | 0.2326 | No |
| 12 | XPO1 |  |  | 5413 | 0.126 | 0.2193 | No |
| 13 | PRDX6 |  |  | 6003 | 0.113 | 0.2079 | No |
| 14 | UAP1 |  |  | 6117 | 0.110 | 0.2191 | No |
| 15 | UCP2 |  |  | 6887 | 0.093 | 0.1959 | No |
| 16 | RRAS2 |  |  | 7000 | 0.091 | 0.2044 | No |
| 17 | IL1RL1 |  |  | 7320 | 0.085 | 0.2018 | No |
| 18 | PRR5 |  |  | 9216 | 0.054 | 0.1179 | No |
| 19 | EREG |  |  | 9524 | 0.049 | 0.1105 | No |
| 20 | PLAC8 |  |  | 9590 | 0.048 | 0.1146 | No |
| 21 | BCL3 |  |  | 10515 | 0.034 | 0.0749 | No |
| 22 | SLC7A6 |  |  | 11130 | 0.026 | 0.0490 | No |
| 23 | TUBB2C |  |  | 13311 | -0.006 | -0.0561 | No |
| 24 | GSTO1 |  |  | 13515 | -0.009 | -0.0646 | No |
| 25 | TSC22D3 |  |  | 16172 | -0.052 | -0.1858 | No |
| 26 | ITGA5 |  |  | 18655 | -0.124 | -0.2876 | No |
| 27 | TEAD4 |  |  | 19054 | -0.144 | -0.2850 | No |
| 28 | SRXN1 |  |  | 19868 | -0.220 | -0.2910 | Yes |
| 29 | PPA1 |  |  | 19945 | -0.231 | -0.2595 | Yes |
| 30 | CEBPB |  |  | 20318 | -0.341 | -0.2258 | Yes |
| 31 | SCD |  |  | 20439 | -0.416 | -0.1683 | Yes |
| 32 | POR |  |  | 20501 | -0.476 | -0.0989 | Yes |
| 33 | HSPH1 |  |  | 20585 | -0.683 | 0.0010 | Yes |
Table: GSEA details [plain text format]

  

Fig 2: ADIP\_DIFF\_CLUSTER3: Random ES distribution      
 Gene set null distribution of ES for **ADIP\_DIFF\_CLUSTER3**

  
