# Supplementary material for: The rapamycin-regulated gene expression signature determines prognosis for breast cancer
Source: Mol Cancer. 2009 Sep 24;8:75. doi: 10.1186/1476-4598-8-75 (PMC2761377; doi:10.1186/1476-4598-8-75)
Supplement: Additional file 2 — Gene set enrichment analysis of in vivo data, time series. The data provided represent the time series of GSEA. This compressed file contains "Time" shortcut file and "GSEA_time" folder. Clicking on "Time" shortcut opens the index file providing access to analysis files contained in the "GSEA_time" folder. [file 1476-4598-8-75-S2.zip › GSEA_time/ADIP_DIFF_CLUSTER4.html]

Details for gene set ADIP\_DIFF\_CLUSTER4[GSEA]

|  || Dataset | gsea\_time\_collapsed |
| Phenotype | NoPhenotypeAvailable |
| Upregulated in class | na\_neg |
| GeneSet | ADIP\_DIFF\_CLUSTER4 |
| Enrichment Score (ES) | -0.38640928 |
| Normalized Enrichment Score (NES) | -1.3810109 |
| Nominal p-value | 0.030612245 |
| FDR q-value | 0.28227943 |
| FWER p-Value | 1.0 |
Table: GSEA Results Summary

  

Fig 1: Enrichment plot: ADIP\_DIFF\_CLUSTER4      
 Profile of the Running ES Score & Positions of GeneSet Members on the Rank Ordered List

  

| PROBE | GENE SYMBOL | GENE\_TITLE | RANK IN GENE LIST | RANK METRIC SCORE | RUNNING ES | CORE ENRICHMENT || 1 | SERPINA3 |  |  | 116 | 0.874 | 0.1208 | No |
| 2 | CDCA7 |  |  | 387 | 0.591 | 0.1932 | No |
| 3 | HELLS |  |  | 1882 | 0.293 | 0.1631 | No |
| 4 | DUT |  |  | 1960 | 0.287 | 0.2009 | No |
| 5 | ABCF2 |  |  | 2590 | 0.242 | 0.2053 | No |
| 6 | SNX5 |  |  | 4174 | 0.166 | 0.1524 | No |
| 7 | CSPG2 |  |  | 4202 | 0.164 | 0.1749 | No |
| 8 | MCM5 |  |  | 4893 | 0.141 | 0.1617 | No |
| 9 | NAP1L4 |  |  | 5164 | 0.133 | 0.1678 | No |
| 10 | RAD51 |  |  | 5681 | 0.120 | 0.1601 | No |
| 11 | DNMT1 |  |  | 5950 | 0.114 | 0.1635 | No |
| 12 | SMC2 |  |  | 6113 | 0.110 | 0.1716 | No |
| 13 | INTS2 |  |  | 6876 | 0.094 | 0.1481 | No |
| 14 | PRPS1 |  |  | 7885 | 0.075 | 0.1099 | No |
| 15 | MCM6 |  |  | 8047 | 0.072 | 0.1125 | No |
| 16 | DDX21 |  |  | 8438 | 0.066 | 0.1031 | No |
| 17 | HMGA1 |  |  | 9145 | 0.055 | 0.0767 | No |
| 18 | RRM2 |  |  | 9889 | 0.044 | 0.0469 | No |
| 19 | ECM1 |  |  | 9997 | 0.042 | 0.0478 | No |
| 20 | HMGB2 |  |  | 13259 | -0.005 | -0.1100 | No |
| 21 | POLA2 |  |  | 13786 | -0.013 | -0.1338 | No |
| 22 | MCM4 |  |  | 14787 | -0.028 | -0.1783 | No |
| 23 | CHAF1B |  |  | 16046 | -0.049 | -0.2323 | No |
| 24 | ASS1 |  |  | 19217 | -0.154 | -0.3641 | Yes |
| 25 | MCM7 |  |  | 19264 | -0.157 | -0.3435 | Yes |
| 26 | ABCF1 |  |  | 19432 | -0.172 | -0.3267 | Yes |
| 27 | GRWD1 |  |  | 19478 | -0.176 | -0.3034 | Yes |
| 28 | FEN1 |  |  | 19563 | -0.185 | -0.2807 | Yes |
| 29 | HAT1 |  |  | 19839 | -0.217 | -0.2626 | Yes |
| 30 | IFRD2 |  |  | 20050 | -0.253 | -0.2361 | Yes |
| 31 | CDC6 |  |  | 20126 | -0.270 | -0.2007 | Yes |
| 32 | MRPL18 |  |  | 20450 | -0.425 | -0.1549 | Yes |
| 33 | ASF1B |  |  | 20451 | -0.427 | -0.0932 | Yes |
| 34 | POLD1 |  |  | 20588 | -0.695 | 0.0008 | Yes |
Table: GSEA details [plain text format]

  

Fig 2: ADIP\_DIFF\_CLUSTER4: Random ES distribution      
 Gene set null distribution of ES for **ADIP\_DIFF\_CLUSTER4**

  
