# Supplementary material for: The rapamycin-regulated gene expression signature determines prognosis for breast cancer
Source: Mol Cancer. 2009 Sep 24;8:75. doi: 10.1186/1476-4598-8-75 (PMC2761377; doi:10.1186/1476-4598-8-75)
Supplement: Additional file 2 — Gene set enrichment analysis of in vivo data, time series. The data provided represent the time series of GSEA. This compressed file contains "Time" shortcut file and "GSEA_time" folder. Clicking on "Time" shortcut opens the index file providing access to analysis files contained in the "GSEA_time" folder. [file 1476-4598-8-75-S2.zip › GSEA_time/AGED_MOUSE_HYPOTH_DN.html]

Details for gene set AGED\_MOUSE\_HYPOTH\_DN[GSEA]

|  || Dataset | gsea\_time\_collapsed |
| Phenotype | NoPhenotypeAvailable |
| Upregulated in class | na\_neg |
| GeneSet | AGED\_MOUSE\_HYPOTH\_DN |
| Enrichment Score (ES) | -0.33465272 |
| Normalized Enrichment Score (NES) | -1.2323091 |
| Nominal p-value | 0.13207547 |
| FDR q-value | 0.38499293 |
| FWER p-Value | 1.0 |
Table: GSEA Results Summary

  

Fig 1: Enrichment plot: AGED\_MOUSE\_HYPOTH\_DN      
 Profile of the Running ES Score & Positions of GeneSet Members on the Rank Ordered List

  

| PROBE | GENE SYMBOL | GENE\_TITLE | RANK IN GENE LIST | RANK METRIC SCORE | RUNNING ES | CORE ENRICHMENT || 1 | VSNL1 |  |  | 200 | 0.725 | 0.0889 | No |
| 2 | KIF5B |  |  | 1377 | 0.343 | 0.0784 | No |
| 3 | RAB18 |  |  | 2304 | 0.261 | 0.0689 | No |
| 4 | DHX30 |  |  | 2307 | 0.261 | 0.1044 | No |
| 5 | ATP6V1E1 |  |  | 2387 | 0.256 | 0.1354 | No |
| 6 | APOE |  |  | 2558 | 0.244 | 0.1603 | No |
| 7 | VTI1B |  |  | 2939 | 0.222 | 0.1721 | No |
| 8 | CAMK2G |  |  | 3156 | 0.211 | 0.1903 | No |
| 9 | ATP9A |  |  | 3395 | 0.200 | 0.2059 | No |
| 10 | EEF1A1 |  |  | 3506 | 0.194 | 0.2269 | No |
| 11 | CANX |  |  | 3877 | 0.179 | 0.2333 | No |
| 12 | SARS |  |  | 4111 | 0.168 | 0.2448 | No |
| 13 | PRKACB |  |  | 5252 | 0.130 | 0.2072 | No |
| 14 | ATP2A2 |  |  | 5878 | 0.115 | 0.1925 | No |
| 15 | DNAJA2 |  |  | 6671 | 0.098 | 0.1673 | No |
| 16 | GPX4 |  |  | 6841 | 0.094 | 0.1719 | No |
| 17 | EPAS1 |  |  | 7386 | 0.084 | 0.1569 | No |
| 18 | MGAT2 |  |  | 7657 | 0.079 | 0.1545 | No |
| 19 | MAGED1 |  |  | 7658 | 0.079 | 0.1652 | No |
| 20 | PTGDS |  |  | 9300 | 0.052 | 0.0925 | No |
| 21 | UBC |  |  | 11289 | 0.024 | -0.0009 | No |
| 22 | GRIA1 |  |  | 13784 | -0.013 | -0.1205 | No |
| 23 | ACHE |  |  | 13844 | -0.013 | -0.1215 | No |
| 24 | DNM1 |  |  | 14187 | -0.019 | -0.1356 | No |
| 25 | ATP6V0C |  |  | 14287 | -0.020 | -0.1376 | No |
| 26 | DLG4 |  |  | 14433 | -0.023 | -0.1416 | No |
| 27 | EPRS |  |  | 16116 | -0.050 | -0.2165 | No |
| 28 | GNAI2 |  |  | 17163 | -0.074 | -0.2573 | No |
| 29 | FSTL1 |  |  | 18675 | -0.125 | -0.3138 | Yes |
| 30 | GDI1 |  |  | 18974 | -0.139 | -0.3093 | Yes |
| 31 | CS |  |  | 19240 | -0.156 | -0.3010 | Yes |
| 32 | AP2M1 |  |  | 19933 | -0.229 | -0.3034 | Yes |
| 33 | DDB1 |  |  | 20231 | -0.305 | -0.2763 | Yes |
| 34 | PPP2R1A |  |  | 20299 | -0.333 | -0.2343 | Yes |
| 35 | STIP1 |  |  | 20368 | -0.365 | -0.1880 | Yes |
| 36 | ACO2 |  |  | 20483 | -0.453 | -0.1318 | Yes |
| 37 | ATP6AP1 |  |  | 20522 | -0.502 | -0.0654 | Yes |
| 38 | PLD3 |  |  | 20528 | -0.510 | 0.0037 | Yes |
Table: GSEA details [plain text format]

  

Fig 2: AGED\_MOUSE\_HYPOTH\_DN: Random ES distribution      
 Gene set null distribution of ES for **AGED\_MOUSE\_HYPOTH\_DN**

  
