# Supplementary material for: The rapamycin-regulated gene expression signature determines prognosis for breast cancer
Source: Mol Cancer. 2009 Sep 24;8:75. doi: 10.1186/1476-4598-8-75 (PMC2761377; doi:10.1186/1476-4598-8-75)
Supplement: Additional file 2 — Gene set enrichment analysis of in vivo data, time series. The data provided represent the time series of GSEA. This compressed file contains "Time" shortcut file and "GSEA_time" folder. Clicking on "Time" shortcut opens the index file providing access to analysis files contained in the "GSEA_time" folder. [file 1476-4598-8-75-S2.zip › GSEA_time/AGUIRRE_PANCREAS_CHR19.html]

Details for gene set AGUIRRE\_PANCREAS\_CHR19[GSEA]

|  || Dataset | gsea\_time\_collapsed |
| Phenotype | NoPhenotypeAvailable |
| Upregulated in class | na\_neg |
| GeneSet | AGUIRRE\_PANCREAS\_CHR19 |
| Enrichment Score (ES) | -0.32247052 |
| Normalized Enrichment Score (NES) | -1.3768523 |
| Nominal p-value | 0.025 |
| FDR q-value | 0.27938175 |
| FWER p-Value | 1.0 |
Table: GSEA Results Summary

  

Fig 1: Enrichment plot: AGUIRRE\_PANCREAS\_CHR19      
 Profile of the Running ES Score & Positions of GeneSet Members on the Rank Ordered List

  

| PROBE | GENE SYMBOL | GENE\_TITLE | RANK IN GENE LIST | RANK METRIC SCORE | RUNNING ES | CORE ENRICHMENT || 1 | ZNF264 |  |  | 694 | 0.475 | 0.0167 | No |
| 2 | ZNF573 |  |  | 1461 | 0.334 | 0.0149 | No |
| 3 | RPL13A |  |  | 1777 | 0.302 | 0.0316 | No |
| 4 | SNRPD2 |  |  | 2700 | 0.236 | 0.0118 | No |
| 5 | HNRPL |  |  | 2753 | 0.233 | 0.0340 | No |
| 6 | ZNF419A |  |  | 2860 | 0.227 | 0.0530 | No |
| 7 | RUVBL2 |  |  | 3020 | 0.218 | 0.0684 | No |
| 8 | MRPS12 |  |  | 3026 | 0.218 | 0.0914 | No |
| 9 | ZNF304 |  |  | 3147 | 0.211 | 0.1080 | No |
| 10 | ZNF444 |  |  | 3581 | 0.191 | 0.1072 | No |
| 11 | RPL18 |  |  | 3626 | 0.189 | 0.1252 | No |
| 12 | EPN1 |  |  | 3898 | 0.178 | 0.1309 | No |
| 13 | ZNF146 |  |  | 4400 | 0.157 | 0.1232 | No |
| 14 | CNOT3 |  |  | 4656 | 0.149 | 0.1266 | No |
| 15 | C5AR1 |  |  | 5084 | 0.135 | 0.1202 | No |
| 16 | PRMT1 |  |  | 5253 | 0.130 | 0.1259 | No |
| 17 | ZNF460 |  |  | 5779 | 0.117 | 0.1128 | No |
| 18 | RCN3 |  |  | 5942 | 0.114 | 0.1170 | No |
| 19 | EIF3S12 |  |  | 6274 | 0.106 | 0.1121 | No |
| 20 | GRLF1 |  |  | 7693 | 0.078 | 0.0513 | No |
| 21 | PRKD2 |  |  | 7964 | 0.073 | 0.0460 | No |
| 22 | OPA3 |  |  | 8576 | 0.063 | 0.0230 | No |
| 23 | SIX5 |  |  | 8629 | 0.063 | 0.0271 | No |
| 24 | ZNF331 |  |  | 8784 | 0.060 | 0.0260 | No |
| 25 | NAPA |  |  | 9182 | 0.054 | 0.0124 | No |
| 26 | KDELR1 |  |  | 9760 | 0.046 | -0.0108 | No |
| 27 | PRPF31 |  |  | 10039 | 0.041 | -0.0200 | No |
| 28 | AP2S1 |  |  | 10156 | 0.040 | -0.0214 | No |
| 29 | RPS9 |  |  | 10297 | 0.038 | -0.0242 | No |
| 30 | EML2 |  |  | 10845 | 0.029 | -0.0477 | No |
| 31 | PAK4 |  |  | 11334 | 0.023 | -0.0691 | No |
| 32 | DBP |  |  | 11398 | 0.022 | -0.0698 | No |
| 33 | ZNF211 |  |  | 11699 | 0.018 | -0.0825 | No |
| 34 | STRN4 |  |  | 11760 | 0.017 | -0.0836 | No |
| 35 | ZNF134 |  |  | 11991 | 0.014 | -0.0934 | No |
| 36 | SIRT2 |  |  | 12156 | 0.012 | -0.1001 | No |
| 37 | KLK13 |  |  | 12215 | 0.011 | -0.1018 | No |
| 38 | DMPK |  |  | 12508 | 0.006 | -0.1153 | No |
| 39 | CD3EAP |  |  | 12765 | 0.002 | -0.1276 | No |
| 40 | MAP4K1 |  |  | 13000 | -0.001 | -0.1388 | No |
| 41 | FUT1 |  |  | 13366 | -0.007 | -0.1559 | No |
| 42 | PPP5C |  |  | 13538 | -0.009 | -0.1632 | No |
| 43 | LHB |  |  | 13904 | -0.014 | -0.1795 | No |
| 44 | NCR1 |  |  | 13956 | -0.015 | -0.1803 | No |
| 45 | SNRP70 |  |  | 14299 | -0.021 | -0.1948 | No |
| 46 | ZDHHC24 |  |  | 14370 | -0.022 | -0.1959 | No |
| 47 | SYT5 |  |  | 14495 | -0.024 | -0.1994 | No |
| 48 | EHD2 |  |  | 14566 | -0.025 | -0.2002 | No |
| 49 | SARS2 |  |  | 14667 | -0.026 | -0.2023 | No |
| 50 | PAF1 |  |  | 14773 | -0.028 | -0.2044 | No |
| 51 | GIPR |  |  | 15103 | -0.033 | -0.2169 | No |
| 52 | LENG4 |  |  | 15477 | -0.039 | -0.2310 | No |
| 53 | U2AF2 |  |  | 16361 | -0.056 | -0.2680 | No |
| 54 | PPP1R13L |  |  | 16544 | -0.060 | -0.2705 | No |
| 55 | LOC126208 |  |  | 17294 | -0.077 | -0.2988 | No |
| 56 | FBXO17 |  |  | 17328 | -0.079 | -0.2920 | No |
| 57 | KIR2DL1 |  |  | 17752 | -0.091 | -0.3029 | No |
| 58 | NFKBIB |  |  | 17851 | -0.094 | -0.2977 | No |
| 59 | BAX |  |  | 18361 | -0.112 | -0.3106 | Yes |
| 60 | TSEN34 |  |  | 18447 | -0.115 | -0.3025 | Yes |
| 61 | NR1H2 |  |  | 18651 | -0.124 | -0.2992 | Yes |
| 62 | DKKL1 |  |  | 18673 | -0.125 | -0.2870 | Yes |
| 63 | NUCB1 |  |  | 19008 | -0.141 | -0.2882 | Yes |
| 64 | DPF1 |  |  | 19329 | -0.163 | -0.2866 | Yes |
| 65 | GRWD1 |  |  | 19478 | -0.176 | -0.2750 | Yes |
| 66 | NOSIP |  |  | 19545 | -0.183 | -0.2587 | Yes |
| 67 | LIG1 |  |  | 19746 | -0.205 | -0.2467 | Yes |
| 68 | PSMD8 |  |  | 19999 | -0.241 | -0.2334 | Yes |
| 69 | NUP62 |  |  | 20063 | -0.255 | -0.2093 | Yes |
| 70 | IRF3 |  |  | 20193 | -0.293 | -0.1844 | Yes |
| 71 | SLC1A5 |  |  | 20296 | -0.332 | -0.1541 | Yes |
| 72 | GYS1 |  |  | 20388 | -0.378 | -0.1184 | Yes |
| 73 | PLEKHA4 |  |  | 20399 | -0.382 | -0.0782 | Yes |
| 74 | ZNF580 |  |  | 20599 | -0.830 | 0.0003 | Yes |
Table: GSEA details [plain text format]

  

Fig 2: AGUIRRE\_PANCREAS\_CHR19: Random ES distribution      
 Gene set null distribution of ES for **AGUIRRE\_PANCREAS\_CHR19**

  
