# Supplementary material for: The rapamycin-regulated gene expression signature determines prognosis for breast cancer
Source: Mol Cancer. 2009 Sep 24;8:75. doi: 10.1186/1476-4598-8-75 (PMC2761377; doi:10.1186/1476-4598-8-75)
Supplement: Additional file 2 — Gene set enrichment analysis of in vivo data, time series. The data provided represent the time series of GSEA. This compressed file contains "Time" shortcut file and "GSEA_time" folder. Clicking on "Time" shortcut opens the index file providing access to analysis files contained in the "GSEA_time" folder. [file 1476-4598-8-75-S2.zip › GSEA_time/ALANINE_AND_ASPARTATE_METABOLISM.html]

Details for gene set ALANINE\_AND\_ASPARTATE\_METABOLISM[GSEA]

|  || Dataset | gsea\_time\_collapsed |
| Phenotype | NoPhenotypeAvailable |
| Upregulated in class | na\_neg |
| GeneSet | ALANINE\_AND\_ASPARTATE\_METABOLISM |
| Enrichment Score (ES) | -0.5127737 |
| Normalized Enrichment Score (NES) | -1.6933725 |
| Nominal p-value | 0.0058139535 |
| FDR q-value | 0.12569165 |
| FWER p-Value | 0.686 |
Table: GSEA Results Summary

  

Fig 1: Enrichment plot: ALANINE\_AND\_ASPARTATE\_METABOLISM      
 Profile of the Running ES Score & Positions of GeneSet Members on the Rank Ordered List

  

| PROBE | GENE SYMBOL | GENE\_TITLE | RANK IN GENE LIST | RANK METRIC SCORE | RUNNING ES | CORE ENRICHMENT || 1 | NARS |  |  | 1891 | 0.293 | 0.0388 | No |
| 2 | DDO |  |  | 4767 | 0.145 | -0.0361 | No |
| 3 | DARS |  |  | 5532 | 0.123 | -0.0184 | No |
| 4 | CRAT |  |  | 9301 | 0.052 | -0.1781 | No |
| 5 | ABAT |  |  | 10578 | 0.034 | -0.2251 | No |
| 6 | GPT |  |  | 10681 | 0.032 | -0.2159 | No |
| 7 | ASNS |  |  | 11419 | 0.022 | -0.2420 | No |
| 8 | ADSS |  |  | 11451 | 0.021 | -0.2341 | No |
| 9 | AGXT2 |  |  | 11739 | 0.017 | -0.2403 | No |
| 10 | GAD1 |  |  | 12033 | 0.013 | -0.2486 | No |
| 11 | GOT1 |  |  | 12172 | 0.012 | -0.2501 | No |
| 12 | ASPA |  |  | 12334 | 0.009 | -0.2539 | No |
| 13 | GAD2 |  |  | 13202 | -0.004 | -0.2941 | No |
| 14 | AGXT |  |  | 14000 | -0.016 | -0.3257 | No |
| 15 | CAD |  |  | 15550 | -0.040 | -0.3830 | No |
| 16 | ADSL |  |  | 17001 | -0.070 | -0.4223 | No |
| 17 | ASL |  |  | 18610 | -0.122 | -0.4460 | Yes |
| 18 | GOT2 |  |  | 19985 | -0.238 | -0.4067 | Yes |
| 19 | PC |  |  | 20000 | -0.241 | -0.3000 | Yes |
| 20 | GPT2 |  |  | 20293 | -0.330 | -0.1672 | Yes |
| 21 | AARS |  |  | 20430 | -0.409 | 0.0085 | Yes |
Table: GSEA details [plain text format]

  

Fig 2: ALANINE\_AND\_ASPARTATE\_METABOLISM: Random ES distribution      
 Gene set null distribution of ES for **ALANINE\_AND\_ASPARTATE\_METABOLISM**

  
