# Supplementary material for: The rapamycin-regulated gene expression signature determines prognosis for breast cancer
Source: Mol Cancer. 2009 Sep 24;8:75. doi: 10.1186/1476-4598-8-75 (PMC2761377; doi:10.1186/1476-4598-8-75)
Supplement: Additional file 2 — Gene set enrichment analysis of in vivo data, time series. The data provided represent the time series of GSEA. This compressed file contains "Time" shortcut file and "GSEA_time" folder. Clicking on "Time" shortcut opens the index file providing access to analysis files contained in the "GSEA_time" folder. [file 1476-4598-8-75-S2.zip › GSEA_time/AS3_FIBRO_DN.html]

Details for gene set AS3\_FIBRO\_DN[GSEA]

|  || Dataset | gsea\_time\_collapsed |
| Phenotype | NoPhenotypeAvailable |
| Upregulated in class | na\_pos |
| GeneSet | AS3\_FIBRO\_DN |
| Enrichment Score (ES) | 0.7077559 |
| Normalized Enrichment Score (NES) | 1.8302349 |
| Nominal p-value | 0.0 |
| FDR q-value | 0.0051166983 |
| FWER p-Value | 0.097 |
Table: GSEA Results Summary

  

Fig 1: Enrichment plot: AS3\_FIBRO\_DN      
 Profile of the Running ES Score & Positions of GeneSet Members on the Rank Ordered List

  

| PROBE | GENE SYMBOL | GENE\_TITLE | RANK IN GENE LIST | RANK METRIC SCORE | RUNNING ES | CORE ENRICHMENT || 1 | CCL2 |  |  | 11 | 1.629 | 0.1557 | Yes |
| 2 | IL8 |  |  | 13 | 1.561 | 0.3054 | Yes |
| 3 | EGFR |  |  | 42 | 1.173 | 0.4165 | Yes |
| 4 | CXCL2 |  |  | 203 | 0.723 | 0.4780 | Yes |
| 5 | PLAU |  |  | 302 | 0.647 | 0.5353 | Yes |
| 6 | ETV6 |  |  | 467 | 0.546 | 0.5797 | Yes |
| 7 | EIF4E |  |  | 1263 | 0.361 | 0.5756 | Yes |
| 8 | NCK1 |  |  | 1399 | 0.341 | 0.6017 | Yes |
| 9 | JUN |  |  | 1522 | 0.326 | 0.6271 | Yes |
| 10 | MYCN |  |  | 1784 | 0.301 | 0.6432 | Yes |
| 11 | F11R |  |  | 2357 | 0.258 | 0.6401 | Yes |
| 12 | CDC42 |  |  | 2404 | 0.255 | 0.6623 | Yes |
| 13 | NPAT |  |  | 2672 | 0.238 | 0.6721 | Yes |
| 14 | MAP3K8 |  |  | 2810 | 0.229 | 0.6875 | Yes |
| 15 | PGF |  |  | 2843 | 0.228 | 0.7078 | Yes |
| 16 | GRB2 |  |  | 3407 | 0.199 | 0.6995 | No |
| 17 | PML |  |  | 4606 | 0.151 | 0.6557 | No |
| 18 | CXCL1 |  |  | 5663 | 0.120 | 0.6159 | No |
| 19 | DARC |  |  | 6447 | 0.102 | 0.5877 | No |
| 20 | TNFAIP6 |  |  | 6873 | 0.094 | 0.5760 | No |
| 21 | EGR1 |  |  | 8018 | 0.072 | 0.5273 | No |
| 22 | CRADD |  |  | 9849 | 0.044 | 0.4426 | No |
| 23 | KRT8 |  |  | 10286 | 0.038 | 0.4250 | No |
| 24 | NR2F6 |  |  | 12512 | 0.006 | 0.3175 | No |
| 25 | JUNB |  |  | 13719 | -0.012 | 0.2600 | No |
| 26 | TAPBP |  |  | 16473 | -0.059 | 0.1318 | No |
| 27 | RAB3A |  |  | 17092 | -0.072 | 0.1087 | No |
| 28 | L1CAM |  |  | 18518 | -0.118 | 0.0507 | No |
| 29 | ELK1 |  |  | 18632 | -0.123 | 0.0570 | No |
| 30 | MAP2K1 |  |  | 19255 | -0.157 | 0.0418 | No |
| 31 | CRABP2 |  |  | 20027 | -0.248 | 0.0281 | No |
Table: GSEA details [plain text format]

  

Fig 2: AS3\_FIBRO\_DN: Random ES distribution      
 Gene set null distribution of ES for **AS3\_FIBRO\_DN**

  
