# Supplementary material for: The rapamycin-regulated gene expression signature determines prognosis for breast cancer
Source: Mol Cancer. 2009 Sep 24;8:75. doi: 10.1186/1476-4598-8-75 (PMC2761377; doi:10.1186/1476-4598-8-75)
Supplement: Additional file 2 — Gene set enrichment analysis of in vivo data, time series. The data provided represent the time series of GSEA. This compressed file contains "Time" shortcut file and "GSEA_time" folder. Clicking on "Time" shortcut opens the index file providing access to analysis files contained in the "GSEA_time" folder. [file 1476-4598-8-75-S2.zip › GSEA_time/ASTON_DEPRESSION_UP.html]

Details for gene set ASTON\_DEPRESSION\_UP[GSEA]

|  || Dataset | gsea\_time\_collapsed |
| Phenotype | NoPhenotypeAvailable |
| Upregulated in class | na\_pos |
| GeneSet | ASTON\_DEPRESSION\_UP |
| Enrichment Score (ES) | 0.67052335 |
| Normalized Enrichment Score (NES) | 1.8224299 |
| Nominal p-value | 0.0 |
| FDR q-value | 0.004805881 |
| FWER p-Value | 0.11 |
Table: GSEA Results Summary

  

Fig 1: Enrichment plot: ASTON\_DEPRESSION\_UP      
 Profile of the Running ES Score & Positions of GeneSet Members on the Rank Ordered List

  

| PROBE | GENE SYMBOL | GENE\_TITLE | RANK IN GENE LIST | RANK METRIC SCORE | RUNNING ES | CORE ENRICHMENT || 1 | IGFBP7 |  |  | 2 | 2.165 | 0.1845 | Yes |
| 2 | CALD1 |  |  | 72 | 0.980 | 0.2647 | Yes |
| 3 | SAMD4A |  |  | 298 | 0.650 | 0.3092 | Yes |
| 4 | TPM2 |  |  | 322 | 0.628 | 0.3616 | Yes |
| 5 | ZNF529 |  |  | 406 | 0.579 | 0.4069 | Yes |
| 6 | CUGBP2 |  |  | 577 | 0.506 | 0.4418 | Yes |
| 7 | PLAGL1 |  |  | 814 | 0.441 | 0.4679 | Yes |
| 8 | RAB2 |  |  | 821 | 0.441 | 0.5052 | Yes |
| 9 | SCP2 |  |  | 896 | 0.424 | 0.5378 | Yes |
| 10 | KIAA0828 |  |  | 1511 | 0.327 | 0.5358 | Yes |
| 11 | JUN |  |  | 1522 | 0.326 | 0.5631 | Yes |
| 12 | C20ORF111 |  |  | 1564 | 0.321 | 0.5885 | Yes |
| 13 | BCL11A |  |  | 1715 | 0.307 | 0.6074 | Yes |
| 14 | MTCP1 |  |  | 1795 | 0.300 | 0.6291 | Yes |
| 15 | DOPEY1 |  |  | 1945 | 0.289 | 0.6465 | Yes |
| 16 | ZNF423 |  |  | 1956 | 0.288 | 0.6705 | Yes |
| 17 | APC |  |  | 2450 | 0.251 | 0.6679 | No |
| 18 | REV3L |  |  | 3016 | 0.219 | 0.6591 | No |
| 19 | TSC1 |  |  | 3760 | 0.184 | 0.6387 | No |
| 20 | DGCR5 |  |  | 3815 | 0.182 | 0.6516 | No |
| 21 | ACD |  |  | 4487 | 0.155 | 0.6321 | No |
| 22 | LTBP1 |  |  | 4628 | 0.150 | 0.6381 | No |
| 23 | OSBPL2 |  |  | 4919 | 0.140 | 0.6359 | No |
| 24 | DLGAP1 |  |  | 5871 | 0.116 | 0.5995 | No |
| 25 | PPFIA4 |  |  | 5910 | 0.115 | 0.6075 | No |
| 26 | ANKRD6 |  |  | 6987 | 0.091 | 0.5629 | No |
| 27 | PTHR1 |  |  | 8030 | 0.072 | 0.5184 | No |
| 28 | CEP250 |  |  | 9151 | 0.055 | 0.4686 | No |
| 29 | DKFZP761N09121 |  |  | 9233 | 0.053 | 0.4692 | No |
| 30 | KAL1 |  |  | 9393 | 0.051 | 0.4658 | No |
| 31 | PENK |  |  | 10538 | 0.034 | 0.4131 | No |
| 32 | HEXA |  |  | 10641 | 0.033 | 0.4109 | No |
| 33 | LOC283824 |  |  | 11681 | 0.018 | 0.3619 | No |
| 34 | GREB1 |  |  | 11903 | 0.015 | 0.3525 | No |
| 35 | TTR |  |  | 11962 | 0.014 | 0.3509 | No |
| 36 | PTPRT |  |  | 12689 | 0.003 | 0.3158 | No |
| 37 | CCKBR |  |  | 12740 | 0.002 | 0.3136 | No |
| 38 | TBC1D1 |  |  | 13646 | -0.011 | 0.2705 | No |
| 39 | GSTM3 |  |  | 15317 | -0.037 | 0.1924 | No |
| 40 | TNFRSF25 |  |  | 15558 | -0.040 | 0.1842 | No |
| 41 | MN1 |  |  | 16342 | -0.056 | 0.1508 | No |
| 42 | COMP |  |  | 16365 | -0.056 | 0.1546 | No |
| 43 | DDX23 |  |  | 19513 | -0.180 | 0.0168 | No |
| 44 | MTHFD2 |  |  | 19664 | -0.196 | 0.0263 | No |
| 45 | CTNNAL1 |  |  | 19916 | -0.228 | 0.0335 | No |
Table: GSEA details [plain text format]

  

Fig 2: ASTON\_DEPRESSION\_UP: Random ES distribution      
 Gene set null distribution of ES for **ASTON\_DEPRESSION\_UP**

  
