# Supplementary material for: The rapamycin-regulated gene expression signature determines prognosis for breast cancer
Source: Mol Cancer. 2009 Sep 24;8:75. doi: 10.1186/1476-4598-8-75 (PMC2761377; doi:10.1186/1476-4598-8-75)
Supplement: Additional file 2 — Gene set enrichment analysis of in vivo data, time series. The data provided represent the time series of GSEA. This compressed file contains "Time" shortcut file and "GSEA_time" folder. Clicking on "Time" shortcut opens the index file providing access to analysis files contained in the "GSEA_time" folder. [file 1476-4598-8-75-S2.zip › GSEA_time/BAF57_BT549_DN.html]

Details for gene set BAF57\_BT549\_DN[GSEA]

|  || Dataset | gsea\_time\_collapsed |
| Phenotype | NoPhenotypeAvailable |
| Upregulated in class | na\_pos |
| GeneSet | BAF57\_BT549\_DN |
| Enrichment Score (ES) | 0.5676399 |
| Normalized Enrichment Score (NES) | 1.7439458 |
| Nominal p-value | 0.0 |
| FDR q-value | 0.012881016 |
| FWER p-Value | 0.426 |
Table: GSEA Results Summary

  

Fig 1: Enrichment plot: BAF57\_BT549\_DN      
 Profile of the Running ES Score & Positions of GeneSet Members on the Rank Ordered List

  

| PROBE | GENE SYMBOL | GENE\_TITLE | RANK IN GENE LIST | RANK METRIC SCORE | RUNNING ES | CORE ENRICHMENT || 1 | S100A2 |  |  | 4 | 2.096 | 0.0258 | Yes |
| 2 | TMEM47 |  |  | 7 | 1.842 | 0.0486 | Yes |
| 3 | FLJ10154 |  |  | 10 | 1.649 | 0.0689 | Yes |
| 4 | IL8 |  |  | 13 | 1.561 | 0.0882 | Yes |
| 5 | ADAMTS1 |  |  | 33 | 1.238 | 0.1027 | Yes |
| 6 | EGFR |  |  | 42 | 1.173 | 0.1168 | Yes |
| 7 | RP1-93H18.5 |  |  | 63 | 1.010 | 0.1284 | Yes |
| 8 | DUSP6 |  |  | 94 | 0.912 | 0.1382 | Yes |
| 9 | SOD2 |  |  | 128 | 0.857 | 0.1472 | Yes |
| 10 | ETS1 |  |  | 130 | 0.846 | 0.1577 | Yes |
| 11 | QDPR |  |  | 148 | 0.805 | 0.1668 | Yes |
| 12 | SLC39A8 |  |  | 153 | 0.797 | 0.1765 | Yes |
| 13 | TMEM46 |  |  | 154 | 0.796 | 0.1864 | Yes |
| 14 | CXCL3 |  |  | 162 | 0.784 | 0.1958 | Yes |
| 15 | SGK |  |  | 172 | 0.769 | 0.2049 | Yes |
| 16 | TM4SF1 |  |  | 183 | 0.754 | 0.2137 | Yes |
| 17 | CXCL2 |  |  | 203 | 0.723 | 0.2218 | Yes |
| 18 | CAV1 |  |  | 234 | 0.693 | 0.2289 | Yes |
| 19 | STC2 |  |  | 256 | 0.679 | 0.2363 | Yes |
| 20 | HNRPD |  |  | 281 | 0.666 | 0.2434 | Yes |
| 21 | GNG11 |  |  | 283 | 0.666 | 0.2516 | Yes |
| 22 | TRIM8 |  |  | 296 | 0.650 | 0.2591 | Yes |
| 23 | BMP2K |  |  | 316 | 0.634 | 0.2660 | Yes |
| 24 | LSM5 |  |  | 318 | 0.633 | 0.2738 | Yes |
| 25 | HK2 |  |  | 330 | 0.621 | 0.2810 | Yes |
| 26 | C6ORF111 |  |  | 348 | 0.608 | 0.2877 | Yes |
| 27 | DBNDD2 |  |  | 354 | 0.605 | 0.2949 | Yes |
| 28 | LOC92482 |  |  | 369 | 0.597 | 0.3017 | Yes |
| 29 | SMURF2 |  |  | 373 | 0.596 | 0.3089 | Yes |
| 30 | RASA1 |  |  | 384 | 0.593 | 0.3158 | Yes |
| 31 | MID1 |  |  | 400 | 0.582 | 0.3223 | Yes |
| 32 | PTGER4 |  |  | 453 | 0.552 | 0.3265 | Yes |
| 33 | LPHN2 |  |  | 554 | 0.514 | 0.3280 | Yes |
| 34 | TFPI2 |  |  | 563 | 0.511 | 0.3339 | Yes |
| 35 | MTAP |  |  | 569 | 0.509 | 0.3400 | Yes |
| 36 | ATRX |  |  | 590 | 0.503 | 0.3453 | Yes |
| 37 | TM4SF18 |  |  | 625 | 0.491 | 0.3497 | Yes |
| 38 | ELOVL6 |  |  | 627 | 0.491 | 0.3557 | Yes |
| 39 | KIAA1622 |  |  | 633 | 0.489 | 0.3615 | Yes |
| 40 | TNFRSF21 |  |  | 637 | 0.488 | 0.3675 | Yes |
| 41 | SFXN1 |  |  | 666 | 0.480 | 0.3720 | Yes |
| 42 | KIAA1794 |  |  | 681 | 0.477 | 0.3773 | Yes |
| 43 | LYPLA1 |  |  | 695 | 0.475 | 0.3825 | Yes |
| 44 | TBC1D4 |  |  | 697 | 0.475 | 0.3884 | Yes |
| 45 | LIF |  |  | 724 | 0.469 | 0.3929 | Yes |
| 46 | PWP1 |  |  | 736 | 0.466 | 0.3981 | Yes |
| 47 | HSPA1B |  |  | 777 | 0.454 | 0.4018 | Yes |
| 48 | HCCS |  |  | 810 | 0.443 | 0.4057 | Yes |
| 49 | TAP2 |  |  | 855 | 0.432 | 0.4089 | Yes |
| 50 | TPM1 |  |  | 870 | 0.428 | 0.4135 | Yes |
| 51 | SNRPA1 |  |  | 874 | 0.428 | 0.4187 | Yes |
| 52 | HS3ST1 |  |  | 875 | 0.427 | 0.4240 | Yes |
| 53 | C3ORF60 |  |  | 879 | 0.427 | 0.4291 | Yes |
| 54 | SRPK1 |  |  | 954 | 0.414 | 0.4306 | Yes |
| 55 | PTP4A1 |  |  | 988 | 0.408 | 0.4341 | Yes |
| 56 | DUSP4 |  |  | 1010 | 0.403 | 0.4380 | Yes |
| 57 | POU4F1 |  |  | 1034 | 0.400 | 0.4418 | Yes |
| 58 | PNN |  |  | 1068 | 0.393 | 0.4451 | Yes |
| 59 | PAQR3 |  |  | 1114 | 0.384 | 0.4476 | Yes |
| 60 | NT5E |  |  | 1124 | 0.381 | 0.4519 | Yes |
| 61 | DYNLT3 |  |  | 1135 | 0.380 | 0.4562 | Yes |
| 62 | SIX2 |  |  | 1147 | 0.378 | 0.4603 | Yes |
| 63 | TCERG1 |  |  | 1150 | 0.378 | 0.4649 | Yes |
| 64 | TWIST1 |  |  | 1175 | 0.375 | 0.4684 | Yes |
| 65 | PDLIM5 |  |  | 1228 | 0.365 | 0.4703 | Yes |
| 66 | EIF4E |  |  | 1263 | 0.361 | 0.4731 | Yes |
| 67 | MRPS17 |  |  | 1271 | 0.359 | 0.4773 | Yes |
| 68 | C9ORF85 |  |  | 1280 | 0.358 | 0.4813 | Yes |
| 69 | OSTM1 |  |  | 1294 | 0.355 | 0.4851 | Yes |
| 70 | NARG1 |  |  | 1323 | 0.351 | 0.4880 | Yes |
| 71 | LBR |  |  | 1467 | 0.334 | 0.4851 | Yes |
| 72 | AASDHPPT |  |  | 1570 | 0.321 | 0.4841 | Yes |
| 73 | SORBS2 |  |  | 1592 | 0.319 | 0.4870 | Yes |
| 74 | SLC1A6 |  |  | 1600 | 0.319 | 0.4906 | Yes |
| 75 | MEF2A |  |  | 1602 | 0.318 | 0.4945 | Yes |
| 76 | HIGD1A |  |  | 1685 | 0.310 | 0.4943 | Yes |
| 77 | HSPA4L |  |  | 1696 | 0.309 | 0.4977 | Yes |
| 78 | CDCP1 |  |  | 1705 | 0.308 | 0.5011 | Yes |
| 79 | PPA2 |  |  | 1740 | 0.305 | 0.5032 | Yes |
| 80 | CSAG2 |  |  | 1780 | 0.302 | 0.5050 | Yes |
| 81 | TLE4 |  |  | 1921 | 0.290 | 0.5017 | Yes |
| 82 | GRTP1 |  |  | 1923 | 0.290 | 0.5053 | Yes |
| 83 | IFT74 |  |  | 2018 | 0.283 | 0.5041 | Yes |
| 84 | C11ORF48 |  |  | 2019 | 0.282 | 0.5076 | Yes |
| 85 | ACOT9 |  |  | 2042 | 0.280 | 0.5100 | Yes |
| 86 | BNIP3 |  |  | 2049 | 0.280 | 0.5132 | Yes |
| 87 | EIF1AX |  |  | 2053 | 0.279 | 0.5165 | Yes |
| 88 | GALNT7 |  |  | 2137 | 0.274 | 0.5158 | Yes |
| 89 | DCK |  |  | 2156 | 0.272 | 0.5183 | Yes |
| 90 | PIGA |  |  | 2268 | 0.263 | 0.5161 | Yes |
| 91 | SLC29A1 |  |  | 2315 | 0.261 | 0.5171 | Yes |
| 92 | KYNU |  |  | 2325 | 0.260 | 0.5199 | Yes |
| 93 | B3GALNT1 |  |  | 2347 | 0.258 | 0.5220 | Yes |
| 94 | F11R |  |  | 2357 | 0.258 | 0.5248 | Yes |
| 95 | CDC42 |  |  | 2404 | 0.255 | 0.5257 | Yes |
| 96 | HSPA9B |  |  | 2458 | 0.251 | 0.5262 | Yes |
| 97 | RAGE |  |  | 2476 | 0.249 | 0.5284 | Yes |
| 98 | DNAJC15 |  |  | 2477 | 0.249 | 0.5315 | Yes |
| 99 | KIAA1598 |  |  | 2484 | 0.248 | 0.5343 | Yes |
| 100 | RNF138 |  |  | 2491 | 0.248 | 0.5371 | Yes |
| 101 | TNFRSF11B |  |  | 2516 | 0.247 | 0.5390 | Yes |
| 102 | PRDX2 |  |  | 2541 | 0.245 | 0.5408 | Yes |
| 103 | MAPK6 |  |  | 2603 | 0.241 | 0.5408 | Yes |
| 104 | SCML1 |  |  | 2614 | 0.241 | 0.5433 | Yes |
| 105 | SEPT9 |  |  | 2673 | 0.238 | 0.5434 | Yes |
| 106 | SMC3 |  |  | 2754 | 0.233 | 0.5423 | Yes |
| 107 | GTF2A2 |  |  | 2758 | 0.233 | 0.5451 | Yes |
| 108 | TPD52 |  |  | 2798 | 0.230 | 0.5460 | Yes |
| 109 | MAEA |  |  | 2895 | 0.225 | 0.5441 | Yes |
| 110 | VRK1 |  |  | 2931 | 0.223 | 0.5451 | Yes |
| 111 | IGF2BP3 |  |  | 3018 | 0.218 | 0.5436 | Yes |
| 112 | TACC2 |  |  | 3024 | 0.218 | 0.5460 | Yes |
| 113 | ING2 |  |  | 3041 | 0.217 | 0.5480 | Yes |
| 114 | EXOSC8 |  |  | 3126 | 0.213 | 0.5464 | Yes |
| 115 | DLEU2 |  |  | 3161 | 0.211 | 0.5474 | Yes |
| 116 | PBK |  |  | 3219 | 0.208 | 0.5472 | Yes |
| 117 | KIAA1797 |  |  | 3266 | 0.206 | 0.5474 | Yes |
| 118 | CSTF3 |  |  | 3293 | 0.204 | 0.5487 | Yes |
| 119 | RNF6 |  |  | 3306 | 0.204 | 0.5506 | Yes |
| 120 | CDC25A |  |  | 3425 | 0.199 | 0.5473 | Yes |
| 121 | SEMA3C |  |  | 3448 | 0.197 | 0.5486 | Yes |
| 122 | SMARCA5 |  |  | 3449 | 0.197 | 0.5511 | Yes |
| 123 | FBXO25 |  |  | 3549 | 0.192 | 0.5486 | Yes |
| 124 | TXNL4A |  |  | 3598 | 0.190 | 0.5486 | Yes |
| 125 | SPON2 |  |  | 3635 | 0.189 | 0.5492 | Yes |
| 126 | ARSJ |  |  | 3650 | 0.189 | 0.5508 | Yes |
| 127 | CAV2 |  |  | 3683 | 0.188 | 0.5515 | Yes |
| 128 | SACM1L |  |  | 3797 | 0.183 | 0.5482 | Yes |
| 129 | MRS2L |  |  | 3817 | 0.182 | 0.5496 | Yes |
| 130 | IL4R |  |  | 3899 | 0.178 | 0.5478 | Yes |
| 131 | C12ORF24 |  |  | 3933 | 0.176 | 0.5483 | Yes |
| 132 | H2AFY |  |  | 3943 | 0.176 | 0.5501 | Yes |
| 133 | MASA |  |  | 3966 | 0.175 | 0.5512 | Yes |
| 134 | PARP12 |  |  | 3981 | 0.174 | 0.5526 | Yes |
| 135 | AK2 |  |  | 3982 | 0.174 | 0.5548 | Yes |
| 136 | SCC-112 |  |  | 3984 | 0.174 | 0.5569 | Yes |
| 137 | NMT2 |  |  | 4000 | 0.174 | 0.5583 | Yes |
| 138 | VPS4B |  |  | 4017 | 0.173 | 0.5597 | Yes |
| 139 | PDLIM3 |  |  | 4032 | 0.172 | 0.5611 | Yes |
| 140 | DVL1 |  |  | 4056 | 0.171 | 0.5621 | Yes |
| 141 | DLAT |  |  | 4067 | 0.171 | 0.5637 | Yes |
| 142 | PRR6 |  |  | 4090 | 0.170 | 0.5648 | Yes |
| 143 | SACS |  |  | 4127 | 0.168 | 0.5651 | Yes |
| 144 | CTSC |  |  | 4134 | 0.167 | 0.5669 | Yes |
| 145 | CSE1L |  |  | 4195 | 0.165 | 0.5659 | Yes |
| 146 | MAGEA1 |  |  | 4203 | 0.164 | 0.5676 | Yes |
| 147 | SPCS3 |  |  | 4339 | 0.159 | 0.5630 | No |
| 148 | SLCO4A1 |  |  | 4434 | 0.157 | 0.5603 | No |
| 149 | PDCD5 |  |  | 4473 | 0.155 | 0.5603 | No |
| 150 | C1ORF41 |  |  | 4501 | 0.154 | 0.5609 | No |
| 151 | SYCP2 |  |  | 4554 | 0.152 | 0.5602 | No |
| 152 | IER5 |  |  | 4583 | 0.151 | 0.5607 | No |
| 153 | PTPLA |  |  | 4586 | 0.151 | 0.5625 | No |
| 154 | ZNF232 |  |  | 4673 | 0.148 | 0.5601 | No |
| 155 | GDF15 |  |  | 4826 | 0.143 | 0.5544 | No |
| 156 | DRAP1 |  |  | 5080 | 0.135 | 0.5436 | No |
| 157 | SFRP2 |  |  | 5182 | 0.132 | 0.5402 | No |
| 158 | C10ORF86 |  |  | 5200 | 0.132 | 0.5410 | No |
| 159 | BDKRB1 |  |  | 5312 | 0.129 | 0.5371 | No |
| 160 | OGFRL1 |  |  | 5319 | 0.128 | 0.5384 | No |
| 161 | CYP2R1 |  |  | 5461 | 0.125 | 0.5330 | No |
| 162 | GRPEL1 |  |  | 5533 | 0.123 | 0.5311 | No |
| 163 | CXCL1 |  |  | 5663 | 0.120 | 0.5262 | No |
| 164 | TRIM9 |  |  | 5664 | 0.120 | 0.5277 | No |
| 165 | TPD52L1 |  |  | 5715 | 0.119 | 0.5267 | No |
| 166 | FIP1L1 |  |  | 5756 | 0.118 | 0.5262 | No |
| 167 | TSPAN13 |  |  | 5795 | 0.117 | 0.5258 | No |
| 168 | MTERFD1 |  |  | 5804 | 0.117 | 0.5268 | No |
| 169 | PLCB4 |  |  | 5873 | 0.116 | 0.5249 | No |
| 170 | DENND2A |  |  | 5994 | 0.113 | 0.5204 | No |
| 171 | BDKRB2 |  |  | 6062 | 0.111 | 0.5185 | No |
| 172 | HADH |  |  | 6339 | 0.105 | 0.5061 | No |
| 173 | MYH10 |  |  | 6473 | 0.102 | 0.5009 | No |
| 174 | GLA |  |  | 6513 | 0.101 | 0.5002 | No |
| 175 | ZNF45 |  |  | 6519 | 0.101 | 0.5012 | No |
| 176 | B3GNT5 |  |  | 6620 | 0.099 | 0.4975 | No |
| 177 | NDUFV2 |  |  | 6742 | 0.097 | 0.4927 | No |
| 178 | NPTX1 |  |  | 6768 | 0.096 | 0.4927 | No |
| 179 | COX5A |  |  | 6819 | 0.095 | 0.4914 | No |
| 180 | SCG2 |  |  | 6840 | 0.094 | 0.4916 | No |
| 181 | IDH3A |  |  | 6869 | 0.094 | 0.4914 | No |
| 182 | TMEM33 |  |  | 6875 | 0.094 | 0.4923 | No |
| 183 | EIF4G3 |  |  | 6929 | 0.092 | 0.4908 | No |
| 184 | RRAS2 |  |  | 7000 | 0.091 | 0.4885 | No |
| 185 | FGF12 |  |  | 7035 | 0.090 | 0.4879 | No |
| 186 | AP2B1 |  |  | 7048 | 0.090 | 0.4885 | No |
| 187 | FLJ20105 |  |  | 7094 | 0.089 | 0.4874 | No |
| 188 | LOC129293 |  |  | 7110 | 0.089 | 0.4877 | No |
| 189 | CCNE1 |  |  | 7255 | 0.087 | 0.4817 | No |
| 190 | SUSD5 |  |  | 7421 | 0.083 | 0.4746 | No |
| 191 | NOLA1 |  |  | 7523 | 0.081 | 0.4706 | No |
| 192 | RGS20 |  |  | 7558 | 0.081 | 0.4700 | No |
| 193 | DOCK1 |  |  | 7581 | 0.080 | 0.4699 | No |
| 194 | ITGA10 |  |  | 7591 | 0.080 | 0.4704 | No |
| 195 | TMSB4Y |  |  | 7604 | 0.080 | 0.4708 | No |
| 196 | PCAF |  |  | 7876 | 0.075 | 0.4584 | No |
| 197 | PDCD2 |  |  | 8004 | 0.073 | 0.4530 | No |
| 198 | NRG1 |  |  | 8035 | 0.072 | 0.4524 | No |
| 199 | CAT |  |  | 8112 | 0.071 | 0.4496 | No |
| 200 | LOXL1 |  |  | 8180 | 0.070 | 0.4471 | No |
| 201 | SLC4A7 |  |  | 8221 | 0.069 | 0.4460 | No |
| 202 | PRG1 |  |  | 8317 | 0.067 | 0.4422 | No |
| 203 | WNT5B |  |  | 8360 | 0.067 | 0.4409 | No |
| 204 | BAALC |  |  | 8387 | 0.066 | 0.4405 | No |
| 205 | SUCLA2 |  |  | 8448 | 0.065 | 0.4383 | No |
| 206 | CD9 |  |  | 8510 | 0.064 | 0.4361 | No |
| 207 | DKFZP686O1327 |  |  | 8672 | 0.062 | 0.4289 | No |
| 208 | MAN1A1 |  |  | 8855 | 0.059 | 0.4207 | No |
| 209 | C4ORF22 |  |  | 8933 | 0.058 | 0.4176 | No |
| 210 | GLRX2 |  |  | 9054 | 0.056 | 0.4124 | No |
| 211 | BAMBI |  |  | 9107 | 0.055 | 0.4105 | No |
| 212 | HMGA1 |  |  | 9145 | 0.055 | 0.4094 | No |
| 213 | SDHB |  |  | 9306 | 0.052 | 0.4021 | No |
| 214 | ADAM12 |  |  | 9345 | 0.052 | 0.4009 | No |
| 215 | ZFPM2 |  |  | 9350 | 0.052 | 0.4013 | No |
| 216 | FLJ20186 |  |  | 9427 | 0.050 | 0.3982 | No |
| 217 | CXCL6 |  |  | 9436 | 0.050 | 0.3985 | No |
| 218 | RTEL1 |  |  | 9528 | 0.049 | 0.3946 | No |
| 219 | CASD1 |  |  | 9534 | 0.049 | 0.3949 | No |
| 220 | PSMB9 |  |  | 9544 | 0.049 | 0.3951 | No |
| 221 | RP2 |  |  | 9698 | 0.047 | 0.3881 | No |
| 222 | C15ORF48 |  |  | 9752 | 0.046 | 0.3861 | No |
| 223 | TRPC4 |  |  | 9768 | 0.046 | 0.3859 | No |
| 224 | OAS3 |  |  | 9921 | 0.043 | 0.3790 | No |
| 225 | TXNL2 |  |  | 10109 | 0.040 | 0.3702 | No |
| 226 | GNL3 |  |  | 10140 | 0.040 | 0.3693 | No |
| 227 | ATP5I |  |  | 10296 | 0.038 | 0.3621 | No |
| 228 | AFF3 |  |  | 10375 | 0.037 | 0.3587 | No |
| 229 | HPSE |  |  | 10443 | 0.036 | 0.3558 | No |
| 230 | LAMA4 |  |  | 10458 | 0.035 | 0.3556 | No |
| 231 | FRAP1 |  |  | 10544 | 0.034 | 0.3518 | No |
| 232 | ABCE1 |  |  | 10660 | 0.032 | 0.3465 | No |
| 233 | CYP1B1 |  |  | 10729 | 0.031 | 0.3436 | No |
| 234 | CITED2 |  |  | 10789 | 0.030 | 0.3410 | No |
| 235 | PRAME |  |  | 10866 | 0.029 | 0.3376 | No |
| 236 | UBE2V2 |  |  | 10927 | 0.028 | 0.3350 | No |
| 237 | MAGEA5 |  |  | 10983 | 0.027 | 0.3327 | No |
| 238 | HSPC111 |  |  | 11074 | 0.026 | 0.3286 | No |
| 239 | FADS2 |  |  | 11277 | 0.024 | 0.3189 | No |
| 240 | DIO3 |  |  | 11547 | 0.020 | 0.3059 | No |
| 241 | CYB5B |  |  | 11608 | 0.019 | 0.3031 | No |
| 242 | SHOC2 |  |  | 11644 | 0.019 | 0.3017 | No |
| 243 | BOLA2 |  |  | 12170 | 0.012 | 0.2759 | No |
| 244 | ETV3 |  |  | 12187 | 0.011 | 0.2753 | No |
| 245 | SDPR |  |  | 12330 | 0.009 | 0.2684 | No |
| 246 | E2F5 |  |  | 12422 | 0.008 | 0.2640 | No |
| 247 | PRR7 |  |  | 12535 | 0.006 | 0.2585 | No |
| 248 | TIAL1 |  |  | 12659 | 0.004 | 0.2525 | No |
| 249 | STAM |  |  | 12754 | 0.002 | 0.2479 | No |
| 250 | CASP7 |  |  | 13003 | -0.001 | 0.2357 | No |
| 251 | CXCL5 |  |  | 13115 | -0.003 | 0.2302 | No |
| 252 | ABI3BP |  |  | 13133 | -0.003 | 0.2294 | No |
| 253 | C4ORF18 |  |  | 13158 | -0.003 | 0.2283 | No |
| 254 | EPHA2 |  |  | 13256 | -0.005 | 0.2236 | No |
| 255 | KIAA0895 |  |  | 13525 | -0.009 | 0.2105 | No |
| 256 | ADAMTS2 |  |  | 13585 | -0.010 | 0.2077 | No |
| 257 | SPATA5L1 |  |  | 13601 | -0.010 | 0.2071 | No |
| 258 | RAB9 |  |  | 13947 | -0.015 | 0.1902 | No |
| 259 | DUSP9 |  |  | 14326 | -0.021 | 0.1719 | No |
| 260 | C11ORF24 |  |  | 14377 | -0.022 | 0.1697 | No |
| 261 | COX7B |  |  | 14618 | -0.025 | 0.1581 | No |
| 262 | CREB3 |  |  | 14973 | -0.031 | 0.1411 | No |
| 263 | FLJ20323 |  |  | 14980 | -0.031 | 0.1412 | No |
| 264 | BST1 |  |  | 15105 | -0.033 | 0.1355 | No |
| 265 | PRDX3 |  |  | 15215 | -0.035 | 0.1305 | No |
| 266 | FGF5 |  |  | 15342 | -0.037 | 0.1248 | No |
| 267 | RAMP1 |  |  | 15361 | -0.038 | 0.1243 | No |
| 268 | SLITRK5 |  |  | 15498 | -0.039 | 0.1181 | No |
| 269 | APIP |  |  | 15508 | -0.039 | 0.1182 | No |
| 270 | CTPS |  |  | 15695 | -0.043 | 0.1095 | No |
| 271 | FH |  |  | 15798 | -0.045 | 0.1050 | No |
| 272 | TNFSF7 |  |  | 16167 | -0.052 | 0.0875 | No |
| 273 | MAD2L1 |  |  | 16264 | -0.054 | 0.0835 | No |
| 274 | SLBP |  |  | 16438 | -0.058 | 0.0757 | No |
| 275 | LAP3 |  |  | 16470 | -0.058 | 0.0749 | No |
| 276 | NCAPG |  |  | 16553 | -0.060 | 0.0716 | No |
| 277 | UCHL1 |  |  | 16647 | -0.062 | 0.0677 | No |
| 278 | KBTBD11 |  |  | 16776 | -0.065 | 0.0622 | No |
| 279 | ZNF659 |  |  | 16844 | -0.066 | 0.0598 | No |
| 280 | MED28 |  |  | 16911 | -0.068 | 0.0573 | No |
| 281 | HMGCS1 |  |  | 16931 | -0.068 | 0.0572 | No |
| 282 | NUDC |  |  | 17185 | -0.075 | 0.0457 | No |
| 283 | RGS4 |  |  | 17417 | -0.081 | 0.0353 | No |
| 284 | PALMD |  |  | 17464 | -0.083 | 0.0341 | No |
| 285 | CACYBP |  |  | 17524 | -0.085 | 0.0322 | No |
| 286 | PLK2 |  |  | 17594 | -0.087 | 0.0299 | No |
| 287 | SLC16A2 |  |  | 17707 | -0.090 | 0.0255 | No |
| 288 | POP7 |  |  | 17758 | -0.091 | 0.0241 | No |
| 289 | FGF2 |  |  | 17847 | -0.094 | 0.0210 | No |
| 290 | MX2 |  |  | 17855 | -0.094 | 0.0218 | No |
| 291 | BST2 |  |  | 17902 | -0.096 | 0.0207 | No |
| 292 | CCR8 |  |  | 17926 | -0.097 | 0.0208 | No |
| 293 | MXRA5 |  |  | 17936 | -0.097 | 0.0215 | No |
| 294 | NOC2L |  |  | 17981 | -0.098 | 0.0206 | No |
| 295 | RSPO3 |  |  | 18186 | -0.105 | 0.0118 | No |
| 296 | TMEM14B |  |  | 18196 | -0.106 | 0.0127 | No |
| 297 | STEAP3 |  |  | 18241 | -0.108 | 0.0119 | No |
| 298 | IL13RA2 |  |  | 18264 | -0.109 | 0.0121 | No |
| 299 | TMEFF1 |  |  | 18345 | -0.111 | 0.0096 | No |
| 300 | PPIF |  |  | 18423 | -0.114 | 0.0072 | No |
| 301 | GALNT14 |  |  | 18463 | -0.116 | 0.0067 | No |
| 302 | CAND2 |  |  | 18473 | -0.116 | 0.0077 | No |
| 303 | GAL |  |  | 18547 | -0.120 | 0.0056 | No |
| 304 | NTN4 |  |  | 18657 | -0.124 | 0.0018 | No |
| 305 | MRPL13 |  |  | 18729 | -0.127 | -0.0002 | No |
| 306 | RP11-393H10.2 |  |  | 18765 | -0.129 | -0.0003 | No |
| 307 | TRIB1 |  |  | 18829 | -0.132 | -0.0018 | No |
| 308 | ALAS1 |  |  | 19244 | -0.156 | -0.0202 | No |
| 309 | ZNHIT1 |  |  | 19293 | -0.160 | -0.0206 | No |
| 310 | GTF2H2 |  |  | 19418 | -0.171 | -0.0246 | No |
| 311 | SMS |  |  | 19420 | -0.171 | -0.0225 | No |
| 312 | C1QBP |  |  | 19463 | -0.176 | -0.0224 | No |
| 313 | HDDC2 |  |  | 19519 | -0.180 | -0.0229 | No |
| 314 | C4ORF27 |  |  | 19525 | -0.181 | -0.0209 | No |
| 315 | S100A8 |  |  | 19583 | -0.187 | -0.0214 | No |
| 316 | PDHA1 |  |  | 19595 | -0.188 | -0.0196 | No |
| 317 | KNTC2 |  |  | 19779 | -0.209 | -0.0261 | No |
| 318 | IFI27 |  |  | 19905 | -0.226 | -0.0294 | No |
| 319 | AKR1B1 |  |  | 20185 | -0.290 | -0.0396 | No |
| 320 | MX1 |  |  | 20243 | -0.310 | -0.0385 | No |
| 321 | CSTB |  |  | 20247 | -0.312 | -0.0348 | No |
| 322 | FJX1 |  |  | 20285 | -0.325 | -0.0326 | No |
| 323 | HSPA4 |  |  | 20344 | -0.351 | -0.0311 | No |
| 324 | RNF182 |  |  | 20366 | -0.364 | -0.0276 | No |
| 325 | HSPA1A |  |  | 20462 | -0.438 | -0.0269 | No |
| 326 | NDUFAB1 |  |  | 20484 | -0.455 | -0.0223 | No |
| 327 | PSMB8 |  |  | 20531 | -0.514 | -0.0182 | No |
| 328 | SAC3D1 |  |  | 20541 | -0.528 | -0.0121 | No |
| 329 | LGALS3BP |  |  | 20546 | -0.534 | -0.0056 | No |
| 330 | HSPH1 |  |  | 20585 | -0.683 | 0.0010 | No |
Table: GSEA details [plain text format]

  

Fig 2: BAF57\_BT549\_DN: Random ES distribution      
 Gene set null distribution of ES for **BAF57\_BT549\_DN**

  
