# Supplementary material for: The rapamycin-regulated gene expression signature determines prognosis for breast cancer
Source: Mol Cancer. 2009 Sep 24;8:75. doi: 10.1186/1476-4598-8-75 (PMC2761377; doi:10.1186/1476-4598-8-75)
Supplement: Additional file 2 — Gene set enrichment analysis of in vivo data, time series. The data provided represent the time series of GSEA. This compressed file contains "Time" shortcut file and "GSEA_time" folder. Clicking on "Time" shortcut opens the index file providing access to analysis files contained in the "GSEA_time" folder. [file 1476-4598-8-75-S2.zip › GSEA_time/BCNU_GLIOMA_NOMGMT_48HRS_DN.html]

Details for gene set BCNU\_GLIOMA\_NOMGMT\_48HRS\_DN[GSEA]

|  || Dataset | gsea\_time\_collapsed |
| Phenotype | NoPhenotypeAvailable |
| Upregulated in class | na\_neg |
| GeneSet | BCNU\_GLIOMA\_NOMGMT\_48HRS\_DN |
| Enrichment Score (ES) | -0.6349945 |
| Normalized Enrichment Score (NES) | -2.2832623 |
| Nominal p-value | 0.0 |
| FDR q-value | 0.004775718 |
| FWER p-Value | 0.0050 |
Table: GSEA Results Summary

  

Fig 1: Enrichment plot: BCNU\_GLIOMA\_NOMGMT\_48HRS\_DN      
 Profile of the Running ES Score & Positions of GeneSet Members on the Rank Ordered List

  

| PROBE | GENE SYMBOL | GENE\_TITLE | RANK IN GENE LIST | RANK METRIC SCORE | RUNNING ES | CORE ENRICHMENT || 1 | FBN1 |  |  | 2330 | 0.260 | -0.0621 | No |
| 2 | KRT17 |  |  | 2702 | 0.236 | -0.0337 | No |
| 3 | PMS2L11 |  |  | 5677 | 0.120 | -0.1546 | No |
| 4 | FYN |  |  | 6142 | 0.110 | -0.1556 | No |
| 5 | LOXL1 |  |  | 8180 | 0.070 | -0.2408 | No |
| 6 | WASPIP |  |  | 8373 | 0.067 | -0.2370 | No |
| 7 | LRCH4 |  |  | 8621 | 0.063 | -0.2367 | No |
| 8 | ACTN1 |  |  | 9090 | 0.055 | -0.2485 | No |
| 9 | ATP1A3 |  |  | 10373 | 0.037 | -0.3037 | No |
| 10 | HMGN2 |  |  | 10507 | 0.035 | -0.3033 | No |
| 11 | ADRA1B |  |  | 11261 | 0.024 | -0.3352 | No |
| 12 | IRS1 |  |  | 12296 | 0.010 | -0.3835 | No |
| 13 | PFN1 |  |  | 12735 | 0.002 | -0.4043 | No |
| 14 | INSL3 |  |  | 14938 | -0.031 | -0.5053 | No |
| 15 | SHOX |  |  | 15081 | -0.033 | -0.5057 | No |
| 16 | ADCYAP1 |  |  | 16208 | -0.053 | -0.5500 | No |
| 17 | INPPL1 |  |  | 17603 | -0.087 | -0.6006 | No |
| 18 | AMH |  |  | 18302 | -0.110 | -0.6129 | Yes |
| 19 | ITGA3 |  |  | 18758 | -0.129 | -0.6096 | Yes |
| 20 | MAP4K2 |  |  | 18761 | -0.129 | -0.5843 | Yes |
| 21 | GTF2F1 |  |  | 18870 | -0.135 | -0.5630 | Yes |
| 22 | AQP8 |  |  | 18888 | -0.135 | -0.5372 | Yes |
| 23 | INSIG1 |  |  | 19038 | -0.143 | -0.5163 | Yes |
| 24 | ACTC1 |  |  | 19679 | -0.197 | -0.5086 | Yes |
| 25 | TUBG1 |  |  | 19755 | -0.206 | -0.4717 | Yes |
| 26 | YWHAH |  |  | 20007 | -0.242 | -0.4361 | Yes |
| 27 | PPM1G |  |  | 20235 | -0.307 | -0.3866 | Yes |
| 28 | NRTN |  |  | 20413 | -0.395 | -0.3174 | Yes |
| 29 | ATP5D |  |  | 20437 | -0.416 | -0.2366 | Yes |
| 30 | EPHX1 |  |  | 20521 | -0.501 | -0.1420 | Yes |
| 31 | MDK |  |  | 20591 | -0.741 | 0.0007 | Yes |
Table: GSEA details [plain text format]

  

Fig 2: BCNU\_GLIOMA\_NOMGMT\_48HRS\_DN: Random ES distribution      
 Gene set null distribution of ES for **BCNU\_GLIOMA\_NOMGMT\_48HRS\_DN**

  
