# Supplementary material for: The rapamycin-regulated gene expression signature determines prognosis for breast cancer
Source: Mol Cancer. 2009 Sep 24;8:75. doi: 10.1186/1476-4598-8-75 (PMC2761377; doi:10.1186/1476-4598-8-75)
Supplement: Additional file 2 — Gene set enrichment analysis of in vivo data, time series. The data provided represent the time series of GSEA. This compressed file contains "Time" shortcut file and "GSEA_time" folder. Clicking on "Time" shortcut opens the index file providing access to analysis files contained in the "GSEA_time" folder. [file 1476-4598-8-75-S2.zip › GSEA_time/BECKER_TAMOXIFEN_RESISTANT_UP.html]

Details for gene set BECKER\_TAMOXIFEN\_RESISTANT\_UP[GSEA]

|  || Dataset | gsea\_time\_collapsed |
| Phenotype | NoPhenotypeAvailable |
| Upregulated in class | na\_neg |
| GeneSet | BECKER\_TAMOXIFEN\_RESISTANT\_UP |
| Enrichment Score (ES) | -0.40170345 |
| Normalized Enrichment Score (NES) | -1.5244572 |
| Nominal p-value | 0.04597701 |
| FDR q-value | 0.21530129 |
| FWER p-Value | 0.969 |
Table: GSEA Results Summary

  

Fig 1: Enrichment plot: BECKER\_TAMOXIFEN\_RESISTANT\_UP      
 Profile of the Running ES Score & Positions of GeneSet Members on the Rank Ordered List

  

| PROBE | GENE SYMBOL | GENE\_TITLE | RANK IN GENE LIST | RANK METRIC SCORE | RUNNING ES | CORE ENRICHMENT || 1 | TAGLN |  |  | 95 | 0.911 | 0.1119 | No |
| 2 | EBAG9 |  |  | 1320 | 0.351 | 0.0973 | No |
| 3 | SEPP1 |  |  | 1538 | 0.325 | 0.1282 | No |
| 4 | CXADR |  |  | 2770 | 0.232 | 0.0980 | No |
| 5 | CPE |  |  | 2813 | 0.229 | 0.1253 | No |
| 6 | DECR1 |  |  | 3765 | 0.184 | 0.1025 | No |
| 7 | PPP2R2A |  |  | 3811 | 0.182 | 0.1236 | No |
| 8 | GAGE1 /// GAGE2 /// |  |  | 3855 | 0.180 | 0.1445 | No |
| 9 | SNX1 |  |  | 4187 | 0.165 | 0.1496 | No |
| 10 | ACTA1 |  |  | 4631 | 0.149 | 0.1471 | No |
| 11 | PROCR |  |  | 4716 | 0.147 | 0.1618 | No |
| 12 | RNASE4 |  |  | 5292 | 0.129 | 0.1504 | No |
| 13 | BCAS1 |  |  | 5603 | 0.122 | 0.1509 | No |
| 14 | B2M |  |  | 5723 | 0.119 | 0.1603 | No |
| 15 | SCGB2A2 |  |  | 5762 | 0.118 | 0.1735 | No |
| 16 | BTG3 |  |  | 6287 | 0.106 | 0.1616 | No |
| 17 | PTK6 |  |  | 7020 | 0.091 | 0.1376 | No |
| 18 | ARL4A |  |  | 7595 | 0.080 | 0.1200 | No |
| 19 | NMI |  |  | 8892 | 0.058 | 0.0644 | No |
| 20 | CRABP1 |  |  | 11048 | 0.027 | -0.0370 | No |
| 21 | NMT1 |  |  | 11087 | 0.026 | -0.0354 | No |
| 22 | CACNB3 |  |  | 12014 | 0.014 | -0.0787 | No |
| 23 | TSPAN3 |  |  | 12944 | -0.000 | -0.1238 | No |
| 24 | TUSC3 |  |  | 13669 | -0.011 | -0.1576 | No |
| 25 | GTF2E2 |  |  | 13967 | -0.015 | -0.1700 | No |
| 26 | NNAT |  |  | 14865 | -0.030 | -0.2098 | No |
| 27 | ISGF3G |  |  | 15927 | -0.047 | -0.2554 | No |
| 28 | UGT2B15 |  |  | 16102 | -0.050 | -0.2575 | No |
| 29 | IFI44 |  |  | 16912 | -0.068 | -0.2881 | No |
| 30 | BST2 |  |  | 17902 | -0.096 | -0.3240 | No |
| 31 | E2F1 |  |  | 18810 | -0.131 | -0.3513 | No |
| 32 | IFI6 |  |  | 19848 | -0.218 | -0.3738 | Yes |
| 33 | IFIT1 |  |  | 19898 | -0.225 | -0.3474 | Yes |
| 34 | SEPHS2 |  |  | 19963 | -0.234 | -0.3206 | Yes |
| 35 | ID1 |  |  | 20093 | -0.262 | -0.2934 | Yes |
| 36 | ISG15 |  |  | 20207 | -0.298 | -0.2608 | Yes |
| 37 | EPHX1 |  |  | 20521 | -0.501 | -0.2120 | Yes |
| 38 | ID3 |  |  | 20523 | -0.502 | -0.1479 | Yes |
| 39 | LGALS3BP |  |  | 20546 | -0.534 | -0.0806 | Yes |
| 40 | PITX1 |  |  | 20578 | -0.652 | 0.0013 | Yes |
Table: GSEA details [plain text format]

  

Fig 2: BECKER\_TAMOXIFEN\_RESISTANT\_UP: Random ES distribution      
 Gene set null distribution of ES for **BECKER\_TAMOXIFEN\_RESISTANT\_UP**

  
