# Supplementary material for: The rapamycin-regulated gene expression signature determines prognosis for breast cancer
Source: Mol Cancer. 2009 Sep 24;8:75. doi: 10.1186/1476-4598-8-75 (PMC2761377; doi:10.1186/1476-4598-8-75)
Supplement: Additional file 2 — Gene set enrichment analysis of in vivo data, time series. The data provided represent the time series of GSEA. This compressed file contains "Time" shortcut file and "GSEA_time" folder. Clicking on "Time" shortcut opens the index file providing access to analysis files contained in the "GSEA_time" folder. [file 1476-4598-8-75-S2.zip › GSEA_time/BENNETT_SLE_UP.html]

Details for gene set BENNETT\_SLE\_UP[GSEA]

|  || Dataset | gsea\_time\_collapsed |
| Phenotype | NoPhenotypeAvailable |
| Upregulated in class | na\_neg |
| GeneSet | BENNETT\_SLE\_UP |
| Enrichment Score (ES) | -0.41311675 |
| Normalized Enrichment Score (NES) | -1.4426934 |
| Nominal p-value | 0.023255814 |
| FDR q-value | 0.26405728 |
| FWER p-Value | 0.995 |
Table: GSEA Results Summary

  

Fig 1: Enrichment plot: BENNETT\_SLE\_UP      
 Profile of the Running ES Score & Positions of GeneSet Members on the Rank Ordered List

  

| PROBE | GENE SYMBOL | GENE\_TITLE | RANK IN GENE LIST | RANK METRIC SCORE | RUNNING ES | CORE ENRICHMENT || 1 | CKAP4 |  |  | 121 | 0.869 | 0.1634 | No |
| 2 | APOBEC3C |  |  | 1099 | 0.387 | 0.1914 | No |
| 3 | PLSCR1 |  |  | 1622 | 0.315 | 0.2275 | No |
| 4 | STAT1 |  |  | 4141 | 0.167 | 0.1377 | No |
| 5 | C2 |  |  | 4885 | 0.141 | 0.1291 | No |
| 6 | OAS2 |  |  | 5619 | 0.121 | 0.1171 | No |
| 7 | FPRL1 |  |  | 6723 | 0.097 | 0.0824 | No |
| 8 | BIRC4BP |  |  | 9494 | 0.050 | -0.0425 | No |
| 9 | OASL |  |  | 10812 | 0.030 | -0.1006 | No |
| 10 | SERPING1 |  |  | 12583 | 0.005 | -0.1856 | No |
| 11 | TDRD7 |  |  | 13023 | -0.001 | -0.2067 | No |
| 12 | AGRIN |  |  | 13257 | -0.005 | -0.2171 | No |
| 13 | TNFSF10 |  |  | 14451 | -0.023 | -0.2706 | No |
| 14 | IFI35 |  |  | 15152 | -0.034 | -0.2980 | No |
| 15 | IFIT3 |  |  | 15416 | -0.038 | -0.3033 | No |
| 16 | IFITM3 |  |  | 15704 | -0.043 | -0.3088 | No |
| 17 | CAMP |  |  | 17785 | -0.092 | -0.3920 | Yes |
| 18 | RNASE2 |  |  | 17833 | -0.094 | -0.3760 | Yes |
| 19 | MX2 |  |  | 17855 | -0.094 | -0.3586 | Yes |
| 20 | TAP1 |  |  | 18553 | -0.120 | -0.3692 | Yes |
| 21 | IFI44L |  |  | 19458 | -0.175 | -0.3790 | Yes |
| 22 | IRF7 |  |  | 19564 | -0.185 | -0.3480 | Yes |
| 23 | S100A8 |  |  | 19583 | -0.187 | -0.3125 | Yes |
| 24 | ISG15 |  |  | 20207 | -0.298 | -0.2846 | Yes |
| 25 | MX1 |  |  | 20243 | -0.310 | -0.2259 | Yes |
| 26 | LY6E |  |  | 20336 | -0.349 | -0.1623 | Yes |
| 27 | OAS1 |  |  | 20370 | -0.365 | -0.0927 | Yes |
| 28 | LGALS3BP |  |  | 20546 | -0.534 | 0.0029 | Yes |
Table: GSEA details [plain text format]

  

Fig 2: BENNETT\_SLE\_UP: Random ES distribution      
 Gene set null distribution of ES for **BENNETT\_SLE\_UP**

  
