# Supplementary material for: The rapamycin-regulated gene expression signature determines prognosis for breast cancer
Source: Mol Cancer. 2009 Sep 24;8:75. doi: 10.1186/1476-4598-8-75 (PMC2761377; doi:10.1186/1476-4598-8-75)
Supplement: Additional file 2 — Gene set enrichment analysis of in vivo data, time series. The data provided represent the time series of GSEA. This compressed file contains "Time" shortcut file and "GSEA_time" folder. Clicking on "Time" shortcut opens the index file providing access to analysis files contained in the "GSEA_time" folder. [file 1476-4598-8-75-S2.zip › GSEA_time/BILE_ACID_BIOSYNTHESIS.html]

Details for gene set BILE\_ACID\_BIOSYNTHESIS[GSEA]

|  || Dataset | gsea\_time\_collapsed |
| Phenotype | NoPhenotypeAvailable |
| Upregulated in class | na\_neg |
| GeneSet | BILE\_ACID\_BIOSYNTHESIS |
| Enrichment Score (ES) | -0.36418253 |
| Normalized Enrichment Score (NES) | -1.2408197 |
| Nominal p-value | 0.176 |
| FDR q-value | 0.3770659 |
| FWER p-Value | 1.0 |
Table: GSEA Results Summary

  

Fig 1: Enrichment plot: BILE\_ACID\_BIOSYNTHESIS      
 Profile of the Running ES Score & Positions of GeneSet Members on the Rank Ordered List

  

| PROBE | GENE SYMBOL | GENE\_TITLE | RANK IN GENE LIST | RANK METRIC SCORE | RUNNING ES | CORE ENRICHMENT || 1 | SRD5A1 |  |  | 342 | 0.611 | 0.1671 | No |
| 2 | ALDH1A3 |  |  | 3109 | 0.214 | 0.0968 | No |
| 3 | ACAA2 |  |  | 3822 | 0.182 | 0.1168 | No |
| 4 | ALDH1B1 |  |  | 4736 | 0.146 | 0.1164 | No |
| 5 | ADH4 |  |  | 7650 | 0.079 | -0.0013 | No |
| 6 | ALDH1A2 |  |  | 7879 | 0.075 | 0.0100 | No |
| 7 | CYP27A1 |  |  | 12343 | 0.009 | -0.2043 | No |
| 8 | ADH1B |  |  | 13211 | -0.004 | -0.2451 | No |
| 9 | HADHB |  |  | 14081 | -0.017 | -0.2821 | No |
| 10 | ADH6 |  |  | 14305 | -0.021 | -0.2867 | No |
| 11 | ALDH2 |  |  | 14955 | -0.031 | -0.3090 | No |
| 12 | ACAA1 |  |  | 16091 | -0.050 | -0.3492 | Yes |
| 13 | ADH7 |  |  | 16133 | -0.051 | -0.3358 | Yes |
| 14 | SRD5A2 |  |  | 16235 | -0.053 | -0.3247 | Yes |
| 15 | ALDH3A1 |  |  | 16424 | -0.057 | -0.3166 | Yes |
| 16 | ADHFE1 |  |  | 16524 | -0.060 | -0.3035 | Yes |
| 17 | ADH1C |  |  | 16543 | -0.060 | -0.2863 | Yes |
| 18 | BAAT |  |  | 17269 | -0.077 | -0.2984 | Yes |
| 19 | AKR1C4 |  |  | 17475 | -0.083 | -0.2834 | Yes |
| 20 | ADH1A |  |  | 18090 | -0.101 | -0.2828 | Yes |
| 21 | ALDH3A2 |  |  | 18618 | -0.123 | -0.2715 | Yes |
| 22 | CYP7A1 |  |  | 18949 | -0.138 | -0.2459 | Yes |
| 23 | CEL |  |  | 19134 | -0.149 | -0.2101 | Yes |
| 24 | AKR1D1 |  |  | 19371 | -0.167 | -0.1714 | Yes |
| 25 | ALDH1A1 |  |  | 19486 | -0.177 | -0.1238 | Yes |
| 26 | SOAT2 |  |  | 19932 | -0.229 | -0.0764 | Yes |
| 27 | ALDH9A1 |  |  | 20364 | -0.363 | 0.0117 | Yes |
Table: GSEA details [plain text format]

  

Fig 2: BILE\_ACID\_BIOSYNTHESIS: Random ES distribution      
 Gene set null distribution of ES for **BILE\_ACID\_BIOSYNTHESIS**

  
