# Supplementary material for: The rapamycin-regulated gene expression signature determines prognosis for breast cancer
Source: Mol Cancer. 2009 Sep 24;8:75. doi: 10.1186/1476-4598-8-75 (PMC2761377; doi:10.1186/1476-4598-8-75)
Supplement: Additional file 2 — Gene set enrichment analysis of in vivo data, time series. The data provided represent the time series of GSEA. This compressed file contains "Time" shortcut file and "GSEA_time" folder. Clicking on "Time" shortcut opens the index file providing access to analysis files contained in the "GSEA_time" folder. [file 1476-4598-8-75-S2.zip › GSEA_time/BRCA1_SW480_UP.html]

Details for gene set BRCA1\_SW480\_UP[GSEA]

|  || Dataset | gsea\_time\_collapsed |
| Phenotype | NoPhenotypeAvailable |
| Upregulated in class | na\_neg |
| GeneSet | BRCA1\_SW480\_UP |
| Enrichment Score (ES) | -0.39352417 |
| Normalized Enrichment Score (NES) | -1.3088738 |
| Nominal p-value | 0.13125 |
| FDR q-value | 0.3188774 |
| FWER p-Value | 1.0 |
Table: GSEA Results Summary

  

Fig 1: Enrichment plot: BRCA1\_SW480\_UP      
 Profile of the Running ES Score & Positions of GeneSet Members on the Rank Ordered List

  

| PROBE | GENE SYMBOL | GENE\_TITLE | RANK IN GENE LIST | RANK METRIC SCORE | RUNNING ES | CORE ENRICHMENT || 1 | MET |  |  | 171 | 0.770 | 0.1509 | No |
| 2 | UBE2B |  |  | 276 | 0.669 | 0.2842 | No |
| 3 | RAC1 |  |  | 2228 | 0.266 | 0.2445 | No |
| 4 | TOP2A |  |  | 3342 | 0.202 | 0.2322 | No |
| 5 | TOP1 |  |  | 4714 | 0.147 | 0.1959 | No |
| 6 | XRCC6 |  |  | 6602 | 0.100 | 0.1248 | No |
| 7 | MAPRE2 |  |  | 7675 | 0.078 | 0.0889 | No |
| 8 | LFNG |  |  | 8386 | 0.066 | 0.0681 | No |
| 9 | GADD45A |  |  | 8579 | 0.063 | 0.0719 | No |
| 10 | KRT2 |  |  | 10256 | 0.038 | -0.0017 | No |
| 11 | CDC34 |  |  | 10411 | 0.036 | -0.0017 | No |
| 12 | RHOA |  |  | 10990 | 0.027 | -0.0241 | No |
| 13 | CTNNA1 |  |  | 11833 | 0.016 | -0.0616 | No |
| 14 | DDIT3 |  |  | 11873 | 0.015 | -0.0603 | No |
| 15 | PCNA |  |  | 12857 | 0.001 | -0.1079 | No |
| 16 | CDKN1A |  |  | 12863 | 0.001 | -0.1080 | No |
| 17 | TIMP2 |  |  | 13940 | -0.015 | -0.1572 | No |
| 18 | ARHGDIA |  |  | 14579 | -0.025 | -0.1831 | No |
| 19 | PCTK1 |  |  | 15235 | -0.035 | -0.2076 | No |
| 20 | GAPDH |  |  | 16748 | -0.064 | -0.2678 | No |
| 21 | IGFBP4 |  |  | 17975 | -0.098 | -0.3072 | No |
| 22 | SOD1 |  |  | 19187 | -0.152 | -0.3345 | Yes |
| 23 | CDK4 |  |  | 20403 | -0.387 | -0.3136 | Yes |
| 24 | TIMP1 |  |  | 20516 | -0.498 | -0.2161 | Yes |
| 25 | IGFBP2 |  |  | 20605 | -1.066 | -0.0000 | Yes |
Table: GSEA details [plain text format]

  

Fig 2: BRCA1\_SW480\_UP: Random ES distribution      
 Gene set null distribution of ES for **BRCA1\_SW480\_UP**

  
