# Supplementary material for: The rapamycin-regulated gene expression signature determines prognosis for breast cancer
Source: Mol Cancer. 2009 Sep 24;8:75. doi: 10.1186/1476-4598-8-75 (PMC2761377; doi:10.1186/1476-4598-8-75)
Supplement: Additional file 2 — Gene set enrichment analysis of in vivo data, time series. The data provided represent the time series of GSEA. This compressed file contains "Time" shortcut file and "GSEA_time" folder. Clicking on "Time" shortcut opens the index file providing access to analysis files contained in the "GSEA_time" folder. [file 1476-4598-8-75-S2.zip › GSEA_time/BRENTANI_REPAIR.html]

Details for gene set BRENTANI\_REPAIR[GSEA]

|  || Dataset | gsea\_time\_collapsed |
| Phenotype | NoPhenotypeAvailable |
| Upregulated in class | na\_neg |
| GeneSet | BRENTANI\_REPAIR |
| Enrichment Score (ES) | -0.34877342 |
| Normalized Enrichment Score (NES) | -1.3142688 |
| Nominal p-value | 0.0927835 |
| FDR q-value | 0.3186843 |
| FWER p-Value | 1.0 |
Table: GSEA Results Summary

  

Fig 1: Enrichment plot: BRENTANI\_REPAIR      
 Profile of the Running ES Score & Positions of GeneSet Members on the Rank Ordered List

  

| PROBE | GENE SYMBOL | GENE\_TITLE | RANK IN GENE LIST | RANK METRIC SCORE | RUNNING ES | CORE ENRICHMENT || 1 | ERCC1 |  |  | 829 | 0.438 | 0.0420 | No |
| 2 | MSH3 |  |  | 1620 | 0.316 | 0.0629 | No |
| 3 | RAD1 |  |  | 3299 | 0.204 | 0.0196 | No |
| 4 | RAD50 |  |  | 3725 | 0.186 | 0.0338 | No |
| 5 | MLH1 |  |  | 3880 | 0.179 | 0.0599 | No |
| 6 | RAD23B |  |  | 4243 | 0.163 | 0.0729 | No |
| 7 | BRCA2 |  |  | 4464 | 0.156 | 0.0915 | No |
| 8 | DDB2 |  |  | 4551 | 0.152 | 0.1160 | No |
| 9 | PMS1 |  |  | 4570 | 0.151 | 0.1435 | No |
| 10 | ERCC5 |  |  | 4609 | 0.150 | 0.1699 | No |
| 11 | RAD51L1 |  |  | 4686 | 0.148 | 0.1939 | No |
| 12 | RAD51 |  |  | 5681 | 0.120 | 0.1681 | No |
| 13 | RAD17 |  |  | 6048 | 0.112 | 0.1712 | No |
| 14 | XRCC5 |  |  | 7454 | 0.083 | 0.1185 | No |
| 15 | OGG1 |  |  | 8987 | 0.057 | 0.0547 | No |
| 16 | MSH6 |  |  | 9065 | 0.056 | 0.0615 | No |
| 17 | RAD52 |  |  | 10387 | 0.037 | 0.0041 | No |
| 18 | FRAP1 |  |  | 10544 | 0.034 | 0.0029 | No |
| 19 | CHEK2 |  |  | 11466 | 0.021 | -0.0379 | No |
| 20 | ERCC3 |  |  | 11610 | 0.019 | -0.0412 | No |
| 21 | BRCA1 |  |  | 12699 | 0.003 | -0.0935 | No |
| 22 | PCNA |  |  | 12857 | 0.001 | -0.1009 | No |
| 23 | XPA |  |  | 13632 | -0.011 | -0.1366 | No |
| 24 | RAD54L |  |  | 15238 | -0.035 | -0.2080 | No |
| 25 | XRCC3 |  |  | 15266 | -0.036 | -0.2025 | No |
| 26 | MSH2 |  |  | 17995 | -0.099 | -0.3166 | No |
| 27 | APEX1 |  |  | 18220 | -0.106 | -0.3076 | No |
| 28 | RAD9A |  |  | 19069 | -0.145 | -0.3216 | Yes |
| 29 | FEN1 |  |  | 19563 | -0.185 | -0.3108 | Yes |
| 30 | ERCC4 |  |  | 19703 | -0.201 | -0.2799 | Yes |
| 31 | EXO1 |  |  | 19934 | -0.230 | -0.2479 | Yes |
| 32 | XPC |  |  | 19943 | -0.231 | -0.2049 | Yes |
| 33 | RAD23A |  |  | 20094 | -0.262 | -0.1631 | Yes |
| 34 | DDB1 |  |  | 20231 | -0.305 | -0.1124 | Yes |
| 35 | POLD1 |  |  | 20588 | -0.695 | 0.0008 | Yes |
Table: GSEA details [plain text format]

  

Fig 2: BRENTANI\_REPAIR: Random ES distribution      
 Gene set null distribution of ES for **BRENTANI\_REPAIR**

  
