# Supplementary material for: The rapamycin-regulated gene expression signature determines prognosis for breast cancer
Source: Mol Cancer. 2009 Sep 24;8:75. doi: 10.1186/1476-4598-8-75 (PMC2761377; doi:10.1186/1476-4598-8-75)
Supplement: Additional file 2 — Gene set enrichment analysis of in vivo data, time series. The data provided represent the time series of GSEA. This compressed file contains "Time" shortcut file and "GSEA_time" folder. Clicking on "Time" shortcut opens the index file providing access to analysis files contained in the "GSEA_time" folder. [file 1476-4598-8-75-S2.zip › GSEA_time/BUT_TSA_UP.html]

Details for gene set BUT\_TSA\_UP[GSEA]

|  || Dataset | gsea\_time\_collapsed |
| Phenotype | NoPhenotypeAvailable |
| Upregulated in class | na\_neg |
| GeneSet | BUT\_TSA\_UP |
| Enrichment Score (ES) | -0.3954773 |
| Normalized Enrichment Score (NES) | -1.2195561 |
| Nominal p-value | 0.19753087 |
| FDR q-value | 0.39351064 |
| FWER p-Value | 1.0 |
Table: GSEA Results Summary

  

Fig 1: Enrichment plot: BUT\_TSA\_UP      
 Profile of the Running ES Score & Positions of GeneSet Members on the Rank Ordered List

  

| PROBE | GENE SYMBOL | GENE\_TITLE | RANK IN GENE LIST | RANK METRIC SCORE | RUNNING ES | CORE ENRICHMENT || 1 | CXCR4 |  |  | 186 | 0.747 | 0.2074 | No |
| 2 | TOB1 |  |  | 1542 | 0.324 | 0.2355 | No |
| 3 | ICAM1 |  |  | 3140 | 0.212 | 0.2192 | No |
| 4 | MAPRE1 |  |  | 3331 | 0.203 | 0.2688 | No |
| 5 | NR4A1 |  |  | 5383 | 0.127 | 0.2060 | No |
| 6 | GADD45A |  |  | 8579 | 0.063 | 0.0692 | No |
| 7 | RNH1 |  |  | 8870 | 0.059 | 0.0721 | No |
| 8 | GATA2 |  |  | 8943 | 0.058 | 0.0853 | No |
| 9 | RHOA |  |  | 10990 | 0.027 | -0.0061 | No |
| 10 | GSTT1 |  |  | 12559 | 0.005 | -0.0807 | No |
| 11 | CDKN1A |  |  | 12863 | 0.001 | -0.0952 | No |
| 12 | NET1 |  |  | 15007 | -0.032 | -0.1901 | No |
| 13 | EPHB3 |  |  | 15117 | -0.034 | -0.1856 | No |
| 14 | PRDX1 |  |  | 16586 | -0.061 | -0.2392 | No |
| 15 | CDC20 |  |  | 18350 | -0.111 | -0.2927 | Yes |
| 16 | PRKCD |  |  | 20468 | -0.443 | -0.2670 | Yes |
| 17 | HSPB1 |  |  | 20498 | -0.468 | -0.1327 | Yes |
| 18 | POR |  |  | 20501 | -0.476 | 0.0051 | Yes |
Table: GSEA details [plain text format]

  

Fig 2: BUT\_TSA\_UP: Random ES distribution      
 Gene set null distribution of ES for **BUT\_TSA\_UP**

  
