# Supplementary material for: The rapamycin-regulated gene expression signature determines prognosis for breast cancer
Source: Mol Cancer. 2009 Sep 24;8:75. doi: 10.1186/1476-4598-8-75 (PMC2761377; doi:10.1186/1476-4598-8-75)
Supplement: Additional file 2 — Gene set enrichment analysis of in vivo data, time series. The data provided represent the time series of GSEA. This compressed file contains "Time" shortcut file and "GSEA_time" folder. Clicking on "Time" shortcut opens the index file providing access to analysis files contained in the "GSEA_time" folder. [file 1476-4598-8-75-S2.zip › GSEA_time/CALRES_MOUSE_UP.html]

Details for gene set CALRES\_MOUSE\_UP[GSEA]

|  || Dataset | gsea\_time\_collapsed |
| Phenotype | NoPhenotypeAvailable |
| Upregulated in class | na\_neg |
| GeneSet | CALRES\_MOUSE\_UP |
| Enrichment Score (ES) | -0.4313392 |
| Normalized Enrichment Score (NES) | -1.5483189 |
| Nominal p-value | 0.024793388 |
| FDR q-value | 0.21153104 |
| FWER p-Value | 0.947 |
Table: GSEA Results Summary

  

Fig 1: Enrichment plot: CALRES\_MOUSE\_UP      
 Profile of the Running ES Score & Positions of GeneSet Members on the Rank Ordered List

  

| PROBE | GENE SYMBOL | GENE\_TITLE | RANK IN GENE LIST | RANK METRIC SCORE | RUNNING ES | CORE ENRICHMENT || 1 | PPP1R2 |  |  | 1183 | 0.374 | 0.0184 | No |
| 2 | PPARD |  |  | 1591 | 0.319 | 0.0635 | No |
| 3 | FZD6 |  |  | 1940 | 0.290 | 0.1054 | No |
| 4 | ADFP |  |  | 3492 | 0.194 | 0.0695 | No |
| 5 | GLUL |  |  | 3828 | 0.181 | 0.0900 | No |
| 6 | SSR4 |  |  | 4058 | 0.171 | 0.1136 | No |
| 7 | FBP2 |  |  | 5129 | 0.134 | 0.0888 | No |
| 8 | LOC129607 |  |  | 5706 | 0.119 | 0.0851 | No |
| 9 | EEF1G |  |  | 6608 | 0.099 | 0.0614 | No |
| 10 | THBD |  |  | 7143 | 0.088 | 0.0534 | No |
| 11 | MYL3 |  |  | 7285 | 0.086 | 0.0640 | No |
| 12 | CLTB |  |  | 9908 | 0.043 | -0.0547 | No |
| 13 | GUCA1A |  |  | 11495 | 0.021 | -0.1276 | No |
| 14 | CTNNA1 |  |  | 11833 | 0.016 | -0.1406 | No |
| 15 | DDIT3 |  |  | 11873 | 0.015 | -0.1394 | No |
| 16 | ALDOC |  |  | 12401 | 0.008 | -0.1633 | No |
| 17 | GIP |  |  | 13609 | -0.010 | -0.2199 | No |
| 18 | PSME1 |  |  | 14347 | -0.021 | -0.2515 | No |
| 19 | ADIPOQ |  |  | 16202 | -0.053 | -0.3308 | No |
| 20 | PEX5 |  |  | 17700 | -0.090 | -0.3853 | No |
| 21 | ACTB |  |  | 18556 | -0.120 | -0.4024 | Yes |
| 22 | PRKCSH |  |  | 19152 | -0.150 | -0.4009 | Yes |
| 23 | MYBPH |  |  | 19520 | -0.180 | -0.3821 | Yes |
| 24 | TKT |  |  | 19574 | -0.186 | -0.3468 | Yes |
| 25 | ACTC1 |  |  | 19679 | -0.197 | -0.3118 | Yes |
| 26 | PPARG |  |  | 19907 | -0.226 | -0.2770 | Yes |
| 27 | SLC1A5 |  |  | 20296 | -0.332 | -0.2283 | Yes |
| 28 | PSMC3 |  |  | 20400 | -0.383 | -0.1556 | Yes |
| 29 | FASN |  |  | 20596 | -0.815 | 0.0004 | Yes |
Table: GSEA details [plain text format]

  

Fig 2: CALRES\_MOUSE\_UP: Random ES distribution      
 Gene set null distribution of ES for **CALRES\_MOUSE\_UP**

  
