# Supplementary material for: The rapamycin-regulated gene expression signature determines prognosis for breast cancer
Source: Mol Cancer. 2009 Sep 24;8:75. doi: 10.1186/1476-4598-8-75 (PMC2761377; doi:10.1186/1476-4598-8-75)
Supplement: Additional file 2 — Gene set enrichment analysis of in vivo data, time series. The data provided represent the time series of GSEA. This compressed file contains "Time" shortcut file and "GSEA_time" folder. Clicking on "Time" shortcut opens the index file providing access to analysis files contained in the "GSEA_time" folder. [file 1476-4598-8-75-S2.zip › GSEA_time/CANCER_UNDIFFERENTIATED_META_UP.html]

Details for gene set CANCER\_UNDIFFERENTIATED\_META\_UP[GSEA]

|  || Dataset | gsea\_time\_collapsed |
| Phenotype | NoPhenotypeAvailable |
| Upregulated in class | na\_neg |
| GeneSet | CANCER\_UNDIFFERENTIATED\_META\_UP |
| Enrichment Score (ES) | -0.38031223 |
| Normalized Enrichment Score (NES) | -1.6664224 |
| Nominal p-value | 0.0 |
| FDR q-value | 0.13696654 |
| FWER p-Value | 0.753 |
Table: GSEA Results Summary

  

Fig 1: Enrichment plot: CANCER\_UNDIFFERENTIATED\_META\_UP      
 Profile of the Running ES Score & Positions of GeneSet Members on the Rank Ordered List

  

| PROBE | GENE SYMBOL | GENE\_TITLE | RANK IN GENE LIST | RANK METRIC SCORE | RUNNING ES | CORE ENRICHMENT || 1 | SLC16A1 |  |  | 187 | 0.746 | 0.0576 | No |
| 2 | BIRC5 |  |  | 745 | 0.463 | 0.0719 | No |
| 3 | GPSM2 |  |  | 1084 | 0.390 | 0.0903 | No |
| 4 | CDC2 |  |  | 1184 | 0.373 | 0.1188 | No |
| 5 | CDKN3 |  |  | 1274 | 0.358 | 0.1465 | No |
| 6 | CCNA2 |  |  | 1525 | 0.326 | 0.1635 | No |
| 7 | PSMB7 |  |  | 2319 | 0.260 | 0.1481 | No |
| 8 | EZH2 |  |  | 2383 | 0.256 | 0.1679 | No |
| 9 | TOP2A |  |  | 3342 | 0.202 | 0.1393 | No |
| 10 | KIF14 |  |  | 3480 | 0.195 | 0.1500 | No |
| 11 | POLR2K |  |  | 3858 | 0.180 | 0.1478 | No |
| 12 | DPM1 |  |  | 4452 | 0.156 | 0.1329 | No |
| 13 | KIAA0101 |  |  | 4566 | 0.151 | 0.1409 | No |
| 14 | PRDX4 |  |  | 4610 | 0.150 | 0.1522 | No |
| 15 | RPA3 |  |  | 4695 | 0.147 | 0.1613 | No |
| 16 | H2AFX |  |  | 6183 | 0.109 | 0.0986 | No |
| 17 | SSBP1 |  |  | 6860 | 0.094 | 0.0741 | No |
| 18 | CKS1B |  |  | 6871 | 0.094 | 0.0820 | No |
| 19 | CXCL9 |  |  | 7134 | 0.089 | 0.0772 | No |
| 20 | NUDT1 |  |  | 7788 | 0.076 | 0.0522 | No |
| 21 | TRIP13 |  |  | 7790 | 0.076 | 0.0589 | No |
| 22 | MCM6 |  |  | 8047 | 0.072 | 0.0528 | No |
| 23 | MELK |  |  | 9282 | 0.053 | -0.0025 | No |
| 24 | GAS6 |  |  | 10032 | 0.042 | -0.0353 | No |
| 25 | KIF23 |  |  | 10825 | 0.030 | -0.0711 | No |
| 26 | NME1 |  |  | 11525 | 0.020 | -0.1034 | No |
| 27 | RFC4 |  |  | 11971 | 0.014 | -0.1238 | No |
| 28 | CCNB1 |  |  | 12244 | 0.011 | -0.1361 | No |
| 29 | PCNA |  |  | 12857 | 0.001 | -0.1658 | No |
| 30 | RAD21 |  |  | 13230 | -0.004 | -0.1835 | No |
| 31 | HMGB2 |  |  | 13259 | -0.005 | -0.1844 | No |
| 32 | NCAPH |  |  | 13338 | -0.006 | -0.1877 | No |
| 33 | SEC61B |  |  | 13930 | -0.015 | -0.2151 | No |
| 34 | CEBPG |  |  | 14610 | -0.025 | -0.2459 | No |
| 35 | CCT6A |  |  | 15444 | -0.038 | -0.2830 | No |
| 36 | MAD2L1 |  |  | 16264 | -0.054 | -0.3181 | No |
| 37 | COL1A2 |  |  | 16667 | -0.063 | -0.3321 | No |
| 38 | DLG7 |  |  | 16705 | -0.063 | -0.3282 | No |
| 39 | H2AFZ |  |  | 16941 | -0.068 | -0.3336 | No |
| 40 | EIF2S2 |  |  | 17192 | -0.075 | -0.3390 | No |
| 41 | TUBB4 |  |  | 17365 | -0.080 | -0.3403 | No |
| 42 | NCAPD2 |  |  | 17630 | -0.088 | -0.3453 | No |
| 43 | CDC20 |  |  | 18350 | -0.111 | -0.3704 | Yes |
| 44 | TMSB10 |  |  | 18365 | -0.112 | -0.3611 | Yes |
| 45 | TAP1 |  |  | 18553 | -0.120 | -0.3595 | Yes |
| 46 | GGH |  |  | 18839 | -0.133 | -0.3615 | Yes |
| 47 | UBE2C |  |  | 18987 | -0.140 | -0.3561 | Yes |
| 48 | ILF2 |  |  | 19034 | -0.143 | -0.3456 | Yes |
| 49 | YBX1 |  |  | 19218 | -0.154 | -0.3407 | Yes |
| 50 | KIF2C |  |  | 19275 | -0.159 | -0.3293 | Yes |
| 51 | SLC7A5 |  |  | 19380 | -0.168 | -0.3193 | Yes |
| 52 | CTSL |  |  | 19382 | -0.168 | -0.3044 | Yes |
| 53 | MCM3 |  |  | 19468 | -0.176 | -0.2928 | Yes |
| 54 | GCLM |  |  | 19568 | -0.186 | -0.2810 | Yes |
| 55 | IFI30 |  |  | 19605 | -0.189 | -0.2659 | Yes |
| 56 | FOXM1 |  |  | 19610 | -0.189 | -0.2492 | Yes |
| 57 | CKS2 |  |  | 19619 | -0.191 | -0.2325 | Yes |
| 58 | PSMD14 |  |  | 19634 | -0.192 | -0.2160 | Yes |
| 59 | MTHFD2 |  |  | 19664 | -0.196 | -0.1999 | Yes |
| 60 | GARS |  |  | 19841 | -0.217 | -0.1891 | Yes |
| 61 | PSMD2 |  |  | 19976 | -0.237 | -0.1745 | Yes |
| 62 | CDC6 |  |  | 20126 | -0.270 | -0.1576 | Yes |
| 63 | TUBB |  |  | 20162 | -0.282 | -0.1341 | Yes |
| 64 | MCM2 |  |  | 20168 | -0.285 | -0.1089 | Yes |
| 65 | MYBL2 |  |  | 20456 | -0.434 | -0.0840 | Yes |
| 66 | CENPA |  |  | 20463 | -0.438 | -0.0452 | Yes |
| 67 | ADRM1 |  |  | 20562 | -0.582 | 0.0021 | Yes |
Table: GSEA details [plain text format]

  

Fig 2: CANCER\_UNDIFFERENTIATED\_META\_UP: Random ES distribution      
 Gene set null distribution of ES for **CANCER\_UNDIFFERENTIATED\_META\_UP**

  
