# Supplementary material for: The rapamycin-regulated gene expression signature determines prognosis for breast cancer
Source: Mol Cancer. 2009 Sep 24;8:75. doi: 10.1186/1476-4598-8-75 (PMC2761377; doi:10.1186/1476-4598-8-75)
Supplement: Additional file 2 — Gene set enrichment analysis of in vivo data, time series. The data provided represent the time series of GSEA. This compressed file contains "Time" shortcut file and "GSEA_time" folder. Clicking on "Time" shortcut opens the index file providing access to analysis files contained in the "GSEA_time" folder. [file 1476-4598-8-75-S2.zip › GSEA_time/CHEN_HOXA5_TARGETS_UP.html]

Details for gene set CHEN\_HOXA5\_TARGETS\_UP[GSEA]

|  || Dataset | gsea\_time\_collapsed |
| Phenotype | NoPhenotypeAvailable |
| Upregulated in class | na\_pos |
| GeneSet | CHEN\_HOXA5\_TARGETS\_UP |
| Enrichment Score (ES) | 0.6053439 |
| Normalized Enrichment Score (NES) | 1.8442624 |
| Nominal p-value | 0.0 |
| FDR q-value | 0.0040729907 |
| FWER p-Value | 0.066 |
Table: GSEA Results Summary

  

Fig 1: Enrichment plot: CHEN\_HOXA5\_TARGETS\_UP      
 Profile of the Running ES Score & Positions of GeneSet Members on the Rank Ordered List

  

| PROBE | GENE SYMBOL | GENE\_TITLE | RANK IN GENE LIST | RANK METRIC SCORE | RUNNING ES | CORE ENRICHMENT || 1 | IL8 |  |  | 13 | 1.561 | 0.0290 | Yes |
| 2 | SAT1 |  |  | 58 | 1.024 | 0.0463 | Yes |
| 3 | BIRC3 |  |  | 61 | 1.013 | 0.0654 | Yes |
| 4 | DUSP6 |  |  | 94 | 0.912 | 0.0812 | Yes |
| 5 | TSC22D2 |  |  | 98 | 0.907 | 0.0982 | Yes |
| 6 | NEDD9 |  |  | 100 | 0.905 | 0.1154 | Yes |
| 7 | MAFF |  |  | 109 | 0.883 | 0.1317 | Yes |
| 8 | CLK4 |  |  | 126 | 0.861 | 0.1473 | Yes |
| 9 | JOSD3 |  |  | 218 | 0.705 | 0.1562 | Yes |
| 10 | FNBP4 |  |  | 226 | 0.700 | 0.1692 | Yes |
| 11 | VEGF |  |  | 252 | 0.681 | 0.1809 | Yes |
| 12 | BCL10 |  |  | 255 | 0.679 | 0.1936 | Yes |
| 13 | STC2 |  |  | 256 | 0.679 | 0.2065 | Yes |
| 14 | ZNF557 |  |  | 344 | 0.610 | 0.2138 | Yes |
| 15 | F3 |  |  | 371 | 0.596 | 0.2239 | Yes |
| 16 | NUPL1 |  |  | 388 | 0.591 | 0.2343 | Yes |
| 17 | CROCC /// MGC12760 / |  |  | 411 | 0.575 | 0.2441 | Yes |
| 18 | RABGGTB |  |  | 419 | 0.570 | 0.2546 | Yes |
| 19 | MCL1 |  |  | 471 | 0.544 | 0.2624 | Yes |
| 20 | PRPF38B |  |  | 472 | 0.543 | 0.2727 | Yes |
| 21 | CLK1 |  |  | 503 | 0.531 | 0.2814 | Yes |
| 22 | GABARAPL1 /// GABARA |  |  | 535 | 0.520 | 0.2897 | Yes |
| 23 | CAB39 |  |  | 617 | 0.493 | 0.2951 | Yes |
| 24 | LIF |  |  | 724 | 0.469 | 0.2988 | Yes |
| 25 | CCNL1 |  |  | 780 | 0.453 | 0.3047 | Yes |
| 26 | CXORF45 |  |  | 792 | 0.447 | 0.3127 | Yes |
| 27 | SFPQ |  |  | 796 | 0.446 | 0.3210 | Yes |
| 28 | DLX2 |  |  | 885 | 0.425 | 0.3247 | Yes |
| 29 | PMAIP1 |  |  | 904 | 0.422 | 0.3319 | Yes |
| 30 | TNFAIP3 |  |  | 911 | 0.421 | 0.3396 | Yes |
| 31 | ZNF267 |  |  | 921 | 0.418 | 0.3471 | Yes |
| 32 | TSC22D1 |  |  | 1008 | 0.403 | 0.3505 | Yes |
| 33 | BIRC2 |  |  | 1020 | 0.402 | 0.3576 | Yes |
| 34 | JMJD1C |  |  | 1042 | 0.397 | 0.3641 | Yes |
| 35 | DCLRE1C |  |  | 1059 | 0.394 | 0.3708 | Yes |
| 36 | ING3 |  |  | 1066 | 0.394 | 0.3780 | Yes |
| 37 | C5ORF5 |  |  | 1097 | 0.388 | 0.3839 | Yes |
| 38 | STRN3 |  |  | 1117 | 0.383 | 0.3902 | Yes |
| 39 | FLJ22028 |  |  | 1146 | 0.378 | 0.3960 | Yes |
| 40 | PTBP2 |  |  | 1213 | 0.368 | 0.3998 | Yes |
| 41 | TIPARP |  |  | 1231 | 0.365 | 0.4059 | Yes |
| 42 | CNOT2 |  |  | 1286 | 0.356 | 0.4100 | Yes |
| 43 | SEC31A |  |  | 1288 | 0.356 | 0.4167 | Yes |
| 44 | ARNTL |  |  | 1336 | 0.349 | 0.4210 | Yes |
| 45 | BACH1 |  |  | 1355 | 0.346 | 0.4267 | Yes |
| 46 | AGTPBP1 |  |  | 1373 | 0.344 | 0.4324 | Yes |
| 47 | FLJ11021 |  |  | 1375 | 0.344 | 0.4389 | Yes |
| 48 | CHD9 |  |  | 1413 | 0.340 | 0.4435 | Yes |
| 49 | ZCCHC8 |  |  | 1440 | 0.336 | 0.4486 | Yes |
| 50 | MORC3 |  |  | 1479 | 0.332 | 0.4530 | Yes |
| 51 | C1ORF108 |  |  | 1499 | 0.329 | 0.4584 | Yes |
| 52 | JUN |  |  | 1522 | 0.326 | 0.4635 | Yes |
| 53 | AKAP10 |  |  | 1681 | 0.310 | 0.4616 | Yes |
| 54 | FBXO38 |  |  | 1732 | 0.305 | 0.4649 | Yes |
| 55 | ZNF20 |  |  | 1735 | 0.305 | 0.4706 | Yes |
| 56 | TUFT1 |  |  | 1744 | 0.304 | 0.4760 | Yes |
| 57 | C14ORF138 |  |  | 1772 | 0.302 | 0.4804 | Yes |
| 58 | ZNF14 |  |  | 1800 | 0.300 | 0.4848 | Yes |
| 59 | MTHFD2L |  |  | 1817 | 0.299 | 0.4897 | Yes |
| 60 | KLF5 |  |  | 1827 | 0.298 | 0.4949 | Yes |
| 61 | ZZZ3 |  |  | 1926 | 0.290 | 0.4956 | Yes |
| 62 | ZNF131 |  |  | 1963 | 0.287 | 0.4992 | Yes |
| 63 | ZNF228 |  |  | 1982 | 0.285 | 0.5038 | Yes |
| 64 | SMCHD1 |  |  | 1993 | 0.285 | 0.5087 | Yes |
| 65 | KLF10 |  |  | 2047 | 0.280 | 0.5114 | Yes |
| 66 | PCTK2 |  |  | 2050 | 0.279 | 0.5166 | Yes |
| 67 | TNFRSF10B |  |  | 2060 | 0.279 | 0.5215 | Yes |
| 68 | SUV420H1 |  |  | 2062 | 0.279 | 0.5267 | Yes |
| 69 | CCNT2 |  |  | 2095 | 0.277 | 0.5304 | Yes |
| 70 | PSPC1 |  |  | 2243 | 0.265 | 0.5282 | Yes |
| 71 | KLF6 |  |  | 2275 | 0.263 | 0.5317 | Yes |
| 72 | TBX3 |  |  | 2346 | 0.259 | 0.5331 | Yes |
| 73 | ZNF435 |  |  | 2353 | 0.258 | 0.5377 | Yes |
| 74 | RBM15 |  |  | 2376 | 0.256 | 0.5415 | Yes |
| 75 | PELI1 |  |  | 2506 | 0.247 | 0.5399 | Yes |
| 76 | ZCCHC6 |  |  | 2508 | 0.247 | 0.5445 | Yes |
| 77 | ATF3 |  |  | 2567 | 0.244 | 0.5463 | Yes |
| 78 | SLTM |  |  | 2594 | 0.242 | 0.5496 | Yes |
| 79 | MARCH7 |  |  | 2664 | 0.238 | 0.5508 | Yes |
| 80 | CREB5 |  |  | 2799 | 0.230 | 0.5486 | Yes |
| 81 | DMTF1 |  |  | 2883 | 0.226 | 0.5488 | Yes |
| 82 | GADD45B |  |  | 2916 | 0.224 | 0.5514 | Yes |
| 83 | SDCCAG1 |  |  | 3030 | 0.218 | 0.5500 | Yes |
| 84 | KIAA0907 |  |  | 3074 | 0.215 | 0.5520 | Yes |
| 85 | ADNP |  |  | 3077 | 0.215 | 0.5560 | Yes |
| 86 | NFIL3 |  |  | 3085 | 0.214 | 0.5597 | Yes |
| 87 | RBBP6 |  |  | 3118 | 0.213 | 0.5622 | Yes |
| 88 | TAF2 |  |  | 3137 | 0.212 | 0.5653 | Yes |
| 89 | LIG4 |  |  | 3151 | 0.211 | 0.5687 | Yes |
| 90 | IFRD1 |  |  | 3206 | 0.209 | 0.5700 | Yes |
| 91 | PPWD1 |  |  | 3237 | 0.207 | 0.5725 | Yes |
| 92 | ZNF432 |  |  | 3242 | 0.207 | 0.5762 | Yes |
| 93 | ZNF44 |  |  | 3245 | 0.206 | 0.5800 | Yes |
| 94 | C1ORF27 |  |  | 3260 | 0.206 | 0.5832 | Yes |
| 95 | C6ORF62 |  |  | 3291 | 0.204 | 0.5856 | Yes |
| 96 | YY1AP1 |  |  | 3303 | 0.204 | 0.5890 | Yes |
| 97 | DPP8 |  |  | 3460 | 0.196 | 0.5850 | Yes |
| 98 | TDG |  |  | 3462 | 0.196 | 0.5887 | Yes |
| 99 | ZCCHC10 |  |  | 3472 | 0.195 | 0.5920 | Yes |
| 100 | ZNF274 |  |  | 3504 | 0.194 | 0.5941 | Yes |
| 101 | ARIH1 |  |  | 3514 | 0.193 | 0.5974 | Yes |
| 102 | KIAA0241 |  |  | 3545 | 0.192 | 0.5995 | Yes |
| 103 | FEM1C |  |  | 3678 | 0.188 | 0.5966 | Yes |
| 104 | FOXO3A |  |  | 3680 | 0.188 | 0.6001 | Yes |
| 105 | RYBP |  |  | 3772 | 0.184 | 0.5992 | Yes |
| 106 | CENPC1 |  |  | 3842 | 0.181 | 0.5992 | Yes |
| 107 | JMJD1A |  |  | 3860 | 0.180 | 0.6018 | Yes |
| 108 | SNX16 |  |  | 3896 | 0.178 | 0.6035 | Yes |
| 109 | ETS2 |  |  | 3927 | 0.177 | 0.6053 | Yes |
| 110 | ZNF468 |  |  | 4071 | 0.171 | 0.6016 | No |
| 111 | SNX5 |  |  | 4174 | 0.166 | 0.5997 | No |
| 112 | TANK |  |  | 4359 | 0.159 | 0.5937 | No |
| 113 | CASP3 |  |  | 4494 | 0.155 | 0.5901 | No |
| 114 | NEK1 |  |  | 4543 | 0.152 | 0.5906 | No |
| 115 | PPIG |  |  | 4555 | 0.152 | 0.5929 | No |
| 116 | SUHW4 |  |  | 4601 | 0.151 | 0.5936 | No |
| 117 | EIF5 |  |  | 4629 | 0.150 | 0.5951 | No |
| 118 | PRKRIP1 |  |  | 4687 | 0.148 | 0.5951 | No |
| 119 | TRPC1 |  |  | 4759 | 0.145 | 0.5944 | No |
| 120 | PTPN2 |  |  | 4886 | 0.141 | 0.5909 | No |
| 121 | RLF |  |  | 4912 | 0.140 | 0.5923 | No |
| 122 | RBM5 |  |  | 5119 | 0.135 | 0.5847 | No |
| 123 | ZNF19 /// ZNF23 |  |  | 5153 | 0.134 | 0.5857 | No |
| 124 | NCOA3 |  |  | 5249 | 0.131 | 0.5835 | No |
| 125 | BDKRB1 |  |  | 5312 | 0.129 | 0.5829 | No |
| 126 | GPATC2 |  |  | 5355 | 0.128 | 0.5833 | No |
| 127 | RBM39 |  |  | 5485 | 0.124 | 0.5793 | No |
| 128 | CTH |  |  | 5594 | 0.122 | 0.5763 | No |
| 129 | DAAM1 |  |  | 5641 | 0.121 | 0.5763 | No |
| 130 | CRY1 |  |  | 5740 | 0.118 | 0.5738 | No |
| 131 | TLK2 |  |  | 5757 | 0.118 | 0.5752 | No |
| 132 | PNRC2 /// LOC644047 |  |  | 5786 | 0.117 | 0.5761 | No |
| 133 | CNOT4 |  |  | 5973 | 0.113 | 0.5691 | No |
| 134 | FAM48A |  |  | 6039 | 0.112 | 0.5680 | No |
| 135 | ZNF195 |  |  | 6076 | 0.111 | 0.5684 | No |
| 136 | PPP1R12A |  |  | 6133 | 0.110 | 0.5677 | No |
| 137 | SCYL3 |  |  | 6190 | 0.109 | 0.5670 | No |
| 138 | PTGS2 |  |  | 6233 | 0.107 | 0.5670 | No |
| 139 | ARID4B |  |  | 6251 | 0.107 | 0.5682 | No |
| 140 | ZNF451 |  |  | 6300 | 0.105 | 0.5678 | No |
| 141 | GEM |  |  | 6405 | 0.103 | 0.5647 | No |
| 142 | RNF111 |  |  | 6416 | 0.103 | 0.5662 | No |
| 143 | SFRS12 |  |  | 6486 | 0.102 | 0.5647 | No |
| 144 | ZNF45 |  |  | 6519 | 0.101 | 0.5650 | No |
| 145 | SEC24A |  |  | 6529 | 0.101 | 0.5665 | No |
| 146 | ZNF3 |  |  | 6750 | 0.096 | 0.5576 | No |
| 147 | CARF |  |  | 6950 | 0.092 | 0.5495 | No |
| 148 | LINS1 |  |  | 7407 | 0.084 | 0.5287 | No |
| 149 | KIN |  |  | 7426 | 0.083 | 0.5294 | No |
| 150 | C17ORF48 |  |  | 7519 | 0.082 | 0.5265 | No |
| 151 | HLX1 |  |  | 7578 | 0.080 | 0.5252 | No |
| 152 | NSUN6 |  |  | 7677 | 0.078 | 0.5218 | No |
| 153 | NOC3L |  |  | 7761 | 0.076 | 0.5192 | No |
| 154 | LOC51315 |  |  | 7919 | 0.074 | 0.5129 | No |
| 155 | EGR1 |  |  | 8018 | 0.072 | 0.5095 | No |
| 156 | CARS |  |  | 8060 | 0.072 | 0.5088 | No |
| 157 | ZNF588 |  |  | 8075 | 0.072 | 0.5095 | No |
| 158 | WAC |  |  | 8094 | 0.071 | 0.5100 | No |
| 159 | PLGLA1 |  |  | 8114 | 0.071 | 0.5104 | No |
| 160 | C13ORF24 |  |  | 8117 | 0.071 | 0.5116 | No |
| 161 | BTN2A1 |  |  | 8280 | 0.068 | 0.5050 | No |
| 162 | THSD1 /// THSD1P |  |  | 8431 | 0.066 | 0.4989 | No |
| 163 | SOX30 |  |  | 8588 | 0.063 | 0.4924 | No |
| 164 | ZNF180 |  |  | 8592 | 0.063 | 0.4934 | No |
| 165 | PTN |  |  | 8684 | 0.062 | 0.4902 | No |
| 166 | DUSP8 |  |  | 8724 | 0.062 | 0.4894 | No |
| 167 | ZNF331 |  |  | 8784 | 0.060 | 0.4877 | No |
| 168 | POLR3F |  |  | 8883 | 0.059 | 0.4840 | No |
| 169 | KIAA1240 |  |  | 9001 | 0.057 | 0.4793 | No |
| 170 | NFKBIA |  |  | 9034 | 0.056 | 0.4788 | No |
| 171 | INTS6 |  |  | 9244 | 0.053 | 0.4696 | No |
| 172 | MARK3 |  |  | 9253 | 0.053 | 0.4702 | No |
| 173 | DSCR1 |  |  | 9255 | 0.053 | 0.4711 | No |
| 174 | CDK7 |  |  | 9483 | 0.050 | 0.4609 | No |
| 175 | PGS1 |  |  | 9556 | 0.049 | 0.4583 | No |
| 176 | IHPK2 |  |  | 9786 | 0.045 | 0.4480 | No |
| 177 | RAP80 |  |  | 9828 | 0.044 | 0.4468 | No |
| 178 | CREM |  |  | 9885 | 0.044 | 0.4449 | No |
| 179 | RCHY1 |  |  | 10088 | 0.041 | 0.4357 | No |
| 180 | C10ORF137 |  |  | 10159 | 0.040 | 0.4330 | No |
| 181 | ZNF136 |  |  | 10203 | 0.039 | 0.4317 | No |
| 182 | NFKBIE |  |  | 10422 | 0.036 | 0.4217 | No |
| 183 | THUMPD2 |  |  | 10465 | 0.035 | 0.4203 | No |
| 184 | SETDB1 |  |  | 10864 | 0.029 | 0.4013 | No |
| 185 | RIC8B |  |  | 10966 | 0.028 | 0.3969 | No |
| 186 | FOS |  |  | 11075 | 0.026 | 0.3921 | No |
| 187 | IFI16 |  |  | 11265 | 0.024 | 0.3832 | No |
| 188 | ASNS |  |  | 11419 | 0.022 | 0.3761 | No |
| 189 | ZNF184 |  |  | 11976 | 0.014 | 0.3491 | No |
| 190 | GAD1 |  |  | 12033 | 0.013 | 0.3466 | No |
| 191 | FOSL2 |  |  | 12117 | 0.012 | 0.3428 | No |
| 192 | HRH1 |  |  | 12203 | 0.011 | 0.3388 | No |
| 193 | ZNF35 |  |  | 12265 | 0.010 | 0.3360 | No |
| 194 | ZNF227 |  |  | 12980 | -0.001 | 0.3010 | No |
| 195 | ZNF155 |  |  | 13116 | -0.003 | 0.2944 | No |
| 196 | TAF1A |  |  | 13139 | -0.003 | 0.2934 | No |
| 197 | RIPK2 |  |  | 13298 | -0.006 | 0.2858 | No |
| 198 | SPATA5L1 |  |  | 13601 | -0.010 | 0.2711 | No |
| 199 | SCG5 |  |  | 13657 | -0.011 | 0.2686 | No |
| 200 | JUNB |  |  | 13719 | -0.012 | 0.2659 | No |
| 201 | ZNF222 |  |  | 14536 | -0.024 | 0.2263 | No |
| 202 | TNFRSF9 |  |  | 14598 | -0.025 | 0.2238 | No |
| 203 | CEBPG |  |  | 14610 | -0.025 | 0.2237 | No |
| 204 | C2ORF42 |  |  | 14723 | -0.027 | 0.2187 | No |
| 205 | CLEC4A |  |  | 15407 | -0.038 | 0.1859 | No |
| 206 | SMA3 /// SMA5 |  |  | 15482 | -0.039 | 0.1830 | No |
| 207 | SNAI2 |  |  | 16833 | -0.066 | 0.1180 | No |
| 208 | KIF18A |  |  | 16881 | -0.067 | 0.1170 | No |
| 209 | ZRF1 |  |  | 17377 | -0.080 | 0.0942 | No |
| 210 | TBK1 |  |  | 17514 | -0.085 | 0.0892 | No |
| 211 | IER2 |  |  | 18093 | -0.102 | 0.0627 | No |
| 212 | RELB |  |  | 18126 | -0.103 | 0.0631 | No |
| 213 | ZNF426 |  |  | 18153 | -0.104 | 0.0638 | No |
| 214 | TMEFF1 |  |  | 18345 | -0.111 | 0.0566 | No |
| 215 | FUSIP1 /// LOC642558 |  |  | 18360 | -0.112 | 0.0580 | No |
| 216 | WDR67 |  |  | 18617 | -0.122 | 0.0478 | No |
| 217 | FAM53C |  |  | 18653 | -0.124 | 0.0484 | No |
| 218 | FOSB |  |  | 18812 | -0.131 | 0.0431 | No |
| 219 | C1ORF103 |  |  | 19168 | -0.151 | 0.0286 | No |
| 220 | C21ORF91 |  |  | 19229 | -0.155 | 0.0286 | No |
| 221 | NRBF2 |  |  | 19449 | -0.174 | 0.0211 | No |
| 222 | IRF7 |  |  | 19564 | -0.185 | 0.0191 | No |
| 223 | TPTE |  |  | 19584 | -0.187 | 0.0217 | No |
| 224 | CHAC1 |  |  | 19637 | -0.192 | 0.0228 | No |
| 225 | DUSP5 |  |  | 19722 | -0.202 | 0.0225 | No |
| 226 | PRPF3 |  |  | 19785 | -0.210 | 0.0234 | No |
| 227 | CEP76 |  |  | 20021 | -0.246 | 0.0166 | No |
| 228 | ZNF165 |  |  | 20187 | -0.290 | 0.0140 | No |
| 229 | CEBPB |  |  | 20318 | -0.341 | 0.0141 | No |
Table: GSEA details [plain text format]

  

Fig 2: CHEN\_HOXA5\_TARGETS\_UP: Random ES distribution      
 Gene set null distribution of ES for **CHEN\_HOXA5\_TARGETS\_UP**

  
