# Supplementary material for: The rapamycin-regulated gene expression signature determines prognosis for breast cancer
Source: Mol Cancer. 2009 Sep 24;8:75. doi: 10.1186/1476-4598-8-75 (PMC2761377; doi:10.1186/1476-4598-8-75)
Supplement: Additional file 2 — Gene set enrichment analysis of in vivo data, time series. The data provided represent the time series of GSEA. This compressed file contains "Time" shortcut file and "GSEA_time" folder. Clicking on "Time" shortcut opens the index file providing access to analysis files contained in the "GSEA_time" folder. [file 1476-4598-8-75-S2.zip › GSEA_time/CITRATE_CYCLE_TCA_CYCLE.html]

Details for gene set CITRATE\_CYCLE\_TCA\_CYCLE[GSEA]

|  || Dataset | gsea\_time\_collapsed |
| Phenotype | NoPhenotypeAvailable |
| Upregulated in class | na\_neg |
| GeneSet | CITRATE\_CYCLE\_TCA\_CYCLE |
| Enrichment Score (ES) | -0.58077174 |
| Normalized Enrichment Score (NES) | -1.8276037 |
| Nominal p-value | 0.0 |
| FDR q-value | 0.06688772 |
| FWER p-Value | 0.345 |
Table: GSEA Results Summary

  

Fig 1: Enrichment plot: CITRATE\_CYCLE\_TCA\_CYCLE      
 Profile of the Running ES Score & Positions of GeneSet Members on the Rank Ordered List

  

| PROBE | GENE SYMBOL | GENE\_TITLE | RANK IN GENE LIST | RANK METRIC SCORE | RUNNING ES | CORE ENRICHMENT || 1 | MDH1 |  |  | 1304 | 0.354 | 0.0426 | No |
| 2 | SUCLG2 |  |  | 5158 | 0.133 | -0.1047 | No |
| 3 | IDH3A |  |  | 6869 | 0.094 | -0.1597 | No |
| 4 | MDH2 |  |  | 7052 | 0.090 | -0.1415 | No |
| 5 | DLD |  |  | 8057 | 0.072 | -0.1688 | No |
| 6 | SUCLA2 |  |  | 8448 | 0.065 | -0.1682 | No |
| 7 | IDH1 |  |  | 9070 | 0.056 | -0.1817 | No |
| 8 | SDHB |  |  | 9306 | 0.052 | -0.1774 | No |
| 9 | DLST /// DLSTP |  |  | 15717 | -0.043 | -0.4758 | No |
| 10 | FH |  |  | 15798 | -0.045 | -0.4663 | No |
| 11 | IDH3G |  |  | 16278 | -0.054 | -0.4733 | No |
| 12 | ACO1 |  |  | 18491 | -0.117 | -0.5458 | Yes |
| 13 | PCK1 |  |  | 19140 | -0.150 | -0.5325 | Yes |
| 14 | CS |  |  | 19240 | -0.156 | -0.4907 | Yes |
| 15 | IDH2 |  |  | 19803 | -0.213 | -0.4544 | Yes |
| 16 | PC |  |  | 20000 | -0.241 | -0.3918 | Yes |
| 17 | SDHA |  |  | 20118 | -0.266 | -0.3177 | Yes |
| 18 | IDH3B |  |  | 20127 | -0.270 | -0.2374 | Yes |
| 19 | SUCLG1 |  |  | 20440 | -0.417 | -0.1278 | Yes |
| 20 | ACO2 |  |  | 20483 | -0.453 | 0.0059 | Yes |
Table: GSEA details [plain text format]

  

Fig 2: CITRATE\_CYCLE\_TCA\_CYCLE: Random ES distribution      
 Gene set null distribution of ES for **CITRATE\_CYCLE\_TCA\_CYCLE**

  
