# Supplementary material for: The rapamycin-regulated gene expression signature determines prognosis for breast cancer
Source: Mol Cancer. 2009 Sep 24;8:75. doi: 10.1186/1476-4598-8-75 (PMC2761377; doi:10.1186/1476-4598-8-75)
Supplement: Additional file 2 — Gene set enrichment analysis of in vivo data, time series. The data provided represent the time series of GSEA. This compressed file contains "Time" shortcut file and "GSEA_time" folder. Clicking on "Time" shortcut opens the index file providing access to analysis files contained in the "GSEA_time" folder. [file 1476-4598-8-75-S2.zip › GSEA_time/CMV-UV_HCMV_6HRS_DN.html]

Details for gene set CMV-UV\_HCMV\_6HRS\_DN[GSEA]

|  || Dataset | gsea\_time\_collapsed |
| Phenotype | NoPhenotypeAvailable |
| Upregulated in class | na\_pos |
| GeneSet | CMV-UV\_HCMV\_6HRS\_DN |
| Enrichment Score (ES) | 0.62872577 |
| Normalized Enrichment Score (NES) | 1.865405 |
| Nominal p-value | 0.0 |
| FDR q-value | 0.0033808232 |
| FWER p-Value | 0.037 |
Table: GSEA Results Summary

  

Fig 1: Enrichment plot: CMV-UV\_HCMV\_6HRS\_DN      
 Profile of the Running ES Score & Positions of GeneSet Members on the Rank Ordered List

  

| PROBE | GENE SYMBOL | GENE\_TITLE | RANK IN GENE LIST | RANK METRIC SCORE | RUNNING ES | CORE ENRICHMENT || 1 | OLFM4 |  |  | 0 | 2.965 | 0.1250 | Yes |
| 2 | RARRES1 |  |  | 113 | 0.880 | 0.1567 | Yes |
| 3 | NR2F2 |  |  | 115 | 0.875 | 0.1935 | Yes |
| 4 | GPRC5A |  |  | 151 | 0.800 | 0.2255 | Yes |
| 5 | TCF7L2 |  |  | 246 | 0.684 | 0.2498 | Yes |
| 6 | C6ORF111 |  |  | 348 | 0.608 | 0.2705 | Yes |
| 7 | CDC42EP3 |  |  | 350 | 0.608 | 0.2961 | Yes |
| 8 | ZF |  |  | 351 | 0.608 | 0.3217 | Yes |
| 9 | SOCS2 |  |  | 404 | 0.579 | 0.3436 | Yes |
| 10 | GAS1 |  |  | 498 | 0.535 | 0.3617 | Yes |
| 11 | TRIB2 |  |  | 573 | 0.507 | 0.3794 | Yes |
| 12 | ZNF292 |  |  | 582 | 0.505 | 0.4003 | Yes |
| 13 | PDE4B |  |  | 685 | 0.476 | 0.4154 | Yes |
| 14 | SR140 |  |  | 698 | 0.475 | 0.4349 | Yes |
| 15 | COL13A1 |  |  | 733 | 0.466 | 0.4529 | Yes |
| 16 | RUNX1 |  |  | 822 | 0.441 | 0.4672 | Yes |
| 17 | NUP210 |  |  | 847 | 0.434 | 0.4843 | Yes |
| 18 | ADM |  |  | 932 | 0.417 | 0.4978 | Yes |
| 19 | MTUS1 |  |  | 1289 | 0.356 | 0.4955 | Yes |
| 20 | SOCS5 |  |  | 1350 | 0.347 | 0.5071 | Yes |
| 21 | NCK1 |  |  | 1399 | 0.341 | 0.5192 | Yes |
| 22 | WEE1 |  |  | 1417 | 0.339 | 0.5326 | Yes |
| 23 | DYRK2 |  |  | 1584 | 0.320 | 0.5380 | Yes |
| 24 | EPM2AIP1 |  |  | 1720 | 0.306 | 0.5443 | Yes |
| 25 | SIAH1 |  |  | 1724 | 0.306 | 0.5571 | Yes |
| 26 | CDKN1B |  |  | 1916 | 0.291 | 0.5600 | Yes |
| 27 | KLF10 |  |  | 2047 | 0.280 | 0.5655 | Yes |
| 28 | BRWD1 |  |  | 2091 | 0.277 | 0.5751 | Yes |
| 29 | ARL4C |  |  | 2355 | 0.258 | 0.5731 | Yes |
| 30 | RANBP6 |  |  | 2359 | 0.258 | 0.5838 | Yes |
| 31 | KLF7 |  |  | 2569 | 0.244 | 0.5839 | Yes |
| 32 | NR2C1 |  |  | 2743 | 0.234 | 0.5853 | Yes |
| 33 | KLHL9 |  |  | 2849 | 0.227 | 0.5898 | Yes |
| 34 | SMAD3 |  |  | 2926 | 0.223 | 0.5955 | Yes |
| 35 | CRSP9 |  |  | 3052 | 0.216 | 0.5985 | Yes |
| 36 | MYO10 |  |  | 3066 | 0.215 | 0.6070 | Yes |
| 37 | CAMK2G |  |  | 3156 | 0.211 | 0.6115 | Yes |
| 38 | DUSP10 |  |  | 3267 | 0.206 | 0.6149 | Yes |
| 39 | CSTF1 |  |  | 3286 | 0.204 | 0.6226 | Yes |
| 40 | RND3 |  |  | 3338 | 0.202 | 0.6286 | Yes |
| 41 | MGMT |  |  | 3512 | 0.193 | 0.6284 | Yes |
| 42 | COIL |  |  | 3668 | 0.188 | 0.6287 | Yes |
| 43 | RBM16 |  |  | 3972 | 0.175 | 0.6213 | No |
| 44 | KIRREL |  |  | 4262 | 0.163 | 0.6141 | No |
| 45 | SOS2 |  |  | 4297 | 0.161 | 0.6192 | No |
| 46 | KCNJ2 |  |  | 4377 | 0.158 | 0.6220 | No |
| 47 | MEIS1 |  |  | 4440 | 0.156 | 0.6256 | No |
| 48 | ERCC5 |  |  | 4609 | 0.150 | 0.6237 | No |
| 49 | PIM1 |  |  | 5180 | 0.132 | 0.6015 | No |
| 50 | KIAA0376 |  |  | 5351 | 0.128 | 0.5986 | No |
| 51 | MATN2 |  |  | 5576 | 0.122 | 0.5928 | No |
| 52 | ZNF148 |  |  | 5767 | 0.118 | 0.5885 | No |
| 53 | DNMBP |  |  | 5861 | 0.116 | 0.5889 | No |
| 54 | BDKRB2 |  |  | 6062 | 0.111 | 0.5838 | No |
| 55 | PPYR1 |  |  | 6354 | 0.104 | 0.5740 | No |
| 56 | MYC |  |  | 6440 | 0.103 | 0.5742 | No |
| 57 | FPRL1 |  |  | 6723 | 0.097 | 0.5645 | No |
| 58 | PRR3 |  |  | 6847 | 0.094 | 0.5625 | No |
| 59 | EMG1 |  |  | 7609 | 0.080 | 0.5287 | No |
| 60 | GLI3 |  |  | 7763 | 0.076 | 0.5245 | No |
| 61 | HOXA11 |  |  | 8002 | 0.073 | 0.5159 | No |
| 62 | ITPKB |  |  | 8720 | 0.062 | 0.4836 | No |
| 63 | DKFZP564O0823 |  |  | 8818 | 0.060 | 0.4814 | No |
| 64 | ASB1 |  |  | 9159 | 0.054 | 0.4671 | No |
| 65 | VPS13D |  |  | 9389 | 0.051 | 0.4580 | No |
| 66 | KIAA1462 |  |  | 9757 | 0.046 | 0.4421 | No |
| 67 | STARD13 |  |  | 9948 | 0.043 | 0.4346 | No |
| 68 | ID2 |  |  | 10102 | 0.040 | 0.4288 | No |
| 69 | RUNX1T1 |  |  | 10223 | 0.038 | 0.4246 | No |
| 70 | FGF7 |  |  | 10279 | 0.038 | 0.4235 | No |
| 71 | AURKA |  |  | 10284 | 0.038 | 0.4249 | No |
| 72 | ZNF205 |  |  | 10657 | 0.032 | 0.4081 | No |
| 73 | ADORA2B |  |  | 10853 | 0.029 | 0.3998 | No |
| 74 | ETFB |  |  | 11137 | 0.026 | 0.3871 | No |
| 75 | RNF113A |  |  | 11193 | 0.025 | 0.3855 | No |
| 76 | ASNS |  |  | 11419 | 0.022 | 0.3754 | No |
| 77 | DOK1 |  |  | 11709 | 0.018 | 0.3621 | No |
| 78 | C14ORF132 |  |  | 11783 | 0.017 | 0.3592 | No |
| 79 | NR1D2 |  |  | 11988 | 0.014 | 0.3499 | No |
| 80 | HARSL |  |  | 12248 | 0.011 | 0.3377 | No |
| 81 | HOXA9 |  |  | 12432 | 0.008 | 0.3291 | No |
| 82 | HSPB3 |  |  | 12451 | 0.007 | 0.3285 | No |
| 83 | PTPRS |  |  | 12560 | 0.005 | 0.3235 | No |
| 84 | LRRC17 |  |  | 12808 | 0.001 | 0.3115 | No |
| 85 | FRMPD4 |  |  | 12844 | 0.001 | 0.3098 | No |
| 86 | PPP1R3C |  |  | 13583 | -0.010 | 0.2742 | No |
| 87 | CXCL12 |  |  | 14323 | -0.021 | 0.2391 | No |
| 88 | ZNF217 |  |  | 14331 | -0.021 | 0.2396 | No |
| 89 | HSPB6 |  |  | 14612 | -0.025 | 0.2270 | No |
| 90 | TCF21 |  |  | 14736 | -0.027 | 0.2222 | No |
| 91 | COLEC10 |  |  | 14769 | -0.028 | 0.2218 | No |
| 92 | CDC42EP2 |  |  | 14838 | -0.029 | 0.2197 | No |
| 93 | ZNF187 |  |  | 14926 | -0.030 | 0.2167 | No |
| 94 | PIWIL1 |  |  | 15527 | -0.040 | 0.1891 | No |
| 95 | NFATC4 |  |  | 15760 | -0.044 | 0.1797 | No |
| 96 | RNF144 |  |  | 16165 | -0.052 | 0.1622 | No |
| 97 | C16ORF51 |  |  | 16268 | -0.054 | 0.1595 | No |
| 98 | MN1 |  |  | 16342 | -0.056 | 0.1582 | No |
| 99 | PIK3R4 |  |  | 16535 | -0.060 | 0.1514 | No |
| 100 | JARID1B |  |  | 16557 | -0.060 | 0.1529 | No |
| 101 | SNAI2 |  |  | 16833 | -0.066 | 0.1423 | No |
| 102 | BCL7A |  |  | 17625 | -0.088 | 0.1074 | No |
| 103 | ARPC1A |  |  | 17798 | -0.092 | 0.1029 | No |
| 104 | ABCA6 |  |  | 18099 | -0.102 | 0.0926 | No |
| 105 | DDIT4 |  |  | 18623 | -0.123 | 0.0722 | No |
| 106 | CDC25B |  |  | 18779 | -0.130 | 0.0701 | No |
| 107 | RRS1 |  |  | 19234 | -0.156 | 0.0546 | No |
| 108 | NR1H3 |  |  | 20189 | -0.291 | 0.0203 | No |
Table: GSEA details [plain text format]

  

Fig 2: CMV-UV\_HCMV\_6HRS\_DN: Random ES distribution      
 Gene set null distribution of ES for **CMV-UV\_HCMV\_6HRS\_DN**

  
