# Supplementary material for: The rapamycin-regulated gene expression signature determines prognosis for breast cancer
Source: Mol Cancer. 2009 Sep 24;8:75. doi: 10.1186/1476-4598-8-75 (PMC2761377; doi:10.1186/1476-4598-8-75)
Supplement: Additional file 2 — Gene set enrichment analysis of in vivo data, time series. The data provided represent the time series of GSEA. This compressed file contains "Time" shortcut file and "GSEA_time" folder. Clicking on "Time" shortcut opens the index file providing access to analysis files contained in the "GSEA_time" folder. [file 1476-4598-8-75-S2.zip › GSEA_time/CMV_HCMV_TIMECOURSE_14HRS_DN.html]

Details for gene set CMV\_HCMV\_TIMECOURSE\_14HRS\_DN[GSEA]

|  || Dataset | gsea\_time\_collapsed |
| Phenotype | NoPhenotypeAvailable |
| Upregulated in class | na\_pos |
| GeneSet | CMV\_HCMV\_TIMECOURSE\_14HRS\_DN |
| Enrichment Score (ES) | 0.6470232 |
| Normalized Enrichment Score (NES) | 1.7357309 |
| Nominal p-value | 0.0 |
| FDR q-value | 0.0137923 |
| FWER p-Value | 0.486 |
Table: GSEA Results Summary

  

Fig 1: Enrichment plot: CMV\_HCMV\_TIMECOURSE\_14HRS\_DN      
 Profile of the Running ES Score & Positions of GeneSet Members on the Rank Ordered List

  

| PROBE | GENE SYMBOL | GENE\_TITLE | RANK IN GENE LIST | RANK METRIC SCORE | RUNNING ES | CORE ENRICHMENT || 1 | EMP1 |  |  | 15 | 1.549 | 0.1430 | Yes |
| 2 | TRIM2 |  |  | 76 | 0.965 | 0.2296 | Yes |
| 3 | NR2F2 |  |  | 115 | 0.875 | 0.3089 | Yes |
| 4 | PMP22 |  |  | 191 | 0.740 | 0.3739 | Yes |
| 5 | HNRPDL |  |  | 207 | 0.717 | 0.4396 | Yes |
| 6 | MID1 |  |  | 400 | 0.582 | 0.4843 | Yes |
| 7 | TBC1D8 |  |  | 553 | 0.514 | 0.5246 | Yes |
| 8 | ADM |  |  | 932 | 0.417 | 0.5449 | Yes |
| 9 | ETV1 |  |  | 1427 | 0.338 | 0.5522 | Yes |
| 10 | ARHGAP29 |  |  | 1593 | 0.319 | 0.5738 | Yes |
| 11 | SMAD6 |  |  | 1630 | 0.315 | 0.6013 | Yes |
| 12 | TRAF3IP2 |  |  | 1682 | 0.310 | 0.6276 | Yes |
| 13 | ACSL3 |  |  | 1999 | 0.284 | 0.6386 | Yes |
| 14 | ANP32A |  |  | 2538 | 0.245 | 0.6351 | Yes |
| 15 | IGF2BP3 |  |  | 3018 | 0.218 | 0.6321 | Yes |
| 16 | MAP3K5 |  |  | 3190 | 0.209 | 0.6432 | Yes |
| 17 | C11ORF41 |  |  | 3484 | 0.195 | 0.6470 | Yes |
| 18 | AHR |  |  | 4047 | 0.172 | 0.6356 | No |
| 19 | STK17A |  |  | 6186 | 0.109 | 0.5417 | No |
| 20 | DENND3 |  |  | 6606 | 0.099 | 0.5306 | No |
| 21 | ARNT2 |  |  | 6940 | 0.092 | 0.5229 | No |
| 22 | TP53I3 |  |  | 7036 | 0.090 | 0.5267 | No |
| 23 | BAG2 |  |  | 7222 | 0.087 | 0.5258 | No |
| 24 | PCF11 |  |  | 7637 | 0.079 | 0.5130 | No |
| 25 | FAS |  |  | 7653 | 0.079 | 0.5196 | No |
| 26 | LDB2 |  |  | 9047 | 0.056 | 0.4571 | No |
| 27 | GABBR2 |  |  | 10240 | 0.038 | 0.4027 | No |
| 28 | SYNE1 |  |  | 10896 | 0.029 | 0.3735 | No |
| 29 | PRIM2A |  |  | 11241 | 0.025 | 0.3591 | No |
| 30 | ARHGEF17 |  |  | 11814 | 0.016 | 0.3328 | No |
| 31 | NR1D2 |  |  | 11988 | 0.014 | 0.3256 | No |
| 32 | IRS1 |  |  | 12296 | 0.010 | 0.3116 | No |
| 33 | UBL3 |  |  | 14647 | -0.026 | 0.1997 | No |
| 34 | ICK |  |  | 15067 | -0.033 | 0.1824 | No |
| 35 | FGF5 |  |  | 15342 | -0.037 | 0.1725 | No |
| 36 | C6ORF32 |  |  | 16836 | -0.066 | 0.1061 | No |
| 37 | SIPA1 |  |  | 17991 | -0.099 | 0.0591 | No |
| 38 | APEX1 |  |  | 18220 | -0.106 | 0.0579 | No |
| 39 | BLVRB |  |  | 18773 | -0.130 | 0.0431 | No |
| 40 | ST5 |  |  | 19046 | -0.144 | 0.0432 | No |
| 41 | HSPA4 |  |  | 20344 | -0.351 | 0.0127 | No |
Table: GSEA details [plain text format]

  

Fig 2: CMV\_HCMV\_TIMECOURSE\_14HRS\_DN: Random ES distribution      
 Gene set null distribution of ES for **CMV\_HCMV\_TIMECOURSE\_14HRS\_DN**

  
