# Supplementary material for: The rapamycin-regulated gene expression signature determines prognosis for breast cancer
Source: Mol Cancer. 2009 Sep 24;8:75. doi: 10.1186/1476-4598-8-75 (PMC2761377; doi:10.1186/1476-4598-8-75)
Supplement: Additional file 2 — Gene set enrichment analysis of in vivo data, time series. The data provided represent the time series of GSEA. This compressed file contains "Time" shortcut file and "GSEA_time" folder. Clicking on "Time" shortcut opens the index file providing access to analysis files contained in the "GSEA_time" folder. [file 1476-4598-8-75-S2.zip › GSEA_time/CMV_HCMV_TIMECOURSE_6HRS_DN.html]

Details for gene set CMV\_HCMV\_TIMECOURSE\_6HRS\_DN[GSEA]

|  || Dataset | gsea\_time\_collapsed |
| Phenotype | NoPhenotypeAvailable |
| Upregulated in class | na\_pos |
| GeneSet | CMV\_HCMV\_TIMECOURSE\_6HRS\_DN |
| Enrichment Score (ES) | 0.6484313 |
| Normalized Enrichment Score (NES) | 1.7680802 |
| Nominal p-value | 0.0 |
| FDR q-value | 0.01011309 |
| FWER p-Value | 0.302 |
Table: GSEA Results Summary

  

Fig 1: Enrichment plot: CMV\_HCMV\_TIMECOURSE\_6HRS\_DN      
 Profile of the Running ES Score & Positions of GeneSet Members on the Rank Ordered List

  

| PROBE | GENE SYMBOL | GENE\_TITLE | RANK IN GENE LIST | RANK METRIC SCORE | RUNNING ES | CORE ENRICHMENT || 1 | TSC22D2 |  |  | 98 | 0.907 | 0.0754 | Yes |
| 2 | GPRC5A |  |  | 151 | 0.800 | 0.1435 | Yes |
| 3 | RBM4B |  |  | 282 | 0.666 | 0.1960 | Yes |
| 4 | C6ORF111 |  |  | 348 | 0.608 | 0.2466 | Yes |
| 5 | ZF |  |  | 351 | 0.608 | 0.3002 | Yes |
| 6 | F3 |  |  | 371 | 0.596 | 0.3519 | Yes |
| 7 | GAS1 |  |  | 498 | 0.535 | 0.3930 | Yes |
| 8 | SOCS5 |  |  | 1350 | 0.347 | 0.3823 | Yes |
| 9 | BACH1 |  |  | 1355 | 0.346 | 0.4127 | Yes |
| 10 | WEE1 |  |  | 1417 | 0.339 | 0.4396 | Yes |
| 11 | YTHDC1 |  |  | 1524 | 0.326 | 0.4632 | Yes |
| 12 | DYRK2 |  |  | 1584 | 0.320 | 0.4886 | Yes |
| 13 | CDKN1B |  |  | 1916 | 0.291 | 0.4982 | Yes |
| 14 | KLF10 |  |  | 2047 | 0.280 | 0.5166 | Yes |
| 15 | KLF6 |  |  | 2275 | 0.263 | 0.5287 | Yes |
| 16 | RANBP6 |  |  | 2359 | 0.258 | 0.5475 | Yes |
| 17 | KLF7 |  |  | 2569 | 0.244 | 0.5588 | Yes |
| 18 | MLLT11 |  |  | 2893 | 0.225 | 0.5630 | Yes |
| 19 | CRSP9 |  |  | 3052 | 0.216 | 0.5744 | Yes |
| 20 | DUSP10 |  |  | 3267 | 0.206 | 0.5822 | Yes |
| 21 | CSTF1 |  |  | 3286 | 0.204 | 0.5993 | Yes |
| 22 | KIAA0241 |  |  | 3545 | 0.192 | 0.6037 | Yes |
| 23 | TSPYL5 |  |  | 3568 | 0.191 | 0.6196 | Yes |
| 24 | ZBTB24 |  |  | 3656 | 0.189 | 0.6320 | Yes |
| 25 | PPP2R1B |  |  | 3747 | 0.185 | 0.6439 | Yes |
| 26 | RBM16 |  |  | 3972 | 0.175 | 0.6484 | Yes |
| 27 | FBXL5 |  |  | 4311 | 0.161 | 0.6462 | No |
| 28 | CSTF2T |  |  | 4715 | 0.147 | 0.6395 | No |
| 29 | ZNF629 |  |  | 4998 | 0.138 | 0.6379 | No |
| 30 | GCNT1 |  |  | 5162 | 0.133 | 0.6417 | No |
| 31 | RAD17 |  |  | 6048 | 0.112 | 0.6085 | No |
| 32 | AMOTL2 |  |  | 6429 | 0.103 | 0.5991 | No |
| 33 | MYC |  |  | 6440 | 0.103 | 0.6077 | No |
| 34 | GNRH1 |  |  | 6836 | 0.095 | 0.5969 | No |
| 35 | TSPYL4 |  |  | 7770 | 0.076 | 0.5583 | No |
| 36 | GADD45A |  |  | 8579 | 0.063 | 0.5246 | No |
| 37 | PATZ1 |  |  | 8771 | 0.061 | 0.5206 | No |
| 38 | RUNX1T1 |  |  | 10223 | 0.038 | 0.4534 | No |
| 39 | CITED2 |  |  | 10789 | 0.030 | 0.4286 | No |
| 40 | ADORA2B |  |  | 10853 | 0.029 | 0.4281 | No |
| 41 | PRKAB1 |  |  | 11671 | 0.018 | 0.3900 | No |
| 42 | CYR61 |  |  | 11770 | 0.017 | 0.3867 | No |
| 43 | BHLHB2 |  |  | 12438 | 0.008 | 0.3549 | No |
| 44 | HMGB2 |  |  | 13259 | -0.005 | 0.3155 | No |
| 45 | SKIV2L |  |  | 13631 | -0.011 | 0.2984 | No |
| 46 | NTF3 |  |  | 13958 | -0.015 | 0.2839 | No |
| 47 | ZNF217 |  |  | 14331 | -0.021 | 0.2676 | No |
| 48 | GTF2E1 |  |  | 14470 | -0.023 | 0.2629 | No |
| 49 | UMPS |  |  | 15453 | -0.039 | 0.2186 | No |
| 50 | SNAI2 |  |  | 16833 | -0.066 | 0.1574 | No |
| 51 | SNUPN |  |  | 18962 | -0.139 | 0.0661 | No |
| 52 | RRS1 |  |  | 19234 | -0.156 | 0.0667 | No |
Table: GSEA details [plain text format]

  

Fig 2: CMV\_HCMV\_TIMECOURSE\_6HRS\_DN: Random ES distribution      
 Gene set null distribution of ES for **CMV\_HCMV\_TIMECOURSE\_6HRS\_DN**

  
