# Supplementary material for: The rapamycin-regulated gene expression signature determines prognosis for breast cancer
Source: Mol Cancer. 2009 Sep 24;8:75. doi: 10.1186/1476-4598-8-75 (PMC2761377; doi:10.1186/1476-4598-8-75)
Supplement: Additional file 2 — Gene set enrichment analysis of in vivo data, time series. The data provided represent the time series of GSEA. This compressed file contains "Time" shortcut file and "GSEA_time" folder. Clicking on "Time" shortcut opens the index file providing access to analysis files contained in the "GSEA_time" folder. [file 1476-4598-8-75-S2.zip › GSEA_time/CORDERO_KRAS_KD_VS_CONTROL_UP.html]

Details for gene set CORDERO\_KRAS\_KD\_VS\_CONTROL\_UP[GSEA]

|  || Dataset | gsea\_time\_collapsed |
| Phenotype | NoPhenotypeAvailable |
| Upregulated in class | na\_pos |
| GeneSet | CORDERO\_KRAS\_KD\_VS\_CONTROL\_UP |
| Enrichment Score (ES) | 0.6576071 |
| Normalized Enrichment Score (NES) | 1.8644277 |
| Nominal p-value | 0.0 |
| FDR q-value | 0.0031814359 |
| FWER p-Value | 0.038 |
Table: GSEA Results Summary

  

Fig 1: Enrichment plot: CORDERO\_KRAS\_KD\_VS\_CONTROL\_UP      
 Profile of the Running ES Score & Positions of GeneSet Members on the Rank Ordered List

  

| PROBE | GENE SYMBOL | GENE\_TITLE | RANK IN GENE LIST | RANK METRIC SCORE | RUNNING ES | CORE ENRICHMENT || 1 | IGFBP7 |  |  | 2 | 2.165 | 0.1054 | Yes |
| 2 | CCL2 |  |  | 11 | 1.629 | 0.1845 | Yes |
| 3 | CALD1 |  |  | 72 | 0.980 | 0.2293 | Yes |
| 4 | TAGLN |  |  | 95 | 0.911 | 0.2726 | Yes |
| 5 | PLEKHC1 |  |  | 129 | 0.852 | 0.3125 | Yes |
| 6 | SGK |  |  | 172 | 0.769 | 0.3480 | Yes |
| 7 | PGLS |  |  | 484 | 0.539 | 0.3591 | Yes |
| 8 | LAMP2 |  |  | 564 | 0.511 | 0.3802 | Yes |
| 9 | TGFB2 |  |  | 630 | 0.490 | 0.4009 | Yes |
| 10 | SERPINE2 |  |  | 849 | 0.433 | 0.4114 | Yes |
| 11 | TPM1 |  |  | 870 | 0.428 | 0.4313 | Yes |
| 12 | COL4A2 |  |  | 901 | 0.423 | 0.4504 | Yes |
| 13 | SOX4 |  |  | 910 | 0.421 | 0.4706 | Yes |
| 14 | ITGAV |  |  | 940 | 0.416 | 0.4895 | Yes |
| 15 | SPARC |  |  | 969 | 0.411 | 0.5081 | Yes |
| 16 | ATP2B4 |  |  | 974 | 0.410 | 0.5279 | Yes |
| 17 | TGFBI |  |  | 1142 | 0.379 | 0.5383 | Yes |
| 18 | DFNA5 |  |  | 1342 | 0.349 | 0.5456 | Yes |
| 19 | COL4A1 |  |  | 1344 | 0.348 | 0.5625 | Yes |
| 20 | JUN |  |  | 1522 | 0.326 | 0.5698 | Yes |
| 21 | IGFBP3 |  |  | 1528 | 0.326 | 0.5854 | Yes |
| 22 | DICER1 |  |  | 1679 | 0.310 | 0.5932 | Yes |
| 23 | TUFT1 |  |  | 1744 | 0.304 | 0.6049 | Yes |
| 24 | GLIPR1 |  |  | 1753 | 0.303 | 0.6193 | Yes |
| 25 | ADCY7 |  |  | 1786 | 0.301 | 0.6324 | Yes |
| 26 | RSU1 |  |  | 1925 | 0.290 | 0.6399 | Yes |
| 27 | SLC16A4 |  |  | 2174 | 0.271 | 0.6410 | Yes |
| 28 | P4HA2 |  |  | 2642 | 0.239 | 0.6299 | Yes |
| 29 | INPP4B |  |  | 2807 | 0.229 | 0.6331 | Yes |
| 30 | MAP3K8 |  |  | 2810 | 0.229 | 0.6442 | Yes |
| 31 | GADD45B |  |  | 2916 | 0.224 | 0.6500 | Yes |
| 32 | NCK2 |  |  | 3081 | 0.215 | 0.6525 | Yes |
| 33 | TMEPAI |  |  | 3232 | 0.207 | 0.6553 | Yes |
| 34 | PDXK |  |  | 3385 | 0.200 | 0.6576 | Yes |
| 35 | MGEA5 |  |  | 3941 | 0.176 | 0.6392 | No |
| 36 | COL1A1 |  |  | 4112 | 0.168 | 0.6391 | No |
| 37 | JAK1 |  |  | 4177 | 0.165 | 0.6440 | No |
| 38 | CHST3 |  |  | 4222 | 0.164 | 0.6499 | No |
| 39 | NALP1 |  |  | 4868 | 0.141 | 0.6253 | No |
| 40 | ACVR1B |  |  | 4923 | 0.139 | 0.6295 | No |
| 41 | SLC22A5 |  |  | 5427 | 0.126 | 0.6111 | No |
| 42 | MATN2 |  |  | 5576 | 0.122 | 0.6099 | No |
| 43 | COL7A1 |  |  | 6058 | 0.112 | 0.5919 | No |
| 44 | NCF2 |  |  | 6161 | 0.109 | 0.5922 | No |
| 45 | MUC3B |  |  | 6715 | 0.097 | 0.5700 | No |
| 46 | NPTX1 |  |  | 6768 | 0.096 | 0.5722 | No |
| 47 | C1S |  |  | 7521 | 0.081 | 0.5395 | No |
| 48 | ADAM19 |  |  | 8098 | 0.071 | 0.5149 | No |
| 49 | GABARAPL1 |  |  | 8208 | 0.069 | 0.5130 | No |
| 50 | WASPIP |  |  | 8373 | 0.067 | 0.5083 | No |
| 51 | MYL9 |  |  | 8586 | 0.063 | 0.5010 | No |
| 52 | IGSF4 |  |  | 8780 | 0.061 | 0.4946 | No |
| 53 | TIMP3 |  |  | 10734 | 0.031 | 0.4010 | No |
| 54 | BMP1 |  |  | 10951 | 0.028 | 0.3918 | No |
| 55 | ULK1 |  |  | 11083 | 0.026 | 0.3867 | No |
| 56 | DKK3 |  |  | 11768 | 0.017 | 0.3542 | No |
| 57 | CDH4 |  |  | 11834 | 0.016 | 0.3519 | No |
| 58 | EPHB2 |  |  | 12098 | 0.013 | 0.3397 | No |
| 59 | FLRT2 |  |  | 12364 | 0.009 | 0.3272 | No |
| 60 | BHLHB2 |  |  | 12438 | 0.008 | 0.3240 | No |
| 61 | SNAI1 |  |  | 12895 | 0.000 | 0.3018 | No |
| 62 | COL5A1 |  |  | 13102 | -0.003 | 0.2919 | No |
| 63 | WISP2 |  |  | 13504 | -0.009 | 0.2728 | No |
| 64 | ECE1 |  |  | 13619 | -0.010 | 0.2678 | No |
| 65 | JUNB |  |  | 13719 | -0.012 | 0.2635 | No |
| 66 | EVL |  |  | 13986 | -0.016 | 0.2513 | No |
| 67 | HSPG2 |  |  | 14854 | -0.029 | 0.2105 | No |
| 68 | MSC |  |  | 17271 | -0.077 | 0.0966 | No |
| 69 | SERPINE1 |  |  | 18412 | -0.114 | 0.0466 | No |
| 70 | CLDN4 |  |  | 18535 | -0.119 | 0.0465 | No |
| 71 | ST5 |  |  | 19046 | -0.144 | 0.0287 | No |
| 72 | GPR56 |  |  | 19992 | -0.239 | -0.0057 | No |
| 73 | DNAJB2 |  |  | 20081 | -0.260 | 0.0026 | No |
| 74 | HSPB1 |  |  | 20498 | -0.468 | 0.0052 | No |
Table: GSEA details [plain text format]

  

Fig 2: CORDERO\_KRAS\_KD\_VS\_CONTROL\_UP: Random ES distribution      
 Gene set null distribution of ES for **CORDERO\_KRAS\_KD\_VS\_CONTROL\_UP**

  
