# Supplementary material for: The rapamycin-regulated gene expression signature determines prognosis for breast cancer
Source: Mol Cancer. 2009 Sep 24;8:75. doi: 10.1186/1476-4598-8-75 (PMC2761377; doi:10.1186/1476-4598-8-75)
Supplement: Additional file 2 — Gene set enrichment analysis of in vivo data, time series. The data provided represent the time series of GSEA. This compressed file contains "Time" shortcut file and "GSEA_time" folder. Clicking on "Time" shortcut opens the index file providing access to analysis files contained in the "GSEA_time" folder. [file 1476-4598-8-75-S2.zip › GSEA_time/CROONQUIST_IL6_RAS_DN.html]

Details for gene set CROONQUIST\_IL6\_RAS\_DN[GSEA]

|  || Dataset | gsea\_time\_collapsed |
| Phenotype | NoPhenotypeAvailable |
| Upregulated in class | na\_neg |
| GeneSet | CROONQUIST\_IL6\_RAS\_DN |
| Enrichment Score (ES) | -0.5295635 |
| Normalized Enrichment Score (NES) | -1.7617117 |
| Nominal p-value | 0.006535948 |
| FDR q-value | 0.09468295 |
| FWER p-Value | 0.497 |
Table: GSEA Results Summary

  

Fig 1: Enrichment plot: CROONQUIST\_IL6\_RAS\_DN      
 Profile of the Running ES Score & Positions of GeneSet Members on the Rank Ordered List

  

| PROBE | GENE SYMBOL | GENE\_TITLE | RANK IN GENE LIST | RANK METRIC SCORE | RUNNING ES | CORE ENRICHMENT || 1 | CDC2 |  |  | 1184 | 0.373 | 0.0191 | No |
| 2 | CDKN3 |  |  | 1274 | 0.358 | 0.0883 | No |
| 3 | WEE1 |  |  | 1417 | 0.339 | 0.1509 | No |
| 4 | MKI67 |  |  | 4043 | 0.172 | 0.0586 | No |
| 5 | CDC25C |  |  | 5343 | 0.128 | 0.0217 | No |
| 6 | CCNB2 |  |  | 5447 | 0.125 | 0.0424 | No |
| 7 | OIP5 |  |  | 6340 | 0.105 | 0.0206 | No |
| 8 | CCNB1 |  |  | 12244 | 0.011 | -0.2641 | No |
| 9 | BUB1B |  |  | 13178 | -0.004 | -0.3086 | No |
| 10 | SBNO2 |  |  | 14710 | -0.027 | -0.3775 | No |
| 11 | TRAIP |  |  | 14940 | -0.031 | -0.3823 | No |
| 12 | GALE |  |  | 16035 | -0.049 | -0.4254 | No |
| 13 | SPBC25 |  |  | 17350 | -0.079 | -0.4729 | No |
| 14 | CDC20 |  |  | 18350 | -0.111 | -0.4987 | No |
| 15 | UBE2C |  |  | 18987 | -0.140 | -0.5007 | Yes |
| 16 | KIF2C |  |  | 19275 | -0.159 | -0.4821 | Yes |
| 17 | MCM3 |  |  | 19468 | -0.176 | -0.4553 | Yes |
| 18 | FOXM1 |  |  | 19610 | -0.189 | -0.4233 | Yes |
| 19 | KIF22 |  |  | 19929 | -0.229 | -0.3916 | Yes |
| 20 | EXO1 |  |  | 19934 | -0.230 | -0.3447 | Yes |
| 21 | CDC6 |  |  | 20126 | -0.270 | -0.2986 | Yes |
| 22 | MYBL2 |  |  | 20456 | -0.434 | -0.2254 | Yes |
| 23 | CENPA |  |  | 20463 | -0.438 | -0.1358 | Yes |
| 24 | POLD1 |  |  | 20588 | -0.695 | 0.0008 | Yes |
Table: GSEA details [plain text format]

  

Fig 2: CROONQUIST\_IL6\_RAS\_DN: Random ES distribution      
 Gene set null distribution of ES for **CROONQUIST\_IL6\_RAS\_DN**

  
