# Supplementary material for: The rapamycin-regulated gene expression signature determines prognosis for breast cancer
Source: Mol Cancer. 2009 Sep 24;8:75. doi: 10.1186/1476-4598-8-75 (PMC2761377; doi:10.1186/1476-4598-8-75)
Supplement: Additional file 2 — Gene set enrichment analysis of in vivo data, time series. The data provided represent the time series of GSEA. This compressed file contains "Time" shortcut file and "GSEA_time" folder. Clicking on "Time" shortcut opens the index file providing access to analysis files contained in the "GSEA_time" folder. [file 1476-4598-8-75-S2.zip › GSEA_time/CROONQUIST_IL6_STARVE_UP.html]

Details for gene set CROONQUIST\_IL6\_STARVE\_UP[GSEA]

|  || Dataset | gsea\_time\_collapsed |
| Phenotype | NoPhenotypeAvailable |
| Upregulated in class | na\_neg |
| GeneSet | CROONQUIST\_IL6\_STARVE\_UP |
| Enrichment Score (ES) | -0.4272404 |
| Normalized Enrichment Score (NES) | -1.562582 |
| Nominal p-value | 0.0076923077 |
| FDR q-value | 0.2085026 |
| FWER p-Value | 0.933 |
Table: GSEA Results Summary

  

Fig 1: Enrichment plot: CROONQUIST\_IL6\_STARVE\_UP      
 Profile of the Running ES Score & Positions of GeneSet Members on the Rank Ordered List

  

| PROBE | GENE SYMBOL | GENE\_TITLE | RANK IN GENE LIST | RANK METRIC SCORE | RUNNING ES | CORE ENRICHMENT || 1 | CDKN3 |  |  | 1274 | 0.358 | 0.0001 | No |
| 2 | PTTG1 |  |  | 1329 | 0.350 | 0.0580 | No |
| 3 | WEE1 |  |  | 1417 | 0.339 | 0.1124 | No |
| 4 | TK1 |  |  | 2459 | 0.250 | 0.1050 | No |
| 5 | CHEK1 |  |  | 2641 | 0.239 | 0.1377 | No |
| 6 | TOP2A |  |  | 3342 | 0.202 | 0.1386 | No |
| 7 | GINS1 |  |  | 4252 | 0.163 | 0.1226 | No |
| 8 | PLK4 |  |  | 4867 | 0.141 | 0.1172 | No |
| 9 | CCNB2 |  |  | 5447 | 0.125 | 0.1107 | No |
| 10 | OIP5 |  |  | 6340 | 0.105 | 0.0855 | No |
| 11 | CSNK1G1 |  |  | 6981 | 0.092 | 0.0703 | No |
| 12 | SPP1 |  |  | 7970 | 0.073 | 0.0349 | No |
| 13 | RRM2 |  |  | 9889 | 0.044 | -0.0508 | No |
| 14 | CCNB1 |  |  | 12244 | 0.011 | -0.1634 | No |
| 15 | BUB1 |  |  | 12250 | 0.011 | -0.1618 | No |
| 16 | CDC45L |  |  | 13121 | -0.003 | -0.2036 | No |
| 17 | BUB1B |  |  | 13178 | -0.004 | -0.2056 | No |
| 18 | CENPE |  |  | 13614 | -0.010 | -0.2250 | No |
| 19 | ZWINT |  |  | 15930 | -0.047 | -0.3294 | No |
| 20 | KIF11 |  |  | 15959 | -0.048 | -0.3225 | No |
| 21 | MAD2L1 |  |  | 16264 | -0.054 | -0.3280 | No |
| 22 | SPBC25 |  |  | 17350 | -0.079 | -0.3670 | No |
| 23 | CDC20 |  |  | 18350 | -0.111 | -0.3963 | No |
| 24 | UBE2C |  |  | 18987 | -0.140 | -0.4029 | Yes |
| 25 | KIF2C |  |  | 19275 | -0.159 | -0.3894 | Yes |
| 26 | KIFC1 |  |  | 19315 | -0.162 | -0.3634 | Yes |
| 27 | FOXM1 |  |  | 19610 | -0.189 | -0.3449 | Yes |
| 28 | LIG1 |  |  | 19746 | -0.205 | -0.3160 | Yes |
| 29 | KIF22 |  |  | 19929 | -0.229 | -0.2852 | Yes |
| 30 | CDC6 |  |  | 20126 | -0.270 | -0.2480 | Yes |
| 31 | MYBL2 |  |  | 20456 | -0.434 | -0.1889 | Yes |
| 32 | CENPA |  |  | 20463 | -0.438 | -0.1134 | Yes |
| 33 | POLD1 |  |  | 20588 | -0.695 | 0.0008 | Yes |
Table: GSEA details [plain text format]

  

Fig 2: CROONQUIST\_IL6\_STARVE\_UP: Random ES distribution      
 Gene set null distribution of ES for **CROONQUIST\_IL6\_STARVE\_UP**

  
