# Supplementary material for: The rapamycin-regulated gene expression signature determines prognosis for breast cancer
Source: Mol Cancer. 2009 Sep 24;8:75. doi: 10.1186/1476-4598-8-75 (PMC2761377; doi:10.1186/1476-4598-8-75)
Supplement: Additional file 2 — Gene set enrichment analysis of in vivo data, time series. The data provided represent the time series of GSEA. This compressed file contains "Time" shortcut file and "GSEA_time" folder. Clicking on "Time" shortcut opens the index file providing access to analysis files contained in the "GSEA_time" folder. [file 1476-4598-8-75-S2.zip › GSEA_time/CROONQUIST_IL6_STROMA_UP.html]

Details for gene set CROONQUIST\_IL6\_STROMA\_UP[GSEA]

|  || Dataset | gsea\_time\_collapsed |
| Phenotype | NoPhenotypeAvailable |
| Upregulated in class | na\_pos |
| GeneSet | CROONQUIST\_IL6\_STROMA\_UP |
| Enrichment Score (ES) | 0.73875105 |
| Normalized Enrichment Score (NES) | 1.9500726 |
| Nominal p-value | 0.0 |
| FDR q-value | 8.03885E-4 |
| FWER p-Value | 0.0040 |
Table: GSEA Results Summary

  

Fig 1: Enrichment plot: CROONQUIST\_IL6\_STROMA\_UP      
 Profile of the Running ES Score & Positions of GeneSet Members on the Rank Ordered List

  

| PROBE | GENE SYMBOL | GENE\_TITLE | RANK IN GENE LIST | RANK METRIC SCORE | RUNNING ES | CORE ENRICHMENT || 1 | IGFBP7 |  |  | 2 | 2.165 | 0.1815 | Yes |
| 2 | RGS2 |  |  | 70 | 0.989 | 0.2612 | Yes |
| 3 | FSCN1 |  |  | 88 | 0.936 | 0.3389 | Yes |
| 4 | CXCL2 |  |  | 203 | 0.723 | 0.3940 | Yes |
| 5 | TPM2 |  |  | 322 | 0.628 | 0.4409 | Yes |
| 6 | NR4A2 |  |  | 353 | 0.607 | 0.4903 | Yes |
| 7 | MICAL2 |  |  | 374 | 0.595 | 0.5393 | Yes |
| 8 | DUSP1 |  |  | 672 | 0.479 | 0.5651 | Yes |
| 9 | NNMT |  |  | 813 | 0.442 | 0.5954 | Yes |
| 10 | ACTA2 |  |  | 923 | 0.418 | 0.6252 | Yes |
| 11 | SPARC |  |  | 969 | 0.411 | 0.6575 | Yes |
| 12 | TGFBI |  |  | 1142 | 0.379 | 0.6809 | Yes |
| 13 | IGFBP3 |  |  | 1528 | 0.326 | 0.6895 | Yes |
| 14 | TGM2 |  |  | 1588 | 0.320 | 0.7135 | Yes |
| 15 | FER1L3 |  |  | 1615 | 0.316 | 0.7388 | Yes |
| 16 | LOX |  |  | 2848 | 0.227 | 0.6979 | No |
| 17 | CTGF |  |  | 3167 | 0.211 | 0.7001 | No |
| 18 | CSPG2 |  |  | 4202 | 0.164 | 0.6637 | No |
| 19 | SULF1 |  |  | 4510 | 0.154 | 0.6617 | No |
| 20 | SLC25A4 |  |  | 4536 | 0.153 | 0.6733 | No |
| 21 | IL6 |  |  | 4954 | 0.138 | 0.6646 | No |
| 22 | COL6A1 |  |  | 5412 | 0.126 | 0.6530 | No |
| 23 | IER3 |  |  | 6090 | 0.111 | 0.6293 | No |
| 24 | SPTBN1 |  |  | 6719 | 0.097 | 0.6069 | No |
| 25 | RPS4Y1 |  |  | 7931 | 0.074 | 0.5542 | No |
| 26 | LOXL1 |  |  | 8180 | 0.070 | 0.5480 | No |
| 27 | NFKBIA |  |  | 9034 | 0.056 | 0.5113 | No |
| 28 | GABBR2 |  |  | 10240 | 0.038 | 0.4559 | No |
| 29 | LAMA4 |  |  | 10458 | 0.035 | 0.4483 | No |
| 30 | FOS |  |  | 11075 | 0.026 | 0.4206 | No |
| 31 | CCL4 |  |  | 14768 | -0.028 | 0.2434 | No |
| 32 | COL6A3 |  |  | 16068 | -0.050 | 0.1844 | No |
| 33 | NOTCH3 |  |  | 16320 | -0.055 | 0.1769 | No |
| 34 | COL1A2 |  |  | 16667 | -0.063 | 0.1653 | No |
| 35 | THBS2 |  |  | 16783 | -0.065 | 0.1651 | No |
| 36 | SERPINE1 |  |  | 18412 | -0.114 | 0.0956 | No |
| 37 | FOSB |  |  | 18812 | -0.131 | 0.0872 | No |
Table: GSEA details [plain text format]

  

Fig 2: CROONQUIST\_IL6\_STROMA\_UP: Random ES distribution      
 Gene set null distribution of ES for **CROONQUIST\_IL6\_STROMA\_UP**

  
