# Supplementary material for: The rapamycin-regulated gene expression signature determines prognosis for breast cancer
Source: Mol Cancer. 2009 Sep 24;8:75. doi: 10.1186/1476-4598-8-75 (PMC2761377; doi:10.1186/1476-4598-8-75)
Supplement: Additional file 2 — Gene set enrichment analysis of in vivo data, time series. The data provided represent the time series of GSEA. This compressed file contains "Time" shortcut file and "GSEA_time" folder. Clicking on "Time" shortcut opens the index file providing access to analysis files contained in the "GSEA_time" folder. [file 1476-4598-8-75-S2.zip › GSEA_time/CROONQUIST_RAS_STROMA_DN.html]

Details for gene set CROONQUIST\_RAS\_STROMA\_DN[GSEA]

|  || Dataset | gsea\_time\_collapsed |
| Phenotype | NoPhenotypeAvailable |
| Upregulated in class | na\_pos |
| GeneSet | CROONQUIST\_RAS\_STROMA\_DN |
| Enrichment Score (ES) | 0.79319704 |
| Normalized Enrichment Score (NES) | 1.8559402 |
| Nominal p-value | 0.0 |
| FDR q-value | 0.003370874 |
| FWER p-Value | 0.047 |
Table: GSEA Results Summary

  

Fig 1: Enrichment plot: CROONQUIST\_RAS\_STROMA\_DN      
 Profile of the Running ES Score & Positions of GeneSet Members on the Rank Ordered List

  

| PROBE | GENE SYMBOL | GENE\_TITLE | RANK IN GENE LIST | RANK METRIC SCORE | RUNNING ES | CORE ENRICHMENT || 1 | IGFBP7 |  |  | 2 | 2.165 | 0.2956 | Yes |
| 2 | FSCN1 |  |  | 88 | 0.936 | 0.4192 | Yes |
| 3 | TPM2 |  |  | 322 | 0.628 | 0.4936 | Yes |
| 4 | NR4A2 |  |  | 353 | 0.607 | 0.5750 | Yes |
| 5 | DUSP1 |  |  | 672 | 0.479 | 0.6250 | Yes |
| 6 | ACTA2 |  |  | 923 | 0.418 | 0.6700 | Yes |
| 7 | SPARC |  |  | 969 | 0.411 | 0.7240 | Yes |
| 8 | TGFBI |  |  | 1142 | 0.379 | 0.7674 | Yes |
| 9 | IGFBP3 |  |  | 1528 | 0.326 | 0.7932 | Yes |
| 10 | CTGF |  |  | 3167 | 0.211 | 0.7424 | No |
| 11 | SULF1 |  |  | 4510 | 0.154 | 0.6982 | No |
| 12 | IL6 |  |  | 4954 | 0.138 | 0.6956 | No |
| 13 | GJA1 |  |  | 5552 | 0.123 | 0.6834 | No |
| 14 | RPS4Y1 |  |  | 7931 | 0.074 | 0.5779 | No |
| 15 | FOS |  |  | 11075 | 0.026 | 0.4288 | No |
| 16 | POSTN |  |  | 14049 | -0.017 | 0.2867 | No |
| 17 | COL6A3 |  |  | 16068 | -0.050 | 0.1955 | No |
| 18 | COL1A2 |  |  | 16667 | -0.063 | 0.1750 | No |
| 19 | CD81 |  |  | 18539 | -0.119 | 0.1004 | No |
Table: GSEA details [plain text format]

  

Fig 2: CROONQUIST\_RAS\_STROMA\_DN: Random ES distribution      
 Gene set null distribution of ES for **CROONQUIST\_RAS\_STROMA\_DN**

  
