# Supplementary material for: The rapamycin-regulated gene expression signature determines prognosis for breast cancer
Source: Mol Cancer. 2009 Sep 24;8:75. doi: 10.1186/1476-4598-8-75 (PMC2761377; doi:10.1186/1476-4598-8-75)
Supplement: Additional file 2 — Gene set enrichment analysis of in vivo data, time series. The data provided represent the time series of GSEA. This compressed file contains "Time" shortcut file and "GSEA_time" folder. Clicking on "Time" shortcut opens the index file providing access to analysis files contained in the "GSEA_time" folder. [file 1476-4598-8-75-S2.zip › GSEA_time/DIAB_NEPH_DN.html]

Details for gene set DIAB\_NEPH\_DN[GSEA]

|  || Dataset | gsea\_time\_collapsed |
| Phenotype | NoPhenotypeAvailable |
| Upregulated in class | na\_pos |
| GeneSet | DIAB\_NEPH\_DN |
| Enrichment Score (ES) | 0.6019783 |
| Normalized Enrichment Score (NES) | 1.8469528 |
| Nominal p-value | 0.0 |
| FDR q-value | 0.0039503346 |
| FWER p-Value | 0.062 |
Table: GSEA Results Summary

  

Fig 1: Enrichment plot: DIAB\_NEPH\_DN      
 Profile of the Running ES Score & Positions of GeneSet Members on the Rank Ordered List

  

| PROBE | GENE SYMBOL | GENE\_TITLE | RANK IN GENE LIST | RANK METRIC SCORE | RUNNING ES | CORE ENRICHMENT || 1 | IGFBP7 |  |  | 2 | 2.165 | 0.0245 | Yes |
| 2 | MYLK |  |  | 5 | 1.990 | 0.0471 | Yes |
| 3 | CRIM1 |  |  | 16 | 1.496 | 0.0636 | Yes |
| 4 | ARF6 |  |  | 55 | 1.045 | 0.0736 | Yes |
| 5 | BIRC3 |  |  | 61 | 1.013 | 0.0849 | Yes |
| 6 | CALD1 |  |  | 72 | 0.980 | 0.0955 | Yes |
| 7 | PDGFA |  |  | 74 | 0.971 | 0.1065 | Yes |
| 8 | NTRK2 |  |  | 79 | 0.956 | 0.1172 | Yes |
| 9 | NEDD9 |  |  | 100 | 0.905 | 0.1265 | Yes |
| 10 | DLG5 |  |  | 112 | 0.881 | 0.1360 | Yes |
| 11 | PRKAR2B |  |  | 122 | 0.868 | 0.1454 | Yes |
| 12 | PLEKHC1 |  |  | 129 | 0.852 | 0.1548 | Yes |
| 13 | RHEB |  |  | 140 | 0.819 | 0.1636 | Yes |
| 14 | GPRC5A |  |  | 151 | 0.800 | 0.1722 | Yes |
| 15 | NBN |  |  | 159 | 0.786 | 0.1808 | Yes |
| 16 | CXCL2 |  |  | 203 | 0.723 | 0.1869 | Yes |
| 17 | HNRPDL |  |  | 207 | 0.717 | 0.1949 | Yes |
| 18 | CROP |  |  | 212 | 0.710 | 0.2028 | Yes |
| 19 | CD47 |  |  | 228 | 0.697 | 0.2099 | Yes |
| 20 | VEGF |  |  | 252 | 0.681 | 0.2165 | Yes |
| 21 | PAM |  |  | 257 | 0.679 | 0.2241 | Yes |
| 22 | CSDA |  |  | 274 | 0.669 | 0.2309 | Yes |
| 23 | IFITM1 |  |  | 275 | 0.669 | 0.2385 | Yes |
| 24 | UGCG |  |  | 335 | 0.617 | 0.2426 | Yes |
| 25 | TOMM34 |  |  | 349 | 0.608 | 0.2489 | Yes |
| 26 | CDC42EP3 |  |  | 350 | 0.608 | 0.2558 | Yes |
| 27 | CFLAR |  |  | 360 | 0.601 | 0.2622 | Yes |
| 28 | F3 |  |  | 371 | 0.596 | 0.2685 | Yes |
| 29 | NEK7 |  |  | 386 | 0.591 | 0.2745 | Yes |
| 30 | HSP90B1 |  |  | 416 | 0.573 | 0.2796 | Yes |
| 31 | DNAJB9 |  |  | 422 | 0.569 | 0.2858 | Yes |
| 32 | PTGER4 |  |  | 453 | 0.552 | 0.2906 | Yes |
| 33 | RGS10 |  |  | 456 | 0.552 | 0.2968 | Yes |
| 34 | GAS1 |  |  | 498 | 0.535 | 0.3008 | Yes |
| 35 | TRIB2 |  |  | 573 | 0.507 | 0.3029 | Yes |
| 36 | NDRG1 |  |  | 599 | 0.500 | 0.3074 | Yes |
| 37 | TOMM20 |  |  | 602 | 0.499 | 0.3130 | Yes |
| 38 | MXRA7 |  |  | 655 | 0.483 | 0.3159 | Yes |
| 39 | DUSP1 |  |  | 672 | 0.479 | 0.3206 | Yes |
| 40 | PDE4B |  |  | 685 | 0.476 | 0.3254 | Yes |
| 41 | CDKN1C |  |  | 693 | 0.475 | 0.3304 | Yes |
| 42 | SERINC5 |  |  | 719 | 0.470 | 0.3346 | Yes |
| 43 | STX7 |  |  | 727 | 0.469 | 0.3395 | Yes |
| 44 | PODXL |  |  | 755 | 0.461 | 0.3435 | Yes |
| 45 | FAT |  |  | 757 | 0.461 | 0.3487 | Yes |
| 46 | NAB1 |  |  | 769 | 0.458 | 0.3533 | Yes |
| 47 | LANCL1 |  |  | 774 | 0.455 | 0.3583 | Yes |
| 48 | DDX17 |  |  | 778 | 0.454 | 0.3633 | Yes |
| 49 | FBXO21 |  |  | 784 | 0.452 | 0.3682 | Yes |
| 50 | DSTN |  |  | 840 | 0.436 | 0.3704 | Yes |
| 51 | GNE |  |  | 890 | 0.425 | 0.3729 | Yes |
| 52 | ADM |  |  | 932 | 0.417 | 0.3756 | Yes |
| 53 | ITGAV |  |  | 940 | 0.416 | 0.3800 | Yes |
| 54 | NAPG |  |  | 945 | 0.415 | 0.3845 | Yes |
| 55 | CALU |  |  | 952 | 0.414 | 0.3889 | Yes |
| 56 | DNM1L |  |  | 970 | 0.411 | 0.3927 | Yes |
| 57 | FAM98A |  |  | 1017 | 0.402 | 0.3950 | Yes |
| 58 | WWP1 |  |  | 1018 | 0.402 | 0.3996 | Yes |
| 59 | BIRC2 |  |  | 1020 | 0.402 | 0.4041 | Yes |
| 60 | NPTN |  |  | 1026 | 0.401 | 0.4084 | Yes |
| 61 | BCAR3 |  |  | 1036 | 0.399 | 0.4125 | Yes |
| 62 | CD55 |  |  | 1041 | 0.398 | 0.4169 | Yes |
| 63 | CTBP1 |  |  | 1057 | 0.395 | 0.4206 | Yes |
| 64 | SEPT10 |  |  | 1081 | 0.391 | 0.4239 | Yes |
| 65 | ARHGAP5 |  |  | 1106 | 0.385 | 0.4271 | Yes |
| 66 | SUB1 |  |  | 1121 | 0.382 | 0.4308 | Yes |
| 67 | CAST |  |  | 1143 | 0.379 | 0.4340 | Yes |
| 68 | CAMSAP1L1 |  |  | 1144 | 0.379 | 0.4383 | Yes |
| 69 | PAK1 |  |  | 1156 | 0.378 | 0.4421 | Yes |
| 70 | TAX1BP1 |  |  | 1168 | 0.376 | 0.4458 | Yes |
| 71 | SCHIP1 |  |  | 1171 | 0.375 | 0.4500 | Yes |
| 72 | IARS |  |  | 1186 | 0.373 | 0.4535 | Yes |
| 73 | PDLIM5 |  |  | 1228 | 0.365 | 0.4557 | Yes |
| 74 | ACBD3 |  |  | 1250 | 0.363 | 0.4588 | Yes |
| 75 | PLS3 |  |  | 1277 | 0.358 | 0.4615 | Yes |
| 76 | HMGN3 |  |  | 1282 | 0.357 | 0.4654 | Yes |
| 77 | CALM2 |  |  | 1318 | 0.351 | 0.4677 | Yes |
| 78 | KIF5B |  |  | 1377 | 0.343 | 0.4687 | Yes |
| 79 | FNBP1L |  |  | 1411 | 0.340 | 0.4709 | Yes |
| 80 | MORC3 |  |  | 1479 | 0.332 | 0.4714 | Yes |
| 81 | ATXN1 |  |  | 1489 | 0.330 | 0.4747 | Yes |
| 82 | FGFR2 |  |  | 1536 | 0.325 | 0.4761 | Yes |
| 83 | TOB1 |  |  | 1542 | 0.324 | 0.4796 | Yes |
| 84 | AASDHPPT |  |  | 1570 | 0.321 | 0.4819 | Yes |
| 85 | N4BP1 |  |  | 1576 | 0.320 | 0.4853 | Yes |
| 86 | GYG1 |  |  | 1598 | 0.319 | 0.4879 | Yes |
| 87 | PLSCR1 |  |  | 1622 | 0.315 | 0.4903 | Yes |
| 88 | EPS15 |  |  | 1632 | 0.315 | 0.4935 | Yes |
| 89 | PTPN11 |  |  | 1675 | 0.311 | 0.4949 | Yes |
| 90 | AKAP10 |  |  | 1681 | 0.310 | 0.4982 | Yes |
| 91 | PCMT1 |  |  | 1709 | 0.307 | 0.5004 | Yes |
| 92 | PAFAH1B1 |  |  | 1731 | 0.305 | 0.5028 | Yes |
| 93 | PALLD |  |  | 1754 | 0.303 | 0.5052 | Yes |
| 94 | TLK1 |  |  | 1757 | 0.303 | 0.5085 | Yes |
| 95 | ARL1 |  |  | 1759 | 0.303 | 0.5119 | Yes |
| 96 | PLCG2 |  |  | 1781 | 0.301 | 0.5143 | Yes |
| 97 | KIAA0232 |  |  | 1789 | 0.300 | 0.5174 | Yes |
| 98 | PBEF1 |  |  | 1807 | 0.299 | 0.5199 | Yes |
| 99 | ITGB5 |  |  | 1900 | 0.293 | 0.5187 | Yes |
| 100 | SET |  |  | 1914 | 0.291 | 0.5214 | Yes |
| 101 | TLE4 |  |  | 1921 | 0.290 | 0.5244 | Yes |
| 102 | COL4A3 |  |  | 1981 | 0.285 | 0.5247 | Yes |
| 103 | PFN2 |  |  | 1990 | 0.285 | 0.5276 | Yes |
| 104 | ACSL3 |  |  | 1999 | 0.284 | 0.5304 | Yes |
| 105 | LOC151162 |  |  | 2010 | 0.284 | 0.5331 | Yes |
| 106 | RABGAP1L |  |  | 2088 | 0.277 | 0.5325 | Yes |
| 107 | CEBPD |  |  | 2123 | 0.275 | 0.5339 | Yes |
| 108 | ATP13A3 |  |  | 2146 | 0.273 | 0.5359 | Yes |
| 109 | SRGAP2 |  |  | 2229 | 0.266 | 0.5349 | Yes |
| 110 | MRCL3 |  |  | 2331 | 0.260 | 0.5329 | Yes |
| 111 | PAWR |  |  | 2337 | 0.259 | 0.5356 | Yes |
| 112 | GMFB |  |  | 2388 | 0.256 | 0.5360 | Yes |
| 113 | RDX |  |  | 2399 | 0.255 | 0.5384 | Yes |
| 114 | SDC2 |  |  | 2411 | 0.254 | 0.5408 | Yes |
| 115 | CDS1 |  |  | 2420 | 0.253 | 0.5432 | Yes |
| 116 | PRKAR1A |  |  | 2467 | 0.250 | 0.5438 | Yes |
| 117 | HNRPC |  |  | 2500 | 0.247 | 0.5450 | Yes |
| 118 | PSMA3 |  |  | 2554 | 0.244 | 0.5452 | Yes |
| 119 | VPS26A |  |  | 2604 | 0.241 | 0.5455 | Yes |
| 120 | CYP51A1 |  |  | 2618 | 0.241 | 0.5476 | Yes |
| 121 | PREPL |  |  | 2623 | 0.240 | 0.5502 | Yes |
| 122 | TBCA |  |  | 2630 | 0.240 | 0.5526 | Yes |
| 123 | DUSP14 |  |  | 2651 | 0.239 | 0.5543 | Yes |
| 124 | SKP1A |  |  | 2693 | 0.236 | 0.5550 | Yes |
| 125 | CD164 |  |  | 2696 | 0.236 | 0.5576 | Yes |
| 126 | SMARCA2 |  |  | 2755 | 0.233 | 0.5573 | Yes |
| 127 | GTF2A2 |  |  | 2758 | 0.233 | 0.5599 | Yes |
| 128 | ING1 |  |  | 2767 | 0.232 | 0.5621 | Yes |
| 129 | CXADR |  |  | 2770 | 0.232 | 0.5647 | Yes |
| 130 | SMARCC1 |  |  | 2815 | 0.229 | 0.5651 | Yes |
| 131 | SART3 |  |  | 2828 | 0.228 | 0.5671 | Yes |
| 132 | OPTN |  |  | 2842 | 0.228 | 0.5691 | Yes |
| 133 | KLHL9 |  |  | 2849 | 0.227 | 0.5713 | Yes |
| 134 | ACTG1 |  |  | 2865 | 0.227 | 0.5732 | Yes |
| 135 | EXPH5 |  |  | 2875 | 0.226 | 0.5753 | Yes |
| 136 | RAI14 |  |  | 2899 | 0.225 | 0.5767 | Yes |
| 137 | FNBP1 |  |  | 2965 | 0.221 | 0.5760 | Yes |
| 138 | DYRK1A |  |  | 2967 | 0.221 | 0.5785 | Yes |
| 139 | WDR42A |  |  | 3040 | 0.217 | 0.5774 | Yes |
| 140 | CCDC6 |  |  | 3076 | 0.215 | 0.5781 | Yes |
| 141 | CTGF |  |  | 3167 | 0.211 | 0.5761 | Yes |
| 142 | CREB3L2 |  |  | 3169 | 0.211 | 0.5784 | Yes |
| 143 | TMED10 |  |  | 3194 | 0.209 | 0.5796 | Yes |
| 144 | SSBP2 |  |  | 3203 | 0.209 | 0.5816 | Yes |
| 145 | ULK2 |  |  | 3209 | 0.208 | 0.5837 | Yes |
| 146 | ZC3HAV1 |  |  | 3282 | 0.205 | 0.5825 | Yes |
| 147 | LETMD1 |  |  | 3392 | 0.200 | 0.5793 | Yes |
| 148 | ATP9A |  |  | 3395 | 0.200 | 0.5815 | Yes |
| 149 | NFASC |  |  | 3400 | 0.200 | 0.5836 | Yes |
| 150 | CD2AP |  |  | 3440 | 0.198 | 0.5839 | Yes |
| 151 | SEMA3C |  |  | 3448 | 0.197 | 0.5858 | Yes |
| 152 | KIAA0143 |  |  | 3535 | 0.192 | 0.5837 | Yes |
| 153 | NFE2L1 |  |  | 3596 | 0.190 | 0.5829 | Yes |
| 154 | HNRPA1 |  |  | 3607 | 0.190 | 0.5846 | Yes |
| 155 | SPOCK2 |  |  | 3633 | 0.189 | 0.5855 | Yes |
| 156 | TRIM37 |  |  | 3675 | 0.188 | 0.5856 | Yes |
| 157 | ABI1 |  |  | 3679 | 0.188 | 0.5876 | Yes |
| 158 | CACNB2 |  |  | 3697 | 0.187 | 0.5889 | Yes |
| 159 | COL4A4 |  |  | 3706 | 0.187 | 0.5906 | Yes |
| 160 | SLK |  |  | 3744 | 0.185 | 0.5909 | Yes |
| 161 | AQP3 |  |  | 3777 | 0.184 | 0.5914 | Yes |
| 162 | SACM1L |  |  | 3797 | 0.183 | 0.5926 | Yes |
| 163 | LITAF |  |  | 3807 | 0.182 | 0.5942 | Yes |
| 164 | PLOD2 |  |  | 3812 | 0.182 | 0.5961 | Yes |
| 165 | GLUL |  |  | 3828 | 0.181 | 0.5974 | Yes |
| 166 | CANX |  |  | 3877 | 0.179 | 0.5970 | Yes |
| 167 | CTDSPL |  |  | 3921 | 0.177 | 0.5969 | Yes |
| 168 | SEPT11 |  |  | 3939 | 0.176 | 0.5981 | Yes |
| 169 | TRAM2 |  |  | 3969 | 0.175 | 0.5986 | Yes |
| 170 | MTSS1 |  |  | 3994 | 0.174 | 0.5994 | Yes |
| 171 | HMGN1 |  |  | 3995 | 0.174 | 0.6014 | Yes |
| 172 | AXL |  |  | 4024 | 0.173 | 0.6020 | Yes |
| 173 | STAT1 |  |  | 4141 | 0.167 | 0.5981 | No |
| 174 | CSPG2 |  |  | 4202 | 0.164 | 0.5971 | No |
| 175 | RAD23B |  |  | 4243 | 0.163 | 0.5969 | No |
| 176 | UBE2D2 |  |  | 4263 | 0.163 | 0.5978 | No |
| 177 | ADD3 |  |  | 4379 | 0.158 | 0.5940 | No |
| 178 | RNF11 |  |  | 4420 | 0.157 | 0.5938 | No |
| 179 | SULF1 |  |  | 4510 | 0.154 | 0.5911 | No |
| 180 | ALDH3B1 |  |  | 4546 | 0.152 | 0.5911 | No |
| 181 | PARD3 |  |  | 4848 | 0.142 | 0.5779 | No |
| 182 | GMDS |  |  | 4984 | 0.138 | 0.5727 | No |
| 183 | PAPSS1 |  |  | 5060 | 0.136 | 0.5706 | No |
| 184 | MYO1B |  |  | 5116 | 0.135 | 0.5694 | No |
| 185 | ANXA1 |  |  | 5196 | 0.132 | 0.5670 | No |
| 186 | PRKACB |  |  | 5252 | 0.130 | 0.5658 | No |
| 187 | NY-REN-7 |  |  | 5278 | 0.130 | 0.5660 | No |
| 188 | AKAP11 |  |  | 5399 | 0.126 | 0.5615 | No |
| 189 | XPO1 |  |  | 5413 | 0.126 | 0.5623 | No |
| 190 | COL4A6 |  |  | 5428 | 0.126 | 0.5630 | No |
| 191 | GJA1 |  |  | 5552 | 0.123 | 0.5583 | No |
| 192 | OAS2 |  |  | 5619 | 0.121 | 0.5565 | No |
| 193 | SNX19 |  |  | 5626 | 0.121 | 0.5575 | No |
| 194 | RSBN1 |  |  | 5716 | 0.119 | 0.5545 | No |
| 195 | MECP2 |  |  | 5751 | 0.118 | 0.5541 | No |
| 196 | ANKRD15 |  |  | 5803 | 0.117 | 0.5530 | No |
| 197 | CG018 |  |  | 5839 | 0.116 | 0.5526 | No |
| 198 | ATP2A2 |  |  | 5878 | 0.115 | 0.5520 | No |
| 199 | PPFIA4 |  |  | 5910 | 0.115 | 0.5518 | No |
| 200 | PRDX6 |  |  | 6003 | 0.113 | 0.5485 | No |
| 201 | GULP1 |  |  | 6078 | 0.111 | 0.5461 | No |
| 202 | UAP1 |  |  | 6117 | 0.110 | 0.5455 | No |
| 203 | FYN |  |  | 6142 | 0.110 | 0.5455 | No |
| 204 | GSTA4 |  |  | 6425 | 0.103 | 0.5328 | No |
| 205 | EXOC7 |  |  | 6541 | 0.101 | 0.5282 | No |
| 206 | CDC14A |  |  | 6560 | 0.101 | 0.5285 | No |
| 207 | MGAT5 |  |  | 6572 | 0.100 | 0.5291 | No |
| 208 | OTUD4 |  |  | 6746 | 0.096 | 0.5216 | No |
| 209 | RARRES2 |  |  | 6751 | 0.096 | 0.5225 | No |
| 210 | F2R |  |  | 6930 | 0.092 | 0.5148 | No |
| 211 | ARID5B |  |  | 6935 | 0.092 | 0.5156 | No |
| 212 | GHR |  |  | 6956 | 0.092 | 0.5157 | No |
| 213 | RBBP4 |  |  | 7022 | 0.091 | 0.5135 | No |
| 214 | PEX14 |  |  | 7106 | 0.089 | 0.5104 | No |
| 215 | CLIC5 |  |  | 7183 | 0.088 | 0.5077 | No |
| 216 | KIAA0350 |  |  | 7245 | 0.087 | 0.5056 | No |
| 217 | PTPRD |  |  | 7393 | 0.084 | 0.4993 | No |
| 218 | CHD3 |  |  | 7398 | 0.084 | 0.5001 | No |
| 219 | CBLB |  |  | 7419 | 0.083 | 0.5000 | No |
| 220 | XRCC5 |  |  | 7454 | 0.083 | 0.4993 | No |
| 221 | SEPT2 |  |  | 7457 | 0.083 | 0.5001 | No |
| 222 | GAB2 |  |  | 7474 | 0.083 | 0.5003 | No |
| 223 | C1S |  |  | 7521 | 0.081 | 0.4989 | No |
| 224 | PLA2R1 |  |  | 7572 | 0.080 | 0.4974 | No |
| 225 | DPYSL3 |  |  | 7667 | 0.078 | 0.4936 | No |
| 226 | PLCE1 |  |  | 7685 | 0.078 | 0.4937 | No |
| 227 | RAB21 |  |  | 7691 | 0.078 | 0.4943 | No |
| 228 | RHOBTB2 |  |  | 7742 | 0.077 | 0.4927 | No |
| 229 | TSPYL4 |  |  | 7770 | 0.076 | 0.4923 | No |
| 230 | CAPN2 |  |  | 7857 | 0.075 | 0.4889 | No |
| 231 | CORO2B |  |  | 7863 | 0.075 | 0.4895 | No |
| 232 | LOC202134 |  |  | 7910 | 0.074 | 0.4880 | No |
| 233 | HSPA12A |  |  | 7939 | 0.074 | 0.4875 | No |
| 234 | SEMA5A |  |  | 8016 | 0.072 | 0.4846 | No |
| 235 | PTHR1 |  |  | 8030 | 0.072 | 0.4847 | No |
| 236 | MCM6 |  |  | 8047 | 0.072 | 0.4848 | No |
| 237 | LOXL1 |  |  | 8180 | 0.070 | 0.4790 | No |
| 238 | CLIPR-59 |  |  | 8202 | 0.069 | 0.4788 | No |
| 239 | EXOSC7 |  |  | 8229 | 0.069 | 0.4783 | No |
| 240 | MAGI2 |  |  | 8309 | 0.068 | 0.4751 | No |
| 241 | TMED5 |  |  | 8401 | 0.066 | 0.4714 | No |
| 242 | TGFBR3 |  |  | 8482 | 0.065 | 0.4682 | No |
| 243 | MAPK10 |  |  | 8543 | 0.064 | 0.4659 | No |
| 244 | GADD45A |  |  | 8579 | 0.063 | 0.4649 | No |
| 245 | TMEM123 |  |  | 8798 | 0.060 | 0.4548 | No |
| 246 | NMI |  |  | 8892 | 0.058 | 0.4509 | No |
| 247 | ANXA2P1 |  |  | 9043 | 0.056 | 0.4441 | No |
| 248 | MXRA8 |  |  | 9077 | 0.056 | 0.4431 | No |
| 249 | HPGD |  |  | 9136 | 0.055 | 0.4409 | No |
| 250 | GRIK2 |  |  | 9247 | 0.053 | 0.4361 | No |
| 251 | DSCR1 |  |  | 9255 | 0.053 | 0.4363 | No |
| 252 | MYO1E |  |  | 9266 | 0.053 | 0.4364 | No |
| 253 | VPS13D |  |  | 9389 | 0.051 | 0.4310 | No |
| 254 | FGF9 |  |  | 9542 | 0.049 | 0.4240 | No |
| 255 | PSMB9 |  |  | 9544 | 0.049 | 0.4245 | No |
| 256 | SCRN1 |  |  | 9570 | 0.049 | 0.4238 | No |
| 257 | UBR2 |  |  | 9580 | 0.049 | 0.4240 | No |
| 258 | PCID1 |  |  | 9638 | 0.048 | 0.4217 | No |
| 259 | ST3GAL6 |  |  | 9680 | 0.047 | 0.4202 | No |
| 260 | SYNPO |  |  | 9795 | 0.045 | 0.4151 | No |
| 261 | PDIA3 |  |  | 9881 | 0.044 | 0.4114 | No |
| 262 | LRIG1 |  |  | 10086 | 0.041 | 0.4017 | No |
| 263 | TBC1D9B |  |  | 10180 | 0.039 | 0.3976 | No |
| 264 | KLK7 |  |  | 10299 | 0.038 | 0.3922 | No |
| 265 | TRIM23 |  |  | 10322 | 0.038 | 0.3915 | No |
| 266 | PLXNB2 |  |  | 10389 | 0.036 | 0.3887 | No |
| 267 | SURB7 |  |  | 10486 | 0.035 | 0.3843 | No |
| 268 | YWHAZ |  |  | 10493 | 0.035 | 0.3844 | No |
| 269 | PSMA2 |  |  | 10547 | 0.034 | 0.3822 | No |
| 270 | USP46 |  |  | 10624 | 0.033 | 0.3788 | No |
| 271 | CYP1B1 |  |  | 10729 | 0.031 | 0.3740 | No |
| 272 | CITED2 |  |  | 10789 | 0.030 | 0.3715 | No |
| 273 | LASS6 |  |  | 11028 | 0.027 | 0.3600 | No |
| 274 | DPP6 |  |  | 11054 | 0.027 | 0.3591 | No |
| 275 | ATXN10 |  |  | 11084 | 0.026 | 0.3579 | No |
| 276 | GALC |  |  | 11252 | 0.024 | 0.3500 | No |
| 277 | MTX2 |  |  | 11433 | 0.021 | 0.3413 | No |
| 278 | HIRA |  |  | 11500 | 0.021 | 0.3383 | No |
| 279 | CMAH |  |  | 11561 | 0.020 | 0.3355 | No |
| 280 | SLIT1 |  |  | 11589 | 0.019 | 0.3344 | No |
| 281 | TMEM23 |  |  | 11590 | 0.019 | 0.3346 | No |
| 282 | TOM1L2 |  |  | 11691 | 0.018 | 0.3299 | No |
| 283 | CYR61 |  |  | 11770 | 0.017 | 0.3262 | No |
| 284 | CTNNA1 |  |  | 11833 | 0.016 | 0.3234 | No |
| 285 | CASP1 |  |  | 11989 | 0.014 | 0.3159 | No |
| 286 | FOSL2 |  |  | 12117 | 0.012 | 0.3097 | No |
| 287 | DAG1 |  |  | 12130 | 0.012 | 0.3093 | No |
| 288 | SEC61G |  |  | 12179 | 0.012 | 0.3070 | No |
| 289 | IRS1 |  |  | 12296 | 0.010 | 0.3014 | No |
| 290 | HOXA9 |  |  | 12432 | 0.008 | 0.2948 | No |
| 291 | TOP2B |  |  | 12494 | 0.007 | 0.2919 | No |
| 292 | CHST2 |  |  | 12566 | 0.005 | 0.2884 | No |
| 293 | IQGAP2 |  |  | 12641 | 0.004 | 0.2848 | No |
| 294 | TIAL1 |  |  | 12659 | 0.004 | 0.2840 | No |
| 295 | OSMR |  |  | 12869 | 0.000 | 0.2737 | No |
| 296 | KIAA0960 |  |  | 12873 | 0.000 | 0.2735 | No |
| 297 | TSPAN3 |  |  | 12944 | -0.000 | 0.2701 | No |
| 298 | NEBL |  |  | 12958 | -0.000 | 0.2695 | No |
| 299 | RAB1A |  |  | 12978 | -0.001 | 0.2685 | No |
| 300 | PRKCI |  |  | 12982 | -0.001 | 0.2684 | No |
| 301 | TYRO3 |  |  | 13090 | -0.002 | 0.2631 | No |
| 302 | AGRIN |  |  | 13257 | -0.005 | 0.2550 | No |
| 303 | TNNT2 |  |  | 13260 | -0.005 | 0.2549 | No |
| 304 | NPHS1 |  |  | 13567 | -0.010 | 0.2399 | No |
| 305 | AIF1 |  |  | 13617 | -0.010 | 0.2376 | No |
| 306 | TUSC3 |  |  | 13669 | -0.011 | 0.2352 | No |
| 307 | NDN |  |  | 13980 | -0.016 | 0.2201 | No |
| 308 | POSTN |  |  | 14049 | -0.017 | 0.2169 | No |
| 309 | CRI1 |  |  | 14076 | -0.017 | 0.2158 | No |
| 310 | AASS |  |  | 14220 | -0.019 | 0.2090 | No |
| 311 | AP3B1 |  |  | 14247 | -0.020 | 0.2079 | No |
| 312 | COL3A1 |  |  | 14444 | -0.023 | 0.1985 | No |
| 313 | TCF21 |  |  | 14736 | -0.027 | 0.1844 | No |
| 314 | PTPN7 |  |  | 14756 | -0.027 | 0.1838 | No |
| 315 | LPL |  |  | 14779 | -0.028 | 0.1830 | No |
| 316 | PSMA6 |  |  | 15054 | -0.033 | 0.1698 | No |
| 317 | B3GNT1 |  |  | 15168 | -0.034 | 0.1646 | No |
| 318 | BMP7 |  |  | 15242 | -0.036 | 0.1614 | No |
| 319 | MEGF9 |  |  | 15529 | -0.040 | 0.1478 | No |
| 320 | WARS |  |  | 15538 | -0.040 | 0.1478 | No |
| 321 | ECM2 |  |  | 15763 | -0.044 | 0.1373 | No |
| 322 | HNRPA3P1 |  |  | 15928 | -0.047 | 0.1297 | No |
| 323 | TSC22D3 |  |  | 16172 | -0.052 | 0.1183 | No |
| 324 | PTPN14 |  |  | 16255 | -0.054 | 0.1148 | No |
| 325 | C3ORF37 |  |  | 16275 | -0.054 | 0.1145 | No |
| 326 | PHYHIP |  |  | 16283 | -0.054 | 0.1148 | No |
| 327 | GRK5 |  |  | 16307 | -0.055 | 0.1143 | No |
| 328 | TAPBP |  |  | 16473 | -0.059 | 0.1068 | No |
| 329 | WT1 |  |  | 16641 | -0.062 | 0.0992 | No |
| 330 | PNMA2 |  |  | 16732 | -0.064 | 0.0955 | No |
| 331 | KBTBD11 |  |  | 16776 | -0.065 | 0.0941 | No |
| 332 | ENPEP |  |  | 17013 | -0.070 | 0.0833 | No |
| 333 | USP9X |  |  | 17313 | -0.078 | 0.0694 | No |
| 334 | PHLPPL |  |  | 17330 | -0.079 | 0.0695 | No |
| 335 | ATP6AP2 |  |  | 17393 | -0.081 | 0.0673 | No |
| 336 | PPP2CB |  |  | 17427 | -0.082 | 0.0666 | No |
| 337 | ARMCX2 |  |  | 17550 | -0.085 | 0.0616 | No |
| 338 | COLQ |  |  | 17663 | -0.088 | 0.0570 | No |
| 339 | C17ORF25 |  |  | 17893 | -0.096 | 0.0468 | No |
| 340 | BST2 |  |  | 17902 | -0.096 | 0.0475 | No |
| 341 | MSH2 |  |  | 17995 | -0.099 | 0.0441 | No |
| 342 | YWHAE |  |  | 18103 | -0.102 | 0.0399 | No |
| 343 | DNAJA1 |  |  | 18123 | -0.103 | 0.0402 | No |
| 344 | DDN |  |  | 18275 | -0.109 | 0.0340 | No |
| 345 | UNG |  |  | 18333 | -0.111 | 0.0324 | No |
| 346 | SERPINE1 |  |  | 18412 | -0.114 | 0.0298 | No |
| 347 | CAND2 |  |  | 18473 | -0.116 | 0.0282 | No |
| 348 | PTPRO |  |  | 18496 | -0.117 | 0.0284 | No |
| 349 | ZNF185 |  |  | 18521 | -0.118 | 0.0286 | No |
| 350 | HSPA5 |  |  | 18613 | -0.122 | 0.0255 | No |
| 351 | APOL1 |  |  | 18686 | -0.125 | 0.0234 | No |
| 352 | ITGA3 |  |  | 18758 | -0.129 | 0.0213 | No |
| 353 | FRY |  |  | 18989 | -0.140 | 0.0115 | No |
| 354 | TEAD4 |  |  | 19054 | -0.144 | 0.0100 | No |
| 355 | APOD |  |  | 19065 | -0.145 | 0.0112 | No |
| 356 | WIT1 |  |  | 19101 | -0.146 | 0.0111 | No |
| 357 | PCK1 |  |  | 19140 | -0.150 | 0.0109 | No |
| 358 | HSP90AA1 |  |  | 19182 | -0.152 | 0.0106 | No |
| 359 | KLK6 |  |  | 19203 | -0.154 | 0.0114 | No |
| 360 | ADORA1 |  |  | 19213 | -0.154 | 0.0127 | No |
| 361 | PARP1 |  |  | 19491 | -0.177 | 0.0010 | No |
| 362 | GPNMB |  |  | 19565 | -0.185 | -0.0005 | No |
| 363 | MTHFD2 |  |  | 19664 | -0.196 | -0.0031 | No |
| 364 | UBE2L6 |  |  | 19740 | -0.204 | -0.0045 | No |
| 365 | BCAM |  |  | 19763 | -0.207 | -0.0032 | No |
| 366 | HTRA1 |  |  | 20114 | -0.266 | -0.0175 | No |
| 367 | UGP2 |  |  | 20180 | -0.289 | -0.0174 | No |
| 368 | ISG15 |  |  | 20207 | -0.298 | -0.0153 | No |
| 369 | TRIM22 |  |  | 20245 | -0.311 | -0.0136 | No |
| 370 | BMP2 |  |  | 20258 | -0.315 | -0.0106 | No |
| 371 | OAS1 |  |  | 20370 | -0.365 | -0.0119 | No |
| 372 | FLNA |  |  | 20402 | -0.383 | -0.0091 | No |
| 373 | MME |  |  | 20568 | -0.607 | -0.0103 | No |
| 374 | IGFBP2 |  |  | 20605 | -1.066 | 0.0000 | No |
Table: GSEA details [plain text format]

  

Fig 2: DIAB\_NEPH\_DN: Random ES distribution      
 Gene set null distribution of ES for **DIAB\_NEPH\_DN**

  
