# Supplementary material for: The rapamycin-regulated gene expression signature determines prognosis for breast cancer
Source: Mol Cancer. 2009 Sep 24;8:75. doi: 10.1186/1476-4598-8-75 (PMC2761377; doi:10.1186/1476-4598-8-75)
Supplement: Additional file 2 — Gene set enrichment analysis of in vivo data, time series. The data provided represent the time series of GSEA. This compressed file contains "Time" shortcut file and "GSEA_time" folder. Clicking on "Time" shortcut opens the index file providing access to analysis files contained in the "GSEA_time" folder. [file 1476-4598-8-75-S2.zip › GSEA_time/DNA_REPLICATION_REACTOME.html]

Details for gene set DNA\_REPLICATION\_REACTOME[GSEA]

|  || Dataset | gsea\_time\_collapsed |
| Phenotype | NoPhenotypeAvailable |
| Upregulated in class | na\_neg |
| GeneSet | DNA\_REPLICATION\_REACTOME |
| Enrichment Score (ES) | -0.3058597 |
| Normalized Enrichment Score (NES) | -1.2099192 |
| Nominal p-value | 0.13095239 |
| FDR q-value | 0.4044164 |
| FWER p-Value | 1.0 |
Table: GSEA Results Summary

  

Fig 1: Enrichment plot: DNA\_REPLICATION\_REACTOME      
 Profile of the Running ES Score & Positions of GeneSet Members on the Rank Ordered List

  

| PROBE | GENE SYMBOL | GENE\_TITLE | RANK IN GENE LIST | RANK METRIC SCORE | RUNNING ES | CORE ENRICHMENT || 1 | ORC6L |  |  | 1370 | 0.345 | -0.0059 | No |
| 2 | DIAPH2 |  |  | 1534 | 0.325 | 0.0433 | No |
| 3 | CDK2 |  |  | 2972 | 0.221 | 0.0123 | No |
| 4 | ORC2L |  |  | 3532 | 0.192 | 0.0190 | No |
| 5 | CDC7 |  |  | 3621 | 0.189 | 0.0480 | No |
| 6 | CDT1 |  |  | 4246 | 0.163 | 0.0464 | No |
| 7 | ORC5L |  |  | 4368 | 0.158 | 0.0684 | No |
| 8 | RPA3 |  |  | 4695 | 0.147 | 0.0784 | No |
| 9 | POLD3 |  |  | 4752 | 0.145 | 0.1012 | No |
| 10 | MCM5 |  |  | 4893 | 0.141 | 0.1192 | No |
| 11 | RFC1 |  |  | 5393 | 0.127 | 0.1173 | No |
| 12 | UBA52 |  |  | 5526 | 0.123 | 0.1325 | No |
| 13 | ORC4L |  |  | 6115 | 0.110 | 0.1233 | No |
| 14 | RFC5 |  |  | 6565 | 0.101 | 0.1192 | No |
| 15 | POLD4 |  |  | 6613 | 0.099 | 0.1343 | No |
| 16 | ORC3L |  |  | 6832 | 0.095 | 0.1404 | No |
| 17 | UBB |  |  | 6944 | 0.092 | 0.1513 | No |
| 18 | RPA1 |  |  | 7444 | 0.083 | 0.1416 | No |
| 19 | MCM6 |  |  | 8047 | 0.072 | 0.1250 | No |
| 20 | NACA |  |  | 8707 | 0.062 | 0.1038 | No |
| 21 | PRIM1 |  |  | 9406 | 0.051 | 0.0788 | No |
| 22 | POLE |  |  | 9810 | 0.045 | 0.0671 | No |
| 23 | RFC3 |  |  | 9814 | 0.045 | 0.0748 | No |
| 24 | PRIM2A |  |  | 11241 | 0.025 | 0.0097 | No |
| 25 | UBC |  |  | 11289 | 0.024 | 0.0116 | No |
| 26 | RFC4 |  |  | 11971 | 0.014 | -0.0190 | No |
| 27 | PCNA |  |  | 12857 | 0.001 | -0.0618 | No |
| 28 | RPA4 |  |  | 12904 | 0.000 | -0.0640 | No |
| 29 | CDC45L |  |  | 13121 | -0.003 | -0.0740 | No |
| 30 | POLA2 |  |  | 13786 | -0.013 | -0.1041 | No |
| 31 | MCM10 |  |  | 13909 | -0.014 | -0.1075 | No |
| 32 | POLE2 |  |  | 13961 | -0.015 | -0.1073 | No |
| 33 | MCM4 |  |  | 14787 | -0.028 | -0.1424 | No |
| 34 | POLD2 |  |  | 15700 | -0.043 | -0.1791 | No |
| 35 | RFC2 |  |  | 18307 | -0.110 | -0.2865 | Yes |
| 36 | RPS27A |  |  | 18420 | -0.114 | -0.2719 | Yes |
| 37 | ORC1L |  |  | 19002 | -0.141 | -0.2752 | Yes |
| 38 | MCM7 |  |  | 19264 | -0.157 | -0.2603 | Yes |
| 39 | RPA2 |  |  | 19317 | -0.162 | -0.2343 | Yes |
| 40 | MCM3 |  |  | 19468 | -0.176 | -0.2107 | Yes |
| 41 | GMNN |  |  | 20087 | -0.260 | -0.1949 | Yes |
| 42 | CDC6 |  |  | 20126 | -0.270 | -0.1493 | Yes |
| 43 | MCM2 |  |  | 20168 | -0.285 | -0.1011 | Yes |
| 44 | POLD1 |  |  | 20588 | -0.695 | 0.0008 | Yes |
Table: GSEA details [plain text format]

  

Fig 2: DNA\_REPLICATION\_REACTOME: Random ES distribution      
 Gene set null distribution of ES for **DNA\_REPLICATION\_REACTOME**

  
