# Supplementary material for: The rapamycin-regulated gene expression signature determines prognosis for breast cancer
Source: Mol Cancer. 2009 Sep 24;8:75. doi: 10.1186/1476-4598-8-75 (PMC2761377; doi:10.1186/1476-4598-8-75)
Supplement: Additional file 2 — Gene set enrichment analysis of in vivo data, time series. The data provided represent the time series of GSEA. This compressed file contains "Time" shortcut file and "GSEA_time" folder. Clicking on "Time" shortcut opens the index file providing access to analysis files contained in the "GSEA_time" folder. [file 1476-4598-8-75-S2.zip › GSEA_time/DNMT1_KO_DN.html]

Details for gene set DNMT1\_KO\_DN[GSEA]

|  || Dataset | gsea\_time\_collapsed |
| Phenotype | NoPhenotypeAvailable |
| Upregulated in class | na\_neg |
| GeneSet | DNMT1\_KO\_DN |
| Enrichment Score (ES) | -0.49903187 |
| Normalized Enrichment Score (NES) | -1.5232029 |
| Nominal p-value | 0.065 |
| FDR q-value | 0.20432252 |
| FWER p-Value | 0.97 |
Table: GSEA Results Summary

  

Fig 1: Enrichment plot: DNMT1\_KO\_DN      
 Profile of the Running ES Score & Positions of GeneSet Members on the Rank Ordered List

  

| PROBE | GENE SYMBOL | GENE\_TITLE | RANK IN GENE LIST | RANK METRIC SCORE | RUNNING ES | CORE ENRICHMENT || 1 | COL11A1 |  |  | 1835 | 0.297 | 0.0143 | No |
| 2 | HMGB1 |  |  | 1851 | 0.296 | 0.1167 | No |
| 3 | HPS1 |  |  | 4395 | 0.157 | 0.0479 | No |
| 4 | COL6A1 |  |  | 5412 | 0.126 | 0.0424 | No |
| 5 | DNMT1 |  |  | 5950 | 0.114 | 0.0560 | No |
| 6 | CKS1B |  |  | 6871 | 0.094 | 0.0439 | No |
| 7 | COL6A2 |  |  | 8122 | 0.071 | 0.0078 | No |
| 8 | GAS2 |  |  | 9863 | 0.044 | -0.0614 | No |
| 9 | LDLR |  |  | 11599 | 0.019 | -0.1390 | No |
| 10 | COL5A1 |  |  | 13102 | -0.003 | -0.2110 | No |
| 11 | EFNB1 |  |  | 15683 | -0.043 | -0.3214 | No |
| 12 | U2AF2 |  |  | 16361 | -0.056 | -0.3348 | No |
| 13 | H19 |  |  | 17376 | -0.080 | -0.3563 | No |
| 14 | JUP |  |  | 20316 | -0.341 | -0.3804 | Yes |
| 15 | CENPA |  |  | 20463 | -0.438 | -0.2350 | Yes |
| 16 | POLD1 |  |  | 20588 | -0.695 | 0.0008 | Yes |
Table: GSEA details [plain text format]

  

Fig 2: DNMT1\_KO\_DN: Random ES distribution      
 Gene set null distribution of ES for **DNMT1\_KO\_DN**

  
