# Supplementary material for: The rapamycin-regulated gene expression signature determines prognosis for breast cancer
Source: Mol Cancer. 2009 Sep 24;8:75. doi: 10.1186/1476-4598-8-75 (PMC2761377; doi:10.1186/1476-4598-8-75)
Supplement: Additional file 2 — Gene set enrichment analysis of in vivo data, time series. The data provided represent the time series of GSEA. This compressed file contains "Time" shortcut file and "GSEA_time" folder. Clicking on "Time" shortcut opens the index file providing access to analysis files contained in the "GSEA_time" folder. [file 1476-4598-8-75-S2.zip › GSEA_time/DORSEY_DOXYCYCLINE_UP.html]

Details for gene set DORSEY\_DOXYCYCLINE\_UP[GSEA]

|  || Dataset | gsea\_time\_collapsed |
| Phenotype | NoPhenotypeAvailable |
| Upregulated in class | na\_pos |
| GeneSet | DORSEY\_DOXYCYCLINE\_UP |
| Enrichment Score (ES) | 0.7723446 |
| Normalized Enrichment Score (NES) | 1.9560547 |
| Nominal p-value | 0.0 |
| FDR q-value | 0.0010056181 |
| FWER p-Value | 0.0030 |
Table: GSEA Results Summary

  

Fig 1: Enrichment plot: DORSEY\_DOXYCYCLINE\_UP      
 Profile of the Running ES Score & Positions of GeneSet Members on the Rank Ordered List

  

| PROBE | GENE SYMBOL | GENE\_TITLE | RANK IN GENE LIST | RANK METRIC SCORE | RUNNING ES | CORE ENRICHMENT || 1 | STC1 |  |  | 12 | 1.592 | 0.1865 | Yes |
| 2 | PHLDA2 |  |  | 68 | 0.996 | 0.3009 | Yes |
| 3 | SMAD7 |  |  | 253 | 0.680 | 0.3719 | Yes |
| 4 | ATP1B1 |  |  | 270 | 0.672 | 0.4501 | Yes |
| 5 | GNG11 |  |  | 283 | 0.666 | 0.5278 | Yes |
| 6 | TFPI2 |  |  | 563 | 0.511 | 0.5743 | Yes |
| 7 | CD44 |  |  | 641 | 0.487 | 0.6278 | Yes |
| 8 | THBS1 |  |  | 673 | 0.478 | 0.6825 | Yes |
| 9 | CRYAB |  |  | 696 | 0.475 | 0.7372 | Yes |
| 10 | SPARC |  |  | 969 | 0.411 | 0.7723 | Yes |
| 11 | AKAP12 |  |  | 2720 | 0.235 | 0.7149 | No |
| 12 | COL1A1 |  |  | 4112 | 0.168 | 0.6671 | No |
| 13 | GRIK1 |  |  | 4615 | 0.150 | 0.6603 | No |
| 14 | ARHGAP25 |  |  | 6424 | 0.103 | 0.5845 | No |
| 15 | GK |  |  | 6675 | 0.098 | 0.5839 | No |
| 16 | ITGB3 |  |  | 6725 | 0.097 | 0.5929 | No |
| 17 | GSN |  |  | 10561 | 0.034 | 0.4105 | No |
| 18 | PRSS1 |  |  | 12105 | 0.013 | 0.3370 | No |
| 19 | REN |  |  | 12918 | 0.000 | 0.2975 | No |
| 20 | CD69 |  |  | 13127 | -0.003 | 0.2878 | No |
| 21 | ACVR1 |  |  | 13608 | -0.010 | 0.2656 | No |
| 22 | ITGA2B |  |  | 14037 | -0.016 | 0.2467 | No |
| 23 | TNFRSF9 |  |  | 14598 | -0.025 | 0.2225 | No |
| 24 | PRSS3 |  |  | 15230 | -0.035 | 0.1959 | No |
| 25 | COL6A3 |  |  | 16068 | -0.050 | 0.1611 | No |
| 26 | MT4 |  |  | 17335 | -0.079 | 0.1088 | No |
| 27 | IL13RA2 |  |  | 18264 | -0.109 | 0.0765 | No |
| 28 | SERPINE1 |  |  | 18412 | -0.114 | 0.0827 | No |
| 29 | DUSP5 |  |  | 19722 | -0.202 | 0.0429 | No |
Table: GSEA details [plain text format]

  

Fig 2: DORSEY\_DOXYCYCLINE\_UP: Random ES distribution      
 Gene set null distribution of ES for **DORSEY\_DOXYCYCLINE\_UP**

  
