# Supplementary material for: The rapamycin-regulated gene expression signature determines prognosis for breast cancer
Source: Mol Cancer. 2009 Sep 24;8:75. doi: 10.1186/1476-4598-8-75 (PMC2761377; doi:10.1186/1476-4598-8-75)
Supplement: Additional file 2 — Gene set enrichment analysis of in vivo data, time series. The data provided represent the time series of GSEA. This compressed file contains "Time" shortcut file and "GSEA_time" folder. Clicking on "Time" shortcut opens the index file providing access to analysis files contained in the "GSEA_time" folder. [file 1476-4598-8-75-S2.zip › GSEA_time/DOX_RESIST_GASTRIC_DN.html]

Details for gene set DOX\_RESIST\_GASTRIC\_DN[GSEA]

|  || Dataset | gsea\_time\_collapsed |
| Phenotype | NoPhenotypeAvailable |
| Upregulated in class | na\_pos |
| GeneSet | DOX\_RESIST\_GASTRIC\_DN |
| Enrichment Score (ES) | 0.770429 |
| Normalized Enrichment Score (NES) | 1.7759751 |
| Nominal p-value | 0.0 |
| FDR q-value | 0.0092043355 |
| FWER p-Value | 0.274 |
Table: GSEA Results Summary

  

Fig 1: Enrichment plot: DOX\_RESIST\_GASTRIC\_DN      
 Profile of the Running ES Score & Positions of GeneSet Members on the Rank Ordered List

  

| PROBE | GENE SYMBOL | GENE\_TITLE | RANK IN GENE LIST | RANK METRIC SCORE | RUNNING ES | CORE ENRICHMENT || 1 | TMEM106B |  |  | 202 | 0.723 | 0.1254 | Yes |
| 2 | CLIC4 |  |  | 303 | 0.646 | 0.2414 | Yes |
| 3 | KIAA1033 |  |  | 315 | 0.635 | 0.3596 | Yes |
| 4 | C1ORF121 |  |  | 496 | 0.537 | 0.4511 | Yes |
| 5 | NFAT5 |  |  | 803 | 0.444 | 0.5194 | Yes |
| 6 | WWP1 |  |  | 1018 | 0.402 | 0.5840 | Yes |
| 7 | TJP1 |  |  | 1663 | 0.312 | 0.6110 | Yes |
| 8 | RB1CC1 |  |  | 1771 | 0.302 | 0.6623 | Yes |
| 9 | ATP2B1 |  |  | 1819 | 0.298 | 0.7158 | Yes |
| 10 | CEBPD |  |  | 2123 | 0.275 | 0.7524 | Yes |
| 11 | ASXL2 |  |  | 2667 | 0.238 | 0.7704 | Yes |
| 12 | TBL1X |  |  | 4946 | 0.139 | 0.6858 | No |
| 13 | NCOA3 |  |  | 5249 | 0.131 | 0.6956 | No |
| 14 | PCYOX1 |  |  | 6882 | 0.093 | 0.6337 | No |
| 15 | PSAT1 |  |  | 7100 | 0.089 | 0.6399 | No |
| 16 | PTP4A2 |  |  | 8083 | 0.071 | 0.6055 | No |
| 17 | NR1D2 |  |  | 11988 | 0.014 | 0.4185 | No |
Table: GSEA details [plain text format]

  

Fig 2: DOX\_RESIST\_GASTRIC\_DN: Random ES distribution      
 Gene set null distribution of ES for **DOX\_RESIST\_GASTRIC\_DN**

  
