# Supplementary material for: The rapamycin-regulated gene expression signature determines prognosis for breast cancer
Source: Mol Cancer. 2009 Sep 24;8:75. doi: 10.1186/1476-4598-8-75 (PMC2761377; doi:10.1186/1476-4598-8-75)
Supplement: Additional file 2 — Gene set enrichment analysis of in vivo data, time series. The data provided represent the time series of GSEA. This compressed file contains "Time" shortcut file and "GSEA_time" folder. Clicking on "Time" shortcut opens the index file providing access to analysis files contained in the "GSEA_time" folder. [file 1476-4598-8-75-S2.zip › GSEA_time/ELECTRON_TRANSPORTER_ACTIVITY.html]

Details for gene set ELECTRON\_TRANSPORTER\_ACTIVITY[GSEA]

|  || Dataset | gsea\_time\_collapsed |
| Phenotype | NoPhenotypeAvailable |
| Upregulated in class | na\_neg |
| GeneSet | ELECTRON\_TRANSPORTER\_ACTIVITY |
| Enrichment Score (ES) | -0.2705619 |
| Normalized Enrichment Score (NES) | -1.2535144 |
| Nominal p-value | 0.0625 |
| FDR q-value | 0.3605083 |
| FWER p-Value | 1.0 |
Table: GSEA Results Summary

  

Fig 1: Enrichment plot: ELECTRON\_TRANSPORTER\_ACTIVITY      
 Profile of the Running ES Score & Positions of GeneSet Members on the Rank Ordered List

  

| PROBE | GENE SYMBOL | GENE\_TITLE | RANK IN GENE LIST | RANK METRIC SCORE | RUNNING ES | CORE ENRICHMENT || 1 | QDPR |  |  | 148 | 0.805 | 0.0369 | No |
| 2 | TXN |  |  | 178 | 0.760 | 0.0771 | No |
| 3 | PAM |  |  | 257 | 0.679 | 0.1105 | No |
| 4 | SRD5A1 |  |  | 342 | 0.611 | 0.1399 | No |
| 5 | ASPH |  |  | 449 | 0.554 | 0.1651 | No |
| 6 | MTRR |  |  | 579 | 0.506 | 0.1865 | No |
| 7 | COX11 |  |  | 1120 | 0.382 | 0.1811 | No |
| 8 | AKR7A2 |  |  | 1535 | 0.325 | 0.1786 | No |
| 9 | BLVRA |  |  | 1710 | 0.307 | 0.1870 | No |
| 10 | KIAA1833 |  |  | 2180 | 0.270 | 0.1789 | No |
| 11 | PRDX2 |  |  | 2541 | 0.245 | 0.1748 | No |
| 12 | GRHPR |  |  | 2544 | 0.245 | 0.1881 | No |
| 13 | P4HA2 |  |  | 2642 | 0.239 | 0.1965 | No |
| 14 | ME2 |  |  | 2889 | 0.225 | 0.1968 | No |
| 15 | DHRS3 |  |  | 2930 | 0.223 | 0.2071 | No |
| 16 | KMO |  |  | 3072 | 0.215 | 0.2120 | No |
| 17 | UGDH |  |  | 3722 | 0.186 | 0.1905 | No |
| 18 | CYP24A1 |  |  | 3928 | 0.176 | 0.1902 | No |
| 19 | WWOX |  |  | 4167 | 0.166 | 0.1876 | No |
| 20 | AOC2 |  |  | 4226 | 0.164 | 0.1938 | No |
| 21 | HNRPM |  |  | 4375 | 0.158 | 0.1952 | No |
| 22 | DDO |  |  | 4767 | 0.145 | 0.1841 | No |
| 23 | UQCR |  |  | 4860 | 0.142 | 0.1874 | No |
| 24 | RRM2B |  |  | 5062 | 0.136 | 0.1850 | No |
| 25 | DHDH |  |  | 5100 | 0.135 | 0.1906 | No |
| 26 | AKR1C2 |  |  | 5425 | 0.126 | 0.1817 | No |
| 27 | PHYH |  |  | 5764 | 0.118 | 0.1717 | No |
| 28 | NCF2 |  |  | 6161 | 0.109 | 0.1583 | No |
| 29 | LOXL2 |  |  | 6308 | 0.105 | 0.1570 | No |
| 30 | ZFP90 |  |  | 6353 | 0.104 | 0.1606 | No |
| 31 | GPX5 |  |  | 6436 | 0.103 | 0.1622 | No |
| 32 | CYP1A2 |  |  | 6454 | 0.102 | 0.1670 | No |
| 33 | COX5A |  |  | 6819 | 0.095 | 0.1544 | No |
| 34 | GPX4 |  |  | 6841 | 0.094 | 0.1586 | No |
| 35 | ALDH5A1 |  |  | 6992 | 0.091 | 0.1562 | No |
| 36 | CYP19A1 |  |  | 7155 | 0.088 | 0.1532 | No |
| 37 | TXNDC5 |  |  | 7411 | 0.084 | 0.1453 | No |
| 38 | HAAO |  |  | 7661 | 0.079 | 0.1375 | No |
| 39 | PPOX |  |  | 7708 | 0.078 | 0.1395 | No |
| 40 | ALDH1A2 |  |  | 7879 | 0.075 | 0.1353 | No |
| 41 | LOXL1 |  |  | 8180 | 0.070 | 0.1245 | No |
| 42 | COX15 |  |  | 8845 | 0.059 | 0.0953 | No |
| 43 | SARDH |  |  | 8911 | 0.058 | 0.0953 | No |
| 44 | ADH5 |  |  | 9035 | 0.056 | 0.0924 | No |
| 45 | HPGD |  |  | 9136 | 0.055 | 0.0906 | No |
| 46 | NNT |  |  | 9250 | 0.053 | 0.0880 | No |
| 47 | CYCS |  |  | 9385 | 0.051 | 0.0842 | No |
| 48 | CYP39A1 |  |  | 9447 | 0.050 | 0.0840 | No |
| 49 | TXNL2 |  |  | 10109 | 0.040 | 0.0540 | No |
| 50 | ALDH4A1 |  |  | 10627 | 0.033 | 0.0306 | No |
| 51 | NQO2 |  |  | 10647 | 0.033 | 0.0314 | No |
| 52 | TSTA3 |  |  | 10651 | 0.032 | 0.0330 | No |
| 53 | CYP1B1 |  |  | 10729 | 0.031 | 0.0310 | No |
| 54 | CYP11A1 |  |  | 10774 | 0.030 | 0.0305 | No |
| 55 | ME3 |  |  | 11093 | 0.026 | 0.0164 | No |
| 56 | ETFB |  |  | 11137 | 0.026 | 0.0158 | No |
| 57 | INDO |  |  | 11609 | 0.019 | -0.0062 | No |
| 58 | H6PD |  |  | 11652 | 0.018 | -0.0072 | No |
| 59 | GPX2 |  |  | 12509 | 0.006 | -0.0486 | No |
| 60 | CYP26A1 |  |  | 12539 | 0.006 | -0.0497 | No |
| 61 | DHRS2 |  |  | 12821 | 0.001 | -0.0634 | No |
| 62 | AKR1A1 |  |  | 12872 | 0.000 | -0.0658 | No |
| 63 | NOX4 |  |  | 12879 | 0.000 | -0.0661 | No |
| 64 | ADH1B |  |  | 13211 | -0.004 | -0.0820 | No |
| 65 | MAOB |  |  | 13720 | -0.012 | -0.1061 | No |
| 66 | HSD17B8 |  |  | 13779 | -0.013 | -0.1083 | No |
| 67 | CDO1 |  |  | 13823 | -0.013 | -0.1096 | No |
| 68 | EPX |  |  | 14002 | -0.016 | -0.1174 | No |
| 69 | ADH6 |  |  | 14305 | -0.021 | -0.1310 | No |
| 70 | TXNDC2 |  |  | 14801 | -0.028 | -0.1536 | No |
| 71 | ALDH2 |  |  | 14955 | -0.031 | -0.1594 | No |
| 72 | ALOX15B |  |  | 15259 | -0.036 | -0.1723 | No |
| 73 | TXNDC3 |  |  | 15458 | -0.039 | -0.1798 | No |
| 74 | DAO |  |  | 15746 | -0.044 | -0.1914 | No |
| 75 | ADH7 |  |  | 16133 | -0.051 | -0.2074 | No |
| 76 | SRD5A2 |  |  | 16235 | -0.053 | -0.2094 | No |
| 77 | ALDH3A1 |  |  | 16424 | -0.057 | -0.2155 | No |
| 78 | ADH1C |  |  | 16543 | -0.060 | -0.2179 | No |
| 79 | GSR |  |  | 17227 | -0.076 | -0.2471 | No |
| 80 | AKR1C4 |  |  | 17475 | -0.083 | -0.2546 | No |
| 81 | HMOX2 |  |  | 17484 | -0.083 | -0.2504 | No |
| 82 | ALOX12B |  |  | 17525 | -0.085 | -0.2477 | No |
| 83 | DHRS4 |  |  | 17739 | -0.091 | -0.2532 | No |
| 84 | AKR7A3 |  |  | 18027 | -0.100 | -0.2617 | No |
| 85 | SDHD |  |  | 18044 | -0.101 | -0.2570 | No |
| 86 | TXN2 |  |  | 18068 | -0.101 | -0.2525 | No |
| 87 | AOC3 |  |  | 18080 | -0.101 | -0.2475 | No |
| 88 | ADH1A |  |  | 18090 | -0.101 | -0.2424 | No |
| 89 | ME1 |  |  | 18364 | -0.112 | -0.2496 | No |
| 90 | SPR |  |  | 18374 | -0.112 | -0.2439 | No |
| 91 | GPX3 |  |  | 18665 | -0.125 | -0.2512 | No |
| 92 | NCF4 |  |  | 19062 | -0.145 | -0.2626 | Yes |
| 93 | P4HB |  |  | 19099 | -0.146 | -0.2564 | Yes |
| 94 | CYP4F11 |  |  | 19308 | -0.161 | -0.2577 | Yes |
| 95 | AKR1D1 |  |  | 19371 | -0.167 | -0.2516 | Yes |
| 96 | ALDH1A1 |  |  | 19486 | -0.177 | -0.2474 | Yes |
| 97 | MTHFD2 |  |  | 19664 | -0.196 | -0.2453 | Yes |
| 98 | PRDX5 |  |  | 19676 | -0.197 | -0.2351 | Yes |
| 99 | QSCN6 |  |  | 19801 | -0.212 | -0.2295 | Yes |
| 100 | SDHA |  |  | 20118 | -0.266 | -0.2303 | Yes |
| 101 | IDH3B |  |  | 20127 | -0.270 | -0.2159 | Yes |
| 102 | AKR1B10 |  |  | 20167 | -0.284 | -0.2023 | Yes |
| 103 | MB |  |  | 20169 | -0.285 | -0.1867 | Yes |
| 104 | AKR1B1 |  |  | 20185 | -0.290 | -0.1715 | Yes |
| 105 | GLDC |  |  | 20246 | -0.311 | -0.1574 | Yes |
| 106 | DHCR7 |  |  | 20271 | -0.320 | -0.1410 | Yes |
| 107 | PGD |  |  | 20349 | -0.354 | -0.1254 | Yes |
| 108 | PHGDH |  |  | 20363 | -0.363 | -0.1062 | Yes |
| 109 | ALDH9A1 |  |  | 20364 | -0.363 | -0.0863 | Yes |
| 110 | AKR1C3 |  |  | 20409 | -0.394 | -0.0668 | Yes |
| 111 | PDHX |  |  | 20441 | -0.417 | -0.0455 | Yes |
| 112 | AKR1C1 |  |  | 20480 | -0.451 | -0.0227 | Yes |
| 113 | CYC1 |  |  | 20538 | -0.524 | 0.0033 | Yes |
Table: GSEA details [plain text format]

  

Fig 2: ELECTRON\_TRANSPORTER\_ACTIVITY: Random ES distribution      
 Gene set null distribution of ES for **ELECTRON\_TRANSPORTER\_ACTIVITY**

  
