# Supplementary material for: The rapamycin-regulated gene expression signature determines prognosis for breast cancer
Source: Mol Cancer. 2009 Sep 24;8:75. doi: 10.1186/1476-4598-8-75 (PMC2761377; doi:10.1186/1476-4598-8-75)
Supplement: Additional file 2 — Gene set enrichment analysis of in vivo data, time series. The data provided represent the time series of GSEA. This compressed file contains "Time" shortcut file and "GSEA_time" folder. Clicking on "Time" shortcut opens the index file providing access to analysis files contained in the "GSEA_time" folder. [file 1476-4598-8-75-S2.zip › GSEA_time/FATTY_ACID_SYNTHESIS.html]

Details for gene set FATTY\_ACID\_SYNTHESIS[GSEA]

|  || Dataset | gsea\_time\_collapsed |
| Phenotype | NoPhenotypeAvailable |
| Upregulated in class | na\_neg |
| GeneSet | FATTY\_ACID\_SYNTHESIS |
| Enrichment Score (ES) | -0.6521213 |
| Normalized Enrichment Score (NES) | -1.9614068 |
| Nominal p-value | 0.0 |
| FDR q-value | 0.067931406 |
| FWER p-Value | 0.132 |
Table: GSEA Results Summary

  

Fig 1: Enrichment plot: FATTY\_ACID\_SYNTHESIS      
 Profile of the Running ES Score & Positions of GeneSet Members on the Rank Ordered List

  

| PROBE | GENE SYMBOL | GENE\_TITLE | RANK IN GENE LIST | RANK METRIC SCORE | RUNNING ES | CORE ENRICHMENT || 1 | PRKAA1 |  |  | 2542 | 0.245 | -0.0410 | No |
| 2 | MYO5B |  |  | 3788 | 0.183 | -0.0397 | No |
| 3 | ACAA2 |  |  | 3822 | 0.182 | 0.0200 | No |
| 4 | ACACA |  |  | 6760 | 0.096 | -0.0904 | No |
| 5 | PRKAB2 |  |  | 8422 | 0.066 | -0.1489 | No |
| 6 | ACACB |  |  | 8480 | 0.065 | -0.1298 | No |
| 7 | PRKAB1 |  |  | 11671 | 0.018 | -0.2785 | No |
| 8 | PRKAA2 |  |  | 11889 | 0.015 | -0.2839 | No |
| 9 | PRKAG3 |  |  | 12606 | 0.005 | -0.3170 | No |
| 10 | PRKAG1 |  |  | 13013 | -0.001 | -0.3363 | No |
| 11 | PRKAG2 |  |  | 15127 | -0.034 | -0.4275 | No |
| 12 | ACLY |  |  | 19752 | -0.206 | -0.5828 | Yes |
| 13 | PC |  |  | 20000 | -0.241 | -0.5136 | Yes |
| 14 | SLC25A1 |  |  | 20394 | -0.380 | -0.4046 | Yes |
| 15 | SCD |  |  | 20439 | -0.416 | -0.2665 | Yes |
| 16 | FASN |  |  | 20596 | -0.815 | 0.0004 | Yes |
Table: GSEA details [plain text format]

  

Fig 2: FATTY\_ACID\_SYNTHESIS: Random ES distribution      
 Gene set null distribution of ES for **FATTY\_ACID\_SYNTHESIS**

  
