# Supplementary material for: The rapamycin-regulated gene expression signature determines prognosis for breast cancer
Source: Mol Cancer. 2009 Sep 24;8:75. doi: 10.1186/1476-4598-8-75 (PMC2761377; doi:10.1186/1476-4598-8-75)
Supplement: Additional file 2 — Gene set enrichment analysis of in vivo data, time series. The data provided represent the time series of GSEA. This compressed file contains "Time" shortcut file and "GSEA_time" folder. Clicking on "Time" shortcut opens the index file providing access to analysis files contained in the "GSEA_time" folder. [file 1476-4598-8-75-S2.zip › GSEA_time/FSH_OVARY_MCV152_DN.html]

Details for gene set FSH\_OVARY\_MCV152\_DN[GSEA]

|  || Dataset | gsea\_time\_collapsed |
| Phenotype | NoPhenotypeAvailable |
| Upregulated in class | na\_pos |
| GeneSet | FSH\_OVARY\_MCV152\_DN |
| Enrichment Score (ES) | 0.6691326 |
| Normalized Enrichment Score (NES) | 1.8257849 |
| Nominal p-value | 0.0 |
| FDR q-value | 0.0051057497 |
| FWER p-Value | 0.107 |
Table: GSEA Results Summary

  

Fig 1: Enrichment plot: FSH\_OVARY\_MCV152\_DN      
 Profile of the Running ES Score & Positions of GeneSet Members on the Rank Ordered List

  

| PROBE | GENE SYMBOL | GENE\_TITLE | RANK IN GENE LIST | RANK METRIC SCORE | RUNNING ES | CORE ENRICHMENT || 1 | EMP1 |  |  | 15 | 1.549 | 0.1477 | Yes |
| 2 | ARF6 |  |  | 55 | 1.045 | 0.2460 | Yes |
| 3 | EIF2S3 |  |  | 292 | 0.658 | 0.2975 | Yes |
| 4 | DUSP1 |  |  | 672 | 0.479 | 0.3250 | Yes |
| 5 | ARHGAP5 |  |  | 1106 | 0.385 | 0.3408 | Yes |
| 6 | SERBP1 |  |  | 1163 | 0.376 | 0.3741 | Yes |
| 7 | ZMYND11 |  |  | 1242 | 0.363 | 0.4051 | Yes |
| 8 | PRPF4B |  |  | 1303 | 0.354 | 0.4361 | Yes |
| 9 | KIF5B |  |  | 1377 | 0.343 | 0.4655 | Yes |
| 10 | SLC2A3P1 |  |  | 1579 | 0.320 | 0.4864 | Yes |
| 11 | PAK2 |  |  | 1787 | 0.301 | 0.5051 | Yes |
| 12 | UBE2G1 |  |  | 1919 | 0.291 | 0.5266 | Yes |
| 13 | ADD1 |  |  | 1959 | 0.287 | 0.5522 | Yes |
| 14 | PITPNB |  |  | 2031 | 0.281 | 0.5757 | Yes |
| 15 | SNAP23 |  |  | 2509 | 0.247 | 0.5762 | Yes |
| 16 | RANBP9 |  |  | 2686 | 0.237 | 0.5903 | Yes |
| 17 | ITGA6 |  |  | 2714 | 0.235 | 0.6115 | Yes |
| 18 | GDI2 |  |  | 3038 | 0.217 | 0.6166 | Yes |
| 19 | GNA13 |  |  | 3073 | 0.215 | 0.6355 | Yes |
| 20 | MED6 |  |  | 3241 | 0.207 | 0.6472 | Yes |
| 21 | TOP2A |  |  | 3342 | 0.202 | 0.6617 | Yes |
| 22 | RAB6A |  |  | 3795 | 0.183 | 0.6572 | Yes |
| 23 | JAK1 |  |  | 4177 | 0.165 | 0.6545 | Yes |
| 24 | CSPG2 |  |  | 4202 | 0.164 | 0.6691 | Yes |
| 25 | MAT2A |  |  | 5356 | 0.128 | 0.6253 | No |
| 26 | GNS |  |  | 5405 | 0.126 | 0.6351 | No |
| 27 | ATP5A1 |  |  | 5563 | 0.123 | 0.6392 | No |
| 28 | DDX3X |  |  | 6332 | 0.105 | 0.6119 | No |
| 29 | PRPS1 |  |  | 7885 | 0.075 | 0.5435 | No |
| 30 | GPR176 |  |  | 8257 | 0.069 | 0.5321 | No |
| 31 | RAF1 |  |  | 8772 | 0.061 | 0.5129 | No |
| 32 | RRM2 |  |  | 9889 | 0.044 | 0.4628 | No |
| 33 | FN1 |  |  | 9897 | 0.043 | 0.4666 | No |
| 34 | CCNF |  |  | 9943 | 0.043 | 0.4685 | No |
| 35 | CDK8 |  |  | 10429 | 0.036 | 0.4483 | No |
| 36 | YWHAZ |  |  | 10493 | 0.035 | 0.4486 | No |
| 37 | CYB5B |  |  | 11608 | 0.019 | 0.3963 | No |
| 38 | TFDP1 |  |  | 12379 | 0.009 | 0.3596 | No |
| 39 | TOP2B |  |  | 12494 | 0.007 | 0.3547 | No |
| 40 | TEGT |  |  | 12537 | 0.006 | 0.3532 | No |
| 41 | BRCA1 |  |  | 12699 | 0.003 | 0.3457 | No |
| 42 | OSMR |  |  | 12869 | 0.000 | 0.3376 | No |
| 43 | FGF5 |  |  | 15342 | -0.037 | 0.2209 | No |
| 44 | FGF2 |  |  | 17847 | -0.094 | 0.1081 | No |
| 45 | SERPINE1 |  |  | 18412 | -0.114 | 0.0916 | No |
| 46 | MAP2K1 |  |  | 19255 | -0.157 | 0.0657 | No |
Table: GSEA details [plain text format]

  

Fig 2: FSH\_OVARY\_MCV152\_DN: Random ES distribution      
 Gene set null distribution of ES for **FSH\_OVARY\_MCV152\_DN**

  
