# Supplementary material for: The rapamycin-regulated gene expression signature determines prognosis for breast cancer
Source: Mol Cancer. 2009 Sep 24;8:75. doi: 10.1186/1476-4598-8-75 (PMC2761377; doi:10.1186/1476-4598-8-75)
Supplement: Additional file 2 — Gene set enrichment analysis of in vivo data, time series. The data provided represent the time series of GSEA. This compressed file contains "Time" shortcut file and "GSEA_time" folder. Clicking on "Time" shortcut opens the index file providing access to analysis files contained in the "GSEA_time" folder. [file 1476-4598-8-75-S2.zip › GSEA_time/GALE_FLT3ANDAPL_DN.html]

Details for gene set GALE\_FLT3ANDAPL\_DN[GSEA]

|  || Dataset | gsea\_time\_collapsed |
| Phenotype | NoPhenotypeAvailable |
| Upregulated in class | na\_neg |
| GeneSet | GALE\_FLT3ANDAPL\_DN |
| Enrichment Score (ES) | -0.45489118 |
| Normalized Enrichment Score (NES) | -1.4700832 |
| Nominal p-value | 0.097826086 |
| FDR q-value | 0.24902448 |
| FWER p-Value | 0.992 |
Table: GSEA Results Summary

  

Fig 1: Enrichment plot: GALE\_FLT3ANDAPL\_DN      
 Profile of the Running ES Score & Positions of GeneSet Members on the Rank Ordered List

  

| PROBE | GENE SYMBOL | GENE\_TITLE | RANK IN GENE LIST | RANK METRIC SCORE | RUNNING ES | CORE ENRICHMENT || 1 | NAPG |  |  | 945 | 0.415 | 0.1090 | No |
| 2 | DEGS1 |  |  | 2553 | 0.245 | 0.1223 | No |
| 3 | DRAP1 |  |  | 5080 | 0.135 | 0.0500 | No |
| 4 | ARF1 |  |  | 6562 | 0.101 | 0.0155 | No |
| 5 | LOC641807 |  |  | 7060 | 0.090 | 0.0249 | No |
| 6 | OASL |  |  | 10812 | 0.030 | -0.1462 | No |
| 7 | ST3GAL5 |  |  | 12083 | 0.013 | -0.2032 | No |
| 8 | PQBP1 |  |  | 12962 | -0.000 | -0.2456 | No |
| 9 | BAD |  |  | 13424 | -0.008 | -0.2652 | No |
| 10 | NDUFV1 |  |  | 16826 | -0.066 | -0.4058 | Yes |
| 11 | NDUFB7 |  |  | 16859 | -0.067 | -0.3824 | Yes |
| 12 | GPR137 |  |  | 17944 | -0.097 | -0.3988 | Yes |
| 13 | P4HB |  |  | 19099 | -0.146 | -0.4004 | Yes |
| 14 | HLA-A /// HLA-H /// |  |  | 19792 | -0.211 | -0.3553 | Yes |
| 15 | HLA-C |  |  | 19820 | -0.215 | -0.2764 | Yes |
| 16 | RABAC1 |  |  | 19996 | -0.240 | -0.1952 | Yes |
| 17 | HLA-G |  |  | 20002 | -0.241 | -0.1056 | Yes |
| 18 | HLA-B |  |  | 20361 | -0.362 | 0.0119 | Yes |
Table: GSEA details [plain text format]

  

Fig 2: GALE\_FLT3ANDAPL\_DN: Random ES distribution      
 Gene set null distribution of ES for **GALE\_FLT3ANDAPL\_DN**

  
