# Supplementary material for: The rapamycin-regulated gene expression signature determines prognosis for breast cancer
Source: Mol Cancer. 2009 Sep 24;8:75. doi: 10.1186/1476-4598-8-75 (PMC2761377; doi:10.1186/1476-4598-8-75)
Supplement: Additional file 2 — Gene set enrichment analysis of in vivo data, time series. The data provided represent the time series of GSEA. This compressed file contains "Time" shortcut file and "GSEA_time" folder. Clicking on "Time" shortcut opens the index file providing access to analysis files contained in the "GSEA_time" folder. [file 1476-4598-8-75-S2.zip › GSEA_time/GLUTATHIONE_METABOLISM.html]

Details for gene set GLUTATHIONE\_METABOLISM[GSEA]

|  || Dataset | gsea\_time\_collapsed |
| Phenotype | NoPhenotypeAvailable |
| Upregulated in class | na\_neg |
| GeneSet | GLUTATHIONE\_METABOLISM |
| Enrichment Score (ES) | -0.35411873 |
| Normalized Enrichment Score (NES) | -1.277797 |
| Nominal p-value | 0.140625 |
| FDR q-value | 0.33291847 |
| FWER p-Value | 1.0 |
Table: GSEA Results Summary

  

Fig 1: Enrichment plot: GLUTATHIONE\_METABOLISM      
 Profile of the Running ES Score & Positions of GeneSet Members on the Rank Ordered List

  

| PROBE | GENE SYMBOL | GENE\_TITLE | RANK IN GENE LIST | RANK METRIC SCORE | RUNNING ES | CORE ENRICHMENT || 1 | ANPEP |  |  | 54 | 1.069 | 0.2138 | No |
| 2 | MGST3 |  |  | 1349 | 0.347 | 0.2212 | No |
| 3 | GCLC |  |  | 3035 | 0.218 | 0.1834 | No |
| 4 | GSTA1 |  |  | 4154 | 0.167 | 0.1628 | No |
| 5 | GSTA4 |  |  | 6425 | 0.103 | 0.0733 | No |
| 6 | GPX5 |  |  | 6436 | 0.103 | 0.0936 | No |
| 7 | GPX4 |  |  | 6841 | 0.094 | 0.0931 | No |
| 8 | MGST1 |  |  | 7995 | 0.073 | 0.0518 | No |
| 9 | IDH1 |  |  | 9070 | 0.056 | 0.0108 | No |
| 10 | GSTT2 |  |  | 9377 | 0.051 | 0.0064 | No |
| 11 | GSTZ1 |  |  | 9867 | 0.044 | -0.0085 | No |
| 12 | GSTM4 |  |  | 10280 | 0.038 | -0.0209 | No |
| 13 | GPX2 |  |  | 12509 | 0.006 | -0.1279 | No |
| 14 | GSTT1 |  |  | 12559 | 0.005 | -0.1292 | No |
| 15 | GSS |  |  | 13233 | -0.004 | -0.1610 | No |
| 16 | GGT1 |  |  | 14025 | -0.016 | -0.1962 | No |
| 17 | GSTA3 |  |  | 14123 | -0.018 | -0.1973 | No |
| 18 | GSTA2 |  |  | 14815 | -0.029 | -0.2250 | No |
| 19 | MGST2 |  |  | 15200 | -0.035 | -0.2367 | No |
| 20 | GSTM3 |  |  | 15317 | -0.037 | -0.2349 | No |
| 21 | GSTM5 |  |  | 15419 | -0.038 | -0.2320 | No |
| 22 | GSTO2 |  |  | 17493 | -0.084 | -0.3158 | No |
| 23 | GSTM1 |  |  | 17772 | -0.092 | -0.3108 | No |
| 24 | GPX3 |  |  | 18665 | -0.125 | -0.3289 | Yes |
| 25 | GSTM2 |  |  | 18986 | -0.140 | -0.3160 | Yes |
| 26 | GCLM |  |  | 19568 | -0.186 | -0.3067 | Yes |
| 27 | IDH2 |  |  | 19803 | -0.213 | -0.2750 | Yes |
| 28 | GSTP1 |  |  | 20188 | -0.290 | -0.2349 | Yes |
| 29 | PGD |  |  | 20349 | -0.354 | -0.1710 | Yes |
| 30 | GPX1 |  |  | 20417 | -0.399 | -0.0936 | Yes |
| 31 | G6PD |  |  | 20527 | -0.507 | 0.0038 | Yes |
Table: GSEA details [plain text format]

  

Fig 2: GLUTATHIONE\_METABOLISM: Random ES distribution      
 Gene set null distribution of ES for **GLUTATHIONE\_METABOLISM**

  
