# Supplementary material for: The rapamycin-regulated gene expression signature determines prognosis for breast cancer
Source: Mol Cancer. 2009 Sep 24;8:75. doi: 10.1186/1476-4598-8-75 (PMC2761377; doi:10.1186/1476-4598-8-75)
Supplement: Additional file 2 — Gene set enrichment analysis of in vivo data, time series. The data provided represent the time series of GSEA. This compressed file contains "Time" shortcut file and "GSEA_time" folder. Clicking on "Time" shortcut opens the index file providing access to analysis files contained in the "GSEA_time" folder. [file 1476-4598-8-75-S2.zip › GSEA_time/gsea_report_for_na_neg_1197940125570.html]

Report for na\_neg 1197940125570 [GSEA]

| GS  follow link to MSigDB | GS DETAILS | SIZE | ES | NES | NOM p-val | FDR q-val | FWER p-val | RANK AT MAX | LEADING EDGE || 1 | BCNU\_GLIOMA\_NOMGMT\_48HRS\_DN | Details ... | 31 | -0.63 | -2.28 | 0.000 | 0.005 | 0.005 | 1849 | tags=42%, list=9%, signal=46% |
| 2 | FATTY\_ACID\_SYNTHESIS | Details ... | 16 | -0.65 | -1.96 | 0.000 | 0.068 | 0.132 | 855 | tags=31%, list=4%, signal=33% |
| 3 | ADIPOCYTE\_PPARG\_UP | Details ... | 15 | -0.64 | -1.92 | 0.000 | 0.060 | 0.171 | 957 | tags=40%, list=5%, signal=42% |
| 4 | TCA | Details ... | 15 | -0.63 | -1.85 | 0.000 | 0.083 | 0.294 | 1367 | tags=40%, list=7%, signal=43% |
| 5 | UVC\_TTD\_4HR\_UP | Details ... | 58 | -0.45 | -1.83 | 0.000 | 0.075 | 0.327 | 1065 | tags=21%, list=5%, signal=22% |
| 6 | CITRATE\_CYCLE\_TCA\_CYCLE | Details ... | 20 | -0.58 | -1.83 | 0.000 | 0.067 | 0.345 | 2116 | tags=45%, list=10%, signal=50% |
| 7 | CROONQUIST\_IL6\_RAS\_DN | Details ... | 24 | -0.53 | -1.76 | 0.007 | 0.095 | 0.497 | 1620 | tags=42%, list=8%, signal=45% |
| 8 | TGFBETA\_C2\_UP | Details ... | 18 | -0.54 | -1.70 | 0.018 | 0.136 | 0.675 | 291 | tags=22%, list=1%, signal=23% |
| 9 | ALANINE\_AND\_ASPARTATE\_METABOLISM | Details ... | 21 | -0.51 | -1.69 | 0.006 | 0.126 | 0.686 | 622 | tags=19%, list=3%, signal=20% |
| 10 | CANCER\_UNDIFFERENTIATED\_META\_UP | Details ... | 67 | -0.38 | -1.67 | 0.000 | 0.137 | 0.753 | 2257 | tags=37%, list=11%, signal=42% |
| 11 | NO2IL12PATHWAY | Details ... | 15 | -0.53 | -1.64 | 0.020 | 0.145 | 0.806 | 2699 | tags=33%, list=13%, signal=38% |
| 12 | SHIPP\_FL\_VS\_DLBCL\_DN | Details ... | 34 | -0.46 | -1.63 | 0.000 | 0.142 | 0.825 | 2428 | tags=47%, list=12%, signal=53% |
| 13 | CROONQUIST\_IL6\_STARVE\_UP | Details ... | 33 | -0.43 | -1.56 | 0.008 | 0.209 | 0.933 | 1620 | tags=30%, list=8%, signal=33% |
| 14 | CALRES\_MOUSE\_UP | Details ... | 29 | -0.43 | -1.55 | 0.025 | 0.212 | 0.947 | 1455 | tags=28%, list=7%, signal=30% |
| 15 | MTA3PATHWAY | Details ... | 15 | -0.53 | -1.54 | 0.078 | 0.208 | 0.958 | 2360 | tags=40%, list=11%, signal=45% |
| 16 | BECKER\_TAMOXIFEN\_RESISTANT\_UP | Details ... | 40 | -0.40 | -1.52 | 0.046 | 0.215 | 0.969 | 759 | tags=23%, list=4%, signal=23% |
| 17 | DNMT1\_KO\_DN | Details ... | 16 | -0.50 | -1.52 | 0.065 | 0.204 | 0.970 | 291 | tags=19%, list=1%, signal=19% |
| 18 | POD1\_KO\_MOST\_UP | Details ... | 32 | -0.42 | -1.49 | 0.023 | 0.234 | 0.989 | 244 | tags=9%, list=1%, signal=9% |
| 19 | GALE\_FLT3ANDAPL\_DN | Details ... | 18 | -0.45 | -1.47 | 0.098 | 0.249 | 0.992 | 1508 | tags=33%, list=7%, signal=36% |
| 20 | PROTEASOME\_DEGRADATION | Details ... | 31 | -0.42 | -1.46 | 0.036 | 0.256 | 0.995 | 2187 | tags=32%, list=11%, signal=36% |
| 21 | BENNETT\_SLE\_UP | Details ... | 28 | -0.41 | -1.44 | 0.023 | 0.264 | 0.995 | 1149 | tags=29%, list=6%, signal=30% |
| 22 | IDX\_TSA\_UP\_CLUSTER5 | Details ... | 104 | -0.32 | -1.43 | 0.000 | 0.265 | 0.998 | 1106 | tags=19%, list=5%, signal=20% |
| 23 | XPB\_TTD-CS\_DN | Details ... | 24 | -0.42 | -1.42 | 0.062 | 0.273 | 0.999 | 60 | tags=17%, list=0%, signal=17% |
| 24 | LYSINE\_DEGRADATION | Details ... | 30 | -0.39 | -1.41 | 0.050 | 0.284 | 1.000 | 1121 | tags=27%, list=5%, signal=28% |
| 25 | VEGF\_HUVEC\_30MIN\_UP | Details ... | 24 | -0.42 | -1.40 | 0.077 | 0.285 | 1.000 | 1818 | tags=25%, list=9%, signal=27% |
| 26 | TSA\_HEPATOMA\_CANCER\_UP | Details ... | 39 | -0.37 | -1.39 | 0.043 | 0.296 | 1.000 | 806 | tags=13%, list=4%, signal=13% |
| 27 | OXSTRESS\_RPETHREE\_DN | Details ... | 28 | -0.40 | -1.38 | 0.086 | 0.292 | 1.000 | 514 | tags=18%, list=2%, signal=18% |
| 28 | ADIP\_DIFF\_CLUSTER4 | Details ... | 34 | -0.39 | -1.38 | 0.031 | 0.282 | 1.000 | 1390 | tags=32%, list=7%, signal=35% |
| 29 | AGUIRRE\_PANCREAS\_CHR19 | Details ... | 74 | -0.32 | -1.38 | 0.025 | 0.279 | 1.000 | 2246 | tags=22%, list=11%, signal=24% |
| 30 | UVC\_TTD\_ALL\_UP | Details ... | 76 | -0.32 | -1.37 | 0.025 | 0.273 | 1.000 | 1065 | tags=16%, list=5%, signal=17% |
| 31 | PENG\_LEUCINE\_DN | Details ... | 139 | -0.29 | -1.37 | 0.000 | 0.266 | 1.000 | 1571 | tags=24%, list=8%, signal=26% |
| 32 | TGFBETA\_EARLY\_UP | Details ... | 47 | -0.37 | -1.35 | 0.052 | 0.290 | 1.000 | 291 | tags=15%, list=1%, signal=15% |
| 33 | KREBS\_TCA\_CYCLE | Details ... | 30 | -0.38 | -1.34 | 0.073 | 0.300 | 1.000 | 1367 | tags=30%, list=7%, signal=32% |
| 34 | TSA\_CD4\_UP | Details ... | 25 | -0.38 | -1.34 | 0.076 | 0.295 | 1.000 | 1305 | tags=24%, list=6%, signal=26% |
| 35 | OVARIAN\_INFERTILITY\_GENES | Details ... | 25 | -0.39 | -1.33 | 0.090 | 0.303 | 1.000 | 959 | tags=20%, list=5%, signal=21% |
| 36 | BRENTANI\_REPAIR | Details ... | 35 | -0.35 | -1.31 | 0.093 | 0.319 | 1.000 | 1538 | tags=23%, list=7%, signal=25% |
| 37 | BRCA1\_SW480\_UP | Details ... | 25 | -0.39 | -1.31 | 0.131 | 0.319 | 1.000 | 204 | tags=12%, list=1%, signal=12% |
| 38 | HPV31\_DN | Details ... | 47 | -0.34 | -1.31 | 0.056 | 0.315 | 1.000 | 709 | tags=19%, list=3%, signal=20% |
| 39 | RADAEVA\_IFNA\_UP | Details ... | 50 | -0.33 | -1.30 | 0.038 | 0.318 | 1.000 | 1450 | tags=28%, list=7%, signal=30% |
| 40 | TFF2\_KO\_UP | Details ... | 23 | -0.39 | -1.29 | 0.146 | 0.334 | 1.000 | 2054 | tags=26%, list=10%, signal=29% |
| 41 | STRESS\_ARSENIC\_SPECIFIC\_DN | Details ... | 27 | -0.37 | -1.28 | 0.125 | 0.331 | 1.000 | 2180 | tags=26%, list=11%, signal=29% |
| 42 | GLUTATHIONE\_METABOLISM | Details ... | 31 | -0.35 | -1.28 | 0.141 | 0.333 | 1.000 | 1942 | tags=26%, list=9%, signal=28% |
| 43 | IFNALPHA\_NL\_HCC\_UP | Details ... | 18 | -0.40 | -1.26 | 0.137 | 0.355 | 1.000 | 867 | tags=28%, list=4%, signal=29% |
| 44 | ELECTRON\_TRANSPORTER\_ACTIVITY | Details ... | 113 | -0.27 | -1.25 | 0.063 | 0.361 | 1.000 | 1545 | tags=19%, list=7%, signal=21% |
| 45 | BILE\_ACID\_BIOSYNTHESIS | Details ... | 27 | -0.36 | -1.24 | 0.176 | 0.377 | 1.000 | 4516 | tags=59%, list=22%, signal=76% |
| 46 | AGED\_MOUSE\_HYPOTH\_DN | Details ... | 38 | -0.33 | -1.23 | 0.132 | 0.385 | 1.000 | 674 | tags=18%, list=3%, signal=19% |
| 47 | IFNALPHA\_NL\_UP | Details ... | 27 | -0.35 | -1.23 | 0.169 | 0.381 | 1.000 | 1450 | tags=33%, list=7%, signal=36% |
| 48 | BUT\_TSA\_UP | Details ... | 18 | -0.40 | -1.22 | 0.198 | 0.394 | 1.000 | 139 | tags=17%, list=1%, signal=17% |
| 49 | DNA\_REPLICATION\_REACTOME | Details ... | 44 | -0.31 | -1.21 | 0.131 | 0.404 | 1.000 | 2300 | tags=23%, list=11%, signal=26% |
| 50 | ADIP\_DIFF\_CLUSTER3 | Details ... | 33 | -0.32 | -1.21 | 0.162 | 0.397 | 1.000 | 739 | tags=18%, list=4%, signal=19% |
| 51 | BUTANOATE\_METABOLISM |  | 27 | -0.34 | -1.18 | 0.191 | 0.441 | 1.000 | 1121 | tags=19%, list=5%, signal=20% |
| 52 | DOX\_RESIST\_GASTRIC\_UP |  | 44 | -0.31 | -1.18 | 0.171 | 0.449 | 1.000 | 1232 | tags=20%, list=6%, signal=22% |
| 53 | CHESLER\_BRAIN\_NEURAL\_HIGH\_GENES |  | 23 | -0.36 | -1.17 | 0.237 | 0.447 | 1.000 | 1907 | tags=22%, list=9%, signal=24% |
| 54 | CIS\_RESIST\_GASTRIC\_UP |  | 16 | -0.38 | -1.17 | 0.244 | 0.441 | 1.000 | 291 | tags=13%, list=1%, signal=13% |
| 55 | BIOGENIC\_AMINE\_SYNTHESIS |  | 15 | -0.39 | -1.16 | 0.234 | 0.451 | 1.000 | 3837 | tags=27%, list=19%, signal=33% |
| 56 | LAL\_KO\_3MO\_UP |  | 46 | -0.31 | -1.16 | 0.154 | 0.443 | 1.000 | 1214 | tags=17%, list=6%, signal=18% |
| 57 | LIAN\_MYELOID\_DIFF\_TF |  | 34 | -0.31 | -1.16 | 0.188 | 0.443 | 1.000 | 514 | tags=12%, list=2%, signal=12% |
| 58 | IFNA\_UV-CMV\_COMMON\_HCMV\_6HRS\_UP |  | 29 | -0.33 | -1.16 | 0.220 | 0.445 | 1.000 | 1149 | tags=17%, list=6%, signal=18% |
| 59 | ZMPSTE24\_KO\_DN |  | 32 | -0.32 | -1.15 | 0.155 | 0.441 | 1.000 | 2914 | tags=38%, list=14%, signal=44% |
| 60 | CMV\_8HRS\_UP |  | 32 | -0.31 | -1.15 | 0.207 | 0.435 | 1.000 | 856 | tags=19%, list=4%, signal=20% |
| 61 | BCNU\_GLIOMA\_MGMT\_48HRS\_DN |  | 158 | -0.24 | -1.15 | 0.200 | 0.429 | 1.000 | 2345 | tags=19%, list=11%, signal=21% |
| 62 | ROS\_MOUSE\_AORTA\_UP |  | 24 | -0.34 | -1.15 | 0.252 | 0.425 | 1.000 | 404 | tags=13%, list=2%, signal=13% |
| 63 | IFNALPHA\_HCC\_UP |  | 29 | -0.33 | -1.15 | 0.205 | 0.420 | 1.000 | 867 | tags=21%, list=4%, signal=22% |
| 64 | SANA\_IFNG\_ENDOTHELIAL\_UP |  | 73 | -0.26 | -1.14 | 0.121 | 0.429 | 1.000 | 1449 | tags=16%, list=7%, signal=18% |
| 65 | INOS\_ALL\_UP |  | 53 | -0.28 | -1.14 | 0.183 | 0.422 | 1.000 | 1977 | tags=26%, list=10%, signal=29% |
| 66 | GLYCOGEN\_METABOLISM |  | 34 | -0.30 | -1.13 | 0.181 | 0.443 | 1.000 | 501 | tags=15%, list=2%, signal=15% |
| 67 | MONOAMINE\_GPCRS |  | 32 | -0.32 | -1.13 | 0.264 | 0.442 | 1.000 | 4975 | tags=41%, list=24%, signal=53% |
| 68 | IL3PATHWAY |  | 15 | -0.37 | -1.12 | 0.308 | 0.444 | 1.000 | 2699 | tags=33%, list=13%, signal=38% |
| 69 | MMS\_HUMAN\_LYMPH\_HIGH\_24HRS\_UP |  | 19 | -0.36 | -1.12 | 0.302 | 0.457 | 1.000 | 1620 | tags=21%, list=8%, signal=23% |
| 70 | VEGF\_HUVEC\_2HRS\_UP |  | 30 | -0.32 | -1.11 | 0.255 | 0.458 | 1.000 | 1818 | tags=20%, list=9%, signal=22% |
| 71 | TSA\_RKO\_UP |  | 17 | -0.37 | -1.10 | 0.291 | 0.468 | 1.000 | 1233 | tags=18%, list=6%, signal=19% |
| 72 | DIAB\_NEPH\_UP |  | 61 | -0.28 | -1.10 | 0.217 | 0.464 | 1.000 | 1721 | tags=20%, list=8%, signal=21% |
| 73 | CHOLESTEROL\_BIOSYNTHESIS |  | 15 | -0.38 | -1.10 | 0.327 | 0.471 | 1.000 | 1309 | tags=27%, list=6%, signal=28% |
| 74 | POMEROY\_MD\_TREATMENT\_GOOD\_VS\_POOR\_UP |  | 29 | -0.32 | -1.09 | 0.339 | 0.489 | 1.000 | 2062 | tags=24%, list=10%, signal=27% |
| 75 | PROTEASOME |  | 17 | -0.35 | -1.09 | 0.332 | 0.484 | 1.000 | 76 | tags=12%, list=0%, signal=12% |
| 76 | INOS\_ALL\_DN |  | 77 | -0.25 | -1.08 | 0.234 | 0.490 | 1.000 | 611 | tags=12%, list=3%, signal=12% |
| 77 | SHIPP\_DLBCL\_CURED\_UP |  | 30 | -0.30 | -1.06 | 0.348 | 0.540 | 1.000 | 1452 | tags=17%, list=7%, signal=18% |
| 78 | CPR\_LOW\_LIVER\_UP |  | 17 | -0.34 | -1.05 | 0.383 | 0.545 | 1.000 | 1719 | tags=29%, list=8%, signal=32% |
| 79 | IGFR\_IR\_UP |  | 18 | -0.35 | -1.04 | 0.399 | 0.558 | 1.000 | 739 | tags=17%, list=4%, signal=17% |
| 80 | LEE\_DENA\_DN |  | 72 | -0.24 | -1.04 | 0.304 | 0.558 | 1.000 | 3095 | tags=26%, list=15%, signal=31% |
| 81 | FLECHNER\_KIDNEY\_TRANSPLANT\_WELL\_DN |  | 22 | -0.32 | -1.03 | 0.369 | 0.567 | 1.000 | 698 | tags=18%, list=3%, signal=19% |
| 82 | LIAN\_MYELOID\_DIFF\_RECEPTORS |  | 29 | -0.29 | -1.02 | 0.429 | 0.603 | 1.000 | 1571 | tags=17%, list=8%, signal=19% |
| 83 | WALLACE\_JAK2\_DIFF |  | 30 | -0.29 | -1.01 | 0.413 | 0.600 | 1.000 | 1867 | tags=27%, list=9%, signal=29% |
| 84 | XU\_CBP\_UP |  | 21 | -0.31 | -1.01 | 0.410 | 0.595 | 1.000 | 740 | tags=14%, list=4%, signal=15% |
| 85 | BCNU\_GLIOMA\_MGMT\_24HRS\_DN |  | 32 | -0.29 | -1.01 | 0.429 | 0.602 | 1.000 | 1737 | tags=16%, list=8%, signal=17% |
| 86 | GOLDRATH\_CELLCYCLE |  | 28 | -0.28 | -1.00 | 0.432 | 0.603 | 1.000 | 988 | tags=18%, list=5%, signal=19% |
| 87 | PROTEASOMEPATHWAY |  | 21 | -0.32 | -1.00 | 0.410 | 0.598 | 1.000 | 973 | tags=19%, list=5%, signal=20% |
| 88 | DAC\_IFN\_BLADDER\_UP |  | 16 | -0.34 | -1.00 | 0.422 | 0.597 | 1.000 | 1043 | tags=25%, list=5%, signal=26% |
| 89 | ARGININE\_AND\_PROLINE\_METABOLISM |  | 42 | -0.26 | -1.00 | 0.471 | 0.594 | 1.000 | 1997 | tags=24%, list=10%, signal=26% |
| 90 | HCC\_SURVIVAL\_GOOD\_VS\_POOR\_UP |  | 136 | -0.20 | -0.99 | 0.714 | 0.603 | 1.000 | 2554 | tags=18%, list=12%, signal=21% |
| 91 | AGUIRRE\_PANCREAS\_CHR8 |  | 61 | -0.24 | -0.98 | 0.484 | 0.630 | 1.000 | 1990 | tags=23%, list=10%, signal=25% |
| 92 | IL6\_SCAR\_FIBRO\_UP |  | 24 | -0.29 | -0.98 | 0.450 | 0.624 | 1.000 | 872 | tags=17%, list=4%, signal=17% |
| 93 | UREA\_CYCLE\_AND\_METABOLISM\_OF\_AMINO\_GROUPS |  | 18 | -0.32 | -0.97 | 0.474 | 0.628 | 1.000 | 1997 | tags=22%, list=10%, signal=25% |
| 94 | TAVOR\_CEBP\_DN |  | 31 | -0.28 | -0.96 | 0.541 | 0.648 | 1.000 | 759 | tags=16%, list=4%, signal=17% |
| 95 | HOFFMANN\_BIVSBII\_IMVM |  | 81 | -0.22 | -0.96 | 0.550 | 0.641 | 1.000 | 1198 | tags=14%, list=6%, signal=14% |
| 96 | CARBON\_FIXATION |  | 21 | -0.30 | -0.96 | 0.463 | 0.635 | 1.000 | 2360 | tags=33%, list=11%, signal=38% |
| 97 | FERRANDO\_T\_CELL\_DIFFERENTIATION\_PATHWAY |  | 18 | -0.30 | -0.95 | 0.518 | 0.656 | 1.000 | 827 | tags=11%, list=4%, signal=12% |
| 98 | TYROSINE\_METABOLISM |  | 31 | -0.25 | -0.93 | 0.650 | 0.693 | 1.000 | 4474 | tags=42%, list=22%, signal=53% |
| 99 | GPCRDB\_CLASS\_A\_RHODOPSIN\_LIKE |  | 174 | -0.19 | -0.92 | 0.750 | 0.709 | 1.000 | 4091 | tags=29%, list=20%, signal=36% |
| 100 | CCR3PATHWAY |  | 22 | -0.28 | -0.92 | 0.588 | 0.712 | 1.000 | 1923 | tags=27%, list=9%, signal=30% |
| 101 | PHENYLALANINE\_METABOLISM |  | 22 | -0.28 | -0.92 | 0.602 | 0.708 | 1.000 | 1161 | tags=18%, list=6%, signal=19% |
| 102 | IGF1RPATHWAY |  | 15 | -0.31 | -0.91 | 0.555 | 0.710 | 1.000 | 1812 | tags=27%, list=9%, signal=29% |
| 103 | TSADAC\_HYPERMETH\_OVCA\_UP |  | 15 | -0.31 | -0.90 | 0.596 | 0.736 | 1.000 | 2377 | tags=27%, list=12%, signal=30% |
| 104 | CPR\_LOW\_LIVER\_DN |  | 22 | -0.28 | -0.89 | 0.624 | 0.742 | 1.000 | 259 | tags=9%, list=1%, signal=9% |
| 105 | VALINE\_LEUCINE\_AND\_ISOLEUCINE\_DEGRADATION |  | 36 | -0.24 | -0.88 | 0.691 | 0.747 | 1.000 | 1989 | tags=17%, list=10%, signal=18% |
| 106 | TIDPATHWAY |  | 18 | -0.28 | -0.88 | 0.637 | 0.748 | 1.000 | 3966 | tags=33%, list=19%, signal=41% |
| 107 | CDMACPATHWAY |  | 16 | -0.30 | -0.88 | 0.624 | 0.743 | 1.000 | 1812 | tags=19%, list=9%, signal=21% |
| 108 | HOGERKORP\_CD44\_DN |  | 22 | -0.26 | -0.87 | 0.727 | 0.748 | 1.000 | 1520 | tags=14%, list=7%, signal=15% |
| 109 | HDACI\_COLON\_CLUSTER7 |  | 15 | -0.30 | -0.87 | 0.619 | 0.745 | 1.000 | 1426 | tags=20%, list=7%, signal=21% |
| 110 | HSP27PATHWAY |  | 15 | -0.30 | -0.86 | 0.654 | 0.754 | 1.000 | 1518 | tags=13%, list=7%, signal=14% |
| 111 | GPCRS\_CLASS\_A\_RHODOPSIN\_LIKE |  | 134 | -0.18 | -0.82 | 1.000 | 0.814 | 1.000 | 4091 | tags=28%, list=20%, signal=35% |
| 112 | GPCRDB\_OTHER |  | 58 | -0.20 | -0.81 | 0.790 | 0.834 | 1.000 | 4960 | tags=40%, list=24%, signal=52% |
| 113 | GLYCEROLIPID\_METABOLISM |  | 44 | -0.21 | -0.80 | 0.831 | 0.837 | 1.000 | 2517 | tags=20%, list=12%, signal=23% |
| 114 | BETA\_ALANINE\_METABOLISM |  | 27 | -0.24 | -0.80 | 0.813 | 0.834 | 1.000 | 2527 | tags=26%, list=12%, signal=30% |
| 115 | HUMAN\_TISSUE\_LIVER |  | 37 | -0.20 | -0.78 | 0.863 | 0.851 | 1.000 | 3437 | tags=30%, list=17%, signal=36% |
| 116 | PEPTIDE\_GPCRS |  | 72 | -0.17 | -0.70 | 0.975 | 0.929 | 1.000 | 4839 | tags=35%, list=23%, signal=45% |
| 117 | UVB\_NHEK4\_6HRS\_DN |  | 19 | -0.23 | -0.69 | 0.884 | 0.928 | 1.000 | 2300 | tags=21%, list=11%, signal=24% |
Table: Gene sets enriched in phenotype **na**[plain text format]****

  
