# Supplementary material for: The rapamycin-regulated gene expression signature determines prognosis for breast cancer
Source: Mol Cancer. 2009 Sep 24;8:75. doi: 10.1186/1476-4598-8-75 (PMC2761377; doi:10.1186/1476-4598-8-75)
Supplement: Additional file 2 — Gene set enrichment analysis of in vivo data, time series. The data provided represent the time series of GSEA. This compressed file contains "Time" shortcut file and "GSEA_time" folder. Clicking on "Time" shortcut opens the index file providing access to analysis files contained in the "GSEA_time" folder. [file 1476-4598-8-75-S2.zip › GSEA_time/gsea_report_for_na_pos_1197940125570.html]

Report for na\_pos 1197940125570 [GSEA]

| GS  follow link to MSigDB | GS DETAILS | SIZE | ES | NES | NOM p-val | FDR q-val | FWER p-val | RANK AT MAX | LEADING EDGE || 1 | WNT\_TARGETS | Details ... | 25 | 0.80 | 1.99 | 0.000 | 0.003 | 0.003 | 1876 | tags=52%, list=9%, signal=57% |
| 2 | LEE\_TCELLS1\_UP | Details ... | 201 | 0.65 | 1.96 | 0.000 | 0.002 | 0.003 | 3589 | tags=50%, list=17%, signal=60% |
| 3 | DORSEY\_DOXYCYCLINE\_UP | Details ... | 29 | 0.77 | 1.96 | 0.000 | 0.001 | 0.003 | 969 | tags=34%, list=5%, signal=36% |
| 4 | LEE\_TCELLS8\_UP | Details ... | 201 | 0.65 | 1.95 | 0.000 | 0.001 | 0.003 | 3589 | tags=50%, list=17%, signal=60% |
| 5 | CROONQUIST\_IL6\_STROMA\_UP | Details ... | 37 | 0.74 | 1.95 | 0.000 | 0.001 | 0.004 | 1615 | tags=41%, list=8%, signal=44% |
| 6 | LEE\_TCELLS10\_UP | Details ... | 201 | 0.65 | 1.94 | 0.000 | 0.001 | 0.006 | 3589 | tags=50%, list=17%, signal=60% |
| 7 | MENSE\_HYPOXIA\_UP | Details ... | 107 | 0.65 | 1.92 | 0.000 | 0.001 | 0.009 | 4533 | tags=60%, list=22%, signal=76% |
| 8 | UVC\_XPCS\_4HR\_DN | Details ... | 242 | 0.63 | 1.91 | 0.000 | 0.002 | 0.016 | 4050 | tags=52%, list=20%, signal=65% |
| 9 | HYPOXIA\_REVIEW | Details ... | 81 | 0.66 | 1.89 | 0.000 | 0.002 | 0.019 | 2845 | tags=44%, list=14%, signal=51% |
| 10 | UVC\_XPCS\_8HR\_DN | Details ... | 408 | 0.61 | 1.87 | 0.000 | 0.003 | 0.031 | 4368 | tags=52%, list=21%, signal=65% |
| 11 | CMV-UV\_HCMV\_6HRS\_DN | Details ... | 108 | 0.63 | 1.87 | 0.000 | 0.003 | 0.037 | 3668 | tags=39%, list=18%, signal=47% |
| 12 | CORDERO\_KRAS\_KD\_VS\_CONTROL\_UP | Details ... | 74 | 0.66 | 1.86 | 0.000 | 0.003 | 0.038 | 3385 | tags=46%, list=16%, signal=55% |
| 13 | HYPOXIA\_REG\_UP | Details ... | 38 | 0.70 | 1.86 | 0.000 | 0.003 | 0.038 | 3860 | tags=58%, list=19%, signal=71% |
| 14 | CROONQUIST\_RAS\_STROMA\_DN | Details ... | 19 | 0.79 | 1.86 | 0.000 | 0.003 | 0.047 | 1528 | tags=47%, list=7%, signal=51% |
| 15 | UVC\_XPCS\_ALL\_DN | Details ... | 478 | 0.60 | 1.85 | 0.000 | 0.004 | 0.053 | 4368 | tags=50%, list=21%, signal=62% |
| 16 | DIAB\_NEPH\_DN | Details ... | 374 | 0.60 | 1.85 | 0.000 | 0.004 | 0.062 | 4024 | tags=46%, list=20%, signal=56% |
| 17 | CHEN\_HOXA5\_TARGETS\_UP | Details ... | 229 | 0.61 | 1.84 | 0.000 | 0.004 | 0.066 | 3927 | tags=48%, list=19%, signal=58% |
| 18 | UVC\_TTD\_4HR\_DN | Details ... | 297 | 0.61 | 1.84 | 0.000 | 0.004 | 0.072 | 4425 | tags=54%, list=21%, signal=68% |
| 19 | NICK\_RHAPC\_UP | Details ... | 32 | 0.71 | 1.84 | 0.000 | 0.004 | 0.078 | 2013 | tags=41%, list=10%, signal=45% |
| 20 | AS3\_FIBRO\_DN | Details ... | 31 | 0.71 | 1.83 | 0.000 | 0.005 | 0.097 | 2843 | tags=48%, list=14%, signal=56% |
| 21 | TAKEDA\_NUP8\_HOXA9\_16D\_DN | Details ... | 228 | 0.61 | 1.83 | 0.000 | 0.005 | 0.100 | 5807 | tags=65%, list=28%, signal=89% |
| 22 | FSH\_OVARY\_MCV152\_DN | Details ... | 46 | 0.67 | 1.83 | 0.000 | 0.005 | 0.107 | 4202 | tags=52%, list=20%, signal=65% |
| 23 | ZHAN\_MM\_CD138\_HP\_VS\_REST | Details ... | 48 | 0.67 | 1.82 | 0.000 | 0.005 | 0.109 | 2633 | tags=44%, list=13%, signal=50% |
| 24 | ASTON\_DEPRESSION\_UP | Details ... | 45 | 0.67 | 1.82 | 0.000 | 0.005 | 0.110 | 1956 | tags=36%, list=9%, signal=39% |
| 25 | HDACI\_COLON\_BUT30MIN\_DN | Details ... | 37 | 0.69 | 1.82 | 0.000 | 0.005 | 0.110 | 3118 | tags=51%, list=15%, signal=60% |
| 26 | UVB\_SCC\_UP | Details ... | 87 | 0.63 | 1.82 | 0.000 | 0.005 | 0.122 | 4557 | tags=53%, list=22%, signal=68% |
| 27 | UV-CMV\_UNIQUE\_HCMV\_6HRS\_DN | Details ... | 82 | 0.63 | 1.82 | 0.000 | 0.005 | 0.124 | 3668 | tags=39%, list=18%, signal=47% |
| 28 | AS3\_FIBRO\_C4 | Details ... | 18 | 0.78 | 1.82 | 0.000 | 0.005 | 0.124 | 3407 | tags=67%, list=17%, signal=80% |
| 29 | UVC\_TTD\_ALL\_DN | Details ... | 358 | 0.59 | 1.82 | 0.000 | 0.005 | 0.125 | 4425 | tags=52%, list=21%, signal=65% |
| 30 | MANALO\_HYPOXIA\_UP | Details ... | 94 | 0.62 | 1.79 | 0.000 | 0.008 | 0.224 | 4024 | tags=50%, list=20%, signal=62% |
| 31 | IRITANI\_ADPROX\_DN | Details ... | 60 | 0.64 | 1.79 | 0.000 | 0.009 | 0.238 | 3018 | tags=40%, list=15%, signal=47% |
| 32 | TSA\_HEPATOMA\_CANCER\_DN | Details ... | 16 | 0.79 | 1.78 | 0.000 | 0.008 | 0.239 | 2735 | tags=63%, list=13%, signal=72% |
| 33 | TAKEDA\_NUP8\_HOXA9\_6H\_UP | Details ... | 85 | 0.62 | 1.78 | 0.000 | 0.009 | 0.260 | 3475 | tags=34%, list=17%, signal=41% |
| 34 | DOX\_RESIST\_GASTRIC\_DN | Details ... | 17 | 0.77 | 1.78 | 0.000 | 0.009 | 0.274 | 2667 | tags=65%, list=13%, signal=74% |
| 35 | SHEPARD\_NEG\_REG\_OF\_CELL\_PROLIFERATION | Details ... | 108 | 0.61 | 1.77 | 0.000 | 0.009 | 0.286 | 3652 | tags=35%, list=18%, signal=43% |
| 36 | CMV\_HCMV\_TIMECOURSE\_6HRS\_DN | Details ... | 52 | 0.65 | 1.77 | 0.000 | 0.010 | 0.302 | 3972 | tags=50%, list=19%, signal=62% |
| 37 | LI\_FETAL\_VS\_WT\_KIDNEY\_UP | Details ... | 179 | 0.58 | 1.76 | 0.000 | 0.011 | 0.331 | 4024 | tags=43%, list=20%, signal=53% |
| 38 | HINATA\_NFKB\_UP | Details ... | 106 | 0.60 | 1.76 | 0.000 | 0.012 | 0.355 | 829 | tags=19%, list=4%, signal=20% |
| 39 | UVB\_NHEK3\_C3 | Details ... | 17 | 0.76 | 1.76 | 0.000 | 0.011 | 0.357 | 1496 | tags=41%, list=7%, signal=44% |
| 40 | UVB\_NHEK1\_C6 | Details ... | 130 | 0.59 | 1.76 | 0.000 | 0.011 | 0.359 | 6142 | tags=69%, list=30%, signal=98% |
| 41 | UVC\_HIGH\_D4\_DN | Details ... | 46 | 0.65 | 1.76 | 0.000 | 0.011 | 0.361 | 3167 | tags=46%, list=15%, signal=54% |
| 42 | ROS\_MOUSE\_AORTA\_DN | Details ... | 76 | 0.61 | 1.75 | 0.000 | 0.011 | 0.372 | 3046 | tags=43%, list=15%, signal=51% |
| 43 | TSADAC\_RKOEXP\_UP | Details ... | 16 | 0.75 | 1.75 | 0.000 | 0.011 | 0.379 | 363 | tags=31%, list=2%, signal=32% |
| 44 | BAF57\_BT549\_DN | Details ... | 330 | 0.57 | 1.74 | 0.000 | 0.013 | 0.426 | 4203 | tags=44%, list=20%, signal=55% |
| 45 | VEGF\_MMMEC\_12HRS\_UP | Details ... | 28 | 0.68 | 1.74 | 0.000 | 0.014 | 0.467 | 3161 | tags=39%, list=15%, signal=46% |
| 46 | HDACI\_COLON\_SUL30MIN\_DN | Details ... | 38 | 0.65 | 1.74 | 0.001 | 0.014 | 0.481 | 2734 | tags=42%, list=13%, signal=48% |
| 47 | POD1\_KO\_MOST\_DN | Details ... | 25 | 0.70 | 1.74 | 0.000 | 0.014 | 0.483 | 1981 | tags=36%, list=10%, signal=40% |
| 48 | CMV\_HCMV\_TIMECOURSE\_14HRS\_DN | Details ... | 41 | 0.65 | 1.74 | 0.000 | 0.014 | 0.486 | 3484 | tags=41%, list=17%, signal=50% |
| 49 | GUO\_HEX\_UP | Details ... | 81 | 0.60 | 1.74 | 0.000 | 0.014 | 0.489 | 5511 | tags=57%, list=27%, signal=77% |
| 50 | IRITANI\_ADPROX\_VASC | Details ... | 151 | 0.58 | 1.73 | 0.000 | 0.014 | 0.494 | 3018 | tags=35%, list=15%, signal=41% |
| 51 | UVC\_TTD\_8HR\_DN |  | 165 | 0.58 | 1.73 | 0.000 | 0.014 | 0.502 | 3652 | tags=45%, list=18%, signal=54% |
| 52 | METASTASIS\_ADENOCARC\_DN |  | 34 | 0.66 | 1.73 | 0.001 | 0.014 | 0.518 | 1871 | tags=24%, list=9%, signal=26% |
| 53 | AGEING\_BRAIN\_UP |  | 208 | 0.57 | 1.73 | 0.000 | 0.014 | 0.532 | 4258 | tags=44%, list=21%, signal=55% |
| 54 | RIBOSOMAL\_PROTEINS |  | 96 | 0.60 | 1.73 | 0.000 | 0.014 | 0.535 | 4666 | tags=49%, list=23%, signal=63% |
| 55 | REOVIRUS\_HEK293\_UP |  | 236 | 0.57 | 1.73 | 0.000 | 0.014 | 0.538 | 4912 | tags=51%, list=24%, signal=66% |
| 56 | UVB\_NHEK1\_DN |  | 270 | 0.56 | 1.72 | 0.000 | 0.015 | 0.566 | 4957 | tags=58%, list=24%, signal=76% |
| 57 | UV\_ESR\_OLD\_UNREG |  | 19 | 0.71 | 1.71 | 0.002 | 0.020 | 0.668 | 3960 | tags=42%, list=19%, signal=52% |
| 58 | IDX\_TSA\_DN\_CLUSTER5 |  | 46 | 0.63 | 1.71 | 0.000 | 0.019 | 0.668 | 5027 | tags=54%, list=24%, signal=72% |
| 59 | UVC\_TTD-XPCS\_COMMON\_DN |  | 144 | 0.57 | 1.70 | 0.000 | 0.020 | 0.687 | 5307 | tags=58%, list=26%, signal=77% |
| 60 | WALKER\_MM\_SNP\_DIFF |  | 44 | 0.62 | 1.70 | 0.000 | 0.021 | 0.709 | 2182 | tags=34%, list=11%, signal=38% |
| 61 | TPA\_SENS\_MIDDLE\_UP |  | 65 | 0.61 | 1.70 | 0.000 | 0.020 | 0.710 | 1353 | tags=23%, list=7%, signal=25% |
| 62 | HTERT\_UP |  | 67 | 0.60 | 1.69 | 0.001 | 0.021 | 0.732 | 1977 | tags=28%, list=10%, signal=31% |
| 63 | GALE\_FLT3ANDAPL\_UP |  | 59 | 0.60 | 1.69 | 0.000 | 0.023 | 0.761 | 4492 | tags=51%, list=22%, signal=65% |
| 64 | UVC\_HIGH\_ALL\_DN |  | 296 | 0.55 | 1.69 | 0.000 | 0.023 | 0.762 | 3783 | tags=43%, list=18%, signal=52% |
| 65 | IGF1\_NIH3T3\_UP |  | 35 | 0.65 | 1.69 | 0.001 | 0.023 | 0.767 | 1944 | tags=31%, list=9%, signal=35% |
| 66 | DSRNA\_UP |  | 38 | 0.64 | 1.69 | 0.001 | 0.023 | 0.773 | 2567 | tags=34%, list=12%, signal=39% |
| 67 | ET743\_HELA\_UP |  | 56 | 0.61 | 1.68 | 0.000 | 0.023 | 0.782 | 1151 | tags=23%, list=6%, signal=25% |
| 68 | BRCA1\_OVEREXP\_UP |  | 159 | 0.56 | 1.68 | 0.000 | 0.024 | 0.798 | 4425 | tags=46%, list=21%, signal=58% |
| 69 | UVB\_NHEK3\_C5 |  | 35 | 0.64 | 1.68 | 0.000 | 0.025 | 0.815 | 4141 | tags=60%, list=20%, signal=75% |
| 70 | INFLAMPATHWAY |  | 29 | 0.66 | 1.67 | 0.001 | 0.026 | 0.831 | 630 | tags=14%, list=3%, signal=14% |
| 71 | IL1\_CORNEA\_UP |  | 62 | 0.59 | 1.67 | 0.002 | 0.028 | 0.858 | 1450 | tags=21%, list=7%, signal=22% |
| 72 | CMV\_HCMV\_TIMECOURSE\_18HRS\_UP |  | 74 | 0.58 | 1.67 | 0.000 | 0.028 | 0.859 | 2035 | tags=31%, list=10%, signal=34% |
| 73 | MUNSHI\_MM\_UP |  | 65 | 0.59 | 1.67 | 0.000 | 0.028 | 0.861 | 4217 | tags=38%, list=20%, signal=48% |
| 74 | BECKER\_TAMOXIFEN\_RESISTANT\_DN |  | 51 | 0.61 | 1.67 | 0.001 | 0.029 | 0.872 | 4249 | tags=49%, list=21%, signal=62% |
| 75 | WELCSH\_BRCA\_UP |  | 39 | 0.62 | 1.67 | 0.005 | 0.028 | 0.872 | 2545 | tags=33%, list=12%, signal=38% |
| 76 | ADIPOGENESIS\_HMSC\_CLASS1\_UP |  | 18 | 0.71 | 1.66 | 0.001 | 0.029 | 0.884 | 1858 | tags=33%, list=9%, signal=37% |
| 77 | RESISTANCE\_XENOGRAFTS\_UP |  | 28 | 0.65 | 1.66 | 0.001 | 0.029 | 0.887 | 594 | tags=21%, list=3%, signal=22% |
| 78 | TRANSLATION\_FACTORS |  | 47 | 0.60 | 1.66 | 0.003 | 0.029 | 0.894 | 3905 | tags=49%, list=19%, signal=60% |
| 79 | LEI\_HOXC8\_DN |  | 15 | 0.74 | 1.66 | 0.004 | 0.029 | 0.894 | 1358 | tags=40%, list=7%, signal=43% |
| 80 | RADMACHER\_AMLNORMALKARYTYPE\_SIG |  | 83 | 0.57 | 1.66 | 0.000 | 0.029 | 0.894 | 3828 | tags=42%, list=19%, signal=52% |
| 81 | HTERT\_DN |  | 70 | 0.59 | 1.66 | 0.001 | 0.029 | 0.906 | 4292 | tags=39%, list=21%, signal=49% |
| 82 | AGUIRRE\_PANCREAS\_CHR9 |  | 24 | 0.67 | 1.66 | 0.007 | 0.029 | 0.908 | 3686 | tags=50%, list=18%, signal=61% |
| 83 | WILLERT\_WNT\_NCCIT\_ALL\_UP |  | 23 | 0.67 | 1.66 | 0.004 | 0.030 | 0.917 | 5621 | tags=74%, list=27%, signal=102% |
| 84 | HINATA\_NFKB\_DN |  | 20 | 0.69 | 1.66 | 0.010 | 0.030 | 0.917 | 1010 | tags=30%, list=5%, signal=32% |
| 85 | UVB\_NHEK1\_C1 |  | 51 | 0.61 | 1.65 | 0.001 | 0.029 | 0.918 | 3938 | tags=43%, list=19%, signal=53% |
| 86 | CCR5PATHWAY |  | 17 | 0.71 | 1.65 | 0.007 | 0.029 | 0.920 | 1522 | tags=29%, list=7%, signal=32% |
| 87 | BRCA\_BRCA1\_POS |  | 106 | 0.57 | 1.65 | 0.000 | 0.030 | 0.925 | 3814 | tags=36%, list=19%, signal=44% |
| 88 | GH\_GHRHR\_KO\_6HRS\_UP |  | 69 | 0.58 | 1.65 | 0.000 | 0.033 | 0.944 | 4667 | tags=45%, list=23%, signal=58% |
| 89 | KRETZSCHMAR\_IL6\_DIFF |  | 145 | 0.55 | 1.64 | 0.000 | 0.033 | 0.948 | 5298 | tags=52%, list=26%, signal=69% |
| 90 | MUNSHI\_MM\_VS\_PCS\_UP |  | 77 | 0.57 | 1.64 | 0.000 | 0.033 | 0.948 | 4217 | tags=36%, list=20%, signal=46% |
| 91 | PASSERINI\_PROLIFERATION |  | 64 | 0.58 | 1.64 | 0.003 | 0.033 | 0.951 | 3585 | tags=33%, list=17%, signal=40% |
| 92 | BAF57\_BT549\_UP |  | 238 | 0.54 | 1.64 | 0.000 | 0.033 | 0.952 | 4858 | tags=41%, list=24%, signal=53% |
| 93 | CROMER\_HYPOPHARYNGEAL\_MET\_VS\_NON\_UP |  | 72 | 0.57 | 1.64 | 0.000 | 0.033 | 0.953 | 1344 | tags=25%, list=7%, signal=27% |
| 94 | HADDAD\_HPCLYMPHO\_ENRICHED |  | 309 | 0.54 | 1.64 | 0.000 | 0.033 | 0.954 | 3814 | tags=37%, list=19%, signal=44% |
| 95 | BYSTROM\_IL5\_DN |  | 57 | 0.59 | 1.64 | 0.001 | 0.033 | 0.956 | 5387 | tags=56%, list=26%, signal=76% |
| 96 | LEE\_TCELLS7\_UP |  | 15 | 0.72 | 1.64 | 0.006 | 0.033 | 0.960 | 1642 | tags=40%, list=8%, signal=43% |
| 97 | ROME\_INSULIN\_2F\_DN |  | 25 | 0.67 | 1.64 | 0.006 | 0.033 | 0.960 | 4220 | tags=44%, list=20%, signal=55% |
| 98 | TGF\_BETA\_SIGNALING\_PATHWAY |  | 49 | 0.60 | 1.64 | 0.001 | 0.033 | 0.960 | 1956 | tags=31%, list=9%, signal=34% |
| 99 | BROCKE\_IL6 |  | 145 | 0.55 | 1.64 | 0.000 | 0.032 | 0.961 | 5298 | tags=52%, list=26%, signal=69% |
| 100 | CHIARETTI\_T\_ALL |  | 255 | 0.54 | 1.64 | 0.000 | 0.033 | 0.967 | 4351 | tags=39%, list=21%, signal=49% |
| 101 | OLDONLY\_FIBRO\_DN |  | 53 | 0.59 | 1.63 | 0.003 | 0.033 | 0.969 | 4900 | tags=55%, list=24%, signal=72% |
| 102 | BRG1\_ALAB\_UP |  | 40 | 0.60 | 1.63 | 0.001 | 0.035 | 0.974 | 2755 | tags=30%, list=13%, signal=35% |
| 103 | VERNELL\_PRB\_CLSTR2 |  | 22 | 0.68 | 1.63 | 0.006 | 0.036 | 0.982 | 3232 | tags=55%, list=16%, signal=65% |
| 104 | CALCINEURINPATHWAY |  | 19 | 0.68 | 1.63 | 0.010 | 0.036 | 0.983 | 4361 | tags=58%, list=21%, signal=73% |
| 105 | BREAST\_CANCER\_ESTROGEN\_SIGNALING |  | 93 | 0.56 | 1.63 | 0.000 | 0.035 | 0.983 | 2064 | tags=29%, list=10%, signal=32% |
| 106 | LVAD\_HEARTFAILURE\_DN |  | 38 | 0.61 | 1.63 | 0.002 | 0.035 | 0.984 | 3993 | tags=39%, list=19%, signal=49% |
| 107 | WERNERONLY\_FIBRO\_DN |  | 64 | 0.58 | 1.63 | 0.001 | 0.035 | 0.985 | 2831 | tags=34%, list=14%, signal=40% |
| 108 | UVC\_HIGH\_D8\_DN |  | 30 | 0.64 | 1.62 | 0.008 | 0.036 | 0.987 | 3342 | tags=43%, list=16%, signal=52% |
| 109 | HDACI\_COLON\_SUL2HRS\_DN |  | 17 | 0.70 | 1.62 | 0.009 | 0.036 | 0.987 | 2994 | tags=53%, list=15%, signal=62% |
| 110 | BREASTCA\_TWO\_CLASSES |  | 138 | 0.55 | 1.62 | 0.000 | 0.036 | 0.989 | 4882 | tags=49%, list=24%, signal=64% |
| 111 | SANA\_TNFA\_ENDOTHELIAL\_UP |  | 80 | 0.56 | 1.62 | 0.000 | 0.037 | 0.989 | 1482 | tags=23%, list=7%, signal=24% |
| 112 | CMV\_UV-CMV\_COMMON\_HCMV\_6HRS\_DN |  | 27 | 0.64 | 1.62 | 0.005 | 0.037 | 0.989 | 4440 | tags=48%, list=22%, signal=61% |
| 113 | CHIARETTI\_T\_ALL\_DIFF |  | 278 | 0.53 | 1.62 | 0.000 | 0.038 | 0.989 | 4351 | tags=37%, list=21%, signal=46% |
| 114 | LINDSTEDT\_DEND\_8H\_VS\_48H\_UP |  | 64 | 0.57 | 1.62 | 0.000 | 0.038 | 0.989 | 3140 | tags=30%, list=15%, signal=35% |
| 115 | OKUMURA\_MC\_LPS |  | 185 | 0.54 | 1.61 | 0.000 | 0.039 | 0.989 | 4414 | tags=41%, list=21%, signal=52% |
| 116 | STRESS\_TPA\_SPECIFIC\_UP |  | 42 | 0.61 | 1.61 | 0.005 | 0.039 | 0.990 | 2575 | tags=31%, list=12%, signal=35% |
| 117 | UVC\_LOW\_ALL\_DN |  | 58 | 0.58 | 1.61 | 0.002 | 0.039 | 0.990 | 3448 | tags=41%, list=17%, signal=50% |
| 118 | JNK\_DN |  | 31 | 0.64 | 1.61 | 0.006 | 0.038 | 0.990 | 4731 | tags=52%, list=23%, signal=67% |
| 119 | AT1RPATHWAY |  | 34 | 0.61 | 1.61 | 0.003 | 0.040 | 0.993 | 4184 | tags=53%, list=20%, signal=66% |
| 120 | RORIE\_ES\_PNET\_UP |  | 27 | 0.64 | 1.61 | 0.013 | 0.040 | 0.993 | 3167 | tags=52%, list=15%, signal=61% |
| 121 | TAKEDA\_NUP8\_HOXA9\_6H\_DN |  | 40 | 0.61 | 1.61 | 0.003 | 0.041 | 0.993 | 573 | tags=23%, list=3%, signal=23% |
| 122 | UVB\_NHEK2\_DN |  | 81 | 0.56 | 1.60 | 0.000 | 0.044 | 0.996 | 2696 | tags=37%, list=13%, signal=42% |
| 123 | ST\_ERK1\_ERK2\_MAPK\_PATHWAY |  | 30 | 0.62 | 1.60 | 0.007 | 0.044 | 0.996 | 4297 | tags=50%, list=21%, signal=63% |
| 124 | H2O2\_CSBDIFF\_C1 |  | 33 | 0.61 | 1.60 | 0.008 | 0.044 | 0.996 | 2438 | tags=39%, list=12%, signal=45% |
| 125 | HADDAD\_HSC\_CD10\_UP |  | 296 | 0.52 | 1.60 | 0.000 | 0.044 | 0.996 | 3814 | tags=35%, list=19%, signal=42% |
| 126 | HDACI\_COLON\_SUL12HRS\_UP |  | 26 | 0.64 | 1.60 | 0.007 | 0.044 | 0.996 | 2727 | tags=46%, list=13%, signal=53% |
| 127 | 4NQO\_ESR\_WS\_UNREG |  | 35 | 0.61 | 1.59 | 0.010 | 0.046 | 0.997 | 4610 | tags=43%, list=22%, signal=55% |
| 128 | MCALPAINPATHWAY |  | 24 | 0.65 | 1.59 | 0.011 | 0.046 | 0.998 | 3325 | tags=46%, list=16%, signal=55% |
| 129 | BRG1\_H1299\_UP |  | 36 | 0.61 | 1.59 | 0.003 | 0.047 | 0.999 | 1630 | tags=28%, list=8%, signal=30% |
| 130 | FSH\_GRANULOSA\_DN |  | 76 | 0.55 | 1.59 | 0.001 | 0.046 | 0.999 | 3043 | tags=38%, list=15%, signal=45% |
| 131 | BRUNO\_IL3\_DN |  | 63 | 0.57 | 1.59 | 0.001 | 0.046 | 0.999 | 2813 | tags=33%, list=14%, signal=38% |
| 132 | CMV\_HCMV\_TIMECOURSE\_ALL\_DN |  | 420 | 0.52 | 1.59 | 0.000 | 0.046 | 0.999 | 4332 | tags=37%, list=21%, signal=46% |
| 133 | ST\_JNK\_MAPK\_PATHWAY |  | 40 | 0.60 | 1.59 | 0.003 | 0.046 | 0.999 | 3445 | tags=43%, list=17%, signal=51% |
| 134 | HDACI\_COLON\_CLUSTER6 |  | 43 | 0.59 | 1.59 | 0.004 | 0.046 | 0.999 | 2490 | tags=35%, list=12%, signal=40% |
| 135 | OLDWERNER\_FIBRO\_UP |  | 25 | 0.65 | 1.59 | 0.004 | 0.046 | 0.999 | 2150 | tags=32%, list=10%, signal=36% |
| 136 | ALZHEIMERS\_INCIPIENT\_UP |  | 342 | 0.52 | 1.59 | 0.000 | 0.046 | 1.000 | 5263 | tags=46%, list=26%, signal=60% |
| 137 | KIM\_TH\_CELLS\_UP |  | 46 | 0.60 | 1.59 | 0.000 | 0.046 | 1.000 | 4209 | tags=46%, list=20%, signal=57% |
| 138 | MMS\_HUMAN\_LYMPH\_LOW\_4HRS\_DN |  | 16 | 0.69 | 1.59 | 0.010 | 0.047 | 1.000 | 3772 | tags=56%, list=18%, signal=69% |
| 139 | CMV\_HCMV\_TIMECOURSE\_8HRS\_DN |  | 16 | 0.69 | 1.58 | 0.015 | 0.048 | 1.000 | 3338 | tags=69%, list=16%, signal=82% |
| 140 | LH\_GRANULOSA\_DN |  | 76 | 0.55 | 1.58 | 0.000 | 0.048 | 1.000 | 3043 | tags=38%, list=15%, signal=45% |
| 141 | IDX\_TSA\_DN\_CLUSTER6 |  | 27 | 0.63 | 1.58 | 0.011 | 0.048 | 1.000 | 793 | tags=22%, list=4%, signal=23% |
| 142 | ZMPSTE24\_KO\_UP |  | 31 | 0.62 | 1.58 | 0.006 | 0.048 | 1.000 | 4257 | tags=45%, list=21%, signal=57% |
| 143 | KNUDSEN\_PMNS\_UP |  | 74 | 0.55 | 1.58 | 0.003 | 0.048 | 1.000 | 6090 | tags=54%, list=30%, signal=76% |
| 144 | IGLESIAS\_E2FMINUS\_UP |  | 136 | 0.53 | 1.58 | 0.000 | 0.048 | 1.000 | 4536 | tags=35%, list=22%, signal=45% |
| 145 | AGEING\_KIDNEY\_SPECIFIC\_DN |  | 132 | 0.53 | 1.58 | 0.001 | 0.050 | 1.000 | 4249 | tags=39%, list=21%, signal=49% |
| 146 | BASSO\_HCL\_DIFF |  | 86 | 0.55 | 1.58 | 0.001 | 0.050 | 1.000 | 4023 | tags=41%, list=20%, signal=50% |
| 147 | PGC1APATHWAY |  | 23 | 0.65 | 1.57 | 0.008 | 0.052 | 1.000 | 4184 | tags=57%, list=20%, signal=71% |
| 148 | EMT\_DN |  | 54 | 0.57 | 1.57 | 0.006 | 0.052 | 1.000 | 3167 | tags=39%, list=15%, signal=46% |
| 149 | LINDSTEDT\_DEND\_UP |  | 50 | 0.58 | 1.57 | 0.005 | 0.052 | 1.000 | 1010 | tags=24%, list=5%, signal=25% |
| 150 | TPA\_SENS\_LATE\_UP |  | 52 | 0.57 | 1.57 | 0.006 | 0.053 | 1.000 | 1102 | tags=21%, list=5%, signal=22% |
| 151 | BRENTANI\_CELL\_ADHESION |  | 92 | 0.54 | 1.57 | 0.000 | 0.054 | 1.000 | 3140 | tags=28%, list=15%, signal=33% |
| 152 | CMV\_HCMV\_6HRS\_DN |  | 55 | 0.57 | 1.57 | 0.006 | 0.054 | 1.000 | 4440 | tags=45%, list=22%, signal=58% |
| 153 | HDACI\_COLON\_SUL\_DN |  | 215 | 0.52 | 1.57 | 0.000 | 0.054 | 1.000 | 3283 | tags=32%, list=16%, signal=37% |
| 154 | ZHAN\_MM\_MOLECULAR\_CLASSI\_DN |  | 50 | 0.57 | 1.56 | 0.010 | 0.057 | 1.000 | 3459 | tags=38%, list=17%, signal=46% |
| 155 | SIG\_CHEMOTAXIS |  | 44 | 0.58 | 1.56 | 0.010 | 0.057 | 1.000 | 4751 | tags=41%, list=23%, signal=53% |
| 156 | LIZUKA\_L0\_GR\_L1 |  | 15 | 0.69 | 1.56 | 0.011 | 0.057 | 1.000 | 310 | tags=13%, list=2%, signal=14% |
| 157 | TSA\_PANC50\_UP |  | 38 | 0.60 | 1.56 | 0.010 | 0.058 | 1.000 | 3167 | tags=34%, list=15%, signal=40% |
| 158 | MENSE\_HYPOXIA\_TRANSPORTER\_GENES |  | 48 | 0.57 | 1.56 | 0.009 | 0.058 | 1.000 | 4269 | tags=40%, list=21%, signal=50% |
| 159 | HEARTFAILURE\_ATRIA\_DN |  | 111 | 0.53 | 1.56 | 0.002 | 0.058 | 1.000 | 3407 | tags=32%, list=17%, signal=39% |
| 160 | PASSERINI\_SIGNAL |  | 338 | 0.51 | 1.56 | 0.000 | 0.058 | 1.000 | 3305 | tags=27%, list=16%, signal=31% |
| 161 | UVC\_HIGH\_D6\_DN |  | 31 | 0.61 | 1.56 | 0.014 | 0.059 | 1.000 | 3783 | tags=58%, list=18%, signal=71% |
| 162 | HDACI\_COLON\_CUR48HRS\_UP |  | 62 | 0.55 | 1.56 | 0.005 | 0.059 | 1.000 | 4239 | tags=47%, list=21%, signal=59% |
| 163 | BRACX\_UP |  | 22 | 0.63 | 1.55 | 0.016 | 0.059 | 1.000 | 3345 | tags=50%, list=16%, signal=60% |
| 164 | CISPLATIN\_PROBCELL\_UP |  | 17 | 0.67 | 1.55 | 0.025 | 0.059 | 1.000 | 4158 | tags=41%, list=20%, signal=52% |
| 165 | TGFBETA\_LATE\_UP |  | 33 | 0.59 | 1.55 | 0.013 | 0.059 | 1.000 | 2398 | tags=30%, list=12%, signal=34% |
| 166 | GH\_AUTOCRINE\_DN |  | 122 | 0.53 | 1.55 | 0.000 | 0.062 | 1.000 | 4213 | tags=35%, list=20%, signal=44% |
| 167 | HYPOXIA\_RCC\_UP |  | 102 | 0.53 | 1.55 | 0.002 | 0.062 | 1.000 | 4692 | tags=49%, list=23%, signal=63% |
| 168 | GH\_GHRHR\_KO\_24HRS\_UP |  | 142 | 0.52 | 1.55 | 0.000 | 0.062 | 1.000 | 4510 | tags=39%, list=22%, signal=49% |
| 169 | HALMOS\_CEBP\_DN |  | 43 | 0.58 | 1.55 | 0.009 | 0.062 | 1.000 | 3267 | tags=44%, list=16%, signal=52% |
| 170 | TPA\_SENS\_EARLY\_UP |  | 47 | 0.57 | 1.55 | 0.010 | 0.062 | 1.000 | 2731 | tags=23%, list=13%, signal=27% |
| 171 | MTORPATHWAY |  | 23 | 0.63 | 1.55 | 0.013 | 0.062 | 1.000 | 4328 | tags=52%, list=21%, signal=66% |
| 172 | FRASOR\_ER\_DN |  | 61 | 0.55 | 1.55 | 0.003 | 0.062 | 1.000 | 5603 | tags=52%, list=27%, signal=72% |
| 173 | LEE\_TCELLS5\_UP |  | 19 | 0.66 | 1.54 | 0.021 | 0.063 | 1.000 | 1209 | tags=26%, list=6%, signal=28% |
| 174 | INSULIN\_NIH3T3\_UP |  | 17 | 0.66 | 1.54 | 0.025 | 0.063 | 1.000 | 3585 | tags=41%, list=17%, signal=50% |
| 175 | GAMMA\_ESR\_WS\_UNREG |  | 28 | 0.59 | 1.54 | 0.019 | 0.063 | 1.000 | 2630 | tags=32%, list=13%, signal=37% |
| 176 | HDACI\_COLON\_CLUSTER10 |  | 43 | 0.57 | 1.54 | 0.011 | 0.064 | 1.000 | 4296 | tags=47%, list=21%, signal=59% |
| 177 | CORDERO\_KRAS\_KD\_VS\_CONTROL\_DN |  | 55 | 0.56 | 1.54 | 0.006 | 0.064 | 1.000 | 4191 | tags=42%, list=20%, signal=52% |
| 178 | HSC\_MATURE\_ADULT |  | 331 | 0.50 | 1.54 | 0.000 | 0.065 | 1.000 | 4425 | tags=34%, list=21%, signal=43% |
| 179 | CREB\_BRAIN\_2WKS\_UP |  | 24 | 0.63 | 1.54 | 0.013 | 0.064 | 1.000 | 3381 | tags=50%, list=16%, signal=60% |
| 180 | WERNER\_FIBRO\_DN |  | 168 | 0.51 | 1.54 | 0.000 | 0.066 | 1.000 | 3432 | tags=33%, list=17%, signal=39% |
| 181 | UVB\_NHEK3\_C2 |  | 43 | 0.58 | 1.54 | 0.008 | 0.065 | 1.000 | 4400 | tags=58%, list=21%, signal=74% |
| 182 | MENSSEN\_MYC\_UP |  | 31 | 0.60 | 1.54 | 0.014 | 0.065 | 1.000 | 2991 | tags=39%, list=15%, signal=45% |
| 183 | RHOPATHWAY |  | 29 | 0.60 | 1.54 | 0.021 | 0.066 | 1.000 | 4748 | tags=45%, list=23%, signal=58% |
| 184 | WANG\_HOXA9\_VS\_MEIS1\_UP |  | 27 | 0.61 | 1.54 | 0.013 | 0.066 | 1.000 | 1289 | tags=15%, list=6%, signal=16% |
| 185 | HCC\_SURVIVAL\_GOOD\_VS\_POOR\_DN |  | 129 | 0.52 | 1.53 | 0.000 | 0.068 | 1.000 | 4985 | tags=51%, list=24%, signal=67% |
| 186 | ZHAN\_MM\_CD1\_VS\_CD2\_UP |  | 91 | 0.53 | 1.53 | 0.002 | 0.067 | 1.000 | 5393 | tags=49%, list=26%, signal=67% |
| 187 | BRCA1KO\_MEF\_DN |  | 79 | 0.54 | 1.53 | 0.002 | 0.068 | 1.000 | 4872 | tags=51%, list=24%, signal=66% |
| 188 | HALMOS\_CEBP\_UP |  | 50 | 0.56 | 1.53 | 0.004 | 0.068 | 1.000 | 1827 | tags=22%, list=9%, signal=24% |
| 189 | ZUCCHI\_EPITHELIAL\_UP |  | 42 | 0.57 | 1.53 | 0.015 | 0.069 | 1.000 | 3217 | tags=40%, list=16%, signal=48% |
| 190 | MOREAUX\_TACI\_HI\_VS\_LOW\_DN |  | 170 | 0.51 | 1.53 | 0.000 | 0.069 | 1.000 | 3587 | tags=42%, list=17%, signal=50% |
| 191 | CHREBPPATHWAY |  | 17 | 0.66 | 1.53 | 0.023 | 0.069 | 1.000 | 3325 | tags=35%, list=16%, signal=42% |
| 192 | TNFA\_NFKB\_DEP\_UP |  | 18 | 0.65 | 1.53 | 0.021 | 0.069 | 1.000 | 911 | tags=17%, list=4%, signal=17% |
| 193 | STEMCELL\_COMMON\_UP |  | 180 | 0.51 | 1.53 | 0.000 | 0.069 | 1.000 | 4719 | tags=47%, list=23%, signal=61% |
| 194 | HDACI\_COLON\_CUR\_UP |  | 108 | 0.52 | 1.53 | 0.001 | 0.069 | 1.000 | 4239 | tags=43%, list=21%, signal=53% |
| 195 | HDACI\_COLON\_SUL24HRS\_UP |  | 64 | 0.54 | 1.53 | 0.005 | 0.069 | 1.000 | 4410 | tags=50%, list=21%, signal=63% |
| 196 | VHL\_NORMAL\_UP |  | 438 | 0.50 | 1.53 | 0.000 | 0.068 | 1.000 | 4501 | tags=40%, list=22%, signal=51% |
| 197 | NOVA2\_KO\_SPLICING |  | 42 | 0.56 | 1.53 | 0.020 | 0.069 | 1.000 | 3156 | tags=31%, list=15%, signal=36% |
| 198 | TSADAC\_HYPOMETH\_OVCA\_UP |  | 52 | 0.55 | 1.52 | 0.008 | 0.069 | 1.000 | 3135 | tags=31%, list=15%, signal=36% |
| 199 | IDX\_TSA\_UP\_CLUSTER1 |  | 25 | 0.61 | 1.52 | 0.025 | 0.070 | 1.000 | 3927 | tags=56%, list=19%, signal=69% |
| 200 | POMEROY\_MD\_TREATMENT\_GOOD\_VS\_POOR\_DN |  | 24 | 0.61 | 1.52 | 0.018 | 0.069 | 1.000 | 2832 | tags=38%, list=14%, signal=43% |
| 201 | LEE\_DENA\_UP |  | 59 | 0.55 | 1.52 | 0.004 | 0.069 | 1.000 | 2188 | tags=31%, list=11%, signal=34% |
| 202 | GREENBAUM\_E2A\_UP |  | 33 | 0.59 | 1.52 | 0.018 | 0.069 | 1.000 | 5447 | tags=58%, list=26%, signal=78% |
| 203 | HDACI\_COLON\_BUT16HRS\_DN |  | 108 | 0.52 | 1.52 | 0.003 | 0.069 | 1.000 | 2261 | tags=28%, list=11%, signal=31% |
| 204 | UVB\_SCC\_DN |  | 105 | 0.52 | 1.52 | 0.000 | 0.069 | 1.000 | 4910 | tags=54%, list=24%, signal=71% |
| 205 | IFN\_BETA\_GLIOMA\_DN |  | 44 | 0.56 | 1.52 | 0.009 | 0.069 | 1.000 | 3396 | tags=32%, list=16%, signal=38% |
| 206 | DORSAM\_HOXA9\_UP |  | 32 | 0.59 | 1.52 | 0.021 | 0.070 | 1.000 | 5629 | tags=53%, list=27%, signal=73% |
| 207 | FLECHNER\_KIDNEY\_TRANSPLANT\_REJECTION\_PBL\_UP |  | 64 | 0.53 | 1.52 | 0.005 | 0.070 | 1.000 | 3877 | tags=39%, list=19%, signal=48% |
| 208 | TENEDINI\_MEGAKARYOCYTIC\_GENES |  | 53 | 0.55 | 1.52 | 0.005 | 0.070 | 1.000 | 6440 | tags=49%, list=31%, signal=71% |
| 209 | RUIZ\_TENASCIN\_TARGETS |  | 79 | 0.53 | 1.52 | 0.003 | 0.070 | 1.000 | 2430 | tags=27%, list=12%, signal=30% |
| 210 | NGUYEN\_KERATO\_DN |  | 81 | 0.53 | 1.52 | 0.000 | 0.069 | 1.000 | 4804 | tags=44%, list=23%, signal=58% |
| 211 | ET743\_SARCOMA\_6HRS\_UP |  | 30 | 0.60 | 1.52 | 0.017 | 0.070 | 1.000 | 3762 | tags=40%, list=18%, signal=49% |
| 212 | ET743\_SARCOMA\_72HRS\_DN |  | 222 | 0.50 | 1.52 | 0.000 | 0.069 | 1.000 | 4752 | tags=44%, list=23%, signal=56% |
| 213 | HSC\_MATURE\_SHARED |  | 251 | 0.51 | 1.52 | 0.000 | 0.070 | 1.000 | 4425 | tags=36%, list=21%, signal=45% |
| 214 | WONG\_IFNA\_HCC\_RESISTANT\_VS\_SENSITIVE\_UP |  | 15 | 0.68 | 1.52 | 0.031 | 0.070 | 1.000 | 3016 | tags=40%, list=15%, signal=47% |
| 215 | AGED\_MOUSE\_CORTEX\_UP |  | 31 | 0.60 | 1.52 | 0.014 | 0.070 | 1.000 | 5005 | tags=61%, list=24%, signal=81% |
| 216 | HDACI\_COLON\_BUT\_DN |  | 248 | 0.50 | 1.52 | 0.000 | 0.070 | 1.000 | 4204 | tags=38%, list=20%, signal=47% |
| 217 | IDX\_TSA\_DN\_CLUSTER1 |  | 42 | 0.56 | 1.52 | 0.012 | 0.070 | 1.000 | 2813 | tags=31%, list=14%, signal=36% |
| 218 | GH\_GHRHR\_KO\_24HRS\_DN |  | 172 | 0.51 | 1.52 | 0.000 | 0.070 | 1.000 | 3954 | tags=33%, list=19%, signal=41% |
| 219 | VEGF\_MMMEC\_6HRS\_UP |  | 50 | 0.55 | 1.52 | 0.011 | 0.070 | 1.000 | 1528 | tags=18%, list=7%, signal=19% |
| 220 | OLD\_FIBRO\_DN |  | 158 | 0.51 | 1.52 | 0.001 | 0.070 | 1.000 | 3549 | tags=35%, list=17%, signal=42% |
| 221 | PASSERINI\_TRANSCRIPTION |  | 74 | 0.53 | 1.52 | 0.010 | 0.070 | 1.000 | 5406 | tags=45%, list=26%, signal=60% |
| 222 | WERNER\_FIBRO\_UP |  | 56 | 0.55 | 1.51 | 0.004 | 0.070 | 1.000 | 4008 | tags=36%, list=19%, signal=44% |
| 223 | HDACI\_COLON\_SUL48HRS\_DN |  | 81 | 0.53 | 1.51 | 0.003 | 0.070 | 1.000 | 2614 | tags=27%, list=13%, signal=31% |
| 224 | HDACI\_COLON\_CUR12HRS\_UP |  | 21 | 0.63 | 1.51 | 0.022 | 0.070 | 1.000 | 2434 | tags=43%, list=12%, signal=49% |
| 225 | NOS1PATHWAY |  | 22 | 0.63 | 1.51 | 0.026 | 0.070 | 1.000 | 5252 | tags=50%, list=25%, signal=67% |
| 226 | OXSTRESS\_RPE\_HNETBH\_DN |  | 47 | 0.55 | 1.51 | 0.014 | 0.070 | 1.000 | 4609 | tags=38%, list=22%, signal=49% |
| 227 | AGEING\_KIDNEY\_SPECIFIC\_UP |  | 183 | 0.50 | 1.51 | 0.000 | 0.071 | 1.000 | 3058 | tags=31%, list=15%, signal=36% |
| 228 | FLOTHO\_CASP8AP2\_MRD\_DIFF |  | 84 | 0.52 | 1.51 | 0.002 | 0.071 | 1.000 | 3921 | tags=39%, list=19%, signal=48% |
| 229 | UVB\_NHEK1\_C2 |  | 21 | 0.63 | 1.51 | 0.019 | 0.072 | 1.000 | 3167 | tags=38%, list=15%, signal=45% |
| 230 | VERNELL\_PRB\_CLSTR1 |  | 67 | 0.53 | 1.51 | 0.007 | 0.072 | 1.000 | 3425 | tags=36%, list=17%, signal=43% |
| 231 | UVC\_HIGH\_D2\_DN |  | 37 | 0.57 | 1.51 | 0.011 | 0.072 | 1.000 | 1997 | tags=38%, list=10%, signal=42% |
| 232 | NTHIPATHWAY |  | 21 | 0.63 | 1.51 | 0.031 | 0.072 | 1.000 | 4050 | tags=38%, list=20%, signal=47% |
| 233 | ST\_TUMOR\_NECROSIS\_FACTOR\_PATHWAY |  | 29 | 0.59 | 1.51 | 0.017 | 0.072 | 1.000 | 4856 | tags=48%, list=24%, signal=63% |
| 234 | INSULIN\_ADIP\_INSENS\_UP |  | 23 | 0.62 | 1.51 | 0.016 | 0.073 | 1.000 | 1227 | tags=22%, list=6%, signal=23% |
| 235 | KIM\_TH\_CELLS\_DN |  | 15 | 0.67 | 1.51 | 0.041 | 0.073 | 1.000 | 1006 | tags=27%, list=5%, signal=28% |
| 236 | ZUCCHI\_EPITHELIAL\_DN |  | 43 | 0.56 | 1.51 | 0.020 | 0.073 | 1.000 | 724 | tags=19%, list=4%, signal=19% |
| 237 | HDACI\_COLON\_TSA\_DN |  | 64 | 0.54 | 1.51 | 0.003 | 0.073 | 1.000 | 3600 | tags=36%, list=17%, signal=43% |
| 238 | VANTVEER\_BREAST\_OUTCOME\_GOOD\_VS\_POOR\_DN |  | 64 | 0.54 | 1.50 | 0.012 | 0.073 | 1.000 | 4439 | tags=45%, list=22%, signal=58% |
| 239 | HDACI\_COLON\_BUT48HRS\_DN |  | 116 | 0.51 | 1.50 | 0.001 | 0.074 | 1.000 | 3927 | tags=40%, list=19%, signal=49% |
| 240 | UVC\_HIGH\_D9\_DN |  | 24 | 0.60 | 1.50 | 0.029 | 0.074 | 1.000 | 5233 | tags=67%, list=25%, signal=89% |
| 241 | LEE\_TCELLS4\_UP |  | 58 | 0.54 | 1.50 | 0.011 | 0.079 | 1.000 | 3899 | tags=38%, list=19%, signal=47% |
| 242 | AGUIRRE\_PANCREAS\_CHR12 |  | 59 | 0.54 | 1.50 | 0.011 | 0.079 | 1.000 | 6133 | tags=64%, list=30%, signal=91% |
| 243 | ZHAN\_MM\_CD138\_LB\_VS\_REST |  | 42 | 0.56 | 1.50 | 0.016 | 0.079 | 1.000 | 2270 | tags=24%, list=11%, signal=27% |
| 244 | INSULIN\_ADIP\_SENS\_DN |  | 17 | 0.64 | 1.49 | 0.031 | 0.080 | 1.000 | 3942 | tags=41%, list=19%, signal=51% |
| 245 | CANCERDRUGS\_PROBCELL\_UP |  | 20 | 0.62 | 1.49 | 0.043 | 0.079 | 1.000 | 1542 | tags=20%, list=7%, signal=22% |
| 246 | ET743\_RESIST\_DN |  | 40 | 0.56 | 1.49 | 0.025 | 0.080 | 1.000 | 3800 | tags=40%, list=18%, signal=49% |
| 247 | ET743PT650\_COLONCA\_DN |  | 44 | 0.55 | 1.49 | 0.019 | 0.081 | 1.000 | 6479 | tags=70%, list=31%, signal=103% |
| 248 | AGED\_MOUSE\_RETINA\_ANY\_UP |  | 21 | 0.61 | 1.49 | 0.039 | 0.081 | 1.000 | 2768 | tags=29%, list=13%, signal=33% |
| 249 | CELL\_ADHESION |  | 173 | 0.50 | 1.49 | 0.000 | 0.080 | 1.000 | 2984 | tags=22%, list=14%, signal=25% |
| 250 | ABRAHAM\_MM\_VS\_AL\_DN |  | 22 | 0.61 | 1.49 | 0.024 | 0.083 | 1.000 | 3349 | tags=50%, list=16%, signal=60% |
| 251 | MMS\_MOUSE\_LYMPH\_HIGH\_4HRS\_UP |  | 35 | 0.58 | 1.49 | 0.022 | 0.083 | 1.000 | 3444 | tags=49%, list=17%, signal=58% |
| 252 | ET743\_SARCOMA\_DN |  | 269 | 0.49 | 1.49 | 0.000 | 0.083 | 1.000 | 4284 | tags=42%, list=21%, signal=52% |
| 253 | ST\_FAS\_SIGNALING\_PATHWAY |  | 61 | 0.53 | 1.49 | 0.015 | 0.083 | 1.000 | 3190 | tags=28%, list=15%, signal=33% |
| 254 | NO1PATHWAY |  | 29 | 0.59 | 1.49 | 0.030 | 0.083 | 1.000 | 4631 | tags=41%, list=22%, signal=53% |
| 255 | TAKEDA\_NUP8\_HOXA9\_3D\_UP |  | 187 | 0.49 | 1.49 | 0.000 | 0.084 | 1.000 | 4209 | tags=34%, list=20%, signal=43% |
| 256 | YANG\_OSTECLASTS\_SIG |  | 38 | 0.56 | 1.49 | 0.022 | 0.084 | 1.000 | 499 | tags=13%, list=2%, signal=13% |
| 257 | ABRAHAM\_AL\_VS\_MM\_UP |  | 23 | 0.61 | 1.48 | 0.035 | 0.084 | 1.000 | 3349 | tags=48%, list=16%, signal=57% |
| 258 | YE\_INTRAMETASTATIC\_HCC\_UP |  | 21 | 0.62 | 1.48 | 0.028 | 0.084 | 1.000 | 1447 | tags=24%, list=7%, signal=26% |
| 259 | PTENPATHWAY |  | 17 | 0.64 | 1.48 | 0.036 | 0.085 | 1.000 | 4837 | tags=65%, list=23%, signal=84% |
| 260 | HUMAN\_TISSUE\_PLACENTA |  | 19 | 0.63 | 1.48 | 0.042 | 0.085 | 1.000 | 2287 | tags=26%, list=11%, signal=30% |
| 261 | ROSS\_CBF\_MYH |  | 54 | 0.54 | 1.48 | 0.017 | 0.084 | 1.000 | 2851 | tags=30%, list=14%, signal=34% |
| 262 | JECHLINGER\_EMT\_DN |  | 40 | 0.56 | 1.48 | 0.025 | 0.084 | 1.000 | 5219 | tags=55%, list=25%, signal=74% |
| 263 | CARIES\_PULP\_UP |  | 205 | 0.49 | 1.48 | 0.000 | 0.085 | 1.000 | 1313 | tags=15%, list=6%, signal=16% |
| 264 | PARP\_KO\_UP |  | 30 | 0.57 | 1.48 | 0.028 | 0.086 | 1.000 | 2545 | tags=23%, list=12%, signal=27% |
| 265 | ESR\_FIBROBLAST\_UP |  | 50 | 0.54 | 1.48 | 0.019 | 0.087 | 1.000 | 3167 | tags=36%, list=15%, signal=42% |
| 266 | LAIRPATHWAY |  | 15 | 0.65 | 1.48 | 0.044 | 0.087 | 1.000 | 3140 | tags=33%, list=15%, signal=39% |
| 267 | AGEING\_KIDNEY\_UP |  | 406 | 0.48 | 1.48 | 0.000 | 0.087 | 1.000 | 4472 | tags=33%, list=22%, signal=41% |
| 268 | ADIPOGENESIS\_HMSC\_CLASS8\_DN |  | 32 | 0.57 | 1.47 | 0.037 | 0.090 | 1.000 | 4826 | tags=44%, list=23%, signal=57% |
| 269 | PROLIFERATION\_GENES |  | 363 | 0.48 | 1.47 | 0.000 | 0.090 | 1.000 | 3659 | tags=26%, list=18%, signal=31% |
| 270 | GN\_CAMP\_GRANULOSA\_DN |  | 61 | 0.53 | 1.47 | 0.007 | 0.090 | 1.000 | 4141 | tags=43%, list=20%, signal=53% |
| 271 | MYOD\_NIH3T3\_DN |  | 56 | 0.53 | 1.47 | 0.015 | 0.090 | 1.000 | 2550 | tags=29%, list=12%, signal=33% |
| 272 | FLECHNER\_KIDNEY\_TRANSPLANT\_REJECTION\_PBL\_DN |  | 51 | 0.54 | 1.47 | 0.022 | 0.090 | 1.000 | 3215 | tags=41%, list=16%, signal=49% |
| 273 | HYPOXIA\_FIBRO\_UP |  | 20 | 0.62 | 1.47 | 0.037 | 0.090 | 1.000 | 3492 | tags=55%, list=17%, signal=66% |
| 274 | BRCA1\_OVEREXP\_PROSTATE\_DN |  | 78 | 0.51 | 1.47 | 0.010 | 0.091 | 1.000 | 2344 | tags=27%, list=11%, signal=30% |
| 275 | BHATTACHARYA\_ESC\_UP |  | 62 | 0.53 | 1.47 | 0.011 | 0.091 | 1.000 | 3502 | tags=35%, list=17%, signal=43% |
| 276 | AGUIRRE\_PANCREAS\_CHR6 |  | 32 | 0.57 | 1.47 | 0.032 | 0.091 | 1.000 | 3656 | tags=44%, list=18%, signal=53% |
| 277 | CELL\_CYCLE\_ARREST |  | 31 | 0.57 | 1.47 | 0.026 | 0.090 | 1.000 | 3195 | tags=39%, list=16%, signal=46% |
| 278 | CMV\_HCMV\_TIMECOURSE\_18HRS\_DN |  | 21 | 0.60 | 1.47 | 0.041 | 0.090 | 1.000 | 1512 | tags=24%, list=7%, signal=26% |
| 279 | ESR\_FIBROBLAST\_DN |  | 18 | 0.62 | 1.47 | 0.047 | 0.092 | 1.000 | 3960 | tags=44%, list=19%, signal=55% |
| 280 | TAKEDA\_NUP8\_HOXA9\_8D\_DN |  | 214 | 0.49 | 1.47 | 0.000 | 0.092 | 1.000 | 5124 | tags=37%, list=25%, signal=49% |
| 281 | PASSERINI\_GROWTH |  | 33 | 0.56 | 1.47 | 0.034 | 0.093 | 1.000 | 5383 | tags=48%, list=26%, signal=66% |
| 282 | SARCOMAS\_LEIOMYOSARCOMA\_CALP\_UP |  | 15 | 0.65 | 1.47 | 0.045 | 0.093 | 1.000 | 474 | tags=13%, list=2%, signal=14% |
| 283 | HSC\_MATURE\_FETAL |  | 325 | 0.48 | 1.47 | 0.000 | 0.093 | 1.000 | 5216 | tags=39%, list=25%, signal=51% |
| 284 | ROSS\_CBF\_LEUKEMIA |  | 65 | 0.52 | 1.47 | 0.011 | 0.092 | 1.000 | 3814 | tags=34%, list=19%, signal=41% |
| 285 | CMV\_24HRS\_DN |  | 71 | 0.52 | 1.46 | 0.014 | 0.094 | 1.000 | 1893 | tags=24%, list=9%, signal=26% |
| 286 | ABBUD\_LIF\_DN |  | 24 | 0.59 | 1.46 | 0.046 | 0.095 | 1.000 | 2714 | tags=29%, list=13%, signal=34% |
| 287 | FALT\_BCLL\_DN |  | 49 | 0.53 | 1.46 | 0.026 | 0.095 | 1.000 | 2884 | tags=35%, list=14%, signal=40% |
| 288 | CHAUHAN\_2ME2 |  | 46 | 0.54 | 1.46 | 0.017 | 0.095 | 1.000 | 1959 | tags=37%, list=10%, signal=41% |
| 289 | CYTOKINEPATHWAY |  | 20 | 0.61 | 1.46 | 0.033 | 0.095 | 1.000 | 529 | tags=10%, list=3%, signal=10% |
| 290 | FCER1PATHWAY |  | 38 | 0.56 | 1.46 | 0.026 | 0.096 | 1.000 | 4361 | tags=47%, list=21%, signal=60% |
| 291 | SA\_PTEN\_PATHWAY |  | 17 | 0.64 | 1.46 | 0.044 | 0.096 | 1.000 | 3407 | tags=41%, list=17%, signal=49% |
| 292 | IL1\_CORNEA\_DN |  | 74 | 0.51 | 1.46 | 0.007 | 0.097 | 1.000 | 4828 | tags=38%, list=23%, signal=49% |
| 293 | HDACI\_COLON\_SUL16HRS\_UP |  | 42 | 0.54 | 1.46 | 0.034 | 0.097 | 1.000 | 4728 | tags=48%, list=23%, signal=62% |
| 294 | NDKDYNAMINPATHWAY |  | 18 | 0.63 | 1.46 | 0.039 | 0.097 | 1.000 | 4184 | tags=61%, list=20%, signal=77% |
| 295 | EGFPATHWAY |  | 27 | 0.57 | 1.46 | 0.040 | 0.098 | 1.000 | 4361 | tags=48%, list=21%, signal=61% |
| 296 | BRCA1\_OVEREXP\_PROSTATE\_UP |  | 159 | 0.48 | 1.46 | 0.005 | 0.099 | 1.000 | 4689 | tags=40%, list=23%, signal=52% |
| 297 | STEMPATHWAY |  | 15 | 0.65 | 1.46 | 0.052 | 0.098 | 1.000 | 13 | tags=7%, list=0%, signal=7% |
| 298 | HDACI\_COLON\_BUT24HRS\_DN |  | 109 | 0.50 | 1.45 | 0.001 | 0.099 | 1.000 | 2261 | tags=28%, list=11%, signal=31% |
| 299 | JAIN\_NEMO\_DIFF |  | 75 | 0.51 | 1.45 | 0.024 | 0.099 | 1.000 | 5061 | tags=47%, list=25%, signal=62% |
| 300 | ST\_GRANULE\_CELL\_SURVIVAL\_PATHWAY |  | 27 | 0.59 | 1.45 | 0.037 | 0.099 | 1.000 | 3178 | tags=33%, list=15%, signal=39% |
| 301 | HDACI\_COLON\_TSA2HRS\_DN |  | 22 | 0.61 | 1.45 | 0.044 | 0.099 | 1.000 | 2735 | tags=41%, list=13%, signal=47% |
| 302 | AGEING\_KIDNEY\_DN |  | 129 | 0.49 | 1.45 | 0.001 | 0.099 | 1.000 | 4249 | tags=34%, list=21%, signal=43% |
| 303 | KNUDSEN\_PMNS\_DN |  | 226 | 0.48 | 1.45 | 0.000 | 0.100 | 1.000 | 4748 | tags=38%, list=23%, signal=49% |
| 304 | HDACI\_COLON\_TSA12HRS\_UP |  | 22 | 0.60 | 1.45 | 0.045 | 0.100 | 1.000 | 4050 | tags=50%, list=20%, signal=62% |
| 305 | BOQUEST\_CD31PLUS\_VS\_CD31MINUS\_DN |  | 265 | 0.48 | 1.45 | 0.000 | 0.100 | 1.000 | 4861 | tags=34%, list=24%, signal=44% |
| 306 | VERHAAK\_AML\_NPM1\_MUT\_VS\_WT\_DN |  | 258 | 0.48 | 1.45 | 0.000 | 0.101 | 1.000 | 4209 | tags=31%, list=20%, signal=38% |
| 307 | UVC\_LOW\_C2\_DN |  | 20 | 0.60 | 1.45 | 0.050 | 0.101 | 1.000 | 6515 | tags=65%, list=32%, signal=95% |
| 308 | CMV\_HCMV\_TIMECOURSE\_20HRS\_DN |  | 40 | 0.55 | 1.45 | 0.028 | 0.102 | 1.000 | 5257 | tags=55%, list=26%, signal=74% |
| 309 | METHOTREXATE\_PROBCELL\_UP |  | 18 | 0.62 | 1.45 | 0.038 | 0.103 | 1.000 | 4158 | tags=33%, list=20%, signal=42% |
| 310 | NOUZOVA\_CPG\_H4\_UP |  | 120 | 0.49 | 1.45 | 0.003 | 0.103 | 1.000 | 3920 | tags=38%, list=19%, signal=47% |
| 311 | LIZUKA\_G2\_GR\_G3 |  | 27 | 0.58 | 1.45 | 0.037 | 0.103 | 1.000 | 6339 | tags=63%, list=31%, signal=91% |
| 312 | 4NQO\_UNIQUE\_FIBRO\_UP |  | 22 | 0.60 | 1.44 | 0.053 | 0.104 | 1.000 | 3783 | tags=50%, list=18%, signal=61% |
| 313 | BRENTANI\_DEATH |  | 70 | 0.51 | 1.44 | 0.015 | 0.105 | 1.000 | 6930 | tags=50%, list=34%, signal=75% |
| 314 | HASLINGER\_B\_CLL\_12 |  | 18 | 0.61 | 1.44 | 0.054 | 0.106 | 1.000 | 1819 | tags=33%, list=9%, signal=37% |
| 315 | STEMCELL\_COMMON\_DN |  | 62 | 0.52 | 1.44 | 0.027 | 0.107 | 1.000 | 3075 | tags=27%, list=15%, signal=32% |
| 316 | ZHAN\_PCS\_MULTIPLE\_MYELOMA\_SPKD |  | 24 | 0.59 | 1.44 | 0.046 | 0.107 | 1.000 | 710 | tags=21%, list=3%, signal=22% |
| 317 | TGFBETA\_C5\_UP |  | 17 | 0.62 | 1.44 | 0.052 | 0.108 | 1.000 | 1187 | tags=24%, list=6%, signal=25% |
| 318 | HSC\_LTHSC\_ADULT |  | 360 | 0.47 | 1.44 | 0.000 | 0.108 | 1.000 | 4158 | tags=28%, list=20%, signal=35% |
| 319 | HSC\_LTHSC\_FETAL |  | 268 | 0.47 | 1.44 | 0.000 | 0.109 | 1.000 | 4229 | tags=30%, list=21%, signal=38% |
| 320 | IDX\_TSA\_DN\_CLUSTER4 |  | 27 | 0.57 | 1.44 | 0.048 | 0.109 | 1.000 | 2877 | tags=41%, list=14%, signal=47% |
| 321 | GAMMA-UV\_FIBRO\_DN |  | 41 | 0.54 | 1.44 | 0.032 | 0.110 | 1.000 | 5477 | tags=49%, list=27%, signal=66% |
| 322 | ZHAN\_MM\_CD1\_VS\_CD2\_DN |  | 54 | 0.52 | 1.44 | 0.028 | 0.110 | 1.000 | 3794 | tags=31%, list=18%, signal=38% |
| 323 | LEE\_ACOX1\_UP |  | 64 | 0.51 | 1.44 | 0.024 | 0.111 | 1.000 | 2290 | tags=33%, list=11%, signal=37% |
| 324 | HSC\_LTHSC\_SHARED |  | 268 | 0.47 | 1.43 | 0.000 | 0.111 | 1.000 | 4229 | tags=30%, list=21%, signal=38% |
| 325 | SIG\_PIP3\_SIGNALING\_IN\_B\_LYMPHOCYTES |  | 35 | 0.55 | 1.43 | 0.048 | 0.112 | 1.000 | 4776 | tags=37%, list=23%, signal=48% |
| 326 | AD12\_ANY\_DN |  | 25 | 0.58 | 1.43 | 0.045 | 0.112 | 1.000 | 1036 | tags=24%, list=5%, signal=25% |
| 327 | OXSTRESS\_BREASTCA\_UP |  | 29 | 0.57 | 1.43 | 0.040 | 0.115 | 1.000 | 1773 | tags=34%, list=9%, signal=38% |
| 328 | STRESS\_ARSENIC\_SPECIFIC\_UP |  | 149 | 0.48 | 1.43 | 0.006 | 0.115 | 1.000 | 4445 | tags=35%, list=22%, signal=44% |
| 329 | AGED\_MOUSE\_HIPPOCAMPUS\_MULTI\_UP |  | 19 | 0.60 | 1.43 | 0.064 | 0.115 | 1.000 | 3381 | tags=42%, list=16%, signal=50% |
| 330 | PASSERINI\_APOPTOSIS |  | 43 | 0.53 | 1.43 | 0.035 | 0.114 | 1.000 | 4494 | tags=40%, list=22%, signal=50% |
| 331 | CARIES\_PULP\_HIGH\_UP |  | 91 | 0.49 | 1.43 | 0.009 | 0.115 | 1.000 | 1153 | tags=13%, list=6%, signal=14% |
| 332 | ELONGINA\_KO\_UP |  | 162 | 0.47 | 1.43 | 0.002 | 0.117 | 1.000 | 3828 | tags=33%, list=19%, signal=40% |
| 333 | JISON\_SICKLECELL\_DIFF |  | 368 | 0.47 | 1.43 | 0.000 | 0.118 | 1.000 | 3506 | tags=30%, list=17%, signal=36% |
| 334 | CHESLER\_HIGHEST\_FOLD\_RANGE\_GENES |  | 37 | 0.53 | 1.43 | 0.043 | 0.117 | 1.000 | 2703 | tags=35%, list=13%, signal=40% |
| 335 | FLECHNER\_KIDNEY\_TRANSPLANT\_WELL\_PBL\_DN |  | 42 | 0.54 | 1.43 | 0.028 | 0.117 | 1.000 | 4337 | tags=45%, list=21%, signal=57% |
| 336 | BRCA\_PROGNOSIS\_NEG |  | 95 | 0.49 | 1.42 | 0.010 | 0.119 | 1.000 | 4127 | tags=40%, list=20%, signal=50% |
| 337 | ROSS\_CBF |  | 82 | 0.49 | 1.42 | 0.011 | 0.119 | 1.000 | 3814 | tags=28%, list=19%, signal=34% |
| 338 | GOLDRATH\_HP |  | 147 | 0.48 | 1.42 | 0.002 | 0.121 | 1.000 | 4679 | tags=47%, list=23%, signal=60% |
| 339 | FLECHNER\_KIDNEY\_TRANSPLANT\_WELL\_PBL\_UP |  | 153 | 0.48 | 1.42 | 0.008 | 0.121 | 1.000 | 3661 | tags=34%, list=18%, signal=41% |
| 340 | CITED1\_KO\_HET\_DN |  | 30 | 0.56 | 1.42 | 0.045 | 0.121 | 1.000 | 2193 | tags=33%, list=11%, signal=37% |
| 341 | CANTHARIDIN\_DN |  | 50 | 0.52 | 1.42 | 0.034 | 0.121 | 1.000 | 3020 | tags=30%, list=15%, signal=35% |
| 342 | UVB\_NHEK4\_6HRS\_UP |  | 28 | 0.56 | 1.42 | 0.064 | 0.120 | 1.000 | 3338 | tags=46%, list=16%, signal=55% |
| 343 | DORSAM\_HOXA9\_DN |  | 30 | 0.55 | 1.42 | 0.056 | 0.121 | 1.000 | 3739 | tags=40%, list=18%, signal=49% |
| 344 | LEE\_ACOX1\_DN |  | 63 | 0.50 | 1.42 | 0.024 | 0.121 | 1.000 | 3333 | tags=24%, list=16%, signal=28% |
| 345 | IRS\_KO\_ADIP\_UP |  | 28 | 0.56 | 1.42 | 0.055 | 0.121 | 1.000 | 2272 | tags=25%, list=11%, signal=28% |
| 346 | LVAD\_HEARTFAILURE\_UP |  | 89 | 0.49 | 1.42 | 0.016 | 0.124 | 1.000 | 4684 | tags=38%, list=23%, signal=49% |
| 347 | VIPPATHWAY |  | 27 | 0.56 | 1.41 | 0.044 | 0.126 | 1.000 | 4361 | tags=41%, list=21%, signal=52% |
| 348 | XU\_ATRA\_PLUSNSC\_UP |  | 15 | 0.63 | 1.41 | 0.068 | 0.126 | 1.000 | 1020 | tags=27%, list=5%, signal=28% |
| 349 | UVB\_NHEK3\_C7 |  | 53 | 0.51 | 1.41 | 0.028 | 0.127 | 1.000 | 3045 | tags=40%, list=15%, signal=46% |
| 350 | SHIPP\_FL\_VS\_DLBCL\_UP |  | 34 | 0.54 | 1.41 | 0.040 | 0.127 | 1.000 | 1753 | tags=26%, list=9%, signal=29% |
| 351 | HYPERME\_COLONCA\_SW48 |  | 18 | 0.61 | 1.41 | 0.057 | 0.127 | 1.000 | 2873 | tags=22%, list=14%, signal=26% |
| 352 | IFN\_BETA\_GLIOMA\_UP |  | 63 | 0.50 | 1.41 | 0.033 | 0.128 | 1.000 | 4346 | tags=29%, list=21%, signal=36% |
| 353 | BLEO\_MOUSE\_LYMPH\_LOW\_24HRS\_DN |  | 27 | 0.57 | 1.41 | 0.053 | 0.128 | 1.000 | 3466 | tags=41%, list=17%, signal=49% |
| 354 | CHIARETTI\_ZAP70\_DIFF |  | 67 | 0.50 | 1.41 | 0.026 | 0.127 | 1.000 | 5302 | tags=42%, list=26%, signal=56% |
| 355 | HDACI\_COLON\_SUL24HRS\_DN |  | 127 | 0.48 | 1.41 | 0.005 | 0.127 | 1.000 | 2614 | tags=27%, list=13%, signal=30% |
| 356 | ET743\_SARCOMA\_48HRS\_DN |  | 188 | 0.47 | 1.41 | 0.002 | 0.127 | 1.000 | 3747 | tags=37%, list=18%, signal=45% |
| 357 | TGFBETA\_C1\_UP |  | 17 | 0.60 | 1.41 | 0.065 | 0.127 | 1.000 | 2048 | tags=41%, list=10%, signal=46% |
| 358 | CMV\_ALL\_DN |  | 102 | 0.48 | 1.41 | 0.012 | 0.128 | 1.000 | 5254 | tags=46%, list=25%, signal=62% |
| 359 | SANSOM\_APC\_LOSS5\_UP |  | 79 | 0.49 | 1.41 | 0.024 | 0.129 | 1.000 | 4045 | tags=33%, list=20%, signal=41% |
| 360 | TNFALPHA\_4HRS\_UP |  | 40 | 0.53 | 1.41 | 0.043 | 0.129 | 1.000 | 3038 | tags=30%, list=15%, signal=35% |
| 361 | HDACI\_COLON\_SUL48HRS\_UP |  | 90 | 0.49 | 1.41 | 0.014 | 0.131 | 1.000 | 4410 | tags=46%, list=21%, signal=58% |
| 362 | AGED\_MOUSE\_CORTEX\_DN |  | 46 | 0.52 | 1.41 | 0.049 | 0.131 | 1.000 | 2693 | tags=37%, list=13%, signal=42% |
| 363 | GH\_EXOGENOUS\_EARLY\_UP |  | 29 | 0.55 | 1.40 | 0.060 | 0.133 | 1.000 | 1323 | tags=21%, list=6%, signal=22% |
| 364 | UVB\_NHEK3\_ALL |  | 393 | 0.46 | 1.40 | 0.000 | 0.133 | 1.000 | 3954 | tags=37%, list=19%, signal=45% |
| 365 | BRENTANI\_TRANSPORT\_OF\_VESICLES |  | 24 | 0.57 | 1.40 | 0.070 | 0.134 | 1.000 | 4023 | tags=42%, list=20%, signal=52% |
| 366 | CMV\_HCMV\_TIMECOURSE\_48HRS\_DN |  | 110 | 0.48 | 1.40 | 0.011 | 0.134 | 1.000 | 5113 | tags=38%, list=25%, signal=51% |
| 367 | SIG\_PIP3\_SIGNALING\_IN\_CARDIAC\_MYOCTES |  | 66 | 0.50 | 1.40 | 0.028 | 0.133 | 1.000 | 3936 | tags=33%, list=19%, signal=41% |
| 368 | LINDSTEDT\_DEND\_DN |  | 65 | 0.50 | 1.40 | 0.029 | 0.133 | 1.000 | 3777 | tags=31%, list=18%, signal=38% |
| 369 | HDACI\_COLON\_TSA2HRS\_UP |  | 60 | 0.50 | 1.40 | 0.025 | 0.134 | 1.000 | 2164 | tags=27%, list=11%, signal=30% |
| 370 | SPRYPATHWAY |  | 18 | 0.60 | 1.40 | 0.071 | 0.134 | 1.000 | 3407 | tags=39%, list=17%, signal=47% |
| 371 | POMEROY\_DESMOPLASIC\_VS\_CLASSIC\_MD\_UP |  | 46 | 0.52 | 1.40 | 0.029 | 0.136 | 1.000 | 3338 | tags=30%, list=16%, signal=36% |
| 372 | LU\_IL4BCELL |  | 66 | 0.50 | 1.40 | 0.026 | 0.135 | 1.000 | 3380 | tags=32%, list=16%, signal=38% |
| 373 | HIF1\_TARGETS |  | 36 | 0.53 | 1.40 | 0.055 | 0.136 | 1.000 | 2845 | tags=39%, list=14%, signal=45% |
| 374 | MRNA\_PROCESSING |  | 42 | 0.52 | 1.40 | 0.059 | 0.135 | 1.000 | 2381 | tags=31%, list=12%, signal=35% |
| 375 | AGED\_RHESUS\_UP |  | 198 | 0.46 | 1.40 | 0.002 | 0.137 | 1.000 | 3075 | tags=24%, list=15%, signal=28% |
| 376 | METPATHWAY |  | 34 | 0.54 | 1.40 | 0.060 | 0.137 | 1.000 | 3407 | tags=38%, list=17%, signal=46% |
| 377 | UEDA\_MOUSE\_SCN |  | 82 | 0.48 | 1.40 | 0.032 | 0.137 | 1.000 | 3470 | tags=32%, list=17%, signal=38% |
| 378 | UVC\_HIGH\_D3\_DN |  | 46 | 0.52 | 1.40 | 0.034 | 0.138 | 1.000 | 3747 | tags=39%, list=18%, signal=48% |
| 379 | ECMPATHWAY |  | 21 | 0.58 | 1.39 | 0.083 | 0.138 | 1.000 | 3367 | tags=33%, list=16%, signal=40% |
| 380 | CREBPATHWAY |  | 27 | 0.56 | 1.39 | 0.071 | 0.138 | 1.000 | 3407 | tags=41%, list=17%, signal=49% |
| 381 | UVC\_LOW\_C3\_DN |  | 19 | 0.60 | 1.39 | 0.077 | 0.139 | 1.000 | 3448 | tags=47%, list=17%, signal=57% |
| 382 | SA\_B\_CELL\_RECEPTOR\_COMPLEXES |  | 24 | 0.56 | 1.39 | 0.077 | 0.140 | 1.000 | 3407 | tags=46%, list=17%, signal=55% |
| 383 | GAMMA-UV\_FIBRO\_UP |  | 35 | 0.54 | 1.39 | 0.044 | 0.140 | 1.000 | 1537 | tags=29%, list=7%, signal=31% |
| 384 | STAEGE\_EFTS\_UP |  | 29 | 0.54 | 1.39 | 0.072 | 0.142 | 1.000 | 2005 | tags=24%, list=10%, signal=27% |
| 385 | NGUYEN\_KERATO\_UP |  | 28 | 0.55 | 1.39 | 0.070 | 0.144 | 1.000 | 1908 | tags=25%, list=9%, signal=28% |
| 386 | ST\_INTEGRIN\_SIGNALING\_PATHWAY |  | 78 | 0.49 | 1.39 | 0.031 | 0.144 | 1.000 | 4910 | tags=38%, list=24%, signal=50% |
| 387 | LE\_MYELIN\_UP |  | 89 | 0.48 | 1.39 | 0.023 | 0.144 | 1.000 | 3621 | tags=35%, list=18%, signal=42% |
| 388 | PRMT5\_KD\_UP |  | 183 | 0.46 | 1.39 | 0.005 | 0.144 | 1.000 | 4043 | tags=36%, list=20%, signal=44% |
| 389 | SIG\_IL4RECEPTOR\_IN\_B\_LYPHOCYTES |  | 27 | 0.55 | 1.39 | 0.075 | 0.145 | 1.000 | 4297 | tags=41%, list=21%, signal=51% |
| 390 | STEFFEN\_AML\_PML\_PLZF\_TRGT |  | 43 | 0.51 | 1.39 | 0.061 | 0.145 | 1.000 | 3287 | tags=28%, list=16%, signal=33% |
| 391 | GOLDRATH\_MEMORY |  | 53 | 0.51 | 1.39 | 0.043 | 0.145 | 1.000 | 3346 | tags=30%, list=16%, signal=36% |
| 392 | P21\_MIDDLE\_DN |  | 15 | 0.61 | 1.39 | 0.104 | 0.145 | 1.000 | 4695 | tags=53%, list=23%, signal=69% |
| 393 | VEGFPATHWAY |  | 27 | 0.55 | 1.38 | 0.067 | 0.145 | 1.000 | 5005 | tags=48%, list=24%, signal=64% |
| 394 | ADDYA\_K562\_HEMIN\_TREATMENT |  | 70 | 0.49 | 1.38 | 0.043 | 0.145 | 1.000 | 2567 | tags=24%, list=12%, signal=28% |
| 395 | INSULIN\_ADIP\_INSENS\_DN |  | 17 | 0.61 | 1.38 | 0.070 | 0.145 | 1.000 | 5793 | tags=53%, list=28%, signal=74% |
| 396 | SERUM\_FIBROBLAST\_CORE\_DN |  | 196 | 0.46 | 1.38 | 0.003 | 0.145 | 1.000 | 3682 | tags=32%, list=18%, signal=38% |
| 397 | UVB\_NHEK3\_C1 |  | 57 | 0.49 | 1.38 | 0.056 | 0.146 | 1.000 | 2659 | tags=33%, list=13%, signal=38% |
| 398 | RUTELLA\_HEPATGFSNDCS\_UP |  | 158 | 0.46 | 1.38 | 0.004 | 0.147 | 1.000 | 3100 | tags=25%, list=15%, signal=30% |
| 399 | PDGFPATHWAY |  | 27 | 0.55 | 1.38 | 0.066 | 0.148 | 1.000 | 4361 | tags=48%, list=21%, signal=61% |
| 400 | KANG\_TERT\_DN |  | 96 | 0.47 | 1.38 | 0.028 | 0.148 | 1.000 | 4446 | tags=28%, list=22%, signal=36% |
| 401 | UVC\_HIGH\_D1\_DN |  | 15 | 0.60 | 1.38 | 0.097 | 0.148 | 1.000 | 4661 | tags=53%, list=23%, signal=69% |
| 402 | CELL\_ADHESION\_MOLECULE\_ACTIVITY |  | 107 | 0.48 | 1.38 | 0.014 | 0.149 | 1.000 | 3464 | tags=26%, list=17%, signal=31% |
| 403 | STRIATED\_MUSCLE\_CONTRACTION |  | 37 | 0.52 | 1.38 | 0.067 | 0.149 | 1.000 | 4164 | tags=46%, list=20%, signal=57% |
| 404 | OLD\_FIBRO\_UP |  | 61 | 0.49 | 1.38 | 0.035 | 0.149 | 1.000 | 2828 | tags=30%, list=14%, signal=34% |
| 405 | LE\_MYELIN\_DN |  | 93 | 0.48 | 1.38 | 0.022 | 0.149 | 1.000 | 2848 | tags=28%, list=14%, signal=32% |
| 406 | PARK\_MSCS\_DIFF |  | 31 | 0.54 | 1.38 | 0.074 | 0.150 | 1.000 | 2991 | tags=35%, list=15%, signal=41% |
| 407 | WERNERONLY\_FIBRO\_UP |  | 31 | 0.53 | 1.38 | 0.064 | 0.151 | 1.000 | 4008 | tags=39%, list=19%, signal=48% |
| 408 | HASLINGER\_B\_CLL\_11Q23 |  | 20 | 0.58 | 1.38 | 0.085 | 0.151 | 1.000 | 5863 | tags=60%, list=28%, signal=84% |
| 409 | APOPTOSIS |  | 67 | 0.49 | 1.38 | 0.036 | 0.151 | 1.000 | 2060 | tags=21%, list=10%, signal=23% |
| 410 | DFOSB\_BRAIN\_2WKS\_UP |  | 38 | 0.52 | 1.38 | 0.069 | 0.151 | 1.000 | 4850 | tags=45%, list=24%, signal=58% |
| 411 | IGF1MTORPATHWAY |  | 20 | 0.58 | 1.38 | 0.081 | 0.151 | 1.000 | 4531 | tags=55%, list=22%, signal=70% |
| 412 | FALT\_BCLL\_IG\_MUTATED\_VS\_WT\_UP |  | 48 | 0.50 | 1.38 | 0.058 | 0.151 | 1.000 | 6050 | tags=58%, list=29%, signal=82% |
| 413 | UVC\_HIGH\_ALL\_UP |  | 19 | 0.59 | 1.37 | 0.092 | 0.151 | 1.000 | 2855 | tags=32%, list=14%, signal=37% |
| 414 | ZHAN\_MMPC\_SIMAL |  | 47 | 0.50 | 1.37 | 0.042 | 0.151 | 1.000 | 4580 | tags=38%, list=22%, signal=49% |
| 415 | LEE\_TCELLS9\_UP |  | 31 | 0.54 | 1.37 | 0.064 | 0.151 | 1.000 | 6431 | tags=55%, list=31%, signal=80% |
| 416 | PASSERINI\_INFLAMMATION |  | 25 | 0.55 | 1.37 | 0.079 | 0.151 | 1.000 | 1153 | tags=12%, list=6%, signal=13% |
| 417 | SIG\_REGULATION\_OF\_THE\_ACTIN\_CYTOSKELETON\_BY\_RHO\_GTPASES |  | 35 | 0.53 | 1.37 | 0.062 | 0.151 | 1.000 | 4689 | tags=40%, list=23%, signal=52% |
| 418 | ELONGINA\_KO\_DN |  | 177 | 0.46 | 1.37 | 0.007 | 0.151 | 1.000 | 3304 | tags=30%, list=16%, signal=35% |
| 419 | CREB\_BRAIN\_8WKS\_DN |  | 49 | 0.50 | 1.37 | 0.053 | 0.151 | 1.000 | 3444 | tags=29%, list=17%, signal=34% |
| 420 | GN\_CAMP\_GRANULOSA\_UP |  | 53 | 0.50 | 1.37 | 0.048 | 0.151 | 1.000 | 3385 | tags=34%, list=16%, signal=41% |
| 421 | HDACI\_COLON\_CUR\_DN |  | 49 | 0.50 | 1.37 | 0.047 | 0.151 | 1.000 | 2960 | tags=31%, list=14%, signal=36% |
| 422 | INTRINSICPATHWAY |  | 22 | 0.56 | 1.37 | 0.079 | 0.153 | 1.000 | 1981 | tags=27%, list=10%, signal=30% |
| 423 | MITOCHONDRIAPATHWAY |  | 20 | 0.57 | 1.37 | 0.093 | 0.153 | 1.000 | 1599 | tags=25%, list=8%, signal=27% |
| 424 | LEE\_E2F1\_UP |  | 60 | 0.49 | 1.37 | 0.059 | 0.153 | 1.000 | 4112 | tags=37%, list=20%, signal=46% |
| 425 | HOGERKORP\_ANTI\_CD44\_UP |  | 23 | 0.56 | 1.37 | 0.072 | 0.153 | 1.000 | 6724 | tags=65%, list=33%, signal=97% |
| 426 | G13\_SIGNALING\_PATHWAY |  | 39 | 0.52 | 1.37 | 0.067 | 0.154 | 1.000 | 4996 | tags=51%, list=24%, signal=68% |
| 427 | HDACI\_COLON\_TSABUT\_DN |  | 22 | 0.56 | 1.37 | 0.088 | 0.154 | 1.000 | 2109 | tags=23%, list=10%, signal=25% |
| 428 | PARK\_MSCS\_BOTH |  | 44 | 0.51 | 1.37 | 0.054 | 0.154 | 1.000 | 5346 | tags=41%, list=26%, signal=55% |
| 429 | KANG\_TERT\_UP |  | 79 | 0.47 | 1.37 | 0.026 | 0.155 | 1.000 | 6073 | tags=43%, list=29%, signal=61% |
| 430 | HDACI\_COLON\_TSA\_UP |  | 120 | 0.46 | 1.37 | 0.028 | 0.155 | 1.000 | 2655 | tags=26%, list=13%, signal=29% |
| 431 | HEMATOP\_STEM\_ALL\_UP |  | 36 | 0.52 | 1.37 | 0.081 | 0.155 | 1.000 | 1568 | tags=22%, list=8%, signal=24% |
| 432 | ZHANG\_EFT\_EWSFLI1\_UP |  | 88 | 0.48 | 1.37 | 0.024 | 0.155 | 1.000 | 5881 | tags=41%, list=29%, signal=57% |
| 433 | HDACI\_COLON\_CUR2HRS\_UP |  | 29 | 0.54 | 1.37 | 0.089 | 0.156 | 1.000 | 2707 | tags=31%, list=13%, signal=36% |
| 434 | TCELL\_ANERGIC\_UP |  | 81 | 0.48 | 1.37 | 0.023 | 0.156 | 1.000 | 6560 | tags=46%, list=32%, signal=67% |
| 435 | HDACI\_COLON\_CLUSTER4 |  | 16 | 0.59 | 1.36 | 0.087 | 0.157 | 1.000 | 1851 | tags=31%, list=9%, signal=34% |
| 436 | BCRABL\_HL60\_CDNA\_DN |  | 28 | 0.53 | 1.36 | 0.082 | 0.157 | 1.000 | 3349 | tags=32%, list=16%, signal=38% |
| 437 | TSADAC\_PANC50\_UP |  | 42 | 0.51 | 1.36 | 0.068 | 0.159 | 1.000 | 2392 | tags=21%, list=12%, signal=24% |
| 438 | PHOSPHATIDYLINOSITOL\_SIGNALING\_SYSTEM |  | 90 | 0.47 | 1.36 | 0.032 | 0.159 | 1.000 | 4923 | tags=36%, list=24%, signal=47% |
| 439 | APOPTOSIS\_GENMAPP |  | 43 | 0.50 | 1.36 | 0.078 | 0.159 | 1.000 | 1599 | tags=19%, list=8%, signal=20% |
| 440 | KANNAN\_P53\_DN |  | 15 | 0.61 | 1.36 | 0.094 | 0.158 | 1.000 | 191 | tags=13%, list=1%, signal=13% |
| 441 | GENOTOXINS\_4HRS\_DISCR |  | 34 | 0.52 | 1.36 | 0.079 | 0.158 | 1.000 | 4377 | tags=38%, list=21%, signal=48% |
| 442 | TOB1PATHWAY |  | 17 | 0.58 | 1.36 | 0.081 | 0.159 | 1.000 | 1542 | tags=24%, list=7%, signal=25% |
| 443 | MAPKPATHWAY |  | 84 | 0.47 | 1.36 | 0.039 | 0.159 | 1.000 | 3445 | tags=35%, list=17%, signal=41% |
| 444 | SCHUMACHER\_MYC\_UP |  | 51 | 0.49 | 1.36 | 0.068 | 0.160 | 1.000 | 4134 | tags=39%, list=20%, signal=49% |
| 445 | 5FU\_RESIST\_GASTRIC\_DN |  | 16 | 0.59 | 1.36 | 0.094 | 0.160 | 1.000 | 756 | tags=19%, list=4%, signal=19% |
| 446 | VEGF\_MMMEC\_3HRS\_UP |  | 66 | 0.49 | 1.36 | 0.046 | 0.160 | 1.000 | 3161 | tags=26%, list=15%, signal=30% |
| 447 | HOFFMANN\_BIVSBII\_BI |  | 97 | 0.47 | 1.36 | 0.028 | 0.161 | 1.000 | 2677 | tags=22%, list=13%, signal=25% |
| 448 | MITOCHONDRIAL\_FATTY\_ACID\_BETAOXIDATION |  | 16 | 0.59 | 1.36 | 0.108 | 0.161 | 1.000 | 5038 | tags=63%, list=24%, signal=83% |
| 449 | OLDWERNER\_FIBRO\_DN |  | 105 | 0.47 | 1.36 | 0.028 | 0.163 | 1.000 | 3432 | tags=30%, list=17%, signal=36% |
| 450 | LIZUKA\_L0\_SM\_L1 |  | 19 | 0.58 | 1.36 | 0.103 | 0.163 | 1.000 | 5067 | tags=47%, list=25%, signal=63% |
| 451 | BRENTANI\_SIGNALING |  | 178 | 0.45 | 1.36 | 0.007 | 0.162 | 1.000 | 6281 | tags=43%, list=30%, signal=61% |
| 452 | ZHAN\_TONSIL\_BONEMARROW |  | 41 | 0.51 | 1.36 | 0.069 | 0.162 | 1.000 | 2016 | tags=22%, list=10%, signal=24% |
| 453 | ATRBRCAPATHWAY |  | 21 | 0.56 | 1.36 | 0.098 | 0.162 | 1.000 | 6048 | tags=57%, list=29%, signal=81% |
| 454 | AGUIRRE\_PANCREAS\_CHR18 |  | 17 | 0.59 | 1.36 | 0.109 | 0.162 | 1.000 | 3598 | tags=41%, list=17%, signal=50% |
| 455 | AGEING\_BRAIN\_DN |  | 120 | 0.46 | 1.35 | 0.016 | 0.163 | 1.000 | 4318 | tags=36%, list=21%, signal=45% |
| 456 | JNK\_UP |  | 30 | 0.52 | 1.35 | 0.106 | 0.163 | 1.000 | 3559 | tags=37%, list=17%, signal=44% |
| 457 | PITUITARY\_FETAL\_UP |  | 17 | 0.58 | 1.35 | 0.098 | 0.163 | 1.000 | 3934 | tags=59%, list=19%, signal=73% |
| 458 | ACTINYPATHWAY |  | 17 | 0.59 | 1.35 | 0.117 | 0.163 | 1.000 | 4651 | tags=47%, list=23%, signal=61% |
| 459 | ST\_PHOSPHOINOSITIDE\_3\_KINASE\_PATHWAY |  | 35 | 0.52 | 1.35 | 0.085 | 0.163 | 1.000 | 4297 | tags=40%, list=21%, signal=50% |
| 460 | HOHENKIRK\_MONOCYTE\_DEND\_DN |  | 119 | 0.46 | 1.35 | 0.010 | 0.164 | 1.000 | 2440 | tags=22%, list=12%, signal=25% |
| 461 | ET743\_SARCOMA\_UP |  | 69 | 0.48 | 1.35 | 0.052 | 0.165 | 1.000 | 2916 | tags=28%, list=14%, signal=32% |
| 462 | UVB\_NHEK1\_UP |  | 173 | 0.45 | 1.35 | 0.013 | 0.165 | 1.000 | 3598 | tags=30%, list=17%, signal=36% |
| 463 | FASPATHWAY |  | 27 | 0.54 | 1.35 | 0.080 | 0.169 | 1.000 | 2504 | tags=37%, list=12%, signal=42% |
| 464 | CELLCYCLEPATHWAY |  | 23 | 0.55 | 1.35 | 0.085 | 0.168 | 1.000 | 3425 | tags=35%, list=17%, signal=42% |
| 465 | HADDAD\_HSC\_CD7\_DN |  | 84 | 0.47 | 1.35 | 0.046 | 0.170 | 1.000 | 2894 | tags=31%, list=14%, signal=36% |
| 466 | CMV\_8HRS\_DN |  | 44 | 0.50 | 1.35 | 0.074 | 0.170 | 1.000 | 5254 | tags=57%, list=25%, signal=76% |
| 467 | HDACI\_COLON\_SUL\_UP |  | 131 | 0.46 | 1.34 | 0.026 | 0.171 | 1.000 | 4410 | tags=42%, list=21%, signal=53% |
| 468 | DAC\_PANC\_UP |  | 365 | 0.44 | 1.34 | 0.000 | 0.172 | 1.000 | 4066 | tags=25%, list=20%, signal=30% |
| 469 | FSH\_GRANULOSA\_UP |  | 78 | 0.47 | 1.34 | 0.046 | 0.172 | 1.000 | 3385 | tags=36%, list=16%, signal=43% |
| 470 | SIG\_INSULIN\_RECEPTOR\_PATHWAY\_IN\_CARDIAC\_MYOCYTES |  | 51 | 0.48 | 1.34 | 0.070 | 0.175 | 1.000 | 3407 | tags=33%, list=17%, signal=40% |
| 471 | HADDAD\_CD45CD7\_PLUS\_VS\_MINUS\_DN |  | 84 | 0.47 | 1.34 | 0.040 | 0.175 | 1.000 | 2894 | tags=31%, list=14%, signal=36% |
| 472 | ROSS\_FAB\_M7 |  | 69 | 0.47 | 1.34 | 0.041 | 0.175 | 1.000 | 4053 | tags=28%, list=20%, signal=34% |
| 473 | ALCALAY\_AML\_NPMC\_DN |  | 187 | 0.44 | 1.34 | 0.007 | 0.175 | 1.000 | 4243 | tags=29%, list=21%, signal=37% |
| 474 | IL6\_FIBRO\_UP |  | 47 | 0.49 | 1.34 | 0.078 | 0.178 | 1.000 | 1264 | tags=17%, list=6%, signal=18% |
| 475 | N\_GLYCAN\_BIOSYNTHESIS |  | 22 | 0.55 | 1.34 | 0.101 | 0.178 | 1.000 | 2579 | tags=32%, list=13%, signal=36% |
| 476 | SHEPARD\_CRASH\_AND\_BURN\_MUT\_VS\_WT\_UP |  | 153 | 0.45 | 1.34 | 0.017 | 0.178 | 1.000 | 4366 | tags=34%, list=21%, signal=43% |
| 477 | STRESS\_GENOTOXIC\_SPECIFIC\_DN |  | 40 | 0.50 | 1.34 | 0.087 | 0.177 | 1.000 | 2575 | tags=25%, list=12%, signal=29% |
| 478 | YAO\_P4\_KO\_VS\_WT\_DN |  | 16 | 0.59 | 1.33 | 0.113 | 0.181 | 1.000 | 2007 | tags=25%, list=10%, signal=28% |
| 479 | BCRPATHWAY |  | 35 | 0.51 | 1.33 | 0.091 | 0.182 | 1.000 | 4361 | tags=43%, list=21%, signal=54% |
| 480 | GUO\_HEX\_DN |  | 61 | 0.48 | 1.33 | 0.061 | 0.182 | 1.000 | 2559 | tags=21%, list=12%, signal=24% |
| 481 | HDACI\_COLON\_SUL16HRS\_DN |  | 72 | 0.47 | 1.33 | 0.053 | 0.182 | 1.000 | 6134 | tags=53%, list=30%, signal=75% |
| 482 | RAC1PATHWAY |  | 22 | 0.55 | 1.33 | 0.109 | 0.183 | 1.000 | 3859 | tags=50%, list=19%, signal=61% |
| 483 | IL1RPATHWAY |  | 31 | 0.52 | 1.33 | 0.099 | 0.183 | 1.000 | 2143 | tags=23%, list=10%, signal=25% |
| 484 | LOTEM\_LEUKEMIA\_UP |  | 22 | 0.55 | 1.33 | 0.105 | 0.183 | 1.000 | 4866 | tags=50%, list=24%, signal=65% |
| 485 | KUMAR\_HOXA\_DIFF |  | 343 | 0.44 | 1.33 | 0.001 | 0.183 | 1.000 | 3398 | tags=22%, list=16%, signal=26% |
| 486 | BRCA2\_BRCA1\_DN |  | 42 | 0.49 | 1.33 | 0.080 | 0.183 | 1.000 | 3870 | tags=31%, list=19%, signal=38% |
| 487 | GILDEA\_BLADDER\_UP |  | 30 | 0.52 | 1.33 | 0.088 | 0.183 | 1.000 | 2817 | tags=33%, list=14%, signal=39% |
| 488 | COCAINE\_BRAIN\_5D\_UP |  | 63 | 0.48 | 1.33 | 0.063 | 0.183 | 1.000 | 4238 | tags=32%, list=21%, signal=40% |
| 489 | ADIP\_DIFF\_CLUSTER1 |  | 56 | 0.48 | 1.33 | 0.072 | 0.183 | 1.000 | 5370 | tags=41%, list=26%, signal=55% |
| 490 | LH\_GRANULOSA\_UP |  | 80 | 0.46 | 1.33 | 0.055 | 0.188 | 1.000 | 3385 | tags=35%, list=16%, signal=42% |
| 491 | YAGI\_AML\_PROG\_FAB |  | 190 | 0.44 | 1.33 | 0.016 | 0.187 | 1.000 | 4079 | tags=30%, list=20%, signal=37% |
| 492 | BRG1\_SW13\_UP |  | 52 | 0.48 | 1.33 | 0.061 | 0.188 | 1.000 | 969 | tags=15%, list=5%, signal=16% |
| 493 | HDACI\_COLON\_BUT48HRS\_UP |  | 96 | 0.45 | 1.33 | 0.048 | 0.187 | 1.000 | 4525 | tags=35%, list=22%, signal=45% |
| 494 | BRG1\_ALAB\_DN |  | 37 | 0.51 | 1.33 | 0.095 | 0.187 | 1.000 | 3637 | tags=35%, list=18%, signal=43% |
| 495 | SASAKI\_ATL\_UP |  | 168 | 0.44 | 1.32 | 0.011 | 0.190 | 1.000 | 3923 | tags=30%, list=19%, signal=36% |
| 496 | BRCA\_BRCA1\_NEG |  | 154 | 0.45 | 1.32 | 0.029 | 0.190 | 1.000 | 2813 | tags=23%, list=14%, signal=26% |
| 497 | ET743\_SARCOMA\_24HRS\_DN |  | 110 | 0.46 | 1.32 | 0.037 | 0.190 | 1.000 | 4263 | tags=35%, list=21%, signal=44% |
| 498 | BRENTANI\_PROTEIN\_MODIFICATION |  | 143 | 0.45 | 1.32 | 0.023 | 0.190 | 1.000 | 5430 | tags=41%, list=26%, signal=56% |
| 499 | KUROKAWA\_5FU\_IFN\_SENSITIVE\_VS\_RESISTANT\_DN |  | 34 | 0.50 | 1.32 | 0.106 | 0.190 | 1.000 | 4474 | tags=38%, list=22%, signal=49% |
| 500 | TCRPATHWAY |  | 44 | 0.49 | 1.32 | 0.082 | 0.191 | 1.000 | 4361 | tags=36%, list=21%, signal=46% |
| 501 | NFATPATHWAY |  | 52 | 0.48 | 1.32 | 0.083 | 0.191 | 1.000 | 4190 | tags=40%, list=20%, signal=51% |
| 502 | CITED1\_KO\_HET\_UP |  | 27 | 0.52 | 1.32 | 0.117 | 0.191 | 1.000 | 2320 | tags=30%, list=11%, signal=33% |
| 503 | HSC\_STHSC\_SHARED |  | 33 | 0.50 | 1.32 | 0.106 | 0.193 | 1.000 | 5165 | tags=52%, list=25%, signal=69% |
| 504 | HEMATOPOESIS\_RELATED\_TRANSCRIPTION\_FACTORS |  | 84 | 0.46 | 1.32 | 0.054 | 0.193 | 1.000 | 5061 | tags=36%, list=25%, signal=47% |
| 505 | CELL\_PROLIFERATION |  | 203 | 0.43 | 1.32 | 0.009 | 0.193 | 1.000 | 4545 | tags=28%, list=22%, signal=35% |
| 506 | NELSON\_ANDROGEN\_UP |  | 60 | 0.47 | 1.32 | 0.080 | 0.193 | 1.000 | 3240 | tags=37%, list=16%, signal=43% |
| 507 | P53\_SIGNALING |  | 93 | 0.45 | 1.32 | 0.047 | 0.194 | 1.000 | 5067 | tags=35%, list=25%, signal=47% |
| 508 | ST\_P38\_MAPK\_PATHWAY |  | 36 | 0.50 | 1.32 | 0.104 | 0.195 | 1.000 | 3435 | tags=39%, list=17%, signal=47% |
| 509 | GLYCEROPHOSPHOLIPID\_METABOLISM |  | 49 | 0.48 | 1.32 | 0.085 | 0.195 | 1.000 | 4361 | tags=39%, list=21%, signal=49% |
| 510 | CMV\_HCMV\_TIMECOURSE\_16HRS\_DN |  | 20 | 0.55 | 1.32 | 0.120 | 0.195 | 1.000 | 3822 | tags=30%, list=19%, signal=37% |
| 511 | SASAKI\_TCELL\_LYMPHOMA\_VS\_CD4\_UP |  | 168 | 0.44 | 1.32 | 0.019 | 0.195 | 1.000 | 3923 | tags=30%, list=19%, signal=36% |
| 512 | SANA\_TNFA\_ENDOTHELIAL\_DN |  | 83 | 0.46 | 1.32 | 0.055 | 0.195 | 1.000 | 5394 | tags=42%, list=26%, signal=57% |
| 513 | ST\_G\_ALPHA\_I\_PATHWAY |  | 34 | 0.51 | 1.31 | 0.097 | 0.198 | 1.000 | 3407 | tags=29%, list=17%, signal=35% |
| 514 | GPCRPATHWAY |  | 35 | 0.51 | 1.31 | 0.111 | 0.199 | 1.000 | 4361 | tags=40%, list=21%, signal=51% |
| 515 | ROME\_INSULIN\_2F\_UP |  | 185 | 0.44 | 1.31 | 0.019 | 0.199 | 1.000 | 5455 | tags=43%, list=26%, signal=58% |
| 516 | BCNU\_GLIOMA\_NOMGMT\_48HRS\_UP |  | 18 | 0.56 | 1.31 | 0.142 | 0.201 | 1.000 | 5406 | tags=44%, list=26%, signal=60% |
| 517 | CHANG\_SERUM\_RESPONSE\_UP |  | 148 | 0.44 | 1.31 | 0.018 | 0.201 | 1.000 | 3840 | tags=33%, list=19%, signal=40% |
| 518 | NAKAJIMA\_MCSMBP\_MAST |  | 46 | 0.48 | 1.31 | 0.100 | 0.201 | 1.000 | 573 | tags=11%, list=3%, signal=11% |
| 519 | WNTPATHWAY |  | 25 | 0.53 | 1.31 | 0.120 | 0.201 | 1.000 | 4652 | tags=52%, list=23%, signal=67% |
| 520 | TPA\_RESIST\_LATE\_UP |  | 37 | 0.50 | 1.31 | 0.095 | 0.201 | 1.000 | 664 | tags=8%, list=3%, signal=8% |
| 521 | SANA\_IFNG\_ENDOTHELIAL\_DN |  | 86 | 0.46 | 1.31 | 0.051 | 0.201 | 1.000 | 4580 | tags=40%, list=22%, signal=51% |
| 522 | GREENBAUM\_E2A\_DN |  | 17 | 0.57 | 1.31 | 0.126 | 0.201 | 1.000 | 15 | tags=6%, list=0%, signal=6% |
| 523 | ZHAN\_MMPC\_LATEVS |  | 45 | 0.49 | 1.31 | 0.116 | 0.201 | 1.000 | 2518 | tags=27%, list=12%, signal=30% |
| 524 | GAMMA\_ESR\_OLD\_UNREG |  | 26 | 0.52 | 1.31 | 0.127 | 0.202 | 1.000 | 4473 | tags=38%, list=22%, signal=49% |
| 525 | AGED\_MOUSE\_HIPPOCAMPUS\_ANY\_UP |  | 45 | 0.49 | 1.31 | 0.106 | 0.203 | 1.000 | 5740 | tags=56%, list=28%, signal=77% |
| 526 | TIS7\_OVEREXP\_DN |  | 19 | 0.56 | 1.31 | 0.133 | 0.203 | 1.000 | 5993 | tags=58%, list=29%, signal=82% |
| 527 | APPEL\_IMATINIB\_UP |  | 31 | 0.51 | 1.31 | 0.108 | 0.203 | 1.000 | 2827 | tags=32%, list=14%, signal=37% |
| 528 | UVC\_HIGH\_D5\_DN |  | 36 | 0.49 | 1.31 | 0.124 | 0.204 | 1.000 | 6811 | tags=58%, list=33%, signal=87% |
| 529 | GATA3PATHWAY |  | 15 | 0.58 | 1.31 | 0.138 | 0.205 | 1.000 | 7045 | tags=53%, list=34%, signal=81% |
| 530 | GALINDO\_ACT\_UP |  | 76 | 0.46 | 1.31 | 0.064 | 0.204 | 1.000 | 1433 | tags=18%, list=7%, signal=20% |
| 531 | IRS1\_KO\_ADIP\_DN |  | 116 | 0.44 | 1.31 | 0.047 | 0.205 | 1.000 | 3807 | tags=33%, list=18%, signal=40% |
| 532 | PASSERINI\_IMMUNE |  | 21 | 0.54 | 1.30 | 0.148 | 0.207 | 1.000 | 4173 | tags=38%, list=20%, signal=48% |
| 533 | IGF\_VS\_PDGF\_UP |  | 71 | 0.46 | 1.30 | 0.079 | 0.207 | 1.000 | 3934 | tags=27%, list=19%, signal=33% |
| 534 | GAY\_YY1\_UP |  | 195 | 0.43 | 1.30 | 0.024 | 0.207 | 1.000 | 4453 | tags=27%, list=22%, signal=34% |
| 535 | XU\_CBP\_DN |  | 36 | 0.49 | 1.30 | 0.118 | 0.207 | 1.000 | 3109 | tags=28%, list=15%, signal=33% |
| 536 | CROONQUIST\_RAS\_STROMA\_UP |  | 24 | 0.53 | 1.30 | 0.131 | 0.207 | 1.000 | 3366 | tags=29%, list=16%, signal=35% |
| 537 | TAKEDA\_NUP8\_HOXA9\_10D\_DN |  | 152 | 0.44 | 1.30 | 0.031 | 0.207 | 1.000 | 5561 | tags=39%, list=27%, signal=53% |
| 538 | SHEPARD\_CELL\_PROLIFERATION |  | 203 | 0.43 | 1.30 | 0.014 | 0.207 | 1.000 | 4545 | tags=28%, list=22%, signal=35% |
| 539 | COCAINE\_BRAIN\_4WKS\_UP |  | 63 | 0.46 | 1.30 | 0.092 | 0.210 | 1.000 | 5543 | tags=41%, list=27%, signal=56% |
| 540 | HSC\_STHSC\_ADULT |  | 41 | 0.49 | 1.30 | 0.108 | 0.210 | 1.000 | 5165 | tags=46%, list=25%, signal=62% |
| 541 | LEE\_MYC\_UP |  | 53 | 0.47 | 1.30 | 0.099 | 0.210 | 1.000 | 5558 | tags=49%, list=27%, signal=67% |
| 542 | TPA\_RESIST\_LATE\_DN |  | 63 | 0.46 | 1.30 | 0.091 | 0.211 | 1.000 | 3995 | tags=30%, list=19%, signal=37% |
| 543 | TPA\_RESIST\_MIDDLE\_DN |  | 108 | 0.44 | 1.30 | 0.054 | 0.211 | 1.000 | 4111 | tags=30%, list=20%, signal=37% |
| 544 | GRANDVAUX\_IRF3\_DN |  | 20 | 0.55 | 1.30 | 0.151 | 0.211 | 1.000 | 1971 | tags=20%, list=10%, signal=22% |
| 545 | DEATHPATHWAY |  | 33 | 0.50 | 1.30 | 0.110 | 0.211 | 1.000 | 2612 | tags=21%, list=13%, signal=24% |
| 546 | KLEIN\_PEL\_UP |  | 50 | 0.48 | 1.30 | 0.086 | 0.211 | 1.000 | 3777 | tags=34%, list=18%, signal=42% |
| 547 | IDX\_TSA\_DN\_CLUSTER2 |  | 62 | 0.46 | 1.30 | 0.083 | 0.211 | 1.000 | 3838 | tags=35%, list=19%, signal=43% |
| 548 | SCHURINGA\_STAT5A\_DN |  | 17 | 0.57 | 1.29 | 0.155 | 0.216 | 1.000 | 3001 | tags=35%, list=15%, signal=41% |
| 549 | HIPPOCAMPUS\_DEVELOPMENT\_POSTNATAL |  | 45 | 0.48 | 1.29 | 0.123 | 0.217 | 1.000 | 1968 | tags=18%, list=10%, signal=20% |
| 550 | ERM\_KO\_TESTES\_DN |  | 21 | 0.53 | 1.29 | 0.149 | 0.217 | 1.000 | 5681 | tags=67%, list=28%, signal=92% |
| 551 | EDG1PATHWAY |  | 25 | 0.52 | 1.29 | 0.157 | 0.217 | 1.000 | 3367 | tags=32%, list=16%, signal=38% |
| 552 | P53GENES\_ALL |  | 17 | 0.56 | 1.29 | 0.132 | 0.217 | 1.000 | 3186 | tags=29%, list=15%, signal=35% |
| 553 | CASPASEPATHWAY |  | 22 | 0.53 | 1.29 | 0.160 | 0.217 | 1.000 | 1599 | tags=27%, list=8%, signal=30% |
| 554 | BIOPEPTIDESPATHWAY |  | 38 | 0.49 | 1.29 | 0.120 | 0.217 | 1.000 | 4361 | tags=34%, list=21%, signal=43% |
| 555 | HSC\_LATEPROGENITORS\_ADULT |  | 463 | 0.42 | 1.29 | 0.004 | 0.217 | 1.000 | 4134 | tags=32%, list=20%, signal=39% |
| 556 | MATSUDA\_VALPHAINKT\_DIFF |  | 408 | 0.42 | 1.29 | 0.003 | 0.217 | 1.000 | 4111 | tags=28%, list=20%, signal=35% |
| 557 | ADIPOGENESIS\_HMSC\_CLASS3\_UP |  | 66 | 0.46 | 1.29 | 0.089 | 0.216 | 1.000 | 1681 | tags=24%, list=8%, signal=26% |
| 558 | PENG\_GLUTAMINE\_UP |  | 227 | 0.43 | 1.29 | 0.013 | 0.218 | 1.000 | 5405 | tags=40%, list=26%, signal=53% |
| 559 | SMITH\_HTERT\_UP |  | 102 | 0.45 | 1.29 | 0.068 | 0.221 | 1.000 | 4818 | tags=42%, list=23%, signal=55% |
| 560 | HUMAN\_CD34\_ENRICHED\_TRANSCRIPTION\_FACTORS |  | 181 | 0.43 | 1.29 | 0.029 | 0.222 | 1.000 | 4760 | tags=36%, list=23%, signal=47% |
| 561 | PARK\_MSCS\_LIN2 |  | 40 | 0.49 | 1.29 | 0.133 | 0.223 | 1.000 | 3148 | tags=33%, list=15%, signal=38% |
| 562 | ZHAN\_MM\_CD138\_PR\_VS\_REST |  | 49 | 0.47 | 1.28 | 0.116 | 0.226 | 1.000 | 5447 | tags=45%, list=26%, signal=61% |
| 563 | MYOD\_NIH3T3\_UP |  | 82 | 0.45 | 1.28 | 0.088 | 0.228 | 1.000 | 4631 | tags=38%, list=22%, signal=49% |
| 564 | HSC\_HSC\_ADULT |  | 299 | 0.42 | 1.28 | 0.006 | 0.229 | 1.000 | 3485 | tags=26%, list=17%, signal=31% |
| 565 | HSC\_STHSC\_FETAL |  | 33 | 0.50 | 1.28 | 0.127 | 0.228 | 1.000 | 5165 | tags=52%, list=25%, signal=69% |
| 566 | HDACI\_COLON\_CURSUL\_UP |  | 42 | 0.48 | 1.28 | 0.118 | 0.229 | 1.000 | 4409 | tags=43%, list=21%, signal=54% |
| 567 | TNF\_AND\_FAS\_NETWORK |  | 17 | 0.56 | 1.28 | 0.147 | 0.229 | 1.000 | 3013 | tags=35%, list=15%, signal=41% |
| 568 | KENNY\_WNT\_DN |  | 45 | 0.47 | 1.28 | 0.133 | 0.230 | 1.000 | 6147 | tags=51%, list=30%, signal=73% |
| 569 | SERUM\_FIBROBLAST\_CORE\_UP |  | 202 | 0.43 | 1.28 | 0.027 | 0.234 | 1.000 | 3933 | tags=31%, list=19%, signal=38% |
| 570 | LIZUKA\_L1\_GR\_G1 |  | 20 | 0.54 | 1.28 | 0.152 | 0.235 | 1.000 | 4580 | tags=40%, list=22%, signal=51% |
| 571 | PARK\_RARALPHA\_MOD |  | 61 | 0.46 | 1.28 | 0.102 | 0.236 | 1.000 | 910 | tags=20%, list=4%, signal=21% |
| 572 | VEGF\_MMMEC\_ALL\_UP |  | 94 | 0.44 | 1.28 | 0.073 | 0.236 | 1.000 | 3161 | tags=21%, list=15%, signal=25% |
| 573 | MOREAUX\_TACI\_HI\_IN\_BMPC |  | 96 | 0.44 | 1.27 | 0.078 | 0.238 | 1.000 | 4554 | tags=28%, list=22%, signal=36% |
| 574 | MAGRANGEAS\_MULTIPLE\_MYELOMA\_IGL\_VS\_IGK\_DN |  | 27 | 0.50 | 1.27 | 0.167 | 0.239 | 1.000 | 1869 | tags=26%, list=9%, signal=28% |
| 575 | CROMER\_HYPOPHARYNGEAL\_MET\_VS\_NON\_DN |  | 81 | 0.44 | 1.27 | 0.083 | 0.244 | 1.000 | 4731 | tags=44%, list=23%, signal=57% |
| 576 | HSC\_HSC\_FETAL |  | 237 | 0.42 | 1.27 | 0.027 | 0.244 | 1.000 | 3341 | tags=26%, list=16%, signal=31% |
| 577 | CALCINEURIN\_NF\_AT\_SIGNALING |  | 93 | 0.44 | 1.27 | 0.074 | 0.246 | 1.000 | 4239 | tags=27%, list=21%, signal=34% |
| 578 | CARIES\_PULP\_DN |  | 70 | 0.45 | 1.27 | 0.096 | 0.248 | 1.000 | 4913 | tags=37%, list=24%, signal=49% |
| 579 | HYPERTROPHY\_MODEL |  | 20 | 0.54 | 1.27 | 0.180 | 0.248 | 1.000 | 3973 | tags=35%, list=19%, signal=43% |
| 580 | ZHAN\_MM\_CD138\_MF\_VS\_REST |  | 48 | 0.46 | 1.27 | 0.127 | 0.248 | 1.000 | 3631 | tags=27%, list=18%, signal=33% |
| 581 | H2O2\_CSBRESCUED\_C1\_UP |  | 42 | 0.47 | 1.27 | 0.129 | 0.249 | 1.000 | 3607 | tags=40%, list=18%, signal=49% |
| 582 | TSADAC\_HYPOMETH\_HYPERAC\_OVCA\_UP |  | 16 | 0.55 | 1.26 | 0.171 | 0.250 | 1.000 | 1783 | tags=31%, list=9%, signal=34% |
| 583 | IFN\_ANY\_UP |  | 81 | 0.44 | 1.26 | 0.095 | 0.250 | 1.000 | 4972 | tags=43%, list=24%, signal=57% |
| 584 | HSC\_LATEPROGENITORS\_SHARED |  | 456 | 0.41 | 1.26 | 0.003 | 0.250 | 1.000 | 4134 | tags=32%, list=20%, signal=39% |
| 585 | CHANG\_SERUM\_RESPONSE\_DN |  | 124 | 0.43 | 1.26 | 0.065 | 0.250 | 1.000 | 3682 | tags=29%, list=18%, signal=35% |
| 586 | CHESLER\_BRAIN\_ONLY\_SUBSET |  | 23 | 0.52 | 1.26 | 0.161 | 0.250 | 1.000 | 4760 | tags=43%, list=23%, signal=56% |
| 587 | RIBAVIRIN\_RSV\_DN |  | 43 | 0.47 | 1.26 | 0.145 | 0.250 | 1.000 | 4523 | tags=35%, list=22%, signal=45% |
| 588 | 41BBPATHWAY |  | 18 | 0.54 | 1.26 | 0.176 | 0.250 | 1.000 | 4425 | tags=33%, list=21%, signal=42% |
| 589 | ARAPPATHWAY |  | 20 | 0.52 | 1.26 | 0.177 | 0.250 | 1.000 | 6562 | tags=45%, list=32%, signal=66% |
| 590 | SHIPP\_DLBCL\_CURED\_DN |  | 37 | 0.47 | 1.26 | 0.165 | 0.250 | 1.000 | 4070 | tags=32%, list=20%, signal=40% |
| 591 | CIS\_XPC\_DN |  | 188 | 0.42 | 1.26 | 0.032 | 0.252 | 1.000 | 5144 | tags=38%, list=25%, signal=50% |
| 592 | MMS\_MOUSE\_LYMPH\_HIGH\_24HRS\_UP |  | 22 | 0.52 | 1.26 | 0.170 | 0.252 | 1.000 | 4776 | tags=55%, list=23%, signal=71% |
| 593 | LINDSTEDT\_DEND\_8H\_VS\_48H\_DN |  | 67 | 0.45 | 1.26 | 0.127 | 0.252 | 1.000 | 5014 | tags=36%, list=24%, signal=47% |
| 594 | IFNALPHA\_RESIST\_DN |  | 19 | 0.54 | 1.26 | 0.165 | 0.253 | 1.000 | 4217 | tags=53%, list=20%, signal=66% |
| 595 | TPA\_SENS\_LATE\_DN |  | 233 | 0.42 | 1.26 | 0.022 | 0.253 | 1.000 | 4133 | tags=28%, list=20%, signal=35% |
| 596 | LEI\_MYB\_REGULATED\_GENES |  | 317 | 0.41 | 1.26 | 0.007 | 0.253 | 1.000 | 3922 | tags=30%, list=19%, signal=36% |
| 597 | HDACI\_COLON\_CUR24HRS\_UP |  | 37 | 0.48 | 1.26 | 0.150 | 0.254 | 1.000 | 1822 | tags=24%, list=9%, signal=27% |
| 598 | AD12\_24HRS\_DN |  | 18 | 0.54 | 1.26 | 0.159 | 0.254 | 1.000 | 2322 | tags=28%, list=11%, signal=31% |
| 599 | HESS\_HOXAANMEIS1\_DN |  | 60 | 0.46 | 1.26 | 0.123 | 0.255 | 1.000 | 4800 | tags=37%, list=23%, signal=48% |
| 600 | BRENTANI\_TRANSCRIPTION\_FACTORS |  | 64 | 0.45 | 1.26 | 0.118 | 0.256 | 1.000 | 5737 | tags=41%, list=28%, signal=56% |
| 601 | ZHAN\_MMPC\_SIM |  | 42 | 0.47 | 1.26 | 0.146 | 0.255 | 1.000 | 5810 | tags=48%, list=28%, signal=66% |
| 602 | GERY\_CEBP\_TARGETS |  | 111 | 0.43 | 1.26 | 0.088 | 0.256 | 1.000 | 2567 | tags=23%, list=12%, signal=26% |
| 603 | HSC\_LATEPROGENITORS\_FETAL |  | 464 | 0.41 | 1.25 | 0.001 | 0.259 | 1.000 | 4134 | tags=31%, list=20%, signal=38% |
| 604 | ATRIA\_UP |  | 196 | 0.42 | 1.25 | 0.052 | 0.259 | 1.000 | 3979 | tags=26%, list=19%, signal=32% |
| 605 | GLYCOLYSIS\_AND\_GLUCONEOGENESIS |  | 42 | 0.47 | 1.25 | 0.149 | 0.260 | 1.000 | 1694 | tags=17%, list=8%, signal=18% |
| 606 | TPA\_SKIN\_DN |  | 16 | 0.55 | 1.25 | 0.189 | 0.259 | 1.000 | 4112 | tags=44%, list=20%, signal=55% |
| 607 | HESS\_HOXAANMEIS1\_UP |  | 60 | 0.46 | 1.25 | 0.120 | 0.259 | 1.000 | 4800 | tags=37%, list=23%, signal=48% |
| 608 | LEE\_MYC\_TGFA\_UP |  | 60 | 0.45 | 1.25 | 0.138 | 0.261 | 1.000 | 4390 | tags=38%, list=21%, signal=49% |
| 609 | TELPATHWAY |  | 15 | 0.55 | 1.25 | 0.198 | 0.261 | 1.000 | 3229 | tags=27%, list=16%, signal=32% |
| 610 | IFN\_BETA\_UP |  | 65 | 0.44 | 1.25 | 0.142 | 0.262 | 1.000 | 4972 | tags=46%, list=24%, signal=61% |
| 611 | IFN\_ALL\_UP |  | 18 | 0.53 | 1.25 | 0.194 | 0.262 | 1.000 | 4972 | tags=44%, list=24%, signal=59% |
| 612 | IFN\_GAMMA\_UP |  | 38 | 0.47 | 1.25 | 0.147 | 0.263 | 1.000 | 4972 | tags=37%, list=24%, signal=48% |
| 613 | IDX\_TSA\_DN\_CLUSTER3 |  | 78 | 0.43 | 1.25 | 0.120 | 0.264 | 1.000 | 2720 | tags=28%, list=13%, signal=32% |
| 614 | PAR1PATHWAY |  | 19 | 0.52 | 1.25 | 0.181 | 0.269 | 1.000 | 7738 | tags=68%, list=38%, signal=109% |
| 615 | HDACI\_COLON\_CUR24HRS\_DN |  | 25 | 0.51 | 1.25 | 0.166 | 0.270 | 1.000 | 4950 | tags=44%, list=24%, signal=58% |
| 616 | BRCA1\_MES\_UP |  | 39 | 0.46 | 1.25 | 0.165 | 0.270 | 1.000 | 3349 | tags=36%, list=16%, signal=43% |
| 617 | PYK2PATHWAY |  | 28 | 0.49 | 1.24 | 0.187 | 0.271 | 1.000 | 4361 | tags=43%, list=21%, signal=54% |
| 618 | GH\_EXOGENOUS\_LATE\_UP |  | 78 | 0.44 | 1.24 | 0.124 | 0.272 | 1.000 | 3994 | tags=24%, list=19%, signal=30% |
| 619 | ST\_MYOCYTE\_AD\_PATHWAY |  | 24 | 0.50 | 1.24 | 0.176 | 0.273 | 1.000 | 3912 | tags=25%, list=19%, signal=31% |
| 620 | NEMETH\_TNF\_UP |  | 80 | 0.43 | 1.24 | 0.127 | 0.273 | 1.000 | 5178 | tags=39%, list=25%, signal=52% |
| 621 | HOFMANN\_MDS\_CD34\_LOW\_RISK |  | 50 | 0.45 | 1.24 | 0.153 | 0.274 | 1.000 | 2651 | tags=28%, list=13%, signal=32% |
| 622 | EIF4PATHWAY |  | 24 | 0.51 | 1.24 | 0.185 | 0.275 | 1.000 | 3367 | tags=29%, list=16%, signal=35% |
| 623 | YU\_CMYC\_UP |  | 30 | 0.48 | 1.24 | 0.174 | 0.277 | 1.000 | 4751 | tags=43%, list=23%, signal=56% |
| 624 | H2O2\_CSBRESCUED\_UP |  | 56 | 0.45 | 1.24 | 0.150 | 0.278 | 1.000 | 3607 | tags=36%, list=18%, signal=43% |
| 625 | GSK3PATHWAY |  | 26 | 0.50 | 1.24 | 0.182 | 0.279 | 1.000 | 5695 | tags=50%, list=28%, signal=69% |
| 626 | UVC\_HIGH\_D7\_DN |  | 32 | 0.48 | 1.24 | 0.171 | 0.278 | 1.000 | 4348 | tags=47%, list=21%, signal=59% |
| 627 | TAKEDA\_NUP8\_HOXA9\_8D\_UP |  | 151 | 0.41 | 1.24 | 0.068 | 0.279 | 1.000 | 3497 | tags=25%, list=17%, signal=29% |
| 628 | NAKAJIMA\_MCSMBP\_EOS |  | 27 | 0.49 | 1.24 | 0.179 | 0.278 | 1.000 | 2534 | tags=22%, list=12%, signal=25% |
| 629 | MRNA\_PROCESSING\_REACTOME |  | 108 | 0.42 | 1.24 | 0.111 | 0.278 | 1.000 | 3614 | tags=31%, list=18%, signal=38% |
| 630 | UVC\_TTD\_8HR\_UP |  | 23 | 0.50 | 1.24 | 0.197 | 0.279 | 1.000 | 2448 | tags=26%, list=12%, signal=30% |
| 631 | BLEO\_HUMAN\_LYMPH\_HIGH\_24HRS\_UP |  | 92 | 0.43 | 1.24 | 0.113 | 0.280 | 1.000 | 3170 | tags=23%, list=15%, signal=27% |
| 632 | ST\_WNT\_CA2\_CYCLIC\_GMP\_PATHWAY |  | 19 | 0.53 | 1.24 | 0.196 | 0.280 | 1.000 | 3156 | tags=32%, list=15%, signal=37% |
| 633 | INNEREAR\_UP |  | 40 | 0.47 | 1.23 | 0.171 | 0.283 | 1.000 | 2258 | tags=15%, list=11%, signal=17% |
| 634 | GO\_ROS |  | 26 | 0.49 | 1.23 | 0.209 | 0.283 | 1.000 | 3321 | tags=31%, list=16%, signal=37% |
| 635 | ST\_DIFFERENTIATION\_PATHWAY\_IN\_PC12\_CELLS |  | 43 | 0.45 | 1.23 | 0.185 | 0.283 | 1.000 | 4050 | tags=33%, list=20%, signal=40% |
| 636 | TUMOR\_SUPRESSOR |  | 23 | 0.51 | 1.23 | 0.181 | 0.282 | 1.000 | 4464 | tags=39%, list=22%, signal=50% |
| 637 | O6BG\_RESIST\_MEDULLOBLASTOMA\_DN |  | 51 | 0.44 | 1.23 | 0.158 | 0.285 | 1.000 | 3282 | tags=31%, list=16%, signal=37% |
| 638 | P38MAPKPATHWAY |  | 39 | 0.47 | 1.23 | 0.179 | 0.285 | 1.000 | 4141 | tags=41%, list=20%, signal=51% |
| 639 | BREASTCA\_THREE\_CLASSES |  | 42 | 0.46 | 1.23 | 0.162 | 0.286 | 1.000 | 4731 | tags=40%, list=23%, signal=52% |
| 640 | IGF\_VS\_PDGF\_DN |  | 43 | 0.46 | 1.23 | 0.180 | 0.289 | 1.000 | 2550 | tags=30%, list=12%, signal=34% |
| 641 | SMITH\_HTERT\_DN |  | 61 | 0.44 | 1.23 | 0.151 | 0.289 | 1.000 | 963 | tags=16%, list=5%, signal=17% |
| 642 | GH\_EXOGENOUS\_ALL\_UP |  | 59 | 0.44 | 1.23 | 0.163 | 0.289 | 1.000 | 4994 | tags=24%, list=24%, signal=31% |
| 643 | SMITH\_HCV\_INDUCED\_HCC\_UP |  | 33 | 0.47 | 1.23 | 0.189 | 0.293 | 1.000 | 5387 | tags=45%, list=26%, signal=61% |
| 644 | CELL\_GROWTH\_AND\_OR\_MAINTENANCE |  | 60 | 0.44 | 1.23 | 0.143 | 0.293 | 1.000 | 4850 | tags=35%, list=24%, signal=46% |
| 645 | HOFMANN\_MANTEL\_LYMPHOMA\_VS\_LYMPH\_NODES\_UP |  | 49 | 0.45 | 1.23 | 0.179 | 0.294 | 1.000 | 3394 | tags=27%, list=16%, signal=32% |
| 646 | TSADAC\_RKOSILENT\_UP |  | 20 | 0.51 | 1.22 | 0.213 | 0.295 | 1.000 | 2325 | tags=20%, list=11%, signal=23% |
| 647 | HSC\_EARLYPROGENITORS\_FETAL |  | 448 | 0.40 | 1.22 | 0.011 | 0.295 | 1.000 | 5074 | tags=34%, list=25%, signal=44% |
| 648 | HSC\_EARLYPROGENITORS\_SHARED |  | 448 | 0.40 | 1.22 | 0.009 | 0.297 | 1.000 | 5074 | tags=34%, list=25%, signal=44% |
| 649 | KANNAN\_P53\_UP |  | 35 | 0.47 | 1.22 | 0.187 | 0.300 | 1.000 | 4558 | tags=37%, list=22%, signal=48% |
| 650 | STANELLE\_E2F1\_UP |  | 28 | 0.48 | 1.22 | 0.203 | 0.302 | 1.000 | 3199 | tags=29%, list=16%, signal=34% |
| 651 | HSC\_HSC\_SHARED |  | 223 | 0.40 | 1.22 | 0.060 | 0.302 | 1.000 | 3341 | tags=25%, list=16%, signal=30% |
| 652 | GCRPATHWAY |  | 17 | 0.52 | 1.22 | 0.205 | 0.301 | 1.000 | 7978 | tags=65%, list=39%, signal=105% |
| 653 | HIPPOCAMPUS\_DEVELOPMENT\_PRENATAL |  | 33 | 0.47 | 1.22 | 0.215 | 0.302 | 1.000 | 5644 | tags=52%, list=27%, signal=71% |
| 654 | PENG\_GLUCOSE\_DN |  | 134 | 0.41 | 1.22 | 0.110 | 0.303 | 1.000 | 5690 | tags=43%, list=28%, signal=58% |
| 655 | TAKEDA\_NUP8\_HOXA9\_10D\_UP |  | 190 | 0.40 | 1.22 | 0.062 | 0.303 | 1.000 | 3135 | tags=20%, list=15%, signal=23% |
| 656 | ADIP\_VS\_FIBRO\_UP |  | 34 | 0.46 | 1.22 | 0.196 | 0.303 | 1.000 | 896 | tags=12%, list=4%, signal=12% |
| 657 | ADIP\_DIFF\_CLUSTER2 |  | 40 | 0.46 | 1.22 | 0.205 | 0.303 | 1.000 | 1417 | tags=23%, list=7%, signal=24% |
| 658 | FSH\_OVARY\_MCV152\_UP |  | 61 | 0.44 | 1.22 | 0.172 | 0.303 | 1.000 | 2618 | tags=30%, list=13%, signal=34% |
| 659 | HSC\_EARLYPROGENITORS\_ADULT |  | 450 | 0.40 | 1.22 | 0.011 | 0.306 | 1.000 | 5074 | tags=34%, list=25%, signal=44% |
| 660 | NING\_COPD\_DN |  | 119 | 0.42 | 1.22 | 0.104 | 0.306 | 1.000 | 4112 | tags=32%, list=20%, signal=40% |
| 661 | OXSTRESS\_RPETWO\_DN |  | 108 | 0.41 | 1.21 | 0.114 | 0.306 | 1.000 | 4609 | tags=34%, list=22%, signal=44% |
| 662 | WANG\_MLL\_CBP\_VS\_GMP\_UP |  | 36 | 0.47 | 1.21 | 0.198 | 0.306 | 1.000 | 4131 | tags=33%, list=20%, signal=42% |
| 663 | CMV\_HCMV\_TIMECOURSE\_8HRS\_UP |  | 20 | 0.50 | 1.21 | 0.237 | 0.310 | 1.000 | 3140 | tags=25%, list=15%, signal=29% |
| 664 | PENG\_RAPAMYCIN\_UP |  | 154 | 0.40 | 1.21 | 0.118 | 0.312 | 1.000 | 4369 | tags=29%, list=21%, signal=37% |
| 665 | TARTE\_PLASMA\_BLASTIC |  | 307 | 0.40 | 1.21 | 0.043 | 0.315 | 1.000 | 3529 | tags=29%, list=17%, signal=34% |
| 666 | IGF1PATHWAY |  | 20 | 0.51 | 1.21 | 0.227 | 0.315 | 1.000 | 4531 | tags=50%, list=22%, signal=64% |
| 667 | HOFMANN\_MDS\_CD34\_HIGH\_RISK |  | 29 | 0.47 | 1.21 | 0.230 | 0.316 | 1.000 | 4573 | tags=34%, list=22%, signal=44% |
| 668 | HDACI\_COLON\_BUT\_UP |  | 180 | 0.40 | 1.21 | 0.083 | 0.318 | 1.000 | 4525 | tags=32%, list=22%, signal=41% |
| 669 | DAC\_PANC50\_UP |  | 42 | 0.45 | 1.21 | 0.190 | 0.318 | 1.000 | 6883 | tags=40%, list=33%, signal=61% |
| 670 | CMV\_HCMV\_TIMECOURSE\_4HRS\_DN |  | 35 | 0.46 | 1.21 | 0.204 | 0.320 | 1.000 | 3053 | tags=29%, list=15%, signal=33% |
| 671 | P21\_P53\_ANY\_DN |  | 49 | 0.44 | 1.21 | 0.179 | 0.320 | 1.000 | 4277 | tags=39%, list=21%, signal=49% |
| 672 | PDGF\_ES\_UP |  | 18 | 0.52 | 1.20 | 0.252 | 0.321 | 1.000 | 4663 | tags=39%, list=23%, signal=50% |
| 673 | GH\_HYPOPHYSECTOMY\_RAT\_DN |  | 15 | 0.53 | 1.20 | 0.220 | 0.322 | 1.000 | 3765 | tags=33%, list=18%, signal=41% |
| 674 | FALT\_BCLL\_IG\_MUTATED\_VS\_WT\_DN |  | 48 | 0.44 | 1.20 | 0.202 | 0.323 | 1.000 | 2282 | tags=23%, list=11%, signal=26% |
| 675 | AGED\_MOUSE\_NEOCORTEX\_UP |  | 67 | 0.42 | 1.20 | 0.174 | 0.323 | 1.000 | 2017 | tags=24%, list=10%, signal=26% |
| 676 | NUCLEAR\_RECEPTORS |  | 40 | 0.44 | 1.20 | 0.227 | 0.323 | 1.000 | 5383 | tags=35%, list=26%, signal=47% |
| 677 | ST\_ADRENERGIC |  | 33 | 0.46 | 1.20 | 0.213 | 0.324 | 1.000 | 3912 | tags=21%, list=19%, signal=26% |
| 678 | BRENTANI\_CELL\_CYCLE |  | 81 | 0.42 | 1.20 | 0.167 | 0.324 | 1.000 | 4220 | tags=36%, list=20%, signal=45% |
| 679 | NI2\_LUNG\_DN |  | 20 | 0.51 | 1.20 | 0.240 | 0.326 | 1.000 | 5757 | tags=55%, list=28%, signal=76% |
| 680 | NI2\_MOUSE\_DN |  | 45 | 0.44 | 1.20 | 0.220 | 0.329 | 1.000 | 4221 | tags=24%, list=20%, signal=31% |
| 681 | TNFALPHA\_ALL\_UP |  | 77 | 0.42 | 1.20 | 0.172 | 0.331 | 1.000 | 3283 | tags=27%, list=16%, signal=32% |
| 682 | RACCYCDPATHWAY |  | 22 | 0.50 | 1.20 | 0.232 | 0.332 | 1.000 | 3367 | tags=36%, list=16%, signal=43% |
| 683 | MARTINELLI\_IFNS\_DIFF |  | 21 | 0.50 | 1.20 | 0.249 | 0.333 | 1.000 | 128 | tags=10%, list=1%, signal=10% |
| 684 | TAVOR\_CEBP\_UP |  | 49 | 0.44 | 1.19 | 0.214 | 0.335 | 1.000 | 3912 | tags=35%, list=19%, signal=43% |
| 685 | MOREAUX\_TACI\_HI\_VS\_LOW\_UP |  | 413 | 0.39 | 1.19 | 0.030 | 0.335 | 1.000 | 4637 | tags=26%, list=23%, signal=33% |
| 686 | HOGERKORP\_CD44\_UP |  | 23 | 0.49 | 1.19 | 0.248 | 0.335 | 1.000 | 6724 | tags=52%, list=33%, signal=77% |
| 687 | HIVNEFPATHWAY |  | 55 | 0.43 | 1.19 | 0.200 | 0.334 | 1.000 | 3190 | tags=27%, list=15%, signal=32% |
| 688 | CMV\_24HRS\_UP |  | 72 | 0.42 | 1.19 | 0.175 | 0.335 | 1.000 | 3349 | tags=33%, list=16%, signal=40% |
| 689 | IRITANI\_ADPROX\_UP |  | 26 | 0.47 | 1.19 | 0.241 | 0.335 | 1.000 | 4252 | tags=42%, list=21%, signal=53% |
| 690 | HDACPATHWAY |  | 30 | 0.47 | 1.19 | 0.231 | 0.336 | 1.000 | 4531 | tags=40%, list=22%, signal=51% |
| 691 | FETAL\_LIVER\_ENRICHED\_TRANSCRIPTION\_FACTORS |  | 75 | 0.42 | 1.19 | 0.175 | 0.337 | 1.000 | 3381 | tags=24%, list=16%, signal=29% |
| 692 | PROSTAGLANDIN\_SYNTHESIS\_REGULATION |  | 28 | 0.48 | 1.19 | 0.245 | 0.336 | 1.000 | 6364 | tags=43%, list=31%, signal=62% |
| 693 | ADIP\_DIFF\_CLUSTER5 |  | 39 | 0.45 | 1.19 | 0.216 | 0.336 | 1.000 | 4060 | tags=41%, list=20%, signal=51% |
| 694 | AGED\_MOUSE\_HYPOTH\_UP |  | 44 | 0.44 | 1.19 | 0.217 | 0.336 | 1.000 | 3256 | tags=32%, list=16%, signal=38% |
| 695 | PPARAPATHWAY |  | 54 | 0.43 | 1.19 | 0.204 | 0.337 | 1.000 | 4281 | tags=35%, list=21%, signal=44% |
| 696 | UVC\_XPCS\_8HR\_UP |  | 58 | 0.42 | 1.19 | 0.210 | 0.339 | 1.000 | 4560 | tags=31%, list=22%, signal=40% |
| 697 | DCPATHWAY |  | 21 | 0.49 | 1.19 | 0.257 | 0.341 | 1.000 | 54 | tags=5%, list=0%, signal=5% |
| 698 | CMV\_HCMV\_TIMECOURSE\_ALL\_UP |  | 464 | 0.39 | 1.19 | 0.016 | 0.341 | 1.000 | 3814 | tags=26%, list=19%, signal=31% |
| 699 | MOREAUX\_TACI\_HI\_IN\_PPC\_UP |  | 73 | 0.41 | 1.19 | 0.207 | 0.341 | 1.000 | 3131 | tags=32%, list=15%, signal=37% |
| 700 | CELL\_MOTILITY |  | 106 | 0.40 | 1.19 | 0.158 | 0.341 | 1.000 | 3170 | tags=22%, list=15%, signal=26% |
| 701 | HYPOXIA\_NORMAL\_UP |  | 216 | 0.39 | 1.19 | 0.101 | 0.341 | 1.000 | 2960 | tags=27%, list=14%, signal=32% |
| 702 | SHEPARD\_CRASH\_AND\_BURN\_MUT\_VS\_WT\_DN |  | 155 | 0.40 | 1.19 | 0.148 | 0.341 | 1.000 | 5143 | tags=32%, list=25%, signal=43% |
| 703 | YAGI\_AML\_PROGNOSIS |  | 34 | 0.45 | 1.19 | 0.236 | 0.341 | 1.000 | 1467 | tags=18%, list=7%, signal=19% |
| 704 | STOSSI\_ER\_UP |  | 47 | 0.44 | 1.19 | 0.238 | 0.340 | 1.000 | 2718 | tags=17%, list=13%, signal=20% |
| 705 | ERKPATHWAY |  | 30 | 0.46 | 1.18 | 0.237 | 0.346 | 1.000 | 3407 | tags=30%, list=17%, signal=36% |
| 706 | SIG\_BCR\_SIGNALING\_PATHWAY |  | 45 | 0.44 | 1.18 | 0.215 | 0.347 | 1.000 | 5640 | tags=38%, list=27%, signal=52% |
| 707 | NAB\_LUNG\_UP |  | 27 | 0.47 | 1.18 | 0.265 | 0.348 | 1.000 | 2255 | tags=22%, list=11%, signal=25% |
| 708 | CALRES\_RHESUS\_UP |  | 67 | 0.43 | 1.18 | 0.201 | 0.348 | 1.000 | 4636 | tags=30%, list=22%, signal=38% |
| 709 | G1PATHWAY |  | 25 | 0.48 | 1.18 | 0.253 | 0.348 | 1.000 | 3425 | tags=36%, list=17%, signal=43% |
| 710 | CIRCADIAN\_EXERCISE |  | 41 | 0.44 | 1.18 | 0.240 | 0.349 | 1.000 | 2241 | tags=24%, list=11%, signal=27% |
| 711 | ASTON\_OLIGODENDROGLIA\_MYELINATION\_SUBSET |  | 17 | 0.51 | 1.18 | 0.276 | 0.350 | 1.000 | 699 | tags=12%, list=3%, signal=12% |
| 712 | ALCALAY\_AML\_NPMC\_UP |  | 139 | 0.40 | 1.18 | 0.152 | 0.350 | 1.000 | 4091 | tags=29%, list=20%, signal=36% |
| 713 | HEARTFAILURE\_ATRIA\_UP |  | 25 | 0.48 | 1.18 | 0.231 | 0.351 | 1.000 | 4093 | tags=24%, list=20%, signal=30% |
| 714 | UV-4NQO\_FIBRO\_UP |  | 28 | 0.47 | 1.18 | 0.248 | 0.350 | 1.000 | 6287 | tags=54%, list=31%, signal=77% |
| 715 | LEE\_TCELLS3\_UP |  | 106 | 0.41 | 1.18 | 0.185 | 0.351 | 1.000 | 4728 | tags=37%, list=23%, signal=48% |
| 716 | IDX\_TSA\_UP\_CLUSTER2 |  | 60 | 0.43 | 1.18 | 0.209 | 0.351 | 1.000 | 3071 | tags=28%, list=15%, signal=33% |
| 717 | WONG\_IFNA\_HCC\_RESISTANT\_VS\_SENSITIVE\_DN |  | 33 | 0.45 | 1.18 | 0.250 | 0.351 | 1.000 | 5252 | tags=48%, list=25%, signal=65% |
| 718 | BRENTANI\_IMMUNE\_FUNCTION |  | 50 | 0.43 | 1.18 | 0.228 | 0.353 | 1.000 | 2344 | tags=14%, list=11%, signal=16% |
| 719 | CHESLER\_BRAIN\_CIS\_GENES |  | 57 | 0.42 | 1.18 | 0.208 | 0.353 | 1.000 | 3806 | tags=33%, list=18%, signal=41% |
| 720 | HPV31\_UP |  | 59 | 0.43 | 1.18 | 0.216 | 0.352 | 1.000 | 3554 | tags=25%, list=17%, signal=31% |
| 721 | ALZHEIMERS\_INCIPIENT\_DN |  | 144 | 0.39 | 1.18 | 0.150 | 0.353 | 1.000 | 4368 | tags=31%, list=21%, signal=39% |
| 722 | CALRES\_RHESUS\_DN |  | 59 | 0.42 | 1.18 | 0.224 | 0.355 | 1.000 | 6796 | tags=47%, list=33%, signal=71% |
| 723 | HDACI\_COLON\_BUT24HRS\_UP |  | 70 | 0.41 | 1.17 | 0.206 | 0.357 | 1.000 | 2313 | tags=21%, list=11%, signal=24% |
| 724 | SHEPARD\_BMYB\_MORPHOLINO\_UP |  | 155 | 0.39 | 1.17 | 0.140 | 0.356 | 1.000 | 4876 | tags=33%, list=24%, signal=43% |
| 725 | AGED\_RHESUS\_DN |  | 108 | 0.40 | 1.17 | 0.183 | 0.356 | 1.000 | 5759 | tags=40%, list=28%, signal=55% |
| 726 | SCHURINGA\_STAT5A\_UP |  | 23 | 0.48 | 1.17 | 0.263 | 0.356 | 1.000 | 991 | tags=22%, list=5%, signal=23% |
| 727 | LEE\_CIP\_UP |  | 60 | 0.42 | 1.17 | 0.220 | 0.356 | 1.000 | 2290 | tags=27%, list=11%, signal=30% |
| 728 | DSRNA\_DN |  | 15 | 0.52 | 1.17 | 0.273 | 0.355 | 1.000 | 5254 | tags=40%, list=25%, signal=54% |
| 729 | HDACI\_COLON\_TSABUT\_UP |  | 72 | 0.41 | 1.17 | 0.209 | 0.359 | 1.000 | 2444 | tags=21%, list=12%, signal=24% |
| 730 | RETT\_UP |  | 40 | 0.44 | 1.17 | 0.241 | 0.358 | 1.000 | 5254 | tags=38%, list=25%, signal=50% |
| 731 | LIZUKA\_G1\_SM\_G2 |  | 25 | 0.47 | 1.17 | 0.269 | 0.362 | 1.000 | 1724 | tags=28%, list=8%, signal=31% |
| 732 | TPA\_SENS\_MIDDLE\_DN |  | 302 | 0.38 | 1.17 | 0.084 | 0.364 | 1.000 | 4202 | tags=26%, list=20%, signal=32% |
| 733 | TNFR1PATHWAY |  | 28 | 0.45 | 1.17 | 0.272 | 0.364 | 1.000 | 2069 | tags=29%, list=10%, signal=32% |
| 734 | UCALPAINPATHWAY |  | 16 | 0.51 | 1.17 | 0.284 | 0.365 | 1.000 | 4945 | tags=50%, list=24%, signal=66% |
| 735 | WELCH\_GATA1 |  | 24 | 0.47 | 1.17 | 0.300 | 0.367 | 1.000 | 1355 | tags=17%, list=7%, signal=18% |
| 736 | HDACI\_COLON\_BUT12HRS\_UP |  | 45 | 0.43 | 1.17 | 0.245 | 0.368 | 1.000 | 4525 | tags=33%, list=22%, signal=43% |
| 737 | AGED\_MOUSE\_HIPPOCAMPUS\_ANY\_DN |  | 42 | 0.43 | 1.17 | 0.247 | 0.369 | 1.000 | 4559 | tags=33%, list=22%, signal=43% |
| 738 | HOHENKIRK\_MONOCYTE\_DEND\_UP |  | 106 | 0.39 | 1.16 | 0.202 | 0.373 | 1.000 | 4985 | tags=29%, list=24%, signal=38% |
| 739 | HSC\_INTERMEDIATEPROGENITORS\_ADULT |  | 144 | 0.39 | 1.16 | 0.176 | 0.373 | 1.000 | 5661 | tags=40%, list=27%, signal=55% |
| 740 | NI2\_MOUSE\_UP |  | 39 | 0.43 | 1.16 | 0.258 | 0.373 | 1.000 | 3912 | tags=33%, list=19%, signal=41% |
| 741 | HBX\_HEP\_UP |  | 18 | 0.49 | 1.16 | 0.285 | 0.373 | 1.000 | 5061 | tags=56%, list=25%, signal=74% |
| 742 | MYOD\_BRG1\_UP |  | 27 | 0.47 | 1.16 | 0.271 | 0.373 | 1.000 | 5910 | tags=63%, list=29%, signal=88% |
| 743 | AGEING\_LYMPH\_DN |  | 17 | 0.50 | 1.16 | 0.302 | 0.373 | 1.000 | 5203 | tags=47%, list=25%, signal=63% |
| 744 | HBX\_HCC\_DN |  | 22 | 0.48 | 1.16 | 0.275 | 0.374 | 1.000 | 5249 | tags=50%, list=25%, signal=67% |
| 745 | IRS1\_KO\_ADIP\_UP |  | 96 | 0.40 | 1.16 | 0.202 | 0.374 | 1.000 | 4961 | tags=31%, list=24%, signal=41% |
| 746 | ALKPATHWAY |  | 33 | 0.45 | 1.16 | 0.260 | 0.380 | 1.000 | 5393 | tags=48%, list=26%, signal=66% |
| 747 | JISON\_SICKLE\_CELL |  | 31 | 0.45 | 1.16 | 0.277 | 0.379 | 1.000 | 5026 | tags=48%, list=24%, signal=64% |
| 748 | GLYCOSPHINGOLIPID\_METABOLISM |  | 23 | 0.47 | 1.16 | 0.287 | 0.381 | 1.000 | 3459 | tags=30%, list=17%, signal=37% |
| 749 | NEMETH\_TNF\_DN |  | 30 | 0.46 | 1.16 | 0.256 | 0.383 | 1.000 | 4368 | tags=40%, list=21%, signal=51% |
| 750 | BRENTANI\_CYTOSKELETON |  | 22 | 0.48 | 1.15 | 0.282 | 0.386 | 1.000 | 3991 | tags=36%, list=19%, signal=45% |
| 751 | CELL\_CYCLE |  | 78 | 0.40 | 1.15 | 0.239 | 0.386 | 1.000 | 3621 | tags=27%, list=18%, signal=33% |
| 752 | FLECHNER\_KIDNEY\_TRANSPLANT\_REJECTION\_UP |  | 86 | 0.40 | 1.15 | 0.233 | 0.386 | 1.000 | 4301 | tags=27%, list=21%, signal=34% |
| 753 | AGUIRRE\_PANCREAS\_CHR1 |  | 31 | 0.45 | 1.15 | 0.290 | 0.386 | 1.000 | 5772 | tags=45%, list=28%, signal=63% |
| 754 | VANASSE\_BCL2\_TARGETS |  | 86 | 0.40 | 1.15 | 0.212 | 0.387 | 1.000 | 3799 | tags=24%, list=18%, signal=30% |
| 755 | TOLLPATHWAY |  | 33 | 0.45 | 1.15 | 0.267 | 0.388 | 1.000 | 3533 | tags=24%, list=17%, signal=29% |
| 756 | ET743\_SARCOMA\_72HRS\_UP |  | 66 | 0.41 | 1.15 | 0.260 | 0.389 | 1.000 | 2567 | tags=23%, list=12%, signal=26% |
| 757 | GENOTOXINS\_24HRS\_DISCR |  | 39 | 0.43 | 1.15 | 0.277 | 0.390 | 1.000 | 5470 | tags=41%, list=27%, signal=56% |
| 758 | ABRAHAM\_MM\_VS\_AL\_UP |  | 19 | 0.48 | 1.15 | 0.301 | 0.390 | 1.000 | 250 | tags=16%, list=1%, signal=16% |
| 759 | FATTY\_ACID\_DEGRADATION |  | 26 | 0.47 | 1.15 | 0.270 | 0.390 | 1.000 | 5334 | tags=46%, list=26%, signal=62% |
| 760 | BYSTRYKH\_HSC\_BRAIN\_TRANS\_GLOCUS |  | 157 | 0.38 | 1.15 | 0.192 | 0.390 | 1.000 | 4607 | tags=31%, list=22%, signal=39% |
| 761 | HDACI\_COLON\_BUT12HRS\_DN |  | 74 | 0.41 | 1.15 | 0.261 | 0.392 | 1.000 | 5691 | tags=47%, list=28%, signal=65% |
| 762 | ABRAHAM\_AL\_VS\_MM\_DN |  | 18 | 0.49 | 1.15 | 0.304 | 0.392 | 1.000 | 250 | tags=17%, list=1%, signal=17% |
| 763 | RADIATION\_SENSITIVITY |  | 24 | 0.47 | 1.15 | 0.278 | 0.393 | 1.000 | 5681 | tags=46%, list=28%, signal=63% |
| 764 | TPA\_SENS\_EARLY\_DN |  | 284 | 0.37 | 1.15 | 0.130 | 0.393 | 1.000 | 4557 | tags=29%, list=22%, signal=37% |
| 765 | DNA\_DAMAGE\_SIGNALING |  | 88 | 0.40 | 1.15 | 0.234 | 0.392 | 1.000 | 5692 | tags=43%, list=28%, signal=59% |
| 766 | SERUM\_FIBROBLAST\_CELLCYCLE |  | 136 | 0.38 | 1.15 | 0.206 | 0.396 | 1.000 | 3752 | tags=32%, list=18%, signal=38% |
| 767 | BASSO\_REGULATORY\_HUBS |  | 138 | 0.39 | 1.15 | 0.200 | 0.395 | 1.000 | 4610 | tags=38%, list=22%, signal=48% |
| 768 | INSULINPATHWAY |  | 21 | 0.48 | 1.15 | 0.298 | 0.396 | 1.000 | 4239 | tags=43%, list=21%, signal=54% |
| 769 | TGFBETA\_ALL\_UP |  | 80 | 0.39 | 1.14 | 0.249 | 0.401 | 1.000 | 2398 | tags=26%, list=12%, signal=30% |
| 770 | UVC\_XPCS\_ALL\_UP |  | 61 | 0.41 | 1.14 | 0.264 | 0.401 | 1.000 | 4560 | tags=30%, list=22%, signal=38% |
| 771 | ST\_WNT\_BETA\_CATENIN\_PATHWAY |  | 31 | 0.45 | 1.14 | 0.306 | 0.401 | 1.000 | 4234 | tags=32%, list=21%, signal=41% |
| 772 | LI\_FETAL\_VS\_WT\_KIDNEY\_DN |  | 159 | 0.38 | 1.14 | 0.201 | 0.403 | 1.000 | 5126 | tags=35%, list=25%, signal=46% |
| 773 | CMV\_HCMV\_TIMECOURSE\_24HRS\_DN |  | 42 | 0.43 | 1.14 | 0.281 | 0.404 | 1.000 | 733 | tags=12%, list=4%, signal=12% |
| 774 | HDACI\_COLON\_SUL12HRS\_DN |  | 27 | 0.45 | 1.14 | 0.312 | 0.404 | 1.000 | 5518 | tags=56%, list=27%, signal=76% |
| 775 | OLDONLY\_FIBRO\_UP |  | 36 | 0.43 | 1.14 | 0.285 | 0.405 | 1.000 | 3232 | tags=31%, list=16%, signal=36% |
| 776 | HYPOPHYSECTOMY\_RAT\_UP |  | 34 | 0.44 | 1.14 | 0.299 | 0.405 | 1.000 | 3200 | tags=29%, list=16%, signal=35% |
| 777 | TPA\_RESIST\_MIDDLE\_UP |  | 49 | 0.41 | 1.14 | 0.294 | 0.412 | 1.000 | 4787 | tags=35%, list=23%, signal=45% |
| 778 | UVC\_LOW\_ALL\_UP |  | 19 | 0.48 | 1.13 | 0.324 | 0.414 | 1.000 | 1542 | tags=21%, list=7%, signal=23% |
| 779 | VERHAAK\_AML\_NPM1\_MUT\_VS\_WT\_UP |  | 189 | 0.38 | 1.13 | 0.191 | 0.415 | 1.000 | 1805 | tags=14%, list=9%, signal=16% |
| 780 | CELL\_SURFACE\_RECEPTOR\_LINKED\_SIGNAL\_TRANSDUCTION |  | 130 | 0.39 | 1.13 | 0.242 | 0.415 | 1.000 | 2344 | tags=12%, list=11%, signal=14% |
| 781 | UV\_UNIQUE\_FIBRO\_DN |  | 32 | 0.44 | 1.13 | 0.304 | 0.417 | 1.000 | 5959 | tags=41%, list=29%, signal=57% |
| 782 | HASLINGER\_B\_CLL\_13Q14 |  | 19 | 0.48 | 1.13 | 0.308 | 0.420 | 1.000 | 3746 | tags=32%, list=18%, signal=39% |
| 783 | LIN\_WNT\_UP |  | 53 | 0.41 | 1.13 | 0.291 | 0.422 | 1.000 | 2784 | tags=25%, list=14%, signal=28% |
| 784 | P53HYPOXIAPATHWAY |  | 19 | 0.48 | 1.13 | 0.320 | 0.422 | 1.000 | 1528 | tags=26%, list=7%, signal=28% |
| 785 | TNFR2PATHWAY |  | 18 | 0.48 | 1.13 | 0.330 | 0.423 | 1.000 | 9289 | tags=83%, list=45%, signal=152% |
| 786 | HYPOXIA\_RCC\_NOVHL\_UP |  | 66 | 0.40 | 1.13 | 0.270 | 0.424 | 1.000 | 4224 | tags=33%, list=20%, signal=42% |
| 787 | EGF\_HDMEC\_UP |  | 42 | 0.42 | 1.13 | 0.327 | 0.424 | 1.000 | 4574 | tags=48%, list=22%, signal=61% |
| 788 | HOX\_GENES |  | 54 | 0.40 | 1.13 | 0.307 | 0.427 | 1.000 | 6438 | tags=39%, list=31%, signal=56% |
| 789 | LEE\_MYC\_E2F1\_UP |  | 55 | 0.41 | 1.13 | 0.293 | 0.427 | 1.000 | 4686 | tags=33%, list=23%, signal=42% |
| 790 | HOFMANN\_MDS\_CD34\_LOW\_AND\_HIGH\_RISK |  | 46 | 0.41 | 1.12 | 0.323 | 0.431 | 1.000 | 4826 | tags=37%, list=23%, signal=48% |
| 791 | MEF2DPATHWAY |  | 20 | 0.47 | 1.12 | 0.324 | 0.434 | 1.000 | 4184 | tags=40%, list=20%, signal=50% |
| 792 | CIS\_XPC\_UP |  | 149 | 0.37 | 1.12 | 0.242 | 0.437 | 1.000 | 2156 | tags=18%, list=10%, signal=20% |
| 793 | KENNY\_WNT\_UP |  | 46 | 0.41 | 1.12 | 0.306 | 0.439 | 1.000 | 4358 | tags=35%, list=21%, signal=44% |
| 794 | VHL\_RCC\_UP |  | 110 | 0.38 | 1.12 | 0.283 | 0.438 | 1.000 | 5362 | tags=40%, list=26%, signal=54% |
| 795 | SHEPARD\_POS\_REG\_OF\_CELL\_PROLIFERATION |  | 93 | 0.39 | 1.12 | 0.273 | 0.440 | 1.000 | 3380 | tags=19%, list=16%, signal=23% |
| 796 | TARTE\_MATURE\_PC |  | 387 | 0.36 | 1.12 | 0.142 | 0.440 | 1.000 | 2646 | tags=15%, list=13%, signal=17% |
| 797 | BLEO\_HUMAN\_LYMPH\_HIGH\_4HRS\_UP |  | 21 | 0.47 | 1.12 | 0.344 | 0.441 | 1.000 | 4972 | tags=43%, list=24%, signal=56% |
| 798 | ADIP\_HUMAN\_UP |  | 60 | 0.40 | 1.12 | 0.290 | 0.441 | 1.000 | 2976 | tags=22%, list=14%, signal=25% |
| 799 | DAVIES\_N |  | 19 | 0.47 | 1.11 | 0.342 | 0.446 | 1.000 | 4301 | tags=26%, list=21%, signal=33% |
| 800 | BASSO\_GERMINAL\_CENTER\_CD40\_DN |  | 70 | 0.39 | 1.11 | 0.303 | 0.447 | 1.000 | 3897 | tags=24%, list=19%, signal=30% |
| 801 | CHEN\_LUNG\_SURVIVAL |  | 20 | 0.46 | 1.11 | 0.312 | 0.449 | 1.000 | 2115 | tags=30%, list=10%, signal=33% |
| 802 | VENTRICLES\_UP |  | 205 | 0.37 | 1.11 | 0.217 | 0.448 | 1.000 | 4002 | tags=28%, list=19%, signal=35% |
| 803 | BYSTRYKH\_HSC\_CIS\_GLOCUS |  | 104 | 0.38 | 1.11 | 0.278 | 0.448 | 1.000 | 4320 | tags=31%, list=21%, signal=39% |
| 804 | EICOSANOID\_SYNTHESIS |  | 17 | 0.47 | 1.11 | 0.350 | 0.455 | 1.000 | 3422 | tags=18%, list=17%, signal=21% |
| 805 | STURLA\_SONIC\_HEDGEHOG |  | 16 | 0.49 | 1.11 | 0.354 | 0.455 | 1.000 | 5413 | tags=56%, list=26%, signal=76% |
| 806 | BLEO\_MOUSE\_LYMPH\_HIGH\_24HRS\_DN |  | 34 | 0.42 | 1.11 | 0.342 | 0.456 | 1.000 | 3796 | tags=41%, list=18%, signal=50% |
| 807 | CALRES\_MOUSE\_NEOCORTEX\_UP |  | 59 | 0.40 | 1.10 | 0.337 | 0.464 | 1.000 | 2761 | tags=20%, list=13%, signal=23% |
| 808 | CELL\_ADHESION\_RECEPTOR\_ACTIVITY |  | 33 | 0.43 | 1.10 | 0.327 | 0.464 | 1.000 | 6873 | tags=55%, list=33%, signal=82% |
| 809 | YAGI\_AML\_PROG\_ASSOC |  | 127 | 0.37 | 1.10 | 0.293 | 0.466 | 1.000 | 5113 | tags=32%, list=25%, signal=43% |
| 810 | GAMMA\_HEXACHLOROCYCLOHEXANE\_DEGRADATION |  | 29 | 0.43 | 1.10 | 0.349 | 0.468 | 1.000 | 8220 | tags=62%, list=40%, signal=103% |
| 811 | KERATINOCYTEPATHWAY |  | 43 | 0.41 | 1.10 | 0.346 | 0.468 | 1.000 | 3190 | tags=23%, list=15%, signal=27% |
| 812 | FERNANDEZ\_MYC\_TARGETS |  | 178 | 0.36 | 1.10 | 0.260 | 0.469 | 1.000 | 3140 | tags=24%, list=15%, signal=28% |
| 813 | UBIQUITIN\_MEDIATED\_PROTEOLYSIS |  | 23 | 0.45 | 1.10 | 0.351 | 0.468 | 1.000 | 4374 | tags=57%, list=21%, signal=72% |
| 814 | HSC\_INTERMEDIATEPROGENITORS\_FETAL |  | 158 | 0.37 | 1.10 | 0.278 | 0.468 | 1.000 | 5661 | tags=39%, list=27%, signal=53% |
| 815 | COLLER\_MYC\_UP |  | 18 | 0.47 | 1.10 | 0.361 | 0.468 | 1.000 | 1807 | tags=28%, list=9%, signal=30% |
| 816 | GH\_EXOGENOUS\_LATE\_DN |  | 80 | 0.38 | 1.10 | 0.316 | 0.470 | 1.000 | 3137 | tags=18%, list=15%, signal=21% |
| 817 | ATP\_SYNTHESIS |  | 21 | 0.45 | 1.10 | 0.356 | 0.471 | 1.000 | 961 | tags=10%, list=5%, signal=10% |
| 818 | GLUCONEOGENESIS |  | 52 | 0.40 | 1.10 | 0.331 | 0.472 | 1.000 | 410 | tags=8%, list=2%, signal=8% |
| 819 | GLYCOLYSIS |  | 52 | 0.40 | 1.10 | 0.334 | 0.473 | 1.000 | 410 | tags=8%, list=2%, signal=8% |
| 820 | HDACI\_COLON\_BUT16HRS\_UP |  | 42 | 0.41 | 1.10 | 0.337 | 0.473 | 1.000 | 2313 | tags=19%, list=11%, signal=21% |
| 821 | TYPE\_III\_SECRETION\_SYSTEM |  | 21 | 0.45 | 1.09 | 0.366 | 0.478 | 1.000 | 961 | tags=10%, list=5%, signal=10% |
| 822 | AS3\_FIBRO\_UP |  | 44 | 0.41 | 1.09 | 0.351 | 0.479 | 1.000 | 3425 | tags=30%, list=17%, signal=35% |
| 823 | HSC\_INTERMEDIATEPROGENITORS\_SHARED |  | 133 | 0.37 | 1.09 | 0.320 | 0.482 | 1.000 | 5661 | tags=38%, list=27%, signal=53% |
| 824 | CAMPTOTHECIN\_PROBCELL\_DN |  | 30 | 0.43 | 1.09 | 0.364 | 0.482 | 1.000 | 3618 | tags=37%, list=18%, signal=44% |
| 825 | FLAGELLAR\_ASSEMBLY |  | 21 | 0.45 | 1.09 | 0.369 | 0.482 | 1.000 | 961 | tags=10%, list=5%, signal=10% |
| 826 | UVB\_NHEK3\_C8 |  | 66 | 0.39 | 1.09 | 0.339 | 0.482 | 1.000 | 5393 | tags=45%, list=26%, signal=61% |
| 827 | PORPHYRIN\_AND\_CHLOROPHYLL\_METABOLISM |  | 20 | 0.46 | 1.09 | 0.360 | 0.485 | 1.000 | 1764 | tags=20%, list=9%, signal=22% |
| 828 | CARDIACEGFPATHWAY |  | 17 | 0.47 | 1.09 | 0.362 | 0.485 | 1.000 | 1522 | tags=12%, list=7%, signal=13% |
| 829 | PRMT5\_KD\_DN |  | 25 | 0.44 | 1.09 | 0.371 | 0.485 | 1.000 | 6347 | tags=44%, list=31%, signal=64% |
| 830 | ST\_GAQ\_PATHWAY |  | 27 | 0.43 | 1.09 | 0.371 | 0.485 | 1.000 | 1353 | tags=15%, list=7%, signal=16% |
| 831 | NF90\_UP |  | 25 | 0.44 | 1.09 | 0.366 | 0.485 | 1.000 | 2558 | tags=24%, list=12%, signal=27% |
| 832 | GH\_EXOGENOUS\_ANY\_UP |  | 291 | 0.36 | 1.09 | 0.234 | 0.485 | 1.000 | 4024 | tags=19%, list=20%, signal=23% |
| 833 | REN\_E2F1\_TARGETS |  | 38 | 0.42 | 1.09 | 0.362 | 0.486 | 1.000 | 5037 | tags=42%, list=24%, signal=56% |
| 834 | TSA\_HEPATOMA\_UP |  | 36 | 0.42 | 1.09 | 0.362 | 0.486 | 1.000 | 3167 | tags=25%, list=15%, signal=29% |
| 835 | DAC\_BLADDER\_UP |  | 28 | 0.43 | 1.09 | 0.361 | 0.485 | 1.000 | 1124 | tags=14%, list=5%, signal=15% |
| 836 | AS3\_FIBRO\_C3 |  | 44 | 0.41 | 1.09 | 0.358 | 0.485 | 1.000 | 3425 | tags=30%, list=17%, signal=35% |
| 837 | BLOOD\_CLOTTING\_CASCADE |  | 20 | 0.46 | 1.09 | 0.372 | 0.490 | 1.000 | 2048 | tags=20%, list=10%, signal=22% |
| 838 | ZHAN\_MM\_MOLECULAR\_CLASSI\_UP |  | 62 | 0.39 | 1.09 | 0.356 | 0.489 | 1.000 | 3132 | tags=21%, list=15%, signal=25% |
| 839 | HOUSTIS\_ROS |  | 30 | 0.42 | 1.08 | 0.369 | 0.493 | 1.000 | 1478 | tags=17%, list=7%, signal=18% |
| 840 | FMLPPATHWAY |  | 35 | 0.41 | 1.08 | 0.379 | 0.493 | 1.000 | 4184 | tags=31%, list=20%, signal=39% |
| 841 | AGUIRRE\_PANCREAS\_CHR22 |  | 60 | 0.39 | 1.08 | 0.359 | 0.495 | 1.000 | 5455 | tags=37%, list=26%, signal=50% |
| 842 | GH\_GHRHR\_KO\_6HRS\_DN |  | 34 | 0.42 | 1.08 | 0.358 | 0.494 | 1.000 | 1554 | tags=18%, list=8%, signal=19% |
| 843 | PGC |  | 344 | 0.35 | 1.08 | 0.255 | 0.494 | 1.000 | 3514 | tags=23%, list=17%, signal=27% |
| 844 | BCL2\_FAMILY\_AND\_REG\_NETWORK |  | 21 | 0.45 | 1.08 | 0.385 | 0.500 | 1.000 | 5612 | tags=43%, list=27%, signal=59% |
| 845 | TAKEDA\_NUP8\_HOXA9\_3D\_DN |  | 30 | 0.42 | 1.08 | 0.378 | 0.502 | 1.000 | 1243 | tags=17%, list=6%, signal=18% |
| 846 | NITROGEN\_METABOLISM |  | 21 | 0.44 | 1.08 | 0.391 | 0.508 | 1.000 | 6634 | tags=29%, list=32%, signal=42% |
| 847 | H2O2\_CSBDIFF\_C2 |  | 33 | 0.42 | 1.07 | 0.374 | 0.509 | 1.000 | 3217 | tags=36%, list=16%, signal=43% |
| 848 | G2PATHWAY |  | 23 | 0.43 | 1.07 | 0.385 | 0.509 | 1.000 | 5343 | tags=35%, list=26%, signal=47% |
| 849 | GENOTOXINS\_ALL\_4HRS\_REG |  | 26 | 0.43 | 1.07 | 0.405 | 0.512 | 1.000 | 4358 | tags=42%, list=21%, signal=54% |
| 850 | ST\_DICTYOSTELIUM\_DISCOIDEUM\_CAMP\_CHEMOTAXIS\_PATHWAY |  | 32 | 0.42 | 1.07 | 0.387 | 0.512 | 1.000 | 3571 | tags=31%, list=17%, signal=38% |
| 851 | CHESLER\_BRAIN\_HIGHEST\_VARIANCE\_GENES |  | 24 | 0.43 | 1.07 | 0.424 | 0.511 | 1.000 | 1344 | tags=21%, list=7%, signal=22% |
| 852 | CMV\_HCMV\_TIMECOURSE\_14HRS\_UP |  | 45 | 0.40 | 1.07 | 0.379 | 0.512 | 1.000 | 4606 | tags=36%, list=22%, signal=46% |
| 853 | CANCERDRUGS\_PROBCELL\_DN |  | 15 | 0.48 | 1.07 | 0.396 | 0.513 | 1.000 | 1685 | tags=33%, list=8%, signal=36% |
| 854 | AGED\_MOUSE\_CEREBELLUM\_DN |  | 47 | 0.39 | 1.07 | 0.378 | 0.518 | 1.000 | 1843 | tags=13%, list=9%, signal=14% |
| 855 | FERRARI\_4HPR\_UP |  | 22 | 0.44 | 1.07 | 0.406 | 0.523 | 1.000 | 3448 | tags=32%, list=17%, signal=38% |
| 856 | HOFFMANN\_BIVSBII\_BI\_TABLE2 |  | 197 | 0.36 | 1.06 | 0.353 | 0.528 | 1.000 | 5133 | tags=34%, list=25%, signal=44% |
| 857 | UEDA\_MOUSE\_LIVER |  | 133 | 0.36 | 1.06 | 0.369 | 0.529 | 1.000 | 3982 | tags=30%, list=19%, signal=37% |
| 858 | DAVIES\_MGUS\_MM |  | 35 | 0.41 | 1.06 | 0.402 | 0.529 | 1.000 | 4470 | tags=31%, list=22%, signal=40% |
| 859 | SANSOM\_APC\_LOSS4\_UP |  | 115 | 0.36 | 1.06 | 0.366 | 0.531 | 1.000 | 4450 | tags=27%, list=22%, signal=34% |
| 860 | HBX\_NL\_UP |  | 22 | 0.44 | 1.06 | 0.419 | 0.532 | 1.000 | 5663 | tags=41%, list=27%, signal=56% |
| 861 | HUMAN\_TISSUE\_PANCREAS |  | 40 | 0.40 | 1.06 | 0.396 | 0.532 | 1.000 | 6302 | tags=40%, list=31%, signal=58% |
| 862 | CMV\_ALL\_UP |  | 93 | 0.36 | 1.06 | 0.383 | 0.534 | 1.000 | 5619 | tags=42%, list=27%, signal=57% |
| 863 | TPOPATHWAY |  | 23 | 0.43 | 1.06 | 0.425 | 0.535 | 1.000 | 4361 | tags=39%, list=21%, signal=50% |
| 864 | STRESSPATHWAY |  | 24 | 0.43 | 1.06 | 0.436 | 0.535 | 1.000 | 2318 | tags=21%, list=11%, signal=23% |
| 865 | LEE\_TCELLS6\_UP |  | 27 | 0.42 | 1.06 | 0.408 | 0.535 | 1.000 | 7240 | tags=41%, list=35%, signal=63% |
| 866 | SA\_CASPASE\_CASCADE |  | 16 | 0.47 | 1.06 | 0.410 | 0.536 | 1.000 | 1599 | tags=19%, list=8%, signal=20% |
| 867 | MAPK\_CASCADE |  | 29 | 0.41 | 1.06 | 0.406 | 0.536 | 1.000 | 3445 | tags=31%, list=17%, signal=37% |
| 868 | FERRANDO\_MLL\_T\_ALL\_DN |  | 83 | 0.37 | 1.05 | 0.403 | 0.543 | 1.000 | 2315 | tags=19%, list=11%, signal=22% |
| 869 | SIG\_CD40PATHWAYMAP |  | 33 | 0.41 | 1.05 | 0.422 | 0.544 | 1.000 | 3367 | tags=21%, list=16%, signal=25% |
| 870 | IFN\_ALPHA\_UP |  | 40 | 0.39 | 1.05 | 0.411 | 0.543 | 1.000 | 5619 | tags=48%, list=27%, signal=65% |
| 871 | ST\_GA13\_PATHWAY |  | 35 | 0.41 | 1.05 | 0.408 | 0.544 | 1.000 | 3190 | tags=31%, list=15%, signal=37% |
| 872 | O6BG\_RESIST\_MEDULLOBLASTOMA\_UP |  | 23 | 0.43 | 1.05 | 0.423 | 0.543 | 1.000 | 2088 | tags=26%, list=10%, signal=29% |
| 873 | HDACI\_COLON\_CLUSTER5 |  | 23 | 0.43 | 1.05 | 0.418 | 0.543 | 1.000 | 3887 | tags=35%, list=19%, signal=43% |
| 874 | INSULIN\_SIGNALING |  | 98 | 0.37 | 1.05 | 0.388 | 0.543 | 1.000 | 1778 | tags=16%, list=9%, signal=18% |
| 875 | DRUG\_RESISTANCE\_AND\_METABOLISM |  | 95 | 0.36 | 1.05 | 0.400 | 0.543 | 1.000 | 4714 | tags=31%, list=23%, signal=39% |
| 876 | STRESS\_IONIZING\_SPECIFIC\_UP |  | 15 | 0.47 | 1.05 | 0.430 | 0.547 | 1.000 | 7744 | tags=53%, list=38%, signal=85% |
| 877 | IRITANI\_ADPROX\_LYMPH |  | 125 | 0.36 | 1.05 | 0.409 | 0.548 | 1.000 | 4379 | tags=30%, list=21%, signal=37% |
| 878 | MAMMARY\_DEV\_UP |  | 55 | 0.38 | 1.05 | 0.420 | 0.548 | 1.000 | 5582 | tags=36%, list=27%, signal=50% |
| 879 | PEART\_HISTONE\_DN |  | 73 | 0.37 | 1.05 | 0.400 | 0.549 | 1.000 | 4893 | tags=34%, list=24%, signal=45% |
| 880 | SHEPARD\_BMYB\_MORPHOLINO\_DN |  | 168 | 0.35 | 1.05 | 0.380 | 0.549 | 1.000 | 4632 | tags=33%, list=22%, signal=42% |
| 881 | ZELLER\_MYC\_UP |  | 23 | 0.43 | 1.05 | 0.431 | 0.549 | 1.000 | 2991 | tags=30%, list=15%, signal=36% |
| 882 | LEE\_MYC\_DN |  | 59 | 0.37 | 1.05 | 0.429 | 0.550 | 1.000 | 666 | tags=10%, list=3%, signal=10% |
| 883 | DER\_IFNB\_UP |  | 93 | 0.36 | 1.05 | 0.429 | 0.552 | 1.000 | 5619 | tags=40%, list=27%, signal=54% |
| 884 | JECHLINGER\_EMT\_UP |  | 54 | 0.38 | 1.04 | 0.415 | 0.557 | 1.000 | 2677 | tags=20%, list=13%, signal=23% |
| 885 | ASTON\_DEPRESSION\_DN |  | 150 | 0.35 | 1.04 | 0.390 | 0.556 | 1.000 | 2461 | tags=19%, list=12%, signal=21% |
| 886 | CAMPTOTHECIN\_PROBCELL\_UP |  | 22 | 0.43 | 1.04 | 0.439 | 0.556 | 1.000 | 4158 | tags=27%, list=20%, signal=34% |
| 887 | INTEGRIN\_MEDIATED\_CELL\_ADHESION\_KEGG |  | 90 | 0.36 | 1.04 | 0.427 | 0.561 | 1.000 | 6142 | tags=40%, list=30%, signal=57% |
| 888 | ICHIBA\_GVHD |  | 238 | 0.35 | 1.04 | 0.396 | 0.560 | 1.000 | 2097 | tags=14%, list=10%, signal=16% |
| 889 | ZHAN\_MM\_CD138\_MS\_VS\_REST |  | 47 | 0.39 | 1.04 | 0.427 | 0.560 | 1.000 | 3069 | tags=23%, list=15%, signal=27% |
| 890 | VEGF\_HUVEC\_UP |  | 15 | 0.46 | 1.04 | 0.429 | 0.561 | 1.000 | 3516 | tags=27%, list=17%, signal=32% |
| 891 | CHEMICALPATHWAY |  | 21 | 0.43 | 1.04 | 0.437 | 0.562 | 1.000 | 6313 | tags=38%, list=31%, signal=55% |
| 892 | FERRANDO\_TAL1\_NEIGHBORS |  | 15 | 0.46 | 1.04 | 0.439 | 0.562 | 1.000 | 2232 | tags=33%, list=11%, signal=37% |
| 893 | REOVIRUS\_HEK293\_DN |  | 230 | 0.34 | 1.04 | 0.396 | 0.563 | 1.000 | 3118 | tags=21%, list=15%, signal=24% |
| 894 | PHOTOSYNTHESIS |  | 22 | 0.43 | 1.04 | 0.440 | 0.568 | 1.000 | 961 | tags=9%, list=5%, signal=10% |
| 895 | PYRIMIDINE\_METABOLISM |  | 58 | 0.37 | 1.03 | 0.440 | 0.571 | 1.000 | 2501 | tags=19%, list=12%, signal=22% |
| 896 | CSKPATHWAY |  | 22 | 0.42 | 1.03 | 0.436 | 0.571 | 1.000 | 6177 | tags=45%, list=30%, signal=65% |
| 897 | GLYCOGEN |  | 19 | 0.44 | 1.03 | 0.457 | 0.570 | 1.000 | 1935 | tags=26%, list=9%, signal=29% |
| 898 | TARTE\_PC |  | 80 | 0.36 | 1.03 | 0.430 | 0.570 | 1.000 | 4349 | tags=33%, list=21%, signal=41% |
| 899 | IRS\_KO\_ADIP\_DN |  | 39 | 0.39 | 1.03 | 0.463 | 0.570 | 1.000 | 5986 | tags=36%, list=29%, signal=50% |
| 900 | RAY\_P210\_DIFF |  | 53 | 0.38 | 1.03 | 0.448 | 0.571 | 1.000 | 1781 | tags=19%, list=9%, signal=21% |
| 901 | NKTPATHWAY |  | 26 | 0.41 | 1.03 | 0.451 | 0.573 | 1.000 | 6966 | tags=38%, list=34%, signal=58% |
| 902 | NADLER\_OBESITY\_DN |  | 36 | 0.39 | 1.03 | 0.440 | 0.573 | 1.000 | 1 | tags=3%, list=0%, signal=3% |
| 903 | P21\_P53\_MIDDLE\_DN |  | 25 | 0.42 | 1.03 | 0.452 | 0.580 | 1.000 | 4004 | tags=36%, list=19%, signal=45% |
| 904 | PARK\_HSC\_VS\_MPP\_UP |  | 16 | 0.45 | 1.03 | 0.444 | 0.579 | 1.000 | 3943 | tags=44%, list=19%, signal=54% |
| 905 | PURINE\_METABOLISM |  | 114 | 0.35 | 1.03 | 0.457 | 0.580 | 1.000 | 3025 | tags=17%, list=15%, signal=19% |
| 906 | DISTECHE\_XINACTIVATED\_GENES |  | 19 | 0.44 | 1.03 | 0.441 | 0.583 | 1.000 | 6332 | tags=53%, list=31%, signal=76% |
| 907 | WNT\_SIGNALING |  | 59 | 0.37 | 1.02 | 0.448 | 0.588 | 1.000 | 2450 | tags=20%, list=12%, signal=23% |
| 908 | PENG\_LEUCINE\_UP |  | 103 | 0.35 | 1.02 | 0.462 | 0.593 | 1.000 | 4755 | tags=33%, list=23%, signal=43% |
| 909 | HDACI\_COLON\_BUT2HRS\_UP |  | 75 | 0.35 | 1.02 | 0.458 | 0.593 | 1.000 | 4419 | tags=27%, list=21%, signal=34% |
| 910 | ROSS\_PML\_RAR |  | 80 | 0.36 | 1.02 | 0.454 | 0.596 | 1.000 | 2082 | tags=19%, list=10%, signal=21% |
| 911 | ROTH\_HTERT\_DIFF |  | 31 | 0.40 | 1.02 | 0.473 | 0.596 | 1.000 | 6440 | tags=35%, list=31%, signal=52% |
| 912 | ADIP\_DIFF\_UP |  | 67 | 0.36 | 1.02 | 0.483 | 0.595 | 1.000 | 4420 | tags=28%, list=21%, signal=36% |
| 913 | UVB\_NHEK3\_C0 |  | 82 | 0.35 | 1.02 | 0.469 | 0.595 | 1.000 | 2758 | tags=23%, list=13%, signal=27% |
| 914 | SANSOM\_APC\_4\_DN |  | 68 | 0.36 | 1.02 | 0.467 | 0.595 | 1.000 | 5260 | tags=31%, list=26%, signal=41% |
| 915 | CK1PATHWAY |  | 17 | 0.44 | 1.02 | 0.463 | 0.597 | 1.000 | 3325 | tags=29%, list=16%, signal=35% |
| 916 | CREB\_BRAIN\_8WKS\_UP |  | 75 | 0.36 | 1.01 | 0.475 | 0.602 | 1.000 | 4411 | tags=29%, list=21%, signal=37% |
| 917 | ZHAN\_MULTIPLE\_MYELOMA\_VS\_NORMAL\_DN |  | 39 | 0.38 | 1.01 | 0.473 | 0.605 | 1.000 | 4391 | tags=26%, list=21%, signal=33% |
| 918 | HBX\_NL\_DN |  | 16 | 0.44 | 1.01 | 0.450 | 0.607 | 1.000 | 5254 | tags=50%, list=25%, signal=67% |
| 919 | PENG\_GLUCOSE\_UP |  | 34 | 0.39 | 1.01 | 0.484 | 0.608 | 1.000 | 5080 | tags=35%, list=25%, signal=47% |
| 920 | BRCA\_PROGNOSIS\_POS |  | 41 | 0.37 | 1.01 | 0.485 | 0.610 | 1.000 | 3571 | tags=22%, list=17%, signal=26% |
| 921 | MANALO\_HYPOXIA\_DN |  | 78 | 0.35 | 1.01 | 0.490 | 0.612 | 1.000 | 3726 | tags=26%, list=18%, signal=31% |
| 922 | STARCH\_AND\_SUCROSE\_METABOLISM |  | 31 | 0.39 | 1.01 | 0.470 | 0.613 | 1.000 | 1764 | tags=16%, list=9%, signal=18% |
| 923 | SCHRAETS\_MLL\_UP |  | 34 | 0.39 | 1.01 | 0.494 | 0.613 | 1.000 | 4689 | tags=29%, list=23%, signal=38% |
| 924 | CORTEX\_ENRICHMENT\_LATE\_UP |  | 20 | 0.42 | 1.01 | 0.496 | 0.616 | 1.000 | 4217 | tags=40%, list=20%, signal=50% |
| 925 | CPR\_NULL-LOW\_LIVER\_UP |  | 17 | 0.43 | 1.01 | 0.499 | 0.616 | 1.000 | 4575 | tags=53%, list=22%, signal=68% |
| 926 | ZHAN\_MMPC\_SIM\_BC\_AND\_MM |  | 45 | 0.37 | 1.00 | 0.488 | 0.617 | 1.000 | 4488 | tags=36%, list=22%, signal=45% |
| 927 | BADPATHWAY |  | 22 | 0.42 | 1.00 | 0.474 | 0.616 | 1.000 | 7534 | tags=50%, list=37%, signal=79% |
| 928 | HISTIDINE\_METABOLISM |  | 25 | 0.40 | 1.00 | 0.466 | 0.618 | 1.000 | 513 | tags=12%, list=2%, signal=12% |
| 929 | GAMMA\_UNIQUE\_FIBRO\_DN |  | 60 | 0.36 | 1.00 | 0.511 | 0.619 | 1.000 | 4750 | tags=28%, list=23%, signal=37% |
| 930 | AMIPATHWAY |  | 22 | 0.42 | 1.00 | 0.480 | 0.618 | 1.000 | 6177 | tags=45%, list=30%, signal=65% |
| 931 | ZHAN\_MM\_CD138\_CD2\_VS\_REST |  | 40 | 0.38 | 1.00 | 0.497 | 0.618 | 1.000 | 4301 | tags=25%, list=21%, signal=32% |
| 932 | ELECTRON\_TRANSPORT\_CHAIN |  | 98 | 0.35 | 1.00 | 0.505 | 0.620 | 1.000 | 5235 | tags=40%, list=25%, signal=53% |
| 933 | HUMAN\_TISSUE\_THYMUS |  | 16 | 0.44 | 1.00 | 0.490 | 0.620 | 1.000 | 3081 | tags=19%, list=15%, signal=22% |
| 934 | OXIDATIVE\_PHOSPHORYLATION |  | 58 | 0.36 | 1.00 | 0.517 | 0.622 | 1.000 | 5294 | tags=33%, list=26%, signal=44% |
| 935 | PENG\_RAPAMYCIN\_DN |  | 188 | 0.34 | 1.00 | 0.496 | 0.623 | 1.000 | 4505 | tags=32%, list=22%, signal=41% |
| 936 | YAO\_P4\_KO\_VS\_WT\_UP |  | 71 | 0.35 | 1.00 | 0.519 | 0.627 | 1.000 | 3373 | tags=25%, list=16%, signal=30% |
| 937 | TAKEDA\_NUP8\_HOXA9\_16D\_UP |  | 172 | 0.33 | 0.99 | 0.530 | 0.631 | 1.000 | 5981 | tags=30%, list=29%, signal=42% |
| 938 | UVB\_NHEK2\_UP |  | 68 | 0.35 | 0.99 | 0.519 | 0.631 | 1.000 | 1255 | tags=13%, list=6%, signal=14% |
| 939 | BRCA1\_SW480\_DN |  | 16 | 0.43 | 0.99 | 0.516 | 0.631 | 1.000 | 4652 | tags=50%, list=23%, signal=65% |
| 940 | ROSS\_AML1\_ETO |  | 82 | 0.34 | 0.99 | 0.510 | 0.633 | 1.000 | 2946 | tags=22%, list=14%, signal=26% |
| 941 | GLEEVECPATHWAY |  | 22 | 0.41 | 0.99 | 0.498 | 0.635 | 1.000 | 4141 | tags=36%, list=20%, signal=45% |
| 942 | MRNA\_SPLICING |  | 49 | 0.36 | 0.99 | 0.528 | 0.635 | 1.000 | 5085 | tags=41%, list=25%, signal=54% |
| 943 | CMV\_HCMV\_TIMECOURSE\_10HRS\_DN |  | 15 | 0.44 | 0.99 | 0.500 | 0.639 | 1.000 | 3492 | tags=33%, list=17%, signal=40% |
| 944 | ST\_T\_CELL\_SIGNAL\_TRANSDUCTION |  | 44 | 0.37 | 0.99 | 0.508 | 0.639 | 1.000 | 4776 | tags=27%, list=23%, signal=35% |
| 945 | GH\_EXOGENOUS\_ANY\_DN |  | 96 | 0.34 | 0.99 | 0.533 | 0.640 | 1.000 | 3137 | tags=15%, list=15%, signal=17% |
| 946 | CHAUVIN\_ANDROGEN\_REGULATED\_GENES |  | 43 | 0.37 | 0.99 | 0.508 | 0.640 | 1.000 | 6756 | tags=47%, list=33%, signal=69% |
| 947 | ETSPATHWAY |  | 18 | 0.42 | 0.99 | 0.529 | 0.640 | 1.000 | 3927 | tags=28%, list=19%, signal=34% |
| 948 | HDACI\_COLON\_CLUSTER9 |  | 65 | 0.35 | 0.99 | 0.532 | 0.642 | 1.000 | 3341 | tags=22%, list=16%, signal=26% |
| 949 | HUMAN\_MITODB\_6\_2002 |  | 382 | 0.32 | 0.99 | 0.553 | 0.642 | 1.000 | 4880 | tags=30%, list=24%, signal=38% |
| 950 | UVC\_TTD-XPCS\_COMMON\_UP |  | 21 | 0.41 | 0.99 | 0.502 | 0.643 | 1.000 | 4522 | tags=33%, list=22%, signal=43% |
| 951 | PASSERINI\_ADHESION |  | 37 | 0.37 | 0.98 | 0.501 | 0.643 | 1.000 | 2714 | tags=16%, list=13%, signal=19% |
| 952 | CALCIUM\_REGULATION\_IN\_CARDIAC\_CELLS |  | 140 | 0.33 | 0.98 | 0.541 | 0.646 | 1.000 | 6212 | tags=32%, list=30%, signal=46% |
| 953 | FRUCTOSE\_AND\_MANNOSE\_METABOLISM |  | 25 | 0.39 | 0.98 | 0.516 | 0.647 | 1.000 | 330 | tags=8%, list=2%, signal=8% |
| 954 | AMINOACYL\_TRNA\_BIOSYNTHESIS |  | 23 | 0.40 | 0.98 | 0.507 | 0.650 | 1.000 | 5532 | tags=43%, list=27%, signal=59% |
| 955 | NING\_COPD\_UP |  | 144 | 0.33 | 0.98 | 0.573 | 0.652 | 1.000 | 3551 | tags=26%, list=17%, signal=31% |
| 956 | HANSON\_NFKAPPB\_IND |  | 18 | 0.42 | 0.98 | 0.521 | 0.652 | 1.000 | 6040 | tags=39%, list=29%, signal=55% |
| 957 | CALRES\_MOUSE\_NEOCORTEX\_DN |  | 66 | 0.35 | 0.98 | 0.550 | 0.652 | 1.000 | 1620 | tags=15%, list=8%, signal=16% |
| 958 | POD1\_KO\_UP |  | 412 | 0.32 | 0.98 | 0.568 | 0.653 | 1.000 | 5490 | tags=31%, list=27%, signal=42% |
| 959 | CMV\_HCMV\_TIMECOURSE\_16HRS\_UP |  | 57 | 0.35 | 0.98 | 0.531 | 0.652 | 1.000 | 3095 | tags=23%, list=15%, signal=27% |
| 960 | AGED\_MOUSE\_MUSCLE\_UP |  | 31 | 0.38 | 0.98 | 0.536 | 0.654 | 1.000 | 1369 | tags=13%, list=7%, signal=14% |
| 961 | SHEPARD\_GENES\_COMMON\_BW\_CB\_MO |  | 68 | 0.35 | 0.97 | 0.549 | 0.660 | 1.000 | 4611 | tags=32%, list=22%, signal=42% |
| 962 | UV-4NQO\_FIBRO\_DN |  | 28 | 0.38 | 0.97 | 0.539 | 0.663 | 1.000 | 2191 | tags=14%, list=11%, signal=16% |
| 963 | CMV\_HCMV\_TIMECOURSE\_20HRS\_UP |  | 84 | 0.33 | 0.97 | 0.568 | 0.665 | 1.000 | 4537 | tags=32%, list=22%, signal=41% |
| 964 | SMOOTH\_MUSCLE\_CONTRACTION |  | 143 | 0.32 | 0.97 | 0.575 | 0.665 | 1.000 | 2567 | tags=16%, list=12%, signal=18% |
| 965 | INOSITOL\_PHOSPHATE\_METABOLISM |  | 25 | 0.39 | 0.97 | 0.533 | 0.665 | 1.000 | 5873 | tags=40%, list=29%, signal=56% |
| 966 | BREAST\_DUCTAL\_CARCINOMA\_GENES |  | 19 | 0.41 | 0.97 | 0.524 | 0.666 | 1.000 | 3342 | tags=37%, list=16%, signal=44% |
| 967 | P21\_ANY\_DN |  | 35 | 0.37 | 0.97 | 0.536 | 0.666 | 1.000 | 4998 | tags=43%, list=24%, signal=56% |
| 968 | GAY\_YY1\_DN |  | 243 | 0.32 | 0.97 | 0.596 | 0.667 | 1.000 | 5576 | tags=30%, list=27%, signal=41% |
| 969 | AGED\_MOUSE\_MUSCLE\_DN |  | 32 | 0.38 | 0.97 | 0.539 | 0.667 | 1.000 | 4112 | tags=38%, list=20%, signal=47% |
| 970 | G1\_TO\_S\_CELL\_CYCLE\_REACTOME |  | 68 | 0.34 | 0.97 | 0.562 | 0.666 | 1.000 | 5026 | tags=29%, list=24%, signal=39% |
| 971 | CMV-UV\_HCMV\_6HRS\_UP |  | 120 | 0.33 | 0.97 | 0.577 | 0.668 | 1.000 | 4957 | tags=28%, list=24%, signal=36% |
| 972 | BYSTRYKH\_HSC\_BRAIN\_CIS\_GLOCUS |  | 51 | 0.35 | 0.97 | 0.532 | 0.670 | 1.000 | 4320 | tags=35%, list=21%, signal=45% |
| 973 | HOFFMANN\_BIVSBII\_LGBII |  | 101 | 0.33 | 0.97 | 0.574 | 0.670 | 1.000 | 5606 | tags=30%, list=27%, signal=41% |
| 974 | AGED\_MOUSE\_NEOCORTEX\_DN |  | 55 | 0.35 | 0.96 | 0.545 | 0.673 | 1.000 | 3673 | tags=20%, list=18%, signal=24% |
| 975 | XPB\_TTD-CS\_UP |  | 26 | 0.39 | 0.96 | 0.544 | 0.675 | 1.000 | 6581 | tags=42%, list=32%, signal=62% |
| 976 | UV-CMV\_UNIQUE\_HCMV\_6HRS\_UP |  | 102 | 0.33 | 0.96 | 0.605 | 0.676 | 1.000 | 4957 | tags=27%, list=24%, signal=36% |
| 977 | PLATELET\_EXPRESSED |  | 33 | 0.37 | 0.96 | 0.541 | 0.679 | 1.000 | 2687 | tags=21%, list=13%, signal=24% |
| 978 | ATMPATHWAY |  | 19 | 0.40 | 0.96 | 0.541 | 0.681 | 1.000 | 5681 | tags=37%, list=28%, signal=51% |
| 979 | LIAN\_MYELOID\_DIFF\_GRANULE |  | 24 | 0.39 | 0.96 | 0.531 | 0.680 | 1.000 | 1764 | tags=17%, list=9%, signal=18% |
| 980 | FERRANDO\_MLL\_T\_ALL\_UP |  | 87 | 0.33 | 0.96 | 0.588 | 0.680 | 1.000 | 4058 | tags=28%, list=20%, signal=34% |
| 981 | CPR\_NULL\_LIVER\_DN |  | 17 | 0.41 | 0.96 | 0.560 | 0.680 | 1.000 | 5479 | tags=41%, list=27%, signal=56% |
| 982 | EMT\_UP |  | 61 | 0.34 | 0.96 | 0.577 | 0.680 | 1.000 | 2677 | tags=18%, list=13%, signal=21% |
| 983 | APOPTOSIS\_KEGG |  | 49 | 0.35 | 0.95 | 0.558 | 0.686 | 1.000 | 7172 | tags=41%, list=35%, signal=62% |
| 984 | YU\_CMYC\_DN |  | 44 | 0.35 | 0.95 | 0.540 | 0.687 | 1.000 | 5405 | tags=23%, list=26%, signal=31% |
| 985 | ARFPATHWAY |  | 16 | 0.42 | 0.95 | 0.563 | 0.696 | 1.000 | 3367 | tags=31%, list=16%, signal=37% |
| 986 | MPRPATHWAY |  | 23 | 0.39 | 0.95 | 0.570 | 0.700 | 1.000 | 3325 | tags=26%, list=16%, signal=31% |
| 987 | WANG\_MLL\_CBP\_VS\_GMP\_DN |  | 40 | 0.35 | 0.94 | 0.573 | 0.705 | 1.000 | 5949 | tags=38%, list=29%, signal=53% |
| 988 | HUMAN\_TISSUE\_TESTIS |  | 59 | 0.34 | 0.94 | 0.590 | 0.707 | 1.000 | 6478 | tags=36%, list=31%, signal=52% |
| 989 | ST\_INTERLEUKIN\_4\_PATHWAY |  | 26 | 0.37 | 0.94 | 0.563 | 0.708 | 1.000 | 4297 | tags=35%, list=21%, signal=44% |
| 990 | WIELAND\_HEPATITIS\_B\_INDUCED |  | 106 | 0.32 | 0.94 | 0.620 | 0.712 | 1.000 | 4260 | tags=24%, list=21%, signal=30% |
| 991 | NF90\_DN |  | 38 | 0.35 | 0.94 | 0.588 | 0.713 | 1.000 | 1107 | tags=16%, list=5%, signal=17% |
| 992 | ZHAN\_MMPC\_PC |  | 22 | 0.39 | 0.93 | 0.591 | 0.718 | 1.000 | 6993 | tags=45%, list=34%, signal=69% |
| 993 | CROONQUIST\_IL6\_RAS\_UP |  | 22 | 0.39 | 0.93 | 0.599 | 0.721 | 1.000 | 468 | tags=9%, list=2%, signal=9% |
| 994 | UVB\_NHEK3\_C6 |  | 30 | 0.37 | 0.93 | 0.592 | 0.723 | 1.000 | 7595 | tags=57%, list=37%, signal=90% |
| 995 | HYPOPHYSECTOMY\_RAT\_DN |  | 49 | 0.34 | 0.93 | 0.616 | 0.722 | 1.000 | 2341 | tags=18%, list=11%, signal=21% |
| 996 | G\_PROTEIN\_SIGNALING |  | 91 | 0.32 | 0.93 | 0.624 | 0.723 | 1.000 | 4184 | tags=27%, list=20%, signal=34% |
| 997 | PTDINSPATHWAY |  | 22 | 0.38 | 0.93 | 0.590 | 0.724 | 1.000 | 4731 | tags=41%, list=23%, signal=53% |
| 998 | CANCER\_NEOPLASTIC\_META\_UP |  | 61 | 0.33 | 0.93 | 0.609 | 0.726 | 1.000 | 4051 | tags=31%, list=20%, signal=39% |
| 999 | ERK5PATHWAY |  | 17 | 0.40 | 0.93 | 0.576 | 0.729 | 1.000 | 4361 | tags=47%, list=21%, signal=60% |
| 1000 | MARSHALL\_SPLEEN\_BAL |  | 21 | 0.38 | 0.92 | 0.587 | 0.736 | 1.000 | 1542 | tags=10%, list=7%, signal=10% |
| 1001 | DNMT1\_KO\_UP |  | 72 | 0.32 | 0.92 | 0.629 | 0.741 | 1.000 | 4716 | tags=36%, list=23%, signal=47% |
| 1002 | HCMVPATHWAY |  | 15 | 0.41 | 0.92 | 0.597 | 0.742 | 1.000 | 3367 | tags=27%, list=16%, signal=32% |
| 1003 | AGUIRRE\_PANCREAS\_CHR17 |  | 70 | 0.32 | 0.92 | 0.644 | 0.743 | 1.000 | 6388 | tags=41%, list=31%, signal=60% |
| 1004 | MITOCHONDRIA |  | 391 | 0.30 | 0.92 | 0.770 | 0.745 | 1.000 | 4860 | tags=29%, list=24%, signal=38% |
| 1005 | PASSERINI\_OXIDATION |  | 19 | 0.38 | 0.91 | 0.609 | 0.751 | 1.000 | 1538 | tags=16%, list=7%, signal=17% |
| 1006 | NOUZOVA\_CPG\_METHLTD |  | 64 | 0.32 | 0.91 | 0.637 | 0.753 | 1.000 | 3147 | tags=19%, list=15%, signal=22% |
| 1007 | ADIP\_VS\_PREADIP\_UP |  | 35 | 0.35 | 0.91 | 0.634 | 0.753 | 1.000 | 896 | tags=6%, list=4%, signal=6% |
| 1008 | ET743\_RESIST\_UP |  | 17 | 0.40 | 0.91 | 0.624 | 0.754 | 1.000 | 2720 | tags=29%, list=13%, signal=34% |
| 1009 | CITED1\_KO\_WT\_UP |  | 16 | 0.40 | 0.90 | 0.623 | 0.765 | 1.000 | 7139 | tags=56%, list=35%, signal=86% |
| 1010 | AMINOSUGARS\_METABOLISM |  | 15 | 0.40 | 0.90 | 0.625 | 0.764 | 1.000 | 1698 | tags=20%, list=8%, signal=22% |
| 1011 | CMV\_HCMV\_TIMECOURSE\_48HRS\_UP |  | 66 | 0.32 | 0.90 | 0.647 | 0.769 | 1.000 | 6040 | tags=33%, list=29%, signal=47% |
| 1012 | NADLER\_OBESITY\_UP |  | 52 | 0.33 | 0.90 | 0.675 | 0.772 | 1.000 | 4369 | tags=33%, list=21%, signal=41% |
| 1013 | CELL\_CYCLE\_REGULATOR |  | 24 | 0.36 | 0.90 | 0.615 | 0.773 | 1.000 | 5387 | tags=42%, list=26%, signal=56% |
| 1014 | BASSO\_GERMINAL\_CENTER\_CD40\_UP |  | 97 | 0.31 | 0.90 | 0.690 | 0.773 | 1.000 | 4892 | tags=25%, list=24%, signal=32% |
| 1015 | RIBAVIRIN\_RSV\_UP |  | 22 | 0.37 | 0.90 | 0.642 | 0.773 | 1.000 | 275 | tags=9%, list=1%, signal=9% |
| 1016 | P53\_BRCA1\_UP |  | 31 | 0.34 | 0.90 | 0.646 | 0.773 | 1.000 | 1887 | tags=13%, list=9%, signal=14% |
| 1017 | HG\_PROGERIA\_DN |  | 25 | 0.37 | 0.89 | 0.644 | 0.773 | 1.000 | 335 | tags=8%, list=2%, signal=8% |
| 1018 | MOOTHA\_VOXPHOS |  | 77 | 0.31 | 0.89 | 0.674 | 0.776 | 1.000 | 5235 | tags=39%, list=25%, signal=52% |
| 1019 | CMV\_UV-CMV\_COMMON\_HCMV\_6HRS\_UP |  | 20 | 0.38 | 0.89 | 0.635 | 0.777 | 1.000 | 3282 | tags=25%, list=16%, signal=30% |
| 1020 | NFKBPATHWAY |  | 23 | 0.36 | 0.89 | 0.642 | 0.779 | 1.000 | 2069 | tags=13%, list=10%, signal=14% |
| 1021 | OXSTRESS\_RPE\_H2O2TBH\_DN |  | 31 | 0.34 | 0.89 | 0.645 | 0.782 | 1.000 | 3150 | tags=26%, list=15%, signal=30% |
| 1022 | TPA\_RESIST\_EARLY\_DN |  | 74 | 0.31 | 0.89 | 0.705 | 0.787 | 1.000 | 3995 | tags=22%, list=19%, signal=27% |
| 1023 | NAKAJIMA\_MCS\_UP |  | 92 | 0.31 | 0.88 | 0.719 | 0.786 | 1.000 | 442 | tags=4%, list=2%, signal=4% |
| 1024 | NAB\_LUNG\_DN |  | 54 | 0.32 | 0.88 | 0.706 | 0.786 | 1.000 | 3292 | tags=19%, list=16%, signal=22% |
| 1025 | CERAMIDEPATHWAY |  | 22 | 0.37 | 0.88 | 0.634 | 0.785 | 1.000 | 5387 | tags=32%, list=26%, signal=43% |
| 1026 | DER\_IFNG\_UP |  | 62 | 0.32 | 0.88 | 0.691 | 0.785 | 1.000 | 4972 | tags=27%, list=24%, signal=36% |
| 1027 | PROPANOATE\_METABOLISM |  | 31 | 0.34 | 0.88 | 0.649 | 0.787 | 1.000 | 4736 | tags=29%, list=23%, signal=38% |
| 1028 | ET743\_HELA\_DN |  | 15 | 0.39 | 0.88 | 0.642 | 0.787 | 1.000 | 5881 | tags=53%, list=29%, signal=75% |
| 1029 | 5FU\_RESIST\_GASTRIC\_UP |  | 21 | 0.37 | 0.88 | 0.675 | 0.791 | 1.000 | 2433 | tags=29%, list=12%, signal=32% |
| 1030 | HASLINGER\_B\_CLL\_17P13 |  | 16 | 0.38 | 0.88 | 0.662 | 0.794 | 1.000 | 896 | tags=13%, list=4%, signal=13% |
| 1031 | CMV\_HCMV\_TIMECOURSE\_1HR\_DN |  | 38 | 0.33 | 0.88 | 0.665 | 0.795 | 1.000 | 5552 | tags=34%, list=27%, signal=47% |
| 1032 | AS3\_FIBRO\_C2 |  | 31 | 0.34 | 0.88 | 0.649 | 0.795 | 1.000 | 3150 | tags=26%, list=15%, signal=30% |
| 1033 | TNFALPHA\_30MIN\_UP |  | 42 | 0.33 | 0.88 | 0.678 | 0.794 | 1.000 | 3283 | tags=24%, list=16%, signal=28% |
| 1034 | HBX\_HCC\_UP |  | 16 | 0.38 | 0.88 | 0.656 | 0.794 | 1.000 | 5663 | tags=38%, list=27%, signal=52% |
| 1035 | IL7PATHWAY |  | 16 | 0.38 | 0.88 | 0.665 | 0.793 | 1.000 | 8892 | tags=63%, list=43%, signal=110% |
| 1036 | AS3\_FIBRO\_C1 |  | 31 | 0.34 | 0.87 | 0.670 | 0.796 | 1.000 | 3150 | tags=26%, list=15%, signal=30% |
| 1037 | NKCELLSPATHWAY |  | 18 | 0.37 | 0.87 | 0.652 | 0.796 | 1.000 | 5723 | tags=39%, list=28%, signal=54% |
| 1038 | REFRACTORY\_GASTRIC\_UP |  | 87 | 0.30 | 0.87 | 0.724 | 0.799 | 1.000 | 5990 | tags=37%, list=29%, signal=52% |
| 1039 | BRCA2\_BRCA1\_UP |  | 48 | 0.32 | 0.87 | 0.710 | 0.803 | 1.000 | 3331 | tags=19%, list=16%, signal=22% |
| 1040 | IL2RBPATHWAY |  | 34 | 0.33 | 0.87 | 0.696 | 0.803 | 1.000 | 6440 | tags=44%, list=31%, signal=64% |
| 1041 | EPOPATHWAY |  | 19 | 0.36 | 0.86 | 0.672 | 0.807 | 1.000 | 4361 | tags=42%, list=21%, signal=53% |
| 1042 | UV\_UNIQUE\_FIBRO\_UP |  | 22 | 0.36 | 0.86 | 0.683 | 0.807 | 1.000 | 3861 | tags=27%, list=19%, signal=34% |
| 1043 | BRENTANI\_DNA\_METHYLATION\_AND\_MODIFICATION |  | 23 | 0.35 | 0.86 | 0.659 | 0.807 | 1.000 | 3210 | tags=22%, list=16%, signal=26% |
| 1044 | ZHAN\_MM\_CD138\_CD1\_VS\_REST |  | 44 | 0.32 | 0.86 | 0.711 | 0.809 | 1.000 | 1181 | tags=9%, list=6%, signal=10% |
| 1045 | INTEGRINPATHWAY |  | 34 | 0.33 | 0.86 | 0.699 | 0.810 | 1.000 | 3707 | tags=32%, list=18%, signal=39% |
| 1046 | CMV\_HCMV\_TIMECOURSE\_24HRS\_UP |  | 72 | 0.30 | 0.86 | 0.750 | 0.814 | 1.000 | 5590 | tags=29%, list=27%, signal=40% |
| 1047 | IDX\_TSA\_UP\_CLUSTER6 |  | 163 | 0.29 | 0.86 | 0.809 | 0.815 | 1.000 | 4679 | tags=28%, list=23%, signal=36% |
| 1048 | IL2PATHWAY |  | 22 | 0.35 | 0.86 | 0.684 | 0.816 | 1.000 | 4239 | tags=32%, list=21%, signal=40% |
| 1049 | TESTIS\_EXPRESSED\_GENES |  | 61 | 0.31 | 0.85 | 0.731 | 0.816 | 1.000 | 5081 | tags=26%, list=25%, signal=35% |
| 1050 | BRCA1\_OVEREXP\_DN |  | 110 | 0.29 | 0.85 | 0.774 | 0.817 | 1.000 | 3840 | tags=26%, list=19%, signal=32% |
| 1051 | ADIP\_VS\_PREADIP\_DN |  | 37 | 0.32 | 0.85 | 0.697 | 0.818 | 1.000 | 4537 | tags=30%, list=22%, signal=38% |
| 1052 | MARCINIAK\_CHOP\_DIFF |  | 21 | 0.34 | 0.85 | 0.679 | 0.823 | 1.000 | 4760 | tags=43%, list=23%, signal=56% |
| 1053 | GPCRS\_CLASS\_B\_SECRETIN\_LIKE |  | 23 | 0.34 | 0.85 | 0.704 | 0.826 | 1.000 | 6784 | tags=35%, list=33%, signal=52% |
| 1054 | HIPPOCAMPUS\_DEVELOPMENT\_NEONATAL |  | 27 | 0.33 | 0.85 | 0.716 | 0.826 | 1.000 | 3049 | tags=30%, list=15%, signal=35% |
| 1055 | UNDERHILL\_PROLIFERATION |  | 18 | 0.36 | 0.84 | 0.692 | 0.829 | 1.000 | 3621 | tags=28%, list=18%, signal=34% |
| 1056 | POMEROY\_DESMOPLASIC\_VS\_CLASSIC\_MD\_DN |  | 41 | 0.31 | 0.84 | 0.724 | 0.829 | 1.000 | 3619 | tags=27%, list=18%, signal=32% |
| 1057 | ADIP\_VS\_FIBRO\_DN |  | 27 | 0.34 | 0.84 | 0.732 | 0.828 | 1.000 | 4769 | tags=33%, list=23%, signal=43% |
| 1058 | TALL1PATHWAY |  | 15 | 0.38 | 0.84 | 0.704 | 0.828 | 1.000 | 8080 | tags=53%, list=39%, signal=88% |
| 1059 | GPCRDB\_CLASS\_B\_SECRETIN\_LIKE |  | 23 | 0.34 | 0.84 | 0.706 | 0.830 | 1.000 | 6784 | tags=35%, list=33%, signal=52% |
| 1060 | NGFPATHWAY |  | 19 | 0.35 | 0.84 | 0.701 | 0.831 | 1.000 | 6462 | tags=53%, list=31%, signal=77% |
| 1061 | IL6PATHWAY |  | 21 | 0.34 | 0.83 | 0.726 | 0.838 | 1.000 | 5225 | tags=48%, list=25%, signal=64% |
| 1062 | HSIAO\_LIVER\_SPECIFIC\_GENES |  | 240 | 0.27 | 0.83 | 0.888 | 0.838 | 1.000 | 4885 | tags=20%, list=24%, signal=26% |
| 1063 | LEE\_MYC\_E2F1\_DN |  | 61 | 0.30 | 0.83 | 0.758 | 0.837 | 1.000 | 2729 | tags=15%, list=13%, signal=17% |
| 1064 | PENG\_GLUTAMINE\_DN |  | 248 | 0.28 | 0.83 | 0.897 | 0.841 | 1.000 | 4844 | tags=32%, list=24%, signal=42% |
| 1065 | MYC\_TARGETS |  | 40 | 0.31 | 0.83 | 0.751 | 0.842 | 1.000 | 2991 | tags=25%, list=15%, signal=29% |
| 1066 | ZHAN\_MMPC\_EARLYVS |  | 48 | 0.30 | 0.83 | 0.758 | 0.843 | 1.000 | 3899 | tags=25%, list=19%, signal=31% |
| 1067 | CHEN\_HOXA5\_TARGETS\_DN |  | 47 | 0.30 | 0.83 | 0.767 | 0.845 | 1.000 | 2634 | tags=15%, list=13%, signal=17% |
| 1068 | ANDROGEN\_GENES |  | 52 | 0.30 | 0.83 | 0.749 | 0.844 | 1.000 | 3465 | tags=19%, list=17%, signal=23% |
| 1069 | ROSS\_MLL\_FUSION |  | 83 | 0.29 | 0.83 | 0.805 | 0.843 | 1.000 | 3982 | tags=23%, list=19%, signal=28% |
| 1070 | AKTPATHWAY |  | 17 | 0.35 | 0.82 | 0.713 | 0.844 | 1.000 | 4328 | tags=29%, list=21%, signal=37% |
| 1071 | GOLUB\_ALL\_VS\_AML\_UP |  | 19 | 0.35 | 0.82 | 0.723 | 0.844 | 1.000 | 7022 | tags=37%, list=34%, signal=56% |
| 1072 | PENTOSE\_PHOSPHATE\_PATHWAY |  | 23 | 0.33 | 0.82 | 0.756 | 0.848 | 1.000 | 2412 | tags=17%, list=12%, signal=20% |
| 1073 | ASTIER\_FN\_DIFF |  | 61 | 0.29 | 0.82 | 0.791 | 0.848 | 1.000 | 3030 | tags=21%, list=15%, signal=25% |
| 1074 | IDX\_TSA\_UP\_CLUSTER3 |  | 88 | 0.28 | 0.82 | 0.807 | 0.847 | 1.000 | 3726 | tags=24%, list=18%, signal=29% |
| 1075 | GH\_AUTOCRINE\_UP |  | 219 | 0.27 | 0.82 | 0.908 | 0.852 | 1.000 | 5845 | tags=29%, list=28%, signal=40% |
| 1076 | MOUSE\_TISSUE\_TESTIS |  | 34 | 0.31 | 0.82 | 0.749 | 0.851 | 1.000 | 3668 | tags=21%, list=18%, signal=25% |
| 1077 | TNFALPHA\_TGZ\_ADIP\_DN |  | 27 | 0.32 | 0.81 | 0.747 | 0.853 | 1.000 | 4420 | tags=30%, list=21%, signal=38% |
| 1078 | SANSOM\_APC\_5\_DN |  | 327 | 0.27 | 0.81 | 0.926 | 0.852 | 1.000 | 2925 | tags=13%, list=14%, signal=15% |
| 1079 | PASSERINI\_EM |  | 35 | 0.31 | 0.81 | 0.760 | 0.857 | 1.000 | 4202 | tags=26%, list=20%, signal=32% |
| 1080 | OLDAGE\_DN |  | 47 | 0.30 | 0.81 | 0.789 | 0.856 | 1.000 | 4414 | tags=30%, list=21%, signal=38% |
| 1081 | NADLER\_OBESITY\_HYPERGLYCEMIA |  | 42 | 0.30 | 0.81 | 0.768 | 0.857 | 1.000 | 7000 | tags=38%, list=34%, signal=58% |
| 1082 | BCNU\_GLIOMA\_MGMT\_48HRS\_UP |  | 18 | 0.34 | 0.81 | 0.730 | 0.861 | 1.000 | 3796 | tags=28%, list=18%, signal=34% |
| 1083 | ZHAN\_TONSIL\_PCBC |  | 43 | 0.30 | 0.80 | 0.791 | 0.861 | 1.000 | 3899 | tags=26%, list=19%, signal=31% |
| 1084 | CARM\_ERPATHWAY |  | 26 | 0.32 | 0.80 | 0.757 | 0.861 | 1.000 | 4125 | tags=23%, list=20%, signal=29% |
| 1085 | CELL\_CYCLE\_KEGG |  | 86 | 0.28 | 0.80 | 0.830 | 0.862 | 1.000 | 5037 | tags=30%, list=24%, signal=40% |
| 1086 | XU\_ATRA\_UP |  | 15 | 0.35 | 0.80 | 0.755 | 0.866 | 1.000 | 5737 | tags=40%, list=28%, signal=55% |
| 1087 | VANTVEER\_BREAST\_OUTCOME\_GOOD\_VS\_POOR\_UP |  | 26 | 0.32 | 0.80 | 0.763 | 0.866 | 1.000 | 3571 | tags=19%, list=17%, signal=23% |
| 1088 | HADDAD\_HSC\_CD7\_UP |  | 64 | 0.28 | 0.80 | 0.797 | 0.866 | 1.000 | 4349 | tags=17%, list=21%, signal=22% |
| 1089 | ADIP\_HUMAN\_DN |  | 27 | 0.32 | 0.79 | 0.772 | 0.870 | 1.000 | 1591 | tags=11%, list=8%, signal=12% |
| 1090 | HADDAD\_CD45CD7\_PLUS\_VS\_MINUS\_UP |  | 64 | 0.28 | 0.79 | 0.820 | 0.874 | 1.000 | 4349 | tags=17%, list=21%, signal=22% |
| 1091 | HDACI\_COLON\_TSA48HRS\_UP |  | 42 | 0.29 | 0.79 | 0.805 | 0.875 | 1.000 | 5516 | tags=31%, list=27%, signal=42% |
| 1092 | TH1TH2PATHWAY |  | 17 | 0.34 | 0.79 | 0.754 | 0.874 | 1.000 | 6221 | tags=29%, list=30%, signal=42% |
| 1093 | ST\_B\_CELL\_ANTIGEN\_RECEPTOR |  | 39 | 0.30 | 0.79 | 0.791 | 0.874 | 1.000 | 4337 | tags=23%, list=21%, signal=29% |
| 1094 | GRANDVAUX\_IFN\_NOT\_IRF3\_UP |  | 15 | 0.34 | 0.79 | 0.762 | 0.873 | 1.000 | 4141 | tags=27%, list=20%, signal=33% |
| 1095 | SPPAPATHWAY |  | 20 | 0.33 | 0.79 | 0.769 | 0.873 | 1.000 | 3154 | tags=25%, list=15%, signal=29% |
| 1096 | LAL\_KO\_6MO\_UP |  | 61 | 0.28 | 0.79 | 0.821 | 0.875 | 1.000 | 1922 | tags=13%, list=9%, signal=14% |
| 1097 | RORIE\_ES\_PNET\_DN |  | 27 | 0.31 | 0.78 | 0.796 | 0.875 | 1.000 | 7342 | tags=33%, list=36%, signal=52% |
| 1098 | CMV\_HCMV\_TIMECOURSE\_12HRS\_UP |  | 26 | 0.31 | 0.78 | 0.784 | 0.875 | 1.000 | 1239 | tags=12%, list=6%, signal=12% |
| 1099 | ABBUD\_LIF\_UP |  | 40 | 0.29 | 0.78 | 0.812 | 0.875 | 1.000 | 4862 | tags=28%, list=24%, signal=36% |
| 1100 | CPR\_NULL\_LIVER\_UP |  | 33 | 0.30 | 0.78 | 0.798 | 0.875 | 1.000 | 4713 | tags=39%, list=23%, signal=51% |
| 1101 | GOLDRATH\_CYTOLYTIC |  | 17 | 0.33 | 0.78 | 0.771 | 0.878 | 1.000 | 641 | tags=6%, list=3%, signal=6% |
| 1102 | ZHAN\_MULTIPLE\_MYELOMA\_SUBCLASSES\_DIFF |  | 30 | 0.30 | 0.78 | 0.808 | 0.881 | 1.000 | 3954 | tags=33%, list=19%, signal=41% |
| 1103 | S1P\_SIGNALING |  | 24 | 0.31 | 0.77 | 0.790 | 0.883 | 1.000 | 3715 | tags=25%, list=18%, signal=30% |
| 1104 | KLEIN\_PEL\_DN |  | 58 | 0.28 | 0.77 | 0.826 | 0.883 | 1.000 | 1715 | tags=12%, list=8%, signal=13% |
| 1105 | PYRUVATE\_METABOLISM |  | 37 | 0.29 | 0.77 | 0.819 | 0.884 | 1.000 | 4736 | tags=27%, list=23%, signal=35% |
| 1106 | ASTIER\_BCELL |  | 60 | 0.28 | 0.77 | 0.847 | 0.883 | 1.000 | 3030 | tags=20%, list=15%, signal=23% |
| 1107 | OXSTRESS\_RPE\_H2O2HNE\_DN |  | 31 | 0.30 | 0.77 | 0.808 | 0.885 | 1.000 | 4905 | tags=39%, list=24%, signal=51% |
| 1108 | ZHAN\_MULTIPLE\_MYELOMA\_VS\_NORMAL\_UP |  | 62 | 0.27 | 0.77 | 0.861 | 0.885 | 1.000 | 3094 | tags=21%, list=15%, signal=25% |
| 1109 | LEE\_E2F1\_DN |  | 62 | 0.28 | 0.77 | 0.832 | 0.886 | 1.000 | 2729 | tags=15%, list=13%, signal=17% |
| 1110 | CMV\_HCMV\_6HRS\_UP |  | 25 | 0.31 | 0.76 | 0.810 | 0.888 | 1.000 | 3405 | tags=24%, list=17%, signal=29% |
| 1111 | FRASOR\_ER\_UP |  | 30 | 0.30 | 0.76 | 0.797 | 0.887 | 1.000 | 3342 | tags=20%, list=16%, signal=24% |
| 1112 | DER\_IFNA\_UP |  | 66 | 0.27 | 0.76 | 0.849 | 0.887 | 1.000 | 5619 | tags=32%, list=27%, signal=44% |
| 1113 | GNATENKO\_PLATELET |  | 44 | 0.28 | 0.76 | 0.827 | 0.892 | 1.000 | 3007 | tags=20%, list=15%, signal=24% |
| 1114 | IFNA\_HCMV\_6HRS\_UP |  | 53 | 0.28 | 0.76 | 0.841 | 0.893 | 1.000 | 4753 | tags=26%, list=23%, signal=34% |
| 1115 | GNATENKO\_PLATELET\_UP |  | 44 | 0.28 | 0.75 | 0.856 | 0.895 | 1.000 | 3007 | tags=20%, list=15%, signal=24% |
| 1116 | STATIN\_PATHWAY\_PHARMGKB |  | 17 | 0.32 | 0.75 | 0.800 | 0.895 | 1.000 | 8130 | tags=47%, list=39%, signal=78% |
| 1117 | SA\_TRKA\_RECEPTOR |  | 16 | 0.33 | 0.75 | 0.798 | 0.895 | 1.000 | 991 | tags=13%, list=5%, signal=13% |
| 1118 | TPA\_SKIN\_UP |  | 20 | 0.31 | 0.75 | 0.794 | 0.895 | 1.000 | 3097 | tags=25%, list=15%, signal=29% |
| 1119 | MATRIX\_METALLOPROTEINASES |  | 30 | 0.29 | 0.75 | 0.815 | 0.894 | 1.000 | 1463 | tags=10%, list=7%, signal=11% |
| 1120 | PROSTAGLANDIN\_AND\_LEUKOTRIENE\_METABOLISM |  | 33 | 0.29 | 0.75 | 0.837 | 0.896 | 1.000 | 3422 | tags=18%, list=17%, signal=22% |
| 1121 | TRNA\_SYNTHETASES |  | 19 | 0.31 | 0.74 | 0.803 | 0.901 | 1.000 | 5532 | tags=42%, list=27%, signal=58% |
| 1122 | DFOSB\_BRAIN\_8WKS\_UP |  | 40 | 0.27 | 0.74 | 0.851 | 0.901 | 1.000 | 5493 | tags=30%, list=27%, signal=41% |
| 1123 | TNFALPHA\_ADIP\_DN |  | 57 | 0.26 | 0.74 | 0.867 | 0.901 | 1.000 | 4420 | tags=25%, list=21%, signal=31% |
| 1124 | HEARTFAILURE\_VENTRICLE\_DN |  | 67 | 0.26 | 0.74 | 0.873 | 0.903 | 1.000 | 6024 | tags=36%, list=29%, signal=50% |
| 1125 | HOFMANN\_MANTEL\_LYMPHOMA\_VS\_LYMPH\_NODES\_DN |  | 39 | 0.28 | 0.74 | 0.853 | 0.903 | 1.000 | 7016 | tags=38%, list=34%, signal=58% |
| 1126 | IDX\_TSA\_UP\_CLUSTER4 |  | 40 | 0.28 | 0.74 | 0.853 | 0.903 | 1.000 | 4195 | tags=23%, list=20%, signal=28% |
| 1127 | AGED\_MOUSE\_CEREBELLUM\_UP |  | 60 | 0.26 | 0.74 | 0.879 | 0.903 | 1.000 | 3197 | tags=22%, list=16%, signal=26% |
| 1128 | LEE\_CIP\_DN |  | 65 | 0.26 | 0.73 | 0.881 | 0.905 | 1.000 | 2729 | tags=12%, list=13%, signal=14% |
| 1129 | CALRES\_MOUSE\_DN |  | 39 | 0.28 | 0.73 | 0.842 | 0.904 | 1.000 | 4819 | tags=33%, list=23%, signal=43% |
| 1130 | CMV\_IE86\_UP |  | 49 | 0.26 | 0.73 | 0.876 | 0.909 | 1.000 | 4893 | tags=35%, list=24%, signal=45% |
| 1131 | INFLAMMATORY\_RESPONSE\_PATHWAY |  | 29 | 0.29 | 0.73 | 0.861 | 0.909 | 1.000 | 7744 | tags=38%, list=38%, signal=61% |
| 1132 | PEART\_HISTONE\_UP |  | 52 | 0.26 | 0.73 | 0.879 | 0.909 | 1.000 | 3465 | tags=23%, list=17%, signal=28% |
| 1133 | ST\_GA12\_PATHWAY |  | 21 | 0.30 | 0.72 | 0.834 | 0.910 | 1.000 | 4074 | tags=29%, list=20%, signal=36% |
| 1134 | GOLUB\_ALL\_VS\_AML\_DN |  | 16 | 0.32 | 0.72 | 0.857 | 0.914 | 1.000 | 5067 | tags=38%, list=25%, signal=50% |
| 1135 | CELL\_CYCLE\_CHECKPOINT |  | 24 | 0.29 | 0.72 | 0.851 | 0.914 | 1.000 | 6048 | tags=38%, list=29%, signal=53% |
| 1136 | BYSTROM\_IL5\_UP |  | 41 | 0.27 | 0.72 | 0.865 | 0.914 | 1.000 | 4886 | tags=27%, list=24%, signal=35% |
| 1137 | DAC\_FIBRO\_UP |  | 17 | 0.30 | 0.71 | 0.829 | 0.920 | 1.000 | 286 | tags=6%, list=1%, signal=6% |
| 1138 | PITX2PATHWAY |  | 16 | 0.31 | 0.71 | 0.832 | 0.921 | 1.000 | 4652 | tags=38%, list=23%, signal=48% |
| 1139 | GALACTOSE\_METABOLISM |  | 23 | 0.29 | 0.71 | 0.887 | 0.921 | 1.000 | 6513 | tags=43%, list=32%, signal=64% |
| 1140 | GENOTOXINS\_ALL\_24HRS\_REG |  | 28 | 0.28 | 0.71 | 0.877 | 0.920 | 1.000 | 3838 | tags=25%, list=19%, signal=31% |
| 1141 | IL12PATHWAY |  | 20 | 0.30 | 0.70 | 0.859 | 0.925 | 1.000 | 2011 | tags=15%, list=10%, signal=17% |
| 1142 | FALT\_BCLL\_UP |  | 45 | 0.26 | 0.70 | 0.891 | 0.925 | 1.000 | 4656 | tags=24%, list=23%, signal=32% |
| 1143 | GLUTAMATE\_METABOLISM |  | 24 | 0.28 | 0.70 | 0.857 | 0.926 | 1.000 | 3828 | tags=17%, list=19%, signal=20% |
| 1144 | GH\_EXOGENOUS\_MIDDLE\_UP |  | 125 | 0.23 | 0.69 | 0.964 | 0.931 | 1.000 | 6716 | tags=29%, list=33%, signal=42% |
| 1145 | NUMATA\_G\_CSF\_DIFF |  | 18 | 0.29 | 0.69 | 0.863 | 0.932 | 1.000 | 6193 | tags=44%, list=30%, signal=63% |
| 1146 | TPA\_RESIST\_EARLY\_UP |  | 30 | 0.27 | 0.69 | 0.882 | 0.931 | 1.000 | 6476 | tags=33%, list=31%, signal=49% |
| 1147 | TARTE\_BCELL |  | 38 | 0.26 | 0.69 | 0.907 | 0.931 | 1.000 | 5737 | tags=32%, list=28%, signal=44% |
| 1148 | FATTY\_ACID\_METABOLISM |  | 82 | 0.23 | 0.67 | 0.960 | 0.940 | 1.000 | 5038 | tags=27%, list=24%, signal=35% |
| 1149 | PARK\_RARALPHA\_UP |  | 38 | 0.26 | 0.67 | 0.922 | 0.940 | 1.000 | 7656 | tags=47%, list=37%, signal=75% |
| 1150 | STRESS\_GENOTOXIC\_SPECIFIC\_UP |  | 34 | 0.26 | 0.67 | 0.913 | 0.945 | 1.000 | 5067 | tags=26%, list=25%, signal=35% |
| 1151 | CXCR4PATHWAY |  | 24 | 0.27 | 0.66 | 0.895 | 0.948 | 1.000 | 4361 | tags=21%, list=21%, signal=26% |
| 1152 | RARRXRPATHWAY |  | 15 | 0.29 | 0.65 | 0.906 | 0.953 | 1.000 | 5249 | tags=27%, list=25%, signal=36% |
| 1153 | RNA\_TRANSCRIPTION\_REACTOME |  | 37 | 0.25 | 0.65 | 0.943 | 0.955 | 1.000 | 6441 | tags=32%, list=31%, signal=47% |
| 1154 | FETAL\_LIVER\_VS\_ADULT\_LIVER\_GNF2 |  | 70 | 0.23 | 0.64 | 0.958 | 0.960 | 1.000 | 1153 | tags=7%, list=6%, signal=8% |
| 1155 | ERM\_KO\_SERTOLI\_DN |  | 18 | 0.27 | 0.63 | 0.909 | 0.960 | 1.000 | 7958 | tags=39%, list=39%, signal=63% |
| 1156 | RASPATHWAY |  | 22 | 0.26 | 0.63 | 0.920 | 0.964 | 1.000 | 3367 | tags=23%, list=16%, signal=27% |
| 1157 | TRYPTOPHAN\_METABOLISM |  | 54 | 0.22 | 0.61 | 0.969 | 0.969 | 1.000 | 7879 | tags=50%, list=38%, signal=81% |
| 1158 | GLYCINE\_SERINE\_AND\_THREONINE\_METABOLISM |  | 35 | 0.23 | 0.61 | 0.957 | 0.969 | 1.000 | 4361 | tags=23%, list=21%, signal=29% |
| 1159 | CTLA4PATHWAY |  | 18 | 0.26 | 0.61 | 0.928 | 0.969 | 1.000 | 3407 | tags=17%, list=17%, signal=20% |
| 1160 | AGUIRRE\_PANCREAS\_CHR7 |  | 48 | 0.23 | 0.61 | 0.962 | 0.971 | 1.000 | 5865 | tags=35%, list=28%, signal=49% |
| 1161 | P53PATHWAY |  | 16 | 0.26 | 0.60 | 0.928 | 0.971 | 1.000 | 391 | tags=6%, list=2%, signal=6% |
| 1162 | ANDROGEN\_AND\_ESTROGEN\_METABOLISM |  | 24 | 0.24 | 0.60 | 0.958 | 0.973 | 1.000 | 2267 | tags=8%, list=11%, signal=9% |
| 1163 | SMALL\_LIGAND\_GPCRS |  | 17 | 0.26 | 0.60 | 0.941 | 0.973 | 1.000 | 453 | tags=6%, list=2%, signal=6% |
| 1164 | ELECTRON\_TRANSPORT |  | 74 | 0.20 | 0.58 | 0.990 | 0.979 | 1.000 | 5259 | tags=22%, list=26%, signal=29% |
| 1165 | LEE\_MYC\_TGFA\_DN |  | 63 | 0.20 | 0.56 | 0.981 | 0.983 | 1.000 | 2475 | tags=8%, list=12%, signal=9% |
| 1166 | GHPATHWAY |  | 27 | 0.22 | 0.56 | 0.974 | 0.983 | 1.000 | 4361 | tags=26%, list=21%, signal=33% |
| 1167 | UVB\_NHEK4\_24HRS\_DN |  | 18 | 0.23 | 0.56 | 0.951 | 0.983 | 1.000 | 9166 | tags=56%, list=44%, signal=100% |
| 1168 | TSA\_CD4\_DN |  | 18 | 0.23 | 0.54 | 0.960 | 0.988 | 1.000 | 5727 | tags=33%, list=28%, signal=46% |
| 1169 | WANG\_HOXA9\_VS\_MEIS1\_DN |  | 23 | 0.22 | 0.53 | 0.971 | 0.988 | 1.000 | 1441 | tags=9%, list=7%, signal=9% |
| 1170 | RELAPATHWAY |  | 16 | 0.21 | 0.47 | 0.988 | 0.996 | 1.000 | 9289 | tags=63%, list=45%, signal=114% |
Table: Gene sets enriched in phenotype **na**[plain text format]****

  
