# Supplementary material for: The rapamycin-regulated gene expression signature determines prognosis for breast cancer
Source: Mol Cancer. 2009 Sep 24;8:75. doi: 10.1186/1476-4598-8-75 (PMC2761377; doi:10.1186/1476-4598-8-75)
Supplement: Additional file 3 — Gene set enrichment analysis of in vivo data, treatment series. The data provided represent the treatment series of GSEA. This compressed file contains "Treatment" shortcut file and "GSEA_treatment" folder. Clicking on "Treatment" shortcut opens the index file providing access to analysis files contained in the "GSEA_treatment" folder. [file 1476-4598-8-75-S3.zip › GSEA_treatment/4NQO_UNIQUE_FIBRO_UP.html]

Details for gene set 4NQO\_UNIQUE\_FIBRO\_UP[GSEA]

|  || Dataset | gsea\_treatment\_collapsed |
| Phenotype | NoPhenotypeAvailable |
| Upregulated in class | na\_neg |
| GeneSet | 4NQO\_UNIQUE\_FIBRO\_UP |
| Enrichment Score (ES) | -0.3142026 |
| Normalized Enrichment Score (NES) | -1.1932431 |
| Nominal p-value | 0.19402985 |
| FDR q-value | 0.30886513 |
| FWER p-Value | 1.0 |
Table: GSEA Results Summary

  

Fig 1: Enrichment plot: 4NQO\_UNIQUE\_FIBRO\_UP      
 Profile of the Running ES Score & Positions of GeneSet Members on the Rank Ordered List

  

| PROBE | GENE SYMBOL | GENE\_TITLE | RANK IN GENE LIST | RANK METRIC SCORE | RUNNING ES | CORE ENRICHMENT || 1 | ITGB5 |  |  | 1080 | 0.323 | 0.0408 | No |
| 2 | IFNGR2 |  |  | 1158 | 0.315 | 0.1279 | No |
| 3 | NET1 |  |  | 1428 | 0.291 | 0.1989 | No |
| 4 | ARPC3 |  |  | 3335 | 0.199 | 0.1636 | No |
| 5 | ESD |  |  | 3445 | 0.196 | 0.2148 | No |
| 6 | MT1H |  |  | 3910 | 0.182 | 0.2449 | No |
| 7 | ANKRD11 |  |  | 4807 | 0.159 | 0.2473 | No |
| 8 | DSCAM |  |  | 5533 | 0.144 | 0.2535 | No |
| 9 | PPA1 |  |  | 9532 | 0.079 | 0.0822 | No |
| 10 | ID1 |  |  | 10922 | 0.061 | 0.0322 | No |
| 11 | SSR3 |  |  | 10941 | 0.061 | 0.0488 | No |
| 12 | POLR2F |  |  | 12407 | 0.041 | -0.0104 | No |
| 13 | CD36 |  |  | 12917 | 0.035 | -0.0250 | No |
| 14 | RHOBTB3 |  |  | 14685 | 0.011 | -0.1078 | No |
| 15 | HSPB8 |  |  | 15924 | -0.009 | -0.1653 | No |
| 16 | ATOX1 |  |  | 16284 | -0.015 | -0.1785 | No |
| 17 | PDGFRL |  |  | 19079 | -0.088 | -0.2889 | Yes |
| 18 | RABGGTB |  |  | 19123 | -0.090 | -0.2651 | Yes |
| 19 | RAGE |  |  | 19675 | -0.118 | -0.2577 | Yes |
| 20 | PCK2 |  |  | 20288 | -0.191 | -0.2322 | Yes |
| 21 | CYP51A1 |  |  | 20531 | -0.354 | -0.1419 | Yes |
| 22 | FBLN2 |  |  | 20574 | -0.504 | 0.0015 | Yes |
Table: GSEA details [plain text format]

  

Fig 2: 4NQO\_UNIQUE\_FIBRO\_UP: Random ES distribution      
 Gene set null distribution of ES for **4NQO\_UNIQUE\_FIBRO\_UP**

  
