# Supplementary material for: The rapamycin-regulated gene expression signature determines prognosis for breast cancer
Source: Mol Cancer. 2009 Sep 24;8:75. doi: 10.1186/1476-4598-8-75 (PMC2761377; doi:10.1186/1476-4598-8-75)
Supplement: Additional file 3 — Gene set enrichment analysis of in vivo data, treatment series. The data provided represent the treatment series of GSEA. This compressed file contains "Treatment" shortcut file and "GSEA_treatment" folder. Clicking on "Treatment" shortcut opens the index file providing access to analysis files contained in the "GSEA_treatment" folder. [file 1476-4598-8-75-S3.zip › GSEA_treatment/5FU_RESIST_GASTRIC_DN.html]

Details for gene set 5FU\_RESIST\_GASTRIC\_DN[GSEA]

|  || Dataset | gsea\_treatment\_collapsed |
| Phenotype | NoPhenotypeAvailable |
| Upregulated in class | na\_neg |
| GeneSet | 5FU\_RESIST\_GASTRIC\_DN |
| Enrichment Score (ES) | -0.6476669 |
| Normalized Enrichment Score (NES) | -2.1987662 |
| Nominal p-value | 0.0 |
| FDR q-value | 0.003294666 |
| FWER p-Value | 0.011 |
Table: GSEA Results Summary

  

Fig 1: Enrichment plot: 5FU\_RESIST\_GASTRIC\_DN      
 Profile of the Running ES Score & Positions of GeneSet Members on the Rank Ordered List

  

| PROBE | GENE SYMBOL | GENE\_TITLE | RANK IN GENE LIST | RANK METRIC SCORE | RUNNING ES | CORE ENRICHMENT || 1 | NFYA |  |  | 1750 | 0.269 | -0.0148 | No |
| 2 | BACE2 |  |  | 2815 | 0.218 | -0.0097 | No |
| 3 | ITGB4 |  |  | 3396 | 0.197 | 0.0136 | No |
| 4 | MST1R |  |  | 4905 | 0.157 | -0.0187 | No |
| 5 | LAMC2 |  |  | 5378 | 0.147 | -0.0032 | No |
| 6 | FLNB |  |  | 8686 | 0.091 | -0.1400 | No |
| 7 | OPLAH |  |  | 11121 | 0.058 | -0.2431 | No |
| 8 | CALB2 |  |  | 12647 | 0.038 | -0.3071 | No |
| 9 | SLC2A1 |  |  | 13804 | 0.023 | -0.3572 | No |
| 10 | SLC43A1 |  |  | 16728 | -0.024 | -0.4930 | No |
| 11 | LDLR |  |  | 17099 | -0.031 | -0.5030 | No |
| 12 | GOT1 |  |  | 20079 | -0.152 | -0.6079 | Yes |
| 13 | PCK2 |  |  | 20288 | -0.191 | -0.5681 | Yes |
| 14 | S100A14 |  |  | 20550 | -0.414 | -0.4729 | Yes |
| 15 | S100P |  |  | 20599 | -0.897 | -0.2414 | Yes |
| 16 | DDIT4 |  |  | 20600 | -0.927 | 0.0002 | Yes |
Table: GSEA details [plain text format]

  

Fig 2: 5FU\_RESIST\_GASTRIC\_DN: Random ES distribution      
 Gene set null distribution of ES for **5FU\_RESIST\_GASTRIC\_DN**

  
