# Supplementary material for: The rapamycin-regulated gene expression signature determines prognosis for breast cancer
Source: Mol Cancer. 2009 Sep 24;8:75. doi: 10.1186/1476-4598-8-75 (PMC2761377; doi:10.1186/1476-4598-8-75)
Supplement: Additional file 3 — Gene set enrichment analysis of in vivo data, treatment series. The data provided represent the treatment series of GSEA. This compressed file contains "Treatment" shortcut file and "GSEA_treatment" folder. Clicking on "Treatment" shortcut opens the index file providing access to analysis files contained in the "GSEA_treatment" folder. [file 1476-4598-8-75-S3.zip › GSEA_treatment/ADIP_DIFF_CLUSTER2.html]

Details for gene set ADIP\_DIFF\_CLUSTER2[GSEA]

|  || Dataset | gsea\_treatment\_collapsed |
| Phenotype | NoPhenotypeAvailable |
| Upregulated in class | na\_neg |
| GeneSet | ADIP\_DIFF\_CLUSTER2 |
| Enrichment Score (ES) | -0.3936366 |
| Normalized Enrichment Score (NES) | -1.6671265 |
| Nominal p-value | 0.025641026 |
| FDR q-value | 0.10053706 |
| FWER p-Value | 0.558 |
Table: GSEA Results Summary

  

Fig 1: Enrichment plot: ADIP\_DIFF\_CLUSTER2      
 Profile of the Running ES Score & Positions of GeneSet Members on the Rank Ordered List

  

| PROBE | GENE SYMBOL | GENE\_TITLE | RANK IN GENE LIST | RANK METRIC SCORE | RUNNING ES | CORE ENRICHMENT || 1 | CTGF |  |  | 12 | 0.922 | 0.1346 | No |
| 2 | CYR61 |  |  | 1299 | 0.302 | 0.1165 | No |
| 3 | THBS1 |  |  | 2263 | 0.242 | 0.1051 | No |
| 4 | DNAJB6 |  |  | 2414 | 0.234 | 0.1322 | No |
| 5 | ERRFI1 |  |  | 2807 | 0.218 | 0.1450 | No |
| 6 | WEE1 |  |  | 2845 | 0.216 | 0.1750 | No |
| 7 | SERPINE1 |  |  | 3043 | 0.209 | 0.1960 | No |
| 8 | CDR2 |  |  | 3972 | 0.180 | 0.1772 | No |
| 9 | CAPN1 |  |  | 4708 | 0.162 | 0.1652 | No |
| 10 | IL6 |  |  | 5899 | 0.137 | 0.1273 | No |
| 11 | TNFRSF1B |  |  | 5948 | 0.136 | 0.1450 | No |
| 12 | CXCR7 |  |  | 7786 | 0.105 | 0.0711 | No |
| 13 | RHOJ |  |  | 8627 | 0.092 | 0.0438 | No |
| 14 | CDR2L |  |  | 8954 | 0.088 | 0.0408 | No |
| 15 | FOXC2 |  |  | 9756 | 0.076 | 0.0130 | No |
| 16 | KLF4 |  |  | 9867 | 0.075 | 0.0187 | No |
| 17 | PHLDA1 |  |  | 9954 | 0.074 | 0.0253 | No |
| 18 | ID1 |  |  | 10922 | 0.061 | -0.0129 | No |
| 19 | SEMA3C |  |  | 12655 | 0.038 | -0.0914 | No |
| 20 | TNFAIP6 |  |  | 12970 | 0.034 | -0.1017 | No |
| 21 | PPP1R15A |  |  | 13595 | 0.026 | -0.1282 | No |
| 22 | BAG3 |  |  | 13939 | 0.022 | -0.1417 | No |
| 23 | CXCL6 |  |  | 14050 | 0.020 | -0.1441 | No |
| 24 | ETS2 |  |  | 14747 | 0.010 | -0.1765 | No |
| 25 | FOSB |  |  | 15095 | 0.004 | -0.1927 | No |
| 26 | ID2 |  |  | 15550 | -0.003 | -0.2144 | No |
| 27 | PTGS2 |  |  | 16374 | -0.017 | -0.2519 | No |
| 28 | DNAJB4 |  |  | 17157 | -0.032 | -0.2853 | No |
| 29 | ZFP36 |  |  | 18077 | -0.054 | -0.3221 | No |
| 30 | DUSP1 |  |  | 18808 | -0.076 | -0.3464 | No |
| 31 | GADD45G |  |  | 19781 | -0.126 | -0.3752 | Yes |
| 32 | CEBPD |  |  | 19851 | -0.131 | -0.3593 | Yes |
| 33 | IER3 |  |  | 20158 | -0.165 | -0.3500 | Yes |
| 34 | PRSS15 |  |  | 20239 | -0.181 | -0.3273 | Yes |
| 35 | SLC20A1 |  |  | 20324 | -0.200 | -0.3021 | Yes |
| 36 | VEGF |  |  | 20367 | -0.211 | -0.2732 | Yes |
| 37 | ID3 |  |  | 20467 | -0.265 | -0.2392 | Yes |
| 38 | DNAJB1 |  |  | 20548 | -0.409 | -0.1830 | Yes |
| 39 | ADM |  |  | 20571 | -0.495 | -0.1115 | Yes |
| 40 | RGS2 |  |  | 20592 | -0.771 | 0.0006 | Yes |
Table: GSEA details [plain text format]

  

Fig 2: ADIP\_DIFF\_CLUSTER2: Random ES distribution      
 Gene set null distribution of ES for **ADIP\_DIFF\_CLUSTER2**

  
