# Supplementary material for: The rapamycin-regulated gene expression signature determines prognosis for breast cancer
Source: Mol Cancer. 2009 Sep 24;8:75. doi: 10.1186/1476-4598-8-75 (PMC2761377; doi:10.1186/1476-4598-8-75)
Supplement: Additional file 3 — Gene set enrichment analysis of in vivo data, treatment series. The data provided represent the treatment series of GSEA. This compressed file contains "Treatment" shortcut file and "GSEA_treatment" folder. Clicking on "Treatment" shortcut opens the index file providing access to analysis files contained in the "GSEA_treatment" folder. [file 1476-4598-8-75-S3.zip › GSEA_treatment/ADIP_DIFF_CLUSTER5.html]

Details for gene set ADIP\_DIFF\_CLUSTER5[GSEA]

|  || Dataset | gsea\_treatment\_collapsed |
| Phenotype | NoPhenotypeAvailable |
| Upregulated in class | na\_pos |
| GeneSet | ADIP\_DIFF\_CLUSTER5 |
| Enrichment Score (ES) | 0.625125 |
| Normalized Enrichment Score (NES) | 1.7175416 |
| Nominal p-value | 0.0010559662 |
| FDR q-value | 0.010266108 |
| FWER p-Value | 0.332 |
Table: GSEA Results Summary

  

Fig 1: Enrichment plot: ADIP\_DIFF\_CLUSTER5      
 Profile of the Running ES Score & Positions of GeneSet Members on the Rank Ordered List

  

| PROBE | GENE SYMBOL | GENE\_TITLE | RANK IN GENE LIST | RANK METRIC SCORE | RUNNING ES | CORE ENRICHMENT || 1 | S100A8 |  |  | 42 | 0.695 | 0.0797 | Yes |
| 2 | ISYNA1 |  |  | 73 | 0.620 | 0.1511 | Yes |
| 3 | C9ORF16 |  |  | 78 | 0.610 | 0.2226 | Yes |
| 4 | TK1 |  |  | 119 | 0.554 | 0.2859 | Yes |
| 5 | LCN2 |  |  | 262 | 0.463 | 0.3334 | Yes |
| 6 | EXOSC5 |  |  | 401 | 0.425 | 0.3766 | Yes |
| 7 | STMN1 |  |  | 586 | 0.389 | 0.4134 | Yes |
| 8 | H2AFX |  |  | 814 | 0.352 | 0.4437 | Yes |
| 9 | RANBP5 |  |  | 876 | 0.345 | 0.4813 | Yes |
| 10 | TRAPPC5 |  |  | 918 | 0.340 | 0.5193 | Yes |
| 11 | SFRS1 |  |  | 923 | 0.339 | 0.5589 | Yes |
| 12 | ELAVL1 |  |  | 1379 | 0.295 | 0.5715 | Yes |
| 13 | RANGAP1 |  |  | 1598 | 0.279 | 0.5937 | Yes |
| 14 | CAD |  |  | 1624 | 0.277 | 0.6251 | Yes |
| 15 | CDC25C |  |  | 2665 | 0.224 | 0.6009 | No |
| 16 | HMMR |  |  | 3097 | 0.207 | 0.6043 | No |
| 17 | KIF22 |  |  | 3842 | 0.184 | 0.5897 | No |
| 18 | CDC2 |  |  | 3984 | 0.179 | 0.6040 | No |
| 19 | AURKB |  |  | 4313 | 0.171 | 0.6082 | No |
| 20 | UBE2C |  |  | 4419 | 0.168 | 0.6228 | No |
| 21 | NUSAP1 |  |  | 4979 | 0.156 | 0.6140 | No |
| 22 | KIF20A |  |  | 5176 | 0.151 | 0.6223 | No |
| 23 | RRM1 |  |  | 6328 | 0.130 | 0.5816 | No |
| 24 | GALK1 |  |  | 6465 | 0.127 | 0.5899 | No |
| 25 | CENPL |  |  | 9925 | 0.074 | 0.4305 | No |
| 26 | PTTG1 |  |  | 10051 | 0.072 | 0.4329 | No |
| 27 | CDC20 |  |  | 13049 | 0.033 | 0.2910 | No |
| 28 | AQP1 |  |  | 14264 | 0.017 | 0.2340 | No |
| 29 | XTP3TPA |  |  | 14598 | 0.012 | 0.2192 | No |
| 30 | SLPI |  |  | 14802 | 0.009 | 0.2104 | No |
| 31 | CCNB1 |  |  | 15866 | -0.008 | 0.1596 | No |
| 32 | BUB1 |  |  | 16158 | -0.013 | 0.1470 | No |
| 33 | CCNA2 |  |  | 17367 | -0.037 | 0.0925 | No |
| 34 | AGT |  |  | 17814 | -0.047 | 0.0764 | No |
| 35 | PRC1 |  |  | 18504 | -0.065 | 0.0506 | No |
| 36 | RACGAP1 |  |  | 19175 | -0.092 | 0.0288 | No |
| 37 | MELK |  |  | 19351 | -0.101 | 0.0322 | No |
| 38 | ANLN |  |  | 19423 | -0.104 | 0.0409 | No |
| 39 | WFDC12 |  |  | 19956 | -0.140 | 0.0316 | No |
Table: GSEA details [plain text format]

  

Fig 2: ADIP\_DIFF\_CLUSTER5: Random ES distribution      
 Gene set null distribution of ES for **ADIP\_DIFF\_CLUSTER5**

  
