# Supplementary material for: The rapamycin-regulated gene expression signature determines prognosis for breast cancer
Source: Mol Cancer. 2009 Sep 24;8:75. doi: 10.1186/1476-4598-8-75 (PMC2761377; doi:10.1186/1476-4598-8-75)
Supplement: Additional file 3 — Gene set enrichment analysis of in vivo data, treatment series. The data provided represent the treatment series of GSEA. This compressed file contains "Treatment" shortcut file and "GSEA_treatment" folder. Clicking on "Treatment" shortcut opens the index file providing access to analysis files contained in the "GSEA_treatment" folder. [file 1476-4598-8-75-S3.zip › GSEA_treatment/ADIP_VS_PREADIP_UP.html]

Details for gene set ADIP\_VS\_PREADIP\_UP[GSEA]

|  || Dataset | gsea\_treatment\_collapsed |
| Phenotype | NoPhenotypeAvailable |
| Upregulated in class | na\_neg |
| GeneSet | ADIP\_VS\_PREADIP\_UP |
| Enrichment Score (ES) | -0.3551574 |
| Normalized Enrichment Score (NES) | -1.5207856 |
| Nominal p-value | 0.027777778 |
| FDR q-value | 0.18149398 |
| FWER p-Value | 0.824 |
Table: GSEA Results Summary

  

Fig 1: Enrichment plot: ADIP\_VS\_PREADIP\_UP      
 Profile of the Running ES Score & Positions of GeneSet Members on the Rank Ordered List

  

| PROBE | GENE SYMBOL | GENE\_TITLE | RANK IN GENE LIST | RANK METRIC SCORE | RUNNING ES | CORE ENRICHMENT || 1 | BCKDHA |  |  | 230 | 0.479 | 0.0599 | No |
| 2 | SCP2 |  |  | 805 | 0.353 | 0.0845 | No |
| 3 | ANAPC1 |  |  | 1136 | 0.317 | 0.1155 | No |
| 4 | ACADVL |  |  | 1357 | 0.297 | 0.1489 | No |
| 5 | STAT1 |  |  | 1401 | 0.294 | 0.1905 | No |
| 6 | PPARG |  |  | 1732 | 0.271 | 0.2146 | No |
| 7 | PC |  |  | 2386 | 0.236 | 0.2179 | No |
| 8 | CPT2 |  |  | 3063 | 0.208 | 0.2159 | No |
| 9 | CRAT |  |  | 3131 | 0.205 | 0.2431 | No |
| 10 | VEGFB |  |  | 3232 | 0.202 | 0.2682 | No |
| 11 | CFD |  |  | 3315 | 0.200 | 0.2939 | No |
| 12 | ADIPOQ |  |  | 5793 | 0.138 | 0.1940 | No |
| 13 | MNT |  |  | 6505 | 0.126 | 0.1782 | No |
| 14 | ACADS |  |  | 7194 | 0.114 | 0.1618 | No |
| 15 | SQSTM1 |  |  | 8746 | 0.090 | 0.0998 | No |
| 16 | PPA1 |  |  | 9532 | 0.079 | 0.0734 | No |
| 17 | STAT3 |  |  | 10284 | 0.069 | 0.0472 | No |
| 18 | GCLM |  |  | 12365 | 0.042 | -0.0477 | No |
| 19 | CIDEC |  |  | 12441 | 0.041 | -0.0452 | No |
| 20 | LIPE |  |  | 12716 | 0.038 | -0.0529 | No |
| 21 | CD36 |  |  | 12917 | 0.035 | -0.0574 | No |
| 22 | FASN |  |  | 13160 | 0.032 | -0.0645 | No |
| 23 | STAT5A |  |  | 13555 | 0.027 | -0.0796 | No |
| 24 | IGF1 |  |  | 14591 | 0.012 | -0.1282 | No |
| 25 | ITGA7 |  |  | 16258 | -0.014 | -0.2070 | No |
| 26 | GHR |  |  | 16307 | -0.015 | -0.2071 | No |
| 27 | AGT |  |  | 17814 | -0.047 | -0.2733 | No |
| 28 | FABP4 |  |  | 19098 | -0.089 | -0.3224 | No |
| 29 | SPARCL1 |  |  | 19437 | -0.105 | -0.3232 | Yes |
| 30 | ADFP |  |  | 19613 | -0.114 | -0.3148 | Yes |
| 31 | DDIT3 |  |  | 20445 | -0.252 | -0.3178 | Yes |
| 32 | GADD45A |  |  | 20478 | -0.277 | -0.2782 | Yes |
| 33 | ALDH2 |  |  | 20530 | -0.348 | -0.2290 | Yes |
| 34 | ACSL1 |  |  | 20590 | -0.711 | -0.1264 | Yes |
| 35 | LDHB |  |  | 20594 | -0.856 | 0.0005 | Yes |
Table: GSEA details [plain text format]

  

Fig 2: ADIP\_VS\_PREADIP\_UP: Random ES distribution      
 Gene set null distribution of ES for **ADIP\_VS\_PREADIP\_UP**

  
