# Supplementary material for: The rapamycin-regulated gene expression signature determines prognosis for breast cancer
Source: Mol Cancer. 2009 Sep 24;8:75. doi: 10.1186/1476-4598-8-75 (PMC2761377; doi:10.1186/1476-4598-8-75)
Supplement: Additional file 3 — Gene set enrichment analysis of in vivo data, treatment series. The data provided represent the treatment series of GSEA. This compressed file contains "Treatment" shortcut file and "GSEA_treatment" folder. Clicking on "Treatment" shortcut opens the index file providing access to analysis files contained in the "GSEA_treatment" folder. [file 1476-4598-8-75-S3.zip › GSEA_treatment/AMINOACYL_TRNA_BIOSYNTHESIS.html]

Details for gene set AMINOACYL\_TRNA\_BIOSYNTHESIS[GSEA]

|  || Dataset | gsea\_treatment\_collapsed |
| Phenotype | NoPhenotypeAvailable |
| Upregulated in class | na\_neg |
| GeneSet | AMINOACYL\_TRNA\_BIOSYNTHESIS |
| Enrichment Score (ES) | -0.31599855 |
| Normalized Enrichment Score (NES) | -1.2532718 |
| Nominal p-value | 0.18032786 |
| FDR q-value | 0.27520576 |
| FWER p-Value | 1.0 |
Table: GSEA Results Summary

  

Fig 1: Enrichment plot: AMINOACYL\_TRNA\_BIOSYNTHESIS      
 Profile of the Running ES Score & Positions of GeneSet Members on the Rank Ordered List

  

| PROBE | GENE SYMBOL | GENE\_TITLE | RANK IN GENE LIST | RANK METRIC SCORE | RUNNING ES | CORE ENRICHMENT || 1 | QARS |  |  | 240 | 0.473 | 0.1239 | No |
| 2 | DARS |  |  | 1276 | 0.304 | 0.1608 | No |
| 3 | WARS2 |  |  | 1985 | 0.256 | 0.1998 | No |
| 4 | HARSL |  |  | 3171 | 0.204 | 0.2007 | No |
| 5 | LARS2 |  |  | 4395 | 0.169 | 0.1898 | No |
| 6 | MARS2 |  |  | 4991 | 0.156 | 0.2056 | No |
| 7 | LARS |  |  | 5106 | 0.153 | 0.2439 | No |
| 8 | FARS2 |  |  | 7245 | 0.114 | 0.1728 | No |
| 9 | RARS |  |  | 10372 | 0.068 | 0.0403 | No |
| 10 | CARS |  |  | 10805 | 0.062 | 0.0371 | No |
| 11 | EPRS |  |  | 12393 | 0.042 | -0.0280 | No |
| 12 | KARS |  |  | 14246 | 0.017 | -0.1130 | No |
| 13 | YARS |  |  | 15535 | -0.002 | -0.1749 | No |
| 14 | HARS |  |  | 16413 | -0.017 | -0.2125 | No |
| 15 | IARS |  |  | 17477 | -0.039 | -0.2528 | No |
| 16 | WARS |  |  | 17621 | -0.043 | -0.2475 | No |
| 17 | FARSLB |  |  | 18660 | -0.071 | -0.2775 | No |
| 18 | MARS |  |  | 19453 | -0.106 | -0.2857 | Yes |
| 19 | SARS |  |  | 19872 | -0.133 | -0.2678 | Yes |
| 20 | TARS |  |  | 19912 | -0.137 | -0.2305 | Yes |
| 21 | NARS |  |  | 20386 | -0.219 | -0.1906 | Yes |
| 22 | AARS |  |  | 20451 | -0.254 | -0.1207 | Yes |
| 23 | GARS |  |  | 20560 | -0.447 | 0.0022 | Yes |
Table: GSEA details [plain text format]

  

Fig 2: AMINOACYL\_TRNA\_BIOSYNTHESIS: Random ES distribution      
 Gene set null distribution of ES for **AMINOACYL\_TRNA\_BIOSYNTHESIS**

  
