# Supplementary material for: The rapamycin-regulated gene expression signature determines prognosis for breast cancer
Source: Mol Cancer. 2009 Sep 24;8:75. doi: 10.1186/1476-4598-8-75 (PMC2761377; doi:10.1186/1476-4598-8-75)
Supplement: Additional file 3 — Gene set enrichment analysis of in vivo data, treatment series. The data provided represent the treatment series of GSEA. This compressed file contains "Treatment" shortcut file and "GSEA_treatment" folder. Clicking on "Treatment" shortcut opens the index file providing access to analysis files contained in the "GSEA_treatment" folder. [file 1476-4598-8-75-S3.zip › GSEA_treatment/APPEL_IMATINIB_UP.html]

Details for gene set APPEL\_IMATINIB\_UP[GSEA]

|  || Dataset | gsea\_treatment\_collapsed |
| Phenotype | NoPhenotypeAvailable |
| Upregulated in class | na\_pos |
| GeneSet | APPEL\_IMATINIB\_UP |
| Enrichment Score (ES) | 0.67288375 |
| Normalized Enrichment Score (NES) | 1.8317794 |
| Nominal p-value | 0.0 |
| FDR q-value | 0.0016349459 |
| FWER p-Value | 0.033 |
Table: GSEA Results Summary

  

Fig 1: Enrichment plot: APPEL\_IMATINIB\_UP      
 Profile of the Running ES Score & Positions of GeneSet Members on the Rank Ordered List

  

| PROBE | GENE SYMBOL | GENE\_TITLE | RANK IN GENE LIST | RANK METRIC SCORE | RUNNING ES | CORE ENRICHMENT || 1 | CTSB |  |  | 14 | 0.888 | 0.1350 | Yes |
| 2 | FUCA1 |  |  | 339 | 0.442 | 0.1867 | Yes |
| 3 | GUSB |  |  | 403 | 0.425 | 0.2486 | Yes |
| 4 | PLSCR1 |  |  | 717 | 0.365 | 0.2891 | Yes |
| 5 | GM2A |  |  | 1129 | 0.318 | 0.3177 | Yes |
| 6 | ASAH1 |  |  | 1168 | 0.314 | 0.3639 | Yes |
| 7 | PLD1 |  |  | 1177 | 0.313 | 0.4113 | Yes |
| 8 | PPGB |  |  | 1493 | 0.287 | 0.4399 | Yes |
| 9 | PPARG |  |  | 1732 | 0.271 | 0.4697 | Yes |
| 10 | CTSD |  |  | 1748 | 0.269 | 0.5101 | Yes |
| 11 | LAMP1 |  |  | 1895 | 0.261 | 0.5429 | Yes |
| 12 | GNS |  |  | 1961 | 0.257 | 0.5790 | Yes |
| 13 | CTSL |  |  | 2193 | 0.246 | 0.6054 | Yes |
| 14 | APOC1 |  |  | 3416 | 0.196 | 0.5760 | Yes |
| 15 | HEXA |  |  | 3462 | 0.195 | 0.6036 | Yes |
| 16 | CTSH |  |  | 3789 | 0.186 | 0.6161 | Yes |
| 17 | ACP5 |  |  | 3998 | 0.179 | 0.6334 | Yes |
| 18 | LAMP2 |  |  | 4224 | 0.174 | 0.6490 | Yes |
| 19 | APOE |  |  | 4275 | 0.172 | 0.6729 | Yes |
| 20 | CD163 |  |  | 9744 | 0.076 | 0.4188 | No |
| 21 | CD300A |  |  | 11559 | 0.053 | 0.3387 | No |
| 22 | MSR1 |  |  | 13282 | 0.030 | 0.2597 | No |
| 23 | ENG |  |  | 13572 | 0.026 | 0.2497 | No |
| 24 | CTSZ |  |  | 15046 | 0.005 | 0.1789 | No |
| 25 | NEU1 |  |  | 15813 | -0.007 | 0.1427 | No |
| 26 | C5AR1 |  |  | 17032 | -0.029 | 0.0880 | No |
| 27 | APOD |  |  | 17810 | -0.047 | 0.0574 | No |
| 28 | CD68 |  |  | 18754 | -0.075 | 0.0230 | No |
| 29 | RARRES1 |  |  | 19548 | -0.111 | 0.0014 | No |
| 30 | HEXB |  |  | 19596 | -0.113 | 0.0164 | No |
| 31 | OSBPL3 |  |  | 20373 | -0.213 | 0.0113 | No |
Table: GSEA details [plain text format]

  

Fig 2: APPEL\_IMATINIB\_UP: Random ES distribution      
 Gene set null distribution of ES for **APPEL\_IMATINIB\_UP**

  
