# Supplementary material for: The rapamycin-regulated gene expression signature determines prognosis for breast cancer
Source: Mol Cancer. 2009 Sep 24;8:75. doi: 10.1186/1476-4598-8-75 (PMC2761377; doi:10.1186/1476-4598-8-75)
Supplement: Additional file 3 — Gene set enrichment analysis of in vivo data, treatment series. The data provided represent the treatment series of GSEA. This compressed file contains "Treatment" shortcut file and "GSEA_treatment" folder. Clicking on "Treatment" shortcut opens the index file providing access to analysis files contained in the "GSEA_treatment" folder. [file 1476-4598-8-75-S3.zip › GSEA_treatment/ASTON_OLIGODENDROGLIA_MYELINATION_SUBSET.html]

Details for gene set ASTON\_OLIGODENDROGLIA\_MYELINATION\_SUBSET[GSEA]

|  || Dataset | gsea\_treatment\_collapsed |
| Phenotype | NoPhenotypeAvailable |
| Upregulated in class | na\_neg |
| GeneSet | ASTON\_OLIGODENDROGLIA\_MYELINATION\_SUBSET |
| Enrichment Score (ES) | -0.41324267 |
| Normalized Enrichment Score (NES) | -1.4457049 |
| Nominal p-value | 0.07446808 |
| FDR q-value | 0.19582045 |
| FWER p-Value | 0.931 |
Table: GSEA Results Summary

  

Fig 1: Enrichment plot: ASTON\_OLIGODENDROGLIA\_MYELINATION\_SUBSET      
 Profile of the Running ES Score & Positions of GeneSet Members on the Rank Ordered List

  

| PROBE | GENE SYMBOL | GENE\_TITLE | RANK IN GENE LIST | RANK METRIC SCORE | RUNNING ES | CORE ENRICHMENT || 1 | ERBB3 |  |  | 223 | 0.483 | 0.1867 | No |
| 2 | CNP |  |  | 3630 | 0.190 | 0.0990 | No |
| 3 | PLLP |  |  | 5614 | 0.142 | 0.0609 | No |
| 4 | MAG |  |  | 6806 | 0.122 | 0.0528 | No |
| 5 | ENPP2 |  |  | 6815 | 0.122 | 0.1021 | No |
| 6 | MOBP |  |  | 10149 | 0.071 | -0.0307 | No |
| 7 | PMP22 |  |  | 11772 | 0.050 | -0.0893 | No |
| 8 | OLIG2 |  |  | 12018 | 0.047 | -0.0821 | No |
| 9 | MAL |  |  | 12889 | 0.035 | -0.1100 | No |
| 10 | MOG |  |  | 13005 | 0.034 | -0.1018 | No |
| 11 | EDG2 |  |  | 13730 | 0.025 | -0.1270 | No |
| 12 | ASPA |  |  | 13912 | 0.022 | -0.1269 | No |
| 13 | SOX10 |  |  | 15270 | 0.001 | -0.1922 | No |
| 14 | PLP1 |  |  | 16523 | -0.019 | -0.2452 | No |
| 15 | TF |  |  | 18049 | -0.053 | -0.2976 | No |
| 16 | UGT8 |  |  | 20430 | -0.239 | -0.3154 | Yes |
| 17 | KLK6 |  |  | 20593 | -0.793 | 0.0006 | Yes |
Table: GSEA details [plain text format]

  

Fig 2: ASTON\_OLIGODENDROGLIA\_MYELINATION\_SUBSET: Random ES distribution      
 Gene set null distribution of ES for **ASTON\_OLIGODENDROGLIA\_MYELINATION\_SUBSET**

  
