# Supplementary material for: The rapamycin-regulated gene expression signature determines prognosis for breast cancer
Source: Mol Cancer. 2009 Sep 24;8:75. doi: 10.1186/1476-4598-8-75 (PMC2761377; doi:10.1186/1476-4598-8-75)
Supplement: Additional file 3 — Gene set enrichment analysis of in vivo data, treatment series. The data provided represent the treatment series of GSEA. This compressed file contains "Treatment" shortcut file and "GSEA_treatment" folder. Clicking on "Treatment" shortcut opens the index file providing access to analysis files contained in the "GSEA_treatment" folder. [file 1476-4598-8-75-S3.zip › GSEA_treatment/BENNETT_SLE_UP.html]

Details for gene set BENNETT\_SLE\_UP[GSEA]

|  || Dataset | gsea\_treatment\_collapsed |
| Phenotype | NoPhenotypeAvailable |
| Upregulated in class | na\_pos |
| GeneSet | BENNETT\_SLE\_UP |
| Enrichment Score (ES) | 0.79718244 |
| Normalized Enrichment Score (NES) | 2.096661 |
| Nominal p-value | 0.0 |
| FDR q-value | 0.0 |
| FWER p-Value | 0.0 |
Table: GSEA Results Summary

  

Fig 1: Enrichment plot: BENNETT\_SLE\_UP      
 Profile of the Running ES Score & Positions of GeneSet Members on the Rank Ordered List

  

| PROBE | GENE SYMBOL | GENE\_TITLE | RANK IN GENE LIST | RANK METRIC SCORE | RUNNING ES | CORE ENRICHMENT || 1 | MX1 |  |  | 24 | 0.773 | 0.0953 | Yes |
| 2 | S100A8 |  |  | 42 | 0.695 | 0.1812 | Yes |
| 3 | ISG15 |  |  | 59 | 0.644 | 0.2608 | Yes |
| 4 | LGALS3BP |  |  | 61 | 0.643 | 0.3410 | Yes |
| 5 | TNFSF10 |  |  | 160 | 0.518 | 0.4009 | Yes |
| 6 | AGRIN |  |  | 198 | 0.492 | 0.4605 | Yes |
| 7 | LY6E |  |  | 251 | 0.467 | 0.5163 | Yes |
| 8 | OAS1 |  |  | 255 | 0.466 | 0.5743 | Yes |
| 9 | TAP1 |  |  | 556 | 0.393 | 0.6088 | Yes |
| 10 | IRF7 |  |  | 639 | 0.378 | 0.6519 | Yes |
| 11 | PLSCR1 |  |  | 717 | 0.365 | 0.6937 | Yes |
| 12 | IFI35 |  |  | 766 | 0.357 | 0.7359 | Yes |
| 13 | SERPING1 |  |  | 936 | 0.338 | 0.7699 | Yes |
| 14 | STAT1 |  |  | 1401 | 0.294 | 0.7841 | Yes |
| 15 | IFIT3 |  |  | 1814 | 0.265 | 0.7972 | Yes |
| 16 | FPRL1 |  |  | 6075 | 0.134 | 0.6069 | No |
| 17 | BIRC4BP |  |  | 7047 | 0.117 | 0.5744 | No |
| 18 | APOBEC3C |  |  | 7232 | 0.114 | 0.5796 | No |
| 19 | IFITM3 |  |  | 8420 | 0.095 | 0.5338 | No |
| 20 | CAMP |  |  | 9069 | 0.086 | 0.5131 | No |
| 21 | OASL |  |  | 12433 | 0.041 | 0.3548 | No |
| 22 | OAS2 |  |  | 12543 | 0.040 | 0.3545 | No |
| 23 | C2 |  |  | 12568 | 0.039 | 0.3583 | No |
| 24 | TDRD7 |  |  | 13343 | 0.029 | 0.3243 | No |
| 25 | IFI44L |  |  | 14255 | 0.017 | 0.2822 | No |
| 26 | CKAP4 |  |  | 16519 | -0.019 | 0.1746 | No |
| 27 | RNASE2 |  |  | 18634 | -0.070 | 0.0806 | No |
| 28 | MX2 |  |  | 19725 | -0.121 | 0.0428 | No |
Table: GSEA details [plain text format]

  

Fig 2: BENNETT\_SLE\_UP: Random ES distribution      
 Gene set null distribution of ES for **BENNETT\_SLE\_UP**

  
