# Supplementary material for: The rapamycin-regulated gene expression signature determines prognosis for breast cancer
Source: Mol Cancer. 2009 Sep 24;8:75. doi: 10.1186/1476-4598-8-75 (PMC2761377; doi:10.1186/1476-4598-8-75)
Supplement: Additional file 3 — Gene set enrichment analysis of in vivo data, treatment series. The data provided represent the treatment series of GSEA. This compressed file contains "Treatment" shortcut file and "GSEA_treatment" folder. Clicking on "Treatment" shortcut opens the index file providing access to analysis files contained in the "GSEA_treatment" folder. [file 1476-4598-8-75-S3.zip › GSEA_treatment/BRCA1_MES_UP.html]

Details for gene set BRCA1\_MES\_UP[GSEA]

|  || Dataset | gsea\_treatment\_collapsed |
| Phenotype | NoPhenotypeAvailable |
| Upregulated in class | na\_neg |
| GeneSet | BRCA1\_MES\_UP |
| Enrichment Score (ES) | -0.3305077 |
| Normalized Enrichment Score (NES) | -1.4940083 |
| Nominal p-value | 0.057142857 |
| FDR q-value | 0.16821682 |
| FWER p-Value | 0.868 |
Table: GSEA Results Summary

  

Fig 1: Enrichment plot: BRCA1\_MES\_UP      
 Profile of the Running ES Score & Positions of GeneSet Members on the Rank Ordered List

  

| PROBE | GENE SYMBOL | GENE\_TITLE | RANK IN GENE LIST | RANK METRIC SCORE | RUNNING ES | CORE ENRICHMENT || 1 | CA2 |  |  | 225 | 0.483 | 0.0576 | No |
| 2 | ACTA2 |  |  | 1058 | 0.325 | 0.0634 | No |
| 3 | DDX3X |  |  | 1290 | 0.303 | 0.0952 | No |
| 4 | FGFR1 |  |  | 1604 | 0.279 | 0.1195 | No |
| 5 | NUMB |  |  | 2128 | 0.250 | 0.1296 | No |
| 6 | APP |  |  | 2143 | 0.248 | 0.1641 | No |
| 7 | PSMC2 |  |  | 2146 | 0.248 | 0.1993 | No |
| 8 | DNAJA1 |  |  | 2804 | 0.218 | 0.1983 | No |
| 9 | RPA2 |  |  | 3050 | 0.209 | 0.2160 | No |
| 10 | TFAP2C |  |  | 3182 | 0.203 | 0.2385 | No |
| 11 | DLG1 |  |  | 3367 | 0.198 | 0.2577 | No |
| 12 | DDX1 |  |  | 3384 | 0.198 | 0.2850 | No |
| 13 | TPM1 |  |  | 3849 | 0.184 | 0.2885 | No |
| 14 | WNT3 |  |  | 5030 | 0.155 | 0.2532 | No |
| 15 | RIT1 |  |  | 5227 | 0.151 | 0.2650 | No |
| 16 | ARF1 |  |  | 5294 | 0.149 | 0.2830 | No |
| 17 | EIF2S1 |  |  | 8649 | 0.092 | 0.1329 | No |
| 18 | KRT8 |  |  | 10715 | 0.063 | 0.0416 | No |
| 19 | ROCK2 |  |  | 10786 | 0.063 | 0.0470 | No |
| 20 | SSH2 |  |  | 10875 | 0.061 | 0.0514 | No |
| 21 | PDIA3 |  |  | 11707 | 0.050 | 0.0182 | No |
| 22 | EPRS |  |  | 12393 | 0.042 | -0.0092 | No |
| 23 | ATP6AP1 |  |  | 13278 | 0.030 | -0.0478 | No |
| 24 | NEFL |  |  | 13388 | 0.029 | -0.0490 | No |
| 25 | DDX24 |  |  | 13722 | 0.025 | -0.0618 | No |
| 26 | TAGLN |  |  | 13864 | 0.023 | -0.0654 | No |
| 27 | TCEA1 |  |  | 14360 | 0.015 | -0.0873 | No |
| 28 | STAM |  |  | 15934 | -0.009 | -0.1625 | No |
| 29 | LXN |  |  | 16060 | -0.011 | -0.1670 | No |
| 30 | ANXA5 |  |  | 17826 | -0.047 | -0.2461 | No |
| 31 | PSMC1 |  |  | 18161 | -0.056 | -0.2544 | No |
| 32 | ANXA3 |  |  | 19727 | -0.122 | -0.3132 | Yes |
| 33 | SARS |  |  | 19872 | -0.133 | -0.3013 | Yes |
| 34 | ACTC1 |  |  | 20123 | -0.160 | -0.2908 | Yes |
| 35 | DDX21 |  |  | 20219 | -0.176 | -0.2703 | Yes |
| 36 | VIM |  |  | 20275 | -0.188 | -0.2463 | Yes |
| 37 | ID3 |  |  | 20467 | -0.265 | -0.2180 | Yes |
| 38 | SFN |  |  | 20552 | -0.415 | -0.1631 | Yes |
| 39 | FGFBP1 |  |  | 20602 | -1.166 | 0.0001 | Yes |
Table: GSEA details [plain text format]

  

Fig 2: BRCA1\_MES\_UP: Random ES distribution      
 Gene set null distribution of ES for **BRCA1\_MES\_UP**

  
