# Supplementary material for: The rapamycin-regulated gene expression signature determines prognosis for breast cancer
Source: Mol Cancer. 2009 Sep 24;8:75. doi: 10.1186/1476-4598-8-75 (PMC2761377; doi:10.1186/1476-4598-8-75)
Supplement: Additional file 3 — Gene set enrichment analysis of in vivo data, treatment series. The data provided represent the treatment series of GSEA. This compressed file contains "Treatment" shortcut file and "GSEA_treatment" folder. Clicking on "Treatment" shortcut opens the index file providing access to analysis files contained in the "GSEA_treatment" folder. [file 1476-4598-8-75-S3.zip › GSEA_treatment/BRCA1_OVEREXP_UP.html]

Details for gene set BRCA1\_OVEREXP\_UP[GSEA]

|  || Dataset | gsea\_treatment\_collapsed |
| Phenotype | NoPhenotypeAvailable |
| Upregulated in class | na\_pos |
| GeneSet | BRCA1\_OVEREXP\_UP |
| Enrichment Score (ES) | 0.57034194 |
| Normalized Enrichment Score (NES) | 1.7396992 |
| Nominal p-value | 0.0 |
| FDR q-value | 0.008465379 |
| FWER p-Value | 0.226 |
Table: GSEA Results Summary

  

Fig 1: Enrichment plot: BRCA1\_OVEREXP\_UP      
 Profile of the Running ES Score & Positions of GeneSet Members on the Rank Ordered List

  

| PROBE | GENE SYMBOL | GENE\_TITLE | RANK IN GENE LIST | RANK METRIC SCORE | RUNNING ES | CORE ENRICHMENT || 1 | TFPI2 |  |  | 0 | 1.316 | 0.0434 | Yes |
| 2 | CIRBP |  |  | 19 | 0.845 | 0.0704 | Yes |
| 3 | SFRS3 |  |  | 123 | 0.550 | 0.0835 | Yes |
| 4 | SERPINB1 |  |  | 169 | 0.511 | 0.0981 | Yes |
| 5 | ALDH7A1 |  |  | 327 | 0.444 | 0.1051 | Yes |
| 6 | VAMP3 |  |  | 384 | 0.429 | 0.1165 | Yes |
| 7 | TCEAL1 |  |  | 455 | 0.412 | 0.1267 | Yes |
| 8 | CD47 |  |  | 481 | 0.408 | 0.1389 | Yes |
| 9 | CCNG2 |  |  | 485 | 0.408 | 0.1522 | Yes |
| 10 | WRB |  |  | 633 | 0.378 | 0.1575 | Yes |
| 11 | FSTL1 |  |  | 636 | 0.378 | 0.1699 | Yes |
| 12 | C21ORF33 |  |  | 655 | 0.374 | 0.1813 | Yes |
| 13 | HMGN4 |  |  | 697 | 0.368 | 0.1915 | Yes |
| 14 | MAT2A |  |  | 706 | 0.367 | 0.2032 | Yes |
| 15 | CITED2 |  |  | 722 | 0.365 | 0.2145 | Yes |
| 16 | SMC1A |  |  | 778 | 0.356 | 0.2235 | Yes |
| 17 | KIFAP3 |  |  | 797 | 0.354 | 0.2343 | Yes |
| 18 | CYBA |  |  | 823 | 0.350 | 0.2446 | Yes |
| 19 | DDOST |  |  | 828 | 0.349 | 0.2560 | Yes |
| 20 | NDUFA4 |  |  | 860 | 0.346 | 0.2659 | Yes |
| 21 | SCAMP1 |  |  | 862 | 0.346 | 0.2772 | Yes |
| 22 | PCBD1 |  |  | 958 | 0.335 | 0.2836 | Yes |
| 23 | DEK |  |  | 988 | 0.333 | 0.2932 | Yes |
| 24 | ACTA2 |  |  | 1058 | 0.325 | 0.3005 | Yes |
| 25 | ID4 |  |  | 1119 | 0.319 | 0.3081 | Yes |
| 26 | NFYC |  |  | 1181 | 0.313 | 0.3155 | Yes |
| 27 | ABCD3 |  |  | 1221 | 0.308 | 0.3237 | Yes |
| 28 | NPAL3 |  |  | 1259 | 0.305 | 0.3320 | Yes |
| 29 | DDX3X |  |  | 1290 | 0.303 | 0.3405 | Yes |
| 30 | SERINC3 |  |  | 1306 | 0.302 | 0.3497 | Yes |
| 31 | CSDA |  |  | 1327 | 0.300 | 0.3586 | Yes |
| 32 | ITGB3BP |  |  | 1398 | 0.294 | 0.3649 | Yes |
| 33 | STAT1 |  |  | 1401 | 0.294 | 0.3745 | Yes |
| 34 | ANXA4 |  |  | 1541 | 0.284 | 0.3771 | Yes |
| 35 | FCGRT |  |  | 1542 | 0.284 | 0.3865 | Yes |
| 36 | TSNAX |  |  | 1563 | 0.282 | 0.3948 | Yes |
| 37 | ZFP36L1 |  |  | 1617 | 0.278 | 0.4013 | Yes |
| 38 | TRAM1 |  |  | 1618 | 0.278 | 0.4105 | Yes |
| 39 | EXOC5 |  |  | 1623 | 0.277 | 0.4195 | Yes |
| 40 | TSG101 |  |  | 1666 | 0.274 | 0.4265 | Yes |
| 41 | CREB1 |  |  | 1694 | 0.273 | 0.4341 | Yes |
| 42 | ACTR1B |  |  | 1742 | 0.270 | 0.4408 | Yes |
| 43 | TM9SF1 |  |  | 1929 | 0.259 | 0.4402 | Yes |
| 44 | ZNF43 |  |  | 2039 | 0.253 | 0.4432 | Yes |
| 45 | TMED2 |  |  | 2099 | 0.250 | 0.4486 | Yes |
| 46 | ADD1 |  |  | 2150 | 0.248 | 0.4543 | Yes |
| 47 | SEPHS2 |  |  | 2254 | 0.242 | 0.4573 | Yes |
| 48 | CXADR |  |  | 2335 | 0.239 | 0.4613 | Yes |
| 49 | TUBB3 |  |  | 2365 | 0.237 | 0.4677 | Yes |
| 50 | KIF11 |  |  | 2725 | 0.222 | 0.4574 | Yes |
| 51 | UBE2D3 |  |  | 2819 | 0.217 | 0.4600 | Yes |
| 52 | SSR1 |  |  | 2980 | 0.211 | 0.4592 | Yes |
| 53 | HMMR |  |  | 3097 | 0.207 | 0.4603 | Yes |
| 54 | SDHC |  |  | 3109 | 0.206 | 0.4666 | Yes |
| 55 | ADD3 |  |  | 3113 | 0.206 | 0.4732 | Yes |
| 56 | PRKAR1A |  |  | 3195 | 0.203 | 0.4760 | Yes |
| 57 | HAGH |  |  | 3289 | 0.200 | 0.4780 | Yes |
| 58 | P2RX4 |  |  | 3334 | 0.199 | 0.4824 | Yes |
| 59 | BNIP3 |  |  | 3398 | 0.197 | 0.4858 | Yes |
| 60 | RAB6A |  |  | 3456 | 0.195 | 0.4895 | Yes |
| 61 | PPP4C |  |  | 3479 | 0.194 | 0.4948 | Yes |
| 62 | RAB1A |  |  | 3525 | 0.193 | 0.4990 | Yes |
| 63 | CD9 |  |  | 3541 | 0.192 | 0.5046 | Yes |
| 64 | LAMA3 |  |  | 3543 | 0.192 | 0.5109 | Yes |
| 65 | KDELR2 |  |  | 3656 | 0.189 | 0.5117 | Yes |
| 66 | NPC2 |  |  | 3729 | 0.188 | 0.5143 | Yes |
| 67 | TAF11 |  |  | 3764 | 0.186 | 0.5188 | Yes |
| 68 | PPP6C |  |  | 3779 | 0.186 | 0.5242 | Yes |
| 69 | CD46 |  |  | 3833 | 0.184 | 0.5277 | Yes |
| 70 | CDC2 |  |  | 3984 | 0.179 | 0.5263 | Yes |
| 71 | IQGAP1 |  |  | 4143 | 0.176 | 0.5244 | Yes |
| 72 | LIMS1 |  |  | 4163 | 0.175 | 0.5292 | Yes |
| 73 | MRCL3 |  |  | 4170 | 0.175 | 0.5347 | Yes |
| 74 | FRAP1 |  |  | 4190 | 0.175 | 0.5395 | Yes |
| 75 | AGPS |  |  | 4287 | 0.172 | 0.5405 | Yes |
| 76 | GPS2 |  |  | 4392 | 0.169 | 0.5410 | Yes |
| 77 | TOP2A |  |  | 4403 | 0.169 | 0.5461 | Yes |
| 78 | PTCRA |  |  | 4408 | 0.169 | 0.5514 | Yes |
| 79 | COPS2 |  |  | 4463 | 0.167 | 0.5543 | Yes |
| 80 | PPIC |  |  | 4479 | 0.167 | 0.5591 | Yes |
| 81 | PLD3 |  |  | 4489 | 0.167 | 0.5642 | Yes |
| 82 | LIPA |  |  | 4577 | 0.165 | 0.5653 | Yes |
| 83 | PGRMC1 |  |  | 4587 | 0.165 | 0.5703 | Yes |
| 84 | LTB4DH |  |  | 4802 | 0.160 | 0.5651 | No |
| 85 | TEGT |  |  | 5004 | 0.155 | 0.5604 | No |
| 86 | SEC23A |  |  | 5054 | 0.154 | 0.5631 | No |
| 87 | EXTL2 |  |  | 5256 | 0.150 | 0.5582 | No |
| 88 | RPS6KA3 |  |  | 5690 | 0.141 | 0.5417 | No |
| 89 | ANXA1 |  |  | 5708 | 0.140 | 0.5455 | No |
| 90 | JAK1 |  |  | 5904 | 0.137 | 0.5405 | No |
| 91 | NDUFS1 |  |  | 6064 | 0.134 | 0.5371 | No |
| 92 | MFGE8 |  |  | 6071 | 0.134 | 0.5413 | No |
| 93 | MAP4K5 |  |  | 6084 | 0.134 | 0.5451 | No |
| 94 | TITF1 |  |  | 6214 | 0.131 | 0.5431 | No |
| 95 | HTATIP |  |  | 6472 | 0.127 | 0.5347 | No |
| 96 | GNAI1 |  |  | 6689 | 0.124 | 0.5282 | No |
| 97 | RDX |  |  | 6786 | 0.122 | 0.5276 | No |
| 98 | ENPP2 |  |  | 6815 | 0.122 | 0.5302 | No |
| 99 | SEPP1 |  |  | 6864 | 0.121 | 0.5318 | No |
| 100 | PPP2R5C |  |  | 7256 | 0.113 | 0.5165 | No |
| 101 | ARPC1A |  |  | 7308 | 0.113 | 0.5177 | No |
| 102 | RAB2 |  |  | 7498 | 0.110 | 0.5121 | No |
| 103 | TMED10 |  |  | 7619 | 0.108 | 0.5097 | No |
| 104 | RRM2 |  |  | 7744 | 0.106 | 0.5072 | No |
| 105 | COPS8 |  |  | 7781 | 0.105 | 0.5089 | No |
| 106 | MEF2C |  |  | 7862 | 0.104 | 0.5084 | No |
| 107 | KIF2A |  |  | 8144 | 0.100 | 0.4980 | No |
| 108 | SFRS5 |  |  | 8966 | 0.088 | 0.4607 | No |
| 109 | PRCP |  |  | 9016 | 0.087 | 0.4611 | No |
| 110 | STC1 |  |  | 9045 | 0.086 | 0.4626 | No |
| 111 | MAP2K4 |  |  | 9253 | 0.083 | 0.4552 | No |
| 112 | NDUFA12 |  |  | 9370 | 0.082 | 0.4523 | No |
| 113 | HIST2H2AA3 |  |  | 9462 | 0.080 | 0.4505 | No |
| 114 | ARF6 |  |  | 9489 | 0.080 | 0.4518 | No |
| 115 | IGF2R |  |  | 9501 | 0.080 | 0.4539 | No |
| 116 | ATP6V0A1 |  |  | 9579 | 0.079 | 0.4528 | No |
| 117 | SUCLG1 |  |  | 9817 | 0.076 | 0.4437 | No |
| 118 | KIF2C |  |  | 9876 | 0.075 | 0.4433 | No |
| 119 | HDAC1 |  |  | 10070 | 0.072 | 0.4362 | No |
| 120 | LUM |  |  | 10113 | 0.072 | 0.4366 | No |
| 121 | GATA3 |  |  | 10631 | 0.064 | 0.4134 | No |
| 122 | PTPN1 |  |  | 11238 | 0.057 | 0.3856 | No |
| 123 | IDH1 |  |  | 11460 | 0.054 | 0.3766 | No |
| 124 | PICALM |  |  | 11612 | 0.052 | 0.3709 | No |
| 125 | DR1 |  |  | 11855 | 0.049 | 0.3607 | No |
| 126 | CALM2 |  |  | 11920 | 0.048 | 0.3591 | No |
| 127 | WWP1 |  |  | 11937 | 0.048 | 0.3599 | No |
| 128 | ZNF148 |  |  | 12121 | 0.045 | 0.3525 | No |
| 129 | TM9SF2 |  |  | 12206 | 0.044 | 0.3498 | No |
| 130 | ZNF267 |  |  | 12359 | 0.042 | 0.3438 | No |
| 131 | MLH1 |  |  | 12496 | 0.040 | 0.3385 | No |
| 132 | MAP2K1 |  |  | 13159 | 0.032 | 0.3071 | No |
| 133 | XIST |  |  | 13876 | 0.023 | 0.2728 | No |
| 134 | HMGN3 |  |  | 13931 | 0.022 | 0.2709 | No |
| 135 | TIMM17A |  |  | 14122 | 0.019 | 0.2622 | No |
| 136 | ANXA2 |  |  | 14618 | 0.012 | 0.2384 | No |
| 137 | NTS |  |  | 14662 | 0.011 | 0.2367 | No |
| 138 | CSNK1A1 |  |  | 15386 | -0.000 | 0.2013 | No |
| 139 | KCNK1 |  |  | 15542 | -0.002 | 0.1938 | No |
| 140 | ID2 |  |  | 15550 | -0.003 | 0.1936 | No |
| 141 | FGF9 |  |  | 15627 | -0.004 | 0.1900 | No |
| 142 | KIF5B |  |  | 15633 | -0.004 | 0.1899 | No |
| 143 | GALNT3 |  |  | 15713 | -0.005 | 0.1862 | No |
| 144 | NEU1 |  |  | 15813 | -0.007 | 0.1816 | No |
| 145 | RAB5B |  |  | 16108 | -0.012 | 0.1676 | No |
| 146 | CDKN1A |  |  | 16349 | -0.016 | 0.1564 | No |
| 147 | PBX3 |  |  | 16357 | -0.016 | 0.1566 | No |
| 148 | ALCAM |  |  | 16942 | -0.027 | 0.1290 | No |
| 149 | NID1 |  |  | 17467 | -0.039 | 0.1046 | No |
| 150 | MPV17 |  |  | 18708 | -0.073 | 0.0464 | No |
| 151 | PAM |  |  | 18827 | -0.078 | 0.0432 | No |
| 152 | RABGGTB |  |  | 19123 | -0.090 | 0.0317 | No |
| 153 | PPP1R1A |  |  | 19142 | -0.091 | 0.0338 | No |
| 154 | ADCYAP1 |  |  | 19285 | -0.097 | 0.0301 | No |
| 155 | QPCT |  |  | 20006 | -0.145 | -0.0004 | No |
| 156 | KRT4 |  |  | 20217 | -0.176 | -0.0048 | No |
| 157 | CKS2 |  |  | 20271 | -0.187 | -0.0012 | No |
| 158 | ID2B |  |  | 20372 | -0.213 | 0.0009 | No |
| 159 | GUCY1B3 |  |  | 20511 | -0.316 | 0.0046 | No |
Table: GSEA details [plain text format]

  

Fig 2: BRCA1\_OVEREXP\_UP: Random ES distribution      
 Gene set null distribution of ES for **BRCA1\_OVEREXP\_UP**

  
