# Supplementary material for: The rapamycin-regulated gene expression signature determines prognosis for breast cancer
Source: Mol Cancer. 2009 Sep 24;8:75. doi: 10.1186/1476-4598-8-75 (PMC2761377; doi:10.1186/1476-4598-8-75)
Supplement: Additional file 3 — Gene set enrichment analysis of in vivo data, treatment series. The data provided represent the treatment series of GSEA. This compressed file contains "Treatment" shortcut file and "GSEA_treatment" folder. Clicking on "Treatment" shortcut opens the index file providing access to analysis files contained in the "GSEA_treatment" folder. [file 1476-4598-8-75-S3.zip › GSEA_treatment/BUT_TSA_UP.html]

Details for gene set BUT\_TSA\_UP[GSEA]

|  || Dataset | gsea\_treatment\_collapsed |
| Phenotype | NoPhenotypeAvailable |
| Upregulated in class | na\_neg |
| GeneSet | BUT\_TSA\_UP |
| Enrichment Score (ES) | -0.34142944 |
| Normalized Enrichment Score (NES) | -1.2644836 |
| Nominal p-value | 0.18666667 |
| FDR q-value | 0.26860994 |
| FWER p-Value | 1.0 |
Table: GSEA Results Summary

  

Fig 1: Enrichment plot: BUT\_TSA\_UP      
 Profile of the Running ES Score & Positions of GeneSet Members on the Rank Ordered List

  

| PROBE | GENE SYMBOL | GENE\_TITLE | RANK IN GENE LIST | RANK METRIC SCORE | RUNNING ES | CORE ENRICHMENT || 1 | ICAM1 |  |  | 408 | 0.423 | 0.1196 | Yes |
| 2 | NET1 |  |  | 1428 | 0.291 | 0.1661 | Yes |
| 3 | PRKCD |  |  | 1691 | 0.273 | 0.2434 | Yes |
| 4 | POR |  |  | 3108 | 0.206 | 0.2426 | Yes |
| 5 | MAPRE1 |  |  | 3518 | 0.193 | 0.2865 | Yes |
| 6 | RHOA |  |  | 4320 | 0.171 | 0.3040 | Yes |
| 7 | NR4A1 |  |  | 7746 | 0.106 | 0.1725 | No |
| 8 | RNH1 |  |  | 10541 | 0.066 | 0.0585 | No |
| 9 | GSTT1 |  |  | 12958 | 0.035 | -0.0475 | No |
| 10 | CDC20 |  |  | 13049 | 0.033 | -0.0409 | No |
| 11 | CXCR4 |  |  | 14667 | 0.011 | -0.1158 | No |
| 12 | GATA2 |  |  | 14848 | 0.008 | -0.1219 | No |
| 13 | CDKN1A |  |  | 16349 | -0.016 | -0.1893 | No |
| 14 | EPHB3 |  |  | 19077 | -0.088 | -0.2930 | No |
| 15 | PRDX1 |  |  | 20076 | -0.151 | -0.2915 | No |
| 16 | TOB1 |  |  | 20356 | -0.208 | -0.2365 | No |
| 17 | GADD45A |  |  | 20478 | -0.277 | -0.1511 | No |
| 18 | HSPB1 |  |  | 20567 | -0.477 | 0.0018 | No |
Table: GSEA details [plain text format]

  

Fig 2: BUT\_TSA\_UP: Random ES distribution      
 Gene set null distribution of ES for **BUT\_TSA\_UP**

  
