# Supplementary material for: The rapamycin-regulated gene expression signature determines prognosis for breast cancer
Source: Mol Cancer. 2009 Sep 24;8:75. doi: 10.1186/1476-4598-8-75 (PMC2761377; doi:10.1186/1476-4598-8-75)
Supplement: Additional file 3 — Gene set enrichment analysis of in vivo data, treatment series. The data provided represent the treatment series of GSEA. This compressed file contains "Treatment" shortcut file and "GSEA_treatment" folder. Clicking on "Treatment" shortcut opens the index file providing access to analysis files contained in the "GSEA_treatment" folder. [file 1476-4598-8-75-S3.zip › GSEA_treatment/CHAUHAN_2ME2.html]

Details for gene set CHAUHAN\_2ME2[GSEA]

|  || Dataset | gsea\_treatment\_collapsed |
| Phenotype | NoPhenotypeAvailable |
| Upregulated in class | na\_pos |
| GeneSet | CHAUHAN\_2ME2 |
| Enrichment Score (ES) | 0.60199076 |
| Normalized Enrichment Score (NES) | 1.6870066 |
| Nominal p-value | 0.0 |
| FDR q-value | 0.014345262 |
| FWER p-Value | 0.499 |
Table: GSEA Results Summary

  

Fig 1: Enrichment plot: CHAUHAN\_2ME2      
 Profile of the Running ES Score & Positions of GeneSet Members on the Rank Ordered List

  

| PROBE | GENE SYMBOL | GENE\_TITLE | RANK IN GENE LIST | RANK METRIC SCORE | RUNNING ES | CORE ENRICHMENT || 1 | MYC |  |  | 75 | 0.615 | 0.0533 | Yes |
| 2 | TAPBP |  |  | 89 | 0.595 | 0.1078 | Yes |
| 3 | SLC25A6 |  |  | 121 | 0.552 | 0.1575 | Yes |
| 4 | NAP1L1 |  |  | 142 | 0.531 | 0.2058 | Yes |
| 5 | EIF3S5 |  |  | 164 | 0.516 | 0.2526 | Yes |
| 6 | UBE1 |  |  | 219 | 0.484 | 0.2948 | Yes |
| 7 | TNFAIP3 |  |  | 406 | 0.424 | 0.3251 | Yes |
| 8 | EIF4B |  |  | 533 | 0.398 | 0.3558 | Yes |
| 9 | LITAF |  |  | 538 | 0.396 | 0.3923 | Yes |
| 10 | ARPC5 |  |  | 1026 | 0.328 | 0.3991 | Yes |
| 11 | ITGB5 |  |  | 1080 | 0.323 | 0.4265 | Yes |
| 12 | TUBB |  |  | 1267 | 0.305 | 0.4457 | Yes |
| 13 | PARP1 |  |  | 1466 | 0.289 | 0.4629 | Yes |
| 14 | LTA4H |  |  | 1589 | 0.279 | 0.4828 | Yes |
| 15 | SFRS6 |  |  | 1636 | 0.277 | 0.5062 | Yes |
| 16 | ST5 |  |  | 1714 | 0.272 | 0.5277 | Yes |
| 17 | CTBP1 |  |  | 1734 | 0.271 | 0.5518 | Yes |
| 18 | MCL1 |  |  | 1881 | 0.262 | 0.5690 | Yes |
| 19 | CSNK1E |  |  | 2147 | 0.248 | 0.5791 | Yes |
| 20 | ADD1 |  |  | 2150 | 0.248 | 0.6020 | Yes |
| 21 | HERPUD1 |  |  | 3327 | 0.199 | 0.5632 | No |
| 22 | EIF5A |  |  | 3517 | 0.193 | 0.5720 | No |
| 23 | UBE2G2 |  |  | 4117 | 0.176 | 0.5592 | No |
| 24 | TUBB2C |  |  | 4295 | 0.172 | 0.5665 | No |
| 25 | GPR161 |  |  | 5312 | 0.149 | 0.5308 | No |
| 26 | UBE2E3 |  |  | 5601 | 0.142 | 0.5300 | No |
| 27 | PIM2 |  |  | 5797 | 0.138 | 0.5333 | No |
| 28 | COX6A1 |  |  | 6192 | 0.132 | 0.5264 | No |
| 29 | CKB |  |  | 6395 | 0.128 | 0.5285 | No |
| 30 | UBE2M |  |  | 7592 | 0.108 | 0.4803 | No |
| 31 | RAF1 |  |  | 8147 | 0.100 | 0.4626 | No |
| 32 | PKM2 |  |  | 8667 | 0.091 | 0.4458 | No |
| 33 | ARHGDIB |  |  | 9762 | 0.076 | 0.3997 | No |
| 34 | PMM2 |  |  | 11565 | 0.052 | 0.3169 | No |
| 35 | MUC1 |  |  | 12069 | 0.046 | 0.2967 | No |
| 36 | HSP90B1 |  |  | 14782 | 0.010 | 0.1657 | No |
| 37 | DECR2 |  |  | 14800 | 0.009 | 0.1657 | No |
| 38 | BTG2 |  |  | 16013 | -0.011 | 0.1077 | No |
| 39 | CRYBB1 |  |  | 17397 | -0.038 | 0.0439 | No |
| 40 | MKI67 |  |  | 17689 | -0.044 | 0.0339 | No |
| 41 | FDPS |  |  | 18142 | -0.055 | 0.0170 | No |
| 42 | LGALS1 |  |  | 19156 | -0.091 | -0.0238 | No |
| 43 | IL2RG |  |  | 19997 | -0.144 | -0.0513 | No |
| 44 | CKS2 |  |  | 20271 | -0.187 | -0.0472 | No |
| 45 | VIM |  |  | 20275 | -0.188 | -0.0299 | No |
| 46 | ADM |  |  | 20571 | -0.495 | 0.0017 | No |
Table: GSEA details [plain text format]

  

Fig 2: CHAUHAN\_2ME2: Random ES distribution      
 Gene set null distribution of ES for **CHAUHAN\_2ME2**

  
