# Supplementary material for: The rapamycin-regulated gene expression signature determines prognosis for breast cancer
Source: Mol Cancer. 2009 Sep 24;8:75. doi: 10.1186/1476-4598-8-75 (PMC2761377; doi:10.1186/1476-4598-8-75)
Supplement: Additional file 3 — Gene set enrichment analysis of in vivo data, treatment series. The data provided represent the treatment series of GSEA. This compressed file contains "Treatment" shortcut file and "GSEA_treatment" folder. Clicking on "Treatment" shortcut opens the index file providing access to analysis files contained in the "GSEA_treatment" folder. [file 1476-4598-8-75-S3.zip › GSEA_treatment/CHOLESTEROL_BIOSYNTHESIS.html]

Details for gene set CHOLESTEROL\_BIOSYNTHESIS[GSEA]

|  || Dataset | gsea\_treatment\_collapsed |
| Phenotype | NoPhenotypeAvailable |
| Upregulated in class | na\_neg |
| GeneSet | CHOLESTEROL\_BIOSYNTHESIS |
| Enrichment Score (ES) | -0.8777282 |
| Normalized Enrichment Score (NES) | -2.920087 |
| Nominal p-value | 0.0 |
| FDR q-value | 0.0 |
| FWER p-Value | 0.0 |
Table: GSEA Results Summary

  

Fig 1: Enrichment plot: CHOLESTEROL\_BIOSYNTHESIS      
 Profile of the Running ES Score & Positions of GeneSet Members on the Rank Ordered List

  

| PROBE | GENE SYMBOL | GENE\_TITLE | RANK IN GENE LIST | RANK METRIC SCORE | RUNNING ES | CORE ENRICHMENT || 1 | PMVK |  |  | 11275 | 0.056 | -0.5298 | No |
| 2 | MVK |  |  | 13158 | 0.032 | -0.6111 | No |
| 3 | FDFT1 |  |  | 17340 | -0.036 | -0.8027 | No |
| 4 | LSS |  |  | 17971 | -0.051 | -0.8172 | No |
| 5 | FDPS |  |  | 18142 | -0.055 | -0.8079 | No |
| 6 | IDI1 |  |  | 19265 | -0.096 | -0.8320 | Yes |
| 7 | SC4MOL |  |  | 20208 | -0.175 | -0.8225 | Yes |
| 8 | SC5DL |  |  | 20250 | -0.183 | -0.7664 | Yes |
| 9 | SQLE |  |  | 20301 | -0.196 | -0.7069 | Yes |
| 10 | NSDHL |  |  | 20464 | -0.263 | -0.6316 | Yes |
| 11 | DHCR7 |  |  | 20521 | -0.327 | -0.5307 | Yes |
| 12 | CYP51A1 |  |  | 20531 | -0.354 | -0.4191 | Yes |
| 13 | HMGCR |  |  | 20532 | -0.358 | -0.3058 | Yes |
| 14 | MVD |  |  | 20536 | -0.372 | -0.1882 | Yes |
| 15 | HMGCS1 |  |  | 20582 | -0.605 | 0.0011 | Yes |
Table: GSEA details [plain text format]

  

Fig 2: CHOLESTEROL\_BIOSYNTHESIS: Random ES distribution      
 Gene set null distribution of ES for **CHOLESTEROL\_BIOSYNTHESIS**

  
