# Supplementary material for: The rapamycin-regulated gene expression signature determines prognosis for breast cancer
Source: Mol Cancer. 2009 Sep 24;8:75. doi: 10.1186/1476-4598-8-75 (PMC2761377; doi:10.1186/1476-4598-8-75)
Supplement: Additional file 3 — Gene set enrichment analysis of in vivo data, treatment series. The data provided represent the treatment series of GSEA. This compressed file contains "Treatment" shortcut file and "GSEA_treatment" folder. Clicking on "Treatment" shortcut opens the index file providing access to analysis files contained in the "GSEA_treatment" folder. [file 1476-4598-8-75-S3.zip › GSEA_treatment/CMV_HCMV_TIMECOURSE_12HRS_UP.html]

Details for gene set CMV\_HCMV\_TIMECOURSE\_12HRS\_UP[GSEA]

|  || Dataset | gsea\_treatment\_collapsed |
| Phenotype | NoPhenotypeAvailable |
| Upregulated in class | na\_pos |
| GeneSet | CMV\_HCMV\_TIMECOURSE\_12HRS\_UP |
| Enrichment Score (ES) | 0.6657304 |
| Normalized Enrichment Score (NES) | 1.7649101 |
| Nominal p-value | 0.0 |
| FDR q-value | 0.0054280083 |
| FWER p-Value | 0.135 |
Table: GSEA Results Summary

  

Fig 1: Enrichment plot: CMV\_HCMV\_TIMECOURSE\_12HRS\_UP      
 Profile of the Running ES Score & Positions of GeneSet Members on the Rank Ordered List

  

| PROBE | GENE SYMBOL | GENE\_TITLE | RANK IN GENE LIST | RANK METRIC SCORE | RUNNING ES | CORE ENRICHMENT || 1 | MX1 |  |  | 24 | 0.773 | 0.1257 | Yes |
| 2 | ISG15 |  |  | 59 | 0.644 | 0.2299 | Yes |
| 3 | SOD2 |  |  | 150 | 0.523 | 0.3114 | Yes |
| 4 | TNFSF10 |  |  | 160 | 0.518 | 0.3960 | Yes |
| 5 | RARRES3 |  |  | 182 | 0.503 | 0.4776 | Yes |
| 6 | OAS1 |  |  | 255 | 0.466 | 0.5507 | Yes |
| 7 | IRF7 |  |  | 639 | 0.378 | 0.5941 | Yes |
| 8 | B4GALT5 |  |  | 1461 | 0.289 | 0.6017 | Yes |
| 9 | IFIT3 |  |  | 1814 | 0.265 | 0.6282 | Yes |
| 10 | RRAD |  |  | 2789 | 0.219 | 0.6168 | Yes |
| 11 | RSAD2 |  |  | 3107 | 0.206 | 0.6353 | Yes |
| 12 | PRPF19 |  |  | 3321 | 0.199 | 0.6576 | Yes |
| 13 | ISG20 |  |  | 3782 | 0.186 | 0.6657 | Yes |
| 14 | RHOB |  |  | 4540 | 0.166 | 0.6562 | No |
| 15 | IL6 |  |  | 5899 | 0.137 | 0.6126 | No |
| 16 | CCL5 |  |  | 8249 | 0.098 | 0.5145 | No |
| 17 | OASL |  |  | 12433 | 0.041 | 0.3181 | No |
| 18 | OAS2 |  |  | 12543 | 0.040 | 0.3193 | No |
| 19 | TNFAIP6 |  |  | 12970 | 0.034 | 0.3042 | No |
| 20 | NCF2 |  |  | 14671 | 0.011 | 0.2234 | No |
| 21 | ZNF264 |  |  | 15149 | 0.004 | 0.2009 | No |
| 22 | CBX4 |  |  | 16452 | -0.018 | 0.1406 | No |
| 23 | CH25H |  |  | 17654 | -0.043 | 0.0893 | No |
| 24 | NR4A3 |  |  | 19262 | -0.096 | 0.0270 | No |
| 25 | GBP2 |  |  | 19549 | -0.111 | 0.0314 | No |
| 26 | MX2 |  |  | 19725 | -0.121 | 0.0428 | No |
Table: GSEA details [plain text format]

  

Fig 2: CMV\_HCMV\_TIMECOURSE\_12HRS\_UP: Random ES distribution      
 Gene set null distribution of ES for **CMV\_HCMV\_TIMECOURSE\_12HRS\_UP**

  
