# Supplementary material for: The rapamycin-regulated gene expression signature determines prognosis for breast cancer
Source: Mol Cancer. 2009 Sep 24;8:75. doi: 10.1186/1476-4598-8-75 (PMC2761377; doi:10.1186/1476-4598-8-75)
Supplement: Additional file 3 — Gene set enrichment analysis of in vivo data, treatment series. The data provided represent the treatment series of GSEA. This compressed file contains "Treatment" shortcut file and "GSEA_treatment" folder. Clicking on "Treatment" shortcut opens the index file providing access to analysis files contained in the "GSEA_treatment" folder. [file 1476-4598-8-75-S3.zip › GSEA_treatment/CMV_UV-CMV_COMMON_HCMV_6HRS_DN.html]

Details for gene set CMV\_UV-CMV\_COMMON\_HCMV\_6HRS\_DN[GSEA]

|  || Dataset | gsea\_treatment\_collapsed |
| Phenotype | NoPhenotypeAvailable |
| Upregulated in class | na\_neg |
| GeneSet | CMV\_UV-CMV\_COMMON\_HCMV\_6HRS\_DN |
| Enrichment Score (ES) | -0.31310478 |
| Normalized Enrichment Score (NES) | -1.2335974 |
| Nominal p-value | 0.21052632 |
| FDR q-value | 0.27821723 |
| FWER p-Value | 1.0 |
Table: GSEA Results Summary

  

Fig 1: Enrichment plot: CMV\_UV-CMV\_COMMON\_HCMV\_6HRS\_DN      
 Profile of the Running ES Score & Positions of GeneSet Members on the Rank Ordered List

  

| PROBE | GENE SYMBOL | GENE\_TITLE | RANK IN GENE LIST | RANK METRIC SCORE | RUNNING ES | CORE ENRICHMENT || 1 | PDE4B |  |  | 616 | 0.382 | 0.0546 | No |
| 2 | RBM16 |  |  | 1672 | 0.274 | 0.0639 | No |
| 3 | DNMBP |  |  | 1998 | 0.255 | 0.1046 | No |
| 4 | SIAH1 |  |  | 2010 | 0.254 | 0.1603 | No |
| 5 | WEE1 |  |  | 2845 | 0.216 | 0.1677 | No |
| 6 | KLHL9 |  |  | 3009 | 0.210 | 0.2063 | No |
| 7 | PPP1R3C |  |  | 3228 | 0.202 | 0.2404 | No |
| 8 | MEIS1 |  |  | 3762 | 0.186 | 0.2557 | No |
| 9 | NR2F2 |  |  | 4891 | 0.158 | 0.2358 | No |
| 10 | CDC42EP2 |  |  | 5818 | 0.138 | 0.2213 | No |
| 11 | SOCS5 |  |  | 6968 | 0.118 | 0.1917 | No |
| 12 | RUNX1 |  |  | 9041 | 0.086 | 0.1101 | No |
| 13 | TCF21 |  |  | 10527 | 0.066 | 0.0525 | No |
| 14 | CXCL12 |  |  | 10698 | 0.063 | 0.0583 | No |
| 15 | MN1 |  |  | 11059 | 0.059 | 0.0538 | No |
| 16 | RND3 |  |  | 11199 | 0.057 | 0.0597 | No |
| 17 | STARD13 |  |  | 11504 | 0.053 | 0.0567 | No |
| 18 | RUNX1T1 |  |  | 11601 | 0.052 | 0.0635 | No |
| 19 | RNF113A |  |  | 11897 | 0.048 | 0.0598 | No |
| 20 | HOXA11 |  |  | 12086 | 0.046 | 0.0608 | No |
| 21 | GAS1 |  |  | 14002 | 0.021 | -0.0277 | No |
| 22 | BDKRB2 |  |  | 14390 | 0.015 | -0.0432 | No |
| 23 | ID2 |  |  | 15550 | -0.003 | -0.0989 | No |
| 24 | KLF10 |  |  | 15567 | -0.003 | -0.0990 | No |
| 25 | SNAI2 |  |  | 19844 | -0.131 | -0.2778 | Yes |
| 26 | ADM |  |  | 20571 | -0.495 | -0.2035 | Yes |
| 27 | DDIT4 |  |  | 20600 | -0.927 | 0.0002 | Yes |
Table: GSEA details [plain text format]

  

Fig 2: CMV\_UV-CMV\_COMMON\_HCMV\_6HRS\_DN: Random ES distribution      
 Gene set null distribution of ES for **CMV\_UV-CMV\_COMMON\_HCMV\_6HRS\_DN**

  
