# Supplementary material for: The rapamycin-regulated gene expression signature determines prognosis for breast cancer
Source: Mol Cancer. 2009 Sep 24;8:75. doi: 10.1186/1476-4598-8-75 (PMC2761377; doi:10.1186/1476-4598-8-75)
Supplement: Additional file 3 — Gene set enrichment analysis of in vivo data, treatment series. The data provided represent the treatment series of GSEA. This compressed file contains "Treatment" shortcut file and "GSEA_treatment" folder. Clicking on "Treatment" shortcut opens the index file providing access to analysis files contained in the "GSEA_treatment" folder. [file 1476-4598-8-75-S3.zip › GSEA_treatment/CPR_LOW_LIVER_UP.html]

Details for gene set CPR\_LOW\_LIVER\_UP[GSEA]

|  || Dataset | gsea\_treatment\_collapsed |
| Phenotype | NoPhenotypeAvailable |
| Upregulated in class | na\_neg |
| GeneSet | CPR\_LOW\_LIVER\_UP |
| Enrichment Score (ES) | -0.69775814 |
| Normalized Enrichment Score (NES) | -2.461506 |
| Nominal p-value | 0.0 |
| FDR q-value | 0.0 |
| FWER p-Value | 0.0 |
Table: GSEA Results Summary

  

Fig 1: Enrichment plot: CPR\_LOW\_LIVER\_UP      
 Profile of the Running ES Score & Positions of GeneSet Members on the Rank Ordered List

  

| PROBE | GENE SYMBOL | GENE\_TITLE | RANK IN GENE LIST | RANK METRIC SCORE | RUNNING ES | CORE ENRICHMENT || 1 | ETHE1 |  |  | 5488 | 0.145 | -0.2161 | No |
| 2 | CES2 |  |  | 6229 | 0.131 | -0.2063 | No |
| 3 | ZNF236 |  |  | 8906 | 0.088 | -0.3056 | No |
| 4 | CYP2C9 |  |  | 9781 | 0.076 | -0.3214 | No |
| 5 | CYP7A1 |  |  | 9799 | 0.076 | -0.2958 | No |
| 6 | GCK |  |  | 12047 | 0.046 | -0.3888 | No |
| 7 | TCEA3 |  |  | 15272 | 0.001 | -0.5449 | No |
| 8 | FDPS |  |  | 18142 | -0.055 | -0.6650 | Yes |
| 9 | CYB5B |  |  | 18431 | -0.063 | -0.6569 | Yes |
| 10 | CYP2B6 |  |  | 19274 | -0.096 | -0.6642 | Yes |
| 11 | AQP8 |  |  | 19951 | -0.140 | -0.6483 | Yes |
| 12 | SC4MOL |  |  | 20208 | -0.175 | -0.5999 | Yes |
| 13 | SQLE |  |  | 20301 | -0.196 | -0.5362 | Yes |
| 14 | SCD |  |  | 20496 | -0.294 | -0.4431 | Yes |
| 15 | DHCR7 |  |  | 20521 | -0.327 | -0.3301 | Yes |
| 16 | CYP51A1 |  |  | 20531 | -0.354 | -0.2072 | Yes |
| 17 | HMGCS1 |  |  | 20582 | -0.605 | 0.0011 | Yes |
Table: GSEA details [plain text format]

  

Fig 2: CPR\_LOW\_LIVER\_UP: Random ES distribution      
 Gene set null distribution of ES for **CPR\_LOW\_LIVER\_UP**

  
