# Supplementary material for: The rapamycin-regulated gene expression signature determines prognosis for breast cancer
Source: Mol Cancer. 2009 Sep 24;8:75. doi: 10.1186/1476-4598-8-75 (PMC2761377; doi:10.1186/1476-4598-8-75)
Supplement: Additional file 3 — Gene set enrichment analysis of in vivo data, treatment series. The data provided represent the treatment series of GSEA. This compressed file contains "Treatment" shortcut file and "GSEA_treatment" folder. Clicking on "Treatment" shortcut opens the index file providing access to analysis files contained in the "GSEA_treatment" folder. [file 1476-4598-8-75-S3.zip › GSEA_treatment/CPR_NULL_LIVER_UP.html]

Details for gene set CPR\_NULL\_LIVER\_UP[GSEA]

|  || Dataset | gsea\_treatment\_collapsed |
| Phenotype | NoPhenotypeAvailable |
| Upregulated in class | na\_neg |
| GeneSet | CPR\_NULL\_LIVER\_UP |
| Enrichment Score (ES) | -0.52309644 |
| Normalized Enrichment Score (NES) | -1.9772087 |
| Nominal p-value | 0.0 |
| FDR q-value | 0.019471519 |
| FWER p-Value | 0.091 |
Table: GSEA Results Summary

  

Fig 1: Enrichment plot: CPR\_NULL\_LIVER\_UP      
 Profile of the Running ES Score & Positions of GeneSet Members on the Rank Ordered List

  

| PROBE | GENE SYMBOL | GENE\_TITLE | RANK IN GENE LIST | RANK METRIC SCORE | RUNNING ES | CORE ENRICHMENT || 1 | C11ORF54 |  |  | 272 | 0.460 | 0.0957 | No |
| 2 | HSPC159 |  |  | 5280 | 0.149 | -0.1124 | No |
| 3 | ETHE1 |  |  | 5488 | 0.145 | -0.0882 | No |
| 4 | CES2 |  |  | 6229 | 0.131 | -0.0931 | No |
| 5 | ZNF236 |  |  | 8906 | 0.088 | -0.2023 | No |
| 6 | ACTG1 |  |  | 9177 | 0.085 | -0.1954 | No |
| 7 | ENTPD5 |  |  | 9644 | 0.078 | -0.1996 | No |
| 8 | CYP2C9 |  |  | 9781 | 0.076 | -0.1881 | No |
| 9 | CYP7A1 |  |  | 9799 | 0.076 | -0.1709 | No |
| 10 | GADD45B |  |  | 11255 | 0.056 | -0.2283 | No |
| 11 | ASNS |  |  | 11946 | 0.048 | -0.2506 | No |
| 12 | CD36 |  |  | 12917 | 0.035 | -0.2894 | No |
| 13 | SLCO1A2 |  |  | 14830 | 0.009 | -0.3803 | No |
| 14 | TMEM97 |  |  | 15155 | 0.003 | -0.3952 | No |
| 15 | HMOX1 |  |  | 15526 | -0.002 | -0.4127 | No |
| 16 | TMEM176A |  |  | 16773 | -0.024 | -0.4675 | No |
| 17 | ANXA5 |  |  | 17826 | -0.047 | -0.5075 | Yes |
| 18 | CSAD |  |  | 17861 | -0.048 | -0.4978 | Yes |
| 19 | FDPS |  |  | 18142 | -0.055 | -0.4983 | Yes |
| 20 | CYB5B |  |  | 18431 | -0.063 | -0.4972 | Yes |
| 21 | LPL |  |  | 18964 | -0.083 | -0.5035 | Yes |
| 22 | DHCR24 |  |  | 19039 | -0.086 | -0.4868 | Yes |
| 23 | LGALS1 |  |  | 19156 | -0.091 | -0.4708 | Yes |
| 24 | CYP2B6 |  |  | 19274 | -0.096 | -0.4537 | Yes |
| 25 | UGDH |  |  | 19592 | -0.113 | -0.4423 | Yes |
| 26 | AHR |  |  | 19747 | -0.123 | -0.4207 | Yes |
| 27 | AQP8 |  |  | 19951 | -0.140 | -0.3974 | Yes |
| 28 | SC4MOL |  |  | 20208 | -0.175 | -0.3685 | Yes |
| 29 | CYP26A1 |  |  | 20272 | -0.188 | -0.3272 | Yes |
| 30 | SQLE |  |  | 20301 | -0.196 | -0.2822 | Yes |
| 31 | SCD |  |  | 20496 | -0.294 | -0.2219 | Yes |
| 32 | CYP51A1 |  |  | 20531 | -0.354 | -0.1397 | Yes |
| 33 | HMGCS1 |  |  | 20582 | -0.605 | 0.0011 | Yes |
Table: GSEA details [plain text format]

  

Fig 2: CPR\_NULL\_LIVER\_UP: Random ES distribution      
 Gene set null distribution of ES for **CPR\_NULL\_LIVER\_UP**

  
