# Supplementary material for: The rapamycin-regulated gene expression signature determines prognosis for breast cancer
Source: Mol Cancer. 2009 Sep 24;8:75. doi: 10.1186/1476-4598-8-75 (PMC2761377; doi:10.1186/1476-4598-8-75)
Supplement: Additional file 3 — Gene set enrichment analysis of in vivo data, treatment series. The data provided represent the treatment series of GSEA. This compressed file contains "Treatment" shortcut file and "GSEA_treatment" folder. Clicking on "Treatment" shortcut opens the index file providing access to analysis files contained in the "GSEA_treatment" folder. [file 1476-4598-8-75-S3.zip › GSEA_treatment/CROMER_HYPOPHARYNGEAL_MET_VS_NON_UP.html]

Details for gene set CROMER\_HYPOPHARYNGEAL\_MET\_VS\_NON\_UP[GSEA]

|  || Dataset | gsea\_treatment\_collapsed |
| Phenotype | NoPhenotypeAvailable |
| Upregulated in class | na\_pos |
| GeneSet | CROMER\_HYPOPHARYNGEAL\_MET\_VS\_NON\_UP |
| Enrichment Score (ES) | 0.58766687 |
| Normalized Enrichment Score (NES) | 1.7243096 |
| Nominal p-value | 0.0 |
| FDR q-value | 0.009871958 |
| FWER p-Value | 0.294 |
Table: GSEA Results Summary

  

Fig 1: Enrichment plot: CROMER\_HYPOPHARYNGEAL\_MET\_VS\_NON\_UP      
 Profile of the Running ES Score & Positions of GeneSet Members on the Rank Ordered List

  

| PROBE | GENE SYMBOL | GENE\_TITLE | RANK IN GENE LIST | RANK METRIC SCORE | RUNNING ES | CORE ENRICHMENT || 1 | CRI1 |  |  | 47 | 0.687 | 0.0503 | Yes |
| 2 | IL8 |  |  | 76 | 0.611 | 0.0957 | Yes |
| 3 | PSMB9 |  |  | 120 | 0.554 | 0.1361 | Yes |
| 4 | SH3BGRL |  |  | 131 | 0.542 | 0.1771 | Yes |
| 5 | COL4A1 |  |  | 681 | 0.369 | 0.1786 | Yes |
| 6 | SMC1A |  |  | 778 | 0.356 | 0.2012 | Yes |
| 7 | KIFAP3 |  |  | 797 | 0.354 | 0.2274 | Yes |
| 8 | MAGED2 |  |  | 907 | 0.341 | 0.2482 | Yes |
| 9 | CD164 |  |  | 921 | 0.339 | 0.2735 | Yes |
| 10 | PPT1 |  |  | 945 | 0.337 | 0.2982 | Yes |
| 11 | DEK |  |  | 988 | 0.333 | 0.3216 | Yes |
| 12 | NFYC |  |  | 1181 | 0.313 | 0.3362 | Yes |
| 13 | PARP1 |  |  | 1466 | 0.289 | 0.3445 | Yes |
| 14 | HSF2 |  |  | 1495 | 0.287 | 0.3651 | Yes |
| 15 | FCGRT |  |  | 1542 | 0.284 | 0.3846 | Yes |
| 16 | ACVR1B |  |  | 1792 | 0.267 | 0.3929 | Yes |
| 17 | KIAA1840 |  |  | 1870 | 0.263 | 0.4093 | Yes |
| 18 | MAGED1 |  |  | 1892 | 0.261 | 0.4282 | Yes |
| 19 | GNS |  |  | 1961 | 0.257 | 0.4446 | Yes |
| 20 | TMEM109 |  |  | 2077 | 0.252 | 0.4583 | Yes |
| 21 | GANAB |  |  | 2260 | 0.242 | 0.4679 | Yes |
| 22 | C5ORF15 |  |  | 2706 | 0.223 | 0.4633 | Yes |
| 23 | GALNT10 |  |  | 2816 | 0.217 | 0.4747 | Yes |
| 24 | BUB3 |  |  | 2885 | 0.215 | 0.4878 | Yes |
| 25 | SLC30A1 |  |  | 3042 | 0.209 | 0.4962 | Yes |
| 26 | CTNNB1 |  |  | 3054 | 0.208 | 0.5116 | Yes |
| 27 | TFAP2C |  |  | 3182 | 0.203 | 0.5210 | Yes |
| 28 | GPR107 |  |  | 3294 | 0.200 | 0.5309 | Yes |
| 29 | SH3BP5 |  |  | 3747 | 0.187 | 0.5232 | Yes |
| 30 | GNB2 |  |  | 3750 | 0.187 | 0.5373 | Yes |
| 31 | CD46 |  |  | 3833 | 0.184 | 0.5474 | Yes |
| 32 | TMEM123 |  |  | 3989 | 0.179 | 0.5536 | Yes |
| 33 | MDC1 |  |  | 4034 | 0.178 | 0.5651 | Yes |
| 34 | CNOT2 |  |  | 4312 | 0.171 | 0.5648 | Yes |
| 35 | FADS1 |  |  | 4351 | 0.170 | 0.5759 | Yes |
| 36 | PGRMC1 |  |  | 4587 | 0.165 | 0.5771 | Yes |
| 37 | C10ORF10 |  |  | 4764 | 0.160 | 0.5808 | Yes |
| 38 | JARID1C |  |  | 4873 | 0.158 | 0.5877 | Yes |
| 39 | PTPRK |  |  | 5366 | 0.148 | 0.5750 | No |
| 40 | XPO1 |  |  | 5531 | 0.144 | 0.5780 | No |
| 41 | GTSE1 |  |  | 5649 | 0.141 | 0.5832 | No |
| 42 | MFGE8 |  |  | 6071 | 0.134 | 0.5729 | No |
| 43 | TMEM63A |  |  | 6642 | 0.125 | 0.5547 | No |
| 44 | ENPP2 |  |  | 6815 | 0.122 | 0.5556 | No |
| 45 | BMI1 |  |  | 7236 | 0.114 | 0.5439 | No |
| 46 | FADS2 |  |  | 7617 | 0.108 | 0.5336 | No |
| 47 | UBXD2 |  |  | 8223 | 0.098 | 0.5117 | No |
| 48 | ATRX |  |  | 8431 | 0.095 | 0.5089 | No |
| 49 | ZHX3 |  |  | 9231 | 0.084 | 0.4764 | No |
| 50 | ARHGDIB |  |  | 9762 | 0.076 | 0.4564 | No |
| 51 | MICB |  |  | 9915 | 0.075 | 0.4547 | No |
| 52 | WAS |  |  | 10906 | 0.061 | 0.4112 | No |
| 53 | DYRK1A |  |  | 11112 | 0.058 | 0.4057 | No |
| 54 | SLC25A36 |  |  | 11642 | 0.051 | 0.3839 | No |
| 55 | GHRH |  |  | 12132 | 0.045 | 0.3635 | No |
| 56 | BEXL1 |  |  | 12176 | 0.044 | 0.3648 | No |
| 57 | TCF12 |  |  | 13110 | 0.032 | 0.3218 | No |
| 58 | CDH11 |  |  | 13551 | 0.027 | 0.3025 | No |
| 59 | GGPS1 |  |  | 13961 | 0.021 | 0.2842 | No |
| 60 | CCDC131 |  |  | 14483 | 0.013 | 0.2598 | No |
| 61 | MTMR2 |  |  | 14488 | 0.013 | 0.2607 | No |
| 62 | DHRSX |  |  | 14499 | 0.013 | 0.2612 | No |
| 63 | CXCR4 |  |  | 14667 | 0.011 | 0.2539 | No |
| 64 | ID2 |  |  | 15550 | -0.003 | 0.2112 | No |
| 65 | TUBB2B |  |  | 16608 | -0.021 | 0.1613 | No |
| 66 | CCND3 |  |  | 16814 | -0.025 | 0.1532 | No |
| 67 | GABBR1 |  |  | 17198 | -0.033 | 0.1371 | No |
| 68 | PPID |  |  | 17555 | -0.041 | 0.1229 | No |
| 69 | PPIH |  |  | 17674 | -0.044 | 0.1205 | No |
| 70 | TSC22D3 |  |  | 18666 | -0.071 | 0.0777 | No |
| 71 | PLEKHC1 |  |  | 19410 | -0.103 | 0.0495 | No |
| 72 | CNN3 |  |  | 19594 | -0.113 | 0.0492 | No |
Table: GSEA details [plain text format]

  

Fig 2: CROMER\_HYPOPHARYNGEAL\_MET\_VS\_NON\_UP: Random ES distribution      
 Gene set null distribution of ES for **CROMER\_HYPOPHARYNGEAL\_MET\_VS\_NON\_UP**

  
