# Supplementary material for: The rapamycin-regulated gene expression signature determines prognosis for breast cancer
Source: Mol Cancer. 2009 Sep 24;8:75. doi: 10.1186/1476-4598-8-75 (PMC2761377; doi:10.1186/1476-4598-8-75)
Supplement: Additional file 3 — Gene set enrichment analysis of in vivo data, treatment series. The data provided represent the treatment series of GSEA. This compressed file contains "Treatment" shortcut file and "GSEA_treatment" folder. Clicking on "Treatment" shortcut opens the index file providing access to analysis files contained in the "GSEA_treatment" folder. [file 1476-4598-8-75-S3.zip › GSEA_treatment/CROONQUIST_RAS_STROMA_DN.html]

Details for gene set CROONQUIST\_RAS\_STROMA\_DN[GSEA]

|  || Dataset | gsea\_treatment\_collapsed |
| Phenotype | NoPhenotypeAvailable |
| Upregulated in class | na\_pos |
| GeneSet | CROONQUIST\_RAS\_STROMA\_DN |
| Enrichment Score (ES) | 0.69440734 |
| Normalized Enrichment Score (NES) | 1.734266 |
| Nominal p-value | 0.0010822511 |
| FDR q-value | 0.008909811 |
| FWER p-Value | 0.252 |
Table: GSEA Results Summary

  

Fig 1: Enrichment plot: CROONQUIST\_RAS\_STROMA\_DN      
 Profile of the Running ES Score & Positions of GeneSet Members on the Rank Ordered List

  

| PROBE | GENE SYMBOL | GENE\_TITLE | RANK IN GENE LIST | RANK METRIC SCORE | RUNNING ES | CORE ENRICHMENT || 1 | CTGF |  |  | 12 | 0.922 | 0.2432 | Yes |
| 2 | IGFBP7 |  |  | 44 | 0.693 | 0.4248 | Yes |
| 3 | CD81 |  |  | 693 | 0.368 | 0.4906 | Yes |
| 4 | TGFBI |  |  | 811 | 0.352 | 0.5781 | Yes |
| 5 | ACTA2 |  |  | 1058 | 0.325 | 0.6521 | Yes |
| 6 | SPARC |  |  | 1676 | 0.274 | 0.6944 | Yes |
| 7 | IL6 |  |  | 5899 | 0.137 | 0.5254 | No |
| 8 | RPS4Y1 |  |  | 6215 | 0.131 | 0.5448 | No |
| 9 | GJA1 |  |  | 12062 | 0.046 | 0.2729 | No |
| 10 | IGFBP3 |  |  | 12384 | 0.042 | 0.2684 | No |
| 11 | SULF1 |  |  | 12751 | 0.037 | 0.2604 | No |
| 12 | POSTN |  |  | 14063 | 0.020 | 0.2019 | No |
| 13 | TPM2 |  |  | 15180 | 0.003 | 0.1486 | No |
| 14 | FSCN1 |  |  | 15375 | -0.000 | 0.1392 | No |
| 15 | NR4A2 |  |  | 16771 | -0.024 | 0.0778 | No |
| 16 | COL1A2 |  |  | 18136 | -0.055 | 0.0261 | No |
| 17 | COL6A3 |  |  | 18784 | -0.076 | 0.0146 | No |
| 18 | DUSP1 |  |  | 18808 | -0.076 | 0.0337 | No |
| 19 | FOS |  |  | 20334 | -0.202 | 0.0132 | No |
Table: GSEA details [plain text format]

  

Fig 2: CROONQUIST\_RAS\_STROMA\_DN: Random ES distribution      
 Gene set null distribution of ES for **CROONQUIST\_RAS\_STROMA\_DN**

  
