# Supplementary material for: The rapamycin-regulated gene expression signature determines prognosis for breast cancer
Source: Mol Cancer. 2009 Sep 24;8:75. doi: 10.1186/1476-4598-8-75 (PMC2761377; doi:10.1186/1476-4598-8-75)
Supplement: Additional file 3 — Gene set enrichment analysis of in vivo data, treatment series. The data provided represent the treatment series of GSEA. This compressed file contains "Treatment" shortcut file and "GSEA_treatment" folder. Clicking on "Treatment" shortcut opens the index file providing access to analysis files contained in the "GSEA_treatment" folder. [file 1476-4598-8-75-S3.zip › GSEA_treatment/CTLA4PATHWAY.html]

Details for gene set CTLA4PATHWAY[GSEA]

|  || Dataset | gsea\_treatment\_collapsed |
| Phenotype | NoPhenotypeAvailable |
| Upregulated in class | na\_neg |
| GeneSet | CTLA4PATHWAY |
| Enrichment Score (ES) | -0.26396775 |
| Normalized Enrichment Score (NES) | -0.9318647 |
| Nominal p-value | 0.5913978 |
| FDR q-value | 0.67843086 |
| FWER p-Value | 1.0 |
Table: GSEA Results Summary

  

Fig 1: Enrichment plot: CTLA4PATHWAY      
 Profile of the Running ES Score & Positions of GeneSet Members on the Rank Ordered List

  

| PROBE | GENE SYMBOL | GENE\_TITLE | RANK IN GENE LIST | RANK METRIC SCORE | RUNNING ES | CORE ENRICHMENT || 1 | HLA-DRB1 |  |  | 4173 | 0.175 | -0.0849 | No |
| 2 | PTPN11 |  |  | 4319 | 0.171 | 0.0234 | No |
| 3 | TRA@ |  |  | 8005 | 0.102 | -0.0870 | No |
| 4 | GRB2 |  |  | 9226 | 0.084 | -0.0899 | No |
| 5 | CD28 |  |  | 9802 | 0.076 | -0.0667 | No |
| 6 | HLA-DRA |  |  | 9918 | 0.074 | -0.0224 | No |
| 7 | LCK |  |  | 10056 | 0.072 | 0.0195 | No |
| 8 | CD3G |  |  | 12673 | 0.038 | -0.0820 | No |
| 9 | PIK3CA |  |  | 12896 | 0.035 | -0.0692 | No |
| 10 | CTLA4 |  |  | 13167 | 0.032 | -0.0609 | No |
| 11 | IL2 |  |  | 14227 | 0.017 | -0.1007 | No |
| 12 | CD3D |  |  | 14533 | 0.013 | -0.1068 | No |
| 13 | PIK3R1 |  |  | 14620 | 0.012 | -0.1032 | No |
| 14 | CD86 |  |  | 17931 | -0.050 | -0.2303 | Yes |
| 15 | ICOS |  |  | 18276 | -0.059 | -0.2075 | Yes |
| 16 | CD80 |  |  | 18514 | -0.066 | -0.1747 | Yes |
| 17 | CD3E |  |  | 19706 | -0.120 | -0.1519 | Yes |
| 18 | ITK |  |  | 20492 | -0.290 | 0.0055 | Yes |
Table: GSEA details [plain text format]

  

Fig 2: CTLA4PATHWAY: Random ES distribution      
 Gene set null distribution of ES for **CTLA4PATHWAY**

  
