# Supplementary material for: The rapamycin-regulated gene expression signature determines prognosis for breast cancer
Source: Mol Cancer. 2009 Sep 24;8:75. doi: 10.1186/1476-4598-8-75 (PMC2761377; doi:10.1186/1476-4598-8-75)
Supplement: Additional file 3 — Gene set enrichment analysis of in vivo data, treatment series. The data provided represent the treatment series of GSEA. This compressed file contains "Treatment" shortcut file and "GSEA_treatment" folder. Clicking on "Treatment" shortcut opens the index file providing access to analysis files contained in the "GSEA_treatment" folder. [file 1476-4598-8-75-S3.zip › GSEA_treatment/DAC_BLADDER_UP.html]

Details for gene set DAC\_BLADDER\_UP[GSEA]

|  || Dataset | gsea\_treatment\_collapsed |
| Phenotype | NoPhenotypeAvailable |
| Upregulated in class | na\_pos |
| GeneSet | DAC\_BLADDER\_UP |
| Enrichment Score (ES) | 0.7361598 |
| Normalized Enrichment Score (NES) | 1.942238 |
| Nominal p-value | 0.0 |
| FDR q-value | 4.487126E-4 |
| FWER p-Value | 0.0050 |
Table: GSEA Results Summary

  

Fig 1: Enrichment plot: DAC\_BLADDER\_UP      
 Profile of the Running ES Score & Positions of GeneSet Members on the Rank Ordered List

  

| PROBE | GENE SYMBOL | GENE\_TITLE | RANK IN GENE LIST | RANK METRIC SCORE | RUNNING ES | CORE ENRICHMENT || 1 | NT5E |  |  | 2 | 1.142 | 0.1590 | Yes |
| 2 | MX1 |  |  | 24 | 0.773 | 0.2656 | Yes |
| 3 | TNFSF10 |  |  | 160 | 0.518 | 0.3312 | Yes |
| 4 | OAS1 |  |  | 255 | 0.466 | 0.3916 | Yes |
| 5 | CCL20 |  |  | 301 | 0.453 | 0.4524 | Yes |
| 6 | TNFAIP3 |  |  | 406 | 0.424 | 0.5064 | Yes |
| 7 | ICAM1 |  |  | 408 | 0.423 | 0.5653 | Yes |
| 8 | CXCL2 |  |  | 457 | 0.412 | 0.6204 | Yes |
| 9 | IRF7 |  |  | 639 | 0.378 | 0.6642 | Yes |
| 10 | C1S |  |  | 714 | 0.366 | 0.7116 | Yes |
| 11 | STAT1 |  |  | 1401 | 0.294 | 0.7192 | Yes |
| 12 | IFIT3 |  |  | 1814 | 0.265 | 0.7362 | Yes |
| 13 | DHRS2 |  |  | 2762 | 0.220 | 0.7207 | No |
| 14 | STX3 |  |  | 4250 | 0.173 | 0.6725 | No |
| 15 | BIRC4BP |  |  | 7047 | 0.117 | 0.5530 | No |
| 16 | CCL5 |  |  | 8249 | 0.098 | 0.5082 | No |
| 17 | UCHL1 |  |  | 8269 | 0.098 | 0.5209 | No |
| 18 | STC1 |  |  | 9045 | 0.086 | 0.4952 | No |
| 19 | HBEGF |  |  | 9327 | 0.082 | 0.4930 | No |
| 20 | HIST2H2AA3 |  |  | 9462 | 0.080 | 0.4977 | No |
| 21 | OAS2 |  |  | 12543 | 0.040 | 0.3536 | No |
| 22 | IFI44L |  |  | 14255 | 0.017 | 0.2729 | No |
| 23 | DAZL |  |  | 14377 | 0.015 | 0.2691 | No |
| 24 | SLPI |  |  | 14802 | 0.009 | 0.2498 | No |
| 25 | HIST1H2AC |  |  | 16391 | -0.017 | 0.1750 | No |
| 26 | MMP1 |  |  | 16811 | -0.025 | 0.1580 | No |
| 27 | IL13RA2 |  |  | 18562 | -0.067 | 0.0824 | No |
| 28 | MX2 |  |  | 19725 | -0.121 | 0.0428 | No |
Table: GSEA details [plain text format]

  

Fig 2: DAC\_BLADDER\_UP: Random ES distribution      
 Gene set null distribution of ES for **DAC\_BLADDER\_UP**

  
