# Supplementary material for: The rapamycin-regulated gene expression signature determines prognosis for breast cancer
Source: Mol Cancer. 2009 Sep 24;8:75. doi: 10.1186/1476-4598-8-75 (PMC2761377; doi:10.1186/1476-4598-8-75)
Supplement: Additional file 3 — Gene set enrichment analysis of in vivo data, treatment series. The data provided represent the treatment series of GSEA. This compressed file contains "Treatment" shortcut file and "GSEA_treatment" folder. Clicking on "Treatment" shortcut opens the index file providing access to analysis files contained in the "GSEA_treatment" folder. [file 1476-4598-8-75-S3.zip › GSEA_treatment/DAC_IFN_BLADDER_UP.html]

Details for gene set DAC\_IFN\_BLADDER\_UP[GSEA]

|  || Dataset | gsea\_treatment\_collapsed |
| Phenotype | NoPhenotypeAvailable |
| Upregulated in class | na\_pos |
| GeneSet | DAC\_IFN\_BLADDER\_UP |
| Enrichment Score (ES) | 0.84676 |
| Normalized Enrichment Score (NES) | 2.0430908 |
| Nominal p-value | 0.0 |
| FDR q-value | 0.0 |
| FWER p-Value | 0.0 |
Table: GSEA Results Summary

  

Fig 1: Enrichment plot: DAC\_IFN\_BLADDER\_UP      
 Profile of the Running ES Score & Positions of GeneSet Members on the Rank Ordered List

  

| PROBE | GENE SYMBOL | GENE\_TITLE | RANK IN GENE LIST | RANK METRIC SCORE | RUNNING ES | CORE ENRICHMENT || 1 | MX1 |  |  | 24 | 0.773 | 0.1501 | Yes |
| 2 | TNFSF10 |  |  | 160 | 0.518 | 0.2450 | Yes |
| 3 | OAS1 |  |  | 255 | 0.466 | 0.3317 | Yes |
| 4 | CCL20 |  |  | 301 | 0.453 | 0.4182 | Yes |
| 5 | TNFAIP3 |  |  | 406 | 0.424 | 0.4962 | Yes |
| 6 | ICAM1 |  |  | 408 | 0.423 | 0.5790 | Yes |
| 7 | CXCL2 |  |  | 457 | 0.412 | 0.6573 | Yes |
| 8 | IRF7 |  |  | 639 | 0.378 | 0.7224 | Yes |
| 9 | C1S |  |  | 714 | 0.366 | 0.7905 | Yes |
| 10 | STAT1 |  |  | 1401 | 0.294 | 0.8148 | Yes |
| 11 | IFIT3 |  |  | 1814 | 0.265 | 0.8468 | Yes |
| 12 | CCL5 |  |  | 8249 | 0.098 | 0.5534 | No |
| 13 | OAS2 |  |  | 12543 | 0.040 | 0.3527 | No |
| 14 | SLPI |  |  | 14802 | 0.009 | 0.2448 | No |
| 15 | IL13RA2 |  |  | 18562 | -0.067 | 0.0754 | No |
| 16 | MX2 |  |  | 19725 | -0.121 | 0.0427 | No |
Table: GSEA details [plain text format]

  

Fig 2: DAC\_IFN\_BLADDER\_UP: Random ES distribution      
 Gene set null distribution of ES for **DAC\_IFN\_BLADDER\_UP**

  
