# Supplementary material for: The rapamycin-regulated gene expression signature determines prognosis for breast cancer
Source: Mol Cancer. 2009 Sep 24;8:75. doi: 10.1186/1476-4598-8-75 (PMC2761377; doi:10.1186/1476-4598-8-75)
Supplement: Additional file 3 — Gene set enrichment analysis of in vivo data, treatment series. The data provided represent the treatment series of GSEA. This compressed file contains "Treatment" shortcut file and "GSEA_treatment" folder. Clicking on "Treatment" shortcut opens the index file providing access to analysis files contained in the "GSEA_treatment" folder. [file 1476-4598-8-75-S3.zip › GSEA_treatment/DER_IFNA_UP.html]

Details for gene set DER\_IFNA\_UP[GSEA]

|  || Dataset | gsea\_treatment\_collapsed |
| Phenotype | NoPhenotypeAvailable |
| Upregulated in class | na\_pos |
| GeneSet | DER\_IFNA\_UP |
| Enrichment Score (ES) | 0.70274675 |
| Normalized Enrichment Score (NES) | 2.042719 |
| Nominal p-value | 0.0 |
| FDR q-value | 0.0 |
| FWER p-Value | 0.0 |
Table: GSEA Results Summary

  

Fig 1: Enrichment plot: DER\_IFNA\_UP      
 Profile of the Running ES Score & Positions of GeneSet Members on the Rank Ordered List

  

| PROBE | GENE SYMBOL | GENE\_TITLE | RANK IN GENE LIST | RANK METRIC SCORE | RUNNING ES | CORE ENRICHMENT || 1 | TRIM22 |  |  | 8 | 1.024 | 0.0586 | Yes |
| 2 | MX1 |  |  | 24 | 0.773 | 0.1025 | Yes |
| 3 | HLA-B |  |  | 41 | 0.695 | 0.1418 | Yes |
| 4 | ISG15 |  |  | 59 | 0.644 | 0.1781 | Yes |
| 5 | IFITM1 |  |  | 79 | 0.609 | 0.2123 | Yes |
| 6 | HLA-E |  |  | 126 | 0.545 | 0.2415 | Yes |
| 7 | GBP1 |  |  | 130 | 0.542 | 0.2726 | Yes |
| 8 | HLA-A |  |  | 195 | 0.496 | 0.2980 | Yes |
| 9 | OAS1 |  |  | 255 | 0.466 | 0.3221 | Yes |
| 10 | ZFP36L2 |  |  | 352 | 0.438 | 0.3426 | Yes |
| 11 | PDXK |  |  | 402 | 0.425 | 0.3648 | Yes |
| 12 | PSMB8 |  |  | 426 | 0.420 | 0.3879 | Yes |
| 13 | PSMB10 |  |  | 447 | 0.415 | 0.4108 | Yes |
| 14 | RBBP4 |  |  | 474 | 0.410 | 0.4332 | Yes |
| 15 | PSME1 |  |  | 513 | 0.402 | 0.4545 | Yes |
| 16 | IRF1 |  |  | 551 | 0.393 | 0.4754 | Yes |
| 17 | TAP1 |  |  | 556 | 0.393 | 0.4978 | Yes |
| 18 | BST2 |  |  | 657 | 0.374 | 0.5145 | Yes |
| 19 | C1S |  |  | 714 | 0.366 | 0.5329 | Yes |
| 20 | IFI6 |  |  | 726 | 0.364 | 0.5533 | Yes |
| 21 | IFI35 |  |  | 766 | 0.357 | 0.5720 | Yes |
| 22 | SKP1A |  |  | 832 | 0.349 | 0.5889 | Yes |
| 23 | ADAR |  |  | 885 | 0.344 | 0.6062 | Yes |
| 24 | CD164 |  |  | 921 | 0.339 | 0.6241 | Yes |
| 25 | IFI44 |  |  | 1039 | 0.327 | 0.6373 | Yes |
| 26 | PLOD2 |  |  | 1140 | 0.316 | 0.6506 | Yes |
| 27 | STAT1 |  |  | 1401 | 0.294 | 0.6549 | Yes |
| 28 | IRF2 |  |  | 1500 | 0.287 | 0.6667 | Yes |
| 29 | IFIT3 |  |  | 1814 | 0.265 | 0.6668 | Yes |
| 30 | SFRS2 |  |  | 2044 | 0.253 | 0.6702 | Yes |
| 31 | BBC3 |  |  | 2497 | 0.230 | 0.6615 | Yes |
| 32 | BTG1 |  |  | 2593 | 0.227 | 0.6699 | Yes |
| 33 | SRP9 |  |  | 2599 | 0.226 | 0.6827 | Yes |
| 34 | CYCS |  |  | 2626 | 0.226 | 0.6944 | Yes |
| 35 | CASP8 |  |  | 2719 | 0.222 | 0.7027 | Yes |
| 36 | FAS |  |  | 3778 | 0.186 | 0.6619 | No |
| 37 | SDCBP |  |  | 3795 | 0.185 | 0.6718 | No |
| 38 | LIPA |  |  | 4577 | 0.165 | 0.6433 | No |
| 39 | BAG1 |  |  | 4977 | 0.156 | 0.6329 | No |
| 40 | TRIM14 |  |  | 4986 | 0.156 | 0.6415 | No |
| 41 | PML |  |  | 5234 | 0.150 | 0.6381 | No |
| 42 | SHFM1 |  |  | 5405 | 0.147 | 0.6383 | No |
| 43 | EPS15 |  |  | 5695 | 0.140 | 0.6323 | No |
| 44 | IL6 |  |  | 5899 | 0.137 | 0.6303 | No |
| 45 | RHOC |  |  | 7051 | 0.117 | 0.5810 | No |
| 46 | POLR2B |  |  | 7649 | 0.107 | 0.5581 | No |
| 47 | XRCC6 |  |  | 7760 | 0.106 | 0.5589 | No |
| 48 | MAP3K10 |  |  | 8577 | 0.093 | 0.5245 | No |
| 49 | TAS2R5 |  |  | 9222 | 0.084 | 0.4980 | No |
| 50 | GMPR |  |  | 10165 | 0.071 | 0.4562 | No |
| 51 | PPP3CA |  |  | 11496 | 0.053 | 0.3945 | No |
| 52 | DDX17 |  |  | 11694 | 0.051 | 0.3879 | No |
| 53 | ATP6V0B |  |  | 12039 | 0.046 | 0.3738 | No |
| 54 | OASL |  |  | 12433 | 0.041 | 0.3570 | No |
| 55 | OAS2 |  |  | 12543 | 0.040 | 0.3540 | No |
| 56 | PYHIN1 |  |  | 13629 | 0.026 | 0.3027 | No |
| 57 | TEAD4 |  |  | 13666 | 0.025 | 0.3024 | No |
| 58 | EIF2B1 |  |  | 13897 | 0.022 | 0.2925 | No |
| 59 | ELAC1 |  |  | 14639 | 0.011 | 0.2570 | No |
| 60 | NMI |  |  | 16092 | -0.012 | 0.1870 | No |
| 61 | TRIM21 |  |  | 16730 | -0.024 | 0.1574 | No |
| 62 | CSRP3 |  |  | 16774 | -0.024 | 0.1567 | No |
| 63 | COL16A1 |  |  | 17177 | -0.033 | 0.1390 | No |
| 64 | VEGFC |  |  | 19585 | -0.113 | 0.0283 | No |
| 65 | MX2 |  |  | 19725 | -0.121 | 0.0285 | No |
| 66 | PMAIP1 |  |  | 20440 | -0.248 | 0.0080 | No |
Table: GSEA details [plain text format]

  

Fig 2: DER\_IFNA\_UP: Random ES distribution      
 Gene set null distribution of ES for **DER\_IFNA\_UP**

  
