# Supplementary material for: The rapamycin-regulated gene expression signature determines prognosis for breast cancer
Source: Mol Cancer. 2009 Sep 24;8:75. doi: 10.1186/1476-4598-8-75 (PMC2761377; doi:10.1186/1476-4598-8-75)
Supplement: Additional file 3 — Gene set enrichment analysis of in vivo data, treatment series. The data provided represent the treatment series of GSEA. This compressed file contains "Treatment" shortcut file and "GSEA_treatment" folder. Clicking on "Treatment" shortcut opens the index file providing access to analysis files contained in the "GSEA_treatment" folder. [file 1476-4598-8-75-S3.zip › GSEA_treatment/DER_IFNG_UP.html]

Details for gene set DER\_IFNG\_UP[GSEA]

|  || Dataset | gsea\_treatment\_collapsed |
| Phenotype | NoPhenotypeAvailable |
| Upregulated in class | na\_pos |
| GeneSet | DER\_IFNG\_UP |
| Enrichment Score (ES) | 0.66669244 |
| Normalized Enrichment Score (NES) | 1.9194344 |
| Nominal p-value | 0.0 |
| FDR q-value | 4.944348E-4 |
| FWER p-Value | 0.0070 |
Table: GSEA Results Summary

  

Fig 1: Enrichment plot: DER\_IFNG\_UP      
 Profile of the Running ES Score & Positions of GeneSet Members on the Rank Ordered List

  

| PROBE | GENE SYMBOL | GENE\_TITLE | RANK IN GENE LIST | RANK METRIC SCORE | RUNNING ES | CORE ENRICHMENT || 1 | HLA-B |  |  | 41 | 0.695 | 0.0442 | Yes |
| 2 | ISG15 |  |  | 59 | 0.644 | 0.0862 | Yes |
| 3 | IFITM1 |  |  | 79 | 0.609 | 0.1257 | Yes |
| 4 | HLA-E |  |  | 126 | 0.545 | 0.1597 | Yes |
| 5 | GBP1 |  |  | 130 | 0.542 | 0.1956 | Yes |
| 6 | HLA-A |  |  | 195 | 0.496 | 0.2254 | Yes |
| 7 | ZFP36L2 |  |  | 352 | 0.438 | 0.2469 | Yes |
| 8 | ICAM1 |  |  | 408 | 0.423 | 0.2724 | Yes |
| 9 | PSMB8 |  |  | 426 | 0.420 | 0.2994 | Yes |
| 10 | PSMB10 |  |  | 447 | 0.415 | 0.3260 | Yes |
| 11 | RBBP4 |  |  | 474 | 0.410 | 0.3520 | Yes |
| 12 | PSME1 |  |  | 513 | 0.402 | 0.3769 | Yes |
| 13 | IRF1 |  |  | 551 | 0.393 | 0.4012 | Yes |
| 14 | TAP1 |  |  | 556 | 0.393 | 0.4271 | Yes |
| 15 | BST2 |  |  | 657 | 0.374 | 0.4471 | Yes |
| 16 | C1S |  |  | 714 | 0.366 | 0.4687 | Yes |
| 17 | IFI35 |  |  | 766 | 0.357 | 0.4899 | Yes |
| 18 | SKP1A |  |  | 832 | 0.349 | 0.5099 | Yes |
| 19 | ADAR |  |  | 885 | 0.344 | 0.5302 | Yes |
| 20 | PLOD2 |  |  | 1140 | 0.316 | 0.5389 | Yes |
| 21 | HADH |  |  | 1330 | 0.300 | 0.5496 | Yes |
| 22 | STAT1 |  |  | 1401 | 0.294 | 0.5658 | Yes |
| 23 | PARP1 |  |  | 1466 | 0.289 | 0.5818 | Yes |
| 24 | SF3A1 |  |  | 1513 | 0.286 | 0.5986 | Yes |
| 25 | IFIT3 |  |  | 1814 | 0.265 | 0.6016 | Yes |
| 26 | HADHB |  |  | 2028 | 0.254 | 0.6081 | Yes |
| 27 | SFRS2 |  |  | 2044 | 0.253 | 0.6242 | Yes |
| 28 | BBC3 |  |  | 2497 | 0.230 | 0.6175 | Yes |
| 29 | BTG1 |  |  | 2593 | 0.227 | 0.6279 | Yes |
| 30 | SRP9 |  |  | 2599 | 0.226 | 0.6427 | Yes |
| 31 | CYCS |  |  | 2626 | 0.226 | 0.6564 | Yes |
| 32 | CASP8 |  |  | 2719 | 0.222 | 0.6667 | Yes |
| 33 | ELK4 |  |  | 3118 | 0.206 | 0.6610 | No |
| 34 | IL15RA |  |  | 3488 | 0.194 | 0.6559 | No |
| 35 | FAS |  |  | 3778 | 0.186 | 0.6542 | No |
| 36 | SDCBP |  |  | 3795 | 0.185 | 0.6657 | No |
| 37 | BAG1 |  |  | 4977 | 0.156 | 0.6186 | No |
| 38 | PML |  |  | 5234 | 0.150 | 0.6161 | No |
| 39 | SHFM1 |  |  | 5405 | 0.147 | 0.6176 | No |
| 40 | EPS15 |  |  | 5695 | 0.140 | 0.6128 | No |
| 41 | IL6 |  |  | 5899 | 0.137 | 0.6120 | No |
| 42 | VAT1 |  |  | 6606 | 0.125 | 0.5860 | No |
| 43 | RHOC |  |  | 7051 | 0.117 | 0.5721 | No |
| 44 | XRCC6 |  |  | 7760 | 0.106 | 0.5447 | No |
| 45 | MAP3K10 |  |  | 8577 | 0.093 | 0.5111 | No |
| 46 | HIF3A |  |  | 8963 | 0.088 | 0.4982 | No |
| 47 | TAS2R5 |  |  | 9222 | 0.084 | 0.4912 | No |
| 48 | PHLDA1 |  |  | 9954 | 0.074 | 0.4605 | No |
| 49 | PPP3CA |  |  | 11496 | 0.053 | 0.3890 | No |
| 50 | ATP6V0B |  |  | 12039 | 0.046 | 0.3657 | No |
| 51 | PYHIN1 |  |  | 13629 | 0.026 | 0.2901 | No |
| 52 | TEAD4 |  |  | 13666 | 0.025 | 0.2900 | No |
| 53 | EIF2B1 |  |  | 13897 | 0.022 | 0.2803 | No |
| 54 | PRAME |  |  | 15340 | 0.000 | 0.2101 | No |
| 55 | NMI |  |  | 16092 | -0.012 | 0.1744 | No |
| 56 | TRIM21 |  |  | 16730 | -0.024 | 0.1449 | No |
| 57 | CSRP3 |  |  | 16774 | -0.024 | 0.1444 | No |
| 58 | COL16A1 |  |  | 17177 | -0.033 | 0.1270 | No |
| 59 | FOSL1 |  |  | 19426 | -0.104 | 0.0246 | No |
| 60 | VEGFC |  |  | 19585 | -0.113 | 0.0244 | No |
| 61 | CEBPD |  |  | 19851 | -0.131 | 0.0202 | No |
| 62 | PMAIP1 |  |  | 20440 | -0.248 | 0.0080 | No |
Table: GSEA details [plain text format]

  

Fig 2: DER\_IFNG\_UP: Random ES distribution      
 Gene set null distribution of ES for **DER\_IFNG\_UP**

  
