# Supplementary material for: The rapamycin-regulated gene expression signature determines prognosis for breast cancer
Source: Mol Cancer. 2009 Sep 24;8:75. doi: 10.1186/1476-4598-8-75 (PMC2761377; doi:10.1186/1476-4598-8-75)
Supplement: Additional file 3 — Gene set enrichment analysis of in vivo data, treatment series. The data provided represent the treatment series of GSEA. This compressed file contains "Treatment" shortcut file and "GSEA_treatment" folder. Clicking on "Treatment" shortcut opens the index file providing access to analysis files contained in the "GSEA_treatment" folder. [file 1476-4598-8-75-S3.zip › GSEA_treatment/DSRNA_UP.html]

Details for gene set DSRNA\_UP[GSEA]

|  || Dataset | gsea\_treatment\_collapsed |
| Phenotype | NoPhenotypeAvailable |
| Upregulated in class | na\_pos |
| GeneSet | DSRNA\_UP |
| Enrichment Score (ES) | 0.6572171 |
| Normalized Enrichment Score (NES) | 1.8345282 |
| Nominal p-value | 0.0 |
| FDR q-value | 0.0016167942 |
| FWER p-Value | 0.031 |
Table: GSEA Results Summary

  

Fig 1: Enrichment plot: DSRNA\_UP      
 Profile of the Running ES Score & Positions of GeneSet Members on the Rank Ordered List

  

| PROBE | GENE SYMBOL | GENE\_TITLE | RANK IN GENE LIST | RANK METRIC SCORE | RUNNING ES | CORE ENRICHMENT || 1 | TFPI2 |  |  | 0 | 1.316 | 0.1484 | Yes |
| 2 | TNFAIP2 |  |  | 22 | 0.790 | 0.2364 | Yes |
| 3 | GBP1 |  |  | 130 | 0.542 | 0.2924 | Yes |
| 4 | SAT1 |  |  | 137 | 0.536 | 0.3526 | Yes |
| 5 | BTN3A3 |  |  | 221 | 0.483 | 0.4030 | Yes |
| 6 | TNFAIP3 |  |  | 406 | 0.424 | 0.4419 | Yes |
| 7 | IRF1 |  |  | 551 | 0.393 | 0.4792 | Yes |
| 8 | TAP1 |  |  | 556 | 0.393 | 0.5233 | Yes |
| 9 | HMGB2 |  |  | 601 | 0.385 | 0.5646 | Yes |
| 10 | FGF2 |  |  | 834 | 0.348 | 0.5925 | Yes |
| 11 | ADAR |  |  | 885 | 0.344 | 0.6289 | Yes |
| 12 | EIF2S3 |  |  | 1453 | 0.290 | 0.6339 | Yes |
| 13 | CFLAR |  |  | 2118 | 0.250 | 0.6298 | Yes |
| 14 | IFIT1 |  |  | 2486 | 0.231 | 0.6380 | Yes |
| 15 | SUOX |  |  | 2614 | 0.226 | 0.6572 | Yes |
| 16 | BNIP3 |  |  | 3398 | 0.197 | 0.6414 | No |
| 17 | AFAP |  |  | 3621 | 0.190 | 0.6521 | No |
| 18 | XPO1 |  |  | 5531 | 0.144 | 0.5754 | No |
| 19 | ANXA1 |  |  | 5708 | 0.140 | 0.5827 | No |
| 20 | PSMA3 |  |  | 6760 | 0.122 | 0.5454 | No |
| 21 | NFKBIA |  |  | 7714 | 0.106 | 0.5110 | No |
| 22 | PLAUR |  |  | 9123 | 0.085 | 0.4522 | No |
| 23 | KLF4 |  |  | 9867 | 0.075 | 0.4245 | No |
| 24 | TRAF1 |  |  | 10005 | 0.073 | 0.4261 | No |
| 25 | PRG1 |  |  | 10469 | 0.066 | 0.4111 | No |
| 26 | ATF3 |  |  | 11262 | 0.056 | 0.3789 | No |
| 27 | ELF3 |  |  | 11345 | 0.055 | 0.3812 | No |
| 28 | TNFAIP6 |  |  | 12970 | 0.034 | 0.3060 | No |
| 29 | CD44 |  |  | 14567 | 0.013 | 0.2299 | No |
| 30 | BIRC2 |  |  | 15484 | -0.001 | 0.1855 | No |
| 31 | CHN1 |  |  | 15566 | -0.003 | 0.1819 | No |
| 32 | NMI |  |  | 16092 | -0.012 | 0.1577 | No |
| 33 | CCL4 |  |  | 16197 | -0.013 | 0.1542 | No |
| 34 | IQGAP2 |  |  | 17125 | -0.031 | 0.1126 | No |
| 35 | UBQLN1 |  |  | 18041 | -0.053 | 0.0741 | No |
| 36 | ACSL3 |  |  | 19146 | -0.091 | 0.0307 | No |
| 37 | GCH1 |  |  | 20092 | -0.155 | 0.0022 | No |
| 38 | IGFBP6 |  |  | 20329 | -0.201 | 0.0134 | No |
Table: GSEA details [plain text format]

  

Fig 2: DSRNA\_UP: Random ES distribution      
 Gene set null distribution of ES for **DSRNA\_UP**

  
