# Supplementary material for: The rapamycin-regulated gene expression signature determines prognosis for breast cancer
Source: Mol Cancer. 2009 Sep 24;8:75. doi: 10.1186/1476-4598-8-75 (PMC2761377; doi:10.1186/1476-4598-8-75)
Supplement: Additional file 3 — Gene set enrichment analysis of in vivo data, treatment series. The data provided represent the treatment series of GSEA. This compressed file contains "Treatment" shortcut file and "GSEA_treatment" folder. Clicking on "Treatment" shortcut opens the index file providing access to analysis files contained in the "GSEA_treatment" folder. [file 1476-4598-8-75-S3.zip › GSEA_treatment/ERM_KO_SERTOLI_DN.html]

Details for gene set ERM\_KO\_SERTOLI\_DN[GSEA]

|  || Dataset | gsea\_treatment\_collapsed |
| Phenotype | NoPhenotypeAvailable |
| Upregulated in class | na\_neg |
| GeneSet | ERM\_KO\_SERTOLI\_DN |
| Enrichment Score (ES) | -0.42156124 |
| Normalized Enrichment Score (NES) | -1.583549 |
| Nominal p-value | 0.026666667 |
| FDR q-value | 0.14089175 |
| FWER p-Value | 0.715 |
Table: GSEA Results Summary

  

Fig 1: Enrichment plot: ERM\_KO\_SERTOLI\_DN      
 Profile of the Running ES Score & Positions of GeneSet Members on the Rank Ordered List

  

| PROBE | GENE SYMBOL | GENE\_TITLE | RANK IN GENE LIST | RANK METRIC SCORE | RUNNING ES | CORE ENRICHMENT || 1 | IL6 |  |  | 5899 | 0.137 | -0.2103 | No |
| 2 | GBF1 |  |  | 7547 | 0.109 | -0.2296 | No |
| 3 | KIAA1199 |  |  | 9059 | 0.086 | -0.2549 | No |
| 4 | CLEC7A |  |  | 9166 | 0.085 | -0.2129 | No |
| 5 | ELAVL2 |  |  | 10332 | 0.068 | -0.2313 | No |
| 6 | CXCL12 |  |  | 10698 | 0.063 | -0.2136 | No |
| 7 | THBD |  |  | 10744 | 0.063 | -0.1806 | No |
| 8 | SEC24D |  |  | 11169 | 0.057 | -0.1693 | No |
| 9 | IGFBP4 |  |  | 11759 | 0.050 | -0.1700 | No |
| 10 | ANGPTL2 |  |  | 12288 | 0.043 | -0.1714 | No |
| 11 | CD36 |  |  | 12917 | 0.035 | -0.1824 | No |
| 12 | CXCL5 |  |  | 14674 | 0.011 | -0.2617 | No |
| 13 | CD53 |  |  | 15773 | -0.006 | -0.3116 | No |
| 14 | PLEK |  |  | 17540 | -0.041 | -0.3745 | No |
| 15 | GCG |  |  | 18386 | -0.062 | -0.3810 | Yes |
| 16 | CCL7 |  |  | 19223 | -0.094 | -0.3689 | Yes |
| 17 | MMP12 |  |  | 19482 | -0.107 | -0.3216 | Yes |
| 18 | GPNMB |  |  | 20588 | -0.674 | 0.0008 | Yes |
Table: GSEA details [plain text format]

  

Fig 2: ERM\_KO\_SERTOLI\_DN: Random ES distribution      
 Gene set null distribution of ES for **ERM\_KO\_SERTOLI\_DN**

  
