# Supplementary material for: The rapamycin-regulated gene expression signature determines prognosis for breast cancer
Source: Mol Cancer. 2009 Sep 24;8:75. doi: 10.1186/1476-4598-8-75 (PMC2761377; doi:10.1186/1476-4598-8-75)
Supplement: Additional file 3 — Gene set enrichment analysis of in vivo data, treatment series. The data provided represent the treatment series of GSEA. This compressed file contains "Treatment" shortcut file and "GSEA_treatment" folder. Clicking on "Treatment" shortcut opens the index file providing access to analysis files contained in the "GSEA_treatment" folder. [file 1476-4598-8-75-S3.zip › GSEA_treatment/FERRANDO_TAL1_NEIGHBORS.html]

Details for gene set FERRANDO\_TAL1\_NEIGHBORS[GSEA]

|  || Dataset | gsea\_treatment\_collapsed |
| Phenotype | NoPhenotypeAvailable |
| Upregulated in class | na\_neg |
| GeneSet | FERRANDO\_TAL1\_NEIGHBORS |
| Enrichment Score (ES) | -0.3609281 |
| Normalized Enrichment Score (NES) | -1.2410561 |
| Nominal p-value | 0.20353982 |
| FDR q-value | 0.27588946 |
| FWER p-Value | 1.0 |
Table: GSEA Results Summary

  

Fig 1: Enrichment plot: FERRANDO\_TAL1\_NEIGHBORS      
 Profile of the Running ES Score & Positions of GeneSet Members on the Rank Ordered List

  

| PROBE | GENE SYMBOL | GENE\_TITLE | RANK IN GENE LIST | RANK METRIC SCORE | RUNNING ES | CORE ENRICHMENT || 1 | FOLR1 |  |  | 520 | 0.401 | 0.1477 | No |
| 2 | ITGB2 |  |  | 2545 | 0.228 | 0.1479 | No |
| 3 | TMSL8 |  |  | 3338 | 0.199 | 0.1952 | No |
| 4 | MYBL2 |  |  | 4721 | 0.161 | 0.1977 | No |
| 5 | TNFRSF1B |  |  | 5948 | 0.136 | 0.1969 | No |
| 6 | RUNX1 |  |  | 9041 | 0.086 | 0.0839 | No |
| 7 | BCL2A1 |  |  | 10950 | 0.060 | 0.0172 | No |
| 8 | FCGR3B |  |  | 11670 | 0.051 | 0.0043 | No |
| 9 | DTYMK |  |  | 12103 | 0.045 | 0.0028 | No |
| 10 | CD6 |  |  | 14236 | 0.017 | -0.0932 | No |
| 11 | TCF7 |  |  | 16237 | -0.014 | -0.1843 | No |
| 12 | CSF1R |  |  | 18317 | -0.060 | -0.2593 | No |
| 13 | CRIP1 |  |  | 20410 | -0.230 | -0.2614 | Yes |
| 14 | LGALS3 /// GALIG |  |  | 20450 | -0.253 | -0.1541 | Yes |
| 15 | CDA |  |  | 20538 | -0.374 | 0.0033 | Yes |
Table: GSEA details [plain text format]

  

Fig 2: FERRANDO\_TAL1\_NEIGHBORS: Random ES distribution      
 Gene set null distribution of ES for **FERRANDO\_TAL1\_NEIGHBORS**

  
