# Supplementary material for: The rapamycin-regulated gene expression signature determines prognosis for breast cancer
Source: Mol Cancer. 2009 Sep 24;8:75. doi: 10.1186/1476-4598-8-75 (PMC2761377; doi:10.1186/1476-4598-8-75)
Supplement: Additional file 3 — Gene set enrichment analysis of in vivo data, treatment series. The data provided represent the treatment series of GSEA. This compressed file contains "Treatment" shortcut file and "GSEA_treatment" folder. Clicking on "Treatment" shortcut opens the index file providing access to analysis files contained in the "GSEA_treatment" folder. [file 1476-4598-8-75-S3.zip › GSEA_treatment/FRASOR_ER_UP.html]

Details for gene set FRASOR\_ER\_UP[GSEA]

|  || Dataset | gsea\_treatment\_collapsed |
| Phenotype | NoPhenotypeAvailable |
| Upregulated in class | na\_neg |
| GeneSet | FRASOR\_ER\_UP |
| Enrichment Score (ES) | -0.1834157 |
| Normalized Enrichment Score (NES) | -0.75504863 |
| Nominal p-value | 0.84782606 |
| FDR q-value | 0.92058307 |
| FWER p-Value | 1.0 |
Table: GSEA Results Summary

  

Fig 1: Enrichment plot: FRASOR\_ER\_UP      
 Profile of the Running ES Score & Positions of GeneSet Members on the Rank Ordered List

  

| PROBE | GENE SYMBOL | GENE\_TITLE | RANK IN GENE LIST | RANK METRIC SCORE | RUNNING ES | CORE ENRICHMENT || 1 | PTGES |  |  | 711 | 0.366 | 0.0821 | Yes |
| 2 | SLC39A6 |  |  | 2873 | 0.215 | 0.0456 | Yes |
| 3 | EGR3 |  |  | 3772 | 0.186 | 0.0612 | Yes |
| 4 | GLRB |  |  | 3943 | 0.181 | 0.1105 | Yes |
| 5 | TOP2A |  |  | 4403 | 0.169 | 0.1419 | Yes |
| 6 | EPB41L3 |  |  | 6142 | 0.133 | 0.0997 | Yes |
| 7 | SDC2 |  |  | 6496 | 0.126 | 0.1228 | Yes |
| 8 | CALCR |  |  | 6943 | 0.119 | 0.1390 | Yes |
| 9 | SLC22A5 |  |  | 7777 | 0.105 | 0.1321 | Yes |
| 10 | NRIP1 |  |  | 8204 | 0.099 | 0.1427 | Yes |
| 11 | GREB1 |  |  | 8900 | 0.088 | 0.1370 | Yes |
| 12 | RAB31 |  |  | 9052 | 0.086 | 0.1570 | Yes |
| 13 | PDZK1 |  |  | 10344 | 0.068 | 0.1160 | No |
| 14 | ADCY9 |  |  | 10691 | 0.064 | 0.1196 | No |
| 15 | CXCL12 |  |  | 10698 | 0.063 | 0.1395 | No |
| 16 | AP1G1 |  |  | 11018 | 0.059 | 0.1428 | No |
| 17 | AREG |  |  | 11292 | 0.056 | 0.1473 | No |
| 18 | IGFBP4 |  |  | 11759 | 0.050 | 0.1405 | No |
| 19 | MYBL1 |  |  | 12369 | 0.042 | 0.1244 | No |
| 20 | MAPT |  |  | 12665 | 0.038 | 0.1223 | No |
| 21 | RASGRP1 |  |  | 12821 | 0.036 | 0.1263 | No |
| 22 | RET |  |  | 13831 | 0.023 | 0.0846 | No |
| 23 | WISP2 |  |  | 14771 | 0.010 | 0.0420 | No |
| 24 | CA12 |  |  | 15493 | -0.002 | 0.0076 | No |
| 25 | CDC6 |  |  | 15583 | -0.003 | 0.0043 | No |
| 26 | RFC4 |  |  | 17365 | -0.037 | -0.0706 | No |
| 27 | SIAH2 |  |  | 19687 | -0.119 | -0.1456 | No |
| 28 | TFF1 |  |  | 20162 | -0.166 | -0.1158 | No |
| 29 | FOS |  |  | 20334 | -0.202 | -0.0597 | No |
| 30 | PEG10 |  |  | 20407 | -0.229 | 0.0096 | No |
Table: GSEA details [plain text format]

  

Fig 2: FRASOR\_ER\_UP: Random ES distribution      
 Gene set null distribution of ES for **FRASOR\_ER\_UP**

  
