# Supplementary material for: The rapamycin-regulated gene expression signature determines prognosis for breast cancer
Source: Mol Cancer. 2009 Sep 24;8:75. doi: 10.1186/1476-4598-8-75 (PMC2761377; doi:10.1186/1476-4598-8-75)
Supplement: Additional file 3 — Gene set enrichment analysis of in vivo data, treatment series. The data provided represent the treatment series of GSEA. This compressed file contains "Treatment" shortcut file and "GSEA_treatment" folder. Clicking on "Treatment" shortcut opens the index file providing access to analysis files contained in the "GSEA_treatment" folder. [file 1476-4598-8-75-S3.zip › GSEA_treatment/GALE_FLT3ANDAPL_DN.html]

Details for gene set GALE\_FLT3ANDAPL\_DN[GSEA]

|  || Dataset | gsea\_treatment\_collapsed |
| Phenotype | NoPhenotypeAvailable |
| Upregulated in class | na\_pos |
| GeneSet | GALE\_FLT3ANDAPL\_DN |
| Enrichment Score (ES) | 0.6968285 |
| Normalized Enrichment Score (NES) | 1.7271228 |
| Nominal p-value | 0.0 |
| FDR q-value | 0.009720272 |
| FWER p-Value | 0.285 |
Table: GSEA Results Summary

  

Fig 1: Enrichment plot: GALE\_FLT3ANDAPL\_DN      
 Profile of the Running ES Score & Positions of GeneSet Members on the Rank Ordered List

  

| PROBE | GENE SYMBOL | GENE\_TITLE | RANK IN GENE LIST | RANK METRIC SCORE | RUNNING ES | CORE ENRICHMENT || 1 | HLA-B |  |  | 41 | 0.695 | 0.1296 | Yes |
| 2 | HLA-C |  |  | 57 | 0.650 | 0.2519 | Yes |
| 3 | HLA-A /// HLA-H /// |  |  | 88 | 0.595 | 0.3631 | Yes |
| 4 | HLA-G |  |  | 107 | 0.567 | 0.4695 | Yes |
| 5 | NDUFB7 |  |  | 656 | 0.374 | 0.5137 | Yes |
| 6 | GPR137 |  |  | 668 | 0.371 | 0.5834 | Yes |
| 7 | BAD |  |  | 1497 | 0.287 | 0.5974 | Yes |
| 8 | PQBP1 |  |  | 1649 | 0.275 | 0.6421 | Yes |
| 9 | NDUFV1 |  |  | 2035 | 0.253 | 0.6714 | Yes |
| 10 | RABAC1 |  |  | 2526 | 0.229 | 0.6909 | Yes |
| 11 | DRAP1 |  |  | 3196 | 0.203 | 0.6968 | Yes |
| 12 | P4HB |  |  | 4307 | 0.172 | 0.6754 | No |
| 13 | ARF1 |  |  | 5294 | 0.149 | 0.6557 | No |
| 14 | DEGS1 |  |  | 6705 | 0.124 | 0.6106 | No |
| 15 | ST3GAL5 |  |  | 11900 | 0.048 | 0.3674 | No |
| 16 | OASL |  |  | 12433 | 0.041 | 0.3494 | No |
| 17 | NAPG |  |  | 14073 | 0.019 | 0.2735 | No |
| 18 | LOC641807 |  |  | 20415 | -0.231 | 0.0092 | No |
Table: GSEA details [plain text format]

  

Fig 2: GALE\_FLT3ANDAPL\_DN: Random ES distribution      
 Gene set null distribution of ES for **GALE\_FLT3ANDAPL\_DN**

  
