# Supplementary material for: The rapamycin-regulated gene expression signature determines prognosis for breast cancer
Source: Mol Cancer. 2009 Sep 24;8:75. doi: 10.1186/1476-4598-8-75 (PMC2761377; doi:10.1186/1476-4598-8-75)
Supplement: Additional file 3 — Gene set enrichment analysis of in vivo data, treatment series. The data provided represent the treatment series of GSEA. This compressed file contains "Treatment" shortcut file and "GSEA_treatment" folder. Clicking on "Treatment" shortcut opens the index file providing access to analysis files contained in the "GSEA_treatment" folder. [file 1476-4598-8-75-S3.zip › GSEA_treatment/GERY_CEBP_TARGETS.html]

Details for gene set GERY\_CEBP\_TARGETS[GSEA]

|  || Dataset | gsea\_treatment\_collapsed |
| Phenotype | NoPhenotypeAvailable |
| Upregulated in class | na\_neg |
| GeneSet | GERY\_CEBP\_TARGETS |
| Enrichment Score (ES) | -0.32127395 |
| Normalized Enrichment Score (NES) | -1.956547 |
| Nominal p-value | 0.0 |
| FDR q-value | 0.019847728 |
| FWER p-Value | 0.106 |
Table: GSEA Results Summary

  

Fig 1: Enrichment plot: GERY\_CEBP\_TARGETS      
 Profile of the Running ES Score & Positions of GeneSet Members on the Rank Ordered List

  

| PROBE | GENE SYMBOL | GENE\_TITLE | RANK IN GENE LIST | RANK METRIC SCORE | RUNNING ES | CORE ENRICHMENT || 1 | S100A8 |  |  | 42 | 0.695 | 0.0382 | No |
| 2 | PLAT |  |  | 132 | 0.541 | 0.0652 | No |
| 3 | SAT1 |  |  | 137 | 0.536 | 0.0961 | No |
| 4 | LCN2 |  |  | 262 | 0.463 | 0.1168 | No |
| 5 | XDH |  |  | 299 | 0.453 | 0.1413 | No |
| 6 | CD47 |  |  | 481 | 0.408 | 0.1561 | No |
| 7 | ACTA2 |  |  | 1058 | 0.325 | 0.1468 | No |
| 8 | PPARG |  |  | 1732 | 0.271 | 0.1297 | No |
| 9 | NRP1 |  |  | 1838 | 0.264 | 0.1399 | No |
| 10 | NR2F6 |  |  | 2138 | 0.249 | 0.1397 | No |
| 11 | ABHD5 |  |  | 2337 | 0.239 | 0.1438 | No |
| 12 | DNAJB9 |  |  | 2364 | 0.237 | 0.1563 | No |
| 13 | TOM1L1 |  |  | 2556 | 0.227 | 0.1601 | No |
| 14 | IER2 |  |  | 2613 | 0.226 | 0.1705 | No |
| 15 | FOXF2 |  |  | 2781 | 0.219 | 0.1751 | No |
| 16 | RRAD |  |  | 2789 | 0.219 | 0.1874 | No |
| 17 | WWC1 |  |  | 2931 | 0.213 | 0.1928 | No |
| 18 | PIM1 |  |  | 3137 | 0.205 | 0.1947 | No |
| 19 | JUNB |  |  | 3199 | 0.203 | 0.2035 | No |
| 20 | S100A3 |  |  | 3264 | 0.201 | 0.2120 | No |
| 21 | DBP |  |  | 3551 | 0.192 | 0.2092 | No |
| 22 | TCTE3 |  |  | 3992 | 0.179 | 0.1981 | No |
| 23 | PEA15 |  |  | 4528 | 0.166 | 0.1817 | No |
| 24 | PER2 |  |  | 4648 | 0.163 | 0.1853 | No |
| 25 | ABCA1 |  |  | 4833 | 0.159 | 0.1855 | No |
| 26 | GIPC2 |  |  | 4885 | 0.158 | 0.1921 | No |
| 27 | ECHS1 |  |  | 5287 | 0.149 | 0.1812 | No |
| 28 | BCKDHB |  |  | 5454 | 0.146 | 0.1816 | No |
| 29 | HIST1H2BC |  |  | 6053 | 0.134 | 0.1602 | No |
| 30 | ANGPTL4 |  |  | 6598 | 0.125 | 0.1408 | No |
| 31 | SERPINB2 |  |  | 6688 | 0.124 | 0.1437 | No |
| 32 | USP36 |  |  | 7006 | 0.118 | 0.1350 | No |
| 33 | CYB5R1 |  |  | 7071 | 0.116 | 0.1386 | No |
| 34 | C1QDC1 |  |  | 7103 | 0.116 | 0.1438 | No |
| 35 | ADSSL1 |  |  | 7655 | 0.107 | 0.1232 | No |
| 36 | CRABP2 |  |  | 7782 | 0.105 | 0.1231 | No |
| 37 | MKNK2 |  |  | 8697 | 0.091 | 0.0838 | No |
| 38 | PROCR |  |  | 8873 | 0.088 | 0.0804 | No |
| 39 | PLAUR |  |  | 9123 | 0.085 | 0.0732 | No |
| 40 | BAIAP2 |  |  | 9276 | 0.083 | 0.0706 | No |
| 41 | HBEGF |  |  | 9327 | 0.082 | 0.0729 | No |
| 42 | KLF4 |  |  | 9867 | 0.075 | 0.0509 | No |
| 43 | MAFK |  |  | 10240 | 0.070 | 0.0368 | No |
| 44 | CLU |  |  | 10425 | 0.067 | 0.0317 | No |
| 45 | RNH1 |  |  | 10541 | 0.066 | 0.0299 | No |
| 46 | IGF2 |  |  | 10823 | 0.062 | 0.0198 | No |
| 47 | EIF1AY |  |  | 11242 | 0.057 | 0.0027 | No |
| 48 | HGF |  |  | 11247 | 0.057 | 0.0058 | No |
| 49 | GADD45B |  |  | 11255 | 0.056 | 0.0087 | No |
| 50 | ATF3 |  |  | 11262 | 0.056 | 0.0117 | No |
| 51 | GREM2 |  |  | 11643 | 0.051 | -0.0039 | No |
| 52 | HP |  |  | 12079 | 0.046 | -0.0224 | No |
| 53 | FHL2 |  |  | 12328 | 0.042 | -0.0321 | No |
| 54 | HIPK1 |  |  | 12349 | 0.042 | -0.0306 | No |
| 55 | NGFB |  |  | 12612 | 0.039 | -0.0412 | No |
| 56 | EREG |  |  | 12711 | 0.038 | -0.0438 | No |
| 57 | ALDH3A1 |  |  | 12761 | 0.037 | -0.0440 | No |
| 58 | CXCL1 |  |  | 12797 | 0.037 | -0.0436 | No |
| 59 | HIST1H3F |  |  | 12809 | 0.036 | -0.0420 | No |
| 60 | HTATIP2 |  |  | 12883 | 0.035 | -0.0436 | No |
| 61 | FGF7 |  |  | 12924 | 0.035 | -0.0435 | No |
| 62 | RBED1 |  |  | 12952 | 0.035 | -0.0428 | No |
| 63 | TNFAIP6 |  |  | 12970 | 0.034 | -0.0417 | No |
| 64 | APBB1IP |  |  | 13353 | 0.029 | -0.0586 | No |
| 65 | SH3GL3 |  |  | 13575 | 0.026 | -0.0678 | No |
| 66 | SAA3P |  |  | 13776 | 0.024 | -0.0762 | No |
| 67 | BAG3 |  |  | 13939 | 0.022 | -0.0829 | No |
| 68 | POSTN |  |  | 14063 | 0.020 | -0.0878 | No |
| 69 | GLA |  |  | 14270 | 0.017 | -0.0968 | No |
| 70 | RGS16 |  |  | 14583 | 0.012 | -0.1114 | No |
| 71 | CXCL5 |  |  | 14674 | 0.011 | -0.1151 | No |
| 72 | TGM3 |  |  | 14730 | 0.010 | -0.1172 | No |
| 73 | PTX3 |  |  | 14933 | 0.007 | -0.1267 | No |
| 74 | GLRX |  |  | 15153 | 0.003 | -0.1372 | No |
| 75 | RASL11B |  |  | 15523 | -0.002 | -0.1550 | No |
| 76 | HMOX1 |  |  | 15526 | -0.002 | -0.1550 | No |
| 77 | ID2 |  |  | 15550 | -0.003 | -0.1559 | No |
| 78 | IFRD1 |  |  | 15829 | -0.007 | -0.1691 | No |
| 79 | RDH11 |  |  | 15894 | -0.008 | -0.1717 | No |
| 80 | BTG2 |  |  | 16013 | -0.011 | -0.1769 | No |
| 81 | KNG1 |  |  | 16629 | -0.022 | -0.2056 | No |
| 82 | DCN |  |  | 16689 | -0.023 | -0.2072 | No |
| 83 | SERPINI1 |  |  | 17300 | -0.035 | -0.2349 | No |
| 84 | NXPH2 |  |  | 17492 | -0.040 | -0.2419 | No |
| 85 | CSPG2 |  |  | 17601 | -0.042 | -0.2448 | No |
| 86 | LSS |  |  | 17971 | -0.051 | -0.2598 | No |
| 87 | ABCD2 |  |  | 18291 | -0.059 | -0.2719 | No |
| 88 | PRNP |  |  | 18358 | -0.061 | -0.2716 | No |
| 89 | CD68 |  |  | 18754 | -0.075 | -0.2866 | No |
| 90 | DUSP1 |  |  | 18808 | -0.076 | -0.2847 | No |
| 91 | LPL |  |  | 18964 | -0.083 | -0.2875 | No |
| 92 | CHORDC1 |  |  | 19655 | -0.116 | -0.3144 | Yes |
| 93 | ORM1 |  |  | 19733 | -0.122 | -0.3111 | Yes |
| 94 | GADD45G |  |  | 19781 | -0.126 | -0.3061 | Yes |
| 95 | GCH1 |  |  | 20092 | -0.155 | -0.3123 | Yes |
| 96 | ELOVL6 |  |  | 20205 | -0.174 | -0.3077 | Yes |
| 97 | CRYAB |  |  | 20259 | -0.185 | -0.2995 | Yes |
| 98 | FOS |  |  | 20334 | -0.202 | -0.2914 | Yes |
| 99 | DDIT3 |  |  | 20445 | -0.252 | -0.2822 | Yes |
| 100 | NSDHL |  |  | 20464 | -0.263 | -0.2679 | Yes |
| 101 | ID3 |  |  | 20467 | -0.265 | -0.2526 | Yes |
| 102 | HSPA1A |  |  | 20495 | -0.294 | -0.2369 | Yes |
| 103 | ZFAND2A |  |  | 20519 | -0.325 | -0.2192 | Yes |
| 104 | HMGCR |  |  | 20532 | -0.358 | -0.1991 | Yes |
| 105 | MVD |  |  | 20536 | -0.372 | -0.1777 | Yes |
| 106 | TRIB3 |  |  | 20539 | -0.374 | -0.1561 | Yes |
| 107 | TMEM45A |  |  | 20542 | -0.392 | -0.1335 | Yes |
| 108 | DNAJB1 |  |  | 20548 | -0.409 | -0.1100 | Yes |
| 109 | HSPA1B |  |  | 20565 | -0.463 | -0.0840 | Yes |
| 110 | ACSL1 |  |  | 20590 | -0.711 | -0.0440 | Yes |
| 111 | RGS2 |  |  | 20592 | -0.771 | 0.0006 | Yes |
Table: GSEA details [plain text format]

  

Fig 2: GERY\_CEBP\_TARGETS: Random ES distribution      
 Gene set null distribution of ES for **GERY\_CEBP\_TARGETS**

  
