# Supplementary material for: The rapamycin-regulated gene expression signature determines prognosis for breast cancer
Source: Mol Cancer. 2009 Sep 24;8:75. doi: 10.1186/1476-4598-8-75 (PMC2761377; doi:10.1186/1476-4598-8-75)
Supplement: Additional file 3 — Gene set enrichment analysis of in vivo data, treatment series. The data provided represent the treatment series of GSEA. This compressed file contains "Treatment" shortcut file and "GSEA_treatment" folder. Clicking on "Treatment" shortcut opens the index file providing access to analysis files contained in the "GSEA_treatment" folder. [file 1476-4598-8-75-S3.zip › GSEA_treatment/GLUCONEOGENESIS.html]

Details for gene set GLUCONEOGENESIS[GSEA]

|  || Dataset | gsea\_treatment\_collapsed |
| Phenotype | NoPhenotypeAvailable |
| Upregulated in class | na\_neg |
| GeneSet | GLUCONEOGENESIS |
| Enrichment Score (ES) | -0.22703601 |
| Normalized Enrichment Score (NES) | -1.0901592 |
| Nominal p-value | 0.26086956 |
| FDR q-value | 0.45408908 |
| FWER p-Value | 1.0 |
Table: GSEA Results Summary

  

Fig 1: Enrichment plot: GLUCONEOGENESIS      
 Profile of the Running ES Score & Positions of GeneSet Members on the Rank Ordered List

  

| PROBE | GENE SYMBOL | GENE\_TITLE | RANK IN GENE LIST | RANK METRIC SCORE | RUNNING ES | CORE ENRICHMENT || 1 | ALDH1A3 |  |  | 201 | 0.491 | 0.0754 | Yes |
| 2 | FBP2 |  |  | 1423 | 0.292 | 0.0666 | Yes |
| 3 | ACYP2 |  |  | 1708 | 0.272 | 0.0999 | Yes |
| 4 | ALDH1A1 |  |  | 1897 | 0.261 | 0.1360 | Yes |
| 5 | BPGM |  |  | 2267 | 0.242 | 0.1599 | Yes |
| 6 | PDHB |  |  | 2557 | 0.227 | 0.1853 | Yes |
| 7 | ENO1 |  |  | 2660 | 0.225 | 0.2193 | Yes |
| 8 | ALDH1B1 |  |  | 5730 | 0.140 | 0.0942 | No |
| 9 | DLD |  |  | 5864 | 0.137 | 0.1115 | No |
| 10 | ALDH3B1 |  |  | 6056 | 0.134 | 0.1255 | No |
| 11 | ALDOB |  |  | 6203 | 0.132 | 0.1412 | No |
| 12 | PGK1 |  |  | 6774 | 0.122 | 0.1346 | No |
| 13 | ALDH3B2 |  |  | 7193 | 0.114 | 0.1341 | No |
| 14 | ADH7 |  |  | 7491 | 0.110 | 0.1387 | No |
| 15 | PKM2 |  |  | 8667 | 0.091 | 0.0973 | No |
| 16 | ENO3 |  |  | 9051 | 0.086 | 0.0936 | No |
| 17 | GAPDH |  |  | 9691 | 0.077 | 0.0759 | No |
| 18 | ALDOA |  |  | 9957 | 0.074 | 0.0757 | No |
| 19 | ADH1B |  |  | 10093 | 0.072 | 0.0816 | No |
| 20 | DLAT |  |  | 10308 | 0.069 | 0.0831 | No |
| 21 | LDHC |  |  | 11189 | 0.057 | 0.0502 | No |
| 22 | AKR1A1 |  |  | 11934 | 0.048 | 0.0223 | No |
| 23 | GCK |  |  | 12047 | 0.046 | 0.0248 | No |
| 24 | ADH4 |  |  | 12242 | 0.044 | 0.0230 | No |
| 25 | ALDH3A1 |  |  | 12761 | 0.037 | 0.0042 | No |
| 26 | TPI1 |  |  | 12932 | 0.035 | 0.0019 | No |
| 27 | PDHA1 |  |  | 13233 | 0.031 | -0.0073 | No |
| 28 | PKLR |  |  | 14035 | 0.020 | -0.0427 | No |
| 29 | ADH1A |  |  | 14143 | 0.019 | -0.0447 | No |
| 30 | PGM3 |  |  | 14215 | 0.018 | -0.0451 | No |
| 31 | ADH6 |  |  | 14218 | 0.018 | -0.0421 | No |
| 32 | HK2 |  |  | 14703 | 0.011 | -0.0638 | No |
| 33 | PDHA2 |  |  | 14914 | 0.007 | -0.0728 | No |
| 34 | ALDOC |  |  | 15589 | -0.003 | -0.1050 | No |
| 35 | PFKM |  |  | 15673 | -0.005 | -0.1082 | No |
| 36 | ACYP1 |  |  | 15884 | -0.008 | -0.1170 | No |
| 37 | G6PC |  |  | 16166 | -0.013 | -0.1284 | No |
| 38 | ALDH3A2 |  |  | 16233 | -0.014 | -0.1292 | No |
| 39 | ENO2 |  |  | 16283 | -0.015 | -0.1290 | No |
| 40 | FBP1 |  |  | 16603 | -0.021 | -0.1408 | No |
| 41 | PGM1 |  |  | 16604 | -0.021 | -0.1372 | No |
| 42 | ALDH9A1 |  |  | 16867 | -0.025 | -0.1455 | No |
| 43 | GPI |  |  | 17414 | -0.038 | -0.1655 | No |
| 44 | ADH1C |  |  | 17433 | -0.038 | -0.1598 | No |
| 45 | ALDH1A2 |  |  | 18290 | -0.059 | -0.1912 | No |
| 46 | HK1 |  |  | 18684 | -0.072 | -0.1978 | No |
| 47 | HK3 |  |  | 19286 | -0.097 | -0.2102 | No |
| 48 | LDHA |  |  | 19554 | -0.112 | -0.2039 | No |
| 49 | PFKP |  |  | 19647 | -0.116 | -0.1882 | No |
| 50 | ADHFE1 |  |  | 20063 | -0.150 | -0.1824 | No |
| 51 | ALDH2 |  |  | 20530 | -0.348 | -0.1448 | No |
| 52 | LDHB |  |  | 20594 | -0.856 | 0.0005 | No |
Table: GSEA details [plain text format]

  

Fig 2: GLUCONEOGENESIS: Random ES distribution      
 Gene set null distribution of ES for **GLUCONEOGENESIS**

  
