# Supplementary material for: The rapamycin-regulated gene expression signature determines prognosis for breast cancer
Source: Mol Cancer. 2009 Sep 24;8:75. doi: 10.1186/1476-4598-8-75 (PMC2761377; doi:10.1186/1476-4598-8-75)
Supplement: Additional file 3 — Gene set enrichment analysis of in vivo data, treatment series. The data provided represent the treatment series of GSEA. This compressed file contains "Treatment" shortcut file and "GSEA_treatment" folder. Clicking on "Treatment" shortcut opens the index file providing access to analysis files contained in the "GSEA_treatment" folder. [file 1476-4598-8-75-S3.zip › GSEA_treatment/GLYCINE_SERINE_AND_THREONINE_METABOLISM.html]

Details for gene set GLYCINE\_SERINE\_AND\_THREONINE\_METABOLISM[GSEA]

|  || Dataset | gsea\_treatment\_collapsed |
| Phenotype | NoPhenotypeAvailable |
| Upregulated in class | na\_neg |
| GeneSet | GLYCINE\_SERINE\_AND\_THREONINE\_METABOLISM |
| Enrichment Score (ES) | -0.23323333 |
| Normalized Enrichment Score (NES) | -0.9753374 |
| Nominal p-value | 0.54761904 |
| FDR q-value | 0.61393327 |
| FWER p-Value | 1.0 |
Table: GSEA Results Summary

  

Fig 1: Enrichment plot: GLYCINE\_SERINE\_AND\_THREONINE\_METABOLISM      
 Profile of the Running ES Score & Positions of GeneSet Members on the Rank Ordered List

  

| PROBE | GENE SYMBOL | GENE\_TITLE | RANK IN GENE LIST | RANK METRIC SCORE | RUNNING ES | CORE ENRICHMENT || 1 | MAOA |  |  | 305 | 0.451 | 0.0920 | No |
| 2 | ABP1 |  |  | 1149 | 0.316 | 0.1259 | No |
| 3 | PEMT |  |  | 1759 | 0.269 | 0.1600 | No |
| 4 | ALAS1 |  |  | 2924 | 0.214 | 0.1540 | No |
| 5 | SHMT1 |  |  | 3269 | 0.201 | 0.1849 | No |
| 6 | PISD |  |  | 4239 | 0.173 | 0.1789 | No |
| 7 | GAMT |  |  | 4867 | 0.158 | 0.1859 | No |
| 8 | DLD |  |  | 5864 | 0.137 | 0.1700 | No |
| 9 | CHKB /// CPT1B |  |  | 6809 | 0.122 | 0.1529 | No |
| 10 | PLCG1 |  |  | 7929 | 0.103 | 0.1229 | No |
| 11 | BHMT |  |  | 8134 | 0.100 | 0.1366 | No |
| 12 | CHDH |  |  | 8506 | 0.094 | 0.1408 | No |
| 13 | ALAS2 |  |  | 9267 | 0.083 | 0.1236 | No |
| 14 | GATM |  |  | 9929 | 0.074 | 0.1090 | No |
| 15 | SARDH |  |  | 10510 | 0.066 | 0.0964 | No |
| 16 | AMT |  |  | 11001 | 0.060 | 0.0867 | No |
| 17 | PLCG2 |  |  | 11276 | 0.056 | 0.0867 | No |
| 18 | GCAT |  |  | 11280 | 0.056 | 0.0999 | No |
| 19 | CHKA |  |  | 11499 | 0.053 | 0.1020 | No |
| 20 | PLCB2 |  |  | 13671 | 0.025 | 0.0024 | No |
| 21 | MAOB |  |  | 14617 | 0.012 | -0.0408 | No |
| 22 | AOC3 |  |  | 14735 | 0.010 | -0.0441 | No |
| 23 | PSPH |  |  | 14851 | 0.008 | -0.0478 | No |
| 24 | AGXT |  |  | 15007 | 0.006 | -0.0539 | No |
| 25 | AOC2 |  |  | 15717 | -0.005 | -0.0872 | No |
| 26 | CTH |  |  | 16261 | -0.014 | -0.1101 | No |
| 27 | DMGDH |  |  | 17199 | -0.033 | -0.1478 | No |
| 28 | CBS |  |  | 18957 | -0.083 | -0.2136 | Yes |
| 29 | AGXT2 |  |  | 18965 | -0.083 | -0.1944 | Yes |
| 30 | DAO |  |  | 18992 | -0.084 | -0.1757 | Yes |
| 31 | SARS |  |  | 19872 | -0.133 | -0.1868 | Yes |
| 32 | TARS |  |  | 19912 | -0.137 | -0.1564 | Yes |
| 33 | GLDC |  |  | 20110 | -0.158 | -0.1284 | Yes |
| 34 | SHMT2 |  |  | 20303 | -0.196 | -0.0913 | Yes |
| 35 | GARS |  |  | 20560 | -0.447 | 0.0022 | Yes |
Table: GSEA details [plain text format]

  

Fig 2: GLYCINE\_SERINE\_AND\_THREONINE\_METABOLISM: Random ES distribution      
 Gene set null distribution of ES for **GLYCINE\_SERINE\_AND\_THREONINE\_METABOLISM**

  
