# Supplementary material for: The rapamycin-regulated gene expression signature determines prognosis for breast cancer
Source: Mol Cancer. 2009 Sep 24;8:75. doi: 10.1186/1476-4598-8-75 (PMC2761377; doi:10.1186/1476-4598-8-75)
Supplement: Additional file 3 — Gene set enrichment analysis of in vivo data, treatment series. The data provided represent the treatment series of GSEA. This compressed file contains "Treatment" shortcut file and "GSEA_treatment" folder. Clicking on "Treatment" shortcut opens the index file providing access to analysis files contained in the "GSEA_treatment" folder. [file 1476-4598-8-75-S3.zip › GSEA_treatment/GLYCOLYSIS_AND_GLUCONEOGENESIS.html]

Details for gene set GLYCOLYSIS\_AND\_GLUCONEOGENESIS[GSEA]

|  || Dataset | gsea\_treatment\_collapsed |
| Phenotype | NoPhenotypeAvailable |
| Upregulated in class | na\_neg |
| GeneSet | GLYCOLYSIS\_AND\_GLUCONEOGENESIS |
| Enrichment Score (ES) | -0.2946566 |
| Normalized Enrichment Score (NES) | -1.2120883 |
| Nominal p-value | 0.125 |
| FDR q-value | 0.29186928 |
| FWER p-Value | 1.0 |
Table: GSEA Results Summary

  

Fig 1: Enrichment plot: GLYCOLYSIS\_AND\_GLUCONEOGENESIS      
 Profile of the Running ES Score & Positions of GeneSet Members on the Rank Ordered List

  

| PROBE | GENE SYMBOL | GENE\_TITLE | RANK IN GENE LIST | RANK METRIC SCORE | RUNNING ES | CORE ENRICHMENT || 1 | PFKL |  |  | 532 | 0.398 | 0.0552 | No |
| 2 | FBP2 |  |  | 1423 | 0.292 | 0.0713 | No |
| 3 | MDH1 |  |  | 2181 | 0.246 | 0.0846 | No |
| 4 | PC |  |  | 2386 | 0.236 | 0.1227 | No |
| 5 | PDHB |  |  | 2557 | 0.227 | 0.1607 | No |
| 6 | ENO1 |  |  | 2660 | 0.225 | 0.2015 | No |
| 7 | DLD |  |  | 5864 | 0.137 | 0.0736 | No |
| 8 | MDH2 |  |  | 5920 | 0.137 | 0.0987 | No |
| 9 | ALDOB |  |  | 6203 | 0.132 | 0.1118 | No |
| 10 | PGK1 |  |  | 6774 | 0.122 | 0.1090 | No |
| 11 | TNFAIP1 |  |  | 7845 | 0.104 | 0.0782 | No |
| 12 | PKM2 |  |  | 8667 | 0.091 | 0.0569 | No |
| 13 | ENO3 |  |  | 9051 | 0.086 | 0.0557 | No |
| 14 | GAPDH |  |  | 9691 | 0.077 | 0.0403 | No |
| 15 | GAPDHS |  |  | 9705 | 0.077 | 0.0554 | No |
| 16 | ALDOA |  |  | 9957 | 0.074 | 0.0581 | No |
| 17 | DLAT |  |  | 10308 | 0.069 | 0.0551 | No |
| 18 | LDHC |  |  | 11189 | 0.057 | 0.0240 | No |
| 19 | GCK |  |  | 12047 | 0.046 | -0.0083 | No |
| 20 | TPI1 |  |  | 12932 | 0.035 | -0.0442 | No |
| 21 | PDHA1 |  |  | 13233 | 0.031 | -0.0525 | No |
| 22 | PKLR |  |  | 14035 | 0.020 | -0.0873 | No |
| 23 | HK2 |  |  | 14703 | 0.011 | -0.1176 | No |
| 24 | PDHA2 |  |  | 14914 | 0.007 | -0.1263 | No |
| 25 | ALDOC |  |  | 15589 | -0.003 | -0.1584 | No |
| 26 | PDHX |  |  | 15594 | -0.003 | -0.1579 | No |
| 27 | PCK1 |  |  | 15655 | -0.004 | -0.1600 | No |
| 28 | PFKM |  |  | 15673 | -0.005 | -0.1598 | No |
| 29 | G6PC |  |  | 16166 | -0.013 | -0.1811 | No |
| 30 | ENO2 |  |  | 16283 | -0.015 | -0.1837 | No |
| 31 | FBP1 |  |  | 16603 | -0.021 | -0.1949 | No |
| 32 | GPI |  |  | 17414 | -0.038 | -0.2266 | No |
| 33 | GOT2 |  |  | 17497 | -0.040 | -0.2224 | No |
| 34 | HK1 |  |  | 18684 | -0.072 | -0.2654 | No |
| 35 | HK3 |  |  | 19286 | -0.097 | -0.2749 | Yes |
| 36 | LDHA |  |  | 19554 | -0.112 | -0.2652 | Yes |
| 37 | PFKP |  |  | 19647 | -0.116 | -0.2460 | Yes |
| 38 | PGAM2 |  |  | 19685 | -0.119 | -0.2237 | Yes |
| 39 | LDHAL6B |  |  | 19989 | -0.143 | -0.2092 | Yes |
| 40 | GOT1 |  |  | 20079 | -0.152 | -0.1825 | Yes |
| 41 | PGK2 |  |  | 20160 | -0.166 | -0.1526 | Yes |
| 42 | LDHB |  |  | 20594 | -0.856 | 0.0005 | Yes |
Table: GSEA details [plain text format]

  

Fig 2: GLYCOLYSIS\_AND\_GLUCONEOGENESIS: Random ES distribution      
 Gene set null distribution of ES for **GLYCOLYSIS\_AND\_GLUCONEOGENESIS**

  
