# Supplementary material for: The rapamycin-regulated gene expression signature determines prognosis for breast cancer
Source: Mol Cancer. 2009 Sep 24;8:75. doi: 10.1186/1476-4598-8-75 (PMC2761377; doi:10.1186/1476-4598-8-75)
Supplement: Additional file 3 — Gene set enrichment analysis of in vivo data, treatment series. The data provided represent the treatment series of GSEA. This compressed file contains "Treatment" shortcut file and "GSEA_treatment" folder. Clicking on "Treatment" shortcut opens the index file providing access to analysis files contained in the "GSEA_treatment" folder. [file 1476-4598-8-75-S3.zip › GSEA_treatment/GPCRS_CLASS_A_RHODOPSIN_LIKE.html]

Details for gene set GPCRS\_CLASS\_A\_RHODOPSIN\_LIKE[GSEA]

|  || Dataset | gsea\_treatment\_collapsed |
| Phenotype | NoPhenotypeAvailable |
| Upregulated in class | na\_neg |
| GeneSet | GPCRS\_CLASS\_A\_RHODOPSIN\_LIKE |
| Enrichment Score (ES) | -0.15002999 |
| Normalized Enrichment Score (NES) | -0.79509026 |
| Nominal p-value | 1.0 |
| FDR q-value | 0.8748029 |
| FWER p-Value | 1.0 |
Table: GSEA Results Summary

  

Fig 1: Enrichment plot: GPCRS\_CLASS\_A\_RHODOPSIN\_LIKE      
 Profile of the Running ES Score & Positions of GeneSet Members on the Rank Ordered List

  

| PROBE | GENE SYMBOL | GENE\_TITLE | RANK IN GENE LIST | RANK METRIC SCORE | RUNNING ES | CORE ENRICHMENT || 1 | F2RL1 |  |  | 172 | 0.509 | 0.0426 | No |
| 2 | BCL9L |  |  | 2590 | 0.227 | -0.0528 | No |
| 3 | DRD3 |  |  | 2728 | 0.222 | -0.0373 | No |
| 4 | EDNRA |  |  | 3771 | 0.186 | -0.0695 | No |
| 5 | OPN3 |  |  | 3807 | 0.185 | -0.0527 | No |
| 6 | TBXA2R |  |  | 4038 | 0.178 | -0.0461 | No |
| 7 | CHRM1 |  |  | 4369 | 0.170 | -0.0451 | No |
| 8 | PTAFR |  |  | 4384 | 0.170 | -0.0288 | No |
| 9 | AVPR1A |  |  | 4521 | 0.166 | -0.0188 | No |
| 10 | PTGER3 |  |  | 4580 | 0.165 | -0.0051 | No |
| 11 | GPR27 |  |  | 4594 | 0.164 | 0.0107 | No |
| 12 | DRD4 |  |  | 4786 | 0.160 | 0.0174 | No |
| 13 | ADRB2 |  |  | 5224 | 0.151 | 0.0111 | No |
| 14 | PTGER1 |  |  | 5895 | 0.137 | -0.0079 | No |
| 15 | FPRL1 |  |  | 6075 | 0.134 | -0.0032 | No |
| 16 | F2R |  |  | 6726 | 0.123 | -0.0226 | No |
| 17 | VIPR1 |  |  | 6954 | 0.119 | -0.0218 | No |
| 18 | RGR |  |  | 7033 | 0.117 | -0.0139 | No |
| 19 | OPRL1 |  |  | 7124 | 0.115 | -0.0067 | No |
| 20 | HRH3 |  |  | 7154 | 0.115 | 0.0034 | No |
| 21 | GPR44 |  |  | 7333 | 0.113 | 0.0060 | No |
| 22 | AVPR2 |  |  | 7357 | 0.112 | 0.0161 | No |
| 23 | PTGER4 |  |  | 7819 | 0.105 | 0.0041 | No |
| 24 | HTR7 |  |  | 7871 | 0.104 | 0.0120 | No |
| 25 | P2RY12 |  |  | 8011 | 0.101 | 0.0154 | No |
| 26 | CX3CR1 |  |  | 8229 | 0.098 | 0.0146 | No |
| 27 | DRD2 |  |  | 8236 | 0.098 | 0.0241 | No |
| 28 | PTGFR |  |  | 8323 | 0.097 | 0.0296 | No |
| 29 | HTR2C |  |  | 8520 | 0.094 | 0.0294 | No |
| 30 | PTGDR |  |  | 8533 | 0.094 | 0.0382 | No |
| 31 | NPY5R |  |  | 8644 | 0.092 | 0.0420 | No |
| 32 | ADRA1D |  |  | 8682 | 0.091 | 0.0494 | No |
| 33 | PPYR1 |  |  | 8797 | 0.090 | 0.0528 | No |
| 34 | GPR4 |  |  | 8830 | 0.089 | 0.0602 | No |
| 35 | ADRA1A |  |  | 8912 | 0.088 | 0.0650 | No |
| 36 | ADRA1B |  |  | 9150 | 0.085 | 0.0619 | No |
| 37 | NPY6R |  |  | 9630 | 0.078 | 0.0463 | No |
| 38 | GALR2 |  |  | 9675 | 0.077 | 0.0519 | No |
| 39 | MC1R |  |  | 9859 | 0.076 | 0.0506 | No |
| 40 | CNR1 |  |  | 10080 | 0.072 | 0.0470 | No |
| 41 | CCKAR |  |  | 10374 | 0.068 | 0.0395 | No |
| 42 | FBXW2 |  |  | 10454 | 0.067 | 0.0423 | No |
| 43 | NPY2R |  |  | 10916 | 0.061 | 0.0259 | No |
| 44 | CCKBR |  |  | 11006 | 0.060 | 0.0275 | No |
| 45 | GPR30 |  |  | 11049 | 0.059 | 0.0314 | No |
| 46 | CXCR3 |  |  | 11288 | 0.056 | 0.0254 | No |
| 47 | BLR1 |  |  | 11324 | 0.056 | 0.0292 | No |
| 48 | GPR35 |  |  | 11856 | 0.049 | 0.0082 | No |
| 49 | LTB4R |  |  | 11865 | 0.049 | 0.0126 | No |
| 50 | GALR3 |  |  | 11979 | 0.047 | 0.0118 | No |
| 51 | TSHR |  |  | 12010 | 0.047 | 0.0151 | No |
| 52 | GPR17 |  |  | 12033 | 0.046 | 0.0186 | No |
| 53 | CCR1 |  |  | 12230 | 0.044 | 0.0134 | No |
| 54 | GPR50 |  |  | 12571 | 0.039 | 0.0008 | No |
| 55 | EDNRB |  |  | 12640 | 0.038 | 0.0013 | No |
| 56 | ADRB1 |  |  | 12920 | 0.035 | -0.0088 | No |
| 57 | HTR1D |  |  | 13099 | 0.033 | -0.0142 | No |
| 58 | CHRM2 |  |  | 13203 | 0.031 | -0.0161 | No |
| 59 | HTR4 |  |  | 13224 | 0.031 | -0.0140 | No |
| 60 | GPR85 |  |  | 13442 | 0.028 | -0.0218 | No |
| 61 | CCR2 |  |  | 13529 | 0.027 | -0.0232 | No |
| 62 | ADRA2C |  |  | 13828 | 0.023 | -0.0355 | No |
| 63 | DRD1 |  |  | 13833 | 0.023 | -0.0333 | No |
| 64 | ADORA3 |  |  | 13837 | 0.023 | -0.0312 | No |
| 65 | HCRTR1 |  |  | 14064 | 0.020 | -0.0403 | No |
| 66 | GRPR |  |  | 14158 | 0.018 | -0.0430 | No |
| 67 | LHCGR |  |  | 14177 | 0.018 | -0.0420 | No |
| 68 | OPRM1 |  |  | 14363 | 0.015 | -0.0495 | No |
| 69 | HTR2A |  |  | 14366 | 0.015 | -0.0481 | No |
| 70 | BDKRB2 |  |  | 14390 | 0.015 | -0.0477 | No |
| 71 | AGTR1 |  |  | 14404 | 0.015 | -0.0469 | No |
| 72 | IL8RA |  |  | 14413 | 0.015 | -0.0458 | No |
| 73 | CMKLR1 |  |  | 14484 | 0.013 | -0.0478 | No |
| 74 | OPRD1 |  |  | 14573 | 0.012 | -0.0509 | No |
| 75 | P2RY1 |  |  | 14631 | 0.012 | -0.0526 | No |
| 76 | CXCR4 |  |  | 14667 | 0.011 | -0.0532 | No |
| 77 | HTR5A |  |  | 14750 | 0.010 | -0.0562 | No |
| 78 | CCR3 |  |  | 14770 | 0.010 | -0.0562 | No |
| 79 | GHSR |  |  | 14783 | 0.010 | -0.0558 | No |
| 80 | IL8RB |  |  | 14882 | 0.008 | -0.0598 | No |
| 81 | FPR1 |  |  | 14968 | 0.006 | -0.0633 | No |
| 82 | CCBP2 |  |  | 15148 | 0.004 | -0.0717 | No |
| 83 | FPRL2 |  |  | 15248 | 0.002 | -0.0763 | No |
| 84 | CHRM5 |  |  | 15344 | 0.000 | -0.0809 | No |
| 85 | AVPR1B |  |  | 15438 | -0.001 | -0.0854 | No |
| 86 | PTGIR |  |  | 15602 | -0.003 | -0.0930 | No |
| 87 | CCRL1 |  |  | 15835 | -0.008 | -0.1036 | No |
| 88 | GPR23 |  |  | 15968 | -0.010 | -0.1090 | No |
| 89 | CCR9 |  |  | 16023 | -0.011 | -0.1106 | No |
| 90 | GALR1 |  |  | 16090 | -0.012 | -0.1126 | No |
| 91 | MTNR1A |  |  | 16234 | -0.014 | -0.1182 | No |
| 92 | CCR7 |  |  | 16267 | -0.014 | -0.1183 | No |
| 93 | ADRB3 |  |  | 16468 | -0.018 | -0.1263 | No |
| 94 | ADORA2A |  |  | 16513 | -0.019 | -0.1265 | No |
| 95 | OR7C1 |  |  | 16633 | -0.022 | -0.1302 | No |
| 96 | CHRM4 |  |  | 16792 | -0.025 | -0.1354 | No |
| 97 | HTR1F |  |  | 16863 | -0.025 | -0.1363 | No |
| 98 | BDKRB1 |  |  | 16892 | -0.026 | -0.1351 | No |
| 99 | GPR63 |  |  | 16964 | -0.028 | -0.1357 | No |
| 100 | HRH1 |  |  | 17053 | -0.030 | -0.1370 | No |
| 101 | TRHR |  |  | 17077 | -0.030 | -0.1351 | No |
| 102 | NMBR |  |  | 17160 | -0.032 | -0.1359 | No |
| 103 | GPR92 |  |  | 17189 | -0.033 | -0.1340 | No |
| 104 | ADORA1 |  |  | 17334 | -0.036 | -0.1374 | No |
| 105 | MTNR1B |  |  | 17389 | -0.037 | -0.1363 | No |
| 106 | HTR1A |  |  | 17510 | -0.040 | -0.1382 | No |
| 107 | AGTR2 |  |  | 17573 | -0.041 | -0.1371 | No |
| 108 | NTSR1 |  |  | 17647 | -0.043 | -0.1363 | No |
| 109 | P2RY5 |  |  | 17693 | -0.044 | -0.1341 | No |
| 110 | P2RY6 |  |  | 17762 | -0.046 | -0.1328 | No |
| 111 | FSHR |  |  | 17800 | -0.047 | -0.1300 | No |
| 112 | HTR1E |  |  | 17910 | -0.050 | -0.1303 | No |
| 113 | F2RL2 |  |  | 17982 | -0.051 | -0.1287 | No |
| 114 | OXTR |  |  | 18220 | -0.057 | -0.1345 | No |
| 115 | HCRTR2 |  |  | 18368 | -0.062 | -0.1356 | No |
| 116 | ADORA2B |  |  | 18557 | -0.067 | -0.1380 | No |
| 117 | ADRA2A |  |  | 18776 | -0.076 | -0.1411 | No |
| 118 | HTR2B |  |  | 18960 | -0.083 | -0.1417 | Yes |
| 119 | HTR1B |  |  | 18991 | -0.084 | -0.1348 | Yes |
| 120 | C3AR1 |  |  | 19264 | -0.096 | -0.1384 | Yes |
| 121 | MAS1 |  |  | 19308 | -0.099 | -0.1307 | Yes |
| 122 | NPY1R |  |  | 19384 | -0.102 | -0.1241 | Yes |
| 123 | CNR2 |  |  | 19427 | -0.104 | -0.1157 | Yes |
| 124 | OPRK1 |  |  | 19478 | -0.107 | -0.1074 | Yes |
| 125 | GPR37 |  |  | 19676 | -0.118 | -0.1051 | Yes |
| 126 | NTSR2 |  |  | 19739 | -0.122 | -0.0959 | Yes |
| 127 | P2RY2 |  |  | 19883 | -0.134 | -0.0895 | Yes |
| 128 | PTGER2 |  |  | 19983 | -0.143 | -0.0801 | Yes |
| 129 | CCR4 |  |  | 20018 | -0.146 | -0.0671 | Yes |
| 130 | AGTRL1 |  |  | 20045 | -0.149 | -0.0535 | Yes |
| 131 | RRH |  |  | 20081 | -0.153 | -0.0399 | Yes |
| 132 | CCR8 |  |  | 20178 | -0.169 | -0.0277 | Yes |
| 133 | OR1F1 |  |  | 20304 | -0.197 | -0.0141 | Yes |
| 134 | GPR87 |  |  | 20490 | -0.287 | 0.0056 | Yes |
Table: GSEA details [plain text format]

  

Fig 2: GPCRS\_CLASS\_A\_RHODOPSIN\_LIKE: Random ES distribution      
 Gene set null distribution of ES for **GPCRS\_CLASS\_A\_RHODOPSIN\_LIKE**

  
