# Supplementary material for: The rapamycin-regulated gene expression signature determines prognosis for breast cancer
Source: Mol Cancer. 2009 Sep 24;8:75. doi: 10.1186/1476-4598-8-75 (PMC2761377; doi:10.1186/1476-4598-8-75)
Supplement: Additional file 3 — Gene set enrichment analysis of in vivo data, treatment series. The data provided represent the treatment series of GSEA. This compressed file contains "Treatment" shortcut file and "GSEA_treatment" folder. Clicking on "Treatment" shortcut opens the index file providing access to analysis files contained in the "GSEA_treatment" folder. [file 1476-4598-8-75-S3.zip › GSEA_treatment/GRANDVAUX_IFN_NOT_IRF3_UP.html]

Details for gene set GRANDVAUX\_IFN\_NOT\_IRF3\_UP[GSEA]

|  || Dataset | gsea\_treatment\_collapsed |
| Phenotype | NoPhenotypeAvailable |
| Upregulated in class | na\_pos |
| GeneSet | GRANDVAUX\_IFN\_NOT\_IRF3\_UP |
| Enrichment Score (ES) | 0.7715657 |
| Normalized Enrichment Score (NES) | 1.843491 |
| Nominal p-value | 0.0011148272 |
| FDR q-value | 0.0016513754 |
| FWER p-Value | 0.03 |
Table: GSEA Results Summary

  

Fig 1: Enrichment plot: GRANDVAUX\_IFN\_NOT\_IRF3\_UP      
 Profile of the Running ES Score & Positions of GeneSet Members on the Rank Ordered List

  

| PROBE | GENE SYMBOL | GENE\_TITLE | RANK IN GENE LIST | RANK METRIC SCORE | RUNNING ES | CORE ENRICHMENT || 1 | MX1 |  |  | 24 | 0.773 | 0.1604 | Yes |
| 2 | IFITM1 |  |  | 79 | 0.609 | 0.2852 | Yes |
| 3 | IRF1 |  |  | 551 | 0.393 | 0.3446 | Yes |
| 4 | ISGF3G |  |  | 612 | 0.383 | 0.4217 | Yes |
| 5 | IRF7 |  |  | 639 | 0.378 | 0.4994 | Yes |
| 6 | IFI6 |  |  | 726 | 0.364 | 0.5712 | Yes |
| 7 | IFI35 |  |  | 766 | 0.357 | 0.6440 | Yes |
| 8 | ADAR |  |  | 885 | 0.344 | 0.7102 | Yes |
| 9 | STAT1 |  |  | 1401 | 0.294 | 0.7467 | Yes |
| 10 | EIF2AK2 |  |  | 1990 | 0.255 | 0.7716 | Yes |
| 11 | ISG20 |  |  | 3782 | 0.186 | 0.7234 | No |
| 12 | IFITM3 |  |  | 8420 | 0.095 | 0.5181 | No |
| 13 | GBP2 |  |  | 19549 | -0.111 | 0.0009 | No |
| 14 | IRGM |  |  | 19690 | -0.119 | 0.0190 | No |
| 15 | MX2 |  |  | 19725 | -0.121 | 0.0427 | No |
Table: GSEA details [plain text format]

  

Fig 2: GRANDVAUX\_IFN\_NOT\_IRF3\_UP: Random ES distribution      
 Gene set null distribution of ES for **GRANDVAUX\_IFN\_NOT\_IRF3\_UP**

  
