# Supplementary material for: The rapamycin-regulated gene expression signature determines prognosis for breast cancer
Source: Mol Cancer. 2009 Sep 24;8:75. doi: 10.1186/1476-4598-8-75 (PMC2761377; doi:10.1186/1476-4598-8-75)
Supplement: Additional file 3 — Gene set enrichment analysis of in vivo data, treatment series. The data provided represent the treatment series of GSEA. This compressed file contains "Treatment" shortcut file and "GSEA_treatment" folder. Clicking on "Treatment" shortcut opens the index file providing access to analysis files contained in the "GSEA_treatment" folder. [file 1476-4598-8-75-S3.zip › GSEA_treatment/gsea_report_for_na_neg_1197924317248.html]

Report for na\_neg 1197924317248 [GSEA]

| GS  follow link to MSigDB | GS DETAILS | SIZE | ES | NES | NOM p-val | FDR q-val | FWER p-val | RANK AT MAX | LEADING EDGE || 1 | CHOLESTEROL\_BIOSYNTHESIS | Details ... | 15 | -0.88 | -2.92 | 0.000 | 0.000 | 0.000 | 399 | tags=60%, list=2%, signal=61% |
| 2 | CPR\_LOW\_LIVER\_UP | Details ... | 17 | -0.70 | -2.46 | 0.000 | 0.000 | 0.000 | 1333 | tags=47%, list=6%, signal=50% |
| 3 | ZHAN\_MM\_MOLECULAR\_CLASSI\_UP | Details ... | 62 | -0.42 | -2.21 | 0.000 | 0.004 | 0.011 | 1497 | tags=34%, list=7%, signal=36% |
| 4 | 5FU\_RESIST\_GASTRIC\_DN | Details ... | 16 | -0.65 | -2.20 | 0.000 | 0.003 | 0.011 | 528 | tags=31%, list=3%, signal=32% |
| 5 | HINATA\_NFKB\_DN | Details ... | 20 | -0.60 | -2.17 | 0.000 | 0.004 | 0.016 | 41 | tags=15%, list=0%, signal=15% |
| 6 | CPR\_NULL\_LIVER\_UP | Details ... | 33 | -0.52 | -1.98 | 0.000 | 0.019 | 0.091 | 1643 | tags=39%, list=8%, signal=43% |
| 7 | GERY\_CEBP\_TARGETS | Details ... | 111 | -0.32 | -1.96 | 0.000 | 0.020 | 0.106 | 515 | tags=15%, list=2%, signal=16% |
| 8 | ZHAN\_MM\_CD138\_CD1\_VS\_REST | Details ... | 44 | -0.44 | -1.94 | 0.000 | 0.022 | 0.132 | 1886 | tags=34%, list=9%, signal=37% |
| 9 | P53GENES\_ALL | Details ... | 17 | -0.51 | -1.74 | 0.011 | 0.071 | 0.405 | 449 | tags=29%, list=2%, signal=30% |
| 10 | ADIP\_DIFF\_CLUSTER2 | Details ... | 40 | -0.39 | -1.67 | 0.026 | 0.101 | 0.558 | 826 | tags=25%, list=4%, signal=26% |
| 11 | ERM\_KO\_SERTOLI\_DN | Details ... | 18 | -0.42 | -1.58 | 0.027 | 0.141 | 0.715 | 1384 | tags=17%, list=7%, signal=18% |
| 12 | ADIP\_VS\_PREADIP\_UP | Details ... | 35 | -0.36 | -1.52 | 0.028 | 0.181 | 0.824 | 162 | tags=14%, list=1%, signal=14% |
| 13 | TSADAC\_PANC50\_UP | Details ... | 42 | -0.36 | -1.51 | 0.000 | 0.174 | 0.836 | 314 | tags=12%, list=2%, signal=12% |
| 14 | LEE\_E2F1\_DN | Details ... | 62 | -0.33 | -1.51 | 0.000 | 0.162 | 0.837 | 1059 | tags=19%, list=5%, signal=20% |
| 15 | BRCA1\_MES\_UP | Details ... | 39 | -0.33 | -1.49 | 0.057 | 0.168 | 0.868 | 880 | tags=21%, list=4%, signal=21% |
| 16 | NO1PATHWAY | Details ... | 29 | -0.36 | -1.45 | 0.042 | 0.203 | 0.928 | 240 | tags=14%, list=1%, signal=14% |
| 17 | ASTON\_OLIGODENDROGLIA\_MYELINATION\_SUBSET | Details ... | 17 | -0.41 | -1.45 | 0.074 | 0.196 | 0.931 | 177 | tags=12%, list=1%, signal=12% |
| 18 | HYPOXIA\_REG\_UP | Details ... | 38 | -0.34 | -1.42 | 0.029 | 0.215 | 0.956 | 994 | tags=26%, list=5%, signal=28% |
| 19 | NADLER\_OBESITY\_DN | Details ... | 36 | -0.33 | -1.41 | 0.033 | 0.217 | 0.964 | 1681 | tags=22%, list=8%, signal=24% |
| 20 | ADIP\_VS\_FIBRO\_UP | Details ... | 34 | -0.35 | -1.40 | 0.053 | 0.212 | 0.970 | 240 | tags=15%, list=1%, signal=15% |
| 21 | UVB\_NHEK3\_C7 | Details ... | 53 | -0.32 | -1.40 | 0.040 | 0.203 | 0.971 | 454 | tags=13%, list=2%, signal=13% |
| 22 | NAKAJIMA\_MCSMBP\_EOS | Details ... | 27 | -0.34 | -1.38 | 0.061 | 0.213 | 0.981 | 1233 | tags=15%, list=6%, signal=16% |
| 23 | TRNA\_SYNTHETASES | Details ... | 19 | -0.37 | -1.37 | 0.127 | 0.212 | 0.982 | 1154 | tags=32%, list=6%, signal=33% |
| 24 | IDX\_TSA\_DN\_CLUSTER4 | Details ... | 27 | -0.35 | -1.32 | 0.089 | 0.261 | 0.996 | 399 | tags=19%, list=2%, signal=19% |
| 25 | LIZUKA\_L0\_GR\_L1 | Details ... | 15 | -0.41 | -1.31 | 0.149 | 0.269 | 0.998 | 6 | tags=7%, list=0%, signal=7% |
| 26 | STRIATED\_MUSCLE\_CONTRACTION | Details ... | 37 | -0.32 | -1.30 | 0.085 | 0.276 | 0.999 | 467 | tags=14%, list=2%, signal=14% |
| 27 | IGF\_VS\_PDGF\_UP | Details ... | 71 | -0.27 | -1.29 | 0.000 | 0.272 | 1.000 | 484 | tags=10%, list=2%, signal=10% |
| 28 | SCHURINGA\_STAT5A\_UP | Details ... | 23 | -0.32 | -1.29 | 0.130 | 0.263 | 1.000 | 249 | tags=17%, list=1%, signal=18% |
| 29 | UVC\_HIGH\_D1\_DN | Details ... | 15 | -0.37 | -1.28 | 0.171 | 0.273 | 1.000 | 343 | tags=13%, list=2%, signal=14% |
| 30 | MYOD\_BRG1\_UP | Details ... | 27 | -0.34 | -1.27 | 0.108 | 0.271 | 1.000 | 123 | tags=15%, list=1%, signal=15% |
| 31 | BUT\_TSA\_UP | Details ... | 18 | -0.34 | -1.26 | 0.187 | 0.269 | 1.000 | 531 | tags=22%, list=3%, signal=23% |
| 32 | AMINOACYL\_TRNA\_BIOSYNTHESIS | Details ... | 23 | -0.32 | -1.25 | 0.180 | 0.275 | 1.000 | 1154 | tags=26%, list=6%, signal=28% |
| 33 | PYRUVATE\_METABOLISM | Details ... | 37 | -0.29 | -1.24 | 0.079 | 0.280 | 1.000 | 77 | tags=8%, list=0%, signal=8% |
| 34 | FERRANDO\_TAL1\_NEIGHBORS | Details ... | 15 | -0.36 | -1.24 | 0.204 | 0.276 | 1.000 | 197 | tags=20%, list=1%, signal=20% |
| 35 | CMV\_UV-CMV\_COMMON\_HCMV\_6HRS\_DN | Details ... | 27 | -0.31 | -1.23 | 0.211 | 0.278 | 1.000 | 36 | tags=7%, list=0%, signal=7% |
| 36 | NEMETH\_TNF\_DN | Details ... | 30 | -0.30 | -1.23 | 0.122 | 0.278 | 1.000 | 273 | tags=13%, list=1%, signal=13% |
| 37 | GLYCOLYSIS\_AND\_GLUCONEOGENESIS | Details ... | 42 | -0.29 | -1.21 | 0.125 | 0.292 | 1.000 | 1321 | tags=19%, list=6%, signal=20% |
| 38 | 4NQO\_UNIQUE\_FIBRO\_UP | Details ... | 22 | -0.31 | -1.19 | 0.194 | 0.309 | 1.000 | 1528 | tags=27%, list=7%, signal=29% |
| 39 | HSP27PATHWAY | Details ... | 15 | -0.34 | -1.11 | 0.328 | 0.436 | 1.000 | 52 | tags=13%, list=0%, signal=13% |
| 40 | WANG\_HOXA9\_VS\_MEIS1\_UP | Details ... | 27 | -0.28 | -1.09 | 0.400 | 0.461 | 1.000 | 11 | tags=4%, list=0%, signal=4% |
| 41 | GLUCONEOGENESIS | Details ... | 52 | -0.23 | -1.09 | 0.261 | 0.454 | 1.000 | 1321 | tags=12%, list=6%, signal=12% |
| 42 | GLYCINE\_SERINE\_AND\_THREONINE\_METABOLISM | Details ... | 35 | -0.23 | -0.98 | 0.548 | 0.614 | 1.000 | 1650 | tags=23%, list=8%, signal=25% |
| 43 | POD1\_KO\_MOST\_DN | Details ... | 25 | -0.26 | -0.95 | 0.594 | 0.661 | 1.000 | 42 | tags=8%, list=0%, signal=8% |
| 44 | CTLA4PATHWAY | Details ... | 18 | -0.26 | -0.93 | 0.591 | 0.678 | 1.000 | 2676 | tags=28%, list=13%, signal=32% |
| 45 | STOSSI\_ER\_UP | Details ... | 47 | -0.21 | -0.93 | 0.556 | 0.675 | 1.000 | 1345 | tags=21%, list=7%, signal=23% |
| 46 | PEPTIDE\_GPCRS | Details ... | 72 | -0.20 | -0.90 | 0.667 | 0.709 | 1.000 | 12378 | tags=92%, list=60%, signal=229% |
| 47 | MTA3PATHWAY | Details ... | 15 | -0.23 | -0.80 | 0.731 | 0.881 | 1.000 | 40 | tags=7%, list=0%, signal=7% |
| 48 | GPCRS\_CLASS\_A\_RHODOPSIN\_LIKE | Details ... | 134 | -0.15 | -0.80 | 1.000 | 0.875 | 1.000 | 1647 | tags=13%, list=8%, signal=14% |
| 49 | FRASOR\_ER\_UP | Details ... | 30 | -0.18 | -0.76 | 0.848 | 0.921 | 1.000 | 920 | tags=13%, list=4%, signal=14% |
| 50 | KIM\_TH\_CELLS\_DN | Details ... | 15 | -0.21 | -0.73 | 0.808 | 0.934 | 1.000 | 1908 | tags=27%, list=9%, signal=29% |
| 51 | CHESLER\_BRAIN\_NEURAL\_HIGH\_GENES |  | 23 | -0.17 | -0.70 | 0.915 | 0.955 | 1.000 | 17024 | tags=100%, list=83%, signal=575% |
| 52 | GAMMA\_HEXACHLOROCYCLOHEXANE\_DEGRADATION |  | 29 | -0.15 | -0.65 | 0.970 | 0.983 | 1.000 | 1333 | tags=10%, list=6%, signal=11% |
| 53 | HUMAN\_TISSUE\_LIVER |  | 37 | -0.17 | -0.65 | 0.880 | 0.967 | 1.000 | 2201 | tags=22%, list=11%, signal=24% |
| 54 | GPCRDB\_OTHER |  | 58 | -0.11 | -0.55 | 1.000 | 0.990 | 1.000 | 16836 | tags=98%, list=82%, signal=536% |
| 55 | GPCRDB\_CLASS\_A\_RHODOPSIN\_LIKE |  | 174 | -0.21 |  |  | 1.000 | 0.000 | 2960 | tags=24%, list=14%, signal=27% |
| 56 | GLYCOLYSIS |  | 52 | -0.23 | -1.09 | 0.294 | 0.444 | 1.000 | 1321 | tags=12%, list=6%, signal=12% |
| 57 | DCPATHWAY |  | 21 | -0.29 | -1.07 | 0.368 | 0.478 | 1.000 | 11210 | tags=95%, list=54%, signal=209% |
| 58 | PENG\_GLUCOSE\_UP |  | 34 | -0.26 | -1.06 | 0.395 | 0.489 | 1.000 | 628 | tags=18%, list=3%, signal=18% |
| 59 | FERRANDO\_T\_CELL\_DIFFERENTIATION\_PATHWAY |  | 18 | -0.29 | -1.03 | 0.397 | 0.527 | 1.000 | 1137 | tags=22%, list=6%, signal=23% |
| 60 | HUMAN\_TISSUE\_THYMUS |  | 16 | -0.28 | -1.01 | 0.388 | 0.550 | 1.000 | 2386 | tags=31%, list=12%, signal=35% |
Table: Gene sets enriched in phenotype **na**[plain text format]****

  
