# Supplementary material for: The rapamycin-regulated gene expression signature determines prognosis for breast cancer
Source: Mol Cancer. 2009 Sep 24;8:75. doi: 10.1186/1476-4598-8-75 (PMC2761377; doi:10.1186/1476-4598-8-75)
Supplement: Additional file 3 — Gene set enrichment analysis of in vivo data, treatment series. The data provided represent the treatment series of GSEA. This compressed file contains "Treatment" shortcut file and "GSEA_treatment" folder. Clicking on "Treatment" shortcut opens the index file providing access to analysis files contained in the "GSEA_treatment" folder. [file 1476-4598-8-75-S3.zip › GSEA_treatment/gsea_report_for_na_pos_1197924317248.html]

Report for na\_pos 1197924317248 [GSEA]

| GS  follow link to MSigDB | GS DETAILS | SIZE | ES | NES | NOM p-val | FDR q-val | FWER p-val | RANK AT MAX | LEADING EDGE || 1 | BENNETT\_SLE\_UP | Details ... | 28 | 0.80 | 2.10 | 0.000 | 0.000 | 0.000 | 1814 | tags=54%, list=9%, signal=59% |
| 2 | IFNALPHA\_NL\_HCC\_UP | Details ... | 18 | 0.85 | 2.08 | 0.000 | 0.000 | 0.000 | 1401 | tags=67%, list=7%, signal=71% |
| 3 | DAC\_IFN\_BLADDER\_UP | Details ... | 16 | 0.85 | 2.04 | 0.000 | 0.000 | 0.000 | 1814 | tags=69%, list=9%, signal=75% |
| 4 | DER\_IFNA\_UP | Details ... | 66 | 0.70 | 2.04 | 0.000 | 0.000 | 0.000 | 2719 | tags=53%, list=13%, signal=61% |
| 5 | SANA\_TNFA\_ENDOTHELIAL\_UP | Details ... | 80 | 0.69 | 2.02 | 0.000 | 0.000 | 0.000 | 3073 | tags=55%, list=15%, signal=64% |
| 6 | RADAEVA\_IFNA\_UP | Details ... | 50 | 0.71 | 2.01 | 0.000 | 0.000 | 0.001 | 3918 | tags=66%, list=19%, signal=81% |
| 7 | SANA\_IFNG\_ENDOTHELIAL\_UP | Details ... | 73 | 0.68 | 2.01 | 0.000 | 0.000 | 0.001 | 2349 | tags=45%, list=11%, signal=51% |
| 8 | IFNALPHA\_NL\_UP | Details ... | 27 | 0.76 | 2.00 | 0.000 | 0.000 | 0.001 | 1401 | tags=56%, list=7%, signal=60% |
| 9 | IFNALPHA\_HCC\_UP | Details ... | 29 | 0.74 | 1.97 | 0.000 | 0.000 | 0.003 | 1757 | tags=52%, list=9%, signal=56% |
| 10 | NF90\_UP | Details ... | 25 | 0.75 | 1.95 | 0.000 | 0.000 | 0.005 | 1814 | tags=48%, list=9%, signal=53% |
| 11 | DAC\_BLADDER\_UP | Details ... | 28 | 0.74 | 1.94 | 0.000 | 0.000 | 0.005 | 1814 | tags=43%, list=9%, signal=47% |
| 12 | TAVOR\_CEBP\_DN | Details ... | 31 | 0.72 | 1.94 | 0.000 | 0.000 | 0.005 | 1224 | tags=35%, list=6%, signal=38% |
| 13 | OXSTRESS\_RPETHREE\_DN | Details ... | 28 | 0.73 | 1.93 | 0.000 | 0.000 | 0.006 | 3398 | tags=54%, list=16%, signal=64% |
| 14 | DER\_IFNG\_UP | Details ... | 62 | 0.67 | 1.92 | 0.000 | 0.000 | 0.007 | 2719 | tags=52%, list=13%, signal=59% |
| 15 | WIELAND\_HEPATITIS\_B\_INDUCED | Details ... | 106 | 0.63 | 1.87 | 0.000 | 0.001 | 0.019 | 1129 | tags=30%, list=5%, signal=32% |
| 16 | IFN\_ALPHA\_UP | Details ... | 40 | 0.68 | 1.87 | 0.000 | 0.001 | 0.021 | 2719 | tags=50%, list=13%, signal=57% |
| 17 | RIBAVIRIN\_RSV\_UP | Details ... | 22 | 0.73 | 1.84 | 0.000 | 0.002 | 0.030 | 3757 | tags=64%, list=18%, signal=78% |
| 18 | GRANDVAUX\_IFN\_NOT\_IRF3\_UP | Details ... | 15 | 0.77 | 1.84 | 0.001 | 0.002 | 0.030 | 1990 | tags=67%, list=10%, signal=74% |
| 19 | DSRNA\_UP | Details ... | 38 | 0.66 | 1.83 | 0.000 | 0.002 | 0.031 | 2614 | tags=39%, list=13%, signal=45% |
| 20 | APPEL\_IMATINIB\_UP | Details ... | 31 | 0.67 | 1.83 | 0.000 | 0.002 | 0.033 | 4275 | tags=61%, list=21%, signal=77% |
| 21 | MRNA\_PROCESSING | Details ... | 42 | 0.66 | 1.83 | 0.000 | 0.002 | 0.033 | 4607 | tags=67%, list=22%, signal=86% |
| 22 | RESISTANCE\_XENOGRAFTS\_UP | Details ... | 28 | 0.69 | 1.81 | 0.000 | 0.003 | 0.057 | 2547 | tags=50%, list=12%, signal=57% |
| 23 | IFN\_BETA\_UP | Details ... | 65 | 0.62 | 1.80 | 0.000 | 0.003 | 0.065 | 2719 | tags=45%, list=13%, signal=51% |
| 24 | TNFALPHA\_ALL\_UP | Details ... | 77 | 0.60 | 1.78 | 0.000 | 0.004 | 0.093 | 3199 | tags=44%, list=16%, signal=52% |
| 25 | IFN\_ANY\_UP | Details ... | 81 | 0.61 | 1.78 | 0.000 | 0.004 | 0.106 | 3795 | tags=48%, list=18%, signal=59% |
| 26 | IFN\_ALL\_UP | Details ... | 18 | 0.72 | 1.77 | 0.002 | 0.005 | 0.119 | 2719 | tags=61%, list=13%, signal=70% |
| 27 | CMV\_HCMV\_TIMECOURSE\_12HRS\_UP | Details ... | 26 | 0.67 | 1.76 | 0.000 | 0.005 | 0.135 | 3782 | tags=50%, list=18%, signal=61% |
| 28 | UVB\_SCC\_UP | Details ... | 87 | 0.59 | 1.75 | 0.000 | 0.006 | 0.169 | 4336 | tags=52%, list=21%, signal=65% |
| 29 | BRCA1\_OVEREXP\_UP | Details ... | 159 | 0.57 | 1.74 | 0.000 | 0.008 | 0.226 | 4587 | tags=52%, list=22%, signal=67% |
| 30 | IFNA\_HCMV\_6HRS\_UP | Details ... | 53 | 0.62 | 1.74 | 0.000 | 0.009 | 0.247 | 2149 | tags=36%, list=10%, signal=40% |
| 31 | CROONQUIST\_RAS\_STROMA\_DN | Details ... | 19 | 0.69 | 1.73 | 0.001 | 0.009 | 0.252 | 1676 | tags=32%, list=8%, signal=34% |
| 32 | HDACI\_COLON\_SUL24HRS\_DN | Details ... | 127 | 0.57 | 1.73 | 0.000 | 0.010 | 0.275 | 4387 | tags=48%, list=21%, signal=61% |
| 33 | GALE\_FLT3ANDAPL\_DN | Details ... | 18 | 0.70 | 1.73 | 0.000 | 0.010 | 0.285 | 3196 | tags=61%, list=16%, signal=72% |
| 34 | CROMER\_HYPOPHARYNGEAL\_MET\_VS\_NON\_UP | Details ... | 72 | 0.59 | 1.72 | 0.000 | 0.010 | 0.294 | 4873 | tags=53%, list=24%, signal=69% |
| 35 | TSA\_PANC50\_UP | Details ... | 38 | 0.62 | 1.72 | 0.000 | 0.010 | 0.316 | 570 | tags=18%, list=3%, signal=19% |
| 36 | HDACI\_COLON\_BUT30MIN\_DN | Details ... | 37 | 0.63 | 1.72 | 0.001 | 0.010 | 0.317 | 4224 | tags=54%, list=20%, signal=68% |
| 37 | TUMOR\_SUPRESSOR | Details ... | 23 | 0.67 | 1.72 | 0.001 | 0.010 | 0.331 | 3846 | tags=52%, list=19%, signal=64% |
| 38 | ADIP\_DIFF\_CLUSTER5 | Details ... | 39 | 0.63 | 1.72 | 0.001 | 0.010 | 0.332 | 1624 | tags=36%, list=8%, signal=39% |
| 39 | UV-4NQO\_FIBRO\_UP | Details ... | 28 | 0.65 | 1.72 | 0.002 | 0.010 | 0.332 | 4175 | tags=57%, list=20%, signal=72% |
| 40 | HIPPOCAMPUS\_DEVELOPMENT\_PRENATAL | Details ... | 33 | 0.64 | 1.71 | 0.000 | 0.010 | 0.339 | 1636 | tags=42%, list=8%, signal=46% |
| 41 | TGFBETA\_EARLY\_UP | Details ... | 47 | 0.61 | 1.71 | 0.000 | 0.010 | 0.346 | 5670 | tags=57%, list=28%, signal=79% |
| 42 | LEE\_TCELLS9\_UP | Details ... | 31 | 0.64 | 1.71 | 0.000 | 0.011 | 0.368 | 3107 | tags=48%, list=15%, signal=57% |
| 43 | IDX\_TSA\_DN\_CLUSTER5 | Details ... | 46 | 0.61 | 1.71 | 0.000 | 0.011 | 0.379 | 3576 | tags=41%, list=17%, signal=50% |
| 44 | TGFBETA\_ALL\_UP | Details ... | 80 | 0.57 | 1.70 | 0.000 | 0.012 | 0.417 | 5781 | tags=55%, list=28%, signal=76% |
| 45 | IFN\_GAMMA\_UP | Details ... | 38 | 0.62 | 1.69 | 0.000 | 0.014 | 0.462 | 2719 | tags=47%, list=13%, signal=54% |
| 46 | PARK\_HSC\_VS\_MPP\_UP | Details ... | 16 | 0.70 | 1.69 | 0.002 | 0.013 | 0.469 | 605 | tags=31%, list=3%, signal=32% |
| 47 | CHAUHAN\_2ME2 | Details ... | 46 | 0.60 | 1.69 | 0.000 | 0.014 | 0.499 | 2150 | tags=43%, list=10%, signal=48% |
| 48 | HDACI\_COLON\_SUL\_DN | Details ... | 215 | 0.54 | 1.68 | 0.000 | 0.015 | 0.515 | 4968 | tags=51%, list=24%, signal=66% |
| 49 | NI2\_MOUSE\_UP | Details ... | 39 | 0.61 | 1.68 | 0.000 | 0.015 | 0.529 | 1881 | tags=31%, list=9%, signal=34% |
| 50 | TNFA\_NFKB\_DEP\_UP | Details ... | 18 | 0.68 | 1.68 | 0.003 | 0.014 | 0.529 | 457 | tags=33%, list=2%, signal=34% |
| 51 | LVAD\_HEARTFAILURE\_DN |  | 38 | 0.61 | 1.68 | 0.000 | 0.014 | 0.537 | 3394 | tags=34%, list=16%, signal=41% |
| 52 | JECHLINGER\_EMT\_DN |  | 40 | 0.61 | 1.68 | 0.000 | 0.014 | 0.537 | 3789 | tags=48%, list=18%, signal=58% |
| 53 | O6BG\_RESIST\_MEDULLOBLASTOMA\_DN |  | 51 | 0.60 | 1.68 | 0.000 | 0.014 | 0.546 | 5169 | tags=53%, list=25%, signal=70% |
| 54 | GILDEA\_BLADDER\_UP |  | 30 | 0.63 | 1.68 | 0.000 | 0.014 | 0.549 | 2898 | tags=37%, list=14%, signal=43% |
| 55 | HDACI\_COLON\_CLUSTER6 |  | 43 | 0.60 | 1.67 | 0.000 | 0.015 | 0.576 | 5140 | tags=60%, list=25%, signal=80% |
| 56 | O6BG\_RESIST\_MEDULLOBLASTOMA\_UP |  | 23 | 0.65 | 1.67 | 0.002 | 0.015 | 0.586 | 3849 | tags=35%, list=19%, signal=43% |
| 57 | CANTHARIDIN\_DN |  | 50 | 0.60 | 1.67 | 0.000 | 0.015 | 0.588 | 3774 | tags=46%, list=18%, signal=56% |
| 58 | STEFFEN\_AML\_PML\_PLZF\_TRGT |  | 43 | 0.59 | 1.67 | 0.001 | 0.016 | 0.626 | 4786 | tags=56%, list=23%, signal=73% |
| 59 | WELCSH\_BRCA\_UP |  | 39 | 0.61 | 1.67 | 0.001 | 0.016 | 0.629 | 2150 | tags=36%, list=10%, signal=40% |
| 60 | ZHAN\_MM\_MOLECULAR\_CLASSI\_DN |  | 50 | 0.59 | 1.67 | 0.000 | 0.016 | 0.629 | 2270 | tags=30%, list=11%, signal=34% |
| 61 | ZUCCHI\_EPITHELIAL\_UP |  | 42 | 0.61 | 1.66 | 0.000 | 0.017 | 0.648 | 3490 | tags=40%, list=17%, signal=49% |
| 62 | PEART\_HISTONE\_DN |  | 73 | 0.57 | 1.66 | 0.000 | 0.017 | 0.664 | 5327 | tags=56%, list=26%, signal=75% |
| 63 | LINDSTEDT\_DEND\_8H\_VS\_48H\_UP |  | 64 | 0.57 | 1.66 | 0.000 | 0.017 | 0.672 | 1758 | tags=27%, list=9%, signal=29% |
| 64 | PARK\_MSCS\_BOTH |  | 44 | 0.60 | 1.66 | 0.000 | 0.017 | 0.682 | 5500 | tags=59%, list=27%, signal=80% |
| 65 | CHEN\_HOXA5\_TARGETS\_DN |  | 47 | 0.59 | 1.66 | 0.001 | 0.017 | 0.683 | 4568 | tags=49%, list=22%, signal=63% |
| 66 | MOREAUX\_TACI\_HI\_VS\_LOW\_DN |  | 170 | 0.54 | 1.65 | 0.000 | 0.019 | 0.716 | 5431 | tags=54%, list=26%, signal=73% |
| 67 | MMS\_HUMAN\_LYMPH\_HIGH\_24HRS\_UP |  | 19 | 0.67 | 1.65 | 0.004 | 0.019 | 0.719 | 5068 | tags=63%, list=25%, signal=84% |
| 68 | LIAN\_MYELOID\_DIFF\_GRANULE |  | 24 | 0.64 | 1.65 | 0.001 | 0.018 | 0.721 | 2545 | tags=38%, list=12%, signal=43% |
| 69 | CMV\_24HRS\_UP |  | 72 | 0.57 | 1.65 | 0.000 | 0.018 | 0.726 | 4980 | tags=51%, list=24%, signal=68% |
| 70 | TNFALPHA\_30MIN\_UP |  | 42 | 0.59 | 1.65 | 0.000 | 0.019 | 0.738 | 3199 | tags=45%, list=16%, signal=53% |
| 71 | IDX\_TSA\_UP\_CLUSTER3 |  | 88 | 0.56 | 1.65 | 0.000 | 0.019 | 0.745 | 5500 | tags=51%, list=27%, signal=69% |
| 72 | HINATA\_NFKB\_UP |  | 106 | 0.55 | 1.65 | 0.000 | 0.019 | 0.750 | 3717 | tags=42%, list=18%, signal=50% |
| 73 | LIN\_WNT\_UP |  | 53 | 0.58 | 1.65 | 0.000 | 0.019 | 0.757 | 3788 | tags=49%, list=18%, signal=60% |
| 74 | HDACI\_COLON\_SUL16HRS\_DN |  | 72 | 0.57 | 1.64 | 0.000 | 0.019 | 0.761 | 5061 | tags=54%, list=25%, signal=72% |
| 75 | BRCA\_BRCA1\_NEG |  | 154 | 0.54 | 1.64 | 0.000 | 0.019 | 0.769 | 4678 | tags=42%, list=23%, signal=53% |
| 76 | PRMT5\_KD\_UP |  | 183 | 0.54 | 1.64 | 0.000 | 0.019 | 0.769 | 5601 | tags=51%, list=27%, signal=70% |
| 77 | JNK\_UP |  | 30 | 0.62 | 1.64 | 0.002 | 0.019 | 0.783 | 4084 | tags=47%, list=20%, signal=58% |
| 78 | HBX\_HEP\_UP |  | 18 | 0.66 | 1.64 | 0.006 | 0.019 | 0.787 | 4738 | tags=61%, list=23%, signal=79% |
| 79 | XU\_ATRA\_PLUSNSC\_UP |  | 15 | 0.69 | 1.64 | 0.005 | 0.020 | 0.799 | 2033 | tags=40%, list=10%, signal=44% |
| 80 | ABRAHAM\_MM\_VS\_AL\_UP |  | 19 | 0.66 | 1.63 | 0.004 | 0.021 | 0.819 | 4385 | tags=63%, list=21%, signal=80% |
| 81 | CMV\_HCMV\_TIMECOURSE\_20HRS\_DN |  | 40 | 0.59 | 1.63 | 0.001 | 0.020 | 0.819 | 4713 | tags=52%, list=23%, signal=68% |
| 82 | LEE\_TCELLS4\_UP |  | 58 | 0.57 | 1.63 | 0.000 | 0.020 | 0.819 | 2311 | tags=28%, list=11%, signal=31% |
| 83 | XU\_ATRA\_UP |  | 15 | 0.68 | 1.63 | 0.002 | 0.021 | 0.826 | 4493 | tags=60%, list=22%, signal=77% |
| 84 | MRNA\_SPLICING |  | 49 | 0.58 | 1.63 | 0.000 | 0.021 | 0.835 | 4607 | tags=51%, list=22%, signal=66% |
| 85 | YANG\_OSTECLASTS\_SIG |  | 38 | 0.59 | 1.63 | 0.001 | 0.021 | 0.840 | 925 | tags=16%, list=4%, signal=17% |
| 86 | LE\_MYELIN\_UP |  | 89 | 0.55 | 1.62 | 0.000 | 0.022 | 0.854 | 5500 | tags=51%, list=27%, signal=69% |
| 87 | REOVIRUS\_HEK293\_DN |  | 230 | 0.52 | 1.62 | 0.000 | 0.022 | 0.858 | 5307 | tags=50%, list=26%, signal=67% |
| 88 | TAKEDA\_NUP8\_HOXA9\_3D\_DN |  | 30 | 0.60 | 1.62 | 0.002 | 0.023 | 0.868 | 2211 | tags=23%, list=11%, signal=26% |
| 89 | DISTECHE\_XINACTIVATED\_GENES |  | 19 | 0.66 | 1.62 | 0.007 | 0.022 | 0.868 | 4989 | tags=63%, list=24%, signal=83% |
| 90 | PENG\_RAPAMYCIN\_UP |  | 154 | 0.53 | 1.62 | 0.000 | 0.022 | 0.868 | 3672 | tags=38%, list=18%, signal=46% |
| 91 | IL6\_FIBRO\_UP |  | 47 | 0.58 | 1.62 | 0.001 | 0.022 | 0.870 | 3974 | tags=40%, list=19%, signal=50% |
| 92 | TNFALPHA\_4HRS\_UP |  | 40 | 0.58 | 1.61 | 0.003 | 0.024 | 0.895 | 2634 | tags=35%, list=13%, signal=40% |
| 93 | DNMT1\_KO\_UP |  | 72 | 0.55 | 1.61 | 0.000 | 0.024 | 0.901 | 3054 | tags=35%, list=15%, signal=41% |
| 94 | AS3\_FIBRO\_DN |  | 31 | 0.60 | 1.61 | 0.002 | 0.024 | 0.902 | 3199 | tags=39%, list=16%, signal=46% |
| 95 | HDACI\_COLON\_SUL30MIN\_DN |  | 38 | 0.59 | 1.61 | 0.002 | 0.025 | 0.904 | 4923 | tags=58%, list=24%, signal=76% |
| 96 | IL6\_SCAR\_FIBRO\_UP |  | 24 | 0.63 | 1.61 | 0.004 | 0.024 | 0.904 | 4779 | tags=46%, list=23%, signal=60% |
| 97 | VHL\_RCC\_UP |  | 110 | 0.54 | 1.61 | 0.000 | 0.024 | 0.909 | 5165 | tags=54%, list=25%, signal=71% |
| 98 | CMV\_8HRS\_UP |  | 32 | 0.59 | 1.61 | 0.001 | 0.025 | 0.918 | 3400 | tags=41%, list=17%, signal=49% |
| 99 | TIS7\_OVEREXP\_DN |  | 19 | 0.64 | 1.61 | 0.005 | 0.026 | 0.920 | 5428 | tags=63%, list=26%, signal=86% |
| 100 | ELECTRON\_TRANSPORT\_CHAIN |  | 98 | 0.53 | 1.60 | 0.000 | 0.026 | 0.925 | 6192 | tags=55%, list=30%, signal=78% |
| 101 | AGUIRRE\_PANCREAS\_CHR1 |  | 31 | 0.60 | 1.60 | 0.003 | 0.026 | 0.934 | 4431 | tags=42%, list=22%, signal=53% |
| 102 | FSH\_GRANULOSA\_DN |  | 76 | 0.55 | 1.60 | 0.000 | 0.026 | 0.934 | 4174 | tags=42%, list=20%, signal=53% |
| 103 | DNA\_REPLICATION\_REACTOME |  | 44 | 0.57 | 1.60 | 0.001 | 0.026 | 0.942 | 4860 | tags=52%, list=24%, signal=68% |
| 104 | FSH\_OVARY\_MCV152\_DN |  | 46 | 0.57 | 1.60 | 0.000 | 0.026 | 0.943 | 5004 | tags=59%, list=24%, signal=77% |
| 105 | HDACI\_COLON\_SUL12HRS\_DN |  | 27 | 0.61 | 1.60 | 0.002 | 0.026 | 0.944 | 6259 | tags=78%, list=30%, signal=112% |
| 106 | MMS\_MOUSE\_LYMPH\_HIGH\_24HRS\_UP |  | 22 | 0.62 | 1.60 | 0.004 | 0.026 | 0.947 | 3601 | tags=45%, list=17%, signal=55% |
| 107 | LH\_GRANULOSA\_DN |  | 76 | 0.55 | 1.60 | 0.000 | 0.027 | 0.951 | 4174 | tags=42%, list=20%, signal=53% |
| 108 | DAVIES\_MGUS\_MM |  | 35 | 0.58 | 1.60 | 0.003 | 0.027 | 0.953 | 4211 | tags=43%, list=20%, signal=54% |
| 109 | NING\_COPD\_UP |  | 144 | 0.53 | 1.60 | 0.000 | 0.027 | 0.953 | 4511 | tags=42%, list=22%, signal=54% |
| 110 | HDACI\_COLON\_SUL48HRS\_DN |  | 81 | 0.55 | 1.60 | 0.000 | 0.027 | 0.955 | 5992 | tags=56%, list=29%, signal=78% |
| 111 | IDX\_TSA\_DN\_CLUSTER3 |  | 78 | 0.55 | 1.60 | 0.000 | 0.027 | 0.955 | 3955 | tags=49%, list=19%, signal=60% |
| 112 | VHL\_NORMAL\_UP |  | 438 | 0.51 | 1.60 | 0.000 | 0.027 | 0.959 | 4472 | tags=43%, list=22%, signal=54% |
| 113 | ZHAN\_MMPC\_SIM |  | 42 | 0.57 | 1.59 | 0.000 | 0.027 | 0.961 | 3400 | tags=40%, list=17%, signal=48% |
| 114 | BLEO\_HUMAN\_LYMPH\_HIGH\_24HRS\_UP |  | 92 | 0.54 | 1.59 | 0.000 | 0.028 | 0.970 | 4708 | tags=41%, list=23%, signal=53% |
| 115 | ROSS\_CBF\_MYH |  | 54 | 0.56 | 1.59 | 0.001 | 0.029 | 0.973 | 4631 | tags=39%, list=22%, signal=50% |
| 116 | CITED1\_KO\_WT\_UP |  | 16 | 0.66 | 1.59 | 0.014 | 0.029 | 0.973 | 4456 | tags=63%, list=22%, signal=80% |
| 117 | ZHAN\_MULTIPLE\_MYELOMA\_SUBCLASSES\_DIFF |  | 30 | 0.60 | 1.59 | 0.003 | 0.029 | 0.976 | 5367 | tags=53%, list=26%, signal=72% |
| 118 | HASLINGER\_B\_CLL\_17P13 |  | 16 | 0.66 | 1.58 | 0.008 | 0.030 | 0.976 | 6068 | tags=75%, list=29%, signal=106% |
| 119 | CANCER\_NEOPLASTIC\_META\_UP |  | 61 | 0.55 | 1.58 | 0.000 | 0.031 | 0.976 | 3984 | tags=46%, list=19%, signal=57% |
| 120 | REN\_E2F1\_TARGETS |  | 38 | 0.57 | 1.58 | 0.002 | 0.030 | 0.976 | 4403 | tags=50%, list=21%, signal=63% |
| 121 | H2O2\_CSBRESCUED\_C1\_UP |  | 42 | 0.57 | 1.58 | 0.000 | 0.030 | 0.976 | 3947 | tags=43%, list=19%, signal=53% |
| 122 | DAC\_FIBRO\_UP |  | 17 | 0.65 | 1.58 | 0.006 | 0.031 | 0.976 | 3857 | tags=41%, list=19%, signal=51% |
| 123 | FALT\_BCLL\_IG\_MUTATED\_VS\_WT\_DN |  | 48 | 0.57 | 1.58 | 0.002 | 0.031 | 0.977 | 5531 | tags=58%, list=27%, signal=80% |
| 124 | PARK\_MSCS\_DIFF |  | 31 | 0.58 | 1.58 | 0.005 | 0.031 | 0.978 | 4843 | tags=52%, list=24%, signal=67% |
| 125 | MRNA\_PROCESSING\_REACTOME |  | 108 | 0.53 | 1.58 | 0.000 | 0.031 | 0.978 | 6270 | tags=62%, list=30%, signal=89% |
| 126 | ADIP\_DIFF\_CLUSTER4 |  | 34 | 0.59 | 1.58 | 0.002 | 0.031 | 0.980 | 5463 | tags=62%, list=27%, signal=84% |
| 127 | BREASTCA\_TWO\_CLASSES |  | 138 | 0.52 | 1.58 | 0.000 | 0.031 | 0.981 | 5341 | tags=48%, list=26%, signal=64% |
| 128 | UVB\_NHEK3\_C2 |  | 43 | 0.57 | 1.58 | 0.001 | 0.031 | 0.981 | 4820 | tags=56%, list=23%, signal=73% |
| 129 | BRCA1\_OVEREXP\_PROSTATE\_UP |  | 159 | 0.52 | 1.57 | 0.000 | 0.032 | 0.983 | 4026 | tags=36%, list=20%, signal=45% |
| 130 | GH\_GHRHR\_KO\_24HRS\_DN |  | 172 | 0.51 | 1.57 | 0.000 | 0.032 | 0.983 | 6529 | tags=54%, list=32%, signal=78% |
| 131 | HOHENKIRK\_MONOCYTE\_DEND\_DN |  | 119 | 0.52 | 1.57 | 0.000 | 0.032 | 0.984 | 2671 | tags=31%, list=13%, signal=36% |
| 132 | FLECHNER\_KIDNEY\_TRANSPLANT\_WELL\_PBL\_UP |  | 153 | 0.52 | 1.57 | 0.000 | 0.032 | 0.984 | 6161 | tags=57%, list=30%, signal=81% |
| 133 | HDACI\_COLON\_BUT2HRS\_UP |  | 75 | 0.53 | 1.57 | 0.000 | 0.032 | 0.984 | 5950 | tags=53%, list=29%, signal=75% |
| 134 | ZHAN\_MMPC\_SIM\_BC\_AND\_MM |  | 45 | 0.57 | 1.57 | 0.002 | 0.033 | 0.987 | 6111 | tags=67%, list=30%, signal=95% |
| 135 | G1\_TO\_S\_CELL\_CYCLE\_REACTOME |  | 68 | 0.54 | 1.57 | 0.001 | 0.033 | 0.987 | 4323 | tags=41%, list=21%, signal=52% |
| 136 | HDACI\_COLON\_CUR48HRS\_UP |  | 62 | 0.54 | 1.57 | 0.001 | 0.033 | 0.987 | 4552 | tags=47%, list=22%, signal=60% |
| 137 | NICK\_RHAPC\_UP |  | 32 | 0.59 | 1.57 | 0.002 | 0.033 | 0.987 | 4989 | tags=53%, list=24%, signal=70% |
| 138 | HDACI\_COLON\_BUT12HRS\_UP |  | 45 | 0.56 | 1.57 | 0.000 | 0.033 | 0.987 | 4540 | tags=47%, list=22%, signal=60% |
| 139 | RETT\_UP |  | 40 | 0.57 | 1.57 | 0.000 | 0.033 | 0.989 | 4794 | tags=35%, list=23%, signal=46% |
| 140 | CALRES\_MOUSE\_NEOCORTEX\_DN |  | 66 | 0.54 | 1.56 | 0.001 | 0.034 | 0.990 | 5292 | tags=50%, list=26%, signal=67% |
| 141 | RIBOSOMAL\_PROTEINS |  | 96 | 0.52 | 1.56 | 0.000 | 0.034 | 0.992 | 6778 | tags=65%, list=33%, signal=96% |
| 142 | EMT\_DN |  | 54 | 0.55 | 1.56 | 0.002 | 0.034 | 0.992 | 3789 | tags=48%, list=18%, signal=59% |
| 143 | CMV\_IE86\_UP |  | 49 | 0.56 | 1.56 | 0.001 | 0.034 | 0.992 | 5118 | tags=57%, list=25%, signal=76% |
| 144 | BECKER\_TAMOXIFEN\_RESISTANT\_UP |  | 40 | 0.56 | 1.56 | 0.002 | 0.034 | 0.992 | 3001 | tags=40%, list=15%, signal=47% |
| 145 | HYPOXIA\_RCC\_NOVHL\_UP |  | 66 | 0.54 | 1.56 | 0.000 | 0.034 | 0.992 | 4727 | tags=45%, list=23%, signal=59% |
| 146 | FLECHNER\_KIDNEY\_TRANSPLANT\_REJECTION\_PBL\_DN |  | 51 | 0.55 | 1.56 | 0.004 | 0.034 | 0.992 | 6642 | tags=65%, list=32%, signal=95% |
| 147 | IFNA\_UV-CMV\_COMMON\_HCMV\_6HRS\_UP |  | 29 | 0.58 | 1.56 | 0.005 | 0.034 | 0.993 | 2149 | tags=31%, list=10%, signal=35% |
| 148 | ET743\_SARCOMA\_UP |  | 69 | 0.53 | 1.56 | 0.000 | 0.034 | 0.993 | 3199 | tags=39%, list=16%, signal=46% |
| 149 | HASLINGER\_B\_CLL\_13Q14 |  | 19 | 0.62 | 1.56 | 0.012 | 0.034 | 0.993 | 2022 | tags=32%, list=10%, signal=35% |
| 150 | AGUIRRE\_PANCREAS\_CHR8 |  | 61 | 0.54 | 1.56 | 0.000 | 0.034 | 0.993 | 4522 | tags=49%, list=22%, signal=63% |
| 151 | FETAL\_LIVER\_ENRICHED\_TRANSCRIPTION\_FACTORS |  | 75 | 0.53 | 1.56 | 0.001 | 0.034 | 0.993 | 4651 | tags=43%, list=23%, signal=55% |
| 152 | SASAKI\_TCELL\_LYMPHOMA\_VS\_CD4\_UP |  | 168 | 0.50 | 1.56 | 0.000 | 0.034 | 0.993 | 5678 | tags=46%, list=28%, signal=64% |
| 153 | WALKER\_MM\_SNP\_DIFF |  | 44 | 0.56 | 1.56 | 0.002 | 0.034 | 0.993 | 3699 | tags=48%, list=18%, signal=58% |
| 154 | GN\_CAMP\_GRANULOSA\_DN |  | 61 | 0.54 | 1.56 | 0.001 | 0.034 | 0.993 | 3729 | tags=39%, list=18%, signal=48% |
| 155 | ABRAHAM\_AL\_VS\_MM\_DN |  | 18 | 0.64 | 1.56 | 0.013 | 0.034 | 0.994 | 4385 | tags=61%, list=21%, signal=78% |
| 156 | HYPOXIA\_RCC\_UP |  | 102 | 0.52 | 1.56 | 0.001 | 0.034 | 0.994 | 4084 | tags=43%, list=20%, signal=54% |
| 157 | LEE\_TCELLS1\_UP |  | 201 | 0.51 | 1.56 | 0.000 | 0.034 | 0.994 | 5009 | tags=44%, list=24%, signal=57% |
| 158 | HOFMANN\_MANTEL\_LYMPHOMA\_VS\_LYMPH\_NODES\_UP |  | 49 | 0.56 | 1.56 | 0.002 | 0.034 | 0.994 | 4771 | tags=43%, list=23%, signal=56% |
| 159 | LEE\_CIP\_UP |  | 60 | 0.54 | 1.56 | 0.002 | 0.034 | 0.994 | 1555 | tags=22%, list=8%, signal=23% |
| 160 | LEE\_TCELLS8\_UP |  | 201 | 0.51 | 1.55 | 0.000 | 0.034 | 0.995 | 5009 | tags=44%, list=24%, signal=57% |
| 161 | 4NQO\_ESR\_WS\_UNREG |  | 35 | 0.57 | 1.55 | 0.006 | 0.034 | 0.997 | 4856 | tags=54%, list=24%, signal=71% |
| 162 | BRCA1\_OVEREXP\_PROSTATE\_DN |  | 78 | 0.53 | 1.55 | 0.000 | 0.035 | 0.997 | 5088 | tags=47%, list=25%, signal=63% |
| 163 | TRANSLATION\_FACTORS |  | 47 | 0.55 | 1.55 | 0.001 | 0.035 | 0.997 | 4326 | tags=43%, list=21%, signal=54% |
| 164 | CELL\_CYCLE\_REGULATOR |  | 24 | 0.60 | 1.55 | 0.008 | 0.035 | 0.997 | 4584 | tags=50%, list=22%, signal=64% |
| 165 | CIS\_XPC\_DN |  | 188 | 0.51 | 1.55 | 0.000 | 0.035 | 0.997 | 5185 | tags=46%, list=25%, signal=61% |
| 166 | LEE\_TCELLS10\_UP |  | 201 | 0.51 | 1.55 | 0.000 | 0.035 | 0.998 | 5009 | tags=44%, list=24%, signal=57% |
| 167 | ZHAN\_MM\_CD138\_HP\_VS\_REST |  | 48 | 0.56 | 1.55 | 0.002 | 0.036 | 0.998 | 3107 | tags=38%, list=15%, signal=44% |
| 168 | MITOCHONDRIA |  | 391 | 0.49 | 1.55 | 0.000 | 0.036 | 0.998 | 6192 | tags=52%, list=30%, signal=74% |
| 169 | BRCA2\_BRCA1\_UP |  | 48 | 0.55 | 1.55 | 0.006 | 0.036 | 0.998 | 5096 | tags=50%, list=25%, signal=66% |
| 170 | STEMCELL\_COMMON\_UP |  | 180 | 0.50 | 1.55 | 0.000 | 0.036 | 0.999 | 5593 | tags=53%, list=27%, signal=73% |
| 171 | PASSERINI\_GROWTH |  | 33 | 0.56 | 1.55 | 0.006 | 0.036 | 0.999 | 1638 | tags=24%, list=8%, signal=26% |
| 172 | CMV\_ALL\_UP |  | 93 | 0.52 | 1.55 | 0.000 | 0.036 | 0.999 | 4980 | tags=46%, list=24%, signal=61% |
| 173 | TAKEDA\_NUP8\_HOXA9\_3D\_UP |  | 187 | 0.50 | 1.54 | 0.000 | 0.036 | 0.999 | 3233 | tags=32%, list=16%, signal=38% |
| 174 | HDACI\_COLON\_CLUSTER9 |  | 65 | 0.53 | 1.54 | 0.004 | 0.037 | 0.999 | 5083 | tags=49%, list=25%, signal=65% |
| 175 | RUIZ\_TENASCIN\_TARGETS |  | 79 | 0.53 | 1.54 | 0.001 | 0.037 | 0.999 | 3849 | tags=39%, list=19%, signal=48% |
| 176 | UVC\_HIGH\_D2\_DN |  | 37 | 0.56 | 1.54 | 0.004 | 0.037 | 0.999 | 3637 | tags=38%, list=18%, signal=46% |
| 177 | TSA\_RKO\_UP |  | 17 | 0.63 | 1.54 | 0.015 | 0.037 | 0.999 | 4336 | tags=59%, list=21%, signal=74% |
| 178 | SASAKI\_ATL\_UP |  | 168 | 0.50 | 1.54 | 0.000 | 0.038 | 1.000 | 5678 | tags=46%, list=28%, signal=64% |
| 179 | ZUCCHI\_EPITHELIAL\_DN |  | 43 | 0.55 | 1.54 | 0.003 | 0.039 | 1.000 | 4480 | tags=49%, list=22%, signal=62% |
| 180 | KENNY\_WNT\_UP |  | 46 | 0.55 | 1.53 | 0.004 | 0.039 | 1.000 | 3064 | tags=41%, list=15%, signal=48% |
| 181 | UBIQUITIN\_MEDIATED\_PROTEOLYSIS |  | 23 | 0.59 | 1.53 | 0.013 | 0.040 | 1.000 | 6949 | tags=78%, list=34%, signal=118% |
| 182 | JISON\_SICKLECELL\_DIFF |  | 368 | 0.49 | 1.53 | 0.000 | 0.041 | 1.000 | 4886 | tags=43%, list=24%, signal=56% |
| 183 | HTERT\_UP |  | 67 | 0.52 | 1.53 | 0.001 | 0.041 | 1.000 | 3737 | tags=36%, list=18%, signal=44% |
| 184 | OLDONLY\_FIBRO\_UP |  | 36 | 0.56 | 1.52 | 0.008 | 0.044 | 1.000 | 5019 | tags=53%, list=24%, signal=70% |
| 185 | MYC\_TARGETS |  | 40 | 0.55 | 1.52 | 0.003 | 0.044 | 1.000 | 5198 | tags=50%, list=25%, signal=67% |
| 186 | CORDERO\_KRAS\_KD\_VS\_CONTROL\_UP |  | 74 | 0.52 | 1.52 | 0.000 | 0.046 | 1.000 | 4255 | tags=38%, list=21%, signal=48% |
| 187 | AGED\_MOUSE\_CORTEX\_DN |  | 46 | 0.55 | 1.52 | 0.005 | 0.046 | 1.000 | 6806 | tags=63%, list=33%, signal=94% |
| 188 | CROONQUIST\_IL6\_RAS\_DN |  | 24 | 0.59 | 1.52 | 0.012 | 0.046 | 1.000 | 5753 | tags=54%, list=28%, signal=75% |
| 189 | GOLDRATH\_HP |  | 147 | 0.50 | 1.52 | 0.000 | 0.046 | 1.000 | 6593 | tags=54%, list=32%, signal=79% |
| 190 | UVB\_NHEK3\_C6 |  | 30 | 0.57 | 1.52 | 0.011 | 0.047 | 1.000 | 3538 | tags=47%, list=17%, signal=56% |
| 191 | DNMT1\_KO\_DN |  | 16 | 0.63 | 1.52 | 0.023 | 0.047 | 1.000 | 3686 | tags=44%, list=18%, signal=53% |
| 192 | HIVNEFPATHWAY |  | 55 | 0.53 | 1.52 | 0.004 | 0.046 | 1.000 | 8111 | tags=76%, list=39%, signal=126% |
| 193 | HDACI\_COLON\_TSA12HRS\_UP |  | 22 | 0.59 | 1.52 | 0.013 | 0.046 | 1.000 | 3910 | tags=41%, list=19%, signal=50% |
| 194 | HDACI\_COLON\_BUT\_DN |  | 248 | 0.49 | 1.52 | 0.000 | 0.047 | 1.000 | 5161 | tags=48%, list=25%, signal=63% |
| 195 | UV\_ESR\_OLD\_UNREG |  | 19 | 0.60 | 1.52 | 0.010 | 0.047 | 1.000 | 5709 | tags=58%, list=28%, signal=80% |
| 196 | BRENTANI\_REPAIR |  | 35 | 0.56 | 1.52 | 0.006 | 0.046 | 1.000 | 5375 | tags=54%, list=26%, signal=73% |
| 197 | OXSTRESS\_RPETWO\_DN |  | 108 | 0.50 | 1.51 | 0.000 | 0.046 | 1.000 | 5198 | tags=45%, list=25%, signal=60% |
| 198 | FERNANDEZ\_MYC\_TARGETS |  | 178 | 0.49 | 1.51 | 0.000 | 0.046 | 1.000 | 6119 | tags=48%, list=30%, signal=68% |
| 199 | BRG1\_ALAB\_UP |  | 40 | 0.55 | 1.51 | 0.002 | 0.047 | 1.000 | 4079 | tags=45%, list=20%, signal=56% |
| 200 | INSULIN\_ADIP\_INSENS\_DN |  | 17 | 0.62 | 1.51 | 0.032 | 0.047 | 1.000 | 1452 | tags=35%, list=7%, signal=38% |
| 201 | AGED\_MOUSE\_CEREBELLUM\_UP |  | 60 | 0.52 | 1.51 | 0.003 | 0.048 | 1.000 | 3789 | tags=33%, list=18%, signal=41% |
| 202 | FALT\_BCLL\_DN |  | 49 | 0.53 | 1.51 | 0.004 | 0.049 | 1.000 | 7352 | tags=67%, list=36%, signal=104% |
| 203 | MOREAUX\_TACI\_HI\_IN\_PPC\_UP |  | 73 | 0.51 | 1.51 | 0.002 | 0.050 | 1.000 | 4085 | tags=45%, list=20%, signal=56% |
| 204 | LIAN\_MYELOID\_DIFF\_TF |  | 34 | 0.57 | 1.51 | 0.011 | 0.050 | 1.000 | 3134 | tags=38%, list=15%, signal=45% |
| 205 | PENG\_GLUCOSE\_DN |  | 134 | 0.50 | 1.51 | 0.000 | 0.050 | 1.000 | 5882 | tags=51%, list=29%, signal=72% |
| 206 | TSA\_HEPATOMA\_UP |  | 36 | 0.55 | 1.51 | 0.005 | 0.050 | 1.000 | 3777 | tags=50%, list=18%, signal=61% |
| 207 | STRESS\_GENOTOXIC\_SPECIFIC\_DN |  | 40 | 0.55 | 1.51 | 0.005 | 0.050 | 1.000 | 3454 | tags=33%, list=17%, signal=39% |
| 208 | ELONGINA\_KO\_UP |  | 162 | 0.50 | 1.51 | 0.000 | 0.049 | 1.000 | 4891 | tags=41%, list=24%, signal=54% |
| 209 | BRENTANI\_CELL\_CYCLE |  | 81 | 0.51 | 1.50 | 0.002 | 0.050 | 1.000 | 5237 | tags=41%, list=25%, signal=54% |
| 210 | CARIES\_PULP\_HIGH\_UP |  | 91 | 0.50 | 1.50 | 0.001 | 0.050 | 1.000 | 2597 | tags=22%, list=13%, signal=25% |
| 211 | MAGRANGEAS\_MULTIPLE\_MYELOMA\_IGL\_VS\_IGK\_DN |  | 27 | 0.57 | 1.50 | 0.016 | 0.050 | 1.000 | 2513 | tags=33%, list=12%, signal=38% |
| 212 | CMV\_HCMV\_TIMECOURSE\_18HRS\_DN |  | 21 | 0.60 | 1.50 | 0.014 | 0.051 | 1.000 | 1838 | tags=29%, list=9%, signal=31% |
| 213 | CELL\_CYCLE\_CHECKPOINT |  | 24 | 0.58 | 1.50 | 0.019 | 0.051 | 1.000 | 5375 | tags=63%, list=26%, signal=84% |
| 214 | EGF\_HDMEC\_UP |  | 42 | 0.54 | 1.50 | 0.007 | 0.051 | 1.000 | 1773 | tags=33%, list=9%, signal=36% |
| 215 | PLATELET\_EXPRESSED |  | 33 | 0.55 | 1.50 | 0.022 | 0.051 | 1.000 | 4587 | tags=48%, list=22%, signal=62% |
| 216 | ZHAN\_MM\_CD1\_VS\_CD2\_DN |  | 54 | 0.53 | 1.50 | 0.004 | 0.053 | 1.000 | 5360 | tags=44%, list=26%, signal=60% |
| 217 | PASSERINI\_APOPTOSIS |  | 43 | 0.54 | 1.50 | 0.006 | 0.053 | 1.000 | 2719 | tags=30%, list=13%, signal=35% |
| 218 | DIAB\_NEPH\_DN |  | 374 | 0.48 | 1.50 | 0.000 | 0.053 | 1.000 | 4779 | tags=39%, list=23%, signal=50% |
| 219 | FRASOR\_ER\_DN |  | 61 | 0.52 | 1.50 | 0.004 | 0.053 | 1.000 | 3907 | tags=44%, list=19%, signal=54% |
| 220 | TSADAC\_RKOEXP\_UP |  | 16 | 0.62 | 1.50 | 0.022 | 0.053 | 1.000 | 3613 | tags=44%, list=18%, signal=53% |
| 221 | CIRCADIAN\_EXERCISE |  | 41 | 0.54 | 1.49 | 0.007 | 0.054 | 1.000 | 5657 | tags=56%, list=27%, signal=77% |
| 222 | MOOTHA\_VOXPHOS |  | 77 | 0.51 | 1.49 | 0.002 | 0.054 | 1.000 | 5547 | tags=51%, list=27%, signal=69% |
| 223 | RORIE\_ES\_PNET\_UP |  | 27 | 0.56 | 1.49 | 0.017 | 0.055 | 1.000 | 2005 | tags=26%, list=10%, signal=29% |
| 224 | LEE\_MYC\_TGFA\_UP |  | 60 | 0.52 | 1.49 | 0.005 | 0.055 | 1.000 | 2167 | tags=23%, list=11%, signal=26% |
| 225 | HUMAN\_CD34\_ENRICHED\_TRANSCRIPTION\_FACTORS |  | 181 | 0.48 | 1.49 | 0.000 | 0.057 | 1.000 | 6268 | tags=50%, list=30%, signal=72% |
| 226 | XU\_CBP\_UP |  | 21 | 0.58 | 1.49 | 0.021 | 0.057 | 1.000 | 5389 | tags=62%, list=26%, signal=84% |
| 227 | NING\_COPD\_DN |  | 119 | 0.49 | 1.49 | 0.000 | 0.057 | 1.000 | 5273 | tags=46%, list=26%, signal=62% |
| 228 | IRS\_KO\_ADIP\_DN |  | 39 | 0.54 | 1.48 | 0.011 | 0.059 | 1.000 | 4385 | tags=33%, list=21%, signal=42% |
| 229 | WALLACE\_JAK2\_DIFF |  | 30 | 0.55 | 1.48 | 0.012 | 0.059 | 1.000 | 6322 | tags=53%, list=31%, signal=77% |
| 230 | ALZHEIMERS\_INCIPIENT\_UP |  | 342 | 0.47 | 1.48 | 0.000 | 0.059 | 1.000 | 5661 | tags=44%, list=27%, signal=59% |
| 231 | OXSTRESS\_RPE\_HNETBH\_DN |  | 47 | 0.52 | 1.48 | 0.008 | 0.059 | 1.000 | 5034 | tags=45%, list=24%, signal=59% |
| 232 | UVB\_NHEK1\_C2 |  | 21 | 0.59 | 1.48 | 0.024 | 0.059 | 1.000 | 1372 | tags=33%, list=7%, signal=36% |
| 233 | ALCALAY\_AML\_NPMC\_DN |  | 187 | 0.48 | 1.48 | 0.000 | 0.059 | 1.000 | 5084 | tags=40%, list=25%, signal=53% |
| 234 | CMV\_HCMV\_TIMECOURSE\_8HRS\_UP |  | 20 | 0.59 | 1.48 | 0.021 | 0.059 | 1.000 | 3242 | tags=35%, list=16%, signal=41% |
| 235 | HUMAN\_MITODB\_6\_2002 |  | 382 | 0.47 | 1.48 | 0.000 | 0.059 | 1.000 | 6199 | tags=50%, list=30%, signal=71% |
| 236 | OLDWERNER\_FIBRO\_DN |  | 105 | 0.49 | 1.48 | 0.000 | 0.059 | 1.000 | 6234 | tags=50%, list=30%, signal=72% |
| 237 | MARTINELLI\_IFNS\_DIFF |  | 21 | 0.60 | 1.48 | 0.014 | 0.059 | 1.000 | 293 | tags=19%, list=1%, signal=19% |
| 238 | BREASTCA\_THREE\_CLASSES |  | 42 | 0.53 | 1.48 | 0.015 | 0.059 | 1.000 | 3894 | tags=48%, list=19%, signal=59% |
| 239 | PASSERINI\_ADHESION |  | 37 | 0.54 | 1.48 | 0.012 | 0.059 | 1.000 | 4079 | tags=38%, list=20%, signal=47% |
| 240 | HG\_PROGERIA\_DN |  | 25 | 0.57 | 1.48 | 0.021 | 0.059 | 1.000 | 4997 | tags=48%, list=24%, signal=63% |
| 241 | SERUM\_FIBROBLAST\_CORE\_DN |  | 196 | 0.48 | 1.48 | 0.000 | 0.059 | 1.000 | 4577 | tags=42%, list=22%, signal=53% |
| 242 | DNA\_DAMAGE\_SIGNALING |  | 88 | 0.50 | 1.48 | 0.003 | 0.059 | 1.000 | 6545 | tags=51%, list=32%, signal=75% |
| 243 | CHANG\_SERUM\_RESPONSE\_UP |  | 148 | 0.49 | 1.48 | 0.000 | 0.059 | 1.000 | 5369 | tags=44%, list=26%, signal=59% |
| 244 | JAIN\_NEMO\_DIFF |  | 75 | 0.50 | 1.48 | 0.001 | 0.059 | 1.000 | 6422 | tags=64%, list=31%, signal=93% |
| 245 | HDACI\_COLON\_BUT\_UP |  | 180 | 0.48 | 1.48 | 0.000 | 0.059 | 1.000 | 6102 | tags=46%, list=30%, signal=65% |
| 246 | ET743\_SARCOMA\_72HRS\_UP |  | 66 | 0.51 | 1.48 | 0.001 | 0.059 | 1.000 | 3781 | tags=39%, list=18%, signal=48% |
| 247 | ROS\_MOUSE\_AORTA\_UP |  | 24 | 0.57 | 1.48 | 0.023 | 0.059 | 1.000 | 6014 | tags=58%, list=29%, signal=82% |
| 248 | GH\_GHRHR\_KO\_6HRS\_DN |  | 34 | 0.54 | 1.48 | 0.013 | 0.059 | 1.000 | 5855 | tags=56%, list=28%, signal=78% |
| 249 | HOUSTIS\_ROS |  | 30 | 0.55 | 1.48 | 0.020 | 0.059 | 1.000 | 4353 | tags=47%, list=21%, signal=59% |
| 250 | VEGF\_MMMEC\_3HRS\_UP |  | 66 | 0.50 | 1.48 | 0.005 | 0.059 | 1.000 | 4540 | tags=41%, list=22%, signal=52% |
| 251 | PENTOSE\_PHOSPHATE\_PATHWAY |  | 23 | 0.57 | 1.47 | 0.030 | 0.060 | 1.000 | 2637 | tags=30%, list=13%, signal=35% |
| 252 | HDACI\_COLON\_BUT24HRS\_UP |  | 70 | 0.51 | 1.47 | 0.004 | 0.060 | 1.000 | 4540 | tags=41%, list=22%, signal=53% |
| 253 | HEARTFAILURE\_VENTRICLE\_DN |  | 67 | 0.51 | 1.47 | 0.003 | 0.061 | 1.000 | 4680 | tags=42%, list=23%, signal=54% |
| 254 | PASSERINI\_EM |  | 35 | 0.54 | 1.47 | 0.012 | 0.061 | 1.000 | 1349 | tags=14%, list=7%, signal=15% |
| 255 | SHEPARD\_BMYB\_MORPHOLINO\_UP |  | 155 | 0.48 | 1.47 | 0.000 | 0.062 | 1.000 | 4466 | tags=38%, list=22%, signal=48% |
| 256 | BRENTANI\_TRANSCRIPTION\_FACTORS |  | 64 | 0.51 | 1.47 | 0.004 | 0.061 | 1.000 | 3340 | tags=28%, list=16%, signal=33% |
| 257 | AS3\_FIBRO\_C4 |  | 18 | 0.60 | 1.47 | 0.032 | 0.061 | 1.000 | 2735 | tags=39%, list=13%, signal=45% |
| 258 | H2O2\_CSBRESCUED\_UP |  | 56 | 0.51 | 1.47 | 0.008 | 0.063 | 1.000 | 3947 | tags=41%, list=19%, signal=51% |
| 259 | IDX\_TSA\_UP\_CLUSTER6 |  | 163 | 0.48 | 1.47 | 0.000 | 0.062 | 1.000 | 4923 | tags=44%, list=24%, signal=58% |
| 260 | ET743\_SARCOMA\_6HRS\_UP |  | 30 | 0.55 | 1.47 | 0.016 | 0.063 | 1.000 | 4126 | tags=43%, list=20%, signal=54% |
| 261 | CALRES\_MOUSE\_UP |  | 29 | 0.56 | 1.47 | 0.010 | 0.063 | 1.000 | 1732 | tags=28%, list=8%, signal=30% |
| 262 | ICHIBA\_GVHD |  | 238 | 0.47 | 1.47 | 0.000 | 0.063 | 1.000 | 3232 | tags=26%, list=16%, signal=31% |
| 263 | KNUDSEN\_PMNS\_DN |  | 226 | 0.47 | 1.47 | 0.000 | 0.063 | 1.000 | 6277 | tags=54%, list=30%, signal=77% |
| 264 | FALT\_BCLL\_UP |  | 45 | 0.53 | 1.47 | 0.009 | 0.063 | 1.000 | 6311 | tags=60%, list=31%, signal=86% |
| 265 | OLDAGE\_DN |  | 47 | 0.52 | 1.47 | 0.005 | 0.063 | 1.000 | 5764 | tags=47%, list=28%, signal=65% |
| 266 | FSH\_OVARY\_MCV152\_UP |  | 61 | 0.51 | 1.46 | 0.003 | 0.065 | 1.000 | 2508 | tags=31%, list=12%, signal=35% |
| 267 | PORPHYRIN\_AND\_CHLOROPHYLL\_METABOLISM |  | 20 | 0.58 | 1.46 | 0.025 | 0.066 | 1.000 | 3469 | tags=35%, list=17%, signal=42% |
| 268 | HDACI\_COLON\_CLUSTER4 |  | 16 | 0.61 | 1.46 | 0.042 | 0.066 | 1.000 | 4502 | tags=56%, list=22%, signal=72% |
| 269 | CMV\_HCMV\_TIMECOURSE\_24HRS\_DN |  | 42 | 0.53 | 1.46 | 0.015 | 0.067 | 1.000 | 4435 | tags=40%, list=22%, signal=51% |
| 270 | MATRIX\_METALLOPROTEINASES |  | 30 | 0.56 | 1.46 | 0.020 | 0.067 | 1.000 | 5663 | tags=50%, list=27%, signal=69% |
| 271 | CHESLER\_HIGHEST\_FOLD\_RANGE\_GENES |  | 37 | 0.53 | 1.46 | 0.026 | 0.067 | 1.000 | 5728 | tags=54%, list=28%, signal=75% |
| 272 | G13\_SIGNALING\_PATHWAY |  | 39 | 0.53 | 1.46 | 0.022 | 0.067 | 1.000 | 5578 | tags=54%, list=27%, signal=74% |
| 273 | TGFBETA\_C1\_UP |  | 17 | 0.59 | 1.46 | 0.039 | 0.067 | 1.000 | 5670 | tags=53%, list=28%, signal=73% |
| 274 | ERM\_KO\_TESTES\_DN |  | 21 | 0.57 | 1.46 | 0.029 | 0.068 | 1.000 | 2672 | tags=33%, list=13%, signal=38% |
| 275 | ST\_GA13\_PATHWAY |  | 35 | 0.52 | 1.46 | 0.024 | 0.068 | 1.000 | 6822 | tags=57%, list=33%, signal=85% |
| 276 | LEE\_E2F1\_UP |  | 60 | 0.50 | 1.46 | 0.003 | 0.068 | 1.000 | 2335 | tags=28%, list=11%, signal=32% |
| 277 | IL1\_CORNEA\_UP |  | 62 | 0.50 | 1.45 | 0.006 | 0.069 | 1.000 | 6688 | tags=47%, list=32%, signal=69% |
| 278 | BRENTANI\_IMMUNE\_FUNCTION |  | 50 | 0.52 | 1.45 | 0.008 | 0.068 | 1.000 | 5507 | tags=34%, list=27%, signal=46% |
| 279 | CROONQUIST\_IL6\_STARVE\_UP |  | 33 | 0.54 | 1.45 | 0.024 | 0.068 | 1.000 | 5874 | tags=52%, list=29%, signal=72% |
| 280 | UVB\_NHEK4\_6HRS\_UP |  | 28 | 0.54 | 1.45 | 0.031 | 0.068 | 1.000 | 4301 | tags=46%, list=21%, signal=59% |
| 281 | ZELLER\_MYC\_UP |  | 23 | 0.56 | 1.45 | 0.028 | 0.068 | 1.000 | 2828 | tags=35%, list=14%, signal=40% |
| 282 | MYOD\_NIH3T3\_DN |  | 56 | 0.51 | 1.45 | 0.008 | 0.068 | 1.000 | 1183 | tags=21%, list=6%, signal=23% |
| 283 | REOVIRUS\_HEK293\_UP |  | 236 | 0.47 | 1.45 | 0.000 | 0.068 | 1.000 | 5874 | tags=46%, list=29%, signal=63% |
| 284 | HDACI\_COLON\_TSA2HRS\_UP |  | 60 | 0.50 | 1.45 | 0.007 | 0.069 | 1.000 | 4954 | tags=43%, list=24%, signal=57% |
| 285 | LIZUKA\_L0\_SM\_L1 |  | 19 | 0.58 | 1.45 | 0.035 | 0.069 | 1.000 | 5423 | tags=58%, list=26%, signal=79% |
| 286 | GAMMA\_ESR\_WS\_UNREG |  | 28 | 0.54 | 1.45 | 0.028 | 0.069 | 1.000 | 3361 | tags=43%, list=16%, signal=51% |
| 287 | AGUIRRE\_PANCREAS\_CHR19 |  | 74 | 0.49 | 1.45 | 0.007 | 0.069 | 1.000 | 6311 | tags=53%, list=31%, signal=76% |
| 288 | IDX\_TSA\_DN\_CLUSTER1 |  | 42 | 0.51 | 1.45 | 0.016 | 0.069 | 1.000 | 1503 | tags=31%, list=7%, signal=33% |
| 289 | WERNER\_FIBRO\_DN |  | 168 | 0.47 | 1.45 | 0.000 | 0.070 | 1.000 | 6234 | tags=47%, list=30%, signal=67% |
| 290 | OKUMURA\_MC\_LPS |  | 185 | 0.47 | 1.45 | 0.000 | 0.071 | 1.000 | 4228 | tags=35%, list=21%, signal=43% |
| 291 | TIDPATHWAY |  | 18 | 0.58 | 1.45 | 0.030 | 0.071 | 1.000 | 1758 | tags=28%, list=9%, signal=30% |
| 292 | YAGI\_AML\_PROGNOSIS |  | 34 | 0.53 | 1.45 | 0.022 | 0.071 | 1.000 | 6606 | tags=59%, list=32%, signal=86% |
| 293 | LI\_FETAL\_VS\_WT\_KIDNEY\_UP |  | 179 | 0.47 | 1.45 | 0.000 | 0.071 | 1.000 | 4275 | tags=37%, list=21%, signal=47% |
| 294 | PENG\_GLUTAMINE\_UP |  | 227 | 0.47 | 1.45 | 0.000 | 0.071 | 1.000 | 4780 | tags=44%, list=23%, signal=57% |
| 295 | UVC\_HIGH\_D7\_DN |  | 32 | 0.54 | 1.45 | 0.021 | 0.072 | 1.000 | 4440 | tags=47%, list=22%, signal=60% |
| 296 | ADIP\_VS\_PREADIP\_DN |  | 37 | 0.52 | 1.44 | 0.024 | 0.072 | 1.000 | 3717 | tags=38%, list=18%, signal=46% |
| 297 | UVB\_NHEK2\_DN |  | 81 | 0.49 | 1.44 | 0.005 | 0.072 | 1.000 | 4591 | tags=46%, list=22%, signal=59% |
| 298 | CELL\_CYCLE\_ARREST |  | 31 | 0.54 | 1.44 | 0.020 | 0.072 | 1.000 | 5628 | tags=48%, list=27%, signal=66% |
| 299 | HOFFMANN\_BIVSBII\_IMVM |  | 81 | 0.49 | 1.44 | 0.004 | 0.072 | 1.000 | 5457 | tags=44%, list=26%, signal=60% |
| 300 | ADIP\_VS\_FIBRO\_DN |  | 27 | 0.55 | 1.44 | 0.029 | 0.072 | 1.000 | 4911 | tags=44%, list=24%, signal=58% |
| 301 | BYSTROM\_IL5\_DN |  | 57 | 0.50 | 1.44 | 0.004 | 0.072 | 1.000 | 7049 | tags=63%, list=34%, signal=96% |
| 302 | BROCKE\_IL6 |  | 145 | 0.47 | 1.44 | 0.000 | 0.073 | 1.000 | 5136 | tags=42%, list=25%, signal=56% |
| 303 | ZHAN\_MM\_CD1\_VS\_CD2\_UP |  | 91 | 0.48 | 1.44 | 0.004 | 0.073 | 1.000 | 3025 | tags=34%, list=15%, signal=40% |
| 304 | GOLUB\_ALL\_VS\_AML\_UP |  | 19 | 0.58 | 1.44 | 0.026 | 0.073 | 1.000 | 5986 | tags=53%, list=29%, signal=74% |
| 305 | TSA\_HEPATOMA\_CANCER\_UP |  | 39 | 0.53 | 1.44 | 0.019 | 0.073 | 1.000 | 4321 | tags=36%, list=21%, signal=45% |
| 306 | TPA\_RESIST\_MIDDLE\_UP |  | 49 | 0.51 | 1.44 | 0.015 | 0.074 | 1.000 | 6028 | tags=47%, list=29%, signal=66% |
| 307 | AGUIRRE\_PANCREAS\_CHR12 |  | 59 | 0.50 | 1.44 | 0.008 | 0.074 | 1.000 | 5343 | tags=51%, list=26%, signal=68% |
| 308 | SANSOM\_APC\_LOSS4\_UP |  | 115 | 0.48 | 1.44 | 0.000 | 0.074 | 1.000 | 4899 | tags=37%, list=24%, signal=49% |
| 309 | BLEO\_MOUSE\_LYMPH\_HIGH\_24HRS\_DN |  | 34 | 0.53 | 1.44 | 0.018 | 0.074 | 1.000 | 7720 | tags=74%, list=37%, signal=117% |
| 310 | VEGF\_MMMEC\_12HRS\_UP |  | 28 | 0.54 | 1.44 | 0.025 | 0.074 | 1.000 | 339 | tags=21%, list=2%, signal=22% |
| 311 | TGFBETA\_C2\_UP |  | 18 | 0.59 | 1.44 | 0.035 | 0.074 | 1.000 | 3717 | tags=44%, list=18%, signal=54% |
| 312 | CHANG\_SERUM\_RESPONSE\_DN |  | 124 | 0.47 | 1.44 | 0.001 | 0.074 | 1.000 | 4577 | tags=41%, list=22%, signal=53% |
| 313 | KRETZSCHMAR\_IL6\_DIFF |  | 145 | 0.47 | 1.44 | 0.000 | 0.074 | 1.000 | 5136 | tags=42%, list=25%, signal=56% |
| 314 | HDACI\_COLON\_BUT16HRS\_DN |  | 108 | 0.48 | 1.44 | 0.001 | 0.074 | 1.000 | 5111 | tags=48%, list=25%, signal=64% |
| 315 | AGEING\_KIDNEY\_SPECIFIC\_DN |  | 132 | 0.47 | 1.44 | 0.002 | 0.075 | 1.000 | 6112 | tags=47%, list=30%, signal=66% |
| 316 | HDACI\_COLON\_BUT24HRS\_DN |  | 109 | 0.48 | 1.43 | 0.001 | 0.076 | 1.000 | 5161 | tags=49%, list=25%, signal=65% |
| 317 | OLD\_FIBRO\_UP |  | 61 | 0.50 | 1.43 | 0.007 | 0.076 | 1.000 | 5019 | tags=43%, list=24%, signal=56% |
| 318 | VEGF\_MMMEC\_ALL\_UP |  | 94 | 0.48 | 1.43 | 0.002 | 0.076 | 1.000 | 3686 | tags=31%, list=18%, signal=37% |
| 319 | PROTEASOME |  | 17 | 0.59 | 1.43 | 0.040 | 0.076 | 1.000 | 6760 | tags=76%, list=33%, signal=114% |
| 320 | KREBS\_TCA\_CYCLE |  | 30 | 0.53 | 1.43 | 0.030 | 0.076 | 1.000 | 6119 | tags=50%, list=30%, signal=71% |
| 321 | ADIP\_HUMAN\_DN |  | 27 | 0.54 | 1.43 | 0.031 | 0.076 | 1.000 | 2389 | tags=33%, list=12%, signal=38% |
| 322 | HDACI\_COLON\_TSABUT\_DN |  | 22 | 0.57 | 1.43 | 0.042 | 0.076 | 1.000 | 3721 | tags=45%, list=18%, signal=55% |
| 323 | GALE\_FLT3ANDAPL\_UP |  | 59 | 0.50 | 1.43 | 0.016 | 0.077 | 1.000 | 5281 | tags=44%, list=26%, signal=59% |
| 324 | WANG\_MLL\_CBP\_VS\_GMP\_DN |  | 40 | 0.51 | 1.43 | 0.025 | 0.077 | 1.000 | 5046 | tags=45%, list=24%, signal=59% |
| 325 | CELL\_ADHESION\_RECEPTOR\_ACTIVITY |  | 33 | 0.53 | 1.43 | 0.027 | 0.077 | 1.000 | 5524 | tags=48%, list=27%, signal=66% |
| 326 | PARK\_MSCS\_LIN2 |  | 40 | 0.51 | 1.43 | 0.022 | 0.077 | 1.000 | 7342 | tags=63%, list=36%, signal=97% |
| 327 | XPB\_TTD-CS\_DN |  | 24 | 0.56 | 1.43 | 0.031 | 0.078 | 1.000 | 3789 | tags=38%, list=18%, signal=46% |
| 328 | MTORPATHWAY |  | 23 | 0.56 | 1.43 | 0.037 | 0.078 | 1.000 | 6236 | tags=48%, list=30%, signal=69% |
| 329 | ADIP\_DIFF\_CLUSTER1 |  | 56 | 0.49 | 1.43 | 0.014 | 0.079 | 1.000 | 3849 | tags=39%, list=19%, signal=48% |
| 330 | SMITH\_HTERT\_DN |  | 61 | 0.49 | 1.43 | 0.010 | 0.079 | 1.000 | 4440 | tags=41%, list=22%, signal=52% |
| 331 | HDACI\_COLON\_TSABUT\_UP |  | 72 | 0.48 | 1.43 | 0.011 | 0.079 | 1.000 | 5844 | tags=44%, list=28%, signal=62% |
| 332 | GNATENKO\_PLATELET\_UP |  | 44 | 0.51 | 1.43 | 0.020 | 0.079 | 1.000 | 2392 | tags=34%, list=12%, signal=38% |
| 333 | SERUM\_FIBROBLAST\_CORE\_UP |  | 202 | 0.46 | 1.43 | 0.000 | 0.079 | 1.000 | 5446 | tags=43%, list=26%, signal=58% |
| 334 | OLD\_FIBRO\_DN |  | 158 | 0.47 | 1.42 | 0.001 | 0.080 | 1.000 | 6423 | tags=48%, list=31%, signal=69% |
| 335 | UVB\_NHEK2\_UP |  | 68 | 0.49 | 1.42 | 0.008 | 0.082 | 1.000 | 7382 | tags=65%, list=36%, signal=100% |
| 336 | LOTEM\_LEUKEMIA\_UP |  | 22 | 0.56 | 1.42 | 0.040 | 0.082 | 1.000 | 6301 | tags=55%, list=31%, signal=78% |
| 337 | SHEPARD\_GENES\_COMMON\_BW\_CB\_MO |  | 68 | 0.49 | 1.42 | 0.009 | 0.081 | 1.000 | 5649 | tags=40%, list=27%, signal=55% |
| 338 | BYSTRYKH\_HSC\_BRAIN\_CIS\_GLOCUS |  | 51 | 0.50 | 1.42 | 0.017 | 0.082 | 1.000 | 6161 | tags=49%, list=30%, signal=70% |
| 339 | HDACI\_COLON\_TSA\_UP |  | 120 | 0.47 | 1.42 | 0.000 | 0.082 | 1.000 | 5099 | tags=38%, list=25%, signal=51% |
| 340 | WONG\_IFNA\_HCC\_RESISTANT\_VS\_SENSITIVE\_UP |  | 15 | 0.60 | 1.42 | 0.039 | 0.082 | 1.000 | 6556 | tags=73%, list=32%, signal=107% |
| 341 | N\_GLYCAN\_BIOSYNTHESIS |  | 22 | 0.55 | 1.42 | 0.041 | 0.083 | 1.000 | 3496 | tags=41%, list=17%, signal=49% |
| 342 | IDX\_TSA\_DN\_CLUSTER6 |  | 27 | 0.54 | 1.42 | 0.034 | 0.083 | 1.000 | 7131 | tags=67%, list=35%, signal=102% |
| 343 | ET743\_SARCOMA\_48HRS\_DN |  | 188 | 0.46 | 1.42 | 0.000 | 0.083 | 1.000 | 5579 | tags=44%, list=27%, signal=60% |
| 344 | ARFPATHWAY |  | 16 | 0.59 | 1.42 | 0.050 | 0.083 | 1.000 | 1285 | tags=25%, list=6%, signal=27% |
| 345 | ET743\_SARCOMA\_DN |  | 269 | 0.46 | 1.42 | 0.000 | 0.083 | 1.000 | 5603 | tags=44%, list=27%, signal=59% |
| 346 | HSC\_EARLYPROGENITORS\_SHARED |  | 448 | 0.45 | 1.42 | 0.000 | 0.084 | 1.000 | 6670 | tags=49%, list=32%, signal=70% |
| 347 | DFOSB\_BRAIN\_8WKS\_UP |  | 40 | 0.51 | 1.42 | 0.023 | 0.085 | 1.000 | 5529 | tags=45%, list=27%, signal=61% |
| 348 | PASSERINI\_OXIDATION |  | 19 | 0.57 | 1.41 | 0.052 | 0.085 | 1.000 | 3131 | tags=42%, list=15%, signal=50% |
| 349 | UVC\_HIGH\_D6\_DN |  | 31 | 0.52 | 1.41 | 0.032 | 0.086 | 1.000 | 2068 | tags=32%, list=10%, signal=36% |
| 350 | HSC\_EARLYPROGENITORS\_ADULT |  | 450 | 0.45 | 1.41 | 0.000 | 0.088 | 1.000 | 6670 | tags=48%, list=32%, signal=70% |
| 351 | VALINE\_LEUCINE\_AND\_ISOLEUCINE\_DEGRADATION |  | 36 | 0.51 | 1.41 | 0.027 | 0.088 | 1.000 | 2479 | tags=36%, list=12%, signal=41% |
| 352 | NKCELLSPATHWAY |  | 18 | 0.57 | 1.41 | 0.043 | 0.089 | 1.000 | 3015 | tags=33%, list=15%, signal=39% |
| 353 | GNATENKO\_PLATELET |  | 44 | 0.51 | 1.41 | 0.021 | 0.089 | 1.000 | 2392 | tags=34%, list=12%, signal=38% |
| 354 | CELL\_CYCLE\_KEGG |  | 86 | 0.48 | 1.41 | 0.004 | 0.089 | 1.000 | 5852 | tags=48%, list=28%, signal=66% |
| 355 | GALINDO\_ACT\_UP |  | 76 | 0.48 | 1.41 | 0.012 | 0.089 | 1.000 | 3783 | tags=30%, list=18%, signal=37% |
| 356 | GLUTATHIONE\_METABOLISM |  | 31 | 0.52 | 1.41 | 0.042 | 0.089 | 1.000 | 6466 | tags=52%, list=31%, signal=75% |
| 357 | MAPKPATHWAY |  | 84 | 0.48 | 1.41 | 0.004 | 0.089 | 1.000 | 7202 | tags=60%, list=35%, signal=91% |
| 358 | BRCA\_PROGNOSIS\_POS |  | 41 | 0.51 | 1.41 | 0.020 | 0.089 | 1.000 | 8741 | tags=76%, list=42%, signal=131% |
| 359 | HSC\_EARLYPROGENITORS\_FETAL |  | 448 | 0.45 | 1.41 | 0.000 | 0.089 | 1.000 | 6670 | tags=49%, list=32%, signal=70% |
| 360 | ASTON\_DEPRESSION\_UP |  | 45 | 0.50 | 1.41 | 0.025 | 0.089 | 1.000 | 4855 | tags=38%, list=24%, signal=49% |
| 361 | TGFBETA\_LATE\_UP |  | 33 | 0.53 | 1.41 | 0.028 | 0.090 | 1.000 | 5781 | tags=52%, list=28%, signal=71% |
| 362 | TPA\_SENS\_MIDDLE\_UP |  | 65 | 0.48 | 1.40 | 0.014 | 0.092 | 1.000 | 4497 | tags=37%, list=22%, signal=47% |
| 363 | NADLER\_OBESITY\_UP |  | 52 | 0.49 | 1.40 | 0.018 | 0.092 | 1.000 | 4710 | tags=42%, list=23%, signal=55% |
| 364 | KNUDSEN\_PMNS\_UP |  | 74 | 0.48 | 1.40 | 0.010 | 0.091 | 1.000 | 4072 | tags=35%, list=20%, signal=44% |
| 365 | AGEING\_KIDNEY\_UP |  | 406 | 0.45 | 1.40 | 0.000 | 0.091 | 1.000 | 4341 | tags=32%, list=21%, signal=39% |
| 366 | HSC\_INTERMEDIATEPROGENITORS\_SHARED |  | 133 | 0.46 | 1.40 | 0.002 | 0.092 | 1.000 | 6074 | tags=44%, list=29%, signal=62% |
| 367 | H2O2\_CSBDIFF\_C2 |  | 33 | 0.52 | 1.40 | 0.038 | 0.093 | 1.000 | 3941 | tags=45%, list=19%, signal=56% |
| 368 | PASSERINI\_INFLAMMATION |  | 25 | 0.53 | 1.40 | 0.042 | 0.093 | 1.000 | 27 | tags=8%, list=0%, signal=8% |
| 369 | PASSERINI\_TRANSCRIPTION |  | 74 | 0.48 | 1.40 | 0.010 | 0.092 | 1.000 | 5005 | tags=42%, list=24%, signal=55% |
| 370 | KUROKAWA\_5FU\_IFN\_SENSITIVE\_VS\_RESISTANT\_DN |  | 34 | 0.52 | 1.40 | 0.027 | 0.092 | 1.000 | 5603 | tags=50%, list=27%, signal=69% |
| 371 | HYPOXIA\_NORMAL\_UP |  | 216 | 0.45 | 1.40 | 0.000 | 0.093 | 1.000 | 4666 | tags=40%, list=23%, signal=52% |
| 372 | BYSTRYKH\_HSC\_BRAIN\_TRANS\_GLOCUS |  | 157 | 0.46 | 1.40 | 0.000 | 0.093 | 1.000 | 4026 | tags=31%, list=20%, signal=38% |
| 373 | HEARTFAILURE\_ATRIA\_DN |  | 111 | 0.47 | 1.40 | 0.003 | 0.094 | 1.000 | 7016 | tags=56%, list=34%, signal=84% |
| 374 | HEARTFAILURE\_ATRIA\_UP |  | 25 | 0.54 | 1.40 | 0.057 | 0.094 | 1.000 | 6311 | tags=56%, list=31%, signal=81% |
| 375 | HCC\_SURVIVAL\_GOOD\_VS\_POOR\_DN |  | 129 | 0.46 | 1.40 | 0.001 | 0.094 | 1.000 | 4860 | tags=40%, list=24%, signal=52% |
| 376 | GENOTOXINS\_ALL\_24HRS\_REG |  | 28 | 0.53 | 1.40 | 0.045 | 0.094 | 1.000 | 3956 | tags=46%, list=19%, signal=57% |
| 377 | ROME\_INSULIN\_2F\_UP |  | 185 | 0.46 | 1.40 | 0.000 | 0.095 | 1.000 | 6082 | tags=48%, list=30%, signal=68% |
| 378 | AS3\_FIBRO\_C2 |  | 31 | 0.52 | 1.40 | 0.048 | 0.095 | 1.000 | 3743 | tags=39%, list=18%, signal=47% |
| 379 | HDACI\_COLON\_BUT48HRS\_DN |  | 116 | 0.46 | 1.40 | 0.004 | 0.095 | 1.000 | 5161 | tags=47%, list=25%, signal=63% |
| 380 | AGUIRRE\_PANCREAS\_CHR17 |  | 70 | 0.48 | 1.39 | 0.020 | 0.096 | 1.000 | 6528 | tags=47%, list=32%, signal=69% |
| 381 | HSC\_INTERMEDIATEPROGENITORS\_ADULT |  | 144 | 0.46 | 1.39 | 0.001 | 0.096 | 1.000 | 6074 | tags=44%, list=29%, signal=62% |
| 382 | FLECHNER\_KIDNEY\_TRANSPLANT\_REJECTION\_PBL\_UP |  | 64 | 0.48 | 1.39 | 0.013 | 0.096 | 1.000 | 6925 | tags=52%, list=34%, signal=77% |
| 383 | BRG1\_H1299\_UP |  | 36 | 0.51 | 1.39 | 0.041 | 0.096 | 1.000 | 8509 | tags=78%, list=41%, signal=132% |
| 384 | BRCA1KO\_MEF\_DN |  | 79 | 0.47 | 1.39 | 0.008 | 0.097 | 1.000 | 7034 | tags=62%, list=34%, signal=94% |
| 385 | BRENTANI\_CELL\_ADHESION |  | 92 | 0.46 | 1.39 | 0.009 | 0.097 | 1.000 | 4032 | tags=34%, list=20%, signal=42% |
| 386 | CELL\_CYCLE |  | 78 | 0.47 | 1.39 | 0.008 | 0.097 | 1.000 | 4323 | tags=41%, list=21%, signal=52% |
| 387 | POMEROY\_MD\_TREATMENT\_GOOD\_VS\_POOR\_UP |  | 29 | 0.52 | 1.39 | 0.046 | 0.098 | 1.000 | 7130 | tags=55%, list=35%, signal=84% |
| 388 | HSC\_LATEPROGENITORS\_FETAL |  | 464 | 0.44 | 1.39 | 0.000 | 0.098 | 1.000 | 5520 | tags=40%, list=27%, signal=54% |
| 389 | UNDERHILL\_PROLIFERATION |  | 18 | 0.57 | 1.39 | 0.072 | 0.098 | 1.000 | 5375 | tags=56%, list=26%, signal=75% |
| 390 | OXSTRESS\_RPE\_H2O2TBH\_DN |  | 31 | 0.52 | 1.39 | 0.053 | 0.098 | 1.000 | 3743 | tags=39%, list=18%, signal=47% |
| 391 | HSC\_LATEPROGENITORS\_ADULT |  | 463 | 0.44 | 1.39 | 0.000 | 0.098 | 1.000 | 5520 | tags=40%, list=27%, signal=54% |
| 392 | ET743\_RESIST\_DN |  | 40 | 0.50 | 1.39 | 0.041 | 0.098 | 1.000 | 2875 | tags=35%, list=14%, signal=41% |
| 393 | ET743\_SARCOMA\_72HRS\_DN |  | 222 | 0.45 | 1.39 | 0.000 | 0.098 | 1.000 | 5603 | tags=44%, list=27%, signal=60% |
| 394 | GAMMA\_ESR\_OLD\_UNREG |  | 26 | 0.53 | 1.39 | 0.041 | 0.098 | 1.000 | 4660 | tags=46%, list=23%, signal=60% |
| 395 | FERRANDO\_MLL\_T\_ALL\_UP |  | 87 | 0.47 | 1.39 | 0.010 | 0.098 | 1.000 | 3789 | tags=32%, list=18%, signal=39% |
| 396 | HALMOS\_CEBP\_UP |  | 50 | 0.49 | 1.39 | 0.028 | 0.098 | 1.000 | 3088 | tags=30%, list=15%, signal=35% |
| 397 | YU\_CMYC\_UP |  | 30 | 0.52 | 1.39 | 0.048 | 0.099 | 1.000 | 5500 | tags=47%, list=27%, signal=64% |
| 398 | HSC\_LATEPROGENITORS\_SHARED |  | 456 | 0.44 | 1.39 | 0.000 | 0.099 | 1.000 | 5520 | tags=40%, list=27%, signal=54% |
| 399 | BRENTANI\_DEATH |  | 70 | 0.48 | 1.39 | 0.015 | 0.100 | 1.000 | 8111 | tags=64%, list=39%, signal=106% |
| 400 | UVB\_NHEK1\_DN |  | 270 | 0.44 | 1.39 | 0.000 | 0.101 | 1.000 | 6194 | tags=47%, list=30%, signal=66% |
| 401 | ATRIA\_UP |  | 196 | 0.45 | 1.39 | 0.000 | 0.101 | 1.000 | 5062 | tags=38%, list=25%, signal=50% |
| 402 | INTRINSICPATHWAY |  | 22 | 0.54 | 1.38 | 0.053 | 0.101 | 1.000 | 936 | tags=18%, list=5%, signal=19% |
| 403 | WERNERONLY\_FIBRO\_UP |  | 31 | 0.51 | 1.38 | 0.048 | 0.101 | 1.000 | 7197 | tags=55%, list=35%, signal=84% |
| 404 | RASPATHWAY |  | 22 | 0.55 | 1.38 | 0.057 | 0.102 | 1.000 | 7202 | tags=68%, list=35%, signal=105% |
| 405 | ADIPOGENESIS\_HMSC\_CLASS1\_UP |  | 18 | 0.56 | 1.38 | 0.062 | 0.102 | 1.000 | 3910 | tags=39%, list=19%, signal=48% |
| 406 | TAKEDA\_NUP8\_HOXA9\_8D\_UP |  | 151 | 0.45 | 1.38 | 0.001 | 0.102 | 1.000 | 3844 | tags=32%, list=19%, signal=39% |
| 407 | LEE\_DENA\_UP |  | 59 | 0.48 | 1.38 | 0.022 | 0.102 | 1.000 | 3007 | tags=34%, list=15%, signal=40% |
| 408 | CITED1\_KO\_HET\_UP |  | 27 | 0.53 | 1.38 | 0.047 | 0.102 | 1.000 | 5904 | tags=37%, list=29%, signal=52% |
| 409 | ROSS\_AML1\_ETO |  | 82 | 0.47 | 1.38 | 0.007 | 0.102 | 1.000 | 4251 | tags=38%, list=21%, signal=47% |
| 410 | AGED\_MOUSE\_HYPOTH\_DN |  | 38 | 0.50 | 1.38 | 0.040 | 0.103 | 1.000 | 5274 | tags=55%, list=26%, signal=74% |
| 411 | HSC\_INTERMEDIATEPROGENITORS\_FETAL |  | 158 | 0.45 | 1.38 | 0.003 | 0.103 | 1.000 | 6074 | tags=42%, list=29%, signal=60% |
| 412 | DEATHPATHWAY |  | 33 | 0.51 | 1.38 | 0.042 | 0.103 | 1.000 | 8111 | tags=70%, list=39%, signal=115% |
| 413 | AGED\_MOUSE\_MUSCLE\_UP |  | 31 | 0.51 | 1.38 | 0.045 | 0.103 | 1.000 | 3525 | tags=45%, list=17%, signal=54% |
| 414 | HOHENKIRK\_MONOCYTE\_DEND\_UP |  | 106 | 0.46 | 1.38 | 0.007 | 0.103 | 1.000 | 5656 | tags=42%, list=27%, signal=58% |
| 415 | DAC\_PANC50\_UP |  | 42 | 0.50 | 1.38 | 0.037 | 0.103 | 1.000 | 3782 | tags=31%, list=18%, signal=38% |
| 416 | FLOTHO\_CASP8AP2\_MRD\_DIFF |  | 84 | 0.47 | 1.38 | 0.004 | 0.104 | 1.000 | 6980 | tags=55%, list=34%, signal=82% |
| 417 | AGED\_RHESUS\_DN |  | 108 | 0.46 | 1.38 | 0.006 | 0.106 | 1.000 | 6677 | tags=49%, list=32%, signal=72% |
| 418 | 5FU\_RESIST\_GASTRIC\_UP |  | 21 | 0.54 | 1.38 | 0.061 | 0.106 | 1.000 | 3030 | tags=43%, list=15%, signal=50% |
| 419 | CHEN\_LUNG\_SURVIVAL |  | 20 | 0.54 | 1.38 | 0.058 | 0.106 | 1.000 | 1658 | tags=25%, list=8%, signal=27% |
| 420 | CMV\_HCMV\_TIMECOURSE\_14HRS\_DN |  | 41 | 0.50 | 1.37 | 0.033 | 0.107 | 1.000 | 5581 | tags=51%, list=27%, signal=70% |
| 421 | UVC\_HIGH\_D4\_DN |  | 46 | 0.49 | 1.37 | 0.040 | 0.107 | 1.000 | 4709 | tags=33%, list=23%, signal=42% |
| 422 | G1PATHWAY |  | 25 | 0.53 | 1.37 | 0.048 | 0.107 | 1.000 | 5626 | tags=60%, list=27%, signal=82% |
| 423 | IGF\_VS\_PDGF\_DN |  | 43 | 0.50 | 1.37 | 0.025 | 0.107 | 1.000 | 3646 | tags=37%, list=18%, signal=45% |
| 424 | INTEGRIN\_MEDIATED\_CELL\_ADHESION\_KEGG |  | 90 | 0.46 | 1.37 | 0.010 | 0.108 | 1.000 | 7140 | tags=51%, list=35%, signal=78% |
| 425 | UVC\_HIGH\_ALL\_DN |  | 296 | 0.44 | 1.37 | 0.000 | 0.108 | 1.000 | 5874 | tags=43%, list=29%, signal=59% |
| 426 | BRENTANI\_DNA\_METHYLATION\_AND\_MODIFICATION |  | 23 | 0.54 | 1.37 | 0.058 | 0.109 | 1.000 | 5680 | tags=52%, list=28%, signal=72% |
| 427 | OXSTRESS\_RPE\_H2O2HNE\_DN |  | 31 | 0.52 | 1.37 | 0.051 | 0.109 | 1.000 | 4978 | tags=48%, list=24%, signal=64% |
| 428 | CCR5PATHWAY |  | 17 | 0.55 | 1.37 | 0.078 | 0.110 | 1.000 | 445 | tags=12%, list=2%, signal=12% |
| 429 | MANALO\_HYPOXIA\_DN |  | 78 | 0.47 | 1.37 | 0.011 | 0.109 | 1.000 | 8004 | tags=62%, list=39%, signal=100% |
| 430 | HYPOXIA\_REVIEW |  | 81 | 0.47 | 1.37 | 0.012 | 0.109 | 1.000 | 3490 | tags=31%, list=17%, signal=37% |
| 431 | SHIPP\_DLBCL\_CURED\_DN |  | 37 | 0.50 | 1.37 | 0.045 | 0.109 | 1.000 | 6237 | tags=57%, list=30%, signal=81% |
| 432 | AS3\_FIBRO\_C1 |  | 31 | 0.52 | 1.37 | 0.047 | 0.109 | 1.000 | 3743 | tags=39%, list=18%, signal=47% |
| 433 | ZHAN\_MM\_CD138\_LB\_VS\_REST |  | 42 | 0.50 | 1.37 | 0.038 | 0.109 | 1.000 | 3732 | tags=36%, list=18%, signal=44% |
| 434 | LEI\_MYB\_REGULATED\_GENES |  | 317 | 0.44 | 1.37 | 0.000 | 0.109 | 1.000 | 4555 | tags=43%, list=22%, signal=55% |
| 435 | APOPTOSIS\_GENMAPP |  | 43 | 0.49 | 1.37 | 0.033 | 0.109 | 1.000 | 4911 | tags=44%, list=24%, signal=58% |
| 436 | SHEPARD\_CRASH\_AND\_BURN\_MUT\_VS\_WT\_UP |  | 153 | 0.45 | 1.37 | 0.004 | 0.109 | 1.000 | 4431 | tags=34%, list=22%, signal=43% |
| 437 | LINDSTEDT\_DEND\_UP |  | 50 | 0.49 | 1.37 | 0.042 | 0.109 | 1.000 | 2593 | tags=34%, list=13%, signal=39% |
| 438 | TNF\_AND\_FAS\_NETWORK |  | 17 | 0.56 | 1.37 | 0.071 | 0.109 | 1.000 | 6754 | tags=65%, list=33%, signal=96% |
| 439 | CMV\_HCMV\_TIMECOURSE\_6HRS\_DN |  | 52 | 0.49 | 1.37 | 0.031 | 0.109 | 1.000 | 3824 | tags=38%, list=19%, signal=47% |
| 440 | APOPTOSIS |  | 67 | 0.47 | 1.37 | 0.016 | 0.109 | 1.000 | 7303 | tags=61%, list=35%, signal=94% |
| 441 | KIM\_TH\_CELLS\_UP |  | 46 | 0.49 | 1.37 | 0.037 | 0.108 | 1.000 | 2420 | tags=26%, list=12%, signal=29% |
| 442 | HYPOPHYSECTOMY\_RAT\_UP |  | 34 | 0.50 | 1.37 | 0.067 | 0.109 | 1.000 | 3398 | tags=41%, list=16%, signal=49% |
| 443 | LEE\_ACOX1\_UP |  | 64 | 0.47 | 1.37 | 0.026 | 0.109 | 1.000 | 2193 | tags=25%, list=11%, signal=28% |
| 444 | NI2\_LUNG\_DN |  | 20 | 0.54 | 1.37 | 0.069 | 0.109 | 1.000 | 6300 | tags=55%, list=31%, signal=79% |
| 445 | TELPATHWAY |  | 15 | 0.58 | 1.37 | 0.065 | 0.109 | 1.000 | 1638 | tags=27%, list=8%, signal=29% |
| 446 | TPA\_SKIN\_UP |  | 20 | 0.54 | 1.36 | 0.078 | 0.111 | 1.000 | 3956 | tags=45%, list=19%, signal=56% |
| 447 | COCAINE\_BRAIN\_4WKS\_UP |  | 63 | 0.47 | 1.36 | 0.032 | 0.112 | 1.000 | 5383 | tags=38%, list=26%, signal=51% |
| 448 | ELONGINA\_KO\_DN |  | 177 | 0.44 | 1.36 | 0.000 | 0.112 | 1.000 | 5451 | tags=45%, list=26%, signal=61% |
| 449 | AGUIRRE\_PANCREAS\_CHR22 |  | 60 | 0.47 | 1.36 | 0.032 | 0.113 | 1.000 | 7132 | tags=60%, list=35%, signal=91% |
| 450 | CARIES\_PULP\_DN |  | 70 | 0.47 | 1.36 | 0.023 | 0.114 | 1.000 | 1826 | tags=19%, list=9%, signal=20% |
| 451 | P21\_MIDDLE\_DN |  | 15 | 0.57 | 1.36 | 0.078 | 0.114 | 1.000 | 3626 | tags=47%, list=18%, signal=57% |
| 452 | GOLDRATH\_MEMORY |  | 53 | 0.48 | 1.36 | 0.039 | 0.114 | 1.000 | 3584 | tags=32%, list=17%, signal=39% |
| 453 | UVC\_TTD\_8HR\_UP |  | 23 | 0.53 | 1.36 | 0.073 | 0.114 | 1.000 | 1764 | tags=26%, list=9%, signal=28% |
| 454 | VEGF\_MMMEC\_6HRS\_UP |  | 50 | 0.48 | 1.36 | 0.031 | 0.114 | 1.000 | 714 | tags=16%, list=3%, signal=17% |
| 455 | UVB\_NHEK3\_C1 |  | 57 | 0.48 | 1.36 | 0.020 | 0.114 | 1.000 | 4997 | tags=37%, list=24%, signal=49% |
| 456 | MANALO\_HYPOXIA\_UP |  | 94 | 0.46 | 1.36 | 0.009 | 0.114 | 1.000 | 2385 | tags=27%, list=12%, signal=30% |
| 457 | BRENTANI\_PROTEIN\_MODIFICATION |  | 143 | 0.45 | 1.36 | 0.005 | 0.114 | 1.000 | 4820 | tags=38%, list=23%, signal=50% |
| 458 | BASSO\_GERMINAL\_CENTER\_CD40\_UP |  | 97 | 0.45 | 1.36 | 0.011 | 0.114 | 1.000 | 2580 | tags=24%, list=13%, signal=27% |
| 459 | ZHAN\_PCS\_MULTIPLE\_MYELOMA\_SPKD |  | 24 | 0.53 | 1.36 | 0.074 | 0.114 | 1.000 | 1814 | tags=21%, list=9%, signal=23% |
| 460 | UVB\_NHEK3\_C0 |  | 82 | 0.46 | 1.36 | 0.015 | 0.115 | 1.000 | 8008 | tags=63%, list=39%, signal=103% |
| 461 | UVC\_TTD\_ALL\_UP |  | 76 | 0.46 | 1.36 | 0.016 | 0.116 | 1.000 | 6113 | tags=54%, list=30%, signal=76% |
| 462 | P38MAPKPATHWAY |  | 39 | 0.49 | 1.36 | 0.054 | 0.117 | 1.000 | 4518 | tags=51%, list=22%, signal=66% |
| 463 | AGED\_MOUSE\_NEOCORTEX\_UP |  | 67 | 0.47 | 1.36 | 0.024 | 0.116 | 1.000 | 3541 | tags=36%, list=17%, signal=43% |
| 464 | TNFR1PATHWAY |  | 28 | 0.51 | 1.35 | 0.066 | 0.117 | 1.000 | 8045 | tags=79%, list=39%, signal=129% |
| 465 | IDX\_TSA\_UP\_CLUSTER1 |  | 25 | 0.51 | 1.35 | 0.077 | 0.118 | 1.000 | 2845 | tags=32%, list=14%, signal=37% |
| 466 | INOS\_ALL\_DN |  | 77 | 0.46 | 1.35 | 0.023 | 0.117 | 1.000 | 4956 | tags=45%, list=24%, signal=60% |
| 467 | NOUZOVA\_CPG\_H4\_UP |  | 120 | 0.45 | 1.35 | 0.008 | 0.118 | 1.000 | 7217 | tags=54%, list=35%, signal=83% |
| 468 | TPA\_SENS\_LATE\_DN |  | 233 | 0.44 | 1.35 | 0.001 | 0.118 | 1.000 | 5319 | tags=39%, list=26%, signal=52% |
| 469 | ST\_WNT\_BETA\_CATENIN\_PATHWAY |  | 31 | 0.49 | 1.35 | 0.078 | 0.118 | 1.000 | 6236 | tags=48%, list=30%, signal=69% |
| 470 | GCRPATHWAY |  | 17 | 0.56 | 1.35 | 0.074 | 0.118 | 1.000 | 7049 | tags=53%, list=34%, signal=80% |
| 471 | ROSS\_FAB\_M7 |  | 69 | 0.46 | 1.35 | 0.025 | 0.118 | 1.000 | 6189 | tags=46%, list=30%, signal=66% |
| 472 | DORSEY\_DOXYCYCLINE\_UP |  | 29 | 0.51 | 1.35 | 0.063 | 0.118 | 1.000 | 3043 | tags=24%, list=15%, signal=28% |
| 473 | CPR\_LOW\_LIVER\_DN |  | 22 | 0.54 | 1.35 | 0.074 | 0.118 | 1.000 | 4642 | tags=55%, list=23%, signal=70% |
| 474 | BCRABL\_HL60\_CDNA\_DN |  | 28 | 0.52 | 1.35 | 0.076 | 0.120 | 1.000 | 5012 | tags=39%, list=24%, signal=52% |
| 475 | PASSERINI\_PROLIFERATION |  | 64 | 0.47 | 1.35 | 0.030 | 0.120 | 1.000 | 3955 | tags=28%, list=19%, signal=35% |
| 476 | RELAPATHWAY |  | 16 | 0.55 | 1.35 | 0.080 | 0.120 | 1.000 | 7714 | tags=75%, list=37%, signal=120% |
| 477 | TARTE\_BCELL |  | 38 | 0.49 | 1.35 | 0.052 | 0.120 | 1.000 | 1643 | tags=18%, list=8%, signal=20% |
| 478 | POD1\_KO\_UP |  | 412 | 0.43 | 1.35 | 0.000 | 0.122 | 1.000 | 4431 | tags=33%, list=22%, signal=41% |
| 479 | NDKDYNAMINPATHWAY |  | 18 | 0.55 | 1.35 | 0.093 | 0.122 | 1.000 | 6065 | tags=50%, list=29%, signal=71% |
| 480 | AGEING\_KIDNEY\_DN |  | 129 | 0.44 | 1.35 | 0.004 | 0.123 | 1.000 | 6112 | tags=43%, list=30%, signal=61% |
| 481 | ADIPOCYTE\_PPARG\_UP |  | 15 | 0.56 | 1.35 | 0.094 | 0.123 | 1.000 | 3315 | tags=33%, list=16%, signal=40% |
| 482 | HDACI\_COLON\_BUT12HRS\_DN |  | 74 | 0.46 | 1.35 | 0.022 | 0.123 | 1.000 | 5161 | tags=43%, list=25%, signal=57% |
| 483 | OXIDATIVE\_PHOSPHORYLATION |  | 58 | 0.47 | 1.35 | 0.022 | 0.123 | 1.000 | 6636 | tags=50%, list=32%, signal=74% |
| 484 | MMS\_HUMAN\_LYMPH\_LOW\_4HRS\_DN |  | 16 | 0.56 | 1.35 | 0.088 | 0.123 | 1.000 | 4134 | tags=44%, list=20%, signal=55% |
| 485 | HOFMANN\_MDS\_CD34\_HIGH\_RISK |  | 29 | 0.51 | 1.34 | 0.076 | 0.124 | 1.000 | 5021 | tags=38%, list=24%, signal=50% |
| 486 | GH\_AUTOCRINE\_DN |  | 122 | 0.45 | 1.34 | 0.008 | 0.124 | 1.000 | 3454 | tags=34%, list=17%, signal=40% |
| 487 | DORSAM\_HOXA9\_DN |  | 30 | 0.50 | 1.34 | 0.077 | 0.125 | 1.000 | 4466 | tags=43%, list=22%, signal=55% |
| 488 | POMEROY\_DESMOPLASIC\_VS\_CLASSIC\_MD\_UP |  | 46 | 0.48 | 1.34 | 0.046 | 0.125 | 1.000 | 1963 | tags=20%, list=10%, signal=22% |
| 489 | PROTEASOME\_DEGRADATION |  | 31 | 0.50 | 1.34 | 0.059 | 0.125 | 1.000 | 6259 | tags=48%, list=30%, signal=69% |
| 490 | ALCALAY\_AML\_NPMC\_UP |  | 139 | 0.44 | 1.34 | 0.004 | 0.125 | 1.000 | 7346 | tags=53%, list=36%, signal=82% |
| 491 | POMEROY\_MD\_TREATMENT\_GOOD\_VS\_POOR\_DN |  | 24 | 0.51 | 1.34 | 0.095 | 0.125 | 1.000 | 6621 | tags=63%, list=32%, signal=92% |
| 492 | BASSO\_GERMINAL\_CENTER\_CD40\_DN |  | 70 | 0.46 | 1.34 | 0.026 | 0.125 | 1.000 | 4814 | tags=39%, list=23%, signal=50% |
| 493 | AGED\_MOUSE\_HIPPOCAMPUS\_MULTI\_UP |  | 19 | 0.54 | 1.34 | 0.086 | 0.125 | 1.000 | 2090 | tags=21%, list=10%, signal=23% |
| 494 | DORSAM\_HOXA9\_UP |  | 32 | 0.50 | 1.34 | 0.065 | 0.126 | 1.000 | 7126 | tags=53%, list=35%, signal=81% |
| 495 | CHEMICALPATHWAY |  | 21 | 0.53 | 1.34 | 0.080 | 0.127 | 1.000 | 8787 | tags=86%, list=43%, signal=149% |
| 496 | IDX\_TSA\_DN\_CLUSTER2 |  | 62 | 0.46 | 1.34 | 0.039 | 0.127 | 1.000 | 7102 | tags=58%, list=34%, signal=88% |
| 497 | SANSOM\_APC\_LOSS5\_UP |  | 79 | 0.45 | 1.34 | 0.017 | 0.128 | 1.000 | 3124 | tags=33%, list=15%, signal=39% |
| 498 | KANG\_TERT\_UP |  | 79 | 0.45 | 1.34 | 0.026 | 0.129 | 1.000 | 7106 | tags=47%, list=34%, signal=71% |
| 499 | TGFBETA\_C5\_UP |  | 17 | 0.54 | 1.34 | 0.095 | 0.129 | 1.000 | 5663 | tags=53%, list=27%, signal=73% |
| 500 | P21\_P53\_MIDDLE\_DN |  | 25 | 0.52 | 1.34 | 0.089 | 0.130 | 1.000 | 4733 | tags=44%, list=23%, signal=57% |
| 501 | HSC\_MATURE\_SHARED |  | 251 | 0.43 | 1.34 | 0.000 | 0.130 | 1.000 | 5878 | tags=41%, list=29%, signal=57% |
| 502 | XU\_CBP\_DN |  | 36 | 0.50 | 1.34 | 0.050 | 0.130 | 1.000 | 4012 | tags=33%, list=19%, signal=41% |
| 503 | MENSE\_HYPOXIA\_TRANSPORTER\_GENES |  | 48 | 0.48 | 1.33 | 0.054 | 0.132 | 1.000 | 5038 | tags=42%, list=24%, signal=55% |
| 504 | BECKER\_TAMOXIFEN\_RESISTANT\_DN |  | 51 | 0.46 | 1.33 | 0.054 | 0.132 | 1.000 | 3634 | tags=31%, list=18%, signal=38% |
| 505 | PHENYLALANINE\_METABOLISM |  | 22 | 0.53 | 1.33 | 0.080 | 0.135 | 1.000 | 1530 | tags=23%, list=7%, signal=25% |
| 506 | BRACX\_UP |  | 22 | 0.52 | 1.33 | 0.099 | 0.135 | 1.000 | 8237 | tags=73%, list=40%, signal=121% |
| 507 | UVB\_NHEK1\_C6 |  | 130 | 0.44 | 1.33 | 0.009 | 0.136 | 1.000 | 6193 | tags=45%, list=30%, signal=63% |
| 508 | CROONQUIST\_RAS\_STROMA\_UP |  | 24 | 0.51 | 1.33 | 0.086 | 0.138 | 1.000 | 4820 | tags=46%, list=23%, signal=60% |
| 509 | LEE\_TCELLS5\_UP |  | 19 | 0.54 | 1.33 | 0.091 | 0.138 | 1.000 | 6162 | tags=47%, list=30%, signal=68% |
| 510 | NAB\_LUNG\_DN |  | 54 | 0.47 | 1.33 | 0.052 | 0.139 | 1.000 | 3752 | tags=33%, list=18%, signal=41% |
| 511 | ZHAN\_MULTIPLE\_MYELOMA\_VS\_NORMAL\_UP |  | 62 | 0.46 | 1.33 | 0.044 | 0.139 | 1.000 | 6677 | tags=52%, list=32%, signal=76% |
| 512 | NTHIPATHWAY |  | 21 | 0.52 | 1.33 | 0.102 | 0.141 | 1.000 | 7910 | tags=67%, list=38%, signal=108% |
| 513 | SMITH\_HCV\_INDUCED\_HCC\_UP |  | 33 | 0.49 | 1.32 | 0.077 | 0.142 | 1.000 | 4403 | tags=39%, list=21%, signal=50% |
| 514 | PGC |  | 344 | 0.42 | 1.32 | 0.000 | 0.142 | 1.000 | 6046 | tags=44%, list=29%, signal=62% |
| 515 | UCALPAINPATHWAY |  | 16 | 0.55 | 1.32 | 0.114 | 0.143 | 1.000 | 5218 | tags=69%, list=25%, signal=92% |
| 516 | HDACI\_COLON\_TSA\_DN |  | 64 | 0.46 | 1.32 | 0.046 | 0.144 | 1.000 | 4287 | tags=36%, list=21%, signal=45% |
| 517 | FERRANDO\_MLL\_T\_ALL\_DN |  | 83 | 0.45 | 1.32 | 0.023 | 0.144 | 1.000 | 5844 | tags=43%, list=28%, signal=60% |
| 518 | CELL\_MOTILITY |  | 106 | 0.44 | 1.32 | 0.018 | 0.144 | 1.000 | 3743 | tags=29%, list=18%, signal=36% |
| 519 | HDACI\_COLON\_CLUSTER10 |  | 43 | 0.48 | 1.32 | 0.065 | 0.144 | 1.000 | 7514 | tags=63%, list=36%, signal=99% |
| 520 | ZHANG\_EFT\_EWSFLI1\_UP |  | 88 | 0.45 | 1.32 | 0.021 | 0.146 | 1.000 | 3235 | tags=28%, list=16%, signal=34% |
| 521 | ESR\_FIBROBLAST\_UP |  | 50 | 0.46 | 1.32 | 0.047 | 0.146 | 1.000 | 5709 | tags=46%, list=28%, signal=63% |
| 522 | ZMPSTE24\_KO\_DN |  | 32 | 0.49 | 1.32 | 0.082 | 0.146 | 1.000 | 3474 | tags=28%, list=17%, signal=34% |
| 523 | CMV\_HCMV\_6HRS\_DN |  | 55 | 0.46 | 1.32 | 0.045 | 0.148 | 1.000 | 2010 | tags=33%, list=10%, signal=36% |
| 524 | CHESLER\_BRAIN\_CIS\_GENES |  | 57 | 0.46 | 1.32 | 0.042 | 0.148 | 1.000 | 4597 | tags=40%, list=22%, signal=52% |
| 525 | KLEIN\_PEL\_UP |  | 50 | 0.47 | 1.32 | 0.066 | 0.148 | 1.000 | 4998 | tags=40%, list=24%, signal=53% |
| 526 | UVC\_XPCS\_ALL\_DN |  | 478 | 0.42 | 1.32 | 0.000 | 0.149 | 1.000 | 6489 | tags=44%, list=31%, signal=62% |
| 527 | LEE\_MYC\_E2F1\_UP |  | 55 | 0.46 | 1.32 | 0.054 | 0.149 | 1.000 | 5176 | tags=42%, list=25%, signal=56% |
| 528 | CHESLER\_BRAIN\_HIGHEST\_VARIANCE\_GENES |  | 24 | 0.51 | 1.31 | 0.078 | 0.150 | 1.000 | 4597 | tags=42%, list=22%, signal=54% |
| 529 | UVB\_NHEK1\_UP |  | 173 | 0.43 | 1.31 | 0.008 | 0.151 | 1.000 | 5287 | tags=40%, list=26%, signal=54% |
| 530 | HDACI\_COLON\_CUR24HRS\_DN |  | 25 | 0.50 | 1.31 | 0.101 | 0.151 | 1.000 | 5731 | tags=48%, list=28%, signal=66% |
| 531 | HDACI\_COLON\_CUR\_UP |  | 108 | 0.44 | 1.31 | 0.025 | 0.151 | 1.000 | 4706 | tags=38%, list=23%, signal=49% |
| 532 | VANASSE\_BCL2\_TARGETS |  | 86 | 0.44 | 1.31 | 0.031 | 0.152 | 1.000 | 2479 | tags=21%, list=12%, signal=24% |
| 533 | ST\_DIFFERENTIATION\_PATHWAY\_IN\_PC12\_CELLS |  | 43 | 0.47 | 1.31 | 0.050 | 0.152 | 1.000 | 4632 | tags=40%, list=22%, signal=51% |
| 534 | ABRAHAM\_MM\_VS\_AL\_DN |  | 22 | 0.52 | 1.31 | 0.105 | 0.152 | 1.000 | 4706 | tags=36%, list=23%, signal=47% |
| 535 | SANA\_IFNG\_ENDOTHELIAL\_DN |  | 86 | 0.44 | 1.31 | 0.037 | 0.152 | 1.000 | 5482 | tags=44%, list=27%, signal=60% |
| 536 | CIS\_RESIST\_GASTRIC\_UP |  | 16 | 0.54 | 1.31 | 0.122 | 0.152 | 1.000 | 4607 | tags=56%, list=22%, signal=72% |
| 537 | ET743\_HELA\_DN |  | 15 | 0.55 | 1.31 | 0.104 | 0.152 | 1.000 | 763 | tags=20%, list=4%, signal=21% |
| 538 | BRG1\_SW13\_UP |  | 52 | 0.47 | 1.31 | 0.061 | 0.152 | 1.000 | 1685 | tags=23%, list=8%, signal=25% |
| 539 | CMV\_HCMV\_TIMECOURSE\_8HRS\_DN |  | 16 | 0.54 | 1.31 | 0.118 | 0.153 | 1.000 | 1757 | tags=25%, list=9%, signal=27% |
| 540 | LINDSTEDT\_DEND\_DN |  | 65 | 0.45 | 1.31 | 0.052 | 0.153 | 1.000 | 4912 | tags=38%, list=24%, signal=50% |
| 541 | JNK\_DN |  | 31 | 0.49 | 1.31 | 0.087 | 0.153 | 1.000 | 3778 | tags=39%, list=18%, signal=47% |
| 542 | PROTEASOMEPATHWAY |  | 21 | 0.52 | 1.31 | 0.108 | 0.153 | 1.000 | 6949 | tags=62%, list=34%, signal=93% |
| 543 | MITOCHONDRIAPATHWAY |  | 20 | 0.53 | 1.31 | 0.110 | 0.153 | 1.000 | 8111 | tags=75%, list=39%, signal=124% |
| 544 | APOPTOSIS\_KEGG |  | 49 | 0.46 | 1.31 | 0.067 | 0.153 | 1.000 | 8111 | tags=65%, list=39%, signal=107% |
| 545 | TPA\_SENS\_MIDDLE\_DN |  | 302 | 0.42 | 1.31 | 0.001 | 0.153 | 1.000 | 7349 | tags=47%, list=36%, signal=72% |
| 546 | ST\_TUMOR\_NECROSIS\_FACTOR\_PATHWAY |  | 29 | 0.49 | 1.31 | 0.092 | 0.155 | 1.000 | 7714 | tags=69%, list=37%, signal=110% |
| 547 | ZHAN\_MM\_CD138\_MF\_VS\_REST |  | 48 | 0.46 | 1.31 | 0.068 | 0.155 | 1.000 | 4814 | tags=38%, list=23%, signal=49% |
| 548 | LI\_FETAL\_VS\_WT\_KIDNEY\_DN |  | 159 | 0.43 | 1.31 | 0.012 | 0.156 | 1.000 | 4918 | tags=38%, list=24%, signal=49% |
| 549 | SCHURINGA\_STAT5A\_DN |  | 17 | 0.53 | 1.31 | 0.127 | 0.155 | 1.000 | 1767 | tags=18%, list=9%, signal=19% |
| 550 | HDACI\_COLON\_CUR\_DN |  | 49 | 0.46 | 1.31 | 0.064 | 0.156 | 1.000 | 5731 | tags=51%, list=28%, signal=71% |
| 551 | PENG\_LEUCINE\_UP |  | 103 | 0.43 | 1.31 | 0.034 | 0.157 | 1.000 | 4255 | tags=34%, list=21%, signal=43% |
| 552 | UVB\_NHEK3\_ALL |  | 393 | 0.41 | 1.30 | 0.000 | 0.157 | 1.000 | 5731 | tags=43%, list=28%, signal=58% |
| 553 | ECMPATHWAY |  | 21 | 0.51 | 1.30 | 0.123 | 0.158 | 1.000 | 5282 | tags=52%, list=26%, signal=70% |
| 554 | RADMACHER\_AMLNORMALKARYTYPE\_SIG |  | 83 | 0.44 | 1.30 | 0.047 | 0.159 | 1.000 | 4891 | tags=39%, list=24%, signal=50% |
| 555 | UV\_UNIQUE\_FIBRO\_DN |  | 32 | 0.49 | 1.30 | 0.094 | 0.160 | 1.000 | 6533 | tags=53%, list=32%, signal=78% |
| 556 | NAKAJIMA\_MCS\_UP |  | 92 | 0.44 | 1.30 | 0.039 | 0.160 | 1.000 | 305 | tags=9%, list=1%, signal=9% |
| 557 | HSC\_MATURE\_ADULT |  | 331 | 0.42 | 1.30 | 0.000 | 0.160 | 1.000 | 5878 | tags=39%, list=29%, signal=53% |
| 558 | CMV\_HCMV\_TIMECOURSE\_16HRS\_DN |  | 20 | 0.52 | 1.30 | 0.126 | 0.160 | 1.000 | 5363 | tags=45%, list=26%, signal=61% |
| 559 | GAMMA-UV\_FIBRO\_DN |  | 41 | 0.47 | 1.30 | 0.077 | 0.160 | 1.000 | 4758 | tags=37%, list=23%, signal=47% |
| 560 | BYSTROM\_IL5\_UP |  | 41 | 0.47 | 1.30 | 0.082 | 0.160 | 1.000 | 6113 | tags=46%, list=30%, signal=66% |
| 561 | HDACI\_COLON\_SUL2HRS\_DN |  | 17 | 0.53 | 1.30 | 0.127 | 0.161 | 1.000 | 4968 | tags=59%, list=24%, signal=77% |
| 562 | IRITANI\_ADPROX\_VASC |  | 151 | 0.43 | 1.30 | 0.011 | 0.161 | 1.000 | 4016 | tags=29%, list=19%, signal=36% |
| 563 | POMEROY\_DESMOPLASIC\_VS\_CLASSIC\_MD\_DN |  | 41 | 0.47 | 1.30 | 0.093 | 0.162 | 1.000 | 5392 | tags=39%, list=26%, signal=53% |
| 564 | SANA\_TNFA\_ENDOTHELIAL\_DN |  | 83 | 0.44 | 1.30 | 0.047 | 0.162 | 1.000 | 3777 | tags=25%, list=18%, signal=31% |
| 565 | AGED\_MOUSE\_MUSCLE\_DN |  | 32 | 0.48 | 1.30 | 0.107 | 0.163 | 1.000 | 6065 | tags=50%, list=29%, signal=71% |
| 566 | BYSTRYKH\_HSC\_CIS\_GLOCUS |  | 104 | 0.44 | 1.30 | 0.028 | 0.163 | 1.000 | 5319 | tags=40%, list=26%, signal=54% |
| 567 | UVB\_NHEK3\_C5 |  | 35 | 0.48 | 1.30 | 0.075 | 0.163 | 1.000 | 8467 | tags=66%, list=41%, signal=111% |
| 568 | IDX\_TSA\_UP\_CLUSTER5 |  | 104 | 0.43 | 1.30 | 0.028 | 0.163 | 1.000 | 6605 | tags=53%, list=32%, signal=77% |
| 569 | RUTELLA\_HEPATGFSNDCS\_UP |  | 158 | 0.42 | 1.30 | 0.005 | 0.163 | 1.000 | 4388 | tags=34%, list=21%, signal=42% |
| 570 | ROSS\_PML\_RAR |  | 80 | 0.44 | 1.30 | 0.042 | 0.163 | 1.000 | 4882 | tags=36%, list=24%, signal=47% |
| 571 | HASLINGER\_B\_CLL\_12 |  | 18 | 0.52 | 1.30 | 0.145 | 0.163 | 1.000 | 5021 | tags=44%, list=24%, signal=59% |
| 572 | BAF57\_BT549\_UP |  | 238 | 0.42 | 1.30 | 0.002 | 0.164 | 1.000 | 3444 | tags=25%, list=17%, signal=30% |
| 573 | ST\_INTERLEUKIN\_4\_PATHWAY |  | 26 | 0.49 | 1.29 | 0.095 | 0.165 | 1.000 | 6236 | tags=50%, list=30%, signal=72% |
| 574 | TAKEDA\_NUP8\_HOXA9\_10D\_UP |  | 190 | 0.42 | 1.29 | 0.009 | 0.165 | 1.000 | 4881 | tags=33%, list=24%, signal=42% |
| 575 | ABRAHAM\_AL\_VS\_MM\_UP |  | 23 | 0.51 | 1.29 | 0.113 | 0.166 | 1.000 | 4706 | tags=35%, list=23%, signal=45% |
| 576 | VERNELL\_PRB\_CLSTR1 |  | 67 | 0.45 | 1.29 | 0.058 | 0.165 | 1.000 | 7074 | tags=49%, list=34%, signal=75% |
| 577 | GOLDRATH\_CYTOLYTIC |  | 17 | 0.53 | 1.29 | 0.136 | 0.167 | 1.000 | 2736 | tags=24%, list=13%, signal=27% |
| 578 | HSC\_MATURE\_FETAL |  | 325 | 0.41 | 1.29 | 0.001 | 0.166 | 1.000 | 5878 | tags=39%, list=29%, signal=54% |
| 579 | HDACI\_COLON\_TSA48HRS\_UP |  | 42 | 0.47 | 1.29 | 0.078 | 0.167 | 1.000 | 7646 | tags=48%, list=37%, signal=76% |
| 580 | GREENBAUM\_E2A\_DN |  | 17 | 0.53 | 1.29 | 0.125 | 0.168 | 1.000 | 2305 | tags=29%, list=11%, signal=33% |
| 581 | UVC\_XPCS\_4HR\_DN |  | 242 | 0.42 | 1.29 | 0.003 | 0.169 | 1.000 | 6482 | tags=44%, list=31%, signal=64% |
| 582 | HDACI\_COLON\_CUR2HRS\_UP |  | 29 | 0.49 | 1.29 | 0.114 | 0.170 | 1.000 | 4222 | tags=38%, list=20%, signal=48% |
| 583 | CMV\_HCMV\_TIMECOURSE\_ALL\_DN |  | 420 | 0.41 | 1.29 | 0.000 | 0.170 | 1.000 | 5375 | tags=36%, list=26%, signal=48% |
| 584 | TSADAC\_HYPOMETH\_OVCA\_UP |  | 52 | 0.46 | 1.29 | 0.092 | 0.171 | 1.000 | 6178 | tags=44%, list=30%, signal=63% |
| 585 | HSC\_STHSC\_ADULT |  | 41 | 0.47 | 1.29 | 0.081 | 0.171 | 1.000 | 5264 | tags=46%, list=26%, signal=62% |
| 586 | YU\_CMYC\_DN |  | 44 | 0.47 | 1.29 | 0.083 | 0.170 | 1.000 | 6231 | tags=41%, list=30%, signal=59% |
| 587 | FLECHNER\_KIDNEY\_TRANSPLANT\_WELL\_DN |  | 22 | 0.50 | 1.29 | 0.122 | 0.171 | 1.000 | 6523 | tags=59%, list=32%, signal=86% |
| 588 | ABBUD\_LIF\_UP |  | 40 | 0.46 | 1.29 | 0.091 | 0.171 | 1.000 | 4073 | tags=35%, list=20%, signal=44% |
| 589 | PYRIMIDINE\_METABOLISM |  | 58 | 0.45 | 1.29 | 0.069 | 0.171 | 1.000 | 4379 | tags=43%, list=21%, signal=55% |
| 590 | MOUSE\_TISSUE\_TESTIS |  | 34 | 0.48 | 1.29 | 0.093 | 0.172 | 1.000 | 2603 | tags=21%, list=13%, signal=24% |
| 591 | TAVOR\_CEBP\_UP |  | 49 | 0.46 | 1.29 | 0.078 | 0.172 | 1.000 | 1031 | tags=20%, list=5%, signal=21% |
| 592 | CMV\_ALL\_DN |  | 102 | 0.43 | 1.29 | 0.035 | 0.172 | 1.000 | 5116 | tags=40%, list=25%, signal=53% |
| 593 | CALRES\_MOUSE\_NEOCORTEX\_UP |  | 59 | 0.45 | 1.29 | 0.055 | 0.173 | 1.000 | 4823 | tags=44%, list=23%, signal=57% |
| 594 | ROS\_MOUSE\_AORTA\_DN |  | 76 | 0.43 | 1.28 | 0.046 | 0.174 | 1.000 | 4520 | tags=36%, list=22%, signal=45% |
| 595 | BRUNO\_IL3\_DN |  | 63 | 0.44 | 1.28 | 0.070 | 0.173 | 1.000 | 3959 | tags=37%, list=19%, signal=45% |
| 596 | HDACI\_COLON\_BUT48HRS\_UP |  | 96 | 0.44 | 1.28 | 0.031 | 0.175 | 1.000 | 6466 | tags=41%, list=31%, signal=59% |
| 597 | TSA\_HEPATOMA\_CANCER\_DN |  | 16 | 0.53 | 1.28 | 0.126 | 0.175 | 1.000 | 4529 | tags=44%, list=22%, signal=56% |
| 598 | UVC\_TTD\_4HR\_DN |  | 297 | 0.41 | 1.28 | 0.001 | 0.176 | 1.000 | 6477 | tags=43%, list=31%, signal=62% |
| 599 | CREB\_BRAIN\_8WKS\_UP |  | 75 | 0.44 | 1.28 | 0.062 | 0.176 | 1.000 | 5123 | tags=32%, list=25%, signal=42% |
| 600 | WNTPATHWAY |  | 25 | 0.49 | 1.28 | 0.117 | 0.176 | 1.000 | 8658 | tags=68%, list=42%, signal=117% |
| 601 | HPV31\_UP |  | 59 | 0.44 | 1.28 | 0.059 | 0.176 | 1.000 | 2548 | tags=25%, list=12%, signal=29% |
| 602 | CMV\_HCMV\_TIMECOURSE\_20HRS\_UP |  | 84 | 0.43 | 1.28 | 0.046 | 0.177 | 1.000 | 5945 | tags=45%, list=29%, signal=63% |
| 603 | TARTE\_PC |  | 80 | 0.44 | 1.28 | 0.054 | 0.177 | 1.000 | 4923 | tags=41%, list=24%, signal=54% |
| 604 | KENNY\_WNT\_DN |  | 45 | 0.45 | 1.28 | 0.113 | 0.178 | 1.000 | 7782 | tags=56%, list=38%, signal=89% |
| 605 | ALZHEIMERS\_INCIPIENT\_DN |  | 144 | 0.42 | 1.28 | 0.018 | 0.177 | 1.000 | 7238 | tags=45%, list=35%, signal=69% |
| 606 | KANNAN\_P53\_DN |  | 15 | 0.54 | 1.28 | 0.150 | 0.178 | 1.000 | 5118 | tags=40%, list=25%, signal=53% |
| 607 | NAKAJIMA\_MCSMBP\_MAST |  | 46 | 0.46 | 1.28 | 0.091 | 0.178 | 1.000 | 6815 | tags=48%, list=33%, signal=71% |
| 608 | HADDAD\_HSC\_CD10\_UP |  | 296 | 0.41 | 1.28 | 0.004 | 0.178 | 1.000 | 6811 | tags=44%, list=33%, signal=64% |
| 609 | UVC\_XPCS\_8HR\_DN |  | 408 | 0.41 | 1.28 | 0.001 | 0.178 | 1.000 | 6489 | tags=42%, list=31%, signal=60% |
| 610 | IRITANI\_ADPROX\_UP |  | 26 | 0.48 | 1.28 | 0.121 | 0.181 | 1.000 | 6733 | tags=58%, list=33%, signal=86% |
| 611 | NKTPATHWAY |  | 26 | 0.49 | 1.28 | 0.118 | 0.181 | 1.000 | 1158 | tags=12%, list=6%, signal=12% |
| 612 | GLYCOSPHINGOLIPID\_METABOLISM |  | 23 | 0.50 | 1.28 | 0.145 | 0.181 | 1.000 | 8598 | tags=70%, list=42%, signal=119% |
| 613 | TPA\_SENS\_EARLY\_DN |  | 284 | 0.41 | 1.28 | 0.003 | 0.182 | 1.000 | 5319 | tags=38%, list=26%, signal=51% |
| 614 | HADDAD\_HPCLYMPHO\_ENRICHED |  | 309 | 0.41 | 1.28 | 0.002 | 0.182 | 1.000 | 6865 | tags=44%, list=33%, signal=65% |
| 615 | ABBUD\_LIF\_DN |  | 24 | 0.49 | 1.27 | 0.126 | 0.183 | 1.000 | 6815 | tags=42%, list=33%, signal=62% |
| 616 | LEE\_MYC\_UP |  | 53 | 0.44 | 1.27 | 0.086 | 0.183 | 1.000 | 6466 | tags=55%, list=31%, signal=80% |
| 617 | AGEING\_KIDNEY\_SPECIFIC\_UP |  | 183 | 0.41 | 1.27 | 0.019 | 0.183 | 1.000 | 4435 | tags=29%, list=22%, signal=37% |
| 618 | HYPERME\_COLONCA\_SW48 |  | 18 | 0.52 | 1.27 | 0.151 | 0.183 | 1.000 | 7206 | tags=56%, list=35%, signal=85% |
| 619 | PARP\_KO\_UP |  | 30 | 0.48 | 1.27 | 0.132 | 0.183 | 1.000 | 4399 | tags=43%, list=21%, signal=55% |
| 620 | RORIE\_ES\_PNET\_DN |  | 27 | 0.48 | 1.27 | 0.128 | 0.184 | 1.000 | 1048 | tags=11%, list=5%, signal=12% |
| 621 | FLECHNER\_KIDNEY\_TRANSPLANT\_REJECTION\_UP |  | 86 | 0.43 | 1.27 | 0.066 | 0.184 | 1.000 | 2545 | tags=21%, list=12%, signal=24% |
| 622 | AGEING\_BRAIN\_UP |  | 208 | 0.41 | 1.27 | 0.011 | 0.184 | 1.000 | 5862 | tags=41%, list=28%, signal=57% |
| 623 | IGF1\_NIH3T3\_UP |  | 35 | 0.47 | 1.27 | 0.111 | 0.185 | 1.000 | 5032 | tags=43%, list=24%, signal=57% |
| 624 | STRESSPATHWAY |  | 24 | 0.49 | 1.27 | 0.138 | 0.184 | 1.000 | 3242 | tags=38%, list=16%, signal=44% |
| 625 | VERNELL\_PRB\_CLSTR2 |  | 22 | 0.50 | 1.27 | 0.125 | 0.185 | 1.000 | 6095 | tags=59%, list=30%, signal=84% |
| 626 | TARTE\_PLASMA\_BLASTIC |  | 307 | 0.41 | 1.27 | 0.002 | 0.185 | 1.000 | 5118 | tags=39%, list=25%, signal=51% |
| 627 | WERNERONLY\_FIBRO\_DN |  | 64 | 0.44 | 1.27 | 0.062 | 0.185 | 1.000 | 5241 | tags=38%, list=25%, signal=50% |
| 628 | BRENTANI\_SIGNALING |  | 178 | 0.42 | 1.27 | 0.021 | 0.186 | 1.000 | 8587 | tags=56%, list=42%, signal=95% |
| 629 | SHEPARD\_BMYB\_MORPHOLINO\_DN |  | 168 | 0.42 | 1.27 | 0.019 | 0.187 | 1.000 | 4912 | tags=33%, list=24%, signal=43% |
| 630 | RACCYCDPATHWAY |  | 22 | 0.50 | 1.27 | 0.149 | 0.187 | 1.000 | 8658 | tags=68%, list=42%, signal=117% |
| 631 | UVC\_TTD\_4HR\_UP |  | 58 | 0.45 | 1.27 | 0.078 | 0.187 | 1.000 | 8096 | tags=72%, list=39%, signal=119% |
| 632 | ROSS\_MLL\_FUSION |  | 83 | 0.43 | 1.27 | 0.053 | 0.188 | 1.000 | 6678 | tags=40%, list=32%, signal=59% |
| 633 | BRCA1\_OVEREXP\_DN |  | 110 | 0.42 | 1.27 | 0.044 | 0.188 | 1.000 | 5623 | tags=45%, list=27%, signal=61% |
| 634 | HBX\_NL\_UP |  | 22 | 0.49 | 1.27 | 0.147 | 0.188 | 1.000 | 7410 | tags=55%, list=36%, signal=85% |
| 635 | UVC\_HIGH\_ALL\_UP |  | 19 | 0.51 | 1.27 | 0.151 | 0.188 | 1.000 | 220 | tags=16%, list=1%, signal=16% |
| 636 | WERNER\_FIBRO\_UP |  | 56 | 0.45 | 1.27 | 0.095 | 0.189 | 1.000 | 6842 | tags=45%, list=33%, signal=67% |
| 637 | MATSUDA\_VALPHAINKT\_DIFF |  | 408 | 0.40 | 1.27 | 0.000 | 0.190 | 1.000 | 4368 | tags=30%, list=21%, signal=37% |
| 638 | ST\_B\_CELL\_ANTIGEN\_RECEPTOR |  | 39 | 0.46 | 1.26 | 0.109 | 0.191 | 1.000 | 9880 | tags=69%, list=48%, signal=133% |
| 639 | HESS\_HOXAANMEIS1\_DN |  | 60 | 0.44 | 1.26 | 0.103 | 0.192 | 1.000 | 4794 | tags=40%, list=23%, signal=52% |
| 640 | NEMETH\_TNF\_UP |  | 80 | 0.43 | 1.26 | 0.054 | 0.192 | 1.000 | 2613 | tags=23%, list=13%, signal=26% |
| 641 | RADIATION\_SENSITIVITY |  | 24 | 0.49 | 1.26 | 0.157 | 0.192 | 1.000 | 5728 | tags=50%, list=28%, signal=69% |
| 642 | NFKBPATHWAY |  | 23 | 0.49 | 1.26 | 0.126 | 0.192 | 1.000 | 7910 | tags=65%, list=38%, signal=106% |
| 643 | CROONQUIST\_IL6\_STROMA\_UP |  | 37 | 0.46 | 1.26 | 0.122 | 0.192 | 1.000 | 1676 | tags=22%, list=8%, signal=23% |
| 644 | OLDONLY\_FIBRO\_DN |  | 53 | 0.44 | 1.26 | 0.100 | 0.192 | 1.000 | 7687 | tags=53%, list=37%, signal=84% |
| 645 | TAKEDA\_NUP8\_HOXA9\_6H\_DN |  | 40 | 0.46 | 1.26 | 0.106 | 0.194 | 1.000 | 4706 | tags=30%, list=23%, signal=39% |
| 646 | ST\_ERK1\_ERK2\_MAPK\_PATHWAY |  | 30 | 0.47 | 1.26 | 0.145 | 0.195 | 1.000 | 9226 | tags=70%, list=45%, signal=127% |
| 647 | GENOTOXINS\_4HRS\_DISCR |  | 34 | 0.46 | 1.26 | 0.116 | 0.195 | 1.000 | 5728 | tags=38%, list=28%, signal=53% |
| 648 | CARM\_ERPATHWAY |  | 26 | 0.49 | 1.26 | 0.136 | 0.195 | 1.000 | 9360 | tags=81%, list=45%, signal=148% |
| 649 | UVC\_TTD\_ALL\_DN |  | 358 | 0.40 | 1.26 | 0.002 | 0.195 | 1.000 | 8204 | tags=53%, list=40%, signal=87% |
| 650 | ST\_INTEGRIN\_SIGNALING\_PATHWAY |  | 78 | 0.43 | 1.26 | 0.066 | 0.195 | 1.000 | 9253 | tags=65%, list=45%, signal=118% |
| 651 | HCMVPATHWAY |  | 15 | 0.53 | 1.26 | 0.157 | 0.196 | 1.000 | 6236 | tags=47%, list=30%, signal=67% |
| 652 | ST\_FAS\_SIGNALING\_PATHWAY |  | 61 | 0.44 | 1.26 | 0.080 | 0.197 | 1.000 | 5605 | tags=46%, list=27%, signal=63% |
| 653 | HESS\_HOXAANMEIS1\_UP |  | 60 | 0.44 | 1.26 | 0.100 | 0.196 | 1.000 | 4794 | tags=40%, list=23%, signal=52% |
| 654 | AGUIRRE\_PANCREAS\_CHR6 |  | 32 | 0.48 | 1.26 | 0.123 | 0.197 | 1.000 | 8951 | tags=78%, list=43%, signal=138% |
| 655 | CALRES\_RHESUS\_DN |  | 59 | 0.44 | 1.26 | 0.080 | 0.197 | 1.000 | 6865 | tags=49%, list=33%, signal=73% |
| 656 | PASSERINI\_SIGNAL |  | 338 | 0.40 | 1.26 | 0.002 | 0.197 | 1.000 | 5732 | tags=35%, list=28%, signal=48% |
| 657 | PTENPATHWAY |  | 17 | 0.51 | 1.26 | 0.167 | 0.198 | 1.000 | 7688 | tags=65%, list=37%, signal=103% |
| 658 | GUO\_HEX\_UP |  | 81 | 0.43 | 1.26 | 0.069 | 0.198 | 1.000 | 6949 | tags=57%, list=34%, signal=85% |
| 659 | TPA\_SENS\_EARLY\_UP |  | 47 | 0.45 | 1.26 | 0.106 | 0.198 | 1.000 | 3929 | tags=34%, list=19%, signal=42% |
| 660 | ASTON\_DEPRESSION\_DN |  | 150 | 0.41 | 1.26 | 0.039 | 0.197 | 1.000 | 5640 | tags=41%, list=27%, signal=56% |
| 661 | CERAMIDEPATHWAY |  | 22 | 0.49 | 1.25 | 0.145 | 0.200 | 1.000 | 8147 | tags=68%, list=40%, signal=113% |
| 662 | GALACTOSE\_METABOLISM |  | 23 | 0.49 | 1.25 | 0.150 | 0.202 | 1.000 | 6670 | tags=43%, list=32%, signal=64% |
| 663 | GUO\_HEX\_DN |  | 61 | 0.44 | 1.25 | 0.090 | 0.202 | 1.000 | 7570 | tags=48%, list=37%, signal=75% |
| 664 | IL1\_CORNEA\_DN |  | 74 | 0.43 | 1.25 | 0.088 | 0.202 | 1.000 | 5209 | tags=38%, list=25%, signal=50% |
| 665 | ST\_GAQ\_PATHWAY |  | 27 | 0.48 | 1.25 | 0.144 | 0.203 | 1.000 | 7714 | tags=67%, list=37%, signal=106% |
| 666 | BRCA\_BRCA1\_POS |  | 106 | 0.42 | 1.25 | 0.056 | 0.204 | 1.000 | 6239 | tags=40%, list=30%, signal=57% |
| 667 | SIG\_PIP3\_SIGNALING\_IN\_CARDIAC\_MYOCTES |  | 66 | 0.43 | 1.25 | 0.093 | 0.204 | 1.000 | 4736 | tags=36%, list=23%, signal=47% |
| 668 | NAB\_LUNG\_UP |  | 27 | 0.47 | 1.25 | 0.132 | 0.205 | 1.000 | 2209 | tags=37%, list=11%, signal=41% |
| 669 | SHIPP\_FL\_VS\_DLBCL\_DN |  | 34 | 0.46 | 1.25 | 0.140 | 0.206 | 1.000 | 4307 | tags=35%, list=21%, signal=45% |
| 670 | ET743\_SARCOMA\_24HRS\_DN |  | 110 | 0.42 | 1.25 | 0.052 | 0.207 | 1.000 | 7537 | tags=49%, list=37%, signal=77% |
| 671 | LEE\_TCELLS3\_UP |  | 106 | 0.42 | 1.25 | 0.057 | 0.207 | 1.000 | 5176 | tags=36%, list=25%, signal=48% |
| 672 | ZHAN\_MMPC\_EARLYVS |  | 48 | 0.44 | 1.25 | 0.112 | 0.207 | 1.000 | 4923 | tags=40%, list=24%, signal=52% |
| 673 | ST\_JNK\_MAPK\_PATHWAY |  | 40 | 0.45 | 1.25 | 0.134 | 0.207 | 1.000 | 8606 | tags=70%, list=42%, signal=120% |
| 674 | ATRBRCAPATHWAY |  | 21 | 0.48 | 1.25 | 0.180 | 0.207 | 1.000 | 7548 | tags=62%, list=37%, signal=98% |
| 675 | CASPASEPATHWAY |  | 22 | 0.48 | 1.25 | 0.171 | 0.210 | 1.000 | 7277 | tags=68%, list=35%, signal=105% |
| 676 | LAIRPATHWAY |  | 15 | 0.53 | 1.25 | 0.184 | 0.210 | 1.000 | 3743 | tags=40%, list=18%, signal=49% |
| 677 | IRITANI\_ADPROX\_DN |  | 60 | 0.43 | 1.25 | 0.096 | 0.211 | 1.000 | 3849 | tags=30%, list=19%, signal=37% |
| 678 | TPA\_SENS\_LATE\_UP |  | 52 | 0.44 | 1.25 | 0.110 | 0.211 | 1.000 | 4497 | tags=35%, list=22%, signal=44% |
| 679 | SERUM\_FIBROBLAST\_CELLCYCLE |  | 136 | 0.41 | 1.24 | 0.039 | 0.211 | 1.000 | 6910 | tags=47%, list=34%, signal=70% |
| 680 | TSADAC\_RKOSILENT\_UP |  | 20 | 0.49 | 1.24 | 0.182 | 0.211 | 1.000 | 4597 | tags=40%, list=22%, signal=51% |
| 681 | RNA\_TRANSCRIPTION\_REACTOME |  | 37 | 0.45 | 1.24 | 0.141 | 0.213 | 1.000 | 8753 | tags=62%, list=42%, signal=108% |
| 682 | NGUYEN\_KERATO\_DN |  | 81 | 0.42 | 1.24 | 0.073 | 0.215 | 1.000 | 4627 | tags=37%, list=22%, signal=48% |
| 683 | UV\_UNIQUE\_FIBRO\_UP |  | 22 | 0.49 | 1.24 | 0.167 | 0.215 | 1.000 | 5093 | tags=45%, list=25%, signal=60% |
| 684 | ST\_GRANULE\_CELL\_SURVIVAL\_PATHWAY |  | 27 | 0.47 | 1.24 | 0.156 | 0.215 | 1.000 | 4740 | tags=44%, list=23%, signal=58% |
| 685 | VANTVEER\_BREAST\_OUTCOME\_GOOD\_VS\_POOR\_DN |  | 64 | 0.42 | 1.24 | 0.124 | 0.216 | 1.000 | 4547 | tags=36%, list=22%, signal=46% |
| 686 | BRCA2\_BRCA1\_DN |  | 42 | 0.45 | 1.24 | 0.113 | 0.216 | 1.000 | 7305 | tags=50%, list=35%, signal=77% |
| 687 | AS3\_FIBRO\_UP |  | 44 | 0.45 | 1.24 | 0.132 | 0.216 | 1.000 | 5728 | tags=43%, list=28%, signal=60% |
| 688 | HEMATOPOESIS\_RELATED\_TRANSCRIPTION\_FACTORS |  | 84 | 0.42 | 1.24 | 0.077 | 0.219 | 1.000 | 5005 | tags=33%, list=24%, signal=44% |
| 689 | SIG\_REGULATION\_OF\_THE\_ACTIN\_CYTOSKELETON\_BY\_RHO\_GTPASES |  | 35 | 0.46 | 1.24 | 0.143 | 0.219 | 1.000 | 3640 | tags=31%, list=18%, signal=38% |
| 690 | FSH\_GRANULOSA\_UP |  | 78 | 0.42 | 1.24 | 0.092 | 0.220 | 1.000 | 5229 | tags=44%, list=25%, signal=58% |
| 691 | SIG\_CHEMOTAXIS |  | 44 | 0.44 | 1.24 | 0.145 | 0.220 | 1.000 | 3640 | tags=30%, list=18%, signal=36% |
| 692 | IGLESIAS\_E2FMINUS\_UP |  | 136 | 0.41 | 1.24 | 0.052 | 0.220 | 1.000 | 4911 | tags=35%, list=24%, signal=46% |
| 693 | UVC\_HIGH\_D8\_DN |  | 30 | 0.46 | 1.24 | 0.162 | 0.222 | 1.000 | 5161 | tags=47%, list=25%, signal=62% |
| 694 | FALT\_BCLL\_IG\_MUTATED\_VS\_WT\_UP |  | 48 | 0.44 | 1.24 | 0.133 | 0.223 | 1.000 | 7525 | tags=63%, list=37%, signal=98% |
| 695 | PURINE\_METABOLISM |  | 114 | 0.41 | 1.23 | 0.066 | 0.223 | 1.000 | 5017 | tags=33%, list=24%, signal=44% |
| 696 | ST\_G\_ALPHA\_I\_PATHWAY |  | 34 | 0.46 | 1.23 | 0.154 | 0.224 | 1.000 | 9226 | tags=65%, list=45%, signal=117% |
| 697 | EIF4PATHWAY |  | 24 | 0.48 | 1.23 | 0.176 | 0.224 | 1.000 | 6236 | tags=42%, list=30%, signal=60% |
| 698 | TCA |  | 15 | 0.52 | 1.23 | 0.192 | 0.224 | 1.000 | 6119 | tags=53%, list=30%, signal=76% |
| 699 | GENOTOXINS\_24HRS\_DISCR |  | 39 | 0.44 | 1.23 | 0.158 | 0.226 | 1.000 | 4983 | tags=46%, list=24%, signal=61% |
| 700 | HDACI\_COLON\_BUT16HRS\_UP |  | 42 | 0.45 | 1.23 | 0.129 | 0.226 | 1.000 | 4540 | tags=36%, list=22%, signal=46% |
| 701 | VEGF\_HUVEC\_UP |  | 15 | 0.52 | 1.23 | 0.187 | 0.226 | 1.000 | 3970 | tags=40%, list=19%, signal=50% |
| 702 | AS3\_FIBRO\_C3 |  | 44 | 0.45 | 1.23 | 0.136 | 0.227 | 1.000 | 5728 | tags=43%, list=28%, signal=60% |
| 703 | GLYCOGEN |  | 19 | 0.49 | 1.23 | 0.186 | 0.227 | 1.000 | 6477 | tags=53%, list=31%, signal=77% |
| 704 | UEDA\_MOUSE\_LIVER |  | 133 | 0.41 | 1.23 | 0.049 | 0.227 | 1.000 | 5116 | tags=39%, list=25%, signal=52% |
| 705 | HOFFMANN\_BIVSBII\_BI\_TABLE2 |  | 197 | 0.40 | 1.23 | 0.023 | 0.227 | 1.000 | 5901 | tags=40%, list=29%, signal=56% |
| 706 | FASPATHWAY |  | 27 | 0.47 | 1.23 | 0.176 | 0.228 | 1.000 | 7277 | tags=67%, list=35%, signal=103% |
| 707 | CANCER\_UNDIFFERENTIATED\_META\_UP |  | 67 | 0.43 | 1.23 | 0.096 | 0.229 | 1.000 | 5848 | tags=46%, list=28%, signal=64% |
| 708 | ZHAN\_MMPC\_SIMAL |  | 47 | 0.44 | 1.23 | 0.132 | 0.228 | 1.000 | 4923 | tags=40%, list=24%, signal=53% |
| 709 | SIG\_BCR\_SIGNALING\_PATHWAY |  | 45 | 0.44 | 1.23 | 0.146 | 0.229 | 1.000 | 6236 | tags=42%, list=30%, signal=60% |
| 710 | ST\_DICTYOSTELIUM\_DISCOIDEUM\_CAMP\_CHEMOTAXIS\_PATHWAY |  | 32 | 0.46 | 1.23 | 0.167 | 0.229 | 1.000 | 4393 | tags=28%, list=21%, signal=36% |
| 711 | CMV\_24HRS\_DN |  | 71 | 0.42 | 1.23 | 0.102 | 0.230 | 1.000 | 4353 | tags=32%, list=21%, signal=41% |
| 712 | VERHAAK\_AML\_NPM1\_MUT\_VS\_WT\_UP |  | 189 | 0.40 | 1.23 | 0.030 | 0.232 | 1.000 | 2263 | tags=19%, list=11%, signal=21% |
| 713 | CMV\_HCMV\_TIMECOURSE\_ALL\_UP |  | 464 | 0.39 | 1.23 | 0.002 | 0.232 | 1.000 | 6291 | tags=41%, list=31%, signal=58% |
| 714 | ARAPPATHWAY |  | 20 | 0.49 | 1.23 | 0.203 | 0.233 | 1.000 | 7626 | tags=60%, list=37%, signal=95% |
| 715 | AKTPATHWAY |  | 17 | 0.51 | 1.23 | 0.201 | 0.233 | 1.000 | 8357 | tags=65%, list=41%, signal=109% |
| 716 | PHOTOSYNTHESIS |  | 22 | 0.48 | 1.23 | 0.183 | 0.233 | 1.000 | 4710 | tags=36%, list=23%, signal=47% |
| 717 | HDACI\_COLON\_TSA2HRS\_DN |  | 22 | 0.48 | 1.22 | 0.174 | 0.233 | 1.000 | 6350 | tags=59%, list=31%, signal=85% |
| 718 | DIAB\_NEPH\_UP |  | 61 | 0.43 | 1.22 | 0.122 | 0.233 | 1.000 | 4079 | tags=30%, list=20%, signal=37% |
| 719 | NADLER\_OBESITY\_HYPERGLYCEMIA |  | 42 | 0.44 | 1.22 | 0.157 | 0.233 | 1.000 | 6300 | tags=43%, list=31%, signal=62% |
| 720 | BAF57\_BT549\_DN |  | 330 | 0.39 | 1.22 | 0.010 | 0.233 | 1.000 | 6422 | tags=43%, list=31%, signal=62% |
| 721 | ADIPOGENESIS\_HMSC\_CLASS8\_DN |  | 32 | 0.45 | 1.22 | 0.161 | 0.234 | 1.000 | 3849 | tags=34%, list=19%, signal=42% |
| 722 | INOS\_ALL\_UP |  | 53 | 0.43 | 1.22 | 0.143 | 0.235 | 1.000 | 5599 | tags=49%, list=27%, signal=67% |
| 723 | ESR\_FIBROBLAST\_DN |  | 18 | 0.50 | 1.22 | 0.186 | 0.234 | 1.000 | 3041 | tags=33%, list=15%, signal=39% |
| 724 | AD12\_ANY\_DN |  | 25 | 0.47 | 1.22 | 0.189 | 0.235 | 1.000 | 3795 | tags=32%, list=18%, signal=39% |
| 725 | UVB\_NHEK1\_C1 |  | 51 | 0.44 | 1.22 | 0.140 | 0.235 | 1.000 | 6778 | tags=49%, list=33%, signal=73% |
| 726 | RAC1PATHWAY |  | 22 | 0.48 | 1.22 | 0.187 | 0.235 | 1.000 | 5911 | tags=45%, list=29%, signal=64% |
| 727 | CALRES\_MOUSE\_DN |  | 39 | 0.44 | 1.22 | 0.150 | 0.235 | 1.000 | 7311 | tags=56%, list=35%, signal=87% |
| 728 | BRCA1\_SW480\_UP |  | 25 | 0.47 | 1.22 | 0.179 | 0.237 | 1.000 | 4850 | tags=44%, list=24%, signal=57% |
| 729 | TFF2\_KO\_UP |  | 23 | 0.48 | 1.22 | 0.179 | 0.237 | 1.000 | 4724 | tags=43%, list=23%, signal=56% |
| 730 | WELCH\_GATA1 |  | 24 | 0.47 | 1.22 | 0.190 | 0.237 | 1.000 | 3292 | tags=33%, list=16%, signal=40% |
| 731 | LH\_GRANULOSA\_UP |  | 80 | 0.41 | 1.22 | 0.108 | 0.238 | 1.000 | 5229 | tags=43%, list=25%, signal=57% |
| 732 | SMITH\_HTERT\_UP |  | 102 | 0.41 | 1.22 | 0.088 | 0.239 | 1.000 | 5167 | tags=36%, list=25%, signal=48% |
| 733 | PITX2PATHWAY |  | 16 | 0.50 | 1.22 | 0.199 | 0.240 | 1.000 | 6472 | tags=50%, list=31%, signal=73% |
| 734 | UVB\_NHEK4\_6HRS\_DN |  | 19 | 0.50 | 1.22 | 0.197 | 0.241 | 1.000 | 7189 | tags=63%, list=35%, signal=97% |
| 735 | AD12\_24HRS\_DN |  | 18 | 0.49 | 1.22 | 0.196 | 0.242 | 1.000 | 7746 | tags=61%, list=38%, signal=98% |
| 736 | MMS\_MOUSE\_LYMPH\_HIGH\_4HRS\_UP |  | 35 | 0.45 | 1.22 | 0.182 | 0.242 | 1.000 | 8272 | tags=51%, list=40%, signal=86% |
| 737 | LAL\_KO\_3MO\_UP |  | 46 | 0.43 | 1.22 | 0.160 | 0.242 | 1.000 | 1009 | tags=11%, list=5%, signal=11% |
| 738 | RAY\_P210\_DIFF |  | 53 | 0.43 | 1.22 | 0.155 | 0.242 | 1.000 | 5012 | tags=40%, list=24%, signal=52% |
| 739 | POD1\_KO\_MOST\_UP |  | 32 | 0.45 | 1.22 | 0.169 | 0.242 | 1.000 | 727 | tags=16%, list=4%, signal=16% |
| 740 | VENTRICLES\_UP |  | 205 | 0.40 | 1.21 | 0.039 | 0.243 | 1.000 | 6140 | tags=37%, list=30%, signal=52% |
| 741 | ZHAN\_TONSIL\_PCBC |  | 43 | 0.44 | 1.21 | 0.158 | 0.245 | 1.000 | 4923 | tags=40%, list=24%, signal=52% |
| 742 | YAGI\_AML\_PROG\_ASSOC |  | 127 | 0.40 | 1.21 | 0.064 | 0.246 | 1.000 | 5531 | tags=39%, list=27%, signal=53% |
| 743 | CREB\_BRAIN\_2WKS\_UP |  | 24 | 0.47 | 1.21 | 0.197 | 0.246 | 1.000 | 5123 | tags=50%, list=25%, signal=66% |
| 744 | P21\_P53\_ANY\_DN |  | 49 | 0.43 | 1.21 | 0.156 | 0.246 | 1.000 | 5691 | tags=39%, list=28%, signal=53% |
| 745 | DSRNA\_DN |  | 15 | 0.50 | 1.21 | 0.220 | 0.247 | 1.000 | 4045 | tags=33%, list=20%, signal=41% |
| 746 | WNT\_SIGNALING |  | 59 | 0.42 | 1.21 | 0.143 | 0.247 | 1.000 | 5610 | tags=32%, list=27%, signal=44% |
| 747 | HDACI\_COLON\_CURSUL\_UP |  | 42 | 0.44 | 1.21 | 0.165 | 0.247 | 1.000 | 3619 | tags=33%, list=18%, signal=40% |
| 748 | DRUG\_RESISTANCE\_AND\_METABOLISM |  | 95 | 0.41 | 1.21 | 0.094 | 0.247 | 1.000 | 7548 | tags=47%, list=37%, signal=74% |
| 749 | BCNU\_GLIOMA\_MGMT\_24HRS\_DN |  | 32 | 0.45 | 1.21 | 0.174 | 0.247 | 1.000 | 3699 | tags=28%, list=18%, signal=34% |
| 750 | HADDAD\_HSC\_CD7\_DN |  | 84 | 0.41 | 1.21 | 0.113 | 0.247 | 1.000 | 4962 | tags=35%, list=24%, signal=45% |
| 751 | UVC\_HIGH\_D3\_DN |  | 46 | 0.43 | 1.21 | 0.148 | 0.248 | 1.000 | 6085 | tags=43%, list=30%, signal=62% |
| 752 | MEF2DPATHWAY |  | 20 | 0.48 | 1.21 | 0.229 | 0.249 | 1.000 | 3705 | tags=30%, list=18%, signal=37% |
| 753 | P21\_ANY\_DN |  | 35 | 0.45 | 1.21 | 0.169 | 0.250 | 1.000 | 5728 | tags=43%, list=28%, signal=59% |
| 754 | H2O2\_CSBDIFF\_C1 |  | 33 | 0.45 | 1.21 | 0.171 | 0.252 | 1.000 | 2883 | tags=39%, list=14%, signal=46% |
| 755 | ET743\_RESIST\_UP |  | 17 | 0.49 | 1.21 | 0.208 | 0.252 | 1.000 | 4423 | tags=47%, list=21%, signal=60% |
| 756 | CANCERDRUGS\_PROBCELL\_DN |  | 15 | 0.50 | 1.21 | 0.222 | 0.253 | 1.000 | 2864 | tags=33%, list=14%, signal=39% |
| 757 | SIG\_CD40PATHWAYMAP |  | 33 | 0.45 | 1.21 | 0.183 | 0.254 | 1.000 | 9757 | tags=73%, list=47%, signal=138% |
| 758 | KUMAR\_HOXA\_DIFF |  | 343 | 0.38 | 1.21 | 0.006 | 0.254 | 1.000 | 7023 | tags=42%, list=34%, signal=62% |
| 759 | INSULIN\_NIH3T3\_UP |  | 17 | 0.49 | 1.21 | 0.217 | 0.255 | 1.000 | 4923 | tags=35%, list=24%, signal=46% |
| 760 | ERK5PATHWAY |  | 17 | 0.50 | 1.21 | 0.201 | 0.255 | 1.000 | 9757 | tags=82%, list=47%, signal=156% |
| 761 | CITRATE\_CYCLE\_TCA\_CYCLE |  | 20 | 0.47 | 1.20 | 0.219 | 0.259 | 1.000 | 6119 | tags=45%, list=30%, signal=64% |
| 762 | UVC\_HIGH\_D9\_DN |  | 24 | 0.46 | 1.20 | 0.193 | 0.260 | 1.000 | 6477 | tags=54%, list=31%, signal=79% |
| 763 | UVC\_TTD\_8HR\_DN |  | 165 | 0.39 | 1.20 | 0.059 | 0.261 | 1.000 | 9723 | tags=65%, list=47%, signal=122% |
| 764 | UVC\_TTD-XPCS\_COMMON\_UP |  | 21 | 0.47 | 1.20 | 0.228 | 0.261 | 1.000 | 1274 | tags=24%, list=6%, signal=25% |
| 765 | NGUYEN\_KERATO\_UP |  | 28 | 0.45 | 1.20 | 0.208 | 0.264 | 1.000 | 6726 | tags=50%, list=33%, signal=74% |
| 766 | WANG\_MLL\_CBP\_VS\_GMP\_UP |  | 36 | 0.44 | 1.20 | 0.211 | 0.264 | 1.000 | 5489 | tags=47%, list=27%, signal=64% |
| 767 | HADDAD\_CD45CD7\_PLUS\_VS\_MINUS\_DN |  | 84 | 0.41 | 1.20 | 0.111 | 0.265 | 1.000 | 4962 | tags=35%, list=24%, signal=45% |
| 768 | YE\_INTRAMETASTATIC\_HCC\_UP |  | 21 | 0.47 | 1.20 | 0.231 | 0.267 | 1.000 | 2787 | tags=29%, list=14%, signal=33% |
| 769 | RARRXRPATHWAY |  | 15 | 0.50 | 1.20 | 0.223 | 0.267 | 1.000 | 8297 | tags=73%, list=40%, signal=123% |
| 770 | UVC\_XPCS\_8HR\_UP |  | 58 | 0.42 | 1.20 | 0.160 | 0.267 | 1.000 | 2924 | tags=28%, list=14%, signal=32% |
| 771 | CARDIACEGFPATHWAY |  | 17 | 0.49 | 1.20 | 0.228 | 0.267 | 1.000 | 1073 | tags=18%, list=5%, signal=19% |
| 772 | IGF1RPATHWAY |  | 15 | 0.51 | 1.20 | 0.229 | 0.267 | 1.000 | 3015 | tags=33%, list=15%, signal=39% |
| 773 | AT1RPATHWAY |  | 34 | 0.44 | 1.19 | 0.206 | 0.270 | 1.000 | 9393 | tags=65%, list=46%, signal=119% |
| 774 | UVB\_SCC\_DN |  | 105 | 0.40 | 1.19 | 0.104 | 0.272 | 1.000 | 3834 | tags=31%, list=19%, signal=38% |
| 775 | UV-4NQO\_FIBRO\_DN |  | 28 | 0.45 | 1.19 | 0.191 | 0.274 | 1.000 | 9259 | tags=61%, list=45%, signal=110% |
| 776 | CMV\_HCMV\_TIMECOURSE\_14HRS\_UP |  | 45 | 0.43 | 1.19 | 0.206 | 0.274 | 1.000 | 5929 | tags=49%, list=29%, signal=68% |
| 777 | HDACI\_COLON\_SUL16HRS\_UP |  | 42 | 0.43 | 1.19 | 0.222 | 0.275 | 1.000 | 2584 | tags=26%, list=13%, signal=30% |
| 778 | HOFFMANN\_BIVSBII\_LGBII |  | 101 | 0.40 | 1.19 | 0.116 | 0.275 | 1.000 | 8030 | tags=53%, list=39%, signal=87% |
| 779 | STEMPATHWAY |  | 15 | 0.51 | 1.19 | 0.232 | 0.275 | 1.000 | 3980 | tags=27%, list=19%, signal=33% |
| 780 | BHATTACHARYA\_ESC\_UP |  | 62 | 0.41 | 1.19 | 0.146 | 0.276 | 1.000 | 4293 | tags=34%, list=21%, signal=43% |
| 781 | CHIARETTI\_ZAP70\_DIFF |  | 67 | 0.41 | 1.19 | 0.164 | 0.278 | 1.000 | 5254 | tags=39%, list=25%, signal=52% |
| 782 | TGF\_BETA\_SIGNALING\_PATHWAY |  | 49 | 0.42 | 1.19 | 0.191 | 0.280 | 1.000 | 4632 | tags=35%, list=22%, signal=45% |
| 783 | CMV\_HCMV\_6HRS\_UP |  | 25 | 0.45 | 1.19 | 0.234 | 0.280 | 1.000 | 3431 | tags=36%, list=17%, signal=43% |
| 784 | BCNU\_GLIOMA\_NOMGMT\_48HRS\_UP |  | 18 | 0.48 | 1.19 | 0.249 | 0.281 | 1.000 | 4859 | tags=44%, list=24%, signal=58% |
| 785 | TENEDINI\_MEGAKARYOCYTIC\_GENES |  | 53 | 0.42 | 1.19 | 0.178 | 0.281 | 1.000 | 1881 | tags=19%, list=9%, signal=21% |
| 786 | IL1RPATHWAY |  | 31 | 0.44 | 1.19 | 0.225 | 0.281 | 1.000 | 7910 | tags=55%, list=38%, signal=89% |
| 787 | ELECTRON\_TRANSPORTER\_ACTIVITY |  | 113 | 0.39 | 1.19 | 0.123 | 0.284 | 1.000 | 5010 | tags=36%, list=24%, signal=48% |
| 788 | PAR1PATHWAY |  | 19 | 0.47 | 1.18 | 0.250 | 0.285 | 1.000 | 9903 | tags=74%, list=48%, signal=142% |
| 789 | ASTIER\_FN\_DIFF |  | 61 | 0.41 | 1.18 | 0.190 | 0.285 | 1.000 | 4434 | tags=33%, list=22%, signal=42% |
| 790 | HSC\_STHSC\_FETAL |  | 33 | 0.44 | 1.18 | 0.221 | 0.285 | 1.000 | 8359 | tags=61%, list=41%, signal=102% |
| 791 | CHEN\_HOXA5\_TARGETS\_UP |  | 229 | 0.38 | 1.18 | 0.047 | 0.286 | 1.000 | 6313 | tags=39%, list=31%, signal=56% |
| 792 | HSC\_STHSC\_SHARED |  | 33 | 0.44 | 1.18 | 0.220 | 0.289 | 1.000 | 8359 | tags=61%, list=41%, signal=102% |
| 793 | IRS1\_KO\_ADIP\_DN |  | 116 | 0.39 | 1.18 | 0.117 | 0.290 | 1.000 | 4581 | tags=34%, list=22%, signal=43% |
| 794 | ET743PT650\_COLONCA\_DN |  | 44 | 0.43 | 1.18 | 0.204 | 0.291 | 1.000 | 8045 | tags=61%, list=39%, signal=100% |
| 795 | HSC\_LTHSC\_ADULT |  | 360 | 0.38 | 1.18 | 0.025 | 0.292 | 1.000 | 7450 | tags=44%, list=36%, signal=67% |
| 796 | STEMCELL\_COMMON\_DN |  | 62 | 0.41 | 1.18 | 0.183 | 0.294 | 1.000 | 5901 | tags=37%, list=29%, signal=52% |
| 797 | MOREAUX\_TACI\_HI\_IN\_BMPC |  | 96 | 0.39 | 1.18 | 0.166 | 0.294 | 1.000 | 6463 | tags=41%, list=31%, signal=59% |
| 798 | SHEPARD\_CRASH\_AND\_BURN\_MUT\_VS\_WT\_DN |  | 155 | 0.39 | 1.18 | 0.085 | 0.293 | 1.000 | 5680 | tags=34%, list=28%, signal=46% |
| 799 | HDACI\_COLON\_SUL12HRS\_UP |  | 26 | 0.45 | 1.18 | 0.239 | 0.293 | 1.000 | 2807 | tags=31%, list=14%, signal=36% |
| 800 | ROSS\_CBF\_LEUKEMIA |  | 65 | 0.41 | 1.18 | 0.193 | 0.293 | 1.000 | 5399 | tags=40%, list=26%, signal=54% |
| 801 | IDX\_TSA\_UP\_CLUSTER2 |  | 60 | 0.41 | 1.18 | 0.179 | 0.295 | 1.000 | 4648 | tags=35%, list=23%, signal=45% |
| 802 | LINDSTEDT\_DEND\_8H\_VS\_48H\_DN |  | 67 | 0.41 | 1.18 | 0.181 | 0.297 | 1.000 | 2213 | tags=18%, list=11%, signal=20% |
| 803 | STRESS\_ARSENIC\_SPECIFIC\_DN |  | 27 | 0.45 | 1.18 | 0.238 | 0.298 | 1.000 | 7016 | tags=56%, list=34%, signal=84% |
| 804 | GN\_CAMP\_GRANULOSA\_UP |  | 53 | 0.41 | 1.17 | 0.213 | 0.298 | 1.000 | 5229 | tags=42%, list=25%, signal=55% |
| 805 | YAGI\_AML\_PROG\_FAB |  | 190 | 0.38 | 1.17 | 0.071 | 0.299 | 1.000 | 4923 | tags=33%, list=24%, signal=43% |
| 806 | CMV\_HCMV\_TIMECOURSE\_10HRS\_DN |  | 15 | 0.49 | 1.17 | 0.267 | 0.300 | 1.000 | 7816 | tags=60%, list=38%, signal=97% |
| 807 | WONG\_IFNA\_HCC\_RESISTANT\_VS\_SENSITIVE\_DN |  | 33 | 0.44 | 1.17 | 0.210 | 0.302 | 1.000 | 4480 | tags=36%, list=22%, signal=46% |
| 808 | EICOSANOID\_SYNTHESIS |  | 17 | 0.48 | 1.17 | 0.259 | 0.302 | 1.000 | 1589 | tags=18%, list=8%, signal=19% |
| 809 | GSK3PATHWAY |  | 26 | 0.45 | 1.17 | 0.246 | 0.304 | 1.000 | 8658 | tags=50%, list=42%, signal=86% |
| 810 | HTERT\_DN |  | 70 | 0.40 | 1.17 | 0.177 | 0.305 | 1.000 | 2902 | tags=23%, list=14%, signal=27% |
| 811 | ERKPATHWAY |  | 30 | 0.44 | 1.17 | 0.239 | 0.307 | 1.000 | 5218 | tags=37%, list=25%, signal=49% |
| 812 | SA\_PTEN\_PATHWAY |  | 17 | 0.48 | 1.17 | 0.260 | 0.307 | 1.000 | 9757 | tags=82%, list=47%, signal=156% |
| 813 | SHEPARD\_NEG\_REG\_OF\_CELL\_PROLIFERATION |  | 108 | 0.39 | 1.17 | 0.153 | 0.307 | 1.000 | 4009 | tags=26%, list=19%, signal=32% |
| 814 | HBX\_HCC\_DN |  | 22 | 0.46 | 1.17 | 0.246 | 0.308 | 1.000 | 4488 | tags=36%, list=22%, signal=46% |
| 815 | MUNSHI\_MM\_UP |  | 65 | 0.40 | 1.17 | 0.203 | 0.314 | 1.000 | 5797 | tags=46%, list=28%, signal=64% |
| 816 | IRITANI\_ADPROX\_LYMPH |  | 125 | 0.39 | 1.16 | 0.139 | 0.316 | 1.000 | 4918 | tags=32%, list=24%, signal=42% |
| 817 | UVC\_HIGH\_D5\_DN |  | 36 | 0.43 | 1.16 | 0.230 | 0.315 | 1.000 | 6456 | tags=44%, list=31%, signal=65% |
| 818 | VANTVEER\_BREAST\_OUTCOME\_GOOD\_VS\_POOR\_UP |  | 26 | 0.45 | 1.16 | 0.255 | 0.316 | 1.000 | 9074 | tags=62%, list=44%, signal=110% |
| 819 | AGED\_MOUSE\_CORTEX\_UP |  | 31 | 0.44 | 1.16 | 0.255 | 0.318 | 1.000 | 6994 | tags=58%, list=34%, signal=88% |
| 820 | PGC1APATHWAY |  | 23 | 0.46 | 1.16 | 0.270 | 0.318 | 1.000 | 8367 | tags=57%, list=41%, signal=95% |
| 821 | CELLCYCLEPATHWAY |  | 23 | 0.45 | 1.16 | 0.254 | 0.319 | 1.000 | 7525 | tags=57%, list=37%, signal=89% |
| 822 | UV-CMV\_UNIQUE\_HCMV\_6HRS\_DN |  | 82 | 0.40 | 1.16 | 0.186 | 0.318 | 1.000 | 6088 | tags=37%, list=30%, signal=52% |
| 823 | GH\_GHRHR\_KO\_6HRS\_UP |  | 69 | 0.40 | 1.16 | 0.207 | 0.319 | 1.000 | 3814 | tags=25%, list=19%, signal=30% |
| 824 | REFRACTORY\_GASTRIC\_UP |  | 87 | 0.39 | 1.16 | 0.177 | 0.319 | 1.000 | 8380 | tags=57%, list=41%, signal=96% |
| 825 | INNEREAR\_UP |  | 40 | 0.42 | 1.16 | 0.241 | 0.320 | 1.000 | 4029 | tags=33%, list=20%, signal=40% |
| 826 | HDACPATHWAY |  | 30 | 0.44 | 1.16 | 0.262 | 0.320 | 1.000 | 8006 | tags=47%, list=39%, signal=76% |
| 827 | TCELL\_ANERGIC\_UP |  | 81 | 0.39 | 1.16 | 0.183 | 0.322 | 1.000 | 3752 | tags=26%, list=18%, signal=32% |
| 828 | ZHAN\_MULTIPLE\_MYELOMA\_VS\_NORMAL\_DN |  | 39 | 0.42 | 1.16 | 0.236 | 0.322 | 1.000 | 5655 | tags=38%, list=27%, signal=53% |
| 829 | KANG\_TERT\_DN |  | 96 | 0.38 | 1.16 | 0.170 | 0.323 | 1.000 | 8058 | tags=42%, list=39%, signal=68% |
| 830 | HSC\_LTHSC\_FETAL |  | 268 | 0.37 | 1.16 | 0.064 | 0.324 | 1.000 | 8306 | tags=50%, list=40%, signal=82% |
| 831 | VIPPATHWAY |  | 27 | 0.44 | 1.16 | 0.257 | 0.324 | 1.000 | 7929 | tags=56%, list=38%, signal=90% |
| 832 | CMV\_8HRS\_DN |  | 44 | 0.42 | 1.16 | 0.235 | 0.324 | 1.000 | 5116 | tags=45%, list=25%, signal=60% |
| 833 | UVB\_NHEK3\_C8 |  | 66 | 0.40 | 1.16 | 0.227 | 0.324 | 1.000 | 4466 | tags=30%, list=22%, signal=39% |
| 834 | MAPK\_CASCADE |  | 29 | 0.44 | 1.16 | 0.249 | 0.324 | 1.000 | 9757 | tags=72%, list=47%, signal=137% |
| 835 | UVC\_XPCS\_ALL\_UP |  | 61 | 0.40 | 1.16 | 0.211 | 0.326 | 1.000 | 2924 | tags=26%, list=14%, signal=30% |
| 836 | CMV-UV\_HCMV\_6HRS\_UP |  | 120 | 0.38 | 1.16 | 0.133 | 0.326 | 1.000 | 3782 | tags=24%, list=18%, signal=29% |
| 837 | 41BBPATHWAY |  | 18 | 0.47 | 1.16 | 0.276 | 0.327 | 1.000 | 7714 | tags=56%, list=37%, signal=89% |
| 838 | HSC\_LTHSC\_SHARED |  | 268 | 0.37 | 1.16 | 0.062 | 0.327 | 1.000 | 8306 | tags=50%, list=40%, signal=82% |
| 839 | AGED\_MOUSE\_HIPPOCAMPUS\_ANY\_UP |  | 45 | 0.41 | 1.15 | 0.235 | 0.327 | 1.000 | 2090 | tags=18%, list=10%, signal=20% |
| 840 | MUNSHI\_MM\_VS\_PCS\_UP |  | 77 | 0.39 | 1.15 | 0.201 | 0.327 | 1.000 | 4846 | tags=36%, list=24%, signal=47% |
| 841 | HSC\_HSC\_FETAL |  | 237 | 0.37 | 1.15 | 0.088 | 0.330 | 1.000 | 7256 | tags=46%, list=35%, signal=70% |
| 842 | TPA\_RESIST\_LATE\_UP |  | 37 | 0.42 | 1.15 | 0.251 | 0.330 | 1.000 | 6406 | tags=41%, list=31%, signal=59% |
| 843 | TSADAC\_HYPOMETH\_HYPERAC\_OVCA\_UP |  | 16 | 0.47 | 1.15 | 0.290 | 0.330 | 1.000 | 8428 | tags=63%, list=41%, signal=106% |
| 844 | GAY\_YY1\_DN |  | 243 | 0.37 | 1.15 | 0.086 | 0.331 | 1.000 | 4860 | tags=31%, list=24%, signal=40% |
| 845 | BLEO\_MOUSE\_LYMPH\_LOW\_24HRS\_DN |  | 27 | 0.44 | 1.15 | 0.270 | 0.331 | 1.000 | 7583 | tags=63%, list=37%, signal=99% |
| 846 | LU\_IL4BCELL |  | 66 | 0.40 | 1.15 | 0.230 | 0.335 | 1.000 | 7010 | tags=41%, list=34%, signal=62% |
| 847 | ST\_MYOCYTE\_AD\_PATHWAY |  | 24 | 0.44 | 1.15 | 0.270 | 0.334 | 1.000 | 7079 | tags=42%, list=34%, signal=63% |
| 848 | TARTE\_MATURE\_PC |  | 387 | 0.37 | 1.15 | 0.047 | 0.335 | 1.000 | 4883 | tags=30%, list=24%, signal=38% |
| 849 | HSC\_HSC\_ADULT |  | 299 | 0.37 | 1.15 | 0.054 | 0.335 | 1.000 | 4521 | tags=30%, list=22%, signal=38% |
| 850 | GAMMA\_UNIQUE\_FIBRO\_DN |  | 60 | 0.40 | 1.15 | 0.226 | 0.336 | 1.000 | 5144 | tags=37%, list=25%, signal=49% |
| 851 | BRCA\_PROGNOSIS\_NEG |  | 95 | 0.38 | 1.15 | 0.193 | 0.339 | 1.000 | 4918 | tags=35%, list=24%, signal=45% |
| 852 | CMV\_HCMV\_TIMECOURSE\_24HRS\_UP |  | 72 | 0.39 | 1.15 | 0.209 | 0.339 | 1.000 | 4855 | tags=36%, list=24%, signal=47% |
| 853 | TOLLPATHWAY |  | 33 | 0.42 | 1.15 | 0.274 | 0.339 | 1.000 | 7910 | tags=55%, list=38%, signal=88% |
| 854 | SANSOM\_APC\_4\_DN |  | 68 | 0.40 | 1.15 | 0.218 | 0.340 | 1.000 | 5132 | tags=34%, list=25%, signal=45% |
| 855 | SIG\_IL4RECEPTOR\_IN\_B\_LYPHOCYTES |  | 27 | 0.44 | 1.15 | 0.289 | 0.340 | 1.000 | 9757 | tags=78%, list=47%, signal=148% |
| 856 | DAVIES\_N |  | 19 | 0.47 | 1.15 | 0.281 | 0.340 | 1.000 | 3789 | tags=32%, list=18%, signal=39% |
| 857 | BIOPEPTIDESPATHWAY |  | 38 | 0.41 | 1.15 | 0.253 | 0.340 | 1.000 | 9757 | tags=61%, list=47%, signal=115% |
| 858 | G\_PROTEIN\_SIGNALING |  | 91 | 0.38 | 1.15 | 0.198 | 0.340 | 1.000 | 8077 | tags=51%, list=39%, signal=83% |
| 859 | CMV\_HCMV\_TIMECOURSE\_1HR\_DN |  | 38 | 0.42 | 1.15 | 0.277 | 0.340 | 1.000 | 9159 | tags=63%, list=44%, signal=113% |
| 860 | CALCINEURINPATHWAY |  | 19 | 0.45 | 1.14 | 0.307 | 0.341 | 1.000 | 7929 | tags=47%, list=38%, signal=77% |
| 861 | BASSO\_HCL\_DIFF |  | 86 | 0.39 | 1.14 | 0.206 | 0.342 | 1.000 | 4635 | tags=30%, list=22%, signal=39% |
| 862 | ASTIER\_BCELL |  | 60 | 0.40 | 1.14 | 0.237 | 0.342 | 1.000 | 4434 | tags=32%, list=22%, signal=40% |
| 863 | IDX\_TSA\_UP\_CLUSTER4 |  | 40 | 0.41 | 1.14 | 0.261 | 0.342 | 1.000 | 4125 | tags=25%, list=20%, signal=31% |
| 864 | PROLIFERATION\_GENES |  | 363 | 0.37 | 1.14 | 0.066 | 0.342 | 1.000 | 4009 | tags=24%, list=19%, signal=29% |
| 865 | GENOTOXINS\_ALL\_4HRS\_REG |  | 26 | 0.43 | 1.14 | 0.286 | 0.343 | 1.000 | 4814 | tags=42%, list=23%, signal=55% |
| 866 | ACTINYPATHWAY |  | 17 | 0.47 | 1.14 | 0.306 | 0.346 | 1.000 | 3918 | tags=47%, list=19%, signal=58% |
| 867 | BCNU\_GLIOMA\_NOMGMT\_48HRS\_DN |  | 31 | 0.43 | 1.14 | 0.281 | 0.346 | 1.000 | 2638 | tags=26%, list=13%, signal=30% |
| 868 | UREA\_CYCLE\_AND\_METABOLISM\_OF\_AMINO\_GROUPS |  | 18 | 0.47 | 1.14 | 0.284 | 0.346 | 1.000 | 4931 | tags=44%, list=24%, signal=58% |
| 869 | VERHAAK\_AML\_NPM1\_MUT\_VS\_WT\_DN |  | 258 | 0.37 | 1.14 | 0.101 | 0.348 | 1.000 | 5696 | tags=32%, list=28%, signal=43% |
| 870 | PENG\_LEUCINE\_DN |  | 139 | 0.37 | 1.14 | 0.165 | 0.347 | 1.000 | 4543 | tags=34%, list=22%, signal=43% |
| 871 | BCL2\_FAMILY\_AND\_REG\_NETWORK |  | 21 | 0.45 | 1.14 | 0.294 | 0.348 | 1.000 | 6236 | tags=57%, list=30%, signal=82% |
| 872 | SIG\_INSULIN\_RECEPTOR\_PATHWAY\_IN\_CARDIAC\_MYOCYTES |  | 51 | 0.41 | 1.14 | 0.253 | 0.350 | 1.000 | 6236 | tags=45%, list=30%, signal=65% |
| 873 | INSULIN\_ADIP\_SENS\_DN |  | 17 | 0.47 | 1.14 | 0.314 | 0.350 | 1.000 | 3984 | tags=47%, list=19%, signal=58% |
| 874 | AGUIRRE\_PANCREAS\_CHR9 |  | 24 | 0.44 | 1.14 | 0.301 | 0.353 | 1.000 | 9492 | tags=79%, list=46%, signal=147% |
| 875 | ROME\_INSULIN\_2F\_DN |  | 25 | 0.44 | 1.13 | 0.309 | 0.357 | 1.000 | 4658 | tags=32%, list=23%, signal=41% |
| 876 | OXSTRESS\_BREASTCA\_UP |  | 29 | 0.42 | 1.13 | 0.300 | 0.357 | 1.000 | 4353 | tags=34%, list=21%, signal=44% |
| 877 | IL2RBPATHWAY |  | 34 | 0.41 | 1.13 | 0.274 | 0.358 | 1.000 | 8587 | tags=62%, list=42%, signal=106% |
| 878 | CPR\_NULL\_LIVER\_DN |  | 17 | 0.46 | 1.13 | 0.300 | 0.357 | 1.000 | 8245 | tags=71%, list=40%, signal=118% |
| 879 | HSC\_HSC\_SHARED |  | 223 | 0.37 | 1.13 | 0.126 | 0.359 | 1.000 | 4502 | tags=30%, list=22%, signal=38% |
| 880 | NOVA2\_KO\_SPLICING |  | 42 | 0.41 | 1.13 | 0.289 | 0.359 | 1.000 | 7339 | tags=45%, list=36%, signal=70% |
| 881 | CMV\_HCMV\_TIMECOURSE\_48HRS\_DN |  | 110 | 0.38 | 1.13 | 0.203 | 0.360 | 1.000 | 4329 | tags=27%, list=21%, signal=34% |
| 882 | UV-CMV\_UNIQUE\_HCMV\_6HRS\_UP |  | 102 | 0.38 | 1.13 | 0.219 | 0.359 | 1.000 | 3199 | tags=21%, list=16%, signal=24% |
| 883 | TYPE\_III\_SECRETION\_SYSTEM |  | 21 | 0.44 | 1.13 | 0.318 | 0.360 | 1.000 | 4710 | tags=33%, list=23%, signal=43% |
| 884 | ROSS\_CBF |  | 82 | 0.38 | 1.13 | 0.238 | 0.360 | 1.000 | 5399 | tags=38%, list=26%, signal=51% |
| 885 | BRENTANI\_TRANSPORT\_OF\_VESICLES |  | 24 | 0.44 | 1.13 | 0.291 | 0.360 | 1.000 | 5230 | tags=46%, list=25%, signal=61% |
| 886 | ATMPATHWAY |  | 19 | 0.46 | 1.13 | 0.310 | 0.360 | 1.000 | 2402 | tags=26%, list=12%, signal=30% |
| 887 | PENG\_GLUTAMINE\_DN |  | 248 | 0.36 | 1.13 | 0.102 | 0.362 | 1.000 | 6054 | tags=41%, list=29%, signal=58% |
| 888 | HIPPOCAMPUS\_DEVELOPMENT\_NEONATAL |  | 27 | 0.43 | 1.13 | 0.313 | 0.362 | 1.000 | 3717 | tags=30%, list=18%, signal=36% |
| 889 | TPA\_RESIST\_EARLY\_DN |  | 74 | 0.39 | 1.13 | 0.242 | 0.363 | 1.000 | 4855 | tags=35%, list=24%, signal=46% |
| 890 | BREAST\_CANCER\_ESTROGEN\_SIGNALING |  | 93 | 0.38 | 1.13 | 0.247 | 0.363 | 1.000 | 3957 | tags=30%, list=19%, signal=37% |
| 891 | SHEPARD\_POS\_REG\_OF\_CELL\_PROLIFERATION |  | 93 | 0.38 | 1.13 | 0.214 | 0.366 | 1.000 | 7009 | tags=38%, list=34%, signal=57% |
| 892 | HIF1\_TARGETS |  | 36 | 0.42 | 1.13 | 0.294 | 0.366 | 1.000 | 3398 | tags=28%, list=16%, signal=33% |
| 893 | P53PATHWAY |  | 16 | 0.47 | 1.13 | 0.314 | 0.368 | 1.000 | 5626 | tags=50%, list=27%, signal=69% |
| 894 | HUMAN\_TISSUE\_PANCREAS |  | 40 | 0.41 | 1.13 | 0.288 | 0.367 | 1.000 | 7462 | tags=45%, list=36%, signal=70% |
| 895 | G2PATHWAY |  | 23 | 0.44 | 1.13 | 0.329 | 0.370 | 1.000 | 3984 | tags=35%, list=19%, signal=43% |
| 896 | RHOPATHWAY |  | 29 | 0.42 | 1.12 | 0.313 | 0.373 | 1.000 | 8346 | tags=69%, list=41%, signal=116% |
| 897 | UVC\_TTD-XPCS\_COMMON\_DN |  | 144 | 0.37 | 1.12 | 0.197 | 0.374 | 1.000 | 9101 | tags=62%, list=44%, signal=110% |
| 898 | AGUIRRE\_PANCREAS\_CHR7 |  | 48 | 0.40 | 1.12 | 0.296 | 0.374 | 1.000 | 7330 | tags=50%, list=36%, signal=77% |
| 899 | DOX\_RESIST\_GASTRIC\_UP |  | 44 | 0.40 | 1.12 | 0.295 | 0.376 | 1.000 | 6910 | tags=45%, list=34%, signal=68% |
| 900 | HPV31\_DN |  | 47 | 0.40 | 1.12 | 0.287 | 0.377 | 1.000 | 1401 | tags=19%, list=7%, signal=20% |
| 901 | INFLAMPATHWAY |  | 29 | 0.43 | 1.12 | 0.290 | 0.377 | 1.000 | 4173 | tags=24%, list=20%, signal=30% |
| 902 | HDACI\_COLON\_CUR12HRS\_UP |  | 21 | 0.44 | 1.12 | 0.312 | 0.377 | 1.000 | 3327 | tags=29%, list=16%, signal=34% |
| 903 | IFN\_BETA\_GLIOMA\_UP |  | 63 | 0.39 | 1.12 | 0.266 | 0.377 | 1.000 | 4707 | tags=30%, list=23%, signal=39% |
| 904 | CXCR4PATHWAY |  | 24 | 0.44 | 1.12 | 0.318 | 0.377 | 1.000 | 4167 | tags=33%, list=20%, signal=42% |
| 905 | FLAGELLAR\_ASSEMBLY |  | 21 | 0.44 | 1.12 | 0.335 | 0.377 | 1.000 | 4710 | tags=33%, list=23%, signal=43% |
| 906 | CARIES\_PULP\_UP |  | 205 | 0.36 | 1.12 | 0.177 | 0.378 | 1.000 | 1426 | tags=12%, list=7%, signal=12% |
| 907 | PRMT5\_KD\_DN |  | 25 | 0.43 | 1.12 | 0.308 | 0.378 | 1.000 | 3331 | tags=24%, list=16%, signal=29% |
| 908 | SIG\_PIP3\_SIGNALING\_IN\_B\_LYMPHOCYTES |  | 35 | 0.41 | 1.12 | 0.305 | 0.378 | 1.000 | 6236 | tags=40%, list=30%, signal=57% |
| 909 | PTDINSPATHWAY |  | 22 | 0.44 | 1.12 | 0.326 | 0.379 | 1.000 | 6236 | tags=45%, list=30%, signal=65% |
| 910 | AGED\_MOUSE\_RETINA\_ANY\_UP |  | 21 | 0.44 | 1.12 | 0.322 | 0.381 | 1.000 | 7860 | tags=52%, list=38%, signal=85% |
| 911 | P53\_BRCA1\_UP |  | 31 | 0.42 | 1.12 | 0.319 | 0.384 | 1.000 | 4399 | tags=39%, list=21%, signal=49% |
| 912 | ATP\_SYNTHESIS |  | 21 | 0.44 | 1.11 | 0.328 | 0.387 | 1.000 | 4710 | tags=33%, list=23%, signal=43% |
| 913 | CMV\_HCMV\_TIMECOURSE\_18HRS\_UP |  | 74 | 0.38 | 1.11 | 0.283 | 0.389 | 1.000 | 6279 | tags=43%, list=30%, signal=62% |
| 914 | ST\_GA12\_PATHWAY |  | 21 | 0.45 | 1.11 | 0.327 | 0.389 | 1.000 | 8593 | tags=67%, list=42%, signal=114% |
| 915 | GREENBAUM\_E2A\_UP |  | 33 | 0.41 | 1.11 | 0.319 | 0.390 | 1.000 | 4979 | tags=33%, list=24%, signal=44% |
| 916 | MOREAUX\_TACI\_HI\_VS\_LOW\_UP |  | 413 | 0.35 | 1.11 | 0.104 | 0.391 | 1.000 | 7097 | tags=42%, list=34%, signal=62% |
| 917 | CHIARETTI\_T\_ALL\_DIFF |  | 278 | 0.36 | 1.11 | 0.152 | 0.394 | 1.000 | 2588 | tags=19%, list=13%, signal=22% |
| 918 | HOX\_GENES |  | 54 | 0.39 | 1.11 | 0.299 | 0.394 | 1.000 | 7236 | tags=44%, list=35%, signal=68% |
| 919 | BLOOD\_CLOTTING\_CASCADE |  | 20 | 0.44 | 1.11 | 0.344 | 0.394 | 1.000 | 3043 | tags=20%, list=15%, signal=23% |
| 920 | IGF1PATHWAY |  | 20 | 0.44 | 1.11 | 0.332 | 0.394 | 1.000 | 8147 | tags=60%, list=40%, signal=99% |
| 921 | HDACI\_COLON\_CUR24HRS\_UP |  | 37 | 0.40 | 1.11 | 0.326 | 0.394 | 1.000 | 4706 | tags=38%, list=23%, signal=49% |
| 922 | PYK2PATHWAY |  | 28 | 0.42 | 1.11 | 0.342 | 0.395 | 1.000 | 9946 | tags=71%, list=48%, signal=138% |
| 923 | INTEGRINPATHWAY |  | 34 | 0.41 | 1.11 | 0.316 | 0.396 | 1.000 | 5309 | tags=47%, list=26%, signal=63% |
| 924 | HDACI\_COLON\_SUL\_UP |  | 131 | 0.37 | 1.11 | 0.226 | 0.396 | 1.000 | 3972 | tags=28%, list=19%, signal=35% |
| 925 | HOGERKORP\_CD44\_DN |  | 22 | 0.44 | 1.11 | 0.336 | 0.395 | 1.000 | 4209 | tags=32%, list=20%, signal=40% |
| 926 | HYPERTROPHY\_MODEL |  | 20 | 0.44 | 1.11 | 0.345 | 0.396 | 1.000 | 1824 | tags=20%, list=9%, signal=22% |
| 927 | ZHAN\_MMPC\_PC |  | 22 | 0.43 | 1.11 | 0.341 | 0.397 | 1.000 | 7613 | tags=41%, list=37%, signal=65% |
| 928 | P53\_SIGNALING |  | 93 | 0.37 | 1.11 | 0.292 | 0.399 | 1.000 | 6975 | tags=49%, list=34%, signal=74% |
| 929 | HDACI\_COLON\_SUL48HRS\_UP |  | 90 | 0.37 | 1.10 | 0.279 | 0.403 | 1.000 | 3972 | tags=29%, list=19%, signal=36% |
| 930 | TPA\_RESIST\_MIDDLE\_DN |  | 108 | 0.37 | 1.10 | 0.279 | 0.403 | 1.000 | 4855 | tags=35%, list=24%, signal=46% |
| 931 | LIZUKA\_G1\_SM\_G2 |  | 25 | 0.42 | 1.10 | 0.333 | 0.406 | 1.000 | 6804 | tags=48%, list=33%, signal=72% |
| 932 | HOFFMANN\_BIVSBII\_BI |  | 97 | 0.37 | 1.10 | 0.272 | 0.411 | 1.000 | 8441 | tags=53%, list=41%, signal=89% |
| 933 | HYPOPHYSECTOMY\_RAT\_DN |  | 49 | 0.39 | 1.10 | 0.333 | 0.411 | 1.000 | 2976 | tags=33%, list=14%, signal=38% |
| 934 | BASSO\_REGULATORY\_HUBS |  | 138 | 0.36 | 1.10 | 0.266 | 0.412 | 1.000 | 6998 | tags=45%, list=34%, signal=68% |
| 935 | MYOD\_NIH3T3\_UP |  | 82 | 0.37 | 1.10 | 0.290 | 0.417 | 1.000 | 1726 | tags=18%, list=8%, signal=20% |
| 936 | CIS\_XPC\_UP |  | 149 | 0.36 | 1.10 | 0.251 | 0.418 | 1.000 | 5115 | tags=34%, list=25%, signal=45% |
| 937 | STRESS\_IONIZING\_SPECIFIC\_UP |  | 15 | 0.46 | 1.10 | 0.363 | 0.420 | 1.000 | 3560 | tags=40%, list=17%, signal=48% |
| 938 | UVC\_LOW\_ALL\_UP |  | 19 | 0.44 | 1.09 | 0.364 | 0.423 | 1.000 | 1194 | tags=21%, list=6%, signal=22% |
| 939 | KERATINOCYTEPATHWAY |  | 43 | 0.39 | 1.09 | 0.346 | 0.428 | 1.000 | 10087 | tags=70%, list=49%, signal=136% |
| 940 | DOX\_RESIST\_GASTRIC\_DN |  | 17 | 0.45 | 1.09 | 0.355 | 0.431 | 1.000 | 4316 | tags=41%, list=21%, signal=52% |
| 941 | STANELLE\_E2F1\_UP |  | 28 | 0.41 | 1.09 | 0.369 | 0.431 | 1.000 | 8942 | tags=57%, list=43%, signal=101% |
| 942 | TNFR2PATHWAY |  | 18 | 0.44 | 1.09 | 0.382 | 0.435 | 1.000 | 7714 | tags=61%, list=37%, signal=98% |
| 943 | ROTH\_HTERT\_DIFF |  | 31 | 0.41 | 1.09 | 0.360 | 0.438 | 1.000 | 7548 | tags=52%, list=37%, signal=81% |
| 944 | CITED1\_KO\_HET\_DN |  | 30 | 0.41 | 1.09 | 0.362 | 0.439 | 1.000 | 4075 | tags=33%, list=20%, signal=41% |
| 945 | CMV\_UV-CMV\_COMMON\_HCMV\_6HRS\_UP |  | 20 | 0.43 | 1.09 | 0.380 | 0.441 | 1.000 | 3431 | tags=35%, list=17%, signal=42% |
| 946 | AGEING\_BRAIN\_DN |  | 120 | 0.36 | 1.08 | 0.303 | 0.442 | 1.000 | 8396 | tags=52%, list=41%, signal=88% |
| 947 | GLUTAMATE\_METABOLISM |  | 24 | 0.41 | 1.08 | 0.384 | 0.448 | 1.000 | 2681 | tags=25%, list=13%, signal=29% |
| 948 | PARK\_RARALPHA\_UP |  | 38 | 0.39 | 1.08 | 0.352 | 0.448 | 1.000 | 3137 | tags=24%, list=15%, signal=28% |
| 949 | NUMATA\_G\_CSF\_DIFF |  | 18 | 0.44 | 1.08 | 0.387 | 0.448 | 1.000 | 6825 | tags=56%, list=33%, signal=83% |
| 950 | GAY\_YY1\_UP |  | 195 | 0.35 | 1.08 | 0.258 | 0.449 | 1.000 | 4446 | tags=26%, list=22%, signal=33% |
| 951 | LVAD\_HEARTFAILURE\_UP |  | 89 | 0.36 | 1.08 | 0.323 | 0.450 | 1.000 | 3958 | tags=25%, list=19%, signal=30% |
| 952 | HYPOXIA\_FIBRO\_UP |  | 20 | 0.43 | 1.08 | 0.369 | 0.452 | 1.000 | 3398 | tags=30%, list=16%, signal=36% |
| 953 | GH\_GHRHR\_KO\_24HRS\_UP |  | 142 | 0.36 | 1.08 | 0.290 | 0.455 | 1.000 | 3860 | tags=25%, list=19%, signal=31% |
| 954 | FETAL\_LIVER\_VS\_ADULT\_LIVER\_GNF2 |  | 70 | 0.37 | 1.08 | 0.345 | 0.455 | 1.000 | 6847 | tags=37%, list=33%, signal=55% |
| 955 | LIZUKA\_L1\_GR\_G1 |  | 20 | 0.43 | 1.08 | 0.362 | 0.456 | 1.000 | 1647 | tags=20%, list=8%, signal=22% |
| 956 | SANSOM\_APC\_5\_DN |  | 327 | 0.34 | 1.07 | 0.220 | 0.462 | 1.000 | 5369 | tags=32%, list=26%, signal=42% |
| 957 | ADIP\_DIFF\_UP |  | 67 | 0.37 | 1.07 | 0.361 | 0.463 | 1.000 | 5072 | tags=42%, list=25%, signal=55% |
| 958 | TH1TH2PATHWAY |  | 17 | 0.44 | 1.07 | 0.399 | 0.464 | 1.000 | 1158 | tags=12%, list=6%, signal=12% |
| 959 | HDACI\_COLON\_CLUSTER7 |  | 15 | 0.45 | 1.07 | 0.407 | 0.465 | 1.000 | 4591 | tags=33%, list=22%, signal=43% |
| 960 | SA\_B\_CELL\_RECEPTOR\_COMPLEXES |  | 24 | 0.41 | 1.07 | 0.379 | 0.470 | 1.000 | 4534 | tags=38%, list=22%, signal=48% |
| 961 | TPA\_RESIST\_EARLY\_UP |  | 30 | 0.40 | 1.07 | 0.397 | 0.470 | 1.000 | 7279 | tags=43%, list=35%, signal=67% |
| 962 | CMV\_HCMV\_TIMECOURSE\_16HRS\_UP |  | 57 | 0.37 | 1.07 | 0.384 | 0.470 | 1.000 | 5516 | tags=37%, list=27%, signal=50% |
| 963 | HOFMANN\_MANTEL\_LYMPHOMA\_VS\_LYMPH\_NODES\_DN |  | 39 | 0.39 | 1.07 | 0.387 | 0.473 | 1.000 | 7581 | tags=54%, list=37%, signal=85% |
| 964 | STRESS\_GENOTOXIC\_SPECIFIC\_UP |  | 34 | 0.40 | 1.07 | 0.397 | 0.473 | 1.000 | 4571 | tags=38%, list=22%, signal=49% |
| 965 | IFN\_BETA\_GLIOMA\_DN |  | 44 | 0.38 | 1.07 | 0.392 | 0.478 | 1.000 | 4978 | tags=41%, list=24%, signal=54% |
| 966 | WILLERT\_WNT\_NCCIT\_ALL\_UP |  | 23 | 0.41 | 1.06 | 0.406 | 0.481 | 1.000 | 5428 | tags=35%, list=26%, signal=47% |
| 967 | ST\_PHOSPHOINOSITIDE\_3\_KINASE\_PATHWAY |  | 35 | 0.39 | 1.06 | 0.398 | 0.488 | 1.000 | 6236 | tags=49%, list=30%, signal=70% |
| 968 | ADIP\_DIFF\_CLUSTER3 |  | 33 | 0.39 | 1.06 | 0.400 | 0.488 | 1.000 | 4295 | tags=30%, list=21%, signal=38% |
| 969 | BCNU\_GLIOMA\_MGMT\_48HRS\_DN |  | 158 | 0.35 | 1.06 | 0.345 | 0.490 | 1.000 | 3289 | tags=20%, list=16%, signal=24% |
| 970 | PARK\_RARALPHA\_MOD |  | 61 | 0.37 | 1.06 | 0.379 | 0.491 | 1.000 | 3317 | tags=28%, list=16%, signal=33% |
| 971 | PHOSPHATIDYLINOSITOL\_SIGNALING\_SYSTEM |  | 90 | 0.36 | 1.06 | 0.361 | 0.490 | 1.000 | 9183 | tags=58%, list=45%, signal=104% |
| 972 | GH\_HYPOPHYSECTOMY\_RAT\_DN |  | 15 | 0.44 | 1.06 | 0.408 | 0.498 | 1.000 | 4009 | tags=33%, list=19%, signal=41% |
| 973 | SA\_TRKA\_RECEPTOR |  | 16 | 0.44 | 1.06 | 0.413 | 0.500 | 1.000 | 7688 | tags=50%, list=37%, signal=80% |
| 974 | CDMACPATHWAY |  | 16 | 0.43 | 1.05 | 0.410 | 0.501 | 1.000 | 3015 | tags=25%, list=15%, signal=29% |
| 975 | NUCLEAR\_RECEPTORS |  | 40 | 0.38 | 1.05 | 0.411 | 0.504 | 1.000 | 9687 | tags=65%, list=47%, signal=122% |
| 976 | CYTOKINEPATHWAY |  | 20 | 0.42 | 1.05 | 0.421 | 0.505 | 1.000 | 2716 | tags=15%, list=13%, signal=17% |
| 977 | EDG1PATHWAY |  | 25 | 0.41 | 1.05 | 0.426 | 0.506 | 1.000 | 6689 | tags=40%, list=32%, signal=59% |
| 978 | TALL1PATHWAY |  | 15 | 0.44 | 1.05 | 0.430 | 0.509 | 1.000 | 8979 | tags=53%, list=44%, signal=94% |
| 979 | ALKPATHWAY |  | 33 | 0.39 | 1.05 | 0.432 | 0.509 | 1.000 | 3653 | tags=27%, list=18%, signal=33% |
| 980 | COCAINE\_BRAIN\_5D\_UP |  | 63 | 0.37 | 1.05 | 0.403 | 0.511 | 1.000 | 6021 | tags=35%, list=29%, signal=49% |
| 981 | CAMPTOTHECIN\_PROBCELL\_DN |  | 30 | 0.40 | 1.05 | 0.412 | 0.514 | 1.000 | 5854 | tags=47%, list=28%, signal=65% |
| 982 | OLDWERNER\_FIBRO\_UP |  | 25 | 0.40 | 1.05 | 0.425 | 0.515 | 1.000 | 4440 | tags=28%, list=22%, signal=36% |
| 983 | TPA\_RESIST\_LATE\_DN |  | 63 | 0.36 | 1.05 | 0.407 | 0.515 | 1.000 | 8206 | tags=51%, list=40%, signal=84% |
| 984 | HALMOS\_CEBP\_DN |  | 43 | 0.37 | 1.05 | 0.425 | 0.516 | 1.000 | 3237 | tags=21%, list=16%, signal=25% |
| 985 | AGED\_RHESUS\_UP |  | 198 | 0.34 | 1.05 | 0.360 | 0.516 | 1.000 | 3752 | tags=21%, list=18%, signal=26% |
| 986 | GHPATHWAY |  | 27 | 0.40 | 1.04 | 0.439 | 0.521 | 1.000 | 10524 | tags=67%, list=51%, signal=136% |
| 987 | AGED\_MOUSE\_HIPPOCAMPUS\_ANY\_DN |  | 42 | 0.38 | 1.04 | 0.414 | 0.522 | 1.000 | 7168 | tags=43%, list=35%, signal=66% |
| 988 | GOLDRATH\_CELLCYCLE |  | 28 | 0.40 | 1.04 | 0.434 | 0.526 | 1.000 | 4552 | tags=32%, list=22%, signal=41% |
| 989 | TAKEDA\_NUP8\_HOXA9\_16D\_DN |  | 228 | 0.34 | 1.04 | 0.380 | 0.525 | 1.000 | 7713 | tags=43%, list=37%, signal=69% |
| 990 | TAKEDA\_NUP8\_HOXA9\_16D\_UP |  | 172 | 0.34 | 1.04 | 0.382 | 0.525 | 1.000 | 5528 | tags=30%, list=27%, signal=40% |
| 991 | AGED\_MOUSE\_HYPOTH\_UP |  | 44 | 0.37 | 1.04 | 0.434 | 0.525 | 1.000 | 5834 | tags=36%, list=28%, signal=51% |
| 992 | GO\_ROS |  | 26 | 0.40 | 1.04 | 0.439 | 0.525 | 1.000 | 7251 | tags=42%, list=35%, signal=65% |
| 993 | CHIARETTI\_T\_ALL |  | 255 | 0.33 | 1.04 | 0.366 | 0.534 | 1.000 | 2588 | tags=18%, list=13%, signal=20% |
| 994 | FMLPPATHWAY |  | 35 | 0.38 | 1.04 | 0.436 | 0.534 | 1.000 | 8147 | tags=40%, list=40%, signal=66% |
| 995 | MITOCHONDRIAL\_FATTY\_ACID\_BETAOXIDATION |  | 16 | 0.43 | 1.04 | 0.442 | 0.534 | 1.000 | 1397 | tags=31%, list=7%, signal=33% |
| 996 | GLEEVECPATHWAY |  | 22 | 0.41 | 1.04 | 0.447 | 0.536 | 1.000 | 3723 | tags=27%, list=18%, signal=33% |
| 997 | LEE\_TCELLS7\_UP |  | 15 | 0.43 | 1.04 | 0.459 | 0.538 | 1.000 | 4820 | tags=40%, list=23%, signal=52% |
| 998 | CELL\_ADHESION |  | 173 | 0.34 | 1.03 | 0.405 | 0.539 | 1.000 | 8343 | tags=42%, list=40%, signal=69% |
| 999 | ADIPOGENESIS\_HMSC\_CLASS3\_UP |  | 66 | 0.35 | 1.03 | 0.437 | 0.542 | 1.000 | 3147 | tags=27%, list=15%, signal=32% |
| 1000 | AGUIRRE\_PANCREAS\_CHR18 |  | 17 | 0.42 | 1.03 | 0.457 | 0.542 | 1.000 | 7150 | tags=59%, list=35%, signal=90% |
| 1001 | CMV-UV\_HCMV\_6HRS\_DN |  | 108 | 0.35 | 1.03 | 0.444 | 0.542 | 1.000 | 5086 | tags=32%, list=25%, signal=43% |
| 1002 | PPARAPATHWAY |  | 54 | 0.36 | 1.03 | 0.447 | 0.548 | 1.000 | 8245 | tags=54%, list=40%, signal=89% |
| 1003 | TYROSINE\_METABOLISM |  | 31 | 0.38 | 1.03 | 0.486 | 0.549 | 1.000 | 1777 | tags=13%, list=9%, signal=14% |
| 1004 | ARGININE\_AND\_PROLINE\_METABOLISM |  | 42 | 0.37 | 1.03 | 0.462 | 0.548 | 1.000 | 5730 | tags=36%, list=28%, signal=49% |
| 1005 | CELL\_SURFACE\_RECEPTOR\_LINKED\_SIGNAL\_TRANSDUCTION |  | 130 | 0.34 | 1.03 | 0.453 | 0.558 | 1.000 | 5986 | tags=25%, list=29%, signal=36% |
| 1006 | HOFMANN\_MDS\_CD34\_LOW\_AND\_HIGH\_RISK |  | 46 | 0.36 | 1.02 | 0.455 | 0.562 | 1.000 | 5269 | tags=37%, list=26%, signal=50% |
| 1007 | SA\_CASPASE\_CASCADE |  | 16 | 0.43 | 1.02 | 0.450 | 0.561 | 1.000 | 5367 | tags=44%, list=26%, signal=59% |
| 1008 | ZHAN\_MMPC\_LATEVS |  | 45 | 0.36 | 1.02 | 0.455 | 0.562 | 1.000 | 3064 | tags=22%, list=15%, signal=26% |
| 1009 | TPA\_SKIN\_DN |  | 16 | 0.42 | 1.02 | 0.459 | 0.562 | 1.000 | 654 | tags=13%, list=3%, signal=13% |
| 1010 | LAL\_KO\_6MO\_UP |  | 61 | 0.36 | 1.02 | 0.478 | 0.566 | 1.000 | 1009 | tags=8%, list=5%, signal=9% |
| 1011 | CELL\_PROLIFERATION |  | 203 | 0.33 | 1.02 | 0.439 | 0.566 | 1.000 | 4590 | tags=26%, list=22%, signal=33% |
| 1012 | SPPAPATHWAY |  | 20 | 0.40 | 1.02 | 0.468 | 0.568 | 1.000 | 9757 | tags=70%, list=47%, signal=133% |
| 1013 | EMT\_UP |  | 61 | 0.35 | 1.02 | 0.465 | 0.568 | 1.000 | 4635 | tags=31%, list=22%, signal=40% |
| 1014 | ELECTRON\_TRANSPORT |  | 74 | 0.35 | 1.02 | 0.467 | 0.568 | 1.000 | 5727 | tags=31%, list=28%, signal=43% |
| 1015 | PDGF\_ES\_UP |  | 18 | 0.41 | 1.02 | 0.490 | 0.568 | 1.000 | 7011 | tags=50%, list=34%, signal=76% |
| 1016 | NELSON\_ANDROGEN\_UP |  | 60 | 0.35 | 1.02 | 0.494 | 0.568 | 1.000 | 5231 | tags=40%, list=25%, signal=53% |
| 1017 | UVB\_NHEK3\_C3 |  | 17 | 0.42 | 1.02 | 0.472 | 0.568 | 1.000 | 3168 | tags=29%, list=15%, signal=35% |
| 1018 | BRG1\_ALAB\_DN |  | 37 | 0.37 | 1.02 | 0.492 | 0.569 | 1.000 | 4639 | tags=27%, list=23%, signal=35% |
| 1019 | ADDYA\_K562\_HEMIN\_TREATMENT |  | 70 | 0.35 | 1.02 | 0.467 | 0.569 | 1.000 | 4409 | tags=36%, list=21%, signal=45% |
| 1020 | TOB1PATHWAY |  | 17 | 0.42 | 1.02 | 0.471 | 0.571 | 1.000 | 1779 | tags=18%, list=9%, signal=19% |
| 1021 | CORTEX\_ENRICHMENT\_LATE\_UP |  | 20 | 0.40 | 1.01 | 0.488 | 0.576 | 1.000 | 6967 | tags=60%, list=34%, signal=91% |
| 1022 | SHEPARD\_CELL\_PROLIFERATION |  | 203 | 0.33 | 1.01 | 0.464 | 0.579 | 1.000 | 4590 | tags=26%, list=22%, signal=33% |
| 1023 | LEE\_TCELLS6\_UP |  | 27 | 0.38 | 1.01 | 0.497 | 0.581 | 1.000 | 5123 | tags=26%, list=25%, signal=34% |
| 1024 | CCR3PATHWAY |  | 22 | 0.39 | 1.01 | 0.501 | 0.581 | 1.000 | 8787 | tags=55%, list=43%, signal=95% |
| 1025 | LEE\_MYC\_TGFA\_DN |  | 63 | 0.35 | 1.01 | 0.477 | 0.584 | 1.000 | 4974 | tags=25%, list=24%, signal=33% |
| 1026 | ZHAN\_MM\_CD138\_MS\_VS\_REST |  | 47 | 0.36 | 1.01 | 0.513 | 0.586 | 1.000 | 10287 | tags=64%, list=50%, signal=127% |
| 1027 | HEMATOP\_STEM\_ALL\_UP |  | 36 | 0.37 | 1.01 | 0.485 | 0.588 | 1.000 | 952 | tags=14%, list=5%, signal=15% |
| 1028 | S1P\_SIGNALING |  | 24 | 0.39 | 1.01 | 0.491 | 0.588 | 1.000 | 7870 | tags=50%, list=38%, signal=81% |
| 1029 | LEE\_CIP\_DN |  | 65 | 0.35 | 1.01 | 0.503 | 0.589 | 1.000 | 3228 | tags=20%, list=16%, signal=24% |
| 1030 | SARCOMAS\_LEIOMYOSARCOMA\_CALP\_UP |  | 15 | 0.42 | 1.01 | 0.481 | 0.589 | 1.000 | 6940 | tags=53%, list=34%, signal=80% |
| 1031 | ZHAN\_MM\_CD138\_CD2\_VS\_REST |  | 40 | 0.36 | 1.01 | 0.491 | 0.591 | 1.000 | 8115 | tags=45%, list=39%, signal=74% |
| 1032 | CREB\_BRAIN\_8WKS\_DN |  | 49 | 0.35 | 1.00 | 0.505 | 0.595 | 1.000 | 7645 | tags=47%, list=37%, signal=74% |
| 1033 | TSA\_CD4\_UP |  | 25 | 0.39 | 1.00 | 0.501 | 0.595 | 1.000 | 5938 | tags=44%, list=29%, signal=62% |
| 1034 | CALCINEURIN\_NF\_AT\_SIGNALING |  | 93 | 0.33 | 0.99 | 0.522 | 0.615 | 1.000 | 6090 | tags=34%, list=30%, signal=49% |
| 1035 | INSULINPATHWAY |  | 21 | 0.40 | 0.99 | 0.507 | 0.615 | 1.000 | 8147 | tags=57%, list=40%, signal=94% |
| 1036 | TAKEDA\_NUP8\_HOXA9\_8D\_DN |  | 214 | 0.32 | 0.99 | 0.547 | 0.619 | 1.000 | 3915 | tags=20%, list=19%, signal=25% |
| 1037 | LYSINE\_DEGRADATION |  | 30 | 0.37 | 0.99 | 0.513 | 0.627 | 1.000 | 3563 | tags=33%, list=17%, signal=40% |
| 1038 | INSULIN\_SIGNALING |  | 98 | 0.33 | 0.99 | 0.535 | 0.627 | 1.000 | 4651 | tags=24%, list=23%, signal=31% |
| 1039 | MENSSEN\_MYC\_UP |  | 31 | 0.37 | 0.99 | 0.545 | 0.629 | 1.000 | 4229 | tags=35%, list=21%, signal=45% |
| 1040 | JECHLINGER\_EMT\_UP |  | 54 | 0.35 | 0.99 | 0.533 | 0.630 | 1.000 | 4635 | tags=33%, list=22%, signal=43% |
| 1041 | GH\_AUTOCRINE\_UP |  | 219 | 0.32 | 0.98 | 0.545 | 0.635 | 1.000 | 6405 | tags=32%, list=31%, signal=45% |
| 1042 | TNFALPHA\_ADIP\_DN |  | 57 | 0.34 | 0.98 | 0.554 | 0.644 | 1.000 | 5072 | tags=40%, list=25%, signal=53% |
| 1043 | CISPLATIN\_PROBCELL\_UP |  | 17 | 0.40 | 0.98 | 0.536 | 0.647 | 1.000 | 296 | tags=12%, list=1%, signal=12% |
| 1044 | CHESLER\_BRAIN\_ONLY\_SUBSET |  | 23 | 0.38 | 0.98 | 0.550 | 0.651 | 1.000 | 4597 | tags=35%, list=22%, signal=45% |
| 1045 | UVC\_LOW\_C3\_DN |  | 19 | 0.40 | 0.98 | 0.552 | 0.653 | 1.000 | 5974 | tags=42%, list=29%, signal=59% |
| 1046 | TRYPTOPHAN\_METABOLISM |  | 54 | 0.34 | 0.98 | 0.551 | 0.653 | 1.000 | 2463 | tags=19%, list=12%, signal=21% |
| 1047 | IRS1\_KO\_ADIP\_UP |  | 96 | 0.33 | 0.98 | 0.575 | 0.652 | 1.000 | 2598 | tags=16%, list=13%, signal=18% |
| 1048 | NI2\_MOUSE\_DN |  | 45 | 0.35 | 0.97 | 0.563 | 0.653 | 1.000 | 3348 | tags=20%, list=16%, signal=24% |
| 1049 | GAMMA-UV\_FIBRO\_UP |  | 35 | 0.36 | 0.97 | 0.562 | 0.654 | 1.000 | 4111 | tags=31%, list=20%, signal=39% |
| 1050 | EGFPATHWAY |  | 27 | 0.37 | 0.97 | 0.534 | 0.654 | 1.000 | 9253 | tags=63%, list=45%, signal=114% |
| 1051 | KANNAN\_P53\_UP |  | 35 | 0.36 | 0.97 | 0.555 | 0.663 | 1.000 | 3806 | tags=26%, list=18%, signal=31% |
| 1052 | HISTIDINE\_METABOLISM |  | 25 | 0.37 | 0.97 | 0.547 | 0.665 | 1.000 | 6093 | tags=36%, list=30%, signal=51% |
| 1053 | FERRARI\_4HPR\_UP |  | 22 | 0.38 | 0.97 | 0.543 | 0.667 | 1.000 | 1552 | tags=27%, list=8%, signal=29% |
| 1054 | PEART\_HISTONE\_UP |  | 52 | 0.34 | 0.97 | 0.570 | 0.667 | 1.000 | 6030 | tags=42%, list=29%, signal=60% |
| 1055 | EPOPATHWAY |  | 19 | 0.39 | 0.97 | 0.544 | 0.668 | 1.000 | 8147 | tags=63%, list=40%, signal=104% |
| 1056 | CHAUVIN\_ANDROGEN\_REGULATED\_GENES |  | 43 | 0.35 | 0.96 | 0.572 | 0.671 | 1.000 | 6536 | tags=33%, list=32%, signal=48% |
| 1057 | UVC\_LOW\_ALL\_DN |  | 58 | 0.34 | 0.96 | 0.579 | 0.674 | 1.000 | 5366 | tags=33%, list=26%, signal=44% |
| 1058 | BLEO\_HUMAN\_LYMPH\_HIGH\_4HRS\_UP |  | 21 | 0.38 | 0.96 | 0.562 | 0.674 | 1.000 | 2593 | tags=38%, list=13%, signal=44% |
| 1059 | LEE\_MYC\_E2F1\_DN |  | 61 | 0.33 | 0.96 | 0.586 | 0.674 | 1.000 | 1426 | tags=13%, list=7%, signal=14% |
| 1060 | PDGFPATHWAY |  | 27 | 0.36 | 0.96 | 0.564 | 0.680 | 1.000 | 9253 | tags=59%, list=45%, signal=107% |
| 1061 | UEDA\_MOUSE\_SCN |  | 82 | 0.33 | 0.96 | 0.600 | 0.680 | 1.000 | 3914 | tags=26%, list=19%, signal=31% |
| 1062 | AGED\_MOUSE\_CEREBELLUM\_DN |  | 47 | 0.34 | 0.96 | 0.574 | 0.680 | 1.000 | 4482 | tags=32%, list=22%, signal=41% |
| 1063 | DAC\_PANC\_UP |  | 365 | 0.31 | 0.96 | 0.680 | 0.679 | 1.000 | 6043 | tags=31%, list=29%, signal=43% |
| 1064 | LIAN\_MYELOID\_DIFF\_RECEPTORS |  | 29 | 0.36 | 0.96 | 0.554 | 0.680 | 1.000 | 1813 | tags=14%, list=9%, signal=15% |
| 1065 | FATTY\_ACID\_DEGRADATION |  | 26 | 0.36 | 0.96 | 0.588 | 0.682 | 1.000 | 8242 | tags=69%, list=40%, signal=115% |
| 1066 | CREBPATHWAY |  | 27 | 0.36 | 0.96 | 0.558 | 0.682 | 1.000 | 9903 | tags=70%, list=48%, signal=135% |
| 1067 | STAEGE\_EFTS\_UP |  | 29 | 0.36 | 0.96 | 0.569 | 0.681 | 1.000 | 9752 | tags=52%, list=47%, signal=98% |
| 1068 | CELL\_GROWTH\_AND\_OR\_MAINTENANCE |  | 60 | 0.33 | 0.96 | 0.576 | 0.681 | 1.000 | 8587 | tags=53%, list=42%, signal=91% |
| 1069 | RIBAVIRIN\_RSV\_DN |  | 43 | 0.34 | 0.95 | 0.590 | 0.689 | 1.000 | 4353 | tags=30%, list=21%, signal=38% |
| 1070 | HDACI\_COLON\_SUL24HRS\_UP |  | 64 | 0.33 | 0.95 | 0.623 | 0.697 | 1.000 | 3207 | tags=23%, list=16%, signal=28% |
| 1071 | MENSE\_HYPOXIA\_UP |  | 107 | 0.31 | 0.95 | 0.623 | 0.697 | 1.000 | 3804 | tags=26%, list=18%, signal=32% |
| 1072 | PITUITARY\_FETAL\_UP |  | 17 | 0.38 | 0.95 | 0.573 | 0.700 | 1.000 | 4739 | tags=53%, list=23%, signal=69% |
| 1073 | IFNALPHA\_RESIST\_DN |  | 19 | 0.38 | 0.95 | 0.576 | 0.702 | 1.000 | 4388 | tags=32%, list=21%, signal=40% |
| 1074 | P53HYPOXIAPATHWAY |  | 19 | 0.38 | 0.94 | 0.594 | 0.706 | 1.000 | 445 | tags=11%, list=2%, signal=11% |
| 1075 | ANDROGEN\_GENES |  | 52 | 0.33 | 0.94 | 0.613 | 0.708 | 1.000 | 5844 | tags=31%, list=28%, signal=43% |
| 1076 | ADIP\_HUMAN\_UP |  | 60 | 0.33 | 0.94 | 0.612 | 0.711 | 1.000 | 2755 | tags=15%, list=13%, signal=17% |
| 1077 | NOUZOVA\_CPG\_METHLTD |  | 64 | 0.32 | 0.93 | 0.651 | 0.724 | 1.000 | 5877 | tags=25%, list=29%, signal=35% |
| 1078 | SHIPP\_FL\_VS\_DLBCL\_UP |  | 34 | 0.34 | 0.93 | 0.599 | 0.725 | 1.000 | 455 | tags=6%, list=2%, signal=6% |
| 1079 | STRESS\_ARSENIC\_SPECIFIC\_UP |  | 149 | 0.30 | 0.92 | 0.710 | 0.745 | 1.000 | 5862 | tags=36%, list=28%, signal=49% |
| 1080 | VEGF\_HUVEC\_30MIN\_UP |  | 24 | 0.35 | 0.92 | 0.632 | 0.755 | 1.000 | 4275 | tags=21%, list=21%, signal=26% |
| 1081 | CK1PATHWAY |  | 17 | 0.37 | 0.92 | 0.630 | 0.758 | 1.000 | 8357 | tags=65%, list=41%, signal=109% |
| 1082 | ET743\_HELA\_UP |  | 56 | 0.32 | 0.91 | 0.665 | 0.760 | 1.000 | 2508 | tags=23%, list=12%, signal=26% |
| 1083 | LEE\_ACOX1\_DN |  | 63 | 0.32 | 0.91 | 0.674 | 0.760 | 1.000 | 3500 | tags=19%, list=17%, signal=23% |
| 1084 | HUMAN\_TISSUE\_TESTIS |  | 59 | 0.32 | 0.91 | 0.688 | 0.761 | 1.000 | 6973 | tags=36%, list=34%, signal=54% |
| 1085 | JISON\_SICKLE\_CELL |  | 31 | 0.34 | 0.91 | 0.661 | 0.765 | 1.000 | 6199 | tags=45%, list=30%, signal=64% |
| 1086 | ST\_ADRENERGIC |  | 33 | 0.34 | 0.91 | 0.659 | 0.764 | 1.000 | 9813 | tags=58%, list=48%, signal=110% |
| 1087 | ZMPSTE24\_KO\_UP |  | 31 | 0.34 | 0.91 | 0.668 | 0.765 | 1.000 | 1852 | tags=16%, list=9%, signal=18% |
| 1088 | CHREBPPATHWAY |  | 17 | 0.36 | 0.91 | 0.650 | 0.773 | 1.000 | 6794 | tags=47%, list=33%, signal=70% |
| 1089 | AGEING\_LYMPH\_DN |  | 17 | 0.37 | 0.90 | 0.636 | 0.775 | 1.000 | 5432 | tags=29%, list=26%, signal=40% |
| 1090 | TAKEDA\_NUP8\_HOXA9\_10D\_DN |  | 152 | 0.30 | 0.90 | 0.759 | 0.775 | 1.000 | 3998 | tags=20%, list=19%, signal=24% |
| 1091 | BADPATHWAY |  | 22 | 0.35 | 0.90 | 0.651 | 0.778 | 1.000 | 8339 | tags=55%, list=40%, signal=92% |
| 1092 | BRCA1\_SW480\_DN |  | 16 | 0.37 | 0.90 | 0.626 | 0.778 | 1.000 | 5728 | tags=50%, list=28%, signal=69% |
| 1093 | IL7PATHWAY |  | 16 | 0.38 | 0.90 | 0.643 | 0.777 | 1.000 | 10056 | tags=69%, list=49%, signal=134% |
| 1094 | YAO\_P4\_KO\_VS\_WT\_UP |  | 71 | 0.31 | 0.90 | 0.699 | 0.777 | 1.000 | 4372 | tags=27%, list=21%, signal=34% |
| 1095 | IL6PATHWAY |  | 21 | 0.35 | 0.90 | 0.661 | 0.777 | 1.000 | 10284 | tags=76%, list=50%, signal=152% |
| 1096 | FATTY\_ACID\_SYNTHESIS |  | 16 | 0.37 | 0.90 | 0.650 | 0.777 | 1.000 | 2733 | tags=25%, list=13%, signal=29% |
| 1097 | SCHUMACHER\_MYC\_UP |  | 51 | 0.32 | 0.90 | 0.698 | 0.779 | 1.000 | 5094 | tags=37%, list=25%, signal=49% |
| 1098 | TCRPATHWAY |  | 44 | 0.32 | 0.90 | 0.664 | 0.780 | 1.000 | 9253 | tags=50%, list=45%, signal=91% |
| 1099 | TNFALPHA\_TGZ\_ADIP\_DN |  | 27 | 0.33 | 0.90 | 0.652 | 0.783 | 1.000 | 4713 | tags=37%, list=23%, signal=48% |
| 1100 | GLYCOGEN\_METABOLISM |  | 34 | 0.33 | 0.90 | 0.684 | 0.783 | 1.000 | 6134 | tags=38%, list=30%, signal=54% |
| 1101 | CELL\_ADHESION\_MOLECULE\_ACTIVITY |  | 107 | 0.30 | 0.89 | 0.746 | 0.783 | 1.000 | 3500 | tags=19%, list=17%, signal=22% |
| 1102 | IGF1MTORPATHWAY |  | 20 | 0.36 | 0.89 | 0.670 | 0.794 | 1.000 | 8978 | tags=50%, list=44%, signal=89% |
| 1103 | UVB\_NHEK4\_24HRS\_DN |  | 18 | 0.36 | 0.89 | 0.685 | 0.794 | 1.000 | 7189 | tags=50%, list=35%, signal=77% |
| 1104 | GOLUB\_ALL\_VS\_AML\_DN |  | 16 | 0.37 | 0.89 | 0.677 | 0.798 | 1.000 | 7714 | tags=56%, list=37%, signal=90% |
| 1105 | STRESS\_TPA\_SPECIFIC\_UP |  | 42 | 0.32 | 0.88 | 0.706 | 0.801 | 1.000 | 8218 | tags=52%, list=40%, signal=87% |
| 1106 | FCER1PATHWAY |  | 38 | 0.32 | 0.88 | 0.699 | 0.802 | 1.000 | 3247 | tags=21%, list=16%, signal=25% |
| 1107 | MCALPAINPATHWAY |  | 24 | 0.34 | 0.88 | 0.674 | 0.802 | 1.000 | 4708 | tags=38%, list=23%, signal=49% |
| 1108 | PROSTAGLANDIN\_AND\_LEUKOTRIENE\_METABOLISM |  | 33 | 0.33 | 0.88 | 0.699 | 0.804 | 1.000 | 2478 | tags=15%, list=12%, signal=17% |
| 1109 | TESTIS\_EXPRESSED\_GENES |  | 61 | 0.31 | 0.88 | 0.727 | 0.806 | 1.000 | 4092 | tags=20%, list=20%, signal=24% |
| 1110 | HOGERKORP\_ANTI\_CD44\_UP |  | 23 | 0.34 | 0.87 | 0.687 | 0.811 | 1.000 | 8086 | tags=39%, list=39%, signal=64% |
| 1111 | HBX\_HCC\_UP |  | 16 | 0.35 | 0.87 | 0.687 | 0.817 | 1.000 | 7410 | tags=50%, list=36%, signal=78% |
| 1112 | IL12PATHWAY |  | 20 | 0.35 | 0.87 | 0.702 | 0.817 | 1.000 | 2328 | tags=15%, list=11%, signal=17% |
| 1113 | GPCRPATHWAY |  | 35 | 0.32 | 0.87 | 0.708 | 0.818 | 1.000 | 8147 | tags=51%, list=40%, signal=85% |
| 1114 | NFATPATHWAY |  | 52 | 0.30 | 0.87 | 0.739 | 0.819 | 1.000 | 8367 | tags=44%, list=41%, signal=74% |
| 1115 | HCC\_SURVIVAL\_GOOD\_VS\_POOR\_UP |  | 136 | 0.28 | 0.87 | 0.816 | 0.820 | 1.000 | 5699 | tags=26%, list=28%, signal=35% |
| 1116 | STURLA\_SONIC\_HEDGEHOG |  | 16 | 0.36 | 0.87 | 0.684 | 0.820 | 1.000 | 7424 | tags=56%, list=36%, signal=88% |
| 1117 | SPRYPATHWAY |  | 18 | 0.35 | 0.86 | 0.707 | 0.833 | 1.000 | 9757 | tags=67%, list=47%, signal=127% |
| 1118 | FRUCTOSE\_AND\_MANNOSE\_METABOLISM |  | 25 | 0.33 | 0.86 | 0.724 | 0.836 | 1.000 | 4442 | tags=24%, list=22%, signal=31% |
| 1119 | INSULIN\_ADIP\_INSENS\_UP |  | 23 | 0.33 | 0.86 | 0.715 | 0.836 | 1.000 | 3972 | tags=30%, list=19%, signal=38% |
| 1120 | ETSPATHWAY |  | 18 | 0.34 | 0.85 | 0.704 | 0.838 | 1.000 | 6043 | tags=39%, list=29%, signal=55% |
| 1121 | PROPANOATE\_METABOLISM |  | 31 | 0.32 | 0.85 | 0.724 | 0.838 | 1.000 | 2479 | tags=26%, list=12%, signal=29% |
| 1122 | ST\_T\_CELL\_SIGNAL\_TRANSDUCTION |  | 44 | 0.30 | 0.85 | 0.746 | 0.841 | 1.000 | 10137 | tags=57%, list=49%, signal=112% |
| 1123 | HASLINGER\_B\_CLL\_11Q23 |  | 20 | 0.34 | 0.85 | 0.713 | 0.842 | 1.000 | 10080 | tags=60%, list=49%, signal=117% |
| 1124 | NF90\_DN |  | 38 | 0.30 | 0.85 | 0.729 | 0.843 | 1.000 | 3538 | tags=24%, list=17%, signal=29% |
| 1125 | WANG\_HOXA9\_VS\_MEIS1\_DN |  | 23 | 0.33 | 0.85 | 0.731 | 0.845 | 1.000 | 9403 | tags=61%, list=46%, signal=112% |
| 1126 | COLLER\_MYC\_UP |  | 18 | 0.34 | 0.85 | 0.707 | 0.844 | 1.000 | 5111 | tags=33%, list=25%, signal=44% |
| 1127 | BETA\_ALANINE\_METABOLISM |  | 27 | 0.32 | 0.85 | 0.728 | 0.844 | 1.000 | 8820 | tags=59%, list=43%, signal=103% |
| 1128 | HDACI\_COLON\_CLUSTER5 |  | 23 | 0.33 | 0.85 | 0.730 | 0.843 | 1.000 | 2722 | tags=26%, list=13%, signal=30% |
| 1129 | XPB\_TTD-CS\_UP |  | 26 | 0.32 | 0.85 | 0.716 | 0.843 | 1.000 | 8876 | tags=50%, list=43%, signal=88% |
| 1130 | GRANDVAUX\_IRF3\_DN |  | 20 | 0.34 | 0.84 | 0.730 | 0.844 | 1.000 | 4826 | tags=35%, list=23%, signal=46% |
| 1131 | VEGFPATHWAY |  | 27 | 0.33 | 0.84 | 0.743 | 0.845 | 1.000 | 8788 | tags=52%, list=43%, signal=90% |
| 1132 | TSA\_CD4\_DN |  | 18 | 0.34 | 0.84 | 0.737 | 0.851 | 1.000 | 8614 | tags=50%, list=42%, signal=86% |
| 1133 | CALRES\_RHESUS\_UP |  | 67 | 0.29 | 0.84 | 0.799 | 0.853 | 1.000 | 2613 | tags=18%, list=13%, signal=20% |
| 1134 | GLYCEROPHOSPHOLIPID\_METABOLISM |  | 49 | 0.29 | 0.83 | 0.795 | 0.863 | 1.000 | 8222 | tags=43%, list=40%, signal=71% |
| 1135 | TPOPATHWAY |  | 23 | 0.32 | 0.83 | 0.765 | 0.863 | 1.000 | 9226 | tags=61%, list=45%, signal=110% |
| 1136 | SMOOTH\_MUSCLE\_CONTRACTION |  | 143 | 0.27 | 0.83 | 0.887 | 0.863 | 1.000 | 6542 | tags=37%, list=32%, signal=54% |
| 1137 | ZHAN\_TONSIL\_BONEMARROW |  | 41 | 0.29 | 0.82 | 0.775 | 0.867 | 1.000 | 3064 | tags=17%, list=15%, signal=20% |
| 1138 | HSIAO\_LIVER\_SPECIFIC\_GENES |  | 240 | 0.26 | 0.82 | 0.940 | 0.872 | 1.000 | 4802 | tags=22%, list=23%, signal=28% |
| 1139 | HOGERKORP\_CD44\_UP |  | 23 | 0.32 | 0.82 | 0.776 | 0.875 | 1.000 | 8086 | tags=39%, list=39%, signal=64% |
| 1140 | BOQUEST\_CD31PLUS\_VS\_CD31MINUS\_DN |  | 265 | 0.26 | 0.81 | 0.951 | 0.876 | 1.000 | 3762 | tags=15%, list=18%, signal=19% |
| 1141 | LEE\_MYC\_DN |  | 59 | 0.28 | 0.81 | 0.828 | 0.879 | 1.000 | 2298 | tags=12%, list=11%, signal=13% |
| 1142 | BUTANOATE\_METABOLISM |  | 27 | 0.31 | 0.81 | 0.784 | 0.879 | 1.000 | 2557 | tags=19%, list=12%, signal=21% |
| 1143 | MAMMARY\_DEV\_UP |  | 55 | 0.29 | 0.81 | 0.826 | 0.880 | 1.000 | 4515 | tags=16%, list=22%, signal=21% |
| 1144 | OVARIAN\_INFERTILITY\_GENES |  | 25 | 0.31 | 0.81 | 0.763 | 0.881 | 1.000 | 4068 | tags=20%, list=20%, signal=25% |
| 1145 | GH\_EXOGENOUS\_EARLY\_UP |  | 29 | 0.30 | 0.81 | 0.793 | 0.881 | 1.000 | 7021 | tags=34%, list=34%, signal=52% |
| 1146 | ST\_P38\_MAPK\_PATHWAY |  | 36 | 0.30 | 0.81 | 0.792 | 0.881 | 1.000 | 6527 | tags=39%, list=32%, signal=57% |
| 1147 | LE\_MYELIN\_DN |  | 93 | 0.27 | 0.81 | 0.856 | 0.881 | 1.000 | 5915 | tags=34%, list=29%, signal=48% |
| 1148 | IL2PATHWAY |  | 22 | 0.32 | 0.80 | 0.767 | 0.883 | 1.000 | 8587 | tags=55%, list=42%, signal=93% |
| 1149 | INOSITOL\_PHOSPHATE\_METABOLISM |  | 25 | 0.31 | 0.80 | 0.772 | 0.884 | 1.000 | 9183 | tags=48%, list=45%, signal=86% |
| 1150 | WNT\_TARGETS |  | 25 | 0.31 | 0.80 | 0.783 | 0.886 | 1.000 | 7644 | tags=36%, list=37%, signal=57% |
| 1151 | GH\_EXOGENOUS\_ANY\_DN |  | 96 | 0.27 | 0.80 | 0.892 | 0.891 | 1.000 | 8025 | tags=34%, list=39%, signal=56% |
| 1152 | NGFPATHWAY |  | 19 | 0.32 | 0.80 | 0.782 | 0.890 | 1.000 | 8147 | tags=47%, list=40%, signal=78% |
| 1153 | DFOSB\_BRAIN\_2WKS\_UP |  | 38 | 0.29 | 0.79 | 0.807 | 0.891 | 1.000 | 3139 | tags=21%, list=15%, signal=25% |
| 1154 | GH\_EXOGENOUS\_LATE\_UP |  | 78 | 0.27 | 0.79 | 0.876 | 0.892 | 1.000 | 7933 | tags=38%, list=38%, signal=62% |
| 1155 | IGFR\_IR\_UP |  | 18 | 0.32 | 0.79 | 0.776 | 0.891 | 1.000 | 5094 | tags=28%, list=25%, signal=37% |
| 1156 | UVC\_LOW\_C2\_DN |  | 20 | 0.31 | 0.79 | 0.787 | 0.895 | 1.000 | 11839 | tags=70%, list=57%, signal=164% |
| 1157 | BRENTANI\_CYTOSKELETON |  | 22 | 0.31 | 0.79 | 0.795 | 0.896 | 1.000 | 2340 | tags=14%, list=11%, signal=15% |
| 1158 | LIZUKA\_G2\_GR\_G3 |  | 27 | 0.30 | 0.79 | 0.800 | 0.896 | 1.000 | 7731 | tags=56%, list=38%, signal=89% |
| 1159 | HADDAD\_HSC\_CD7\_UP |  | 64 | 0.27 | 0.79 | 0.859 | 0.895 | 1.000 | 5350 | tags=22%, list=26%, signal=29% |
| 1160 | HADDAD\_CD45CD7\_PLUS\_VS\_MINUS\_UP |  | 64 | 0.27 | 0.79 | 0.858 | 0.895 | 1.000 | 5350 | tags=22%, list=26%, signal=29% |
| 1161 | NOS1PATHWAY |  | 22 | 0.31 | 0.78 | 0.796 | 0.901 | 1.000 | 6794 | tags=41%, list=33%, signal=61% |
| 1162 | CALCIUM\_REGULATION\_IN\_CARDIAC\_CELLS |  | 140 | 0.26 | 0.78 | 0.934 | 0.905 | 1.000 | 8912 | tags=48%, list=43%, signal=84% |
| 1163 | AMINOSUGARS\_METABOLISM |  | 15 | 0.32 | 0.77 | 0.804 | 0.905 | 1.000 | 3462 | tags=20%, list=17%, signal=24% |
| 1164 | BCNU\_GLIOMA\_MGMT\_48HRS\_UP |  | 18 | 0.31 | 0.77 | 0.798 | 0.905 | 1.000 | 4443 | tags=28%, list=22%, signal=35% |
| 1165 | CMV\_HCMV\_TIMECOURSE\_48HRS\_UP |  | 66 | 0.27 | 0.77 | 0.883 | 0.904 | 1.000 | 9948 | tags=50%, list=48%, signal=96% |
| 1166 | BILE\_ACID\_BIOSYNTHESIS |  | 27 | 0.29 | 0.77 | 0.828 | 0.906 | 1.000 | 2028 | tags=15%, list=10%, signal=16% |
| 1167 | MPRPATHWAY |  | 23 | 0.30 | 0.77 | 0.809 | 0.905 | 1.000 | 7202 | tags=52%, list=35%, signal=80% |
| 1168 | HIPPOCAMPUS\_DEVELOPMENT\_POSTNATAL |  | 45 | 0.27 | 0.77 | 0.848 | 0.906 | 1.000 | 5758 | tags=31%, list=28%, signal=43% |
| 1169 | CAMPTOTHECIN\_PROBCELL\_UP |  | 22 | 0.30 | 0.77 | 0.820 | 0.908 | 1.000 | 6894 | tags=36%, list=33%, signal=55% |
| 1170 | ST\_WNT\_CA2\_CYCLIC\_GMP\_PATHWAY |  | 19 | 0.31 | 0.77 | 0.807 | 0.908 | 1.000 | 4111 | tags=21%, list=20%, signal=26% |
| 1171 | CROMER\_HYPOPHARYNGEAL\_MET\_VS\_NON\_DN |  | 81 | 0.26 | 0.76 | 0.907 | 0.914 | 1.000 | 4255 | tags=28%, list=21%, signal=36% |
| 1172 | STARCH\_AND\_SUCROSE\_METABOLISM |  | 31 | 0.28 | 0.76 | 0.852 | 0.914 | 1.000 | 4801 | tags=19%, list=23%, signal=25% |
| 1173 | HUMAN\_TISSUE\_PLACENTA |  | 19 | 0.30 | 0.75 | 0.838 | 0.917 | 1.000 | 12685 | tags=74%, list=62%, signal=192% |
| 1174 | HBX\_NL\_DN |  | 16 | 0.31 | 0.75 | 0.819 | 0.917 | 1.000 | 6734 | tags=44%, list=33%, signal=65% |
| 1175 | BIOGENIC\_AMINE\_SYNTHESIS |  | 15 | 0.31 | 0.75 | 0.819 | 0.918 | 1.000 | 305 | tags=7%, list=1%, signal=7% |
| 1176 | PENG\_RAPAMYCIN\_DN |  | 188 | 0.24 | 0.75 | 0.973 | 0.919 | 1.000 | 5688 | tags=30%, list=28%, signal=41% |
| 1177 | SCHRAETS\_MLL\_UP |  | 34 | 0.27 | 0.74 | 0.867 | 0.924 | 1.000 | 9762 | tags=50%, list=47%, signal=95% |
| 1178 | METHOTREXATE\_PROBCELL\_UP |  | 18 | 0.30 | 0.74 | 0.836 | 0.927 | 1.000 | 6810 | tags=39%, list=33%, signal=58% |
| 1179 | AGED\_MOUSE\_NEOCORTEX\_DN |  | 55 | 0.26 | 0.74 | 0.891 | 0.926 | 1.000 | 6262 | tags=27%, list=30%, signal=39% |
| 1180 | VEGF\_HUVEC\_2HRS\_UP |  | 30 | 0.27 | 0.73 | 0.864 | 0.929 | 1.000 | 5645 | tags=37%, list=27%, signal=50% |
| 1181 | INFLAMMATORY\_RESPONSE\_PATHWAY |  | 29 | 0.28 | 0.73 | 0.872 | 0.928 | 1.000 | 8241 | tags=38%, list=40%, signal=63% |
| 1182 | LEI\_HOXC8\_DN |  | 15 | 0.30 | 0.73 | 0.843 | 0.930 | 1.000 | 3567 | tags=20%, list=17%, signal=24% |
| 1183 | METPATHWAY |  | 34 | 0.27 | 0.73 | 0.869 | 0.931 | 1.000 | 5218 | tags=29%, list=25%, signal=39% |
| 1184 | ALANINE\_AND\_ASPARTATE\_METABOLISM |  | 21 | 0.29 | 0.73 | 0.859 | 0.932 | 1.000 | 3131 | tags=29%, list=15%, signal=34% |
| 1185 | SMALL\_LIGAND\_GPCRS |  | 17 | 0.30 | 0.72 | 0.851 | 0.933 | 1.000 | 8533 | tags=53%, list=41%, signal=90% |
| 1186 | HANSON\_NFKAPPB\_IND |  | 18 | 0.29 | 0.72 | 0.862 | 0.939 | 1.000 | 8505 | tags=50%, list=41%, signal=85% |
| 1187 | PROSTAGLANDIN\_SYNTHESIS\_REGULATION |  | 28 | 0.27 | 0.71 | 0.873 | 0.939 | 1.000 | 9151 | tags=46%, list=44%, signal=83% |
| 1188 | CORDERO\_KRAS\_KD\_VS\_CONTROL\_DN |  | 55 | 0.25 | 0.71 | 0.908 | 0.939 | 1.000 | 6421 | tags=38%, list=31%, signal=55% |
| 1189 | TAKEDA\_NUP8\_HOXA9\_6H\_UP |  | 85 | 0.24 | 0.71 | 0.950 | 0.939 | 1.000 | 7230 | tags=33%, list=35%, signal=51% |
| 1190 | NITROGEN\_METABOLISM |  | 21 | 0.28 | 0.71 | 0.860 | 0.940 | 1.000 | 6649 | tags=29%, list=32%, signal=42% |
| 1191 | GH\_EXOGENOUS\_ALL\_UP |  | 59 | 0.25 | 0.71 | 0.918 | 0.940 | 1.000 | 6030 | tags=24%, list=29%, signal=33% |
| 1192 | GH\_EXOGENOUS\_ANY\_UP |  | 291 | 0.22 | 0.69 | 0.997 | 0.949 | 1.000 | 7162 | tags=27%, list=35%, signal=42% |
| 1193 | ZHAN\_MM\_CD138\_PR\_VS\_REST |  | 49 | 0.25 | 0.69 | 0.938 | 0.949 | 1.000 | 8429 | tags=41%, list=41%, signal=69% |
| 1194 | SHIPP\_DLBCL\_CURED\_UP |  | 30 | 0.26 | 0.69 | 0.898 | 0.949 | 1.000 | 2183 | tags=13%, list=11%, signal=15% |
| 1195 | GH\_EXOGENOUS\_LATE\_DN |  | 80 | 0.23 | 0.69 | 0.954 | 0.949 | 1.000 | 9141 | tags=41%, list=44%, signal=74% |
| 1196 | AMIPATHWAY |  | 22 | 0.26 | 0.68 | 0.891 | 0.953 | 1.000 | 6794 | tags=36%, list=33%, signal=54% |
| 1197 | CMV\_HCMV\_TIMECOURSE\_4HRS\_DN |  | 35 | 0.25 | 0.68 | 0.907 | 0.954 | 1.000 | 5619 | tags=37%, list=27%, signal=51% |
| 1198 | ANDROGEN\_AND\_ESTROGEN\_METABOLISM |  | 24 | 0.26 | 0.67 | 0.911 | 0.960 | 1.000 | 4160 | tags=17%, list=20%, signal=21% |
| 1199 | IL3PATHWAY |  | 15 | 0.28 | 0.67 | 0.897 | 0.959 | 1.000 | 9650 | tags=60%, list=47%, signal=113% |
| 1200 | CSKPATHWAY |  | 22 | 0.26 | 0.67 | 0.906 | 0.959 | 1.000 | 6794 | tags=36%, list=33%, signal=54% |
| 1201 | LEE\_DENA\_DN |  | 72 | 0.23 | 0.67 | 0.960 | 0.959 | 1.000 | 5010 | tags=22%, list=24%, signal=29% |
| 1202 | YAO\_P4\_KO\_VS\_WT\_DN |  | 16 | 0.27 | 0.66 | 0.907 | 0.960 | 1.000 | 5198 | tags=31%, list=25%, signal=42% |
| 1203 | METASTASIS\_ADENOCARC\_DN |  | 34 | 0.24 | 0.65 | 0.939 | 0.964 | 1.000 | 9720 | tags=53%, list=47%, signal=100% |
| 1204 | PASSERINI\_IMMUNE |  | 21 | 0.25 | 0.64 | 0.926 | 0.968 | 1.000 | 10439 | tags=48%, list=51%, signal=96% |
| 1205 | IRS\_KO\_ADIP\_UP |  | 28 | 0.24 | 0.64 | 0.940 | 0.970 | 1.000 | 2598 | tags=14%, list=13%, signal=16% |
| 1206 | GPCRS\_CLASS\_B\_SECRETIN\_LIKE |  | 23 | 0.25 | 0.63 | 0.928 | 0.971 | 1.000 | 8723 | tags=39%, list=42%, signal=68% |
| 1207 | HOFMANN\_MDS\_CD34\_LOW\_RISK |  | 50 | 0.23 | 0.63 | 0.959 | 0.972 | 1.000 | 1874 | tags=12%, list=9%, signal=13% |
| 1208 | GPCRDB\_CLASS\_B\_SECRETIN\_LIKE |  | 23 | 0.25 | 0.63 | 0.926 | 0.972 | 1.000 | 8723 | tags=39%, list=42%, signal=68% |
| 1209 | MARSHALL\_SPLEEN\_BAL |  | 21 | 0.24 | 0.62 | 0.932 | 0.973 | 1.000 | 7160 | tags=38%, list=35%, signal=58% |
| 1210 | CANCERDRUGS\_PROBCELL\_UP |  | 20 | 0.25 | 0.62 | 0.940 | 0.973 | 1.000 | 5541 | tags=25%, list=27%, signal=34% |
| 1211 | CROONQUIST\_IL6\_RAS\_UP |  | 22 | 0.24 | 0.61 | 0.932 | 0.978 | 1.000 | 8249 | tags=36%, list=40%, signal=61% |
| 1212 | GLYCEROLIPID\_METABOLISM |  | 44 | 0.21 | 0.60 | 0.966 | 0.981 | 1.000 | 8222 | tags=39%, list=40%, signal=64% |
| 1213 | GH\_EXOGENOUS\_MIDDLE\_UP |  | 125 | 0.20 | 0.60 | 0.996 | 0.980 | 1.000 | 6123 | tags=21%, list=30%, signal=29% |
| 1214 | CARBON\_FIXATION |  | 21 | 0.23 | 0.59 | 0.953 | 0.982 | 1.000 | 2181 | tags=14%, list=11%, signal=16% |
| 1215 | BREAST\_DUCTAL\_CARCINOMA\_GENES |  | 19 | 0.24 | 0.59 | 0.938 | 0.982 | 1.000 | 5500 | tags=32%, list=27%, signal=43% |
| 1216 | FATTY\_ACID\_METABOLISM |  | 82 | 0.20 | 0.59 | 0.993 | 0.981 | 1.000 | 6310 | tags=29%, list=31%, signal=42% |
| 1217 | TSADAC\_HYPERMETH\_OVCA\_UP |  | 15 | 0.24 | 0.58 | 0.952 | 0.983 | 1.000 | 1645 | tags=13%, list=8%, signal=14% |
| 1218 | MARCINIAK\_CHOP\_DIFF |  | 21 | 0.23 | 0.57 | 0.964 | 0.985 | 1.000 | 8339 | tags=43%, list=40%, signal=72% |
| 1219 | NO2IL12PATHWAY |  | 15 | 0.24 | 0.56 | 0.957 | 0.986 | 1.000 | 1822 | tags=7%, list=9%, signal=7% |
| 1220 | STATIN\_PATHWAY\_PHARMGKB |  | 17 | 0.23 | 0.56 | 0.968 | 0.986 | 1.000 | 4833 | tags=29%, list=23%, signal=38% |
| 1221 | CPR\_NULL-LOW\_LIVER\_UP |  | 17 | 0.21 | 0.53 | 0.973 | 0.992 | 1.000 | 9799 | tags=47%, list=48%, signal=90% |
| 1222 | MONOAMINE\_GPCRS |  | 32 | 0.18 | 0.49 | 0.983 | 0.997 | 1.000 | 9150 | tags=31%, list=44%, signal=56% |
| 1223 | GATA3PATHWAY |  | 15 | 0.21 | 0.49 | 0.983 | 0.997 | 1.000 | 6794 | tags=33%, list=33%, signal=50% |
| 1224 | DER\_IFNB\_UP |  | 93 | 0.65 | 1.91 | 0.000 | 0.000 | 0.007 | 3425 | tags=51%, list=17%, signal=60% |
| 1225 | FLECHNER\_KIDNEY\_TRANSPLANT\_WELL\_PBL\_DN |  | 42 | 0.48 | 1.32 | 0.060 | 0.061 | 1.000 | 5933 | tags=52%, list=29%, signal=73% |
| 1226 | KLEIN\_PEL\_DN |  | 58 | 0.46 | 1.32 | 0.051 | 0.063 | 1.000 | 5746 | tags=43%, list=28%, signal=60% |
| 1227 | BCRPATHWAY |  | 35 | 0.38 | 1.06 | 0.408 | 0.390 | 1.000 | 8147 | tags=51%, list=40%, signal=85% |
Table: Gene sets enriched in phenotype **na**[plain text format]****

  
