# Supplementary material for: The rapamycin-regulated gene expression signature determines prognosis for breast cancer
Source: Mol Cancer. 2009 Sep 24;8:75. doi: 10.1186/1476-4598-8-75 (PMC2761377; doi:10.1186/1476-4598-8-75)
Supplement: Additional file 2 — Gene set enrichment analysis of in vivo data, time series. The data provided represent the time series of GSEA. This compressed file contains "Time" shortcut file and "GSEA_time" folder. Clicking on "Time" shortcut opens the index file providing access to analysis files contained in the "GSEA_time" folder. [file 1476-4598-8-75-S2.zip › GSEA_time/GUO_HEX_UP.html]

Details for gene set GUO\_HEX\_UP[GSEA]

|  || Dataset | gsea\_time\_collapsed |
| Phenotype | NoPhenotypeAvailable |
| Upregulated in class | na\_pos |
| GeneSet | GUO\_HEX\_UP |
| Enrichment Score (ES) | 0.601588 |
| Normalized Enrichment Score (NES) | 1.7353402 |
| Nominal p-value | 0.0 |
| FDR q-value | 0.013634211 |
| FWER p-Value | 0.489 |
Table: GSEA Results Summary

  

Fig 1: Enrichment plot: GUO\_HEX\_UP      
 Profile of the Running ES Score & Positions of GeneSet Members on the Rank Ordered List

  

| PROBE | GENE SYMBOL | GENE\_TITLE | RANK IN GENE LIST | RANK METRIC SCORE | RUNNING ES | CORE ENRICHMENT || 1 | EXT1 |  |  | 230 | 0.695 | 0.0339 | Yes |
| 2 | PTEN |  |  | 250 | 0.681 | 0.0773 | Yes |
| 3 | VEGF |  |  | 252 | 0.681 | 0.1215 | Yes |
| 4 | FBLN2 |  |  | 271 | 0.671 | 0.1642 | Yes |
| 5 | SYNCRIP |  |  | 308 | 0.642 | 0.2041 | Yes |
| 6 | SFRS7 |  |  | 366 | 0.598 | 0.2402 | Yes |
| 7 | NRP1 |  |  | 608 | 0.496 | 0.2606 | Yes |
| 8 | ELOVL6 |  |  | 627 | 0.491 | 0.2916 | Yes |
| 9 | LUC7L2 |  |  | 934 | 0.417 | 0.3038 | Yes |
| 10 | SP1 |  |  | 956 | 0.413 | 0.3296 | Yes |
| 11 | ATP6V1A |  |  | 961 | 0.413 | 0.3562 | Yes |
| 12 | NARG1 |  |  | 1323 | 0.351 | 0.3614 | Yes |
| 13 | CMTM6 |  |  | 1405 | 0.340 | 0.3796 | Yes |
| 14 | PJA1 |  |  | 1774 | 0.302 | 0.3813 | Yes |
| 15 | IQGAP1 |  |  | 1950 | 0.288 | 0.3914 | Yes |
| 16 | GTF2H1 |  |  | 2136 | 0.274 | 0.4002 | Yes |
| 17 | PCGF2 |  |  | 2185 | 0.270 | 0.4154 | Yes |
| 18 | TBX3 |  |  | 2346 | 0.259 | 0.4244 | Yes |
| 19 | PTPN13 |  |  | 2504 | 0.247 | 0.4328 | Yes |
| 20 | KLF7 |  |  | 2569 | 0.244 | 0.4455 | Yes |
| 21 | MTF2 |  |  | 2607 | 0.241 | 0.4594 | Yes |
| 22 | CD99 |  |  | 2687 | 0.237 | 0.4709 | Yes |
| 23 | AKAP12 |  |  | 2720 | 0.235 | 0.4846 | Yes |
| 24 | SMARCC1 |  |  | 2815 | 0.229 | 0.4949 | Yes |
| 25 | PGF |  |  | 2843 | 0.228 | 0.5084 | Yes |
| 26 | WWTR1 |  |  | 2873 | 0.226 | 0.5217 | Yes |
| 27 | HRB |  |  | 3092 | 0.214 | 0.5250 | Yes |
| 28 | PCM1 |  |  | 3148 | 0.211 | 0.5360 | Yes |
| 29 | AKAP8 |  |  | 3348 | 0.202 | 0.5394 | Yes |
| 30 | SPG21 |  |  | 3806 | 0.182 | 0.5290 | Yes |
| 31 | RBMS1 |  |  | 3919 | 0.177 | 0.5350 | Yes |
| 32 | CALM3 |  |  | 3967 | 0.175 | 0.5441 | Yes |
| 33 | SLC6A6 |  |  | 3988 | 0.174 | 0.5544 | Yes |
| 34 | SNX5 |  |  | 4174 | 0.166 | 0.5562 | Yes |
| 35 | IMPAD1 |  |  | 4219 | 0.164 | 0.5647 | Yes |
| 36 | DHX36 |  |  | 4298 | 0.161 | 0.5714 | Yes |
| 37 | UBE3A |  |  | 4374 | 0.158 | 0.5780 | Yes |
| 38 | ABCD4 |  |  | 4453 | 0.156 | 0.5843 | Yes |
| 39 | IGF1R |  |  | 4531 | 0.153 | 0.5905 | Yes |
| 40 | TBL1X |  |  | 4946 | 0.139 | 0.5794 | Yes |
| 41 | SFRP2 |  |  | 5182 | 0.132 | 0.5765 | Yes |
| 42 | ATBF1 |  |  | 5300 | 0.129 | 0.5792 | Yes |
| 43 | TES |  |  | 5362 | 0.127 | 0.5845 | Yes |
| 44 | ENPP5 |  |  | 5477 | 0.125 | 0.5871 | Yes |
| 45 | RBM39 |  |  | 5485 | 0.124 | 0.5948 | Yes |
| 46 | FZD4 |  |  | 5511 | 0.124 | 0.6016 | Yes |
| 47 | ENY2 |  |  | 5760 | 0.118 | 0.5972 | No |
| 48 | STIL |  |  | 5918 | 0.115 | 0.5970 | No |
| 49 | ERF |  |  | 5985 | 0.113 | 0.6011 | No |
| 50 | SOX11 |  |  | 6343 | 0.105 | 0.5905 | No |
| 51 | WDR26 |  |  | 6977 | 0.092 | 0.5657 | No |
| 52 | UTX |  |  | 7186 | 0.088 | 0.5613 | No |
| 53 | HOXD1 |  |  | 7516 | 0.082 | 0.5505 | No |
| 54 | ATXN2 |  |  | 8014 | 0.073 | 0.5310 | No |
| 55 | WWC2 |  |  | 8481 | 0.065 | 0.5126 | No |
| 56 | PURA |  |  | 8897 | 0.058 | 0.4961 | No |
| 57 | POU2F1 |  |  | 9338 | 0.052 | 0.4780 | No |
| 58 | MARK2 |  |  | 9594 | 0.048 | 0.4687 | No |
| 59 | GRIK5 |  |  | 10136 | 0.040 | 0.4450 | No |
| 60 | SATB1 |  |  | 10714 | 0.032 | 0.4189 | No |
| 61 | BMP4 |  |  | 11047 | 0.027 | 0.4045 | No |
| 62 | MAGEL2 |  |  | 11163 | 0.025 | 0.4006 | No |
| 63 | EPHA4 |  |  | 11620 | 0.019 | 0.3796 | No |
| 64 | GLG1 |  |  | 11950 | 0.014 | 0.3645 | No |
| 65 | MMP14 |  |  | 11968 | 0.014 | 0.3646 | No |
| 66 | PTPN1 |  |  | 12551 | 0.006 | 0.3366 | No |
| 67 | RTN1 |  |  | 12603 | 0.005 | 0.3345 | No |
| 68 | EFNA2 |  |  | 12673 | 0.003 | 0.3313 | No |
| 69 | ISLR2 |  |  | 12810 | 0.001 | 0.3248 | No |
| 70 | ARSA |  |  | 13137 | -0.003 | 0.3091 | No |
| 71 | FGD1 |  |  | 13283 | -0.005 | 0.3024 | No |
| 72 | CD2BP2 |  |  | 13440 | -0.008 | 0.2953 | No |
| 73 | HNRPU |  |  | 13589 | -0.010 | 0.2887 | No |
| 74 | FTS |  |  | 13607 | -0.010 | 0.2885 | No |
| 75 | NDN |  |  | 13980 | -0.016 | 0.2714 | No |
| 76 | NNAT |  |  | 14865 | -0.030 | 0.2303 | No |
| 77 | GNG3 |  |  | 16462 | -0.058 | 0.1563 | No |
| 78 | REST |  |  | 18344 | -0.111 | 0.0719 | No |
| 79 | RRAGD |  |  | 19023 | -0.142 | 0.0481 | No |
| 80 | CIC |  |  | 19735 | -0.204 | 0.0267 | No |
| 81 | EFNA3 |  |  | 19998 | -0.240 | 0.0296 | No |
Table: GSEA details [plain text format]

  

Fig 2: GUO\_HEX\_UP: Random ES distribution      
 Gene set null distribution of ES for **GUO\_HEX\_UP**
