# Supplementary material for: The rapamycin-regulated gene expression signature determines prognosis for breast cancer
Source: Mol Cancer. 2009 Sep 24;8:75. doi: 10.1186/1476-4598-8-75 (PMC2761377; doi:10.1186/1476-4598-8-75)
Supplement: Additional file 2 — Gene set enrichment analysis of in vivo data, time series. The data provided represent the time series of GSEA. This compressed file contains "Time" shortcut file and "GSEA_time" folder. Clicking on "Time" shortcut opens the index file providing access to analysis files contained in the "GSEA_time" folder. [file 1476-4598-8-75-S2.zip › GSEA_time/HDACI_COLON_BUT30MIN_DN.html]

Details for gene set HDACI\_COLON\_BUT30MIN\_DN[GSEA]

|  || Dataset | gsea\_time\_collapsed |
| Phenotype | NoPhenotypeAvailable |
| Upregulated in class | na\_pos |
| GeneSet | HDACI\_COLON\_BUT30MIN\_DN |
| Enrichment Score (ES) | 0.6865061 |
| Normalized Enrichment Score (NES) | 1.8217912 |
| Nominal p-value | 0.0 |
| FDR q-value | 0.0046944544 |
| FWER p-Value | 0.11 |
Table: GSEA Results Summary

  

Fig 1: Enrichment plot: HDACI\_COLON\_BUT30MIN\_DN      
 Profile of the Running ES Score & Positions of GeneSet Members on the Rank Ordered List

  

| PROBE | GENE SYMBOL | GENE\_TITLE | RANK IN GENE LIST | RANK METRIC SCORE | RUNNING ES | CORE ENRICHMENT || 1 | CALD1 |  |  | 72 | 0.980 | 0.0942 | Yes |
| 2 | MRPS31 |  |  | 118 | 0.873 | 0.1791 | Yes |
| 3 | TTC3 |  |  | 197 | 0.730 | 0.2481 | Yes |
| 4 | C18ORF1 |  |  | 306 | 0.642 | 0.3069 | Yes |
| 5 | SEH1L |  |  | 440 | 0.556 | 0.3559 | Yes |
| 6 | LAMP2 |  |  | 564 | 0.511 | 0.4009 | Yes |
| 7 | HNRPA3 |  |  | 703 | 0.473 | 0.4414 | Yes |
| 8 | MBNL2 |  |  | 718 | 0.470 | 0.4876 | Yes |
| 9 | DLG1 |  |  | 845 | 0.434 | 0.5248 | Yes |
| 10 | EIF4E |  |  | 1263 | 0.361 | 0.5405 | Yes |
| 11 | KIAA0828 |  |  | 1511 | 0.327 | 0.5612 | Yes |
| 12 | FNDC3B |  |  | 1760 | 0.303 | 0.5793 | Yes |
| 13 | HMGB1 |  |  | 1851 | 0.296 | 0.6045 | Yes |
| 14 | OFD1 |  |  | 2130 | 0.274 | 0.6183 | Yes |
| 15 | PRKAR1A |  |  | 2467 | 0.250 | 0.6269 | Yes |
| 16 | ITGA6 |  |  | 2714 | 0.235 | 0.6384 | Yes |
| 17 | MGA |  |  | 2734 | 0.234 | 0.6608 | Yes |
| 18 | NARG2 |  |  | 2781 | 0.231 | 0.6816 | Yes |
| 19 | RBBP6 |  |  | 3118 | 0.213 | 0.6865 | Yes |
| 20 | RIOK1 |  |  | 3694 | 0.187 | 0.6772 | No |
| 21 | TGOLN2 |  |  | 4193 | 0.165 | 0.6694 | No |
| 22 | KIAA0101 |  |  | 4566 | 0.151 | 0.6665 | No |
| 23 | ZRANB2 |  |  | 4914 | 0.140 | 0.6636 | No |
| 24 | GTPBP4 |  |  | 5796 | 0.117 | 0.6324 | No |
| 25 | SCLT1 |  |  | 6711 | 0.097 | 0.5976 | No |
| 26 | FAM49B |  |  | 6877 | 0.094 | 0.5990 | No |
| 27 | IKIP |  |  | 7458 | 0.083 | 0.5790 | No |
| 28 | TCEAL4 |  |  | 7536 | 0.081 | 0.5834 | No |
| 29 | RGL1 |  |  | 8184 | 0.070 | 0.5589 | No |
| 30 | GOLGB1 |  |  | 9029 | 0.056 | 0.5235 | No |
| 31 | PSEN1 |  |  | 9756 | 0.046 | 0.4927 | No |
| 32 | CREB3L1 |  |  | 10234 | 0.038 | 0.4734 | No |
| 33 | CTNNA1 |  |  | 11833 | 0.016 | 0.3973 | No |
| 34 | PROX1 |  |  | 12016 | 0.014 | 0.3898 | No |
| 35 | LARP4 |  |  | 15021 | -0.032 | 0.2470 | No |
| 36 | C11ORF46 |  |  | 18567 | -0.120 | 0.0867 | No |
| 37 | ITGA5 |  |  | 18655 | -0.124 | 0.0948 | No |
Table: GSEA details [plain text format]

  

Fig 2: HDACI\_COLON\_BUT30MIN\_DN: Random ES distribution      
 Gene set null distribution of ES for **HDACI\_COLON\_BUT30MIN\_DN**
