# Supplementary material for: The rapamycin-regulated gene expression signature determines prognosis for breast cancer
Source: Mol Cancer. 2009 Sep 24;8:75. doi: 10.1186/1476-4598-8-75 (PMC2761377; doi:10.1186/1476-4598-8-75)
Supplement: Additional file 2 — Gene set enrichment analysis of in vivo data, time series. The data provided represent the time series of GSEA. This compressed file contains "Time" shortcut file and "GSEA_time" folder. Clicking on "Time" shortcut opens the index file providing access to analysis files contained in the "GSEA_time" folder. [file 1476-4598-8-75-S2.zip › GSEA_time/HDACI_COLON_SUL30MIN_DN.html]

Details for gene set HDACI\_COLON\_SUL30MIN\_DN[GSEA]

|  || Dataset | gsea\_time\_collapsed |
| Phenotype | NoPhenotypeAvailable |
| Upregulated in class | na\_pos |
| GeneSet | HDACI\_COLON\_SUL30MIN\_DN |
| Enrichment Score (ES) | 0.65371144 |
| Normalized Enrichment Score (NES) | 1.7362764 |
| Nominal p-value | 0.0011185682 |
| FDR q-value | 0.014283473 |
| FWER p-Value | 0.481 |
Table: GSEA Results Summary

  

Fig 1: Enrichment plot: HDACI\_COLON\_SUL30MIN\_DN      
 Profile of the Running ES Score & Positions of GeneSet Members on the Rank Ordered List

  

| PROBE | GENE SYMBOL | GENE\_TITLE | RANK IN GENE LIST | RANK METRIC SCORE | RUNNING ES | CORE ENRICHMENT || 1 | FAM3C |  |  | 81 | 0.949 | 0.0895 | Yes |
| 2 | NBN |  |  | 159 | 0.786 | 0.1632 | Yes |
| 3 | TTC3 |  |  | 197 | 0.730 | 0.2334 | Yes |
| 4 | HP1BP3 |  |  | 211 | 0.711 | 0.3027 | Yes |
| 5 | TMF1 |  |  | 291 | 0.659 | 0.3638 | Yes |
| 6 | PHLDA1 |  |  | 377 | 0.595 | 0.4182 | Yes |
| 7 | PRPF38B |  |  | 472 | 0.543 | 0.4671 | Yes |
| 8 | BIRC6 |  |  | 691 | 0.476 | 0.5034 | Yes |
| 9 | MBNL2 |  |  | 718 | 0.470 | 0.5484 | Yes |
| 10 | DLG1 |  |  | 845 | 0.434 | 0.5851 | Yes |
| 11 | SSR1 |  |  | 1380 | 0.343 | 0.5929 | Yes |
| 12 | ZMYM3 |  |  | 1706 | 0.308 | 0.6074 | Yes |
| 13 | THRAP3 |  |  | 2371 | 0.257 | 0.6004 | Yes |
| 14 | PRKAR1A |  |  | 2467 | 0.250 | 0.6204 | Yes |
| 15 | ITGA6 |  |  | 2714 | 0.235 | 0.6316 | Yes |
| 16 | MGA |  |  | 2734 | 0.234 | 0.6537 | Yes |
| 17 | PPP2R2A |  |  | 3811 | 0.182 | 0.6193 | No |
| 18 | TGOLN2 |  |  | 4193 | 0.165 | 0.6170 | No |
| 19 | TXNDC1 |  |  | 4398 | 0.157 | 0.6226 | No |
| 20 | SKIV2L2 |  |  | 4616 | 0.150 | 0.6268 | No |
| 21 | AMMECR1 |  |  | 5297 | 0.129 | 0.6065 | No |
| 22 | ATP2A2 |  |  | 5878 | 0.115 | 0.5897 | No |
| 23 | GPIAP1 |  |  | 6165 | 0.109 | 0.5865 | No |
| 24 | SCLT1 |  |  | 6711 | 0.097 | 0.5696 | No |
| 25 | TMEM123 |  |  | 8798 | 0.060 | 0.4741 | No |
| 26 | GOLGB1 |  |  | 9029 | 0.056 | 0.4684 | No |
| 27 | C20ORF94 |  |  | 11095 | 0.026 | 0.3706 | No |
| 28 | SLC9A9 |  |  | 11753 | 0.017 | 0.3403 | No |
| 29 | MTBP |  |  | 11823 | 0.016 | 0.3386 | No |
| 30 | CTNNA1 |  |  | 11833 | 0.016 | 0.3398 | No |
| 31 | IDE |  |  | 13060 | -0.002 | 0.2804 | No |
| 32 | AASS |  |  | 14220 | -0.019 | 0.2259 | No |
| 33 | XPO7 |  |  | 15005 | -0.032 | 0.1909 | No |
| 34 | SNTB1 |  |  | 15017 | -0.032 | 0.1935 | No |
| 35 | LARP4 |  |  | 15021 | -0.032 | 0.1965 | No |
| 36 | PTPRN |  |  | 16319 | -0.055 | 0.1389 | No |
| 37 | STK11IP |  |  | 17971 | -0.098 | 0.0683 | No |
| 38 | MME |  |  | 20568 | -0.607 | 0.0018 | No |
Table: GSEA details [plain text format]

  

Fig 2: HDACI\_COLON\_SUL30MIN\_DN: Random ES distribution      
 Gene set null distribution of ES for **HDACI\_COLON\_SUL30MIN\_DN**
