# Supplementary material for: The rapamycin-regulated gene expression signature determines prognosis for breast cancer
Source: Mol Cancer. 2009 Sep 24;8:75. doi: 10.1186/1476-4598-8-75 (PMC2761377; doi:10.1186/1476-4598-8-75)
Supplement: Additional file 2 — Gene set enrichment analysis of in vivo data, time series. The data provided represent the time series of GSEA. This compressed file contains "Time" shortcut file and "GSEA_time" folder. Clicking on "Time" shortcut opens the index file providing access to analysis files contained in the "GSEA_time" folder. [file 1476-4598-8-75-S2.zip › GSEA_time/HINATA_NFKB_UP.html]

Details for gene set HINATA\_NFKB\_UP[GSEA]

|  || Dataset | gsea\_time\_collapsed |
| Phenotype | NoPhenotypeAvailable |
| Upregulated in class | na\_pos |
| GeneSet | HINATA\_NFKB\_UP |
| Enrichment Score (ES) | 0.59965324 |
| Normalized Enrichment Score (NES) | 1.7576963 |
| Nominal p-value | 0.0 |
| FDR q-value | 0.011560915 |
| FWER p-Value | 0.355 |
Table: GSEA Results Summary

  

Fig 1: Enrichment plot: HINATA\_NFKB\_UP      
 Profile of the Running ES Score & Positions of GeneSet Members on the Rank Ordered List

  

| PROBE | GENE SYMBOL | GENE\_TITLE | RANK IN GENE LIST | RANK METRIC SCORE | RUNNING ES | CORE ENRICHMENT || 1 | CCL2 |  |  | 11 | 1.629 | 0.0599 | Yes |
| 2 | IL8 |  |  | 13 | 1.561 | 0.1177 | Yes |
| 3 | WFDC2 |  |  | 21 | 1.357 | 0.1677 | Yes |
| 4 | KRT6B |  |  | 24 | 1.322 | 0.2166 | Yes |
| 5 | KRT14 |  |  | 35 | 1.225 | 0.2616 | Yes |
| 6 | ARF6 |  |  | 55 | 1.045 | 0.2995 | Yes |
| 7 | RARRES1 |  |  | 113 | 0.880 | 0.3293 | Yes |
| 8 | SOD2 |  |  | 128 | 0.857 | 0.3604 | Yes |
| 9 | ETS1 |  |  | 130 | 0.846 | 0.3917 | Yes |
| 10 | CXCL3 |  |  | 162 | 0.784 | 0.4193 | Yes |
| 11 | CXCL2 |  |  | 203 | 0.723 | 0.4442 | Yes |
| 12 | ARHGDIB |  |  | 251 | 0.681 | 0.4671 | Yes |
| 13 | PLAU |  |  | 302 | 0.647 | 0.4887 | Yes |
| 14 | CFLAR |  |  | 360 | 0.601 | 0.5082 | Yes |
| 15 | SAA1 |  |  | 405 | 0.579 | 0.5275 | Yes |
| 16 | EFNA1 |  |  | 480 | 0.541 | 0.5440 | Yes |
| 17 | CXCL11 |  |  | 499 | 0.535 | 0.5629 | Yes |
| 18 | IL15 |  |  | 529 | 0.522 | 0.5809 | Yes |
| 19 | TAF15 |  |  | 762 | 0.460 | 0.5866 | Yes |
| 20 | ERCC1 |  |  | 829 | 0.438 | 0.5997 | Yes |
| 21 | ADAMTS3 |  |  | 1376 | 0.343 | 0.5858 | No |
| 22 | RABEP1 |  |  | 1485 | 0.331 | 0.5928 | No |
| 23 | CDH2 |  |  | 1872 | 0.294 | 0.5848 | No |
| 24 | PLAT |  |  | 2048 | 0.280 | 0.5867 | No |
| 25 | KRT7 |  |  | 2115 | 0.276 | 0.5937 | No |
| 26 | RAC1 |  |  | 2228 | 0.266 | 0.5981 | No |
| 27 | ICAM1 |  |  | 3140 | 0.212 | 0.5615 | No |
| 28 | ABCC1 |  |  | 3799 | 0.182 | 0.5362 | No |
| 29 | LITAF |  |  | 3807 | 0.182 | 0.5426 | No |
| 30 | IFNAR2 |  |  | 3820 | 0.182 | 0.5487 | No |
| 31 | PTX3 |  |  | 3912 | 0.177 | 0.5509 | No |
| 32 | MAP1B |  |  | 3991 | 0.174 | 0.5535 | No |
| 33 | TIAM1 |  |  | 4045 | 0.172 | 0.5573 | No |
| 34 | MAPK9 |  |  | 4818 | 0.143 | 0.5249 | No |
| 35 | IL6 |  |  | 4954 | 0.138 | 0.5235 | No |
| 36 | MMP1 |  |  | 5254 | 0.130 | 0.5137 | No |
| 37 | CXCL1 |  |  | 5663 | 0.120 | 0.4983 | No |
| 38 | TNC |  |  | 5876 | 0.116 | 0.4922 | No |
| 39 | CSF2 |  |  | 5899 | 0.115 | 0.4954 | No |
| 40 | IER3 |  |  | 6090 | 0.111 | 0.4903 | No |
| 41 | CCL5 |  |  | 6144 | 0.109 | 0.4917 | No |
| 42 | IL1RN |  |  | 6333 | 0.105 | 0.4864 | No |
| 43 | IL1A |  |  | 6347 | 0.105 | 0.4897 | No |
| 44 | SEC24A |  |  | 6529 | 0.101 | 0.4846 | No |
| 45 | ESM1 |  |  | 6714 | 0.097 | 0.4792 | No |
| 46 | SPTBN1 |  |  | 6719 | 0.097 | 0.4826 | No |
| 47 | TNFAIP6 |  |  | 6873 | 0.094 | 0.4787 | No |
| 48 | CDH3 |  |  | 6883 | 0.093 | 0.4817 | No |
| 49 | CPNE5 |  |  | 6999 | 0.091 | 0.4795 | No |
| 50 | AP2B1 |  |  | 7048 | 0.090 | 0.4805 | No |
| 51 | TNFAIP2 |  |  | 7179 | 0.088 | 0.4774 | No |
| 52 | UTX |  |  | 7186 | 0.088 | 0.4804 | No |
| 53 | GABBR1 |  |  | 7468 | 0.083 | 0.4697 | No |
| 54 | STAT5A |  |  | 7862 | 0.075 | 0.4533 | No |
| 55 | NFKB1 |  |  | 7978 | 0.073 | 0.4504 | No |
| 56 | CD9 |  |  | 8510 | 0.064 | 0.4269 | No |
| 57 | GADD45A |  |  | 8579 | 0.063 | 0.4260 | No |
| 58 | EDN1 |  |  | 8935 | 0.058 | 0.4108 | No |
| 59 | RELA |  |  | 9068 | 0.056 | 0.4064 | No |
| 60 | GCH1 |  |  | 9218 | 0.054 | 0.4012 | No |
| 61 | CXCL6 |  |  | 9436 | 0.050 | 0.3924 | No |
| 62 | GPR37 |  |  | 9835 | 0.044 | 0.3747 | No |
| 63 | FN1 |  |  | 9897 | 0.043 | 0.3733 | No |
| 64 | NPR1 |  |  | 9980 | 0.042 | 0.3709 | No |
| 65 | TNF |  |  | 10008 | 0.042 | 0.3711 | No |
| 66 | IL7R |  |  | 10261 | 0.038 | 0.3602 | No |
| 67 | SLC7A2 |  |  | 10780 | 0.030 | 0.3361 | No |
| 68 | KRT10 |  |  | 11399 | 0.022 | 0.3068 | No |
| 69 | NFKB2 |  |  | 11438 | 0.021 | 0.3057 | No |
| 70 | TNIP1 |  |  | 11755 | 0.017 | 0.2909 | No |
| 71 | CYP27B1 |  |  | 11787 | 0.017 | 0.2900 | No |
| 72 | MMP14 |  |  | 11968 | 0.014 | 0.2818 | No |
| 73 | CCNB1 |  |  | 12244 | 0.011 | 0.2687 | No |
| 74 | IRF1 |  |  | 12849 | 0.001 | 0.2393 | No |
| 75 | CDKN1A |  |  | 12863 | 0.001 | 0.2387 | No |
| 76 | CCL20 |  |  | 12960 | -0.000 | 0.2340 | No |
| 77 | CXCL5 |  |  | 13115 | -0.003 | 0.2266 | No |
| 78 | CTSE |  |  | 13249 | -0.005 | 0.2203 | No |
| 79 | OLR1 |  |  | 13378 | -0.007 | 0.2143 | No |
| 80 | IL1B |  |  | 13976 | -0.016 | 0.1858 | No |
| 81 | RAC2 |  |  | 14529 | -0.024 | 0.1598 | No |
| 82 | MMP9 |  |  | 14832 | -0.029 | 0.1461 | No |
| 83 | MYBL1 |  |  | 15250 | -0.036 | 0.1271 | No |
| 84 | FGF5 |  |  | 15342 | -0.037 | 0.1240 | No |
| 85 | HGFAC |  |  | 15867 | -0.046 | 0.1002 | No |
| 86 | WNT5A |  |  | 16212 | -0.053 | 0.0854 | No |
| 87 | CD83 |  |  | 16957 | -0.069 | 0.0516 | No |
| 88 | MSC |  |  | 17271 | -0.077 | 0.0392 | No |
| 89 | BTN3A3 |  |  | 17395 | -0.081 | 0.0362 | No |
| 90 | GBP1 |  |  | 17410 | -0.081 | 0.0385 | No |
| 91 | CXCL10 |  |  | 17747 | -0.091 | 0.0255 | No |
| 92 | FGF2 |  |  | 17847 | -0.094 | 0.0242 | No |
| 93 | RELB |  |  | 18126 | -0.103 | 0.0144 | No |
| 94 | BCL2A1 |  |  | 18179 | -0.105 | 0.0158 | No |
| 95 | SERPINE1 |  |  | 18412 | -0.114 | 0.0087 | No |
| 96 | TAP1 |  |  | 18553 | -0.120 | 0.0063 | No |
| 97 | SPRR1B |  |  | 19001 | -0.141 | -0.0102 | No |
| 98 | SOD1 |  |  | 19187 | -0.152 | -0.0136 | No |
| 99 | RPA2 |  |  | 19317 | -0.162 | -0.0139 | No |
| 100 | SELE |  |  | 19762 | -0.207 | -0.0279 | No |
| 101 | XPC |  |  | 19943 | -0.231 | -0.0281 | No |
| 102 | GTF2H4 |  |  | 19969 | -0.235 | -0.0206 | No |
| 103 | PNRC1 |  |  | 20123 | -0.269 | -0.0181 | No |
| 104 | SERPINB2 |  |  | 20152 | -0.277 | -0.0092 | No |
| 105 | BMP2 |  |  | 20258 | -0.315 | -0.0026 | No |
| 106 | ITGB6 |  |  | 20539 | -0.526 | 0.0032 | No |
Table: GSEA details [plain text format]

  

Fig 2: HINATA\_NFKB\_UP: Random ES distribution      
 Gene set null distribution of ES for **HINATA\_NFKB\_UP**
