# Supplementary material for: The rapamycin-regulated gene expression signature determines prognosis for breast cancer
Source: Mol Cancer. 2009 Sep 24;8:75. doi: 10.1186/1476-4598-8-75 (PMC2761377; doi:10.1186/1476-4598-8-75)
Supplement: Additional file 2 — Gene set enrichment analysis of in vivo data, time series. The data provided represent the time series of GSEA. This compressed file contains "Time" shortcut file and "GSEA_time" folder. Clicking on "Time" shortcut opens the index file providing access to analysis files contained in the "GSEA_time" folder. [file 1476-4598-8-75-S2.zip › GSEA_time/HPV31_DN.html]

Details for gene set HPV31\_DN[GSEA]

|  || Dataset | gsea\_time\_collapsed |
| Phenotype | NoPhenotypeAvailable |
| Upregulated in class | na\_neg |
| GeneSet | HPV31\_DN |
| Enrichment Score (ES) | -0.34358576 |
| Normalized Enrichment Score (NES) | -1.3059479 |
| Nominal p-value | 0.055555556 |
| FDR q-value | 0.31537333 |
| FWER p-Value | 1.0 |
Table: GSEA Results Summary

  

Fig 1: Enrichment plot: HPV31\_DN      
 Profile of the Running ES Score & Positions of GeneSet Members on the Rank Ordered List

  

| PROBE | GENE SYMBOL | GENE\_TITLE | RANK IN GENE LIST | RANK METRIC SCORE | RUNNING ES | CORE ENRICHMENT || 1 | IFITM1 |  |  | 275 | 0.669 | 0.0691 | No |
| 2 | DMKN |  |  | 547 | 0.515 | 0.1193 | No |
| 3 | SNRPA1 |  |  | 874 | 0.428 | 0.1561 | No |
| 4 | DSP |  |  | 2398 | 0.255 | 0.1135 | No |
| 5 | DEGS1 |  |  | 2553 | 0.245 | 0.1362 | No |
| 6 | UBE3B |  |  | 2736 | 0.234 | 0.1561 | No |
| 7 | THRB |  |  | 2740 | 0.234 | 0.1848 | No |
| 8 | SFN |  |  | 3186 | 0.210 | 0.1890 | No |
| 9 | CBFA2T2 |  |  | 3391 | 0.200 | 0.2037 | No |
| 10 | NMT2 |  |  | 4000 | 0.174 | 0.1955 | No |
| 11 | STAT1 |  |  | 4141 | 0.167 | 0.2093 | No |
| 12 | OAS2 |  |  | 5619 | 0.121 | 0.1524 | No |
| 13 | ENTPD3 |  |  | 5988 | 0.113 | 0.1484 | No |
| 14 | MXD1 |  |  | 6428 | 0.103 | 0.1398 | No |
| 15 | DSC2 |  |  | 6517 | 0.101 | 0.1480 | No |
| 16 | AR |  |  | 7692 | 0.078 | 0.1005 | No |
| 17 | PHF7 |  |  | 7813 | 0.076 | 0.1040 | No |
| 18 | LAMA3 |  |  | 8659 | 0.062 | 0.0706 | No |
| 19 | CTSL2 |  |  | 9243 | 0.053 | 0.0488 | No |
| 20 | PRIM1 |  |  | 9406 | 0.051 | 0.0472 | No |
| 21 | KLK7 |  |  | 10299 | 0.038 | 0.0084 | No |
| 22 | LRP1 |  |  | 10328 | 0.037 | 0.0116 | No |
| 23 | DSG1 |  |  | 11221 | 0.025 | -0.0288 | No |
| 24 | GPA33 |  |  | 11276 | 0.024 | -0.0284 | No |
| 25 | BPGM |  |  | 11483 | 0.021 | -0.0359 | No |
| 26 | ACVR2B |  |  | 12155 | 0.012 | -0.0671 | No |
| 27 | SAMD9 |  |  | 12726 | 0.002 | -0.0945 | No |
| 28 | TNRC6C |  |  | 12739 | 0.002 | -0.0948 | No |
| 29 | C13ORF16 |  |  | 13166 | -0.004 | -0.1151 | No |
| 30 | SMCP |  |  | 13606 | -0.010 | -0.1352 | No |
| 31 | LYST |  |  | 14862 | -0.030 | -0.1925 | No |
| 32 | HMGCS1 |  |  | 16931 | -0.068 | -0.2847 | No |
| 33 | SPRR1A |  |  | 17631 | -0.088 | -0.3079 | No |
| 34 | MX2 |  |  | 17855 | -0.094 | -0.3071 | No |
| 35 | PLA2G1B |  |  | 18298 | -0.110 | -0.3151 | No |
| 36 | PTPRU |  |  | 18384 | -0.113 | -0.3054 | No |
| 37 | TRIB1 |  |  | 18829 | -0.132 | -0.3107 | No |
| 38 | KLK6 |  |  | 19203 | -0.154 | -0.3098 | No |
| 39 | IFIT1 |  |  | 19898 | -0.225 | -0.3158 | Yes |
| 40 | IFI27 |  |  | 19905 | -0.226 | -0.2883 | Yes |
| 41 | SERPINB2 |  |  | 20152 | -0.277 | -0.2661 | Yes |
| 42 | MX1 |  |  | 20243 | -0.310 | -0.2322 | Yes |
| 43 | TRIM22 |  |  | 20245 | -0.311 | -0.1939 | Yes |
| 44 | SULT2B1 |  |  | 20352 | -0.355 | -0.1553 | Yes |
| 45 | IL1R2 |  |  | 20419 | -0.400 | -0.1092 | Yes |
| 46 | SLPI |  |  | 20486 | -0.458 | -0.0560 | Yes |
| 47 | MVD |  |  | 20520 | -0.501 | 0.0041 | Yes |
Table: GSEA details [plain text format]

  

Fig 2: HPV31\_DN: Random ES distribution      
 Gene set null distribution of ES for **HPV31\_DN**
