# Supplementary material for: The rapamycin-regulated gene expression signature determines prognosis for breast cancer
Source: Mol Cancer. 2009 Sep 24;8:75. doi: 10.1186/1476-4598-8-75 (PMC2761377; doi:10.1186/1476-4598-8-75)
Supplement: Additional file 2 — Gene set enrichment analysis of in vivo data, time series. The data provided represent the time series of GSEA. This compressed file contains "Time" shortcut file and "GSEA_time" folder. Clicking on "Time" shortcut opens the index file providing access to analysis files contained in the "GSEA_time" folder. [file 1476-4598-8-75-S2.zip › GSEA_time/HYPOXIA_REG_UP.html]

Details for gene set HYPOXIA\_REG\_UP[GSEA]

|  || Dataset | gsea\_time\_collapsed |
| Phenotype | NoPhenotypeAvailable |
| Upregulated in class | na\_pos |
| GeneSet | HYPOXIA\_REG\_UP |
| Enrichment Score (ES) | 0.7031822 |
| Normalized Enrichment Score (NES) | 1.862042 |
| Nominal p-value | 0.0 |
| FDR q-value | 0.0029367101 |
| FWER p-Value | 0.038 |
Table: GSEA Results Summary

  

Fig 1: Enrichment plot: HYPOXIA\_REG\_UP      
 Profile of the Running ES Score & Positions of GeneSet Members on the Rank Ordered List

  

| PROBE | GENE SYMBOL | GENE\_TITLE | RANK IN GENE LIST | RANK METRIC SCORE | RUNNING ES | CORE ENRICHMENT || 1 | SLC2A1 |  |  | 96 | 0.908 | 0.0806 | Yes |
| 2 | PGK1 |  |  | 101 | 0.899 | 0.1647 | Yes |
| 3 | GPRC5A |  |  | 151 | 0.800 | 0.2374 | Yes |
| 4 | VEGF |  |  | 252 | 0.681 | 0.2964 | Yes |
| 5 | STC2 |  |  | 256 | 0.679 | 0.3599 | Yes |
| 6 | F3 |  |  | 371 | 0.596 | 0.4103 | Yes |
| 7 | EFNA1 |  |  | 480 | 0.541 | 0.4558 | Yes |
| 8 | TGFA |  |  | 613 | 0.494 | 0.4957 | Yes |
| 9 | C10ORF10 |  |  | 658 | 0.482 | 0.5388 | Yes |
| 10 | DUSP1 |  |  | 672 | 0.479 | 0.5831 | Yes |
| 11 | ADM |  |  | 932 | 0.417 | 0.6097 | Yes |
| 12 | CDKN1B |  |  | 1916 | 0.291 | 0.5892 | Yes |
| 13 | FUS |  |  | 1973 | 0.286 | 0.6133 | Yes |
| 14 | BNIP3 |  |  | 2049 | 0.280 | 0.6359 | Yes |
| 15 | CCNG2 |  |  | 2273 | 0.263 | 0.6497 | Yes |
| 16 | P4HA2 |  |  | 2642 | 0.239 | 0.6542 | Yes |
| 17 | NFIL3 |  |  | 3085 | 0.214 | 0.6529 | Yes |
| 18 | ADFP |  |  | 3492 | 0.194 | 0.6514 | Yes |
| 19 | HNRPA1 |  |  | 3607 | 0.190 | 0.6636 | Yes |
| 20 | PDGFB |  |  | 3659 | 0.189 | 0.6788 | Yes |
| 21 | SLC16A3 |  |  | 3800 | 0.182 | 0.6891 | Yes |
| 22 | JMJD1A |  |  | 3860 | 0.180 | 0.7032 | Yes |
| 23 | PFKP |  |  | 4731 | 0.146 | 0.6746 | No |
| 24 | PHC2 |  |  | 5302 | 0.129 | 0.6590 | No |
| 25 | PFKFB4 |  |  | 6375 | 0.104 | 0.6166 | No |
| 26 | SAP30 |  |  | 7828 | 0.076 | 0.5531 | No |
| 27 | MUC1 |  |  | 8233 | 0.069 | 0.5399 | No |
| 28 | RASGRP1 |  |  | 10275 | 0.038 | 0.4443 | No |
| 29 | PTDSR |  |  | 10810 | 0.030 | 0.4212 | No |
| 30 | ADORA2B |  |  | 10853 | 0.029 | 0.4219 | No |
| 31 | FOSL2 |  |  | 12117 | 0.012 | 0.3616 | No |
| 32 | BHLHB2 |  |  | 12438 | 0.008 | 0.3467 | No |
| 33 | PPP1R3C |  |  | 13583 | -0.010 | 0.2920 | No |
| 34 | JUNB |  |  | 13719 | -0.012 | 0.2866 | No |
| 35 | ALDOA |  |  | 18247 | -0.108 | 0.0766 | No |
| 36 | SERPINE1 |  |  | 18412 | -0.114 | 0.0793 | No |
| 37 | DDIT4 |  |  | 18623 | -0.123 | 0.0806 | No |
| 38 | SLC7A5 |  |  | 19380 | -0.168 | 0.0596 | No |
Table: GSEA details [plain text format]

  

Fig 2: HYPOXIA\_REG\_UP: Random ES distribution      
 Gene set null distribution of ES for **HYPOXIA\_REG\_UP**
