# Supplementary material for: The rapamycin-regulated gene expression signature determines prognosis for breast cancer
Source: Mol Cancer. 2009 Sep 24;8:75. doi: 10.1186/1476-4598-8-75 (PMC2761377; doi:10.1186/1476-4598-8-75)
Supplement: Additional file 2 — Gene set enrichment analysis of in vivo data, time series. The data provided represent the time series of GSEA. This compressed file contains "Time" shortcut file and "GSEA_time" folder. Clicking on "Time" shortcut opens the index file providing access to analysis files contained in the "GSEA_time" folder. [file 1476-4598-8-75-S2.zip › GSEA_time/HYPOXIA_REVIEW.html]

Details for gene set HYPOXIA\_REVIEW[GSEA]

|  || Dataset | gsea\_time\_collapsed |
| Phenotype | NoPhenotypeAvailable |
| Upregulated in class | na\_pos |
| GeneSet | HYPOXIA\_REVIEW |
| Enrichment Score (ES) | 0.6603841 |
| Normalized Enrichment Score (NES) | 1.8946134 |
| Nominal p-value | 0.0 |
| FDR q-value | 0.0021255754 |
| FWER p-Value | 0.019 |
Table: GSEA Results Summary

  

Fig 1: Enrichment plot: HYPOXIA\_REVIEW      
 Profile of the Running ES Score & Positions of GeneSet Members on the Rank Ordered List

  

| PROBE | GENE SYMBOL | GENE\_TITLE | RANK IN GENE LIST | RANK METRIC SCORE | RUNNING ES | CORE ENRICHMENT || 1 | CCL2 |  |  | 11 | 1.629 | 0.0603 | Yes |
| 2 | STC1 |  |  | 12 | 1.592 | 0.1198 | Yes |
| 3 | IL8 |  |  | 13 | 1.561 | 0.1782 | Yes |
| 4 | SAT1 |  |  | 58 | 1.024 | 0.2143 | Yes |
| 5 | TAGLN |  |  | 95 | 0.911 | 0.2466 | Yes |
| 6 | SLC2A1 |  |  | 96 | 0.908 | 0.2805 | Yes |
| 7 | PGK1 |  |  | 101 | 0.899 | 0.3139 | Yes |
| 8 | TXN |  |  | 178 | 0.760 | 0.3387 | Yes |
| 9 | CP |  |  | 189 | 0.741 | 0.3658 | Yes |
| 10 | FTL |  |  | 193 | 0.733 | 0.3931 | Yes |
| 11 | VEGF |  |  | 252 | 0.681 | 0.4157 | Yes |
| 12 | SLC2A3 |  |  | 278 | 0.668 | 0.4395 | Yes |
| 13 | HK2 |  |  | 330 | 0.621 | 0.4602 | Yes |
| 14 | BIK |  |  | 340 | 0.613 | 0.4826 | Yes |
| 15 | HDAC9 |  |  | 364 | 0.600 | 0.5039 | Yes |
| 16 | F3 |  |  | 371 | 0.596 | 0.5259 | Yes |
| 17 | ENO1 |  |  | 410 | 0.577 | 0.5456 | Yes |
| 18 | TGFA |  |  | 613 | 0.494 | 0.5542 | Yes |
| 19 | BNIP3L |  |  | 873 | 0.428 | 0.5576 | Yes |
| 20 | HIF1A |  |  | 889 | 0.425 | 0.5727 | Yes |
| 21 | ADM |  |  | 932 | 0.417 | 0.5863 | Yes |
| 22 | AK3 |  |  | 1022 | 0.401 | 0.5969 | Yes |
| 23 | VIM |  |  | 1054 | 0.395 | 0.6102 | Yes |
| 24 | PLAUR |  |  | 1102 | 0.387 | 0.6224 | Yes |
| 25 | JUN |  |  | 1522 | 0.326 | 0.6141 | Yes |
| 26 | IGFBP3 |  |  | 1528 | 0.326 | 0.6260 | Yes |
| 27 | TGM2 |  |  | 1588 | 0.320 | 0.6351 | Yes |
| 28 | CDKN1B |  |  | 1916 | 0.291 | 0.6301 | Yes |
| 29 | P4HA1 |  |  | 1987 | 0.285 | 0.6373 | Yes |
| 30 | BNIP3 |  |  | 2049 | 0.280 | 0.6448 | Yes |
| 31 | CCNG2 |  |  | 2273 | 0.263 | 0.6437 | Yes |
| 32 | LRP8 |  |  | 2444 | 0.252 | 0.6448 | Yes |
| 33 | CA9 |  |  | 2610 | 0.241 | 0.6458 | Yes |
| 34 | CD99 |  |  | 2687 | 0.237 | 0.6510 | Yes |
| 35 | PGF |  |  | 2843 | 0.228 | 0.6519 | Yes |
| 36 | TF |  |  | 2845 | 0.227 | 0.6604 | Yes |
| 37 | PDGFB |  |  | 3659 | 0.189 | 0.6278 | No |
| 38 | PFKP |  |  | 4731 | 0.146 | 0.5811 | No |
| 39 | IL6 |  |  | 4954 | 0.138 | 0.5755 | No |
| 40 | TGFB3 |  |  | 5054 | 0.136 | 0.5757 | No |
| 41 | IGFBP1 |  |  | 5212 | 0.131 | 0.5730 | No |
| 42 | TFRC |  |  | 5832 | 0.116 | 0.5472 | No |
| 43 | PTGS2 |  |  | 6233 | 0.107 | 0.5317 | No |
| 44 | CA12 |  |  | 6270 | 0.106 | 0.5339 | No |
| 45 | XRCC6 |  |  | 6602 | 0.100 | 0.5215 | No |
| 46 | PKM2 |  |  | 6653 | 0.098 | 0.5227 | No |
| 47 | TFF3 |  |  | 7274 | 0.086 | 0.4957 | No |
| 48 | EPAS1 |  |  | 7386 | 0.084 | 0.4935 | No |
| 49 | XRCC5 |  |  | 7454 | 0.083 | 0.4933 | No |
| 50 | PRPS1 |  |  | 7885 | 0.075 | 0.4751 | No |
| 51 | SPP1 |  |  | 7970 | 0.073 | 0.4738 | No |
| 52 | NFKB1 |  |  | 7978 | 0.073 | 0.4761 | No |
| 53 | LDHA |  |  | 8499 | 0.064 | 0.4532 | No |
| 54 | ANGPT2 |  |  | 8923 | 0.058 | 0.4348 | No |
| 55 | EDN1 |  |  | 8935 | 0.058 | 0.4364 | No |
| 56 | HGF |  |  | 8955 | 0.058 | 0.4377 | No |
| 57 | EDN2 |  |  | 9166 | 0.054 | 0.4295 | No |
| 58 | FLT1 |  |  | 10090 | 0.041 | 0.3860 | No |
| 59 | FOS |  |  | 11075 | 0.026 | 0.3391 | No |
| 60 | IGF2 |  |  | 11640 | 0.019 | 0.3123 | No |
| 61 | DDIT3 |  |  | 11873 | 0.015 | 0.3015 | No |
| 62 | BHLHB2 |  |  | 12438 | 0.008 | 0.2744 | No |
| 63 | FGF3 |  |  | 12664 | 0.004 | 0.2635 | No |
| 64 | CDKN1A |  |  | 12863 | 0.001 | 0.2539 | No |
| 65 | COL5A1 |  |  | 13102 | -0.003 | 0.2424 | No |
| 66 | TEK |  |  | 14907 | -0.030 | 0.1557 | No |
| 67 | TGFB1 |  |  | 15318 | -0.037 | 0.1371 | No |
| 68 | EPO |  |  | 15935 | -0.047 | 0.1088 | No |
| 69 | GAPDH |  |  | 16748 | -0.064 | 0.0717 | No |
| 70 | MMP13 |  |  | 16772 | -0.065 | 0.0730 | No |
| 71 | ENPEP |  |  | 17013 | -0.070 | 0.0639 | No |
| 72 | APEX1 |  |  | 18220 | -0.106 | 0.0091 | No |
| 73 | ALDOA |  |  | 18247 | -0.108 | 0.0119 | No |
| 74 | L1CAM |  |  | 18518 | -0.118 | 0.0031 | No |
| 75 | RP1 |  |  | 19199 | -0.153 | -0.0243 | No |
| 76 | PFKL |  |  | 19334 | -0.163 | -0.0247 | No |
| 77 | TH |  |  | 19681 | -0.198 | -0.0342 | No |
| 78 | MIF |  |  | 19823 | -0.215 | -0.0330 | No |
| 79 | HK1 |  |  | 20105 | -0.264 | -0.0368 | No |
| 80 | HMOX1 |  |  | 20558 | -0.570 | -0.0376 | No |
| 81 | IGFBP2 |  |  | 20605 | -1.066 | 0.0000 | No |
Table: GSEA details [plain text format]

  

Fig 2: HYPOXIA\_REVIEW: Random ES distribution      
 Gene set null distribution of ES for **HYPOXIA\_REVIEW**
