# Supplementary material for: The rapamycin-regulated gene expression signature determines prognosis for breast cancer
Source: Mol Cancer. 2009 Sep 24;8:75. doi: 10.1186/1476-4598-8-75 (PMC2761377; doi:10.1186/1476-4598-8-75)
Supplement: Additional file 2 — Gene set enrichment analysis of in vivo data, time series. The data provided represent the time series of GSEA. This compressed file contains "Time" shortcut file and "GSEA_time" folder. Clicking on "Time" shortcut opens the index file providing access to analysis files contained in the "GSEA_time" folder. [file 1476-4598-8-75-S2.zip › GSEA_time/IDX_TSA_UP_CLUSTER5.html]

Details for gene set IDX\_TSA\_UP\_CLUSTER5[GSEA]

|  || Dataset | gsea\_time\_collapsed |
| Phenotype | NoPhenotypeAvailable |
| Upregulated in class | na\_neg |
| GeneSet | IDX\_TSA\_UP\_CLUSTER5 |
| Enrichment Score (ES) | -0.32272777 |
| Normalized Enrichment Score (NES) | -1.4329728 |
| Nominal p-value | 0.0 |
| FDR q-value | 0.26509914 |
| FWER p-Value | 0.998 |
Table: GSEA Results Summary

  

Fig 1: Enrichment plot: IDX\_TSA\_UP\_CLUSTER5      
 Profile of the Running ES Score & Positions of GeneSet Members on the Rank Ordered List

  

| PROBE | GENE SYMBOL | GENE\_TITLE | RANK IN GENE LIST | RANK METRIC SCORE | RUNNING ES | CORE ENRICHMENT || 1 | DNAJB9 |  |  | 422 | 0.569 | 0.0111 | No |
| 2 | CHP |  |  | 668 | 0.480 | 0.0259 | No |
| 3 | CBR3 |  |  | 1257 | 0.362 | 0.0174 | No |
| 4 | PIM3 |  |  | 1279 | 0.358 | 0.0363 | No |
| 5 | MGST3 |  |  | 1349 | 0.347 | 0.0522 | No |
| 6 | MKNK2 |  |  | 1388 | 0.342 | 0.0695 | No |
| 7 | GPI |  |  | 1486 | 0.331 | 0.0831 | No |
| 8 | NDUFA8 |  |  | 1673 | 0.311 | 0.0914 | No |
| 9 | USMG5 |  |  | 1796 | 0.300 | 0.1021 | No |
| 10 | ARL6IP2 |  |  | 1809 | 0.299 | 0.1182 | No |
| 11 | MRPS2 |  |  | 2124 | 0.275 | 0.1182 | No |
| 12 | COQ9 |  |  | 2288 | 0.262 | 0.1248 | No |
| 13 | ACSL1 |  |  | 2341 | 0.259 | 0.1367 | No |
| 14 | MRPL12 |  |  | 2462 | 0.250 | 0.1448 | No |
| 15 | PSMA1 |  |  | 2524 | 0.246 | 0.1555 | No |
| 16 | MAPK6 |  |  | 2603 | 0.241 | 0.1652 | No |
| 17 | FAM73B |  |  | 2671 | 0.238 | 0.1751 | No |
| 18 | PLA2G12A |  |  | 2917 | 0.224 | 0.1756 | No |
| 19 | COX17 |  |  | 3094 | 0.214 | 0.1790 | No |
| 20 | C20ORF45 |  |  | 3120 | 0.213 | 0.1896 | No |
| 21 | C6ORF72 |  |  | 3310 | 0.203 | 0.1917 | No |
| 22 | PTRF |  |  | 3890 | 0.178 | 0.1734 | No |
| 23 | AK2 |  |  | 3982 | 0.174 | 0.1787 | No |
| 24 | TOMM40 |  |  | 4173 | 0.166 | 0.1786 | No |
| 25 | TIMM17A |  |  | 4221 | 0.164 | 0.1855 | No |
| 26 | ACADM |  |  | 4321 | 0.160 | 0.1896 | No |
| 27 | TMEM97 |  |  | 4713 | 0.147 | 0.1787 | No |
| 28 | UQCR |  |  | 4860 | 0.142 | 0.1794 | No |
| 29 | COX6A1 |  |  | 5235 | 0.131 | 0.1685 | No |
| 30 | GRPEL1 |  |  | 5533 | 0.123 | 0.1609 | No |
| 31 | FDX1 |  |  | 5768 | 0.118 | 0.1560 | No |
| 32 | JAGN1 |  |  | 6146 | 0.109 | 0.1437 | No |
| 33 | IFNGR1 |  |  | 6221 | 0.108 | 0.1461 | No |
| 34 | GNPAT |  |  | 6645 | 0.099 | 0.1309 | No |
| 35 | GK |  |  | 6675 | 0.098 | 0.1350 | No |
| 36 | C18ORF8 |  |  | 6704 | 0.098 | 0.1391 | No |
| 37 | ZNRF2 |  |  | 6806 | 0.095 | 0.1394 | No |
| 38 | LPIN1 |  |  | 6890 | 0.093 | 0.1406 | No |
| 39 | SORBS1 |  |  | 7041 | 0.090 | 0.1383 | No |
| 40 | PEX14 |  |  | 7106 | 0.089 | 0.1402 | No |
| 41 | TXNDC14 |  |  | 7475 | 0.083 | 0.1268 | No |
| 42 | TIMM9 |  |  | 7494 | 0.082 | 0.1305 | No |
| 43 | COX4NB |  |  | 7721 | 0.077 | 0.1238 | No |
| 44 | LMAN2 |  |  | 7783 | 0.076 | 0.1251 | No |
| 45 | PRPS1 |  |  | 7885 | 0.075 | 0.1243 | No |
| 46 | RPP14 |  |  | 7916 | 0.074 | 0.1270 | No |
| 47 | BCL2L13 |  |  | 9180 | 0.054 | 0.0684 | No |
| 48 | CRAT |  |  | 9301 | 0.052 | 0.0655 | No |
| 49 | ADAM12 |  |  | 9345 | 0.052 | 0.0663 | No |
| 50 | ATP5G1 |  |  | 9663 | 0.047 | 0.0534 | No |
| 51 | RREB1 |  |  | 9683 | 0.047 | 0.0551 | No |
| 52 | IVD |  |  | 10027 | 0.042 | 0.0407 | No |
| 53 | PSMA5 |  |  | 10237 | 0.038 | 0.0327 | No |
| 54 | FAM82C |  |  | 10327 | 0.037 | 0.0304 | No |
| 55 | ABHD5 |  |  | 10366 | 0.037 | 0.0306 | No |
| 56 | TIMM8A |  |  | 10428 | 0.036 | 0.0296 | No |
| 57 | ETFB |  |  | 11137 | 0.026 | -0.0035 | No |
| 58 | MTX2 |  |  | 11433 | 0.021 | -0.0167 | No |
| 59 | TBL2 |  |  | 11635 | 0.019 | -0.0255 | No |
| 60 | ORMDL3 |  |  | 12012 | 0.014 | -0.0431 | No |
| 61 | LRRC59 |  |  | 12741 | 0.002 | -0.0784 | No |
| 62 | PDCD8 |  |  | 12867 | 0.001 | -0.0845 | No |
| 63 | BRP44 |  |  | 13036 | -0.002 | -0.0926 | No |
| 64 | LOC642393 |  |  | 13295 | -0.006 | -0.1048 | No |
| 65 | GPHN |  |  | 13442 | -0.008 | -0.1115 | No |
| 66 | ESRRA |  |  | 13733 | -0.012 | -0.1250 | No |
| 67 | KIAA1161 |  |  | 14375 | -0.022 | -0.1551 | No |
| 68 | PDIA6 |  |  | 14486 | -0.023 | -0.1591 | No |
| 69 | PRDX3 |  |  | 15215 | -0.035 | -0.1927 | No |
| 70 | TYSND1 |  |  | 15357 | -0.038 | -0.1975 | No |
| 71 | RASD1 |  |  | 15554 | -0.040 | -0.2048 | No |
| 72 | MRPS26 |  |  | 15997 | -0.048 | -0.2237 | No |
| 73 | PHB |  |  | 17102 | -0.073 | -0.2735 | No |
| 74 | TALDO1 |  |  | 17228 | -0.076 | -0.2753 | No |
| 75 | C22ORF13 |  |  | 17249 | -0.076 | -0.2721 | No |
| 76 | PTGES2 |  |  | 17959 | -0.098 | -0.3012 | No |
| 77 | SIPA1 |  |  | 17991 | -0.099 | -0.2972 | No |
| 78 | SDHD |  |  | 18044 | -0.101 | -0.2942 | No |
| 79 | ALDOA |  |  | 18247 | -0.108 | -0.2980 | No |
| 80 | TIMM23 |  |  | 18252 | -0.108 | -0.2922 | No |
| 81 | NDUFB10 |  |  | 18287 | -0.109 | -0.2878 | No |
| 82 | MRPL15 |  |  | 18855 | -0.134 | -0.3080 | No |
| 83 | C2ORF7 |  |  | 19056 | -0.144 | -0.3097 | No |
| 84 | PEX11A |  |  | 19216 | -0.154 | -0.3089 | No |
| 85 | BCAP31 |  |  | 19501 | -0.179 | -0.3128 | Yes |
| 86 | HBLD1 |  |  | 19598 | -0.189 | -0.3069 | Yes |
| 87 | PHB2 |  |  | 19772 | -0.208 | -0.3038 | Yes |
| 88 | ECHS1 |  |  | 19778 | -0.209 | -0.2924 | Yes |
| 89 | SLC5A6 |  |  | 19876 | -0.222 | -0.2848 | Yes |
| 90 | PPARG |  |  | 19907 | -0.226 | -0.2736 | Yes |
| 91 | PPA1 |  |  | 19945 | -0.231 | -0.2626 | Yes |
| 92 | PC |  |  | 20000 | -0.241 | -0.2518 | Yes |
| 93 | MRPL34 |  |  | 20267 | -0.319 | -0.2470 | Yes |
| 94 | NRIP1 |  |  | 20273 | -0.320 | -0.2294 | Yes |
| 95 | XBP1 |  |  | 20381 | -0.370 | -0.2140 | Yes |
| 96 | GYS1 |  |  | 20388 | -0.378 | -0.1933 | Yes |
| 97 | DBI |  |  | 20414 | -0.395 | -0.1725 | Yes |
| 98 | MRPS34 |  |  | 20443 | -0.417 | -0.1506 | Yes |
| 99 | MRPL18 |  |  | 20450 | -0.425 | -0.1272 | Yes |
| 100 | ACO2 |  |  | 20483 | -0.453 | -0.1035 | Yes |
| 101 | NDUFAB1 |  |  | 20484 | -0.455 | -0.0782 | Yes |
| 102 | POR |  |  | 20501 | -0.476 | -0.0525 | Yes |
| 103 | G6PD |  |  | 20527 | -0.507 | -0.0254 | Yes |
| 104 | CYC1 |  |  | 20538 | -0.524 | 0.0033 | Yes |
Table: GSEA details [plain text format]

  

Fig 2: IDX\_TSA\_UP\_CLUSTER5: Random ES distribution      
 Gene set null distribution of ES for **IDX\_TSA\_UP\_CLUSTER5**
