# Supplementary material for: The rapamycin-regulated gene expression signature determines prognosis for breast cancer
Source: Mol Cancer. 2009 Sep 24;8:75. doi: 10.1186/1476-4598-8-75 (PMC2761377; doi:10.1186/1476-4598-8-75)
Supplement: Additional file 2 — Gene set enrichment analysis of in vivo data, time series. The data provided represent the time series of GSEA. This compressed file contains "Time" shortcut file and "GSEA_time" folder. Clicking on "Time" shortcut opens the index file providing access to analysis files contained in the "GSEA_time" folder. [file 1476-4598-8-75-S2.zip › GSEA_time/IFNALPHA_NL_HCC_UP.html]

Details for gene set IFNALPHA\_NL\_HCC\_UP[GSEA]

|  || Dataset | gsea\_time\_collapsed |
| Phenotype | NoPhenotypeAvailable |
| Upregulated in class | na\_neg |
| GeneSet | IFNALPHA\_NL\_HCC\_UP |
| Enrichment Score (ES) | -0.39618167 |
| Normalized Enrichment Score (NES) | -1.2610109 |
| Nominal p-value | 0.13736264 |
| FDR q-value | 0.35543057 |
| FWER p-Value | 1.0 |
Table: GSEA Results Summary

  

Fig 1: Enrichment plot: IFNALPHA\_NL\_HCC\_UP      
 Profile of the Running ES Score & Positions of GeneSet Members on the Rank Ordered List

  

| PROBE | GENE SYMBOL | GENE\_TITLE | RANK IN GENE LIST | RANK METRIC SCORE | RUNNING ES | CORE ENRICHMENT || 1 | IFITM1 |  |  | 275 | 0.669 | 0.1729 | No |
| 2 | AKR7A2 |  |  | 1535 | 0.325 | 0.2021 | No |
| 3 | PLSCR1 |  |  | 1622 | 0.315 | 0.2858 | No |
| 4 | STAT1 |  |  | 4141 | 0.167 | 0.2101 | No |
| 5 | E2F2 |  |  | 4728 | 0.146 | 0.2223 | No |
| 6 | KIAA0409 |  |  | 10407 | 0.036 | -0.0434 | No |
| 7 | IRF1 |  |  | 12849 | 0.001 | -0.1617 | No |
| 8 | KLK3 |  |  | 13193 | -0.004 | -0.1773 | No |
| 9 | PSME1 |  |  | 14347 | -0.021 | -0.2274 | No |
| 10 | VCAM1 |  |  | 15109 | -0.034 | -0.2550 | No |
| 11 | MX2 |  |  | 17855 | -0.094 | -0.3621 | Yes |
| 12 | BST2 |  |  | 17902 | -0.096 | -0.3377 | Yes |
| 13 | RFC2 |  |  | 18307 | -0.110 | -0.3266 | Yes |
| 14 | UBE2L6 |  |  | 19740 | -0.204 | -0.3393 | Yes |
| 15 | IFI27 |  |  | 19905 | -0.226 | -0.2843 | Yes |
| 16 | ISG15 |  |  | 20207 | -0.298 | -0.2159 | Yes |
| 17 | MX1 |  |  | 20243 | -0.310 | -0.1312 | Yes |
| 18 | LGALS3BP |  |  | 20546 | -0.534 | 0.0029 | Yes |
Table: GSEA details [plain text format]

  

Fig 2: IFNALPHA\_NL\_HCC\_UP: Random ES distribution      
 Gene set null distribution of ES for **IFNALPHA\_NL\_HCC\_UP**
