# Supplementary material for: The rapamycin-regulated gene expression signature determines prognosis for breast cancer
Source: Mol Cancer. 2009 Sep 24;8:75. doi: 10.1186/1476-4598-8-75 (PMC2761377; doi:10.1186/1476-4598-8-75)
Supplement: Additional file 2 — Gene set enrichment analysis of in vivo data, time series. The data provided represent the time series of GSEA. This compressed file contains "Time" shortcut file and "GSEA_time" folder. Clicking on "Time" shortcut opens the index file providing access to analysis files contained in the "GSEA_time" folder. [file 1476-4598-8-75-S2.zip › GSEA_time/IRITANI_ADPROX_DN.html]

Details for gene set IRITANI\_ADPROX\_DN[GSEA]

|  || Dataset | gsea\_time\_collapsed |
| Phenotype | NoPhenotypeAvailable |
| Upregulated in class | na\_pos |
| GeneSet | IRITANI\_ADPROX\_DN |
| Enrichment Score (ES) | 0.6371046 |
| Normalized Enrichment Score (NES) | 1.785971 |
| Nominal p-value | 0.0 |
| FDR q-value | 0.008509755 |
| FWER p-Value | 0.238 |
Table: GSEA Results Summary

  

Fig 1: Enrichment plot: IRITANI\_ADPROX\_DN      
 Profile of the Running ES Score & Positions of GeneSet Members on the Rank Ordered List

  

| PROBE | GENE SYMBOL | GENE\_TITLE | RANK IN GENE LIST | RANK METRIC SCORE | RUNNING ES | CORE ENRICHMENT || 1 | LDHB |  |  | 1 | 2.284 | 0.1253 | Yes |
| 2 | CCL2 |  |  | 11 | 1.629 | 0.2143 | Yes |
| 3 | LY75 |  |  | 18 | 1.465 | 0.2944 | Yes |
| 4 | ARHGDIB |  |  | 251 | 0.681 | 0.3205 | Yes |
| 5 | PRNP |  |  | 279 | 0.668 | 0.3558 | Yes |
| 6 | PLAU |  |  | 302 | 0.647 | 0.3903 | Yes |
| 7 | SMURF2 |  |  | 373 | 0.596 | 0.4196 | Yes |
| 8 | IL32 |  |  | 594 | 0.502 | 0.4364 | Yes |
| 9 | NRP1 |  |  | 608 | 0.496 | 0.4630 | Yes |
| 10 | TNFRSF21 |  |  | 637 | 0.488 | 0.4884 | Yes |
| 11 | CD44 |  |  | 641 | 0.487 | 0.5150 | Yes |
| 12 | NUAK1 |  |  | 689 | 0.476 | 0.5388 | Yes |
| 13 | EMP3 |  |  | 708 | 0.473 | 0.5639 | Yes |
| 14 | NNMT |  |  | 813 | 0.442 | 0.5831 | Yes |
| 15 | TPM1 |  |  | 870 | 0.428 | 0.6039 | Yes |
| 16 | STAT6 |  |  | 1149 | 0.378 | 0.6112 | Yes |
| 17 | ANXA6 |  |  | 1248 | 0.363 | 0.6263 | Yes |
| 18 | CDH2 |  |  | 1872 | 0.294 | 0.6121 | Yes |
| 19 | KRT7 |  |  | 2115 | 0.276 | 0.6155 | Yes |
| 20 | CD59 |  |  | 2344 | 0.259 | 0.6186 | Yes |
| 21 | PFKM |  |  | 2412 | 0.254 | 0.6292 | Yes |
| 22 | PLA2G4A |  |  | 2718 | 0.235 | 0.6273 | Yes |
| 23 | MLLT11 |  |  | 2893 | 0.225 | 0.6312 | Yes |
| 24 | IGF2BP3 |  |  | 3018 | 0.218 | 0.6371 | Yes |
| 25 | GLCE |  |  | 3705 | 0.187 | 0.6140 | No |
| 26 | ZNF238 |  |  | 3934 | 0.176 | 0.6125 | No |
| 27 | TRAM2 |  |  | 3969 | 0.175 | 0.6205 | No |
| 28 | AXL |  |  | 4024 | 0.173 | 0.6273 | No |
| 29 | CSPG2 |  |  | 4202 | 0.164 | 0.6277 | No |
| 30 | TANK |  |  | 4359 | 0.159 | 0.6289 | No |
| 31 | PROCR |  |  | 4716 | 0.147 | 0.6196 | No |
| 32 | CAP2 |  |  | 4775 | 0.145 | 0.6247 | No |
| 33 | SRPX |  |  | 4858 | 0.142 | 0.6285 | No |
| 34 | IL6 |  |  | 4954 | 0.138 | 0.6315 | No |
| 35 | PRKACB |  |  | 5252 | 0.130 | 0.6242 | No |
| 36 | ARHGAP22 |  |  | 6044 | 0.112 | 0.5918 | No |
| 37 | GPR126 |  |  | 6157 | 0.109 | 0.5924 | No |
| 38 | LTBP2 |  |  | 6325 | 0.105 | 0.5900 | No |
| 39 | PDLIM4 |  |  | 6570 | 0.100 | 0.5836 | No |
| 40 | ARL6IP5 |  |  | 6587 | 0.100 | 0.5883 | No |
| 41 | LPXN |  |  | 7085 | 0.089 | 0.5690 | No |
| 42 | BMP6 |  |  | 7325 | 0.085 | 0.5621 | No |
| 43 | ICAM2 |  |  | 7358 | 0.085 | 0.5652 | No |
| 44 | RRAS |  |  | 7849 | 0.075 | 0.5454 | No |
| 45 | PRG1 |  |  | 8317 | 0.067 | 0.5264 | No |
| 46 | GBP2 |  |  | 8805 | 0.060 | 0.5060 | No |
| 47 | FAP |  |  | 9123 | 0.055 | 0.4936 | No |
| 48 | LYL1 |  |  | 9983 | 0.042 | 0.4541 | No |
| 49 | TFEC |  |  | 10170 | 0.039 | 0.4472 | No |
| 50 | NRCAM |  |  | 10964 | 0.028 | 0.4101 | No |
| 51 | MMP14 |  |  | 11968 | 0.014 | 0.3621 | No |
| 52 | SLC38A6 |  |  | 12891 | 0.000 | 0.3173 | No |
| 53 | FAM107A |  |  | 14562 | -0.025 | 0.2373 | No |
| 54 | RGS4 |  |  | 17417 | -0.081 | 0.1029 | No |
| 55 | SERPINE1 |  |  | 18412 | -0.114 | 0.0608 | No |
| 56 | SELP |  |  | 18627 | -0.123 | 0.0571 | No |
| 57 | ITGA5 |  |  | 18655 | -0.124 | 0.0626 | No |
| 58 | TCN2 |  |  | 18767 | -0.129 | 0.0643 | No |
| 59 | MAPKAPK3 |  |  | 19089 | -0.146 | 0.0567 | No |
| 60 | TRIM22 |  |  | 20245 | -0.311 | 0.0175 | No |
Table: GSEA details [plain text format]

  

Fig 2: IRITANI\_ADPROX\_DN: Random ES distribution      
 Gene set null distribution of ES for **IRITANI\_ADPROX\_DN**
