# Supplementary material for: The rapamycin-regulated gene expression signature determines prognosis for breast cancer
Source: Mol Cancer. 2009 Sep 24;8:75. doi: 10.1186/1476-4598-8-75 (PMC2761377; doi:10.1186/1476-4598-8-75)
Supplement: Additional file 2 — Gene set enrichment analysis of in vivo data, time series. The data provided represent the time series of GSEA. This compressed file contains "Time" shortcut file and "GSEA_time" folder. Clicking on "Time" shortcut opens the index file providing access to analysis files contained in the "GSEA_time" folder. [file 1476-4598-8-75-S2.zip › GSEA_time/IRITANI_ADPROX_VASC.html]

Details for gene set IRITANI\_ADPROX\_VASC[GSEA]

|  || Dataset | gsea\_time\_collapsed |
| Phenotype | NoPhenotypeAvailable |
| Upregulated in class | na\_pos |
| GeneSet | IRITANI\_ADPROX\_VASC |
| Enrichment Score (ES) | 0.57849115 |
| Normalized Enrichment Score (NES) | 1.7341518 |
| Nominal p-value | 0.0 |
| FDR q-value | 0.01354171 |
| FWER p-Value | 0.494 |
Table: GSEA Results Summary

  

Fig 1: Enrichment plot: IRITANI\_ADPROX\_VASC      
 Profile of the Running ES Score & Positions of GeneSet Members on the Rank Ordered List

  

| PROBE | GENE SYMBOL | GENE\_TITLE | RANK IN GENE LIST | RANK METRIC SCORE | RUNNING ES | CORE ENRICHMENT || 1 | LDHB |  |  | 1 | 2.284 | 0.0575 | Yes |
| 2 | CCL2 |  |  | 11 | 1.629 | 0.0980 | Yes |
| 3 | IL8 |  |  | 13 | 1.561 | 0.1373 | Yes |
| 4 | LY75 |  |  | 18 | 1.465 | 0.1740 | Yes |
| 5 | ANPEP |  |  | 54 | 1.069 | 0.1992 | Yes |
| 6 | TAGLN |  |  | 95 | 0.911 | 0.2202 | Yes |
| 7 | NR2F2 |  |  | 115 | 0.875 | 0.2413 | Yes |
| 8 | CXCR4 |  |  | 186 | 0.747 | 0.2567 | Yes |
| 9 | EXT1 |  |  | 230 | 0.695 | 0.2721 | Yes |
| 10 | ARHGDIB |  |  | 251 | 0.681 | 0.2883 | Yes |
| 11 | ATP1B1 |  |  | 270 | 0.672 | 0.3043 | Yes |
| 12 | PRNP |  |  | 279 | 0.668 | 0.3207 | Yes |
| 13 | PLAU |  |  | 302 | 0.647 | 0.3360 | Yes |
| 14 | TPM2 |  |  | 322 | 0.628 | 0.3508 | Yes |
| 15 | CDC42EP3 |  |  | 350 | 0.608 | 0.3648 | Yes |
| 16 | SMURF2 |  |  | 373 | 0.596 | 0.3787 | Yes |
| 17 | RGS10 |  |  | 456 | 0.552 | 0.3886 | Yes |
| 18 | LPHN2 |  |  | 554 | 0.514 | 0.3968 | Yes |
| 19 | LAMP2 |  |  | 564 | 0.511 | 0.4093 | Yes |
| 20 | IL32 |  |  | 594 | 0.502 | 0.4205 | Yes |
| 21 | TNFRSF21 |  |  | 637 | 0.488 | 0.4307 | Yes |
| 22 | CD44 |  |  | 641 | 0.487 | 0.4428 | Yes |
| 23 | NUAK1 |  |  | 689 | 0.476 | 0.4525 | Yes |
| 24 | COL8A1 |  |  | 713 | 0.472 | 0.4633 | Yes |
| 25 | EPS8 |  |  | 715 | 0.471 | 0.4751 | Yes |
| 26 | NNMT |  |  | 813 | 0.442 | 0.4815 | Yes |
| 27 | TPM1 |  |  | 870 | 0.428 | 0.4895 | Yes |
| 28 | ACTA2 |  |  | 923 | 0.418 | 0.4975 | Yes |
| 29 | ITGAV |  |  | 940 | 0.416 | 0.5072 | Yes |
| 30 | DKK1 |  |  | 1024 | 0.401 | 0.5133 | Yes |
| 31 | TGFBI |  |  | 1142 | 0.379 | 0.5171 | Yes |
| 32 | STAT6 |  |  | 1149 | 0.378 | 0.5264 | Yes |
| 33 | ANXA6 |  |  | 1248 | 0.363 | 0.5307 | Yes |
| 34 | DFNA5 |  |  | 1342 | 0.349 | 0.5349 | Yes |
| 35 | JUN |  |  | 1522 | 0.326 | 0.5344 | Yes |
| 36 | TGM2 |  |  | 1588 | 0.320 | 0.5393 | Yes |
| 37 | CDH2 |  |  | 1872 | 0.294 | 0.5328 | Yes |
| 38 | ITGB5 |  |  | 1900 | 0.293 | 0.5389 | Yes |
| 39 | PFN2 |  |  | 1990 | 0.285 | 0.5417 | Yes |
| 40 | KRT7 |  |  | 2115 | 0.276 | 0.5426 | Yes |
| 41 | MVP |  |  | 2148 | 0.273 | 0.5479 | Yes |
| 42 | SLC1A1 |  |  | 2300 | 0.261 | 0.5471 | Yes |
| 43 | CD59 |  |  | 2344 | 0.259 | 0.5515 | Yes |
| 44 | ARL4C |  |  | 2355 | 0.258 | 0.5575 | Yes |
| 45 | PFKM |  |  | 2412 | 0.254 | 0.5612 | Yes |
| 46 | UPP1 |  |  | 2501 | 0.247 | 0.5631 | Yes |
| 47 | KLHL21 |  |  | 2629 | 0.240 | 0.5629 | Yes |
| 48 | PLA2G4A |  |  | 2718 | 0.235 | 0.5645 | Yes |
| 49 | BGN |  |  | 2751 | 0.233 | 0.5688 | Yes |
| 50 | PGF |  |  | 2843 | 0.228 | 0.5701 | Yes |
| 51 | MLLT11 |  |  | 2893 | 0.225 | 0.5734 | Yes |
| 52 | SMAD3 |  |  | 2926 | 0.223 | 0.5774 | Yes |
| 53 | IGF2BP3 |  |  | 3018 | 0.218 | 0.5785 | Yes |
| 54 | ICAM1 |  |  | 3140 | 0.212 | 0.5779 | No |
| 55 | LAMC2 |  |  | 3324 | 0.203 | 0.5741 | No |
| 56 | GLCE |  |  | 3705 | 0.187 | 0.5602 | No |
| 57 | PLOD2 |  |  | 3812 | 0.182 | 0.5596 | No |
| 58 | IL4R |  |  | 3899 | 0.178 | 0.5599 | No |
| 59 | ZNF238 |  |  | 3934 | 0.176 | 0.5626 | No |
| 60 | TRAM2 |  |  | 3969 | 0.175 | 0.5654 | No |
| 61 | NMT2 |  |  | 4000 | 0.174 | 0.5683 | No |
| 62 | AXL |  |  | 4024 | 0.173 | 0.5715 | No |
| 63 | CTSC |  |  | 4134 | 0.167 | 0.5704 | No |
| 64 | CSPG2 |  |  | 4202 | 0.164 | 0.5713 | No |
| 65 | TANK |  |  | 4359 | 0.159 | 0.5676 | No |
| 66 | PLTP |  |  | 4369 | 0.158 | 0.5712 | No |
| 67 | EIF2B2 |  |  | 4505 | 0.154 | 0.5685 | No |
| 68 | TACSTD2 |  |  | 4511 | 0.154 | 0.5721 | No |
| 69 | PROCR |  |  | 4716 | 0.147 | 0.5658 | No |
| 70 | CAP2 |  |  | 4775 | 0.145 | 0.5666 | No |
| 71 | SRPX |  |  | 4858 | 0.142 | 0.5662 | No |
| 72 | MCM5 |  |  | 4893 | 0.141 | 0.5681 | No |
| 73 | IL6 |  |  | 4954 | 0.138 | 0.5686 | No |
| 74 | SLC6A8 |  |  | 5081 | 0.135 | 0.5659 | No |
| 75 | ENO2 |  |  | 5094 | 0.135 | 0.5687 | No |
| 76 | PRKACB |  |  | 5252 | 0.130 | 0.5643 | No |
| 77 | MMP1 |  |  | 5254 | 0.130 | 0.5675 | No |
| 78 | SORD |  |  | 5370 | 0.127 | 0.5651 | No |
| 79 | COL6A1 |  |  | 5412 | 0.126 | 0.5663 | No |
| 80 | CLDN7 |  |  | 5473 | 0.125 | 0.5665 | No |
| 81 | CXCL1 |  |  | 5663 | 0.120 | 0.5603 | No |
| 82 | ARHGAP22 |  |  | 6044 | 0.112 | 0.5445 | No |
| 83 | IER3 |  |  | 6090 | 0.111 | 0.5451 | No |
| 84 | UAP1 |  |  | 6117 | 0.110 | 0.5466 | No |
| 85 | GPR126 |  |  | 6157 | 0.109 | 0.5474 | No |
| 86 | SRPX2 |  |  | 6174 | 0.109 | 0.5494 | No |
| 87 | LOXL2 |  |  | 6308 | 0.105 | 0.5456 | No |
| 88 | LTBP2 |  |  | 6325 | 0.105 | 0.5474 | No |
| 89 | PDLIM4 |  |  | 6570 | 0.100 | 0.5380 | No |
| 90 | ARL6IP5 |  |  | 6587 | 0.100 | 0.5397 | No |
| 91 | NPTX1 |  |  | 6768 | 0.096 | 0.5333 | No |
| 92 | TP53I3 |  |  | 7036 | 0.090 | 0.5226 | No |
| 93 | LPXN |  |  | 7085 | 0.089 | 0.5225 | No |
| 94 | BMP6 |  |  | 7325 | 0.085 | 0.5129 | No |
| 95 | ICAM2 |  |  | 7358 | 0.085 | 0.5135 | No |
| 96 | LAMA5 |  |  | 7478 | 0.082 | 0.5098 | No |
| 97 | RRAS |  |  | 7849 | 0.075 | 0.4936 | No |
| 98 | CHST1 |  |  | 7994 | 0.073 | 0.4883 | No |
| 99 | PRG1 |  |  | 8317 | 0.067 | 0.4743 | No |
| 100 | TXNRD2 |  |  | 8356 | 0.067 | 0.4741 | No |
| 101 | GADD45A |  |  | 8579 | 0.063 | 0.4649 | No |
| 102 | RHOB |  |  | 8609 | 0.063 | 0.4650 | No |
| 103 | SLC7A7 |  |  | 8744 | 0.061 | 0.4600 | No |
| 104 | GBP2 |  |  | 8805 | 0.060 | 0.4586 | No |
| 105 | FAP |  |  | 9123 | 0.055 | 0.4445 | No |
| 106 | CLU |  |  | 9929 | 0.043 | 0.4062 | No |
| 107 | LYL1 |  |  | 9983 | 0.042 | 0.4047 | No |
| 108 | FLT1 |  |  | 10090 | 0.041 | 0.4005 | No |
| 109 | TFEC |  |  | 10170 | 0.039 | 0.3977 | No |
| 110 | EFEMP1 |  |  | 10302 | 0.038 | 0.3922 | No |
| 111 | MT3 |  |  | 10371 | 0.037 | 0.3898 | No |
| 112 | FBXL2 |  |  | 10618 | 0.033 | 0.3786 | No |
| 113 | NRCAM |  |  | 10964 | 0.028 | 0.3624 | No |
| 114 | BASP1 |  |  | 11212 | 0.025 | 0.3510 | No |
| 115 | CYFIP2 |  |  | 11549 | 0.020 | 0.3351 | No |
| 116 | ANXA3 |  |  | 11767 | 0.017 | 0.3249 | No |
| 117 | MMP14 |  |  | 11968 | 0.014 | 0.3155 | No |
| 118 | NISCH |  |  | 12066 | 0.013 | 0.3110 | No |
| 119 | CDKN1A |  |  | 12863 | 0.001 | 0.2722 | No |
| 120 | SLC38A6 |  |  | 12891 | 0.000 | 0.2708 | No |
| 121 | MT1F |  |  | 12932 | -0.000 | 0.2689 | No |
| 122 | AGRIN |  |  | 13257 | -0.005 | 0.2532 | No |
| 123 | ITM2A |  |  | 13336 | -0.006 | 0.2495 | No |
| 124 | ECE1 |  |  | 13619 | -0.010 | 0.2360 | No |
| 125 | RAC2 |  |  | 14529 | -0.024 | 0.1922 | No |
| 126 | FAM107A |  |  | 14562 | -0.025 | 0.1912 | No |
| 127 | ALDH2 |  |  | 14955 | -0.031 | 0.1728 | No |
| 128 | LENG4 |  |  | 15477 | -0.039 | 0.1483 | No |
| 129 | UCHL1 |  |  | 16647 | -0.062 | 0.0927 | No |
| 130 | COL1A2 |  |  | 16667 | -0.063 | 0.0934 | No |
| 131 | MT1X |  |  | 17171 | -0.075 | 0.0707 | No |
| 132 | RGS4 |  |  | 17417 | -0.081 | 0.0607 | No |
| 133 | PKIA |  |  | 17654 | -0.088 | 0.0514 | No |
| 134 | MT1H |  |  | 17868 | -0.095 | 0.0434 | No |
| 135 | BTG2 |  |  | 18002 | -0.099 | 0.0394 | No |
| 136 | VEGFC |  |  | 18210 | -0.106 | 0.0319 | No |
| 137 | SERPINE1 |  |  | 18412 | -0.114 | 0.0250 | No |
| 138 | SELP |  |  | 18627 | -0.123 | 0.0176 | No |
| 139 | ITGA5 |  |  | 18655 | -0.124 | 0.0194 | No |
| 140 | RCN1 |  |  | 18750 | -0.128 | 0.0181 | No |
| 141 | TCN2 |  |  | 18767 | -0.129 | 0.0205 | No |
| 142 | MAPKAPK3 |  |  | 19089 | -0.146 | 0.0085 | No |
| 143 | SGSH |  |  | 19211 | -0.154 | 0.0065 | No |
| 144 | MT2A |  |  | 19579 | -0.187 | -0.0067 | No |
| 145 | FHL2 |  |  | 19668 | -0.196 | -0.0061 | No |
| 146 | SELE |  |  | 19762 | -0.207 | -0.0054 | No |
| 147 | IFI27 |  |  | 19905 | -0.226 | -0.0067 | No |
| 148 | ISG15 |  |  | 20207 | -0.298 | -0.0139 | No |
| 149 | TRIM22 |  |  | 20245 | -0.311 | -0.0079 | No |
| 150 | SLC3A2 |  |  | 20347 | -0.352 | -0.0040 | No |
| 151 | CAPG |  |  | 20581 | -0.656 | 0.0012 | No |
Table: GSEA details [plain text format]

  

Fig 2: IRITANI\_ADPROX\_VASC: Random ES distribution      
 Gene set null distribution of ES for **IRITANI\_ADPROX\_VASC**
