# Supplementary material for: The rapamycin-regulated gene expression signature determines prognosis for breast cancer
Source: Mol Cancer. 2009 Sep 24;8:75. doi: 10.1186/1476-4598-8-75 (PMC2761377; doi:10.1186/1476-4598-8-75)
Supplement: Additional file 2 — Gene set enrichment analysis of in vivo data, time series. The data provided represent the time series of GSEA. This compressed file contains "Time" shortcut file and "GSEA_time" folder. Clicking on "Time" shortcut opens the index file providing access to analysis files contained in the "GSEA_time" folder. [file 1476-4598-8-75-S2.zip › GSEA_time/KREBS_TCA_CYCLE.html]

Details for gene set KREBS\_TCA\_CYCLE[GSEA]

|  || Dataset | gsea\_time\_collapsed |
| Phenotype | NoPhenotypeAvailable |
| Upregulated in class | na\_neg |
| GeneSet | KREBS\_TCA\_CYCLE |
| Enrichment Score (ES) | -0.3768868 |
| Normalized Enrichment Score (NES) | -1.340738 |
| Nominal p-value | 0.0729927 |
| FDR q-value | 0.3002522 |
| FWER p-Value | 1.0 |
Table: GSEA Results Summary

  

Fig 1: Enrichment plot: KREBS\_TCA\_CYCLE      
 Profile of the Running ES Score & Positions of GeneSet Members on the Rank Ordered List

  

| PROBE | GENE SYMBOL | GENE\_TITLE | RANK IN GENE LIST | RANK METRIC SCORE | RUNNING ES | CORE ENRICHMENT || 1 | PDK1 |  |  | 355 | 0.604 | 0.0848 | No |
| 2 | MDH1 |  |  | 1304 | 0.354 | 0.0985 | No |
| 3 | PDK3 |  |  | 1385 | 0.343 | 0.1526 | No |
| 4 | PPM2C |  |  | 2313 | 0.261 | 0.1516 | No |
| 5 | PDK4 |  |  | 3942 | 0.176 | 0.1022 | No |
| 6 | SDHC |  |  | 4051 | 0.171 | 0.1259 | No |
| 7 | DLAT |  |  | 4067 | 0.171 | 0.1540 | No |
| 8 | PDHB |  |  | 4317 | 0.160 | 0.1689 | No |
| 9 | SUCLG2 |  |  | 5158 | 0.133 | 0.1506 | No |
| 10 | OGDH |  |  | 5727 | 0.119 | 0.1431 | No |
| 11 | IDH3A |  |  | 6869 | 0.094 | 0.1035 | No |
| 12 | MDH2 |  |  | 7052 | 0.090 | 0.1099 | No |
| 13 | DLD |  |  | 8057 | 0.072 | 0.0732 | No |
| 14 | SUCLA2 |  |  | 8448 | 0.065 | 0.0653 | No |
| 15 | SDHB |  |  | 9306 | 0.052 | 0.0325 | No |
| 16 | FH |  |  | 15798 | -0.045 | -0.2754 | No |
| 17 | PDHA2 |  |  | 15899 | -0.047 | -0.2724 | No |
| 18 | IDH3G |  |  | 16278 | -0.054 | -0.2816 | No |
| 19 | PDK2 |  |  | 17777 | -0.092 | -0.3388 | No |
| 20 | PDP2 |  |  | 17856 | -0.094 | -0.3267 | No |
| 21 | SDHD |  |  | 18044 | -0.101 | -0.3188 | No |
| 22 | CS |  |  | 19240 | -0.156 | -0.3506 | Yes |
| 23 | PDHA1 |  |  | 19595 | -0.188 | -0.3360 | Yes |
| 24 | IDH2 |  |  | 19803 | -0.213 | -0.3101 | Yes |
| 25 | PC |  |  | 20000 | -0.241 | -0.2790 | Yes |
| 26 | SDHA |  |  | 20118 | -0.266 | -0.2396 | Yes |
| 27 | IDH3B |  |  | 20127 | -0.270 | -0.1944 | Yes |
| 28 | SUCLG1 |  |  | 20440 | -0.417 | -0.1391 | Yes |
| 29 | PDHX |  |  | 20441 | -0.417 | -0.0687 | Yes |
| 30 | ACO2 |  |  | 20483 | -0.453 | 0.0059 | Yes |
Table: GSEA details [plain text format]

  

Fig 2: KREBS\_TCA\_CYCLE: Random ES distribution      
 Gene set null distribution of ES for **KREBS\_TCA\_CYCLE**
