# Supplementary material for: The rapamycin-regulated gene expression signature determines prognosis for breast cancer
Source: Mol Cancer. 2009 Sep 24;8:75. doi: 10.1186/1476-4598-8-75 (PMC2761377; doi:10.1186/1476-4598-8-75)
Supplement: Additional file 2 — Gene set enrichment analysis of in vivo data, time series. The data provided represent the time series of GSEA. This compressed file contains "Time" shortcut file and "GSEA_time" folder. Clicking on "Time" shortcut opens the index file providing access to analysis files contained in the "GSEA_time" folder. [file 1476-4598-8-75-S2.zip › GSEA_time/LEE_TCELLS1_UP.html]

Details for gene set LEE\_TCELLS1\_UP[GSEA]

|  || Dataset | gsea\_time\_collapsed |
| Phenotype | NoPhenotypeAvailable |
| Upregulated in class | na\_pos |
| GeneSet | LEE\_TCELLS1\_UP |
| Enrichment Score (ES) | 0.64783025 |
| Normalized Enrichment Score (NES) | 1.9637622 |
| Nominal p-value | 0.0 |
| FDR q-value | 0.0015084273 |
| FWER p-Value | 0.0030 |
Table: GSEA Results Summary

  

Fig 1: Enrichment plot: LEE\_TCELLS1\_UP      
 Profile of the Running ES Score & Positions of GeneSet Members on the Rank Ordered List

  

| PROBE | GENE SYMBOL | GENE\_TITLE | RANK IN GENE LIST | RANK METRIC SCORE | RUNNING ES | CORE ENRICHMENT || 1 | RP1-93H18.5 |  |  | 63 | 1.010 | 0.0182 | Yes |
| 2 | FAM3C |  |  | 81 | 0.949 | 0.0373 | Yes |
| 3 | ZC3HAV1L |  |  | 119 | 0.871 | 0.0538 | Yes |
| 4 | ANKRD10 |  |  | 131 | 0.845 | 0.0710 | Yes |
| 5 | SLC25A37 |  |  | 157 | 0.793 | 0.0865 | Yes |
| 6 | ANKRD37 |  |  | 179 | 0.759 | 0.1014 | Yes |
| 7 | OXR1 |  |  | 184 | 0.747 | 0.1169 | Yes |
| 8 | LOC440667 /// LOC440 |  |  | 245 | 0.685 | 0.1284 | Yes |
| 9 | CSDA |  |  | 274 | 0.669 | 0.1411 | Yes |
| 10 | SLC2A3 |  |  | 278 | 0.668 | 0.1550 | Yes |
| 11 | ATP11A |  |  | 290 | 0.660 | 0.1683 | Yes |
| 12 | NFKBIZ |  |  | 313 | 0.639 | 0.1807 | Yes |
| 13 | RASA2 |  |  | 317 | 0.634 | 0.1939 | Yes |
| 14 | DAPK1 |  |  | 343 | 0.610 | 0.2055 | Yes |
| 15 | MICAL2 |  |  | 374 | 0.595 | 0.2165 | Yes |
| 16 | CDCA7 |  |  | 387 | 0.591 | 0.2284 | Yes |
| 17 | PTGER4 |  |  | 453 | 0.552 | 0.2368 | Yes |
| 18 | ABCC10 |  |  | 458 | 0.551 | 0.2482 | Yes |
| 19 | NFATC2IP |  |  | 464 | 0.547 | 0.2594 | Yes |
| 20 | C12ORF38 |  |  | 470 | 0.544 | 0.2706 | Yes |
| 21 | CDCA7L |  |  | 481 | 0.540 | 0.2815 | Yes |
| 22 | SKAP2 |  |  | 537 | 0.519 | 0.2897 | Yes |
| 23 | ARL6IP6 |  |  | 541 | 0.517 | 0.3005 | Yes |
| 24 | MYSM1 |  |  | 561 | 0.512 | 0.3103 | Yes |
| 25 | EEF1E1 |  |  | 592 | 0.502 | 0.3194 | Yes |
| 26 | SIDT2 |  |  | 624 | 0.491 | 0.3282 | Yes |
| 27 | LOC346887 |  |  | 639 | 0.488 | 0.3378 | Yes |
| 28 | LYPLA1 |  |  | 695 | 0.475 | 0.3451 | Yes |
| 29 | LOC641845 /// LOC647 |  |  | 707 | 0.473 | 0.3545 | Yes |
| 30 | PGM2 |  |  | 791 | 0.447 | 0.3598 | Yes |
| 31 | KCTD18 |  |  | 795 | 0.446 | 0.3691 | Yes |
| 32 | SOX4 |  |  | 910 | 0.421 | 0.3723 | Yes |
| 33 | TNFAIP3 |  |  | 911 | 0.421 | 0.3812 | Yes |
| 34 | TAF4B |  |  | 937 | 0.417 | 0.3887 | Yes |
| 35 | ING3 |  |  | 1066 | 0.394 | 0.3907 | Yes |
| 36 | PABPC1 |  |  | 1079 | 0.391 | 0.3984 | Yes |
| 37 | IFT80 |  |  | 1093 | 0.388 | 0.4059 | Yes |
| 38 | ZAK |  |  | 1123 | 0.382 | 0.4125 | Yes |
| 39 | TBC1D22A |  |  | 1134 | 0.380 | 0.4200 | Yes |
| 40 | PAK1 |  |  | 1156 | 0.378 | 0.4269 | Yes |
| 41 | ACY1L2 |  |  | 1267 | 0.360 | 0.4291 | Yes |
| 42 | BTBD3 |  |  | 1321 | 0.351 | 0.4339 | Yes |
| 43 | LCORL |  |  | 1332 | 0.350 | 0.4408 | Yes |
| 44 | CDK2AP1 |  |  | 1363 | 0.345 | 0.4466 | Yes |
| 45 | KLHL8 |  |  | 1406 | 0.340 | 0.4517 | Yes |
| 46 | STS-1 |  |  | 1410 | 0.340 | 0.4587 | Yes |
| 47 | SMC4 |  |  | 1414 | 0.340 | 0.4657 | Yes |
| 48 | AEBP2 |  |  | 1424 | 0.338 | 0.4723 | Yes |
| 49 | ZNF677 |  |  | 1432 | 0.337 | 0.4791 | Yes |
| 50 | TMEM65 |  |  | 1442 | 0.336 | 0.4857 | Yes |
| 51 | JUN |  |  | 1522 | 0.326 | 0.4887 | Yes |
| 52 | TOB1 |  |  | 1542 | 0.324 | 0.4946 | Yes |
| 53 | BIRC4 |  |  | 1599 | 0.319 | 0.4985 | Yes |
| 54 | CSRP2BP |  |  | 1621 | 0.316 | 0.5041 | Yes |
| 55 | PLSCR1 |  |  | 1622 | 0.315 | 0.5108 | Yes |
| 56 | RAB6IP1 |  |  | 1703 | 0.308 | 0.5133 | Yes |
| 57 | BCL11A |  |  | 1715 | 0.307 | 0.5193 | Yes |
| 58 | MTHFD2L |  |  | 1817 | 0.299 | 0.5206 | Yes |
| 59 | ZNF776 |  |  | 1844 | 0.297 | 0.5255 | Yes |
| 60 | ERICH1 |  |  | 1861 | 0.295 | 0.5310 | Yes |
| 61 | ID2 /// ID2B |  |  | 1876 | 0.294 | 0.5365 | Yes |
| 62 | LYPLAL1 |  |  | 1932 | 0.290 | 0.5399 | Yes |
| 63 | ACBD5 |  |  | 1938 | 0.290 | 0.5457 | Yes |
| 64 | HERC2P2 /// LOC44024 |  |  | 2038 | 0.281 | 0.5468 | Yes |
| 65 | DPY19L4 |  |  | 2074 | 0.278 | 0.5509 | Yes |
| 66 | TRIO |  |  | 2122 | 0.275 | 0.5544 | Yes |
| 67 | CHML |  |  | 2145 | 0.273 | 0.5591 | Yes |
| 68 | MTHFD1L |  |  | 2154 | 0.272 | 0.5644 | Yes |
| 69 | PLCXD2 |  |  | 2294 | 0.262 | 0.5631 | Yes |
| 70 | RAB18 |  |  | 2304 | 0.261 | 0.5681 | Yes |
| 71 | USP32 |  |  | 2311 | 0.261 | 0.5733 | Yes |
| 72 | SERF2 |  |  | 2323 | 0.260 | 0.5783 | Yes |
| 73 | OTUD6B |  |  | 2356 | 0.258 | 0.5821 | Yes |
| 74 | THRAP3 |  |  | 2371 | 0.257 | 0.5868 | Yes |
| 75 | VPS13C |  |  | 2372 | 0.257 | 0.5922 | Yes |
| 76 | PDCD4 |  |  | 2443 | 0.252 | 0.5941 | Yes |
| 77 | RNF138 |  |  | 2491 | 0.248 | 0.5970 | Yes |
| 78 | ZNF295 |  |  | 2581 | 0.243 | 0.5977 | Yes |
| 79 | LOC152485 |  |  | 2593 | 0.242 | 0.6023 | Yes |
| 80 | LOC374443 |  |  | 2749 | 0.233 | 0.5996 | Yes |
| 81 | YARS |  |  | 2775 | 0.231 | 0.6032 | Yes |
| 82 | VEZT |  |  | 2785 | 0.231 | 0.6077 | Yes |
| 83 | MGC33302 |  |  | 2818 | 0.229 | 0.6109 | Yes |
| 84 | RAI1 |  |  | 2981 | 0.220 | 0.6076 | Yes |
| 85 | IGHM |  |  | 3001 | 0.219 | 0.6113 | Yes |
| 86 | IGF2BP3 |  |  | 3018 | 0.218 | 0.6151 | Yes |
| 87 | CENPK |  |  | 3115 | 0.213 | 0.6149 | Yes |
| 88 | CBL |  |  | 3122 | 0.213 | 0.6190 | Yes |
| 89 | PAPOLA |  |  | 3124 | 0.213 | 0.6235 | Yes |
| 90 | RBM7 |  |  | 3130 | 0.212 | 0.6277 | Yes |
| 91 | VARSL |  |  | 3189 | 0.209 | 0.6292 | Yes |
| 92 | TMEPAI |  |  | 3232 | 0.207 | 0.6315 | Yes |
| 93 | NADK |  |  | 3234 | 0.207 | 0.6358 | Yes |
| 94 | HACE1 |  |  | 3249 | 0.206 | 0.6395 | Yes |
| 95 | LOC286170 |  |  | 3343 | 0.202 | 0.6392 | Yes |
| 96 | ZNF542 |  |  | 3397 | 0.200 | 0.6408 | Yes |
| 97 | PPAPDC1B |  |  | 3413 | 0.199 | 0.6442 | Yes |
| 98 | NIPSNAP3A |  |  | 3510 | 0.193 | 0.6436 | Yes |
| 99 | CEP57 |  |  | 3582 | 0.191 | 0.6441 | Yes |
| 100 | TMOD3 |  |  | 3589 | 0.190 | 0.6478 | Yes |
| 101 | NEDD1 |  |  | 3689 | 0.187 | 0.6469 | No |
| 102 | FAM126A |  |  | 3804 | 0.182 | 0.6452 | No |
| 103 | KIAA1450 |  |  | 3944 | 0.176 | 0.6420 | No |
| 104 | TOX |  |  | 4209 | 0.164 | 0.6326 | No |
| 105 | IGL@ /// IGLC1 /// I |  |  | 4260 | 0.163 | 0.6335 | No |
| 106 | SERF1A |  |  | 4439 | 0.156 | 0.6281 | No |
| 107 | SOCS3 |  |  | 4571 | 0.151 | 0.6249 | No |
| 108 | CSTF2T |  |  | 4715 | 0.147 | 0.6209 | No |
| 109 | PCGF5 |  |  | 4815 | 0.143 | 0.6191 | No |
| 110 | C9ORF77 |  |  | 4816 | 0.143 | 0.6221 | No |
| 111 | HEG1 |  |  | 4833 | 0.143 | 0.6243 | No |
| 112 | BCNP1 |  |  | 5070 | 0.136 | 0.6156 | No |
| 113 | CACHD1 |  |  | 5179 | 0.133 | 0.6131 | No |
| 114 | PRMT1 |  |  | 5253 | 0.130 | 0.6123 | No |
| 115 | HERC2 |  |  | 5281 | 0.130 | 0.6137 | No |
| 116 | ARL5A |  |  | 5394 | 0.127 | 0.6109 | No |
| 117 | SENP7 |  |  | 5398 | 0.126 | 0.6134 | No |
| 118 | PDDC1 |  |  | 5451 | 0.125 | 0.6135 | No |
| 119 | DARS |  |  | 5532 | 0.123 | 0.6121 | No |
| 120 | CAMK4 |  |  | 5697 | 0.119 | 0.6066 | No |
| 121 | CRY1 |  |  | 5740 | 0.118 | 0.6070 | No |
| 122 | TSPAN13 |  |  | 5795 | 0.117 | 0.6069 | No |
| 123 | FLJ11171 |  |  | 5805 | 0.117 | 0.6089 | No |
| 124 | CG018 |  |  | 5839 | 0.116 | 0.6097 | No |
| 125 | C6ORF79 |  |  | 6032 | 0.112 | 0.6027 | No |
| 126 | TIMM8B |  |  | 6205 | 0.108 | 0.5965 | No |
| 127 | IBRDC2 |  |  | 6289 | 0.106 | 0.5947 | No |
| 128 | PDE4D |  |  | 6296 | 0.106 | 0.5966 | No |
| 129 | AUTS2 |  |  | 6692 | 0.098 | 0.5793 | No |
| 130 | AKAP2 /// PALM2-AKAP |  |  | 6747 | 0.096 | 0.5787 | No |
| 131 | SH2D1A |  |  | 6905 | 0.093 | 0.5729 | No |
| 132 | ZNF711 |  |  | 7047 | 0.090 | 0.5679 | No |
| 133 | ZNF271 |  |  | 7055 | 0.090 | 0.5695 | No |
| 134 | C5ORF13 |  |  | 7342 | 0.085 | 0.5572 | No |
| 135 | RPS26 /// RPS26L /// |  |  | 7442 | 0.083 | 0.5541 | No |
| 136 | USP38 |  |  | 7540 | 0.081 | 0.5511 | No |
| 137 | AUH |  |  | 7640 | 0.079 | 0.5479 | No |
| 138 | FLJ22536 |  |  | 7895 | 0.075 | 0.5370 | No |
| 139 | RPL18A /// LOC390354 |  |  | 7971 | 0.073 | 0.5349 | No |
| 140 | TAGLN2 |  |  | 8153 | 0.070 | 0.5275 | No |
| 141 | PDLIM1 |  |  | 8198 | 0.069 | 0.5268 | No |
| 142 | ANKRD55 |  |  | 8301 | 0.068 | 0.5232 | No |
| 143 | C7ORF44 |  |  | 8470 | 0.065 | 0.5163 | No |
| 144 | CYSLTR1 |  |  | 8888 | 0.059 | 0.4971 | No |
| 145 | SYTL2 |  |  | 9133 | 0.055 | 0.4863 | No |
| 146 | HPGD |  |  | 9136 | 0.055 | 0.4874 | No |
| 147 | PLAC8 |  |  | 9590 | 0.048 | 0.4662 | No |
| 148 | ID2 |  |  | 10102 | 0.040 | 0.4420 | No |
| 149 | CD93 |  |  | 10190 | 0.039 | 0.4386 | No |
| 150 | RGS1 |  |  | 10491 | 0.035 | 0.4246 | No |
| 151 | GNAI1 |  |  | 10557 | 0.034 | 0.4221 | No |
| 152 | TCF4 |  |  | 10696 | 0.032 | 0.4160 | No |
| 153 | NDUFA7 |  |  | 11068 | 0.026 | 0.3984 | No |
| 154 | C4ORF32 |  |  | 11165 | 0.025 | 0.3942 | No |
| 155 | IFI16 |  |  | 11265 | 0.024 | 0.3899 | No |
| 156 | GPA33 |  |  | 11276 | 0.024 | 0.3899 | No |
| 157 | ZNF720 |  |  | 11306 | 0.024 | 0.3890 | No |
| 158 | NME1 |  |  | 11525 | 0.020 | 0.3787 | No |
| 159 | CSAD |  |  | 11540 | 0.020 | 0.3784 | No |
| 160 | GPR146 |  |  | 11666 | 0.018 | 0.3727 | No |
| 161 | QSCN6L1 |  |  | 11817 | 0.016 | 0.3657 | No |
| 162 | MPP7 |  |  | 12076 | 0.013 | 0.3533 | No |
| 163 | PAICS |  |  | 12225 | 0.011 | 0.3463 | No |
| 164 | KBTBD6 |  |  | 12473 | 0.007 | 0.3343 | No |
| 165 | TMEM156 |  |  | 12489 | 0.007 | 0.3337 | No |
| 166 | RHOU |  |  | 12701 | 0.003 | 0.3235 | No |
| 167 | ITM2A |  |  | 13336 | -0.006 | 0.2925 | No |
| 168 | FLJ37440 |  |  | 13420 | -0.008 | 0.2886 | No |
| 169 | HIST2H2AA /// LOC653 |  |  | 13472 | -0.008 | 0.2863 | No |
| 170 | AIF1 |  |  | 13617 | -0.010 | 0.2795 | No |
| 171 | PSMB2 |  |  | 13941 | -0.015 | 0.2639 | No |
| 172 | LOC148203 |  |  | 13992 | -0.016 | 0.2618 | No |
| 173 | RNASE6 |  |  | 14262 | -0.020 | 0.2491 | No |
| 174 | GZMA |  |  | 14269 | -0.020 | 0.2492 | No |
| 175 | PFDN6 |  |  | 14291 | -0.020 | 0.2486 | No |
| 176 | GATA3 |  |  | 14301 | -0.021 | 0.2486 | No |
| 177 | TNFSF10 |  |  | 14451 | -0.023 | 0.2418 | No |
| 178 | B3GNT2 |  |  | 15049 | -0.033 | 0.2132 | No |
| 179 | VAV3 |  |  | 15157 | -0.034 | 0.2087 | No |
| 180 | KIAA0319L |  |  | 15319 | -0.037 | 0.2015 | No |
| 181 | NAPSB |  |  | 15322 | -0.037 | 0.2022 | No |
| 182 | CTPS |  |  | 15695 | -0.043 | 0.1849 | No |
| 183 | SFRS10 |  |  | 16396 | -0.057 | 0.1518 | No |
| 184 | CCR7 |  |  | 16616 | -0.062 | 0.1423 | No |
| 185 | GPR171 |  |  | 16812 | -0.065 | 0.1341 | No |
| 186 | HBG1 /// HBG2 |  |  | 16923 | -0.068 | 0.1302 | No |
| 187 | RHOBTB3 |  |  | 16997 | -0.070 | 0.1281 | No |
| 188 | HIST1H2BK |  |  | 17029 | -0.071 | 0.1280 | No |
| 189 | ZCCHC9 |  |  | 17198 | -0.075 | 0.1214 | No |
| 190 | LOC205251 |  |  | 18106 | -0.102 | 0.0791 | No |
| 191 | P2RX5 |  |  | 18164 | -0.105 | 0.0785 | No |
| 192 | MPEG1 |  |  | 18314 | -0.110 | 0.0735 | No |
| 193 | FUT8 |  |  | 18841 | -0.133 | 0.0505 | No |
| 194 | HIST1H2AC |  |  | 19190 | -0.152 | 0.0367 | No |
| 195 | TKT |  |  | 19574 | -0.186 | 0.0218 | No |
| 196 | CKS2 |  |  | 19619 | -0.191 | 0.0237 | No |
| 197 | PRPF19 |  |  | 19713 | -0.202 | 0.0234 | No |
| 198 | HDAC2 |  |  | 19736 | -0.204 | 0.0266 | No |
| 199 | MIF |  |  | 19823 | -0.215 | 0.0269 | No |
| 200 | HIST1H2BD |  |  | 19997 | -0.240 | 0.0235 | No |
| 201 | ISG15 |  |  | 20207 | -0.298 | 0.0195 | No |
Table: GSEA details [plain text format]

  

Fig 2: LEE\_TCELLS1\_UP: Random ES distribution      
 Gene set null distribution of ES for **LEE\_TCELLS1\_UP**
