# Supplementary material for: The rapamycin-regulated gene expression signature determines prognosis for breast cancer
Source: Mol Cancer. 2009 Sep 24;8:75. doi: 10.1186/1476-4598-8-75 (PMC2761377; doi:10.1186/1476-4598-8-75)
Supplement: Additional file 2 — Gene set enrichment analysis of in vivo data, time series. The data provided represent the time series of GSEA. This compressed file contains "Time" shortcut file and "GSEA_time" folder. Clicking on "Time" shortcut opens the index file providing access to analysis files contained in the "GSEA_time" folder. [file 1476-4598-8-75-S2.zip › GSEA_time/LI_FETAL_VS_WT_KIDNEY_UP.html]

Details for gene set LI\_FETAL\_VS\_WT\_KIDNEY\_UP[GSEA]

|  || Dataset | gsea\_time\_collapsed |
| Phenotype | NoPhenotypeAvailable |
| Upregulated in class | na\_pos |
| GeneSet | LI\_FETAL\_VS\_WT\_KIDNEY\_UP |
| Enrichment Score (ES) | 0.5831474 |
| Normalized Enrichment Score (NES) | 1.7617252 |
| Nominal p-value | 0.0 |
| FDR q-value | 0.011004881 |
| FWER p-Value | 0.331 |
Table: GSEA Results Summary

  

Fig 1: Enrichment plot: LI\_FETAL\_VS\_WT\_KIDNEY\_UP      
 Profile of the Running ES Score & Positions of GeneSet Members on the Rank Ordered List

  

| PROBE | GENE SYMBOL | GENE\_TITLE | RANK IN GENE LIST | RANK METRIC SCORE | RUNNING ES | CORE ENRICHMENT || 1 | IGFBP7 |  |  | 2 | 2.165 | 0.0495 | Yes |
| 2 | MYLK |  |  | 5 | 1.990 | 0.0950 | Yes |
| 3 | CRIM1 |  |  | 16 | 1.496 | 0.1288 | Yes |
| 4 | TRIM2 |  |  | 76 | 0.965 | 0.1480 | Yes |
| 5 | FAM3C |  |  | 81 | 0.949 | 0.1696 | Yes |
| 6 | PRPS2 |  |  | 152 | 0.799 | 0.1844 | Yes |
| 7 | PMP22 |  |  | 191 | 0.740 | 0.1995 | Yes |
| 8 | VEGF |  |  | 252 | 0.681 | 0.2122 | Yes |
| 9 | ATP1B1 |  |  | 270 | 0.672 | 0.2268 | Yes |
| 10 | PRNP |  |  | 279 | 0.668 | 0.2417 | Yes |
| 11 | GNG11 |  |  | 283 | 0.666 | 0.2568 | Yes |
| 12 | ASAH1 |  |  | 286 | 0.662 | 0.2719 | Yes |
| 13 | PLAU |  |  | 302 | 0.647 | 0.2860 | Yes |
| 14 | SLC30A1 |  |  | 334 | 0.618 | 0.2986 | Yes |
| 15 | UGCG |  |  | 335 | 0.617 | 0.3128 | Yes |
| 16 | GNG12 |  |  | 372 | 0.596 | 0.3247 | Yes |
| 17 | GUCY1A3 |  |  | 376 | 0.595 | 0.3382 | Yes |
| 18 | CARD10 |  |  | 486 | 0.539 | 0.3452 | Yes |
| 19 | LAMP2 |  |  | 564 | 0.511 | 0.3531 | Yes |
| 20 | LEPR |  |  | 595 | 0.501 | 0.3631 | Yes |
| 21 | ITM2B |  |  | 596 | 0.501 | 0.3746 | Yes |
| 22 | DAB2 |  |  | 659 | 0.482 | 0.3826 | Yes |
| 23 | CDKN1C |  |  | 693 | 0.475 | 0.3919 | Yes |
| 24 | CRYAB |  |  | 696 | 0.475 | 0.4027 | Yes |
| 25 | DST |  |  | 702 | 0.473 | 0.4133 | Yes |
| 26 | SERINC5 |  |  | 719 | 0.470 | 0.4233 | Yes |
| 27 | PODXL |  |  | 755 | 0.461 | 0.4321 | Yes |
| 28 | DSTN |  |  | 840 | 0.436 | 0.4380 | Yes |
| 29 | ANK3 |  |  | 869 | 0.429 | 0.4464 | Yes |
| 30 | SCP2 |  |  | 896 | 0.424 | 0.4549 | Yes |
| 31 | KDR |  |  | 957 | 0.413 | 0.4614 | Yes |
| 32 | IL10RB |  |  | 1028 | 0.401 | 0.4672 | Yes |
| 33 | SLC35A3 |  |  | 1138 | 0.380 | 0.4705 | Yes |
| 34 | CAST |  |  | 1143 | 0.379 | 0.4790 | Yes |
| 35 | SP100 |  |  | 1230 | 0.365 | 0.4832 | Yes |
| 36 | PLS3 |  |  | 1277 | 0.358 | 0.4891 | Yes |
| 37 | ANGPT1 |  |  | 1448 | 0.335 | 0.4885 | Yes |
| 38 | SEPP1 |  |  | 1538 | 0.325 | 0.4915 | Yes |
| 39 | TOB1 |  |  | 1542 | 0.324 | 0.4988 | Yes |
| 40 | FER1L3 |  |  | 1615 | 0.316 | 0.5026 | Yes |
| 41 | PLCG2 |  |  | 1781 | 0.301 | 0.5014 | Yes |
| 42 | ALDH6A1 |  |  | 1825 | 0.298 | 0.5061 | Yes |
| 43 | NR3C1 |  |  | 1893 | 0.293 | 0.5095 | Yes |
| 44 | KIAA1026 |  |  | 1935 | 0.290 | 0.5142 | Yes |
| 45 | IQGAP1 |  |  | 1950 | 0.288 | 0.5201 | Yes |
| 46 | PDE8A |  |  | 1979 | 0.286 | 0.5253 | Yes |
| 47 | S100A10 |  |  | 2017 | 0.283 | 0.5299 | Yes |
| 48 | CEBPD |  |  | 2123 | 0.275 | 0.5311 | Yes |
| 49 | SC4MOL |  |  | 2128 | 0.275 | 0.5372 | Yes |
| 50 | PPAP2A |  |  | 2289 | 0.262 | 0.5353 | Yes |
| 51 | ACSL1 |  |  | 2341 | 0.259 | 0.5388 | Yes |
| 52 | ATP5L |  |  | 2417 | 0.253 | 0.5409 | Yes |
| 53 | CDS1 |  |  | 2420 | 0.253 | 0.5466 | Yes |
| 54 | TPST1 |  |  | 2422 | 0.253 | 0.5524 | Yes |
| 55 | TOM1L1 |  |  | 2515 | 0.247 | 0.5535 | Yes |
| 56 | RBPMS |  |  | 2550 | 0.245 | 0.5575 | Yes |
| 57 | APOE |  |  | 2558 | 0.244 | 0.5627 | Yes |
| 58 | KCTD12 |  |  | 2703 | 0.236 | 0.5611 | Yes |
| 59 | CREBL2 |  |  | 2705 | 0.235 | 0.5664 | Yes |
| 60 | PEX7 |  |  | 2812 | 0.229 | 0.5665 | Yes |
| 61 | MTMR6 |  |  | 2910 | 0.224 | 0.5669 | Yes |
| 62 | TGFBR2 |  |  | 3160 | 0.211 | 0.5595 | Yes |
| 63 | ZNF44 |  |  | 3245 | 0.206 | 0.5601 | Yes |
| 64 | SERPINF1 |  |  | 3273 | 0.205 | 0.5635 | Yes |
| 65 | PLS1 |  |  | 3382 | 0.200 | 0.5628 | Yes |
| 66 | PDXK |  |  | 3385 | 0.200 | 0.5673 | Yes |
| 67 | CD2AP |  |  | 3440 | 0.198 | 0.5692 | Yes |
| 68 | SEMA3C |  |  | 3448 | 0.197 | 0.5734 | Yes |
| 69 | KIAA0143 |  |  | 3535 | 0.192 | 0.5735 | Yes |
| 70 | SSFA2 |  |  | 3584 | 0.191 | 0.5756 | Yes |
| 71 | CAV2 |  |  | 3683 | 0.188 | 0.5751 | Yes |
| 72 | MYH9 |  |  | 3732 | 0.185 | 0.5770 | Yes |
| 73 | HRSP12 |  |  | 3810 | 0.182 | 0.5774 | Yes |
| 74 | CTDSPL |  |  | 3921 | 0.177 | 0.5760 | Yes |
| 75 | ETS2 |  |  | 3927 | 0.177 | 0.5798 | Yes |
| 76 | TRAM2 |  |  | 3969 | 0.175 | 0.5818 | Yes |
| 77 | AXL |  |  | 4024 | 0.173 | 0.5831 | Yes |
| 78 | TGOLN2 |  |  | 4193 | 0.165 | 0.5787 | No |
| 79 | MITF |  |  | 4295 | 0.161 | 0.5774 | No |
| 80 | LYN |  |  | 4337 | 0.160 | 0.5791 | No |
| 81 | TACSTD2 |  |  | 4511 | 0.154 | 0.5742 | No |
| 82 | PRCP |  |  | 4580 | 0.151 | 0.5743 | No |
| 83 | CYB5A |  |  | 4648 | 0.149 | 0.5744 | No |
| 84 | ALCAM |  |  | 4881 | 0.141 | 0.5663 | No |
| 85 | ELF1 |  |  | 4957 | 0.138 | 0.5658 | No |
| 86 | NCOA3 |  |  | 5249 | 0.131 | 0.5545 | No |
| 87 | SLC22A5 |  |  | 5427 | 0.126 | 0.5487 | No |
| 88 | PDZRN3 |  |  | 5490 | 0.124 | 0.5486 | No |
| 89 | TNFAIP8 |  |  | 5514 | 0.124 | 0.5503 | No |
| 90 | MATN2 |  |  | 5576 | 0.122 | 0.5501 | No |
| 91 | CDH5 |  |  | 5806 | 0.117 | 0.5415 | No |
| 92 | GPRC5B |  |  | 6098 | 0.111 | 0.5298 | No |
| 93 | CA12 |  |  | 6270 | 0.106 | 0.5239 | No |
| 94 | ANXA4 |  |  | 6364 | 0.104 | 0.5217 | No |
| 95 | AHNAK |  |  | 6393 | 0.104 | 0.5227 | No |
| 96 | AMOTL2 |  |  | 6429 | 0.103 | 0.5234 | No |
| 97 | PGRMC2 |  |  | 6460 | 0.102 | 0.5243 | No |
| 98 | HPN |  |  | 7196 | 0.088 | 0.4903 | No |
| 99 | LRRC32 |  |  | 7266 | 0.086 | 0.4889 | No |
| 100 | ERG |  |  | 7314 | 0.085 | 0.4885 | No |
| 101 | REEP5 |  |  | 7413 | 0.084 | 0.4856 | No |
| 102 | MAN2A1 |  |  | 7584 | 0.080 | 0.4792 | No |
| 103 | ZFHX1B |  |  | 7962 | 0.073 | 0.4624 | No |
| 104 | SPP1 |  |  | 7970 | 0.073 | 0.4637 | No |
| 105 | RGL1 |  |  | 8184 | 0.070 | 0.4549 | No |
| 106 | PDE6B |  |  | 8281 | 0.068 | 0.4517 | No |
| 107 | MAGI2 |  |  | 8309 | 0.068 | 0.4520 | No |
| 108 | LLGL2 |  |  | 8364 | 0.067 | 0.4509 | No |
| 109 | TMED5 |  |  | 8401 | 0.066 | 0.4506 | No |
| 110 | CTSO |  |  | 8557 | 0.064 | 0.4445 | No |
| 111 | GADD45A |  |  | 8579 | 0.063 | 0.4449 | No |
| 112 | TFPI |  |  | 8641 | 0.063 | 0.4434 | No |
| 113 | DYNC2LI1 |  |  | 8698 | 0.062 | 0.4420 | No |
| 114 | PATZ1 |  |  | 8771 | 0.061 | 0.4399 | No |
| 115 | IL27RA |  |  | 8787 | 0.060 | 0.4406 | No |
| 116 | MAF |  |  | 9000 | 0.057 | 0.4315 | No |
| 117 | LDB2 |  |  | 9047 | 0.056 | 0.4305 | No |
| 118 | HERPUD1 |  |  | 9397 | 0.051 | 0.4146 | No |
| 119 | FGF9 |  |  | 9542 | 0.049 | 0.4087 | No |
| 120 | RAB22A |  |  | 9674 | 0.047 | 0.4033 | No |
| 121 | ST3GAL6 |  |  | 9680 | 0.047 | 0.4042 | No |
| 122 | CRADD |  |  | 9849 | 0.044 | 0.3970 | No |
| 123 | ZNF124 |  |  | 9874 | 0.044 | 0.3968 | No |
| 124 | CLU |  |  | 9929 | 0.043 | 0.3951 | No |
| 125 | CTSH |  |  | 9994 | 0.042 | 0.3930 | No |
| 126 | GNAI1 |  |  | 10557 | 0.034 | 0.3662 | No |
| 127 | ALDH4A1 |  |  | 10627 | 0.033 | 0.3636 | No |
| 128 | ACSM3 |  |  | 10949 | 0.028 | 0.3485 | No |
| 129 | KIFC3 |  |  | 11002 | 0.027 | 0.3466 | No |
| 130 | SMPDL3A |  |  | 11086 | 0.026 | 0.3432 | No |
| 131 | OXCT1 |  |  | 11286 | 0.024 | 0.3339 | No |
| 132 | BPGM |  |  | 11483 | 0.021 | 0.3248 | No |
| 133 | SLC37A4 |  |  | 11656 | 0.018 | 0.3168 | No |
| 134 | CLDN3 |  |  | 11742 | 0.017 | 0.3131 | No |
| 135 | ANXA3 |  |  | 11767 | 0.017 | 0.3123 | No |
| 136 | KIF13B |  |  | 12043 | 0.013 | 0.2991 | No |
| 137 | OLFML2A |  |  | 12284 | 0.010 | 0.2876 | No |
| 138 | IQGAP2 |  |  | 12641 | 0.004 | 0.2703 | No |
| 139 | ATP1A1 |  |  | 12729 | 0.002 | 0.2661 | No |
| 140 | KIAA0960 |  |  | 12873 | 0.000 | 0.2591 | No |
| 141 | CDH1 |  |  | 12976 | -0.001 | 0.2541 | No |
| 142 | BRP44 |  |  | 13036 | -0.002 | 0.2513 | No |
| 143 | AGRIN |  |  | 13257 | -0.005 | 0.2406 | No |
| 144 | COL4A5 |  |  | 13539 | -0.009 | 0.2270 | No |
| 145 | TBC1D9 |  |  | 13572 | -0.010 | 0.2257 | No |
| 146 | PPP1R3C |  |  | 13583 | -0.010 | 0.2254 | No |
| 147 | GLUD2 |  |  | 13593 | -0.010 | 0.2252 | No |
| 148 | IL1R1 |  |  | 13760 | -0.012 | 0.2174 | No |
| 149 | DUSP9 |  |  | 14326 | -0.021 | 0.1902 | No |
| 150 | TCF21 |  |  | 14736 | -0.027 | 0.1708 | No |
| 151 | TEK |  |  | 14907 | -0.030 | 0.1632 | No |
| 152 | PPP2R5A |  |  | 14908 | -0.030 | 0.1639 | No |
| 153 | APOM |  |  | 15846 | -0.046 | 0.1190 | No |
| 154 | IQCK |  |  | 16377 | -0.056 | 0.0944 | No |
| 155 | FPGT |  |  | 16436 | -0.058 | 0.0929 | No |
| 156 | COL1A2 |  |  | 16667 | -0.063 | 0.0830 | No |
| 157 | FUCA1 |  |  | 16857 | -0.067 | 0.0753 | No |
| 158 | ENPEP |  |  | 17013 | -0.070 | 0.0693 | No |
| 159 | TSPO |  |  | 17026 | -0.071 | 0.0704 | No |
| 160 | CRYZ |  |  | 17418 | -0.081 | 0.0531 | No |
| 161 | FGFR3 |  |  | 17911 | -0.096 | 0.0312 | No |
| 162 | PPL |  |  | 17935 | -0.097 | 0.0323 | No |
| 163 | SDHD |  |  | 18044 | -0.101 | 0.0293 | No |
| 164 | MERTK |  |  | 18330 | -0.111 | 0.0179 | No |
| 165 | ATP10D |  |  | 18461 | -0.116 | 0.0142 | No |
| 166 | VLDLR |  |  | 18736 | -0.127 | 0.0037 | No |
| 167 | BLVRB |  |  | 18773 | -0.130 | 0.0049 | No |
| 168 | FRY |  |  | 18989 | -0.140 | -0.0024 | No |
| 169 | ALDH1A1 |  |  | 19486 | -0.177 | -0.0226 | No |
| 170 | IFIT1 |  |  | 19898 | -0.225 | -0.0376 | No |
| 171 | SEPHS2 |  |  | 19963 | -0.234 | -0.0354 | No |
| 172 | KRT19 |  |  | 20041 | -0.252 | -0.0334 | No |
| 173 | SYNGR2 |  |  | 20125 | -0.269 | -0.0313 | No |
| 174 | ACOT2 /// ACOT1 /// |  |  | 20252 | -0.312 | -0.0303 | No |
| 175 | CX3CL1 |  |  | 20359 | -0.360 | -0.0272 | No |
| 176 | XBP1 |  |  | 20381 | -0.370 | -0.0198 | No |
| 177 | AKR1C3 |  |  | 20409 | -0.394 | -0.0121 | No |
| 178 | IMPA2 |  |  | 20423 | -0.403 | -0.0035 | No |
| 179 | CPVL |  |  | 20549 | -0.540 | 0.0027 | No |
Table: GSEA details [plain text format]

  

Fig 2: LI\_FETAL\_VS\_WT\_KIDNEY\_UP: Random ES distribution      
 Gene set null distribution of ES for **LI\_FETAL\_VS\_WT\_KIDNEY\_UP**
