# Supplementary material for: The rapamycin-regulated gene expression signature determines prognosis for breast cancer
Source: Mol Cancer. 2009 Sep 24;8:75. doi: 10.1186/1476-4598-8-75 (PMC2761377; doi:10.1186/1476-4598-8-75)
Supplement: Additional file 2 — Gene set enrichment analysis of in vivo data, time series. The data provided represent the time series of GSEA. This compressed file contains "Time" shortcut file and "GSEA_time" folder. Clicking on "Time" shortcut opens the index file providing access to analysis files contained in the "GSEA_time" folder. [file 1476-4598-8-75-S2.zip › GSEA_time/LYSINE_DEGRADATION.html]

Details for gene set LYSINE\_DEGRADATION[GSEA]

|  || Dataset | gsea\_time\_collapsed |
| Phenotype | NoPhenotypeAvailable |
| Upregulated in class | na\_neg |
| GeneSet | LYSINE\_DEGRADATION |
| Enrichment Score (ES) | -0.3925971 |
| Normalized Enrichment Score (NES) | -1.4066535 |
| Nominal p-value | 0.05 |
| FDR q-value | 0.2840819 |
| FWER p-Value | 1.0 |
Table: GSEA Results Summary

  

Fig 1: Enrichment plot: LYSINE\_DEGRADATION      
 Profile of the Running ES Score & Positions of GeneSet Members on the Rank Ordered List

  

| PROBE | GENE SYMBOL | GENE\_TITLE | RANK IN GENE LIST | RANK METRIC SCORE | RUNNING ES | CORE ENRICHMENT || 1 | AASDHPPT |  |  | 1570 | 0.321 | -0.0114 | No |
| 2 | TMLHE |  |  | 2391 | 0.255 | 0.0005 | No |
| 3 | HADHA |  |  | 2731 | 0.234 | 0.0314 | No |
| 4 | ALDH1A3 |  |  | 3109 | 0.214 | 0.0564 | No |
| 5 | AASDH |  |  | 3437 | 0.198 | 0.0805 | No |
| 6 | PLOD2 |  |  | 3812 | 0.182 | 0.0991 | No |
| 7 | EHHADH |  |  | 3947 | 0.176 | 0.1282 | No |
| 8 | ACAT1 |  |  | 4720 | 0.147 | 0.1204 | No |
| 9 | ALDH1B1 |  |  | 4736 | 0.146 | 0.1493 | No |
| 10 | SHMT2 |  |  | 7160 | 0.088 | 0.0494 | No |
| 11 | ALDH1A2 |  |  | 7879 | 0.075 | 0.0296 | No |
| 12 | DOT1L |  |  | 8746 | 0.061 | -0.0001 | No |
| 13 | SDS |  |  | 8851 | 0.059 | 0.0068 | No |
| 14 | SHMT1 |  |  | 9175 | 0.054 | 0.0021 | No |
| 15 | EHMT2 |  |  | 10250 | 0.038 | -0.0424 | No |
| 16 | AASS |  |  | 14220 | -0.019 | -0.2314 | No |
| 17 | ALDH2 |  |  | 14955 | -0.031 | -0.2608 | No |
| 18 | DLST /// DLSTP |  |  | 15717 | -0.043 | -0.2890 | No |
| 19 | ALDH3A1 |  |  | 16424 | -0.057 | -0.3117 | No |
| 20 | AADAT |  |  | 17996 | -0.099 | -0.3681 | Yes |
| 21 | EHMT1 |  |  | 18402 | -0.113 | -0.3648 | Yes |
| 22 | ALDH3A2 |  |  | 18618 | -0.123 | -0.3505 | Yes |
| 23 | ALDH1A1 |  |  | 19486 | -0.177 | -0.3568 | Yes |
| 24 | PLOD3 |  |  | 19683 | -0.198 | -0.3263 | Yes |
| 25 | BBOX1 |  |  | 19756 | -0.206 | -0.2881 | Yes |
| 26 | ECHS1 |  |  | 19778 | -0.209 | -0.2469 | Yes |
| 27 | GCDH |  |  | 19987 | -0.239 | -0.2087 | Yes |
| 28 | PLOD1 |  |  | 20259 | -0.316 | -0.1579 | Yes |
| 29 | ALDH9A1 |  |  | 20364 | -0.363 | -0.0894 | Yes |
| 30 | ACAT2 |  |  | 20518 | -0.499 | 0.0042 | Yes |
Table: GSEA details [plain text format]

  

Fig 2: LYSINE\_DEGRADATION: Random ES distribution      
 Gene set null distribution of ES for **LYSINE\_DEGRADATION**
