# Supplementary material for: The rapamycin-regulated gene expression signature determines prognosis for breast cancer
Source: Mol Cancer. 2009 Sep 24;8:75. doi: 10.1186/1476-4598-8-75 (PMC2761377; doi:10.1186/1476-4598-8-75)
Supplement: Additional file 2 — Gene set enrichment analysis of in vivo data, time series. The data provided represent the time series of GSEA. This compressed file contains "Time" shortcut file and "GSEA_time" folder. Clicking on "Time" shortcut opens the index file providing access to analysis files contained in the "GSEA_time" folder. [file 1476-4598-8-75-S2.zip › GSEA_time/MANALO_HYPOXIA_UP.html]

Details for gene set MANALO\_HYPOXIA\_UP[GSEA]

|  || Dataset | gsea\_time\_collapsed |
| Phenotype | NoPhenotypeAvailable |
| Upregulated in class | na\_pos |
| GeneSet | MANALO\_HYPOXIA\_UP |
| Enrichment Score (ES) | 0.62467384 |
| Normalized Enrichment Score (NES) | 1.789723 |
| Nominal p-value | 0.0 |
| FDR q-value | 0.008225988 |
| FWER p-Value | 0.224 |
Table: GSEA Results Summary

  

Fig 1: Enrichment plot: MANALO\_HYPOXIA\_UP      
 Profile of the Running ES Score & Positions of GeneSet Members on the Rank Ordered List

  

| PROBE | GENE SYMBOL | GENE\_TITLE | RANK IN GENE LIST | RANK METRIC SCORE | RUNNING ES | CORE ENRICHMENT || 1 | STC1 |  |  | 12 | 1.592 | 0.0604 | Yes |
| 2 | FKBP9 |  |  | 46 | 1.138 | 0.1024 | Yes |
| 3 | INHBA |  |  | 69 | 0.995 | 0.1395 | Yes |
| 4 | DUSP6 |  |  | 94 | 0.912 | 0.1733 | Yes |
| 5 | MAFF |  |  | 109 | 0.883 | 0.2064 | Yes |
| 6 | CXCR4 |  |  | 186 | 0.747 | 0.2314 | Yes |
| 7 | VEGF |  |  | 252 | 0.681 | 0.2543 | Yes |
| 8 | STC2 |  |  | 256 | 0.679 | 0.2801 | Yes |
| 9 | PAM |  |  | 257 | 0.679 | 0.3061 | Yes |
| 10 | LIMS1 |  |  | 261 | 0.677 | 0.3319 | Yes |
| 11 | PTGIS |  |  | 273 | 0.669 | 0.3571 | Yes |
| 12 | EGLN3 |  |  | 297 | 0.650 | 0.3808 | Yes |
| 13 | ZNF292 |  |  | 582 | 0.505 | 0.3863 | Yes |
| 14 | LEPR |  |  | 595 | 0.501 | 0.4050 | Yes |
| 15 | NDRG1 |  |  | 599 | 0.500 | 0.4240 | Yes |
| 16 | HES1 |  |  | 618 | 0.493 | 0.4420 | Yes |
| 17 | ITPR2 |  |  | 665 | 0.481 | 0.4582 | Yes |
| 18 | CDKN1C |  |  | 693 | 0.475 | 0.4751 | Yes |
| 19 | COL4A2 |  |  | 901 | 0.423 | 0.4812 | Yes |
| 20 | SOX4 |  |  | 910 | 0.421 | 0.4969 | Yes |
| 21 | ADM |  |  | 932 | 0.417 | 0.5119 | Yes |
| 22 | AK3 |  |  | 1022 | 0.401 | 0.5229 | Yes |
| 23 | GABRP |  |  | 1206 | 0.370 | 0.5282 | Yes |
| 24 | ERO1L |  |  | 1273 | 0.358 | 0.5387 | Yes |
| 25 | COL4A1 |  |  | 1344 | 0.348 | 0.5486 | Yes |
| 26 | IGFBP3 |  |  | 1528 | 0.326 | 0.5522 | Yes |
| 27 | PTPRR |  |  | 1532 | 0.325 | 0.5645 | Yes |
| 28 | MEF2A |  |  | 1602 | 0.318 | 0.5733 | Yes |
| 29 | PLCG2 |  |  | 1781 | 0.301 | 0.5762 | Yes |
| 30 | MXI1 |  |  | 1838 | 0.297 | 0.5848 | Yes |
| 31 | CDH2 |  |  | 1872 | 0.294 | 0.5945 | Yes |
| 32 | TNFRSF10B |  |  | 2060 | 0.279 | 0.5961 | Yes |
| 33 | TRIO |  |  | 2122 | 0.275 | 0.6036 | Yes |
| 34 | CCNG2 |  |  | 2273 | 0.263 | 0.6064 | Yes |
| 35 | P4HA2 |  |  | 2642 | 0.239 | 0.5976 | Yes |
| 36 | TLE1 |  |  | 2761 | 0.232 | 0.6007 | Yes |
| 37 | PGF |  |  | 2843 | 0.228 | 0.6055 | Yes |
| 38 | LOX |  |  | 2848 | 0.227 | 0.6140 | Yes |
| 39 | GADD45B |  |  | 2916 | 0.224 | 0.6193 | Yes |
| 40 | ZBTB1 |  |  | 3053 | 0.216 | 0.6210 | Yes |
| 41 | CREB3L2 |  |  | 3169 | 0.211 | 0.6235 | Yes |
| 42 | NPAS2 |  |  | 3432 | 0.198 | 0.6183 | Yes |
| 43 | EPOR |  |  | 3498 | 0.194 | 0.6226 | Yes |
| 44 | PDGFB |  |  | 3659 | 0.189 | 0.6220 | Yes |
| 45 | PLOD2 |  |  | 3812 | 0.182 | 0.6215 | Yes |
| 46 | RPS6KA2 |  |  | 3936 | 0.176 | 0.6223 | Yes |
| 47 | AXL |  |  | 4024 | 0.173 | 0.6247 | Yes |
| 48 | TXNIP |  |  | 4158 | 0.166 | 0.6246 | No |
| 49 | EGLN1 |  |  | 4430 | 0.157 | 0.6174 | No |
| 50 | TNFRSF14 |  |  | 4460 | 0.156 | 0.6219 | No |
| 51 | RGS3 |  |  | 4747 | 0.146 | 0.6136 | No |
| 52 | ACVR1B |  |  | 4923 | 0.139 | 0.6104 | No |
| 53 | BCL6 |  |  | 5124 | 0.134 | 0.6058 | No |
| 54 | HIVEP2 |  |  | 5168 | 0.133 | 0.6088 | No |
| 55 | PTPRB |  |  | 5430 | 0.126 | 0.6008 | No |
| 56 | ADORA2A |  |  | 5493 | 0.124 | 0.6026 | No |
| 57 | OPN3 |  |  | 5957 | 0.114 | 0.5844 | No |
| 58 | ANGPTL4 |  |  | 6010 | 0.113 | 0.5861 | No |
| 59 | LOXL2 |  |  | 6308 | 0.105 | 0.5757 | No |
| 60 | WASF2 |  |  | 7296 | 0.086 | 0.5309 | No |
| 61 | RRAS |  |  | 7849 | 0.075 | 0.5068 | No |
| 62 | INHBE |  |  | 7963 | 0.073 | 0.5041 | No |
| 63 | CASK |  |  | 8354 | 0.067 | 0.4877 | No |
| 64 | INSR |  |  | 8647 | 0.063 | 0.4758 | No |
| 65 | GDF10 |  |  | 8840 | 0.060 | 0.4688 | No |
| 66 | EDN1 |  |  | 8935 | 0.058 | 0.4664 | No |
| 67 | NOTCH4 |  |  | 9132 | 0.055 | 0.4589 | No |
| 68 | ZHX2 |  |  | 9402 | 0.051 | 0.4478 | No |
| 69 | PTPRF |  |  | 9518 | 0.050 | 0.4441 | No |
| 70 | NPR1 |  |  | 9980 | 0.042 | 0.4232 | No |
| 71 | HIF3A |  |  | 10033 | 0.042 | 0.4223 | No |
| 72 | RLN1 |  |  | 10313 | 0.038 | 0.4101 | No |
| 73 | RHOBTB1 |  |  | 10755 | 0.031 | 0.3898 | No |
| 74 | COL18A1 |  |  | 11082 | 0.026 | 0.3749 | No |
| 75 | COL9A1 |  |  | 12359 | 0.009 | 0.3130 | No |
| 76 | PTGS1 |  |  | 12430 | 0.008 | 0.3099 | No |
| 77 | BHLHB2 |  |  | 12438 | 0.008 | 0.3099 | No |
| 78 | ENPP1 |  |  | 12454 | 0.007 | 0.3094 | No |
| 79 | SIRT3 |  |  | 12848 | 0.001 | 0.2903 | No |
| 80 | COL5A1 |  |  | 13102 | -0.003 | 0.2781 | No |
| 81 | CUBN |  |  | 13331 | -0.006 | 0.2672 | No |
| 82 | ITM2A |  |  | 13336 | -0.006 | 0.2673 | No |
| 83 | NFATC4 |  |  | 15760 | -0.044 | 0.1508 | No |
| 84 | TCF8 |  |  | 16274 | -0.054 | 0.1279 | No |
| 85 | GRK5 |  |  | 16307 | -0.055 | 0.1284 | No |
| 86 | COL1A2 |  |  | 16667 | -0.063 | 0.1133 | No |
| 87 | PELI2 |  |  | 17337 | -0.079 | 0.0837 | No |
| 88 | VEGFC |  |  | 18210 | -0.106 | 0.0453 | No |
| 89 | VLDLR |  |  | 18736 | -0.127 | 0.0246 | No |
| 90 | SHOX2 |  |  | 18969 | -0.139 | 0.0186 | No |
| 91 | PPARG |  |  | 19907 | -0.226 | -0.0184 | No |
| 92 | TCF7L1 |  |  | 20343 | -0.351 | -0.0262 | No |
| 93 | CX3CL1 |  |  | 20359 | -0.360 | -0.0132 | No |
| 94 | RASSF2 |  |  | 20580 | -0.655 | 0.0012 | No |
Table: GSEA details [plain text format]

  

Fig 2: MANALO\_HYPOXIA\_UP: Random ES distribution      
 Gene set null distribution of ES for **MANALO\_HYPOXIA\_UP**
