# Supplementary material for: The rapamycin-regulated gene expression signature determines prognosis for breast cancer
Source: Mol Cancer. 2009 Sep 24;8:75. doi: 10.1186/1476-4598-8-75 (PMC2761377; doi:10.1186/1476-4598-8-75)
Supplement: Additional file 2 — Gene set enrichment analysis of in vivo data, time series. The data provided represent the time series of GSEA. This compressed file contains "Time" shortcut file and "GSEA_time" folder. Clicking on "Time" shortcut opens the index file providing access to analysis files contained in the "GSEA_time" folder. [file 1476-4598-8-75-S2.zip › GSEA_time/MENSE_HYPOXIA_UP.html]

Details for gene set MENSE\_HYPOXIA\_UP[GSEA]

|  || Dataset | gsea\_time\_collapsed |
| Phenotype | NoPhenotypeAvailable |
| Upregulated in class | na\_pos |
| GeneSet | MENSE\_HYPOXIA\_UP |
| Enrichment Score (ES) | 0.65340114 |
| Normalized Enrichment Score (NES) | 1.9247814 |
| Nominal p-value | 0.0 |
| FDR q-value | 0.0012965453 |
| FWER p-Value | 0.0090 |
Table: GSEA Results Summary

  

Fig 1: Enrichment plot: MENSE\_HYPOXIA\_UP      
 Profile of the Running ES Score & Positions of GeneSet Members on the Rank Ordered List

  

| PROBE | GENE SYMBOL | GENE\_TITLE | RANK IN GENE LIST | RANK METRIC SCORE | RUNNING ES | CORE ENRICHMENT || 1 | ELL2 |  |  | 30 | 1.258 | 0.0400 | Yes |
| 2 | PFKFB3 |  |  | 37 | 1.222 | 0.0801 | Yes |
| 3 | PGK1 |  |  | 101 | 0.899 | 0.1067 | Yes |
| 4 | MAFF |  |  | 109 | 0.883 | 0.1355 | Yes |
| 5 | SOD2 |  |  | 128 | 0.857 | 0.1629 | Yes |
| 6 | JMJD2B |  |  | 138 | 0.829 | 0.1898 | Yes |
| 7 | WSB1 |  |  | 155 | 0.795 | 0.2153 | Yes |
| 8 | BHLHB3 |  |  | 174 | 0.767 | 0.2397 | Yes |
| 9 | ANKRD37 |  |  | 179 | 0.759 | 0.2646 | Yes |
| 10 | VEGF |  |  | 252 | 0.681 | 0.2835 | Yes |
| 11 | STC2 |  |  | 256 | 0.679 | 0.3058 | Yes |
| 12 | SLC2A3 |  |  | 278 | 0.668 | 0.3268 | Yes |
| 13 | HK2 |  |  | 330 | 0.621 | 0.3448 | Yes |
| 14 | PDK1 |  |  | 355 | 0.604 | 0.3636 | Yes |
| 15 | ASPH |  |  | 449 | 0.554 | 0.3773 | Yes |
| 16 | PPP3CA |  |  | 519 | 0.524 | 0.3912 | Yes |
| 17 | RBPSUH |  |  | 585 | 0.504 | 0.4047 | Yes |
| 18 | NDRG1 |  |  | 599 | 0.500 | 0.4206 | Yes |
| 19 | UFM1 |  |  | 606 | 0.497 | 0.4367 | Yes |
| 20 | BNIP3L |  |  | 873 | 0.428 | 0.4378 | Yes |
| 21 | ADM |  |  | 932 | 0.417 | 0.4487 | Yes |
| 22 | CCNI |  |  | 1095 | 0.388 | 0.4537 | Yes |
| 23 | WDR54 |  |  | 1101 | 0.387 | 0.4662 | Yes |
| 24 | CDC2L6 |  |  | 1111 | 0.384 | 0.4784 | Yes |
| 25 | CTTN |  |  | 1136 | 0.380 | 0.4898 | Yes |
| 26 | TIPARP |  |  | 1231 | 0.365 | 0.4972 | Yes |
| 27 | KLHL24 |  |  | 1238 | 0.364 | 0.5090 | Yes |
| 28 | RAB40C |  |  | 1251 | 0.363 | 0.5203 | Yes |
| 29 | ERO1L |  |  | 1273 | 0.358 | 0.5311 | Yes |
| 30 | LCORL |  |  | 1332 | 0.350 | 0.5399 | Yes |
| 31 | C6ORF166 |  |  | 1379 | 0.343 | 0.5489 | Yes |
| 32 | FNBP1L |  |  | 1411 | 0.340 | 0.5586 | Yes |
| 33 | TMEM65 |  |  | 1442 | 0.336 | 0.5683 | Yes |
| 34 | GPI |  |  | 1486 | 0.331 | 0.5771 | Yes |
| 35 | RIOK3 |  |  | 1721 | 0.306 | 0.5758 | Yes |
| 36 | PBEF1 /// LOC646309 |  |  | 1805 | 0.300 | 0.5816 | Yes |
| 37 | PBEF1 |  |  | 1807 | 0.299 | 0.5914 | Yes |
| 38 | MXI1 |  |  | 1838 | 0.297 | 0.5998 | Yes |
| 39 | ERICH1 |  |  | 1861 | 0.295 | 0.6084 | Yes |
| 40 | P4HA1 |  |  | 1987 | 0.285 | 0.6118 | Yes |
| 41 | BNIP3 |  |  | 2049 | 0.280 | 0.6180 | Yes |
| 42 | INSIG2 |  |  | 2070 | 0.278 | 0.6262 | Yes |
| 43 | CEBPD |  |  | 2123 | 0.275 | 0.6328 | Yes |
| 44 | LOC401152 |  |  | 2153 | 0.273 | 0.6403 | Yes |
| 45 | STK4 |  |  | 2481 | 0.249 | 0.6326 | Yes |
| 46 | ATF3 |  |  | 2567 | 0.244 | 0.6365 | Yes |
| 47 | KLF7 |  |  | 2569 | 0.244 | 0.6445 | Yes |
| 48 | P4HA2 |  |  | 2642 | 0.239 | 0.6489 | Yes |
| 49 | LOX |  |  | 2848 | 0.227 | 0.6464 | Yes |
| 50 | NFIL3 |  |  | 3085 | 0.214 | 0.6420 | Yes |
| 51 | MED6 |  |  | 3241 | 0.207 | 0.6412 | Yes |
| 52 | NADSYN1 |  |  | 3323 | 0.203 | 0.6440 | Yes |
| 53 | ADFP |  |  | 3492 | 0.194 | 0.6422 | Yes |
| 54 | FAM110C |  |  | 3669 | 0.188 | 0.6398 | Yes |
| 55 | PLOD2 |  |  | 3812 | 0.182 | 0.6389 | Yes |
| 56 | JMJD1A |  |  | 3860 | 0.180 | 0.6425 | Yes |
| 57 | DIDO1 |  |  | 3891 | 0.178 | 0.6470 | Yes |
| 58 | SLC6A6 |  |  | 3988 | 0.174 | 0.6480 | Yes |
| 59 | JMJD2C |  |  | 4180 | 0.165 | 0.6442 | Yes |
| 60 | ABCB6 |  |  | 4205 | 0.164 | 0.6484 | Yes |
| 61 | MTAC2D1 |  |  | 4269 | 0.162 | 0.6507 | Yes |
| 62 | CLPB |  |  | 4367 | 0.158 | 0.6512 | Yes |
| 63 | EGLN1 |  |  | 4430 | 0.157 | 0.6533 | Yes |
| 64 | NOL3 |  |  | 4533 | 0.153 | 0.6534 | Yes |
| 65 | ENO2 |  |  | 5094 | 0.135 | 0.6305 | No |
| 66 | RORA |  |  | 5192 | 0.132 | 0.6302 | No |
| 67 | C3ORF28 |  |  | 5223 | 0.131 | 0.6330 | No |
| 68 | GOSR2 |  |  | 5263 | 0.130 | 0.6354 | No |
| 69 | RNASE4 |  |  | 5292 | 0.129 | 0.6383 | No |
| 70 | HIG2 |  |  | 5305 | 0.129 | 0.6420 | No |
| 71 | PRPSAP1 |  |  | 5483 | 0.125 | 0.6375 | No |
| 72 | ANG /// RNASE4 |  |  | 5556 | 0.123 | 0.6380 | No |
| 73 | RP11-529I10.4 |  |  | 5788 | 0.117 | 0.6306 | No |
| 74 | PPFIA4 |  |  | 5910 | 0.115 | 0.6285 | No |
| 75 | SPAG4 |  |  | 5987 | 0.113 | 0.6285 | No |
| 76 | ANGPTL4 |  |  | 6010 | 0.113 | 0.6312 | No |
| 77 | WDR5B |  |  | 6086 | 0.111 | 0.6312 | No |
| 78 | GBE1 |  |  | 6178 | 0.109 | 0.6303 | No |
| 79 | PFKFB4 |  |  | 6375 | 0.104 | 0.6242 | No |
| 80 | KLF4 |  |  | 7984 | 0.073 | 0.5482 | No |
| 81 | FOXD1 |  |  | 8226 | 0.069 | 0.5387 | No |
| 82 | GADD45A |  |  | 8579 | 0.063 | 0.5236 | No |
| 83 | MGC21644 |  |  | 8849 | 0.059 | 0.5124 | No |
| 84 | LOC285513 |  |  | 9369 | 0.051 | 0.4888 | No |
| 85 | TSLP |  |  | 9400 | 0.051 | 0.4890 | No |
| 86 | SERGEF |  |  | 9725 | 0.046 | 0.4747 | No |
| 87 | FAM13A1 |  |  | 9759 | 0.046 | 0.4746 | No |
| 88 | LOC400027 |  |  | 9800 | 0.045 | 0.4742 | No |
| 89 | PTDSR |  |  | 10810 | 0.030 | 0.4260 | No |
| 90 | FAM119B |  |  | 11810 | 0.016 | 0.3778 | No |
| 91 | LARP6 |  |  | 12026 | 0.013 | 0.3677 | No |
| 92 | ALDOC |  |  | 12401 | 0.008 | 0.3497 | No |
| 93 | BHLHB2 |  |  | 12438 | 0.008 | 0.3482 | No |
| 94 | PEX13 |  |  | 12668 | 0.004 | 0.3372 | No |
| 95 | OSMR |  |  | 12869 | 0.000 | 0.3275 | No |
| 96 | PPP1R3C |  |  | 13583 | -0.010 | 0.2930 | No |
| 97 | PPP1R15A |  |  | 14202 | -0.019 | 0.2635 | No |
| 98 | ZBTB25 |  |  | 14670 | -0.026 | 0.2416 | No |
| 99 | LOC154761 |  |  | 15864 | -0.046 | 0.1849 | No |
| 100 | STARD4 |  |  | 16810 | -0.065 | 0.1409 | No |
| 101 | THAP8 |  |  | 17521 | -0.085 | 0.1091 | No |
| 102 | CLK3 |  |  | 17567 | -0.086 | 0.1097 | No |
| 103 | KIAA2013 |  |  | 18448 | -0.115 | 0.0706 | No |
| 104 | INSIG1 |  |  | 19038 | -0.143 | 0.0466 | No |
| 105 | HCFC1R1 |  |  | 19049 | -0.144 | 0.0508 | No |
| 106 | CEBPB |  |  | 20318 | -0.341 | 0.0002 | No |
| 107 | SCD |  |  | 20439 | -0.416 | 0.0081 | No |
Table: GSEA details [plain text format]

  

Fig 2: MENSE\_HYPOXIA\_UP: Random ES distribution      
 Gene set null distribution of ES for **MENSE\_HYPOXIA\_UP**
