# Supplementary material for: The rapamycin-regulated gene expression signature determines prognosis for breast cancer
Source: Mol Cancer. 2009 Sep 24;8:75. doi: 10.1186/1476-4598-8-75 (PMC2761377; doi:10.1186/1476-4598-8-75)
Supplement: Additional file 2 — Gene set enrichment analysis of in vivo data, time series. The data provided represent the time series of GSEA. This compressed file contains "Time" shortcut file and "GSEA_time" folder. Clicking on "Time" shortcut opens the index file providing access to analysis files contained in the "GSEA_time" folder. [file 1476-4598-8-75-S2.zip › GSEA_time/MTA3PATHWAY.html]

Details for gene set MTA3PATHWAY[GSEA]

|  || Dataset | gsea\_time\_collapsed |
| Phenotype | NoPhenotypeAvailable |
| Upregulated in class | na\_neg |
| GeneSet | MTA3PATHWAY |
| Enrichment Score (ES) | -0.5299117 |
| Normalized Enrichment Score (NES) | -1.5410365 |
| Nominal p-value | 0.07798165 |
| FDR q-value | 0.2077209 |
| FWER p-Value | 0.958 |
Table: GSEA Results Summary

  

Fig 1: Enrichment plot: MTA3PATHWAY      
 Profile of the Running ES Score & Positions of GeneSet Members on the Rank Ordered List

  

| PROBE | GENE SYMBOL | GENE\_TITLE | RANK IN GENE LIST | RANK METRIC SCORE | RUNNING ES | CORE ENRICHMENT || 1 | TUBA3 |  |  | 2634 | 0.240 | -0.0090 | No |
| 2 | CTSD |  |  | 5067 | 0.136 | -0.0599 | No |
| 3 | MTA3 |  |  | 5291 | 0.129 | -0.0066 | No |
| 4 | ESR1 |  |  | 6242 | 0.107 | 0.0004 | No |
| 5 | PDZK1 |  |  | 9012 | 0.057 | -0.1060 | No |
| 6 | GREB1 |  |  | 11903 | 0.015 | -0.2389 | No |
| 7 | TUBA1 |  |  | 13103 | -0.003 | -0.2957 | No |
| 8 | HSPB2 |  |  | 13950 | -0.015 | -0.3292 | No |
| 9 | TUBA8 |  |  | 13994 | -0.016 | -0.3234 | No |
| 10 | ALDOA |  |  | 18247 | -0.108 | -0.4765 | Yes |
| 11 | MTA1 |  |  | 18532 | -0.119 | -0.4315 | Yes |
| 12 | TUBA4 |  |  | 18645 | -0.124 | -0.3756 | Yes |
| 13 | TUBA2 |  |  | 19993 | -0.239 | -0.3224 | Yes |
| 14 | TUBA6 |  |  | 20005 | -0.242 | -0.2030 | Yes |
| 15 | HSPB1 |  |  | 20498 | -0.468 | 0.0052 | Yes |
Table: GSEA details [plain text format]

  

Fig 2: MTA3PATHWAY: Random ES distribution      
 Gene set null distribution of ES for **MTA3PATHWAY**
