# Supplementary material for: The rapamycin-regulated gene expression signature determines prognosis for breast cancer
Source: Mol Cancer. 2009 Sep 24;8:75. doi: 10.1186/1476-4598-8-75 (PMC2761377; doi:10.1186/1476-4598-8-75)
Supplement: Additional file 2 — Gene set enrichment analysis of in vivo data, time series. The data provided represent the time series of GSEA. This compressed file contains "Time" shortcut file and "GSEA_time" folder. Clicking on "Time" shortcut opens the index file providing access to analysis files contained in the "GSEA_time" folder. [file 1476-4598-8-75-S2.zip › GSEA_time/NICK_RHAPC_UP.html]

Details for gene set NICK\_RHAPC\_UP[GSEA]

|  || Dataset | gsea\_time\_collapsed |
| Phenotype | NoPhenotypeAvailable |
| Upregulated in class | na\_pos |
| GeneSet | NICK\_RHAPC\_UP |
| Enrichment Score (ES) | 0.7129402 |
| Normalized Enrichment Score (NES) | 1.837833 |
| Nominal p-value | 0.0 |
| FDR q-value | 0.0043311007 |
| FWER p-Value | 0.078 |
Table: GSEA Results Summary

  

Fig 1: Enrichment plot: NICK\_RHAPC\_UP      
 Profile of the Running ES Score & Positions of GeneSet Members on the Rank Ordered List

  

| PROBE | GENE SYMBOL | GENE\_TITLE | RANK IN GENE LIST | RANK METRIC SCORE | RUNNING ES | CORE ENRICHMENT || 1 | RPL38 |  |  | 36 | 1.222 | 0.1212 | Yes |
| 2 | CDC42SE1 |  |  | 45 | 1.142 | 0.2358 | Yes |
| 3 | ARTS-1 |  |  | 82 | 0.947 | 0.3293 | Yes |
| 4 | MAFF |  |  | 109 | 0.883 | 0.4169 | Yes |
| 5 | EIF2S3 |  |  | 292 | 0.658 | 0.4742 | Yes |
| 6 | HDGFRP3 |  |  | 534 | 0.520 | 0.5149 | Yes |
| 7 | ITPR2 |  |  | 665 | 0.481 | 0.5569 | Yes |
| 8 | FUBP1 |  |  | 830 | 0.438 | 0.5930 | Yes |
| 9 | ATP6V1A |  |  | 961 | 0.413 | 0.6282 | Yes |
| 10 | AHCYL1 |  |  | 976 | 0.410 | 0.6688 | Yes |
| 11 | ZFX |  |  | 1311 | 0.352 | 0.6879 | Yes |
| 12 | TLK1 |  |  | 1757 | 0.303 | 0.6968 | Yes |
| 13 | BRD1 |  |  | 2013 | 0.283 | 0.7129 | Yes |
| 14 | ICAM1 |  |  | 3140 | 0.212 | 0.6795 | No |
| 15 | SSFA2 |  |  | 3584 | 0.191 | 0.6772 | No |
| 16 | KIAA1840 |  |  | 4286 | 0.162 | 0.6594 | No |
| 17 | ETNK1 |  |  | 4353 | 0.159 | 0.6721 | No |
| 18 | RTF1 |  |  | 4907 | 0.140 | 0.6594 | No |
| 19 | EDD1 |  |  | 5233 | 0.131 | 0.6568 | No |
| 20 | ECHDC2 |  |  | 5261 | 0.130 | 0.6686 | No |
| 21 | NFYC |  |  | 5860 | 0.116 | 0.6512 | No |
| 22 | ALOX5 |  |  | 8205 | 0.069 | 0.5442 | No |
| 23 | C19ORF6 |  |  | 10210 | 0.039 | 0.4507 | No |
| 24 | ZNF665 |  |  | 10321 | 0.038 | 0.4491 | No |
| 25 | PRKAB1 |  |  | 11671 | 0.018 | 0.3854 | No |
| 26 | LRAP |  |  | 13312 | -0.006 | 0.3063 | No |
| 27 | FLOT1 |  |  | 13529 | -0.009 | 0.2967 | No |
| 28 | DYNC1LI1 |  |  | 13942 | -0.015 | 0.2782 | No |
| 29 | CASP9 |  |  | 15764 | -0.044 | 0.1941 | No |
| 30 | ZPBP |  |  | 15891 | -0.047 | 0.1927 | No |
| 31 | ZNF434 |  |  | 17390 | -0.080 | 0.1280 | No |
| 32 | CRELD2 |  |  | 20158 | -0.281 | 0.0217 | No |
Table: GSEA details [plain text format]

  

Fig 2: NICK\_RHAPC\_UP: Random ES distribution      
 Gene set null distribution of ES for **NICK\_RHAPC\_UP**
