# Supplementary material for: The rapamycin-regulated gene expression signature determines prognosis for breast cancer
Source: Mol Cancer. 2009 Sep 24;8:75. doi: 10.1186/1476-4598-8-75 (PMC2761377; doi:10.1186/1476-4598-8-75)
Supplement: Additional file 2 — Gene set enrichment analysis of in vivo data, time series. The data provided represent the time series of GSEA. This compressed file contains "Time" shortcut file and "GSEA_time" folder. Clicking on "Time" shortcut opens the index file providing access to analysis files contained in the "GSEA_time" folder. [file 1476-4598-8-75-S2.zip › GSEA_time/NO2IL12PATHWAY.html]

Details for gene set NO2IL12PATHWAY[GSEA]

|  || Dataset | gsea\_time\_collapsed |
| Phenotype | NoPhenotypeAvailable |
| Upregulated in class | na\_neg |
| GeneSet | NO2IL12PATHWAY |
| Enrichment Score (ES) | -0.53458434 |
| Normalized Enrichment Score (NES) | -1.6434014 |
| Nominal p-value | 0.02 |
| FDR q-value | 0.14495908 |
| FWER p-Value | 0.806 |
Table: GSEA Results Summary

  

Fig 1: Enrichment plot: NO2IL12PATHWAY      
 Profile of the Running ES Score & Positions of GeneSet Members on the Rank Ordered List

  

| PROBE | GENE SYMBOL | GENE\_TITLE | RANK IN GENE LIST | RANK METRIC SCORE | RUNNING ES | CORE ENRICHMENT || 1 | NOS2A |  |  | 5587 | 0.122 | -0.1729 | No |
| 2 | CD4 |  |  | 6147 | 0.109 | -0.1117 | No |
| 3 | CD3E |  |  | 9692 | 0.047 | -0.2461 | No |
| 4 | IL12A |  |  | 10438 | 0.036 | -0.2535 | No |
| 5 | STAT4 |  |  | 13565 | -0.010 | -0.3975 | No |
| 6 | IFNG |  |  | 13670 | -0.011 | -0.3936 | No |
| 7 | CXCR3 |  |  | 13674 | -0.011 | -0.3848 | No |
| 8 | CD2 |  |  | 13728 | -0.012 | -0.3780 | No |
| 9 | IL12RB1 |  |  | 14107 | -0.018 | -0.3820 | No |
| 10 | IL12B |  |  | 15489 | -0.039 | -0.4172 | No |
| 11 | JAK2 |  |  | 17908 | -0.096 | -0.4572 | Yes |
| 12 | IL12RB2 |  |  | 18100 | -0.102 | -0.3839 | Yes |
| 13 | CD3G |  |  | 18662 | -0.125 | -0.3104 | Yes |
| 14 | CD3D |  |  | 19309 | -0.161 | -0.2114 | Yes |
| 15 | TYK2 |  |  | 20309 | -0.339 | 0.0144 | Yes |
Table: GSEA details [plain text format]

  

Fig 2: NO2IL12PATHWAY: Random ES distribution      
 Gene set null distribution of ES for **NO2IL12PATHWAY**
