# Supplementary material for: The rapamycin-regulated gene expression signature determines prognosis for breast cancer
Source: Mol Cancer. 2009 Sep 24;8:75. doi: 10.1186/1476-4598-8-75 (PMC2761377; doi:10.1186/1476-4598-8-75)
Supplement: Additional file 2 — Gene set enrichment analysis of in vivo data, time series. The data provided represent the time series of GSEA. This compressed file contains "Time" shortcut file and "GSEA_time" folder. Clicking on "Time" shortcut opens the index file providing access to analysis files contained in the "GSEA_time" folder. [file 1476-4598-8-75-S2.zip › GSEA_time/OVARIAN_INFERTILITY_GENES.html]

Details for gene set OVARIAN\_INFERTILITY\_GENES[GSEA]

|  || Dataset | gsea\_time\_collapsed |
| Phenotype | NoPhenotypeAvailable |
| Upregulated in class | na\_neg |
| GeneSet | OVARIAN\_INFERTILITY\_GENES |
| Enrichment Score (ES) | -0.38714597 |
| Normalized Enrichment Score (NES) | -1.328881 |
| Nominal p-value | 0.090277776 |
| FDR q-value | 0.3029313 |
| FWER p-Value | 1.0 |
Table: GSEA Results Summary

  

Fig 1: Enrichment plot: OVARIAN\_INFERTILITY\_GENES      
 Profile of the Running ES Score & Positions of GeneSet Members on the Rank Ordered List

  

| PROBE | GENE SYMBOL | GENE\_TITLE | RANK IN GENE LIST | RANK METRIC SCORE | RUNNING ES | CORE ENRICHMENT || 1 | CDKN1B |  |  | 1916 | 0.291 | -0.0111 | No |
| 2 | MSH5 |  |  | 3662 | 0.188 | -0.0429 | No |
| 3 | MLH1 |  |  | 3880 | 0.179 | -0.0030 | No |
| 4 | VDR |  |  | 4278 | 0.162 | 0.0234 | No |
| 5 | NCOR1 |  |  | 4281 | 0.162 | 0.0688 | No |
| 6 | ATM |  |  | 5026 | 0.137 | 0.0712 | No |
| 7 | DMC1 |  |  | 5150 | 0.134 | 0.1029 | No |
| 8 | GJA4 |  |  | 6069 | 0.111 | 0.0896 | No |
| 9 | NR5A1 |  |  | 6599 | 0.100 | 0.0919 | No |
| 10 | INHA |  |  | 7293 | 0.086 | 0.0824 | No |
| 11 | EGR1 |  |  | 8018 | 0.072 | 0.0675 | No |
| 12 | CCND2 |  |  | 8369 | 0.067 | 0.0694 | No |
| 13 | ESR2 |  |  | 10867 | 0.029 | -0.0437 | No |
| 14 | PGR |  |  | 11474 | 0.021 | -0.0673 | No |
| 15 | SMPD1 |  |  | 12789 | 0.002 | -0.1306 | No |
| 16 | BMPR1B |  |  | 13877 | -0.014 | -0.1795 | No |
| 17 | PRLR |  |  | 14487 | -0.023 | -0.2026 | No |
| 18 | PTGER2 |  |  | 16827 | -0.066 | -0.2977 | No |
| 19 | ZP2 |  |  | 17142 | -0.074 | -0.2920 | No |
| 20 | LHCGR |  |  | 17861 | -0.094 | -0.3004 | No |
| 21 | FSHR |  |  | 19648 | -0.194 | -0.3325 | Yes |
| 22 | DAZL |  |  | 20202 | -0.296 | -0.2759 | Yes |
| 23 | NRIP1 |  |  | 20273 | -0.320 | -0.1890 | Yes |
| 24 | CEBPB |  |  | 20318 | -0.341 | -0.0951 | Yes |
| 25 | CDK4 |  |  | 20403 | -0.387 | 0.0098 | Yes |
Table: GSEA details [plain text format]

  

Fig 2: OVARIAN\_INFERTILITY\_GENES: Random ES distribution      
 Gene set null distribution of ES for **OVARIAN\_INFERTILITY\_GENES**
