# Supplementary material for: The rapamycin-regulated gene expression signature determines prognosis for breast cancer
Source: Mol Cancer. 2009 Sep 24;8:75. doi: 10.1186/1476-4598-8-75 (PMC2761377; doi:10.1186/1476-4598-8-75)
Supplement: Additional file 2 — Gene set enrichment analysis of in vivo data, time series. The data provided represent the time series of GSEA. This compressed file contains "Time" shortcut file and "GSEA_time" folder. Clicking on "Time" shortcut opens the index file providing access to analysis files contained in the "GSEA_time" folder. [file 1476-4598-8-75-S2.zip › GSEA_time/OXSTRESS_RPETHREE_DN.html]

Details for gene set OXSTRESS\_RPETHREE\_DN[GSEA]

|  || Dataset | gsea\_time\_collapsed |
| Phenotype | NoPhenotypeAvailable |
| Upregulated in class | na\_neg |
| GeneSet | OXSTRESS\_RPETHREE\_DN |
| Enrichment Score (ES) | -0.40007946 |
| Normalized Enrichment Score (NES) | -1.3818444 |
| Nominal p-value | 0.0859375 |
| FDR q-value | 0.2916141 |
| FWER p-Value | 1.0 |
Table: GSEA Results Summary

  

Fig 1: Enrichment plot: OXSTRESS\_RPETHREE\_DN      
 Profile of the Running ES Score & Positions of GeneSet Members on the Rank Ordered List

  

| PROBE | GENE SYMBOL | GENE\_TITLE | RANK IN GENE LIST | RANK METRIC SCORE | RUNNING ES | CORE ENRICHMENT || 1 | DAPK1 |  |  | 343 | 0.610 | 0.0747 | No |
| 2 | GNAS |  |  | 1005 | 0.404 | 0.1031 | No |
| 3 | RBL2 |  |  | 1365 | 0.345 | 0.1374 | No |
| 4 | CCNA2 |  |  | 1525 | 0.326 | 0.1784 | No |
| 5 | CUTL1 |  |  | 1928 | 0.290 | 0.2024 | No |
| 6 | BNIP3 |  |  | 2049 | 0.280 | 0.2385 | No |
| 7 | NFIX |  |  | 2923 | 0.223 | 0.2294 | No |
| 8 | AMPH |  |  | 3366 | 0.201 | 0.2381 | No |
| 9 | PIK3R2 |  |  | 4488 | 0.155 | 0.2068 | No |
| 10 | JUND |  |  | 6231 | 0.108 | 0.1382 | No |
| 11 | CDH3 |  |  | 6883 | 0.093 | 0.1206 | No |
| 12 | COMT |  |  | 7486 | 0.082 | 0.1036 | No |
| 13 | NF2 |  |  | 7606 | 0.080 | 0.1098 | No |
| 14 | NFKB1 |  |  | 7978 | 0.073 | 0.1027 | No |
| 15 | CASK |  |  | 8354 | 0.067 | 0.0945 | No |
| 16 | HMGA1 |  |  | 9145 | 0.055 | 0.0643 | No |
| 17 | LCAT |  |  | 9892 | 0.044 | 0.0346 | No |
| 18 | ZNF91 |  |  | 10037 | 0.041 | 0.0338 | No |
| 19 | C6ORF108 |  |  | 10645 | 0.033 | 0.0092 | No |
| 20 | ARHGDIA |  |  | 14579 | -0.025 | -0.1783 | No |
| 21 | MCM4 |  |  | 14787 | -0.028 | -0.1841 | No |
| 22 | ITGA5 |  |  | 18655 | -0.124 | -0.3534 | No |
| 23 | ADORA1 |  |  | 19213 | -0.154 | -0.3574 | No |
| 24 | ID1 |  |  | 20093 | -0.262 | -0.3609 | Yes |
| 25 | CAPNS1 |  |  | 20183 | -0.290 | -0.3219 | Yes |
| 26 | ITGB6 |  |  | 20539 | -0.526 | -0.2605 | Yes |
| 27 | POLD1 |  |  | 20588 | -0.695 | -0.1588 | Yes |
| 28 | IGFBP2 |  |  | 20605 | -1.066 | -0.0000 | Yes |
Table: GSEA details [plain text format]

  

Fig 2: OXSTRESS\_RPETHREE\_DN: Random ES distribution      
 Gene set null distribution of ES for **OXSTRESS\_RPETHREE\_DN**
