# Supplementary material for: The rapamycin-regulated gene expression signature determines prognosis for breast cancer
Source: Mol Cancer. 2009 Sep 24;8:75. doi: 10.1186/1476-4598-8-75 (PMC2761377; doi:10.1186/1476-4598-8-75)
Supplement: Additional file 2 — Gene set enrichment analysis of in vivo data, time series. The data provided represent the time series of GSEA. This compressed file contains "Time" shortcut file and "GSEA_time" folder. Clicking on "Time" shortcut opens the index file providing access to analysis files contained in the "GSEA_time" folder. [file 1476-4598-8-75-S2.zip › GSEA_time/PENG_LEUCINE_DN.html]

Details for gene set PENG\_LEUCINE\_DN[GSEA]

|  || Dataset | gsea\_time\_collapsed |
| Phenotype | NoPhenotypeAvailable |
| Upregulated in class | na\_neg |
| GeneSet | PENG\_LEUCINE\_DN |
| Enrichment Score (ES) | -0.28827995 |
| Normalized Enrichment Score (NES) | -1.3733014 |
| Nominal p-value | 0.0 |
| FDR q-value | 0.26603016 |
| FWER p-Value | 1.0 |
Table: GSEA Results Summary

  

Fig 1: Enrichment plot: PENG\_LEUCINE\_DN      
 Profile of the Running ES Score & Positions of GeneSet Members on the Rank Ordered List

  

| PROBE | GENE SYMBOL | GENE\_TITLE | RANK IN GENE LIST | RANK METRIC SCORE | RUNNING ES | CORE ENRICHMENT || 1 | LDHB |  |  | 1 | 2.284 | 0.0803 | No |
| 2 | GART |  |  | 83 | 0.946 | 0.1096 | No |
| 3 | PGK1 |  |  | 101 | 0.899 | 0.1404 | No |
| 4 | CALR |  |  | 461 | 0.550 | 0.1423 | No |
| 5 | EMP3 |  |  | 708 | 0.473 | 0.1469 | No |
| 6 | SNRPA1 |  |  | 874 | 0.428 | 0.1539 | No |
| 7 | CCNA2 |  |  | 1525 | 0.326 | 0.1336 | No |
| 8 | ERH |  |  | 1884 | 0.293 | 0.1264 | No |
| 9 | PSMF1 |  |  | 2075 | 0.278 | 0.1269 | No |
| 10 | SQLE |  |  | 2134 | 0.274 | 0.1337 | No |
| 11 | AMD1 |  |  | 2150 | 0.273 | 0.1426 | No |
| 12 | CCT5 |  |  | 2167 | 0.271 | 0.1513 | No |
| 13 | ALG8 |  |  | 2190 | 0.270 | 0.1598 | No |
| 14 | NBL1 |  |  | 2224 | 0.267 | 0.1675 | No |
| 15 | SLC29A1 |  |  | 2315 | 0.261 | 0.1723 | No |
| 16 | PSMB7 |  |  | 2319 | 0.260 | 0.1813 | No |
| 17 | PFKM |  |  | 2412 | 0.254 | 0.1858 | No |
| 18 | PDCD4 |  |  | 2443 | 0.252 | 0.1931 | No |
| 19 | TK1 |  |  | 2459 | 0.250 | 0.2012 | No |
| 20 | MRPL12 |  |  | 2462 | 0.250 | 0.2099 | No |
| 21 | GSPT1 |  |  | 2606 | 0.241 | 0.2114 | No |
| 22 | TCP1 |  |  | 2874 | 0.226 | 0.2063 | No |
| 23 | ME2 |  |  | 2889 | 0.225 | 0.2136 | No |
| 24 | VRK1 |  |  | 2931 | 0.223 | 0.2194 | No |
| 25 | UBE2N |  |  | 2954 | 0.222 | 0.2261 | No |
| 26 | DYRK1A |  |  | 2967 | 0.221 | 0.2333 | No |
| 27 | MRPS12 |  |  | 3026 | 0.218 | 0.2381 | No |
| 28 | PAPOLA |  |  | 3124 | 0.213 | 0.2409 | No |
| 29 | SSB |  |  | 3200 | 0.209 | 0.2446 | No |
| 30 | XPO6 |  |  | 3230 | 0.207 | 0.2504 | No |
| 31 | TDG |  |  | 3462 | 0.196 | 0.2460 | No |
| 32 | NUTF2 |  |  | 3530 | 0.192 | 0.2495 | No |
| 33 | GLO1 |  |  | 3790 | 0.183 | 0.2433 | No |
| 34 | MTHFD1 |  |  | 3840 | 0.181 | 0.2473 | No |
| 35 | WDR1 |  |  | 3852 | 0.180 | 0.2531 | No |
| 36 | CTSC |  |  | 4134 | 0.167 | 0.2453 | No |
| 37 | TOMM40 |  |  | 4173 | 0.166 | 0.2492 | No |
| 38 | CSE1L |  |  | 4195 | 0.165 | 0.2540 | No |
| 39 | TIMM17A |  |  | 4221 | 0.164 | 0.2586 | No |
| 40 | NOL5A |  |  | 4448 | 0.156 | 0.2530 | No |
| 41 | BRD2 |  |  | 4522 | 0.153 | 0.2549 | No |
| 42 | EIF5 |  |  | 4629 | 0.150 | 0.2549 | No |
| 43 | NCL |  |  | 4653 | 0.149 | 0.2590 | No |
| 44 | TOP1 |  |  | 4714 | 0.147 | 0.2613 | No |
| 45 | RNF126 |  |  | 4842 | 0.142 | 0.2601 | No |
| 46 | NUP98 |  |  | 5012 | 0.137 | 0.2566 | No |
| 47 | PSMD1 |  |  | 5155 | 0.133 | 0.2544 | No |
| 48 | TFRC |  |  | 5832 | 0.116 | 0.2255 | No |
| 49 | ILF3 |  |  | 5891 | 0.115 | 0.2267 | No |
| 50 | IDI1 |  |  | 6013 | 0.113 | 0.2247 | No |
| 51 | PSME4 |  |  | 6649 | 0.098 | 0.1972 | No |
| 52 | PTBP1 |  |  | 6672 | 0.098 | 0.1995 | No |
| 53 | LIMK2 |  |  | 6739 | 0.097 | 0.1997 | No |
| 54 | XRCC5 |  |  | 7454 | 0.083 | 0.1677 | No |
| 55 | PIK3R3 |  |  | 7480 | 0.082 | 0.1694 | No |
| 56 | TRIP13 |  |  | 7790 | 0.076 | 0.1570 | No |
| 57 | HPRT1 |  |  | 7814 | 0.076 | 0.1585 | No |
| 58 | DDX21 |  |  | 8438 | 0.066 | 0.1304 | No |
| 59 | LDHA |  |  | 8499 | 0.064 | 0.1297 | No |
| 60 | POLRMT |  |  | 8655 | 0.062 | 0.1244 | No |
| 61 | GGA2 |  |  | 8745 | 0.061 | 0.1222 | No |
| 62 | TCEB1 |  |  | 9050 | 0.056 | 0.1093 | No |
| 63 | CYCS |  |  | 9385 | 0.051 | 0.0948 | No |
| 64 | EIF4G2 |  |  | 9737 | 0.046 | 0.0792 | No |
| 65 | PTMS |  |  | 10103 | 0.040 | 0.0628 | No |
| 66 | PSMA5 |  |  | 10237 | 0.038 | 0.0577 | No |
| 67 | DHX9 |  |  | 10330 | 0.037 | 0.0545 | No |
| 68 | EI24 |  |  | 10349 | 0.037 | 0.0549 | No |
| 69 | EMD |  |  | 10489 | 0.035 | 0.0493 | No |
| 70 | NOL1 |  |  | 10955 | 0.028 | 0.0276 | No |
| 71 | HSPC111 |  |  | 11074 | 0.026 | 0.0228 | No |
| 72 | NMT1 |  |  | 11087 | 0.026 | 0.0231 | No |
| 73 | UBC |  |  | 11289 | 0.024 | 0.0141 | No |
| 74 | POLG |  |  | 11347 | 0.023 | 0.0122 | No |
| 75 | NP |  |  | 11569 | 0.020 | 0.0020 | No |
| 76 | CCNB1 |  |  | 12244 | 0.011 | -0.0305 | No |
| 77 | ETF1 |  |  | 12780 | 0.002 | -0.0566 | No |
| 78 | TUBA1 |  |  | 13103 | -0.003 | -0.0722 | No |
| 79 | SFRS2 |  |  | 13287 | -0.005 | -0.0810 | No |
| 80 | SEC61B |  |  | 13930 | -0.015 | -0.1118 | No |
| 81 | MCM4 |  |  | 14787 | -0.028 | -0.1526 | No |
| 82 | PSMA7 |  |  | 15031 | -0.032 | -0.1634 | No |
| 83 | TGFB1 |  |  | 15318 | -0.037 | -0.1761 | No |
| 84 | EIF4G1 |  |  | 15437 | -0.038 | -0.1805 | No |
| 85 | CCT6A |  |  | 15444 | -0.038 | -0.1794 | No |
| 86 | UMPS |  |  | 15453 | -0.039 | -0.1784 | No |
| 87 | CAD |  |  | 15550 | -0.040 | -0.1817 | No |
| 88 | HBD |  |  | 15644 | -0.042 | -0.1848 | No |
| 89 | MLF2 |  |  | 15937 | -0.047 | -0.1974 | No |
| 90 | PSMD11 |  |  | 16612 | -0.062 | -0.2282 | No |
| 91 | KIAA0179 |  |  | 16866 | -0.067 | -0.2382 | No |
| 92 | SPINT2 |  |  | 16995 | -0.070 | -0.2420 | No |
| 93 | KIAA0152 |  |  | 17172 | -0.075 | -0.2479 | No |
| 94 | EBP |  |  | 17473 | -0.083 | -0.2597 | No |
| 95 | BST2 |  |  | 17902 | -0.096 | -0.2772 | No |
| 96 | COPB2 |  |  | 17939 | -0.097 | -0.2756 | No |
| 97 | EIF2B4 |  |  | 17952 | -0.097 | -0.2727 | No |
| 98 | DNAJA1 |  |  | 18123 | -0.103 | -0.2774 | No |
| 99 | ALDOA |  |  | 18247 | -0.108 | -0.2796 | No |
| 100 | UNG |  |  | 18333 | -0.111 | -0.2799 | No |
| 101 | CDC20 |  |  | 18350 | -0.111 | -0.2767 | No |
| 102 | BYSL |  |  | 18494 | -0.117 | -0.2796 | No |
| 103 | VDAC1 |  |  | 18505 | -0.118 | -0.2759 | No |
| 104 | HSPA5 |  |  | 18613 | -0.122 | -0.2769 | No |
| 105 | MRPL3 |  |  | 18730 | -0.127 | -0.2781 | No |
| 106 | HSPA8 |  |  | 18824 | -0.132 | -0.2780 | No |
| 107 | TNFRSF1A |  |  | 19036 | -0.143 | -0.2833 | Yes |
| 108 | DNAJB6 |  |  | 19071 | -0.145 | -0.2798 | Yes |
| 109 | HSPE1 |  |  | 19133 | -0.149 | -0.2776 | Yes |
| 110 | OGFR |  |  | 19153 | -0.150 | -0.2732 | Yes |
| 111 | FKBP2 |  |  | 19226 | -0.155 | -0.2713 | Yes |
| 112 | DHRS1 |  |  | 19252 | -0.156 | -0.2670 | Yes |
| 113 | FDPS |  |  | 19298 | -0.160 | -0.2636 | Yes |
| 114 | FKBP4 |  |  | 19302 | -0.161 | -0.2581 | Yes |
| 115 | MMP15 |  |  | 19560 | -0.185 | -0.2641 | Yes |
| 116 | PSMD14 |  |  | 19634 | -0.192 | -0.2609 | Yes |
| 117 | DHCR24 |  |  | 19744 | -0.205 | -0.2590 | Yes |
| 118 | PPIB |  |  | 19777 | -0.209 | -0.2533 | Yes |
| 119 | CCT2 |  |  | 19842 | -0.217 | -0.2487 | Yes |
| 120 | DDX1 |  |  | 19881 | -0.223 | -0.2428 | Yes |
| 121 | PSMD2 |  |  | 19976 | -0.237 | -0.2390 | Yes |
| 122 | GCDH |  |  | 19987 | -0.239 | -0.2311 | Yes |
| 123 | MRPL28 |  |  | 20028 | -0.248 | -0.2244 | Yes |
| 124 | SF3B4 |  |  | 20194 | -0.294 | -0.2221 | Yes |
| 125 | CCT4 |  |  | 20205 | -0.297 | -0.2121 | Yes |
| 126 | NXF1 |  |  | 20221 | -0.302 | -0.2022 | Yes |
| 127 | PTPN6 |  |  | 20233 | -0.307 | -0.1919 | Yes |
| 128 | AHSA1 |  |  | 20270 | -0.320 | -0.1824 | Yes |
| 129 | HSPA4 |  |  | 20344 | -0.351 | -0.1737 | Yes |
| 130 | PSMD3 |  |  | 20351 | -0.354 | -0.1615 | Yes |
| 131 | RBM14 |  |  | 20418 | -0.400 | -0.1506 | Yes |
| 132 | SCD |  |  | 20439 | -0.416 | -0.1370 | Yes |
| 133 | SRM |  |  | 20508 | -0.487 | -0.1232 | Yes |
| 134 | ACAT2 |  |  | 20518 | -0.499 | -0.1060 | Yes |
| 135 | CYC1 |  |  | 20538 | -0.524 | -0.0885 | Yes |
| 136 | ARMET |  |  | 20551 | -0.552 | -0.0697 | Yes |
| 137 | ADRM1 |  |  | 20562 | -0.582 | -0.0497 | Yes |
| 138 | RASSF2 |  |  | 20580 | -0.655 | -0.0275 | Yes |
| 139 | FASN |  |  | 20596 | -0.815 | 0.0004 | Yes |
Table: GSEA details [plain text format]

  

Fig 2: PENG\_LEUCINE\_DN: Random ES distribution      
 Gene set null distribution of ES for **PENG\_LEUCINE\_DN**
