# Supplementary material for: The rapamycin-regulated gene expression signature determines prognosis for breast cancer
Source: Mol Cancer. 2009 Sep 24;8:75. doi: 10.1186/1476-4598-8-75 (PMC2761377; doi:10.1186/1476-4598-8-75)
Supplement: Additional file 2 — Gene set enrichment analysis of in vivo data, time series. The data provided represent the time series of GSEA. This compressed file contains "Time" shortcut file and "GSEA_time" folder. Clicking on "Time" shortcut opens the index file providing access to analysis files contained in the "GSEA_time" folder. [file 1476-4598-8-75-S2.zip › GSEA_time/POD1_KO_MOST_DN.html]

Details for gene set POD1\_KO\_MOST\_DN[GSEA]

|  || Dataset | gsea\_time\_collapsed |
| Phenotype | NoPhenotypeAvailable |
| Upregulated in class | na\_pos |
| GeneSet | POD1\_KO\_MOST\_DN |
| Enrichment Score (ES) | 0.69808286 |
| Normalized Enrichment Score (NES) | 1.7359935 |
| Nominal p-value | 0.0 |
| FDR q-value | 0.0140221575 |
| FWER p-Value | 0.483 |
Table: GSEA Results Summary

  

Fig 1: Enrichment plot: POD1\_KO\_MOST\_DN      
 Profile of the Running ES Score & Positions of GeneSet Members on the Rank Ordered List

  

| PROBE | GENE SYMBOL | GENE\_TITLE | RANK IN GENE LIST | RANK METRIC SCORE | RUNNING ES | CORE ENRICHMENT || 1 | SLC12A2 |  |  | 85 | 0.941 | 0.1694 | Yes |
| 2 | LRRFIP1 |  |  | 365 | 0.600 | 0.2665 | Yes |
| 3 | PTGER4 |  |  | 453 | 0.552 | 0.3642 | Yes |
| 4 | HSPA1B |  |  | 777 | 0.454 | 0.4324 | Yes |
| 5 | ADM |  |  | 932 | 0.417 | 0.5019 | Yes |
| 6 | ART3 |  |  | 996 | 0.406 | 0.5737 | Yes |
| 7 | TSPAN2 |  |  | 1441 | 0.336 | 0.6141 | Yes |
| 8 | MAPT |  |  | 1664 | 0.312 | 0.6608 | Yes |
| 9 | COL4A3 |  |  | 1981 | 0.285 | 0.6981 | Yes |
| 10 | RHPN1 |  |  | 4092 | 0.170 | 0.6269 | No |
| 11 | TOP1MT |  |  | 5688 | 0.120 | 0.5715 | No |
| 12 | CLIC5 |  |  | 7183 | 0.088 | 0.5151 | No |
| 13 | SH3BGRL2 |  |  | 7254 | 0.087 | 0.5277 | No |
| 14 | SEMA3E |  |  | 8456 | 0.065 | 0.4814 | No |
| 15 | PDE10A |  |  | 9912 | 0.043 | 0.4187 | No |
| 16 | UMOD |  |  | 10891 | 0.029 | 0.3765 | No |
| 17 | GALC |  |  | 11252 | 0.024 | 0.3635 | No |
| 18 | NPHS2 |  |  | 11689 | 0.018 | 0.3456 | No |
| 19 | GABRA4 |  |  | 11735 | 0.017 | 0.3466 | No |
| 20 | SGIP1 |  |  | 12311 | 0.010 | 0.3204 | No |
| 21 | TLR4 |  |  | 13724 | -0.012 | 0.2539 | No |
| 22 | EGF |  |  | 15076 | -0.033 | 0.1944 | No |
| 23 | HIST1H3G |  |  | 17174 | -0.075 | 0.1063 | No |
| 24 | MERTK |  |  | 18330 | -0.111 | 0.0706 | No |
| 25 | PARP4 |  |  | 19833 | -0.216 | 0.0375 | No |
Table: GSEA details [plain text format]

  

Fig 2: POD1\_KO\_MOST\_DN: Random ES distribution      
 Gene set null distribution of ES for **POD1\_KO\_MOST\_DN**
