# Supplementary material for: The rapamycin-regulated gene expression signature determines prognosis for breast cancer
Source: Mol Cancer. 2009 Sep 24;8:75. doi: 10.1186/1476-4598-8-75 (PMC2761377; doi:10.1186/1476-4598-8-75)
Supplement: Additional file 2 — Gene set enrichment analysis of in vivo data, time series. The data provided represent the time series of GSEA. This compressed file contains "Time" shortcut file and "GSEA_time" folder. Clicking on "Time" shortcut opens the index file providing access to analysis files contained in the "GSEA_time" folder. [file 1476-4598-8-75-S2.zip › GSEA_time/POD1_KO_MOST_UP.html]

Details for gene set POD1\_KO\_MOST\_UP[GSEA]

|  || Dataset | gsea\_time\_collapsed |
| Phenotype | NoPhenotypeAvailable |
| Upregulated in class | na\_neg |
| GeneSet | POD1\_KO\_MOST\_UP |
| Enrichment Score (ES) | -0.41685197 |
| Normalized Enrichment Score (NES) | -1.4916968 |
| Nominal p-value | 0.023076924 |
| FDR q-value | 0.23410487 |
| FWER p-Value | 0.989 |
Table: GSEA Results Summary

  

Fig 1: Enrichment plot: POD1\_KO\_MOST\_UP      
 Profile of the Running ES Score & Positions of GeneSet Members on the Rank Ordered List

  

| PROBE | GENE SYMBOL | GENE\_TITLE | RANK IN GENE LIST | RANK METRIC SCORE | RUNNING ES | CORE ENRICHMENT || 1 | NCAM1 |  |  | 1358 | 0.346 | 0.0023 | No |
| 2 | CDKN1B |  |  | 1916 | 0.291 | 0.0327 | No |
| 3 | HMMR |  |  | 2085 | 0.277 | 0.0793 | No |
| 4 | LHFPL2 |  |  | 2643 | 0.239 | 0.0996 | No |
| 5 | SERPINF1 |  |  | 3273 | 0.205 | 0.1096 | No |
| 6 | DKFZP762E1312 |  |  | 3711 | 0.187 | 0.1252 | No |
| 7 | CSPG2 |  |  | 4202 | 0.164 | 0.1339 | No |
| 8 | LBP |  |  | 4333 | 0.160 | 0.1591 | No |
| 9 | COL6A1 |  |  | 5412 | 0.126 | 0.1316 | No |
| 10 | SOX11 |  |  | 6343 | 0.105 | 0.1071 | No |
| 11 | COL6A2 |  |  | 8122 | 0.071 | 0.0346 | No |
| 12 | CCND2 |  |  | 8369 | 0.067 | 0.0359 | No |
| 13 | DCN |  |  | 8493 | 0.065 | 0.0427 | No |
| 14 | PTN |  |  | 8684 | 0.062 | 0.0457 | No |
| 15 | IGSF4 |  |  | 8780 | 0.061 | 0.0531 | No |
| 16 | FAP |  |  | 9123 | 0.055 | 0.0473 | No |
| 17 | NOPE |  |  | 10355 | 0.037 | -0.0053 | No |
| 18 | PENK |  |  | 10538 | 0.034 | -0.0074 | No |
| 19 | EPHA4 |  |  | 11620 | 0.019 | -0.0562 | No |
| 20 | RBP1 |  |  | 11919 | 0.015 | -0.0677 | No |
| 21 | CFH |  |  | 12230 | 0.011 | -0.0807 | No |
| 22 | STARD10 |  |  | 13327 | -0.006 | -0.1327 | No |
| 23 | HOMER2 |  |  | 13898 | -0.014 | -0.1576 | No |
| 24 | COL3A1 |  |  | 14444 | -0.023 | -0.1796 | No |
| 25 | CDH6 |  |  | 14878 | -0.030 | -0.1948 | No |
| 26 | CLDN6 |  |  | 15176 | -0.035 | -0.2024 | No |
| 27 | CYP7B1 |  |  | 15274 | -0.036 | -0.1999 | No |
| 28 | COL8A2 |  |  | 16663 | -0.063 | -0.2551 | No |
| 29 | LUM |  |  | 17751 | -0.091 | -0.2899 | No |
| 30 | PHGDH |  |  | 20363 | -0.363 | -0.3452 | Yes |
| 31 | MDK |  |  | 20591 | -0.741 | -0.2099 | Yes |
| 32 | IGFBP2 |  |  | 20605 | -1.066 | -0.0000 | Yes |
Table: GSEA details [plain text format]

  

Fig 2: POD1\_KO\_MOST\_UP: Random ES distribution      
 Gene set null distribution of ES for **POD1\_KO\_MOST\_UP**
