# Supplementary material for: The rapamycin-regulated gene expression signature determines prognosis for breast cancer
Source: Mol Cancer. 2009 Sep 24;8:75. doi: 10.1186/1476-4598-8-75 (PMC2761377; doi:10.1186/1476-4598-8-75)
Supplement: Additional file 2 — Gene set enrichment analysis of in vivo data, time series. The data provided represent the time series of GSEA. This compressed file contains "Time" shortcut file and "GSEA_time" folder. Clicking on "Time" shortcut opens the index file providing access to analysis files contained in the "GSEA_time" folder. [file 1476-4598-8-75-S2.zip › GSEA_time/PROTEASOME_DEGRADATION.html]

Details for gene set PROTEASOME\_DEGRADATION[GSEA]

|  || Dataset | gsea\_time\_collapsed |
| Phenotype | NoPhenotypeAvailable |
| Upregulated in class | na\_neg |
| GeneSet | PROTEASOME\_DEGRADATION |
| Enrichment Score (ES) | -0.41681567 |
| Normalized Enrichment Score (NES) | -1.4566813 |
| Nominal p-value | 0.036036037 |
| FDR q-value | 0.25630018 |
| FWER p-Value | 0.995 |
Table: GSEA Results Summary

  

Fig 1: Enrichment plot: PROTEASOME\_DEGRADATION      
 Profile of the Running ES Score & Positions of GeneSet Members on the Rank Ordered List

  

| PROBE | GENE SYMBOL | GENE\_TITLE | RANK IN GENE LIST | RANK METRIC SCORE | RUNNING ES | CORE ENRICHMENT || 1 | UBE2D3 |  |  | 1103 | 0.386 | 0.0244 | No |
| 2 | PSMB7 |  |  | 2319 | 0.260 | 0.0178 | No |
| 3 | PSMA1 |  |  | 2524 | 0.246 | 0.0576 | No |
| 4 | PSMD4 |  |  | 3335 | 0.202 | 0.0591 | No |
| 5 | MKI67 |  |  | 4043 | 0.172 | 0.0594 | No |
| 6 | UBE2D2 |  |  | 4263 | 0.163 | 0.0816 | No |
| 7 | PSMD1 |  |  | 5155 | 0.133 | 0.0652 | No |
| 8 | UBA52 |  |  | 5526 | 0.123 | 0.0721 | No |
| 9 | H2AFX |  |  | 6183 | 0.109 | 0.0621 | No |
| 10 | UBE2D1 |  |  | 6315 | 0.105 | 0.0770 | No |
| 11 | UBE1L |  |  | 7269 | 0.086 | 0.0481 | No |
| 12 | PSMB9 |  |  | 9544 | 0.049 | -0.0525 | No |
| 13 | PSMD5 |  |  | 9584 | 0.048 | -0.0447 | No |
| 14 | UBC |  |  | 11289 | 0.024 | -0.1228 | No |
| 15 | PSME2 |  |  | 11951 | 0.014 | -0.1520 | No |
| 16 | IFNG |  |  | 13670 | -0.011 | -0.2332 | No |
| 17 | PSMA4 |  |  | 14050 | -0.017 | -0.2483 | No |
| 18 | PSMA7 |  |  | 15031 | -0.032 | -0.2894 | No |
| 19 | PSMA6 |  |  | 15054 | -0.033 | -0.2839 | No |
| 20 | H2AFZ |  |  | 16941 | -0.068 | -0.3617 | No |
| 21 | PSMC2 |  |  | 17461 | -0.083 | -0.3703 | No |
| 22 | RPS27A |  |  | 18420 | -0.114 | -0.3938 | Yes |
| 23 | PSMC5 |  |  | 18440 | -0.115 | -0.3715 | Yes |
| 24 | PSMC1 |  |  | 18470 | -0.116 | -0.3494 | Yes |
| 25 | UBE1 |  |  | 18507 | -0.118 | -0.3274 | Yes |
| 26 | PSMD2 |  |  | 19976 | -0.237 | -0.3510 | Yes |
| 27 | PSMD8 |  |  | 19999 | -0.241 | -0.3034 | Yes |
| 28 | PSMD3 |  |  | 20351 | -0.354 | -0.2489 | Yes |
| 29 | PSMC6 |  |  | 20385 | -0.375 | -0.1749 | Yes |
| 30 | PSMC3 |  |  | 20400 | -0.383 | -0.0983 | Yes |
| 31 | PSMB6 |  |  | 20548 | -0.536 | 0.0028 | Yes |
Table: GSEA details [plain text format]

  

Fig 2: PROTEASOME\_DEGRADATION: Random ES distribution      
 Gene set null distribution of ES for **PROTEASOME\_DEGRADATION**
