# Supplementary material for: The rapamycin-regulated gene expression signature determines prognosis for breast cancer
Source: Mol Cancer. 2009 Sep 24;8:75. doi: 10.1186/1476-4598-8-75 (PMC2761377; doi:10.1186/1476-4598-8-75)
Supplement: Additional file 2 — Gene set enrichment analysis of in vivo data, time series. The data provided represent the time series of GSEA. This compressed file contains "Time" shortcut file and "GSEA_time" folder. Clicking on "Time" shortcut opens the index file providing access to analysis files contained in the "GSEA_time" folder. [file 1476-4598-8-75-S2.zip › GSEA_time/RADAEVA_IFNA_UP.html]

Details for gene set RADAEVA\_IFNA\_UP[GSEA]

|  || Dataset | gsea\_time\_collapsed |
| Phenotype | NoPhenotypeAvailable |
| Upregulated in class | na\_neg |
| GeneSet | RADAEVA\_IFNA\_UP |
| Enrichment Score (ES) | -0.33254912 |
| Normalized Enrichment Score (NES) | -1.2992606 |
| Nominal p-value | 0.03846154 |
| FDR q-value | 0.31822297 |
| FWER p-Value | 1.0 |
Table: GSEA Results Summary

  

Fig 1: Enrichment plot: RADAEVA\_IFNA\_UP      
 Profile of the Running ES Score & Positions of GeneSet Members on the Rank Ordered List

  

| PROBE | GENE SYMBOL | GENE\_TITLE | RANK IN GENE LIST | RANK METRIC SCORE | RUNNING ES | CORE ENRICHMENT || 1 | IFITM1 |  |  | 275 | 0.669 | 0.0608 | No |
| 2 | CALR |  |  | 461 | 0.550 | 0.1129 | No |
| 3 | CXCL11 |  |  | 499 | 0.535 | 0.1704 | No |
| 4 | UBE2D3 |  |  | 1103 | 0.386 | 0.1839 | No |
| 5 | AKR7A2 |  |  | 1535 | 0.325 | 0.1990 | No |
| 6 | PLSCR1 |  |  | 1622 | 0.315 | 0.2298 | No |
| 7 | CDKN1B |  |  | 1916 | 0.291 | 0.2478 | No |
| 8 | EIF2AK2 |  |  | 3540 | 0.192 | 0.1901 | No |
| 9 | STAT1 |  |  | 4141 | 0.167 | 0.1795 | No |
| 10 | CASP3 |  |  | 4494 | 0.155 | 0.1796 | No |
| 11 | E2F2 |  |  | 4728 | 0.146 | 0.1845 | No |
| 12 | SOCS1 |  |  | 5204 | 0.132 | 0.1760 | No |
| 13 | BCL2 |  |  | 5387 | 0.127 | 0.1812 | No |
| 14 | IFIT2 |  |  | 5508 | 0.124 | 0.1891 | No |
| 15 | IFITM2 |  |  | 5678 | 0.120 | 0.1942 | No |
| 16 | RSAD2 |  |  | 9879 | 0.044 | -0.0053 | No |
| 17 | KIAA0409 |  |  | 10407 | 0.036 | -0.0269 | No |
| 18 | ADAR |  |  | 12811 | 0.001 | -0.1437 | No |
| 19 | IRF1 |  |  | 12849 | 0.001 | -0.1454 | No |
| 20 | KLK3 |  |  | 13193 | -0.004 | -0.1616 | No |
| 21 | PSME1 |  |  | 14347 | -0.021 | -0.2154 | No |
| 22 | TNFSF10 |  |  | 14451 | -0.023 | -0.2179 | No |
| 23 | VCAM1 |  |  | 15109 | -0.034 | -0.2461 | No |
| 24 | IFI35 |  |  | 15152 | -0.034 | -0.2444 | No |
| 25 | NTRK1 |  |  | 15360 | -0.038 | -0.2503 | No |
| 26 | IFITM3 |  |  | 15704 | -0.043 | -0.2622 | No |
| 27 | MXD4 |  |  | 15829 | -0.045 | -0.2632 | No |
| 28 | IFI44 |  |  | 16912 | -0.068 | -0.3083 | No |
| 29 | PHB |  |  | 17102 | -0.073 | -0.3094 | No |
| 30 | CACNA2D2 |  |  | 17527 | -0.085 | -0.3207 | No |
| 31 | CXCL10 |  |  | 17747 | -0.091 | -0.3212 | Yes |
| 32 | MX2 |  |  | 17855 | -0.094 | -0.3160 | Yes |
| 33 | BST2 |  |  | 17902 | -0.096 | -0.3076 | Yes |
| 34 | IL12RB2 |  |  | 18100 | -0.102 | -0.3059 | Yes |
| 35 | RFC2 |  |  | 18307 | -0.110 | -0.3037 | Yes |
| 36 | BAX |  |  | 18361 | -0.112 | -0.2939 | Yes |
| 37 | PIAS4 |  |  | 19157 | -0.151 | -0.3158 | Yes |
| 38 | HLA-E |  |  | 19158 | -0.151 | -0.2991 | Yes |
| 39 | VAT1 |  |  | 19351 | -0.164 | -0.2902 | Yes |
| 40 | IFI44L |  |  | 19458 | -0.175 | -0.2760 | Yes |
| 41 | IRF7 |  |  | 19564 | -0.185 | -0.2605 | Yes |
| 42 | UBE2L6 |  |  | 19740 | -0.204 | -0.2464 | Yes |
| 43 | IFI6 |  |  | 19848 | -0.218 | -0.2274 | Yes |
| 44 | IFI27 |  |  | 19905 | -0.226 | -0.2050 | Yes |
| 45 | TUBB |  |  | 20162 | -0.282 | -0.1861 | Yes |
| 46 | ISG15 |  |  | 20207 | -0.298 | -0.1552 | Yes |
| 47 | MX1 |  |  | 20243 | -0.310 | -0.1225 | Yes |
| 48 | HLA-B |  |  | 20361 | -0.362 | -0.0881 | Yes |
| 49 | OAS1 |  |  | 20370 | -0.365 | -0.0479 | Yes |
| 50 | LGALS3BP |  |  | 20546 | -0.534 | 0.0029 | Yes |
Table: GSEA details [plain text format]

  

Fig 2: RADAEVA\_IFNA\_UP: Random ES distribution      
 Gene set null distribution of ES for **RADAEVA\_IFNA\_UP**
