# Supplementary material for: The rapamycin-regulated gene expression signature determines prognosis for breast cancer
Source: Mol Cancer. 2009 Sep 24;8:75. doi: 10.1186/1476-4598-8-75 (PMC2761377; doi:10.1186/1476-4598-8-75)
Supplement: Additional file 2 — Gene set enrichment analysis of in vivo data, time series. The data provided represent the time series of GSEA. This compressed file contains "Time" shortcut file and "GSEA_time" folder. Clicking on "Time" shortcut opens the index file providing access to analysis files contained in the "GSEA_time" folder. [file 1476-4598-8-75-S2.zip › GSEA_time/ROS_MOUSE_AORTA_DN.html]

Details for gene set ROS\_MOUSE\_AORTA\_DN[GSEA]

|  || Dataset | gsea\_time\_collapsed |
| Phenotype | NoPhenotypeAvailable |
| Upregulated in class | na\_pos |
| GeneSet | ROS\_MOUSE\_AORTA\_DN |
| Enrichment Score (ES) | 0.6146004 |
| Normalized Enrichment Score (NES) | 1.7540138 |
| Nominal p-value | 0.0 |
| FDR q-value | 0.011223552 |
| FWER p-Value | 0.372 |
Table: GSEA Results Summary

  

Fig 1: Enrichment plot: ROS\_MOUSE\_AORTA\_DN      
 Profile of the Running ES Score & Positions of GeneSet Members on the Rank Ordered List

  

| PROBE | GENE SYMBOL | GENE\_TITLE | RANK IN GENE LIST | RANK METRIC SCORE | RUNNING ES | CORE ENRICHMENT || 1 | SAT1 |  |  | 58 | 1.024 | 0.0496 | Yes |
| 2 | RGS2 |  |  | 70 | 0.989 | 0.0997 | Yes |
| 3 | SERPINA3 |  |  | 116 | 0.874 | 0.1422 | Yes |
| 4 | CXCL3 |  |  | 162 | 0.784 | 0.1802 | Yes |
| 5 | PMP22 |  |  | 191 | 0.740 | 0.2167 | Yes |
| 6 | LIMS1 |  |  | 261 | 0.677 | 0.2480 | Yes |
| 7 | PKP4 |  |  | 262 | 0.677 | 0.2826 | Yes |
| 8 | PTGIS |  |  | 273 | 0.669 | 0.3164 | Yes |
| 9 | NFKBIZ |  |  | 313 | 0.639 | 0.3472 | Yes |
| 10 | MGP |  |  | 412 | 0.574 | 0.3718 | Yes |
| 11 | EMP3 |  |  | 708 | 0.473 | 0.3817 | Yes |
| 12 | DSTN |  |  | 840 | 0.436 | 0.3976 | Yes |
| 13 | TPM1 |  |  | 870 | 0.428 | 0.4181 | Yes |
| 14 | HIF1A |  |  | 889 | 0.425 | 0.4390 | Yes |
| 15 | CTSS |  |  | 960 | 0.413 | 0.4567 | Yes |
| 16 | IL10RB |  |  | 1028 | 0.401 | 0.4739 | Yes |
| 17 | CD14 |  |  | 1153 | 0.378 | 0.4872 | Yes |
| 18 | PHLDB2 |  |  | 1193 | 0.372 | 0.5044 | Yes |
| 19 | AADACL1 |  |  | 1482 | 0.331 | 0.5073 | Yes |
| 20 | ARG2 |  |  | 1498 | 0.329 | 0.5234 | Yes |
| 21 | MEF2A |  |  | 1602 | 0.318 | 0.5347 | Yes |
| 22 | ARL6IP2 |  |  | 1809 | 0.299 | 0.5400 | Yes |
| 23 | CGGBP1 |  |  | 2108 | 0.276 | 0.5396 | Yes |
| 24 | ENAH |  |  | 2261 | 0.264 | 0.5457 | Yes |
| 25 | ATF3 |  |  | 2567 | 0.244 | 0.5433 | Yes |
| 26 | LCP1 |  |  | 2589 | 0.242 | 0.5547 | Yes |
| 27 | TMEM59 |  |  | 2616 | 0.241 | 0.5657 | Yes |
| 28 | BGN |  |  | 2751 | 0.233 | 0.5711 | Yes |
| 29 | CPE |  |  | 2813 | 0.229 | 0.5799 | Yes |
| 30 | IGFBP5 |  |  | 2817 | 0.229 | 0.5915 | Yes |
| 31 | ACTG1 |  |  | 2865 | 0.227 | 0.6008 | Yes |
| 32 | GADD45B |  |  | 2916 | 0.224 | 0.6098 | Yes |
| 33 | CAPZB |  |  | 3046 | 0.217 | 0.6146 | Yes |
| 34 | RPL18 |  |  | 3626 | 0.189 | 0.5961 | No |
| 35 | ARG1 |  |  | 3699 | 0.187 | 0.6022 | No |
| 36 | OGN |  |  | 4446 | 0.156 | 0.5738 | No |
| 37 | IL11RA |  |  | 4769 | 0.145 | 0.5656 | No |
| 38 | CSRP1 |  |  | 4979 | 0.138 | 0.5624 | No |
| 39 | SFRP2 |  |  | 5182 | 0.132 | 0.5594 | No |
| 40 | S100A6 |  |  | 6056 | 0.112 | 0.5226 | No |
| 41 | BTG3 |  |  | 6287 | 0.106 | 0.5168 | No |
| 42 | NPNT |  |  | 7064 | 0.090 | 0.4836 | No |
| 43 | MYH11 |  |  | 7282 | 0.086 | 0.4774 | No |
| 44 | EXOSC7 |  |  | 8229 | 0.069 | 0.4348 | No |
| 45 | ELN |  |  | 8254 | 0.069 | 0.4372 | No |
| 46 | CD9 |  |  | 8510 | 0.064 | 0.4281 | No |
| 47 | CXCL6 |  |  | 9436 | 0.050 | 0.3856 | No |
| 48 | HDC |  |  | 9799 | 0.045 | 0.3703 | No |
| 49 | CLU |  |  | 9929 | 0.043 | 0.3662 | No |
| 50 | RPS5 |  |  | 11119 | 0.026 | 0.3096 | No |
| 51 | ANXA3 |  |  | 11767 | 0.017 | 0.2790 | No |
| 52 | RBP1 |  |  | 11919 | 0.015 | 0.2724 | No |
| 53 | BHLHB2 |  |  | 12438 | 0.008 | 0.2475 | No |
| 54 | SERPING1 |  |  | 12583 | 0.005 | 0.2408 | No |
| 55 | PON1 |  |  | 13271 | -0.005 | 0.2076 | No |
| 56 | ALAS2 |  |  | 13376 | -0.007 | 0.2029 | No |
| 57 | CRIP1 |  |  | 13665 | -0.011 | 0.1894 | No |
| 58 | CD34 |  |  | 13771 | -0.013 | 0.1849 | No |
| 59 | FXYD5 |  |  | 13826 | -0.013 | 0.1830 | No |
| 60 | SYN1 |  |  | 13894 | -0.014 | 0.1805 | No |
| 61 | IL1B |  |  | 13976 | -0.016 | 0.1773 | No |
| 62 | LYZ |  |  | 13985 | -0.016 | 0.1778 | No |
| 63 | MMP9 |  |  | 14832 | -0.029 | 0.1380 | No |
| 64 | CYBA |  |  | 15150 | -0.034 | 0.1243 | No |
| 65 | PTGES3 |  |  | 15739 | -0.044 | 0.0979 | No |
| 66 | GNAI2 |  |  | 17163 | -0.074 | 0.0324 | No |
| 67 | CD5L |  |  | 17189 | -0.075 | 0.0350 | No |
| 68 | ACTB |  |  | 18556 | -0.120 | -0.0253 | No |
| 69 | GPX3 |  |  | 18665 | -0.125 | -0.0242 | No |
| 70 | RCN2 |  |  | 18671 | -0.125 | -0.0181 | No |
| 71 | PTPN23 |  |  | 19286 | -0.159 | -0.0399 | No |
| 72 | ALDH1A1 |  |  | 19486 | -0.177 | -0.0405 | No |
| 73 | SEPX1 |  |  | 20062 | -0.255 | -0.0554 | No |
| 74 | SLC25A1 |  |  | 20394 | -0.380 | -0.0521 | No |
| 75 | IL1R2 |  |  | 20419 | -0.400 | -0.0328 | No |
| 76 | S100A9 |  |  | 20597 | -0.817 | 0.0004 | No |
Table: GSEA details [plain text format]

  

Fig 2: ROS\_MOUSE\_AORTA\_DN: Random ES distribution      
 Gene set null distribution of ES for **ROS\_MOUSE\_AORTA\_DN**
