# Supplementary material for: The rapamycin-regulated gene expression signature determines prognosis for breast cancer
Source: Mol Cancer. 2009 Sep 24;8:75. doi: 10.1186/1476-4598-8-75 (PMC2761377; doi:10.1186/1476-4598-8-75)
Supplement: Additional file 2 — Gene set enrichment analysis of in vivo data, time series. The data provided represent the time series of GSEA. This compressed file contains "Time" shortcut file and "GSEA_time" folder. Clicking on "Time" shortcut opens the index file providing access to analysis files contained in the "GSEA_time" folder. [file 1476-4598-8-75-S2.zip › GSEA_time/SHEPARD_NEG_REG_OF_CELL_PROLIFERATION.html]

Details for gene set SHEPARD\_NEG\_REG\_OF\_CELL\_PROLIFERATION[GSEA]

|  || Dataset | gsea\_time\_collapsed |
| Phenotype | NoPhenotypeAvailable |
| Upregulated in class | na\_pos |
| GeneSet | SHEPARD\_NEG\_REG\_OF\_CELL\_PROLIFERATION |
| Enrichment Score (ES) | 0.6091831 |
| Normalized Enrichment Score (NES) | 1.7726154 |
| Nominal p-value | 0.0 |
| FDR q-value | 0.009427761 |
| FWER p-Value | 0.286 |
Table: GSEA Results Summary

  

Fig 1: Enrichment plot: SHEPARD\_NEG\_REG\_OF\_CELL\_PROLIFERATION      
 Profile of the Running ES Score & Positions of GeneSet Members on the Rank Ordered List

  

| PROBE | GENE SYMBOL | GENE\_TITLE | RANK IN GENE LIST | RANK METRIC SCORE | RUNNING ES | CORE ENRICHMENT || 1 | IGFBP7 |  |  | 2 | 2.165 | 0.0914 | Yes |
| 2 | IL8 |  |  | 13 | 1.561 | 0.1569 | Yes |
| 3 | ADAMTS1 |  |  | 33 | 1.238 | 0.2083 | Yes |
| 4 | DLG5 |  |  | 112 | 0.881 | 0.2416 | Yes |
| 5 | RARRES1 |  |  | 113 | 0.880 | 0.2788 | Yes |
| 6 | MDM4 |  |  | 114 | 0.878 | 0.3159 | Yes |
| 7 | ETS1 |  |  | 130 | 0.846 | 0.3509 | Yes |
| 8 | TOB2 |  |  | 132 | 0.843 | 0.3865 | Yes |
| 9 | PMP22 |  |  | 191 | 0.740 | 0.4149 | Yes |
| 10 | GAS1 |  |  | 498 | 0.535 | 0.4226 | Yes |
| 11 | CDKN1C |  |  | 693 | 0.475 | 0.4332 | Yes |
| 12 | EMP3 |  |  | 708 | 0.473 | 0.4525 | Yes |
| 13 | CUL5 |  |  | 836 | 0.437 | 0.4648 | Yes |
| 14 | CDK10 |  |  | 962 | 0.412 | 0.4761 | Yes |
| 15 | CTBP1 |  |  | 1057 | 0.395 | 0.4882 | Yes |
| 16 | CDKN3 |  |  | 1274 | 0.358 | 0.4928 | Yes |
| 17 | TOB1 |  |  | 1542 | 0.324 | 0.4935 | Yes |
| 18 | PRKRIR |  |  | 1561 | 0.322 | 0.5062 | Yes |
| 19 | CDKN2C |  |  | 1578 | 0.320 | 0.5189 | Yes |
| 20 | MXI1 |  |  | 1838 | 0.297 | 0.5188 | Yes |
| 21 | CUL4A |  |  | 1869 | 0.295 | 0.5298 | Yes |
| 22 | CDKN1B |  |  | 1916 | 0.291 | 0.5399 | Yes |
| 23 | COL4A3 |  |  | 1981 | 0.285 | 0.5488 | Yes |
| 24 | ZRANB1 |  |  | 2238 | 0.266 | 0.5475 | Yes |
| 25 | PAWR |  |  | 2337 | 0.259 | 0.5537 | Yes |
| 26 | PRKRA |  |  | 2427 | 0.253 | 0.5601 | Yes |
| 27 | FABP6 |  |  | 2583 | 0.243 | 0.5628 | Yes |
| 28 | CHEK1 |  |  | 2641 | 0.239 | 0.5701 | Yes |
| 29 | CD164 |  |  | 2696 | 0.236 | 0.5774 | Yes |
| 30 | ING1 |  |  | 2767 | 0.232 | 0.5838 | Yes |
| 31 | CUL1 |  |  | 2956 | 0.222 | 0.5840 | Yes |
| 32 | NCK2 |  |  | 3081 | 0.215 | 0.5870 | Yes |
| 33 | CUL2 |  |  | 3195 | 0.209 | 0.5904 | Yes |
| 34 | FABP3 |  |  | 3330 | 0.203 | 0.5924 | Yes |
| 35 | APRIN |  |  | 3465 | 0.196 | 0.5941 | Yes |
| 36 | VHL |  |  | 3585 | 0.191 | 0.5964 | Yes |
| 37 | CDC7 |  |  | 3621 | 0.189 | 0.6027 | Yes |
| 38 | CTBP2 |  |  | 3652 | 0.189 | 0.6092 | Yes |
| 39 | MNT |  |  | 4412 | 0.157 | 0.5788 | No |
| 40 | IL6 |  |  | 4954 | 0.138 | 0.5583 | No |
| 41 | BTG1 |  |  | 4972 | 0.138 | 0.5633 | No |
| 42 | CNOT8 |  |  | 5147 | 0.134 | 0.5604 | No |
| 43 | BCL2 |  |  | 5387 | 0.127 | 0.5541 | No |
| 44 | CXCL1 |  |  | 5663 | 0.120 | 0.5458 | No |
| 45 | BTG3 |  |  | 6287 | 0.106 | 0.5199 | No |
| 46 | SSTR3 |  |  | 6331 | 0.105 | 0.5222 | No |
| 47 | IL1A |  |  | 6347 | 0.105 | 0.5259 | No |
| 48 | POU1F1 |  |  | 6379 | 0.104 | 0.5288 | No |
| 49 | TGFB1I1 |  |  | 6469 | 0.102 | 0.5288 | No |
| 50 | TP53I11 |  |  | 6780 | 0.095 | 0.5177 | No |
| 51 | GNRH1 |  |  | 6836 | 0.095 | 0.5190 | No |
| 52 | MUC12 |  |  | 6996 | 0.091 | 0.5151 | No |
| 53 | RBBP4 |  |  | 7022 | 0.091 | 0.5177 | No |
| 54 | CCL23 |  |  | 7073 | 0.090 | 0.5191 | No |
| 55 | ADRA1A |  |  | 7088 | 0.089 | 0.5222 | No |
| 56 | SSTR4 |  |  | 7326 | 0.085 | 0.5142 | No |
| 57 | NF2 |  |  | 7606 | 0.080 | 0.5040 | No |
| 58 | PCAF |  |  | 7876 | 0.075 | 0.4940 | No |
| 59 | KLF4 |  |  | 7984 | 0.073 | 0.4918 | No |
| 60 | CDKN2A |  |  | 8063 | 0.072 | 0.4911 | No |
| 61 | TNFRSF8 |  |  | 8674 | 0.062 | 0.4639 | No |
| 62 | SSTR1 |  |  | 8976 | 0.057 | 0.4517 | No |
| 63 | ADAMTS8 |  |  | 9138 | 0.055 | 0.4461 | No |
| 64 | CD33 |  |  | 9307 | 0.052 | 0.4401 | No |
| 65 | IGFBP6 |  |  | 9820 | 0.045 | 0.4171 | No |
| 66 | NF1 |  |  | 10017 | 0.042 | 0.4093 | No |
| 67 | FRK |  |  | 10169 | 0.039 | 0.4036 | No |
| 68 | CDKN2D |  |  | 10282 | 0.038 | 0.3997 | No |
| 69 | PTHLH |  |  | 10555 | 0.034 | 0.3879 | No |
| 70 | MDM2 |  |  | 10709 | 0.032 | 0.3818 | No |
| 71 | SSTR5 |  |  | 10874 | 0.029 | 0.3750 | No |
| 72 | UMOD |  |  | 10891 | 0.029 | 0.3754 | No |
| 73 | COL18A1 |  |  | 11082 | 0.026 | 0.3673 | No |
| 74 | RARRES3 |  |  | 11274 | 0.024 | 0.3590 | No |
| 75 | ATP8A2 |  |  | 11577 | 0.020 | 0.3451 | No |
| 76 | CGRRF1 |  |  | 12055 | 0.013 | 0.3224 | No |
| 77 | DLEC1 |  |  | 12210 | 0.011 | 0.3153 | No |
| 78 | FABP7 |  |  | 12242 | 0.011 | 0.3142 | No |
| 79 | CGREF1 |  |  | 12335 | 0.009 | 0.3101 | No |
| 80 | GML |  |  | 12406 | 0.008 | 0.3071 | No |
| 81 | S100A11 |  |  | 12776 | 0.002 | 0.2892 | No |
| 82 | CDKN1A |  |  | 12863 | 0.001 | 0.2850 | No |
| 83 | OSM |  |  | 12954 | -0.000 | 0.2806 | No |
| 84 | AIF1 |  |  | 13617 | -0.010 | 0.2488 | No |
| 85 | IL1B |  |  | 13976 | -0.016 | 0.2320 | No |
| 86 | NDN |  |  | 13980 | -0.016 | 0.2325 | No |
| 87 | SIRPB2 |  |  | 13998 | -0.016 | 0.2323 | No |
| 88 | SSTR2 |  |  | 14041 | -0.017 | 0.2310 | No |
| 89 | TNFRSF9 |  |  | 14598 | -0.025 | 0.2049 | No |
| 90 | MLLT7 |  |  | 14683 | -0.026 | 0.2019 | No |
| 91 | PLG |  |  | 15132 | -0.034 | 0.1815 | No |
| 92 | BAI1 |  |  | 15269 | -0.036 | 0.1764 | No |
| 93 | HGS |  |  | 15531 | -0.040 | 0.1654 | No |
| 94 | WARS |  |  | 15538 | -0.040 | 0.1668 | No |
| 95 | MXD4 |  |  | 15829 | -0.045 | 0.1545 | No |
| 96 | SESN1 |  |  | 16097 | -0.050 | 0.1436 | No |
| 97 | DEC1 |  |  | 16841 | -0.066 | 0.1102 | No |
| 98 | PPM1D |  |  | 16996 | -0.070 | 0.1056 | No |
| 99 | OPRM1 |  |  | 17338 | -0.079 | 0.0923 | No |
| 100 | BTG2 |  |  | 18002 | -0.099 | 0.0642 | No |
| 101 | SST |  |  | 18329 | -0.111 | 0.0529 | No |
| 102 | TM4SF4 |  |  | 18722 | -0.126 | 0.0391 | No |
| 103 | LDOC1 |  |  | 19097 | -0.146 | 0.0271 | No |
| 104 | GPNMB |  |  | 19565 | -0.185 | 0.0121 | No |
| 105 | CDKN2B |  |  | 19799 | -0.212 | 0.0097 | No |
| 106 | QSCN6 |  |  | 19801 | -0.212 | 0.0186 | No |
| 107 | BAP1 |  |  | 19828 | -0.216 | 0.0265 | No |
| 108 | CDC6 |  |  | 20126 | -0.270 | 0.0234 | No |
Table: GSEA details [plain text format]

  

Fig 2: SHEPARD\_NEG\_REG\_OF\_CELL\_PROLIFERATION: Random ES distribution      
 Gene set null distribution of ES for **SHEPARD\_NEG\_REG\_OF\_CELL\_PROLIFERATION**
