# Supplementary material for: The rapamycin-regulated gene expression signature determines prognosis for breast cancer
Source: Mol Cancer. 2009 Sep 24;8:75. doi: 10.1186/1476-4598-8-75 (PMC2761377; doi:10.1186/1476-4598-8-75)
Supplement: Additional file 2 — Gene set enrichment analysis of in vivo data, time series. The data provided represent the time series of GSEA. This compressed file contains "Time" shortcut file and "GSEA_time" folder. Clicking on "Time" shortcut opens the index file providing access to analysis files contained in the "GSEA_time" folder. [file 1476-4598-8-75-S2.zip › GSEA_time/SHIPP_FL_VS_DLBCL_DN.html]

Details for gene set SHIPP\_FL\_VS\_DLBCL\_DN[GSEA]

|  || Dataset | gsea\_time\_collapsed |
| Phenotype | NoPhenotypeAvailable |
| Upregulated in class | na\_neg |
| GeneSet | SHIPP\_FL\_VS\_DLBCL\_DN |
| Enrichment Score (ES) | -0.45687586 |
| Normalized Enrichment Score (NES) | -1.6324515 |
| Nominal p-value | 0.0 |
| FDR q-value | 0.14229107 |
| FWER p-Value | 0.825 |
Table: GSEA Results Summary

  

Fig 1: Enrichment plot: SHIPP\_FL\_VS\_DLBCL\_DN      
 Profile of the Running ES Score & Positions of GeneSet Members on the Rank Ordered List

  

| PROBE | GENE SYMBOL | GENE\_TITLE | RANK IN GENE LIST | RANK METRIC SCORE | RUNNING ES | CORE ENRICHMENT || 1 | HSPD1 |  |  | 336 | 0.617 | 0.0897 | No |
| 2 | CDKN3 |  |  | 1274 | 0.358 | 0.1057 | No |
| 3 | CCT5 |  |  | 2167 | 0.271 | 0.1090 | No |
| 4 | SLC29A1 |  |  | 2315 | 0.261 | 0.1466 | No |
| 5 | PFKP |  |  | 4731 | 0.146 | 0.0544 | No |
| 6 | CTSD |  |  | 5067 | 0.136 | 0.0614 | No |
| 7 | TFRC |  |  | 5832 | 0.116 | 0.0443 | No |
| 8 | GM2A |  |  | 5959 | 0.114 | 0.0577 | No |
| 9 | PKM2 |  |  | 6653 | 0.098 | 0.0409 | No |
| 10 | EXOSC7 |  |  | 8229 | 0.069 | -0.0238 | No |
| 11 | LDHA |  |  | 8499 | 0.064 | -0.0258 | No |
| 12 | MELK |  |  | 9282 | 0.053 | -0.0547 | No |
| 13 | NME1 |  |  | 11525 | 0.020 | -0.1603 | No |
| 14 | PSME2 |  |  | 11951 | 0.014 | -0.1784 | No |
| 15 | CCNB1 |  |  | 12244 | 0.011 | -0.1908 | No |
| 16 | CLTA |  |  | 13239 | -0.005 | -0.2383 | No |
| 17 | PRDX1 |  |  | 16586 | -0.061 | -0.3905 | No |
| 18 | DLG7 |  |  | 16705 | -0.063 | -0.3853 | No |
| 19 | BCL2A1 |  |  | 18179 | -0.105 | -0.4388 | Yes |
| 20 | ALDOA |  |  | 18247 | -0.108 | -0.4235 | Yes |
| 21 | PSMC1 |  |  | 18470 | -0.116 | -0.4143 | Yes |
| 22 | TRAP1 |  |  | 18667 | -0.125 | -0.4024 | Yes |
| 23 | P4HB |  |  | 19099 | -0.146 | -0.3982 | Yes |
| 24 | HSP90AA1 |  |  | 19182 | -0.152 | -0.3761 | Yes |
| 25 | MCM7 |  |  | 19264 | -0.157 | -0.3530 | Yes |
| 26 | IFI30 |  |  | 19605 | -0.189 | -0.3371 | Yes |
| 27 | MTHFD2 |  |  | 19664 | -0.196 | -0.3062 | Yes |
| 28 | IDH2 |  |  | 19803 | -0.213 | -0.2764 | Yes |
| 29 | CCT3 |  |  | 19813 | -0.214 | -0.2401 | Yes |
| 30 | ATIC |  |  | 20008 | -0.242 | -0.2079 | Yes |
| 31 | HSP90AB1 |  |  | 20186 | -0.290 | -0.1665 | Yes |
| 32 | PLOD1 |  |  | 20259 | -0.316 | -0.1157 | Yes |
| 33 | SLC1A5 |  |  | 20296 | -0.332 | -0.0603 | Yes |
| 34 | CENPA |  |  | 20463 | -0.438 | 0.0069 | Yes |
Table: GSEA details [plain text format]

  

Fig 2: SHIPP\_FL\_VS\_DLBCL\_DN: Random ES distribution      
 Gene set null distribution of ES for **SHIPP\_FL\_VS\_DLBCL\_DN**
