# Supplementary material for: The rapamycin-regulated gene expression signature determines prognosis for breast cancer
Source: Mol Cancer. 2009 Sep 24;8:75. doi: 10.1186/1476-4598-8-75 (PMC2761377; doi:10.1186/1476-4598-8-75)
Supplement: Additional file 2 — Gene set enrichment analysis of in vivo data, time series. The data provided represent the time series of GSEA. This compressed file contains "Time" shortcut file and "GSEA_time" folder. Clicking on "Time" shortcut opens the index file providing access to analysis files contained in the "GSEA_time" folder. [file 1476-4598-8-75-S2.zip › GSEA_time/STRESS_ARSENIC_SPECIFIC_DN.html]

Details for gene set STRESS\_ARSENIC\_SPECIFIC\_DN[GSEA]

|  || Dataset | gsea\_time\_collapsed |
| Phenotype | NoPhenotypeAvailable |
| Upregulated in class | na\_neg |
| GeneSet | STRESS\_ARSENIC\_SPECIFIC\_DN |
| Enrichment Score (ES) | -0.3693949 |
| Normalized Enrichment Score (NES) | -1.2836587 |
| Nominal p-value | 0.125 |
| FDR q-value | 0.33055553 |
| FWER p-Value | 1.0 |
Table: GSEA Results Summary

  

Fig 1: Enrichment plot: STRESS\_ARSENIC\_SPECIFIC\_DN      
 Profile of the Running ES Score & Positions of GeneSet Members on the Rank Ordered List

  

| PROBE | GENE SYMBOL | GENE\_TITLE | RANK IN GENE LIST | RANK METRIC SCORE | RUNNING ES | CORE ENRICHMENT || 1 | NKTR |  |  | 1378 | 0.343 | 0.0292 | No |
| 2 | RALGDS |  |  | 2644 | 0.239 | 0.0349 | No |
| 3 | DLC1 |  |  | 3135 | 0.212 | 0.0705 | No |
| 4 | TIMM17A |  |  | 4221 | 0.164 | 0.0637 | No |
| 5 | MMD |  |  | 4985 | 0.138 | 0.0652 | No |
| 6 | PTPRB |  |  | 5430 | 0.126 | 0.0787 | No |
| 7 | SPARCL1 |  |  | 5676 | 0.120 | 0.1004 | No |
| 8 | HNRPAB |  |  | 7415 | 0.083 | 0.0392 | No |
| 9 | TRIP13 |  |  | 7790 | 0.076 | 0.0424 | No |
| 10 | CCT8 |  |  | 7990 | 0.073 | 0.0532 | No |
| 11 | PEX11B |  |  | 8936 | 0.058 | 0.0235 | No |
| 12 | PAXIP1 |  |  | 9346 | 0.052 | 0.0182 | No |
| 13 | CRSP2 |  |  | 11981 | 0.014 | -0.1059 | No |
| 14 | DAG1 |  |  | 12130 | 0.012 | -0.1097 | No |
| 15 | FUT4 |  |  | 12792 | 0.001 | -0.1414 | No |
| 16 | CLEC2B |  |  | 13002 | -0.001 | -0.1512 | No |
| 17 | JUNB |  |  | 13719 | -0.012 | -0.1827 | No |
| 18 | ESRRA |  |  | 13733 | -0.012 | -0.1800 | No |
| 19 | CLK2 |  |  | 16626 | -0.062 | -0.3031 | No |
| 20 | RPUSD2 |  |  | 17202 | -0.076 | -0.3099 | No |
| 21 | AKAP1 |  |  | 18427 | -0.114 | -0.3373 | Yes |
| 22 | SH2B1 |  |  | 18465 | -0.116 | -0.3067 | Yes |
| 23 | HYAL2 |  |  | 18886 | -0.135 | -0.2892 | Yes |
| 24 | RRAGA |  |  | 19902 | -0.226 | -0.2753 | Yes |
| 25 | MAPK7 |  |  | 20151 | -0.277 | -0.2096 | Yes |
| 26 | FJX1 |  |  | 20285 | -0.325 | -0.1250 | Yes |
| 27 | ID3 |  |  | 20523 | -0.502 | 0.0040 | Yes |
Table: GSEA details [plain text format]

  

Fig 2: STRESS\_ARSENIC\_SPECIFIC\_DN: Random ES distribution      
 Gene set null distribution of ES for **STRESS\_ARSENIC\_SPECIFIC\_DN**
