# Supplementary material for: The rapamycin-regulated gene expression signature determines prognosis for breast cancer
Source: Mol Cancer. 2009 Sep 24;8:75. doi: 10.1186/1476-4598-8-75 (PMC2761377; doi:10.1186/1476-4598-8-75)
Supplement: Additional file 2 — Gene set enrichment analysis of in vivo data, time series. The data provided represent the time series of GSEA. This compressed file contains "Time" shortcut file and "GSEA_time" folder. Clicking on "Time" shortcut opens the index file providing access to analysis files contained in the "GSEA_time" folder. [file 1476-4598-8-75-S2.zip › GSEA_time/TAKEDA_NUP8_HOXA9_16D_DN.html]

Details for gene set TAKEDA\_NUP8\_HOXA9\_16D\_DN[GSEA]

|  || Dataset | gsea\_time\_collapsed |
| Phenotype | NoPhenotypeAvailable |
| Upregulated in class | na\_pos |
| GeneSet | TAKEDA\_NUP8\_HOXA9\_16D\_DN |
| Enrichment Score (ES) | 0.60518587 |
| Normalized Enrichment Score (NES) | 1.8289824 |
| Nominal p-value | 0.0 |
| FDR q-value | 0.005016165 |
| FWER p-Value | 0.1 |
Table: GSEA Results Summary

  

Fig 1: Enrichment plot: TAKEDA\_NUP8\_HOXA9\_16D\_DN      
 Profile of the Running ES Score & Positions of GeneSet Members on the Rank Ordered List

  

| PROBE | GENE SYMBOL | GENE\_TITLE | RANK IN GENE LIST | RANK METRIC SCORE | RUNNING ES | CORE ENRICHMENT || 1 | ELL2 |  |  | 30 | 1.258 | 0.0217 | Yes |
| 2 | ITGA9 |  |  | 49 | 1.128 | 0.0415 | Yes |
| 3 | TNCRNA |  |  | 51 | 1.121 | 0.0621 | Yes |
| 4 | ANKRD12 |  |  | 60 | 1.015 | 0.0804 | Yes |
| 5 | DCUN1D1 |  |  | 103 | 0.896 | 0.0948 | Yes |
| 6 | RARRES1 |  |  | 113 | 0.880 | 0.1105 | Yes |
| 7 | KIAA1641 |  |  | 146 | 0.807 | 0.1238 | Yes |
| 8 | S100P |  |  | 156 | 0.795 | 0.1380 | Yes |
| 9 | MAML2 |  |  | 166 | 0.780 | 0.1519 | Yes |
| 10 | BHLHB3 |  |  | 174 | 0.767 | 0.1656 | Yes |
| 11 | QKI |  |  | 293 | 0.656 | 0.1719 | Yes |
| 12 | CFLAR |  |  | 360 | 0.601 | 0.1797 | Yes |
| 13 | LRRFIP1 |  |  | 365 | 0.600 | 0.1906 | Yes |
| 14 | MLL5 |  |  | 375 | 0.595 | 0.2011 | Yes |
| 15 | TRA2A |  |  | 389 | 0.590 | 0.2113 | Yes |
| 16 | DKFZP313A2432 |  |  | 414 | 0.574 | 0.2207 | Yes |
| 17 | PAN3 |  |  | 417 | 0.572 | 0.2311 | Yes |
| 18 | USP12 |  |  | 427 | 0.566 | 0.2411 | Yes |
| 19 | ETV6 |  |  | 467 | 0.546 | 0.2492 | Yes |
| 20 | USP31 |  |  | 504 | 0.530 | 0.2572 | Yes |
| 21 | MBNL1 |  |  | 514 | 0.528 | 0.2664 | Yes |
| 22 | SYNJ2 |  |  | 525 | 0.523 | 0.2756 | Yes |
| 23 | TBC1D8 |  |  | 553 | 0.514 | 0.2837 | Yes |
| 24 | CUGBP2 |  |  | 577 | 0.506 | 0.2919 | Yes |
| 25 | LOC150759 |  |  | 578 | 0.506 | 0.3012 | Yes |
| 26 | ZNF644 |  |  | 605 | 0.498 | 0.3091 | Yes |
| 27 | NRP1 |  |  | 608 | 0.496 | 0.3181 | Yes |
| 28 | FLJ31951 |  |  | 629 | 0.491 | 0.3261 | Yes |
| 29 | ZNF395 |  |  | 636 | 0.488 | 0.3348 | Yes |
| 30 | CENTB2 |  |  | 661 | 0.481 | 0.3425 | Yes |
| 31 | DIP2A |  |  | 775 | 0.455 | 0.3453 | Yes |
| 32 | HS3ST1 |  |  | 875 | 0.427 | 0.3483 | Yes |
| 33 | PFAAP5 |  |  | 877 | 0.427 | 0.3561 | Yes |
| 34 | KIAA1267 |  |  | 915 | 0.420 | 0.3620 | Yes |
| 35 | FCHSD2 |  |  | 925 | 0.418 | 0.3693 | Yes |
| 36 | IMMP2L |  |  | 959 | 0.413 | 0.3753 | Yes |
| 37 | SPARC |  |  | 969 | 0.411 | 0.3824 | Yes |
| 38 | SVIL |  |  | 1006 | 0.404 | 0.3881 | Yes |
| 39 | NPTN |  |  | 1026 | 0.401 | 0.3945 | Yes |
| 40 | JMJD1C |  |  | 1042 | 0.397 | 0.4011 | Yes |
| 41 | RNF141 |  |  | 1062 | 0.394 | 0.4074 | Yes |
| 42 | ANKRD11 |  |  | 1088 | 0.389 | 0.4133 | Yes |
| 43 | COL4A3BP |  |  | 1174 | 0.375 | 0.4160 | Yes |
| 44 | PPFIA1 |  |  | 1204 | 0.370 | 0.4214 | Yes |
| 45 | RASEF |  |  | 1226 | 0.365 | 0.4271 | Yes |
| 46 | B4GALT5 |  |  | 1239 | 0.364 | 0.4332 | Yes |
| 47 | MTUS1 |  |  | 1289 | 0.356 | 0.4373 | Yes |
| 48 | FARP1 |  |  | 1291 | 0.356 | 0.4438 | Yes |
| 49 | ZNF518 |  |  | 1315 | 0.352 | 0.4492 | Yes |
| 50 | LOC153561 |  |  | 1368 | 0.345 | 0.4530 | Yes |
| 51 | AGTPBP1 |  |  | 1373 | 0.344 | 0.4591 | Yes |
| 52 | AFF1 |  |  | 1382 | 0.343 | 0.4650 | Yes |
| 53 | RASAL2 |  |  | 1402 | 0.341 | 0.4704 | Yes |
| 54 | TSPAN2 |  |  | 1441 | 0.336 | 0.4747 | Yes |
| 55 | ATXN1 |  |  | 1489 | 0.330 | 0.4784 | Yes |
| 56 | TGFBR1 |  |  | 1497 | 0.329 | 0.4842 | Yes |
| 57 | SEPP1 |  |  | 1538 | 0.325 | 0.4882 | Yes |
| 58 | PIAS1 |  |  | 1571 | 0.321 | 0.4925 | Yes |
| 59 | N4BP1 |  |  | 1576 | 0.320 | 0.4982 | Yes |
| 60 | ZNF398 |  |  | 1678 | 0.310 | 0.4989 | Yes |
| 61 | HP /// HPR |  |  | 1712 | 0.307 | 0.5030 | Yes |
| 62 | MTMR3 |  |  | 1997 | 0.284 | 0.4943 | Yes |
| 63 | ARID2 |  |  | 2076 | 0.278 | 0.4955 | Yes |
| 64 | FAM119A |  |  | 2159 | 0.272 | 0.4965 | Yes |
| 65 | LOC644655 |  |  | 2181 | 0.270 | 0.5005 | Yes |
| 66 | C6ORF105 |  |  | 2270 | 0.263 | 0.5010 | Yes |
| 67 | NT5C2 |  |  | 2314 | 0.261 | 0.5037 | Yes |
| 68 | CDYL |  |  | 2316 | 0.261 | 0.5084 | Yes |
| 69 | CD59 |  |  | 2344 | 0.259 | 0.5118 | Yes |
| 70 | VPS8 |  |  | 2425 | 0.253 | 0.5126 | Yes |
| 71 | STK4 |  |  | 2481 | 0.249 | 0.5145 | Yes |
| 72 | ZCCHC11 |  |  | 2492 | 0.248 | 0.5185 | Yes |
| 73 | PELI1 |  |  | 2506 | 0.247 | 0.5224 | Yes |
| 74 | PRDX2 |  |  | 2541 | 0.245 | 0.5253 | Yes |
| 75 | APOE |  |  | 2558 | 0.244 | 0.5290 | Yes |
| 76 | THRAP2 |  |  | 2617 | 0.241 | 0.5306 | Yes |
| 77 | NSF |  |  | 2640 | 0.239 | 0.5339 | Yes |
| 78 | ASXL2 |  |  | 2667 | 0.238 | 0.5370 | Yes |
| 79 | MSRA |  |  | 2728 | 0.234 | 0.5383 | Yes |
| 80 | GGA1 |  |  | 2737 | 0.234 | 0.5423 | Yes |
| 81 | TLE1 |  |  | 2761 | 0.232 | 0.5454 | Yes |
| 82 | DMXL1 |  |  | 2764 | 0.232 | 0.5496 | Yes |
| 83 | THRAP1 |  |  | 2780 | 0.231 | 0.5531 | Yes |
| 84 | CHI3L1 |  |  | 2851 | 0.227 | 0.5538 | Yes |
| 85 | PAPD4 |  |  | 2888 | 0.225 | 0.5562 | Yes |
| 86 | CDC14B |  |  | 2894 | 0.225 | 0.5601 | Yes |
| 87 | ZNF638 |  |  | 2947 | 0.222 | 0.5616 | Yes |
| 88 | IQWD1 |  |  | 2960 | 0.221 | 0.5651 | Yes |
| 89 | TFCP2 |  |  | 2976 | 0.220 | 0.5684 | Yes |
| 90 | AMPD3 |  |  | 3025 | 0.218 | 0.5701 | Yes |
| 91 | CAMK2G |  |  | 3156 | 0.211 | 0.5676 | Yes |
| 92 | DLEU2 |  |  | 3161 | 0.211 | 0.5713 | Yes |
| 93 | KPNA3 |  |  | 3192 | 0.209 | 0.5736 | Yes |
| 94 | CLTC |  |  | 3193 | 0.209 | 0.5775 | Yes |
| 95 | LOC153561 /// LOC643 |  |  | 3274 | 0.205 | 0.5773 | Yes |
| 96 | KBTBD10 |  |  | 3288 | 0.204 | 0.5804 | Yes |
| 97 | C21ORF96 |  |  | 3317 | 0.203 | 0.5828 | Yes |
| 98 | FGFR1 |  |  | 3349 | 0.202 | 0.5850 | Yes |
| 99 | PPAP2B |  |  | 3459 | 0.196 | 0.5833 | Yes |
| 100 | FLJ35740 |  |  | 3526 | 0.193 | 0.5836 | Yes |
| 101 | CCDC52 |  |  | 3538 | 0.192 | 0.5866 | Yes |
| 102 | DOCK4 |  |  | 3631 | 0.189 | 0.5855 | Yes |
| 103 | GPD2 |  |  | 3671 | 0.188 | 0.5871 | Yes |
| 104 | ARG1 |  |  | 3699 | 0.187 | 0.5892 | Yes |
| 105 | CORO2A |  |  | 3702 | 0.187 | 0.5925 | Yes |
| 106 | SLFN13 |  |  | 3791 | 0.183 | 0.5916 | Yes |
| 107 | LRRFIP2 |  |  | 3847 | 0.181 | 0.5922 | Yes |
| 108 | PTX3 |  |  | 3912 | 0.177 | 0.5923 | Yes |
| 109 | RBMS1 |  |  | 3919 | 0.177 | 0.5953 | Yes |
| 110 | RPS6KA2 |  |  | 3936 | 0.176 | 0.5978 | Yes |
| 111 | KIAA1450 |  |  | 3944 | 0.176 | 0.6006 | Yes |
| 112 | PRPF40A |  |  | 4076 | 0.170 | 0.5973 | Yes |
| 113 | LRP3 |  |  | 4081 | 0.170 | 0.6003 | Yes |
| 114 | CRISP3 |  |  | 4207 | 0.164 | 0.5972 | Yes |
| 115 | ERC1 |  |  | 4250 | 0.163 | 0.5981 | Yes |
| 116 | NCOR1 |  |  | 4281 | 0.162 | 0.5996 | Yes |
| 117 | PLTP |  |  | 4369 | 0.158 | 0.5983 | Yes |
| 118 | RPS27 /// LOC646233 |  |  | 4480 | 0.155 | 0.5957 | Yes |
| 119 | TACSTD2 |  |  | 4511 | 0.154 | 0.5971 | Yes |
| 120 | TNRC15 |  |  | 4542 | 0.152 | 0.5984 | Yes |
| 121 | RGR |  |  | 4562 | 0.152 | 0.6003 | Yes |
| 122 | FRMD4A |  |  | 4693 | 0.147 | 0.5966 | Yes |
| 123 | PROCR |  |  | 4716 | 0.147 | 0.5982 | Yes |
| 124 | MGAM /// LOC642103 |  |  | 4806 | 0.143 | 0.5965 | Yes |
| 125 | ZFAND3 |  |  | 4849 | 0.142 | 0.5970 | Yes |
| 126 | GLMN |  |  | 4933 | 0.139 | 0.5955 | Yes |
| 127 | CEBPE |  |  | 4938 | 0.139 | 0.5979 | Yes |
| 128 | SNF1LK2 |  |  | 4958 | 0.138 | 0.5995 | Yes |
| 129 | CYBRD1 |  |  | 4970 | 0.138 | 0.6015 | Yes |
| 130 | SPAG1 |  |  | 4992 | 0.138 | 0.6030 | Yes |
| 131 | ALPK1 |  |  | 5043 | 0.136 | 0.6031 | Yes |
| 132 | EDD1 |  |  | 5233 | 0.131 | 0.5962 | Yes |
| 133 | MMP1 |  |  | 5254 | 0.130 | 0.5976 | Yes |
| 134 | OLFML2B |  |  | 5267 | 0.130 | 0.5994 | Yes |
| 135 | SCN11A |  |  | 5361 | 0.127 | 0.5972 | Yes |
| 136 | DOCK8 |  |  | 5364 | 0.127 | 0.5994 | Yes |
| 137 | RAP1A |  |  | 5366 | 0.127 | 0.6017 | Yes |
| 138 | SLC11A2 |  |  | 5379 | 0.127 | 0.6035 | Yes |
| 139 | C6ORF192 |  |  | 5454 | 0.125 | 0.6022 | Yes |
| 140 | TNFAIP8 |  |  | 5514 | 0.124 | 0.6015 | Yes |
| 141 | TIA1 |  |  | 5518 | 0.124 | 0.6037 | Yes |
| 142 | PADI4 |  |  | 5561 | 0.123 | 0.6039 | Yes |
| 143 | LOC285540 |  |  | 5591 | 0.122 | 0.6047 | Yes |
| 144 | CXCL1 |  |  | 5663 | 0.120 | 0.6034 | Yes |
| 145 | URG4 |  |  | 5749 | 0.118 | 0.6014 | Yes |
| 146 | GPR137B |  |  | 5752 | 0.118 | 0.6035 | Yes |
| 147 | ZNF148 |  |  | 5767 | 0.118 | 0.6050 | Yes |
| 148 | NPL |  |  | 5807 | 0.117 | 0.6052 | Yes |
| 149 | CAPN3 |  |  | 5958 | 0.114 | 0.5999 | No |
| 150 | OLIG1 |  |  | 6081 | 0.111 | 0.5960 | No |
| 151 | DNAH11 |  |  | 6195 | 0.108 | 0.5924 | No |
| 152 | PDE4D |  |  | 6296 | 0.106 | 0.5895 | No |
| 153 | NIBP |  |  | 6328 | 0.105 | 0.5899 | No |
| 154 | LOC400960 |  |  | 6368 | 0.104 | 0.5899 | No |
| 155 | MAML3 |  |  | 6431 | 0.103 | 0.5887 | No |
| 156 | C20ORF74 |  |  | 6489 | 0.102 | 0.5878 | No |
| 157 | CDC14A |  |  | 6560 | 0.101 | 0.5862 | No |
| 158 | MGAT5 |  |  | 6572 | 0.100 | 0.5875 | No |
| 159 | KIAA0922 |  |  | 7082 | 0.089 | 0.5642 | No |
| 160 | CEACAM1 |  |  | 7267 | 0.086 | 0.5567 | No |
| 161 | ERG |  |  | 7314 | 0.085 | 0.5560 | No |
| 162 | ABCB5 |  |  | 7327 | 0.085 | 0.5570 | No |
| 163 | STIM1 |  |  | 7361 | 0.085 | 0.5569 | No |
| 164 | FLI1 |  |  | 7539 | 0.081 | 0.5498 | No |
| 165 | NRP2 |  |  | 7542 | 0.081 | 0.5511 | No |
| 166 | TMEM37 |  |  | 7543 | 0.081 | 0.5526 | No |
| 167 | GPR97 |  |  | 7877 | 0.075 | 0.5377 | No |
| 168 | SCGB3A1 |  |  | 7926 | 0.074 | 0.5367 | No |
| 169 | SFXN5 |  |  | 7948 | 0.074 | 0.5370 | No |
| 170 | ZFHX1B |  |  | 7962 | 0.073 | 0.5377 | No |
| 171 | C13ORF24 |  |  | 8117 | 0.071 | 0.5315 | No |
| 172 | ALOX5 |  |  | 8205 | 0.069 | 0.5285 | No |
| 173 | EXOSC7 |  |  | 8229 | 0.069 | 0.5286 | No |
| 174 | LOC643837 |  |  | 8395 | 0.066 | 0.5217 | No |
| 175 | LGALS12 |  |  | 8520 | 0.064 | 0.5168 | No |
| 176 | TFPI |  |  | 8641 | 0.063 | 0.5121 | No |
| 177 | KLF2 |  |  | 8795 | 0.060 | 0.5057 | No |
| 178 | SDS |  |  | 8851 | 0.059 | 0.5041 | No |
| 179 | MAF |  |  | 9000 | 0.057 | 0.4978 | No |
| 180 | C10ORF33 |  |  | 9131 | 0.055 | 0.4925 | No |
| 181 | KAL1 |  |  | 9393 | 0.051 | 0.4806 | No |
| 182 | HP |  |  | 9448 | 0.050 | 0.4789 | No |
| 183 | ALK |  |  | 9516 | 0.050 | 0.4765 | No |
| 184 | PISD |  |  | 9567 | 0.049 | 0.4749 | No |
| 185 | PIP3-E |  |  | 9604 | 0.048 | 0.4741 | No |
| 186 | ARFGEF1 |  |  | 10047 | 0.041 | 0.4531 | No |
| 187 | PQLC1 |  |  | 10151 | 0.040 | 0.4488 | No |
| 188 | CEACAM6 |  |  | 10266 | 0.038 | 0.4439 | No |
| 189 | CD24 |  |  | 10395 | 0.036 | 0.4383 | No |
| 190 | FCGBP |  |  | 10490 | 0.035 | 0.4343 | No |
| 191 | SULT1C1 |  |  | 10502 | 0.035 | 0.4344 | No |
| 192 | VSTM1 |  |  | 10638 | 0.033 | 0.4284 | No |
| 193 | HEXA |  |  | 10641 | 0.033 | 0.4289 | No |
| 194 | ABCA13 |  |  | 11096 | 0.026 | 0.4071 | No |
| 195 | DEFA4 |  |  | 11281 | 0.024 | 0.3985 | No |
| 196 | ANXA3 |  |  | 11767 | 0.017 | 0.3750 | No |
| 197 | RBP1 |  |  | 11919 | 0.015 | 0.3679 | No |
| 198 | CYP4F3 |  |  | 12189 | 0.011 | 0.3549 | No |
| 199 | SLC16A10 |  |  | 12580 | 0.005 | 0.3359 | No |
| 200 | BPI |  |  | 12779 | 0.002 | 0.3262 | No |
| 201 | DPP4 |  |  | 13052 | -0.002 | 0.3129 | No |
| 202 | CXCL5 |  |  | 13115 | -0.003 | 0.3099 | No |
| 203 | TCN1 |  |  | 13788 | -0.013 | 0.2771 | No |
| 204 | PSMB2 |  |  | 13941 | -0.015 | 0.2699 | No |
| 205 | TNS1 |  |  | 14095 | -0.017 | 0.2628 | No |
| 206 | NCF1 /// LOC653361 / |  |  | 14195 | -0.019 | 0.2582 | No |
| 207 | PGA5 /// LOC643834 / |  |  | 14640 | -0.026 | 0.2369 | No |
| 208 | MMP9 |  |  | 14832 | -0.029 | 0.2281 | No |
| 209 | ARHGAP15 |  |  | 15075 | -0.033 | 0.2168 | No |
| 210 | GGTA1 |  |  | 15093 | -0.033 | 0.2166 | No |
| 211 | ARMCX1 |  |  | 15462 | -0.039 | 0.1993 | No |
| 212 | MGC24103 |  |  | 15858 | -0.046 | 0.1807 | No |
| 213 | FOLR3 |  |  | 16279 | -0.054 | 0.1611 | No |
| 214 | CEACAM8 |  |  | 16337 | -0.055 | 0.1593 | No |
| 215 | KIAA0754 |  |  | 16484 | -0.059 | 0.1532 | No |
| 216 | ADORA3 |  |  | 16582 | -0.061 | 0.1496 | No |
| 217 | LOC648998 /// LOC653 |  |  | 16949 | -0.069 | 0.1329 | No |
| 218 | LTF /// LOC643349 |  |  | 17333 | -0.079 | 0.1156 | No |
| 219 | PGLYRP1 |  |  | 17388 | -0.080 | 0.1144 | No |
| 220 | MMP8 |  |  | 17741 | -0.091 | 0.0988 | No |
| 221 | KIF21B |  |  | 17776 | -0.092 | 0.0988 | No |
| 222 | IGFBP4 |  |  | 17975 | -0.098 | 0.0909 | No |
| 223 | CD24 /// LOC647456 |  |  | 18206 | -0.106 | 0.0815 | No |
| 224 | CSTB |  |  | 20247 | -0.312 | -0.0128 | No |
| 225 | CPNE2 |  |  | 20300 | -0.334 | -0.0092 | No |
| 226 | IL1R2 |  |  | 20419 | -0.400 | -0.0077 | No |
| 227 | C10ORF58 |  |  | 20476 | -0.450 | -0.0021 | No |
| 228 | SLPI |  |  | 20486 | -0.458 | 0.0058 | No |
Table: GSEA details [plain text format]

  

Fig 2: TAKEDA\_NUP8\_HOXA9\_16D\_DN: Random ES distribution      
 Gene set null distribution of ES for **TAKEDA\_NUP8\_HOXA9\_16D\_DN**
