# Supplementary material for: The rapamycin-regulated gene expression signature determines prognosis for breast cancer
Source: Mol Cancer. 2009 Sep 24;8:75. doi: 10.1186/1476-4598-8-75 (PMC2761377; doi:10.1186/1476-4598-8-75)
Supplement: Additional file 2 — Gene set enrichment analysis of in vivo data, time series. The data provided represent the time series of GSEA. This compressed file contains "Time" shortcut file and "GSEA_time" folder. Clicking on "Time" shortcut opens the index file providing access to analysis files contained in the "GSEA_time" folder. [file 1476-4598-8-75-S2.zip › GSEA_time/TAKEDA_NUP8_HOXA9_6H_UP.html]

Details for gene set TAKEDA\_NUP8\_HOXA9\_6H\_UP[GSEA]

|  || Dataset | gsea\_time\_collapsed |
| Phenotype | NoPhenotypeAvailable |
| Upregulated in class | na\_pos |
| GeneSet | TAKEDA\_NUP8\_HOXA9\_6H\_UP |
| Enrichment Score (ES) | 0.6161591 |
| Normalized Enrichment Score (NES) | 1.7792703 |
| Nominal p-value | 0.0 |
| FDR q-value | 0.008906297 |
| FWER p-Value | 0.26 |
Table: GSEA Results Summary

  

Fig 1: Enrichment plot: TAKEDA\_NUP8\_HOXA9\_6H\_UP      
 Profile of the Running ES Score & Positions of GeneSet Members on the Rank Ordered List

  

| PROBE | GENE SYMBOL | GENE\_TITLE | RANK IN GENE LIST | RANK METRIC SCORE | RUNNING ES | CORE ENRICHMENT || 1 | CRIM1 |  |  | 16 | 1.496 | 0.0872 | Yes |
| 2 | GLYATL2 |  |  | 48 | 1.133 | 0.1524 | Yes |
| 3 | NME7 |  |  | 59 | 1.021 | 0.2120 | Yes |
| 4 | FRMD6 |  |  | 97 | 0.908 | 0.2636 | Yes |
| 5 | TMEM46 |  |  | 154 | 0.796 | 0.3078 | Yes |
| 6 | HOXA3 |  |  | 195 | 0.732 | 0.3489 | Yes |
| 7 | HOXC6 |  |  | 565 | 0.511 | 0.3610 | Yes |
| 8 | PDE4DIP |  |  | 612 | 0.494 | 0.3879 | Yes |
| 9 | SLC25A36 |  |  | 653 | 0.484 | 0.4144 | Yes |
| 10 | MMP7 |  |  | 835 | 0.437 | 0.4313 | Yes |
| 11 | ITGB8 |  |  | 841 | 0.436 | 0.4567 | Yes |
| 12 | DLX2 |  |  | 885 | 0.425 | 0.4796 | Yes |
| 13 | HOXB3 |  |  | 899 | 0.423 | 0.5039 | Yes |
| 14 | SPRED1 |  |  | 1259 | 0.361 | 0.5076 | Yes |
| 15 | GLIPR1 |  |  | 1753 | 0.303 | 0.5015 | Yes |
| 16 | KLF5 |  |  | 1827 | 0.298 | 0.5154 | Yes |
| 17 | S100A10 |  |  | 2017 | 0.283 | 0.5229 | Yes |
| 18 | USP32 |  |  | 2311 | 0.261 | 0.5239 | Yes |
| 19 | STK4 |  |  | 2481 | 0.249 | 0.5303 | Yes |
| 20 | PTPN13 |  |  | 2504 | 0.247 | 0.5438 | Yes |
| 21 | DKFZP686A01247 |  |  | 2601 | 0.241 | 0.5533 | Yes |
| 22 | SCML1 |  |  | 2614 | 0.241 | 0.5669 | Yes |
| 23 | FIGN |  |  | 2615 | 0.241 | 0.5811 | Yes |
| 24 | PLA2G4A |  |  | 2718 | 0.235 | 0.5900 | Yes |
| 25 | PCDH9 |  |  | 2784 | 0.231 | 0.6004 | Yes |
| 26 | INPP4B |  |  | 2807 | 0.229 | 0.6128 | Yes |
| 27 | KMO |  |  | 3072 | 0.215 | 0.6126 | Yes |
| 28 | SEMA3C |  |  | 3448 | 0.197 | 0.6059 | Yes |
| 29 | LOC90355 |  |  | 3475 | 0.195 | 0.6162 | Yes |
| 30 | IL18 |  |  | 3973 | 0.175 | 0.6022 | No |
| 31 | TOX |  |  | 4209 | 0.164 | 0.6004 | No |
| 32 | FGF7 /// KGFLP1 /// |  |  | 4399 | 0.157 | 0.6005 | No |
| 33 | CEP70 |  |  | 5367 | 0.127 | 0.5609 | No |
| 34 | EVI1 |  |  | 5606 | 0.121 | 0.5564 | No |
| 35 | LOC404266 |  |  | 5879 | 0.115 | 0.5499 | No |
| 36 | HOXA5 |  |  | 5912 | 0.115 | 0.5551 | No |
| 37 | DMXL2 |  |  | 6527 | 0.101 | 0.5312 | No |
| 38 | CALCRL |  |  | 6582 | 0.100 | 0.5344 | No |
| 39 | ADRBK2 |  |  | 6718 | 0.097 | 0.5335 | No |
| 40 | PCOLCE2 |  |  | 6720 | 0.097 | 0.5392 | No |
| 41 | FLJ20366 |  |  | 6880 | 0.093 | 0.5369 | No |
| 42 | DHRS9 |  |  | 7184 | 0.088 | 0.5274 | No |
| 43 | PLCL1 |  |  | 7280 | 0.086 | 0.5278 | No |
| 44 | PBX3 |  |  | 7567 | 0.081 | 0.5186 | No |
| 45 | XCL1 /// XCL2 |  |  | 7776 | 0.076 | 0.5129 | No |
| 46 | ZNF503 |  |  | 8022 | 0.072 | 0.5053 | No |
| 47 | TYRP1 |  |  | 8321 | 0.067 | 0.4947 | No |
| 48 | SLCO4C1 |  |  | 8948 | 0.058 | 0.4676 | No |
| 49 | HPGD |  |  | 9136 | 0.055 | 0.4617 | No |
| 50 | SCN2A2 |  |  | 9241 | 0.053 | 0.4598 | No |
| 51 | GNAI1 |  |  | 10557 | 0.034 | 0.3977 | No |
| 52 | TEX15 |  |  | 10988 | 0.027 | 0.3783 | No |
| 53 | CMAH |  |  | 11561 | 0.020 | 0.3516 | No |
| 54 | SDPR |  |  | 12330 | 0.009 | 0.3147 | No |
| 55 | CA8 |  |  | 12369 | 0.009 | 0.3134 | No |
| 56 | MSX1 |  |  | 12402 | 0.008 | 0.3123 | No |
| 57 | HOXA9 |  |  | 12432 | 0.008 | 0.3114 | No |
| 58 | P2RY12 |  |  | 12687 | 0.003 | 0.2992 | No |
| 59 | C14ORF125 |  |  | 12901 | 0.000 | 0.2888 | No |
| 60 | NRG4 |  |  | 12983 | -0.001 | 0.2849 | No |
| 61 | CD69 |  |  | 13127 | -0.003 | 0.2781 | No |
| 62 | C4ORF18 |  |  | 13158 | -0.003 | 0.2769 | No |
| 63 | IFNB1 |  |  | 13417 | -0.008 | 0.2647 | No |
| 64 | STAT4 |  |  | 13565 | -0.010 | 0.2581 | No |
| 65 | PLN |  |  | 14440 | -0.023 | 0.2169 | No |
| 66 | DACH1 |  |  | 14796 | -0.028 | 0.2013 | No |
| 67 | MCTP1 |  |  | 15056 | -0.033 | 0.1906 | No |
| 68 | IGJ |  |  | 15232 | -0.035 | 0.1841 | No |
| 69 | P2RY5 |  |  | 15294 | -0.036 | 0.1833 | No |
| 70 | EVI2A |  |  | 15481 | -0.039 | 0.1765 | No |
| 71 | OLFM3 |  |  | 15537 | -0.040 | 0.1762 | No |
| 72 | FLT3 |  |  | 15586 | -0.041 | 0.1762 | No |
| 73 | GAS2L3 |  |  | 15774 | -0.044 | 0.1697 | No |
| 74 | LOC647248 /// LOC647 |  |  | 16090 | -0.050 | 0.1573 | No |
| 75 | HOXA4 |  |  | 16105 | -0.050 | 0.1596 | No |
| 76 | TSPAN12 |  |  | 16147 | -0.051 | 0.1606 | No |
| 77 | SERPINI1 |  |  | 16456 | -0.058 | 0.1490 | No |
| 78 | LOC645591 |  |  | 16538 | -0.060 | 0.1486 | No |
| 79 | LOC401097 |  |  | 17457 | -0.082 | 0.1087 | No |
| 80 | XCL2 |  |  | 17763 | -0.091 | 0.0992 | No |
| 81 | CDH9 |  |  | 18165 | -0.105 | 0.0859 | No |
| 82 | MS4A4A |  |  | 18283 | -0.109 | 0.0866 | No |
| 83 | ST6GALNAC1 |  |  | 18335 | -0.111 | 0.0907 | No |
| 84 | GPR21 |  |  | 19135 | -0.149 | 0.0605 | No |
| 85 | FKBP5 |  |  | 19601 | -0.189 | 0.0489 | No |
Table: GSEA details [plain text format]

  

Fig 2: TAKEDA\_NUP8\_HOXA9\_6H\_UP: Random ES distribution      
 Gene set null distribution of ES for **TAKEDA\_NUP8\_HOXA9\_6H\_UP**
