# Supplementary material for: The rapamycin-regulated gene expression signature determines prognosis for breast cancer
Source: Mol Cancer. 2009 Sep 24;8:75. doi: 10.1186/1476-4598-8-75 (PMC2761377; doi:10.1186/1476-4598-8-75)
Supplement: Additional file 2 — Gene set enrichment analysis of in vivo data, time series. The data provided represent the time series of GSEA. This compressed file contains "Time" shortcut file and "GSEA_time" folder. Clicking on "Time" shortcut opens the index file providing access to analysis files contained in the "GSEA_time" folder. [file 1476-4598-8-75-S2.zip › GSEA_time/TCA.html]

Details for gene set TCA[GSEA]

|  || Dataset | gsea\_time\_collapsed |
| Phenotype | NoPhenotypeAvailable |
| Upregulated in class | na\_neg |
| GeneSet | TCA |
| Enrichment Score (ES) | -0.62553877 |
| Normalized Enrichment Score (NES) | -1.8530695 |
| Nominal p-value | 0.0 |
| FDR q-value | 0.08266473 |
| FWER p-Value | 0.294 |
Table: GSEA Results Summary

  

Fig 1: Enrichment plot: TCA      
 Profile of the Running ES Score & Positions of GeneSet Members on the Rank Ordered List

  

| PROBE | GENE SYMBOL | GENE\_TITLE | RANK IN GENE LIST | RANK METRIC SCORE | RUNNING ES | CORE ENRICHMENT || 1 | SDHC |  |  | 4051 | 0.171 | -0.1300 | No |
| 2 | OGDH |  |  | 5727 | 0.119 | -0.1651 | No |
| 3 | IDH3A |  |  | 6869 | 0.094 | -0.1840 | No |
| 4 | MDH2 |  |  | 7052 | 0.090 | -0.1576 | No |
| 5 | SUCLA2 |  |  | 8448 | 0.065 | -0.1999 | No |
| 6 | SDHB |  |  | 9306 | 0.052 | -0.2210 | No |
| 7 | FH |  |  | 15798 | -0.045 | -0.5189 | No |
| 8 | IDH3G |  |  | 16278 | -0.054 | -0.5209 | No |
| 9 | SDHD |  |  | 18044 | -0.101 | -0.5675 | Yes |
| 10 | CS |  |  | 19240 | -0.156 | -0.5648 | Yes |
| 11 | IDH2 |  |  | 19803 | -0.213 | -0.5093 | Yes |
| 12 | SDHA |  |  | 20118 | -0.266 | -0.4208 | Yes |
| 13 | IDH3B |  |  | 20127 | -0.270 | -0.3160 | Yes |
| 14 | SUCLG1 |  |  | 20440 | -0.417 | -0.1687 | Yes |
| 15 | ACO2 |  |  | 20483 | -0.453 | 0.0059 | Yes |
Table: GSEA details [plain text format]

  

Fig 2: TCA: Random ES distribution      
 Gene set null distribution of ES for **TCA**
