# Supplementary material for: The rapamycin-regulated gene expression signature determines prognosis for breast cancer
Source: Mol Cancer. 2009 Sep 24;8:75. doi: 10.1186/1476-4598-8-75 (PMC2761377; doi:10.1186/1476-4598-8-75)
Supplement: Additional file 2 — Gene set enrichment analysis of in vivo data, time series. The data provided represent the time series of GSEA. This compressed file contains "Time" shortcut file and "GSEA_time" folder. Clicking on "Time" shortcut opens the index file providing access to analysis files contained in the "GSEA_time" folder. [file 1476-4598-8-75-S2.zip › GSEA_time/TFF2_KO_UP.html]

Details for gene set TFF2\_KO\_UP[GSEA]

|  || Dataset | gsea\_time\_collapsed |
| Phenotype | NoPhenotypeAvailable |
| Upregulated in class | na\_neg |
| GeneSet | TFF2\_KO\_UP |
| Enrichment Score (ES) | -0.39229825 |
| Normalized Enrichment Score (NES) | -1.2861965 |
| Nominal p-value | 0.14583333 |
| FDR q-value | 0.33400252 |
| FWER p-Value | 1.0 |
Table: GSEA Results Summary

  

Fig 1: Enrichment plot: TFF2\_KO\_UP      
 Profile of the Running ES Score & Positions of GeneSet Members on the Rank Ordered List

  

| PROBE | GENE SYMBOL | GENE\_TITLE | RANK IN GENE LIST | RANK METRIC SCORE | RUNNING ES | CORE ENRICHMENT || 1 | KDELR2 |  |  | 1225 | 0.365 | 0.0796 | No |
| 2 | SAMHD1 |  |  | 4537 | 0.153 | -0.0229 | No |
| 3 | GNB1 |  |  | 5953 | 0.114 | -0.0482 | No |
| 4 | CCL8 |  |  | 5970 | 0.113 | -0.0057 | No |
| 5 | PTPN22 |  |  | 6678 | 0.098 | -0.0028 | No |
| 6 | APOA4 |  |  | 6889 | 0.093 | 0.0225 | No |
| 7 | TFF3 |  |  | 7274 | 0.086 | 0.0367 | No |
| 8 | ABCG2 |  |  | 9089 | 0.055 | -0.0303 | No |
| 9 | DPEP1 |  |  | 10510 | 0.035 | -0.0861 | No |
| 10 | MS4A1 |  |  | 11058 | 0.027 | -0.1024 | No |
| 11 | BCAT2 |  |  | 11369 | 0.023 | -0.1089 | No |
| 12 | LTB |  |  | 12563 | 0.005 | -0.1648 | No |
| 13 | CRIP1 |  |  | 13665 | -0.011 | -0.2141 | No |
| 14 | CD52 |  |  | 14645 | -0.026 | -0.2518 | No |
| 15 | DGKA |  |  | 15227 | -0.035 | -0.2666 | No |
| 16 | ZG16 |  |  | 15492 | -0.039 | -0.2644 | No |
| 17 | TCF8 |  |  | 16274 | -0.054 | -0.2816 | No |
| 18 | TAP1 |  |  | 18553 | -0.120 | -0.3466 | Yes |
| 19 | SLC2A2 |  |  | 19077 | -0.145 | -0.3167 | Yes |
| 20 | OGFR |  |  | 19153 | -0.150 | -0.2631 | Yes |
| 21 | RBP2 |  |  | 19467 | -0.176 | -0.2114 | Yes |
| 22 | IRF7 |  |  | 19564 | -0.185 | -0.1455 | Yes |
| 23 | PSMB8 |  |  | 20531 | -0.514 | 0.0036 | Yes |
Table: GSEA details [plain text format]

  

Fig 2: TFF2\_KO\_UP: Random ES distribution      
 Gene set null distribution of ES for **TFF2\_KO\_UP**
