# Supplementary material for: The rapamycin-regulated gene expression signature determines prognosis for breast cancer
Source: Mol Cancer. 2009 Sep 24;8:75. doi: 10.1186/1476-4598-8-75 (PMC2761377; doi:10.1186/1476-4598-8-75)
Supplement: Additional file 2 — Gene set enrichment analysis of in vivo data, time series. The data provided represent the time series of GSEA. This compressed file contains "Time" shortcut file and "GSEA_time" folder. Clicking on "Time" shortcut opens the index file providing access to analysis files contained in the "GSEA_time" folder. [file 1476-4598-8-75-S2.zip › GSEA_time/TGFBETA_C2_UP.html]

Details for gene set TGFBETA\_C2\_UP[GSEA]

|  || Dataset | gsea\_time\_collapsed |
| Phenotype | NoPhenotypeAvailable |
| Upregulated in class | na\_neg |
| GeneSet | TGFBETA\_C2\_UP |
| Enrichment Score (ES) | -0.54105914 |
| Normalized Enrichment Score (NES) | -1.6992931 |
| Nominal p-value | 0.01764706 |
| FDR q-value | 0.13563196 |
| FWER p-Value | 0.675 |
Table: GSEA Results Summary

  

Fig 1: Enrichment plot: TGFBETA\_C2\_UP      
 Profile of the Running ES Score & Positions of GeneSet Members on the Rank Ordered List

  

| PROBE | GENE SYMBOL | GENE\_TITLE | RANK IN GENE LIST | RANK METRIC SCORE | RUNNING ES | CORE ENRICHMENT || 1 | ITGB2 |  |  | 438 | 0.560 | 0.1500 | No |
| 2 | ICAM1 |  |  | 3140 | 0.212 | 0.0835 | No |
| 3 | DVL1 |  |  | 4056 | 0.171 | 0.0915 | No |
| 4 | COL6A1 |  |  | 5412 | 0.126 | 0.0642 | No |
| 5 | FN1 |  |  | 9897 | 0.043 | -0.1404 | No |
| 6 | LRP1 |  |  | 10328 | 0.037 | -0.1499 | No |
| 7 | MMP14 |  |  | 11968 | 0.014 | -0.2251 | No |
| 8 | COL3A1 |  |  | 14444 | -0.023 | -0.3384 | No |
| 9 | ARHGDIA |  |  | 14579 | -0.025 | -0.3374 | No |
| 10 | CDH6 |  |  | 14878 | -0.030 | -0.3428 | No |
| 11 | WNT2B |  |  | 14886 | -0.030 | -0.3340 | No |
| 12 | COL6A3 |  |  | 16068 | -0.050 | -0.3762 | No |
| 13 | COL1A2 |  |  | 16667 | -0.063 | -0.3861 | No |
| 14 | MMP3 |  |  | 17111 | -0.073 | -0.3854 | No |
| 15 | JUP |  |  | 20316 | -0.341 | -0.4367 | Yes |
| 16 | RHOG |  |  | 20391 | -0.378 | -0.3249 | Yes |
| 17 | TIMP1 |  |  | 20516 | -0.498 | -0.1786 | Yes |
| 18 | MARCKSL1 |  |  | 20565 | -0.598 | 0.0019 | Yes |
Table: GSEA details [plain text format]

  

Fig 2: TGFBETA\_C2\_UP: Random ES distribution      
 Gene set null distribution of ES for **TGFBETA\_C2\_UP**
