# Supplementary material for: The rapamycin-regulated gene expression signature determines prognosis for breast cancer
Source: Mol Cancer. 2009 Sep 24;8:75. doi: 10.1186/1476-4598-8-75 (PMC2761377; doi:10.1186/1476-4598-8-75)
Supplement: Additional file 2 — Gene set enrichment analysis of in vivo data, time series. The data provided represent the time series of GSEA. This compressed file contains "Time" shortcut file and "GSEA_time" folder. Clicking on "Time" shortcut opens the index file providing access to analysis files contained in the "GSEA_time" folder. [file 1476-4598-8-75-S2.zip › GSEA_time/TGFBETA_EARLY_UP.html]

Details for gene set TGFBETA\_EARLY\_UP[GSEA]

|  || Dataset | gsea\_time\_collapsed |
| Phenotype | NoPhenotypeAvailable |
| Upregulated in class | na\_neg |
| GeneSet | TGFBETA\_EARLY\_UP |
| Enrichment Score (ES) | -0.3667477 |
| Normalized Enrichment Score (NES) | -1.3528827 |
| Nominal p-value | 0.05154639 |
| FDR q-value | 0.2895258 |
| FWER p-Value | 1.0 |
Table: GSEA Results Summary

  

Fig 1: Enrichment plot: TGFBETA\_EARLY\_UP      
 Profile of the Running ES Score & Positions of GeneSet Members on the Rank Ordered List

  

| PROBE | GENE SYMBOL | GENE\_TITLE | RANK IN GENE LIST | RANK METRIC SCORE | RUNNING ES | CORE ENRICHMENT || 1 | TCF7L2 |  |  | 246 | 0.684 | 0.0527 | No |
| 2 | ITGB2 |  |  | 438 | 0.560 | 0.0964 | No |
| 3 | CD44 |  |  | 641 | 0.487 | 0.1326 | No |
| 4 | SPARC |  |  | 969 | 0.411 | 0.1556 | No |
| 5 | PAK1 |  |  | 1156 | 0.378 | 0.1822 | No |
| 6 | IGFBP3 |  |  | 1528 | 0.326 | 0.1949 | No |
| 7 | RHOQ |  |  | 1568 | 0.321 | 0.2234 | No |
| 8 | ADAM9 |  |  | 1582 | 0.320 | 0.2530 | No |
| 9 | ITGB5 |  |  | 1900 | 0.293 | 0.2653 | No |
| 10 | PLAT |  |  | 2048 | 0.280 | 0.2846 | No |
| 11 | CD59 |  |  | 2344 | 0.259 | 0.2947 | No |
| 12 | IGFBP5 |  |  | 2817 | 0.229 | 0.2934 | No |
| 13 | ICAM1 |  |  | 3140 | 0.212 | 0.2977 | No |
| 14 | RND3 |  |  | 3338 | 0.202 | 0.3073 | No |
| 15 | DVL1 |  |  | 4056 | 0.171 | 0.2886 | No |
| 16 | CSPG2 |  |  | 4202 | 0.164 | 0.2971 | No |
| 17 | NOTCH2 |  |  | 5110 | 0.135 | 0.2657 | No |
| 18 | COL6A1 |  |  | 5412 | 0.126 | 0.2630 | No |
| 19 | IGF2R |  |  | 7796 | 0.076 | 0.1543 | No |
| 20 | RHOB |  |  | 8609 | 0.063 | 0.1207 | No |
| 21 | FN1 |  |  | 9897 | 0.043 | 0.0622 | No |
| 22 | LRP1 |  |  | 10328 | 0.037 | 0.0448 | No |
| 23 | LAMA4 |  |  | 10458 | 0.035 | 0.0419 | No |
| 24 | RHOA |  |  | 10990 | 0.027 | 0.0186 | No |
| 25 | MMP14 |  |  | 11968 | 0.014 | -0.0275 | No |
| 26 | LRRC17 |  |  | 12808 | 0.001 | -0.0682 | No |
| 27 | MMP19 |  |  | 13884 | -0.014 | -0.1192 | No |
| 28 | COL3A1 |  |  | 14444 | -0.023 | -0.1442 | No |
| 29 | PXN |  |  | 14552 | -0.024 | -0.1472 | No |
| 30 | ARHGDIA |  |  | 14579 | -0.025 | -0.1461 | No |
| 31 | HSPG2 |  |  | 14854 | -0.029 | -0.1567 | No |
| 32 | CDH6 |  |  | 14878 | -0.030 | -0.1550 | No |
| 33 | WNT2B |  |  | 14886 | -0.030 | -0.1525 | No |
| 34 | EPHB3 |  |  | 15117 | -0.034 | -0.1605 | No |
| 35 | COL6A3 |  |  | 16068 | -0.050 | -0.2020 | No |
| 36 | NID1 |  |  | 16638 | -0.062 | -0.2238 | No |
| 37 | COL1A2 |  |  | 16667 | -0.063 | -0.2193 | No |
| 38 | MMP3 |  |  | 17111 | -0.073 | -0.2340 | No |
| 39 | SERPINE1 |  |  | 18412 | -0.114 | -0.2864 | No |
| 40 | ITGA3 |  |  | 18758 | -0.129 | -0.2910 | No |
| 41 | JUP |  |  | 20316 | -0.341 | -0.3345 | Yes |
| 42 | RHOG |  |  | 20391 | -0.378 | -0.3024 | Yes |
| 43 | BSG |  |  | 20511 | -0.492 | -0.2617 | Yes |
| 44 | TIMP1 |  |  | 20516 | -0.498 | -0.2148 | Yes |
| 45 | MARCKSL1 |  |  | 20565 | -0.598 | -0.1607 | Yes |
| 46 | ZYX |  |  | 20579 | -0.653 | -0.0995 | Yes |
| 47 | IGFBP2 |  |  | 20605 | -1.066 | -0.0000 | Yes |
Table: GSEA details [plain text format]

  

Fig 2: TGFBETA\_EARLY\_UP: Random ES distribution      
 Gene set null distribution of ES for **TGFBETA\_EARLY\_UP**
