# Supplementary material for: The rapamycin-regulated gene expression signature determines prognosis for breast cancer
Source: Mol Cancer. 2009 Sep 24;8:75. doi: 10.1186/1476-4598-8-75 (PMC2761377; doi:10.1186/1476-4598-8-75)
Supplement: Additional file 2 — Gene set enrichment analysis of in vivo data, time series. The data provided represent the time series of GSEA. This compressed file contains "Time" shortcut file and "GSEA_time" folder. Clicking on "Time" shortcut opens the index file providing access to analysis files contained in the "GSEA_time" folder. [file 1476-4598-8-75-S2.zip › GSEA_time/TSADAC_RKOEXP_UP.html]

Details for gene set TSADAC\_RKOEXP\_UP[GSEA]

|  || Dataset | gsea\_time\_collapsed |
| Phenotype | NoPhenotypeAvailable |
| Upregulated in class | na\_pos |
| GeneSet | TSADAC\_RKOEXP\_UP |
| Enrichment Score (ES) | 0.75063896 |
| Normalized Enrichment Score (NES) | 1.7527285 |
| Nominal p-value | 0.0 |
| FDR q-value | 0.011243024 |
| FWER p-Value | 0.379 |
Table: GSEA Results Summary

  

Fig 1: Enrichment plot: TSADAC\_RKOEXP\_UP      
 Profile of the Running ES Score & Positions of GeneSet Members on the Rank Ordered List

  

| PROBE | GENE SYMBOL | GENE\_TITLE | RANK IN GENE LIST | RANK METRIC SCORE | RUNNING ES | CORE ENRICHMENT || 1 | CXCL3 |  |  | 162 | 0.784 | 0.1710 | Yes |
| 2 | IFITM1 |  |  | 275 | 0.669 | 0.3181 | Yes |
| 3 | HNRPD |  |  | 281 | 0.666 | 0.4698 | Yes |
| 4 | MAP3K13 |  |  | 300 | 0.648 | 0.6167 | Yes |
| 5 | ZNF532 |  |  | 363 | 0.600 | 0.7506 | Yes |
| 6 | C8ORF4 |  |  | 4182 | 0.165 | 0.6029 | No |
| 7 | HOXA1 |  |  | 6729 | 0.097 | 0.5013 | No |
| 8 | RIMS2 |  |  | 7374 | 0.084 | 0.4893 | No |
| 9 | LAMA2 |  |  | 8477 | 0.065 | 0.4506 | No |
| 10 | PIP3-E |  |  | 9604 | 0.048 | 0.4068 | No |
| 11 | HMGCS2 |  |  | 11183 | 0.025 | 0.3359 | No |
| 12 | IGF2 |  |  | 11640 | 0.019 | 0.3180 | No |
| 13 | ODZ2 |  |  | 12718 | 0.003 | 0.2664 | No |
| 14 | DLX4 |  |  | 14839 | -0.029 | 0.1701 | No |
| 15 | GATS |  |  | 17110 | -0.073 | 0.0764 | No |
| 16 | YKT6 |  |  | 20429 | -0.409 | 0.0085 | No |
Table: GSEA details [plain text format]

  

Fig 2: TSADAC\_RKOEXP\_UP: Random ES distribution      
 Gene set null distribution of ES for **TSADAC\_RKOEXP\_UP**
