# Supplementary material for: The rapamycin-regulated gene expression signature determines prognosis for breast cancer
Source: Mol Cancer. 2009 Sep 24;8:75. doi: 10.1186/1476-4598-8-75 (PMC2761377; doi:10.1186/1476-4598-8-75)
Supplement: Additional file 2 — Gene set enrichment analysis of in vivo data, time series. The data provided represent the time series of GSEA. This compressed file contains "Time" shortcut file and "GSEA_time" folder. Clicking on "Time" shortcut opens the index file providing access to analysis files contained in the "GSEA_time" folder. [file 1476-4598-8-75-S2.zip › GSEA_time/TSA_CD4_UP.html]

Details for gene set TSA\_CD4\_UP[GSEA]

|  || Dataset | gsea\_time\_collapsed |
| Phenotype | NoPhenotypeAvailable |
| Upregulated in class | na\_neg |
| GeneSet | TSA\_CD4\_UP |
| Enrichment Score (ES) | -0.38433462 |
| Normalized Enrichment Score (NES) | -1.3382279 |
| Nominal p-value | 0.07638889 |
| FDR q-value | 0.29505152 |
| FWER p-Value | 1.0 |
Table: GSEA Results Summary

  

Fig 1: Enrichment plot: TSA\_CD4\_UP      
 Profile of the Running ES Score & Positions of GeneSet Members on the Rank Ordered List

  

| PROBE | GENE SYMBOL | GENE\_TITLE | RANK IN GENE LIST | RANK METRIC SCORE | RUNNING ES | CORE ENRICHMENT || 1 | PSMB4 |  |  | 611 | 0.495 | 0.0817 | No |
| 2 | HSPA1B |  |  | 777 | 0.454 | 0.1759 | No |
| 3 | HMGN1 |  |  | 3995 | 0.174 | 0.0587 | No |
| 4 | SSR4 |  |  | 4058 | 0.171 | 0.0941 | No |
| 5 | CD6 |  |  | 4627 | 0.150 | 0.1002 | No |
| 6 | ENO2 |  |  | 5094 | 0.135 | 0.1079 | No |
| 7 | RARA |  |  | 5219 | 0.131 | 0.1314 | No |
| 8 | ITGB4BP |  |  | 5583 | 0.122 | 0.1413 | No |
| 9 | MYC |  |  | 6440 | 0.103 | 0.1229 | No |
| 10 | EIF4A1 |  |  | 7154 | 0.088 | 0.1081 | No |
| 11 | TP53 |  |  | 8842 | 0.060 | 0.0396 | No |
| 12 | SELPLG |  |  | 10338 | 0.037 | -0.0247 | No |
| 13 | USF2 |  |  | 12416 | 0.008 | -0.1239 | No |
| 14 | CD2 |  |  | 13728 | -0.012 | -0.1850 | No |
| 15 | COX8A |  |  | 14409 | -0.022 | -0.2131 | No |
| 16 | TGFB1 |  |  | 15318 | -0.037 | -0.2490 | No |
| 17 | PA2G4 |  |  | 15688 | -0.043 | -0.2573 | No |
| 18 | IGFBP4 |  |  | 17975 | -0.098 | -0.3464 | No |
| 19 | BYSL |  |  | 18494 | -0.117 | -0.3451 | No |
| 20 | FKBP4 |  |  | 19302 | -0.161 | -0.3482 | Yes |
| 21 | MIF |  |  | 19823 | -0.215 | -0.3251 | Yes |
| 22 | YWHAH |  |  | 20007 | -0.242 | -0.2794 | Yes |
| 23 | HSP90AB1 |  |  | 20186 | -0.290 | -0.2227 | Yes |
| 24 | HSPA1A |  |  | 20462 | -0.438 | -0.1375 | Yes |
| 25 | CD82 |  |  | 20576 | -0.642 | 0.0014 | Yes |
Table: GSEA details [plain text format]

  

Fig 2: TSA\_CD4\_UP: Random ES distribution      
 Gene set null distribution of ES for **TSA\_CD4\_UP**
