# Supplementary material for: The rapamycin-regulated gene expression signature determines prognosis for breast cancer
Source: Mol Cancer. 2009 Sep 24;8:75. doi: 10.1186/1476-4598-8-75 (PMC2761377; doi:10.1186/1476-4598-8-75)
Supplement: Additional file 2 — Gene set enrichment analysis of in vivo data, time series. The data provided represent the time series of GSEA. This compressed file contains "Time" shortcut file and "GSEA_time" folder. Clicking on "Time" shortcut opens the index file providing access to analysis files contained in the "GSEA_time" folder. [file 1476-4598-8-75-S2.zip › GSEA_time/TSA_HEPATOMA_CANCER_DN.html]

Details for gene set TSA\_HEPATOMA\_CANCER\_DN[GSEA]

|  || Dataset | gsea\_time\_collapsed |
| Phenotype | NoPhenotypeAvailable |
| Upregulated in class | na\_pos |
| GeneSet | TSA\_HEPATOMA\_CANCER\_DN |
| Enrichment Score (ES) | 0.7921782 |
| Normalized Enrichment Score (NES) | 1.7847999 |
| Nominal p-value | 0.0 |
| FDR q-value | 0.008337205 |
| FWER p-Value | 0.239 |
Table: GSEA Results Summary

  

Fig 1: Enrichment plot: TSA\_HEPATOMA\_CANCER\_DN      
 Profile of the Running ES Score & Positions of GeneSet Members on the Rank Ordered List

  

| PROBE | GENE SYMBOL | GENE\_TITLE | RANK IN GENE LIST | RANK METRIC SCORE | RUNNING ES | CORE ENRICHMENT || 1 | IL8 |  |  | 13 | 1.561 | 0.2624 | Yes |
| 2 | EGFR |  |  | 42 | 1.173 | 0.4587 | Yes |
| 3 | TGFB2 |  |  | 630 | 0.490 | 0.5127 | Yes |
| 4 | HNRPH1 |  |  | 670 | 0.480 | 0.5917 | Yes |
| 5 | ITGAV |  |  | 940 | 0.416 | 0.6488 | Yes |
| 6 | ADAM9 |  |  | 1582 | 0.320 | 0.6715 | Yes |
| 7 | GRB10 |  |  | 1618 | 0.316 | 0.7231 | Yes |
| 8 | TPR |  |  | 2389 | 0.256 | 0.7288 | Yes |
| 9 | MAPK6 |  |  | 2603 | 0.241 | 0.7591 | Yes |
| 10 | PTK2 |  |  | 2735 | 0.234 | 0.7922 | Yes |
| 11 | MAN1A2 |  |  | 4934 | 0.139 | 0.7089 | No |
| 12 | ROCK1 |  |  | 5834 | 0.116 | 0.6849 | No |
| 13 | CEACAM1 |  |  | 7267 | 0.086 | 0.6298 | No |
| 14 | LIG3 |  |  | 9620 | 0.048 | 0.5236 | No |
| 15 | MDM2 |  |  | 10709 | 0.032 | 0.4761 | No |
| 16 | TP53BP2 |  |  | 11080 | 0.026 | 0.4626 | No |
Table: GSEA details [plain text format]

  

Fig 2: TSA\_HEPATOMA\_CANCER\_DN: Random ES distribution      
 Gene set null distribution of ES for **TSA\_HEPATOMA\_CANCER\_DN**
