# Supplementary material for: The rapamycin-regulated gene expression signature determines prognosis for breast cancer
Source: Mol Cancer. 2009 Sep 24;8:75. doi: 10.1186/1476-4598-8-75 (PMC2761377; doi:10.1186/1476-4598-8-75)
Supplement: Additional file 2 — Gene set enrichment analysis of in vivo data, time series. The data provided represent the time series of GSEA. This compressed file contains "Time" shortcut file and "GSEA_time" folder. Clicking on "Time" shortcut opens the index file providing access to analysis files contained in the "GSEA_time" folder. [file 1476-4598-8-75-S2.zip › GSEA_time/TSA_HEPATOMA_CANCER_UP.html]

Details for gene set TSA\_HEPATOMA\_CANCER\_UP[GSEA]

|  || Dataset | gsea\_time\_collapsed |
| Phenotype | NoPhenotypeAvailable |
| Upregulated in class | na\_neg |
| GeneSet | TSA\_HEPATOMA\_CANCER\_UP |
| Enrichment Score (ES) | -0.37360376 |
| Normalized Enrichment Score (NES) | -1.3863266 |
| Nominal p-value | 0.04255319 |
| FDR q-value | 0.2956085 |
| FWER p-Value | 1.0 |
Table: GSEA Results Summary

  

Fig 1: Enrichment plot: TSA\_HEPATOMA\_CANCER\_UP      
 Profile of the Running ES Score & Positions of GeneSet Members on the Rank Ordered List

  

| PROBE | GENE SYMBOL | GENE\_TITLE | RANK IN GENE LIST | RANK METRIC SCORE | RUNNING ES | CORE ENRICHMENT || 1 | ARHGDIB |  |  | 251 | 0.681 | 0.1007 | No |
| 2 | CDKN2C |  |  | 1578 | 0.320 | 0.0893 | No |
| 3 | KRT7 |  |  | 2115 | 0.276 | 0.1089 | No |
| 4 | CTGF |  |  | 3167 | 0.211 | 0.0928 | No |
| 5 | CAV2 |  |  | 3683 | 0.188 | 0.0988 | No |
| 6 | EFNB3 |  |  | 4018 | 0.173 | 0.1113 | No |
| 7 | NGFR |  |  | 5494 | 0.124 | 0.0601 | No |
| 8 | TP53I11 |  |  | 6780 | 0.095 | 0.0134 | No |
| 9 | CD36 |  |  | 7019 | 0.091 | 0.0169 | No |
| 10 | VTN |  |  | 7071 | 0.090 | 0.0294 | No |
| 11 | ANK1 |  |  | 7864 | 0.075 | 0.0033 | No |
| 12 | PLA2G6 |  |  | 7961 | 0.073 | 0.0107 | No |
| 13 | CD9 |  |  | 8510 | 0.064 | -0.0052 | No |
| 14 | HBEGF |  |  | 9871 | 0.044 | -0.0641 | No |
| 15 | MMP11 |  |  | 10419 | 0.036 | -0.0847 | No |
| 16 | CD8B |  |  | 11187 | 0.025 | -0.1179 | No |
| 17 | FBN2 |  |  | 11612 | 0.019 | -0.1353 | No |
| 18 | BAD |  |  | 13424 | -0.008 | -0.2221 | No |
| 19 | PDGFRB |  |  | 13560 | -0.010 | -0.2270 | No |
| 20 | JUNB |  |  | 13719 | -0.012 | -0.2328 | No |
| 21 | MMP19 |  |  | 13884 | -0.014 | -0.2385 | No |
| 22 | TIMP2 |  |  | 13940 | -0.015 | -0.2387 | No |
| 23 | ITGA7 |  |  | 14818 | -0.029 | -0.2765 | No |
| 24 | TGFB1 |  |  | 15318 | -0.037 | -0.2947 | No |
| 25 | GLRX |  |  | 15511 | -0.040 | -0.2974 | No |
| 26 | CASP9 |  |  | 15764 | -0.044 | -0.3024 | No |
| 27 | COL1A2 |  |  | 16667 | -0.063 | -0.3359 | No |
| 28 | MMP13 |  |  | 16772 | -0.065 | -0.3302 | No |
| 29 | TRIP6 |  |  | 16807 | -0.065 | -0.3210 | No |
| 30 | LUM |  |  | 17751 | -0.091 | -0.3517 | Yes |
| 31 | FASTK |  |  | 18079 | -0.101 | -0.3508 | Yes |
| 32 | VEGFC |  |  | 18210 | -0.106 | -0.3395 | Yes |
| 33 | ITGA3 |  |  | 18758 | -0.129 | -0.3448 | Yes |
| 34 | FOSB |  |  | 18812 | -0.131 | -0.3256 | Yes |
| 35 | QSCN6 |  |  | 19801 | -0.212 | -0.3384 | Yes |
| 36 | JUP |  |  | 20316 | -0.341 | -0.3069 | Yes |
| 37 | RHOG |  |  | 20391 | -0.378 | -0.2479 | Yes |
| 38 | BSG |  |  | 20511 | -0.492 | -0.1722 | Yes |
| 39 | IGFBP2 |  |  | 20605 | -1.066 | -0.0000 | Yes |
Table: GSEA details [plain text format]

  

Fig 2: TSA\_HEPATOMA\_CANCER\_UP: Random ES distribution      
 Gene set null distribution of ES for **TSA\_HEPATOMA\_CANCER\_UP**
