# Supplementary material for: The rapamycin-regulated gene expression signature determines prognosis for breast cancer
Source: Mol Cancer. 2009 Sep 24;8:75. doi: 10.1186/1476-4598-8-75 (PMC2761377; doi:10.1186/1476-4598-8-75)
Supplement: Additional file 2 — Gene set enrichment analysis of in vivo data, time series. The data provided represent the time series of GSEA. This compressed file contains "Time" shortcut file and "GSEA_time" folder. Clicking on "Time" shortcut opens the index file providing access to analysis files contained in the "GSEA_time" folder. [file 1476-4598-8-75-S2.zip › GSEA_time/UV-CMV_UNIQUE_HCMV_6HRS_DN.html]

Details for gene set UV-CMV\_UNIQUE\_HCMV\_6HRS\_DN[GSEA]

|  || Dataset | gsea\_time\_collapsed |
| Phenotype | NoPhenotypeAvailable |
| Upregulated in class | na\_pos |
| GeneSet | UV-CMV\_UNIQUE\_HCMV\_6HRS\_DN |
| Enrichment Score (ES) | 0.6346756 |
| Normalized Enrichment Score (NES) | 1.8177636 |
| Nominal p-value | 0.0 |
| FDR q-value | 0.004869252 |
| FWER p-Value | 0.124 |
Table: GSEA Results Summary

  

Fig 1: Enrichment plot: UV-CMV\_UNIQUE\_HCMV\_6HRS\_DN      
 Profile of the Running ES Score & Positions of GeneSet Members on the Rank Ordered List

  

| PROBE | GENE SYMBOL | GENE\_TITLE | RANK IN GENE LIST | RANK METRIC SCORE | RUNNING ES | CORE ENRICHMENT || 1 | OLFM4 |  |  | 0 | 2.965 | 0.1594 | Yes |
| 2 | RARRES1 |  |  | 113 | 0.880 | 0.2012 | Yes |
| 3 | GPRC5A |  |  | 151 | 0.800 | 0.2424 | Yes |
| 4 | TCF7L2 |  |  | 246 | 0.684 | 0.2747 | Yes |
| 5 | C6ORF111 |  |  | 348 | 0.608 | 0.3024 | Yes |
| 6 | CDC42EP3 |  |  | 350 | 0.608 | 0.3351 | Yes |
| 7 | ZF |  |  | 351 | 0.608 | 0.3678 | Yes |
| 8 | SOCS2 |  |  | 404 | 0.579 | 0.3964 | Yes |
| 9 | TRIB2 |  |  | 573 | 0.507 | 0.4154 | Yes |
| 10 | ZNF292 |  |  | 582 | 0.505 | 0.4422 | Yes |
| 11 | SR140 |  |  | 698 | 0.475 | 0.4621 | Yes |
| 12 | COL13A1 |  |  | 733 | 0.466 | 0.4855 | Yes |
| 13 | RUNX1 |  |  | 822 | 0.441 | 0.5049 | Yes |
| 14 | NUP210 |  |  | 847 | 0.434 | 0.5271 | Yes |
| 15 | MTUS1 |  |  | 1289 | 0.356 | 0.5247 | Yes |
| 16 | NCK1 |  |  | 1399 | 0.341 | 0.5378 | Yes |
| 17 | DYRK2 |  |  | 1584 | 0.320 | 0.5460 | Yes |
| 18 | EPM2AIP1 |  |  | 1720 | 0.306 | 0.5559 | Yes |
| 19 | CDKN1B |  |  | 1916 | 0.291 | 0.5620 | Yes |
| 20 | BRWD1 |  |  | 2091 | 0.277 | 0.5684 | Yes |
| 21 | ARL4C |  |  | 2355 | 0.258 | 0.5695 | Yes |
| 22 | RANBP6 |  |  | 2359 | 0.258 | 0.5832 | Yes |
| 23 | KLF7 |  |  | 2569 | 0.244 | 0.5861 | Yes |
| 24 | NR2C1 |  |  | 2743 | 0.234 | 0.5902 | Yes |
| 25 | SMAD3 |  |  | 2926 | 0.223 | 0.5934 | Yes |
| 26 | CRSP9 |  |  | 3052 | 0.216 | 0.5989 | Yes |
| 27 | MYO10 |  |  | 3066 | 0.215 | 0.6099 | Yes |
| 28 | CAMK2G |  |  | 3156 | 0.211 | 0.6169 | Yes |
| 29 | DUSP10 |  |  | 3267 | 0.206 | 0.6226 | Yes |
| 30 | CSTF1 |  |  | 3286 | 0.204 | 0.6327 | Yes |
| 31 | MGMT |  |  | 3512 | 0.193 | 0.6321 | Yes |
| 32 | COIL |  |  | 3668 | 0.188 | 0.6347 | Yes |
| 33 | KIRREL |  |  | 4262 | 0.163 | 0.6145 | No |
| 34 | SOS2 |  |  | 4297 | 0.161 | 0.6215 | No |
| 35 | KCNJ2 |  |  | 4377 | 0.158 | 0.6262 | No |
| 36 | ERCC5 |  |  | 4609 | 0.150 | 0.6230 | No |
| 37 | PIM1 |  |  | 5180 | 0.132 | 0.6023 | No |
| 38 | KIAA0376 |  |  | 5351 | 0.128 | 0.6009 | No |
| 39 | MATN2 |  |  | 5576 | 0.122 | 0.5966 | No |
| 40 | ZNF148 |  |  | 5767 | 0.118 | 0.5937 | No |
| 41 | PPYR1 |  |  | 6354 | 0.104 | 0.5707 | No |
| 42 | MYC |  |  | 6440 | 0.103 | 0.5721 | No |
| 43 | FPRL1 |  |  | 6723 | 0.097 | 0.5636 | No |
| 44 | PRR3 |  |  | 6847 | 0.094 | 0.5627 | No |
| 45 | EMG1 |  |  | 7609 | 0.080 | 0.5299 | No |
| 46 | GLI3 |  |  | 7763 | 0.076 | 0.5265 | No |
| 47 | ITPKB |  |  | 8720 | 0.062 | 0.4833 | No |
| 48 | DKFZP564O0823 |  |  | 8818 | 0.060 | 0.4818 | No |
| 49 | ASB1 |  |  | 9159 | 0.054 | 0.4681 | No |
| 50 | VPS13D |  |  | 9389 | 0.051 | 0.4597 | No |
| 51 | KIAA1462 |  |  | 9757 | 0.046 | 0.4443 | No |
| 52 | FGF7 |  |  | 10279 | 0.038 | 0.4209 | No |
| 53 | AURKA |  |  | 10284 | 0.038 | 0.4228 | No |
| 54 | ZNF205 |  |  | 10657 | 0.032 | 0.4064 | No |
| 55 | ADORA2B |  |  | 10853 | 0.029 | 0.3984 | No |
| 56 | ETFB |  |  | 11137 | 0.026 | 0.3861 | No |
| 57 | ASNS |  |  | 11419 | 0.022 | 0.3735 | No |
| 58 | DOK1 |  |  | 11709 | 0.018 | 0.3604 | No |
| 59 | C14ORF132 |  |  | 11783 | 0.017 | 0.3577 | No |
| 60 | NR1D2 |  |  | 11988 | 0.014 | 0.3486 | No |
| 61 | HARSL |  |  | 12248 | 0.011 | 0.3365 | No |
| 62 | HOXA9 |  |  | 12432 | 0.008 | 0.3280 | No |
| 63 | HSPB3 |  |  | 12451 | 0.007 | 0.3275 | No |
| 64 | PTPRS |  |  | 12560 | 0.005 | 0.3225 | No |
| 65 | LRRC17 |  |  | 12808 | 0.001 | 0.3106 | No |
| 66 | FRMPD4 |  |  | 12844 | 0.001 | 0.3089 | No |
| 67 | ZNF217 |  |  | 14331 | -0.021 | 0.2377 | No |
| 68 | HSPB6 |  |  | 14612 | -0.025 | 0.2254 | No |
| 69 | COLEC10 |  |  | 14769 | -0.028 | 0.2193 | No |
| 70 | ZNF187 |  |  | 14926 | -0.030 | 0.2133 | No |
| 71 | PIWIL1 |  |  | 15527 | -0.040 | 0.1862 | No |
| 72 | NFATC4 |  |  | 15760 | -0.044 | 0.1773 | No |
| 73 | RNF144 |  |  | 16165 | -0.052 | 0.1604 | No |
| 74 | C16ORF51 |  |  | 16268 | -0.054 | 0.1583 | No |
| 75 | PIK3R4 |  |  | 16535 | -0.060 | 0.1486 | No |
| 76 | JARID1B |  |  | 16557 | -0.060 | 0.1508 | No |
| 77 | BCL7A |  |  | 17625 | -0.088 | 0.1035 | No |
| 78 | ARPC1A |  |  | 17798 | -0.092 | 0.1001 | No |
| 79 | ABCA6 |  |  | 18099 | -0.102 | 0.0910 | No |
| 80 | CDC25B |  |  | 18779 | -0.130 | 0.0649 | No |
| 81 | RRS1 |  |  | 19234 | -0.156 | 0.0511 | No |
| 82 | NR1H3 |  |  | 20189 | -0.291 | 0.0203 | No |
Table: GSEA details [plain text format]

  

Fig 2: UV-CMV\_UNIQUE\_HCMV\_6HRS\_DN: Random ES distribution      
 Gene set null distribution of ES for **UV-CMV\_UNIQUE\_HCMV\_6HRS\_DN**
