# Supplementary material for: The rapamycin-regulated gene expression signature determines prognosis for breast cancer
Source: Mol Cancer. 2009 Sep 24;8:75. doi: 10.1186/1476-4598-8-75 (PMC2761377; doi:10.1186/1476-4598-8-75)
Supplement: Additional file 2 — Gene set enrichment analysis of in vivo data, time series. The data provided represent the time series of GSEA. This compressed file contains "Time" shortcut file and "GSEA_time" folder. Clicking on "Time" shortcut opens the index file providing access to analysis files contained in the "GSEA_time" folder. [file 1476-4598-8-75-S2.zip › GSEA_time/UVB_NHEK1_C6.html]

Details for gene set UVB\_NHEK1\_C6[GSEA]

|  || Dataset | gsea\_time\_collapsed |
| Phenotype | NoPhenotypeAvailable |
| Upregulated in class | na\_pos |
| GeneSet | UVB\_NHEK1\_C6 |
| Enrichment Score (ES) | 0.58971375 |
| Normalized Enrichment Score (NES) | 1.7561975 |
| Nominal p-value | 0.0 |
| FDR q-value | 0.011183434 |
| FWER p-Value | 0.359 |
Table: GSEA Results Summary

  

Fig 1: Enrichment plot: UVB\_NHEK1\_C6      
 Profile of the Running ES Score & Positions of GeneSet Members on the Rank Ordered List

  

| PROBE | GENE SYMBOL | GENE\_TITLE | RANK IN GENE LIST | RANK METRIC SCORE | RUNNING ES | CORE ENRICHMENT || 1 | PLEKHC1 |  |  | 129 | 0.852 | 0.0254 | Yes |
| 2 | VPS13B |  |  | 331 | 0.620 | 0.0386 | Yes |
| 3 | MICAL2 |  |  | 374 | 0.595 | 0.0587 | Yes |
| 4 | TBC1D8 |  |  | 553 | 0.514 | 0.0691 | Yes |
| 5 | LPHN2 |  |  | 554 | 0.514 | 0.0882 | Yes |
| 6 | SLC7A1 |  |  | 570 | 0.508 | 0.1063 | Yes |
| 7 | RBPSUH |  |  | 585 | 0.504 | 0.1244 | Yes |
| 8 | SLC25A36 |  |  | 653 | 0.484 | 0.1391 | Yes |
| 9 | SP3 |  |  | 746 | 0.463 | 0.1518 | Yes |
| 10 | FAT |  |  | 757 | 0.461 | 0.1684 | Yes |
| 11 | SMC5 |  |  | 859 | 0.431 | 0.1795 | Yes |
| 12 | GNE |  |  | 890 | 0.425 | 0.1938 | Yes |
| 13 | TUBGCP3 |  |  | 913 | 0.420 | 0.2084 | Yes |
| 14 | BIRC2 |  |  | 1020 | 0.402 | 0.2181 | Yes |
| 15 | IRAK1BP1 |  |  | 1065 | 0.394 | 0.2306 | Yes |
| 16 | WWC1 |  |  | 1244 | 0.363 | 0.2354 | Yes |
| 17 | CNOT2 |  |  | 1286 | 0.356 | 0.2466 | Yes |
| 18 | MTUS1 |  |  | 1289 | 0.356 | 0.2598 | Yes |
| 19 | FNBP1L |  |  | 1411 | 0.340 | 0.2665 | Yes |
| 20 | SMC4 |  |  | 1414 | 0.340 | 0.2790 | Yes |
| 21 | HMGA2 |  |  | 1443 | 0.336 | 0.2901 | Yes |
| 22 | RNF13 |  |  | 1483 | 0.331 | 0.3005 | Yes |
| 23 | FGFR2 |  |  | 1536 | 0.325 | 0.3101 | Yes |
| 24 | MAST4 |  |  | 1580 | 0.320 | 0.3199 | Yes |
| 25 | PAFAH1B1 |  |  | 1731 | 0.305 | 0.3239 | Yes |
| 26 | TLK1 |  |  | 1757 | 0.303 | 0.3339 | Yes |
| 27 | ATP2B1 |  |  | 1819 | 0.298 | 0.3420 | Yes |
| 28 | EIF2C2 |  |  | 1822 | 0.298 | 0.3530 | Yes |
| 29 | TLE4 |  |  | 1921 | 0.290 | 0.3590 | Yes |
| 30 | ZZZ3 |  |  | 1926 | 0.290 | 0.3696 | Yes |
| 31 | DOPEY1 |  |  | 1945 | 0.289 | 0.3795 | Yes |
| 32 | ATF2 |  |  | 1949 | 0.289 | 0.3901 | Yes |
| 33 | INSIG2 |  |  | 2070 | 0.278 | 0.3945 | Yes |
| 34 | RABGAP1L |  |  | 2088 | 0.277 | 0.4040 | Yes |
| 35 | APPBP2 |  |  | 2120 | 0.275 | 0.4127 | Yes |
| 36 | CEBPD |  |  | 2123 | 0.275 | 0.4228 | Yes |
| 37 | MLH3 |  |  | 2144 | 0.273 | 0.4320 | Yes |
| 38 | ATP13A3 |  |  | 2146 | 0.273 | 0.4421 | Yes |
| 39 | LSM14A |  |  | 2301 | 0.261 | 0.4443 | Yes |
| 40 | CDYL |  |  | 2316 | 0.261 | 0.4533 | Yes |
| 41 | CLASP1 |  |  | 2407 | 0.254 | 0.4583 | Yes |
| 42 | ZCCHC11 |  |  | 2492 | 0.248 | 0.4635 | Yes |
| 43 | FOXO1A |  |  | 2551 | 0.245 | 0.4697 | Yes |
| 44 | KLF7 |  |  | 2569 | 0.244 | 0.4780 | Yes |
| 45 | PTK2 |  |  | 2735 | 0.234 | 0.4786 | Yes |
| 46 | KLHL9 |  |  | 2849 | 0.227 | 0.4815 | Yes |
| 47 | HDHD1A |  |  | 2864 | 0.227 | 0.4893 | Yes |
| 48 | SMAD3 |  |  | 2926 | 0.223 | 0.4946 | Yes |
| 49 | ZNF638 |  |  | 2947 | 0.222 | 0.5019 | Yes |
| 50 | CUL1 |  |  | 2956 | 0.222 | 0.5097 | Yes |
| 51 | REV3L |  |  | 3016 | 0.219 | 0.5150 | Yes |
| 52 | IGF2BP3 |  |  | 3018 | 0.218 | 0.5230 | Yes |
| 53 | CREB3L2 |  |  | 3169 | 0.211 | 0.5235 | Yes |
| 54 | MAP3K5 |  |  | 3190 | 0.209 | 0.5303 | Yes |
| 55 | SSBP2 |  |  | 3203 | 0.209 | 0.5375 | Yes |
| 56 | TOP2A |  |  | 3342 | 0.202 | 0.5383 | Yes |
| 57 | NPAS2 |  |  | 3432 | 0.198 | 0.5413 | Yes |
| 58 | KIF14 |  |  | 3480 | 0.195 | 0.5462 | Yes |
| 59 | ORC2L |  |  | 3532 | 0.192 | 0.5509 | Yes |
| 60 | USP6 |  |  | 3542 | 0.192 | 0.5576 | Yes |
| 61 | DOCK4 |  |  | 3631 | 0.189 | 0.5603 | Yes |
| 62 | CTBP2 |  |  | 3652 | 0.189 | 0.5663 | Yes |
| 63 | FOXO3A |  |  | 3680 | 0.188 | 0.5720 | Yes |
| 64 | CENPC1 |  |  | 3842 | 0.181 | 0.5708 | Yes |
| 65 | MKI67 |  |  | 4043 | 0.172 | 0.5674 | Yes |
| 66 | FAT2 |  |  | 4277 | 0.162 | 0.5621 | Yes |
| 67 | CASP8 |  |  | 4348 | 0.159 | 0.5646 | Yes |
| 68 | ZNF146 |  |  | 4400 | 0.157 | 0.5679 | Yes |
| 69 | MEIS1 |  |  | 4440 | 0.156 | 0.5718 | Yes |
| 70 | TP73L |  |  | 4573 | 0.151 | 0.5710 | Yes |
| 71 | C1ORF9 |  |  | 4578 | 0.151 | 0.5764 | Yes |
| 72 | FNDC3A |  |  | 4710 | 0.147 | 0.5755 | Yes |
| 73 | POLD3 |  |  | 4752 | 0.145 | 0.5789 | Yes |
| 74 | BAZ1A |  |  | 4753 | 0.145 | 0.5843 | Yes |
| 75 | AYTL2 |  |  | 4865 | 0.142 | 0.5841 | Yes |
| 76 | ARHGEF7 |  |  | 4910 | 0.140 | 0.5872 | Yes |
| 77 | EDD1 |  |  | 5233 | 0.131 | 0.5764 | Yes |
| 78 | KIAA0802 |  |  | 5283 | 0.130 | 0.5788 | Yes |
| 79 | XPO1 |  |  | 5413 | 0.126 | 0.5772 | Yes |
| 80 | CCNB2 |  |  | 5447 | 0.125 | 0.5802 | Yes |
| 81 | PHLPP |  |  | 5510 | 0.124 | 0.5818 | Yes |
| 82 | ADCY9 |  |  | 5523 | 0.124 | 0.5858 | Yes |
| 83 | MECP2 |  |  | 5751 | 0.118 | 0.5791 | Yes |
| 84 | TLK2 |  |  | 5757 | 0.118 | 0.5832 | Yes |
| 85 | DOCK9 |  |  | 5923 | 0.114 | 0.5794 | Yes |
| 86 | CNOT4 |  |  | 5973 | 0.113 | 0.5812 | Yes |
| 87 | MYST3 |  |  | 5997 | 0.113 | 0.5843 | Yes |
| 88 | PIP5K3 |  |  | 6008 | 0.113 | 0.5880 | Yes |
| 89 | PPP1R12A |  |  | 6133 | 0.110 | 0.5860 | Yes |
| 90 | FYN |  |  | 6142 | 0.110 | 0.5897 | Yes |
| 91 | ZNF451 |  |  | 6300 | 0.105 | 0.5860 | No |
| 92 | NAV3 |  |  | 6515 | 0.101 | 0.5793 | No |
| 93 | AUTS2 |  |  | 6692 | 0.098 | 0.5743 | No |
| 94 | OTUD4 |  |  | 6746 | 0.096 | 0.5753 | No |
| 95 | MYST4 |  |  | 7079 | 0.089 | 0.5624 | No |
| 96 | LARP7 |  |  | 7569 | 0.081 | 0.5415 | No |
| 97 | NFKB1 |  |  | 7978 | 0.073 | 0.5243 | No |
| 98 | USP24 |  |  | 8116 | 0.071 | 0.5203 | No |
| 99 | PRKCBP1 |  |  | 8234 | 0.069 | 0.5171 | No |
| 100 | KIF2A |  |  | 8603 | 0.063 | 0.5015 | No |
| 101 | RAI17 |  |  | 8832 | 0.060 | 0.4925 | No |
| 102 | PIP5K2B |  |  | 9057 | 0.056 | 0.4837 | No |
| 103 | ZHX2 |  |  | 9402 | 0.051 | 0.4688 | No |
| 104 | PIK3C3 |  |  | 9468 | 0.050 | 0.4675 | No |
| 105 | FRYL |  |  | 9894 | 0.044 | 0.4484 | No |
| 106 | C10ORF137 |  |  | 10159 | 0.040 | 0.4369 | No |
| 107 | PHF3 |  |  | 10469 | 0.035 | 0.4232 | No |
| 108 | WAPAL |  |  | 10750 | 0.031 | 0.4106 | No |
| 109 | KIF23 |  |  | 10825 | 0.030 | 0.4081 | No |
| 110 | SHOC2 |  |  | 11644 | 0.019 | 0.3689 | No |
| 111 | IRS1 |  |  | 12296 | 0.010 | 0.3374 | No |
| 112 | E2F5 |  |  | 12422 | 0.008 | 0.3316 | No |
| 113 | AJAP1 |  |  | 13575 | -0.010 | 0.2757 | No |
| 114 | XPA |  |  | 13632 | -0.011 | 0.2734 | No |
| 115 | POLE2 |  |  | 13961 | -0.015 | 0.2579 | No |
| 116 | UBL3 |  |  | 14647 | -0.026 | 0.2254 | No |
| 117 | MEGF9 |  |  | 15529 | -0.040 | 0.1839 | No |
| 118 | KIF11 |  |  | 15959 | -0.048 | 0.1647 | No |
| 119 | KIAA0423 |  |  | 16104 | -0.050 | 0.1596 | No |
| 120 | MN1 |  |  | 16342 | -0.056 | 0.1501 | No |
| 121 | SLBP |  |  | 16438 | -0.058 | 0.1476 | No |
| 122 | SPOP |  |  | 16690 | -0.063 | 0.1377 | No |
| 123 | KIAA0179 |  |  | 16866 | -0.067 | 0.1316 | No |
| 124 | BCL7A |  |  | 17625 | -0.088 | 0.0978 | No |
| 125 | VEGFC |  |  | 18210 | -0.106 | 0.0733 | No |
| 126 | STRN |  |  | 18468 | -0.116 | 0.0650 | No |
| 127 | RRS1 |  |  | 19234 | -0.156 | 0.0335 | No |
| 128 | SLC9A6 |  |  | 19687 | -0.199 | 0.0188 | No |
| 129 | ABL1 |  |  | 20089 | -0.261 | 0.0089 | No |
| 130 | CENPA |  |  | 20463 | -0.438 | 0.0069 | No |
Table: GSEA details [plain text format]

  

Fig 2: UVB\_NHEK1\_C6: Random ES distribution      
 Gene set null distribution of ES for **UVB\_NHEK1\_C6**
