# Supplementary material for: The rapamycin-regulated gene expression signature determines prognosis for breast cancer
Source: Mol Cancer. 2009 Sep 24;8:75. doi: 10.1186/1476-4598-8-75 (PMC2761377; doi:10.1186/1476-4598-8-75)
Supplement: Additional file 2 — Gene set enrichment analysis of in vivo data, time series. The data provided represent the time series of GSEA. This compressed file contains "Time" shortcut file and "GSEA_time" folder. Clicking on "Time" shortcut opens the index file providing access to analysis files contained in the "GSEA_time" folder. [file 1476-4598-8-75-S2.zip › GSEA_time/UVB_NHEK3_C3.html]

Details for gene set UVB\_NHEK3\_C3[GSEA]

|  || Dataset | gsea\_time\_collapsed |
| Phenotype | NoPhenotypeAvailable |
| Upregulated in class | na\_pos |
| GeneSet | UVB\_NHEK3\_C3 |
| Enrichment Score (ES) | 0.7593501 |
| Normalized Enrichment Score (NES) | 1.7572204 |
| Nominal p-value | 0.0 |
| FDR q-value | 0.0113417115 |
| FWER p-Value | 0.357 |
Table: GSEA Results Summary

  

Fig 1: Enrichment plot: UVB\_NHEK3\_C3      
 Profile of the Running ES Score & Positions of GeneSet Members on the Rank Ordered List

  

| PROBE | GENE SYMBOL | GENE\_TITLE | RANK IN GENE LIST | RANK METRIC SCORE | RUNNING ES | CORE ENRICHMENT || 1 | IL8 |  |  | 13 | 1.561 | 0.2432 | Yes |
| 2 | SOX9 |  |  | 40 | 1.200 | 0.4293 | Yes |
| 3 | PHLDA2 |  |  | 68 | 0.996 | 0.5836 | Yes |
| 4 | DUSP1 |  |  | 672 | 0.479 | 0.6291 | Yes |
| 5 | PMAIP1 |  |  | 904 | 0.422 | 0.6838 | Yes |
| 6 | EIF2S1 |  |  | 1423 | 0.338 | 0.7114 | Yes |
| 7 | TAX1BP3 |  |  | 1496 | 0.329 | 0.7594 | Yes |
| 8 | ATF3 |  |  | 2567 | 0.244 | 0.7455 | No |
| 9 | RND3 |  |  | 3338 | 0.202 | 0.7397 | No |
| 10 | BRD2 |  |  | 4522 | 0.153 | 0.7062 | No |
| 11 | IER3 |  |  | 6090 | 0.111 | 0.6473 | No |
| 12 | H2AFX |  |  | 6183 | 0.109 | 0.6598 | No |
| 13 | HIST2H2AA3 |  |  | 8794 | 0.060 | 0.5424 | No |
| 14 | HBEGF |  |  | 9871 | 0.044 | 0.4970 | No |
| 15 | MYD88 |  |  | 14849 | -0.029 | 0.2599 | No |
| 16 | ATP6V0D1 |  |  | 16314 | -0.055 | 0.1974 | No |
| 17 | MAD2L1BP |  |  | 17006 | -0.070 | 0.1748 | No |
Table: GSEA details [plain text format]

  

Fig 2: UVB\_NHEK3\_C3: Random ES distribution      
 Gene set null distribution of ES for **UVB\_NHEK3\_C3**
