# Supplementary material for: The rapamycin-regulated gene expression signature determines prognosis for breast cancer
Source: Mol Cancer. 2009 Sep 24;8:75. doi: 10.1186/1476-4598-8-75 (PMC2761377; doi:10.1186/1476-4598-8-75)
Supplement: Additional file 2 — Gene set enrichment analysis of in vivo data, time series. The data provided represent the time series of GSEA. This compressed file contains "Time" shortcut file and "GSEA_time" folder. Clicking on "Time" shortcut opens the index file providing access to analysis files contained in the "GSEA_time" folder. [file 1476-4598-8-75-S2.zip › GSEA_time/UVB_SCC_UP.html]

Details for gene set UVB\_SCC\_UP[GSEA]

|  || Dataset | gsea\_time\_collapsed |
| Phenotype | NoPhenotypeAvailable |
| Upregulated in class | na\_pos |
| GeneSet | UVB\_SCC\_UP |
| Enrichment Score (ES) | 0.6272609 |
| Normalized Enrichment Score (NES) | 1.8184605 |
| Nominal p-value | 0.0 |
| FDR q-value | 0.0049796663 |
| FWER p-Value | 0.122 |
Table: GSEA Results Summary

  

Fig 1: Enrichment plot: UVB\_SCC\_UP      
 Profile of the Running ES Score & Positions of GeneSet Members on the Rank Ordered List

  

| PROBE | GENE SYMBOL | GENE\_TITLE | RANK IN GENE LIST | RANK METRIC SCORE | RUNNING ES | CORE ENRICHMENT || 1 | IGFBP7 |  |  | 2 | 2.165 | 0.1006 | Yes |
| 2 | PGK1 |  |  | 101 | 0.899 | 0.1376 | Yes |
| 3 | CXCL3 |  |  | 162 | 0.784 | 0.1711 | Yes |
| 4 | CXCL2 |  |  | 203 | 0.723 | 0.2027 | Yes |
| 5 | ARHGDIB |  |  | 251 | 0.681 | 0.2321 | Yes |
| 6 | EIF2S3 |  |  | 292 | 0.658 | 0.2607 | Yes |
| 7 | NAP1L1 |  |  | 548 | 0.515 | 0.2722 | Yes |
| 8 | LAMP2 |  |  | 564 | 0.511 | 0.2952 | Yes |
| 9 | ITM2B |  |  | 596 | 0.501 | 0.3170 | Yes |
| 10 | AP1S2 |  |  | 671 | 0.479 | 0.3357 | Yes |
| 11 | COPB1 |  |  | 677 | 0.478 | 0.3577 | Yes |
| 12 | CDKN1C |  |  | 693 | 0.475 | 0.3790 | Yes |
| 13 | DSG2 |  |  | 712 | 0.472 | 0.4001 | Yes |
| 14 | PMAIP1 |  |  | 904 | 0.422 | 0.4104 | Yes |
| 15 | SH3BGRL |  |  | 1033 | 0.400 | 0.4227 | Yes |
| 16 | TM2D1 |  |  | 1080 | 0.391 | 0.4387 | Yes |
| 17 | SEPT10 |  |  | 1081 | 0.391 | 0.4568 | Yes |
| 18 | UBE2D3 |  |  | 1103 | 0.386 | 0.4738 | Yes |
| 19 | RECQL |  |  | 1128 | 0.381 | 0.4903 | Yes |
| 20 | DYNLT3 |  |  | 1135 | 0.380 | 0.5077 | Yes |
| 21 | CDC2 |  |  | 1184 | 0.373 | 0.5227 | Yes |
| 22 | SSR1 |  |  | 1380 | 0.343 | 0.5292 | Yes |
| 23 | TOB1 |  |  | 1542 | 0.324 | 0.5364 | Yes |
| 24 | PLSCR1 |  |  | 1622 | 0.315 | 0.5472 | Yes |
| 25 | SFRS1 |  |  | 1728 | 0.306 | 0.5563 | Yes |
| 26 | SNX3 |  |  | 2022 | 0.282 | 0.5551 | Yes |
| 27 | C18ORF10 |  |  | 2052 | 0.279 | 0.5667 | Yes |
| 28 | ANXA2 |  |  | 2188 | 0.270 | 0.5727 | Yes |
| 29 | KYNU |  |  | 2325 | 0.260 | 0.5781 | Yes |
| 30 | RTN4 |  |  | 2463 | 0.250 | 0.5831 | Yes |
| 31 | PRKAR1A |  |  | 2467 | 0.250 | 0.5945 | Yes |
| 32 | ATF3 |  |  | 2567 | 0.244 | 0.6010 | Yes |
| 33 | SKP1A |  |  | 2693 | 0.236 | 0.6059 | Yes |
| 34 | CCBP2 |  |  | 2715 | 0.235 | 0.6158 | Yes |
| 35 | ME2 |  |  | 2889 | 0.225 | 0.6179 | Yes |
| 36 | PAPOLA |  |  | 3124 | 0.213 | 0.6163 | Yes |
| 37 | TAF2 |  |  | 3137 | 0.212 | 0.6256 | Yes |
| 38 | SRI |  |  | 3627 | 0.189 | 0.6106 | Yes |
| 39 | UQCRC2 |  |  | 3768 | 0.184 | 0.6123 | Yes |
| 40 | HRSP12 |  |  | 3810 | 0.182 | 0.6188 | Yes |
| 41 | UBE2E1 |  |  | 4073 | 0.171 | 0.6139 | Yes |
| 42 | AGL |  |  | 4218 | 0.164 | 0.6145 | Yes |
| 43 | PTK9 |  |  | 4258 | 0.163 | 0.6202 | Yes |
| 44 | RBMXL1 |  |  | 4416 | 0.157 | 0.6198 | Yes |
| 45 | TACSTD2 |  |  | 4511 | 0.154 | 0.6224 | Yes |
| 46 | SCYE1 |  |  | 4557 | 0.152 | 0.6273 | Yes |
| 47 | IL6 |  |  | 4954 | 0.138 | 0.6144 | No |
| 48 | SCFD1 |  |  | 5161 | 0.133 | 0.6105 | No |
| 49 | ATP5A1 |  |  | 5563 | 0.123 | 0.5967 | No |
| 50 | B2M |  |  | 5723 | 0.119 | 0.5945 | No |
| 51 | JUND |  |  | 6231 | 0.108 | 0.5748 | No |
| 52 | HEXIM1 |  |  | 6330 | 0.105 | 0.5749 | No |
| 53 | CCNC |  |  | 6337 | 0.105 | 0.5794 | No |
| 54 | RFC5 |  |  | 6565 | 0.101 | 0.5730 | No |
| 55 | RBBP4 |  |  | 7022 | 0.091 | 0.5550 | No |
| 56 | ADK |  |  | 7469 | 0.083 | 0.5372 | No |
| 57 | SLC11A1 |  |  | 7696 | 0.078 | 0.5298 | No |
| 58 | HPRT1 |  |  | 7814 | 0.076 | 0.5276 | No |
| 59 | ALDH7A1 |  |  | 7822 | 0.076 | 0.5308 | No |
| 60 | GADD45A |  |  | 8579 | 0.063 | 0.4969 | No |
| 61 | DYNC2LI1 |  |  | 8698 | 0.062 | 0.4940 | No |
| 62 | RRM2 |  |  | 9889 | 0.044 | 0.4380 | No |
| 63 | ID2 |  |  | 10102 | 0.040 | 0.4296 | No |
| 64 | YWHAZ |  |  | 10493 | 0.035 | 0.4122 | No |
| 65 | AP4S1 |  |  | 10674 | 0.032 | 0.4049 | No |
| 66 | CITED2 |  |  | 10789 | 0.030 | 0.4007 | No |
| 67 | SCARB2 |  |  | 11717 | 0.017 | 0.3564 | No |
| 68 | ANXA3 |  |  | 11767 | 0.017 | 0.3548 | No |
| 69 | IL13RA1 |  |  | 12637 | 0.004 | 0.3126 | No |
| 70 | CCL20 |  |  | 12960 | -0.000 | 0.2969 | No |
| 71 | TAGLN3 |  |  | 13370 | -0.007 | 0.2773 | No |
| 72 | PFDN6 |  |  | 14291 | -0.020 | 0.2334 | No |
| 73 | PDIA6 |  |  | 14486 | -0.023 | 0.2250 | No |
| 74 | PRDX3 |  |  | 15215 | -0.035 | 0.1912 | No |
| 75 | PTGES3 |  |  | 15739 | -0.044 | 0.1677 | No |
| 76 | MGC14376 |  |  | 15969 | -0.048 | 0.1588 | No |
| 77 | TSC22D3 |  |  | 16172 | -0.052 | 0.1514 | No |
| 78 | RALB |  |  | 16851 | -0.067 | 0.1214 | No |
| 79 | HIST1H2BK |  |  | 17029 | -0.071 | 0.1161 | No |
| 80 | ATP6AP2 |  |  | 17393 | -0.081 | 0.1021 | No |
| 81 | C11ORF51 |  |  | 17904 | -0.096 | 0.0817 | No |
| 82 | SDHD |  |  | 18044 | -0.101 | 0.0796 | No |
| 83 | RCN2 |  |  | 18671 | -0.125 | 0.0549 | No |
| 84 | RAD51C |  |  | 19206 | -0.154 | 0.0360 | No |
| 85 | MAP2K1 |  |  | 19255 | -0.157 | 0.0410 | No |
| 86 | PSMD8 |  |  | 19999 | -0.241 | 0.0160 | No |
| 87 | HSP90AB1 |  |  | 20186 | -0.290 | 0.0204 | No |
Table: GSEA details [plain text format]

  

Fig 2: UVB\_SCC\_UP: Random ES distribution      
 Gene set null distribution of ES for **UVB\_SCC\_UP**
