# Supplementary material for: The rapamycin-regulated gene expression signature determines prognosis for breast cancer
Source: Mol Cancer. 2009 Sep 24;8:75. doi: 10.1186/1476-4598-8-75 (PMC2761377; doi:10.1186/1476-4598-8-75)
Supplement: Additional file 2 — Gene set enrichment analysis of in vivo data, time series. The data provided represent the time series of GSEA. This compressed file contains "Time" shortcut file and "GSEA_time" folder. Clicking on "Time" shortcut opens the index file providing access to analysis files contained in the "GSEA_time" folder. [file 1476-4598-8-75-S2.zip › GSEA_time/UVC_HIGH_D4_DN.html]

Details for gene set UVC\_HIGH\_D4\_DN[GSEA]

|  || Dataset | gsea\_time\_collapsed |
| Phenotype | NoPhenotypeAvailable |
| Upregulated in class | na\_pos |
| GeneSet | UVC\_HIGH\_D4\_DN |
| Enrichment Score (ES) | 0.6501748 |
| Normalized Enrichment Score (NES) | 1.7559531 |
| Nominal p-value | 0.0 |
| FDR q-value | 0.010984282 |
| FWER p-Value | 0.361 |
Table: GSEA Results Summary

  

Fig 1: Enrichment plot: UVC\_HIGH\_D4\_DN      
 Profile of the Running ES Score & Positions of GeneSet Members on the Rank Ordered List

  

| PROBE | GENE SYMBOL | GENE\_TITLE | RANK IN GENE LIST | RANK METRIC SCORE | RUNNING ES | CORE ENRICHMENT || 1 | ARF6 |  |  | 55 | 1.045 | 0.0949 | Yes |
| 2 | CHSY1 |  |  | 295 | 0.652 | 0.1441 | Yes |
| 3 | SAMD4A |  |  | 298 | 0.650 | 0.2046 | Yes |
| 4 | SMURF2 |  |  | 373 | 0.596 | 0.2566 | Yes |
| 5 | THUMPD1 |  |  | 451 | 0.553 | 0.3044 | Yes |
| 6 | DDX17 |  |  | 778 | 0.454 | 0.3308 | Yes |
| 7 | GNE |  |  | 890 | 0.425 | 0.3651 | Yes |
| 8 | SEC24B |  |  | 895 | 0.424 | 0.4044 | Yes |
| 9 | FZD7 |  |  | 912 | 0.421 | 0.4429 | Yes |
| 10 | RAPGEF2 |  |  | 1129 | 0.381 | 0.4679 | Yes |
| 11 | HMGA2 |  |  | 1443 | 0.336 | 0.4840 | Yes |
| 12 | USP1 |  |  | 1566 | 0.321 | 0.5080 | Yes |
| 13 | DICER1 |  |  | 1679 | 0.310 | 0.5315 | Yes |
| 14 | SIAH1 |  |  | 1724 | 0.306 | 0.5579 | Yes |
| 15 | UBE2G1 |  |  | 1919 | 0.291 | 0.5756 | Yes |
| 16 | ATP13A3 |  |  | 2146 | 0.273 | 0.5901 | Yes |
| 17 | TPST1 |  |  | 2422 | 0.253 | 0.6004 | Yes |
| 18 | MAPK6 |  |  | 2603 | 0.241 | 0.6141 | Yes |
| 19 | POGZ |  |  | 2627 | 0.240 | 0.6354 | Yes |
| 20 | KRIT1 |  |  | 2841 | 0.228 | 0.6463 | Yes |
| 21 | CTGF |  |  | 3167 | 0.211 | 0.6502 | Yes |
| 22 | SPEN |  |  | 3757 | 0.184 | 0.6387 | No |
| 23 | DOC1 |  |  | 3895 | 0.178 | 0.6487 | No |
| 24 | PPP2R5C |  |  | 4755 | 0.145 | 0.6205 | No |
| 25 | RBM5 |  |  | 5119 | 0.135 | 0.6154 | No |
| 26 | PHC2 |  |  | 5302 | 0.129 | 0.6186 | No |
| 27 | SLC20A2 |  |  | 5400 | 0.126 | 0.6257 | No |
| 28 | XPO1 |  |  | 5413 | 0.126 | 0.6368 | No |
| 29 | ANKRD15 |  |  | 5803 | 0.117 | 0.6288 | No |
| 30 | ATP2A2 |  |  | 5878 | 0.115 | 0.6360 | No |
| 31 | UAP1 |  |  | 6117 | 0.110 | 0.6347 | No |
| 32 | DDX3X |  |  | 6332 | 0.105 | 0.6340 | No |
| 33 | ARID5B |  |  | 6935 | 0.092 | 0.6134 | No |
| 34 | CLINT1 |  |  | 8726 | 0.061 | 0.5320 | No |
| 35 | GATA2 |  |  | 8943 | 0.058 | 0.5269 | No |
| 36 | FHL5 |  |  | 8971 | 0.057 | 0.5309 | No |
| 37 | BMPR1A |  |  | 9101 | 0.055 | 0.5298 | No |
| 38 | AURKA |  |  | 10284 | 0.038 | 0.4758 | No |
| 39 | GNAI1 |  |  | 10557 | 0.034 | 0.4657 | No |
| 40 | PDCD10 |  |  | 11300 | 0.024 | 0.4318 | No |
| 41 | USP10 |  |  | 11999 | 0.014 | 0.3992 | No |
| 42 | BHLHB2 |  |  | 12438 | 0.008 | 0.3786 | No |
| 43 | RAB1A |  |  | 12978 | -0.001 | 0.3525 | No |
| 44 | RAD21 |  |  | 13230 | -0.004 | 0.3407 | No |
| 45 | HMGB2 |  |  | 13259 | -0.005 | 0.3398 | No |
| 46 | WDR57 |  |  | 19591 | -0.188 | 0.0493 | No |
Table: GSEA details [plain text format]

  

Fig 2: UVC\_HIGH\_D4\_DN: Random ES distribution      
 Gene set null distribution of ES for **UVC\_HIGH\_D4\_DN**
