# Supplementary material for: The rapamycin-regulated gene expression signature determines prognosis for breast cancer
Source: Mol Cancer. 2009 Sep 24;8:75. doi: 10.1186/1476-4598-8-75 (PMC2761377; doi:10.1186/1476-4598-8-75)
Supplement: Additional file 2 — Gene set enrichment analysis of in vivo data, time series. The data provided represent the time series of GSEA. This compressed file contains "Time" shortcut file and "GSEA_time" folder. Clicking on "Time" shortcut opens the index file providing access to analysis files contained in the "GSEA_time" folder. [file 1476-4598-8-75-S2.zip › GSEA_time/UVC_TTD_4HR_DN.html]

Details for gene set UVC\_TTD\_4HR\_DN[GSEA]

|  || Dataset | gsea\_time\_collapsed |
| Phenotype | NoPhenotypeAvailable |
| Upregulated in class | na\_pos |
| GeneSet | UVC\_TTD\_4HR\_DN |
| Enrichment Score (ES) | 0.6057611 |
| Normalized Enrichment Score (NES) | 1.8425989 |
| Nominal p-value | 0.0 |
| FDR q-value | 0.004180416 |
| FWER p-Value | 0.072 |
Table: GSEA Results Summary

  

Fig 1: Enrichment plot: UVC\_TTD\_4HR\_DN      
 Profile of the Running ES Score & Positions of GeneSet Members on the Rank Ordered List

  

| PROBE | GENE SYMBOL | GENE\_TITLE | RANK IN GENE LIST | RANK METRIC SCORE | RUNNING ES | CORE ENRICHMENT || 1 | EGFR |  |  | 42 | 1.173 | 0.0162 | Yes |
| 2 | OGT |  |  | 56 | 1.039 | 0.0317 | Yes |
| 3 | PLEKHC1 |  |  | 129 | 0.852 | 0.0414 | Yes |
| 4 | HNRPDL |  |  | 207 | 0.717 | 0.0488 | Yes |
| 5 | EXT1 |  |  | 230 | 0.695 | 0.0585 | Yes |
| 6 | TCF7L2 |  |  | 246 | 0.684 | 0.0684 | Yes |
| 7 | NIPBL |  |  | 268 | 0.673 | 0.0778 | Yes |
| 8 | SAMD4A |  |  | 298 | 0.650 | 0.0865 | Yes |
| 9 | WDR43 |  |  | 305 | 0.646 | 0.0963 | Yes |
| 10 | BMP2K |  |  | 316 | 0.634 | 0.1056 | Yes |
| 11 | UGCG |  |  | 335 | 0.617 | 0.1143 | Yes |
| 12 | DAPK1 |  |  | 343 | 0.610 | 0.1235 | Yes |
| 13 | KIAA1012 |  |  | 345 | 0.610 | 0.1329 | Yes |
| 14 | C6ORF111 |  |  | 348 | 0.608 | 0.1423 | Yes |
| 15 | F3 |  |  | 371 | 0.596 | 0.1505 | Yes |
| 16 | SMURF2 |  |  | 373 | 0.596 | 0.1597 | Yes |
| 17 | NEK7 |  |  | 386 | 0.591 | 0.1683 | Yes |
| 18 | MID1 |  |  | 400 | 0.582 | 0.1767 | Yes |
| 19 | BTAF1 |  |  | 523 | 0.523 | 0.1788 | Yes |
| 20 | SYNJ2 |  |  | 525 | 0.523 | 0.1869 | Yes |
| 21 | ZNF292 |  |  | 582 | 0.505 | 0.1920 | Yes |
| 22 | RBPSUH |  |  | 585 | 0.504 | 0.1998 | Yes |
| 23 | CEP350 |  |  | 626 | 0.491 | 0.2054 | Yes |
| 24 | TGFB2 |  |  | 630 | 0.490 | 0.2129 | Yes |
| 25 | SR140 |  |  | 698 | 0.475 | 0.2170 | Yes |
| 26 | ANKRD28 |  |  | 767 | 0.459 | 0.2208 | Yes |
| 27 | RUNX1 |  |  | 822 | 0.441 | 0.2250 | Yes |
| 28 | FOXJ3 |  |  | 828 | 0.439 | 0.2316 | Yes |
| 29 | FUBP1 |  |  | 830 | 0.438 | 0.2383 | Yes |
| 30 | SEC24B |  |  | 895 | 0.424 | 0.2418 | Yes |
| 31 | TUBGCP3 |  |  | 913 | 0.420 | 0.2475 | Yes |
| 32 | FCHSD2 |  |  | 925 | 0.418 | 0.2534 | Yes |
| 33 | HDAC4 |  |  | 935 | 0.417 | 0.2595 | Yes |
| 34 | R3HDM2 |  |  | 950 | 0.414 | 0.2652 | Yes |
| 35 | SOS1 |  |  | 991 | 0.407 | 0.2696 | Yes |
| 36 | BAT2D1 |  |  | 1014 | 0.402 | 0.2748 | Yes |
| 37 | GAPVD1 |  |  | 1019 | 0.402 | 0.2808 | Yes |
| 38 | BCAR3 |  |  | 1036 | 0.399 | 0.2862 | Yes |
| 39 | IRAK1BP1 |  |  | 1065 | 0.394 | 0.2910 | Yes |
| 40 | PKN2 |  |  | 1072 | 0.393 | 0.2968 | Yes |
| 41 | STRN3 |  |  | 1117 | 0.383 | 0.3006 | Yes |
| 42 | RAPGEF2 |  |  | 1129 | 0.381 | 0.3060 | Yes |
| 43 | CAMSAP1L1 |  |  | 1144 | 0.379 | 0.3112 | Yes |
| 44 | FXR1 |  |  | 1152 | 0.378 | 0.3167 | Yes |
| 45 | SCHIP1 |  |  | 1171 | 0.375 | 0.3217 | Yes |
| 46 | WWC1 |  |  | 1244 | 0.363 | 0.3238 | Yes |
| 47 | USP34 |  |  | 1252 | 0.363 | 0.3291 | Yes |
| 48 | EIF4E |  |  | 1263 | 0.361 | 0.3342 | Yes |
| 49 | SNX13 |  |  | 1265 | 0.361 | 0.3397 | Yes |
| 50 | CNOT2 |  |  | 1286 | 0.356 | 0.3443 | Yes |
| 51 | MYCBP2 |  |  | 1296 | 0.355 | 0.3494 | Yes |
| 52 | SOCS5 |  |  | 1350 | 0.347 | 0.3522 | Yes |
| 53 | KIAA0999 |  |  | 1374 | 0.344 | 0.3564 | Yes |
| 54 | LOC220594 |  |  | 1381 | 0.343 | 0.3614 | Yes |
| 55 | AFF1 |  |  | 1382 | 0.343 | 0.3668 | Yes |
| 56 | FNBP1L |  |  | 1411 | 0.340 | 0.3707 | Yes |
| 57 | LRP6 |  |  | 1447 | 0.336 | 0.3742 | Yes |
| 58 | E2F3 |  |  | 1473 | 0.332 | 0.3781 | Yes |
| 59 | RNF13 |  |  | 1483 | 0.331 | 0.3828 | Yes |
| 60 | ATXN1 |  |  | 1489 | 0.330 | 0.3877 | Yes |
| 61 | UST |  |  | 1491 | 0.330 | 0.3928 | Yes |
| 62 | ANKRD17 |  |  | 1508 | 0.328 | 0.3971 | Yes |
| 63 | MEIS2 |  |  | 1555 | 0.323 | 0.3999 | Yes |
| 64 | ARHGAP29 |  |  | 1593 | 0.319 | 0.4030 | Yes |
| 65 | MSH3 |  |  | 1620 | 0.316 | 0.4066 | Yes |
| 66 | SLC25A24 |  |  | 1730 | 0.305 | 0.4060 | Yes |
| 67 | PAFAH1B1 |  |  | 1731 | 0.305 | 0.4108 | Yes |
| 68 | ZMYM2 |  |  | 1746 | 0.304 | 0.4148 | Yes |
| 69 | TLK1 |  |  | 1757 | 0.303 | 0.4190 | Yes |
| 70 | ATP2B1 |  |  | 1819 | 0.298 | 0.4207 | Yes |
| 71 | TLE4 |  |  | 1921 | 0.290 | 0.4202 | Yes |
| 72 | ZZZ3 |  |  | 1926 | 0.290 | 0.4246 | Yes |
| 73 | CUTL1 |  |  | 1928 | 0.290 | 0.4290 | Yes |
| 74 | KIAA1026 |  |  | 1935 | 0.290 | 0.4332 | Yes |
| 75 | RAB3GAP1 |  |  | 1947 | 0.289 | 0.4372 | Yes |
| 76 | ATF2 |  |  | 1949 | 0.289 | 0.4416 | Yes |
| 77 | MTMR3 |  |  | 1997 | 0.284 | 0.4437 | Yes |
| 78 | MDN1 |  |  | 2001 | 0.284 | 0.4480 | Yes |
| 79 | LOC151162 |  |  | 2010 | 0.284 | 0.4520 | Yes |
| 80 | PARG |  |  | 2020 | 0.282 | 0.4560 | Yes |
| 81 | KLF10 |  |  | 2047 | 0.280 | 0.4590 | Yes |
| 82 | PCTK2 |  |  | 2050 | 0.279 | 0.4633 | Yes |
| 83 | TRIO |  |  | 2122 | 0.275 | 0.4641 | Yes |
| 84 | IL1RAP |  |  | 2143 | 0.273 | 0.4673 | Yes |
| 85 | ATP13A3 |  |  | 2146 | 0.273 | 0.4715 | Yes |
| 86 | SETD2 |  |  | 2166 | 0.271 | 0.4748 | Yes |
| 87 | VPS13A |  |  | 2172 | 0.271 | 0.4787 | Yes |
| 88 | JARID1A |  |  | 2179 | 0.270 | 0.4826 | Yes |
| 89 | PTPRK |  |  | 2191 | 0.270 | 0.4863 | Yes |
| 90 | SRGAP2 |  |  | 2229 | 0.266 | 0.4886 | Yes |
| 91 | CDYL |  |  | 2316 | 0.261 | 0.4884 | Yes |
| 92 | TRIM33 |  |  | 2405 | 0.255 | 0.4881 | Yes |
| 93 | CLASP1 |  |  | 2407 | 0.254 | 0.4920 | Yes |
| 94 | ZCCHC11 |  |  | 2492 | 0.248 | 0.4917 | Yes |
| 95 | KLF7 |  |  | 2569 | 0.244 | 0.4918 | Yes |
| 96 | MAPK6 |  |  | 2603 | 0.241 | 0.4939 | Yes |
| 97 | MTF2 |  |  | 2607 | 0.241 | 0.4975 | Yes |
| 98 | THRAP2 |  |  | 2617 | 0.241 | 0.5008 | Yes |
| 99 | RSN |  |  | 2621 | 0.240 | 0.5044 | Yes |
| 100 | LHFPL2 |  |  | 2643 | 0.239 | 0.5071 | Yes |
| 101 | MARCH7 |  |  | 2664 | 0.238 | 0.5098 | Yes |
| 102 | PTK2 |  |  | 2735 | 0.234 | 0.5100 | Yes |
| 103 | TLE1 |  |  | 2761 | 0.232 | 0.5124 | Yes |
| 104 | KIAA1109 |  |  | 2776 | 0.231 | 0.5153 | Yes |
| 105 | THRAP1 |  |  | 2780 | 0.231 | 0.5187 | Yes |
| 106 | GOLGA4 |  |  | 2786 | 0.231 | 0.5221 | Yes |
| 107 | CREB5 |  |  | 2799 | 0.230 | 0.5251 | Yes |
| 108 | FBXW11 |  |  | 2804 | 0.230 | 0.5284 | Yes |
| 109 | C3ORF63 |  |  | 2833 | 0.228 | 0.5306 | Yes |
| 110 | SOCS6 |  |  | 2839 | 0.228 | 0.5339 | Yes |
| 111 | SMAD3 |  |  | 2926 | 0.223 | 0.5331 | Yes |
| 112 | ZNF638 |  |  | 2947 | 0.222 | 0.5356 | Yes |
| 113 | DYRK1A |  |  | 2967 | 0.221 | 0.5381 | Yes |
| 114 | ACVR2A |  |  | 2997 | 0.220 | 0.5401 | Yes |
| 115 | BICD1 |  |  | 3000 | 0.219 | 0.5434 | Yes |
| 116 | REV3L |  |  | 3016 | 0.219 | 0.5461 | Yes |
| 117 | IGF2BP3 |  |  | 3018 | 0.218 | 0.5494 | Yes |
| 118 | RBM6 |  |  | 3019 | 0.218 | 0.5528 | Yes |
| 119 | FALZ |  |  | 3061 | 0.216 | 0.5542 | Yes |
| 120 | MYO10 |  |  | 3066 | 0.215 | 0.5573 | Yes |
| 121 | DLC1 |  |  | 3135 | 0.212 | 0.5573 | Yes |
| 122 | KIAA1128 |  |  | 3146 | 0.212 | 0.5601 | Yes |
| 123 | CTGF |  |  | 3167 | 0.211 | 0.5624 | Yes |
| 124 | SSBP2 |  |  | 3203 | 0.209 | 0.5639 | Yes |
| 125 | IFRD1 |  |  | 3206 | 0.209 | 0.5670 | Yes |
| 126 | STK3 |  |  | 3287 | 0.204 | 0.5663 | Yes |
| 127 | CD2AP |  |  | 3440 | 0.198 | 0.5619 | Yes |
| 128 | SEMA3C |  |  | 3448 | 0.197 | 0.5646 | Yes |
| 129 | PPAP2B |  |  | 3459 | 0.196 | 0.5671 | Yes |
| 130 | KIF14 |  |  | 3480 | 0.195 | 0.5692 | Yes |
| 131 | ARIH1 |  |  | 3514 | 0.193 | 0.5706 | Yes |
| 132 | ORC2L |  |  | 3532 | 0.192 | 0.5727 | Yes |
| 133 | USP6 |  |  | 3542 | 0.192 | 0.5753 | Yes |
| 134 | SRPK2 |  |  | 3614 | 0.189 | 0.5747 | Yes |
| 135 | CTBP2 |  |  | 3652 | 0.189 | 0.5758 | Yes |
| 136 | ABI1 |  |  | 3679 | 0.188 | 0.5775 | Yes |
| 137 | FOXO3A |  |  | 3680 | 0.188 | 0.5804 | Yes |
| 138 | NUP153 |  |  | 3690 | 0.187 | 0.5828 | Yes |
| 139 | ZDHHC17 |  |  | 3704 | 0.187 | 0.5851 | Yes |
| 140 | ITSN2 |  |  | 3803 | 0.182 | 0.5831 | Yes |
| 141 | PPP2R2A |  |  | 3811 | 0.182 | 0.5856 | Yes |
| 142 | DCUN1D4 |  |  | 3851 | 0.180 | 0.5865 | Yes |
| 143 | HELZ |  |  | 3857 | 0.180 | 0.5890 | Yes |
| 144 | TRAM2 |  |  | 3969 | 0.175 | 0.5863 | Yes |
| 145 | RBM16 |  |  | 3972 | 0.175 | 0.5889 | Yes |
| 146 | SCC-112 |  |  | 3984 | 0.174 | 0.5911 | Yes |
| 147 | EVI5 |  |  | 4021 | 0.173 | 0.5920 | Yes |
| 148 | ANKS1A |  |  | 4038 | 0.172 | 0.5939 | Yes |
| 149 | CREBBP |  |  | 4050 | 0.171 | 0.5960 | Yes |
| 150 | MPHOSPH1 |  |  | 4133 | 0.167 | 0.5946 | Yes |
| 151 | CEP170 |  |  | 4144 | 0.167 | 0.5967 | Yes |
| 152 | JMJD2C |  |  | 4180 | 0.165 | 0.5975 | Yes |
| 153 | KLHL20 |  |  | 4211 | 0.164 | 0.5986 | Yes |
| 154 | GINS1 |  |  | 4252 | 0.163 | 0.5992 | Yes |
| 155 | MITF |  |  | 4295 | 0.161 | 0.5996 | Yes |
| 156 | LYN |  |  | 4337 | 0.160 | 0.6001 | Yes |
| 157 | CASP8 |  |  | 4348 | 0.159 | 0.6021 | Yes |
| 158 | ORC5L |  |  | 4368 | 0.158 | 0.6036 | Yes |
| 159 | ANAPC10 |  |  | 4388 | 0.158 | 0.6051 | Yes |
| 160 | MAP4K5 |  |  | 4425 | 0.157 | 0.6058 | Yes |
| 161 | FNDC3A |  |  | 4710 | 0.147 | 0.5941 | No |
| 162 | BAZ1A |  |  | 4753 | 0.145 | 0.5942 | No |
| 163 | HEG1 |  |  | 4833 | 0.143 | 0.5926 | No |
| 164 | PARD3 |  |  | 4848 | 0.142 | 0.5941 | No |
| 165 | PTPN2 |  |  | 4886 | 0.141 | 0.5945 | No |
| 166 | WDFY3 |  |  | 4908 | 0.140 | 0.5956 | No |
| 167 | ARHGEF7 |  |  | 4910 | 0.140 | 0.5978 | No |
| 168 | TBL1X |  |  | 4946 | 0.139 | 0.5982 | No |
| 169 | PPP2R5E |  |  | 4950 | 0.139 | 0.6002 | No |
| 170 | RGS7 |  |  | 5006 | 0.137 | 0.5996 | No |
| 171 | NUP98 |  |  | 5012 | 0.137 | 0.6015 | No |
| 172 | NCOA3 |  |  | 5249 | 0.131 | 0.5919 | No |
| 173 | KIAA0802 |  |  | 5283 | 0.130 | 0.5923 | No |
| 174 | ATBF1 |  |  | 5300 | 0.129 | 0.5936 | No |
| 175 | KIAA0528 |  |  | 5307 | 0.129 | 0.5953 | No |
| 176 | PSD3 |  |  | 5325 | 0.128 | 0.5964 | No |
| 177 | RBM39 |  |  | 5485 | 0.124 | 0.5905 | No |
| 178 | PHLPP |  |  | 5510 | 0.124 | 0.5913 | No |
| 179 | DDX10 |  |  | 5590 | 0.122 | 0.5893 | No |
| 180 | DAAM1 |  |  | 5641 | 0.121 | 0.5887 | No |
| 181 | CLASP2 |  |  | 5665 | 0.120 | 0.5894 | No |
| 182 | XRCC4 |  |  | 5692 | 0.119 | 0.5900 | No |
| 183 | CRY1 |  |  | 5740 | 0.118 | 0.5895 | No |
| 184 | TLK2 |  |  | 5757 | 0.118 | 0.5906 | No |
| 185 | ZNF148 |  |  | 5767 | 0.118 | 0.5920 | No |
| 186 | BARD1 |  |  | 5810 | 0.117 | 0.5917 | No |
| 187 | DNMBP |  |  | 5861 | 0.116 | 0.5910 | No |
| 188 | DHX15 |  |  | 5867 | 0.116 | 0.5926 | No |
| 189 | CNOT4 |  |  | 5973 | 0.113 | 0.5892 | No |
| 190 | MYST3 |  |  | 5997 | 0.113 | 0.5898 | No |
| 191 | PIP5K3 |  |  | 6008 | 0.113 | 0.5911 | No |
| 192 | FAM48A |  |  | 6039 | 0.112 | 0.5913 | No |
| 193 | CHD1 |  |  | 6055 | 0.112 | 0.5923 | No |
| 194 | DDEF2 |  |  | 6066 | 0.111 | 0.5936 | No |
| 195 | GULP1 |  |  | 6078 | 0.111 | 0.5948 | No |
| 196 | EHBP1 |  |  | 6094 | 0.111 | 0.5957 | No |
| 197 | INPP5F |  |  | 6106 | 0.110 | 0.5969 | No |
| 198 | PPP1R12A |  |  | 6133 | 0.110 | 0.5973 | No |
| 199 | FYN |  |  | 6142 | 0.110 | 0.5987 | No |
| 200 | ZBTB20 |  |  | 6187 | 0.109 | 0.5982 | No |
| 201 | ROR1 |  |  | 6196 | 0.108 | 0.5995 | No |
| 202 | GNAQ |  |  | 6212 | 0.108 | 0.6004 | No |
| 203 | PUM2 |  |  | 6445 | 0.102 | 0.5906 | No |
| 204 | MGAT5 |  |  | 6572 | 0.100 | 0.5859 | No |
| 205 | SWAP70 |  |  | 6576 | 0.100 | 0.5873 | No |
| 206 | EDG2 |  |  | 6647 | 0.099 | 0.5854 | No |
| 207 | FBXL7 |  |  | 6656 | 0.098 | 0.5866 | No |
| 208 | CTAGE5 |  |  | 6805 | 0.095 | 0.5807 | No |
| 209 | ZHX3 |  |  | 6811 | 0.095 | 0.5820 | No |
| 210 | EIF4G3 |  |  | 6929 | 0.092 | 0.5777 | No |
| 211 | ARID5B |  |  | 6935 | 0.092 | 0.5788 | No |
| 212 | ARNT2 |  |  | 6940 | 0.092 | 0.5801 | No |
| 213 | PTPN12 |  |  | 6991 | 0.091 | 0.5790 | No |
| 214 | RRAS2 |  |  | 7000 | 0.091 | 0.5801 | No |
| 215 | MYST4 |  |  | 7079 | 0.089 | 0.5776 | No |
| 216 | PEX14 |  |  | 7106 | 0.089 | 0.5777 | No |
| 217 | C2ORF10 |  |  | 7112 | 0.089 | 0.5789 | No |
| 218 | HOMER1 |  |  | 7141 | 0.088 | 0.5789 | No |
| 219 | CENTG2 |  |  | 7174 | 0.088 | 0.5787 | No |
| 220 | KIAA0350 |  |  | 7245 | 0.087 | 0.5766 | No |
| 221 | ARHGAP12 |  |  | 7286 | 0.086 | 0.5759 | No |
| 222 | CBLB |  |  | 7419 | 0.083 | 0.5707 | No |
| 223 | SIPA1L1 |  |  | 7491 | 0.082 | 0.5685 | No |
| 224 | PBX3 |  |  | 7567 | 0.081 | 0.5661 | No |
| 225 | AUH |  |  | 7640 | 0.079 | 0.5638 | No |
| 226 | PRKCA |  |  | 7718 | 0.077 | 0.5612 | No |
| 227 | ATXN2 |  |  | 8014 | 0.073 | 0.5478 | No |
| 228 | RGL1 |  |  | 8184 | 0.070 | 0.5405 | No |
| 229 | SLC4A7 |  |  | 8221 | 0.069 | 0.5398 | No |
| 230 | PRKCBP1 |  |  | 8234 | 0.069 | 0.5403 | No |
| 231 | TSPAN5 |  |  | 8279 | 0.068 | 0.5392 | No |
| 232 | MNAT1 |  |  | 8332 | 0.067 | 0.5377 | No |
| 233 | KIF2A |  |  | 8603 | 0.063 | 0.5254 | No |
| 234 | RAI17 |  |  | 8832 | 0.060 | 0.5151 | No |
| 235 | KIAA1240 |  |  | 9001 | 0.057 | 0.5077 | No |
| 236 | MELK |  |  | 9282 | 0.053 | 0.4947 | No |
| 237 | ZHX2 |  |  | 9402 | 0.051 | 0.4897 | No |
| 238 | TCF12 |  |  | 9479 | 0.050 | 0.4867 | No |
| 239 | BAZ2B |  |  | 9510 | 0.050 | 0.4860 | No |
| 240 | TRIM24 |  |  | 9572 | 0.049 | 0.4838 | No |
| 241 | CEP135 |  |  | 9645 | 0.048 | 0.4809 | No |
| 242 | FRYL |  |  | 9894 | 0.044 | 0.4694 | No |
| 243 | PPP2R3A |  |  | 9898 | 0.043 | 0.4699 | No |
| 244 | LARS2 |  |  | 9901 | 0.043 | 0.4705 | No |
| 245 | NCOA1 |  |  | 9947 | 0.043 | 0.4690 | No |
| 246 | STARD13 |  |  | 9948 | 0.043 | 0.4696 | No |
| 247 | ARFGEF1 |  |  | 10047 | 0.041 | 0.4655 | No |
| 248 | LRIG1 |  |  | 10086 | 0.041 | 0.4642 | No |
| 249 | IL7R |  |  | 10261 | 0.038 | 0.4562 | No |
| 250 | CDK8 |  |  | 10429 | 0.036 | 0.4486 | No |
| 251 | PHF3 |  |  | 10469 | 0.035 | 0.4472 | No |
| 252 | GNAI1 |  |  | 10557 | 0.034 | 0.4434 | No |
| 253 | WAPAL |  |  | 10750 | 0.031 | 0.4345 | No |
| 254 | KIF23 |  |  | 10825 | 0.030 | 0.4313 | No |
| 255 | RNGTT |  |  | 11156 | 0.025 | 0.4154 | No |
| 256 | PRIM2A |  |  | 11241 | 0.025 | 0.4117 | No |
| 257 | HIRA |  |  | 11500 | 0.021 | 0.3993 | No |
| 258 | SLIT1 |  |  | 11589 | 0.019 | 0.3953 | No |
| 259 | TMEM23 |  |  | 11590 | 0.019 | 0.3956 | No |
| 260 | ITSN1 |  |  | 11613 | 0.019 | 0.3948 | No |
| 261 | EPHA4 |  |  | 11620 | 0.019 | 0.3948 | No |
| 262 | BLM |  |  | 11778 | 0.017 | 0.3873 | No |
| 263 | STAG1 |  |  | 12080 | 0.013 | 0.3727 | No |
| 264 | BHLHB2 |  |  | 12438 | 0.008 | 0.3552 | No |
| 265 | SUMO4 |  |  | 12700 | 0.003 | 0.3424 | No |
| 266 | TOPBP1 |  |  | 12733 | 0.002 | 0.3409 | No |
| 267 | WDHD1 |  |  | 13246 | -0.005 | 0.3158 | No |
| 268 | UPF2 |  |  | 13306 | -0.006 | 0.3129 | No |
| 269 | WDR7 |  |  | 13545 | -0.009 | 0.3014 | No |
| 270 | ACVR1 |  |  | 13608 | -0.010 | 0.2985 | No |
| 271 | CENPE |  |  | 13614 | -0.010 | 0.2984 | No |
| 272 | AQR |  |  | 13997 | -0.016 | 0.2798 | No |
| 273 | STK39 |  |  | 14174 | -0.019 | 0.2714 | No |
| 274 | DOPEY2 |  |  | 14242 | -0.020 | 0.2685 | No |
| 275 | UBL3 |  |  | 14647 | -0.026 | 0.2490 | No |
| 276 | CXORF6 |  |  | 14833 | -0.029 | 0.2403 | No |
| 277 | LARP4 |  |  | 15021 | -0.032 | 0.2316 | No |
| 278 | MAP2K4 |  |  | 15096 | -0.033 | 0.2285 | No |
| 279 | SLC16A7 |  |  | 15265 | -0.036 | 0.2208 | No |
| 280 | FGF5 |  |  | 15342 | -0.037 | 0.2176 | No |
| 281 | KIAA0888 |  |  | 15796 | -0.045 | 0.1960 | No |
| 282 | C10ORF38 |  |  | 16379 | -0.056 | 0.1682 | No |
| 283 | HAS2 |  |  | 16483 | -0.059 | 0.1640 | No |
| 284 | SPOP |  |  | 16690 | -0.063 | 0.1549 | No |
| 285 | ZFYVE9 |  |  | 17155 | -0.074 | 0.1332 | No |
| 286 | SPBC25 |  |  | 17350 | -0.079 | 0.1249 | No |
| 287 | AFAP |  |  | 17366 | -0.080 | 0.1254 | No |
| 288 | ZRF1 |  |  | 17377 | -0.080 | 0.1261 | No |
| 289 | FGF2 |  |  | 17847 | -0.094 | 0.1045 | No |
| 290 | ROCK2 |  |  | 18023 | -0.100 | 0.0974 | No |
| 291 | VEGFC |  |  | 18210 | -0.106 | 0.0899 | No |
| 292 | MPP6 |  |  | 19658 | -0.195 | 0.0217 | No |
| 293 | KNTC2 |  |  | 19779 | -0.209 | 0.0191 | No |
| 294 | ID1 |  |  | 20093 | -0.262 | 0.0077 | No |
| 295 | NRIP1 |  |  | 20273 | -0.320 | 0.0039 | No |
| 296 | GSK3B |  |  | 20356 | -0.357 | 0.0054 | No |
| 297 | CENPA |  |  | 20463 | -0.438 | 0.0070 | No |
Table: GSEA details [plain text format]

  

Fig 2: UVC\_TTD\_4HR\_DN: Random ES distribution      
 Gene set null distribution of ES for **UVC\_TTD\_4HR\_DN**
