# Supplementary material for: The rapamycin-regulated gene expression signature determines prognosis for breast cancer
Source: Mol Cancer. 2009 Sep 24;8:75. doi: 10.1186/1476-4598-8-75 (PMC2761377; doi:10.1186/1476-4598-8-75)
Supplement: Additional file 2 — Gene set enrichment analysis of in vivo data, time series. The data provided represent the time series of GSEA. This compressed file contains "Time" shortcut file and "GSEA_time" folder. Clicking on "Time" shortcut opens the index file providing access to analysis files contained in the "GSEA_time" folder. [file 1476-4598-8-75-S2.zip › GSEA_time/UVC_TTD_4HR_UP.html]

Details for gene set UVC\_TTD\_4HR\_UP[GSEA]

|  || Dataset | gsea\_time\_collapsed |
| Phenotype | NoPhenotypeAvailable |
| Upregulated in class | na\_neg |
| GeneSet | UVC\_TTD\_4HR\_UP |
| Enrichment Score (ES) | -0.4452534 |
| Normalized Enrichment Score (NES) | -1.8349736 |
| Nominal p-value | 0.0 |
| FDR q-value | 0.07545741 |
| FWER p-Value | 0.327 |
Table: GSEA Results Summary

  

Fig 1: Enrichment plot: UVC\_TTD\_4HR\_UP      
 Profile of the Running ES Score & Positions of GeneSet Members on the Rank Ordered List

  

| PROBE | GENE SYMBOL | GENE\_TITLE | RANK IN GENE LIST | RANK METRIC SCORE | RUNNING ES | CORE ENRICHMENT || 1 | EIF2S3 |  |  | 292 | 0.658 | 0.0488 | No |
| 2 | CA5BL |  |  | 1476 | 0.332 | 0.0231 | No |
| 3 | TPBG |  |  | 1854 | 0.296 | 0.0331 | No |
| 4 | ADD1 |  |  | 1959 | 0.287 | 0.0555 | No |
| 5 | KISS1 |  |  | 2287 | 0.262 | 0.0647 | No |
| 6 | PGF |  |  | 2843 | 0.228 | 0.0596 | No |
| 7 | MRPS12 |  |  | 3026 | 0.218 | 0.0717 | No |
| 8 | PLCB3 |  |  | 3313 | 0.203 | 0.0772 | No |
| 9 | PTRF |  |  | 3890 | 0.178 | 0.0663 | No |
| 10 | ABCB6 |  |  | 4205 | 0.164 | 0.0668 | No |
| 11 | BRD2 |  |  | 4522 | 0.153 | 0.0661 | No |
| 12 | ERBB2 |  |  | 4524 | 0.153 | 0.0808 | No |
| 13 | STK25 |  |  | 5197 | 0.132 | 0.0607 | No |
| 14 | CBX4 |  |  | 6080 | 0.111 | 0.0284 | No |
| 15 | HEXIM1 |  |  | 6330 | 0.105 | 0.0263 | No |
| 16 | TNFSF9 |  |  | 8085 | 0.071 | -0.0522 | No |
| 17 | TREX1 |  |  | 8170 | 0.070 | -0.0496 | No |
| 18 | PLEKHQ1 |  |  | 8551 | 0.064 | -0.0620 | No |
| 19 | RHOB |  |  | 8609 | 0.063 | -0.0587 | No |
| 20 | KCNJ4 |  |  | 8693 | 0.062 | -0.0569 | No |
| 21 | RELA |  |  | 9068 | 0.056 | -0.0697 | No |
| 22 | AMACR |  |  | 9621 | 0.048 | -0.0920 | No |
| 23 | HOXD9 |  |  | 9729 | 0.046 | -0.0928 | No |
| 24 | IGFBP6 |  |  | 9820 | 0.045 | -0.0929 | No |
| 25 | CLTB |  |  | 9908 | 0.043 | -0.0930 | No |
| 26 | CDC34 |  |  | 10411 | 0.036 | -0.1140 | No |
| 27 | STX16 |  |  | 10911 | 0.028 | -0.1355 | No |
| 28 | GALNT2 |  |  | 12030 | 0.013 | -0.1886 | No |
| 29 | ZFPL1 |  |  | 12757 | 0.002 | -0.2238 | No |
| 30 | BAD |  |  | 13424 | -0.008 | -0.2555 | No |
| 31 | CNP |  |  | 14086 | -0.017 | -0.2860 | No |
| 32 | GNL1 |  |  | 14688 | -0.026 | -0.3127 | No |
| 33 | AES |  |  | 15214 | -0.035 | -0.3349 | No |
| 34 | PCTK1 |  |  | 15235 | -0.035 | -0.3325 | No |
| 35 | LENG4 |  |  | 15477 | -0.039 | -0.3405 | No |
| 36 | TSPYL2 |  |  | 15743 | -0.044 | -0.3492 | No |
| 37 | TRIM25 |  |  | 15766 | -0.044 | -0.3460 | No |
| 38 | ACAA1 |  |  | 16091 | -0.050 | -0.3570 | No |
| 39 | C21ORF33 |  |  | 17362 | -0.080 | -0.4111 | No |
| 40 | PIM2 |  |  | 17396 | -0.081 | -0.4050 | No |
| 41 | SMARCD2 |  |  | 17416 | -0.081 | -0.3981 | No |
| 42 | FGFR3 |  |  | 17911 | -0.096 | -0.4130 | No |
| 43 | RPS6KB2 |  |  | 18263 | -0.108 | -0.4197 | No |
| 44 | PPIF |  |  | 18423 | -0.114 | -0.4165 | No |
| 45 | TMEM109 |  |  | 18740 | -0.127 | -0.4196 | No |
| 46 | ERCC2 |  |  | 18966 | -0.139 | -0.4173 | No |
| 47 | OSR2 |  |  | 19542 | -0.183 | -0.4277 | Yes |
| 48 | CDKN2B |  |  | 19799 | -0.212 | -0.4199 | Yes |
| 49 | MRPL28 |  |  | 20028 | -0.248 | -0.4073 | Yes |
| 50 | ASNA1 |  |  | 20324 | -0.344 | -0.3886 | Yes |
| 51 | FLNA |  |  | 20402 | -0.383 | -0.3556 | Yes |
| 52 | MYBL2 |  |  | 20456 | -0.434 | -0.3166 | Yes |
| 53 | BSG |  |  | 20511 | -0.492 | -0.2721 | Yes |
| 54 | MVD |  |  | 20520 | -0.501 | -0.2245 | Yes |
| 55 | EPHX1 |  |  | 20521 | -0.501 | -0.1765 | Yes |
| 56 | PLD3 |  |  | 20528 | -0.510 | -0.1279 | Yes |
| 57 | AAMP |  |  | 20573 | -0.631 | -0.0695 | Yes |
| 58 | MDK |  |  | 20591 | -0.741 | 0.0007 | Yes |
Table: GSEA details [plain text format]

  

Fig 2: UVC\_TTD\_4HR\_UP: Random ES distribution      
 Gene set null distribution of ES for **UVC\_TTD\_4HR\_UP**
