# Supplementary material for: The rapamycin-regulated gene expression signature determines prognosis for breast cancer
Source: Mol Cancer. 2009 Sep 24;8:75. doi: 10.1186/1476-4598-8-75 (PMC2761377; doi:10.1186/1476-4598-8-75)
Supplement: Additional file 2 — Gene set enrichment analysis of in vivo data, time series. The data provided represent the time series of GSEA. This compressed file contains "Time" shortcut file and "GSEA_time" folder. Clicking on "Time" shortcut opens the index file providing access to analysis files contained in the "GSEA_time" folder. [file 1476-4598-8-75-S2.zip › GSEA_time/UVC_TTD_ALL_DN.html]

Details for gene set UVC\_TTD\_ALL\_DN[GSEA]

|  || Dataset | gsea\_time\_collapsed |
| Phenotype | NoPhenotypeAvailable |
| Upregulated in class | na\_pos |
| GeneSet | UVC\_TTD\_ALL\_DN |
| Enrichment Score (ES) | 0.5926993 |
| Normalized Enrichment Score (NES) | 1.8175688 |
| Nominal p-value | 0.0 |
| FDR q-value | 0.0045681912 |
| FWER p-Value | 0.125 |
Table: GSEA Results Summary

  

Fig 1: Enrichment plot: UVC\_TTD\_ALL\_DN      
 Profile of the Running ES Score & Positions of GeneSet Members on the Rank Ordered List

  

| PROBE | GENE SYMBOL | GENE\_TITLE | RANK IN GENE LIST | RANK METRIC SCORE | RUNNING ES | CORE ENRICHMENT || 1 | EGFR |  |  | 42 | 1.173 | 0.0127 | Yes |
| 2 | NFIB |  |  | 53 | 1.093 | 0.0260 | Yes |
| 3 | OGT |  |  | 56 | 1.039 | 0.0390 | Yes |
| 4 | PLEKHC1 |  |  | 129 | 0.852 | 0.0462 | Yes |
| 5 | HNRPDL |  |  | 207 | 0.717 | 0.0515 | Yes |
| 6 | EXT1 |  |  | 230 | 0.695 | 0.0592 | Yes |
| 7 | TCF7L2 |  |  | 246 | 0.684 | 0.0671 | Yes |
| 8 | SMAD7 |  |  | 253 | 0.680 | 0.0753 | Yes |
| 9 | PKP4 |  |  | 262 | 0.677 | 0.0835 | Yes |
| 10 | NIPBL |  |  | 268 | 0.673 | 0.0917 | Yes |
| 11 | ENC1 |  |  | 277 | 0.669 | 0.0998 | Yes |
| 12 | SAMD4A |  |  | 298 | 0.650 | 0.1070 | Yes |
| 13 | WDR43 |  |  | 305 | 0.646 | 0.1149 | Yes |
| 14 | BMP2K |  |  | 316 | 0.634 | 0.1224 | Yes |
| 15 | UGCG |  |  | 335 | 0.617 | 0.1293 | Yes |
| 16 | DAPK1 |  |  | 343 | 0.610 | 0.1366 | Yes |
| 17 | KIAA1012 |  |  | 345 | 0.610 | 0.1443 | Yes |
| 18 | C6ORF111 |  |  | 348 | 0.608 | 0.1519 | Yes |
| 19 | F3 |  |  | 371 | 0.596 | 0.1583 | Yes |
| 20 | SMURF2 |  |  | 373 | 0.596 | 0.1658 | Yes |
| 21 | NEK7 |  |  | 386 | 0.591 | 0.1726 | Yes |
| 22 | CCND1 |  |  | 391 | 0.589 | 0.1799 | Yes |
| 23 | MID1 |  |  | 400 | 0.582 | 0.1868 | Yes |
| 24 | FTO |  |  | 508 | 0.529 | 0.1882 | Yes |
| 25 | MBNL1 |  |  | 514 | 0.528 | 0.1946 | Yes |
| 26 | BTAF1 |  |  | 523 | 0.523 | 0.2008 | Yes |
| 27 | SYNJ2 |  |  | 525 | 0.523 | 0.2074 | Yes |
| 28 | SKAP2 |  |  | 537 | 0.519 | 0.2134 | Yes |
| 29 | ZNF292 |  |  | 582 | 0.505 | 0.2176 | Yes |
| 30 | RBPSUH |  |  | 585 | 0.504 | 0.2239 | Yes |
| 31 | ATRX |  |  | 590 | 0.503 | 0.2300 | Yes |
| 32 | CEP350 |  |  | 626 | 0.491 | 0.2345 | Yes |
| 33 | TGFB2 |  |  | 630 | 0.490 | 0.2405 | Yes |
| 34 | DUSP1 |  |  | 672 | 0.479 | 0.2445 | Yes |
| 35 | SHB |  |  | 674 | 0.478 | 0.2505 | Yes |
| 36 | SR140 |  |  | 698 | 0.475 | 0.2554 | Yes |
| 37 | FAT |  |  | 757 | 0.461 | 0.2583 | Yes |
| 38 | ANKRD28 |  |  | 767 | 0.459 | 0.2637 | Yes |
| 39 | RUNX1 |  |  | 822 | 0.441 | 0.2666 | Yes |
| 40 | FOXJ3 |  |  | 828 | 0.439 | 0.2719 | Yes |
| 41 | FUBP1 |  |  | 830 | 0.438 | 0.2773 | Yes |
| 42 | SEC24B |  |  | 895 | 0.424 | 0.2795 | Yes |
| 43 | TUBGCP3 |  |  | 913 | 0.420 | 0.2840 | Yes |
| 44 | FCHSD2 |  |  | 925 | 0.418 | 0.2887 | Yes |
| 45 | HDAC4 |  |  | 935 | 0.417 | 0.2936 | Yes |
| 46 | R3HDM2 |  |  | 950 | 0.414 | 0.2981 | Yes |
| 47 | SOS1 |  |  | 991 | 0.407 | 0.3012 | Yes |
| 48 | BAT2D1 |  |  | 1014 | 0.402 | 0.3052 | Yes |
| 49 | GAPVD1 |  |  | 1019 | 0.402 | 0.3101 | Yes |
| 50 | BCAR3 |  |  | 1036 | 0.399 | 0.3144 | Yes |
| 51 | IRAK1BP1 |  |  | 1065 | 0.394 | 0.3179 | Yes |
| 52 | PKN2 |  |  | 1072 | 0.393 | 0.3226 | Yes |
| 53 | CDC2L6 |  |  | 1111 | 0.384 | 0.3256 | Yes |
| 54 | STRN3 |  |  | 1117 | 0.383 | 0.3302 | Yes |
| 55 | RAPGEF2 |  |  | 1129 | 0.381 | 0.3344 | Yes |
| 56 | CAMSAP1L1 |  |  | 1144 | 0.379 | 0.3385 | Yes |
| 57 | FXR1 |  |  | 1152 | 0.378 | 0.3430 | Yes |
| 58 | SCHIP1 |  |  | 1171 | 0.375 | 0.3468 | Yes |
| 59 | PDLIM5 |  |  | 1228 | 0.365 | 0.3486 | Yes |
| 60 | ZMYND11 |  |  | 1242 | 0.363 | 0.3526 | Yes |
| 61 | WWC1 |  |  | 1244 | 0.363 | 0.3571 | Yes |
| 62 | USP34 |  |  | 1252 | 0.363 | 0.3613 | Yes |
| 63 | EIF4E |  |  | 1263 | 0.361 | 0.3654 | Yes |
| 64 | SNX13 |  |  | 1265 | 0.361 | 0.3699 | Yes |
| 65 | CNOT2 |  |  | 1286 | 0.356 | 0.3734 | Yes |
| 66 | MYCBP2 |  |  | 1296 | 0.355 | 0.3775 | Yes |
| 67 | SOCS5 |  |  | 1350 | 0.347 | 0.3792 | Yes |
| 68 | KIAA0999 |  |  | 1374 | 0.344 | 0.3824 | Yes |
| 69 | LOC220594 |  |  | 1381 | 0.343 | 0.3865 | Yes |
| 70 | AFF1 |  |  | 1382 | 0.343 | 0.3908 | Yes |
| 71 | FNBP1L |  |  | 1411 | 0.340 | 0.3937 | Yes |
| 72 | SPAG9 |  |  | 1425 | 0.338 | 0.3973 | Yes |
| 73 | LRP6 |  |  | 1447 | 0.336 | 0.4005 | Yes |
| 74 | E2F3 |  |  | 1473 | 0.332 | 0.4035 | Yes |
| 75 | RNF13 |  |  | 1483 | 0.331 | 0.4072 | Yes |
| 76 | ATXN1 |  |  | 1489 | 0.330 | 0.4111 | Yes |
| 77 | UST |  |  | 1491 | 0.330 | 0.4153 | Yes |
| 78 | ANKRD17 |  |  | 1508 | 0.328 | 0.4186 | Yes |
| 79 | MEIS2 |  |  | 1555 | 0.323 | 0.4204 | Yes |
| 80 | ARHGAP29 |  |  | 1593 | 0.319 | 0.4226 | Yes |
| 81 | MSH3 |  |  | 1620 | 0.316 | 0.4253 | Yes |
| 82 | SLC25A24 |  |  | 1730 | 0.305 | 0.4238 | Yes |
| 83 | PAFAH1B1 |  |  | 1731 | 0.305 | 0.4276 | Yes |
| 84 | ZMYM2 |  |  | 1746 | 0.304 | 0.4308 | Yes |
| 85 | TLK1 |  |  | 1757 | 0.303 | 0.4341 | Yes |
| 86 | ATP2B1 |  |  | 1819 | 0.298 | 0.4349 | Yes |
| 87 | ASCC3 |  |  | 1860 | 0.295 | 0.4366 | Yes |
| 88 | TMEM131 |  |  | 1874 | 0.294 | 0.4397 | Yes |
| 89 | TLE4 |  |  | 1921 | 0.290 | 0.4411 | Yes |
| 90 | ZZZ3 |  |  | 1926 | 0.290 | 0.4445 | Yes |
| 91 | CUTL1 |  |  | 1928 | 0.290 | 0.4482 | Yes |
| 92 | KIAA1026 |  |  | 1935 | 0.290 | 0.4515 | Yes |
| 93 | RAB3GAP1 |  |  | 1947 | 0.289 | 0.4546 | Yes |
| 94 | ATF2 |  |  | 1949 | 0.289 | 0.4582 | Yes |
| 95 | SMCHD1 |  |  | 1993 | 0.285 | 0.4597 | Yes |
| 96 | MTMR3 |  |  | 1997 | 0.284 | 0.4631 | Yes |
| 97 | MDN1 |  |  | 2001 | 0.284 | 0.4666 | Yes |
| 98 | LOC151162 |  |  | 2010 | 0.284 | 0.4698 | Yes |
| 99 | PARG |  |  | 2020 | 0.282 | 0.4729 | Yes |
| 100 | MAST2 |  |  | 2025 | 0.282 | 0.4762 | Yes |
| 101 | KLF10 |  |  | 2047 | 0.280 | 0.4787 | Yes |
| 102 | PCTK2 |  |  | 2050 | 0.279 | 0.4822 | Yes |
| 103 | RABGAP1L |  |  | 2088 | 0.277 | 0.4838 | Yes |
| 104 | TRIO |  |  | 2122 | 0.275 | 0.4857 | Yes |
| 105 | IL1RAP |  |  | 2143 | 0.273 | 0.4881 | Yes |
| 106 | ATP13A3 |  |  | 2146 | 0.273 | 0.4915 | Yes |
| 107 | SETD2 |  |  | 2166 | 0.271 | 0.4940 | Yes |
| 108 | VPS13A |  |  | 2172 | 0.271 | 0.4971 | Yes |
| 109 | JARID1A |  |  | 2179 | 0.270 | 0.5002 | Yes |
| 110 | PTPRK |  |  | 2191 | 0.270 | 0.5031 | Yes |
| 111 | SRGAP2 |  |  | 2229 | 0.266 | 0.5046 | Yes |
| 112 | CDYL |  |  | 2316 | 0.261 | 0.5037 | Yes |
| 113 | TRIM33 |  |  | 2405 | 0.255 | 0.5026 | Yes |
| 114 | CLASP1 |  |  | 2407 | 0.254 | 0.5057 | Yes |
| 115 | ZCCHC11 |  |  | 2492 | 0.248 | 0.5047 | Yes |
| 116 | KLF7 |  |  | 2569 | 0.244 | 0.5040 | Yes |
| 117 | DKFZP686A01247 |  |  | 2601 | 0.241 | 0.5055 | Yes |
| 118 | MAPK6 |  |  | 2603 | 0.241 | 0.5085 | Yes |
| 119 | MTF2 |  |  | 2607 | 0.241 | 0.5114 | Yes |
| 120 | THRAP2 |  |  | 2617 | 0.241 | 0.5140 | Yes |
| 121 | RSN |  |  | 2621 | 0.240 | 0.5169 | Yes |
| 122 | LHFPL2 |  |  | 2643 | 0.239 | 0.5189 | Yes |
| 123 | MARCH7 |  |  | 2664 | 0.238 | 0.5209 | Yes |
| 124 | SEPT9 |  |  | 2673 | 0.238 | 0.5235 | Yes |
| 125 | PTK2 |  |  | 2735 | 0.234 | 0.5235 | Yes |
| 126 | TLE1 |  |  | 2761 | 0.232 | 0.5252 | Yes |
| 127 | KIAA1109 |  |  | 2776 | 0.231 | 0.5274 | Yes |
| 128 | THRAP1 |  |  | 2780 | 0.231 | 0.5302 | Yes |
| 129 | GOLGA4 |  |  | 2786 | 0.231 | 0.5328 | Yes |
| 130 | CREB5 |  |  | 2799 | 0.230 | 0.5351 | Yes |
| 131 | FBXW11 |  |  | 2804 | 0.230 | 0.5378 | Yes |
| 132 | C3ORF63 |  |  | 2833 | 0.228 | 0.5393 | Yes |
| 133 | SOCS6 |  |  | 2839 | 0.228 | 0.5420 | Yes |
| 134 | SMAD3 |  |  | 2926 | 0.223 | 0.5405 | Yes |
| 135 | ZNF638 |  |  | 2947 | 0.222 | 0.5423 | Yes |
| 136 | DYRK1A |  |  | 2967 | 0.221 | 0.5442 | Yes |
| 137 | ACVR2A |  |  | 2997 | 0.220 | 0.5455 | Yes |
| 138 | BICD1 |  |  | 3000 | 0.219 | 0.5482 | Yes |
| 139 | REV3L |  |  | 3016 | 0.219 | 0.5502 | Yes |
| 140 | IGF2BP3 |  |  | 3018 | 0.218 | 0.5529 | Yes |
| 141 | RBM6 |  |  | 3019 | 0.218 | 0.5557 | Yes |
| 142 | FALZ |  |  | 3061 | 0.216 | 0.5564 | Yes |
| 143 | MYO10 |  |  | 3066 | 0.215 | 0.5589 | Yes |
| 144 | DLC1 |  |  | 3135 | 0.212 | 0.5582 | Yes |
| 145 | KIAA1128 |  |  | 3146 | 0.212 | 0.5604 | Yes |
| 146 | CTGF |  |  | 3167 | 0.211 | 0.5621 | Yes |
| 147 | SSBP2 |  |  | 3203 | 0.209 | 0.5630 | Yes |
| 148 | IFRD1 |  |  | 3206 | 0.209 | 0.5655 | Yes |
| 149 | STK3 |  |  | 3287 | 0.204 | 0.5641 | Yes |
| 150 | AMPH |  |  | 3366 | 0.201 | 0.5628 | Yes |
| 151 | CD2AP |  |  | 3440 | 0.198 | 0.5617 | Yes |
| 152 | SEMA3C |  |  | 3448 | 0.197 | 0.5639 | Yes |
| 153 | PPAP2B |  |  | 3459 | 0.196 | 0.5658 | Yes |
| 154 | KIF14 |  |  | 3480 | 0.195 | 0.5673 | Yes |
| 155 | ARIH1 |  |  | 3514 | 0.193 | 0.5681 | Yes |
| 156 | ORC2L |  |  | 3532 | 0.192 | 0.5697 | Yes |
| 157 | USP6 |  |  | 3542 | 0.192 | 0.5717 | Yes |
| 158 | SRPK2 |  |  | 3614 | 0.189 | 0.5706 | Yes |
| 159 | CTBP2 |  |  | 3652 | 0.189 | 0.5711 | Yes |
| 160 | ABI1 |  |  | 3679 | 0.188 | 0.5722 | Yes |
| 161 | FOXO3A |  |  | 3680 | 0.188 | 0.5746 | Yes |
| 162 | NUP153 |  |  | 3690 | 0.187 | 0.5765 | Yes |
| 163 | ZDHHC17 |  |  | 3704 | 0.187 | 0.5782 | Yes |
| 164 | ITSN2 |  |  | 3803 | 0.182 | 0.5757 | Yes |
| 165 | PPP2R2A |  |  | 3811 | 0.182 | 0.5776 | Yes |
| 166 | DCUN1D4 |  |  | 3851 | 0.180 | 0.5780 | Yes |
| 167 | HELZ |  |  | 3857 | 0.180 | 0.5800 | Yes |
| 168 | TRAM2 |  |  | 3969 | 0.175 | 0.5767 | Yes |
| 169 | RBM16 |  |  | 3972 | 0.175 | 0.5788 | Yes |
| 170 | SCC-112 |  |  | 3984 | 0.174 | 0.5805 | Yes |
| 171 | MAD1L1 |  |  | 4007 | 0.173 | 0.5816 | Yes |
| 172 | EVI5 |  |  | 4021 | 0.173 | 0.5831 | Yes |
| 173 | ANKS1A |  |  | 4038 | 0.172 | 0.5845 | Yes |
| 174 | CREBBP |  |  | 4050 | 0.171 | 0.5861 | Yes |
| 175 | MPHOSPH1 |  |  | 4133 | 0.167 | 0.5842 | Yes |
| 176 | CEP170 |  |  | 4144 | 0.167 | 0.5858 | Yes |
| 177 | JMJD2C |  |  | 4180 | 0.165 | 0.5861 | Yes |
| 178 | KLHL20 |  |  | 4211 | 0.164 | 0.5867 | Yes |
| 179 | ERC1 |  |  | 4250 | 0.163 | 0.5869 | Yes |
| 180 | GINS1 |  |  | 4252 | 0.163 | 0.5889 | Yes |
| 181 | MITF |  |  | 4295 | 0.161 | 0.5889 | Yes |
| 182 | LYN |  |  | 4337 | 0.160 | 0.5889 | Yes |
| 183 | CASP8 |  |  | 4348 | 0.159 | 0.5904 | Yes |
| 184 | ORC5L |  |  | 4368 | 0.158 | 0.5914 | Yes |
| 185 | ANAPC10 |  |  | 4388 | 0.158 | 0.5925 | Yes |
| 186 | MAP4K5 |  |  | 4425 | 0.157 | 0.5927 | Yes |
| 187 | SKIV2L2 |  |  | 4616 | 0.150 | 0.5852 | No |
| 188 | FNDC3A |  |  | 4710 | 0.147 | 0.5825 | No |
| 189 | GALK2 |  |  | 4749 | 0.146 | 0.5824 | No |
| 190 | BAZ1A |  |  | 4753 | 0.145 | 0.5841 | No |
| 191 | CAP2 |  |  | 4775 | 0.145 | 0.5849 | No |
| 192 | HEG1 |  |  | 4833 | 0.143 | 0.5839 | No |
| 193 | PARD3 |  |  | 4848 | 0.142 | 0.5850 | No |
| 194 | COL5A2 |  |  | 4850 | 0.142 | 0.5867 | No |
| 195 | PTPN2 |  |  | 4886 | 0.141 | 0.5868 | No |
| 196 | WDFY3 |  |  | 4908 | 0.140 | 0.5875 | No |
| 197 | ARHGEF7 |  |  | 4910 | 0.140 | 0.5893 | No |
| 198 | TBL1X |  |  | 4946 | 0.139 | 0.5893 | No |
| 199 | PPP2R5E |  |  | 4950 | 0.139 | 0.5909 | No |
| 200 | RGS7 |  |  | 5006 | 0.137 | 0.5899 | No |
| 201 | NUP98 |  |  | 5012 | 0.137 | 0.5914 | No |
| 202 | MYO1B |  |  | 5116 | 0.135 | 0.5880 | No |
| 203 | CDC42BPA |  |  | 5135 | 0.134 | 0.5888 | No |
| 204 | NCOA3 |  |  | 5249 | 0.131 | 0.5849 | No |
| 205 | PRKACB |  |  | 5252 | 0.130 | 0.5864 | No |
| 206 | TRAK1 |  |  | 5257 | 0.130 | 0.5879 | No |
| 207 | KIAA0802 |  |  | 5283 | 0.130 | 0.5883 | No |
| 208 | ATBF1 |  |  | 5300 | 0.129 | 0.5891 | No |
| 209 | KIAA0528 |  |  | 5307 | 0.129 | 0.5904 | No |
| 210 | PSD3 |  |  | 5325 | 0.128 | 0.5912 | No |
| 211 | RBM39 |  |  | 5485 | 0.124 | 0.5849 | No |
| 212 | SLC25A13 |  |  | 5507 | 0.124 | 0.5855 | No |
| 213 | PHLPP |  |  | 5510 | 0.124 | 0.5869 | No |
| 214 | DDX10 |  |  | 5590 | 0.122 | 0.5846 | No |
| 215 | DAAM1 |  |  | 5641 | 0.121 | 0.5836 | No |
| 216 | CLASP2 |  |  | 5665 | 0.120 | 0.5840 | No |
| 217 | DPYD |  |  | 5686 | 0.120 | 0.5845 | No |
| 218 | XRCC4 |  |  | 5692 | 0.119 | 0.5858 | No |
| 219 | CRY1 |  |  | 5740 | 0.118 | 0.5849 | No |
| 220 | TLK2 |  |  | 5757 | 0.118 | 0.5856 | No |
| 221 | ZNF148 |  |  | 5767 | 0.118 | 0.5867 | No |
| 222 | BARD1 |  |  | 5810 | 0.117 | 0.5861 | No |
| 223 | DNMBP |  |  | 5861 | 0.116 | 0.5851 | No |
| 224 | DHX15 |  |  | 5867 | 0.116 | 0.5863 | No |
| 225 | CNOT4 |  |  | 5973 | 0.113 | 0.5825 | No |
| 226 | MYST3 |  |  | 5997 | 0.113 | 0.5828 | No |
| 227 | PIP5K3 |  |  | 6008 | 0.113 | 0.5837 | No |
| 228 | FAM48A |  |  | 6039 | 0.112 | 0.5837 | No |
| 229 | CHD1 |  |  | 6055 | 0.112 | 0.5843 | No |
| 230 | DDEF2 |  |  | 6066 | 0.111 | 0.5853 | No |
| 231 | GULP1 |  |  | 6078 | 0.111 | 0.5861 | No |
| 232 | EHBP1 |  |  | 6094 | 0.111 | 0.5868 | No |
| 233 | INPP5F |  |  | 6106 | 0.110 | 0.5876 | No |
| 234 | PPP1R12A |  |  | 6133 | 0.110 | 0.5877 | No |
| 235 | FYN |  |  | 6142 | 0.110 | 0.5887 | No |
| 236 | GBE1 |  |  | 6178 | 0.109 | 0.5883 | No |
| 237 | ZBTB20 |  |  | 6187 | 0.109 | 0.5893 | No |
| 238 | ROR1 |  |  | 6196 | 0.108 | 0.5903 | No |
| 239 | GNAQ |  |  | 6212 | 0.108 | 0.5909 | No |
| 240 | RFTN1 |  |  | 6275 | 0.106 | 0.5892 | No |
| 241 | SATB2 |  |  | 6369 | 0.104 | 0.5859 | No |
| 242 | MYC |  |  | 6440 | 0.103 | 0.5837 | No |
| 243 | PUM2 |  |  | 6445 | 0.102 | 0.5848 | No |
| 244 | KIAA0146 |  |  | 6479 | 0.102 | 0.5845 | No |
| 245 | MGAT5 |  |  | 6572 | 0.100 | 0.5812 | No |
| 246 | SWAP70 |  |  | 6576 | 0.100 | 0.5823 | No |
| 247 | EDG2 |  |  | 6647 | 0.099 | 0.5801 | No |
| 248 | FBXL7 |  |  | 6656 | 0.098 | 0.5810 | No |
| 249 | FLNB |  |  | 6706 | 0.098 | 0.5798 | No |
| 250 | CTAGE5 |  |  | 6805 | 0.095 | 0.5761 | No |
| 251 | ZHX3 |  |  | 6811 | 0.095 | 0.5771 | No |
| 252 | EIF4G3 |  |  | 6929 | 0.092 | 0.5725 | No |
| 253 | ARID5B |  |  | 6935 | 0.092 | 0.5734 | No |
| 254 | ARNT2 |  |  | 6940 | 0.092 | 0.5744 | No |
| 255 | PTPN12 |  |  | 6991 | 0.091 | 0.5730 | No |
| 256 | RRAS2 |  |  | 7000 | 0.091 | 0.5738 | No |
| 257 | MYST4 |  |  | 7079 | 0.089 | 0.5711 | No |
| 258 | PEX14 |  |  | 7106 | 0.089 | 0.5709 | No |
| 259 | C2ORF10 |  |  | 7112 | 0.089 | 0.5718 | No |
| 260 | HOMER1 |  |  | 7141 | 0.088 | 0.5715 | No |
| 261 | CENTG2 |  |  | 7174 | 0.088 | 0.5711 | No |
| 262 | KIAA0350 |  |  | 7245 | 0.087 | 0.5687 | No |
| 263 | ARHGAP12 |  |  | 7286 | 0.086 | 0.5678 | No |
| 264 | FARS2 |  |  | 7373 | 0.084 | 0.5646 | No |
| 265 | CBLB |  |  | 7419 | 0.083 | 0.5635 | No |
| 266 | SIPA1L1 |  |  | 7491 | 0.082 | 0.5610 | No |
| 267 | RGS20 |  |  | 7558 | 0.081 | 0.5587 | No |
| 268 | PBX3 |  |  | 7567 | 0.081 | 0.5594 | No |
| 269 | AUH |  |  | 7640 | 0.079 | 0.5568 | No |
| 270 | GRLF1 |  |  | 7693 | 0.078 | 0.5552 | No |
| 271 | PRKCA |  |  | 7718 | 0.077 | 0.5550 | No |
| 272 | TDRD3 |  |  | 7904 | 0.074 | 0.5468 | No |
| 273 | ATXN2 |  |  | 8014 | 0.073 | 0.5424 | No |
| 274 | NRG1 |  |  | 8035 | 0.072 | 0.5423 | No |
| 275 | RGL1 |  |  | 8184 | 0.070 | 0.5358 | No |
| 276 | SLC4A7 |  |  | 8221 | 0.069 | 0.5349 | No |
| 277 | PRKCBP1 |  |  | 8234 | 0.069 | 0.5352 | No |
| 278 | GPR176 |  |  | 8257 | 0.069 | 0.5350 | No |
| 279 | TSPAN5 |  |  | 8279 | 0.068 | 0.5348 | No |
| 280 | MNAT1 |  |  | 8332 | 0.067 | 0.5331 | No |
| 281 | CASK |  |  | 8354 | 0.067 | 0.5329 | No |
| 282 | KIF2A |  |  | 8603 | 0.063 | 0.5215 | No |
| 283 | RAI17 |  |  | 8832 | 0.060 | 0.5110 | No |
| 284 | KIAA1240 |  |  | 9001 | 0.057 | 0.5034 | No |
| 285 | MELK |  |  | 9282 | 0.053 | 0.4902 | No |
| 286 | ZHX2 |  |  | 9402 | 0.051 | 0.4850 | No |
| 287 | TCF12 |  |  | 9479 | 0.050 | 0.4819 | No |
| 288 | BAZ2B |  |  | 9510 | 0.050 | 0.4810 | No |
| 289 | TRIM24 |  |  | 9572 | 0.049 | 0.4786 | No |
| 290 | CEP135 |  |  | 9645 | 0.048 | 0.4756 | No |
| 291 | DOK5 |  |  | 9654 | 0.047 | 0.4758 | No |
| 292 | PTPRM |  |  | 9708 | 0.046 | 0.4738 | No |
| 293 | CRADD |  |  | 9849 | 0.044 | 0.4675 | No |
| 294 | FRYL |  |  | 9894 | 0.044 | 0.4658 | No |
| 295 | PPP2R3A |  |  | 9898 | 0.043 | 0.4662 | No |
| 296 | LARS2 |  |  | 9901 | 0.043 | 0.4667 | No |
| 297 | NCOA1 |  |  | 9947 | 0.043 | 0.4650 | No |
| 298 | STARD13 |  |  | 9948 | 0.043 | 0.4655 | No |
| 299 | ARFGEF1 |  |  | 10047 | 0.041 | 0.4612 | No |
| 300 | LRIG1 |  |  | 10086 | 0.041 | 0.4599 | No |
| 301 | IL7R |  |  | 10261 | 0.038 | 0.4517 | No |
| 302 | CDK8 |  |  | 10429 | 0.036 | 0.4439 | No |
| 303 | PHF3 |  |  | 10469 | 0.035 | 0.4425 | No |
| 304 | GNAI1 |  |  | 10557 | 0.034 | 0.4386 | No |
| 305 | WAPAL |  |  | 10750 | 0.031 | 0.4295 | No |
| 306 | KIF23 |  |  | 10825 | 0.030 | 0.4262 | No |
| 307 | LASS6 |  |  | 11028 | 0.027 | 0.4166 | No |
| 308 | RNGTT |  |  | 11156 | 0.025 | 0.4106 | No |
| 309 | PRIM2A |  |  | 11241 | 0.025 | 0.4068 | No |
| 310 | HIRA |  |  | 11500 | 0.021 | 0.3943 | No |
| 311 | SLIT1 |  |  | 11589 | 0.019 | 0.3902 | No |
| 312 | TMEM23 |  |  | 11590 | 0.019 | 0.3905 | No |
| 313 | ITSN1 |  |  | 11613 | 0.019 | 0.3896 | No |
| 314 | EPHA4 |  |  | 11620 | 0.019 | 0.3896 | No |
| 315 | KIAA1199 |  |  | 11680 | 0.018 | 0.3869 | No |
| 316 | BLM |  |  | 11778 | 0.017 | 0.3823 | No |
| 317 | STAG1 |  |  | 12080 | 0.013 | 0.3676 | No |
| 318 | BHLHB2 |  |  | 12438 | 0.008 | 0.3501 | No |
| 319 | SUMO4 |  |  | 12700 | 0.003 | 0.3372 | No |
| 320 | TOPBP1 |  |  | 12733 | 0.002 | 0.3357 | No |
| 321 | WDHD1 |  |  | 13246 | -0.005 | 0.3104 | No |
| 322 | UPF2 |  |  | 13306 | -0.006 | 0.3076 | No |
| 323 | WDR7 |  |  | 13545 | -0.009 | 0.2960 | No |
| 324 | ACVR1 |  |  | 13608 | -0.010 | 0.2930 | No |
| 325 | CENPE |  |  | 13614 | -0.010 | 0.2929 | No |
| 326 | AQR |  |  | 13997 | -0.016 | 0.2742 | No |
| 327 | STK39 |  |  | 14174 | -0.019 | 0.2658 | No |
| 328 | DOPEY2 |  |  | 14242 | -0.020 | 0.2627 | No |
| 329 | GTF2F2 |  |  | 14316 | -0.021 | 0.2594 | No |
| 330 | CXCL12 |  |  | 14323 | -0.021 | 0.2593 | No |
| 331 | UBL3 |  |  | 14647 | -0.026 | 0.2437 | No |
| 332 | CXORF6 |  |  | 14833 | -0.029 | 0.2350 | No |
| 333 | LARP4 |  |  | 15021 | -0.032 | 0.2261 | No |
| 334 | MAP2K4 |  |  | 15096 | -0.033 | 0.2229 | No |
| 335 | SLC16A7 |  |  | 15265 | -0.036 | 0.2150 | No |
| 336 | FGF5 |  |  | 15342 | -0.037 | 0.2118 | No |
| 337 | KIAA0888 |  |  | 15796 | -0.045 | 0.1900 | No |
| 338 | C10ORF38 |  |  | 16379 | -0.056 | 0.1619 | No |
| 339 | HAS2 |  |  | 16483 | -0.059 | 0.1576 | No |
| 340 | SPOP |  |  | 16690 | -0.063 | 0.1482 | No |
| 341 | ZFYVE9 |  |  | 17155 | -0.074 | 0.1262 | No |
| 342 | SPBC25 |  |  | 17350 | -0.079 | 0.1176 | No |
| 343 | AFAP |  |  | 17366 | -0.080 | 0.1179 | No |
| 344 | ZRF1 |  |  | 17377 | -0.080 | 0.1184 | No |
| 345 | MGLL |  |  | 17408 | -0.081 | 0.1180 | No |
| 346 | FGF2 |  |  | 17847 | -0.094 | 0.0975 | No |
| 347 | ROCK2 |  |  | 18023 | -0.100 | 0.0901 | No |
| 348 | VEGFC |  |  | 18210 | -0.106 | 0.0823 | No |
| 349 | SERPINE1 |  |  | 18412 | -0.114 | 0.0738 | No |
| 350 | FUT8 |  |  | 18841 | -0.133 | 0.0543 | No |
| 351 | MPP6 |  |  | 19658 | -0.195 | 0.0165 | No |
| 352 | KNTC2 |  |  | 19779 | -0.209 | 0.0132 | No |
| 353 | ID1 |  |  | 20093 | -0.262 | 0.0011 | No |
| 354 | SERPINB2 |  |  | 20152 | -0.277 | 0.0017 | No |
| 355 | NRIP1 |  |  | 20273 | -0.320 | -0.0002 | No |
| 356 | GSK3B |  |  | 20356 | -0.357 | 0.0003 | No |
| 357 | CENPA |  |  | 20463 | -0.438 | 0.0006 | No |
| 358 | G6PD |  |  | 20527 | -0.507 | 0.0039 | No |
Table: GSEA details [plain text format]

  

Fig 2: UVC\_TTD\_ALL\_DN: Random ES distribution      
 Gene set null distribution of ES for **UVC\_TTD\_ALL\_DN**
