# Supplementary material for: The rapamycin-regulated gene expression signature determines prognosis for breast cancer
Source: Mol Cancer. 2009 Sep 24;8:75. doi: 10.1186/1476-4598-8-75 (PMC2761377; doi:10.1186/1476-4598-8-75)
Supplement: Additional file 2 — Gene set enrichment analysis of in vivo data, time series. The data provided represent the time series of GSEA. This compressed file contains "Time" shortcut file and "GSEA_time" folder. Clicking on "Time" shortcut opens the index file providing access to analysis files contained in the "GSEA_time" folder. [file 1476-4598-8-75-S2.zip › GSEA_time/UVC_TTD_ALL_UP.html]

Details for gene set UVC\_TTD\_ALL\_UP[GSEA]

|  || Dataset | gsea\_time\_collapsed |
| Phenotype | NoPhenotypeAvailable |
| Upregulated in class | na\_neg |
| GeneSet | UVC\_TTD\_ALL\_UP |
| Enrichment Score (ES) | -0.3185052 |
| Normalized Enrichment Score (NES) | -1.374505 |
| Nominal p-value | 0.025 |
| FDR q-value | 0.2732962 |
| FWER p-Value | 1.0 |
Table: GSEA Results Summary

  

Fig 1: Enrichment plot: UVC\_TTD\_ALL\_UP      
 Profile of the Running ES Score & Positions of GeneSet Members on the Rank Ordered List

  

| PROBE | GENE SYMBOL | GENE\_TITLE | RANK IN GENE LIST | RANK METRIC SCORE | RUNNING ES | CORE ENRICHMENT || 1 | EIF2S3 |  |  | 292 | 0.658 | 0.0327 | No |
| 2 | DMN |  |  | 324 | 0.625 | 0.0759 | No |
| 3 | EFNA1 |  |  | 480 | 0.541 | 0.1069 | No |
| 4 | CDKN1C |  |  | 693 | 0.475 | 0.1305 | No |
| 5 | PER2 |  |  | 1195 | 0.372 | 0.1326 | No |
| 6 | CA5BL |  |  | 1476 | 0.332 | 0.1427 | No |
| 7 | TPBG |  |  | 1854 | 0.296 | 0.1454 | No |
| 8 | ADD1 |  |  | 1959 | 0.287 | 0.1609 | No |
| 9 | CEBPD |  |  | 2123 | 0.275 | 0.1725 | No |
| 10 | KISS1 |  |  | 2287 | 0.262 | 0.1833 | No |
| 11 | FAM8A1 |  |  | 2448 | 0.252 | 0.1935 | No |
| 12 | PGF |  |  | 2843 | 0.228 | 0.1905 | No |
| 13 | MRPS12 |  |  | 3026 | 0.218 | 0.1973 | No |
| 14 | PLCB3 |  |  | 3313 | 0.203 | 0.1978 | No |
| 15 | GLUL |  |  | 3828 | 0.181 | 0.1858 | No |
| 16 | PTRF |  |  | 3890 | 0.178 | 0.1955 | No |
| 17 | ABCB6 |  |  | 4205 | 0.164 | 0.1920 | No |
| 18 | MTX1 |  |  | 4461 | 0.156 | 0.1907 | No |
| 19 | BRD2 |  |  | 4522 | 0.153 | 0.1987 | No |
| 20 | ERBB2 |  |  | 4524 | 0.153 | 0.2096 | No |
| 21 | STK25 |  |  | 5197 | 0.132 | 0.1863 | No |
| 22 | TMEM24 |  |  | 5680 | 0.120 | 0.1713 | No |
| 23 | CBX4 |  |  | 6080 | 0.111 | 0.1598 | No |
| 24 | HEXIM1 |  |  | 6330 | 0.105 | 0.1552 | No |
| 25 | TP53I3 |  |  | 7036 | 0.090 | 0.1273 | No |
| 26 | TNFSF9 |  |  | 8085 | 0.071 | 0.0813 | No |
| 27 | TREX1 |  |  | 8170 | 0.070 | 0.0822 | No |
| 28 | GABARAPL1 |  |  | 8208 | 0.069 | 0.0853 | No |
| 29 | PLEKHQ1 |  |  | 8551 | 0.064 | 0.0732 | No |
| 30 | RHOB |  |  | 8609 | 0.063 | 0.0750 | No |
| 31 | KCNJ4 |  |  | 8693 | 0.062 | 0.0753 | No |
| 32 | RELA |  |  | 9068 | 0.056 | 0.0611 | No |
| 33 | GCH1 |  |  | 9218 | 0.054 | 0.0577 | No |
| 34 | CTSL2 |  |  | 9243 | 0.053 | 0.0603 | No |
| 35 | AMACR |  |  | 9621 | 0.048 | 0.0453 | No |
| 36 | HOXD9 |  |  | 9729 | 0.046 | 0.0434 | No |
| 37 | IGFBP6 |  |  | 9820 | 0.045 | 0.0422 | No |
| 38 | CLTB |  |  | 9908 | 0.043 | 0.0411 | No |
| 39 | CDC34 |  |  | 10411 | 0.036 | 0.0192 | No |
| 40 | STX16 |  |  | 10911 | 0.028 | -0.0031 | No |
| 41 | RSL1D1 |  |  | 11155 | 0.025 | -0.0131 | No |
| 42 | RNF103 |  |  | 11701 | 0.018 | -0.0384 | No |
| 43 | GALNT2 |  |  | 12030 | 0.013 | -0.0534 | No |
| 44 | ZFPL1 |  |  | 12757 | 0.002 | -0.0886 | No |
| 45 | BAD |  |  | 13424 | -0.008 | -0.1205 | No |
| 46 | CNP |  |  | 14086 | -0.017 | -0.1514 | No |
| 47 | GNL1 |  |  | 14688 | -0.026 | -0.1788 | No |
| 48 | AES |  |  | 15214 | -0.035 | -0.2019 | No |
| 49 | PCTK1 |  |  | 15235 | -0.035 | -0.2004 | No |
| 50 | LENG4 |  |  | 15477 | -0.039 | -0.2093 | No |
| 51 | TSPYL2 |  |  | 15743 | -0.044 | -0.2191 | No |
| 52 | TRIM25 |  |  | 15766 | -0.044 | -0.2170 | No |
| 53 | ACAA1 |  |  | 16091 | -0.050 | -0.2292 | No |
| 54 | SLC30A3 |  |  | 16252 | -0.054 | -0.2332 | No |
| 55 | LHX2 |  |  | 17255 | -0.076 | -0.2765 | No |
| 56 | C21ORF33 |  |  | 17362 | -0.080 | -0.2760 | No |
| 57 | PIM2 |  |  | 17396 | -0.081 | -0.2718 | No |
| 58 | SMARCD2 |  |  | 17416 | -0.081 | -0.2670 | No |
| 59 | FGFR3 |  |  | 17911 | -0.096 | -0.2842 | No |
| 60 | RPS6KB2 |  |  | 18263 | -0.108 | -0.2935 | No |
| 61 | PPIF |  |  | 18423 | -0.114 | -0.2931 | No |
| 62 | TMEM109 |  |  | 18740 | -0.127 | -0.2994 | No |
| 63 | ERCC2 |  |  | 18966 | -0.139 | -0.3005 | No |
| 64 | SHOX2 |  |  | 18969 | -0.139 | -0.2906 | No |
| 65 | OSR2 |  |  | 19542 | -0.183 | -0.3055 | Yes |
| 66 | CDKN2B |  |  | 19799 | -0.212 | -0.3028 | Yes |
| 67 | MRPL28 |  |  | 20028 | -0.248 | -0.2962 | Yes |
| 68 | ASNA1 |  |  | 20324 | -0.344 | -0.2860 | Yes |
| 69 | FLNA |  |  | 20402 | -0.383 | -0.2624 | Yes |
| 70 | MYBL2 |  |  | 20456 | -0.434 | -0.2340 | Yes |
| 71 | BSG |  |  | 20511 | -0.492 | -0.2015 | Yes |
| 72 | MVD |  |  | 20520 | -0.501 | -0.1662 | Yes |
| 73 | EPHX1 |  |  | 20521 | -0.501 | -0.1304 | Yes |
| 74 | PLD3 |  |  | 20528 | -0.510 | -0.0943 | Yes |
| 75 | AAMP |  |  | 20573 | -0.631 | -0.0514 | Yes |
| 76 | MDK |  |  | 20591 | -0.741 | 0.0007 | Yes |
Table: GSEA details [plain text format]

  

Fig 2: UVC\_TTD\_ALL\_UP: Random ES distribution      
 Gene set null distribution of ES for **UVC\_TTD\_ALL\_UP**
