# Supplementary material for: The rapamycin-regulated gene expression signature determines prognosis for breast cancer
Source: Mol Cancer. 2009 Sep 24;8:75. doi: 10.1186/1476-4598-8-75 (PMC2761377; doi:10.1186/1476-4598-8-75)
Supplement: Additional file 2 — Gene set enrichment analysis of in vivo data, time series. The data provided represent the time series of GSEA. This compressed file contains "Time" shortcut file and "GSEA_time" folder. Clicking on "Time" shortcut opens the index file providing access to analysis files contained in the "GSEA_time" folder. [file 1476-4598-8-75-S2.zip › GSEA_time/UVC_XPCS_4HR_DN.html]

Details for gene set UVC\_XPCS\_4HR\_DN[GSEA]

|  || Dataset | gsea\_time\_collapsed |
| Phenotype | NoPhenotypeAvailable |
| Upregulated in class | na\_pos |
| GeneSet | UVC\_XPCS\_4HR\_DN |
| Enrichment Score (ES) | 0.6278768 |
| Normalized Enrichment Score (NES) | 1.906259 |
| Nominal p-value | 0.0 |
| FDR q-value | 0.002014979 |
| FWER p-Value | 0.016 |
Table: GSEA Results Summary

  

Fig 1: Enrichment plot: UVC\_XPCS\_4HR\_DN      
 Profile of the Running ES Score & Positions of GeneSet Members on the Rank Ordered List

  

| PROBE | GENE SYMBOL | GENE\_TITLE | RANK IN GENE LIST | RANK METRIC SCORE | RUNNING ES | CORE ENRICHMENT || 1 | EGFR |  |  | 42 | 1.173 | 0.0190 | Yes |
| 2 | NFIB |  |  | 53 | 1.093 | 0.0381 | Yes |
| 3 | TRIM2 |  |  | 76 | 0.965 | 0.0544 | Yes |
| 4 | TSC22D2 |  |  | 98 | 0.907 | 0.0696 | Yes |
| 5 | PLEKHC1 |  |  | 129 | 0.852 | 0.0834 | Yes |
| 6 | EXT1 |  |  | 230 | 0.695 | 0.0910 | Yes |
| 7 | TCF7L2 |  |  | 246 | 0.684 | 0.1025 | Yes |
| 8 | NIPBL |  |  | 268 | 0.673 | 0.1136 | Yes |
| 9 | CHSY1 |  |  | 295 | 0.652 | 0.1240 | Yes |
| 10 | SAMD4A |  |  | 298 | 0.650 | 0.1356 | Yes |
| 11 | WDR43 |  |  | 305 | 0.646 | 0.1469 | Yes |
| 12 | VPS13B |  |  | 331 | 0.620 | 0.1568 | Yes |
| 13 | UGCG |  |  | 335 | 0.617 | 0.1677 | Yes |
| 14 | DAPK1 |  |  | 343 | 0.610 | 0.1783 | Yes |
| 15 | C6ORF111 |  |  | 348 | 0.608 | 0.1891 | Yes |
| 16 | F3 |  |  | 371 | 0.596 | 0.1987 | Yes |
| 17 | SMURF2 |  |  | 373 | 0.596 | 0.2093 | Yes |
| 18 | RASA1 |  |  | 384 | 0.593 | 0.2195 | Yes |
| 19 | MID1 |  |  | 400 | 0.582 | 0.2292 | Yes |
| 20 | TMCC1 |  |  | 425 | 0.568 | 0.2382 | Yes |
| 21 | CDC2L5 |  |  | 562 | 0.512 | 0.2407 | Yes |
| 22 | MTAP |  |  | 569 | 0.509 | 0.2496 | Yes |
| 23 | ZNF292 |  |  | 582 | 0.505 | 0.2580 | Yes |
| 24 | RBPSUH |  |  | 585 | 0.504 | 0.2670 | Yes |
| 25 | CEP350 |  |  | 626 | 0.491 | 0.2738 | Yes |
| 26 | TGFB2 |  |  | 630 | 0.490 | 0.2825 | Yes |
| 27 | CENTB2 |  |  | 661 | 0.481 | 0.2897 | Yes |
| 28 | SHB |  |  | 674 | 0.478 | 0.2977 | Yes |
| 29 | NUAK1 |  |  | 689 | 0.476 | 0.3055 | Yes |
| 30 | RUNX1 |  |  | 822 | 0.441 | 0.3069 | Yes |
| 31 | C19ORF7 |  |  | 824 | 0.440 | 0.3148 | Yes |
| 32 | FOXJ3 |  |  | 828 | 0.439 | 0.3225 | Yes |
| 33 | SEC24B |  |  | 895 | 0.424 | 0.3269 | Yes |
| 34 | TUBGCP3 |  |  | 913 | 0.420 | 0.3336 | Yes |
| 35 | FCHSD2 |  |  | 925 | 0.418 | 0.3406 | Yes |
| 36 | SOS1 |  |  | 991 | 0.407 | 0.3447 | Yes |
| 37 | BCAR3 |  |  | 1036 | 0.399 | 0.3497 | Yes |
| 38 | IRAK1BP1 |  |  | 1065 | 0.394 | 0.3554 | Yes |
| 39 | STRN3 |  |  | 1117 | 0.383 | 0.3598 | Yes |
| 40 | RAPGEF2 |  |  | 1129 | 0.381 | 0.3660 | Yes |
| 41 | CAMSAP1L1 |  |  | 1144 | 0.379 | 0.3722 | Yes |
| 42 | FXR1 |  |  | 1152 | 0.378 | 0.3786 | Yes |
| 43 | SCHIP1 |  |  | 1171 | 0.375 | 0.3845 | Yes |
| 44 | TIPARP |  |  | 1231 | 0.365 | 0.3881 | Yes |
| 45 | WWC1 |  |  | 1244 | 0.363 | 0.3940 | Yes |
| 46 | SNX13 |  |  | 1265 | 0.361 | 0.3995 | Yes |
| 47 | CNOT2 |  |  | 1286 | 0.356 | 0.4049 | Yes |
| 48 | SOCS5 |  |  | 1350 | 0.347 | 0.4081 | Yes |
| 49 | KIAA0999 |  |  | 1374 | 0.344 | 0.4131 | Yes |
| 50 | LOC220594 |  |  | 1381 | 0.343 | 0.4190 | Yes |
| 51 | AFF1 |  |  | 1382 | 0.343 | 0.4252 | Yes |
| 52 | NCK1 |  |  | 1399 | 0.341 | 0.4305 | Yes |
| 53 | RNF13 |  |  | 1483 | 0.331 | 0.4324 | Yes |
| 54 | ANKRD17 |  |  | 1508 | 0.328 | 0.4371 | Yes |
| 55 | MEIS2 |  |  | 1555 | 0.323 | 0.4406 | Yes |
| 56 | ARHGAP29 |  |  | 1593 | 0.319 | 0.4445 | Yes |
| 57 | MALT1 |  |  | 1633 | 0.315 | 0.4482 | Yes |
| 58 | AKAP10 |  |  | 1681 | 0.310 | 0.4515 | Yes |
| 59 | PAFAH1B1 |  |  | 1731 | 0.305 | 0.4546 | Yes |
| 60 | ZMYM2 |  |  | 1746 | 0.304 | 0.4593 | Yes |
| 61 | TLK1 |  |  | 1757 | 0.303 | 0.4643 | Yes |
| 62 | ATP2B1 |  |  | 1819 | 0.298 | 0.4667 | Yes |
| 63 | EIF2C2 |  |  | 1822 | 0.298 | 0.4719 | Yes |
| 64 | TLE4 |  |  | 1921 | 0.290 | 0.4723 | Yes |
| 65 | ZZZ3 |  |  | 1926 | 0.290 | 0.4773 | Yes |
| 66 | CUTL1 |  |  | 1928 | 0.290 | 0.4825 | Yes |
| 67 | KIAA1026 |  |  | 1935 | 0.290 | 0.4874 | Yes |
| 68 | ATF2 |  |  | 1949 | 0.289 | 0.4919 | Yes |
| 69 | MTMR3 |  |  | 1997 | 0.284 | 0.4947 | Yes |
| 70 | ACSL3 |  |  | 1999 | 0.284 | 0.4998 | Yes |
| 71 | PARG |  |  | 2020 | 0.282 | 0.5039 | Yes |
| 72 | KLF10 |  |  | 2047 | 0.280 | 0.5076 | Yes |
| 73 | PCTK2 |  |  | 2050 | 0.279 | 0.5125 | Yes |
| 74 | APPBP2 |  |  | 2120 | 0.275 | 0.5141 | Yes |
| 75 | TRIO |  |  | 2122 | 0.275 | 0.5190 | Yes |
| 76 | IL1RAP |  |  | 2143 | 0.273 | 0.5229 | Yes |
| 77 | SETD2 |  |  | 2166 | 0.271 | 0.5267 | Yes |
| 78 | PTPRK |  |  | 2191 | 0.270 | 0.5303 | Yes |
| 79 | CCDC93 |  |  | 2220 | 0.267 | 0.5338 | Yes |
| 80 | SRGAP2 |  |  | 2229 | 0.266 | 0.5382 | Yes |
| 81 | KLF6 |  |  | 2275 | 0.263 | 0.5407 | Yes |
| 82 | CDYL |  |  | 2316 | 0.261 | 0.5434 | Yes |
| 83 | TRIM33 |  |  | 2405 | 0.255 | 0.5436 | Yes |
| 84 | ZCCHC11 |  |  | 2492 | 0.248 | 0.5439 | Yes |
| 85 | KLF7 |  |  | 2569 | 0.244 | 0.5445 | Yes |
| 86 | DKFZP686A01247 |  |  | 2601 | 0.241 | 0.5473 | Yes |
| 87 | MTF2 |  |  | 2607 | 0.241 | 0.5514 | Yes |
| 88 | THRAP2 |  |  | 2617 | 0.241 | 0.5553 | Yes |
| 89 | RSN |  |  | 2621 | 0.240 | 0.5595 | Yes |
| 90 | LHFPL2 |  |  | 2643 | 0.239 | 0.5627 | Yes |
| 91 | MARCH7 |  |  | 2664 | 0.238 | 0.5660 | Yes |
| 92 | PTK2 |  |  | 2735 | 0.234 | 0.5668 | Yes |
| 93 | TLE1 |  |  | 2761 | 0.232 | 0.5697 | Yes |
| 94 | THRAP1 |  |  | 2780 | 0.231 | 0.5730 | Yes |
| 95 | FBXW11 |  |  | 2804 | 0.230 | 0.5760 | Yes |
| 96 | DYRK1A |  |  | 2967 | 0.221 | 0.5720 | Yes |
| 97 | BICD1 |  |  | 3000 | 0.219 | 0.5743 | Yes |
| 98 | WDR37 |  |  | 3014 | 0.219 | 0.5776 | Yes |
| 99 | REV3L |  |  | 3016 | 0.219 | 0.5815 | Yes |
| 100 | MYO10 |  |  | 3066 | 0.215 | 0.5830 | Yes |
| 101 | DLC1 |  |  | 3135 | 0.212 | 0.5834 | Yes |
| 102 | KIAA1128 |  |  | 3146 | 0.212 | 0.5868 | Yes |
| 103 | CTGF |  |  | 3167 | 0.211 | 0.5896 | Yes |
| 104 | IFRD1 |  |  | 3206 | 0.209 | 0.5914 | Yes |
| 105 | STK3 |  |  | 3287 | 0.204 | 0.5912 | Yes |
| 106 | CENTD1 |  |  | 3424 | 0.199 | 0.5881 | Yes |
| 107 | CD2AP |  |  | 3440 | 0.198 | 0.5909 | Yes |
| 108 | APRIN |  |  | 3465 | 0.196 | 0.5932 | Yes |
| 109 | MICAL3 |  |  | 3513 | 0.193 | 0.5944 | Yes |
| 110 | USP6 |  |  | 3542 | 0.192 | 0.5964 | Yes |
| 111 | KIAA0241 |  |  | 3545 | 0.192 | 0.5998 | Yes |
| 112 | USP15 |  |  | 3595 | 0.190 | 0.6008 | Yes |
| 113 | SRPK2 |  |  | 3614 | 0.189 | 0.6033 | Yes |
| 114 | AKAP9 |  |  | 3620 | 0.189 | 0.6065 | Yes |
| 115 | DOCK4 |  |  | 3631 | 0.189 | 0.6094 | Yes |
| 116 | CTBP2 |  |  | 3652 | 0.189 | 0.6118 | Yes |
| 117 | NUP153 |  |  | 3690 | 0.187 | 0.6133 | Yes |
| 118 | SPEN |  |  | 3757 | 0.184 | 0.6134 | Yes |
| 119 | ITSN2 |  |  | 3803 | 0.182 | 0.6144 | Yes |
| 120 | CENPC1 |  |  | 3842 | 0.181 | 0.6158 | Yes |
| 121 | HELZ |  |  | 3857 | 0.180 | 0.6184 | Yes |
| 122 | DOC1 |  |  | 3895 | 0.178 | 0.6198 | Yes |
| 123 | RBM16 |  |  | 3972 | 0.175 | 0.6192 | Yes |
| 124 | LARP5 |  |  | 4002 | 0.174 | 0.6208 | Yes |
| 125 | ANKS1A |  |  | 4038 | 0.172 | 0.6222 | Yes |
| 126 | TIAM1 |  |  | 4045 | 0.172 | 0.6250 | Yes |
| 127 | CREBBP |  |  | 4050 | 0.171 | 0.6279 | Yes |
| 128 | CEP170 |  |  | 4144 | 0.167 | 0.6263 | No |
| 129 | SOS2 |  |  | 4297 | 0.161 | 0.6217 | No |
| 130 | SLC25A12 |  |  | 4332 | 0.160 | 0.6229 | No |
| 131 | ORC5L |  |  | 4368 | 0.158 | 0.6241 | No |
| 132 | IGF1R |  |  | 4531 | 0.153 | 0.6188 | No |
| 133 | FNDC3A |  |  | 4710 | 0.147 | 0.6127 | No |
| 134 | BAZ1A |  |  | 4753 | 0.145 | 0.6133 | No |
| 135 | HEG1 |  |  | 4833 | 0.143 | 0.6120 | No |
| 136 | ARHGEF7 |  |  | 4910 | 0.140 | 0.6108 | No |
| 137 | IL6 |  |  | 4954 | 0.138 | 0.6111 | No |
| 138 | RGS7 |  |  | 5006 | 0.137 | 0.6111 | No |
| 139 | EDD1 |  |  | 5233 | 0.131 | 0.6023 | No |
| 140 | NCOA3 |  |  | 5249 | 0.131 | 0.6040 | No |
| 141 | KIAA0802 |  |  | 5283 | 0.130 | 0.6047 | No |
| 142 | RBM39 |  |  | 5485 | 0.124 | 0.5970 | No |
| 143 | PHLPP |  |  | 5510 | 0.124 | 0.5981 | No |
| 144 | DDX10 |  |  | 5590 | 0.122 | 0.5964 | No |
| 145 | HMG2L1 |  |  | 5620 | 0.121 | 0.5971 | No |
| 146 | CLASP2 |  |  | 5665 | 0.120 | 0.5971 | No |
| 147 | MECP2 |  |  | 5751 | 0.118 | 0.5950 | No |
| 148 | TLK2 |  |  | 5757 | 0.118 | 0.5969 | No |
| 149 | ZNF148 |  |  | 5767 | 0.118 | 0.5986 | No |
| 150 | BARD1 |  |  | 5810 | 0.117 | 0.5986 | No |
| 151 | DNMBP |  |  | 5861 | 0.116 | 0.5982 | No |
| 152 | STIL |  |  | 5918 | 0.115 | 0.5976 | No |
| 153 | CNOT4 |  |  | 5973 | 0.113 | 0.5969 | No |
| 154 | MYST3 |  |  | 5997 | 0.113 | 0.5978 | No |
| 155 | PIP5K3 |  |  | 6008 | 0.113 | 0.5994 | No |
| 156 | CHD1 |  |  | 6055 | 0.112 | 0.5991 | No |
| 157 | DDEF2 |  |  | 6066 | 0.111 | 0.6006 | No |
| 158 | EHBP1 |  |  | 6094 | 0.111 | 0.6013 | No |
| 159 | PPP1R12A |  |  | 6133 | 0.110 | 0.6014 | No |
| 160 | FYN |  |  | 6142 | 0.110 | 0.6030 | No |
| 161 | GNAQ |  |  | 6212 | 0.108 | 0.6015 | No |
| 162 | ASXL1 |  |  | 6269 | 0.106 | 0.6007 | No |
| 163 | SATB2 |  |  | 6369 | 0.104 | 0.5977 | No |
| 164 | MYC |  |  | 6440 | 0.103 | 0.5961 | No |
| 165 | PUM2 |  |  | 6445 | 0.102 | 0.5977 | No |
| 166 | NAV3 |  |  | 6515 | 0.101 | 0.5962 | No |
| 167 | KIAA0182 |  |  | 6542 | 0.101 | 0.5967 | No |
| 168 | SWAP70 |  |  | 6576 | 0.100 | 0.5969 | No |
| 169 | FBXL7 |  |  | 6656 | 0.098 | 0.5947 | No |
| 170 | CREB1 |  |  | 6802 | 0.095 | 0.5893 | No |
| 171 | ZHX3 |  |  | 6811 | 0.095 | 0.5906 | No |
| 172 | PRR3 |  |  | 6847 | 0.094 | 0.5906 | No |
| 173 | UBXD7 |  |  | 6961 | 0.092 | 0.5867 | No |
| 174 | PTPN12 |  |  | 6991 | 0.091 | 0.5869 | No |
| 175 | RRAS2 |  |  | 7000 | 0.091 | 0.5882 | No |
| 176 | MYST4 |  |  | 7079 | 0.089 | 0.5860 | No |
| 177 | KIAA0922 |  |  | 7082 | 0.089 | 0.5875 | No |
| 178 | C2ORF10 |  |  | 7112 | 0.089 | 0.5876 | No |
| 179 | CENTG2 |  |  | 7174 | 0.088 | 0.5862 | No |
| 180 | KIAA0350 |  |  | 7245 | 0.087 | 0.5843 | No |
| 181 | ARHGAP12 |  |  | 7286 | 0.086 | 0.5839 | No |
| 182 | GLRB |  |  | 7331 | 0.085 | 0.5833 | No |
| 183 | CBLB |  |  | 7419 | 0.083 | 0.5805 | No |
| 184 | PBX3 |  |  | 7567 | 0.081 | 0.5747 | No |
| 185 | ZFHX1B |  |  | 7962 | 0.073 | 0.5567 | No |
| 186 | ATXN2 |  |  | 8014 | 0.073 | 0.5555 | No |
| 187 | GPATC8 |  |  | 8017 | 0.072 | 0.5567 | No |
| 188 | PRKCBP1 |  |  | 8234 | 0.069 | 0.5473 | No |
| 189 | TSPAN5 |  |  | 8279 | 0.068 | 0.5464 | No |
| 190 | BTRC |  |  | 8340 | 0.067 | 0.5446 | No |
| 191 | TGFBR3 |  |  | 8482 | 0.065 | 0.5389 | No |
| 192 | C4ORF8 |  |  | 8502 | 0.064 | 0.5391 | No |
| 193 | KIF2A |  |  | 8603 | 0.063 | 0.5353 | No |
| 194 | RAI17 |  |  | 8832 | 0.060 | 0.5252 | No |
| 195 | PVRL3 |  |  | 8950 | 0.058 | 0.5205 | No |
| 196 | KIAA1240 |  |  | 9001 | 0.057 | 0.5191 | No |
| 197 | BMPR1A |  |  | 9101 | 0.055 | 0.5152 | No |
| 198 | MARK3 |  |  | 9253 | 0.053 | 0.5087 | No |
| 199 | ZHX2 |  |  | 9402 | 0.051 | 0.5024 | No |
| 200 | BAZ2B |  |  | 9510 | 0.050 | 0.4980 | No |
| 201 | UBR2 |  |  | 9580 | 0.049 | 0.4955 | No |
| 202 | CEP135 |  |  | 9645 | 0.048 | 0.4932 | No |
| 203 | FRYL |  |  | 9894 | 0.044 | 0.4818 | No |
| 204 | NCOA1 |  |  | 9947 | 0.043 | 0.4800 | No |
| 205 | STARD13 |  |  | 9948 | 0.043 | 0.4808 | No |
| 206 | ARFGEF1 |  |  | 10047 | 0.041 | 0.4767 | No |
| 207 | CDK8 |  |  | 10429 | 0.036 | 0.4587 | No |
| 208 | PHF3 |  |  | 10469 | 0.035 | 0.4574 | No |
| 209 | GNAI1 |  |  | 10557 | 0.034 | 0.4537 | No |
| 210 | WAPAL |  |  | 10750 | 0.031 | 0.4448 | No |
| 211 | TP53BP2 |  |  | 11080 | 0.026 | 0.4292 | No |
| 212 | RNGTT |  |  | 11156 | 0.025 | 0.4259 | No |
| 213 | HIRA |  |  | 11500 | 0.021 | 0.4095 | No |
| 214 | EPHA4 |  |  | 11620 | 0.019 | 0.4040 | No |
| 215 | KIAA1199 |  |  | 11680 | 0.018 | 0.4014 | No |
| 216 | CYR61 |  |  | 11770 | 0.017 | 0.3973 | No |
| 217 | BLM |  |  | 11778 | 0.017 | 0.3973 | No |
| 218 | FOXK2 |  |  | 12281 | 0.010 | 0.3728 | No |
| 219 | E2F5 |  |  | 12422 | 0.008 | 0.3661 | No |
| 220 | BHLHB2 |  |  | 12438 | 0.008 | 0.3655 | No |
| 221 | SUMO4 |  |  | 12700 | 0.003 | 0.3527 | No |
| 222 | TOPBP1 |  |  | 12733 | 0.002 | 0.3512 | No |
| 223 | STAM |  |  | 12754 | 0.002 | 0.3503 | No |
| 224 | RCOR1 |  |  | 12990 | -0.001 | 0.3387 | No |
| 225 | UPF2 |  |  | 13306 | -0.006 | 0.3234 | No |
| 226 | AHDC1 |  |  | 13379 | -0.007 | 0.3199 | No |
| 227 | ACVR1 |  |  | 13608 | -0.010 | 0.3089 | No |
| 228 | CENPE |  |  | 13614 | -0.010 | 0.3089 | No |
| 229 | TAF4 |  |  | 13934 | -0.015 | 0.2935 | No |
| 230 | CXORF6 |  |  | 14833 | -0.029 | 0.2499 | No |
| 231 | FGF5 |  |  | 15342 | -0.037 | 0.2256 | No |
| 232 | WNT5A |  |  | 16212 | -0.053 | 0.1839 | No |
| 233 | ZFYVE9 |  |  | 17155 | -0.074 | 0.1390 | No |
| 234 | TERF1 |  |  | 17257 | -0.076 | 0.1354 | No |
| 235 | PHLPPL |  |  | 17330 | -0.079 | 0.1333 | No |
| 236 | AFAP |  |  | 17366 | -0.080 | 0.1330 | No |
| 237 | FGF2 |  |  | 17847 | -0.094 | 0.1111 | No |
| 238 | VEGFC |  |  | 18210 | -0.106 | 0.0952 | No |
| 239 | KNTC2 |  |  | 19779 | -0.209 | 0.0220 | No |
| 240 | HOXB2 |  |  | 20124 | -0.269 | 0.0099 | No |
| 241 | NRIP1 |  |  | 20273 | -0.320 | 0.0084 | No |
| 242 | CENPA |  |  | 20463 | -0.438 | 0.0070 | No |
Table: GSEA details [plain text format]

  

Fig 2: UVC\_XPCS\_4HR\_DN: Random ES distribution      
 Gene set null distribution of ES for **UVC\_XPCS\_4HR\_DN**
