# Supplementary material for: The rapamycin-regulated gene expression signature determines prognosis for breast cancer
Source: Mol Cancer. 2009 Sep 24;8:75. doi: 10.1186/1476-4598-8-75 (PMC2761377; doi:10.1186/1476-4598-8-75)
Supplement: Additional file 2 — Gene set enrichment analysis of in vivo data, time series. The data provided represent the time series of GSEA. This compressed file contains "Time" shortcut file and "GSEA_time" folder. Clicking on "Time" shortcut opens the index file providing access to analysis files contained in the "GSEA_time" folder. [file 1476-4598-8-75-S2.zip › GSEA_time/UVC_XPCS_8HR_DN.html]

Details for gene set UVC\_XPCS\_8HR\_DN[GSEA]

|  || Dataset | gsea\_time\_collapsed |
| Phenotype | NoPhenotypeAvailable |
| Upregulated in class | na\_pos |
| GeneSet | UVC\_XPCS\_8HR\_DN |
| Enrichment Score (ES) | 0.60812205 |
| Normalized Enrichment Score (NES) | 1.8721706 |
| Nominal p-value | 0.0 |
| FDR q-value | 0.003115863 |
| FWER p-Value | 0.031 |
Table: GSEA Results Summary

  

Fig 1: Enrichment plot: UVC\_XPCS\_8HR\_DN      
 Profile of the Running ES Score & Positions of GeneSet Members on the Rank Ordered List

  

| PROBE | GENE SYMBOL | GENE\_TITLE | RANK IN GENE LIST | RANK METRIC SCORE | RUNNING ES | CORE ENRICHMENT || 1 | CRIM1 |  |  | 16 | 1.496 | 0.0155 | Yes |
| 2 | EGFR |  |  | 42 | 1.173 | 0.0271 | Yes |
| 3 | OGT |  |  | 56 | 1.039 | 0.0378 | Yes |
| 4 | TRIM2 |  |  | 76 | 0.965 | 0.0474 | Yes |
| 5 | TSC22D2 |  |  | 98 | 0.907 | 0.0562 | Yes |
| 6 | NR2F2 |  |  | 115 | 0.875 | 0.0650 | Yes |
| 7 | PLEKHC1 |  |  | 129 | 0.852 | 0.0736 | Yes |
| 8 | NBN |  |  | 159 | 0.786 | 0.0808 | Yes |
| 9 | HNRPDL |  |  | 207 | 0.717 | 0.0862 | Yes |
| 10 | EXT1 |  |  | 230 | 0.695 | 0.0927 | Yes |
| 11 | SUZ12 |  |  | 233 | 0.693 | 0.1002 | Yes |
| 12 | TCF7L2 |  |  | 246 | 0.684 | 0.1071 | Yes |
| 13 | PTEN |  |  | 250 | 0.681 | 0.1144 | Yes |
| 14 | PKP4 |  |  | 262 | 0.677 | 0.1212 | Yes |
| 15 | NIPBL |  |  | 268 | 0.673 | 0.1283 | Yes |
| 16 | SAMD4A |  |  | 298 | 0.650 | 0.1339 | Yes |
| 17 | WDR43 |  |  | 305 | 0.646 | 0.1407 | Yes |
| 18 | KIAA1033 |  |  | 315 | 0.635 | 0.1472 | Yes |
| 19 | VPS13B |  |  | 331 | 0.620 | 0.1532 | Yes |
| 20 | DAPK1 |  |  | 343 | 0.610 | 0.1593 | Yes |
| 21 | KIAA1012 |  |  | 345 | 0.610 | 0.1659 | Yes |
| 22 | C6ORF111 |  |  | 348 | 0.608 | 0.1724 | Yes |
| 23 | F3 |  |  | 371 | 0.596 | 0.1779 | Yes |
| 24 | SMURF2 |  |  | 373 | 0.596 | 0.1843 | Yes |
| 25 | RASA1 |  |  | 384 | 0.593 | 0.1903 | Yes |
| 26 | NEK7 |  |  | 386 | 0.591 | 0.1967 | Yes |
| 27 | MID1 |  |  | 400 | 0.582 | 0.2024 | Yes |
| 28 | USP12 |  |  | 427 | 0.566 | 0.2073 | Yes |
| 29 | SEC14L1 |  |  | 507 | 0.530 | 0.2091 | Yes |
| 30 | MBNL1 |  |  | 514 | 0.528 | 0.2146 | Yes |
| 31 | CDC2L5 |  |  | 562 | 0.512 | 0.2179 | Yes |
| 32 | MTAP |  |  | 569 | 0.509 | 0.2231 | Yes |
| 33 | SLC7A1 |  |  | 570 | 0.508 | 0.2287 | Yes |
| 34 | RBPSUH |  |  | 585 | 0.504 | 0.2335 | Yes |
| 35 | ATRX |  |  | 590 | 0.503 | 0.2387 | Yes |
| 36 | CEP350 |  |  | 626 | 0.491 | 0.2424 | Yes |
| 37 | TGFB2 |  |  | 630 | 0.490 | 0.2476 | Yes |
| 38 | PHF14 |  |  | 644 | 0.486 | 0.2522 | Yes |
| 39 | CENTB2 |  |  | 661 | 0.481 | 0.2567 | Yes |
| 40 | PICALM |  |  | 669 | 0.480 | 0.2616 | Yes |
| 41 | SR140 |  |  | 698 | 0.475 | 0.2654 | Yes |
| 42 | EPS8 |  |  | 715 | 0.471 | 0.2697 | Yes |
| 43 | ATR |  |  | 742 | 0.464 | 0.2735 | Yes |
| 44 | FAT |  |  | 757 | 0.461 | 0.2778 | Yes |
| 45 | SON |  |  | 768 | 0.458 | 0.2823 | Yes |
| 46 | OSBPL8 |  |  | 783 | 0.452 | 0.2866 | Yes |
| 47 | ATP2C1 |  |  | 790 | 0.447 | 0.2911 | Yes |
| 48 | SFRS3 |  |  | 819 | 0.441 | 0.2946 | Yes |
| 49 | RUNX1 |  |  | 822 | 0.441 | 0.2993 | Yes |
| 50 | C19ORF7 |  |  | 824 | 0.440 | 0.3040 | Yes |
| 51 | FOXJ3 |  |  | 828 | 0.439 | 0.3087 | Yes |
| 52 | DLG1 |  |  | 845 | 0.434 | 0.3126 | Yes |
| 53 | SMC5 |  |  | 859 | 0.431 | 0.3167 | Yes |
| 54 | SEC24B |  |  | 895 | 0.424 | 0.3195 | Yes |
| 55 | TUBGCP3 |  |  | 913 | 0.420 | 0.3233 | Yes |
| 56 | FCHSD2 |  |  | 925 | 0.418 | 0.3273 | Yes |
| 57 | R3HDM2 |  |  | 950 | 0.414 | 0.3306 | Yes |
| 58 | BAT2D1 |  |  | 1014 | 0.402 | 0.3319 | Yes |
| 59 | WWP1 |  |  | 1018 | 0.402 | 0.3361 | Yes |
| 60 | GAPVD1 |  |  | 1019 | 0.402 | 0.3405 | Yes |
| 61 | BCAR3 |  |  | 1036 | 0.399 | 0.3441 | Yes |
| 62 | IRAK1BP1 |  |  | 1065 | 0.394 | 0.3470 | Yes |
| 63 | PKN2 |  |  | 1072 | 0.393 | 0.3510 | Yes |
| 64 | TJP2 |  |  | 1083 | 0.390 | 0.3547 | Yes |
| 65 | STRN3 |  |  | 1117 | 0.383 | 0.3573 | Yes |
| 66 | RAPGEF2 |  |  | 1129 | 0.381 | 0.3609 | Yes |
| 67 | CAMSAP1L1 |  |  | 1144 | 0.379 | 0.3643 | Yes |
| 68 | FXR1 |  |  | 1152 | 0.378 | 0.3681 | Yes |
| 69 | SCHIP1 |  |  | 1171 | 0.375 | 0.3713 | Yes |
| 70 | TRIP12 |  |  | 1210 | 0.369 | 0.3735 | Yes |
| 71 | PDLIM5 |  |  | 1228 | 0.365 | 0.3766 | Yes |
| 72 | TIPARP |  |  | 1231 | 0.365 | 0.3805 | Yes |
| 73 | ZMYND11 |  |  | 1242 | 0.363 | 0.3839 | Yes |
| 74 | WWC1 |  |  | 1244 | 0.363 | 0.3879 | Yes |
| 75 | EIF4E |  |  | 1263 | 0.361 | 0.3909 | Yes |
| 76 | SNX13 |  |  | 1265 | 0.361 | 0.3948 | Yes |
| 77 | CNOT2 |  |  | 1286 | 0.356 | 0.3977 | Yes |
| 78 | MYCBP2 |  |  | 1296 | 0.355 | 0.4011 | Yes |
| 79 | SOCS5 |  |  | 1350 | 0.347 | 0.4023 | Yes |
| 80 | KIAA0999 |  |  | 1374 | 0.344 | 0.4049 | Yes |
| 81 | LOC220594 |  |  | 1381 | 0.343 | 0.4083 | Yes |
| 82 | AFF1 |  |  | 1382 | 0.343 | 0.4121 | Yes |
| 83 | FNBP1L |  |  | 1411 | 0.340 | 0.4144 | Yes |
| 84 | OPA1 |  |  | 1472 | 0.333 | 0.4151 | Yes |
| 85 | RNF13 |  |  | 1483 | 0.331 | 0.4182 | Yes |
| 86 | STAU2 |  |  | 1500 | 0.329 | 0.4210 | Yes |
| 87 | ANKRD17 |  |  | 1508 | 0.328 | 0.4242 | Yes |
| 88 | MEIS2 |  |  | 1555 | 0.323 | 0.4254 | Yes |
| 89 | ARHGAP29 |  |  | 1593 | 0.319 | 0.4271 | Yes |
| 90 | EPS15 |  |  | 1632 | 0.315 | 0.4286 | Yes |
| 91 | MALT1 |  |  | 1633 | 0.315 | 0.4321 | Yes |
| 92 | RAB6IP1 |  |  | 1703 | 0.308 | 0.4320 | Yes |
| 93 | PCMT1 |  |  | 1709 | 0.307 | 0.4351 | Yes |
| 94 | PAFAH1B1 |  |  | 1731 | 0.305 | 0.4374 | Yes |
| 95 | ZMYM2 |  |  | 1746 | 0.304 | 0.4400 | Yes |
| 96 | TLK1 |  |  | 1757 | 0.303 | 0.4428 | Yes |
| 97 | RB1CC1 |  |  | 1771 | 0.302 | 0.4455 | Yes |
| 98 | RPS6KA3 |  |  | 1776 | 0.302 | 0.4486 | Yes |
| 99 | ATP2B1 |  |  | 1819 | 0.298 | 0.4498 | Yes |
| 100 | LRPPRC |  |  | 1830 | 0.298 | 0.4525 | Yes |
| 101 | ASCC3 |  |  | 1860 | 0.295 | 0.4543 | Yes |
| 102 | CDH2 |  |  | 1872 | 0.294 | 0.4570 | Yes |
| 103 | TMEM131 |  |  | 1874 | 0.294 | 0.4601 | Yes |
| 104 | UBE2G1 |  |  | 1919 | 0.291 | 0.4611 | Yes |
| 105 | TLE4 |  |  | 1921 | 0.290 | 0.4642 | Yes |
| 106 | ZZZ3 |  |  | 1926 | 0.290 | 0.4672 | Yes |
| 107 | CUTL1 |  |  | 1928 | 0.290 | 0.4703 | Yes |
| 108 | KIAA1026 |  |  | 1935 | 0.290 | 0.4732 | Yes |
| 109 | RAB3GAP1 |  |  | 1947 | 0.289 | 0.4758 | Yes |
| 110 | ATF2 |  |  | 1949 | 0.289 | 0.4789 | Yes |
| 111 | RANBP2 |  |  | 1961 | 0.287 | 0.4815 | Yes |
| 112 | PDE8A |  |  | 1979 | 0.286 | 0.4838 | Yes |
| 113 | SMCHD1 |  |  | 1993 | 0.285 | 0.4862 | Yes |
| 114 | ACSL3 |  |  | 1999 | 0.284 | 0.4891 | Yes |
| 115 | MDN1 |  |  | 2001 | 0.284 | 0.4921 | Yes |
| 116 | LOC151162 |  |  | 2010 | 0.284 | 0.4948 | Yes |
| 117 | PARG |  |  | 2020 | 0.282 | 0.4975 | Yes |
| 118 | APPBP2 |  |  | 2120 | 0.275 | 0.4956 | Yes |
| 119 | TRIO |  |  | 2122 | 0.275 | 0.4985 | Yes |
| 120 | IL1RAP |  |  | 2143 | 0.273 | 0.5005 | Yes |
| 121 | ATP13A3 |  |  | 2146 | 0.273 | 0.5034 | Yes |
| 122 | SETD2 |  |  | 2166 | 0.271 | 0.5054 | Yes |
| 123 | VPS13A |  |  | 2172 | 0.271 | 0.5081 | Yes |
| 124 | STK24 |  |  | 2182 | 0.270 | 0.5106 | Yes |
| 125 | PTPRK |  |  | 2191 | 0.270 | 0.5131 | Yes |
| 126 | ATP11B |  |  | 2214 | 0.268 | 0.5150 | Yes |
| 127 | SRGAP2 |  |  | 2229 | 0.266 | 0.5172 | Yes |
| 128 | KLF6 |  |  | 2275 | 0.263 | 0.5178 | Yes |
| 129 | PRMT3 |  |  | 2308 | 0.261 | 0.5191 | Yes |
| 130 | NT5C2 |  |  | 2314 | 0.261 | 0.5217 | Yes |
| 131 | CDYL |  |  | 2316 | 0.261 | 0.5245 | Yes |
| 132 | PAWR |  |  | 2337 | 0.259 | 0.5263 | Yes |
| 133 | TRIM33 |  |  | 2405 | 0.255 | 0.5258 | Yes |
| 134 | CLASP1 |  |  | 2407 | 0.254 | 0.5285 | Yes |
| 135 | RAGE |  |  | 2476 | 0.249 | 0.5279 | Yes |
| 136 | PJA2 |  |  | 2487 | 0.248 | 0.5301 | Yes |
| 137 | ZCCHC11 |  |  | 2492 | 0.248 | 0.5326 | Yes |
| 138 | CDS2 |  |  | 2507 | 0.247 | 0.5346 | Yes |
| 139 | KLF7 |  |  | 2569 | 0.244 | 0.5342 | Yes |
| 140 | DKFZP686A01247 |  |  | 2601 | 0.241 | 0.5353 | Yes |
| 141 | THRAP2 |  |  | 2617 | 0.241 | 0.5372 | Yes |
| 142 | RSN |  |  | 2621 | 0.240 | 0.5397 | Yes |
| 143 | LHFPL2 |  |  | 2643 | 0.239 | 0.5412 | Yes |
| 144 | SEPT9 |  |  | 2673 | 0.238 | 0.5424 | Yes |
| 145 | PTK2 |  |  | 2735 | 0.234 | 0.5419 | Yes |
| 146 | SMARCA2 |  |  | 2755 | 0.233 | 0.5435 | Yes |
| 147 | TLE1 |  |  | 2761 | 0.232 | 0.5458 | Yes |
| 148 | KIAA1109 |  |  | 2776 | 0.231 | 0.5477 | Yes |
| 149 | GOLGA4 |  |  | 2786 | 0.231 | 0.5497 | Yes |
| 150 | PAIP1 |  |  | 2794 | 0.230 | 0.5519 | Yes |
| 151 | FBXW11 |  |  | 2804 | 0.230 | 0.5540 | Yes |
| 152 | ADARB1 |  |  | 2826 | 0.228 | 0.5554 | Yes |
| 153 | CENPF |  |  | 2832 | 0.228 | 0.5576 | Yes |
| 154 | C3ORF63 |  |  | 2833 | 0.228 | 0.5601 | Yes |
| 155 | PPM1B |  |  | 2862 | 0.227 | 0.5612 | Yes |
| 156 | SMAD3 |  |  | 2926 | 0.223 | 0.5605 | Yes |
| 157 | ZNF638 |  |  | 2947 | 0.222 | 0.5620 | Yes |
| 158 | CUL1 |  |  | 2956 | 0.222 | 0.5640 | Yes |
| 159 | DYRK1A |  |  | 2967 | 0.221 | 0.5659 | Yes |
| 160 | PIK3C2A |  |  | 2974 | 0.220 | 0.5680 | Yes |
| 161 | REV3L |  |  | 3016 | 0.219 | 0.5684 | Yes |
| 162 | IGF2BP3 |  |  | 3018 | 0.218 | 0.5707 | Yes |
| 163 | FALZ |  |  | 3061 | 0.216 | 0.5710 | Yes |
| 164 | MYO10 |  |  | 3066 | 0.215 | 0.5731 | Yes |
| 165 | DLC1 |  |  | 3135 | 0.212 | 0.5721 | Yes |
| 166 | KIAA1128 |  |  | 3146 | 0.212 | 0.5739 | Yes |
| 167 | DLEU2 |  |  | 3161 | 0.211 | 0.5755 | Yes |
| 168 | CREB3L2 |  |  | 3169 | 0.211 | 0.5774 | Yes |
| 169 | IBTK |  |  | 3191 | 0.209 | 0.5787 | Yes |
| 170 | KPNA3 |  |  | 3192 | 0.209 | 0.5810 | Yes |
| 171 | CUL2 |  |  | 3195 | 0.209 | 0.5831 | Yes |
| 172 | SSBP2 |  |  | 3203 | 0.209 | 0.5851 | Yes |
| 173 | IFRD1 |  |  | 3206 | 0.209 | 0.5873 | Yes |
| 174 | XPO6 |  |  | 3230 | 0.207 | 0.5884 | Yes |
| 175 | STK3 |  |  | 3287 | 0.204 | 0.5878 | Yes |
| 176 | TOP2A |  |  | 3342 | 0.202 | 0.5874 | Yes |
| 177 | CD2AP |  |  | 3440 | 0.198 | 0.5847 | Yes |
| 178 | SEMA3C |  |  | 3448 | 0.197 | 0.5865 | Yes |
| 179 | KIF14 |  |  | 3480 | 0.195 | 0.5871 | Yes |
| 180 | MICAL3 |  |  | 3513 | 0.193 | 0.5876 | Yes |
| 181 | ARIH1 |  |  | 3514 | 0.193 | 0.5897 | Yes |
| 182 | ORC2L |  |  | 3532 | 0.192 | 0.5910 | Yes |
| 183 | USP6 |  |  | 3542 | 0.192 | 0.5927 | Yes |
| 184 | SRPK2 |  |  | 3614 | 0.189 | 0.5912 | Yes |
| 185 | DOCK4 |  |  | 3631 | 0.189 | 0.5925 | Yes |
| 186 | CTBP2 |  |  | 3652 | 0.189 | 0.5935 | Yes |
| 187 | TRIM37 |  |  | 3675 | 0.188 | 0.5945 | Yes |
| 188 | SLK |  |  | 3744 | 0.185 | 0.5932 | Yes |
| 189 | RAB6A |  |  | 3795 | 0.183 | 0.5927 | Yes |
| 190 | ITSN2 |  |  | 3803 | 0.182 | 0.5943 | Yes |
| 191 | DCUN1D4 |  |  | 3851 | 0.180 | 0.5940 | Yes |
| 192 | HELZ |  |  | 3857 | 0.180 | 0.5957 | Yes |
| 193 | DOC1 |  |  | 3895 | 0.178 | 0.5958 | Yes |
| 194 | PTX3 |  |  | 3912 | 0.177 | 0.5969 | Yes |
| 195 | TRAM2 |  |  | 3969 | 0.175 | 0.5961 | Yes |
| 196 | RBM16 |  |  | 3972 | 0.175 | 0.5979 | Yes |
| 197 | SCC-112 |  |  | 3984 | 0.174 | 0.5992 | Yes |
| 198 | NMT2 |  |  | 4000 | 0.174 | 0.6004 | Yes |
| 199 | LARP5 |  |  | 4002 | 0.174 | 0.6022 | Yes |
| 200 | ANKS1A |  |  | 4038 | 0.172 | 0.6024 | Yes |
| 201 | TIAM1 |  |  | 4045 | 0.172 | 0.6039 | Yes |
| 202 | CREBBP |  |  | 4050 | 0.171 | 0.6056 | Yes |
| 203 | MPHOSPH1 |  |  | 4133 | 0.167 | 0.6034 | Yes |
| 204 | CEP170 |  |  | 4144 | 0.167 | 0.6047 | Yes |
| 205 | ARHGEF10 |  |  | 4156 | 0.167 | 0.6060 | Yes |
| 206 | DKFZP586P0123 |  |  | 4231 | 0.164 | 0.6041 | Yes |
| 207 | RAD23B |  |  | 4243 | 0.163 | 0.6053 | Yes |
| 208 | UBE2D2 |  |  | 4263 | 0.163 | 0.6062 | Yes |
| 209 | SOS2 |  |  | 4297 | 0.161 | 0.6063 | Yes |
| 210 | SLC25A12 |  |  | 4332 | 0.160 | 0.6063 | Yes |
| 211 | LYN |  |  | 4337 | 0.160 | 0.6079 | Yes |
| 212 | ORC5L |  |  | 4368 | 0.158 | 0.6081 | Yes |
| 213 | IGF1R |  |  | 4531 | 0.153 | 0.6018 | No |
| 214 | PTENP1 |  |  | 4534 | 0.153 | 0.6033 | No |
| 215 | SKIV2L2 |  |  | 4616 | 0.150 | 0.6010 | No |
| 216 | FNDC3A |  |  | 4710 | 0.147 | 0.5980 | No |
| 217 | PPP2R5C |  |  | 4755 | 0.145 | 0.5974 | No |
| 218 | MARCH6 |  |  | 4787 | 0.144 | 0.5974 | No |
| 219 | HEG1 |  |  | 4833 | 0.143 | 0.5967 | No |
| 220 | PARD3 |  |  | 4848 | 0.142 | 0.5976 | No |
| 221 | MKLN1 |  |  | 4874 | 0.141 | 0.5979 | No |
| 222 | PTPN2 |  |  | 4886 | 0.141 | 0.5989 | No |
| 223 | ARHGEF7 |  |  | 4910 | 0.140 | 0.5993 | No |
| 224 | TBL1X |  |  | 4946 | 0.139 | 0.5991 | No |
| 225 | PPP2R5E |  |  | 4950 | 0.139 | 0.6004 | No |
| 226 | HERC4 |  |  | 4976 | 0.138 | 0.6007 | No |
| 227 | NUMB |  |  | 4980 | 0.138 | 0.6021 | No |
| 228 | RGS7 |  |  | 5006 | 0.137 | 0.6023 | No |
| 229 | NUP98 |  |  | 5012 | 0.137 | 0.6036 | No |
| 230 | ATP8B1 |  |  | 5024 | 0.137 | 0.6045 | No |
| 231 | MYO1B |  |  | 5116 | 0.135 | 0.6015 | No |
| 232 | CDC42BPA |  |  | 5135 | 0.134 | 0.6020 | No |
| 233 | EDD1 |  |  | 5233 | 0.131 | 0.5987 | No |
| 234 | NCOA3 |  |  | 5249 | 0.131 | 0.5994 | No |
| 235 | PRKACB |  |  | 5252 | 0.130 | 0.6007 | No |
| 236 | TRAK1 |  |  | 5257 | 0.130 | 0.6019 | No |
| 237 | KIAA0802 |  |  | 5283 | 0.130 | 0.6021 | No |
| 238 | KIAA0528 |  |  | 5307 | 0.129 | 0.6024 | No |
| 239 | KIAA0376 |  |  | 5351 | 0.128 | 0.6016 | No |
| 240 | PHF21A |  |  | 5397 | 0.127 | 0.6008 | No |
| 241 | XPO1 |  |  | 5413 | 0.126 | 0.6014 | No |
| 242 | SLC25A13 |  |  | 5507 | 0.124 | 0.5982 | No |
| 243 | PHLPP |  |  | 5510 | 0.124 | 0.5994 | No |
| 244 | ADCY9 |  |  | 5523 | 0.124 | 0.6002 | No |
| 245 | DDX10 |  |  | 5590 | 0.122 | 0.5982 | No |
| 246 | R3HDM1 |  |  | 5649 | 0.120 | 0.5967 | No |
| 247 | CLASP2 |  |  | 5665 | 0.120 | 0.5972 | No |
| 248 | DPYD |  |  | 5686 | 0.120 | 0.5975 | No |
| 249 | XRCC4 |  |  | 5692 | 0.119 | 0.5986 | No |
| 250 | TLK2 |  |  | 5757 | 0.118 | 0.5967 | No |
| 251 | ZNF148 |  |  | 5767 | 0.118 | 0.5975 | No |
| 252 | BARD1 |  |  | 5810 | 0.117 | 0.5967 | No |
| 253 | NFYC |  |  | 5860 | 0.116 | 0.5956 | No |
| 254 | DNMBP |  |  | 5861 | 0.116 | 0.5968 | No |
| 255 | DHX15 |  |  | 5867 | 0.116 | 0.5979 | No |
| 256 | PLCB4 |  |  | 5873 | 0.116 | 0.5989 | No |
| 257 | OXSR1 |  |  | 5888 | 0.115 | 0.5994 | No |
| 258 | DOCK9 |  |  | 5923 | 0.114 | 0.5990 | No |
| 259 | INTS3 |  |  | 5968 | 0.113 | 0.5981 | No |
| 260 | MYST3 |  |  | 5997 | 0.113 | 0.5979 | No |
| 261 | CHD1 |  |  | 6055 | 0.112 | 0.5963 | No |
| 262 | DDEF2 |  |  | 6066 | 0.111 | 0.5970 | No |
| 263 | UTP18 |  |  | 6071 | 0.111 | 0.5980 | No |
| 264 | EHBP1 |  |  | 6094 | 0.111 | 0.5982 | No |
| 265 | INPP5F |  |  | 6106 | 0.110 | 0.5988 | No |
| 266 | PPP1R12A |  |  | 6133 | 0.110 | 0.5987 | No |
| 267 | FYN |  |  | 6142 | 0.110 | 0.5995 | No |
| 268 | GBE1 |  |  | 6178 | 0.109 | 0.5990 | No |
| 269 | GNAQ |  |  | 6212 | 0.108 | 0.5985 | No |
| 270 | KIAA0427 |  |  | 6244 | 0.107 | 0.5981 | No |
| 271 | ASXL1 |  |  | 6269 | 0.106 | 0.5981 | No |
| 272 | RFTN1 |  |  | 6275 | 0.106 | 0.5990 | No |
| 273 | SATB2 |  |  | 6369 | 0.104 | 0.5956 | No |
| 274 | MYC |  |  | 6440 | 0.103 | 0.5932 | No |
| 275 | PUM2 |  |  | 6445 | 0.102 | 0.5941 | No |
| 276 | HIVEP1 |  |  | 6464 | 0.102 | 0.5944 | No |
| 277 | NAV3 |  |  | 6515 | 0.101 | 0.5930 | No |
| 278 | ZHX3 |  |  | 6811 | 0.095 | 0.5794 | No |
| 279 | EIF4G3 |  |  | 6929 | 0.092 | 0.5746 | No |
| 280 | MYO9B |  |  | 6949 | 0.092 | 0.5747 | No |
| 281 | PTPN12 |  |  | 6991 | 0.091 | 0.5737 | No |
| 282 | RRAS2 |  |  | 7000 | 0.091 | 0.5743 | No |
| 283 | KIAA0020 |  |  | 7029 | 0.090 | 0.5739 | No |
| 284 | MYST4 |  |  | 7079 | 0.089 | 0.5724 | No |
| 285 | C2ORF10 |  |  | 7112 | 0.089 | 0.5718 | No |
| 286 | HOMER1 |  |  | 7141 | 0.088 | 0.5714 | No |
| 287 | CENTG2 |  |  | 7174 | 0.088 | 0.5708 | No |
| 288 | MPHOSPH9 |  |  | 7197 | 0.088 | 0.5706 | No |
| 289 | KIAA0350 |  |  | 7245 | 0.087 | 0.5692 | No |
| 290 | GLRB |  |  | 7331 | 0.085 | 0.5660 | No |
| 291 | C5ORF13 |  |  | 7342 | 0.085 | 0.5664 | No |
| 292 | ATRN |  |  | 7352 | 0.085 | 0.5669 | No |
| 293 | FARS2 |  |  | 7373 | 0.084 | 0.5668 | No |
| 294 | CBLB |  |  | 7419 | 0.083 | 0.5655 | No |
| 295 | RGS20 |  |  | 7558 | 0.081 | 0.5595 | No |
| 296 | PBX3 |  |  | 7567 | 0.081 | 0.5600 | No |
| 297 | MAN2A1 |  |  | 7584 | 0.080 | 0.5601 | No |
| 298 | CLOCK |  |  | 7607 | 0.080 | 0.5599 | No |
| 299 | AUH |  |  | 7640 | 0.079 | 0.5592 | No |
| 300 | PLCE1 |  |  | 7685 | 0.078 | 0.5578 | No |
| 301 | GRLF1 |  |  | 7693 | 0.078 | 0.5583 | No |
| 302 | PRKCA |  |  | 7718 | 0.077 | 0.5580 | No |
| 303 | ZFHX1B |  |  | 7962 | 0.073 | 0.5468 | No |
| 304 | NFKB1 |  |  | 7978 | 0.073 | 0.5468 | No |
| 305 | ATXN2 |  |  | 8014 | 0.073 | 0.5459 | No |
| 306 | NRG1 |  |  | 8035 | 0.072 | 0.5457 | No |
| 307 | USP24 |  |  | 8116 | 0.071 | 0.5425 | No |
| 308 | SLC4A7 |  |  | 8221 | 0.069 | 0.5381 | No |
| 309 | PRKCBP1 |  |  | 8234 | 0.069 | 0.5382 | No |
| 310 | TSPAN5 |  |  | 8279 | 0.068 | 0.5368 | No |
| 311 | RYK |  |  | 8314 | 0.067 | 0.5359 | No |
| 312 | PKD2 |  |  | 8323 | 0.067 | 0.5362 | No |
| 313 | CASK |  |  | 8354 | 0.067 | 0.5354 | No |
| 314 | C4ORF8 |  |  | 8502 | 0.064 | 0.5289 | No |
| 315 | ANKMY2 |  |  | 8633 | 0.063 | 0.5231 | No |
| 316 | NUP133 |  |  | 8752 | 0.061 | 0.5179 | No |
| 317 | PCNT |  |  | 8810 | 0.060 | 0.5158 | No |
| 318 | RAI17 |  |  | 8832 | 0.060 | 0.5154 | No |
| 319 | PEX3 |  |  | 8930 | 0.058 | 0.5112 | No |
| 320 | KIAA1240 |  |  | 9001 | 0.057 | 0.5084 | No |
| 321 | MARK3 |  |  | 9253 | 0.053 | 0.4965 | No |
| 322 | MELK |  |  | 9282 | 0.053 | 0.4957 | No |
| 323 | PIK3C3 |  |  | 9468 | 0.050 | 0.4871 | No |
| 324 | TCF12 |  |  | 9479 | 0.050 | 0.4871 | No |
| 325 | UBR2 |  |  | 9580 | 0.049 | 0.4827 | No |
| 326 | CEP135 |  |  | 9645 | 0.048 | 0.4801 | No |
| 327 | DNAJC13 |  |  | 9732 | 0.046 | 0.4763 | No |
| 328 | CRADD |  |  | 9849 | 0.044 | 0.4711 | No |
| 329 | FRYL |  |  | 9894 | 0.044 | 0.4694 | No |
| 330 | LARS2 |  |  | 9901 | 0.043 | 0.4695 | No |
| 331 | STARD13 |  |  | 9948 | 0.043 | 0.4677 | No |
| 332 | ARFGEF1 |  |  | 10047 | 0.041 | 0.4633 | No |
| 333 | IL7R |  |  | 10261 | 0.038 | 0.4532 | No |
| 334 | CDK8 |  |  | 10429 | 0.036 | 0.4453 | No |
| 335 | PHF3 |  |  | 10469 | 0.035 | 0.4438 | No |
| 336 | SLIT2 |  |  | 10587 | 0.033 | 0.4383 | No |
| 337 | ABCE1 |  |  | 10660 | 0.032 | 0.4351 | No |
| 338 | WAPAL |  |  | 10750 | 0.031 | 0.4311 | No |
| 339 | NPEPPS |  |  | 10831 | 0.030 | 0.4274 | No |
| 340 | LASS6 |  |  | 11028 | 0.027 | 0.4180 | No |
| 341 | RNGTT |  |  | 11156 | 0.025 | 0.4120 | No |
| 342 | ADAM10 |  |  | 11232 | 0.025 | 0.4086 | No |
| 343 | PRIM2A |  |  | 11241 | 0.025 | 0.4084 | No |
| 344 | MTX2 |  |  | 11433 | 0.021 | 0.3992 | No |
| 345 | ITSN1 |  |  | 11613 | 0.019 | 0.3906 | No |
| 346 | EPHA4 |  |  | 11620 | 0.019 | 0.3905 | No |
| 347 | PCCA |  |  | 11658 | 0.018 | 0.3888 | No |
| 348 | KIAA1199 |  |  | 11680 | 0.018 | 0.3880 | No |
| 349 | RPGR |  |  | 11924 | 0.015 | 0.3761 | No |
| 350 | C21ORF25 |  |  | 11943 | 0.015 | 0.3754 | No |
| 351 | STAG1 |  |  | 12080 | 0.013 | 0.3688 | No |
| 352 | CDC27 |  |  | 12319 | 0.009 | 0.3571 | No |
| 353 | E2F5 |  |  | 12422 | 0.008 | 0.3522 | No |
| 354 | SUMO4 |  |  | 12700 | 0.003 | 0.3385 | No |
| 355 | TOPBP1 |  |  | 12733 | 0.002 | 0.3369 | No |
| 356 | PRKCI |  |  | 12982 | -0.001 | 0.3246 | No |
| 357 | TDRD7 |  |  | 13023 | -0.001 | 0.3227 | No |
| 358 | KIAA0280 |  |  | 13106 | -0.003 | 0.3187 | No |
| 359 | PARN |  |  | 13134 | -0.003 | 0.3173 | No |
| 360 | HNRPH3 |  |  | 13305 | -0.006 | 0.3090 | No |
| 361 | UPF2 |  |  | 13306 | -0.006 | 0.3091 | No |
| 362 | KIF5C |  |  | 13415 | -0.007 | 0.3038 | No |
| 363 | WDR7 |  |  | 13545 | -0.009 | 0.2975 | No |
| 364 | CENPE |  |  | 13614 | -0.010 | 0.2942 | No |
| 365 | WASF3 |  |  | 13952 | -0.015 | 0.2777 | No |
| 366 | STK39 |  |  | 14174 | -0.019 | 0.2670 | No |
| 367 | DOPEY2 |  |  | 14242 | -0.020 | 0.2639 | No |
| 368 | GTF2F2 |  |  | 14316 | -0.021 | 0.2605 | No |
| 369 | CXCL12 |  |  | 14323 | -0.021 | 0.2604 | No |
| 370 | UBL3 |  |  | 14647 | -0.026 | 0.2447 | No |
| 371 | CXORF6 |  |  | 14833 | -0.029 | 0.2359 | No |
| 372 | LARP4 |  |  | 15021 | -0.032 | 0.2270 | No |
| 373 | UCK2 |  |  | 15072 | -0.033 | 0.2249 | No |
| 374 | MAP2K4 |  |  | 15096 | -0.033 | 0.2241 | No |
| 375 | CKAP5 |  |  | 15202 | -0.035 | 0.2193 | No |
| 376 | FGF5 |  |  | 15342 | -0.037 | 0.2128 | No |
| 377 | KIAA0888 |  |  | 15796 | -0.045 | 0.1908 | No |
| 378 | KIF11 |  |  | 15959 | -0.048 | 0.1833 | No |
| 379 | PMM2 |  |  | 16072 | -0.050 | 0.1783 | No |
| 380 | KIAA0423 |  |  | 16104 | -0.050 | 0.1774 | No |
| 381 | GRK5 |  |  | 16307 | -0.055 | 0.1679 | No |
| 382 | C10ORF38 |  |  | 16379 | -0.056 | 0.1650 | No |
| 383 | NEDD4 |  |  | 16536 | -0.060 | 0.1580 | No |
| 384 | NVL |  |  | 16763 | -0.064 | 0.1475 | No |
| 385 | RB1 |  |  | 16764 | -0.064 | 0.1482 | No |
| 386 | THBS2 |  |  | 16783 | -0.065 | 0.1480 | No |
| 387 | TRRAP |  |  | 16986 | -0.070 | 0.1388 | No |
| 388 | ZFYVE9 |  |  | 17155 | -0.074 | 0.1313 | No |
| 389 | USP9X |  |  | 17313 | -0.078 | 0.1243 | No |
| 390 | PHLPPL |  |  | 17330 | -0.079 | 0.1244 | No |
| 391 | AFAP |  |  | 17366 | -0.080 | 0.1236 | No |
| 392 | MGLL |  |  | 17408 | -0.081 | 0.1224 | No |
| 393 | FGF2 |  |  | 17847 | -0.094 | 0.1017 | No |
| 394 | MSH2 |  |  | 17995 | -0.099 | 0.0955 | No |
| 395 | ROCK2 |  |  | 18023 | -0.100 | 0.0953 | No |
| 396 | VEGFC |  |  | 18210 | -0.106 | 0.0873 | No |
| 397 | SERPINE1 |  |  | 18412 | -0.114 | 0.0785 | No |
| 398 | FUT8 |  |  | 18841 | -0.133 | 0.0588 | No |
| 399 | USP13 |  |  | 18919 | -0.137 | 0.0565 | No |
| 400 | RAD51C |  |  | 19206 | -0.154 | 0.0440 | No |
| 401 | MAP2K1 |  |  | 19255 | -0.157 | 0.0433 | No |
| 402 | DUSP5 |  |  | 19722 | -0.202 | 0.0225 | No |
| 403 | GMPS |  |  | 19753 | -0.206 | 0.0232 | No |
| 404 | ID1 |  |  | 20093 | -0.262 | 0.0093 | No |
| 405 | HOXB2 |  |  | 20124 | -0.269 | 0.0107 | No |
| 406 | NRIP1 |  |  | 20273 | -0.320 | 0.0069 | No |
| 407 | GSK3B |  |  | 20356 | -0.357 | 0.0067 | No |
| 408 | G6PD |  |  | 20527 | -0.507 | 0.0039 | No |
Table: GSEA details [plain text format]

  

Fig 2: UVC\_XPCS\_8HR\_DN: Random ES distribution      
 Gene set null distribution of ES for **UVC\_XPCS\_8HR\_DN**
