# Supplementary material for: The rapamycin-regulated gene expression signature determines prognosis for breast cancer
Source: Mol Cancer. 2009 Sep 24;8:75. doi: 10.1186/1476-4598-8-75 (PMC2761377; doi:10.1186/1476-4598-8-75)
Supplement: Additional file 2 — Gene set enrichment analysis of in vivo data, time series. The data provided represent the time series of GSEA. This compressed file contains "Time" shortcut file and "GSEA_time" folder. Clicking on "Time" shortcut opens the index file providing access to analysis files contained in the "GSEA_time" folder. [file 1476-4598-8-75-S2.zip › GSEA_time/UVC_XPCS_ALL_DN.html]

Details for gene set UVC\_XPCS\_ALL\_DN[GSEA]

|  || Dataset | gsea\_time\_collapsed |
| Phenotype | NoPhenotypeAvailable |
| Upregulated in class | na\_pos |
| GeneSet | UVC\_XPCS\_ALL\_DN |
| Enrichment Score (ES) | 0.601183 |
| Normalized Enrichment Score (NES) | 1.8522178 |
| Nominal p-value | 0.0 |
| FDR q-value | 0.0036137057 |
| FWER p-Value | 0.053 |
Table: GSEA Results Summary

  

Fig 1: Enrichment plot: UVC\_XPCS\_ALL\_DN      
 Profile of the Running ES Score & Positions of GeneSet Members on the Rank Ordered List

  

| PROBE | GENE SYMBOL | GENE\_TITLE | RANK IN GENE LIST | RANK METRIC SCORE | RUNNING ES | CORE ENRICHMENT || 1 | CRIM1 |  |  | 16 | 1.496 | 0.0135 | Yes |
| 2 | EGFR |  |  | 42 | 1.173 | 0.0235 | Yes |
| 3 | NFIB |  |  | 53 | 1.093 | 0.0334 | Yes |
| 4 | OGT |  |  | 56 | 1.039 | 0.0433 | Yes |
| 5 | TRIM2 |  |  | 76 | 0.965 | 0.0515 | Yes |
| 6 | TSC22D2 |  |  | 98 | 0.907 | 0.0592 | Yes |
| 7 | NR2F2 |  |  | 115 | 0.875 | 0.0667 | Yes |
| 8 | PLEKHC1 |  |  | 129 | 0.852 | 0.0742 | Yes |
| 9 | NBN |  |  | 159 | 0.786 | 0.0803 | Yes |
| 10 | HNRPDL |  |  | 207 | 0.717 | 0.0848 | Yes |
| 11 | EXT1 |  |  | 230 | 0.695 | 0.0904 | Yes |
| 12 | SUZ12 |  |  | 233 | 0.693 | 0.0969 | Yes |
| 13 | TCF7L2 |  |  | 246 | 0.684 | 0.1028 | Yes |
| 14 | PTEN |  |  | 250 | 0.681 | 0.1092 | Yes |
| 15 | PKP4 |  |  | 262 | 0.677 | 0.1151 | Yes |
| 16 | NIPBL |  |  | 268 | 0.673 | 0.1213 | Yes |
| 17 | CHSY1 |  |  | 295 | 0.652 | 0.1263 | Yes |
| 18 | SAMD4A |  |  | 298 | 0.650 | 0.1324 | Yes |
| 19 | WDR43 |  |  | 305 | 0.646 | 0.1382 | Yes |
| 20 | KIAA1033 |  |  | 315 | 0.635 | 0.1439 | Yes |
| 21 | VPS13B |  |  | 331 | 0.620 | 0.1490 | Yes |
| 22 | UGCG |  |  | 335 | 0.617 | 0.1548 | Yes |
| 23 | DAPK1 |  |  | 343 | 0.610 | 0.1603 | Yes |
| 24 | KIAA1012 |  |  | 345 | 0.610 | 0.1661 | Yes |
| 25 | C6ORF111 |  |  | 348 | 0.608 | 0.1718 | Yes |
| 26 | F3 |  |  | 371 | 0.596 | 0.1764 | Yes |
| 27 | SMURF2 |  |  | 373 | 0.596 | 0.1820 | Yes |
| 28 | RASA1 |  |  | 384 | 0.593 | 0.1872 | Yes |
| 29 | NEK7 |  |  | 386 | 0.591 | 0.1928 | Yes |
| 30 | MID1 |  |  | 400 | 0.582 | 0.1977 | Yes |
| 31 | TMCC1 |  |  | 425 | 0.568 | 0.2019 | Yes |
| 32 | USP12 |  |  | 427 | 0.566 | 0.2073 | Yes |
| 33 | SEC14L1 |  |  | 507 | 0.530 | 0.2085 | Yes |
| 34 | MBNL1 |  |  | 514 | 0.528 | 0.2132 | Yes |
| 35 | CDC2L5 |  |  | 562 | 0.512 | 0.2158 | Yes |
| 36 | MTAP |  |  | 569 | 0.509 | 0.2203 | Yes |
| 37 | SLC7A1 |  |  | 570 | 0.508 | 0.2252 | Yes |
| 38 | ZNF292 |  |  | 582 | 0.505 | 0.2295 | Yes |
| 39 | RBPSUH |  |  | 585 | 0.504 | 0.2342 | Yes |
| 40 | ATRX |  |  | 590 | 0.503 | 0.2388 | Yes |
| 41 | CEP350 |  |  | 626 | 0.491 | 0.2417 | Yes |
| 42 | TGFB2 |  |  | 630 | 0.490 | 0.2463 | Yes |
| 43 | PHF14 |  |  | 644 | 0.486 | 0.2503 | Yes |
| 44 | CENTB2 |  |  | 661 | 0.481 | 0.2541 | Yes |
| 45 | PICALM |  |  | 669 | 0.480 | 0.2583 | Yes |
| 46 | SHB |  |  | 674 | 0.478 | 0.2627 | Yes |
| 47 | NUAK1 |  |  | 689 | 0.476 | 0.2666 | Yes |
| 48 | SR140 |  |  | 698 | 0.475 | 0.2707 | Yes |
| 49 | EPS8 |  |  | 715 | 0.471 | 0.2744 | Yes |
| 50 | ATR |  |  | 742 | 0.464 | 0.2775 | Yes |
| 51 | FAT |  |  | 757 | 0.461 | 0.2812 | Yes |
| 52 | SON |  |  | 768 | 0.458 | 0.2851 | Yes |
| 53 | OSBPL8 |  |  | 783 | 0.452 | 0.2888 | Yes |
| 54 | ATP2C1 |  |  | 790 | 0.447 | 0.2927 | Yes |
| 55 | SFRS3 |  |  | 819 | 0.441 | 0.2956 | Yes |
| 56 | RUNX1 |  |  | 822 | 0.441 | 0.2997 | Yes |
| 57 | C19ORF7 |  |  | 824 | 0.440 | 0.3038 | Yes |
| 58 | FOXJ3 |  |  | 828 | 0.439 | 0.3079 | Yes |
| 59 | DLG1 |  |  | 845 | 0.434 | 0.3112 | Yes |
| 60 | SMC5 |  |  | 859 | 0.431 | 0.3147 | Yes |
| 61 | SEC24B |  |  | 895 | 0.424 | 0.3170 | Yes |
| 62 | TUBGCP3 |  |  | 913 | 0.420 | 0.3202 | Yes |
| 63 | FCHSD2 |  |  | 925 | 0.418 | 0.3236 | Yes |
| 64 | R3HDM2 |  |  | 950 | 0.414 | 0.3264 | Yes |
| 65 | SOS1 |  |  | 991 | 0.407 | 0.3283 | Yes |
| 66 | BAT2D1 |  |  | 1014 | 0.402 | 0.3311 | Yes |
| 67 | WWP1 |  |  | 1018 | 0.402 | 0.3347 | Yes |
| 68 | GAPVD1 |  |  | 1019 | 0.402 | 0.3386 | Yes |
| 69 | BCAR3 |  |  | 1036 | 0.399 | 0.3416 | Yes |
| 70 | IRAK1BP1 |  |  | 1065 | 0.394 | 0.3440 | Yes |
| 71 | PKN2 |  |  | 1072 | 0.393 | 0.3474 | Yes |
| 72 | TJP2 |  |  | 1083 | 0.390 | 0.3507 | Yes |
| 73 | STRN3 |  |  | 1117 | 0.383 | 0.3527 | Yes |
| 74 | RAPGEF2 |  |  | 1129 | 0.381 | 0.3558 | Yes |
| 75 | CAMSAP1L1 |  |  | 1144 | 0.379 | 0.3587 | Yes |
| 76 | FXR1 |  |  | 1152 | 0.378 | 0.3620 | Yes |
| 77 | SCHIP1 |  |  | 1171 | 0.375 | 0.3647 | Yes |
| 78 | TRIP12 |  |  | 1210 | 0.369 | 0.3663 | Yes |
| 79 | PDLIM5 |  |  | 1228 | 0.365 | 0.3690 | Yes |
| 80 | TIPARP |  |  | 1231 | 0.365 | 0.3723 | Yes |
| 81 | ZMYND11 |  |  | 1242 | 0.363 | 0.3753 | Yes |
| 82 | WWC1 |  |  | 1244 | 0.363 | 0.3787 | Yes |
| 83 | EIF4E |  |  | 1263 | 0.361 | 0.3813 | Yes |
| 84 | SNX13 |  |  | 1265 | 0.361 | 0.3847 | Yes |
| 85 | CNOT2 |  |  | 1286 | 0.356 | 0.3871 | Yes |
| 86 | MYCBP2 |  |  | 1296 | 0.355 | 0.3901 | Yes |
| 87 | SOCS5 |  |  | 1350 | 0.347 | 0.3907 | Yes |
| 88 | KIAA0999 |  |  | 1374 | 0.344 | 0.3929 | Yes |
| 89 | LOC220594 |  |  | 1381 | 0.343 | 0.3959 | Yes |
| 90 | AFF1 |  |  | 1382 | 0.343 | 0.3991 | Yes |
| 91 | NCK1 |  |  | 1399 | 0.341 | 0.4016 | Yes |
| 92 | FNBP1L |  |  | 1411 | 0.340 | 0.4043 | Yes |
| 93 | OPA1 |  |  | 1472 | 0.333 | 0.4045 | Yes |
| 94 | RNF13 |  |  | 1483 | 0.331 | 0.4072 | Yes |
| 95 | STAU2 |  |  | 1500 | 0.329 | 0.4095 | Yes |
| 96 | ANKRD17 |  |  | 1508 | 0.328 | 0.4123 | Yes |
| 97 | MEIS2 |  |  | 1555 | 0.323 | 0.4131 | Yes |
| 98 | ARHGAP29 |  |  | 1593 | 0.319 | 0.4143 | Yes |
| 99 | EPS15 |  |  | 1632 | 0.315 | 0.4154 | Yes |
| 100 | MALT1 |  |  | 1633 | 0.315 | 0.4185 | Yes |
| 101 | AKAP10 |  |  | 1681 | 0.310 | 0.4191 | Yes |
| 102 | RAB6IP1 |  |  | 1703 | 0.308 | 0.4210 | Yes |
| 103 | PCMT1 |  |  | 1709 | 0.307 | 0.4237 | Yes |
| 104 | PAFAH1B1 |  |  | 1731 | 0.305 | 0.4255 | Yes |
| 105 | ZMYM2 |  |  | 1746 | 0.304 | 0.4278 | Yes |
| 106 | TLK1 |  |  | 1757 | 0.303 | 0.4302 | Yes |
| 107 | RB1CC1 |  |  | 1771 | 0.302 | 0.4324 | Yes |
| 108 | RPS6KA3 |  |  | 1776 | 0.302 | 0.4351 | Yes |
| 109 | ATP2B1 |  |  | 1819 | 0.298 | 0.4359 | Yes |
| 110 | EIF2C2 |  |  | 1822 | 0.298 | 0.4386 | Yes |
| 111 | LRPPRC |  |  | 1830 | 0.298 | 0.4411 | Yes |
| 112 | ASCC3 |  |  | 1860 | 0.295 | 0.4425 | Yes |
| 113 | CDH2 |  |  | 1872 | 0.294 | 0.4447 | Yes |
| 114 | TMEM131 |  |  | 1874 | 0.294 | 0.4475 | Yes |
| 115 | UBE2G1 |  |  | 1919 | 0.291 | 0.4481 | Yes |
| 116 | TLE4 |  |  | 1921 | 0.290 | 0.4508 | Yes |
| 117 | ZZZ3 |  |  | 1926 | 0.290 | 0.4534 | Yes |
| 118 | CUTL1 |  |  | 1928 | 0.290 | 0.4561 | Yes |
| 119 | KIAA1026 |  |  | 1935 | 0.290 | 0.4586 | Yes |
| 120 | RAB3GAP1 |  |  | 1947 | 0.289 | 0.4608 | Yes |
| 121 | ATF2 |  |  | 1949 | 0.289 | 0.4635 | Yes |
| 122 | RANBP2 |  |  | 1961 | 0.287 | 0.4657 | Yes |
| 123 | PDE8A |  |  | 1979 | 0.286 | 0.4676 | Yes |
| 124 | SMCHD1 |  |  | 1993 | 0.285 | 0.4697 | Yes |
| 125 | MTMR3 |  |  | 1997 | 0.284 | 0.4722 | Yes |
| 126 | ACSL3 |  |  | 1999 | 0.284 | 0.4749 | Yes |
| 127 | MDN1 |  |  | 2001 | 0.284 | 0.4776 | Yes |
| 128 | LOC151162 |  |  | 2010 | 0.284 | 0.4799 | Yes |
| 129 | PARG |  |  | 2020 | 0.282 | 0.4821 | Yes |
| 130 | KLF10 |  |  | 2047 | 0.280 | 0.4835 | Yes |
| 131 | PCTK2 |  |  | 2050 | 0.279 | 0.4861 | Yes |
| 132 | APPBP2 |  |  | 2120 | 0.275 | 0.4853 | Yes |
| 133 | TRIO |  |  | 2122 | 0.275 | 0.4879 | Yes |
| 134 | IL1RAP |  |  | 2143 | 0.273 | 0.4895 | Yes |
| 135 | ATP13A3 |  |  | 2146 | 0.273 | 0.4920 | Yes |
| 136 | SETD2 |  |  | 2166 | 0.271 | 0.4937 | Yes |
| 137 | VPS13A |  |  | 2172 | 0.271 | 0.4960 | Yes |
| 138 | STK24 |  |  | 2182 | 0.270 | 0.4981 | Yes |
| 139 | PTPRK |  |  | 2191 | 0.270 | 0.5003 | Yes |
| 140 | ATP11B |  |  | 2214 | 0.268 | 0.5018 | Yes |
| 141 | CCDC93 |  |  | 2220 | 0.267 | 0.5041 | Yes |
| 142 | SRGAP2 |  |  | 2229 | 0.266 | 0.5062 | Yes |
| 143 | KLF6 |  |  | 2275 | 0.263 | 0.5065 | Yes |
| 144 | PRMT3 |  |  | 2308 | 0.261 | 0.5074 | Yes |
| 145 | NT5C2 |  |  | 2314 | 0.261 | 0.5097 | Yes |
| 146 | CDYL |  |  | 2316 | 0.261 | 0.5121 | Yes |
| 147 | PAWR |  |  | 2337 | 0.259 | 0.5136 | Yes |
| 148 | TRIM33 |  |  | 2405 | 0.255 | 0.5127 | Yes |
| 149 | CLASP1 |  |  | 2407 | 0.254 | 0.5151 | Yes |
| 150 | RAGE |  |  | 2476 | 0.249 | 0.5141 | Yes |
| 151 | PJA2 |  |  | 2487 | 0.248 | 0.5160 | Yes |
| 152 | ZCCHC11 |  |  | 2492 | 0.248 | 0.5181 | Yes |
| 153 | CDS2 |  |  | 2507 | 0.247 | 0.5198 | Yes |
| 154 | KLF7 |  |  | 2569 | 0.244 | 0.5191 | Yes |
| 155 | DKFZP686A01247 |  |  | 2601 | 0.241 | 0.5199 | Yes |
| 156 | MTF2 |  |  | 2607 | 0.241 | 0.5219 | Yes |
| 157 | THRAP2 |  |  | 2617 | 0.241 | 0.5238 | Yes |
| 158 | RSN |  |  | 2621 | 0.240 | 0.5259 | Yes |
| 159 | LHFPL2 |  |  | 2643 | 0.239 | 0.5272 | Yes |
| 160 | MARCH7 |  |  | 2664 | 0.238 | 0.5285 | Yes |
| 161 | SEPT9 |  |  | 2673 | 0.238 | 0.5303 | Yes |
| 162 | PTK2 |  |  | 2735 | 0.234 | 0.5295 | Yes |
| 163 | SMARCA2 |  |  | 2755 | 0.233 | 0.5308 | Yes |
| 164 | TLE1 |  |  | 2761 | 0.232 | 0.5328 | Yes |
| 165 | KIAA1109 |  |  | 2776 | 0.231 | 0.5343 | Yes |
| 166 | THRAP1 |  |  | 2780 | 0.231 | 0.5364 | Yes |
| 167 | GOLGA4 |  |  | 2786 | 0.231 | 0.5383 | Yes |
| 168 | PAIP1 |  |  | 2794 | 0.230 | 0.5402 | Yes |
| 169 | FBXW11 |  |  | 2804 | 0.230 | 0.5419 | Yes |
| 170 | ADARB1 |  |  | 2826 | 0.228 | 0.5431 | Yes |
| 171 | CENPF |  |  | 2832 | 0.228 | 0.5450 | Yes |
| 172 | C3ORF63 |  |  | 2833 | 0.228 | 0.5472 | Yes |
| 173 | PPM1B |  |  | 2862 | 0.227 | 0.5480 | Yes |
| 174 | SMAD3 |  |  | 2926 | 0.223 | 0.5470 | Yes |
| 175 | ZNF638 |  |  | 2947 | 0.222 | 0.5481 | Yes |
| 176 | CUL1 |  |  | 2956 | 0.222 | 0.5498 | Yes |
| 177 | DYRK1A |  |  | 2967 | 0.221 | 0.5514 | Yes |
| 178 | PIK3C2A |  |  | 2974 | 0.220 | 0.5532 | Yes |
| 179 | BICD1 |  |  | 3000 | 0.219 | 0.5541 | Yes |
| 180 | WDR37 |  |  | 3014 | 0.219 | 0.5555 | Yes |
| 181 | REV3L |  |  | 3016 | 0.219 | 0.5576 | Yes |
| 182 | IGF2BP3 |  |  | 3018 | 0.218 | 0.5596 | Yes |
| 183 | FALZ |  |  | 3061 | 0.216 | 0.5596 | Yes |
| 184 | MYO10 |  |  | 3066 | 0.215 | 0.5614 | Yes |
| 185 | DLC1 |  |  | 3135 | 0.212 | 0.5601 | Yes |
| 186 | KIAA1128 |  |  | 3146 | 0.212 | 0.5616 | Yes |
| 187 | DLEU2 |  |  | 3161 | 0.211 | 0.5629 | Yes |
| 188 | CTGF |  |  | 3167 | 0.211 | 0.5647 | Yes |
| 189 | CREB3L2 |  |  | 3169 | 0.211 | 0.5667 | Yes |
| 190 | IBTK |  |  | 3191 | 0.209 | 0.5676 | Yes |
| 191 | KPNA3 |  |  | 3192 | 0.209 | 0.5696 | Yes |
| 192 | CUL2 |  |  | 3195 | 0.209 | 0.5715 | Yes |
| 193 | SSBP2 |  |  | 3203 | 0.209 | 0.5732 | Yes |
| 194 | IFRD1 |  |  | 3206 | 0.209 | 0.5751 | Yes |
| 195 | XPO6 |  |  | 3230 | 0.207 | 0.5759 | Yes |
| 196 | STK3 |  |  | 3287 | 0.204 | 0.5751 | Yes |
| 197 | TOP2A |  |  | 3342 | 0.202 | 0.5743 | Yes |
| 198 | CENTD1 |  |  | 3424 | 0.199 | 0.5722 | Yes |
| 199 | CD2AP |  |  | 3440 | 0.198 | 0.5733 | Yes |
| 200 | SEMA3C |  |  | 3448 | 0.197 | 0.5749 | Yes |
| 201 | APRIN |  |  | 3465 | 0.196 | 0.5760 | Yes |
| 202 | KIF14 |  |  | 3480 | 0.195 | 0.5771 | Yes |
| 203 | MICAL3 |  |  | 3513 | 0.193 | 0.5774 | Yes |
| 204 | ARIH1 |  |  | 3514 | 0.193 | 0.5792 | Yes |
| 205 | ORC2L |  |  | 3532 | 0.192 | 0.5802 | Yes |
| 206 | USP6 |  |  | 3542 | 0.192 | 0.5816 | Yes |
| 207 | KIAA0241 |  |  | 3545 | 0.192 | 0.5833 | Yes |
| 208 | USP15 |  |  | 3595 | 0.190 | 0.5827 | Yes |
| 209 | SRPK2 |  |  | 3614 | 0.189 | 0.5836 | Yes |
| 210 | AKAP9 |  |  | 3620 | 0.189 | 0.5852 | Yes |
| 211 | DOCK4 |  |  | 3631 | 0.189 | 0.5865 | Yes |
| 212 | CTBP2 |  |  | 3652 | 0.189 | 0.5873 | Yes |
| 213 | TRIM37 |  |  | 3675 | 0.188 | 0.5880 | Yes |
| 214 | NUP153 |  |  | 3690 | 0.187 | 0.5891 | Yes |
| 215 | SLK |  |  | 3744 | 0.185 | 0.5883 | Yes |
| 216 | SPEN |  |  | 3757 | 0.184 | 0.5894 | Yes |
| 217 | RAB6A |  |  | 3795 | 0.183 | 0.5893 | Yes |
| 218 | ITSN2 |  |  | 3803 | 0.182 | 0.5907 | Yes |
| 219 | CENPC1 |  |  | 3842 | 0.181 | 0.5906 | Yes |
| 220 | DCUN1D4 |  |  | 3851 | 0.180 | 0.5919 | Yes |
| 221 | HELZ |  |  | 3857 | 0.180 | 0.5934 | Yes |
| 222 | DOC1 |  |  | 3895 | 0.178 | 0.5932 | Yes |
| 223 | PTX3 |  |  | 3912 | 0.177 | 0.5941 | Yes |
| 224 | TRAM2 |  |  | 3969 | 0.175 | 0.5930 | Yes |
| 225 | RBM16 |  |  | 3972 | 0.175 | 0.5946 | Yes |
| 226 | SCC-112 |  |  | 3984 | 0.174 | 0.5957 | Yes |
| 227 | NMT2 |  |  | 4000 | 0.174 | 0.5966 | Yes |
| 228 | LARP5 |  |  | 4002 | 0.174 | 0.5982 | Yes |
| 229 | ANKS1A |  |  | 4038 | 0.172 | 0.5981 | Yes |
| 230 | TIAM1 |  |  | 4045 | 0.172 | 0.5995 | Yes |
| 231 | CREBBP |  |  | 4050 | 0.171 | 0.6009 | Yes |
| 232 | MPHOSPH1 |  |  | 4133 | 0.167 | 0.5984 | Yes |
| 233 | CEP170 |  |  | 4144 | 0.167 | 0.5995 | Yes |
| 234 | ARHGEF10 |  |  | 4156 | 0.167 | 0.6006 | Yes |
| 235 | DKFZP586P0123 |  |  | 4231 | 0.164 | 0.5985 | Yes |
| 236 | RAD23B |  |  | 4243 | 0.163 | 0.5995 | Yes |
| 237 | UBE2D2 |  |  | 4263 | 0.163 | 0.6001 | Yes |
| 238 | SOS2 |  |  | 4297 | 0.161 | 0.6000 | Yes |
| 239 | SLC25A12 |  |  | 4332 | 0.160 | 0.5998 | Yes |
| 240 | LYN |  |  | 4337 | 0.160 | 0.6012 | Yes |
| 241 | ORC5L |  |  | 4368 | 0.158 | 0.6012 | Yes |
| 242 | IGF1R |  |  | 4531 | 0.153 | 0.5946 | No |
| 243 | PTENP1 |  |  | 4534 | 0.153 | 0.5960 | No |
| 244 | SKIV2L2 |  |  | 4616 | 0.150 | 0.5934 | No |
| 245 | FNDC3A |  |  | 4710 | 0.147 | 0.5902 | No |
| 246 | BAZ1A |  |  | 4753 | 0.145 | 0.5895 | No |
| 247 | PPP2R5C |  |  | 4755 | 0.145 | 0.5908 | No |
| 248 | MARCH6 |  |  | 4787 | 0.144 | 0.5906 | No |
| 249 | HEG1 |  |  | 4833 | 0.143 | 0.5898 | No |
| 250 | PARD3 |  |  | 4848 | 0.142 | 0.5904 | No |
| 251 | MKLN1 |  |  | 4874 | 0.141 | 0.5905 | No |
| 252 | PTPN2 |  |  | 4886 | 0.141 | 0.5913 | No |
| 253 | ARHGEF7 |  |  | 4910 | 0.140 | 0.5915 | No |
| 254 | TBL1X |  |  | 4946 | 0.139 | 0.5911 | No |
| 255 | PPP2R5E |  |  | 4950 | 0.139 | 0.5923 | No |
| 256 | IL6 |  |  | 4954 | 0.138 | 0.5935 | No |
| 257 | HERC4 |  |  | 4976 | 0.138 | 0.5938 | No |
| 258 | NUMB |  |  | 4980 | 0.138 | 0.5949 | No |
| 259 | RGS7 |  |  | 5006 | 0.137 | 0.5950 | No |
| 260 | NUP98 |  |  | 5012 | 0.137 | 0.5961 | No |
| 261 | ATP8B1 |  |  | 5024 | 0.137 | 0.5968 | No |
| 262 | MYO1B |  |  | 5116 | 0.135 | 0.5936 | No |
| 263 | CDC42BPA |  |  | 5135 | 0.134 | 0.5940 | No |
| 264 | EDD1 |  |  | 5233 | 0.131 | 0.5904 | No |
| 265 | NCOA3 |  |  | 5249 | 0.131 | 0.5909 | No |
| 266 | PRKACB |  |  | 5252 | 0.130 | 0.5921 | No |
| 267 | TRAK1 |  |  | 5257 | 0.130 | 0.5931 | No |
| 268 | KIAA0802 |  |  | 5283 | 0.130 | 0.5931 | No |
| 269 | KIAA0528 |  |  | 5307 | 0.129 | 0.5932 | No |
| 270 | KIAA0376 |  |  | 5351 | 0.128 | 0.5923 | No |
| 271 | PHF21A |  |  | 5397 | 0.127 | 0.5913 | No |
| 272 | XPO1 |  |  | 5413 | 0.126 | 0.5917 | No |
| 273 | RBM39 |  |  | 5485 | 0.124 | 0.5894 | No |
| 274 | SLC25A13 |  |  | 5507 | 0.124 | 0.5895 | No |
| 275 | PHLPP |  |  | 5510 | 0.124 | 0.5906 | No |
| 276 | ADCY9 |  |  | 5523 | 0.124 | 0.5912 | No |
| 277 | DDX10 |  |  | 5590 | 0.122 | 0.5891 | No |
| 278 | HMG2L1 |  |  | 5620 | 0.121 | 0.5888 | No |
| 279 | R3HDM1 |  |  | 5649 | 0.120 | 0.5885 | No |
| 280 | CLASP2 |  |  | 5665 | 0.120 | 0.5889 | No |
| 281 | DPYD |  |  | 5686 | 0.120 | 0.5891 | No |
| 282 | XRCC4 |  |  | 5692 | 0.119 | 0.5900 | No |
| 283 | MECP2 |  |  | 5751 | 0.118 | 0.5882 | No |
| 284 | TLK2 |  |  | 5757 | 0.118 | 0.5891 | No |
| 285 | ZNF148 |  |  | 5767 | 0.118 | 0.5898 | No |
| 286 | BARD1 |  |  | 5810 | 0.117 | 0.5888 | No |
| 287 | NFYC |  |  | 5860 | 0.116 | 0.5875 | No |
| 288 | DNMBP |  |  | 5861 | 0.116 | 0.5886 | No |
| 289 | DHX15 |  |  | 5867 | 0.116 | 0.5895 | No |
| 290 | PLCB4 |  |  | 5873 | 0.116 | 0.5903 | No |
| 291 | OXSR1 |  |  | 5888 | 0.115 | 0.5907 | No |
| 292 | STIL |  |  | 5918 | 0.115 | 0.5904 | No |
| 293 | DOCK9 |  |  | 5923 | 0.114 | 0.5913 | No |
| 294 | INTS3 |  |  | 5968 | 0.113 | 0.5902 | No |
| 295 | CNOT4 |  |  | 5973 | 0.113 | 0.5911 | No |
| 296 | MYST3 |  |  | 5997 | 0.113 | 0.5910 | No |
| 297 | PIP5K3 |  |  | 6008 | 0.113 | 0.5916 | No |
| 298 | CHD1 |  |  | 6055 | 0.112 | 0.5904 | No |
| 299 | DDEF2 |  |  | 6066 | 0.111 | 0.5909 | No |
| 300 | UTP18 |  |  | 6071 | 0.111 | 0.5918 | No |
| 301 | EHBP1 |  |  | 6094 | 0.111 | 0.5917 | No |
| 302 | INPP5F |  |  | 6106 | 0.110 | 0.5923 | No |
| 303 | PPP1R12A |  |  | 6133 | 0.110 | 0.5920 | No |
| 304 | FYN |  |  | 6142 | 0.110 | 0.5927 | No |
| 305 | GBE1 |  |  | 6178 | 0.109 | 0.5920 | No |
| 306 | GNAQ |  |  | 6212 | 0.108 | 0.5913 | No |
| 307 | KIAA0427 |  |  | 6244 | 0.107 | 0.5908 | No |
| 308 | ASXL1 |  |  | 6269 | 0.106 | 0.5907 | No |
| 309 | RFTN1 |  |  | 6275 | 0.106 | 0.5914 | No |
| 310 | SATB2 |  |  | 6369 | 0.104 | 0.5878 | No |
| 311 | MYC |  |  | 6440 | 0.103 | 0.5853 | No |
| 312 | PUM2 |  |  | 6445 | 0.102 | 0.5861 | No |
| 313 | HIVEP1 |  |  | 6464 | 0.102 | 0.5862 | No |
| 314 | NAV3 |  |  | 6515 | 0.101 | 0.5847 | No |
| 315 | KIAA0182 |  |  | 6542 | 0.101 | 0.5843 | No |
| 316 | SWAP70 |  |  | 6576 | 0.100 | 0.5836 | No |
| 317 | FBXL7 |  |  | 6656 | 0.098 | 0.5806 | No |
| 318 | CREB1 |  |  | 6802 | 0.095 | 0.5744 | No |
| 319 | ZHX3 |  |  | 6811 | 0.095 | 0.5749 | No |
| 320 | PRR3 |  |  | 6847 | 0.094 | 0.5740 | No |
| 321 | EIF4G3 |  |  | 6929 | 0.092 | 0.5709 | No |
| 322 | MYO9B |  |  | 6949 | 0.092 | 0.5708 | No |
| 323 | UBXD7 |  |  | 6961 | 0.092 | 0.5712 | No |
| 324 | PTPN12 |  |  | 6991 | 0.091 | 0.5706 | No |
| 325 | RRAS2 |  |  | 7000 | 0.091 | 0.5711 | No |
| 326 | KIAA0020 |  |  | 7029 | 0.090 | 0.5705 | No |
| 327 | MYST4 |  |  | 7079 | 0.089 | 0.5690 | No |
| 328 | KIAA0922 |  |  | 7082 | 0.089 | 0.5697 | No |
| 329 | C2ORF10 |  |  | 7112 | 0.089 | 0.5691 | No |
| 330 | HOMER1 |  |  | 7141 | 0.088 | 0.5686 | No |
| 331 | CENTG2 |  |  | 7174 | 0.088 | 0.5678 | No |
| 332 | MPHOSPH9 |  |  | 7197 | 0.088 | 0.5676 | No |
| 333 | KIAA0350 |  |  | 7245 | 0.087 | 0.5661 | No |
| 334 | ARHGAP12 |  |  | 7286 | 0.086 | 0.5649 | No |
| 335 | GLRB |  |  | 7331 | 0.085 | 0.5635 | No |
| 336 | C5ORF13 |  |  | 7342 | 0.085 | 0.5638 | No |
| 337 | ATRN |  |  | 7352 | 0.085 | 0.5642 | No |
| 338 | FARS2 |  |  | 7373 | 0.084 | 0.5640 | No |
| 339 | CBLB |  |  | 7419 | 0.083 | 0.5626 | No |
| 340 | RGS20 |  |  | 7558 | 0.081 | 0.5565 | No |
| 341 | PBX3 |  |  | 7567 | 0.081 | 0.5569 | No |
| 342 | MAN2A1 |  |  | 7584 | 0.080 | 0.5568 | No |
| 343 | CLOCK |  |  | 7607 | 0.080 | 0.5565 | No |
| 344 | AUH |  |  | 7640 | 0.079 | 0.5557 | No |
| 345 | PLCE1 |  |  | 7685 | 0.078 | 0.5542 | No |
| 346 | GRLF1 |  |  | 7693 | 0.078 | 0.5546 | No |
| 347 | PRKCA |  |  | 7718 | 0.077 | 0.5542 | No |
| 348 | ZFHX1B |  |  | 7962 | 0.073 | 0.5428 | No |
| 349 | NFKB1 |  |  | 7978 | 0.073 | 0.5428 | No |
| 350 | ATXN2 |  |  | 8014 | 0.073 | 0.5417 | No |
| 351 | GPATC8 |  |  | 8017 | 0.072 | 0.5423 | No |
| 352 | NRG1 |  |  | 8035 | 0.072 | 0.5422 | No |
| 353 | USP24 |  |  | 8116 | 0.071 | 0.5389 | No |
| 354 | SLC4A7 |  |  | 8221 | 0.069 | 0.5344 | No |
| 355 | PRKCBP1 |  |  | 8234 | 0.069 | 0.5344 | No |
| 356 | TSPAN5 |  |  | 8279 | 0.068 | 0.5329 | No |
| 357 | RYK |  |  | 8314 | 0.067 | 0.5318 | No |
| 358 | PKD2 |  |  | 8323 | 0.067 | 0.5321 | No |
| 359 | BTRC |  |  | 8340 | 0.067 | 0.5319 | No |
| 360 | CASK |  |  | 8354 | 0.067 | 0.5319 | No |
| 361 | TGFBR3 |  |  | 8482 | 0.065 | 0.5262 | No |
| 362 | C4ORF8 |  |  | 8502 | 0.064 | 0.5259 | No |
| 363 | KIF2A |  |  | 8603 | 0.063 | 0.5215 | No |
| 364 | ANKMY2 |  |  | 8633 | 0.063 | 0.5207 | No |
| 365 | NUP133 |  |  | 8752 | 0.061 | 0.5154 | No |
| 366 | PCNT |  |  | 8810 | 0.060 | 0.5132 | No |
| 367 | RAI17 |  |  | 8832 | 0.060 | 0.5127 | No |
| 368 | PEX3 |  |  | 8930 | 0.058 | 0.5084 | No |
| 369 | PVRL3 |  |  | 8950 | 0.058 | 0.5080 | No |
| 370 | KIAA1240 |  |  | 9001 | 0.057 | 0.5061 | No |
| 371 | BMPR1A |  |  | 9101 | 0.055 | 0.5017 | No |
| 372 | MARK3 |  |  | 9253 | 0.053 | 0.4947 | No |
| 373 | MELK |  |  | 9282 | 0.053 | 0.4938 | No |
| 374 | ZHX2 |  |  | 9402 | 0.051 | 0.4884 | No |
| 375 | PIK3C3 |  |  | 9468 | 0.050 | 0.4856 | No |
| 376 | TCF12 |  |  | 9479 | 0.050 | 0.4856 | No |
| 377 | BAZ2B |  |  | 9510 | 0.050 | 0.4846 | No |
| 378 | UBR2 |  |  | 9580 | 0.049 | 0.4816 | No |
| 379 | CEP135 |  |  | 9645 | 0.048 | 0.4789 | No |
| 380 | DNAJC13 |  |  | 9732 | 0.046 | 0.4751 | No |
| 381 | CRADD |  |  | 9849 | 0.044 | 0.4697 | No |
| 382 | FRYL |  |  | 9894 | 0.044 | 0.4680 | No |
| 383 | LARS2 |  |  | 9901 | 0.043 | 0.4681 | No |
| 384 | NCOA1 |  |  | 9947 | 0.043 | 0.4663 | No |
| 385 | STARD13 |  |  | 9948 | 0.043 | 0.4667 | No |
| 386 | ARFGEF1 |  |  | 10047 | 0.041 | 0.4622 | No |
| 387 | IL7R |  |  | 10261 | 0.038 | 0.4520 | No |
| 388 | CDK8 |  |  | 10429 | 0.036 | 0.4440 | No |
| 389 | PHF3 |  |  | 10469 | 0.035 | 0.4424 | No |
| 390 | GNAI1 |  |  | 10557 | 0.034 | 0.4384 | No |
| 391 | SLIT2 |  |  | 10587 | 0.033 | 0.4373 | No |
| 392 | ABCE1 |  |  | 10660 | 0.032 | 0.4340 | No |
| 393 | WAPAL |  |  | 10750 | 0.031 | 0.4299 | No |
| 394 | NPEPPS |  |  | 10831 | 0.030 | 0.4262 | No |
| 395 | LASS6 |  |  | 11028 | 0.027 | 0.4167 | No |
| 396 | TP53BP2 |  |  | 11080 | 0.026 | 0.4145 | No |
| 397 | RNGTT |  |  | 11156 | 0.025 | 0.4110 | No |
| 398 | ADAM10 |  |  | 11232 | 0.025 | 0.4075 | No |
| 399 | PRIM2A |  |  | 11241 | 0.025 | 0.4073 | No |
| 400 | MTX2 |  |  | 11433 | 0.021 | 0.3980 | No |
| 401 | HIRA |  |  | 11500 | 0.021 | 0.3950 | No |
| 402 | ITSN1 |  |  | 11613 | 0.019 | 0.3896 | No |
| 403 | EPHA4 |  |  | 11620 | 0.019 | 0.3895 | No |
| 404 | PCCA |  |  | 11658 | 0.018 | 0.3878 | No |
| 405 | KIAA1199 |  |  | 11680 | 0.018 | 0.3869 | No |
| 406 | CYR61 |  |  | 11770 | 0.017 | 0.3827 | No |
| 407 | BLM |  |  | 11778 | 0.017 | 0.3825 | No |
| 408 | RPGR |  |  | 11924 | 0.015 | 0.3754 | No |
| 409 | C21ORF25 |  |  | 11943 | 0.015 | 0.3747 | No |
| 410 | STAG1 |  |  | 12080 | 0.013 | 0.3680 | No |
| 411 | FOXK2 |  |  | 12281 | 0.010 | 0.3582 | No |
| 412 | CDC27 |  |  | 12319 | 0.009 | 0.3564 | No |
| 413 | E2F5 |  |  | 12422 | 0.008 | 0.3514 | No |
| 414 | BHLHB2 |  |  | 12438 | 0.008 | 0.3508 | No |
| 415 | SUMO4 |  |  | 12700 | 0.003 | 0.3378 | No |
| 416 | TOPBP1 |  |  | 12733 | 0.002 | 0.3363 | No |
| 417 | STAM |  |  | 12754 | 0.002 | 0.3353 | No |
| 418 | PRKCI |  |  | 12982 | -0.001 | 0.3240 | No |
| 419 | RCOR1 |  |  | 12990 | -0.001 | 0.3237 | No |
| 420 | TDRD7 |  |  | 13023 | -0.001 | 0.3221 | No |
| 421 | KIAA0280 |  |  | 13106 | -0.003 | 0.3181 | No |
| 422 | PARN |  |  | 13134 | -0.003 | 0.3168 | No |
| 423 | HNRPH3 |  |  | 13305 | -0.006 | 0.3084 | No |
| 424 | UPF2 |  |  | 13306 | -0.006 | 0.3084 | No |
| 425 | AHDC1 |  |  | 13379 | -0.007 | 0.3049 | No |
| 426 | KIF5C |  |  | 13415 | -0.007 | 0.3032 | No |
| 427 | WDR7 |  |  | 13545 | -0.009 | 0.2969 | No |
| 428 | ACVR1 |  |  | 13608 | -0.010 | 0.2939 | No |
| 429 | CENPE |  |  | 13614 | -0.010 | 0.2938 | No |
| 430 | TAF4 |  |  | 13934 | -0.015 | 0.2781 | No |
| 431 | WASF3 |  |  | 13952 | -0.015 | 0.2774 | No |
| 432 | STK39 |  |  | 14174 | -0.019 | 0.2666 | No |
| 433 | DOPEY2 |  |  | 14242 | -0.020 | 0.2634 | No |
| 434 | GTF2F2 |  |  | 14316 | -0.021 | 0.2600 | No |
| 435 | CXCL12 |  |  | 14323 | -0.021 | 0.2599 | No |
| 436 | UBL3 |  |  | 14647 | -0.026 | 0.2441 | No |
| 437 | CXORF6 |  |  | 14833 | -0.029 | 0.2352 | No |
| 438 | LARP4 |  |  | 15021 | -0.032 | 0.2262 | No |
| 439 | UCK2 |  |  | 15072 | -0.033 | 0.2240 | No |
| 440 | MAP2K4 |  |  | 15096 | -0.033 | 0.2232 | No |
| 441 | CKAP5 |  |  | 15202 | -0.035 | 0.2183 | No |
| 442 | FGF5 |  |  | 15342 | -0.037 | 0.2118 | No |
| 443 | KIAA0888 |  |  | 15796 | -0.045 | 0.1897 | No |
| 444 | KIF11 |  |  | 15959 | -0.048 | 0.1821 | No |
| 445 | PMM2 |  |  | 16072 | -0.050 | 0.1770 | No |
| 446 | KIAA0423 |  |  | 16104 | -0.050 | 0.1760 | No |
| 447 | WNT5A |  |  | 16212 | -0.053 | 0.1711 | No |
| 448 | GRK5 |  |  | 16307 | -0.055 | 0.1670 | No |
| 449 | C10ORF38 |  |  | 16379 | -0.056 | 0.1640 | No |
| 450 | NEDD4 |  |  | 16536 | -0.060 | 0.1568 | No |
| 451 | NVL |  |  | 16763 | -0.064 | 0.1462 | No |
| 452 | RB1 |  |  | 16764 | -0.064 | 0.1468 | No |
| 453 | THBS2 |  |  | 16783 | -0.065 | 0.1466 | No |
| 454 | TRRAP |  |  | 16986 | -0.070 | 0.1372 | No |
| 455 | ZFYVE9 |  |  | 17155 | -0.074 | 0.1296 | No |
| 456 | TERF1 |  |  | 17257 | -0.076 | 0.1253 | No |
| 457 | USP9X |  |  | 17313 | -0.078 | 0.1233 | No |
| 458 | PHLPPL |  |  | 17330 | -0.079 | 0.1232 | No |
| 459 | AFAP |  |  | 17366 | -0.080 | 0.1223 | No |
| 460 | MGLL |  |  | 17408 | -0.081 | 0.1210 | No |
| 461 | FGF2 |  |  | 17847 | -0.094 | 0.1002 | No |
| 462 | MSH2 |  |  | 17995 | -0.099 | 0.0938 | No |
| 463 | ROCK2 |  |  | 18023 | -0.100 | 0.0934 | No |
| 464 | VEGFC |  |  | 18210 | -0.106 | 0.0852 | No |
| 465 | SERPINE1 |  |  | 18412 | -0.114 | 0.0763 | No |
| 466 | FUT8 |  |  | 18841 | -0.133 | 0.0563 | No |
| 467 | USP13 |  |  | 18919 | -0.137 | 0.0538 | No |
| 468 | RAD51C |  |  | 19206 | -0.154 | 0.0410 | No |
| 469 | MAP2K1 |  |  | 19255 | -0.157 | 0.0401 | No |
| 470 | DUSP5 |  |  | 19722 | -0.202 | 0.0189 | No |
| 471 | GMPS |  |  | 19753 | -0.206 | 0.0194 | No |
| 472 | KNTC2 |  |  | 19779 | -0.209 | 0.0202 | No |
| 473 | ID1 |  |  | 20093 | -0.262 | 0.0071 | No |
| 474 | HOXB2 |  |  | 20124 | -0.269 | 0.0082 | No |
| 475 | NRIP1 |  |  | 20273 | -0.320 | 0.0039 | No |
| 476 | GSK3B |  |  | 20356 | -0.357 | 0.0032 | No |
| 477 | CENPA |  |  | 20463 | -0.438 | 0.0022 | No |
| 478 | G6PD |  |  | 20527 | -0.507 | 0.0039 | No |
Table: GSEA details [plain text format]

  

Fig 2: UVC\_XPCS\_ALL\_DN: Random ES distribution      
 Gene set null distribution of ES for **UVC\_XPCS\_ALL\_DN**
