# Supplementary material for: The rapamycin-regulated gene expression signature determines prognosis for breast cancer
Source: Mol Cancer. 2009 Sep 24;8:75. doi: 10.1186/1476-4598-8-75 (PMC2761377; doi:10.1186/1476-4598-8-75)
Supplement: Additional file 2 — Gene set enrichment analysis of in vivo data, time series. The data provided represent the time series of GSEA. This compressed file contains "Time" shortcut file and "GSEA_time" folder. Clicking on "Time" shortcut opens the index file providing access to analysis files contained in the "GSEA_time" folder. [file 1476-4598-8-75-S2.zip › GSEA_time/VEGF_HUVEC_30MIN_UP.html]

Details for gene set VEGF\_HUVEC\_30MIN\_UP[GSEA]

|  || Dataset | gsea\_time\_collapsed |
| Phenotype | NoPhenotypeAvailable |
| Upregulated in class | na\_neg |
| GeneSet | VEGF\_HUVEC\_30MIN\_UP |
| Enrichment Score (ES) | -0.41561562 |
| Normalized Enrichment Score (NES) | -1.3992481 |
| Nominal p-value | 0.07692308 |
| FDR q-value | 0.28489694 |
| FWER p-Value | 1.0 |
Table: GSEA Results Summary

  

Fig 1: Enrichment plot: VEGF\_HUVEC\_30MIN\_UP      
 Profile of the Running ES Score & Positions of GeneSet Members on the Rank Ordered List

  

| PROBE | GENE SYMBOL | GENE\_TITLE | RANK IN GENE LIST | RANK METRIC SCORE | RUNNING ES | CORE ENRICHMENT || 1 | APOE |  |  | 2558 | 0.244 | -0.0098 | No |
| 2 | C2 |  |  | 4885 | 0.141 | -0.0568 | No |
| 3 | NR4A1 |  |  | 5383 | 0.127 | -0.0215 | No |
| 4 | EGR1 |  |  | 8018 | 0.072 | -0.1156 | No |
| 5 | DSCR1 |  |  | 9255 | 0.053 | -0.1507 | No |
| 6 | PPY |  |  | 9333 | 0.052 | -0.1301 | No |
| 7 | SCRN1 |  |  | 9570 | 0.049 | -0.1188 | No |
| 8 | HDC |  |  | 9799 | 0.045 | -0.1087 | No |
| 9 | MYO7A |  |  | 11129 | 0.026 | -0.1611 | No |
| 10 | ELAVL4 |  |  | 11386 | 0.022 | -0.1632 | No |
| 11 | DBC1 |  |  | 11462 | 0.021 | -0.1569 | No |
| 12 | CYR61 |  |  | 11770 | 0.017 | -0.1639 | No |
| 13 | PCDH17 |  |  | 12067 | 0.013 | -0.1722 | No |
| 14 | ALAD |  |  | 13444 | -0.008 | -0.2355 | No |
| 15 | TAC1 |  |  | 13534 | -0.009 | -0.2355 | No |
| 16 | CD1B |  |  | 15353 | -0.038 | -0.3063 | No |
| 17 | GZMK |  |  | 15974 | -0.048 | -0.3139 | No |
| 18 | EGR3 |  |  | 17176 | -0.075 | -0.3373 | No |
| 19 | TKTL1 |  |  | 18789 | -0.130 | -0.3546 | Yes |
| 20 | TRIB1 |  |  | 18829 | -0.132 | -0.2945 | Yes |
| 21 | EGR2 |  |  | 18847 | -0.133 | -0.2329 | Yes |
| 22 | DUSP5 |  |  | 19722 | -0.202 | -0.1805 | Yes |
| 23 | VARS |  |  | 19875 | -0.222 | -0.0838 | Yes |
| 24 | AURKC |  |  | 20059 | -0.254 | 0.0265 | Yes |
Table: GSEA details [plain text format]

  

Fig 2: VEGF\_HUVEC\_30MIN\_UP: Random ES distribution      
 Gene set null distribution of ES for **VEGF\_HUVEC\_30MIN\_UP**
