# Supplementary material for: The rapamycin-regulated gene expression signature determines prognosis for breast cancer
Source: Mol Cancer. 2009 Sep 24;8:75. doi: 10.1186/1476-4598-8-75 (PMC2761377; doi:10.1186/1476-4598-8-75)
Supplement: Additional file 2 — Gene set enrichment analysis of in vivo data, time series. The data provided represent the time series of GSEA. This compressed file contains "Time" shortcut file and "GSEA_time" folder. Clicking on "Time" shortcut opens the index file providing access to analysis files contained in the "GSEA_time" folder. [file 1476-4598-8-75-S2.zip › GSEA_time/VEGF_MMMEC_12HRS_UP.html]

Details for gene set VEGF\_MMMEC\_12HRS\_UP[GSEA]

|  || Dataset | gsea\_time\_collapsed |
| Phenotype | NoPhenotypeAvailable |
| Upregulated in class | na\_pos |
| GeneSet | VEGF\_MMMEC\_12HRS\_UP |
| Enrichment Score (ES) | 0.6844742 |
| Normalized Enrichment Score (NES) | 1.7388452 |
| Nominal p-value | 0.0 |
| FDR q-value | 0.013997789 |
| FWER p-Value | 0.467 |
Table: GSEA Results Summary

  

Fig 1: Enrichment plot: VEGF\_MMMEC\_12HRS\_UP      
 Profile of the Running ES Score & Positions of GeneSet Members on the Rank Ordered List

  

| PROBE | GENE SYMBOL | GENE\_TITLE | RANK IN GENE LIST | RANK METRIC SCORE | RUNNING ES | CORE ENRICHMENT || 1 | CCL2 |  |  | 11 | 1.629 | 0.2074 | Yes |
| 2 | SAT1 |  |  | 58 | 1.024 | 0.3359 | Yes |
| 3 | SDC4 |  |  | 110 | 0.882 | 0.4460 | Yes |
| 4 | MGP |  |  | 412 | 0.574 | 0.5047 | Yes |
| 5 | TFPI2 |  |  | 563 | 0.511 | 0.5627 | Yes |
| 6 | SP3 |  |  | 746 | 0.463 | 0.6129 | Yes |
| 7 | NNMT |  |  | 813 | 0.442 | 0.6661 | Yes |
| 8 | IGFBP3 |  |  | 1528 | 0.326 | 0.6730 | Yes |
| 9 | FGFBP1 |  |  | 2222 | 0.267 | 0.6734 | Yes |
| 10 | BGN |  |  | 2751 | 0.233 | 0.6775 | Yes |
| 11 | DLEU2 |  |  | 3161 | 0.211 | 0.6845 | Yes |
| 12 | SLC6A8 |  |  | 5081 | 0.135 | 0.6085 | No |
| 13 | GEM |  |  | 6405 | 0.103 | 0.5574 | No |
| 14 | OXTR |  |  | 7477 | 0.082 | 0.5158 | No |
| 15 | SULT1E1 |  |  | 7847 | 0.075 | 0.5075 | No |
| 16 | ELN |  |  | 8254 | 0.069 | 0.4965 | No |
| 17 | RHOB |  |  | 8609 | 0.063 | 0.4873 | No |
| 18 | ANKRD1 |  |  | 8664 | 0.062 | 0.4926 | No |
| 19 | THY1 |  |  | 8773 | 0.061 | 0.4951 | No |
| 20 | MMP2 |  |  | 8977 | 0.057 | 0.4926 | No |
| 21 | MEST |  |  | 9084 | 0.056 | 0.4945 | No |
| 22 | VCAM1 |  |  | 15109 | -0.034 | 0.2061 | No |
| 23 | COL6A3 |  |  | 16068 | -0.050 | 0.1659 | No |
| 24 | COL1A2 |  |  | 16667 | -0.063 | 0.1448 | No |
| 25 | FUCA1 |  |  | 16857 | -0.067 | 0.1441 | No |
| 26 | SGCE |  |  | 17372 | -0.080 | 0.1293 | No |
| 27 | AQP7 |  |  | 17951 | -0.097 | 0.1136 | No |
| 28 | DIRC2 |  |  | 18551 | -0.120 | 0.0998 | No |
Table: GSEA details [plain text format]

  

Fig 2: VEGF\_MMMEC\_12HRS\_UP: Random ES distribution      
 Gene set null distribution of ES for **VEGF\_MMMEC\_12HRS\_UP**
