# Supplementary material for: The rapamycin-regulated gene expression signature determines prognosis for breast cancer
Source: Mol Cancer. 2009 Sep 24;8:75. doi: 10.1186/1476-4598-8-75 (PMC2761377; doi:10.1186/1476-4598-8-75)
Supplement: Additional file 2 — Gene set enrichment analysis of in vivo data, time series. The data provided represent the time series of GSEA. This compressed file contains "Time" shortcut file and "GSEA_time" folder. Clicking on "Time" shortcut opens the index file providing access to analysis files contained in the "GSEA_time" folder. [file 1476-4598-8-75-S2.zip › GSEA_time/WNT_TARGETS.html]

Details for gene set WNT\_TARGETS[GSEA]

|  || Dataset | gsea\_time\_collapsed |
| Phenotype | NoPhenotypeAvailable |
| Upregulated in class | na\_pos |
| GeneSet | WNT\_TARGETS |
| Enrichment Score (ES) | 0.79870623 |
| Normalized Enrichment Score (NES) | 1.9857295 |
| Nominal p-value | 0.0 |
| FDR q-value | 0.0030168546 |
| FWER p-Value | 0.0030 |
Table: GSEA Results Summary

  

Fig 1: Enrichment plot: WNT\_TARGETS      
 Profile of the Running ES Score & Positions of GeneSet Members on the Rank Ordered List

  

| PROBE | GENE SYMBOL | GENE\_TITLE | RANK IN GENE LIST | RANK METRIC SCORE | RUNNING ES | CORE ENRICHMENT || 1 | FST |  |  | 19 | 1.450 | 0.1682 | Yes |
| 2 | CLDN1 |  |  | 65 | 1.004 | 0.2830 | Yes |
| 3 | MET |  |  | 171 | 0.770 | 0.3677 | Yes |
| 4 | VEGF |  |  | 252 | 0.681 | 0.4432 | Yes |
| 5 | CCND1 |  |  | 391 | 0.589 | 0.5052 | Yes |
| 6 | CD44 |  |  | 641 | 0.487 | 0.5499 | Yes |
| 7 | BIRC5 |  |  | 745 | 0.463 | 0.5988 | Yes |
| 8 | MMP7 |  |  | 835 | 0.437 | 0.6454 | Yes |
| 9 | FZD7 |  |  | 912 | 0.421 | 0.6908 | Yes |
| 10 | PLAUR |  |  | 1102 | 0.387 | 0.7267 | Yes |
| 11 | JUN |  |  | 1522 | 0.326 | 0.7443 | Yes |
| 12 | PPARD |  |  | 1591 | 0.319 | 0.7783 | Yes |
| 13 | ID2 /// ID2B |  |  | 1876 | 0.294 | 0.7987 | Yes |
| 14 | FOSL1 |  |  | 4191 | 0.165 | 0.7055 | No |
| 15 | FGF18 |  |  | 7676 | 0.078 | 0.5454 | No |
| 16 | FOXN1 |  |  | 7782 | 0.076 | 0.5492 | No |
| 17 | LEF1 |  |  | 9031 | 0.056 | 0.4951 | No |
| 18 | ID2 |  |  | 10102 | 0.040 | 0.4478 | No |
| 19 | IGSF4C |  |  | 10145 | 0.040 | 0.4504 | No |
| 20 | NRCAM |  |  | 10964 | 0.028 | 0.4139 | No |
| 21 | BMP4 |  |  | 11047 | 0.027 | 0.4131 | No |
| 22 | GAST |  |  | 13418 | -0.008 | 0.2988 | No |
| 23 | EFNB1 |  |  | 15683 | -0.043 | 0.1938 | No |
| 24 | MMP26 |  |  | 19328 | -0.163 | 0.0357 | No |
| 25 | ATOH1 |  |  | 19903 | -0.226 | 0.0341 | No |
Table: GSEA details [plain text format]

  

Fig 2: WNT\_TARGETS: Random ES distribution      
 Gene set null distribution of ES for **WNT\_TARGETS**
