# Supplementary material for: The rapamycin-regulated gene expression signature determines prognosis for breast cancer
Source: Mol Cancer. 2009 Sep 24;8:75. doi: 10.1186/1476-4598-8-75 (PMC2761377; doi:10.1186/1476-4598-8-75)
Supplement: Additional file 2 — Gene set enrichment analysis of in vivo data, time series. The data provided represent the time series of GSEA. This compressed file contains "Time" shortcut file and "GSEA_time" folder. Clicking on "Time" shortcut opens the index file providing access to analysis files contained in the "GSEA_time" folder. [file 1476-4598-8-75-S2.zip › GSEA_time/XPB_TTD-CS_DN.html]

Details for gene set XPB\_TTD-CS\_DN[GSEA]

|  || Dataset | gsea\_time\_collapsed |
| Phenotype | NoPhenotypeAvailable |
| Upregulated in class | na\_neg |
| GeneSet | XPB\_TTD-CS\_DN |
| Enrichment Score (ES) | -0.42129174 |
| Normalized Enrichment Score (NES) | -1.420626 |
| Nominal p-value | 0.062068965 |
| FDR q-value | 0.2726287 |
| FWER p-Value | 0.999 |
Table: GSEA Results Summary

  

Fig 1: Enrichment plot: XPB\_TTD-CS\_DN      
 Profile of the Running ES Score & Positions of GeneSet Members on the Rank Ordered List

  

| PROBE | GENE SYMBOL | GENE\_TITLE | RANK IN GENE LIST | RANK METRIC SCORE | RUNNING ES | CORE ENRICHMENT || 1 | ANPEP |  |  | 54 | 1.069 | 0.1486 | No |
| 2 | EFNB2 |  |  | 106 | 0.890 | 0.2721 | No |
| 3 | NNMT |  |  | 813 | 0.442 | 0.3004 | No |
| 4 | TGM2 |  |  | 1588 | 0.320 | 0.3080 | No |
| 5 | NMB |  |  | 2440 | 0.252 | 0.3023 | No |
| 6 | ISG20 |  |  | 3814 | 0.182 | 0.2614 | No |
| 7 | PTX3 |  |  | 3912 | 0.177 | 0.2817 | No |
| 8 | HOXB5 |  |  | 6438 | 0.103 | 0.1736 | No |
| 9 | RIMS2 |  |  | 7374 | 0.084 | 0.1401 | No |
| 10 | IGSF3 |  |  | 9015 | 0.057 | 0.0685 | No |
| 11 | MEST |  |  | 9084 | 0.056 | 0.0730 | No |
| 12 | IGFBP6 |  |  | 9820 | 0.045 | 0.0437 | No |
| 13 | CTSH |  |  | 9994 | 0.042 | 0.0412 | No |
| 14 | E2F5 |  |  | 12422 | 0.008 | -0.0756 | No |
| 15 | EPB41L3 |  |  | 13597 | -0.010 | -0.1312 | No |
| 16 | CXCL12 |  |  | 14323 | -0.021 | -0.1634 | No |
| 17 | PCDH7 |  |  | 14706 | -0.027 | -0.1782 | No |
| 18 | PTPRN |  |  | 16319 | -0.055 | -0.2487 | No |
| 19 | LHX2 |  |  | 17255 | -0.076 | -0.2833 | No |
| 20 | ADORA1 |  |  | 19213 | -0.154 | -0.3565 | Yes |
| 21 | GNG4 |  |  | 20547 | -0.535 | -0.3456 | Yes |
| 22 | RASSF2 |  |  | 20580 | -0.655 | -0.2545 | Yes |
| 23 | MDK |  |  | 20591 | -0.741 | -0.1501 | Yes |
| 24 | IGFBP2 |  |  | 20605 | -1.066 | 0.0000 | Yes |
Table: GSEA details [plain text format]

  

Fig 2: XPB\_TTD-CS\_DN: Random ES distribution      
 Gene set null distribution of ES for **XPB\_TTD-CS\_DN**
