# Supplementary material for: The rapamycin-regulated gene expression signature determines prognosis for breast cancer
Source: Mol Cancer. 2009 Sep 24;8:75. doi: 10.1186/1476-4598-8-75 (PMC2761377; doi:10.1186/1476-4598-8-75)
Supplement: Additional file 2 — Gene set enrichment analysis of in vivo data, time series. The data provided represent the time series of GSEA. This compressed file contains "Time" shortcut file and "GSEA_time" folder. Clicking on "Time" shortcut opens the index file providing access to analysis files contained in the "GSEA_time" folder. [file 1476-4598-8-75-S2.zip › GSEA_time/ZHAN_MM_CD138_HP_VS_REST.html]

Details for gene set ZHAN\_MM\_CD138\_HP\_VS\_REST[GSEA]

|  || Dataset | gsea\_time\_collapsed |
| Phenotype | NoPhenotypeAvailable |
| Upregulated in class | na\_pos |
| GeneSet | ZHAN\_MM\_CD138\_HP\_VS\_REST |
| Enrichment Score (ES) | 0.6712943 |
| Normalized Enrichment Score (NES) | 1.8234591 |
| Nominal p-value | 0.0 |
| FDR q-value | 0.0049713915 |
| FWER p-Value | 0.109 |
Table: GSEA Results Summary

  

Fig 1: Enrichment plot: ZHAN\_MM\_CD138\_HP\_VS\_REST      
 Profile of the Running ES Score & Positions of GeneSet Members on the Rank Ordered List

  

| PROBE | GENE SYMBOL | GENE\_TITLE | RANK IN GENE LIST | RANK METRIC SCORE | RUNNING ES | CORE ENRICHMENT || 1 | CDV3 |  |  | 9 | 1.706 | 0.1264 | Yes |
| 2 | AMIGO2 |  |  | 91 | 0.926 | 0.1913 | Yes |
| 3 | C5ORF26 |  |  | 134 | 0.840 | 0.2517 | Yes |
| 4 | RBM4B |  |  | 282 | 0.666 | 0.2941 | Yes |
| 5 | GNG11 |  |  | 283 | 0.666 | 0.3436 | Yes |
| 6 | GLTSCR2 |  |  | 304 | 0.646 | 0.3907 | Yes |
| 7 | IPO7 |  |  | 593 | 0.502 | 0.4139 | Yes |
| 8 | RPL37 |  |  | 616 | 0.493 | 0.4496 | Yes |
| 9 | STX5 |  |  | 660 | 0.482 | 0.4833 | Yes |
| 10 | MNAB |  |  | 867 | 0.429 | 0.5053 | Yes |
| 11 | FCHSD2 |  |  | 925 | 0.418 | 0.5336 | Yes |
| 12 | PAK1 |  |  | 1156 | 0.378 | 0.5505 | Yes |
| 13 | POLR1D |  |  | 1308 | 0.352 | 0.5693 | Yes |
| 14 | RPS17 /// LOC402057 |  |  | 1611 | 0.317 | 0.5782 | Yes |
| 15 | RPL7A /// RPL7AP8 // |  |  | 1645 | 0.313 | 0.5999 | Yes |
| 16 | RPL13A |  |  | 1777 | 0.302 | 0.6159 | Yes |
| 17 | THG1L |  |  | 2004 | 0.284 | 0.6260 | Yes |
| 18 | SEMA4D |  |  | 2090 | 0.277 | 0.6425 | Yes |
| 19 | ATP5L |  |  | 2417 | 0.253 | 0.6454 | Yes |
| 20 | CSNK1G3 |  |  | 2493 | 0.248 | 0.6602 | Yes |
| 21 | DDX58 |  |  | 2633 | 0.240 | 0.6713 | Yes |
| 22 | KIAA0141 |  |  | 3431 | 0.198 | 0.6473 | No |
| 23 | SNHG7 |  |  | 3451 | 0.197 | 0.6610 | No |
| 24 | ZNF291 |  |  | 4378 | 0.158 | 0.6277 | No |
| 25 | ISL2 |  |  | 4678 | 0.148 | 0.6241 | No |
| 26 | PIP5K1B |  |  | 5339 | 0.128 | 0.6016 | No |
| 27 | OAS2 |  |  | 5619 | 0.121 | 0.5970 | No |
| 28 | XRCC4 |  |  | 5692 | 0.119 | 0.6024 | No |
| 29 | TALDO1 /// HSUP1 |  |  | 5900 | 0.115 | 0.6008 | No |
| 30 | FRZB |  |  | 6156 | 0.109 | 0.5966 | No |
| 31 | F2R |  |  | 6930 | 0.092 | 0.5658 | No |
| 32 | SCYL2 |  |  | 7056 | 0.090 | 0.5664 | No |
| 33 | LOC129293 |  |  | 7110 | 0.089 | 0.5705 | No |
| 34 | FLI1 |  |  | 7539 | 0.081 | 0.5557 | No |
| 35 | CNTN5 |  |  | 9727 | 0.046 | 0.4528 | No |
| 36 | RSAD2 |  |  | 9879 | 0.044 | 0.4487 | No |
| 37 | CCRL2 /// LOC642312 |  |  | 10096 | 0.041 | 0.4412 | No |
| 38 | ELOVL7 |  |  | 10208 | 0.039 | 0.4387 | No |
| 39 | CCDC85A |  |  | 11527 | 0.020 | 0.3761 | No |
| 40 | LAG3 |  |  | 12329 | 0.009 | 0.3378 | No |
| 41 | GTF2F2 |  |  | 14316 | -0.021 | 0.2427 | No |
| 42 | TNFSF10 |  |  | 14451 | -0.023 | 0.2379 | No |
| 43 | GBA3 |  |  | 15463 | -0.039 | 0.1916 | No |
| 44 | SIDT1 |  |  | 17759 | -0.091 | 0.0868 | No |
| 45 | EPHB1 |  |  | 18161 | -0.105 | 0.0751 | No |
| 46 | LAMP3 |  |  | 19114 | -0.147 | 0.0397 | No |
| 47 | CCR5 /// LOC653725 |  |  | 19826 | -0.215 | 0.0211 | No |
| 48 | C11ORF2 |  |  | 19894 | -0.225 | 0.0346 | No |
Table: GSEA details [plain text format]

  

Fig 2: ZHAN\_MM\_CD138\_HP\_VS\_REST: Random ES distribution      
 Gene set null distribution of ES for **ZHAN\_MM\_CD138\_HP\_VS\_REST**
