# Supplementary material for: The rapamycin-regulated gene expression signature determines prognosis for breast cancer
Source: Mol Cancer. 2009 Sep 24;8:75. doi: 10.1186/1476-4598-8-75 (PMC2761377; doi:10.1186/1476-4598-8-75)
Supplement: Additional file 3 — Gene set enrichment analysis of in vivo data, treatment series. The data provided represent the treatment series of GSEA. This compressed file contains "Treatment" shortcut file and "GSEA_treatment" folder. Clicking on "Treatment" shortcut opens the index file providing access to analysis files contained in the "GSEA_treatment" folder. [file 1476-4598-8-75-S3.zip › GSEA_treatment/GSEA.zip!/index.html]

Index for xtools.gsea.GseaPreranked my\_analysis.GseaPreranked.1197924317248

### GSEA Report for Dataset gsea\_treatment

#### Enrichment in phenotype: **na**

- 1227 / 1287 gene sets are upregulated in phenotype **na\_pos**- 755 gene sets are significant at FDR < 25%- 333 gene sets are significantly enriched at nominal pvalue < 1%- 524 gene sets are significantly enriched at nominal pvalue < 5%- Snapshot of enrichment results- Detailed enrichment results in html format- Detailed enrichment results in excel format (tab delimited text)- Guide to interpret results

#### Enrichment in phenotype: **na**

- 60 / 1287 gene sets are upregulated in phenotype **na\_neg**- 24 gene sets are significantly enriched at FDR < 25%- 11 gene sets are significantly enriched at nominal pvalue < 1%- 19 gene sets are significantly enriched at nominal pvalue < 5%- Snapshot of enrichment results- Detailed enrichment results in html format- Detailed enrichment results in excel format (tab delimited text)- Guide to interpret results

#### Dataset details

- The dataset has 54675 native features- After collapsing features into gene symbols, there are: 20606 genes

#### Gene set details

- Gene set size filters (min=15, max=500) resulted in filtering out 400 / 1687 gene sets- The remaining 1287 gene sets were used in the analysis- List of gene sets used and their sizes (restricted to features in the specified dataset)

#### Gene markers for the **na\_pos** *versus* **na\_neg** comparison

- The dataset has 20606 features (genes)- Detailed rank ordered gene list for all features in the dataset

#### Global statistics and plots

- Plot of p-values *vs.* NES- Global ES histogram

#### Other

- Parameters used for this analysis

---

Report: my\_analysis.GseaPreranked.1197924317248.rpt   by user: spy

xtools.gsea.GseaPreranked [Mon, Dec 17, '07 2 PM 45]

Website: www.broad.mit.edu/GSEA
Questions & Suggestions: Email
