# Supplementary material for: The rapamycin-regulated gene expression signature determines prognosis for breast cancer
Source: Mol Cancer. 2009 Sep 24;8:75. doi: 10.1186/1476-4598-8-75 (PMC2761377; doi:10.1186/1476-4598-8-75)
Supplement: Additional file 3 — Gene set enrichment analysis of in vivo data, treatment series. The data provided represent the treatment series of GSEA. This compressed file contains "Treatment" shortcut file and "GSEA_treatment" folder. Clicking on "Treatment" shortcut opens the index file providing access to analysis files contained in the "GSEA_treatment" folder. [file 1476-4598-8-75-S3.zip › GSEA_treatment/HDACI_COLON_BUT30MIN_DN.html]

Details for gene set HDACI\_COLON\_BUT30MIN\_DN[GSEA]

|  || Dataset | gsea\_treatment\_collapsed |
| Phenotype | NoPhenotypeAvailable |
| Upregulated in class | na\_pos |
| GeneSet | HDACI\_COLON\_BUT30MIN\_DN |
| Enrichment Score (ES) | 0.63186246 |
| Normalized Enrichment Score (NES) | 1.7197202 |
| Nominal p-value | 0.0010341262 |
| FDR q-value | 0.010258512 |
| FWER p-Value | 0.317 |
Table: GSEA Results Summary

  

Fig 1: Enrichment plot: HDACI\_COLON\_BUT30MIN\_DN      
 Profile of the Running ES Score & Positions of GeneSet Members on the Rank Ordered List

  

| PROBE | GENE SYMBOL | GENE\_TITLE | RANK IN GENE LIST | RANK METRIC SCORE | RUNNING ES | CORE ENRICHMENT || 1 | C11ORF46 |  |  | 143 | 0.530 | 0.0673 | Yes |
| 2 | CALD1 |  |  | 177 | 0.506 | 0.1366 | Yes |
| 3 | HMGB1 |  |  | 247 | 0.469 | 0.1989 | Yes |
| 4 | RGL1 |  |  | 420 | 0.421 | 0.2494 | Yes |
| 5 | RBBP6 |  |  | 635 | 0.378 | 0.2919 | Yes |
| 6 | IKIP |  |  | 707 | 0.367 | 0.3399 | Yes |
| 7 | NARG2 |  |  | 1186 | 0.312 | 0.3603 | Yes |
| 8 | TCEAL4 |  |  | 1242 | 0.307 | 0.4006 | Yes |
| 9 | PSEN1 |  |  | 1310 | 0.302 | 0.4396 | Yes |
| 10 | TGOLN2 |  |  | 1472 | 0.288 | 0.4721 | Yes |
| 11 | LARP4 |  |  | 1965 | 0.257 | 0.4841 | Yes |
| 12 | GOLGB1 |  |  | 1993 | 0.255 | 0.5186 | Yes |
| 13 | C18ORF1 |  |  | 2680 | 0.224 | 0.5166 | Yes |
| 14 | HNRPA3 |  |  | 2861 | 0.216 | 0.5380 | Yes |
| 15 | PRKAR1A |  |  | 3195 | 0.203 | 0.5503 | Yes |
| 16 | MRPS31 |  |  | 3320 | 0.200 | 0.5722 | Yes |
| 17 | DLG1 |  |  | 3367 | 0.198 | 0.5977 | Yes |
| 18 | TTC3 |  |  | 3721 | 0.188 | 0.6068 | Yes |
| 19 | FAM49B |  |  | 4002 | 0.179 | 0.6183 | Yes |
| 20 | LAMP2 |  |  | 4224 | 0.174 | 0.6319 | Yes |
| 21 | ITGA6 |  |  | 4923 | 0.157 | 0.6199 | No |
| 22 | MGA |  |  | 6318 | 0.130 | 0.5704 | No |
| 23 | OFD1 |  |  | 6525 | 0.126 | 0.5780 | No |
| 24 | ITGA5 |  |  | 7140 | 0.115 | 0.5643 | No |
| 25 | KIAA0101 |  |  | 7666 | 0.107 | 0.5538 | No |
| 26 | FNDC3B |  |  | 7687 | 0.107 | 0.5678 | No |
| 27 | SEH1L |  |  | 8151 | 0.100 | 0.5592 | No |
| 28 | SCLT1 |  |  | 8162 | 0.099 | 0.5726 | No |
| 29 | MBNL2 |  |  | 11140 | 0.058 | 0.4359 | No |
| 30 | CTNNA1 |  |  | 12390 | 0.042 | 0.3811 | No |
| 31 | EIF4E |  |  | 13536 | 0.027 | 0.3292 | No |
| 32 | PROX1 |  |  | 14148 | 0.019 | 0.3021 | No |
| 33 | CREB3L1 |  |  | 14302 | 0.016 | 0.2969 | No |
| 34 | ZRANB2 |  |  | 14982 | 0.006 | 0.2648 | No |
| 35 | KIAA0828 |  |  | 15577 | -0.003 | 0.2364 | No |
| 36 | GTPBP4 |  |  | 16809 | -0.025 | 0.1800 | No |
| 37 | RIOK1 |  |  | 17173 | -0.032 | 0.1669 | No |
Table: GSEA details [plain text format]

  

Fig 2: HDACI\_COLON\_BUT30MIN\_DN: Random ES distribution      
 Gene set null distribution of ES for **HDACI\_COLON\_BUT30MIN\_DN**
