# Supplementary material for: The rapamycin-regulated gene expression signature determines prognosis for breast cancer
Source: Mol Cancer. 2009 Sep 24;8:75. doi: 10.1186/1476-4598-8-75 (PMC2761377; doi:10.1186/1476-4598-8-75)
Supplement: Additional file 3 — Gene set enrichment analysis of in vivo data, treatment series. The data provided represent the treatment series of GSEA. This compressed file contains "Treatment" shortcut file and "GSEA_treatment" folder. Clicking on "Treatment" shortcut opens the index file providing access to analysis files contained in the "GSEA_treatment" folder. [file 1476-4598-8-75-S3.zip › GSEA_treatment/HDACI_COLON_SUL24HRS_DN.html]

Details for gene set HDACI\_COLON\_SUL24HRS\_DN[GSEA]

|  || Dataset | gsea\_treatment\_collapsed |
| Phenotype | NoPhenotypeAvailable |
| Upregulated in class | na\_pos |
| GeneSet | HDACI\_COLON\_SUL24HRS\_DN |
| Enrichment Score (ES) | 0.5665155 |
| Normalized Enrichment Score (NES) | 1.7288872 |
| Nominal p-value | 0.0 |
| FDR q-value | 0.009559602 |
| FWER p-Value | 0.275 |
Table: GSEA Results Summary

  

Fig 1: Enrichment plot: HDACI\_COLON\_SUL24HRS\_DN      
 Profile of the Running ES Score & Positions of GeneSet Members on the Rank Ordered List

  

| PROBE | GENE SYMBOL | GENE\_TITLE | RANK IN GENE LIST | RANK METRIC SCORE | RUNNING ES | CORE ENRICHMENT || 1 | CCL28 |  |  | 98 | 0.584 | 0.0223 | Yes |
| 2 | NAP1L1 |  |  | 142 | 0.531 | 0.0448 | Yes |
| 3 | TIA1 |  |  | 167 | 0.513 | 0.0674 | Yes |
| 4 | CALD1 |  |  | 177 | 0.506 | 0.0904 | Yes |
| 5 | HMGB1 |  |  | 247 | 0.469 | 0.1088 | Yes |
| 6 | PRKCBP1 |  |  | 294 | 0.454 | 0.1276 | Yes |
| 7 | PPP1CB |  |  | 345 | 0.441 | 0.1455 | Yes |
| 8 | HMGB2 |  |  | 601 | 0.385 | 0.1509 | Yes |
| 9 | RBBP6 |  |  | 635 | 0.378 | 0.1668 | Yes |
| 10 | FAIM |  |  | 644 | 0.377 | 0.1839 | Yes |
| 11 | SFRS1 |  |  | 923 | 0.339 | 0.1860 | Yes |
| 12 | CGNL1 |  |  | 924 | 0.339 | 0.2017 | Yes |
| 13 | ORMDL2 |  |  | 951 | 0.336 | 0.2160 | Yes |
| 14 | LASP1 |  |  | 1010 | 0.330 | 0.2284 | Yes |
| 15 | DHX9 |  |  | 1028 | 0.328 | 0.2428 | Yes |
| 16 | SUV420H1 |  |  | 1146 | 0.316 | 0.2517 | Yes |
| 17 | ABP1 |  |  | 1149 | 0.316 | 0.2663 | Yes |
| 18 | BAT1 |  |  | 1235 | 0.308 | 0.2764 | Yes |
| 19 | DARS |  |  | 1276 | 0.304 | 0.2885 | Yes |
| 20 | UNC5A |  |  | 1294 | 0.303 | 0.3017 | Yes |
| 21 | BCLAF1 |  |  | 1313 | 0.301 | 0.3148 | Yes |
| 22 | CHCHD7 |  |  | 1409 | 0.293 | 0.3237 | Yes |
| 23 | TSNAX |  |  | 1563 | 0.282 | 0.3293 | Yes |
| 24 | CREBBP |  |  | 1584 | 0.280 | 0.3413 | Yes |
| 25 | ERGIC3 |  |  | 1796 | 0.267 | 0.3434 | Yes |
| 26 | ACP1 |  |  | 1846 | 0.264 | 0.3532 | Yes |
| 27 | CDC7 |  |  | 1919 | 0.260 | 0.3617 | Yes |
| 28 | EPB41 |  |  | 1943 | 0.258 | 0.3726 | Yes |
| 29 | C10ORF32 |  |  | 2064 | 0.252 | 0.3784 | Yes |
| 30 | GTF3A |  |  | 2073 | 0.252 | 0.3897 | Yes |
| 31 | UPF3A |  |  | 2087 | 0.251 | 0.4006 | Yes |
| 32 | CRYL1 |  |  | 2178 | 0.246 | 0.4077 | Yes |
| 33 | SEPT3 |  |  | 2245 | 0.243 | 0.4157 | Yes |
| 34 | MAP9 |  |  | 2302 | 0.240 | 0.4241 | Yes |
| 35 | CUL4B |  |  | 2339 | 0.239 | 0.4334 | Yes |
| 36 | TBC1D23 |  |  | 2561 | 0.227 | 0.4331 | Yes |
| 37 | SNX12 |  |  | 2634 | 0.225 | 0.4400 | Yes |
| 38 | PPP1R1B |  |  | 2704 | 0.223 | 0.4470 | Yes |
| 39 | AKAP1 |  |  | 2939 | 0.213 | 0.4454 | Yes |
| 40 | BMP4 |  |  | 2962 | 0.212 | 0.4542 | Yes |
| 41 | FXYD6 |  |  | 2982 | 0.211 | 0.4630 | Yes |
| 42 | RHBDF1 |  |  | 3049 | 0.209 | 0.4695 | Yes |
| 43 | ADD3 |  |  | 3113 | 0.206 | 0.4759 | Yes |
| 44 | VEGFB |  |  | 3232 | 0.202 | 0.4795 | Yes |
| 45 | KHDRBS1 |  |  | 3282 | 0.200 | 0.4864 | Yes |
| 46 | SRA1 |  |  | 3421 | 0.196 | 0.4888 | Yes |
| 47 | IFITM2 |  |  | 3605 | 0.191 | 0.4887 | Yes |
| 48 | ST18 |  |  | 3639 | 0.190 | 0.4959 | Yes |
| 49 | ZNF529 |  |  | 3719 | 0.188 | 0.5007 | Yes |
| 50 | TTC3 |  |  | 3721 | 0.188 | 0.5093 | Yes |
| 51 | SORT1 |  |  | 3791 | 0.186 | 0.5146 | Yes |
| 52 | MAPK4 |  |  | 3860 | 0.183 | 0.5197 | Yes |
| 53 | PAPOLA |  |  | 3905 | 0.182 | 0.5260 | Yes |
| 54 | SFRS11 |  |  | 4134 | 0.176 | 0.5230 | Yes |
| 55 | STRA6 |  |  | 4210 | 0.174 | 0.5274 | Yes |
| 56 | BMP1 |  |  | 4255 | 0.173 | 0.5333 | Yes |
| 57 | TUBB2C |  |  | 4295 | 0.172 | 0.5393 | Yes |
| 58 | APTX |  |  | 4342 | 0.171 | 0.5450 | Yes |
| 59 | HRBL |  |  | 4375 | 0.170 | 0.5513 | Yes |
| 60 | DNAJC19 |  |  | 4377 | 0.170 | 0.5591 | Yes |
| 61 | MSI2 |  |  | 4387 | 0.169 | 0.5665 | Yes |
| 62 | MME |  |  | 4779 | 0.160 | 0.5548 | No |
| 63 | ORMDL1 |  |  | 4792 | 0.160 | 0.5616 | No |
| 64 | A1BG |  |  | 4901 | 0.158 | 0.5637 | No |
| 65 | RNF180 |  |  | 5061 | 0.154 | 0.5630 | No |
| 66 | GNL1 |  |  | 5685 | 0.141 | 0.5391 | No |
| 67 | ADIPOQ |  |  | 5793 | 0.138 | 0.5403 | No |
| 68 | WDR89 |  |  | 5828 | 0.138 | 0.5451 | No |
| 69 | SCML1 |  |  | 5992 | 0.135 | 0.5434 | No |
| 70 | ANK1 |  |  | 6189 | 0.132 | 0.5399 | No |
| 71 | PSMA1 |  |  | 6259 | 0.131 | 0.5426 | No |
| 72 | FLJ20628 |  |  | 6484 | 0.127 | 0.5375 | No |
| 73 | OFD1 |  |  | 6525 | 0.126 | 0.5414 | No |
| 74 | APOC2 |  |  | 6678 | 0.124 | 0.5397 | No |
| 75 | RDX |  |  | 6786 | 0.122 | 0.5401 | No |
| 76 | SEMA3A |  |  | 6852 | 0.121 | 0.5425 | No |
| 77 | LRIG2 |  |  | 7199 | 0.114 | 0.5310 | No |
| 78 | GPLD1 |  |  | 7626 | 0.108 | 0.5151 | No |
| 79 | CHI3L2 |  |  | 8014 | 0.101 | 0.5009 | No |
| 80 | QRSL1 |  |  | 8303 | 0.097 | 0.4914 | No |
| 81 | CACNA1D |  |  | 8422 | 0.095 | 0.4900 | No |
| 82 | MYB |  |  | 8473 | 0.094 | 0.4920 | No |
| 83 | BAIAP2 |  |  | 9276 | 0.083 | 0.4567 | No |
| 84 | C9ORF85 |  |  | 9292 | 0.083 | 0.4598 | No |
| 85 | PTPRM |  |  | 9369 | 0.082 | 0.4598 | No |
| 86 | PCF11 |  |  | 9848 | 0.076 | 0.4400 | No |
| 87 | SGCD |  |  | 10004 | 0.073 | 0.4358 | No |
| 88 | SLC7A8 |  |  | 10253 | 0.070 | 0.4269 | No |
| 89 | C10ORF46 |  |  | 10710 | 0.063 | 0.4076 | No |
| 90 | COL9A1 |  |  | 10841 | 0.062 | 0.4041 | No |
| 91 | BTK |  |  | 10976 | 0.060 | 0.4003 | No |
| 92 | SESTD1 |  |  | 11388 | 0.055 | 0.3828 | No |
| 93 | SAMD4A |  |  | 11435 | 0.054 | 0.3831 | No |
| 94 | TMEM63C |  |  | 12031 | 0.046 | 0.3562 | No |
| 95 | C2 |  |  | 12568 | 0.039 | 0.3318 | No |
| 96 | PRKCA |  |  | 12613 | 0.039 | 0.3315 | No |
| 97 | SLIT3 |  |  | 12668 | 0.038 | 0.3306 | No |
| 98 | TPM3 |  |  | 13045 | 0.033 | 0.3138 | No |
| 99 | CDH11 |  |  | 13551 | 0.027 | 0.2904 | No |
| 100 | STAT5A |  |  | 13555 | 0.027 | 0.2915 | No |
| 101 | PLCB2 |  |  | 13671 | 0.025 | 0.2870 | No |
| 102 | GALNT5 |  |  | 13717 | 0.025 | 0.2860 | No |
| 103 | ISOC2 |  |  | 13760 | 0.024 | 0.2850 | No |
| 104 | PPIB |  |  | 13763 | 0.024 | 0.2860 | No |
| 105 | LIPC |  |  | 14029 | 0.020 | 0.2740 | No |
| 106 | CCDC14 |  |  | 14038 | 0.020 | 0.2745 | No |
| 107 | PDSS2 |  |  | 14134 | 0.019 | 0.2708 | No |
| 108 | AASS |  |  | 14250 | 0.017 | 0.2659 | No |
| 109 | AQP1 |  |  | 14264 | 0.017 | 0.2661 | No |
| 110 | VIL1 |  |  | 14613 | 0.012 | 0.2496 | No |
| 111 | IL16 |  |  | 14614 | 0.012 | 0.2502 | No |
| 112 | ITM2A |  |  | 14817 | 0.009 | 0.2407 | No |
| 113 | DPYSL3 |  |  | 14893 | 0.008 | 0.2374 | No |
| 114 | FER1L3 |  |  | 15587 | -0.003 | 0.2037 | No |
| 115 | ABCC6 |  |  | 15619 | -0.004 | 0.2024 | No |
| 116 | NEUROD6 |  |  | 15741 | -0.006 | 0.1968 | No |
| 117 | CACNA2D4 |  |  | 16199 | -0.013 | 0.1751 | No |
| 118 | LTBP2 |  |  | 16524 | -0.019 | 0.1601 | No |
| 119 | EEF1G |  |  | 16706 | -0.023 | 0.1524 | No |
| 120 | SDHB |  |  | 16716 | -0.024 | 0.1530 | No |
| 121 | C14ORF37 |  |  | 17023 | -0.029 | 0.1394 | No |
| 122 | LOC284998 |  |  | 17211 | -0.033 | 0.1318 | No |
| 123 | PVALB |  |  | 17537 | -0.041 | 0.1179 | No |
| 124 | LRP8 |  |  | 17774 | -0.046 | 0.1085 | No |
| 125 | KIAA0114 |  |  | 20099 | -0.156 | 0.0022 | No |
| 126 | FOS |  |  | 20334 | -0.202 | 0.0002 | No |
| 127 | KRT23 |  |  | 20480 | -0.280 | 0.0061 | No |
Table: GSEA details [plain text format]

  

Fig 2: HDACI\_COLON\_SUL24HRS\_DN: Random ES distribution      
 Gene set null distribution of ES for **HDACI\_COLON\_SUL24HRS\_DN**
