# Supplementary material for: The rapamycin-regulated gene expression signature determines prognosis for breast cancer
Source: Mol Cancer. 2009 Sep 24;8:75. doi: 10.1186/1476-4598-8-75 (PMC2761377; doi:10.1186/1476-4598-8-75)
Supplement: Additional file 3 — Gene set enrichment analysis of in vivo data, treatment series. The data provided represent the treatment series of GSEA. This compressed file contains "Treatment" shortcut file and "GSEA_treatment" folder. Clicking on "Treatment" shortcut opens the index file providing access to analysis files contained in the "GSEA_treatment" folder. [file 1476-4598-8-75-S3.zip › GSEA_treatment/HDACI_COLON_SUL_DN.html]

Details for gene set HDACI\_COLON\_SUL\_DN[GSEA]

|  || Dataset | gsea\_treatment\_collapsed |
| Phenotype | NoPhenotypeAvailable |
| Upregulated in class | na\_pos |
| GeneSet | HDACI\_COLON\_SUL\_DN |
| Enrichment Score (ES) | 0.5433744 |
| Normalized Enrichment Score (NES) | 1.6844815 |
| Nominal p-value | 0.0 |
| FDR q-value | 0.014542011 |
| FWER p-Value | 0.515 |
Table: GSEA Results Summary

  

Fig 1: Enrichment plot: HDACI\_COLON\_SUL\_DN      
 Profile of the Running ES Score & Positions of GeneSet Members on the Rank Ordered List

  

| PROBE | GENE SYMBOL | GENE\_TITLE | RANK IN GENE LIST | RANK METRIC SCORE | RUNNING ES | CORE ENRICHMENT || 1 | GPIAP1 |  |  | 28 | 0.746 | 0.0196 | Yes |
| 2 | CCL28 |  |  | 98 | 0.584 | 0.0326 | Yes |
| 3 | NAP1L1 |  |  | 142 | 0.531 | 0.0454 | Yes |
| 4 | TIA1 |  |  | 167 | 0.513 | 0.0586 | Yes |
| 5 | CALD1 |  |  | 177 | 0.506 | 0.0724 | Yes |
| 6 | HMGB1 |  |  | 247 | 0.469 | 0.0822 | Yes |
| 7 | PRKCBP1 |  |  | 294 | 0.454 | 0.0927 | Yes |
| 8 | PPP1CB |  |  | 345 | 0.441 | 0.1026 | Yes |
| 9 | NUB1 |  |  | 422 | 0.420 | 0.1106 | Yes |
| 10 | RBBP4 |  |  | 474 | 0.410 | 0.1197 | Yes |
| 11 | HMGB2 |  |  | 601 | 0.385 | 0.1243 | Yes |
| 12 | RBBP6 |  |  | 635 | 0.378 | 0.1333 | Yes |
| 13 | FAIM |  |  | 644 | 0.377 | 0.1434 | Yes |
| 14 | SFRS1 |  |  | 923 | 0.339 | 0.1393 | Yes |
| 15 | CGNL1 |  |  | 924 | 0.339 | 0.1488 | Yes |
| 16 | ORMDL2 |  |  | 951 | 0.336 | 0.1570 | Yes |
| 17 | TMF1 |  |  | 953 | 0.336 | 0.1664 | Yes |
| 18 | LASP1 |  |  | 1010 | 0.330 | 0.1729 | Yes |
| 19 | RAB13 |  |  | 1020 | 0.329 | 0.1817 | Yes |
| 20 | DHX9 |  |  | 1028 | 0.328 | 0.1905 | Yes |
| 21 | SUV420H1 |  |  | 1146 | 0.316 | 0.1937 | Yes |
| 22 | ABP1 |  |  | 1149 | 0.316 | 0.2024 | Yes |
| 23 | BAT1 |  |  | 1235 | 0.308 | 0.2069 | Yes |
| 24 | SFPQ |  |  | 1260 | 0.305 | 0.2143 | Yes |
| 25 | DARS |  |  | 1276 | 0.304 | 0.2221 | Yes |
| 26 | UNC5A |  |  | 1294 | 0.303 | 0.2298 | Yes |
| 27 | BCLAF1 |  |  | 1313 | 0.301 | 0.2373 | Yes |
| 28 | CHCHD7 |  |  | 1409 | 0.293 | 0.2409 | Yes |
| 29 | TGOLN2 |  |  | 1472 | 0.288 | 0.2460 | Yes |
| 30 | NBN |  |  | 1540 | 0.284 | 0.2506 | Yes |
| 31 | TSNAX |  |  | 1563 | 0.282 | 0.2575 | Yes |
| 32 | TXNDC1 |  |  | 1578 | 0.280 | 0.2647 | Yes |
| 33 | CREBBP |  |  | 1584 | 0.280 | 0.2723 | Yes |
| 34 | LOC388969 |  |  | 1592 | 0.279 | 0.2798 | Yes |
| 35 | ERGIC3 |  |  | 1796 | 0.267 | 0.2773 | Yes |
| 36 | ACP1 |  |  | 1846 | 0.264 | 0.2823 | Yes |
| 37 | ZMYM3 |  |  | 1874 | 0.263 | 0.2884 | Yes |
| 38 | CDC7 |  |  | 1919 | 0.260 | 0.2935 | Yes |
| 39 | EPB41 |  |  | 1943 | 0.258 | 0.2996 | Yes |
| 40 | LARP4 |  |  | 1965 | 0.257 | 0.3058 | Yes |
| 41 | GOLGB1 |  |  | 1993 | 0.255 | 0.3116 | Yes |
| 42 | C10ORF32 |  |  | 2064 | 0.252 | 0.3153 | Yes |
| 43 | GTF3A |  |  | 2073 | 0.252 | 0.3220 | Yes |
| 44 | UPF3A |  |  | 2087 | 0.251 | 0.3284 | Yes |
| 45 | AZI2 |  |  | 2137 | 0.249 | 0.3329 | Yes |
| 46 | RAD51L1 |  |  | 2167 | 0.247 | 0.3385 | Yes |
| 47 | CRYL1 |  |  | 2178 | 0.246 | 0.3449 | Yes |
| 48 | MTBP |  |  | 2244 | 0.243 | 0.3485 | Yes |
| 49 | SEPT3 |  |  | 2245 | 0.243 | 0.3553 | Yes |
| 50 | MAP9 |  |  | 2302 | 0.240 | 0.3593 | Yes |
| 51 | CUL4B |  |  | 2339 | 0.239 | 0.3643 | Yes |
| 52 | STK11IP |  |  | 2536 | 0.228 | 0.3611 | Yes |
| 53 | TBC1D23 |  |  | 2561 | 0.227 | 0.3663 | Yes |
| 54 | THRAP3 |  |  | 2571 | 0.227 | 0.3722 | Yes |
| 55 | SNX12 |  |  | 2634 | 0.225 | 0.3755 | Yes |
| 56 | PPP1R1B |  |  | 2704 | 0.223 | 0.3784 | Yes |
| 57 | HP1BP3 |  |  | 2713 | 0.222 | 0.3842 | Yes |
| 58 | FAU |  |  | 2866 | 0.216 | 0.3828 | Yes |
| 59 | AKAP1 |  |  | 2939 | 0.213 | 0.3853 | Yes |
| 60 | BMP4 |  |  | 2962 | 0.212 | 0.3901 | Yes |
| 61 | SSR1 |  |  | 2980 | 0.211 | 0.3952 | Yes |
| 62 | FXYD6 |  |  | 2982 | 0.211 | 0.4011 | Yes |
| 63 | NUPL1 |  |  | 3005 | 0.210 | 0.4059 | Yes |
| 64 | IDE |  |  | 3023 | 0.210 | 0.4110 | Yes |
| 65 | RHBDF1 |  |  | 3049 | 0.209 | 0.4156 | Yes |
| 66 | ADD3 |  |  | 3113 | 0.206 | 0.4183 | Yes |
| 67 | DAP |  |  | 3192 | 0.203 | 0.4202 | Yes |
| 68 | PRKAR1A |  |  | 3195 | 0.203 | 0.4258 | Yes |
| 69 | VEGFB |  |  | 3232 | 0.202 | 0.4297 | Yes |
| 70 | TMPO |  |  | 3240 | 0.202 | 0.4350 | Yes |
| 71 | NDUFB6 |  |  | 3272 | 0.201 | 0.4391 | Yes |
| 72 | KHDRBS1 |  |  | 3282 | 0.200 | 0.4443 | Yes |
| 73 | DLG1 |  |  | 3367 | 0.198 | 0.4457 | Yes |
| 74 | SRA1 |  |  | 3421 | 0.196 | 0.4486 | Yes |
| 75 | RTN4RL1 |  |  | 3473 | 0.194 | 0.4516 | Yes |
| 76 | IFITM2 |  |  | 3605 | 0.191 | 0.4505 | Yes |
| 77 | ST18 |  |  | 3639 | 0.190 | 0.4542 | Yes |
| 78 | ZNF529 |  |  | 3719 | 0.188 | 0.4556 | Yes |
| 79 | TTC3 |  |  | 3721 | 0.188 | 0.4609 | Yes |
| 80 | SORT1 |  |  | 3791 | 0.186 | 0.4627 | Yes |
| 81 | CD46 |  |  | 3833 | 0.184 | 0.4658 | Yes |
| 82 | MAPK4 |  |  | 3860 | 0.183 | 0.4697 | Yes |
| 83 | PAPOLA |  |  | 3905 | 0.182 | 0.4727 | Yes |
| 84 | TMEM123 |  |  | 3989 | 0.179 | 0.4736 | Yes |
| 85 | KIF1B |  |  | 4043 | 0.178 | 0.4760 | Yes |
| 86 | SFRS11 |  |  | 4134 | 0.176 | 0.4765 | Yes |
| 87 | LIMS1 |  |  | 4163 | 0.175 | 0.4801 | Yes |
| 88 | ATP2A2 |  |  | 4174 | 0.175 | 0.4845 | Yes |
| 89 | STRA6 |  |  | 4210 | 0.174 | 0.4877 | Yes |
| 90 | BMP1 |  |  | 4255 | 0.173 | 0.4903 | Yes |
| 91 | TUBB2C |  |  | 4295 | 0.172 | 0.4932 | Yes |
| 92 | P4HB |  |  | 4307 | 0.172 | 0.4975 | Yes |
| 93 | APTX |  |  | 4342 | 0.171 | 0.5007 | Yes |
| 94 | HRBL |  |  | 4375 | 0.170 | 0.5038 | Yes |
| 95 | DNAJC19 |  |  | 4377 | 0.170 | 0.5086 | Yes |
| 96 | MSI2 |  |  | 4387 | 0.169 | 0.5129 | Yes |
| 97 | PTGER3 |  |  | 4580 | 0.165 | 0.5081 | Yes |
| 98 | CDK6 |  |  | 4584 | 0.165 | 0.5126 | Yes |
| 99 | ARHGAP5 |  |  | 4629 | 0.164 | 0.5150 | Yes |
| 100 | SENP7 |  |  | 4684 | 0.162 | 0.5169 | Yes |
| 101 | PPP2R3A |  |  | 4741 | 0.161 | 0.5187 | Yes |
| 102 | MME |  |  | 4779 | 0.160 | 0.5213 | Yes |
| 103 | ORMDL1 |  |  | 4792 | 0.160 | 0.5252 | Yes |
| 104 | FAM3C |  |  | 4877 | 0.158 | 0.5255 | Yes |
| 105 | MITF |  |  | 4883 | 0.158 | 0.5297 | Yes |
| 106 | A1BG |  |  | 4901 | 0.158 | 0.5333 | Yes |
| 107 | ITGA6 |  |  | 4923 | 0.157 | 0.5367 | Yes |
| 108 | ARPP-19 |  |  | 4936 | 0.157 | 0.5405 | Yes |
| 109 | LRDD |  |  | 4968 | 0.156 | 0.5434 | Yes |
| 110 | RNF180 |  |  | 5061 | 0.154 | 0.5432 | No |
| 111 | EIF4G2 |  |  | 5482 | 0.145 | 0.5266 | No |
| 112 | PDZD2 |  |  | 5612 | 0.142 | 0.5243 | No |
| 113 | XPO7 |  |  | 5645 | 0.141 | 0.5267 | No |
| 114 | GNL1 |  |  | 5685 | 0.141 | 0.5288 | No |
| 115 | ADIPOQ |  |  | 5793 | 0.138 | 0.5274 | No |
| 116 | WDR89 |  |  | 5828 | 0.138 | 0.5296 | No |
| 117 | SULT1C1 |  |  | 5971 | 0.136 | 0.5264 | No |
| 118 | SCML1 |  |  | 5992 | 0.135 | 0.5293 | No |
| 119 | SNTB1 |  |  | 6102 | 0.134 | 0.5277 | No |
| 120 | C13ORF1 |  |  | 6110 | 0.133 | 0.5311 | No |
| 121 | ANK1 |  |  | 6189 | 0.132 | 0.5309 | No |
| 122 | PSMA1 |  |  | 6259 | 0.131 | 0.5312 | No |
| 123 | MGA |  |  | 6318 | 0.130 | 0.5320 | No |
| 124 | FLJ20628 |  |  | 6484 | 0.127 | 0.5275 | No |
| 125 | OFD1 |  |  | 6525 | 0.126 | 0.5291 | No |
| 126 | APOC2 |  |  | 6678 | 0.124 | 0.5251 | No |
| 127 | RDX |  |  | 6786 | 0.122 | 0.5233 | No |
| 128 | SEMA3A |  |  | 6852 | 0.121 | 0.5235 | No |
| 129 | MEGF9 |  |  | 7098 | 0.116 | 0.5147 | No |
| 130 | LRIG2 |  |  | 7199 | 0.114 | 0.5130 | No |
| 131 | GPLD1 |  |  | 7626 | 0.108 | 0.4951 | No |
| 132 | CHI3L2 |  |  | 8014 | 0.101 | 0.4790 | No |
| 133 | SCLT1 |  |  | 8162 | 0.099 | 0.4746 | No |
| 134 | PRPF38B |  |  | 8201 | 0.099 | 0.4755 | No |
| 135 | EHHADH |  |  | 8245 | 0.098 | 0.4761 | No |
| 136 | QRSL1 |  |  | 8303 | 0.097 | 0.4761 | No |
| 137 | CACNA1D |  |  | 8422 | 0.095 | 0.4729 | No |
| 138 | NT5DC2 |  |  | 8463 | 0.095 | 0.4736 | No |
| 139 | MYB |  |  | 8473 | 0.094 | 0.4758 | No |
| 140 | SKIV2L2 |  |  | 8582 | 0.093 | 0.4732 | No |
| 141 | COBRA1 |  |  | 9000 | 0.087 | 0.4551 | No |
| 142 | NACA |  |  | 9079 | 0.086 | 0.4537 | No |
| 143 | FLJ10154 |  |  | 9228 | 0.084 | 0.4488 | No |
| 144 | BAIAP2 |  |  | 9276 | 0.083 | 0.4488 | No |
| 145 | C9ORF85 |  |  | 9292 | 0.083 | 0.4504 | No |
| 146 | PPP2R2A |  |  | 9332 | 0.082 | 0.4508 | No |
| 147 | PTPRM |  |  | 9369 | 0.082 | 0.4514 | No |
| 148 | AGPAT1 |  |  | 9636 | 0.078 | 0.4405 | No |
| 149 | GAPDH |  |  | 9691 | 0.077 | 0.4400 | No |
| 150 | PCF11 |  |  | 9848 | 0.076 | 0.4345 | No |
| 151 | PHLDA1 |  |  | 9954 | 0.074 | 0.4314 | No |
| 152 | SGCD |  |  | 10004 | 0.073 | 0.4310 | No |
| 153 | SLC7A8 |  |  | 10253 | 0.070 | 0.4208 | No |
| 154 | C10ORF46 |  |  | 10710 | 0.063 | 0.4003 | No |
| 155 | COL9A1 |  |  | 10841 | 0.062 | 0.3956 | No |
| 156 | SERGEF |  |  | 10859 | 0.062 | 0.3965 | No |
| 157 | BTK |  |  | 10976 | 0.060 | 0.3925 | No |
| 158 | MBNL2 |  |  | 11140 | 0.058 | 0.3861 | No |
| 159 | MRPL23 |  |  | 11384 | 0.055 | 0.3757 | No |
| 160 | SESTD1 |  |  | 11388 | 0.055 | 0.3771 | No |
| 161 | SAMD4A |  |  | 11435 | 0.054 | 0.3764 | No |
| 162 | CHD1L |  |  | 11555 | 0.053 | 0.3721 | No |
| 163 | PTPRN |  |  | 11779 | 0.050 | 0.3625 | No |
| 164 | CAMSAP1L1 |  |  | 11790 | 0.050 | 0.3634 | No |
| 165 | TMEM63C |  |  | 12031 | 0.046 | 0.3529 | No |
| 166 | CTNNA1 |  |  | 12390 | 0.042 | 0.3366 | No |
| 167 | C2 |  |  | 12568 | 0.039 | 0.3290 | No |
| 168 | PRKCA |  |  | 12613 | 0.039 | 0.3279 | No |
| 169 | SLIT3 |  |  | 12668 | 0.038 | 0.3263 | No |
| 170 | FOLR2 |  |  | 12807 | 0.036 | 0.3206 | No |
| 171 | ALDH1L2 |  |  | 12967 | 0.035 | 0.3138 | No |
| 172 | TPM3 |  |  | 13045 | 0.033 | 0.3109 | No |
| 173 | AMMECR1 |  |  | 13136 | 0.032 | 0.3074 | No |
| 174 | CDH11 |  |  | 13551 | 0.027 | 0.2879 | No |
| 175 | STAT5A |  |  | 13555 | 0.027 | 0.2885 | No |
| 176 | PLCB2 |  |  | 13671 | 0.025 | 0.2835 | No |
| 177 | C20ORF94 |  |  | 13703 | 0.025 | 0.2827 | No |
| 178 | GALNT5 |  |  | 13717 | 0.025 | 0.2827 | No |
| 179 | ISOC2 |  |  | 13760 | 0.024 | 0.2814 | No |
| 180 | PPIB |  |  | 13763 | 0.024 | 0.2819 | No |
| 181 | LIPC |  |  | 14029 | 0.020 | 0.2695 | No |
| 182 | CCDC14 |  |  | 14038 | 0.020 | 0.2697 | No |
| 183 | PDSS2 |  |  | 14134 | 0.019 | 0.2655 | No |
| 184 | AASS |  |  | 14250 | 0.017 | 0.2604 | No |
| 185 | AQP1 |  |  | 14264 | 0.017 | 0.2602 | No |
| 186 | KIAA0802 |  |  | 14599 | 0.012 | 0.2441 | No |
| 187 | VIL1 |  |  | 14613 | 0.012 | 0.2438 | No |
| 188 | IL16 |  |  | 14614 | 0.012 | 0.2442 | No |
| 189 | SLC9A9 |  |  | 14630 | 0.012 | 0.2437 | No |
| 190 | ITM2A |  |  | 14817 | 0.009 | 0.2349 | No |
| 191 | DPYSL3 |  |  | 14893 | 0.008 | 0.2314 | No |
| 192 | PCCA |  |  | 15013 | 0.006 | 0.2257 | No |
| 193 | FER1L3 |  |  | 15587 | -0.003 | 0.1977 | No |
| 194 | TJP2 |  |  | 15609 | -0.004 | 0.1968 | No |
| 195 | ABCC6 |  |  | 15619 | -0.004 | 0.1965 | No |
| 196 | NEUROD6 |  |  | 15741 | -0.006 | 0.1907 | No |
| 197 | CACNA2D4 |  |  | 16199 | -0.013 | 0.1687 | No |
| 198 | LTBP2 |  |  | 16524 | -0.019 | 0.1533 | No |
| 199 | EEF1G |  |  | 16706 | -0.023 | 0.1451 | No |
| 200 | SDHB |  |  | 16716 | -0.024 | 0.1453 | No |
| 201 | C14ORF37 |  |  | 17023 | -0.029 | 0.1311 | No |
| 202 | LOC284998 |  |  | 17211 | -0.033 | 0.1229 | No |
| 203 | BIRC6 |  |  | 17264 | -0.034 | 0.1213 | No |
| 204 | PVALB |  |  | 17537 | -0.041 | 0.1091 | No |
| 205 | LRP8 |  |  | 17774 | -0.046 | 0.0988 | No |
| 206 | ATP1A3 |  |  | 17812 | -0.047 | 0.0983 | No |
| 207 | LOC200420 |  |  | 18064 | -0.053 | 0.0875 | No |
| 208 | ARMCX1 |  |  | 19110 | -0.089 | 0.0388 | No |
| 209 | C8B |  |  | 19743 | -0.123 | 0.0112 | No |
| 210 | PHLPPL |  |  | 19802 | -0.127 | 0.0119 | No |
| 211 | FOXM1 |  |  | 19824 | -0.129 | 0.0145 | No |
| 212 | KIAA0114 |  |  | 20099 | -0.156 | 0.0055 | No |
| 213 | ORC6L |  |  | 20326 | -0.201 | 0.0000 | No |
| 214 | FOS |  |  | 20334 | -0.202 | 0.0054 | No |
| 215 | KRT23 |  |  | 20480 | -0.280 | 0.0061 | No |
Table: GSEA details [plain text format]

  

Fig 2: HDACI\_COLON\_SUL\_DN: Random ES distribution      
 Gene set null distribution of ES for **HDACI\_COLON\_SUL\_DN**
