# Supplementary material for: The rapamycin-regulated gene expression signature determines prognosis for breast cancer
Source: Mol Cancer. 2009 Sep 24;8:75. doi: 10.1186/1476-4598-8-75 (PMC2761377; doi:10.1186/1476-4598-8-75)
Supplement: Additional file 3 — Gene set enrichment analysis of in vivo data, treatment series. The data provided represent the treatment series of GSEA. This compressed file contains "Treatment" shortcut file and "GSEA_treatment" folder. Clicking on "Treatment" shortcut opens the index file providing access to analysis files contained in the "GSEA_treatment" folder. [file 1476-4598-8-75-S3.zip › GSEA_treatment/HINATA_NFKB_DN.html]

Details for gene set HINATA\_NFKB\_DN[GSEA]

|  || Dataset | gsea\_treatment\_collapsed |
| Phenotype | NoPhenotypeAvailable |
| Upregulated in class | na\_neg |
| GeneSet | HINATA\_NFKB\_DN |
| Enrichment Score (ES) | -0.6008895 |
| Normalized Enrichment Score (NES) | -2.166713 |
| Nominal p-value | 0.0 |
| FDR q-value | 0.0038487478 |
| FWER p-Value | 0.016 |
Table: GSEA Results Summary

  

Fig 1: Enrichment plot: HINATA\_NFKB\_DN      
 Profile of the Running ES Score & Positions of GeneSet Members on the Rank Ordered List

  

| PROBE | GENE SYMBOL | GENE\_TITLE | RANK IN GENE LIST | RANK METRIC SCORE | RUNNING ES | CORE ENRICHMENT || 1 | ZNF185 |  |  | 2161 | 0.247 | -0.0315 | No |
| 2 | THBS1 |  |  | 2263 | 0.242 | 0.0355 | No |
| 3 | DUSP4 |  |  | 4820 | 0.159 | -0.0414 | No |
| 4 | RUNX1 |  |  | 9041 | 0.086 | -0.2208 | No |
| 5 | KIAA0350 |  |  | 9562 | 0.079 | -0.2226 | No |
| 6 | SCEL |  |  | 11868 | 0.049 | -0.3202 | No |
| 7 | RIMS2 |  |  | 12763 | 0.037 | -0.3526 | No |
| 8 | C4BPA |  |  | 13674 | 0.025 | -0.3894 | No |
| 9 | SULT2B1 |  |  | 13736 | 0.024 | -0.3852 | No |
| 10 | DAPK1 |  |  | 14673 | 0.011 | -0.4273 | No |
| 11 | CEACAM6 |  |  | 17308 | -0.036 | -0.5447 | No |
| 12 | HYAL1 |  |  | 17320 | -0.036 | -0.5347 | No |
| 13 | INA |  |  | 17408 | -0.038 | -0.5278 | No |
| 14 | UPK1B |  |  | 17533 | -0.041 | -0.5216 | No |
| 15 | PTHLH |  |  | 17572 | -0.041 | -0.5112 | No |
| 16 | KLK7 |  |  | 18619 | -0.069 | -0.5414 | No |
| 17 | PPL |  |  | 19672 | -0.118 | -0.5575 | Yes |
| 18 | DSG3 |  |  | 20566 | -0.464 | -0.4630 | Yes |
| 19 | RGS2 |  |  | 20592 | -0.771 | -0.2349 | Yes |
| 20 | KLK6 |  |  | 20593 | -0.793 | 0.0006 | Yes |
Table: GSEA details [plain text format]

  

Fig 2: HINATA\_NFKB\_DN: Random ES distribution      
 Gene set null distribution of ES for **HINATA\_NFKB\_DN**
