# Supplementary material for: The rapamycin-regulated gene expression signature determines prognosis for breast cancer
Source: Mol Cancer. 2009 Sep 24;8:75. doi: 10.1186/1476-4598-8-75 (PMC2761377; doi:10.1186/1476-4598-8-75)
Supplement: Additional file 3 — Gene set enrichment analysis of in vivo data, treatment series. The data provided represent the treatment series of GSEA. This compressed file contains "Treatment" shortcut file and "GSEA_treatment" folder. Clicking on "Treatment" shortcut opens the index file providing access to analysis files contained in the "GSEA_treatment" folder. [file 1476-4598-8-75-S3.zip › GSEA_treatment/HIPPOCAMPUS_DEVELOPMENT_PRENATAL.html]

Details for gene set HIPPOCAMPUS\_DEVELOPMENT\_PRENATAL[GSEA]

|  || Dataset | gsea\_treatment\_collapsed |
| Phenotype | NoPhenotypeAvailable |
| Upregulated in class | na\_pos |
| GeneSet | HIPPOCAMPUS\_DEVELOPMENT\_PRENATAL |
| Enrichment Score (ES) | 0.63653487 |
| Normalized Enrichment Score (NES) | 1.7141614 |
| Nominal p-value | 0.0 |
| FDR q-value | 0.010173239 |
| FWER p-Value | 0.339 |
Table: GSEA Results Summary

  

Fig 1: Enrichment plot: HIPPOCAMPUS\_DEVELOPMENT\_PRENATAL      
 Profile of the Running ES Score & Positions of GeneSet Members on the Rank Ordered List

  

| PROBE | GENE SYMBOL | GENE\_TITLE | RANK IN GENE LIST | RANK METRIC SCORE | RUNNING ES | CORE ENRICHMENT || 1 | H2AFV |  |  | 127 | 0.543 | 0.0702 | Yes |
| 2 | EEF1A1 |  |  | 318 | 0.446 | 0.1236 | Yes |
| 3 | CCNG2 |  |  | 485 | 0.408 | 0.1728 | Yes |
| 4 | RPL13A |  |  | 598 | 0.385 | 0.2215 | Yes |
| 5 | LSM4 |  |  | 600 | 0.385 | 0.2755 | Yes |
| 6 | CDK4 |  |  | 682 | 0.369 | 0.3234 | Yes |
| 7 | NFYB |  |  | 685 | 0.369 | 0.3751 | Yes |
| 8 | H2AFX |  |  | 814 | 0.352 | 0.4184 | Yes |
| 9 | RPL37 |  |  | 983 | 0.333 | 0.4570 | Yes |
| 10 | RPL13 |  |  | 1062 | 0.325 | 0.4989 | Yes |
| 11 | SFPQ |  |  | 1260 | 0.305 | 0.5322 | Yes |
| 12 | HNRPA0 |  |  | 1311 | 0.302 | 0.5722 | Yes |
| 13 | WBP1 |  |  | 1407 | 0.293 | 0.6088 | Yes |
| 14 | SFRS6 |  |  | 1636 | 0.277 | 0.6365 | Yes |
| 15 | DDX48 |  |  | 3263 | 0.201 | 0.5857 | No |
| 16 | PCNA |  |  | 3626 | 0.190 | 0.5949 | No |
| 17 | CCNB2 |  |  | 3748 | 0.187 | 0.6152 | No |
| 18 | SNRPB2 |  |  | 4240 | 0.173 | 0.6157 | No |
| 19 | UBE2C |  |  | 4419 | 0.168 | 0.6307 | No |
| 20 | RPS8 |  |  | 5983 | 0.135 | 0.5737 | No |
| 21 | MAP7 |  |  | 6114 | 0.133 | 0.5861 | No |
| 22 | RPS18 |  |  | 6126 | 0.133 | 0.6043 | No |
| 23 | RPL9 |  |  | 9040 | 0.086 | 0.4748 | No |
| 24 | POLR1C |  |  | 9979 | 0.074 | 0.4395 | No |
| 25 | RPL8 |  |  | 11376 | 0.055 | 0.3794 | No |
| 26 | MYT1 |  |  | 12177 | 0.044 | 0.3468 | No |
| 27 | RBPSUH |  |  | 12844 | 0.036 | 0.3194 | No |
| 28 | H1F0 |  |  | 16155 | -0.013 | 0.1603 | No |
| 29 | NEUROG2 |  |  | 16636 | -0.022 | 0.1400 | No |
| 30 | EEF1G |  |  | 16706 | -0.023 | 0.1399 | No |
| 31 | CCND2 |  |  | 17509 | -0.040 | 0.1065 | No |
| 32 | GADD45G |  |  | 19781 | -0.126 | 0.0137 | No |
| 33 | CKS2 |  |  | 20271 | -0.187 | 0.0162 | No |
Table: GSEA details [plain text format]

  

Fig 2: HIPPOCAMPUS\_DEVELOPMENT\_PRENATAL: Random ES distribution      
 Gene set null distribution of ES for **HIPPOCAMPUS\_DEVELOPMENT\_PRENATAL**
