# Supplementary material for: The rapamycin-regulated gene expression signature determines prognosis for breast cancer
Source: Mol Cancer. 2009 Sep 24;8:75. doi: 10.1186/1476-4598-8-75 (PMC2761377; doi:10.1186/1476-4598-8-75)
Supplement: Additional file 3 — Gene set enrichment analysis of in vivo data, treatment series. The data provided represent the treatment series of GSEA. This compressed file contains "Treatment" shortcut file and "GSEA_treatment" folder. Clicking on "Treatment" shortcut opens the index file providing access to analysis files contained in the "GSEA_treatment" folder. [file 1476-4598-8-75-S3.zip › GSEA_treatment/HSP27PATHWAY.html]

Details for gene set HSP27PATHWAY[GSEA]

|  || Dataset | gsea\_treatment\_collapsed |
| Phenotype | NoPhenotypeAvailable |
| Upregulated in class | na\_neg |
| GeneSet | HSP27PATHWAY |
| Enrichment Score (ES) | -0.33850884 |
| Normalized Enrichment Score (NES) | -1.1115144 |
| Nominal p-value | 0.32786885 |
| FDR q-value | 0.43584436 |
| FWER p-Value | 1.0 |
Table: GSEA Results Summary

  

Fig 1: Enrichment plot: HSP27PATHWAY      
 Profile of the Running ES Score & Positions of GeneSet Members on the Rank Ordered List

  

| PROBE | GENE SYMBOL | GENE\_TITLE | RANK IN GENE LIST | RANK METRIC SCORE | RUNNING ES | CORE ENRICHMENT || 1 | TNF |  |  | 1758 | 0.269 | 0.0160 | Yes |
| 2 | MAPKAPK2 |  |  | 2298 | 0.240 | 0.0805 | Yes |
| 3 | CYCS |  |  | 2626 | 0.226 | 0.1496 | Yes |
| 4 | DAXX |  |  | 3145 | 0.205 | 0.2019 | Yes |
| 5 | FAS |  |  | 3778 | 0.186 | 0.2412 | Yes |
| 6 | MAPKAPK3 |  |  | 5688 | 0.141 | 0.2016 | No |
| 7 | CASP3 |  |  | 7277 | 0.113 | 0.1673 | No |
| 8 | BCL2 |  |  | 8111 | 0.100 | 0.1645 | No |
| 9 | HSPB2 |  |  | 8872 | 0.088 | 0.1610 | No |
| 10 | CASP9 |  |  | 10305 | 0.069 | 0.1174 | No |
| 11 | APAF1 |  |  | 11413 | 0.055 | 0.0843 | No |
| 12 | FASLG |  |  | 16465 | -0.018 | -0.1541 | No |
| 13 | IL1A |  |  | 17407 | -0.038 | -0.1857 | No |
| 14 | ACTA1 |  |  | 20555 | -0.427 | -0.1775 | No |
| 15 | HSPB1 |  |  | 20567 | -0.477 | 0.0018 | No |
Table: GSEA details [plain text format]

  

Fig 2: HSP27PATHWAY: Random ES distribution      
 Gene set null distribution of ES for **HSP27PATHWAY**
