# Supplementary material for: The rapamycin-regulated gene expression signature determines prognosis for breast cancer
Source: Mol Cancer. 2009 Sep 24;8:75. doi: 10.1186/1476-4598-8-75 (PMC2761377; doi:10.1186/1476-4598-8-75)
Supplement: Additional file 3 — Gene set enrichment analysis of in vivo data, treatment series. The data provided represent the treatment series of GSEA. This compressed file contains "Treatment" shortcut file and "GSEA_treatment" folder. Clicking on "Treatment" shortcut opens the index file providing access to analysis files contained in the "GSEA_treatment" folder. [file 1476-4598-8-75-S3.zip › GSEA_treatment/HYPOXIA_REG_UP.html]

Details for gene set HYPOXIA\_REG\_UP[GSEA]

|  || Dataset | gsea\_treatment\_collapsed |
| Phenotype | NoPhenotypeAvailable |
| Upregulated in class | na\_neg |
| GeneSet | HYPOXIA\_REG\_UP |
| Enrichment Score (ES) | -0.33958682 |
| Normalized Enrichment Score (NES) | -1.4183044 |
| Nominal p-value | 0.029411765 |
| FDR q-value | 0.21480945 |
| FWER p-Value | 0.956 |
Table: GSEA Results Summary

  

Fig 1: Enrichment plot: HYPOXIA\_REG\_UP      
 Profile of the Running ES Score & Positions of GeneSet Members on the Rank Ordered List

  

| PROBE | GENE SYMBOL | GENE\_TITLE | RANK IN GENE LIST | RANK METRIC SCORE | RUNNING ES | CORE ENRICHMENT || 1 | EFNA1 |  |  | 109 | 0.566 | 0.0631 | No |
| 2 | CCNG2 |  |  | 485 | 0.408 | 0.0941 | No |
| 3 | PHC2 |  |  | 537 | 0.397 | 0.1395 | No |
| 4 | PDGFB |  |  | 1249 | 0.307 | 0.1420 | No |
| 5 | FUS |  |  | 1639 | 0.276 | 0.1564 | No |
| 6 | F3 |  |  | 1690 | 0.273 | 0.1869 | No |
| 7 | CDKN1B |  |  | 1757 | 0.269 | 0.2162 | No |
| 8 | FOSL2 |  |  | 1767 | 0.268 | 0.2481 | No |
| 9 | HNRPA1 |  |  | 2259 | 0.242 | 0.2535 | No |
| 10 | SERPINE1 |  |  | 3043 | 0.209 | 0.2406 | No |
| 11 | JUNB |  |  | 3199 | 0.203 | 0.2576 | No |
| 12 | PPP1R3C |  |  | 3228 | 0.202 | 0.2806 | No |
| 13 | JMJD1A |  |  | 3353 | 0.199 | 0.2986 | No |
| 14 | BNIP3 |  |  | 3398 | 0.197 | 0.3202 | No |
| 15 | SLC16A3 |  |  | 4120 | 0.176 | 0.3064 | No |
| 16 | SAP30 |  |  | 4318 | 0.171 | 0.3175 | No |
| 17 | C10ORF10 |  |  | 4764 | 0.160 | 0.3152 | No |
| 18 | PGK1 |  |  | 6774 | 0.122 | 0.2323 | No |
| 19 | ALDOA |  |  | 9957 | 0.074 | 0.0864 | No |
| 20 | P4HA2 |  |  | 10485 | 0.066 | 0.0688 | No |
| 21 | MUC1 |  |  | 12069 | 0.046 | -0.0026 | No |
| 22 | RASGRP1 |  |  | 12821 | 0.036 | -0.0348 | No |
| 23 | SLC2A1 |  |  | 13804 | 0.023 | -0.0797 | No |
| 24 | PTDSR |  |  | 14440 | 0.014 | -0.1089 | No |
| 25 | PFKFB4 |  |  | 15027 | 0.006 | -0.1367 | No |
| 26 | TGFA |  |  | 16591 | -0.021 | -0.2102 | No |
| 27 | ADORA2B |  |  | 18557 | -0.067 | -0.2976 | No |
| 28 | DUSP1 |  |  | 18808 | -0.076 | -0.3005 | No |
| 29 | ADFP |  |  | 19613 | -0.114 | -0.3258 | Yes |
| 30 | PFKP |  |  | 19647 | -0.116 | -0.3134 | Yes |
| 31 | NFIL3 |  |  | 19908 | -0.137 | -0.3095 | Yes |
| 32 | GPRC5A |  |  | 20118 | -0.159 | -0.3005 | Yes |
| 33 | VEGF |  |  | 20367 | -0.211 | -0.2871 | Yes |
| 34 | BHLHB2 |  |  | 20403 | -0.226 | -0.2615 | Yes |
| 35 | SLC7A5 |  |  | 20535 | -0.371 | -0.2231 | Yes |
| 36 | STC2 |  |  | 20562 | -0.453 | -0.1697 | Yes |
| 37 | ADM |  |  | 20571 | -0.495 | -0.1103 | Yes |
| 38 | DDIT4 |  |  | 20600 | -0.927 | 0.0002 | Yes |
Table: GSEA details [plain text format]

  

Fig 2: HYPOXIA\_REG\_UP: Random ES distribution      
 Gene set null distribution of ES for **HYPOXIA\_REG\_UP**
