# Supplementary material for: The rapamycin-regulated gene expression signature determines prognosis for breast cancer
Source: Mol Cancer. 2009 Sep 24;8:75. doi: 10.1186/1476-4598-8-75 (PMC2761377; doi:10.1186/1476-4598-8-75)
Supplement: Additional file 3 — Gene set enrichment analysis of in vivo data, treatment series. The data provided represent the treatment series of GSEA. This compressed file contains "Treatment" shortcut file and "GSEA_treatment" folder. Clicking on "Treatment" shortcut opens the index file providing access to analysis files contained in the "GSEA_treatment" folder. [file 1476-4598-8-75-S3.zip › GSEA_treatment/IDX_TSA_DN_CLUSTER4.html]

Details for gene set IDX\_TSA\_DN\_CLUSTER4[GSEA]

|  || Dataset | gsea\_treatment\_collapsed |
| Phenotype | NoPhenotypeAvailable |
| Upregulated in class | na\_neg |
| GeneSet | IDX\_TSA\_DN\_CLUSTER4 |
| Enrichment Score (ES) | -0.35012835 |
| Normalized Enrichment Score (NES) | -1.3236711 |
| Nominal p-value | 0.08928572 |
| FDR q-value | 0.2608205 |
| FWER p-Value | 0.996 |
Table: GSEA Results Summary

  

Fig 1: Enrichment plot: IDX\_TSA\_DN\_CLUSTER4      
 Profile of the Running ES Score & Positions of GeneSet Members on the Rank Ordered List

  

| PROBE | GENE SYMBOL | GENE\_TITLE | RANK IN GENE LIST | RANK METRIC SCORE | RUNNING ES | CORE ENRICHMENT || 1 | CCNG2 |  |  | 485 | 0.408 | 0.0724 | No |
| 2 | SEPT10 |  |  | 728 | 0.364 | 0.1463 | No |
| 3 | FLOT1 |  |  | 1947 | 0.258 | 0.1479 | No |
| 4 | UBE1C |  |  | 2152 | 0.248 | 0.1963 | No |
| 5 | ZFAND6 |  |  | 2667 | 0.224 | 0.2241 | No |
| 6 | PYGB |  |  | 3167 | 0.204 | 0.2480 | No |
| 7 | ATP6V1C1 |  |  | 4710 | 0.162 | 0.2111 | No |
| 8 | PKP2 |  |  | 7070 | 0.117 | 0.1240 | No |
| 9 | GYG1 |  |  | 9515 | 0.080 | 0.0240 | No |
| 10 | TMEM37 |  |  | 9609 | 0.078 | 0.0379 | No |
| 11 | STRBP |  |  | 9701 | 0.077 | 0.0516 | No |
| 12 | DOCK9 |  |  | 10553 | 0.065 | 0.0257 | No |
| 13 | LRRFIP1 |  |  | 12522 | 0.040 | -0.0606 | No |
| 14 | VCL |  |  | 12648 | 0.038 | -0.0576 | No |
| 15 | SDPR |  |  | 14058 | 0.020 | -0.1214 | No |
| 16 | MTM1 |  |  | 14706 | 0.011 | -0.1504 | No |
| 17 | CPEB2 |  |  | 15765 | -0.006 | -0.2003 | No |
| 18 | FDFT1 |  |  | 17340 | -0.036 | -0.2683 | No |
| 19 | ACADL |  |  | 17607 | -0.042 | -0.2713 | No |
| 20 | FDPS |  |  | 18142 | -0.055 | -0.2842 | No |
| 21 | ANXA6 |  |  | 18158 | -0.056 | -0.2718 | No |
| 22 | RAB3IL1 |  |  | 19121 | -0.090 | -0.2974 | No |
| 23 | SC4MOL |  |  | 20208 | -0.175 | -0.3090 | Yes |
| 24 | SQLE |  |  | 20301 | -0.196 | -0.2674 | Yes |
| 25 | NSDHL |  |  | 20464 | -0.263 | -0.2135 | Yes |
| 26 | ANKRD57 |  |  | 20523 | -0.330 | -0.1385 | Yes |
| 27 | HMGCS1 |  |  | 20582 | -0.605 | 0.0011 | Yes |
Table: GSEA details [plain text format]

  

Fig 2: IDX\_TSA\_DN\_CLUSTER4: Random ES distribution      
 Gene set null distribution of ES for **IDX\_TSA\_DN\_CLUSTER4**
