# Supplementary material for: The rapamycin-regulated gene expression signature determines prognosis for breast cancer
Source: Mol Cancer. 2009 Sep 24;8:75. doi: 10.1186/1476-4598-8-75 (PMC2761377; doi:10.1186/1476-4598-8-75)
Supplement: Additional file 3 — Gene set enrichment analysis of in vivo data, treatment series. The data provided represent the treatment series of GSEA. This compressed file contains "Treatment" shortcut file and "GSEA_treatment" folder. Clicking on "Treatment" shortcut opens the index file providing access to analysis files contained in the "GSEA_treatment" folder. [file 1476-4598-8-75-S3.zip › GSEA_treatment/IDX_TSA_DN_CLUSTER5.html]

Details for gene set IDX\_TSA\_DN\_CLUSTER5[GSEA]

|  || Dataset | gsea\_treatment\_collapsed |
| Phenotype | NoPhenotypeAvailable |
| Upregulated in class | na\_pos |
| GeneSet | IDX\_TSA\_DN\_CLUSTER5 |
| Enrichment Score (ES) | 0.6126426 |
| Normalized Enrichment Score (NES) | 1.7070421 |
| Nominal p-value | 0.0 |
| FDR q-value | 0.010843774 |
| FWER p-Value | 0.379 |
Table: GSEA Results Summary

  

Fig 1: Enrichment plot: IDX\_TSA\_DN\_CLUSTER5      
 Profile of the Running ES Score & Positions of GeneSet Members on the Rank Ordered List

  

| PROBE | GENE SYMBOL | GENE\_TITLE | RANK IN GENE LIST | RANK METRIC SCORE | RUNNING ES | CORE ENRICHMENT || 1 | MARCKS |  |  | 101 | 0.575 | 0.0676 | Yes |
| 2 | C11ORF46 |  |  | 143 | 0.530 | 0.1324 | Yes |
| 3 | ATP6AP2 |  |  | 258 | 0.466 | 0.1855 | Yes |
| 4 | SLC12A2 |  |  | 449 | 0.414 | 0.2284 | Yes |
| 5 | IRF1 |  |  | 551 | 0.393 | 0.2730 | Yes |
| 6 | ZMYM6 |  |  | 562 | 0.392 | 0.3219 | Yes |
| 7 | NFIX |  |  | 605 | 0.384 | 0.3683 | Yes |
| 8 | ATF1 |  |  | 917 | 0.340 | 0.3960 | Yes |
| 9 | ZNF644 |  |  | 1013 | 0.330 | 0.4330 | Yes |
| 10 | ATF2 |  |  | 1317 | 0.301 | 0.4562 | Yes |
| 11 | TOP2B |  |  | 1484 | 0.288 | 0.4844 | Yes |
| 12 | PB1 |  |  | 1525 | 0.285 | 0.5184 | Yes |
| 13 | SMARCA2 |  |  | 1977 | 0.256 | 0.5287 | Yes |
| 14 | PAWR |  |  | 2003 | 0.255 | 0.5596 | Yes |
| 15 | RAB9 |  |  | 2180 | 0.246 | 0.5821 | Yes |
| 16 | OGN |  |  | 3311 | 0.200 | 0.5523 | Yes |
| 17 | SNAPAP |  |  | 3434 | 0.196 | 0.5710 | Yes |
| 18 | RNF13 |  |  | 3553 | 0.192 | 0.5895 | Yes |
| 19 | ACVR2A |  |  | 3576 | 0.192 | 0.6126 | Yes |
| 20 | LIMS1 |  |  | 4163 | 0.175 | 0.6062 | No |
| 21 | NR3C1 |  |  | 7049 | 0.117 | 0.4807 | No |
| 22 | RASA1 |  |  | 8100 | 0.100 | 0.4422 | No |
| 23 | SMS |  |  | 8820 | 0.089 | 0.4185 | No |
| 24 | PKD2 |  |  | 9830 | 0.076 | 0.3789 | No |
| 25 | ARFGEF1 |  |  | 9924 | 0.074 | 0.3838 | No |
| 26 | SLC25A36 |  |  | 11642 | 0.051 | 0.3067 | No |
| 27 | SHOX2 |  |  | 11650 | 0.051 | 0.3129 | No |
| 28 | RBMS1 |  |  | 11967 | 0.047 | 0.3034 | No |
| 29 | TBK1 |  |  | 11995 | 0.047 | 0.3081 | No |
| 30 | C20ORF23 |  |  | 12011 | 0.047 | 0.3132 | No |
| 31 | CDH11 |  |  | 13551 | 0.027 | 0.2418 | No |
| 32 | KIAA1212 |  |  | 13587 | 0.026 | 0.2434 | No |
| 33 | EDG2 |  |  | 13730 | 0.025 | 0.2396 | No |
| 34 | GAS1 |  |  | 14002 | 0.021 | 0.2290 | No |
| 35 | AAK1 |  |  | 14396 | 0.015 | 0.2118 | No |
| 36 | LMO7 |  |  | 15466 | -0.001 | 0.1599 | No |
| 37 | KIF5B |  |  | 15633 | -0.004 | 0.1524 | No |
| 38 | RBM39 |  |  | 15989 | -0.010 | 0.1364 | No |
| 39 | FNBP4 |  |  | 16905 | -0.026 | 0.0953 | No |
| 40 | ALCAM |  |  | 16942 | -0.027 | 0.0970 | No |
| 41 | AMOT |  |  | 17337 | -0.036 | 0.0823 | No |
| 42 | FTS |  |  | 17945 | -0.050 | 0.0591 | No |
| 43 | COL3A1 |  |  | 19040 | -0.086 | 0.0167 | No |
| 44 | EPHB3 |  |  | 19077 | -0.088 | 0.0260 | No |
| 45 | SNAI2 |  |  | 19844 | -0.131 | 0.0052 | No |
| 46 | DDIT3 |  |  | 20445 | -0.252 | 0.0078 | No |
Table: GSEA details [plain text format]

  

Fig 2: IDX\_TSA\_DN\_CLUSTER5: Random ES distribution      
 Gene set null distribution of ES for **IDX\_TSA\_DN\_CLUSTER5**
