# Supplementary material for: The rapamycin-regulated gene expression signature determines prognosis for breast cancer
Source: Mol Cancer. 2009 Sep 24;8:75. doi: 10.1186/1476-4598-8-75 (PMC2761377; doi:10.1186/1476-4598-8-75)
Supplement: Additional file 3 — Gene set enrichment analysis of in vivo data, treatment series. The data provided represent the treatment series of GSEA. This compressed file contains "Treatment" shortcut file and "GSEA_treatment" folder. Clicking on "Treatment" shortcut opens the index file providing access to analysis files contained in the "GSEA_treatment" folder. [file 1476-4598-8-75-S3.zip › GSEA_treatment/IFNALPHA_HCC_UP.html]

Details for gene set IFNALPHA\_HCC\_UP[GSEA]

|  || Dataset | gsea\_treatment\_collapsed |
| Phenotype | NoPhenotypeAvailable |
| Upregulated in class | na\_pos |
| GeneSet | IFNALPHA\_HCC\_UP |
| Enrichment Score (ES) | 0.73801184 |
| Normalized Enrichment Score (NES) | 1.9670085 |
| Nominal p-value | 0.0 |
| FDR q-value | 3.3029262E-4 |
| FWER p-Value | 0.0030 |
Table: GSEA Results Summary

  

Fig 1: Enrichment plot: IFNALPHA\_HCC\_UP      
 Profile of the Running ES Score & Positions of GeneSet Members on the Rank Ordered List

  

| PROBE | GENE SYMBOL | GENE\_TITLE | RANK IN GENE LIST | RANK METRIC SCORE | RUNNING ES | CORE ENRICHMENT || 1 | MX1 |  |  | 24 | 0.773 | 0.0920 | Yes |
| 2 | IFI27 |  |  | 32 | 0.725 | 0.1790 | Yes |
| 3 | ISG15 |  |  | 59 | 0.644 | 0.2554 | Yes |
| 4 | LGALS3BP |  |  | 61 | 0.643 | 0.3329 | Yes |
| 5 | IFITM1 |  |  | 79 | 0.609 | 0.4055 | Yes |
| 6 | PSME1 |  |  | 513 | 0.402 | 0.4329 | Yes |
| 7 | IRF1 |  |  | 551 | 0.393 | 0.4785 | Yes |
| 8 | BST2 |  |  | 657 | 0.374 | 0.5185 | Yes |
| 9 | PLSCR1 |  |  | 717 | 0.365 | 0.5595 | Yes |
| 10 | AKR7A2 |  |  | 809 | 0.352 | 0.5976 | Yes |
| 11 | ADAR |  |  | 885 | 0.344 | 0.6354 | Yes |
| 12 | UBE2L6 |  |  | 980 | 0.333 | 0.6710 | Yes |
| 13 | TUBB |  |  | 1267 | 0.305 | 0.6939 | Yes |
| 14 | STAT1 |  |  | 1401 | 0.294 | 0.7229 | Yes |
| 15 | CDKN1B |  |  | 1757 | 0.269 | 0.7380 | Yes |
| 16 | MXD4 |  |  | 3253 | 0.201 | 0.6896 | No |
| 17 | IFITM2 |  |  | 3605 | 0.191 | 0.6956 | No |
| 18 | RFC2 |  |  | 3877 | 0.183 | 0.7044 | No |
| 19 | PHB |  |  | 6528 | 0.126 | 0.5908 | No |
| 20 | CASP3 |  |  | 7277 | 0.113 | 0.5682 | No |
| 21 | BCL2 |  |  | 8111 | 0.100 | 0.5397 | No |
| 22 | IFITM3 |  |  | 8420 | 0.095 | 0.5362 | No |
| 23 | SOCS1 |  |  | 8423 | 0.095 | 0.5476 | No |
| 24 | KLK3 |  |  | 8478 | 0.094 | 0.5564 | No |
| 25 | KIAA0409 |  |  | 9465 | 0.080 | 0.5181 | No |
| 26 | VCAM1 |  |  | 13537 | 0.027 | 0.3235 | No |
| 27 | IL12RB2 |  |  | 15366 | 0.000 | 0.2347 | No |
| 28 | E2F2 |  |  | 17657 | -0.043 | 0.1286 | No |
| 29 | MX2 |  |  | 19725 | -0.121 | 0.0428 | No |
Table: GSEA details [plain text format]

  

Fig 2: IFNALPHA\_HCC\_UP: Random ES distribution      
 Gene set null distribution of ES for **IFNALPHA\_HCC\_UP**
