# Supplementary material for: The rapamycin-regulated gene expression signature determines prognosis for breast cancer
Source: Mol Cancer. 2009 Sep 24;8:75. doi: 10.1186/1476-4598-8-75 (PMC2761377; doi:10.1186/1476-4598-8-75)
Supplement: Additional file 3 — Gene set enrichment analysis of in vivo data, treatment series. The data provided represent the treatment series of GSEA. This compressed file contains "Treatment" shortcut file and "GSEA_treatment" folder. Clicking on "Treatment" shortcut opens the index file providing access to analysis files contained in the "GSEA_treatment" folder. [file 1476-4598-8-75-S3.zip › GSEA_treatment/IFNALPHA_NL_UP.html]

Details for gene set IFNALPHA\_NL\_UP[GSEA]

|  || Dataset | gsea\_treatment\_collapsed |
| Phenotype | NoPhenotypeAvailable |
| Upregulated in class | na\_pos |
| GeneSet | IFNALPHA\_NL\_UP |
| Enrichment Score (ES) | 0.75754046 |
| Normalized Enrichment Score (NES) | 1.9984746 |
| Nominal p-value | 0.0 |
| FDR q-value | 1.2358985E-4 |
| FWER p-Value | 0.0010 |
Table: GSEA Results Summary

  

Fig 1: Enrichment plot: IFNALPHA\_NL\_UP      
 Profile of the Running ES Score & Positions of GeneSet Members on the Rank Ordered List

  

| PROBE | GENE SYMBOL | GENE\_TITLE | RANK IN GENE LIST | RANK METRIC SCORE | RUNNING ES | CORE ENRICHMENT || 1 | MX1 |  |  | 24 | 0.773 | 0.0856 | Yes |
| 2 | IFI27 |  |  | 32 | 0.725 | 0.1666 | Yes |
| 3 | ISG15 |  |  | 59 | 0.644 | 0.2377 | Yes |
| 4 | LGALS3BP |  |  | 61 | 0.643 | 0.3098 | Yes |
| 5 | IFITM1 |  |  | 79 | 0.609 | 0.3774 | Yes |
| 6 | HLA-E |  |  | 126 | 0.545 | 0.4363 | Yes |
| 7 | TNFSF10 |  |  | 160 | 0.518 | 0.4929 | Yes |
| 8 | PSME1 |  |  | 513 | 0.402 | 0.5209 | Yes |
| 9 | IRF1 |  |  | 551 | 0.393 | 0.5632 | Yes |
| 10 | IRF7 |  |  | 639 | 0.378 | 0.6014 | Yes |
| 11 | BST2 |  |  | 657 | 0.374 | 0.6426 | Yes |
| 12 | PLSCR1 |  |  | 717 | 0.365 | 0.6806 | Yes |
| 13 | AKR7A2 |  |  | 809 | 0.352 | 0.7158 | Yes |
| 14 | UBE2L6 |  |  | 980 | 0.333 | 0.7449 | Yes |
| 15 | STAT1 |  |  | 1401 | 0.294 | 0.7575 | Yes |
| 16 | CALR |  |  | 2783 | 0.219 | 0.7150 | No |
| 17 | UBE2D3 |  |  | 2819 | 0.217 | 0.7377 | No |
| 18 | RSAD2 |  |  | 3107 | 0.206 | 0.7470 | No |
| 19 | RFC2 |  |  | 3877 | 0.183 | 0.7301 | No |
| 20 | NTRK1 |  |  | 3918 | 0.182 | 0.7486 | No |
| 21 | PIAS4 |  |  | 4415 | 0.169 | 0.7434 | No |
| 22 | KLK3 |  |  | 8478 | 0.094 | 0.5566 | No |
| 23 | KIAA0409 |  |  | 9465 | 0.080 | 0.5177 | No |
| 24 | VCAM1 |  |  | 13537 | 0.027 | 0.3229 | No |
| 25 | IFI44L |  |  | 14255 | 0.017 | 0.2900 | No |
| 26 | E2F2 |  |  | 17657 | -0.043 | 0.1296 | No |
| 27 | MX2 |  |  | 19725 | -0.121 | 0.0428 | No |
Table: GSEA details [plain text format]

  

Fig 2: IFNALPHA\_NL\_UP: Random ES distribution      
 Gene set null distribution of ES for **IFNALPHA\_NL\_UP**
