# Supplementary material for: The rapamycin-regulated gene expression signature determines prognosis for breast cancer
Source: Mol Cancer. 2009 Sep 24;8:75. doi: 10.1186/1476-4598-8-75 (PMC2761377; doi:10.1186/1476-4598-8-75)
Supplement: Additional file 3 — Gene set enrichment analysis of in vivo data, treatment series. The data provided represent the treatment series of GSEA. This compressed file contains "Treatment" shortcut file and "GSEA_treatment" folder. Clicking on "Treatment" shortcut opens the index file providing access to analysis files contained in the "GSEA_treatment" folder. [file 1476-4598-8-75-S3.zip › GSEA_treatment/IFNA_HCMV_6HRS_UP.html]

Details for gene set IFNA\_HCMV\_6HRS\_UP[GSEA]

|  || Dataset | gsea\_treatment\_collapsed |
| Phenotype | NoPhenotypeAvailable |
| Upregulated in class | na\_pos |
| GeneSet | IFNA\_HCMV\_6HRS\_UP |
| Enrichment Score (ES) | 0.61756986 |
| Normalized Enrichment Score (NES) | 1.7350956 |
| Nominal p-value | 0.0 |
| FDR q-value | 0.009041482 |
| FWER p-Value | 0.247 |
Table: GSEA Results Summary

  

Fig 1: Enrichment plot: IFNA\_HCMV\_6HRS\_UP      
 Profile of the Running ES Score & Positions of GeneSet Members on the Rank Ordered List

  

| PROBE | GENE SYMBOL | GENE\_TITLE | RANK IN GENE LIST | RANK METRIC SCORE | RUNNING ES | CORE ENRICHMENT || 1 | TRIM22 |  |  | 8 | 1.024 | 0.0823 | Yes |
| 2 | MX1 |  |  | 24 | 0.773 | 0.1441 | Yes |
| 3 | IFI27 |  |  | 32 | 0.725 | 0.2023 | Yes |
| 4 | ISG15 |  |  | 59 | 0.644 | 0.2531 | Yes |
| 5 | PSMB9 |  |  | 120 | 0.554 | 0.2950 | Yes |
| 6 | TNFSF10 |  |  | 160 | 0.518 | 0.3349 | Yes |
| 7 | RARRES3 |  |  | 182 | 0.503 | 0.3746 | Yes |
| 8 | OAS1 |  |  | 255 | 0.466 | 0.4088 | Yes |
| 9 | RGL1 |  |  | 420 | 0.421 | 0.4348 | Yes |
| 10 | IRF7 |  |  | 639 | 0.378 | 0.4547 | Yes |
| 11 | BST2 |  |  | 657 | 0.374 | 0.4841 | Yes |
| 12 | IFIT5 |  |  | 667 | 0.372 | 0.5137 | Yes |
| 13 | PLSCR1 |  |  | 717 | 0.365 | 0.5408 | Yes |
| 14 | CASP1 |  |  | 732 | 0.363 | 0.5694 | Yes |
| 15 | UBE2L6 |  |  | 980 | 0.333 | 0.5843 | Yes |
| 16 | PSCD1 |  |  | 1265 | 0.305 | 0.5951 | Yes |
| 17 | STAT1 |  |  | 1401 | 0.294 | 0.6124 | Yes |
| 18 | IFIT3 |  |  | 1814 | 0.265 | 0.6138 | Yes |
| 19 | SAMHD1 |  |  | 2149 | 0.248 | 0.6176 | Yes |
| 20 | IL15 |  |  | 2716 | 0.222 | 0.6080 | No |
| 21 | RSAD2 |  |  | 3107 | 0.206 | 0.6057 | No |
| 22 | IL15RA |  |  | 3488 | 0.194 | 0.6029 | No |
| 23 | ISG20 |  |  | 3782 | 0.186 | 0.6036 | No |
| 24 | ECGF1 |  |  | 4751 | 0.161 | 0.5695 | No |
| 25 | TRIM14 |  |  | 4986 | 0.156 | 0.5707 | No |
| 26 | TLR3 |  |  | 5194 | 0.151 | 0.5728 | No |
| 27 | PML |  |  | 5234 | 0.150 | 0.5830 | No |
| 28 | PDZD2 |  |  | 5612 | 0.142 | 0.5762 | No |
| 29 | INDO |  |  | 5656 | 0.141 | 0.5855 | No |
| 30 | TREX1 |  |  | 5987 | 0.135 | 0.5804 | No |
| 31 | BIRC4BP |  |  | 7047 | 0.117 | 0.5384 | No |
| 32 | RBCK1 |  |  | 7510 | 0.109 | 0.5247 | No |
| 33 | MYD88 |  |  | 7910 | 0.103 | 0.5136 | No |
| 34 | ZBTB20 |  |  | 8368 | 0.096 | 0.4992 | No |
| 35 | KIF5C |  |  | 9192 | 0.084 | 0.4659 | No |
| 36 | BAZ1A |  |  | 9626 | 0.078 | 0.4512 | No |
| 37 | GMPR |  |  | 10165 | 0.071 | 0.4307 | No |
| 38 | CCL8 |  |  | 10622 | 0.064 | 0.4137 | No |
| 39 | STX11 |  |  | 10806 | 0.062 | 0.4098 | No |
| 40 | OASL |  |  | 12433 | 0.041 | 0.3341 | No |
| 41 | OAS2 |  |  | 12543 | 0.040 | 0.3320 | No |
| 42 | TDRD7 |  |  | 13343 | 0.029 | 0.2955 | No |
| 43 | IFI44L |  |  | 14255 | 0.017 | 0.2526 | No |
| 44 | NMI |  |  | 16092 | -0.012 | 0.1642 | No |
| 45 | TRIM21 |  |  | 16730 | -0.024 | 0.1351 | No |
| 46 | MAP2 |  |  | 17018 | -0.029 | 0.1235 | No |
| 47 | RABGAP1L |  |  | 17466 | -0.039 | 0.1049 | No |
| 48 | WARS |  |  | 17621 | -0.043 | 0.1008 | No |
| 49 | C1ORF38 |  |  | 18233 | -0.058 | 0.0758 | No |
| 50 | MELK |  |  | 19351 | -0.101 | 0.0296 | No |
| 51 | GBP2 |  |  | 19549 | -0.111 | 0.0290 | No |
| 52 | MX2 |  |  | 19725 | -0.121 | 0.0303 | No |
| 53 | GCH1 |  |  | 20092 | -0.155 | 0.0250 | No |
Table: GSEA details [plain text format]

  

Fig 2: IFNA\_HCMV\_6HRS\_UP: Random ES distribution      
 Gene set null distribution of ES for **IFNA\_HCMV\_6HRS\_UP**
