# Supplementary material for: The rapamycin-regulated gene expression signature determines prognosis for breast cancer
Source: Mol Cancer. 2009 Sep 24;8:75. doi: 10.1186/1476-4598-8-75 (PMC2761377; doi:10.1186/1476-4598-8-75)
Supplement: Additional file 3 — Gene set enrichment analysis of in vivo data, treatment series. The data provided represent the treatment series of GSEA. This compressed file contains "Treatment" shortcut file and "GSEA_treatment" folder. Clicking on "Treatment" shortcut opens the index file providing access to analysis files contained in the "GSEA_treatment" folder. [file 1476-4598-8-75-S3.zip › GSEA_treatment/IFN_ALL_UP.html]

Details for gene set IFN\_ALL\_UP[GSEA]

|  || Dataset | gsea\_treatment\_collapsed |
| Phenotype | NoPhenotypeAvailable |
| Upregulated in class | na\_pos |
| GeneSet | IFN\_ALL\_UP |
| Enrichment Score (ES) | 0.7234134 |
| Normalized Enrichment Score (NES) | 1.7701024 |
| Nominal p-value | 0.002173913 |
| FDR q-value | 0.004951242 |
| FWER p-Value | 0.119 |
Table: GSEA Results Summary

  

Fig 1: Enrichment plot: IFN\_ALL\_UP      
 Profile of the Running ES Score & Positions of GeneSet Members on the Rank Ordered List

  

| PROBE | GENE SYMBOL | GENE\_TITLE | RANK IN GENE LIST | RANK METRIC SCORE | RUNNING ES | CORE ENRICHMENT || 1 | IFITM1 |  |  | 79 | 0.609 | 0.1266 | Yes |
| 2 | HLA-E |  |  | 126 | 0.545 | 0.2411 | Yes |
| 3 | RBBP4 |  |  | 474 | 0.410 | 0.3121 | Yes |
| 4 | IRF1 |  |  | 551 | 0.393 | 0.3926 | Yes |
| 5 | C1S |  |  | 714 | 0.366 | 0.4631 | Yes |
| 6 | ADAR |  |  | 885 | 0.344 | 0.5285 | Yes |
| 7 | PLOD2 |  |  | 1140 | 0.316 | 0.5840 | Yes |
| 8 | STAT1 |  |  | 1401 | 0.294 | 0.6344 | Yes |
| 9 | IFIT3 |  |  | 1814 | 0.265 | 0.6712 | Yes |
| 10 | BTG1 |  |  | 2593 | 0.227 | 0.6819 | Yes |
| 11 | CASP8 |  |  | 2719 | 0.222 | 0.7234 | Yes |
| 12 | BAG1 |  |  | 4977 | 0.156 | 0.6473 | No |
| 13 | PML |  |  | 5234 | 0.150 | 0.6670 | No |
| 14 | IL6 |  |  | 5899 | 0.137 | 0.6640 | No |
| 15 | XRCC6 |  |  | 7760 | 0.106 | 0.5963 | No |
| 16 | MAP3K10 |  |  | 8577 | 0.093 | 0.5765 | No |
| 17 | NMI |  |  | 16092 | -0.012 | 0.2141 | No |
| 18 | TRIM21 |  |  | 16730 | -0.024 | 0.1882 | No |
Table: GSEA details [plain text format]

  

Fig 2: IFN\_ALL\_UP: Random ES distribution      
 Gene set null distribution of ES for **IFN\_ALL\_UP**
