# Supplementary material for: The rapamycin-regulated gene expression signature determines prognosis for breast cancer
Source: Mol Cancer. 2009 Sep 24;8:75. doi: 10.1186/1476-4598-8-75 (PMC2761377; doi:10.1186/1476-4598-8-75)
Supplement: Additional file 3 — Gene set enrichment analysis of in vivo data, treatment series. The data provided represent the treatment series of GSEA. This compressed file contains "Treatment" shortcut file and "GSEA_treatment" folder. Clicking on "Treatment" shortcut opens the index file providing access to analysis files contained in the "GSEA_treatment" folder. [file 1476-4598-8-75-S3.zip › GSEA_treatment/IFN_ALPHA_UP.html]

Details for gene set IFN\_ALPHA\_UP[GSEA]

|  || Dataset | gsea\_treatment\_collapsed |
| Phenotype | NoPhenotypeAvailable |
| Upregulated in class | na\_pos |
| GeneSet | IFN\_ALPHA\_UP |
| Enrichment Score (ES) | 0.6795792 |
| Normalized Enrichment Score (NES) | 1.867954 |
| Nominal p-value | 0.0 |
| FDR q-value | 0.0013003831 |
| FWER p-Value | 0.021 |
Table: GSEA Results Summary

  

Fig 1: Enrichment plot: IFN\_ALPHA\_UP      
 Profile of the Running ES Score & Positions of GeneSet Members on the Rank Ordered List

  

| PROBE | GENE SYMBOL | GENE\_TITLE | RANK IN GENE LIST | RANK METRIC SCORE | RUNNING ES | CORE ENRICHMENT || 1 | TRIM22 |  |  | 8 | 1.024 | 0.0965 | Yes |
| 2 | MX1 |  |  | 24 | 0.773 | 0.1690 | Yes |
| 3 | ISG15 |  |  | 59 | 0.644 | 0.2283 | Yes |
| 4 | IFITM1 |  |  | 79 | 0.609 | 0.2851 | Yes |
| 5 | HLA-E |  |  | 126 | 0.545 | 0.3345 | Yes |
| 6 | OAS1 |  |  | 255 | 0.466 | 0.3724 | Yes |
| 7 | PDXK |  |  | 402 | 0.425 | 0.4056 | Yes |
| 8 | RBBP4 |  |  | 474 | 0.410 | 0.4409 | Yes |
| 9 | IRF1 |  |  | 551 | 0.393 | 0.4745 | Yes |
| 10 | BST2 |  |  | 657 | 0.374 | 0.5048 | Yes |
| 11 | C1S |  |  | 714 | 0.366 | 0.5367 | Yes |
| 12 | SKP1A |  |  | 832 | 0.349 | 0.5641 | Yes |
| 13 | ADAR |  |  | 885 | 0.344 | 0.5941 | Yes |
| 14 | PLOD2 |  |  | 1140 | 0.316 | 0.6117 | Yes |
| 15 | STAT1 |  |  | 1401 | 0.294 | 0.6269 | Yes |
| 16 | IRF2 |  |  | 1500 | 0.287 | 0.6493 | Yes |
| 17 | IFIT3 |  |  | 1814 | 0.265 | 0.6592 | Yes |
| 18 | BBC3 |  |  | 2497 | 0.230 | 0.6478 | Yes |
| 19 | BTG1 |  |  | 2593 | 0.227 | 0.6646 | Yes |
| 20 | CASP8 |  |  | 2719 | 0.222 | 0.6796 | Yes |
| 21 | FAS |  |  | 3778 | 0.186 | 0.6457 | No |
| 22 | SDCBP |  |  | 3795 | 0.185 | 0.6625 | No |
| 23 | BAG1 |  |  | 4977 | 0.156 | 0.6198 | No |
| 24 | TRIM14 |  |  | 4986 | 0.156 | 0.6342 | No |
| 25 | PML |  |  | 5234 | 0.150 | 0.6364 | No |
| 26 | SHFM1 |  |  | 5405 | 0.147 | 0.6420 | No |
| 27 | EPS15 |  |  | 5695 | 0.140 | 0.6413 | No |
| 28 | IL6 |  |  | 5899 | 0.137 | 0.6443 | No |
| 29 | XRCC6 |  |  | 7760 | 0.106 | 0.5639 | No |
| 30 | MAP3K10 |  |  | 8577 | 0.093 | 0.5330 | No |
| 31 | GMPR |  |  | 10165 | 0.071 | 0.4625 | No |
| 32 | PPP3CA |  |  | 11496 | 0.053 | 0.4029 | No |
| 33 | DDX17 |  |  | 11694 | 0.051 | 0.3982 | No |
| 34 | OAS2 |  |  | 12543 | 0.040 | 0.3607 | No |
| 35 | TEAD4 |  |  | 13666 | 0.025 | 0.3085 | No |
| 36 | NMI |  |  | 16092 | -0.012 | 0.1917 | No |
| 37 | TRIM21 |  |  | 16730 | -0.024 | 0.1630 | No |
| 38 | COL16A1 |  |  | 17177 | -0.033 | 0.1444 | No |
| 39 | VEGFC |  |  | 19585 | -0.113 | 0.0381 | No |
| 40 | MX2 |  |  | 19725 | -0.121 | 0.0428 | No |
Table: GSEA details [plain text format]

  

Fig 2: IFN\_ALPHA\_UP: Random ES distribution      
 Gene set null distribution of ES for **IFN\_ALPHA\_UP**
