# Supplementary material for: The rapamycin-regulated gene expression signature determines prognosis for breast cancer
Source: Mol Cancer. 2009 Sep 24;8:75. doi: 10.1186/1476-4598-8-75 (PMC2761377; doi:10.1186/1476-4598-8-75)
Supplement: Additional file 3 — Gene set enrichment analysis of in vivo data, treatment series. The data provided represent the treatment series of GSEA. This compressed file contains "Treatment" shortcut file and "GSEA_treatment" folder. Clicking on "Treatment" shortcut opens the index file providing access to analysis files contained in the "GSEA_treatment" folder. [file 1476-4598-8-75-S3.zip › GSEA_treatment/IFN_BETA_UP.html]

Details for gene set IFN\_BETA\_UP[GSEA]

|  || Dataset | gsea\_treatment\_collapsed |
| Phenotype | NoPhenotypeAvailable |
| Upregulated in class | na\_pos |
| GeneSet | IFN\_BETA\_UP |
| Enrichment Score (ES) | 0.62303126 |
| Normalized Enrichment Score (NES) | 1.798259 |
| Nominal p-value | 0.0 |
| FDR q-value | 0.0028859098 |
| FWER p-Value | 0.065 |
Table: GSEA Results Summary

  

Fig 1: Enrichment plot: IFN\_BETA\_UP      
 Profile of the Running ES Score & Positions of GeneSet Members on the Rank Ordered List

  

| PROBE | GENE SYMBOL | GENE\_TITLE | RANK IN GENE LIST | RANK METRIC SCORE | RUNNING ES | CORE ENRICHMENT || 1 | TRIM22 |  |  | 8 | 1.024 | 0.0668 | Yes |
| 2 | MX1 |  |  | 24 | 0.773 | 0.1167 | Yes |
| 3 | ISG15 |  |  | 59 | 0.644 | 0.1573 | Yes |
| 4 | IFITM1 |  |  | 79 | 0.609 | 0.1964 | Yes |
| 5 | HLA-E |  |  | 126 | 0.545 | 0.2299 | Yes |
| 6 | CALD1 |  |  | 177 | 0.506 | 0.2606 | Yes |
| 7 | OAS1 |  |  | 255 | 0.466 | 0.2875 | Yes |
| 8 | PDXK |  |  | 402 | 0.425 | 0.3083 | Yes |
| 9 | GMFB |  |  | 439 | 0.416 | 0.3338 | Yes |
| 10 | RBBP4 |  |  | 474 | 0.410 | 0.3591 | Yes |
| 11 | IRF1 |  |  | 551 | 0.393 | 0.3812 | Yes |
| 12 | BST2 |  |  | 657 | 0.374 | 0.4006 | Yes |
| 13 | IFIT5 |  |  | 667 | 0.372 | 0.4246 | Yes |
| 14 | C1S |  |  | 714 | 0.366 | 0.4463 | Yes |
| 15 | ADAR |  |  | 885 | 0.344 | 0.4606 | Yes |
| 16 | CD164 |  |  | 921 | 0.339 | 0.4811 | Yes |
| 17 | PLOD2 |  |  | 1140 | 0.316 | 0.4913 | Yes |
| 18 | BCLAF1 |  |  | 1313 | 0.301 | 0.5026 | Yes |
| 19 | HADH |  |  | 1330 | 0.300 | 0.5215 | Yes |
| 20 | STAT1 |  |  | 1401 | 0.294 | 0.5374 | Yes |
| 21 | PARP1 |  |  | 1466 | 0.289 | 0.5532 | Yes |
| 22 | IRF2 |  |  | 1500 | 0.287 | 0.5704 | Yes |
| 23 | IFIT3 |  |  | 1814 | 0.265 | 0.5726 | Yes |
| 24 | EIF2AK2 |  |  | 1990 | 0.255 | 0.5808 | Yes |
| 25 | HADHB |  |  | 2028 | 0.254 | 0.5957 | Yes |
| 26 | PARP4 |  |  | 2069 | 0.252 | 0.6103 | Yes |
| 27 | BTG1 |  |  | 2593 | 0.227 | 0.5997 | Yes |
| 28 | SRP9 |  |  | 2599 | 0.226 | 0.6143 | Yes |
| 29 | CASP8 |  |  | 2719 | 0.222 | 0.6230 | Yes |
| 30 | DDX1 |  |  | 3384 | 0.198 | 0.6037 | No |
| 31 | CTR9 |  |  | 3425 | 0.196 | 0.6146 | No |
| 32 | IQGAP1 |  |  | 4143 | 0.176 | 0.5912 | No |
| 33 | PTPN11 |  |  | 4319 | 0.171 | 0.5939 | No |
| 34 | FUBP1 |  |  | 4739 | 0.161 | 0.5840 | No |
| 35 | BAG1 |  |  | 4977 | 0.156 | 0.5827 | No |
| 36 | TRIM14 |  |  | 4986 | 0.156 | 0.5926 | No |
| 37 | PML |  |  | 5234 | 0.150 | 0.5904 | No |
| 38 | SHFM1 |  |  | 5405 | 0.147 | 0.5917 | No |
| 39 | RBMX |  |  | 5643 | 0.141 | 0.5895 | No |
| 40 | EPS15 |  |  | 5695 | 0.140 | 0.5962 | No |
| 41 | IL6 |  |  | 5899 | 0.137 | 0.5953 | No |
| 42 | PPP5C |  |  | 6311 | 0.130 | 0.5838 | No |
| 43 | ARID3A |  |  | 6473 | 0.127 | 0.5843 | No |
| 44 | XRCC6 |  |  | 7760 | 0.106 | 0.5286 | No |
| 45 | MAP3K10 |  |  | 8577 | 0.093 | 0.4949 | No |
| 46 | PLAUR |  |  | 9123 | 0.085 | 0.4740 | No |
| 47 | PHLDA1 |  |  | 9954 | 0.074 | 0.4384 | No |
| 48 | ELF1 |  |  | 11114 | 0.058 | 0.3858 | No |
| 49 | PPP3CA |  |  | 11496 | 0.053 | 0.3708 | No |
| 50 | EPRS |  |  | 12393 | 0.042 | 0.3299 | No |
| 51 | OAS2 |  |  | 12543 | 0.040 | 0.3252 | No |
| 52 | ADAM9 |  |  | 12576 | 0.039 | 0.3263 | No |
| 53 | ZRF1 |  |  | 13268 | 0.031 | 0.2947 | No |
| 54 | ETS2 |  |  | 14747 | 0.010 | 0.2234 | No |
| 55 | TMEM1 |  |  | 15308 | 0.001 | 0.1962 | No |
| 56 | HIF1A |  |  | 15637 | -0.004 | 0.1805 | No |
| 57 | NMI |  |  | 16092 | -0.012 | 0.1592 | No |
| 58 | TRIM21 |  |  | 16730 | -0.024 | 0.1297 | No |
| 59 | CSRP3 |  |  | 16774 | -0.024 | 0.1292 | No |
| 60 | AP3M2 |  |  | 19087 | -0.088 | 0.0224 | No |
| 61 | FOSL1 |  |  | 19426 | -0.104 | 0.0128 | No |
| 62 | MX2 |  |  | 19725 | -0.121 | 0.0063 | No |
| 63 | CEBPD |  |  | 19851 | -0.131 | 0.0088 | No |
| 64 | DDX21 |  |  | 20219 | -0.176 | 0.0025 | No |
| 65 | PMAIP1 |  |  | 20440 | -0.248 | 0.0080 | No |
Table: GSEA details [plain text format]

  

Fig 2: IFN\_BETA\_UP: Random ES distribution      
 Gene set null distribution of ES for **IFN\_BETA\_UP**
