# Supplementary material for: The rapamycin-regulated gene expression signature determines prognosis for breast cancer
Source: Mol Cancer. 2009 Sep 24;8:75. doi: 10.1186/1476-4598-8-75 (PMC2761377; doi:10.1186/1476-4598-8-75)
Supplement: Additional file 3 — Gene set enrichment analysis of in vivo data, treatment series. The data provided represent the treatment series of GSEA. This compressed file contains "Treatment" shortcut file and "GSEA_treatment" folder. Clicking on "Treatment" shortcut opens the index file providing access to analysis files contained in the "GSEA_treatment" folder. [file 1476-4598-8-75-S3.zip › GSEA_treatment/IFN_GAMMA_UP.html]

Details for gene set IFN\_GAMMA\_UP[GSEA]

|  || Dataset | gsea\_treatment\_collapsed |
| Phenotype | NoPhenotypeAvailable |
| Upregulated in class | na\_pos |
| GeneSet | IFN\_GAMMA\_UP |
| Enrichment Score (ES) | 0.61835366 |
| Normalized Enrichment Score (NES) | 1.6926198 |
| Nominal p-value | 0.0 |
| FDR q-value | 0.013552965 |
| FWER p-Value | 0.462 |
Table: GSEA Results Summary

  

Fig 1: Enrichment plot: IFN\_GAMMA\_UP      
 Profile of the Running ES Score & Positions of GeneSet Members on the Rank Ordered List

  

| PROBE | GENE SYMBOL | GENE\_TITLE | RANK IN GENE LIST | RANK METRIC SCORE | RUNNING ES | CORE ENRICHMENT || 1 | IFITM1 |  |  | 79 | 0.609 | 0.0701 | Yes |
| 2 | HLA-E |  |  | 126 | 0.545 | 0.1340 | Yes |
| 3 | ICAM1 |  |  | 408 | 0.423 | 0.1717 | Yes |
| 4 | RBBP4 |  |  | 474 | 0.410 | 0.2184 | Yes |
| 5 | PSME1 |  |  | 513 | 0.402 | 0.2653 | Yes |
| 6 | IRF1 |  |  | 551 | 0.393 | 0.3113 | Yes |
| 7 | C1S |  |  | 714 | 0.366 | 0.3479 | Yes |
| 8 | ADAR |  |  | 885 | 0.344 | 0.3813 | Yes |
| 9 | PLOD2 |  |  | 1140 | 0.316 | 0.4074 | Yes |
| 10 | HADH |  |  | 1330 | 0.300 | 0.4346 | Yes |
| 11 | STAT1 |  |  | 1401 | 0.294 | 0.4669 | Yes |
| 12 | PARP1 |  |  | 1466 | 0.289 | 0.4989 | Yes |
| 13 | SF3A1 |  |  | 1513 | 0.286 | 0.5313 | Yes |
| 14 | IFIT3 |  |  | 1814 | 0.265 | 0.5490 | Yes |
| 15 | HADHB |  |  | 2028 | 0.254 | 0.5694 | Yes |
| 16 | BBC3 |  |  | 2497 | 0.230 | 0.5746 | Yes |
| 17 | BTG1 |  |  | 2593 | 0.227 | 0.5975 | Yes |
| 18 | CASP8 |  |  | 2719 | 0.222 | 0.6184 | Yes |
| 19 | IL15RA |  |  | 3488 | 0.194 | 0.6045 | No |
| 20 | FAS |  |  | 3778 | 0.186 | 0.6130 | No |
| 21 | BAG1 |  |  | 4977 | 0.156 | 0.5738 | No |
| 22 | PML |  |  | 5234 | 0.150 | 0.5795 | No |
| 23 | IL6 |  |  | 5899 | 0.137 | 0.5638 | No |
| 24 | PPP5C |  |  | 6311 | 0.130 | 0.5596 | No |
| 25 | VAT1 |  |  | 6606 | 0.125 | 0.5605 | No |
| 26 | XRCC6 |  |  | 7760 | 0.106 | 0.5173 | No |
| 27 | MAP3K10 |  |  | 8577 | 0.093 | 0.4889 | No |
| 28 | PHLDA1 |  |  | 9954 | 0.074 | 0.4309 | No |
| 29 | TEAD4 |  |  | 13666 | 0.025 | 0.2535 | No |
| 30 | EIF2B1 |  |  | 13897 | 0.022 | 0.2450 | No |
| 31 | PRAME |  |  | 15340 | 0.000 | 0.1750 | No |
| 32 | NMI |  |  | 16092 | -0.012 | 0.1399 | No |
| 33 | TRIM21 |  |  | 16730 | -0.024 | 0.1118 | No |
| 34 | COL16A1 |  |  | 17177 | -0.033 | 0.0941 | No |
| 35 | FOSL1 |  |  | 19426 | -0.104 | -0.0025 | No |
| 36 | VEGFC |  |  | 19585 | -0.113 | 0.0035 | No |
| 37 | CEBPD |  |  | 19851 | -0.131 | 0.0065 | No |
| 38 | PMAIP1 |  |  | 20440 | -0.248 | 0.0080 | No |
Table: GSEA details [plain text format]

  

Fig 2: IFN\_GAMMA\_UP: Random ES distribution      
 Gene set null distribution of ES for **IFN\_GAMMA\_UP**
