# Supplementary material for: The rapamycin-regulated gene expression signature determines prognosis for breast cancer
Source: Mol Cancer. 2009 Sep 24;8:75. doi: 10.1186/1476-4598-8-75 (PMC2761377; doi:10.1186/1476-4598-8-75)
Supplement: Additional file 3 — Gene set enrichment analysis of in vivo data, treatment series. The data provided represent the treatment series of GSEA. This compressed file contains "Treatment" shortcut file and "GSEA_treatment" folder. Clicking on "Treatment" shortcut opens the index file providing access to analysis files contained in the "GSEA_treatment" folder. [file 1476-4598-8-75-S3.zip › GSEA_treatment/IGF_VS_PDGF_UP.html]

Details for gene set IGF\_VS\_PDGF\_UP[GSEA]

|  || Dataset | gsea\_treatment\_collapsed |
| Phenotype | NoPhenotypeAvailable |
| Upregulated in class | na\_neg |
| GeneSet | IGF\_VS\_PDGF\_UP |
| Enrichment Score (ES) | -0.27434698 |
| Normalized Enrichment Score (NES) | -1.2915024 |
| Nominal p-value | 0.0 |
| FDR q-value | 0.27215612 |
| FWER p-Value | 1.0 |
Table: GSEA Results Summary

  

Fig 1: Enrichment plot: IGF\_VS\_PDGF\_UP      
 Profile of the Running ES Score & Positions of GeneSet Members on the Rank Ordered List

  

| PROBE | GENE SYMBOL | GENE\_TITLE | RANK IN GENE LIST | RANK METRIC SCORE | RUNNING ES | CORE ENRICHMENT || 1 | ERBB3 |  |  | 223 | 0.483 | 0.0633 | No |
| 2 | BRWD1 |  |  | 3161 | 0.204 | -0.0483 | No |
| 3 | TPM1 |  |  | 3849 | 0.184 | -0.0536 | No |
| 4 | MYH3 |  |  | 3857 | 0.184 | -0.0257 | No |
| 5 | C11ORF67 |  |  | 3869 | 0.183 | 0.0019 | No |
| 6 | DAAM2 |  |  | 4238 | 0.173 | 0.0105 | No |
| 7 | DDC |  |  | 6093 | 0.134 | -0.0592 | No |
| 8 | HDAC11 |  |  | 7458 | 0.110 | -0.1087 | No |
| 9 | ZNF238 |  |  | 7549 | 0.109 | -0.0964 | No |
| 10 | SMTN |  |  | 7857 | 0.104 | -0.0954 | No |
| 11 | TTN |  |  | 7875 | 0.104 | -0.0802 | No |
| 12 | NCAM1 |  |  | 8315 | 0.097 | -0.0868 | No |
| 13 | SYNPO2L |  |  | 8569 | 0.093 | -0.0848 | No |
| 14 | TNNC1 |  |  | 8600 | 0.092 | -0.0721 | No |
| 15 | HSPB2 |  |  | 8872 | 0.088 | -0.0717 | No |
| 16 | FLJ30294 |  |  | 8893 | 0.088 | -0.0592 | No |
| 17 | MYL4 |  |  | 8971 | 0.087 | -0.0496 | No |
| 18 | C1QTNF3 |  |  | 9085 | 0.086 | -0.0420 | No |
| 19 | FAM8A1 |  |  | 9482 | 0.080 | -0.0489 | No |
| 20 | KBTBD5 |  |  | 9527 | 0.079 | -0.0389 | No |
| 21 | LDB3 |  |  | 9588 | 0.078 | -0.0298 | No |
| 22 | CKM |  |  | 9592 | 0.078 | -0.0179 | No |
| 23 | ACTN3 |  |  | 9607 | 0.078 | -0.0065 | No |
| 24 | GATM |  |  | 9929 | 0.074 | -0.0108 | No |
| 25 | CNR1 |  |  | 10080 | 0.072 | -0.0071 | No |
| 26 | ATP2A1 |  |  | 10251 | 0.070 | -0.0047 | No |
| 27 | DCAMKL1 |  |  | 10592 | 0.065 | -0.0113 | No |
| 28 | DKFZP564O0823 |  |  | 10867 | 0.062 | -0.0151 | No |
| 29 | SGCA |  |  | 10886 | 0.061 | -0.0066 | No |
| 30 | ENAH |  |  | 11461 | 0.054 | -0.0263 | No |
| 31 | NEIL1 |  |  | 11663 | 0.051 | -0.0282 | No |
| 32 | FLJ22349 |  |  | 12549 | 0.040 | -0.0652 | No |
| 33 | ATP2B4 |  |  | 12873 | 0.036 | -0.0755 | No |
| 34 | MYOM2 |  |  | 13122 | 0.032 | -0.0826 | No |
| 35 | FBXL22 |  |  | 13209 | 0.031 | -0.0820 | No |
| 36 | TNNT2 |  |  | 13404 | 0.029 | -0.0870 | No |
| 37 | SMYD1 |  |  | 13883 | 0.022 | -0.1069 | No |
| 38 | SDPR |  |  | 14058 | 0.020 | -0.1123 | No |
| 39 | TNNT1 |  |  | 14123 | 0.019 | -0.1126 | No |
| 40 | PKIA |  |  | 14455 | 0.014 | -0.1265 | No |
| 41 | PYGM |  |  | 14584 | 0.012 | -0.1309 | No |
| 42 | USP2 |  |  | 14593 | 0.012 | -0.1295 | No |
| 43 | FST |  |  | 14625 | 0.012 | -0.1292 | No |
| 44 | TPM2 |  |  | 15180 | 0.003 | -0.1557 | No |
| 45 | SOX8 |  |  | 15229 | 0.002 | -0.1576 | No |
| 46 | NPNT |  |  | 15468 | -0.001 | -0.1690 | No |
| 47 | SRL |  |  | 15768 | -0.006 | -0.1826 | No |
| 48 | COX6A2 |  |  | 15854 | -0.008 | -0.1856 | No |
| 49 | TNNT3 |  |  | 16723 | -0.024 | -0.2242 | No |
| 50 | RYR1 |  |  | 16770 | -0.024 | -0.2228 | No |
| 51 | TMEM38A |  |  | 16817 | -0.025 | -0.2212 | No |
| 52 | SMPX |  |  | 16849 | -0.025 | -0.2188 | No |
| 53 | FMO1 |  |  | 16947 | -0.027 | -0.2193 | No |
| 54 | MYOG |  |  | 17462 | -0.039 | -0.2383 | No |
| 55 | DUSP27 |  |  | 17471 | -0.039 | -0.2327 | No |
| 56 | RB1 |  |  | 17665 | -0.043 | -0.2354 | No |
| 57 | PPFIA4 |  |  | 17673 | -0.044 | -0.2290 | No |
| 58 | SGCG |  |  | 17687 | -0.044 | -0.2229 | No |
| 59 | UNC45B |  |  | 17722 | -0.045 | -0.2177 | No |
| 60 | TNNI1 |  |  | 18043 | -0.053 | -0.2251 | No |
| 61 | HFE2 |  |  | 18184 | -0.056 | -0.2233 | No |
| 62 | MYBPH |  |  | 18380 | -0.062 | -0.2233 | No |
| 63 | CMYA1 |  |  | 19343 | -0.101 | -0.2547 | Yes |
| 64 | PGAM2 |  |  | 19685 | -0.119 | -0.2531 | Yes |
| 65 | ACTC1 |  |  | 20123 | -0.160 | -0.2498 | Yes |
| 66 | PRKAG3 |  |  | 20222 | -0.177 | -0.2274 | Yes |
| 67 | FBXO17 |  |  | 20332 | -0.202 | -0.2017 | Yes |
| 68 | ITGB1BP2 |  |  | 20422 | -0.235 | -0.1700 | Yes |
| 69 | GPT2 |  |  | 20527 | -0.344 | -0.1222 | Yes |
| 70 | MYLPF |  |  | 20543 | -0.395 | -0.0622 | Yes |
| 71 | TNNC2 |  |  | 20554 | -0.425 | 0.0025 | Yes |
Table: GSEA details [plain text format]

  

Fig 2: IGF\_VS\_PDGF\_UP: Random ES distribution      
 Gene set null distribution of ES for **IGF\_VS\_PDGF\_UP**
