# Supplementary material for: The rapamycin-regulated gene expression signature determines prognosis for breast cancer
Source: Mol Cancer. 2009 Sep 24;8:75. doi: 10.1186/1476-4598-8-75 (PMC2761377; doi:10.1186/1476-4598-8-75)
Supplement: Additional file 3 — Gene set enrichment analysis of in vivo data, treatment series. The data provided represent the treatment series of GSEA. This compressed file contains "Treatment" shortcut file and "GSEA_treatment" folder. Clicking on "Treatment" shortcut opens the index file providing access to analysis files contained in the "GSEA_treatment" folder. [file 1476-4598-8-75-S3.zip › GSEA_treatment/KIM_TH_CELLS_DN.html]

Details for gene set KIM\_TH\_CELLS\_DN[GSEA]

|  || Dataset | gsea\_treatment\_collapsed |
| Phenotype | NoPhenotypeAvailable |
| Upregulated in class | na\_neg |
| GeneSet | KIM\_TH\_CELLS\_DN |
| Enrichment Score (ES) | -0.21297239 |
| Normalized Enrichment Score (NES) | -0.73121715 |
| Nominal p-value | 0.8076923 |
| FDR q-value | 0.93445885 |
| FWER p-Value | 1.0 |
Table: GSEA Results Summary

  

Fig 1: Enrichment plot: KIM\_TH\_CELLS\_DN      
 Profile of the Running ES Score & Positions of GeneSet Members on the Rank Ordered List

  

| PROBE | GENE SYMBOL | GENE\_TITLE | RANK IN GENE LIST | RANK METRIC SCORE | RUNNING ES | CORE ENRICHMENT || 1 | USP12 |  |  | 3397 | 0.197 | -0.0136 | Yes |
| 2 | DHRS3 |  |  | 4713 | 0.162 | 0.0466 | Yes |
| 3 | RORA |  |  | 6132 | 0.133 | 0.0797 | Yes |
| 4 | SPTBN1 |  |  | 6140 | 0.133 | 0.1813 | Yes |
| 5 | CLASP2 |  |  | 9582 | 0.079 | 0.0747 | No |
| 6 | OSBP |  |  | 9810 | 0.076 | 0.1220 | No |
| 7 | SVIL |  |  | 12494 | 0.040 | 0.0228 | No |
| 8 | DUSP8 |  |  | 15962 | -0.010 | -0.1382 | No |
| 9 | PER1 |  |  | 16113 | -0.012 | -0.1363 | No |
| 10 | ELL2 |  |  | 17124 | -0.031 | -0.1613 | No |
| 11 | RYBP |  |  | 17174 | -0.032 | -0.1390 | No |
| 12 | EDG1 |  |  | 18699 | -0.073 | -0.1572 | No |
| 13 | ZNF331 |  |  | 19186 | -0.093 | -0.1095 | No |
| 14 | PFTK1 |  |  | 19634 | -0.116 | -0.0423 | No |
| 15 | IRS2 |  |  | 19654 | -0.116 | 0.0462 | No |
Table: GSEA details [plain text format]

  

Fig 2: KIM\_TH\_CELLS\_DN: Random ES distribution      
 Gene set null distribution of ES for **KIM\_TH\_CELLS\_DN**
