# Supplementary material for: The rapamycin-regulated gene expression signature determines prognosis for breast cancer
Source: Mol Cancer. 2009 Sep 24;8:75. doi: 10.1186/1476-4598-8-75 (PMC2761377; doi:10.1186/1476-4598-8-75)
Supplement: Additional file 3 — Gene set enrichment analysis of in vivo data, treatment series. The data provided represent the treatment series of GSEA. This compressed file contains "Treatment" shortcut file and "GSEA_treatment" folder. Clicking on "Treatment" shortcut opens the index file providing access to analysis files contained in the "GSEA_treatment" folder. [file 1476-4598-8-75-S3.zip › GSEA_treatment/LEE_E2F1_DN.html]

Details for gene set LEE\_E2F1\_DN[GSEA]

|  || Dataset | gsea\_treatment\_collapsed |
| Phenotype | NoPhenotypeAvailable |
| Upregulated in class | na\_neg |
| GeneSet | LEE\_E2F1\_DN |
| Enrichment Score (ES) | -0.33406043 |
| Normalized Enrichment Score (NES) | -1.5137354 |
| Nominal p-value | 0.0 |
| FDR q-value | 0.16204031 |
| FWER p-Value | 0.837 |
Table: GSEA Results Summary

  

Fig 1: Enrichment plot: LEE\_E2F1\_DN      
 Profile of the Running ES Score & Positions of GeneSet Members on the Rank Ordered List

  

| PROBE | GENE SYMBOL | GENE\_TITLE | RANK IN GENE LIST | RANK METRIC SCORE | RUNNING ES | CORE ENRICHMENT || 1 | EFNA4 |  |  | 434 | 0.418 | 0.0324 | No |
| 2 | OAT |  |  | 970 | 0.334 | 0.0492 | No |
| 3 | PXMP2 |  |  | 1123 | 0.319 | 0.0827 | No |
| 4 | KYNU |  |  | 1426 | 0.292 | 0.1054 | No |
| 5 | GSTO1 |  |  | 1819 | 0.265 | 0.1203 | No |
| 6 | ALDH1A1 |  |  | 1897 | 0.261 | 0.1500 | No |
| 7 | MDH1 |  |  | 2181 | 0.246 | 0.1677 | No |
| 8 | MAPKAPK2 |  |  | 2298 | 0.240 | 0.1929 | No |
| 9 | HADH2 |  |  | 2946 | 0.213 | 0.1887 | No |
| 10 | NR1H3 |  |  | 3084 | 0.207 | 0.2086 | No |
| 11 | C1ORF160 |  |  | 4606 | 0.164 | 0.1555 | No |
| 12 | PPCS |  |  | 5153 | 0.152 | 0.1484 | No |
| 13 | AKAP11 |  |  | 6478 | 0.127 | 0.1002 | No |
| 14 | DCT |  |  | 7514 | 0.109 | 0.0639 | No |
| 15 | BHMT |  |  | 8134 | 0.100 | 0.0465 | No |
| 16 | CFH |  |  | 8562 | 0.093 | 0.0377 | No |
| 17 | EGFR |  |  | 9161 | 0.085 | 0.0194 | No |
| 18 | CYP2F1 |  |  | 9490 | 0.080 | 0.0137 | No |
| 19 | MCM10 |  |  | 9898 | 0.075 | 0.0035 | No |
| 20 | ELOVL5 |  |  | 10255 | 0.070 | -0.0049 | No |
| 21 | PNLIPRP1 |  |  | 10370 | 0.068 | -0.0018 | No |
| 22 | PNLIP |  |  | 10568 | 0.065 | -0.0030 | No |
| 23 | NETO2 |  |  | 10650 | 0.064 | 0.0013 | No |
| 24 | HAL |  |  | 11017 | 0.059 | -0.0089 | No |
| 25 | ITPR1 |  |  | 11562 | 0.053 | -0.0286 | No |
| 26 | MDS025 |  |  | 13387 | 0.029 | -0.1137 | No |
| 27 | IL1RAP |  |  | 13465 | 0.028 | -0.1139 | No |
| 28 | C4BPA |  |  | 13674 | 0.025 | -0.1208 | No |
| 29 | REPS1 |  |  | 14242 | 0.017 | -0.1462 | No |
| 30 | AASS |  |  | 14250 | 0.017 | -0.1443 | No |
| 31 | ORM1 /// ORM2 |  |  | 14475 | 0.014 | -0.1534 | No |
| 32 | IGF1 |  |  | 14591 | 0.012 | -0.1575 | No |
| 33 | BAAT |  |  | 15134 | 0.004 | -0.1834 | No |
| 34 | REG1A |  |  | 15204 | 0.003 | -0.1864 | No |
| 35 | PCK1 |  |  | 15655 | -0.004 | -0.2077 | No |
| 36 | FABP1 |  |  | 15669 | -0.004 | -0.2078 | No |
| 37 | CTRC |  |  | 15729 | -0.006 | -0.2099 | No |
| 38 | HPD |  |  | 15810 | -0.007 | -0.2129 | No |
| 39 | RGN |  |  | 16243 | -0.014 | -0.2321 | No |
| 40 | ABCG2 |  |  | 16294 | -0.015 | -0.2326 | No |
| 41 | GHR |  |  | 16307 | -0.015 | -0.2312 | No |
| 42 | OTC |  |  | 16525 | -0.019 | -0.2393 | No |
| 43 | C14ORF140 |  |  | 16639 | -0.022 | -0.2420 | No |
| 44 | CYP2E1 |  |  | 17100 | -0.031 | -0.2604 | No |
| 45 | ELA2 |  |  | 17121 | -0.031 | -0.2574 | No |
| 46 | TDO2 |  |  | 17749 | -0.046 | -0.2820 | No |
| 47 | MOSC1 |  |  | 18100 | -0.054 | -0.2922 | No |
| 48 | HSD11B1 |  |  | 18550 | -0.067 | -0.3055 | No |
| 49 | HAO1 |  |  | 18643 | -0.070 | -0.3010 | No |
| 50 | ZG16 |  |  | 19013 | -0.085 | -0.3081 | No |
| 51 | RARRES1 |  |  | 19548 | -0.111 | -0.3198 | Yes |
| 52 | ME1 |  |  | 19559 | -0.112 | -0.3060 | Yes |
| 53 | GPR37 |  |  | 19676 | -0.118 | -0.2965 | Yes |
| 54 | AQP8 |  |  | 19951 | -0.140 | -0.2919 | Yes |
| 55 | LECT2 |  |  | 20068 | -0.151 | -0.2783 | Yes |
| 56 | GOT1 |  |  | 20079 | -0.152 | -0.2592 | Yes |
| 57 | ADIPOR2 |  |  | 20122 | -0.160 | -0.2408 | Yes |
| 58 | SERPINF1 |  |  | 20362 | -0.209 | -0.2256 | Yes |
| 59 | DBI |  |  | 20533 | -0.363 | -0.1874 | Yes |
| 60 | AKR1C1 |  |  | 20541 | -0.382 | -0.1388 | Yes |
| 61 | KLK5 |  |  | 20544 | -0.396 | -0.0882 | Yes |
| 62 | ACSL1 |  |  | 20590 | -0.711 | 0.0007 | Yes |
Table: GSEA details [plain text format]

  

Fig 2: LEE\_E2F1\_DN: Random ES distribution      
 Gene set null distribution of ES for **LEE\_E2F1\_DN**
