# Supplementary material for: The rapamycin-regulated gene expression signature determines prognosis for breast cancer
Source: Mol Cancer. 2009 Sep 24;8:75. doi: 10.1186/1476-4598-8-75 (PMC2761377; doi:10.1186/1476-4598-8-75)
Supplement: Additional file 3 — Gene set enrichment analysis of in vivo data, treatment series. The data provided represent the treatment series of GSEA. This compressed file contains "Treatment" shortcut file and "GSEA_treatment" folder. Clicking on "Treatment" shortcut opens the index file providing access to analysis files contained in the "GSEA_treatment" folder. [file 1476-4598-8-75-S3.zip › GSEA_treatment/LEE_TCELLS9_UP.html]

Details for gene set LEE\_TCELLS9\_UP[GSEA]

|  || Dataset | gsea\_treatment\_collapsed |
| Phenotype | NoPhenotypeAvailable |
| Upregulated in class | na\_pos |
| GeneSet | LEE\_TCELLS9\_UP |
| Enrichment Score (ES) | 0.63786083 |
| Normalized Enrichment Score (NES) | 1.7082914 |
| Nominal p-value | 0.0 |
| FDR q-value | 0.01081925 |
| FWER p-Value | 0.368 |
Table: GSEA Results Summary

  

Fig 1: Enrichment plot: LEE\_TCELLS9\_UP      
 Profile of the Running ES Score & Positions of GeneSet Members on the Rank Ordered List

  

| PROBE | GENE SYMBOL | GENE\_TITLE | RANK IN GENE LIST | RANK METRIC SCORE | RUNNING ES | CORE ENRICHMENT || 1 | C5ORF13 |  |  | 37 | 0.717 | 0.1072 | Yes |
| 2 | MARCKS |  |  | 101 | 0.575 | 0.1915 | Yes |
| 3 | LOC129607 |  |  | 263 | 0.463 | 0.2540 | Yes |
| 4 | CCNG2 |  |  | 485 | 0.408 | 0.3053 | Yes |
| 5 | PXDN |  |  | 631 | 0.378 | 0.3557 | Yes |
| 6 | FAM79B |  |  | 686 | 0.369 | 0.4092 | Yes |
| 7 | SOX4 |  |  | 1194 | 0.311 | 0.4318 | Yes |
| 8 | C20ORF100 |  |  | 1270 | 0.305 | 0.4745 | Yes |
| 9 | BCOR |  |  | 1309 | 0.302 | 0.5185 | Yes |
| 10 | LZTFL1 |  |  | 1548 | 0.283 | 0.5500 | Yes |
| 11 | MAML3 |  |  | 2537 | 0.228 | 0.5367 | Yes |
| 12 | TYMS |  |  | 2859 | 0.216 | 0.5539 | Yes |
| 13 | TIMP2 |  |  | 2941 | 0.213 | 0.5823 | Yes |
| 14 | DSG2 |  |  | 2974 | 0.212 | 0.6129 | Yes |
| 15 | RSAD2 |  |  | 3107 | 0.206 | 0.6379 | Yes |
| 16 | RP4-691N24.1 |  |  | 3940 | 0.181 | 0.6249 | No |
| 17 | TOP2A |  |  | 4403 | 0.169 | 0.6281 | No |
| 18 | SPRED2 |  |  | 6038 | 0.135 | 0.5692 | No |
| 19 | TOX |  |  | 7145 | 0.115 | 0.5329 | No |
| 20 | KIAA0101 |  |  | 7666 | 0.107 | 0.5240 | No |
| 21 | CHI3L2 |  |  | 8014 | 0.101 | 0.5225 | No |
| 22 | PDE4D |  |  | 9669 | 0.077 | 0.4539 | No |
| 23 | MICAL2 |  |  | 11264 | 0.056 | 0.3850 | No |
| 24 | P2RY1 |  |  | 14631 | 0.012 | 0.2231 | No |
| 25 | CCRL2 /// LOC642312 |  |  | 14666 | 0.011 | 0.2232 | No |
| 26 | CCR9 |  |  | 16023 | -0.011 | 0.1589 | No |
| 27 | EGR2 |  |  | 16050 | -0.011 | 0.1593 | No |
| 28 | NR4A2 |  |  | 16771 | -0.024 | 0.1279 | No |
| 29 | HTR2B |  |  | 18960 | -0.083 | 0.0342 | No |
| 30 | CD1B |  |  | 19917 | -0.138 | 0.0086 | No |
| 31 | SPRY2 |  |  | 20144 | -0.163 | 0.0224 | No |
Table: GSEA details [plain text format]

  

Fig 2: LEE\_TCELLS9\_UP: Random ES distribution      
 Gene set null distribution of ES for **LEE\_TCELLS9\_UP**
