# Supplementary material for: The rapamycin-regulated gene expression signature determines prognosis for breast cancer
Source: Mol Cancer. 2009 Sep 24;8:75. doi: 10.1186/1476-4598-8-75 (PMC2761377; doi:10.1186/1476-4598-8-75)
Supplement: Additional file 3 — Gene set enrichment analysis of in vivo data, treatment series. The data provided represent the treatment series of GSEA. This compressed file contains "Treatment" shortcut file and "GSEA_treatment" folder. Clicking on "Treatment" shortcut opens the index file providing access to analysis files contained in the "GSEA_treatment" folder. [file 1476-4598-8-75-S3.zip › GSEA_treatment/LIZUKA_L0_GR_L1.html]

Details for gene set LIZUKA\_L0\_GR\_L1[GSEA]

|  || Dataset | gsea\_treatment\_collapsed |
| Phenotype | NoPhenotypeAvailable |
| Upregulated in class | na\_neg |
| GeneSet | LIZUKA\_L0\_GR\_L1 |
| Enrichment Score (ES) | -0.40555286 |
| Normalized Enrichment Score (NES) | -1.3086631 |
| Nominal p-value | 0.14851485 |
| FDR q-value | 0.26934034 |
| FWER p-Value | 0.998 |
Table: GSEA Results Summary

  

Fig 1: Enrichment plot: LIZUKA\_L0\_GR\_L1      
 Profile of the Running ES Score & Positions of GeneSet Members on the Rank Ordered List

  

| PROBE | GENE SYMBOL | GENE\_TITLE | RANK IN GENE LIST | RANK METRIC SCORE | RUNNING ES | CORE ENRICHMENT || 1 | CNBP |  |  | 1780 | 0.267 | 0.0201 | No |
| 2 | ZFPL1 |  |  | 2499 | 0.230 | 0.0768 | No |
| 3 | PTPN18 |  |  | 3238 | 0.202 | 0.1214 | No |
| 4 | RMND5A |  |  | 3763 | 0.186 | 0.1701 | No |
| 5 | RNF103 |  |  | 5830 | 0.138 | 0.1247 | No |
| 6 | DMD |  |  | 7222 | 0.114 | 0.1026 | No |
| 7 | ZNF337 |  |  | 10555 | 0.065 | -0.0332 | No |
| 8 | PCBP3 |  |  | 11176 | 0.057 | -0.0405 | No |
| 9 | FN1 |  |  | 11951 | 0.047 | -0.0593 | No |
| 10 | APOB |  |  | 12743 | 0.037 | -0.0830 | No |
| 11 | SULT2B1 |  |  | 13736 | 0.024 | -0.1216 | No |
| 12 | TUBB2B |  |  | 16608 | -0.021 | -0.2526 | No |
| 13 | ZNF96 |  |  | 16626 | -0.022 | -0.2448 | No |
| 14 | C9ORF61 |  |  | 18900 | -0.081 | -0.3230 | Yes |
| 15 | S100A2 |  |  | 20601 | -1.019 | 0.0002 | Yes |
Table: GSEA details [plain text format]

  

Fig 2: LIZUKA\_L0\_GR\_L1: Random ES distribution      
 Gene set null distribution of ES for **LIZUKA\_L0\_GR\_L1**
