# Supplementary material for: The rapamycin-regulated gene expression signature determines prognosis for breast cancer
Source: Mol Cancer. 2009 Sep 24;8:75. doi: 10.1186/1476-4598-8-75 (PMC2761377; doi:10.1186/1476-4598-8-75)
Supplement: Additional file 3 — Gene set enrichment analysis of in vivo data, treatment series. The data provided represent the treatment series of GSEA. This compressed file contains "Treatment" shortcut file and "GSEA_treatment" folder. Clicking on "Treatment" shortcut opens the index file providing access to analysis files contained in the "GSEA_treatment" folder. [file 1476-4598-8-75-S3.zip › GSEA_treatment/MRNA_PROCESSING.html]

Details for gene set MRNA\_PROCESSING[GSEA]

|  || Dataset | gsea\_treatment\_collapsed |
| Phenotype | NoPhenotypeAvailable |
| Upregulated in class | na\_pos |
| GeneSet | MRNA\_PROCESSING |
| Enrichment Score (ES) | 0.65998596 |
| Normalized Enrichment Score (NES) | 1.8306223 |
| Nominal p-value | 0.0 |
| FDR q-value | 0.0015570913 |
| FWER p-Value | 0.033 |
Table: GSEA Results Summary

  

Fig 1: Enrichment plot: MRNA\_PROCESSING      
 Profile of the Running ES Score & Positions of GeneSet Members on the Rank Ordered List

  

| PROBE | GENE SYMBOL | GENE\_TITLE | RANK IN GENE LIST | RANK METRIC SCORE | RUNNING ES | CORE ENRICHMENT || 1 | SF3B4 |  |  | 312 | 0.448 | 0.0395 | Yes |
| 2 | NONO |  |  | 591 | 0.388 | 0.0733 | Yes |
| 3 | SFRS2IP |  |  | 776 | 0.356 | 0.1079 | Yes |
| 4 | SFRS1 |  |  | 923 | 0.339 | 0.1422 | Yes |
| 5 | CPSF6 |  |  | 941 | 0.337 | 0.1825 | Yes |
| 6 | U2AF2 |  |  | 1091 | 0.322 | 0.2146 | Yes |
| 7 | LSM5 |  |  | 1230 | 0.308 | 0.2455 | Yes |
| 8 | SFPQ |  |  | 1260 | 0.305 | 0.2813 | Yes |
| 9 | HNRPA0 |  |  | 1311 | 0.302 | 0.3157 | Yes |
| 10 | SF3A1 |  |  | 1513 | 0.286 | 0.3408 | Yes |
| 11 | KHSRP |  |  | 1514 | 0.286 | 0.3757 | Yes |
| 12 | SIP1 |  |  | 1665 | 0.274 | 0.4018 | Yes |
| 13 | PTBP1 |  |  | 1914 | 0.260 | 0.4215 | Yes |
| 14 | SFRS2 |  |  | 2044 | 0.253 | 0.4462 | Yes |
| 15 | HNRPA1 |  |  | 2259 | 0.242 | 0.4654 | Yes |
| 16 | SPOP |  |  | 2466 | 0.232 | 0.4837 | Yes |
| 17 | SF3A3 |  |  | 2579 | 0.227 | 0.5059 | Yes |
| 18 | SFRS7 |  |  | 3064 | 0.208 | 0.5078 | Yes |
| 19 | SFRS10 |  |  | 3150 | 0.205 | 0.5286 | Yes |
| 20 | KHDRBS1 |  |  | 3282 | 0.200 | 0.5467 | Yes |
| 21 | CUGBP1 |  |  | 3298 | 0.200 | 0.5704 | Yes |
| 22 | PRPF8 |  |  | 3514 | 0.193 | 0.5835 | Yes |
| 23 | SNRP70 |  |  | 3559 | 0.192 | 0.6048 | Yes |
| 24 | SFRS9 |  |  | 3633 | 0.190 | 0.6245 | Yes |
| 25 | SFRS11 |  |  | 4134 | 0.176 | 0.6216 | Yes |
| 26 | SNRPB2 |  |  | 4240 | 0.173 | 0.6376 | Yes |
| 27 | SLU7 |  |  | 4602 | 0.164 | 0.6402 | Yes |
| 28 | SF3B3 |  |  | 4607 | 0.164 | 0.6600 | Yes |
| 29 | SLBP |  |  | 6085 | 0.134 | 0.6045 | No |
| 30 | LSM3 |  |  | 6889 | 0.120 | 0.5801 | No |
| 31 | U2AF1 |  |  | 7733 | 0.106 | 0.5521 | No |
| 32 | PRPF18 |  |  | 7776 | 0.105 | 0.5629 | No |
| 33 | SFRS8 |  |  | 8899 | 0.088 | 0.5191 | No |
| 34 | SFRS5 |  |  | 8966 | 0.088 | 0.5266 | No |
| 35 | DHX15 |  |  | 9093 | 0.086 | 0.5309 | No |
| 36 | HNRPR |  |  | 10423 | 0.067 | 0.4745 | No |
| 37 | LSM1 |  |  | 11382 | 0.055 | 0.4346 | No |
| 38 | ELAVL4 |  |  | 13693 | 0.025 | 0.3253 | No |
| 39 | SFRS4 |  |  | 15330 | 0.000 | 0.2458 | No |
| 40 | SMNDC1 |  |  | 15503 | -0.002 | 0.2377 | No |
| 41 | USP39 |  |  | 17276 | -0.035 | 0.1557 | No |
| 42 | PRPF3 |  |  | 17944 | -0.050 | 0.1294 | No |
Table: GSEA details [plain text format]

  

Fig 2: MRNA\_PROCESSING: Random ES distribution      
 Gene set null distribution of ES for **MRNA\_PROCESSING**
