# Supplementary material for: The rapamycin-regulated gene expression signature determines prognosis for breast cancer
Source: Mol Cancer. 2009 Sep 24;8:75. doi: 10.1186/1476-4598-8-75 (PMC2761377; doi:10.1186/1476-4598-8-75)
Supplement: Additional file 3 — Gene set enrichment analysis of in vivo data, treatment series. The data provided represent the treatment series of GSEA. This compressed file contains "Treatment" shortcut file and "GSEA_treatment" folder. Clicking on "Treatment" shortcut opens the index file providing access to analysis files contained in the "GSEA_treatment" folder. [file 1476-4598-8-75-S3.zip › GSEA_treatment/MTA3PATHWAY.html]

Details for gene set MTA3PATHWAY[GSEA]

|  || Dataset | gsea\_treatment\_collapsed |
| Phenotype | NoPhenotypeAvailable |
| Upregulated in class | na\_neg |
| GeneSet | MTA3PATHWAY |
| Enrichment Score (ES) | -0.23090906 |
| Normalized Enrichment Score (NES) | -0.80094445 |
| Nominal p-value | 0.7307692 |
| FDR q-value | 0.8806453 |
| FWER p-Value | 1.0 |
Table: GSEA Results Summary

  

Fig 1: Enrichment plot: MTA3PATHWAY      
 Profile of the Running ES Score & Positions of GeneSet Members on the Rank Ordered List

  

| PROBE | GENE SYMBOL | GENE\_TITLE | RANK IN GENE LIST | RANK METRIC SCORE | RUNNING ES | CORE ENRICHMENT || 1 | TUBA3 |  |  | 897 | 0.342 | 0.1233 | Yes |
| 2 | CTSD |  |  | 1748 | 0.269 | 0.2134 | Yes |
| 3 | MTA3 |  |  | 3435 | 0.196 | 0.2270 | Yes |
| 4 | MTA1 |  |  | 5781 | 0.139 | 0.1809 | No |
| 5 | TUBA2 |  |  | 8674 | 0.091 | 0.0850 | No |
| 6 | HSPB2 |  |  | 8872 | 0.088 | 0.1186 | No |
| 7 | GREB1 |  |  | 8900 | 0.088 | 0.1603 | No |
| 8 | ESR1 |  |  | 8903 | 0.088 | 0.2031 | No |
| 9 | ALDOA |  |  | 9957 | 0.074 | 0.1879 | No |
| 10 | PDZK1 |  |  | 10344 | 0.068 | 0.2024 | No |
| 11 | TUBA6 |  |  | 11808 | 0.049 | 0.1553 | No |
| 12 | TUBA1 |  |  | 13598 | 0.026 | 0.0811 | No |
| 13 | TUBA4 |  |  | 13873 | 0.023 | 0.0788 | No |
| 14 | TUBA8 |  |  | 17141 | -0.031 | -0.0646 | No |
| 15 | HSPB1 |  |  | 20567 | -0.477 | 0.0018 | No |
Table: GSEA details [plain text format]

  

Fig 2: MTA3PATHWAY: Random ES distribution      
 Gene set null distribution of ES for **MTA3PATHWAY**
