# Supplementary material for: The rapamycin-regulated gene expression signature determines prognosis for breast cancer
Source: Mol Cancer. 2009 Sep 24;8:75. doi: 10.1186/1476-4598-8-75 (PMC2761377; doi:10.1186/1476-4598-8-75)
Supplement: Additional file 3 — Gene set enrichment analysis of in vivo data, treatment series. The data provided represent the treatment series of GSEA. This compressed file contains "Treatment" shortcut file and "GSEA_treatment" folder. Clicking on "Treatment" shortcut opens the index file providing access to analysis files contained in the "GSEA_treatment" folder. [file 1476-4598-8-75-S3.zip › GSEA_treatment/MYOD_BRG1_UP.html]

Details for gene set MYOD\_BRG1\_UP[GSEA]

|  || Dataset | gsea\_treatment\_collapsed |
| Phenotype | NoPhenotypeAvailable |
| Upregulated in class | na\_neg |
| GeneSet | MYOD\_BRG1\_UP |
| Enrichment Score (ES) | -0.33593625 |
| Normalized Enrichment Score (NES) | -1.2703727 |
| Nominal p-value | 0.10769231 |
| FDR q-value | 0.27085248 |
| FWER p-Value | 1.0 |
Table: GSEA Results Summary

  

Fig 1: Enrichment plot: MYOD\_BRG1\_UP      
 Profile of the Running ES Score & Positions of GeneSet Members on the Rank Ordered List

  

| PROBE | GENE SYMBOL | GENE\_TITLE | RANK IN GENE LIST | RANK METRIC SCORE | RUNNING ES | CORE ENRICHMENT || 1 | IGFBP5 |  |  | 26 | 0.757 | 0.1678 | No |
| 2 | CPSF6 |  |  | 941 | 0.337 | 0.1986 | No |
| 3 | PLEKHO1 |  |  | 1594 | 0.279 | 0.2293 | No |
| 4 | MYH3 |  |  | 3857 | 0.184 | 0.1604 | No |
| 5 | BIN1 |  |  | 5217 | 0.151 | 0.1280 | No |
| 6 | GYS1 |  |  | 6046 | 0.134 | 0.1177 | No |
| 7 | SCRIB |  |  | 7557 | 0.109 | 0.0686 | No |
| 8 | VKORC1 |  |  | 7568 | 0.108 | 0.0922 | No |
| 9 | MYL4 |  |  | 8971 | 0.087 | 0.0435 | No |
| 10 | ENO3 |  |  | 9051 | 0.086 | 0.0589 | No |
| 11 | KIFC3 |  |  | 9149 | 0.085 | 0.0731 | No |
| 12 | DYSF |  |  | 9205 | 0.084 | 0.0892 | No |
| 13 | CKM |  |  | 9592 | 0.078 | 0.0879 | No |
| 14 | ATP2A1 |  |  | 10251 | 0.070 | 0.0715 | No |
| 15 | PDLIM3 |  |  | 10444 | 0.067 | 0.0771 | No |
| 16 | HRC |  |  | 11475 | 0.054 | 0.0391 | No |
| 17 | TNNT2 |  |  | 13404 | 0.029 | -0.0482 | No |
| 18 | SCHIP1 |  |  | 15349 | 0.000 | -0.1425 | No |
| 19 | TNNT3 |  |  | 16723 | -0.024 | -0.2040 | No |
| 20 | MYOG |  |  | 17462 | -0.039 | -0.2311 | No |
| 21 | PPFIA4 |  |  | 17673 | -0.044 | -0.2316 | No |
| 22 | MYBPH |  |  | 18380 | -0.062 | -0.2520 | No |
| 23 | TNNI2 |  |  | 18920 | -0.082 | -0.2600 | No |
| 24 | MYL1 |  |  | 20484 | -0.284 | -0.2726 | Yes |
| 25 | MYLPF |  |  | 20543 | -0.395 | -0.1872 | Yes |
| 26 | TNNC2 |  |  | 20554 | -0.425 | -0.0929 | Yes |
| 27 | ACTA1 |  |  | 20555 | -0.427 | 0.0024 | Yes |
Table: GSEA details [plain text format]

  

Fig 2: MYOD\_BRG1\_UP: Random ES distribution      
 Gene set null distribution of ES for **MYOD\_BRG1\_UP**
