# Supplementary material for: The rapamycin-regulated gene expression signature determines prognosis for breast cancer
Source: Mol Cancer. 2009 Sep 24;8:75. doi: 10.1186/1476-4598-8-75 (PMC2761377; doi:10.1186/1476-4598-8-75)
Supplement: Additional file 3 — Gene set enrichment analysis of in vivo data, treatment series. The data provided represent the treatment series of GSEA. This compressed file contains "Treatment" shortcut file and "GSEA_treatment" folder. Clicking on "Treatment" shortcut opens the index file providing access to analysis files contained in the "GSEA_treatment" folder. [file 1476-4598-8-75-S3.zip › GSEA_treatment/NADLER_OBESITY_DN.html]

Details for gene set NADLER\_OBESITY\_DN[GSEA]

|  || Dataset | gsea\_treatment\_collapsed |
| Phenotype | NoPhenotypeAvailable |
| Upregulated in class | na\_neg |
| GeneSet | NADLER\_OBESITY\_DN |
| Enrichment Score (ES) | -0.3328053 |
| Normalized Enrichment Score (NES) | -1.405237 |
| Nominal p-value | 0.033333335 |
| FDR q-value | 0.21710946 |
| FWER p-Value | 0.964 |
Table: GSEA Results Summary

  

Fig 1: Enrichment plot: NADLER\_OBESITY\_DN      
 Profile of the Running ES Score & Positions of GeneSet Members on the Rank Ordered List

  

| PROBE | GENE SYMBOL | GENE\_TITLE | RANK IN GENE LIST | RANK METRIC SCORE | RUNNING ES | CORE ENRICHMENT || 1 | EEF1A1 |  |  | 318 | 0.446 | 0.0691 | No |
| 2 | DDT |  |  | 973 | 0.334 | 0.1007 | No |
| 3 | UCK1 |  |  | 1619 | 0.278 | 0.1220 | No |
| 4 | B2M |  |  | 2291 | 0.240 | 0.1351 | No |
| 5 | PYGB |  |  | 3167 | 0.204 | 0.1313 | No |
| 6 | CFD |  |  | 3315 | 0.200 | 0.1620 | No |
| 7 | APOE |  |  | 4275 | 0.172 | 0.1481 | No |
| 8 | BCAT2 |  |  | 4420 | 0.168 | 0.1730 | No |
| 9 | GPD2 |  |  | 5032 | 0.155 | 0.1727 | No |
| 10 | RBMS2 |  |  | 7013 | 0.118 | 0.0988 | No |
| 11 | COX8A |  |  | 8948 | 0.088 | 0.0214 | No |
| 12 | PPA1 |  |  | 9532 | 0.079 | 0.0081 | No |
| 13 | SREBF1 |  |  | 9596 | 0.078 | 0.0199 | No |
| 14 | ALDOA |  |  | 9957 | 0.074 | 0.0163 | No |
| 15 | ASNS |  |  | 11946 | 0.048 | -0.0713 | No |
| 16 | TSHR |  |  | 12010 | 0.047 | -0.0655 | No |
| 17 | HP |  |  | 12079 | 0.046 | -0.0601 | No |
| 18 | C2 |  |  | 12568 | 0.039 | -0.0764 | No |
| 19 | SOD1 |  |  | 12888 | 0.035 | -0.0852 | No |
| 20 | RBP4 |  |  | 13125 | 0.032 | -0.0906 | No |
| 21 | PCK1 |  |  | 15655 | -0.004 | -0.2127 | No |
| 22 | CDKN2C |  |  | 15692 | -0.005 | -0.2135 | No |
| 23 | UBB |  |  | 16027 | -0.011 | -0.2278 | No |
| 24 | ADRB3 |  |  | 16468 | -0.018 | -0.2457 | No |
| 25 | FMO1 |  |  | 16947 | -0.027 | -0.2637 | No |
| 26 | FDFT1 |  |  | 17340 | -0.036 | -0.2759 | No |
| 27 | AGT |  |  | 17814 | -0.047 | -0.2900 | No |
| 28 | NNAT |  |  | 18283 | -0.059 | -0.3016 | No |
| 29 | RASD1 |  |  | 18926 | -0.082 | -0.3173 | Yes |
| 30 | FABP4 |  |  | 19098 | -0.089 | -0.3087 | Yes |
| 31 | THRSP |  |  | 19377 | -0.102 | -0.3028 | Yes |
| 32 | GSTA3 |  |  | 19574 | -0.112 | -0.2911 | Yes |
| 33 | GBE1 |  |  | 20409 | -0.230 | -0.2880 | Yes |
| 34 | ALDH2 |  |  | 20530 | -0.348 | -0.2278 | Yes |
| 35 | DBI |  |  | 20533 | -0.363 | -0.1590 | Yes |
| 36 | LDHB |  |  | 20594 | -0.856 | 0.0005 | Yes |
Table: GSEA details [plain text format]

  

Fig 2: NADLER\_OBESITY\_DN: Random ES distribution      
 Gene set null distribution of ES for **NADLER\_OBESITY\_DN**
