# Supplementary material for: The rapamycin-regulated gene expression signature determines prognosis for breast cancer
Source: Mol Cancer. 2009 Sep 24;8:75. doi: 10.1186/1476-4598-8-75 (PMC2761377; doi:10.1186/1476-4598-8-75)
Supplement: Additional file 3 — Gene set enrichment analysis of in vivo data, treatment series. The data provided represent the treatment series of GSEA. This compressed file contains "Treatment" shortcut file and "GSEA_treatment" folder. Clicking on "Treatment" shortcut opens the index file providing access to analysis files contained in the "GSEA_treatment" folder. [file 1476-4598-8-75-S3.zip › GSEA_treatment/NAKAJIMA_MCSMBP_EOS.html]

Details for gene set NAKAJIMA\_MCSMBP\_EOS[GSEA]

|  || Dataset | gsea\_treatment\_collapsed |
| Phenotype | NoPhenotypeAvailable |
| Upregulated in class | na\_neg |
| GeneSet | NAKAJIMA\_MCSMBP\_EOS |
| Enrichment Score (ES) | -0.34451312 |
| Normalized Enrichment Score (NES) | -1.3793715 |
| Nominal p-value | 0.06122449 |
| FDR q-value | 0.21307528 |
| FWER p-Value | 0.981 |
Table: GSEA Results Summary

  

Fig 1: Enrichment plot: NAKAJIMA\_MCSMBP\_EOS      
 Profile of the Running ES Score & Positions of GeneSet Members on the Rank Ordered List

  

| PROBE | GENE SYMBOL | GENE\_TITLE | RANK IN GENE LIST | RANK METRIC SCORE | RUNNING ES | CORE ENRICHMENT || 1 | MARCKSL1 |  |  | 413 | 0.423 | 0.1051 | No |
| 2 | SLC29A1 |  |  | 2801 | 0.218 | 0.0537 | No |
| 3 | CFD |  |  | 3315 | 0.200 | 0.0879 | No |
| 4 | INDO |  |  | 5656 | 0.141 | 0.0160 | No |
| 5 | CREM |  |  | 6167 | 0.132 | 0.0304 | No |
| 6 | OLR1 |  |  | 6307 | 0.130 | 0.0621 | No |
| 7 | IL5RA |  |  | 7239 | 0.114 | 0.0506 | No |
| 8 | GPR44 |  |  | 7333 | 0.113 | 0.0794 | No |
| 9 | PLAUR |  |  | 9123 | 0.085 | 0.0176 | No |
| 10 | TFF3 |  |  | 10102 | 0.072 | -0.0087 | No |
| 11 | CAMK1 |  |  | 11334 | 0.055 | -0.0521 | No |
| 12 | OLIG2 |  |  | 12018 | 0.047 | -0.0715 | No |
| 13 | SYNE1 |  |  | 12827 | 0.036 | -0.1001 | No |
| 14 | ADORA3 |  |  | 13837 | 0.023 | -0.1424 | No |
| 15 | CCR3 |  |  | 14770 | 0.010 | -0.1849 | No |
| 16 | P2RY10 |  |  | 14796 | 0.009 | -0.1834 | No |
| 17 | CLC |  |  | 15578 | -0.003 | -0.2203 | No |
| 18 | EMR1 |  |  | 15745 | -0.006 | -0.2267 | No |
| 19 | CCL4 |  |  | 16197 | -0.013 | -0.2446 | No |
| 20 | FBP1 |  |  | 16603 | -0.021 | -0.2580 | No |
| 21 | HES1 |  |  | 16915 | -0.026 | -0.2653 | No |
| 22 | P2RY14 |  |  | 18522 | -0.066 | -0.3239 | Yes |
| 23 | RNASE2 |  |  | 18634 | -0.070 | -0.3086 | Yes |
| 24 | ALOX15 |  |  | 19374 | -0.102 | -0.3142 | Yes |
| 25 | CNR2 |  |  | 19427 | -0.104 | -0.2858 | Yes |
| 26 | SMAD7 |  |  | 20463 | -0.262 | -0.2587 | Yes |
| 27 | S100P |  |  | 20599 | -0.897 | 0.0003 | Yes |
Table: GSEA details [plain text format]

  

Fig 2: NAKAJIMA\_MCSMBP\_EOS: Random ES distribution      
 Gene set null distribution of ES for **NAKAJIMA\_MCSMBP\_EOS**
