# Supplementary material for: The rapamycin-regulated gene expression signature determines prognosis for breast cancer
Source: Mol Cancer. 2009 Sep 24;8:75. doi: 10.1186/1476-4598-8-75 (PMC2761377; doi:10.1186/1476-4598-8-75)
Supplement: Additional file 3 — Gene set enrichment analysis of in vivo data, treatment series. The data provided represent the treatment series of GSEA. This compressed file contains "Treatment" shortcut file and "GSEA_treatment" folder. Clicking on "Treatment" shortcut opens the index file providing access to analysis files contained in the "GSEA_treatment" folder. [file 1476-4598-8-75-S3.zip › GSEA_treatment/NEMETH_TNF_DN.html]

Details for gene set NEMETH\_TNF\_DN[GSEA]

|  || Dataset | gsea\_treatment\_collapsed |
| Phenotype | NoPhenotypeAvailable |
| Upregulated in class | na\_neg |
| GeneSet | NEMETH\_TNF\_DN |
| Enrichment Score (ES) | -0.3020747 |
| Normalized Enrichment Score (NES) | -1.2275817 |
| Nominal p-value | 0.12244898 |
| FDR q-value | 0.27804378 |
| FWER p-Value | 1.0 |
Table: GSEA Results Summary

  

Fig 1: Enrichment plot: NEMETH\_TNF\_DN      
 Profile of the Running ES Score & Positions of GeneSet Members on the Rank Ordered List

  

| PROBE | GENE SYMBOL | GENE\_TITLE | RANK IN GENE LIST | RANK METRIC SCORE | RUNNING ES | CORE ENRICHMENT || 1 | HELLS |  |  | 1078 | 0.323 | 0.0138 | No |
| 2 | APEX1 |  |  | 1111 | 0.321 | 0.0780 | No |
| 3 | GTF2I |  |  | 1536 | 0.284 | 0.1157 | No |
| 4 | ZFP36L1 |  |  | 1617 | 0.278 | 0.1688 | No |
| 5 | NUMB |  |  | 2128 | 0.250 | 0.1951 | No |
| 6 | ORC5L |  |  | 4096 | 0.176 | 0.1357 | No |
| 7 | CCNF |  |  | 4552 | 0.165 | 0.1475 | No |
| 8 | ACADM |  |  | 4730 | 0.161 | 0.1719 | No |
| 9 | ABCG1 |  |  | 5424 | 0.146 | 0.1682 | No |
| 10 | RBM38 |  |  | 5516 | 0.144 | 0.1933 | No |
| 11 | SLBP |  |  | 6085 | 0.134 | 0.1931 | No |
| 12 | MXI1 |  |  | 6948 | 0.119 | 0.1756 | No |
| 13 | FEN1 |  |  | 8272 | 0.098 | 0.1313 | No |
| 14 | APOBEC1 |  |  | 9374 | 0.082 | 0.0945 | No |
| 15 | MBP |  |  | 9412 | 0.081 | 0.1094 | No |
| 16 | CHEK1 |  |  | 9905 | 0.075 | 0.1008 | No |
| 17 | ITGA4 |  |  | 11022 | 0.059 | 0.0586 | No |
| 18 | ST8SIA4 |  |  | 11704 | 0.050 | 0.0359 | No |
| 19 | HIST1H2AB |  |  | 12539 | 0.040 | 0.0035 | No |
| 20 | LMO4 |  |  | 13678 | 0.025 | -0.0466 | No |
| 21 | LYL1 |  |  | 14844 | 0.009 | -0.1015 | No |
| 22 | AURKA |  |  | 15848 | -0.008 | -0.1487 | No |
| 23 | MKI67 |  |  | 17689 | -0.044 | -0.2290 | No |
| 24 | ZMAT3 |  |  | 18659 | -0.071 | -0.2615 | No |
| 25 | RASSF5 |  |  | 18799 | -0.076 | -0.2526 | No |
| 26 | SLC6A12 |  |  | 19736 | -0.122 | -0.2731 | Yes |
| 27 | FOS |  |  | 20334 | -0.202 | -0.2606 | Yes |
| 28 | HSPA4L |  |  | 20473 | -0.269 | -0.2121 | Yes |
| 29 | SCD |  |  | 20496 | -0.294 | -0.1528 | Yes |
| 30 | RGS2 |  |  | 20592 | -0.771 | 0.0006 | Yes |
Table: GSEA details [plain text format]

  

Fig 2: NEMETH\_TNF\_DN: Random ES distribution      
 Gene set null distribution of ES for **NEMETH\_TNF\_DN**
