# Supplementary material for: The rapamycin-regulated gene expression signature determines prognosis for breast cancer
Source: Mol Cancer. 2009 Sep 24;8:75. doi: 10.1186/1476-4598-8-75 (PMC2761377; doi:10.1186/1476-4598-8-75)
Supplement: Additional file 3 — Gene set enrichment analysis of in vivo data, treatment series. The data provided represent the treatment series of GSEA. This compressed file contains "Treatment" shortcut file and "GSEA_treatment" folder. Clicking on "Treatment" shortcut opens the index file providing access to analysis files contained in the "GSEA_treatment" folder. [file 1476-4598-8-75-S3.zip › GSEA_treatment/NF90_UP.html]

Details for gene set NF90\_UP[GSEA]

|  || Dataset | gsea\_treatment\_collapsed |
| Phenotype | NoPhenotypeAvailable |
| Upregulated in class | na\_pos |
| GeneSet | NF90\_UP |
| Enrichment Score (ES) | 0.7484567 |
| Normalized Enrichment Score (NES) | 1.9458092 |
| Nominal p-value | 0.0 |
| FDR q-value | 4.9358385E-4 |
| FWER p-Value | 0.0050 |
Table: GSEA Results Summary

  

Fig 1: Enrichment plot: NF90\_UP      
 Profile of the Running ES Score & Positions of GeneSet Members on the Rank Ordered List

  

| PROBE | GENE SYMBOL | GENE\_TITLE | RANK IN GENE LIST | RANK METRIC SCORE | RUNNING ES | CORE ENRICHMENT || 1 | NT5E |  |  | 2 | 1.142 | 0.1335 | Yes |
| 2 | TRIM22 |  |  | 8 | 1.024 | 0.2530 | Yes |
| 3 | MX1 |  |  | 24 | 0.773 | 0.3427 | Yes |
| 4 | IFI27 |  |  | 32 | 0.725 | 0.4272 | Yes |
| 5 | ISG15 |  |  | 59 | 0.644 | 0.5014 | Yes |
| 6 | IFITM1 |  |  | 79 | 0.609 | 0.5717 | Yes |
| 7 | HLA-E |  |  | 126 | 0.545 | 0.6332 | Yes |
| 8 | OAS1 |  |  | 255 | 0.466 | 0.6816 | Yes |
| 9 | CTSO |  |  | 812 | 0.352 | 0.6958 | Yes |
| 10 | TUBB |  |  | 1267 | 0.305 | 0.7095 | Yes |
| 11 | STAT1 |  |  | 1401 | 0.294 | 0.7374 | Yes |
| 12 | IFIT3 |  |  | 1814 | 0.265 | 0.7485 | Yes |
| 13 | APOE |  |  | 4275 | 0.172 | 0.6491 | No |
| 14 | BLZF1 |  |  | 4477 | 0.167 | 0.6588 | No |
| 15 | TSPAN8 |  |  | 5269 | 0.150 | 0.6379 | No |
| 16 | TBX5 |  |  | 6721 | 0.123 | 0.5818 | No |
| 17 | BIRC4BP |  |  | 7047 | 0.117 | 0.5797 | No |
| 18 | CLEC2B |  |  | 8592 | 0.093 | 0.5156 | No |
| 19 | BDKRB2 |  |  | 14390 | 0.015 | 0.2356 | No |
| 20 | HIST1H2AC |  |  | 16391 | -0.017 | 0.1404 | No |
| 21 | RGS4 |  |  | 17924 | -0.050 | 0.0718 | No |
| 22 | FAP |  |  | 19333 | -0.100 | 0.0151 | No |
| 23 | EMP1 |  |  | 19407 | -0.103 | 0.0237 | No |
| 24 | MX2 |  |  | 19725 | -0.121 | 0.0225 | No |
| 25 | PROS1 |  |  | 20202 | -0.173 | 0.0196 | No |
Table: GSEA details [plain text format]

  

Fig 2: NF90\_UP: Random ES distribution      
 Gene set null distribution of ES for **NF90\_UP**
