# Supplementary material for: The rapamycin-regulated gene expression signature determines prognosis for breast cancer
Source: Mol Cancer. 2009 Sep 24;8:75. doi: 10.1186/1476-4598-8-75 (PMC2761377; doi:10.1186/1476-4598-8-75)
Supplement: Additional file 3 — Gene set enrichment analysis of in vivo data, treatment series. The data provided represent the treatment series of GSEA. This compressed file contains "Treatment" shortcut file and "GSEA_treatment" folder. Clicking on "Treatment" shortcut opens the index file providing access to analysis files contained in the "GSEA_treatment" folder. [file 1476-4598-8-75-S3.zip › GSEA_treatment/NI2_MOUSE_UP.html]

Details for gene set NI2\_MOUSE\_UP[GSEA]

|  || Dataset | gsea\_treatment\_collapsed |
| Phenotype | NoPhenotypeAvailable |
| Upregulated in class | na\_pos |
| GeneSet | NI2\_MOUSE\_UP |
| Enrichment Score (ES) | 0.6143024 |
| Normalized Enrichment Score (NES) | 1.6825601 |
| Nominal p-value | 0.0 |
| FDR q-value | 0.014689801 |
| FWER p-Value | 0.529 |
Table: GSEA Results Summary

  

Fig 1: Enrichment plot: NI2\_MOUSE\_UP      
 Profile of the Running ES Score & Positions of GeneSet Members on the Rank Ordered List

  

| PROBE | GENE SYMBOL | GENE\_TITLE | RANK IN GENE LIST | RANK METRIC SCORE | RUNNING ES | CORE ENRICHMENT || 1 | RBM3 |  |  | 4 | 1.110 | 0.1357 | Yes |
| 2 | S100A8 |  |  | 42 | 0.695 | 0.2189 | Yes |
| 3 | MYC |  |  | 75 | 0.615 | 0.2925 | Yes |
| 4 | LCN2 |  |  | 262 | 0.463 | 0.3401 | Yes |
| 5 | AKAP12 |  |  | 365 | 0.435 | 0.3883 | Yes |
| 6 | LITAF |  |  | 538 | 0.396 | 0.4284 | Yes |
| 7 | TXNRD1 |  |  | 584 | 0.390 | 0.4739 | Yes |
| 8 | OSMR |  |  | 640 | 0.378 | 0.5174 | Yes |
| 9 | PTGES |  |  | 711 | 0.366 | 0.5588 | Yes |
| 10 | TGFBI |  |  | 811 | 0.352 | 0.5972 | Yes |
| 11 | CYR61 |  |  | 1299 | 0.302 | 0.6105 | Yes |
| 12 | MCL1 |  |  | 1881 | 0.262 | 0.6143 | Yes |
| 13 | TNC |  |  | 3191 | 0.203 | 0.5755 | No |
| 14 | TPM1 |  |  | 3849 | 0.184 | 0.5661 | No |
| 15 | LRG1 |  |  | 5389 | 0.147 | 0.5093 | No |
| 16 | NDUFS1 |  |  | 6064 | 0.134 | 0.4929 | No |
| 17 | ANGPTL4 |  |  | 6598 | 0.125 | 0.4823 | No |
| 18 | NFKBIA |  |  | 7714 | 0.106 | 0.4411 | No |
| 19 | NR4A1 |  |  | 7746 | 0.106 | 0.4525 | No |
| 20 | KLHL2 |  |  | 9076 | 0.086 | 0.3984 | No |
| 21 | ELN |  |  | 10887 | 0.061 | 0.3178 | No |
| 22 | EIF1AY |  |  | 11242 | 0.057 | 0.3076 | No |
| 23 | SPP1 |  |  | 11797 | 0.049 | 0.2866 | No |
| 24 | SPHK1 |  |  | 12096 | 0.046 | 0.2777 | No |
| 25 | RETNLB |  |  | 12481 | 0.041 | 0.2641 | No |
| 26 | MAL |  |  | 12889 | 0.035 | 0.2486 | No |
| 27 | CSTB |  |  | 13690 | 0.025 | 0.2127 | No |
| 28 | PLA2G7 |  |  | 14342 | 0.016 | 0.1830 | No |
| 29 | STEAP4 |  |  | 14711 | 0.011 | 0.1664 | No |
| 30 | PTX3 |  |  | 14933 | 0.007 | 0.1566 | No |
| 31 | BTG2 |  |  | 16013 | -0.011 | 0.1054 | No |
| 32 | CDKN1A |  |  | 16349 | -0.016 | 0.0911 | No |
| 33 | ZFP36 |  |  | 18077 | -0.054 | 0.0137 | No |
| 34 | DUSP1 |  |  | 18808 | -0.076 | -0.0124 | No |
| 35 | LOX |  |  | 18954 | -0.083 | -0.0094 | No |
| 36 | COL3A1 |  |  | 19040 | -0.086 | -0.0030 | No |
| 37 | SERPIND1 |  |  | 19547 | -0.111 | -0.0140 | No |
| 38 | KLF6 |  |  | 19691 | -0.119 | -0.0064 | No |
| 39 | SFN |  |  | 20552 | -0.415 | 0.0026 | No |
Table: GSEA details [plain text format]

  

Fig 2: NI2\_MOUSE\_UP: Random ES distribution      
 Gene set null distribution of ES for **NI2\_MOUSE\_UP**
