# Supplementary material for: The rapamycin-regulated gene expression signature determines prognosis for breast cancer
Source: Mol Cancer. 2009 Sep 24;8:75. doi: 10.1186/1476-4598-8-75 (PMC2761377; doi:10.1186/1476-4598-8-75)
Supplement: Additional file 3 — Gene set enrichment analysis of in vivo data, treatment series. The data provided represent the treatment series of GSEA. This compressed file contains "Treatment" shortcut file and "GSEA_treatment" folder. Clicking on "Treatment" shortcut opens the index file providing access to analysis files contained in the "GSEA_treatment" folder. [file 1476-4598-8-75-S3.zip › GSEA_treatment/NO1PATHWAY.html]

Details for gene set NO1PATHWAY[GSEA]

|  || Dataset | gsea\_treatment\_collapsed |
| Phenotype | NoPhenotypeAvailable |
| Upregulated in class | na\_neg |
| GeneSet | NO1PATHWAY |
| Enrichment Score (ES) | -0.35796675 |
| Normalized Enrichment Score (NES) | -1.4508518 |
| Nominal p-value | 0.041666668 |
| FDR q-value | 0.20268998 |
| FWER p-Value | 0.928 |
Table: GSEA Results Summary

  

Fig 1: Enrichment plot: NO1PATHWAY      
 Profile of the Running ES Score & Positions of GeneSet Members on the Rank Ordered List

  

| PROBE | GENE SYMBOL | GENE\_TITLE | RANK IN GENE LIST | RANK METRIC SCORE | RUNNING ES | CORE ENRICHMENT || 1 | NOS3 |  |  | 3079 | 0.207 | -0.0888 | No |
| 2 | PRKAR1A |  |  | 3195 | 0.203 | -0.0348 | No |
| 3 | CHRM1 |  |  | 4369 | 0.170 | -0.0418 | No |
| 4 | PDE3B |  |  | 4393 | 0.169 | 0.0068 | No |
| 5 | KDR |  |  | 5172 | 0.151 | 0.0135 | No |
| 6 | CALM3 |  |  | 5529 | 0.144 | 0.0384 | No |
| 7 | AKT1 |  |  | 6236 | 0.131 | 0.0426 | No |
| 8 | PRKAR2A |  |  | 6428 | 0.128 | 0.0709 | No |
| 9 | FLT4 |  |  | 6733 | 0.123 | 0.0921 | No |
| 10 | PRKACB |  |  | 6794 | 0.122 | 0.1249 | No |
| 11 | SLC7A1 |  |  | 8382 | 0.096 | 0.0759 | No |
| 12 | PDE3A |  |  | 9858 | 0.076 | 0.0264 | No |
| 13 | CALM2 |  |  | 11920 | 0.048 | -0.0598 | No |
| 14 | FLT1 |  |  | 12509 | 0.040 | -0.0765 | No |
| 15 | CALM1 |  |  | 13965 | 0.021 | -0.1410 | No |
| 16 | CHRNA1 |  |  | 13988 | 0.021 | -0.1360 | No |
| 17 | BDKRB2 |  |  | 14390 | 0.015 | -0.1511 | No |
| 18 | RYR2 |  |  | 14505 | 0.013 | -0.1528 | No |
| 19 | PRKG1 |  |  | 14960 | 0.007 | -0.1729 | No |
| 20 | SYT1 |  |  | 15423 | -0.001 | -0.1951 | No |
| 21 | PRKACG |  |  | 16957 | -0.027 | -0.2615 | No |
| 22 | PDE2A |  |  | 17685 | -0.044 | -0.2839 | No |
| 23 | TNNI1 |  |  | 18043 | -0.053 | -0.2857 | No |
| 24 | PRKG2 |  |  | 18511 | -0.066 | -0.2890 | No |
| 25 | PRKAR1B |  |  | 18689 | -0.072 | -0.2765 | No |
| 26 | VEGF |  |  | 20367 | -0.211 | -0.2960 | Yes |
| 27 | PRKAR2B |  |  | 20498 | -0.300 | -0.2142 | Yes |
| 28 | CAV1 |  |  | 20514 | -0.320 | -0.1210 | Yes |
| 29 | ACTA1 |  |  | 20555 | -0.427 | 0.0024 | Yes |
Table: GSEA details [plain text format]

  

Fig 2: NO1PATHWAY: Random ES distribution      
 Gene set null distribution of ES for **NO1PATHWAY**
