# Supplementary material for: The rapamycin-regulated gene expression signature determines prognosis for breast cancer
Source: Mol Cancer. 2009 Sep 24;8:75. doi: 10.1186/1476-4598-8-75 (PMC2761377; doi:10.1186/1476-4598-8-75)
Supplement: Additional file 3 — Gene set enrichment analysis of in vivo data, treatment series. The data provided represent the treatment series of GSEA. This compressed file contains "Treatment" shortcut file and "GSEA_treatment" folder. Clicking on "Treatment" shortcut opens the index file providing access to analysis files contained in the "GSEA_treatment" folder. [file 1476-4598-8-75-S3.zip › GSEA_treatment/OXSTRESS_RPETHREE_DN.html]

Details for gene set OXSTRESS\_RPETHREE\_DN[GSEA]

|  || Dataset | gsea\_treatment\_collapsed |
| Phenotype | NoPhenotypeAvailable |
| Upregulated in class | na\_pos |
| GeneSet | OXSTRESS\_RPETHREE\_DN |
| Enrichment Score (ES) | 0.7291468 |
| Normalized Enrichment Score (NES) | 1.9270035 |
| Nominal p-value | 0.0 |
| FDR q-value | 4.5591948E-4 |
| FWER p-Value | 0.0060 |
Table: GSEA Results Summary

  

Fig 1: Enrichment plot: OXSTRESS\_RPETHREE\_DN      
 Profile of the Running ES Score & Positions of GeneSet Members on the Rank Ordered List

  

| PROBE | GENE SYMBOL | GENE\_TITLE | RANK IN GENE LIST | RANK METRIC SCORE | RUNNING ES | CORE ENRICHMENT || 1 | ITGB6 |  |  | 63 | 0.640 | 0.1064 | Yes |
| 2 | PIK3R2 |  |  | 199 | 0.492 | 0.1839 | Yes |
| 3 | CAPNS1 |  |  | 372 | 0.434 | 0.2498 | Yes |
| 4 | IGFBP2 |  |  | 575 | 0.390 | 0.3067 | Yes |
| 5 | NFIX |  |  | 605 | 0.384 | 0.3709 | Yes |
| 6 | C6ORF108 |  |  | 648 | 0.376 | 0.4332 | Yes |
| 7 | ARHGDIA |  |  | 763 | 0.358 | 0.4889 | Yes |
| 8 | MCM4 |  |  | 1070 | 0.324 | 0.5295 | Yes |
| 9 | HMGA1 |  |  | 1086 | 0.323 | 0.5839 | Yes |
| 10 | CUTL1 |  |  | 1171 | 0.314 | 0.6334 | Yes |
| 11 | JUND |  |  | 1372 | 0.296 | 0.6744 | Yes |
| 12 | GNAS |  |  | 1773 | 0.268 | 0.7007 | Yes |
| 13 | NF2 |  |  | 2740 | 0.221 | 0.6915 | Yes |
| 14 | POLD1 |  |  | 3035 | 0.209 | 0.7130 | Yes |
| 15 | BNIP3 |  |  | 3398 | 0.197 | 0.7291 | Yes |
| 16 | ITGA5 |  |  | 7140 | 0.115 | 0.5671 | No |
| 17 | COMT |  |  | 9599 | 0.078 | 0.4610 | No |
| 18 | ID1 |  |  | 10922 | 0.061 | 0.4072 | No |
| 19 | CASK |  |  | 10953 | 0.060 | 0.4160 | No |
| 20 | AMPH |  |  | 11470 | 0.054 | 0.4001 | No |
| 21 | LCAT |  |  | 11716 | 0.050 | 0.3968 | No |
| 22 | NFKB1 |  |  | 12483 | 0.041 | 0.3666 | No |
| 23 | RBL2 |  |  | 12582 | 0.039 | 0.3686 | No |
| 24 | ZNF91 |  |  | 13008 | 0.034 | 0.3537 | No |
| 25 | DAPK1 |  |  | 14673 | 0.011 | 0.2747 | No |
| 26 | CDH3 |  |  | 15025 | 0.006 | 0.2586 | No |
| 27 | ADORA1 |  |  | 17334 | -0.036 | 0.1527 | No |
| 28 | CCNA2 |  |  | 17367 | -0.037 | 0.1574 | No |
Table: GSEA details [plain text format]

  

Fig 2: OXSTRESS\_RPETHREE\_DN: Random ES distribution      
 Gene set null distribution of ES for **OXSTRESS\_RPETHREE\_DN**
