# Supplementary material for: The rapamycin-regulated gene expression signature determines prognosis for breast cancer
Source: Mol Cancer. 2009 Sep 24;8:75. doi: 10.1186/1476-4598-8-75 (PMC2761377; doi:10.1186/1476-4598-8-75)
Supplement: Additional file 3 — Gene set enrichment analysis of in vivo data, treatment series. The data provided represent the treatment series of GSEA. This compressed file contains "Treatment" shortcut file and "GSEA_treatment" folder. Clicking on "Treatment" shortcut opens the index file providing access to analysis files contained in the "GSEA_treatment" folder. [file 1476-4598-8-75-S3.zip › GSEA_treatment/P53GENES_ALL.html]

Details for gene set P53GENES\_ALL[GSEA]

|  || Dataset | gsea\_treatment\_collapsed |
| Phenotype | NoPhenotypeAvailable |
| Upregulated in class | na\_neg |
| GeneSet | P53GENES\_ALL |
| Enrichment Score (ES) | -0.50912374 |
| Normalized Enrichment Score (NES) | -1.7378845 |
| Nominal p-value | 0.010526316 |
| FDR q-value | 0.07101882 |
| FWER p-Value | 0.405 |
Table: GSEA Results Summary

  

Fig 1: Enrichment plot: P53GENES\_ALL      
 Profile of the Running ES Score & Positions of GeneSet Members on the Rank Ordered List

  

| PROBE | GENE SYMBOL | GENE\_TITLE | RANK IN GENE LIST | RANK METRIC SCORE | RUNNING ES | CORE ENRICHMENT || 1 | THBS1 |  |  | 2263 | 0.242 | -0.0117 | No |
| 2 | BBC3 |  |  | 2497 | 0.230 | 0.0702 | No |
| 3 | PCNA |  |  | 3626 | 0.190 | 0.0927 | No |
| 4 | CCNG1 |  |  | 4399 | 0.169 | 0.1237 | No |
| 5 | BAX |  |  | 4911 | 0.157 | 0.1627 | No |
| 6 | TNFRSF10B |  |  | 10316 | 0.068 | -0.0720 | No |
| 7 | IGFBP3 |  |  | 12384 | 0.042 | -0.1554 | No |
| 8 | P53AIP1 |  |  | 13800 | 0.023 | -0.2148 | No |
| 9 | SESN1 |  |  | 15353 | 0.000 | -0.2902 | No |
| 10 | MDM2 |  |  | 15404 | -0.000 | -0.2924 | No |
| 11 | CDKN1A |  |  | 16349 | -0.016 | -0.3316 | No |
| 12 | FASLG |  |  | 16465 | -0.018 | -0.3298 | No |
| 13 | IER3 |  |  | 20158 | -0.165 | -0.4420 | Yes |
| 14 | FOS |  |  | 20334 | -0.202 | -0.3683 | Yes |
| 15 | PMAIP1 |  |  | 20440 | -0.248 | -0.2729 | Yes |
| 16 | GADD45A |  |  | 20478 | -0.277 | -0.1623 | Yes |
| 17 | SFN |  |  | 20552 | -0.415 | 0.0026 | Yes |
Table: GSEA details [plain text format]

  

Fig 2: P53GENES\_ALL: Random ES distribution      
 Gene set null distribution of ES for **P53GENES\_ALL**
