# Supplementary material for: The rapamycin-regulated gene expression signature determines prognosis for breast cancer
Source: Mol Cancer. 2009 Sep 24;8:75. doi: 10.1186/1476-4598-8-75 (PMC2761377; doi:10.1186/1476-4598-8-75)
Supplement: Additional file 3 — Gene set enrichment analysis of in vivo data, treatment series. The data provided represent the treatment series of GSEA. This compressed file contains "Treatment" shortcut file and "GSEA_treatment" folder. Clicking on "Treatment" shortcut opens the index file providing access to analysis files contained in the "GSEA_treatment" folder. [file 1476-4598-8-75-S3.zip › GSEA_treatment/PARK_HSC_VS_MPP_UP.html]

Details for gene set PARK\_HSC\_VS\_MPP\_UP[GSEA]

|  || Dataset | gsea\_treatment\_collapsed |
| Phenotype | NoPhenotypeAvailable |
| Upregulated in class | na\_pos |
| GeneSet | PARK\_HSC\_VS\_MPP\_UP |
| Enrichment Score (ES) | 0.7021376 |
| Normalized Enrichment Score (NES) | 1.6917294 |
| Nominal p-value | 0.0022222223 |
| FDR q-value | 0.013451697 |
| FWER p-Value | 0.469 |
Table: GSEA Results Summary

  

Fig 1: Enrichment plot: PARK\_HSC\_VS\_MPP\_UP      
 Profile of the Running ES Score & Positions of GeneSet Members on the Rank Ordered List

  

| PROBE | GENE SYMBOL | GENE\_TITLE | RANK IN GENE LIST | RANK METRIC SCORE | RUNNING ES | CORE ENRICHMENT || 1 | RBM3 |  |  | 4 | 1.110 | 0.2882 | Yes |
| 2 | HMGB1 |  |  | 247 | 0.469 | 0.3982 | Yes |
| 3 | CAPNS1 |  |  | 372 | 0.434 | 0.5049 | Yes |
| 4 | TMEM14A |  |  | 430 | 0.419 | 0.6109 | Yes |
| 5 | NFIX |  |  | 605 | 0.384 | 0.7021 | Yes |
| 6 | CYLN2 |  |  | 2567 | 0.227 | 0.6659 | No |
| 7 | PRKAR1A |  |  | 3195 | 0.203 | 0.6881 | No |
| 8 | PABPC1 |  |  | 4326 | 0.171 | 0.6777 | No |
| 9 | H2AFY |  |  | 6421 | 0.128 | 0.6092 | No |
| 10 | HAT1 |  |  | 8918 | 0.088 | 0.5109 | No |
| 11 | CUGBP2 |  |  | 11073 | 0.059 | 0.4215 | No |
| 12 | NEDD4 |  |  | 11137 | 0.058 | 0.4334 | No |
| 13 | RAMP2 |  |  | 12195 | 0.044 | 0.3936 | No |
| 14 | CCT7 |  |  | 13351 | 0.029 | 0.3451 | No |
| 15 | GIMAP6 |  |  | 13844 | 0.023 | 0.3271 | No |
| 16 | RPGRIP1 |  |  | 15057 | 0.005 | 0.2695 | No |
Table: GSEA details [plain text format]

  

Fig 2: PARK\_HSC\_VS\_MPP\_UP: Random ES distribution      
 Gene set null distribution of ES for **PARK\_HSC\_VS\_MPP\_UP**
