# Supplementary material for: The rapamycin-regulated gene expression signature determines prognosis for breast cancer
Source: Mol Cancer. 2009 Sep 24;8:75. doi: 10.1186/1476-4598-8-75 (PMC2761377; doi:10.1186/1476-4598-8-75)
Supplement: Additional file 3 — Gene set enrichment analysis of in vivo data, treatment series. The data provided represent the treatment series of GSEA. This compressed file contains "Treatment" shortcut file and "GSEA_treatment" folder. Clicking on "Treatment" shortcut opens the index file providing access to analysis files contained in the "GSEA_treatment" folder. [file 1476-4598-8-75-S3.zip › GSEA_treatment/PEPTIDE_GPCRS.html]

Details for gene set PEPTIDE\_GPCRS[GSEA]

|  || Dataset | gsea\_treatment\_collapsed |
| Phenotype | NoPhenotypeAvailable |
| Upregulated in class | na\_neg |
| GeneSet | PEPTIDE\_GPCRS |
| Enrichment Score (ES) | -0.19635999 |
| Normalized Enrichment Score (NES) | -0.90214264 |
| Nominal p-value | 0.6666667 |
| FDR q-value | 0.709476 |
| FWER p-Value | 1.0 |
Table: GSEA Results Summary

  

Fig 1: Enrichment plot: PEPTIDE\_GPCRS      
 Profile of the Running ES Score & Positions of GeneSet Members on the Rank Ordered List

  

| PROBE | GENE SYMBOL | GENE\_TITLE | RANK IN GENE LIST | RANK METRIC SCORE | RUNNING ES | CORE ENRICHMENT || 1 | EDNRA |  |  | 3771 | 0.186 | -0.1398 | No |
| 2 | AVPR1A |  |  | 4521 | 0.166 | -0.1373 | No |
| 3 | GALT |  |  | 5083 | 0.153 | -0.1285 | No |
| 4 | FPRL1 |  |  | 6075 | 0.134 | -0.1452 | No |
| 5 | OPRL1 |  |  | 7124 | 0.115 | -0.1690 | No |
| 6 | AVPR2 |  |  | 7357 | 0.112 | -0.1539 | No |
| 7 | CX3CR1 |  |  | 8229 | 0.098 | -0.1733 | Yes |
| 8 | GNB2L1 |  |  | 8560 | 0.093 | -0.1674 | Yes |
| 9 | NPY5R |  |  | 8644 | 0.092 | -0.1498 | Yes |
| 10 | PPYR1 |  |  | 8797 | 0.090 | -0.1360 | Yes |
| 11 | GNRHR |  |  | 9054 | 0.086 | -0.1282 | Yes |
| 12 | TACR1 |  |  | 9486 | 0.080 | -0.1303 | Yes |
| 13 | NPY6R |  |  | 9630 | 0.078 | -0.1189 | Yes |
| 14 | GALR2 |  |  | 9675 | 0.077 | -0.1029 | Yes |
| 15 | MC1R |  |  | 9859 | 0.076 | -0.0940 | Yes |
| 16 | CCKAR |  |  | 10374 | 0.068 | -0.1031 | Yes |
| 17 | TACR3 |  |  | 10620 | 0.064 | -0.0998 | Yes |
| 18 | TAC4 |  |  | 10634 | 0.064 | -0.0853 | Yes |
| 19 | NPY2R |  |  | 10916 | 0.061 | -0.0847 | Yes |
| 20 | ATP8A1 |  |  | 10947 | 0.060 | -0.0720 | Yes |
| 21 | CCKBR |  |  | 11006 | 0.060 | -0.0608 | Yes |
| 22 | MC3R |  |  | 11008 | 0.060 | -0.0468 | Yes |
| 23 | SSTR2 |  |  | 11258 | 0.056 | -0.0457 | Yes |
| 24 | ELA3A |  |  | 11273 | 0.056 | -0.0332 | Yes |
| 25 | CXCR6 |  |  | 11283 | 0.056 | -0.0204 | Yes |
| 26 | CXCR3 |  |  | 11288 | 0.056 | -0.0073 | Yes |
| 27 | BLR1 |  |  | 11324 | 0.056 | 0.0041 | Yes |
| 28 | MC4R |  |  | 11355 | 0.055 | 0.0157 | Yes |
| 29 | SSTR4 |  |  | 11448 | 0.054 | 0.0240 | Yes |
| 30 | SSTR1 |  |  | 11852 | 0.049 | 0.0158 | Yes |
| 31 | GALR3 |  |  | 11979 | 0.047 | 0.0208 | Yes |
| 32 | TSHR |  |  | 12010 | 0.047 | 0.0303 | Yes |
| 33 | CCR1 |  |  | 12230 | 0.044 | 0.0299 | Yes |
| 34 | CCR10 |  |  | 12366 | 0.042 | 0.0333 | Yes |
| 35 | EDNRB |  |  | 12640 | 0.038 | 0.0291 | Yes |
| 36 | SSTR3 |  |  | 13398 | 0.029 | -0.0010 | Yes |
| 37 | CCR2 |  |  | 13529 | 0.027 | -0.0009 | Yes |
| 38 | GRPR |  |  | 14158 | 0.018 | -0.0272 | Yes |
| 39 | LHCGR |  |  | 14177 | 0.018 | -0.0237 | Yes |
| 40 | OPRM1 |  |  | 14363 | 0.015 | -0.0291 | Yes |
| 41 | BDKRB2 |  |  | 14390 | 0.015 | -0.0269 | Yes |
| 42 | AGTR1 |  |  | 14404 | 0.015 | -0.0240 | Yes |
| 43 | IL8RA |  |  | 14413 | 0.015 | -0.0209 | Yes |
| 44 | OPRD1 |  |  | 14573 | 0.012 | -0.0258 | Yes |
| 45 | CXCR4 |  |  | 14667 | 0.011 | -0.0277 | Yes |
| 46 | CCR3 |  |  | 14770 | 0.010 | -0.0304 | Yes |
| 47 | GHSR |  |  | 14783 | 0.010 | -0.0288 | Yes |
| 48 | MC2R |  |  | 14843 | 0.009 | -0.0296 | Yes |
| 49 | IL8RB |  |  | 14882 | 0.008 | -0.0296 | Yes |
| 50 | FPR1 |  |  | 14968 | 0.006 | -0.0323 | Yes |
| 51 | CCR6 |  |  | 15236 | 0.002 | -0.0447 | Yes |
| 52 | FPRL2 |  |  | 15248 | 0.002 | -0.0448 | Yes |
| 53 | AVPR1B |  |  | 15438 | -0.001 | -0.0538 | Yes |
| 54 | GPR77 |  |  | 15852 | -0.008 | -0.0721 | Yes |
| 55 | GALR1 |  |  | 16090 | -0.012 | -0.0808 | Yes |
| 56 | CCR7 |  |  | 16267 | -0.014 | -0.0860 | Yes |
| 57 | BDKRB1 |  |  | 16892 | -0.026 | -0.1103 | Yes |
| 58 | BRS3 |  |  | 17048 | -0.030 | -0.1108 | Yes |
| 59 | TRHR |  |  | 17077 | -0.030 | -0.1050 | Yes |
| 60 | NMBR |  |  | 17160 | -0.032 | -0.1014 | Yes |
| 61 | AGTR2 |  |  | 17573 | -0.041 | -0.1118 | Yes |
| 62 | NTSR1 |  |  | 17647 | -0.043 | -0.1051 | Yes |
| 63 | TACR2 |  |  | 17655 | -0.043 | -0.0953 | Yes |
| 64 | FSHR |  |  | 17800 | -0.047 | -0.0913 | Yes |
| 65 | OXTR |  |  | 18220 | -0.057 | -0.0983 | Yes |
| 66 | C3AR1 |  |  | 19264 | -0.096 | -0.1264 | Yes |
| 67 | NPY1R |  |  | 19384 | -0.102 | -0.1081 | Yes |
| 68 | OPRK1 |  |  | 19478 | -0.107 | -0.0874 | Yes |
| 69 | NTSR2 |  |  | 19739 | -0.122 | -0.0713 | Yes |
| 70 | CCR4 |  |  | 20018 | -0.146 | -0.0505 | Yes |
| 71 | MC5R |  |  | 20168 | -0.167 | -0.0185 | Yes |
| 72 | CCR8 |  |  | 20178 | -0.169 | 0.0208 | Yes |
Table: GSEA details [plain text format]

  

Fig 2: PEPTIDE\_GPCRS: Random ES distribution      
 Gene set null distribution of ES for **PEPTIDE\_GPCRS**
