# Supplementary material for: The rapamycin-regulated gene expression signature determines prognosis for breast cancer
Source: Mol Cancer. 2009 Sep 24;8:75. doi: 10.1186/1476-4598-8-75 (PMC2761377; doi:10.1186/1476-4598-8-75)
Supplement: Additional file 3 — Gene set enrichment analysis of in vivo data, treatment series. The data provided represent the treatment series of GSEA. This compressed file contains "Treatment" shortcut file and "GSEA_treatment" folder. Clicking on "Treatment" shortcut opens the index file providing access to analysis files contained in the "GSEA_treatment" folder. [file 1476-4598-8-75-S3.zip › GSEA_treatment/POD1_KO_MOST_DN.html]

Details for gene set POD1\_KO\_MOST\_DN[GSEA]

|  || Dataset | gsea\_treatment\_collapsed |
| Phenotype | NoPhenotypeAvailable |
| Upregulated in class | na\_neg |
| GeneSet | POD1\_KO\_MOST\_DN |
| Enrichment Score (ES) | -0.2639802 |
| Normalized Enrichment Score (NES) | -0.9462774 |
| Nominal p-value | 0.59375 |
| FDR q-value | 0.66139996 |
| FWER p-Value | 1.0 |
Table: GSEA Results Summary

  

Fig 1: Enrichment plot: POD1\_KO\_MOST\_DN      
 Profile of the Running ES Score & Positions of GeneSet Members on the Rank Ordered List

  

| PROBE | GENE SYMBOL | GENE\_TITLE | RANK IN GENE LIST | RANK METRIC SCORE | RUNNING ES | CORE ENRICHMENT || 1 | SLC12A2 |  |  | 449 | 0.414 | 0.0929 | Yes |
| 2 | PARP4 |  |  | 2069 | 0.252 | 0.0842 | Yes |
| 3 | TOP1MT |  |  | 3404 | 0.197 | 0.0739 | Yes |
| 4 | RHPN1 |  |  | 3482 | 0.194 | 0.1241 | Yes |
| 5 | SH3BGRL2 |  |  | 4036 | 0.178 | 0.1467 | Yes |
| 6 | MERTK |  |  | 4211 | 0.174 | 0.1865 | Yes |
| 7 | ART3 |  |  | 5024 | 0.155 | 0.1900 | Yes |
| 8 | HIST1H3G |  |  | 5045 | 0.154 | 0.2319 | Yes |
| 9 | PDE10A |  |  | 7213 | 0.114 | 0.1582 | No |
| 10 | COL4A3 |  |  | 7621 | 0.108 | 0.1683 | No |
| 11 | PTGER4 |  |  | 7819 | 0.105 | 0.1878 | No |
| 12 | TSPAN2 |  |  | 9078 | 0.086 | 0.1504 | No |
| 13 | SEMA3E |  |  | 9382 | 0.082 | 0.1584 | No |
| 14 | UMOD |  |  | 12305 | 0.043 | 0.0283 | No |
| 15 | LRRFIP1 |  |  | 12522 | 0.040 | 0.0289 | No |
| 16 | MAPT |  |  | 12665 | 0.038 | 0.0326 | No |
| 17 | CLIC5 |  |  | 12923 | 0.035 | 0.0299 | No |
| 18 | TLR4 |  |  | 13895 | 0.022 | -0.0112 | No |
| 19 | GABRA4 |  |  | 14677 | 0.011 | -0.0462 | No |
| 20 | SGIP1 |  |  | 15241 | 0.002 | -0.0730 | No |
| 21 | EGF |  |  | 18352 | -0.061 | -0.2072 | No |
| 22 | NPHS2 |  |  | 19106 | -0.089 | -0.2191 | No |
| 23 | GALC |  |  | 19205 | -0.093 | -0.1979 | No |
| 24 | HSPA1B |  |  | 20565 | -0.463 | -0.1355 | No |
| 25 | ADM |  |  | 20571 | -0.495 | 0.0017 | No |
Table: GSEA details [plain text format]

  

Fig 2: POD1\_KO\_MOST\_DN: Random ES distribution      
 Gene set null distribution of ES for **POD1\_KO\_MOST\_DN**
