# Supplementary material for: The rapamycin-regulated gene expression signature determines prognosis for breast cancer
Source: Mol Cancer. 2009 Sep 24;8:75. doi: 10.1186/1476-4598-8-75 (PMC2761377; doi:10.1186/1476-4598-8-75)
Supplement: Additional file 3 — Gene set enrichment analysis of in vivo data, treatment series. The data provided represent the treatment series of GSEA. This compressed file contains "Treatment" shortcut file and "GSEA_treatment" folder. Clicking on "Treatment" shortcut opens the index file providing access to analysis files contained in the "GSEA_treatment" folder. [file 1476-4598-8-75-S3.zip › GSEA_treatment/PYRUVATE_METABOLISM.html]

Details for gene set PYRUVATE\_METABOLISM[GSEA]

|  || Dataset | gsea\_treatment\_collapsed |
| Phenotype | NoPhenotypeAvailable |
| Upregulated in class | na\_neg |
| GeneSet | PYRUVATE\_METABOLISM |
| Enrichment Score (ES) | -0.28750563 |
| Normalized Enrichment Score (NES) | -1.244269 |
| Nominal p-value | 0.078947365 |
| FDR q-value | 0.2802519 |
| FWER p-Value | 1.0 |
Table: GSEA Results Summary

  

Fig 1: Enrichment plot: PYRUVATE\_METABOLISM      
 Profile of the Running ES Score & Positions of GeneSet Members on the Rank Ordered List

  

| PROBE | GENE SYMBOL | GENE\_TITLE | RANK IN GENE LIST | RANK METRIC SCORE | RUNNING ES | CORE ENRICHMENT || 1 | ALDH1A3 |  |  | 201 | 0.491 | 0.0736 | Yes |
| 2 | AKR1B1 |  |  | 1224 | 0.308 | 0.0763 | Yes |
| 3 | ACYP2 |  |  | 1708 | 0.272 | 0.0990 | Yes |
| 4 | ALDH1A1 |  |  | 1897 | 0.261 | 0.1341 | Yes |
| 5 | MDH1 |  |  | 2181 | 0.246 | 0.1621 | Yes |
| 6 | PC |  |  | 2386 | 0.236 | 0.1922 | Yes |
| 7 | PDHB |  |  | 2557 | 0.227 | 0.2225 | Yes |
| 8 | GRHPR |  |  | 2906 | 0.214 | 0.2419 | Yes |
| 9 | HAGH |  |  | 3289 | 0.200 | 0.2573 | Yes |
| 10 | ADH5 |  |  | 3527 | 0.193 | 0.2785 | Yes |
| 11 | ALDH1B1 |  |  | 5730 | 0.140 | 0.1952 | No |
| 12 | DLD |  |  | 5864 | 0.137 | 0.2120 | No |
| 13 | MDH2 |  |  | 5920 | 0.137 | 0.2325 | No |
| 14 | HAGHL |  |  | 7385 | 0.112 | 0.1803 | No |
| 15 | ME3 |  |  | 7881 | 0.103 | 0.1738 | No |
| 16 | GLO1 |  |  | 8636 | 0.092 | 0.1527 | No |
| 17 | PKM2 |  |  | 8667 | 0.091 | 0.1667 | No |
| 18 | DLAT |  |  | 10308 | 0.069 | 0.0987 | No |
| 19 | LDHD |  |  | 10784 | 0.063 | 0.0862 | No |
| 20 | ME2 |  |  | 10948 | 0.060 | 0.0885 | No |
| 21 | LDHC |  |  | 11189 | 0.057 | 0.0865 | No |
| 22 | ALDH3A1 |  |  | 12761 | 0.037 | 0.0164 | No |
| 23 | PDHA1 |  |  | 13233 | 0.031 | -0.0012 | No |
| 24 | PKLR |  |  | 14035 | 0.020 | -0.0367 | No |
| 25 | PDHA2 |  |  | 14914 | 0.007 | -0.0781 | No |
| 26 | ACACA |  |  | 15028 | 0.005 | -0.0827 | No |
| 27 | PCK1 |  |  | 15655 | -0.004 | -0.1124 | No |
| 28 | ACYP1 |  |  | 15884 | -0.008 | -0.1221 | No |
| 29 | ALDH3A2 |  |  | 16233 | -0.014 | -0.1367 | No |
| 30 | ALDH9A1 |  |  | 16867 | -0.025 | -0.1631 | No |
| 31 | ACAT1 |  |  | 17187 | -0.033 | -0.1731 | No |
| 32 | ALDH1A2 |  |  | 18290 | -0.059 | -0.2166 | No |
| 33 | LDHA |  |  | 19554 | -0.112 | -0.2591 | No |
| 34 | ME1 |  |  | 19559 | -0.112 | -0.2403 | No |
| 35 | ALDH2 |  |  | 20530 | -0.348 | -0.2284 | No |
| 36 | ACAT2 |  |  | 20577 | -0.511 | -0.1440 | No |
| 37 | LDHB |  |  | 20594 | -0.856 | 0.0005 | No |
Table: GSEA details [plain text format]

  

Fig 2: PYRUVATE\_METABOLISM: Random ES distribution      
 Gene set null distribution of ES for **PYRUVATE\_METABOLISM**
