# Supplementary material for: The rapamycin-regulated gene expression signature determines prognosis for breast cancer
Source: Mol Cancer. 2009 Sep 24;8:75. doi: 10.1186/1476-4598-8-75 (PMC2761377; doi:10.1186/1476-4598-8-75)
Supplement: Additional file 3 — Gene set enrichment analysis of in vivo data, treatment series. The data provided represent the treatment series of GSEA. This compressed file contains "Treatment" shortcut file and "GSEA_treatment" folder. Clicking on "Treatment" shortcut opens the index file providing access to analysis files contained in the "GSEA_treatment" folder. [file 1476-4598-8-75-S3.zip › GSEA_treatment/RADAEVA_IFNA_UP.html]

Details for gene set RADAEVA\_IFNA\_UP[GSEA]

|  || Dataset | gsea\_treatment\_collapsed |
| Phenotype | NoPhenotypeAvailable |
| Upregulated in class | na\_pos |
| GeneSet | RADAEVA\_IFNA\_UP |
| Enrichment Score (ES) | 0.70738435 |
| Normalized Enrichment Score (NES) | 2.011078 |
| Nominal p-value | 0.0 |
| FDR q-value | 1.6478646E-4 |
| FWER p-Value | 0.0010 |
Table: GSEA Results Summary

  

Fig 1: Enrichment plot: RADAEVA\_IFNA\_UP      
 Profile of the Running ES Score & Positions of GeneSet Members on the Rank Ordered List

  

| PROBE | GENE SYMBOL | GENE\_TITLE | RANK IN GENE LIST | RANK METRIC SCORE | RUNNING ES | CORE ENRICHMENT || 1 | MX1 |  |  | 24 | 0.773 | 0.0529 | Yes |
| 2 | IFI27 |  |  | 32 | 0.725 | 0.1032 | Yes |
| 3 | HLA-B |  |  | 41 | 0.695 | 0.1515 | Yes |
| 4 | ISG15 |  |  | 59 | 0.644 | 0.1957 | Yes |
| 5 | LGALS3BP |  |  | 61 | 0.643 | 0.2406 | Yes |
| 6 | IFITM1 |  |  | 79 | 0.609 | 0.2824 | Yes |
| 7 | HLA-E |  |  | 126 | 0.545 | 0.3183 | Yes |
| 8 | TNFSF10 |  |  | 160 | 0.518 | 0.3529 | Yes |
| 9 | CXCL11 |  |  | 228 | 0.479 | 0.3831 | Yes |
| 10 | OAS1 |  |  | 255 | 0.466 | 0.4145 | Yes |
| 11 | PSME1 |  |  | 513 | 0.402 | 0.4301 | Yes |
| 12 | IRF1 |  |  | 551 | 0.393 | 0.4558 | Yes |
| 13 | IRF7 |  |  | 639 | 0.378 | 0.4779 | Yes |
| 14 | BST2 |  |  | 657 | 0.374 | 0.5033 | Yes |
| 15 | PLSCR1 |  |  | 717 | 0.365 | 0.5259 | Yes |
| 16 | IFI6 |  |  | 726 | 0.364 | 0.5509 | Yes |
| 17 | IFI35 |  |  | 766 | 0.357 | 0.5740 | Yes |
| 18 | AKR7A2 |  |  | 809 | 0.352 | 0.5966 | Yes |
| 19 | ADAR |  |  | 885 | 0.344 | 0.6170 | Yes |
| 20 | UBE2L6 |  |  | 980 | 0.333 | 0.6357 | Yes |
| 21 | IFI44 |  |  | 1039 | 0.327 | 0.6558 | Yes |
| 22 | TUBB |  |  | 1267 | 0.305 | 0.6661 | Yes |
| 23 | STAT1 |  |  | 1401 | 0.294 | 0.6802 | Yes |
| 24 | CDKN1B |  |  | 1757 | 0.269 | 0.6818 | Yes |
| 25 | EIF2AK2 |  |  | 1990 | 0.255 | 0.6883 | Yes |
| 26 | CALR |  |  | 2783 | 0.219 | 0.6651 | Yes |
| 27 | UBE2D3 |  |  | 2819 | 0.217 | 0.6786 | Yes |
| 28 | IFIT2 |  |  | 3056 | 0.208 | 0.6817 | Yes |
| 29 | RSAD2 |  |  | 3107 | 0.206 | 0.6937 | Yes |
| 30 | MXD4 |  |  | 3253 | 0.201 | 0.7007 | Yes |
| 31 | IFITM2 |  |  | 3605 | 0.191 | 0.6970 | Yes |
| 32 | RFC2 |  |  | 3877 | 0.183 | 0.6966 | Yes |
| 33 | NTRK1 |  |  | 3918 | 0.182 | 0.7074 | Yes |
| 34 | PIAS4 |  |  | 4415 | 0.169 | 0.6951 | No |
| 35 | BAX |  |  | 4911 | 0.157 | 0.6820 | No |
| 36 | PHB |  |  | 6528 | 0.126 | 0.6122 | No |
| 37 | VAT1 |  |  | 6606 | 0.125 | 0.6172 | No |
| 38 | CASP3 |  |  | 7277 | 0.113 | 0.5925 | No |
| 39 | BCL2 |  |  | 8111 | 0.100 | 0.5590 | No |
| 40 | IFITM3 |  |  | 8420 | 0.095 | 0.5507 | No |
| 41 | SOCS1 |  |  | 8423 | 0.095 | 0.5572 | No |
| 42 | KLK3 |  |  | 8478 | 0.094 | 0.5612 | No |
| 43 | KIAA0409 |  |  | 9465 | 0.080 | 0.5188 | No |
| 44 | CXCL10 |  |  | 10182 | 0.071 | 0.4889 | No |
| 45 | VCAM1 |  |  | 13537 | 0.027 | 0.3277 | No |
| 46 | IFI44L |  |  | 14255 | 0.017 | 0.2940 | No |
| 47 | IL12RB2 |  |  | 15366 | 0.000 | 0.2400 | No |
| 48 | E2F2 |  |  | 17657 | -0.043 | 0.1316 | No |
| 49 | CACNA2D2 |  |  | 17782 | -0.046 | 0.1288 | No |
| 50 | MX2 |  |  | 19725 | -0.121 | 0.0428 | No |
Table: GSEA details [plain text format]

  

Fig 2: RADAEVA\_IFNA\_UP: Random ES distribution      
 Gene set null distribution of ES for **RADAEVA\_IFNA\_UP**
