# Supplementary material for: The rapamycin-regulated gene expression signature determines prognosis for breast cancer
Source: Mol Cancer. 2009 Sep 24;8:75. doi: 10.1186/1476-4598-8-75 (PMC2761377; doi:10.1186/1476-4598-8-75)
Supplement: Additional file 3 — Gene set enrichment analysis of in vivo data, treatment series. The data provided represent the treatment series of GSEA. This compressed file contains "Treatment" shortcut file and "GSEA_treatment" folder. Clicking on "Treatment" shortcut opens the index file providing access to analysis files contained in the "GSEA_treatment" folder. [file 1476-4598-8-75-S3.zip › GSEA_treatment/RESISTANCE_XENOGRAFTS_UP.html]

Details for gene set RESISTANCE\_XENOGRAFTS\_UP[GSEA]

|  || Dataset | gsea\_treatment\_collapsed |
| Phenotype | NoPhenotypeAvailable |
| Upregulated in class | na\_pos |
| GeneSet | RESISTANCE\_XENOGRAFTS\_UP |
| Enrichment Score (ES) | 0.69069046 |
| Normalized Enrichment Score (NES) | 1.8077762 |
| Nominal p-value | 0.0 |
| FDR q-value | 0.0025666333 |
| FWER p-Value | 0.057 |
Table: GSEA Results Summary

  

Fig 1: Enrichment plot: RESISTANCE\_XENOGRAFTS\_UP      
 Profile of the Running ES Score & Positions of GeneSet Members on the Rank Ordered List

  

| PROBE | GENE SYMBOL | GENE\_TITLE | RANK IN GENE LIST | RANK METRIC SCORE | RUNNING ES | CORE ENRICHMENT || 1 | CD74 |  |  | 25 | 0.764 | 0.1176 | Yes |
| 2 | CFB |  |  | 45 | 0.688 | 0.2236 | Yes |
| 3 | ABLIM1 |  |  | 111 | 0.564 | 0.3081 | Yes |
| 4 | SYNGR2 |  |  | 569 | 0.391 | 0.3468 | Yes |
| 5 | RNASET2 |  |  | 661 | 0.373 | 0.4004 | Yes |
| 6 | ABP1 |  |  | 1149 | 0.316 | 0.4258 | Yes |
| 7 | ATP1B1 |  |  | 1248 | 0.307 | 0.4688 | Yes |
| 8 | ANXA4 |  |  | 1541 | 0.284 | 0.4987 | Yes |
| 9 | FCGRT |  |  | 1542 | 0.284 | 0.5428 | Yes |
| 10 | IL32 |  |  | 1685 | 0.274 | 0.5784 | Yes |
| 11 | TNFRSF14 |  |  | 1872 | 0.263 | 0.6102 | Yes |
| 12 | SERPINA1 |  |  | 2104 | 0.250 | 0.6379 | Yes |
| 13 | TMEPAI |  |  | 2119 | 0.250 | 0.6760 | Yes |
| 14 | ACSL5 |  |  | 2547 | 0.228 | 0.6907 | Yes |
| 15 | C11ORF9 |  |  | 3754 | 0.187 | 0.6611 | No |
| 16 | PDIA4 |  |  | 4956 | 0.156 | 0.6270 | No |
| 17 | LAMB3 |  |  | 5853 | 0.138 | 0.6049 | No |
| 18 | MYH14 |  |  | 6693 | 0.124 | 0.5833 | No |
| 19 | MVP |  |  | 8012 | 0.101 | 0.5350 | No |
| 20 | USH1C |  |  | 9550 | 0.079 | 0.4727 | No |
| 21 | CYP3A5 |  |  | 10432 | 0.067 | 0.4403 | No |
| 22 | MUC13 |  |  | 12070 | 0.046 | 0.3678 | No |
| 23 | PCDH1 |  |  | 12330 | 0.042 | 0.3618 | No |
| 24 | ARSE |  |  | 12588 | 0.039 | 0.3555 | No |
| 25 | LGALS4 |  |  | 17033 | -0.029 | 0.1441 | No |
| 26 | LLGL2 |  |  | 18185 | -0.056 | 0.0969 | No |
| 27 | TSPAN18 |  |  | 18191 | -0.056 | 0.1054 | No |
| 28 | GJB1 |  |  | 18804 | -0.076 | 0.0875 | No |
Table: GSEA details [plain text format]

  

Fig 2: RESISTANCE\_XENOGRAFTS\_UP: Random ES distribution      
 Gene set null distribution of ES for **RESISTANCE\_XENOGRAFTS\_UP**
