# Supplementary material for: The rapamycin-regulated gene expression signature determines prognosis for breast cancer
Source: Mol Cancer. 2009 Sep 24;8:75. doi: 10.1186/1476-4598-8-75 (PMC2761377; doi:10.1186/1476-4598-8-75)
Supplement: Additional file 3 — Gene set enrichment analysis of in vivo data, treatment series. The data provided represent the treatment series of GSEA. This compressed file contains "Treatment" shortcut file and "GSEA_treatment" folder. Clicking on "Treatment" shortcut opens the index file providing access to analysis files contained in the "GSEA_treatment" folder. [file 1476-4598-8-75-S3.zip › GSEA_treatment/RIBAVIRIN_RSV_UP.html]

Details for gene set RIBAVIRIN\_RSV\_UP[GSEA]

|  || Dataset | gsea\_treatment\_collapsed |
| Phenotype | NoPhenotypeAvailable |
| Upregulated in class | na\_pos |
| GeneSet | RIBAVIRIN\_RSV\_UP |
| Enrichment Score (ES) | 0.7293255 |
| Normalized Enrichment Score (NES) | 1.844149 |
| Nominal p-value | 0.0 |
| FDR q-value | 0.0017485152 |
| FWER p-Value | 0.03 |
Table: GSEA Results Summary

  

Fig 1: Enrichment plot: RIBAVIRIN\_RSV\_UP      
 Profile of the Running ES Score & Positions of GeneSet Members on the Rank Ordered List

  

| PROBE | GENE SYMBOL | GENE\_TITLE | RANK IN GENE LIST | RANK METRIC SCORE | RUNNING ES | CORE ENRICHMENT || 1 | MDK |  |  | 3 | 1.126 | 0.1671 | Yes |
| 2 | MX1 |  |  | 24 | 0.773 | 0.2809 | Yes |
| 3 | IFI27 |  |  | 32 | 0.725 | 0.3883 | Yes |
| 4 | IFITM1 |  |  | 79 | 0.609 | 0.4765 | Yes |
| 5 | LCN2 |  |  | 262 | 0.463 | 0.5364 | Yes |
| 6 | IRF7 |  |  | 639 | 0.378 | 0.5742 | Yes |
| 7 | CD81 |  |  | 693 | 0.368 | 0.6263 | Yes |
| 8 | C1S |  |  | 714 | 0.366 | 0.6797 | Yes |
| 9 | STAT1 |  |  | 1401 | 0.294 | 0.6901 | Yes |
| 10 | MYH11 |  |  | 2320 | 0.239 | 0.6811 | Yes |
| 11 | SOX9 |  |  | 3007 | 0.210 | 0.6790 | Yes |
| 12 | CD99 |  |  | 3154 | 0.205 | 0.7023 | Yes |
| 13 | ASS1 |  |  | 3567 | 0.192 | 0.7108 | Yes |
| 14 | TFDP1 |  |  | 3757 | 0.187 | 0.7293 | Yes |
| 15 | PIM2 |  |  | 5797 | 0.138 | 0.6508 | No |
| 16 | APOL1 |  |  | 6770 | 0.122 | 0.6218 | No |
| 17 | IFITM3 |  |  | 8420 | 0.095 | 0.5558 | No |
| 18 | TIAL1 |  |  | 9872 | 0.075 | 0.4964 | No |
| 19 | GJA1 |  |  | 12062 | 0.046 | 0.3969 | No |
| 20 | GPX2 |  |  | 12528 | 0.040 | 0.3802 | No |
| 21 | VTN |  |  | 12551 | 0.040 | 0.3851 | No |
| 22 | PDLIM1 |  |  | 17551 | -0.041 | 0.1484 | No |
Table: GSEA details [plain text format]

  

Fig 2: RIBAVIRIN\_RSV\_UP: Random ES distribution      
 Gene set null distribution of ES for **RIBAVIRIN\_RSV\_UP**
