# Supplementary material for: The rapamycin-regulated gene expression signature determines prognosis for breast cancer
Source: Mol Cancer. 2009 Sep 24;8:75. doi: 10.1186/1476-4598-8-75 (PMC2761377; doi:10.1186/1476-4598-8-75)
Supplement: Additional file 3 — Gene set enrichment analysis of in vivo data, treatment series. The data provided represent the treatment series of GSEA. This compressed file contains "Treatment" shortcut file and "GSEA_treatment" folder. Clicking on "Treatment" shortcut opens the index file providing access to analysis files contained in the "GSEA_treatment" folder. [file 1476-4598-8-75-S3.zip › GSEA_treatment/SANA_IFNG_ENDOTHELIAL_UP.html]

Details for gene set SANA\_IFNG\_ENDOTHELIAL\_UP[GSEA]

|  || Dataset | gsea\_treatment\_collapsed |
| Phenotype | NoPhenotypeAvailable |
| Upregulated in class | na\_pos |
| GeneSet | SANA\_IFNG\_ENDOTHELIAL\_UP |
| Enrichment Score (ES) | 0.6812302 |
| Normalized Enrichment Score (NES) | 2.0078294 |
| Nominal p-value | 0.0 |
| FDR q-value | 1.4124555E-4 |
| FWER p-Value | 0.0010 |
Table: GSEA Results Summary

  

Fig 1: Enrichment plot: SANA\_IFNG\_ENDOTHELIAL\_UP      
 Profile of the Running ES Score & Positions of GeneSet Members on the Rank Ordered List

  

| PROBE | GENE SYMBOL | GENE\_TITLE | RANK IN GENE LIST | RANK METRIC SCORE | RUNNING ES | CORE ENRICHMENT || 1 | TRIM22 |  |  | 8 | 1.024 | 0.0523 | Yes |
| 2 | MX1 |  |  | 24 | 0.773 | 0.0914 | Yes |
| 3 | CD74 |  |  | 25 | 0.764 | 0.1307 | Yes |
| 4 | HLA-B |  |  | 41 | 0.695 | 0.1658 | Yes |
| 5 | HLA-C |  |  | 57 | 0.650 | 0.1985 | Yes |
| 6 | LGALS3BP |  |  | 61 | 0.643 | 0.2315 | Yes |
| 7 | CX3CL1 |  |  | 94 | 0.586 | 0.2601 | Yes |
| 8 | LGALS9 |  |  | 95 | 0.585 | 0.2902 | Yes |
| 9 | HLA-E |  |  | 126 | 0.545 | 0.3168 | Yes |
| 10 | GBP1 |  |  | 130 | 0.542 | 0.3446 | Yes |
| 11 | TNFSF10 |  |  | 160 | 0.518 | 0.3698 | Yes |
| 12 | RARRES3 |  |  | 182 | 0.503 | 0.3947 | Yes |
| 13 | HLA-A |  |  | 195 | 0.496 | 0.4197 | Yes |
| 14 | CXCL11 |  |  | 228 | 0.479 | 0.4428 | Yes |
| 15 | OAS1 |  |  | 255 | 0.466 | 0.4655 | Yes |
| 16 | PARP14 |  |  | 291 | 0.455 | 0.4872 | Yes |
| 17 | DTX3L |  |  | 311 | 0.449 | 0.5094 | Yes |
| 18 | PARP9 |  |  | 529 | 0.399 | 0.5194 | Yes |
| 19 | LAP3 |  |  | 609 | 0.383 | 0.5353 | Yes |
| 20 | APOL3 |  |  | 632 | 0.378 | 0.5536 | Yes |
| 21 | BST2 |  |  | 657 | 0.374 | 0.5717 | Yes |
| 22 | C1S |  |  | 714 | 0.366 | 0.5878 | Yes |
| 23 | CASP1 |  |  | 732 | 0.363 | 0.6057 | Yes |
| 24 | FLJ20035 |  |  | 753 | 0.359 | 0.6232 | Yes |
| 25 | IFI35 |  |  | 766 | 0.357 | 0.6410 | Yes |
| 26 | SERPING1 |  |  | 936 | 0.338 | 0.6502 | Yes |
| 27 | UBE2L6 |  |  | 980 | 0.333 | 0.6652 | Yes |
| 28 | VAMP5 |  |  | 1350 | 0.298 | 0.6626 | Yes |
| 29 | STAT1 |  |  | 1401 | 0.294 | 0.6753 | Yes |
| 30 | GBP3 |  |  | 1775 | 0.268 | 0.6709 | Yes |
| 31 | IL23A |  |  | 2017 | 0.254 | 0.6723 | Yes |
| 32 | SAMHD1 |  |  | 2149 | 0.248 | 0.6787 | Yes |
| 33 | HLA-DPA1 |  |  | 2349 | 0.238 | 0.6812 | Yes |
| 34 | C17ORF27 |  |  | 2852 | 0.216 | 0.6679 | No |
| 35 | CEACAM1 |  |  | 2983 | 0.211 | 0.6725 | No |
| 36 | IFIH1 |  |  | 3449 | 0.195 | 0.6599 | No |
| 37 | APOL2 |  |  | 3667 | 0.189 | 0.6590 | No |
| 38 | HLA-DRB1 |  |  | 4173 | 0.175 | 0.6434 | No |
| 39 | INDO |  |  | 5656 | 0.141 | 0.5785 | No |
| 40 | SSPN |  |  | 6231 | 0.131 | 0.5573 | No |
| 41 | ATP6V0A4 |  |  | 6636 | 0.125 | 0.5441 | No |
| 42 | HLA-DQB1 |  |  | 6637 | 0.125 | 0.5505 | No |
| 43 | APOL1 |  |  | 6770 | 0.122 | 0.5504 | No |
| 44 | RAC3 |  |  | 6826 | 0.121 | 0.5539 | No |
| 45 | DHRS8 |  |  | 7169 | 0.115 | 0.5432 | No |
| 46 | NOD27 |  |  | 7215 | 0.114 | 0.5469 | No |
| 47 | BATF2 |  |  | 8344 | 0.096 | 0.4969 | No |
| 48 | CFH |  |  | 8562 | 0.093 | 0.4911 | No |
| 49 | GOLPH2 |  |  | 8700 | 0.091 | 0.4891 | No |
| 50 | ETV7 |  |  | 9260 | 0.083 | 0.4662 | No |
| 51 | GATA5 |  |  | 10086 | 0.072 | 0.4297 | No |
| 52 | HLA-DMB |  |  | 10103 | 0.072 | 0.4326 | No |
| 53 | CXCL10 |  |  | 10182 | 0.071 | 0.4324 | No |
| 54 | KCNH2 |  |  | 10272 | 0.069 | 0.4317 | No |
| 55 | APOL4 |  |  | 11002 | 0.060 | 0.3992 | No |
| 56 | PPP3CA |  |  | 11496 | 0.053 | 0.3780 | No |
| 57 | HLA-DQA1 |  |  | 11649 | 0.051 | 0.3732 | No |
| 58 | ST8SIA4 |  |  | 11704 | 0.050 | 0.3732 | No |
| 59 | SAMD9L |  |  | 12059 | 0.046 | 0.3583 | No |
| 60 | OAS2 |  |  | 12543 | 0.040 | 0.3368 | No |
| 61 | MMP25 |  |  | 13489 | 0.028 | 0.2923 | No |
| 62 | HLA-DRB4 |  |  | 14176 | 0.018 | 0.2598 | No |
| 63 | IFI44L |  |  | 14255 | 0.017 | 0.2569 | No |
| 64 | SLC25A28 |  |  | 15250 | 0.002 | 0.2086 | No |
| 65 | SEPT4 |  |  | 15753 | -0.006 | 0.1844 | No |
| 66 | CD274 |  |  | 17104 | -0.031 | 0.1203 | No |
| 67 | IL18BP |  |  | 17202 | -0.033 | 0.1173 | No |
| 68 | WARS |  |  | 17621 | -0.043 | 0.0991 | No |
| 69 | CXCL9 |  |  | 19477 | -0.107 | 0.0143 | No |
| 70 | PLA1A |  |  | 20000 | -0.144 | -0.0037 | No |
| 71 | MLKL |  |  | 20151 | -0.164 | -0.0026 | No |
| 72 | TNCRNA |  |  | 20196 | -0.171 | 0.0041 | No |
| 73 | LIPG |  |  | 20501 | -0.306 | 0.0051 | No |
Table: GSEA details [plain text format]

  

Fig 2: SANA\_IFNG\_ENDOTHELIAL\_UP: Random ES distribution      
 Gene set null distribution of ES for **SANA\_IFNG\_ENDOTHELIAL\_UP**
