# Supplementary material for: The rapamycin-regulated gene expression signature determines prognosis for breast cancer
Source: Mol Cancer. 2009 Sep 24;8:75. doi: 10.1186/1476-4598-8-75 (PMC2761377; doi:10.1186/1476-4598-8-75)
Supplement: Additional file 3 — Gene set enrichment analysis of in vivo data, treatment series. The data provided represent the treatment series of GSEA. This compressed file contains "Treatment" shortcut file and "GSEA_treatment" folder. Clicking on "Treatment" shortcut opens the index file providing access to analysis files contained in the "GSEA_treatment" folder. [file 1476-4598-8-75-S3.zip › GSEA_treatment/SANA_TNFA_ENDOTHELIAL_UP.html]

Details for gene set SANA\_TNFA\_ENDOTHELIAL\_UP[GSEA]

|  || Dataset | gsea\_treatment\_collapsed |
| Phenotype | NoPhenotypeAvailable |
| Upregulated in class | na\_pos |
| GeneSet | SANA\_TNFA\_ENDOTHELIAL\_UP |
| Enrichment Score (ES) | 0.69043696 |
| Normalized Enrichment Score (NES) | 2.0241473 |
| Nominal p-value | 0.0 |
| FDR q-value | 0.0 |
| FWER p-Value | 0.0 |
Table: GSEA Results Summary

  

Fig 1: Enrichment plot: SANA\_TNFA\_ENDOTHELIAL\_UP      
 Profile of the Running ES Score & Positions of GeneSet Members on the Rank Ordered List

  

| PROBE | GENE SYMBOL | GENE\_TITLE | RANK IN GENE LIST | RANK METRIC SCORE | RUNNING ES | CORE ENRICHMENT || 1 | CCL2 |  |  | 1 | 1.308 | 0.0551 | Yes |
| 2 | TNFAIP2 |  |  | 22 | 0.790 | 0.0874 | Yes |
| 3 | MX1 |  |  | 24 | 0.773 | 0.1199 | Yes |
| 4 | HLA-B |  |  | 41 | 0.695 | 0.1484 | Yes |
| 5 | HLA-C |  |  | 57 | 0.650 | 0.1751 | Yes |
| 6 | LGALS3BP |  |  | 61 | 0.643 | 0.2020 | Yes |
| 7 | IL8 |  |  | 76 | 0.611 | 0.2271 | Yes |
| 8 | TAPBP |  |  | 89 | 0.595 | 0.2516 | Yes |
| 9 | CX3CL1 |  |  | 94 | 0.586 | 0.2761 | Yes |
| 10 | LGALS9 |  |  | 95 | 0.585 | 0.3008 | Yes |
| 11 | HLA-E |  |  | 126 | 0.545 | 0.3223 | Yes |
| 12 | GBP1 |  |  | 130 | 0.542 | 0.3450 | Yes |
| 13 | SAT1 |  |  | 137 | 0.536 | 0.3673 | Yes |
| 14 | SOD2 |  |  | 150 | 0.523 | 0.3887 | Yes |
| 15 | TNIP1 |  |  | 165 | 0.515 | 0.4097 | Yes |
| 16 | HLA-A |  |  | 195 | 0.496 | 0.4292 | Yes |
| 17 | CXCL11 |  |  | 228 | 0.479 | 0.4478 | Yes |
| 18 | OAS1 |  |  | 255 | 0.466 | 0.4662 | Yes |
| 19 | PARP14 |  |  | 291 | 0.455 | 0.4837 | Yes |
| 20 | CCL20 |  |  | 301 | 0.453 | 0.5023 | Yes |
| 21 | TNFAIP3 |  |  | 406 | 0.424 | 0.5151 | Yes |
| 22 | ICAM1 |  |  | 408 | 0.423 | 0.5329 | Yes |
| 23 | CXCL3 |  |  | 427 | 0.419 | 0.5497 | Yes |
| 24 | CXCL2 |  |  | 457 | 0.412 | 0.5656 | Yes |
| 25 | C1QTNF1 |  |  | 483 | 0.408 | 0.5816 | Yes |
| 26 | FTH1 |  |  | 495 | 0.407 | 0.5982 | Yes |
| 27 | INHBA |  |  | 615 | 0.382 | 0.6085 | Yes |
| 28 | APOL3 |  |  | 632 | 0.378 | 0.6237 | Yes |
| 29 | BST2 |  |  | 657 | 0.374 | 0.6383 | Yes |
| 30 | C1S |  |  | 714 | 0.366 | 0.6510 | Yes |
| 31 | CASP1 |  |  | 732 | 0.363 | 0.6654 | Yes |
| 32 | FLJ20035 |  |  | 753 | 0.359 | 0.6796 | Yes |
| 33 | SLC15A3 |  |  | 1009 | 0.331 | 0.6811 | Yes |
| 34 | IL32 |  |  | 1685 | 0.274 | 0.6597 | Yes |
| 35 | OAS3 |  |  | 2108 | 0.250 | 0.6497 | Yes |
| 36 | BIRC3 |  |  | 2126 | 0.250 | 0.6594 | Yes |
| 37 | SAMHD1 |  |  | 2149 | 0.248 | 0.6688 | Yes |
| 38 | BPGM |  |  | 2267 | 0.242 | 0.6733 | Yes |
| 39 | OXR1 |  |  | 2299 | 0.240 | 0.6819 | Yes |
| 40 | IFIT1 |  |  | 2486 | 0.231 | 0.6826 | Yes |
| 41 | AADACL1 |  |  | 2589 | 0.227 | 0.6871 | Yes |
| 42 | DNAJA1 |  |  | 2804 | 0.218 | 0.6859 | Yes |
| 43 | SERPINE1 |  |  | 3043 | 0.209 | 0.6831 | Yes |
| 44 | ICOSLG |  |  | 3073 | 0.208 | 0.6904 | Yes |
| 45 | IFIH1 |  |  | 3449 | 0.195 | 0.6804 | No |
| 46 | CSF1 |  |  | 3752 | 0.187 | 0.6735 | No |
| 47 | ITGAV |  |  | 4016 | 0.179 | 0.6683 | No |
| 48 | RHOB |  |  | 4540 | 0.166 | 0.6498 | No |
| 49 | ATP13A3 |  |  | 4709 | 0.162 | 0.6484 | No |
| 50 | MMP10 |  |  | 5268 | 0.150 | 0.6275 | No |
| 51 | SMAD3 |  |  | 5619 | 0.142 | 0.6164 | No |
| 52 | LAMB3 |  |  | 5853 | 0.138 | 0.6109 | No |
| 53 | IL7R |  |  | 5986 | 0.135 | 0.6102 | No |
| 54 | SSPN |  |  | 6231 | 0.131 | 0.6038 | No |
| 55 | MULK |  |  | 6256 | 0.131 | 0.6081 | No |
| 56 | APOL1 |  |  | 6770 | 0.122 | 0.5883 | No |
| 57 | RAC3 |  |  | 6826 | 0.121 | 0.5907 | No |
| 58 | DHRS8 |  |  | 7169 | 0.115 | 0.5789 | No |
| 59 | DRAM |  |  | 7346 | 0.112 | 0.5750 | No |
| 60 | NFKBIA |  |  | 7714 | 0.106 | 0.5616 | No |
| 61 | CXCR7 |  |  | 7786 | 0.105 | 0.5626 | No |
| 62 | CXCL10 |  |  | 10182 | 0.071 | 0.4489 | No |
| 63 | TMEM16J |  |  | 10401 | 0.067 | 0.4411 | No |
| 64 | CCL8 |  |  | 10622 | 0.064 | 0.4331 | No |
| 65 | SPAG9 |  |  | 11397 | 0.055 | 0.3977 | No |
| 66 | SAMD9L |  |  | 12059 | 0.046 | 0.3675 | No |
| 67 | OAS2 |  |  | 12543 | 0.040 | 0.3456 | No |
| 68 | SLC7A2 |  |  | 13514 | 0.027 | 0.2995 | No |
| 69 | VCAM1 |  |  | 13537 | 0.027 | 0.2996 | No |
| 70 | CXCL6 |  |  | 14050 | 0.020 | 0.2755 | No |
| 71 | IFI44L |  |  | 14255 | 0.017 | 0.2663 | No |
| 72 | TLR2 |  |  | 14880 | 0.008 | 0.2362 | No |
| 73 | MMP3 |  |  | 15163 | 0.003 | 0.2226 | No |
| 74 | CCL11 |  |  | 17002 | -0.029 | 0.1343 | No |
| 75 | RIPK2 |  |  | 17192 | -0.033 | 0.1264 | No |
| 76 | CCL7 |  |  | 19223 | -0.094 | 0.0315 | No |
| 77 | MX2 |  |  | 19725 | -0.121 | 0.0122 | No |
| 78 | PLA1A |  |  | 20000 | -0.144 | 0.0049 | No |
| 79 | CTHRC1 |  |  | 20475 | -0.274 | -0.0066 | No |
| 80 | LIPG |  |  | 20501 | -0.306 | 0.0051 | No |
Table: GSEA details [plain text format]

  

Fig 2: SANA\_TNFA\_ENDOTHELIAL\_UP: Random ES distribution      
 Gene set null distribution of ES for **SANA\_TNFA\_ENDOTHELIAL\_UP**
