# Supplementary material for: The rapamycin-regulated gene expression signature determines prognosis for breast cancer
Source: Mol Cancer. 2009 Sep 24;8:75. doi: 10.1186/1476-4598-8-75 (PMC2761377; doi:10.1186/1476-4598-8-75)
Supplement: Additional file 3 — Gene set enrichment analysis of in vivo data, treatment series. The data provided represent the treatment series of GSEA. This compressed file contains "Treatment" shortcut file and "GSEA_treatment" folder. Clicking on "Treatment" shortcut opens the index file providing access to analysis files contained in the "GSEA_treatment" folder. [file 1476-4598-8-75-S3.zip › GSEA_treatment/SCHURINGA_STAT5A_UP.html]

Details for gene set SCHURINGA\_STAT5A\_UP[GSEA]

|  || Dataset | gsea\_treatment\_collapsed |
| Phenotype | NoPhenotypeAvailable |
| Upregulated in class | na\_neg |
| GeneSet | SCHURINGA\_STAT5A\_UP |
| Enrichment Score (ES) | -0.32156613 |
| Normalized Enrichment Score (NES) | -1.2905928 |
| Nominal p-value | 0.12962963 |
| FDR q-value | 0.26342446 |
| FWER p-Value | 1.0 |
Table: GSEA Results Summary

  

Fig 1: Enrichment plot: SCHURINGA\_STAT5A\_UP      
 Profile of the Running ES Score & Positions of GeneSet Members on the Rank Ordered List

  

| PROBE | GENE SYMBOL | GENE\_TITLE | RANK IN GENE LIST | RANK METRIC SCORE | RUNNING ES | CORE ENRICHMENT || 1 | MAOA |  |  | 305 | 0.451 | 0.1076 | No |
| 2 | LEPR |  |  | 1190 | 0.312 | 0.1494 | No |
| 3 | TRAF4 |  |  | 2072 | 0.252 | 0.1751 | No |
| 4 | HBE1 |  |  | 2211 | 0.245 | 0.2349 | No |
| 5 | PIM1 |  |  | 3137 | 0.205 | 0.2458 | No |
| 6 | CISH |  |  | 6034 | 0.135 | 0.1417 | No |
| 7 | ANK1 |  |  | 6189 | 0.132 | 0.1700 | No |
| 8 | RAC3 |  |  | 6826 | 0.121 | 0.1720 | No |
| 9 | GYPA |  |  | 7231 | 0.114 | 0.1834 | No |
| 10 | SOS1 |  |  | 7688 | 0.107 | 0.1903 | No |
| 11 | CD36 |  |  | 12917 | 0.035 | -0.0542 | No |
| 12 | HBZ |  |  | 13283 | 0.030 | -0.0637 | No |
| 13 | FOSB |  |  | 15095 | 0.004 | -0.1505 | No |
| 14 | PDAP1 |  |  | 15357 | 0.000 | -0.1632 | No |
| 15 | RHAG |  |  | 17137 | -0.031 | -0.2411 | No |
| 16 | LIF |  |  | 17567 | -0.041 | -0.2507 | No |
| 17 | GYPB |  |  | 18670 | -0.071 | -0.2849 | Yes |
| 18 | HBG1 |  |  | 18910 | -0.081 | -0.2744 | Yes |
| 19 | AQP3 |  |  | 19019 | -0.085 | -0.2566 | Yes |
| 20 | XK |  |  | 20358 | -0.208 | -0.2650 | Yes |
| 21 | VEGF |  |  | 20367 | -0.211 | -0.2080 | Yes |
| 22 | HBA1 |  |  | 20507 | -0.311 | -0.1302 | Yes |
| 23 | HBA2 |  |  | 20572 | -0.496 | 0.0016 | Yes |
Table: GSEA details [plain text format]

  

Fig 2: SCHURINGA\_STAT5A\_UP: Random ES distribution      
 Gene set null distribution of ES for **SCHURINGA\_STAT5A\_UP**
