# Supplementary material for: The rapamycin-regulated gene expression signature determines prognosis for breast cancer
Source: Mol Cancer. 2009 Sep 24;8:75. doi: 10.1186/1476-4598-8-75 (PMC2761377; doi:10.1186/1476-4598-8-75)
Supplement: Additional file 3 — Gene set enrichment analysis of in vivo data, treatment series. The data provided represent the treatment series of GSEA. This compressed file contains "Treatment" shortcut file and "GSEA_treatment" folder. Clicking on "Treatment" shortcut opens the index file providing access to analysis files contained in the "GSEA_treatment" folder. [file 1476-4598-8-75-S3.zip › GSEA_treatment/STOSSI_ER_UP.html]

Details for gene set STOSSI\_ER\_UP[GSEA]

|  || Dataset | gsea\_treatment\_collapsed |
| Phenotype | NoPhenotypeAvailable |
| Upregulated in class | na\_neg |
| GeneSet | STOSSI\_ER\_UP |
| Enrichment Score (ES) | -0.21495277 |
| Normalized Enrichment Score (NES) | -0.92630285 |
| Nominal p-value | 0.5555556 |
| FDR q-value | 0.67463815 |
| FWER p-Value | 1.0 |
Table: GSEA Results Summary

  

Fig 1: Enrichment plot: STOSSI\_ER\_UP      
 Profile of the Running ES Score & Positions of GeneSet Members on the Rank Ordered List

  

| PROBE | GENE SYMBOL | GENE\_TITLE | RANK IN GENE LIST | RANK METRIC SCORE | RUNNING ES | CORE ENRICHMENT || 1 | IL8 |  |  | 76 | 0.611 | 0.1050 | Yes |
| 2 | SERPINA6 |  |  | 85 | 0.603 | 0.2118 | Yes |
| 3 | BIRC3 |  |  | 2126 | 0.250 | 0.1569 | No |
| 4 | TLE3 |  |  | 2304 | 0.240 | 0.1910 | No |
| 5 | RASGRF1 |  |  | 3648 | 0.189 | 0.1594 | No |
| 6 | LRIG1 |  |  | 4264 | 0.173 | 0.1601 | No |
| 7 | ADK |  |  | 4336 | 0.171 | 0.1870 | No |
| 8 | ITGA6 |  |  | 4923 | 0.157 | 0.1865 | No |
| 9 | MSMB |  |  | 6177 | 0.132 | 0.1491 | No |
| 10 | SEPP1 |  |  | 6864 | 0.121 | 0.1372 | No |
| 11 | PPP1R12B |  |  | 6940 | 0.119 | 0.1547 | No |
| 12 | GK3P |  |  | 6972 | 0.118 | 0.1742 | No |
| 13 | NRIP1 |  |  | 8204 | 0.099 | 0.1319 | No |
| 14 | SKIL |  |  | 8583 | 0.093 | 0.1300 | No |
| 15 | GREB1 |  |  | 8900 | 0.088 | 0.1303 | No |
| 16 | CDH19 |  |  | 9450 | 0.081 | 0.1179 | No |
| 17 | RALA |  |  | 10270 | 0.069 | 0.0904 | No |
| 18 | PDZK1 |  |  | 10344 | 0.068 | 0.0990 | No |
| 19 | GJA1 |  |  | 12062 | 0.046 | 0.0236 | No |
| 20 | IL24 |  |  | 12530 | 0.040 | 0.0080 | No |
| 21 | ACOX2 |  |  | 12536 | 0.040 | 0.0148 | No |
| 22 | ADAM3A |  |  | 12867 | 0.036 | 0.0051 | No |
| 23 | HLXB9 |  |  | 13970 | 0.021 | -0.0447 | No |
| 24 | BMP6 |  |  | 14332 | 0.016 | -0.0595 | No |
| 25 | NTS |  |  | 14662 | 0.011 | -0.0735 | No |
| 26 | RHOBTB3 |  |  | 14685 | 0.011 | -0.0727 | No |
| 27 | WISP2 |  |  | 14771 | 0.010 | -0.0751 | No |
| 28 | CA12 |  |  | 15493 | -0.002 | -0.1098 | No |
| 29 | PTGS2 |  |  | 16374 | -0.017 | -0.1496 | No |
| 30 | HYAL1 |  |  | 17320 | -0.036 | -0.1893 | No |
| 31 | PTHLH |  |  | 17572 | -0.041 | -0.1941 | No |
| 32 | AGT |  |  | 17814 | -0.047 | -0.1975 | No |
| 33 | GRK5 |  |  | 17915 | -0.050 | -0.1935 | No |
| 34 | KRT17 |  |  | 17959 | -0.050 | -0.1866 | No |
| 35 | KLRC3 |  |  | 18420 | -0.063 | -0.1977 | No |
| 36 | TCN1 |  |  | 18499 | -0.065 | -0.1899 | No |
| 37 | IL13RA2 |  |  | 18562 | -0.067 | -0.1810 | No |
| 38 | NR4A3 |  |  | 19262 | -0.096 | -0.1979 | No |
| 39 | F13A1 |  |  | 19310 | -0.099 | -0.1826 | No |
| 40 | SPARCL1 |  |  | 19437 | -0.105 | -0.1700 | No |
| 41 | CD34 |  |  | 19470 | -0.107 | -0.1526 | No |
| 42 | KLRK1 |  |  | 19783 | -0.126 | -0.1455 | No |
| 43 | PLA2G4A |  |  | 20153 | -0.164 | -0.1342 | No |
| 44 | TFF1 |  |  | 20162 | -0.166 | -0.1051 | No |
| 45 | SLC9A3R1 |  |  | 20316 | -0.199 | -0.0773 | No |
| 46 | OPHN1 |  |  | 20417 | -0.232 | -0.0409 | No |
| 47 | DEFB1 |  |  | 20482 | -0.281 | 0.0060 | No |
Table: GSEA details [plain text format]

  

Fig 2: STOSSI\_ER\_UP: Random ES distribution      
 Gene set null distribution of ES for **STOSSI\_ER\_UP**
