# Supplementary material for: The rapamycin-regulated gene expression signature determines prognosis for breast cancer
Source: Mol Cancer. 2009 Sep 24;8:75. doi: 10.1186/1476-4598-8-75 (PMC2761377; doi:10.1186/1476-4598-8-75)
Supplement: Additional file 3 — Gene set enrichment analysis of in vivo data, treatment series. The data provided represent the treatment series of GSEA. This compressed file contains "Treatment" shortcut file and "GSEA_treatment" folder. Clicking on "Treatment" shortcut opens the index file providing access to analysis files contained in the "GSEA_treatment" folder. [file 1476-4598-8-75-S3.zip › GSEA_treatment/STRIATED_MUSCLE_CONTRACTION.html]

Details for gene set STRIATED\_MUSCLE\_CONTRACTION[GSEA]

|  || Dataset | gsea\_treatment\_collapsed |
| Phenotype | NoPhenotypeAvailable |
| Upregulated in class | na\_neg |
| GeneSet | STRIATED\_MUSCLE\_CONTRACTION |
| Enrichment Score (ES) | -0.31692436 |
| Normalized Enrichment Score (NES) | -1.2961552 |
| Nominal p-value | 0.08510638 |
| FDR q-value | 0.2758484 |
| FWER p-Value | 0.999 |
Table: GSEA Results Summary

  

Fig 1: Enrichment plot: STRIATED\_MUSCLE\_CONTRACTION      
 Profile of the Running ES Score & Positions of GeneSet Members on the Rank Ordered List

  

| PROBE | GENE SYMBOL | GENE\_TITLE | RANK IN GENE LIST | RANK METRIC SCORE | RUNNING ES | CORE ENRICHMENT || 1 | ACTA2 |  |  | 1058 | 0.325 | 0.0228 | No |
| 2 | ACTN4 |  |  | 1631 | 0.277 | 0.0582 | No |
| 3 | TPM4 |  |  | 2682 | 0.224 | 0.0583 | No |
| 4 | TPM3 /// TPM4 |  |  | 3229 | 0.202 | 0.0778 | No |
| 5 | MYBPC1 |  |  | 3296 | 0.200 | 0.1203 | No |
| 6 | TPM1 |  |  | 3849 | 0.184 | 0.1354 | No |
| 7 | MYH3 |  |  | 3857 | 0.184 | 0.1770 | No |
| 8 | DMD |  |  | 7222 | 0.114 | 0.0395 | No |
| 9 | TTN |  |  | 7875 | 0.104 | 0.0315 | No |
| 10 | ACTN2 |  |  | 8050 | 0.101 | 0.0461 | No |
| 11 | MYH6 /// MYH7 |  |  | 8623 | 0.092 | 0.0394 | No |
| 12 | MYL4 |  |  | 8971 | 0.087 | 0.0424 | No |
| 13 | ACTN3 |  |  | 9607 | 0.078 | 0.0295 | No |
| 14 | MYOM1 |  |  | 10176 | 0.071 | 0.0180 | No |
| 15 | DES /// FAM48A |  |  | 11102 | 0.058 | -0.0137 | No |
| 16 | MYBPC3 |  |  | 11385 | 0.055 | -0.0149 | No |
| 17 | MYL9 |  |  | 12410 | 0.041 | -0.0552 | No |
| 18 | MYBPC2 |  |  | 13239 | 0.031 | -0.0885 | No |
| 19 | TNNT2 |  |  | 13404 | 0.029 | -0.0898 | No |
| 20 | DES |  |  | 13511 | 0.027 | -0.0887 | No |
| 21 | TNNT1 |  |  | 14123 | 0.019 | -0.1142 | No |
| 22 | TNNI3 |  |  | 14178 | 0.018 | -0.1126 | No |
| 23 | MYL3 |  |  | 14356 | 0.015 | -0.1177 | No |
| 24 | TCAP |  |  | 14566 | 0.013 | -0.1250 | No |
| 25 | TPM2 |  |  | 15180 | 0.003 | -0.1540 | No |
| 26 | NEB |  |  | 16605 | -0.021 | -0.2184 | No |
| 27 | TNNT3 |  |  | 16723 | -0.024 | -0.2187 | No |
| 28 | MYH6 |  |  | 17793 | -0.047 | -0.2601 | No |
| 29 | TNNI1 |  |  | 18043 | -0.053 | -0.2601 | No |
| 30 | MYH8 |  |  | 18214 | -0.057 | -0.2553 | No |
| 31 | TMOD1 |  |  | 18229 | -0.058 | -0.2428 | No |
| 32 | TNNI2 |  |  | 18920 | -0.082 | -0.2577 | No |
| 33 | MYL2 |  |  | 20140 | -0.163 | -0.2798 | Yes |
| 34 | VIM |  |  | 20275 | -0.188 | -0.2434 | Yes |
| 35 | MYL1 |  |  | 20484 | -0.284 | -0.1887 | Yes |
| 36 | TNNC2 |  |  | 20554 | -0.425 | -0.0951 | Yes |
| 37 | ACTA1 |  |  | 20555 | -0.427 | 0.0024 | Yes |
Table: GSEA details [plain text format]

  

Fig 2: STRIATED\_MUSCLE\_CONTRACTION: Random ES distribution      
 Gene set null distribution of ES for **STRIATED\_MUSCLE\_CONTRACTION**
