# Supplementary material for: The rapamycin-regulated gene expression signature determines prognosis for breast cancer
Source: Mol Cancer. 2009 Sep 24;8:75. doi: 10.1186/1476-4598-8-75 (PMC2761377; doi:10.1186/1476-4598-8-75)
Supplement: Additional file 3 — Gene set enrichment analysis of in vivo data, treatment series. The data provided represent the treatment series of GSEA. This compressed file contains "Treatment" shortcut file and "GSEA_treatment" folder. Clicking on "Treatment" shortcut opens the index file providing access to analysis files contained in the "GSEA_treatment" folder. [file 1476-4598-8-75-S3.zip › GSEA_treatment/TAVOR_CEBP_DN.html]

Details for gene set TAVOR\_CEBP\_DN[GSEA]

|  || Dataset | gsea\_treatment\_collapsed |
| Phenotype | NoPhenotypeAvailable |
| Upregulated in class | na\_pos |
| GeneSet | TAVOR\_CEBP\_DN |
| Enrichment Score (ES) | 0.72374254 |
| Normalized Enrichment Score (NES) | 1.9419627 |
| Nominal p-value | 0.0 |
| FDR q-value | 4.1131987E-4 |
| FWER p-Value | 0.0050 |
Table: GSEA Results Summary

  

Fig 1: Enrichment plot: TAVOR\_CEBP\_DN      
 Profile of the Running ES Score & Positions of GeneSet Members on the Rank Ordered List

  

| PROBE | GENE SYMBOL | GENE\_TITLE | RANK IN GENE LIST | RANK METRIC SCORE | RUNNING ES | CORE ENRICHMENT || 1 | IFI27 |  |  | 32 | 0.725 | 0.0978 | Yes |
| 2 | H1FX |  |  | 40 | 0.696 | 0.1927 | Yes |
| 3 | HLA-B |  |  | 41 | 0.695 | 0.2879 | Yes |
| 4 | ISG15 |  |  | 59 | 0.644 | 0.3753 | Yes |
| 5 | MYC |  |  | 75 | 0.615 | 0.4588 | Yes |
| 6 | TNFSF10 |  |  | 160 | 0.518 | 0.5256 | Yes |
| 7 | SERPINB1 |  |  | 169 | 0.511 | 0.5952 | Yes |
| 8 | IFI6 |  |  | 726 | 0.364 | 0.6180 | Yes |
| 9 | ITGB5 |  |  | 1080 | 0.323 | 0.6451 | Yes |
| 10 | PLOD2 |  |  | 1140 | 0.316 | 0.6856 | Yes |
| 11 | AKR1B1 |  |  | 1224 | 0.308 | 0.7237 | Yes |
| 12 | ZBTB48 |  |  | 3119 | 0.206 | 0.6599 | No |
| 13 | GAL |  |  | 3317 | 0.200 | 0.6776 | No |
| 14 | A2M |  |  | 7877 | 0.104 | 0.4703 | No |
| 15 | CRADD |  |  | 8045 | 0.101 | 0.4760 | No |
| 16 | RIN1 |  |  | 9535 | 0.079 | 0.4145 | No |
| 17 | MC1R |  |  | 9859 | 0.076 | 0.4091 | No |
| 18 | FXR2 |  |  | 9871 | 0.075 | 0.4189 | No |
| 19 | GP9 |  |  | 10346 | 0.068 | 0.4052 | No |
| 20 | THBD |  |  | 10744 | 0.063 | 0.3945 | No |
| 21 | TBXAS1 |  |  | 11971 | 0.047 | 0.3414 | No |
| 22 | GJA1 |  |  | 12062 | 0.046 | 0.3433 | No |
| 23 | OAS2 |  |  | 12543 | 0.040 | 0.3254 | No |
| 24 | FOXO1A |  |  | 14214 | 0.018 | 0.2467 | No |
| 25 | LYL1 |  |  | 14844 | 0.009 | 0.2173 | No |
| 26 | GATA2 |  |  | 14848 | 0.008 | 0.2183 | No |
| 27 | SULT1A3 |  |  | 17234 | -0.034 | 0.1069 | No |
| 28 | MMRN1 |  |  | 17925 | -0.050 | 0.0803 | No |
| 29 | ICAM2 |  |  | 18088 | -0.054 | 0.0798 | No |
| 30 | KLRK1 |  |  | 19783 | -0.126 | 0.0146 | No |
| 31 | PFN2 |  |  | 20256 | -0.185 | 0.0170 | No |
Table: GSEA details [plain text format]

  

Fig 2: TAVOR\_CEBP\_DN: Random ES distribution      
 Gene set null distribution of ES for **TAVOR\_CEBP\_DN**
