# Supplementary material for: The rapamycin-regulated gene expression signature determines prognosis for breast cancer
Source: Mol Cancer. 2009 Sep 24;8:75. doi: 10.1186/1476-4598-8-75 (PMC2761377; doi:10.1186/1476-4598-8-75)
Supplement: Additional file 3 — Gene set enrichment analysis of in vivo data, treatment series. The data provided represent the treatment series of GSEA. This compressed file contains "Treatment" shortcut file and "GSEA_treatment" folder. Clicking on "Treatment" shortcut opens the index file providing access to analysis files contained in the "GSEA_treatment" folder. [file 1476-4598-8-75-S3.zip › GSEA_treatment/TGFBETA_ALL_UP.html]

Details for gene set TGFBETA\_ALL\_UP[GSEA]

|  || Dataset | gsea\_treatment\_collapsed |
| Phenotype | NoPhenotypeAvailable |
| Upregulated in class | na\_pos |
| GeneSet | TGFBETA\_ALL\_UP |
| Enrichment Score (ES) | 0.5749683 |
| Normalized Enrichment Score (NES) | 1.6992923 |
| Nominal p-value | 0.0 |
| FDR q-value | 0.012105382 |
| FWER p-Value | 0.417 |
Table: GSEA Results Summary

  

Fig 1: Enrichment plot: TGFBETA\_ALL\_UP      
 Profile of the Running ES Score & Positions of GeneSet Members on the Rank Ordered List

  

| PROBE | GENE SYMBOL | GENE\_TITLE | RANK IN GENE LIST | RANK METRIC SCORE | RUNNING ES | CORE ENRICHMENT || 1 | IGFBP5 |  |  | 26 | 0.757 | 0.0504 | Yes |
| 2 | CD59 |  |  | 86 | 0.600 | 0.0884 | Yes |
| 3 | MMP2 |  |  | 124 | 0.548 | 0.1239 | Yes |
| 4 | PLAT |  |  | 132 | 0.541 | 0.1605 | Yes |
| 5 | BSG |  |  | 267 | 0.461 | 0.1854 | Yes |
| 6 | ICAM1 |  |  | 408 | 0.423 | 0.2074 | Yes |
| 7 | MARCKSL1 |  |  | 413 | 0.423 | 0.2360 | Yes |
| 8 | LAMB1 |  |  | 498 | 0.405 | 0.2596 | Yes |
| 9 | LAMA4 |  |  | 560 | 0.392 | 0.2833 | Yes |
| 10 | IGFBP2 |  |  | 575 | 0.390 | 0.3093 | Yes |
| 11 | ZYX |  |  | 595 | 0.387 | 0.3347 | Yes |
| 12 | ARHGDIA |  |  | 763 | 0.358 | 0.3510 | Yes |
| 13 | RHOQ |  |  | 952 | 0.336 | 0.3647 | Yes |
| 14 | ITGB5 |  |  | 1080 | 0.323 | 0.3805 | Yes |
| 15 | ARHGAP1 |  |  | 1161 | 0.315 | 0.3981 | Yes |
| 16 | RAC1 |  |  | 1254 | 0.306 | 0.4145 | Yes |
| 17 | TIMP1 |  |  | 1349 | 0.298 | 0.4302 | Yes |
| 18 | SPARC |  |  | 1676 | 0.274 | 0.4329 | Yes |
| 19 | ITGA3 |  |  | 1816 | 0.265 | 0.4443 | Yes |
| 20 | JUP |  |  | 1878 | 0.262 | 0.4592 | Yes |
| 21 | THBS1 |  |  | 2263 | 0.242 | 0.4569 | Yes |
| 22 | SEMA3F |  |  | 2450 | 0.232 | 0.4637 | Yes |
| 23 | ITGB2 |  |  | 2545 | 0.228 | 0.4747 | Yes |
| 24 | NEO1 |  |  | 2635 | 0.225 | 0.4857 | Yes |
| 25 | FGD1 |  |  | 2832 | 0.217 | 0.4909 | Yes |
| 26 | SERPINE1 |  |  | 3043 | 0.209 | 0.4949 | Yes |
| 27 | CTNNB1 |  |  | 3054 | 0.208 | 0.5086 | Yes |
| 28 | ITGB8 |  |  | 3125 | 0.206 | 0.5192 | Yes |
| 29 | TNC |  |  | 3191 | 0.203 | 0.5299 | Yes |
| 30 | PAK2 |  |  | 3200 | 0.203 | 0.5433 | Yes |
| 31 | PXN |  |  | 3423 | 0.196 | 0.5459 | Yes |
| 32 | DVL3 |  |  | 3507 | 0.193 | 0.5550 | Yes |
| 33 | COL6A1 |  |  | 3686 | 0.189 | 0.5592 | Yes |
| 34 | MMP14 |  |  | 3717 | 0.188 | 0.5705 | Yes |
| 35 | RHOA |  |  | 4320 | 0.171 | 0.5529 | Yes |
| 36 | RHOB |  |  | 4540 | 0.166 | 0.5535 | Yes |
| 37 | SMO |  |  | 4600 | 0.164 | 0.5618 | Yes |
| 38 | EPHB2 |  |  | 4740 | 0.161 | 0.5660 | Yes |
| 39 | NOTCH2 |  |  | 5049 | 0.154 | 0.5615 | Yes |
| 40 | TCF7L2 |  |  | 5086 | 0.153 | 0.5702 | Yes |
| 41 | DVL1 |  |  | 5610 | 0.142 | 0.5544 | Yes |
| 42 | MMP11 |  |  | 5663 | 0.141 | 0.5615 | Yes |
| 43 | HSPG2 |  |  | 5670 | 0.141 | 0.5709 | Yes |
| 44 | MTA1 |  |  | 5781 | 0.139 | 0.5750 | Yes |
| 45 | RHOG |  |  | 6070 | 0.134 | 0.5701 | No |
| 46 | ITGA5 |  |  | 7140 | 0.115 | 0.5259 | No |
| 47 | TNFRSF1A |  |  | 7581 | 0.108 | 0.5118 | No |
| 48 | COL8A1 |  |  | 8624 | 0.092 | 0.4673 | No |
| 49 | EPHB4 |  |  | 8866 | 0.088 | 0.4616 | No |
| 50 | LRP1 |  |  | 9113 | 0.085 | 0.4554 | No |
| 51 | IGF2R |  |  | 9501 | 0.080 | 0.4420 | No |
| 52 | ITGB3 |  |  | 9633 | 0.078 | 0.4410 | No |
| 53 | ITGA4 |  |  | 11022 | 0.059 | 0.3774 | No |
| 54 | RND3 |  |  | 11199 | 0.057 | 0.3727 | No |
| 55 | CDH6 |  |  | 11540 | 0.053 | 0.3597 | No |
| 56 | IGFBP4 |  |  | 11759 | 0.050 | 0.3525 | No |
| 57 | FN1 |  |  | 11951 | 0.047 | 0.3464 | No |
| 58 | IGFBP3 |  |  | 12384 | 0.042 | 0.3282 | No |
| 59 | CTNNA1 |  |  | 12390 | 0.042 | 0.3308 | No |
| 60 | MMP17 |  |  | 12557 | 0.039 | 0.3254 | No |
| 61 | ADAM9 |  |  | 12576 | 0.039 | 0.3272 | No |
| 62 | PAK1 |  |  | 12893 | 0.035 | 0.3142 | No |
| 63 | WNT2B |  |  | 13532 | 0.027 | 0.2850 | No |
| 64 | EFNA5 |  |  | 14111 | 0.019 | 0.2581 | No |
| 65 | CD44 |  |  | 14567 | 0.013 | 0.2368 | No |
| 66 | MMP3 |  |  | 15163 | 0.003 | 0.2080 | No |
| 67 | MMP1 |  |  | 16811 | -0.025 | 0.1295 | No |
| 68 | EPHA2 |  |  | 16925 | -0.027 | 0.1258 | No |
| 69 | COL16A1 |  |  | 17177 | -0.033 | 0.1158 | No |
| 70 | NID1 |  |  | 17467 | -0.039 | 0.1044 | No |
| 71 | CSPG2 |  |  | 17601 | -0.042 | 0.1008 | No |
| 72 | DSP |  |  | 18062 | -0.053 | 0.0820 | No |
| 73 | COL1A2 |  |  | 18136 | -0.055 | 0.0822 | No |
| 74 | WNT8B |  |  | 18148 | -0.055 | 0.0855 | No |
| 75 | MMP19 |  |  | 18609 | -0.069 | 0.0677 | No |
| 76 | COL6A3 |  |  | 18784 | -0.076 | 0.0644 | No |
| 77 | LRRC17 |  |  | 18837 | -0.078 | 0.0672 | No |
| 78 | COL3A1 |  |  | 19040 | -0.086 | 0.0632 | No |
| 79 | EPHB3 |  |  | 19077 | -0.088 | 0.0674 | No |
| 80 | THBS2 |  |  | 19385 | -0.102 | 0.0594 | No |
Table: GSEA details [plain text format]

  

Fig 2: TGFBETA\_ALL\_UP: Random ES distribution      
 Gene set null distribution of ES for **TGFBETA\_ALL\_UP**
