# Supplementary material for: The rapamycin-regulated gene expression signature determines prognosis for breast cancer
Source: Mol Cancer. 2009 Sep 24;8:75. doi: 10.1186/1476-4598-8-75 (PMC2761377; doi:10.1186/1476-4598-8-75)
Supplement: Additional file 3 — Gene set enrichment analysis of in vivo data, treatment series. The data provided represent the treatment series of GSEA. This compressed file contains "Treatment" shortcut file and "GSEA_treatment" folder. Clicking on "Treatment" shortcut opens the index file providing access to analysis files contained in the "GSEA_treatment" folder. [file 1476-4598-8-75-S3.zip › GSEA_treatment/TGFBETA_EARLY_UP.html]

Details for gene set TGFBETA\_EARLY\_UP[GSEA]

|  || Dataset | gsea\_treatment\_collapsed |
| Phenotype | NoPhenotypeAvailable |
| Upregulated in class | na\_pos |
| GeneSet | TGFBETA\_EARLY\_UP |
| Enrichment Score (ES) | 0.6056075 |
| Normalized Enrichment Score (NES) | 1.7126486 |
| Nominal p-value | 0.0 |
| FDR q-value | 0.010190245 |
| FWER p-Value | 0.346 |
Table: GSEA Results Summary

  

Fig 1: Enrichment plot: TGFBETA\_EARLY\_UP      
 Profile of the Running ES Score & Positions of GeneSet Members on the Rank Ordered List

  

| PROBE | GENE SYMBOL | GENE\_TITLE | RANK IN GENE LIST | RANK METRIC SCORE | RUNNING ES | CORE ENRICHMENT || 1 | IGFBP5 |  |  | 26 | 0.757 | 0.0778 | Yes |
| 2 | CD59 |  |  | 86 | 0.600 | 0.1376 | Yes |
| 3 | PLAT |  |  | 132 | 0.541 | 0.1920 | Yes |
| 4 | BSG |  |  | 267 | 0.461 | 0.2336 | Yes |
| 5 | ICAM1 |  |  | 408 | 0.423 | 0.2710 | Yes |
| 6 | MARCKSL1 |  |  | 413 | 0.423 | 0.3149 | Yes |
| 7 | LAMA4 |  |  | 560 | 0.392 | 0.3488 | Yes |
| 8 | IGFBP2 |  |  | 575 | 0.390 | 0.3889 | Yes |
| 9 | ZYX |  |  | 595 | 0.387 | 0.4284 | Yes |
| 10 | ARHGDIA |  |  | 763 | 0.358 | 0.4577 | Yes |
| 11 | RHOQ |  |  | 952 | 0.336 | 0.4836 | Yes |
| 12 | ITGB5 |  |  | 1080 | 0.323 | 0.5111 | Yes |
| 13 | TIMP1 |  |  | 1349 | 0.298 | 0.5292 | Yes |
| 14 | SPARC |  |  | 1676 | 0.274 | 0.5419 | Yes |
| 15 | ITGA3 |  |  | 1816 | 0.265 | 0.5628 | Yes |
| 16 | JUP |  |  | 1878 | 0.262 | 0.5872 | Yes |
| 17 | ITGB2 |  |  | 2545 | 0.228 | 0.5787 | Yes |
| 18 | SERPINE1 |  |  | 3043 | 0.209 | 0.5763 | Yes |
| 19 | PXN |  |  | 3423 | 0.196 | 0.5783 | Yes |
| 20 | COL6A1 |  |  | 3686 | 0.189 | 0.5853 | Yes |
| 21 | MMP14 |  |  | 3717 | 0.188 | 0.6034 | Yes |
| 22 | RHOA |  |  | 4320 | 0.171 | 0.5920 | Yes |
| 23 | RHOB |  |  | 4540 | 0.166 | 0.5987 | Yes |
| 24 | NOTCH2 |  |  | 5049 | 0.154 | 0.5901 | Yes |
| 25 | TCF7L2 |  |  | 5086 | 0.153 | 0.6043 | Yes |
| 26 | DVL1 |  |  | 5610 | 0.142 | 0.5938 | Yes |
| 27 | HSPG2 |  |  | 5670 | 0.141 | 0.6056 | Yes |
| 28 | RHOG |  |  | 6070 | 0.134 | 0.6002 | No |
| 29 | LRP1 |  |  | 9113 | 0.085 | 0.4611 | No |
| 30 | IGF2R |  |  | 9501 | 0.080 | 0.4506 | No |
| 31 | RND3 |  |  | 11199 | 0.057 | 0.3741 | No |
| 32 | CDH6 |  |  | 11540 | 0.053 | 0.3631 | No |
| 33 | FN1 |  |  | 11951 | 0.047 | 0.3480 | No |
| 34 | IGFBP3 |  |  | 12384 | 0.042 | 0.3314 | No |
| 35 | ADAM9 |  |  | 12576 | 0.039 | 0.3262 | No |
| 36 | PAK1 |  |  | 12893 | 0.035 | 0.3145 | No |
| 37 | WNT2B |  |  | 13532 | 0.027 | 0.2863 | No |
| 38 | CD44 |  |  | 14567 | 0.013 | 0.2373 | No |
| 39 | MMP3 |  |  | 15163 | 0.003 | 0.2087 | No |
| 40 | NID1 |  |  | 17467 | -0.039 | 0.1008 | No |
| 41 | CSPG2 |  |  | 17601 | -0.042 | 0.0987 | No |
| 42 | COL1A2 |  |  | 18136 | -0.055 | 0.0785 | No |
| 43 | MMP19 |  |  | 18609 | -0.069 | 0.0627 | No |
| 44 | COL6A3 |  |  | 18784 | -0.076 | 0.0622 | No |
| 45 | LRRC17 |  |  | 18837 | -0.078 | 0.0678 | No |
| 46 | COL3A1 |  |  | 19040 | -0.086 | 0.0669 | No |
| 47 | EPHB3 |  |  | 19077 | -0.088 | 0.0743 | No |
Table: GSEA details [plain text format]

  

Fig 2: TGFBETA\_EARLY\_UP: Random ES distribution      
 Gene set null distribution of ES for **TGFBETA\_EARLY\_UP**
