# Supplementary material for: The rapamycin-regulated gene expression signature determines prognosis for breast cancer
Source: Mol Cancer. 2009 Sep 24;8:75. doi: 10.1186/1476-4598-8-75 (PMC2761377; doi:10.1186/1476-4598-8-75)
Supplement: Additional file 3 — Gene set enrichment analysis of in vivo data, treatment series. The data provided represent the treatment series of GSEA. This compressed file contains "Treatment" shortcut file and "GSEA_treatment" folder. Clicking on "Treatment" shortcut opens the index file providing access to analysis files contained in the "GSEA_treatment" folder. [file 1476-4598-8-75-S3.zip › GSEA_treatment/TNFALPHA_ALL_UP.html]

Details for gene set TNFALPHA\_ALL\_UP[GSEA]

|  || Dataset | gsea\_treatment\_collapsed |
| Phenotype | NoPhenotypeAvailable |
| Upregulated in class | na\_pos |
| GeneSet | TNFALPHA\_ALL\_UP |
| Enrichment Score (ES) | 0.60324883 |
| Normalized Enrichment Score (NES) | 1.7840966 |
| Nominal p-value | 0.0 |
| FDR q-value | 0.004043432 |
| FWER p-Value | 0.093 |
Table: GSEA Results Summary

  

Fig 1: Enrichment plot: TNFALPHA\_ALL\_UP      
 Profile of the Running ES Score & Positions of GeneSet Members on the Rank Ordered List

  

| PROBE | GENE SYMBOL | GENE\_TITLE | RANK IN GENE LIST | RANK METRIC SCORE | RUNNING ES | CORE ENRICHMENT || 1 | CCL2 |  |  | 1 | 1.308 | 0.0755 | Yes |
| 2 | IGFBP5 |  |  | 26 | 0.757 | 0.1180 | Yes |
| 3 | IFI27 |  |  | 32 | 0.725 | 0.1597 | Yes |
| 4 | CD59 |  |  | 86 | 0.600 | 0.1918 | Yes |
| 5 | SOD2 |  |  | 150 | 0.523 | 0.2189 | Yes |
| 6 | MCM7 |  |  | 153 | 0.521 | 0.2488 | Yes |
| 7 | TSPAN3 |  |  | 158 | 0.519 | 0.2786 | Yes |
| 8 | TNIP1 |  |  | 165 | 0.515 | 0.3081 | Yes |
| 9 | KIT |  |  | 211 | 0.487 | 0.3340 | Yes |
| 10 | TNFAIP3 |  |  | 406 | 0.424 | 0.3490 | Yes |
| 11 | PSMB8 |  |  | 426 | 0.420 | 0.3724 | Yes |
| 12 | INADL |  |  | 466 | 0.411 | 0.3942 | Yes |
| 13 | ATP5G2 |  |  | 469 | 0.410 | 0.4178 | Yes |
| 14 | PRDX2 |  |  | 800 | 0.354 | 0.4221 | Yes |
| 15 | CMPK |  |  | 913 | 0.340 | 0.4364 | Yes |
| 16 | TMF1 |  |  | 953 | 0.336 | 0.4538 | Yes |
| 17 | HMGA1 |  |  | 1086 | 0.323 | 0.4660 | Yes |
| 18 | PPP2R1A |  |  | 1488 | 0.288 | 0.4631 | Yes |
| 19 | PRDX5 |  |  | 1530 | 0.285 | 0.4776 | Yes |
| 20 | IL32 |  |  | 1685 | 0.274 | 0.4859 | Yes |
| 21 | OPRS1 |  |  | 1707 | 0.272 | 0.5005 | Yes |
| 22 | ERGIC3 |  |  | 1796 | 0.267 | 0.5117 | Yes |
| 23 | NDUFA13 |  |  | 1939 | 0.259 | 0.5197 | Yes |
| 24 | GINS2 |  |  | 2158 | 0.248 | 0.5234 | Yes |
| 25 | TRAP1 |  |  | 2175 | 0.247 | 0.5368 | Yes |
| 26 | ATP5E |  |  | 2203 | 0.245 | 0.5497 | Yes |
| 27 | SLC39A1 |  |  | 2330 | 0.239 | 0.5574 | Yes |
| 28 | ITGB2 |  |  | 2545 | 0.228 | 0.5601 | Yes |
| 29 | SF3A3 |  |  | 2579 | 0.227 | 0.5716 | Yes |
| 30 | IER2 |  |  | 2613 | 0.226 | 0.5830 | Yes |
| 31 | SNX12 |  |  | 2634 | 0.225 | 0.5950 | Yes |
| 32 | CTNNB1 |  |  | 3054 | 0.208 | 0.5866 | Yes |
| 33 | HNRPU |  |  | 3135 | 0.205 | 0.5946 | Yes |
| 34 | JUNB |  |  | 3199 | 0.203 | 0.6032 | Yes |
| 35 | GDI2 |  |  | 3640 | 0.190 | 0.5928 | No |
| 36 | RPS23 |  |  | 4424 | 0.168 | 0.5644 | No |
| 37 | COPS2 |  |  | 4463 | 0.167 | 0.5722 | No |
| 38 | PTMS |  |  | 4969 | 0.156 | 0.5566 | No |
| 39 | TEGT |  |  | 5004 | 0.155 | 0.5639 | No |
| 40 | SHFM1 |  |  | 5405 | 0.147 | 0.5529 | No |
| 41 | TPR |  |  | 5709 | 0.140 | 0.5462 | No |
| 42 | PPP1R10 |  |  | 5933 | 0.136 | 0.5432 | No |
| 43 | CDC34 |  |  | 6030 | 0.135 | 0.5463 | No |
| 44 | KALRN |  |  | 6094 | 0.134 | 0.5510 | No |
| 45 | PHB |  |  | 6528 | 0.126 | 0.5372 | No |
| 46 | OAZ1 |  |  | 6725 | 0.123 | 0.5347 | No |
| 47 | MRPL36 |  |  | 7280 | 0.113 | 0.5143 | No |
| 48 | PLP2 |  |  | 7352 | 0.112 | 0.5173 | No |
| 49 | NFKBIA |  |  | 7714 | 0.106 | 0.5058 | No |
| 50 | CLIC1 |  |  | 8047 | 0.101 | 0.4955 | No |
| 51 | RNASEN |  |  | 9554 | 0.079 | 0.4267 | No |
| 52 | CCNC |  |  | 9800 | 0.076 | 0.4192 | No |
| 53 | PIR |  |  | 9964 | 0.074 | 0.4155 | No |
| 54 | ELOVL5 |  |  | 10255 | 0.070 | 0.4054 | No |
| 55 | PSMB7 |  |  | 10529 | 0.066 | 0.3959 | No |
| 56 | CHD1L |  |  | 11555 | 0.053 | 0.3490 | No |
| 57 | EGR1 |  |  | 12189 | 0.044 | 0.3207 | No |
| 58 | NFKB1 |  |  | 12483 | 0.041 | 0.3088 | No |
| 59 | CXCL1 |  |  | 12797 | 0.037 | 0.2957 | No |
| 60 | MAP2K1 |  |  | 13159 | 0.032 | 0.2799 | No |
| 61 | CD5 |  |  | 13498 | 0.027 | 0.2651 | No |
| 62 | TUBA1 |  |  | 13598 | 0.026 | 0.2617 | No |
| 63 | KEL |  |  | 15209 | 0.002 | 0.1834 | No |
| 64 | SF3B2 |  |  | 15489 | -0.001 | 0.1699 | No |
| 65 | KLF10 |  |  | 15567 | -0.003 | 0.1664 | No |
| 66 | C10ORF7 |  |  | 15774 | -0.006 | 0.1567 | No |
| 67 | PCMT1 |  |  | 16232 | -0.014 | 0.1352 | No |
| 68 | FBN1 |  |  | 17185 | -0.033 | 0.0907 | No |
| 69 | ACLY |  |  | 17617 | -0.043 | 0.0722 | No |
| 70 | EMR3 |  |  | 17703 | -0.045 | 0.0707 | No |
| 71 | TGFBR3 |  |  | 18591 | -0.068 | 0.0314 | No |
| 72 | MAN2A1 |  |  | 18765 | -0.075 | 0.0273 | No |
| 73 | DUSP1 |  |  | 18808 | -0.076 | 0.0297 | No |
| 74 | ATF4 |  |  | 19204 | -0.093 | 0.0158 | No |
| 75 | PRDX1 |  |  | 20076 | -0.151 | -0.0179 | No |
| 76 | GADD45A |  |  | 20478 | -0.277 | -0.0214 | No |
| 77 | HSPB1 |  |  | 20567 | -0.477 | 0.0019 | No |
Table: GSEA details [plain text format]

  

Fig 2: TNFALPHA\_ALL\_UP: Random ES distribution      
 Gene set null distribution of ES for **TNFALPHA\_ALL\_UP**
