# Supplementary material for: The rapamycin-regulated gene expression signature determines prognosis for breast cancer
Source: Mol Cancer. 2009 Sep 24;8:75. doi: 10.1186/1476-4598-8-75 (PMC2761377; doi:10.1186/1476-4598-8-75)
Supplement: Additional file 3 — Gene set enrichment analysis of in vivo data, treatment series. The data provided represent the treatment series of GSEA. This compressed file contains "Treatment" shortcut file and "GSEA_treatment" folder. Clicking on "Treatment" shortcut opens the index file providing access to analysis files contained in the "GSEA_treatment" folder. [file 1476-4598-8-75-S3.zip › GSEA_treatment/TNFA_NFKB_DEP_UP.html]

Details for gene set TNFA\_NFKB\_DEP\_UP[GSEA]

|  || Dataset | gsea\_treatment\_collapsed |
| Phenotype | NoPhenotypeAvailable |
| Upregulated in class | na\_pos |
| GeneSet | TNFA\_NFKB\_DEP\_UP |
| Enrichment Score (ES) | 0.68391454 |
| Normalized Enrichment Score (NES) | 1.6823889 |
| Nominal p-value | 0.0032327587 |
| FDR q-value | 0.014396005 |
| FWER p-Value | 0.529 |
Table: GSEA Results Summary

  

Fig 1: Enrichment plot: TNFA\_NFKB\_DEP\_UP      
 Profile of the Running ES Score & Positions of GeneSet Members on the Rank Ordered List

  

| PROBE | GENE SYMBOL | GENE\_TITLE | RANK IN GENE LIST | RANK METRIC SCORE | RUNNING ES | CORE ENRICHMENT || 1 | TNFAIP2 |  |  | 22 | 0.790 | 0.1840 | Yes |
| 2 | TNIP1 |  |  | 165 | 0.515 | 0.2978 | Yes |
| 3 | CCL20 |  |  | 301 | 0.453 | 0.3972 | Yes |
| 4 | TNFAIP3 |  |  | 406 | 0.424 | 0.4915 | Yes |
| 5 | CXCL3 |  |  | 427 | 0.419 | 0.5888 | Yes |
| 6 | CXCL2 |  |  | 457 | 0.412 | 0.6839 | Yes |
| 7 | RELB |  |  | 2451 | 0.232 | 0.6415 | No |
| 8 | NFKB2 |  |  | 4591 | 0.164 | 0.5762 | No |
| 9 | IL6 |  |  | 5899 | 0.137 | 0.5447 | No |
| 10 | CD83 |  |  | 7305 | 0.113 | 0.5029 | No |
| 11 | NFKBIA |  |  | 7714 | 0.106 | 0.5080 | No |
| 12 | NFKBIE |  |  | 9077 | 0.086 | 0.4619 | No |
| 13 | TNFRSF9 |  |  | 9451 | 0.081 | 0.4627 | No |
| 14 | TRAF1 |  |  | 10005 | 0.073 | 0.4529 | No |
| 15 | NFKB1 |  |  | 12483 | 0.041 | 0.3422 | No |
| 16 | GFPT2 |  |  | 15707 | -0.005 | 0.1869 | No |
| 17 | PLK2 |  |  | 18412 | -0.063 | 0.0702 | No |
| 18 | GCH1 |  |  | 20092 | -0.155 | 0.0249 | No |
Table: GSEA details [plain text format]

  

Fig 2: TNFA\_NFKB\_DEP\_UP: Random ES distribution      
 Gene set null distribution of ES for **TNFA\_NFKB\_DEP\_UP**
