# Supplementary material for: The rapamycin-regulated gene expression signature determines prognosis for breast cancer
Source: Mol Cancer. 2009 Sep 24;8:75. doi: 10.1186/1476-4598-8-75 (PMC2761377; doi:10.1186/1476-4598-8-75)
Supplement: Additional file 3 — Gene set enrichment analysis of in vivo data, treatment series. The data provided represent the treatment series of GSEA. This compressed file contains "Treatment" shortcut file and "GSEA_treatment" folder. Clicking on "Treatment" shortcut opens the index file providing access to analysis files contained in the "GSEA_treatment" folder. [file 1476-4598-8-75-S3.zip › GSEA_treatment/TRNA_SYNTHETASES.html]

Details for gene set TRNA\_SYNTHETASES[GSEA]

|  || Dataset | gsea\_treatment\_collapsed |
| Phenotype | NoPhenotypeAvailable |
| Upregulated in class | na\_neg |
| GeneSet | TRNA\_SYNTHETASES |
| Enrichment Score (ES) | -0.36577412 |
| Normalized Enrichment Score (NES) | -1.3718283 |
| Nominal p-value | 0.12676056 |
| FDR q-value | 0.21184234 |
| FWER p-Value | 0.982 |
Table: GSEA Results Summary

  

Fig 1: Enrichment plot: TRNA\_SYNTHETASES      
 Profile of the Running ES Score & Positions of GeneSet Members on the Rank Ordered List

  

| PROBE | GENE SYMBOL | GENE\_TITLE | RANK IN GENE LIST | RANK METRIC SCORE | RUNNING ES | CORE ENRICHMENT || 1 | QARS |  |  | 240 | 0.473 | 0.1421 | No |
| 2 | KIAA1522 |  |  | 796 | 0.354 | 0.2304 | No |
| 3 | DARS |  |  | 1276 | 0.304 | 0.3060 | No |
| 4 | HARSL |  |  | 3171 | 0.204 | 0.2803 | No |
| 5 | LARS |  |  | 5106 | 0.153 | 0.2361 | No |
| 6 | RARS |  |  | 10372 | 0.068 | 0.0024 | No |
| 7 | CARS |  |  | 10805 | 0.062 | 0.0016 | No |
| 8 | EPRS |  |  | 12393 | 0.042 | -0.0619 | No |
| 9 | KARS |  |  | 14246 | 0.017 | -0.1462 | No |
| 10 | YARS |  |  | 15535 | -0.002 | -0.2080 | No |
| 11 | HARS |  |  | 16413 | -0.017 | -0.2450 | No |
| 12 | IARS |  |  | 17477 | -0.039 | -0.2838 | No |
| 13 | WARS |  |  | 17621 | -0.043 | -0.2768 | No |
| 14 | MARS |  |  | 19453 | -0.106 | -0.3314 | Yes |
| 15 | SARS |  |  | 19872 | -0.133 | -0.3083 | Yes |
| 16 | TARS |  |  | 19912 | -0.137 | -0.2658 | Yes |
| 17 | NARS |  |  | 20386 | -0.219 | -0.2175 | Yes |
| 18 | AARS |  |  | 20451 | -0.254 | -0.1379 | Yes |
| 19 | GARS |  |  | 20560 | -0.447 | 0.0022 | Yes |
Table: GSEA details [plain text format]

  

Fig 2: TRNA\_SYNTHETASES: Random ES distribution      
 Gene set null distribution of ES for **TRNA\_SYNTHETASES**
