# Supplementary material for: The rapamycin-regulated gene expression signature determines prognosis for breast cancer
Source: Mol Cancer. 2009 Sep 24;8:75. doi: 10.1186/1476-4598-8-75 (PMC2761377; doi:10.1186/1476-4598-8-75)
Supplement: Additional file 3 — Gene set enrichment analysis of in vivo data, treatment series. The data provided represent the treatment series of GSEA. This compressed file contains "Treatment" shortcut file and "GSEA_treatment" folder. Clicking on "Treatment" shortcut opens the index file providing access to analysis files contained in the "GSEA_treatment" folder. [file 1476-4598-8-75-S3.zip › GSEA_treatment/TSADAC_PANC50_UP.html]

Details for gene set TSADAC\_PANC50\_UP[GSEA]

|  || Dataset | gsea\_treatment\_collapsed |
| Phenotype | NoPhenotypeAvailable |
| Upregulated in class | na\_neg |
| GeneSet | TSADAC\_PANC50\_UP |
| Enrichment Score (ES) | -0.3561082 |
| Normalized Enrichment Score (NES) | -1.5142332 |
| Nominal p-value | 0.0 |
| FDR q-value | 0.1741627 |
| FWER p-Value | 0.836 |
Table: GSEA Results Summary

  

Fig 1: Enrichment plot: TSADAC\_PANC50\_UP      
 Profile of the Running ES Score & Positions of GeneSet Members on the Rank Ordered List

  

| PROBE | GENE SYMBOL | GENE\_TITLE | RANK IN GENE LIST | RANK METRIC SCORE | RUNNING ES | CORE ENRICHMENT || 1 | BIK |  |  | 333 | 0.443 | 0.0462 | No |
| 2 | VIL2 |  |  | 461 | 0.411 | 0.0980 | No |
| 3 | BST2 |  |  | 657 | 0.374 | 0.1413 | No |
| 4 | TP53I11 |  |  | 760 | 0.358 | 0.1868 | No |
| 5 | FGF2 |  |  | 834 | 0.348 | 0.2323 | No |
| 6 | SFRP1 |  |  | 1048 | 0.327 | 0.2680 | No |
| 7 | IL32 |  |  | 1685 | 0.274 | 0.2756 | No |
| 8 | F11R |  |  | 2735 | 0.221 | 0.2558 | No |
| 9 | BNIP3 |  |  | 3398 | 0.197 | 0.2514 | No |
| 10 | NALP2 |  |  | 6767 | 0.122 | 0.1048 | No |
| 11 | LHX2 |  |  | 6965 | 0.119 | 0.1120 | No |
| 12 | SALL1 |  |  | 7496 | 0.110 | 0.1017 | No |
| 13 | SPANXC |  |  | 7820 | 0.105 | 0.1007 | No |
| 14 | CLDN7 |  |  | 7949 | 0.102 | 0.1089 | No |
| 15 | UCHL1 |  |  | 8269 | 0.098 | 0.1072 | No |
| 16 | DAPK2 |  |  | 9514 | 0.080 | 0.0579 | No |
| 17 | PRG1 |  |  | 10469 | 0.066 | 0.0209 | No |
| 18 | ARC |  |  | 10685 | 0.064 | 0.0195 | No |
| 19 | PAX6 |  |  | 11128 | 0.058 | 0.0062 | No |
| 20 | CASP5 |  |  | 11183 | 0.057 | 0.0116 | No |
| 21 | MAGEC1 |  |  | 11850 | 0.049 | -0.0139 | No |
| 22 | CRLF1 |  |  | 11976 | 0.047 | -0.0134 | No |
| 23 | MLH1 |  |  | 12496 | 0.040 | -0.0329 | No |
| 24 | TERT |  |  | 13235 | 0.031 | -0.0644 | No |
| 25 | CDC25A |  |  | 13448 | 0.028 | -0.0707 | No |
| 26 | GAS1 |  |  | 14002 | 0.021 | -0.0947 | No |
| 27 | LIPC |  |  | 14029 | 0.020 | -0.0931 | No |
| 28 | DAZL |  |  | 14377 | 0.015 | -0.1078 | No |
| 29 | SGCE |  |  | 14684 | 0.011 | -0.1212 | No |
| 30 | WISP2 |  |  | 14771 | 0.010 | -0.1240 | No |
| 31 | TKTL1 |  |  | 15021 | 0.006 | -0.1353 | No |
| 32 | CSF3 |  |  | 15914 | -0.009 | -0.1775 | No |
| 33 | AURKC |  |  | 16500 | -0.019 | -0.2033 | No |
| 34 | ITM2C |  |  | 16678 | -0.023 | -0.2087 | No |
| 35 | WIT1 |  |  | 16896 | -0.026 | -0.2156 | No |
| 36 | MMP8 |  |  | 18780 | -0.076 | -0.2965 | No |
| 37 | DDX4 |  |  | 19316 | -0.099 | -0.3086 | No |
| 38 | CCNA1 |  |  | 20293 | -0.193 | -0.3289 | Yes |
| 39 | CRIP1 |  |  | 20410 | -0.230 | -0.3020 | Yes |
| 40 | HSPA1A |  |  | 20495 | -0.294 | -0.2647 | Yes |
| 41 | S100P |  |  | 20599 | -0.897 | -0.1433 | Yes |
| 42 | S100A2 |  |  | 20601 | -1.019 | 0.0002 | Yes |
Table: GSEA details [plain text format]

  

Fig 2: TSADAC\_PANC50\_UP: Random ES distribution      
 Gene set null distribution of ES for **TSADAC\_PANC50\_UP**
