# Supplementary material for: The rapamycin-regulated gene expression signature determines prognosis for breast cancer
Source: Mol Cancer. 2009 Sep 24;8:75. doi: 10.1186/1476-4598-8-75 (PMC2761377; doi:10.1186/1476-4598-8-75)
Supplement: Additional file 3 — Gene set enrichment analysis of in vivo data, treatment series. The data provided represent the treatment series of GSEA. This compressed file contains "Treatment" shortcut file and "GSEA_treatment" folder. Clicking on "Treatment" shortcut opens the index file providing access to analysis files contained in the "GSEA_treatment" folder. [file 1476-4598-8-75-S3.zip › GSEA_treatment/TSA_PANC50_UP.html]

Details for gene set TSA\_PANC50\_UP[GSEA]

|  || Dataset | gsea\_treatment\_collapsed |
| Phenotype | NoPhenotypeAvailable |
| Upregulated in class | na\_pos |
| GeneSet | TSA\_PANC50\_UP |
| Enrichment Score (ES) | 0.6247527 |
| Normalized Enrichment Score (NES) | 1.720281 |
| Nominal p-value | 0.0 |
| FDR q-value | 0.010495069 |
| FWER p-Value | 0.316 |
Table: GSEA Results Summary

  

Fig 1: Enrichment plot: TSA\_PANC50\_UP      
 Profile of the Running ES Score & Positions of GeneSet Members on the Rank Ordered List

  

| PROBE | GENE SYMBOL | GENE\_TITLE | RANK IN GENE LIST | RANK METRIC SCORE | RUNNING ES | CORE ENRICHMENT || 1 | TFPI2 |  |  | 0 | 1.316 | 0.1779 | Yes |
| 2 | CTGF |  |  | 12 | 0.922 | 0.3020 | Yes |
| 3 | CDKN1C |  |  | 46 | 0.688 | 0.3933 | Yes |
| 4 | IL8 |  |  | 76 | 0.611 | 0.4745 | Yes |
| 5 | UCP2 |  |  | 297 | 0.454 | 0.5252 | Yes |
| 6 | BIK |  |  | 333 | 0.443 | 0.5833 | Yes |
| 7 | NOTCH3 |  |  | 570 | 0.391 | 0.6248 | Yes |
| 8 | DHRS2 |  |  | 2762 | 0.220 | 0.5479 | No |
| 9 | PHC1 |  |  | 3583 | 0.191 | 0.5339 | No |
| 10 | GDF15 |  |  | 5068 | 0.154 | 0.4826 | No |
| 11 | WNT4 |  |  | 5348 | 0.148 | 0.4890 | No |
| 12 | ABCB1 |  |  | 5631 | 0.142 | 0.4945 | No |
| 13 | HIST1H1C |  |  | 7360 | 0.112 | 0.4256 | No |
| 14 | CACNA1G |  |  | 7488 | 0.110 | 0.4343 | No |
| 15 | TUSC4 |  |  | 7629 | 0.108 | 0.4420 | No |
| 16 | STC1 |  |  | 9045 | 0.086 | 0.3848 | No |
| 17 | KLK12 |  |  | 9787 | 0.076 | 0.3591 | No |
| 18 | EGR4 |  |  | 9950 | 0.074 | 0.3612 | No |
| 19 | CDH8 |  |  | 10144 | 0.071 | 0.3615 | No |
| 20 | ING1 |  |  | 10405 | 0.067 | 0.3579 | No |
| 21 | GADD45B |  |  | 11255 | 0.056 | 0.3242 | No |
| 22 | ATF3 |  |  | 11262 | 0.056 | 0.3316 | No |
| 23 | AREG |  |  | 11292 | 0.056 | 0.3377 | No |
| 24 | MBD1 |  |  | 11350 | 0.055 | 0.3424 | No |
| 25 | ANGPT1 |  |  | 11596 | 0.052 | 0.3375 | No |
| 26 | EREG |  |  | 12711 | 0.038 | 0.2884 | No |
| 27 | NGFR |  |  | 13261 | 0.031 | 0.2659 | No |
| 28 | HPSE |  |  | 13325 | 0.030 | 0.2668 | No |
| 29 | CGREF1 |  |  | 14574 | 0.012 | 0.2078 | No |
| 30 | HMOX1 |  |  | 15526 | -0.002 | 0.1619 | No |
| 31 | ID2 |  |  | 15550 | -0.003 | 0.1611 | No |
| 32 | SSX1 |  |  | 15919 | -0.009 | 0.1444 | No |
| 33 | PER1 |  |  | 16113 | -0.012 | 0.1367 | No |
| 34 | CLDN15 |  |  | 16432 | -0.018 | 0.1236 | No |
| 35 | MMP1 |  |  | 16811 | -0.025 | 0.1085 | No |
| 36 | ULBP2 |  |  | 17071 | -0.030 | 0.1000 | No |
| 37 | SERPINI1 |  |  | 17300 | -0.035 | 0.0937 | No |
| 38 | ADM |  |  | 20571 | -0.495 | 0.0017 | No |
Table: GSEA details [plain text format]

  

Fig 2: TSA\_PANC50\_UP: Random ES distribution      
 Gene set null distribution of ES for **TSA\_PANC50\_UP**
