# Supplementary material for: The rapamycin-regulated gene expression signature determines prognosis for breast cancer
Source: Mol Cancer. 2009 Sep 24;8:75. doi: 10.1186/1476-4598-8-75 (PMC2761377; doi:10.1186/1476-4598-8-75)
Supplement: Additional file 3 — Gene set enrichment analysis of in vivo data, treatment series. The data provided represent the treatment series of GSEA. This compressed file contains "Treatment" shortcut file and "GSEA_treatment" folder. Clicking on "Treatment" shortcut opens the index file providing access to analysis files contained in the "GSEA_treatment" folder. [file 1476-4598-8-75-S3.zip › GSEA_treatment/TUMOR_SUPRESSOR.html]

Details for gene set TUMOR\_SUPRESSOR[GSEA]

|  || Dataset | gsea\_treatment\_collapsed |
| Phenotype | NoPhenotypeAvailable |
| Upregulated in class | na\_pos |
| GeneSet | TUMOR\_SUPRESSOR |
| Enrichment Score (ES) | 0.6657629 |
| Normalized Enrichment Score (NES) | 1.718026 |
| Nominal p-value | 0.0010729614 |
| FDR q-value | 0.01046334 |
| FWER p-Value | 0.331 |
Table: GSEA Results Summary

  

Fig 1: Enrichment plot: TUMOR\_SUPRESSOR      
 Profile of the Running ES Score & Positions of GeneSet Members on the Rank Ordered List

  

| PROBE | GENE SYMBOL | GENE\_TITLE | RANK IN GENE LIST | RANK METRIC SCORE | RUNNING ES | CORE ENRICHMENT || 1 | CDKN1C |  |  | 46 | 0.688 | 0.1343 | Yes |
| 2 | TP53 |  |  | 67 | 0.630 | 0.2585 | Yes |
| 3 | IRF1 |  |  | 551 | 0.393 | 0.3132 | Yes |
| 4 | TGFBR1 |  |  | 700 | 0.367 | 0.3790 | Yes |
| 5 | CFL1 |  |  | 737 | 0.362 | 0.4490 | Yes |
| 6 | MSH2 |  |  | 787 | 0.355 | 0.5171 | Yes |
| 7 | TSC2 |  |  | 789 | 0.354 | 0.5874 | Yes |
| 8 | CREBBP |  |  | 1584 | 0.280 | 0.6044 | Yes |
| 9 | CDKN1B |  |  | 1757 | 0.269 | 0.6495 | Yes |
| 10 | NF2 |  |  | 2740 | 0.221 | 0.6456 | Yes |
| 11 | EP300 |  |  | 3705 | 0.188 | 0.6361 | Yes |
| 12 | LCMT2 |  |  | 3846 | 0.184 | 0.6658 | Yes |
| 13 | ACTB |  |  | 5728 | 0.140 | 0.6022 | No |
| 14 | TGFBR2 |  |  | 6423 | 0.128 | 0.5938 | No |
| 15 | CDKN2D |  |  | 7525 | 0.109 | 0.5620 | No |
| 16 | VHL |  |  | 8788 | 0.090 | 0.5186 | No |
| 17 | BRCA1 |  |  | 9360 | 0.082 | 0.5072 | No |
| 18 | BRCA2 |  |  | 10504 | 0.066 | 0.4647 | No |
| 19 | APC |  |  | 13244 | 0.031 | 0.3378 | No |
| 20 | PTEN |  |  | 13509 | 0.027 | 0.3304 | No |
| 21 | WT1 |  |  | 14347 | 0.016 | 0.2929 | No |
| 22 | CCL26 |  |  | 16143 | -0.013 | 0.2081 | No |
| 23 | RB1 |  |  | 17665 | -0.043 | 0.1428 | No |
Table: GSEA details [plain text format]

  

Fig 2: TUMOR\_SUPRESSOR: Random ES distribution      
 Gene set null distribution of ES for **TUMOR\_SUPRESSOR**
