# Supplementary material for: The rapamycin-regulated gene expression signature determines prognosis for breast cancer
Source: Mol Cancer. 2009 Sep 24;8:75. doi: 10.1186/1476-4598-8-75 (PMC2761377; doi:10.1186/1476-4598-8-75)
Supplement: Additional file 3 — Gene set enrichment analysis of in vivo data, treatment series. The data provided represent the treatment series of GSEA. This compressed file contains "Treatment" shortcut file and "GSEA_treatment" folder. Clicking on "Treatment" shortcut opens the index file providing access to analysis files contained in the "GSEA_treatment" folder. [file 1476-4598-8-75-S3.zip › GSEA_treatment/UV-4NQO_FIBRO_UP.html]

Details for gene set UV-4NQO\_FIBRO\_UP[GSEA]

|  || Dataset | gsea\_treatment\_collapsed |
| Phenotype | NoPhenotypeAvailable |
| Upregulated in class | na\_pos |
| GeneSet | UV-4NQO\_FIBRO\_UP |
| Enrichment Score (ES) | 0.65439296 |
| Normalized Enrichment Score (NES) | 1.7171655 |
| Nominal p-value | 0.002096436 |
| FDR q-value | 0.010002876 |
| FWER p-Value | 0.332 |
Table: GSEA Results Summary

  

Fig 1: Enrichment plot: UV-4NQO\_FIBRO\_UP      
 Profile of the Running ES Score & Positions of GeneSet Members on the Rank Ordered List

  

| PROBE | GENE SYMBOL | GENE\_TITLE | RANK IN GENE LIST | RANK METRIC SCORE | RUNNING ES | CORE ENRICHMENT || 1 | TFPI2 |  |  | 0 | 1.316 | 0.2063 | Yes |
| 2 | IFNGR1 |  |  | 82 | 0.606 | 0.2974 | Yes |
| 3 | ITGB1 |  |  | 746 | 0.360 | 0.3216 | Yes |
| 4 | PTK9 |  |  | 782 | 0.355 | 0.3756 | Yes |
| 5 | PAFAH1B3 |  |  | 995 | 0.332 | 0.4174 | Yes |
| 6 | NMT1 |  |  | 1273 | 0.304 | 0.4516 | Yes |
| 7 | RIN2 |  |  | 1404 | 0.294 | 0.4914 | Yes |
| 8 | IRF2 |  |  | 1500 | 0.287 | 0.5317 | Yes |
| 9 | SUOX |  |  | 2614 | 0.226 | 0.5130 | Yes |
| 10 | SEMA4D |  |  | 2676 | 0.224 | 0.5451 | Yes |
| 11 | RAB7 |  |  | 3146 | 0.205 | 0.5544 | Yes |
| 12 | TLE1 |  |  | 3151 | 0.205 | 0.5864 | Yes |
| 13 | PPP2R2B |  |  | 3365 | 0.198 | 0.6071 | Yes |
| 14 | PCNA |  |  | 3626 | 0.190 | 0.6243 | Yes |
| 15 | ITGAL |  |  | 3743 | 0.187 | 0.6480 | Yes |
| 16 | PRMT1 |  |  | 4175 | 0.175 | 0.6544 | Yes |
| 17 | RAP2A |  |  | 5008 | 0.155 | 0.6383 | No |
| 18 | SERPINB2 |  |  | 6688 | 0.124 | 0.5761 | No |
| 19 | SLC35B1 |  |  | 7120 | 0.115 | 0.5732 | No |
| 20 | MYD88 |  |  | 7910 | 0.103 | 0.5511 | No |
| 21 | BTG3 |  |  | 13091 | 0.033 | 0.3045 | No |
| 22 | PRKAB1 |  |  | 13189 | 0.032 | 0.3048 | No |
| 23 | CDC25A |  |  | 13448 | 0.028 | 0.2967 | No |
| 24 | IGF1 |  |  | 14591 | 0.012 | 0.2431 | No |
| 25 | C1ORF61 |  |  | 17007 | -0.029 | 0.1302 | No |
| 26 | CDC37L1 |  |  | 17738 | -0.045 | 0.1018 | No |
| 27 | TGFB3 |  |  | 19003 | -0.084 | 0.0536 | No |
| 28 | INPP1 |  |  | 20091 | -0.154 | 0.0250 | No |
Table: GSEA details [plain text format]

  

Fig 2: UV-4NQO\_FIBRO\_UP: Random ES distribution      
 Gene set null distribution of ES for **UV-4NQO\_FIBRO\_UP**
