# Supplementary material for: The rapamycin-regulated gene expression signature determines prognosis for breast cancer
Source: Mol Cancer. 2009 Sep 24;8:75. doi: 10.1186/1476-4598-8-75 (PMC2761377; doi:10.1186/1476-4598-8-75)
Supplement: Additional file 3 — Gene set enrichment analysis of in vivo data, treatment series. The data provided represent the treatment series of GSEA. This compressed file contains "Treatment" shortcut file and "GSEA_treatment" folder. Clicking on "Treatment" shortcut opens the index file providing access to analysis files contained in the "GSEA_treatment" folder. [file 1476-4598-8-75-S3.zip › GSEA_treatment/UVB_NHEK3_C7.html]

Details for gene set UVB\_NHEK3\_C7[GSEA]

|  || Dataset | gsea\_treatment\_collapsed |
| Phenotype | NoPhenotypeAvailable |
| Upregulated in class | na\_neg |
| GeneSet | UVB\_NHEK3\_C7 |
| Enrichment Score (ES) | -0.3232341 |
| Normalized Enrichment Score (NES) | -1.3988101 |
| Nominal p-value | 0.04 |
| FDR q-value | 0.2030706 |
| FWER p-Value | 0.971 |
Table: GSEA Results Summary

  

Fig 1: Enrichment plot: UVB\_NHEK3\_C7      
 Profile of the Running ES Score & Positions of GeneSet Members on the Rank Ordered List

  

| PROBE | GENE SYMBOL | GENE\_TITLE | RANK IN GENE LIST | RANK METRIC SCORE | RUNNING ES | CORE ENRICHMENT || 1 | CIRBP |  |  | 19 | 0.845 | 0.0801 | No |
| 2 | IFITM1 |  |  | 79 | 0.609 | 0.1356 | No |
| 3 | HMGB1 |  |  | 247 | 0.469 | 0.1724 | No |
| 4 | ITGB1 |  |  | 746 | 0.360 | 0.1827 | No |
| 5 | RPL3 |  |  | 1238 | 0.308 | 0.1883 | No |
| 6 | TAF10 |  |  | 1825 | 0.265 | 0.1852 | No |
| 7 | MTHFD1 |  |  | 2345 | 0.238 | 0.1827 | No |
| 8 | PGD |  |  | 2637 | 0.225 | 0.1901 | No |
| 9 | ESD |  |  | 3445 | 0.196 | 0.1696 | No |
| 10 | MT1X |  |  | 3564 | 0.192 | 0.1822 | No |
| 11 | ASS1 |  |  | 3567 | 0.192 | 0.2005 | No |
| 12 | IDH2 |  |  | 3672 | 0.189 | 0.2136 | No |
| 13 | SRD5A1 |  |  | 3710 | 0.188 | 0.2298 | No |
| 14 | ADK |  |  | 4336 | 0.171 | 0.2158 | No |
| 15 | UBE2C |  |  | 4419 | 0.168 | 0.2279 | No |
| 16 | ITGA6 |  |  | 4923 | 0.157 | 0.2185 | No |
| 17 | MMP10 |  |  | 5268 | 0.150 | 0.2161 | No |
| 18 | CTSC |  |  | 5510 | 0.144 | 0.2182 | No |
| 19 | HNRPA2B1 |  |  | 5691 | 0.140 | 0.2229 | No |
| 20 | BLVRA |  |  | 6199 | 0.132 | 0.2109 | No |
| 21 | NCAPD2 |  |  | 7264 | 0.113 | 0.1700 | No |
| 22 | PLP2 |  |  | 7352 | 0.112 | 0.1765 | No |
| 23 | KIAA0101 |  |  | 7666 | 0.107 | 0.1716 | No |
| 24 | ATIC |  |  | 7692 | 0.107 | 0.1806 | No |
| 25 | PRCP |  |  | 9016 | 0.087 | 0.1245 | No |
| 26 | HSD17B4 |  |  | 9688 | 0.077 | 0.0993 | No |
| 27 | AKR1A1 |  |  | 11934 | 0.048 | -0.0054 | No |
| 28 | ASNS |  |  | 11946 | 0.048 | -0.0014 | No |
| 29 | ATP1B3 |  |  | 12238 | 0.044 | -0.0113 | No |
| 30 | CDC25B |  |  | 12425 | 0.041 | -0.0164 | No |
| 31 | EXOSC7 |  |  | 12459 | 0.041 | -0.0141 | No |
| 32 | MLH1 |  |  | 12496 | 0.040 | -0.0120 | No |
| 33 | CDKN3 |  |  | 12962 | 0.035 | -0.0313 | No |
| 34 | CDC20 |  |  | 13049 | 0.033 | -0.0323 | No |
| 35 | XBP1 |  |  | 13339 | 0.029 | -0.0435 | No |
| 36 | PPIB |  |  | 13763 | 0.024 | -0.0619 | No |
| 37 | YARS |  |  | 15535 | -0.002 | -0.1478 | No |
| 38 | CBR1 |  |  | 15607 | -0.004 | -0.1509 | No |
| 39 | CKAP4 |  |  | 16519 | -0.019 | -0.1934 | No |
| 40 | MMP1 |  |  | 16811 | -0.025 | -0.2052 | No |
| 41 | LIF |  |  | 17567 | -0.041 | -0.2379 | No |
| 42 | TCN1 |  |  | 18499 | -0.065 | -0.2770 | No |
| 43 | PLCD1 |  |  | 18547 | -0.067 | -0.2729 | No |
| 44 | PAM |  |  | 18827 | -0.078 | -0.2790 | No |
| 45 | EPHX2 |  |  | 18840 | -0.078 | -0.2720 | No |
| 46 | CEBPD |  |  | 19851 | -0.131 | -0.3086 | Yes |
| 47 | PLA2G4A |  |  | 20153 | -0.164 | -0.3075 | Yes |
| 48 | PCK2 |  |  | 20288 | -0.191 | -0.2957 | Yes |
| 49 | AARS |  |  | 20451 | -0.254 | -0.2792 | Yes |
| 50 | VSNL1 |  |  | 20578 | -0.533 | -0.2342 | Yes |
| 51 | MALL |  |  | 20580 | -0.579 | -0.1788 | Yes |
| 52 | ACSL1 |  |  | 20590 | -0.711 | -0.1111 | Yes |
| 53 | FGFBP1 |  |  | 20602 | -1.166 | 0.0001 | Yes |
Table: GSEA details [plain text format]

  

Fig 2: UVB\_NHEK3\_C7: Random ES distribution      
 Gene set null distribution of ES for **UVB\_NHEK3\_C7**
