# Supplementary material for: The rapamycin-regulated gene expression signature determines prognosis for breast cancer
Source: Mol Cancer. 2009 Sep 24;8:75. doi: 10.1186/1476-4598-8-75 (PMC2761377; doi:10.1186/1476-4598-8-75)
Supplement: Additional file 3 — Gene set enrichment analysis of in vivo data, treatment series. The data provided represent the treatment series of GSEA. This compressed file contains "Treatment" shortcut file and "GSEA_treatment" folder. Clicking on "Treatment" shortcut opens the index file providing access to analysis files contained in the "GSEA_treatment" folder. [file 1476-4598-8-75-S3.zip › GSEA_treatment/UVB_SCC_UP.html]

Details for gene set UVB\_SCC\_UP[GSEA]

|  || Dataset | gsea\_treatment\_collapsed |
| Phenotype | NoPhenotypeAvailable |
| Upregulated in class | na\_pos |
| GeneSet | UVB\_SCC\_UP |
| Enrichment Score (ES) | 0.5915697 |
| Normalized Enrichment Score (NES) | 1.7541487 |
| Nominal p-value | 0.0 |
| FDR q-value | 0.0064708777 |
| FWER p-Value | 0.169 |
Table: GSEA Results Summary

  

Fig 1: Enrichment plot: UVB\_SCC\_UP      
 Profile of the Running ES Score & Positions of GeneSet Members on the Rank Ordered List

  

| PROBE | GENE SYMBOL | GENE\_TITLE | RANK IN GENE LIST | RANK METRIC SCORE | RUNNING ES | CORE ENRICHMENT || 1 | IGFBP7 |  |  | 44 | 0.693 | 0.0347 | Yes |
| 2 | CDKN1C |  |  | 46 | 0.688 | 0.0712 | Yes |
| 3 | SH3BGRL |  |  | 131 | 0.542 | 0.0960 | Yes |
| 4 | NAP1L1 |  |  | 142 | 0.531 | 0.1238 | Yes |
| 5 | HIST1H2BK |  |  | 203 | 0.491 | 0.1470 | Yes |
| 6 | ITM2B |  |  | 234 | 0.478 | 0.1709 | Yes |
| 7 | ATP6AP2 |  |  | 258 | 0.466 | 0.1945 | Yes |
| 8 | CCL20 |  |  | 301 | 0.453 | 0.2165 | Yes |
| 9 | ALDH7A1 |  |  | 327 | 0.444 | 0.2389 | Yes |
| 10 | CXCL3 |  |  | 427 | 0.419 | 0.2564 | Yes |
| 11 | CXCL2 |  |  | 457 | 0.412 | 0.2769 | Yes |
| 12 | RBBP4 |  |  | 474 | 0.410 | 0.2979 | Yes |
| 13 | RCN2 |  |  | 540 | 0.396 | 0.3158 | Yes |
| 14 | C11ORF51 |  |  | 578 | 0.390 | 0.3348 | Yes |
| 15 | SCARB2 |  |  | 596 | 0.386 | 0.3544 | Yes |
| 16 | DYNC2LI1 |  |  | 662 | 0.373 | 0.3711 | Yes |
| 17 | PLSCR1 |  |  | 717 | 0.365 | 0.3879 | Yes |
| 18 | CITED2 |  |  | 722 | 0.365 | 0.4071 | Yes |
| 19 | SEPT10 |  |  | 728 | 0.364 | 0.4262 | Yes |
| 20 | PTK9 |  |  | 782 | 0.355 | 0.4425 | Yes |
| 21 | HPRT1 |  |  | 803 | 0.354 | 0.4603 | Yes |
| 22 | SKP1A |  |  | 832 | 0.349 | 0.4775 | Yes |
| 23 | SFRS1 |  |  | 923 | 0.339 | 0.4912 | Yes |
| 24 | C18ORF10 |  |  | 1015 | 0.330 | 0.5043 | Yes |
| 25 | JUND |  |  | 1372 | 0.296 | 0.5027 | Yes |
| 26 | YWHAZ |  |  | 1408 | 0.293 | 0.5166 | Yes |
| 27 | KYNU |  |  | 1426 | 0.292 | 0.5313 | Yes |
| 28 | EIF2S3 |  |  | 1453 | 0.290 | 0.5454 | Yes |
| 29 | UQCRC2 |  |  | 1695 | 0.273 | 0.5481 | Yes |
| 30 | UBE2E1 |  |  | 2055 | 0.253 | 0.5441 | Yes |
| 31 | B2M |  |  | 2291 | 0.240 | 0.5454 | Yes |
| 32 | RECQL |  |  | 2744 | 0.221 | 0.5351 | Yes |
| 33 | UBE2D3 |  |  | 2819 | 0.217 | 0.5431 | Yes |
| 34 | DSG2 |  |  | 2974 | 0.212 | 0.5468 | Yes |
| 35 | SSR1 |  |  | 2980 | 0.211 | 0.5578 | Yes |
| 36 | PRKAR1A |  |  | 3195 | 0.203 | 0.5582 | Yes |
| 37 | PRDX3 |  |  | 3225 | 0.202 | 0.5675 | Yes |
| 38 | PDIA6 |  |  | 3331 | 0.199 | 0.5730 | Yes |
| 39 | IL13RA1 |  |  | 3429 | 0.196 | 0.5787 | Yes |
| 40 | TM2D1 |  |  | 3787 | 0.186 | 0.5712 | Yes |
| 41 | PAPOLA |  |  | 3905 | 0.182 | 0.5751 | Yes |
| 42 | CDC2 |  |  | 3984 | 0.179 | 0.5809 | Yes |
| 43 | HRSP12 |  |  | 4098 | 0.176 | 0.5848 | Yes |
| 44 | LAMP2 |  |  | 4224 | 0.174 | 0.5879 | Yes |
| 45 | ADK |  |  | 4336 | 0.171 | 0.5916 | Yes |
| 46 | AGL |  |  | 4770 | 0.160 | 0.5790 | No |
| 47 | SDHD |  |  | 4967 | 0.156 | 0.5777 | No |
| 48 | SRI |  |  | 5209 | 0.151 | 0.5740 | No |
| 49 | SNX3 |  |  | 5673 | 0.141 | 0.5590 | No |
| 50 | IL6 |  |  | 5899 | 0.137 | 0.5553 | No |
| 51 | AP4S1 |  |  | 6413 | 0.128 | 0.5371 | No |
| 52 | PGK1 |  |  | 6774 | 0.122 | 0.5260 | No |
| 53 | SCFD1 |  |  | 6877 | 0.120 | 0.5275 | No |
| 54 | SCYE1 |  |  | 7288 | 0.113 | 0.5135 | No |
| 55 | RRM2 |  |  | 7744 | 0.106 | 0.4970 | No |
| 56 | RFC5 |  |  | 7909 | 0.103 | 0.4945 | No |
| 57 | TAF2 |  |  | 8019 | 0.101 | 0.4946 | No |
| 58 | RBMXL1 |  |  | 8216 | 0.098 | 0.4902 | No |
| 59 | PSMD8 |  |  | 9137 | 0.085 | 0.4499 | No |
| 60 | ATP5A1 |  |  | 9641 | 0.078 | 0.4295 | No |
| 61 | ARHGDIB |  |  | 9762 | 0.076 | 0.4278 | No |
| 62 | CCNC |  |  | 9800 | 0.076 | 0.4300 | No |
| 63 | RAD51C |  |  | 9806 | 0.076 | 0.4338 | No |
| 64 | ME2 |  |  | 10948 | 0.060 | 0.3814 | No |
| 65 | HEXIM1 |  |  | 11120 | 0.058 | 0.3761 | No |
| 66 | ATF3 |  |  | 11262 | 0.056 | 0.3723 | No |
| 67 | MAP2K1 |  |  | 13159 | 0.032 | 0.2816 | No |
| 68 | COPB1 |  |  | 13178 | 0.032 | 0.2824 | No |
| 69 | AP1S2 |  |  | 13332 | 0.029 | 0.2765 | No |
| 70 | DYNLT3 |  |  | 13573 | 0.026 | 0.2662 | No |
| 71 | SLC11A1 |  |  | 13673 | 0.025 | 0.2627 | No |
| 72 | HSP90AB1 |  |  | 13758 | 0.024 | 0.2599 | No |
| 73 | RALB |  |  | 13761 | 0.024 | 0.2610 | No |
| 74 | TACSTD2 |  |  | 13916 | 0.022 | 0.2547 | No |
| 75 | ANXA2 |  |  | 14618 | 0.012 | 0.2211 | No |
| 76 | CCBP2 |  |  | 15148 | 0.004 | 0.1955 | No |
| 77 | ID2 |  |  | 15550 | -0.003 | 0.1762 | No |
| 78 | PFDN6 |  |  | 15699 | -0.005 | 0.1692 | No |
| 79 | RTN4 |  |  | 15733 | -0.006 | 0.1679 | No |
| 80 | PTGES3 |  |  | 15743 | -0.006 | 0.1678 | No |
| 81 | TSC22D3 |  |  | 18666 | -0.071 | 0.0291 | No |
| 82 | TAGLN3 |  |  | 19346 | -0.101 | 0.0014 | No |
| 83 | ANXA3 |  |  | 19727 | -0.122 | -0.0106 | No |
| 84 | TOB1 |  |  | 20356 | -0.208 | -0.0302 | No |
| 85 | PMAIP1 |  |  | 20440 | -0.248 | -0.0210 | No |
| 86 | MGC14376 |  |  | 20472 | -0.268 | -0.0083 | No |
| 87 | GADD45A |  |  | 20478 | -0.277 | 0.0062 | No |
Table: GSEA details [plain text format]

  

Fig 2: UVB\_SCC\_UP: Random ES distribution      
 Gene set null distribution of ES for **UVB\_SCC\_UP**
