# Supplementary material for: The rapamycin-regulated gene expression signature determines prognosis for breast cancer
Source: Mol Cancer. 2009 Sep 24;8:75. doi: 10.1186/1476-4598-8-75 (PMC2761377; doi:10.1186/1476-4598-8-75)
Supplement: Additional file 3 — Gene set enrichment analysis of in vivo data, treatment series. The data provided represent the treatment series of GSEA. This compressed file contains "Treatment" shortcut file and "GSEA_treatment" folder. Clicking on "Treatment" shortcut opens the index file providing access to analysis files contained in the "GSEA_treatment" folder. [file 1476-4598-8-75-S3.zip › GSEA_treatment/UVC_HIGH_D1_DN.html]

Details for gene set UVC\_HIGH\_D1\_DN[GSEA]

|  || Dataset | gsea\_treatment\_collapsed |
| Phenotype | NoPhenotypeAvailable |
| Upregulated in class | na\_neg |
| GeneSet | UVC\_HIGH\_D1\_DN |
| Enrichment Score (ES) | -0.37344405 |
| Normalized Enrichment Score (NES) | -1.2759359 |
| Nominal p-value | 0.17117117 |
| FDR q-value | 0.2731322 |
| FWER p-Value | 1.0 |
Table: GSEA Results Summary

  

Fig 1: Enrichment plot: UVC\_HIGH\_D1\_DN      
 Profile of the Running ES Score & Positions of GeneSet Members on the Rank Ordered List

  

| PROBE | GENE SYMBOL | GENE\_TITLE | RANK IN GENE LIST | RANK METRIC SCORE | RUNNING ES | CORE ENRICHMENT || 1 | PHTF2 |  |  | 1480 | 0.288 | 0.0289 | No |
| 2 | STXBP3 |  |  | 1568 | 0.282 | 0.1234 | No |
| 3 | WEE1 |  |  | 2845 | 0.216 | 0.1373 | No |
| 4 | TOPBP1 |  |  | 3536 | 0.193 | 0.1714 | No |
| 5 | STX3 |  |  | 4250 | 0.173 | 0.1972 | No |
| 6 | ITGA6 |  |  | 4923 | 0.157 | 0.2197 | No |
| 7 | CASP3 |  |  | 7277 | 0.113 | 0.1452 | No |
| 8 | GREM1 |  |  | 10583 | 0.065 | 0.0074 | No |
| 9 | RND3 |  |  | 11199 | 0.057 | -0.0024 | No |
| 10 | U1SNRNPBP |  |  | 11746 | 0.050 | -0.0114 | No |
| 11 | E2F3 |  |  | 12095 | 0.046 | -0.0123 | No |
| 12 | HSPC111 |  |  | 16808 | -0.025 | -0.2325 | No |
| 13 | DUSP1 |  |  | 18808 | -0.076 | -0.3028 | No |
| 14 | GEM |  |  | 20264 | -0.186 | -0.3082 | Yes |
| 15 | DDIT4 |  |  | 20600 | -0.927 | 0.0002 | Yes |
Table: GSEA details [plain text format]

  

Fig 2: UVC\_HIGH\_D1\_DN: Random ES distribution      
 Gene set null distribution of ES for **UVC\_HIGH\_D1\_DN**
