# Supplementary material for: The rapamycin-regulated gene expression signature determines prognosis for breast cancer
Source: Mol Cancer. 2009 Sep 24;8:75. doi: 10.1186/1476-4598-8-75 (PMC2761377; doi:10.1186/1476-4598-8-75)
Supplement: Additional file 3 — Gene set enrichment analysis of in vivo data, treatment series. The data provided represent the treatment series of GSEA. This compressed file contains "Treatment" shortcut file and "GSEA_treatment" folder. Clicking on "Treatment" shortcut opens the index file providing access to analysis files contained in the "GSEA_treatment" folder. [file 1476-4598-8-75-S3.zip › GSEA_treatment/WANG_HOXA9_VS_MEIS1_UP.html]

Details for gene set WANG\_HOXA9\_VS\_MEIS1\_UP[GSEA]

|  || Dataset | gsea\_treatment\_collapsed |
| Phenotype | NoPhenotypeAvailable |
| Upregulated in class | na\_neg |
| GeneSet | WANG\_HOXA9\_VS\_MEIS1\_UP |
| Enrichment Score (ES) | -0.27665263 |
| Normalized Enrichment Score (NES) | -1.0924048 |
| Nominal p-value | 0.4 |
| FDR q-value | 0.46054423 |
| FWER p-Value | 1.0 |
Table: GSEA Results Summary

  

Fig 1: Enrichment plot: WANG\_HOXA9\_VS\_MEIS1\_UP      
 Profile of the Running ES Score & Positions of GeneSet Members on the Rank Ordered List

  

| PROBE | GENE SYMBOL | GENE\_TITLE | RANK IN GENE LIST | RANK METRIC SCORE | RUNNING ES | CORE ENRICHMENT || 1 | CSF2RA |  |  | 1813 | 0.265 | -0.0037 | No |
| 2 | THBS1 |  |  | 2263 | 0.242 | 0.0513 | No |
| 3 | ALAS1 |  |  | 2924 | 0.214 | 0.0871 | No |
| 4 | CTNND1 |  |  | 3114 | 0.206 | 0.1435 | No |
| 5 | SORT1 |  |  | 3791 | 0.186 | 0.1696 | No |
| 6 | BMI1 |  |  | 7236 | 0.114 | 0.0385 | No |
| 7 | ABCA13 |  |  | 8226 | 0.098 | 0.0216 | No |
| 8 | MYB |  |  | 8473 | 0.094 | 0.0396 | No |
| 9 | RHOJ |  |  | 8627 | 0.092 | 0.0615 | No |
| 10 | IGSF6 |  |  | 9060 | 0.086 | 0.0679 | No |
| 11 | CLEC7A |  |  | 9166 | 0.085 | 0.0897 | No |
| 12 | MPO |  |  | 10623 | 0.064 | 0.0394 | No |
| 13 | GDA |  |  | 10975 | 0.060 | 0.0415 | No |
| 14 | TFEC |  |  | 11972 | 0.047 | 0.0081 | No |
| 15 | MS4A3 |  |  | 12160 | 0.045 | 0.0132 | No |
| 16 | TLR4 |  |  | 13895 | 0.022 | -0.0641 | No |
| 17 | TNFSF12 |  |  | 13947 | 0.021 | -0.0598 | No |
| 18 | PRTN3 |  |  | 15579 | -0.003 | -0.1380 | No |
| 19 | CCNB1 |  |  | 15866 | -0.008 | -0.1494 | No |
| 20 | MTUS1 |  |  | 16149 | -0.013 | -0.1592 | No |
| 21 | ELA2 |  |  | 17121 | -0.031 | -0.1964 | No |
| 22 | CCND2 |  |  | 17509 | -0.040 | -0.2025 | No |
| 23 | PDE2A |  |  | 17685 | -0.044 | -0.1970 | No |
| 24 | CD177 |  |  | 18270 | -0.059 | -0.2067 | Yes |
| 25 | HSD11B1 |  |  | 18550 | -0.067 | -0.1990 | Yes |
| 26 | TGFBR3 |  |  | 18591 | -0.068 | -0.1793 | Yes |
| 27 | RNF128 |  |  | 20596 | -0.872 | 0.0004 | Yes |
Table: GSEA details [plain text format]

  

Fig 2: WANG\_HOXA9\_VS\_MEIS1\_UP: Random ES distribution      
 Gene set null distribution of ES for **WANG\_HOXA9\_VS\_MEIS1\_UP**
