# Supplementary material for: The rapamycin-regulated gene expression signature determines prognosis for breast cancer
Source: Mol Cancer. 2009 Sep 24;8:75. doi: 10.1186/1476-4598-8-75 (PMC2761377; doi:10.1186/1476-4598-8-75)
Supplement: Additional file 3 — Gene set enrichment analysis of in vivo data, treatment series. The data provided represent the treatment series of GSEA. This compressed file contains "Treatment" shortcut file and "GSEA_treatment" folder. Clicking on "Treatment" shortcut opens the index file providing access to analysis files contained in the "GSEA_treatment" folder. [file 1476-4598-8-75-S3.zip › GSEA_treatment/WIELAND_HEPATITIS_B_INDUCED.html]

Details for gene set WIELAND\_HEPATITIS\_B\_INDUCED[GSEA]

|  || Dataset | gsea\_treatment\_collapsed |
| Phenotype | NoPhenotypeAvailable |
| Upregulated in class | na\_pos |
| GeneSet | WIELAND\_HEPATITIS\_B\_INDUCED |
| Enrichment Score (ES) | 0.6286084 |
| Normalized Enrichment Score (NES) | 1.8749474 |
| Nominal p-value | 0.0 |
| FDR q-value | 0.0012555113 |
| FWER p-Value | 0.019 |
Table: GSEA Results Summary

  

Fig 1: Enrichment plot: WIELAND\_HEPATITIS\_B\_INDUCED      
 Profile of the Running ES Score & Positions of GeneSet Members on the Rank Ordered List

  

| PROBE | GENE SYMBOL | GENE\_TITLE | RANK IN GENE LIST | RANK METRIC SCORE | RUNNING ES | CORE ENRICHMENT || 1 | TRIM22 |  |  | 8 | 1.024 | 0.0424 | Yes |
| 2 | CTSB |  |  | 14 | 0.888 | 0.0793 | Yes |
| 3 | CD74 |  |  | 25 | 0.764 | 0.1108 | Yes |
| 4 | IFI27 |  |  | 32 | 0.725 | 0.1408 | Yes |
| 5 | HLA-B |  |  | 41 | 0.695 | 0.1695 | Yes |
| 6 | HLA-C |  |  | 57 | 0.650 | 0.1960 | Yes |
| 7 | BTN3A3 /// BTN3A2 |  |  | 71 | 0.625 | 0.2215 | Yes |
| 8 | TAPBP |  |  | 89 | 0.595 | 0.2456 | Yes |
| 9 | PSMB9 |  |  | 120 | 0.554 | 0.2673 | Yes |
| 10 | HLA-E |  |  | 126 | 0.545 | 0.2898 | Yes |
| 11 | GBP1 |  |  | 130 | 0.542 | 0.3124 | Yes |
| 12 | SOD2 |  |  | 150 | 0.523 | 0.3333 | Yes |
| 13 | RARRES3 |  |  | 182 | 0.503 | 0.3528 | Yes |
| 14 | HLA-F |  |  | 184 | 0.502 | 0.3738 | Yes |
| 15 | HLA-A |  |  | 195 | 0.496 | 0.3940 | Yes |
| 16 | HLA-DMA |  |  | 202 | 0.491 | 0.4143 | Yes |
| 17 | CXCL11 |  |  | 228 | 0.479 | 0.4331 | Yes |
| 18 | UCP2 |  |  | 297 | 0.454 | 0.4488 | Yes |
| 19 | GABBR1 /// UBD |  |  | 347 | 0.441 | 0.4648 | Yes |
| 20 | RGL1 |  |  | 420 | 0.421 | 0.4789 | Yes |
| 21 | RAB20 |  |  | 435 | 0.418 | 0.4957 | Yes |
| 22 | PSMB10 |  |  | 447 | 0.415 | 0.5125 | Yes |
| 23 | PSME1 |  |  | 513 | 0.402 | 0.5261 | Yes |
| 24 | LAP3 |  |  | 609 | 0.383 | 0.5375 | Yes |
| 25 | APOL3 |  |  | 632 | 0.378 | 0.5523 | Yes |
| 26 | RNASET2 |  |  | 661 | 0.373 | 0.5665 | Yes |
| 27 | CFL1 |  |  | 737 | 0.362 | 0.5780 | Yes |
| 28 | COTL1 |  |  | 840 | 0.348 | 0.5876 | Yes |
| 29 | PPT1 |  |  | 945 | 0.337 | 0.5966 | Yes |
| 30 | UBE2L6 |  |  | 980 | 0.333 | 0.6089 | Yes |
| 31 | TPP1 |  |  | 1065 | 0.325 | 0.6184 | Yes |
| 32 | GM2A |  |  | 1129 | 0.318 | 0.6286 | Yes |
| 33 | STAT1 |  |  | 1401 | 0.294 | 0.6277 | No |
| 34 | RFX5 |  |  | 1751 | 0.269 | 0.6219 | No |
| 35 | HLA-DPB1 |  |  | 2213 | 0.245 | 0.6097 | No |
| 36 | B2M |  |  | 2291 | 0.240 | 0.6160 | No |
| 37 | HLA-DPA1 |  |  | 2349 | 0.238 | 0.6232 | No |
| 38 | TYMS |  |  | 2859 | 0.216 | 0.6074 | No |
| 39 | IFI16 |  |  | 2898 | 0.214 | 0.6145 | No |
| 40 | SP110 |  |  | 3051 | 0.209 | 0.6158 | No |
| 41 | ISG20 |  |  | 3782 | 0.186 | 0.5880 | No |
| 42 | HLA-DRB1 |  |  | 4173 | 0.175 | 0.5763 | No |
| 43 | UBE2C |  |  | 4419 | 0.168 | 0.5713 | No |
| 44 | MCM6 |  |  | 4860 | 0.158 | 0.5565 | No |
| 45 | TYROBP |  |  | 5350 | 0.148 | 0.5388 | No |
| 46 | CTSC |  |  | 5510 | 0.144 | 0.5371 | No |
| 47 | FGL2 |  |  | 5915 | 0.137 | 0.5231 | No |
| 48 | HLA-DQB1 |  |  | 6637 | 0.125 | 0.4931 | No |
| 49 | RAC2 |  |  | 6758 | 0.122 | 0.4924 | No |
| 50 | PARP12 |  |  | 7072 | 0.116 | 0.4820 | No |
| 51 | CD83 |  |  | 7305 | 0.113 | 0.4754 | No |
| 52 | DRAM |  |  | 7346 | 0.112 | 0.4782 | No |
| 53 | PSME2 |  |  | 7628 | 0.108 | 0.4689 | No |
| 54 | RRM2 |  |  | 7744 | 0.106 | 0.4678 | No |
| 55 | IGL@ /// IGLC1 /// I |  |  | 7811 | 0.105 | 0.4690 | No |
| 56 | CCL5 |  |  | 8249 | 0.098 | 0.4517 | No |
| 57 | RAB31 |  |  | 9052 | 0.086 | 0.4162 | No |
| 58 | IGSF6 |  |  | 9060 | 0.086 | 0.4195 | No |
| 59 | IFI30 |  |  | 9639 | 0.078 | 0.3945 | No |
| 60 | PIK3CD |  |  | 9671 | 0.077 | 0.3962 | No |
| 61 | ARHGDIB |  |  | 9762 | 0.076 | 0.3951 | No |
| 62 | HLA-DRA |  |  | 9918 | 0.074 | 0.3906 | No |
| 63 | PTTG1 |  |  | 10051 | 0.072 | 0.3872 | No |
| 64 | HLA-DMB |  |  | 10103 | 0.072 | 0.3877 | No |
| 65 | LPXN |  |  | 10135 | 0.071 | 0.3891 | No |
| 66 | CXCL10 |  |  | 10182 | 0.071 | 0.3899 | No |
| 67 | PRG1 |  |  | 10469 | 0.066 | 0.3787 | No |
| 68 | LAPTM5 |  |  | 11330 | 0.056 | 0.3391 | No |
| 69 | DENND2D |  |  | 11798 | 0.049 | 0.3183 | No |
| 70 | LYZ /// LILRB1 |  |  | 12395 | 0.042 | 0.2910 | No |
| 71 | GZMK |  |  | 13258 | 0.031 | 0.2502 | No |
| 72 | KPNA2 /// LOC643995 |  |  | 13346 | 0.029 | 0.2472 | No |
| 73 | IGH@ /// IGHG1 /// I |  |  | 13771 | 0.024 | 0.2275 | No |
| 74 | FCGR1A /// LOC440607 |  |  | 13917 | 0.022 | 0.2214 | No |
| 75 | PLAC8 |  |  | 14358 | 0.015 | 0.2005 | No |
| 76 | SLC7A7 |  |  | 14463 | 0.014 | 0.1961 | No |
| 77 | CD3D |  |  | 14533 | 0.013 | 0.1932 | No |
| 78 | CD38 |  |  | 14579 | 0.012 | 0.1915 | No |
| 79 | GZMA |  |  | 14608 | 0.012 | 0.1907 | No |
| 80 | CXCR4 |  |  | 14667 | 0.011 | 0.1883 | No |
| 81 | IL10RA |  |  | 14740 | 0.010 | 0.1852 | No |
| 82 | CD5L |  |  | 14884 | 0.008 | 0.1786 | No |
| 83 | PBK |  |  | 15601 | -0.003 | 0.1438 | No |
| 84 | EMR1 |  |  | 15745 | -0.006 | 0.1370 | No |
| 85 | CD53 |  |  | 15773 | -0.006 | 0.1360 | No |
| 86 | AURKA |  |  | 15848 | -0.008 | 0.1327 | No |
| 87 | TRGC2 /// TRGV2 /// |  |  | 15935 | -0.009 | 0.1289 | No |
| 88 | TRBV19 /// TRBC1 |  |  | 15993 | -0.010 | 0.1265 | No |
| 89 | GIMAP5 |  |  | 16576 | -0.021 | 0.0990 | No |
| 90 | CD48 |  |  | 16886 | -0.026 | 0.0850 | No |
| 91 | C1ORF54 |  |  | 17293 | -0.035 | 0.0667 | No |
| 92 | VSIG4 |  |  | 17372 | -0.037 | 0.0644 | No |
| 93 | WARS |  |  | 17621 | -0.043 | 0.0541 | No |
| 94 | C1ORF38 |  |  | 18233 | -0.058 | 0.0267 | No |
| 95 | FCER1G |  |  | 18311 | -0.060 | 0.0255 | No |
| 96 | PRC1 |  |  | 18504 | -0.065 | 0.0188 | No |
| 97 | CD68 |  |  | 18754 | -0.075 | 0.0098 | No |
| 98 | C3AR1 |  |  | 19264 | -0.096 | -0.0110 | No |
| 99 | CXCL9 |  |  | 19477 | -0.107 | -0.0169 | No |
| 100 | GBP2 |  |  | 19549 | -0.111 | -0.0157 | No |
| 101 | RAB27A |  |  | 20046 | -0.149 | -0.0337 | No |
| 102 | C1QB |  |  | 20058 | -0.150 | -0.0279 | No |
| 103 | CECR1 |  |  | 20183 | -0.169 | -0.0269 | No |
| 104 | S100A10 |  |  | 20345 | -0.205 | -0.0262 | No |
| 105 | LGALS3 /// GALIG |  |  | 20450 | -0.253 | -0.0207 | No |
| 106 | GPNMB |  |  | 20588 | -0.674 | 0.0008 | No |
Table: GSEA details [plain text format]

  

Fig 2: WIELAND\_HEPATITIS\_B\_INDUCED: Random ES distribution      
 Gene set null distribution of ES for **WIELAND\_HEPATITIS\_B\_INDUCED**
