# Supplementary material for: The rapamycin-regulated gene expression signature determines prognosis for breast cancer
Source: Mol Cancer. 2009 Sep 24;8:75. doi: 10.1186/1476-4598-8-75 (PMC2761377; doi:10.1186/1476-4598-8-75)
Supplement: Additional file 3 — Gene set enrichment analysis of in vivo data, treatment series. The data provided represent the treatment series of GSEA. This compressed file contains "Treatment" shortcut file and "GSEA_treatment" folder. Clicking on "Treatment" shortcut opens the index file providing access to analysis files contained in the "GSEA_treatment" folder. [file 1476-4598-8-75-S3.zip › GSEA_treatment/ZHAN_MM_CD138_CD1_VS_REST.html]

Details for gene set ZHAN\_MM\_CD138\_CD1\_VS\_REST[GSEA]

|  || Dataset | gsea\_treatment\_collapsed |
| Phenotype | NoPhenotypeAvailable |
| Upregulated in class | na\_neg |
| GeneSet | ZHAN\_MM\_CD138\_CD1\_VS\_REST |
| Enrichment Score (ES) | -0.44228056 |
| Normalized Enrichment Score (NES) | -1.9382955 |
| Nominal p-value | 0.0 |
| FDR q-value | 0.021609046 |
| FWER p-Value | 0.132 |
Table: GSEA Results Summary

  

Fig 1: Enrichment plot: ZHAN\_MM\_CD138\_CD1\_VS\_REST      
 Profile of the Running ES Score & Positions of GeneSet Members on the Rank Ordered List

  

| PROBE | GENE SYMBOL | GENE\_TITLE | RANK IN GENE LIST | RANK METRIC SCORE | RUNNING ES | CORE ENRICHMENT || 1 | C20ORF100 |  |  | 1270 | 0.305 | -0.0103 | No |
| 2 | HADH |  |  | 1330 | 0.300 | 0.0375 | No |
| 3 | TMC6 |  |  | 2580 | 0.227 | 0.0150 | No |
| 4 | CEBPB |  |  | 2588 | 0.227 | 0.0529 | No |
| 5 | ITGAL |  |  | 3743 | 0.187 | 0.0284 | No |
| 6 | TBC1D16 |  |  | 3951 | 0.180 | 0.0488 | No |
| 7 | ATF5 |  |  | 4158 | 0.175 | 0.0683 | No |
| 8 | NSUN7 |  |  | 5560 | 0.143 | 0.0244 | No |
| 9 | TM6SF1 |  |  | 8321 | 0.097 | -0.0935 | No |
| 10 | SYTL1 |  |  | 8856 | 0.089 | -0.1044 | No |
| 11 | RAB33A |  |  | 8945 | 0.088 | -0.0939 | No |
| 12 | RAB31 |  |  | 9052 | 0.086 | -0.0845 | No |
| 13 | FYN |  |  | 9174 | 0.085 | -0.0761 | No |
| 14 | TXNDC13 |  |  | 9373 | 0.082 | -0.0719 | No |
| 15 | ZNF70 |  |  | 9809 | 0.076 | -0.0803 | No |
| 16 | MTG1 |  |  | 10331 | 0.068 | -0.0941 | No |
| 17 | ERN1 |  |  | 12895 | 0.035 | -0.2128 | No |
| 18 | G0S2 |  |  | 13020 | 0.034 | -0.2131 | No |
| 19 | PCDH20 |  |  | 13683 | 0.025 | -0.2411 | No |
| 20 | RGAG4 |  |  | 14410 | 0.015 | -0.2739 | No |
| 21 | EIF4EBP1 |  |  | 14651 | 0.011 | -0.2837 | No |
| 22 | CLEC2D |  |  | 16188 | -0.013 | -0.3561 | No |
| 23 | CTH |  |  | 16261 | -0.014 | -0.3572 | No |
| 24 | VPS37A |  |  | 16311 | -0.015 | -0.3570 | No |
| 25 | TMC8 |  |  | 16486 | -0.019 | -0.3623 | No |
| 26 | GPR63 |  |  | 16964 | -0.028 | -0.3807 | No |
| 27 | SLC7A11 |  |  | 17273 | -0.035 | -0.3899 | No |
| 28 | RBP1 |  |  | 17843 | -0.048 | -0.4095 | No |
| 29 | MFAP4 |  |  | 18241 | -0.058 | -0.4190 | No |
| 30 | SNFT |  |  | 18721 | -0.073 | -0.4299 | Yes |
| 31 | ALPK2 |  |  | 18753 | -0.075 | -0.4188 | Yes |
| 32 | BGLAP |  |  | 18985 | -0.084 | -0.4159 | Yes |
| 33 | NID2 |  |  | 19125 | -0.090 | -0.4075 | Yes |
| 34 | ASB2 |  |  | 19169 | -0.092 | -0.3941 | Yes |
| 35 | KLHL4 |  |  | 19298 | -0.098 | -0.3837 | Yes |
| 36 | PTGER2 |  |  | 19983 | -0.143 | -0.3929 | Yes |
| 37 | PCK2 |  |  | 20288 | -0.191 | -0.3753 | Yes |
| 38 | SESN2 |  |  | 20456 | -0.257 | -0.3401 | Yes |
| 39 | INHBE |  |  | 20468 | -0.265 | -0.2958 | Yes |
| 40 | PHGDH |  |  | 20474 | -0.271 | -0.2502 | Yes |
| 41 | GPT2 |  |  | 20527 | -0.344 | -0.1946 | Yes |
| 42 | CSTA |  |  | 20529 | -0.346 | -0.1362 | Yes |
| 43 | TRIB3 |  |  | 20539 | -0.374 | -0.0734 | Yes |
| 44 | STC2 |  |  | 20562 | -0.453 | 0.0021 | Yes |
Table: GSEA details [plain text format]

  

Fig 2: ZHAN\_MM\_CD138\_CD1\_VS\_REST: Random ES distribution      
 Gene set null distribution of ES for **ZHAN\_MM\_CD138\_CD1\_VS\_REST**
