# Supplementary material for: The rapamycin-regulated gene expression signature determines prognosis for breast cancer
Source: Mol Cancer. 2009 Sep 24;8:75. doi: 10.1186/1476-4598-8-75 (PMC2761377; doi:10.1186/1476-4598-8-75)
Supplement: Additional file 3 — Gene set enrichment analysis of in vivo data, treatment series. The data provided represent the treatment series of GSEA. This compressed file contains "Treatment" shortcut file and "GSEA_treatment" folder. Clicking on "Treatment" shortcut opens the index file providing access to analysis files contained in the "GSEA_treatment" folder. [file 1476-4598-8-75-S3.zip › GSEA_treatment/ZHAN_MM_MOLECULAR_CLASSI_UP.html]

Details for gene set ZHAN\_MM\_MOLECULAR\_CLASSI\_UP[GSEA]

|  || Dataset | gsea\_treatment\_collapsed |
| Phenotype | NoPhenotypeAvailable |
| Upregulated in class | na\_neg |
| GeneSet | ZHAN\_MM\_MOLECULAR\_CLASSI\_UP |
| Enrichment Score (ES) | -0.42109096 |
| Normalized Enrichment Score (NES) | -2.2058208 |
| Nominal p-value | 0.0 |
| FDR q-value | 0.004392888 |
| FWER p-Value | 0.011 |
Table: GSEA Results Summary

  

Fig 1: Enrichment plot: ZHAN\_MM\_MOLECULAR\_CLASSI\_UP      
 Profile of the Running ES Score & Positions of GeneSet Members on the Rank Ordered List

  

| PROBE | GENE SYMBOL | GENE\_TITLE | RANK IN GENE LIST | RANK METRIC SCORE | RUNNING ES | CORE ENRICHMENT || 1 | WFDC2 |  |  | 355 | 0.438 | 0.0287 | No |
| 2 | LOC162073 |  |  | 357 | 0.437 | 0.0745 | No |
| 3 | RTN3 |  |  | 1134 | 0.317 | 0.0700 | No |
| 4 | C1ORF24 |  |  | 1192 | 0.311 | 0.0998 | No |
| 5 | HADH |  |  | 1330 | 0.300 | 0.1246 | No |
| 6 | LOC340061 |  |  | 2554 | 0.227 | 0.0889 | No |
| 7 | CEBPB |  |  | 2588 | 0.227 | 0.1111 | No |
| 8 | STOM |  |  | 3147 | 0.205 | 0.1054 | No |
| 9 | ASS1 |  |  | 3567 | 0.192 | 0.1051 | No |
| 10 | ITGAL |  |  | 3743 | 0.187 | 0.1162 | No |
| 11 | TBC1D16 |  |  | 3951 | 0.180 | 0.1251 | No |
| 12 | ATF5 |  |  | 4158 | 0.175 | 0.1334 | No |
| 13 | DNAJC12 |  |  | 5672 | 0.141 | 0.0745 | No |
| 14 | KCNK12 |  |  | 6158 | 0.132 | 0.0648 | No |
| 15 | CPNE8 |  |  | 7025 | 0.118 | 0.0350 | No |
| 16 | SETD7 |  |  | 7119 | 0.115 | 0.0426 | No |
| 17 | TM6SF1 |  |  | 8321 | 0.097 | -0.0057 | No |
| 18 | RAB33A |  |  | 8945 | 0.088 | -0.0269 | No |
| 19 | RAB31 |  |  | 9052 | 0.086 | -0.0230 | No |
| 20 | PCDH18 |  |  | 9191 | 0.084 | -0.0209 | No |
| 21 | KLF4 |  |  | 9867 | 0.075 | -0.0459 | No |
| 22 | NPAS3 |  |  | 10535 | 0.066 | -0.0714 | No |
| 23 | USP32 |  |  | 10704 | 0.063 | -0.0730 | No |
| 24 | ASNS |  |  | 11946 | 0.048 | -0.1284 | No |
| 25 | TRAF3IP3 |  |  | 12161 | 0.045 | -0.1341 | No |
| 26 | FAM14B |  |  | 12833 | 0.036 | -0.1630 | No |
| 27 | ERN1 |  |  | 12895 | 0.035 | -0.1623 | No |
| 28 | ALDH1L2 |  |  | 12967 | 0.035 | -0.1621 | No |
| 29 | G0S2 |  |  | 13020 | 0.034 | -0.1611 | No |
| 30 | PCDH20 |  |  | 13683 | 0.025 | -0.1907 | No |
| 31 | RGAG4 |  |  | 14410 | 0.015 | -0.2245 | No |
| 32 | SLA |  |  | 14437 | 0.014 | -0.2242 | No |
| 33 | EIF4EBP1 |  |  | 14651 | 0.011 | -0.2334 | No |
| 34 | GALNT3 |  |  | 15713 | -0.005 | -0.2845 | No |
| 35 | CTAGE5 |  |  | 16654 | -0.022 | -0.3280 | No |
| 36 | GPR63 |  |  | 16964 | -0.028 | -0.3401 | No |
| 37 | DMGDH |  |  | 17199 | -0.033 | -0.3480 | No |
| 38 | SLC7A11 |  |  | 17273 | -0.035 | -0.3479 | No |
| 39 | WARS |  |  | 17621 | -0.043 | -0.3603 | No |
| 40 | RBP1 |  |  | 17843 | -0.048 | -0.3661 | No |
| 41 | LOC283143 |  |  | 18415 | -0.063 | -0.3873 | No |
| 42 | ARMCX1 |  |  | 19110 | -0.089 | -0.4118 | Yes |
| 43 | NID2 |  |  | 19125 | -0.090 | -0.4030 | Yes |
| 44 | ASB2 |  |  | 19169 | -0.092 | -0.3955 | Yes |
| 45 | KLHL4 |  |  | 19298 | -0.098 | -0.3914 | Yes |
| 46 | MIRN21 |  |  | 19447 | -0.106 | -0.3875 | Yes |
| 47 | C9ORF150 |  |  | 19627 | -0.115 | -0.3841 | Yes |
| 48 | C20ORF103 |  |  | 19763 | -0.124 | -0.3777 | Yes |
| 49 | CEBPG |  |  | 19811 | -0.128 | -0.3666 | Yes |
| 50 | MTHFD2 |  |  | 20131 | -0.162 | -0.3651 | Yes |
| 51 | PCK2 |  |  | 20288 | -0.191 | -0.3527 | Yes |
| 52 | CORO1A |  |  | 20425 | -0.237 | -0.3344 | Yes |
| 53 | KIF21B |  |  | 20429 | -0.239 | -0.3095 | Yes |
| 54 | SESN2 |  |  | 20456 | -0.257 | -0.2838 | Yes |
| 55 | INHBE |  |  | 20468 | -0.265 | -0.2565 | Yes |
| 56 | PHGDH |  |  | 20474 | -0.271 | -0.2283 | Yes |
| 57 | VLDLR |  |  | 20512 | -0.317 | -0.1968 | Yes |
| 58 | GPT2 |  |  | 20527 | -0.344 | -0.1614 | Yes |
| 59 | SLC7A5 |  |  | 20535 | -0.371 | -0.1228 | Yes |
| 60 | TRIB3 |  |  | 20539 | -0.374 | -0.0837 | Yes |
| 61 | TPBG |  |  | 20540 | -0.375 | -0.0444 | Yes |
| 62 | STC2 |  |  | 20562 | -0.453 | 0.0021 | Yes |
Table: GSEA details [plain text format]

  

Fig 2: ZHAN\_MM\_MOLECULAR\_CLASSI\_UP: Random ES distribution      
 Gene set null distribution of ES for **ZHAN\_MM\_MOLECULAR\_CLASSI\_UP**
